# Decoding drug-responsive cell subpopulations in triple-negative breast cancer using single-cell multiomics

**DOI:** 10.1016/j.isci.2026.115445

**Published:** 2026-03-21

**Authors:** Yue Wang, Santiago Haase, Austin Whitman, Adriana S. Beltran, Philip M. Spanheimer, Elizabeth Brunk

**Affiliations:** 1 Department of Pharmacology, University of North Carolina at Chapel Hill, Chapel Hill, NC, USA; 2 Integrative Program for Biological and Genome Sciences (IBGS), University of North Carolina at Chapel Hill, Chapel Hill, NC, USA; 3 Lineberger Comprehensive Cancer Center, University of North Carolina at Chapel Hill, Chapel Hill, NC, USA; 4 Department of Genetics, University of North Carolina at Chapel Hill, Chapel Hill, NC, USA; 5 Department of Surgery, University of North Carolina at Chapel Hill, Chapel Hill, NC, USA; 6 Computational Medicine Program, University of North Carolina at Chapel Hill, Chapel Hill, NC, USA; 7 Department of Chemistry, University of North Carolina at Chapel Hill, Chapel Hill, NC, USA

**Keywords:** Classification of bioinformatical subject, Expression study, Cancer, Transcriptomics

## Abstract

Understanding how individual cancer cells adapt to drug treatment is a fundamental challenge limiting precision medicine cancer therapy strategies. Here, we present a multimodal framework that integrates bulk and single-cell treated and untreated transcriptomics data to identify drug-responsive cell populations in triple-negative breast cancer (TNBC). Our framework defines seven bulk-level “identities,” each representing unique combinations of biologically relevant genes. These trackable identities are further mapped onto single cells and uncover global patterns of how cell populations respond to drug treatment. By capturing the evolving nature of cellular states, we show that a select few identities dominate and drive population-level responses during treatment, which allows us to better predict how entire tumors respond to treatment. This insight is essential for designing precise combination therapies tailored to the unique heterogeneity of patient tumors, addressing the single-cell variations that ultimately determine therapeutic outcomes.

## Highlights

• A framework bridging bulk and single-cell omics data identifies drug-responsive cells
• The framework defined 7 validated identities in triple-negative breast cancer
• Identity-specific cancer cell clusters drive drug adaptation in tumors
• Epigenetic regulation accompanies transcriptomic identity shifts

## Graphical abstract

**Figure:**
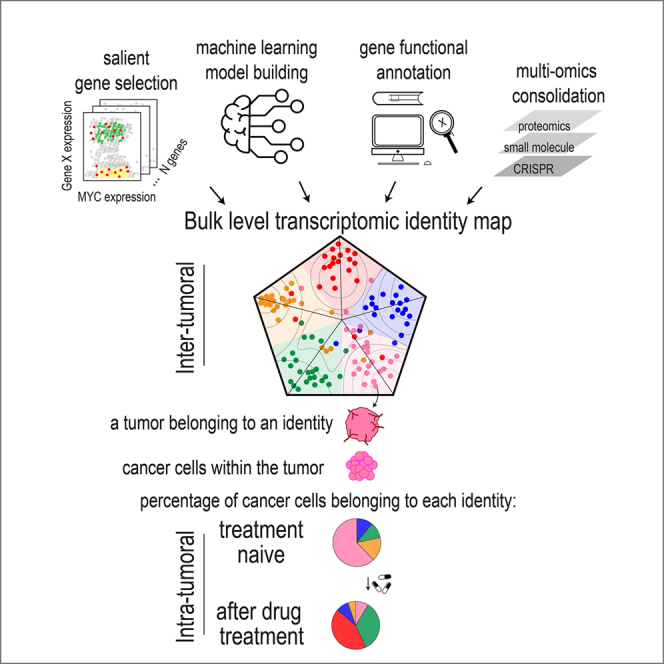

## Introduction

How cancer cells adapt to drug treatment is one of the most pressing challenges in cancer research. Understanding how tumor cells respond, adapt, and resist therapies is essential for developing strategies that target these distinct populations within tumors. Within a single tumor, cells can respond to therapy in dramatically different ways^1^: some are eliminated, while others adapt, survive, and drive resistance.^2^^,^^3^ This response heterogeneity undermines even the most promising therapies,^4^^,^^5^^,^^6^ making it critical to identify which subpopulations of cells are responsible for emerging resistance.

In recent years, the explosion of omics technologies has produced an unprecedented wealth of data. We now have access to transcriptomic profiles spanning diverse cancer types, experimental conditions, and molecular modalities at bulk and single-cell levels. This includes untreated and drug-treated samples, offering a rare opportunity to explore how cancer cells adapt at the molecular level. However, the sheer volume and complexity of these datasets present significant challenges. Distinguishing meaningful biological signals from noise amid thousands of single cells remains a daunting computational task.^7^^,^^8^^,^^9^^,^^10^ In the context of clinical research, this challenge extends to aggregating data into therapeutically meaningful patterns while maintaining the granularity needed to characterize rare or low-abundance cell populations. Despite numerous efforts to address this complexity,^11^^,^^12^^,^^13^^,^^14^^,^^15^^,^^16^^,^^17^^,^^18^ current approaches often focus on predicting drug responses and lack the corresponding single-cell omics data in the drug treatment setting, leaving many adaptive mechanisms unresolved and limiting our ability to uncover global trends in drug response.

Efforts to integrate datasets across untreated and treated conditions have been hindered by the lack of unified repositories and comprehensive analytical tools, though longitudinal datasets exist.^19^^,^^20^^,^^21^^,^^22^^,^^23^ As a result, most studies of drug response focus on deep investigations of individual cell lines or tumor models.^24^^,^^25^^,^^26^^,^^27^^,^^28^ While these studies offer valuable mechanistic insights, they are often limited in scope and provide little opportunity to identify trends that are broadly applicable across samples in a population. The field urgently needs scalable methods capable of uncovering population-wide patterns—tools that can illuminate how adaptation arises, predict drug responses across diverse cancers, and inform the development of more effective therapies. Moreover, fully leveraging the wealth of available multiomics data requires approaches that bridge bulk and single-cell datasets,^29^^,^^30^^,^^31^ integrating large-scale trends with single-cell granularity. Addressing these gaps would represent a significant step forward in advancing precision medicine.

In this study, we tackle these challenges by introducing a multimodal framework that integrates bulk and single-cell transcriptomics data to uncover population-scale trends in drug response. We apply this framework to triple-negative breast cancer (TNBC), a highly aggressive subtype of breast cancer that lacks effective targeted therapies and is marked by variable treatment responses.^32^^,^^33^ By harmonizing bulk and single-cell datasets from untreated and drug-treated conditions, our framework identifies transcriptionally distinct subpopulations with specific molecular “identities” within TNBC cell lines and tumors. By grouping thousands of cells into biologically relevant identities, this approach enables the identification and characterization of subpopulations of cells that may be driving treatment-induced changes, offering a critical lens into how tumors adapt and evolve under therapeutic pressure.

By capturing population-scale patterns and linking them to single-cell behaviors, our framework addresses a critical unmet need in cancer research. It enables the identification of nuanced, biologically meaningful shifts in cellular identities that drive adaptation and resistance, ensuring critical signals are not lost in complex data. Our analysis revealed striking adaptive signatures, with the largest transcriptional shifts often associated with a small number of highly adaptive subpopulations of cells characterized by key identities. As a proof of principle, we applied our framework to our TNBC organoid model, collecting single-cell multiomics data from untreated and treated samples following treatment with epigenetic remodeling drugs. Analysis of this model validated that the predicted identity shifts correspond to distinct epigenetic regulatory mechanisms, highlighting the biological relevance of our approach. Beyond TNBC, this approach has the potential to uncover actionable molecular vulnerabilities across cancer types, paving the way for precision medicine strategies to outpace resistance and improve patient outcomes.

## Results

### Harmonizing bulk and single-cell transcriptomics for defining subpopulation identities

**Figure 1:**
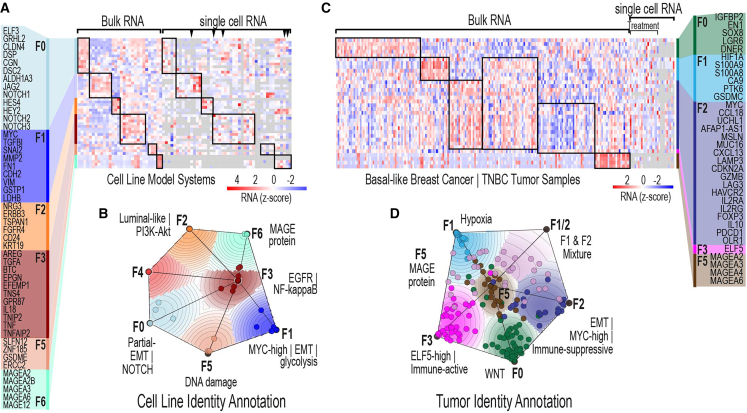
Gene expression identity landscapes for TNBC cell lines and tumor samples (A) Heatmap illustrates the overall transcriptomic data resource for CCLE TNBC cell lines. Each column represents a cell line, and each row corresponds to the expression of genes defining specific identities. Identities and their representative gene groups are annotated on the left side of the heatmap. Samples associated with the same identity are outlined with black boxes, while single-cell RNA-seq data with corresponding drug treatment conditions are indicated along the top edge of the heatmap. Black triangles indicate drug-treated samples. (B) Transcriptomic identity map for 30 TNBC cell lines, highlighting the identity groupings derived from CCLE data. (C) Heatmap of the transcriptomic data resource for basal-like breast cancer and TNBC tumor samples. Each column represents a tumor sample, and each row corresponds to the expression of genes defining specific identities. Identities and their associated gene groups are annotated on the right side of the heatmap. Samples grouped by the same identity are outlined with black boxes, and patient single-cell RNA-seq data with corresponding drug treatment conditions are marked along the top edge of the heatmap. (D) Transcriptomic identity map for 185 TCGA basal-like breast tumor samples, highlighting the identity clusters derived from TCGA data.

To establish a comprehensive framework for defining subpopulation identities, we curated publicly available datasets focusing on TNBC. We identified 30 cell lines annotated as TNBC from the Cancer Cell Line Encyclopedia (CCLE) and dependency map (DepMap), which included 76 transcriptomic datasets, 30 bulk and 46 single cell datasets.^34^^,^^35^^,^^36^ For the single cell data, five have corresponding drug-treatment datasets.^37^^,^^38^^,^^39^ These datasets offered a robust foundation for exploring the molecular diversity of TNBC, encompassing both untreated and treated conditions. The availability of multiomics data allowed us to harmonize bulk and single-cell transcriptomics, enabling detailed analysis of cell subpopulation variability (Figure 1A).

To identify the genes that best define distinct subpopulations, we performed a genome-wide analysis to pinpoint those with the highest variance across TNBC cell lines. Given the well-established role of MYC as a global gene regulator impacting TNBC,^40^^,^^41^^,^^42^^,^^43^ we prioritized significantly varied genes and examined pairwise relationships between MYC and each gene to identify those that most distinctly separated the TNBC cell lines into discrete clusters. This analysis identified 523 genes with highly divergent expression patterns, providing a basis for separating transcriptionally distinct subpopulations. As expected, these genes highlighted diverse biological pathways, including MYC dysregulation, a hallmark of TNBC biology, as well as other processes critical for understanding adaptive responses to treatment, including NOTCH, PI3K-Akt, and EGFR signaling.

Using the most divergent genes, we applied non-negative matrix factorization (NMF) to identify transcriptional groups, or “identities,” among TNBC cell lines. This approach revealed seven subpopulation identities, each characterized by unique gene expression (GE) profiles that capture the heterogeneity across 30 TNBC cell lines. These identities were further visualized using a two-dimensional cluster map, which clearly illustrated the distinct groupings of TNBC cell lines (Figure 1B).

To provide biological context, we performed detailed literature searches (Table S1) to annotate each identity, focusing on the most relevant biological features associated with the defining genes (Figures S1–S7). Identity F2 was enriched for luminal-like genes and PI3K-Akt signaling, while Identity F1 was associated with markers of epithelial-to-mesenchymal transition (EMT) and glycolysis. Three cell lines classified as F2 (MDAMB453, SUM185PE, and MFM223) had previously been assigned to the luminal androgen receptor (LAR) subtype, as defined by Lehmann et al*.*^44^ To explore this relationship further, we compared the F2-defining genes with the LAR subtype gene set and found partial overlap (14 of 89 genes; Figure S8A). To determine whether these two programs were co-expressed within the same cells, we analyzed single-cell RNA-seq (scRNA-seq) data from two F2-identity cell lines (MDAMB453 and MFM223) and found significant correlations between the expression of F2- and LAR-associated gene sets across individual cells (Figures S8B–S8D). While the correlation coefficients were moderate (r = 0.34, 0.17, and 0.25, respectively), they were statistically significant and consistent across datasets, suggesting biological co-variation. These findings indicate that the F2 identity captures key features of the LAR subtype but is not fully defined by androgen receptor signaling alone. Instead, the F2 identity represents a broader luminal-like transcriptional state that integrates AR activity with other signaling programs such as PI3K-Akt. This broader functional context may offer a refined molecular lens for interpreting TNBC heterogeneity and for identifying responsive subpopulations in the context of emerging AR-targeted therapies. Overall, these annotations illuminated the diverse molecular processes driving subpopulation variability, offering insights into potential therapeutic targets.

We extended our analysis to The Cancer Genome Atlas (TCGA) basal-like breast cancer datasets to evaluate the relevance of these subpopulation identities in patient samples (Figure 1C). Notably, several subpopulations identified in cell lines were conserved in TCGA tumors, suggesting that these transcriptional identities are biologically meaningful and clinically relevant (Figures 1D and S9–S13). For instance, Identities F5 and F1-and-F2-mixture characterized by genes involved in the MAGE protein family and high-MYC and hypoxia/glycolysis programs, respectively, were consistently observed across both datasets (Figure S9A).

We observed that TCGA samples assigned to different identities may display distinct survival trends, although these differences were not statistically significant in small subgroup sample sizes (Figure S9B). Specifically, subpopulation F1-and-F2-mixture, distinguished by both high hypoxia and immune suppressive genes, showed a trend toward better overall survival compared with other subpopulations. While these observations did not reach statistical significance, they suggest that subpopulation-specific transcriptional identities may capture biologically meaningful heterogeneity with potential relevance to patient outcomes. Future studies in larger, independent cohorts will be required to determine whether these subpopulation-defined patterns can be leveraged for robust prognostic stratification or therapeutic decision-making.

Through bulk transcriptomics analysis, we identified transcriptionally distinct subpopulations, referred to as identities, that capture the inherent heterogeneity of TNBC. These identities lay the groundwork for understanding the molecular processes underlying variability in TNBC outcomes and demonstrate their clinical relevance, presenting opportunities for precision medicine in this aggressive cancer subtype. Additionally, these findings establish a baseline understanding of TNBC cell identities in treatment-naive conditions, providing a crucial foundation for investigating drug response trajectories under therapeutic pressure.

### Integrating multimodal data to define and validate subpopulation identities in the CCLE tumor-intrinsic model

**Figure 2:**
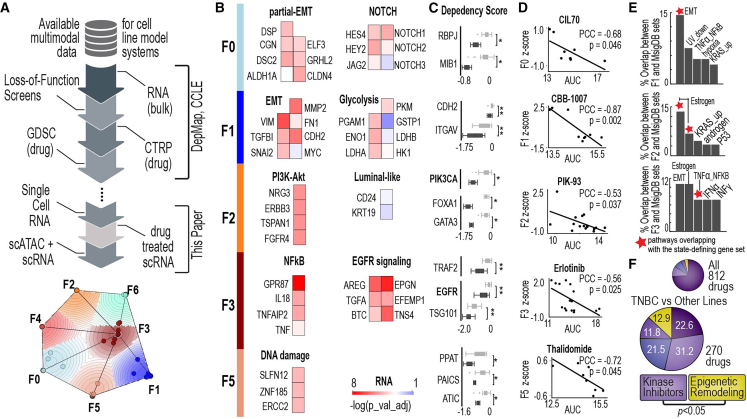
Integrating multimodal data to define and validate subpopulation identities in the CCLE tumor-intrinsic model (A) The workflow for annotating TNBC cell line identities, providing an overview of the methodology used to assign biologically meaningful features to these identities. (B) Gene expression evidence support the biology of TNBC cell line identities. The heatmap presents the adjusted *p*-values from Wilcoxon rank-sum tests comparing gene expression levels in cell lines belonging to a specific identity versus all others. Genes representative of each identity show significantly higher expression within the associated samples, confirming their relevance to the defined biological features. (C) CRISPR dependency data validate the biological characteristics of each TNBC cell line identity. Dependency scores are shown for cell lines belonging to each identity compared to those outside it. Dark gray boxplots represent cell lines associated with the identity, while light gray boxplots represent others. For example, RBPJ and MIB1 highlight NOTCH signaling in the F0 identity; CDH2 and ITGAV emphasize EMT features in F1; PIK3CA, FOXA1, and GATA3 define PI3K-Akt signaling and luminal-like traits in F2; and TRAF2, EGFR, and TSG101 highlight NF-κB and EGFR signaling in F3. Boxplot center line indicates median; box bounds represent the interquartile range (IQR); whiskers extend to 1.5×IQR. (D) Linking drug sensitivity to gene expression identities within TNBC cell lines. Correlations between identity levels and drug sensitivity are depicted with Pearson’s correlation coefficients (PCCs) and *p*-values. Key examples include drugs such as CIL70 (ferroptosis inducer), CBB-1007 (histone demethylase inhibitor), PIK-93 (PI3K inhibitor), Erlotinib (EGFR inhibitor), and Thalidomide (immunomodulatory drug). (E) The overlap between genes defining TNBC cell line identities and MsigDB hallmark gene sets. Bar plots show the percentage of overlapping genes across hallmark sets, with notable overlaps including EMT, TNFα signaling via NF-κB, estrogen responses (early and late), KRAS signaling, and interferon responses. These findings further validate the biological significance of the identified identities and their connections to known pathways. (F) The enriched drug-like molecule classes that show significant differential responses between TNBC and other cancer types by chi-squared test. Together, these panels demonstrate the utility of integrating multi-omics data to define and validate biologically relevant subpopulation identities within TNBC cell lines. ∗*p* < 0.05 and ∗∗*p* < 0.01 by the Wilcoxon rank-sum test (C).

We used harmonized multimodal data to validate the transcriptionally distinct subpopulation identities defined in TNBC cell lines. This harmonized dataset integrates bulk RNA sequencing, CRISPR loss-of-function screens, and drug sensitivity data from repositories such as the Cancer Therapeutics Response Portal (CTRP)^45^^,^^46^ and the Genomics of Drug Sensitivity in Cancer (GDSC)^47^^,^^48^ (Figure 2A). By combining these distinct phenotypic datasets, we aimed to independently validate and characterize the biological relevance of the TNBC subpopulation identities identified through NMF.

We analyzed GE data to identify the most significantly differentially expressed genes in each subpopulation compared to other subpopulations. Some of the most significant genes were independently validated, confirming their biological relevance. For example, in the F3 subpopulation, genes associated with EGFR signaling were significantly upregulated (Figure 2B), and MYC expression is associated with F1 identity (Figure 2B).

We examined CRISPR loss-of-function screen data to identify unique gene dependencies of specific subpopulations. Notably, in the F2 and F3 subpopulations, PIK3CA and EGFR emerged as the top genes showing significant differential effects (Figure 2C). These findings align with the biological identities of these subpopulations: F2 is associated with luminal-like and PI3K-Akt signaling signatures, while F3 is associated with EGFR and NF-κB signaling. In addition, the F0 subpopulation demonstrated dependencies on RBPJ and MIB1, genes critical to Notch signaling,^49^^,^^50^ further reinforcing the link between this subpopulation’s identity and its molecular characteristics.

Using large-scale population-wide drug sensitivity curves from GDSC and CTRP, we investigated drugs whose efficacy varied according to the “weight” or explained variance score derived from the NMF matrix, which quantifies the association of each cell line with a given subpopulation identity. Because the F2 identity is characterized by PI3K-Akt signaling and PI3K gene dependency, we hypothesized that cell lines with increasing association with F2 identity would have enhanced sensitivity to the PI3K and Akt inhibitors. As predicted, we observed correlation between F2 identity and sensitivity to PI3K inhibitors, such as PIK-93 (Figures 2D, S14A, and S14B) and Akt inhibitors, Afuresertib and Uprosertib (Figures S14C and S14D). Additionally, we observed correlations between F3 identity and sensitivity to EGFR inhibitors, namely erlotinib, gefitinib, and PD-153035 (Figures 2D, S14E, and S14F). The F3 identity represents a transcriptional program enriched for EGFR and NF-κB signaling, two pathways known to drive aggressive biology, therapeutic resistance, and immune evasion in TNBC. Importantly, this is the only identity in our framework with the concurrent upregulation of both EGFR and NF-κB, suggesting a uniquely plastic and survival-oriented state. The retrospective validation of the F3-EGFR dependency using independent functional studies is particularly striking: Among multiple TNBC lines tested, only the F3-identity line HCC1937 showed robust sensitivity to EGFR inhibition at both the signaling (pAKT), cell-cycle (G1 arrest), and combinatorial therapy levels.^51^ This level of concordance between a computationally derived identity and experimentally verified drug susceptibility underscores the biological coherence of the F3 state and highlights its translational significance as a therapeutically actionable subpopulation. These findings suggest that subpopulation identities derived from highly variable GE levels directly associate with drug response *in vitro*.

To further validate the biological context of these identities, we compared their defining genes to hallmark gene sets from MsigDB.^52^ For some identities, 10–15% of genes also appear in key MsigDB hallmark gene sets (Figure 2E). For example, the F2 identity strongly overlaps with hallmark gene sets related to estrogen response (early and late), consistent with its luminal-like characteristics (Figure 2E). The F2 identity also overlaps with the androgen response hallmark gene set, which is consistent with the LAR subtype of TNBC.^44^^,^^53^ Importantly, our framework offers insights that go beyond those provided by hallmark gene sets; hallmark gene sets are typically derived from single perturbation experiments, often using controlled cell line models subjected to specific interventions, such as CRISPR knockouts or drug treatments. These sets capture changes in GE or chromatin states driven by the dynamics of the experimental system. In contrast, our approach provides a global, population-wide view of heterogeneity, identifying gene “signatures” that reflect variability across the population rather than within the context of a specific experimental perturbation.

Finally, we analyzed drug response data in TNBC cell lines from CCLE, GDSC, and CTRP to identify classes of drugs with significantly different effects in TNBC compared to other cancer types. Out of 812 drugs, epigenetic remodeling agents stood out as the only class showing significant enrichment and efficacy in TNBC cell lines (Figure 2F). These agents modify chromatin accessibility, influencing GE, and are increasingly being explored for their therapeutic potential in TNBC.^54^^,^^55^ Notably, the F1 identity exhibited a significant sensitivity to CBB-1007, a histone demethylase inhibitor, further underscoring the relevance of epigenetic regulation in this subpopulation (Figure 2D). The enrichment of epigenetic remodeling agents in TNBC highlights their promise for targeting this aggressive and therapeutically challenging cancer subtype.

### Deriving single-cell identities and tracking drug-responsiveness in TNBC cell lines

**Figure 3:**
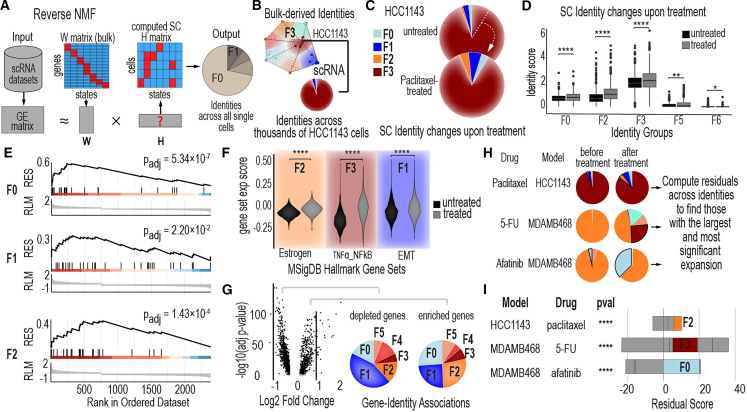
Deriving single-cell identities and tracking drug-responsiveness in TNBC cell lines (A) Workflow illustrates the mapping of bulk-level cancer cell line identities onto single cells in TNBC cell lines, enabling single-cell-level analysis of identity changes. (B) Single-cell identity composition of HCC1143, a TNBC cell line associated with the F3 identity in the CCLE model. The pie chart displays the distribution of single cells across different identities within this cell line prior to treatment. (C) Single-cell identity shifts in HCC1143 after paclitaxel treatment. Pie charts illustrate the percentage of single cells belonging to each identity before and after treatment, revealing changes in the identity composition. (D) Identity scores for single cells in HCC1143 before and after paclitaxel treatment. These scores, derived from the reverse NMF H matrix, indicate the intensity of gene expression identities at the single-cell level. Boxplot center line indicates median; box bounds represent the interquartile range (IQR); whiskers extend to 1.5×IQR. (E) Gene set enrichment analysis (GSEA) results for F0, F1, and F2 gene sets after paclitaxel treatment. The analysis highlights the enrichment or depletion of these gene sets in treated single cells, providing insights into identity-specific responses to treatment. (F) Expression scores for three MsigDB hallmark gene sets in HCC1143 single cells before and after treatment. The hallmark gene sets analyzed include estrogen response (early and late combined), TNFα signaling via NF-κB, and epithelial-mesenchymal transition (EMT), showcasing treatment-induced changes in key biological pathways. Violin plots show the distribution of expression scores. (G) Volcano plots of differentially expressed genes (DEGs) in HCC1143 single cells after paclitaxel treatment. For upregulated genes, the pie chart on the right illustrates the proportion of DEGs belonging to each identity as defined in the CCLE model. Similarly, for downregulated genes, the pie chart on the left shows the identity distribution of DEGs. (H) Illustration of residual score computation to quantify identity changes after drug treatments. Residual scores provide a measure of how much each identity expands or contracts in response to treatment. (I) Global characterization of identity changes across different TNBC cell lines treated with various drugs/compounds. Chi-squared test *p*-values assess the significance of identity changes for each cell line after treatment. Stacked bar plots depict the residual scores for each identity, with positive residual scores indicating identity expansion and negative residual scores indicating identity shrinkage. ∗*p* < 0.05, ∗∗*p* < 0.01, ∗∗∗*p* < 0.001, and ∗∗∗∗*p* < 0.0001 by the Wilcoxon rank-sum test (D and F) and chi-squared test (I).

To connect bulk-derived subpopulation identities to single-cell resolution, we used a reverse NMF approach^56^ (Figure 3A). This method enables the mapping of subpopulation identities derived from bulk RNA-seq data to individual cells within scRNA-seq datasets. Conceptually, the bulk RNA-seq data provides a framework for defining overarching subpopulation identities, represented as a matrix of GE patterns (W matrix). For a given single-cell dataset, we input its GE matrix and solve for the contribution of each subpopulation (H matrix). Here, the W matrix comes from bulk RNA-seq data from CCLE TNBC cell lines, and the H matrix represents the single-cell labels assigned to each subpopulation (Figure 3A). This allows us to annotate each cell in a single-cell dataset with a specific identity from the bulk-derived framework (Figure S15).

As a proof-of-principle case study, we analyzed scRNA-seq data from paclitaxel-treated and untreated HCC1143 cells. Using our framework, we mapped individual cells to their closest matching identity, revealing that 94% of the HCC1143 population belonged to the F3 subpopulation, while 6% were distributed among F0, F1, and F2 identities (2% F0, 3% F1, 1% F2). The F3 subpopulation is characterized by distinct molecular features, including upregulated EGFR and NF-κB signaling (Figure 3B). Notably, the predominance of F3 cells aligns with unmatched bulk RNA-seq data from the same cell line, where HCC1143 is classified as having an F3 identity at the bulk level due to the dominance of this subpopulation.

Paclitaxel treatment caused a notable expansion of the non-F3 subpopulations, increasing overall population heterogeneity (Figure 3C). Subpopulation identity scores before and after treatment revealed significant shifts in most identities (Figure 3D), demonstrating how drug treatment reshapes the population’s identity composition, with nearly all identity scores showing dynamic changes.

To validate the significance of these shifts at the gene level and understand their impact on the population as a whole, we performed gene set enrichment analysis (GSEA) on the scRNA-seq data. GSEA provided quantitative metrics, revealing significant enrichment and depletion of gene sets associated with distinct subpopulation identities. This analysis confirmed that genes associated with the F0, F1, and F2 identities were significantly enriched following drug treatment (Figure 3E). Additionally, hallmark gene sets from MsigDB most closely aligned with these changing subpopulations—such as estrogen response for F2, TNFα signaling via NF-κB for F3, and EMT for F1—exhibited significant changes, providing complementary evidence of the biological relevance of these identities and their treatment-induced shifts (Figure 3F). These findings highlight how these subpopulations are linked to distinct biological processes that are dynamically regulated in response to treatment.

We aimed to determine whether specific genes disproportionately contributed to identity shifts during drug treatment. To address this, we performed differential GE analysis between treated and untreated conditions (Figure 3G), identifying the most significantly altered genes and mapping them to their associated subpopulation identities. While gene-level changes were observed across all identities, the F0, F2, and F3 subpopulations exhibited the most substantial changes in HCC1143, suggesting a potential role for these genes and identities in drug responsiveness. Our analysis further revealed that certain identities played a dominant role in driving the observed shifts. For instance, in HCC1143, the F2 identity emerged as the primary driver of change, contributing most significantly to the overall subpopulation-level shifts observed after treatment (Figure 3I). Extending this analysis to other TNBC cell lines (Figure 3H) confirmed that specific identities, such as F2 and F3, were consistently associated with the most adaptive and dynamic subpopulations (Figures 3I and S16).

Our proof-of-principle analysis demonstrates the strength of integrating bulk-derived subpopulation identities with single-cell data to uncover how distinct subpopulations shift and drive adaptation during drug treatment. By defining cell populations through subpopulation-level identities, we show that certain identities—or their combinations—are closely linked to drug responsiveness, offering valuable insights into the population-level mechanisms underlying treatment adaptation.

### Decoding drug response in patient samples using single-cell identity annotation

**Figure 4:**
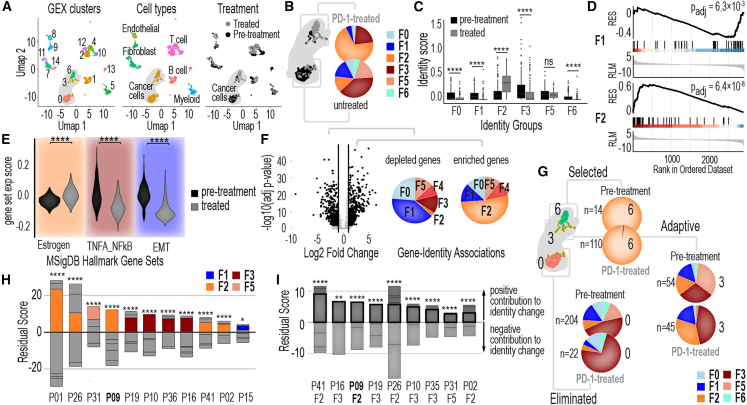
Decoding drug response in patient samples using single-cell identity annotation (A) UMAP plots show cluster identifications, cell types, and treatment conditions for all single cells in the BIOKEY_9 (B9) tumor. These plots provide an overview of the cellular landscape within the tumor, highlighting distinct clusters and their associations with treatment conditions. (B) Single-cell identity changes in the cancer cells of TNBC patient BIOKEY_9 (B9) after pembrolizumab treatment. Pie charts illustrate the distribution of single cells across different identities within the cancer cell population before and after treatment. (C) Identity scores for single cells in the BIOKEY_9 (B9) cancer cell population before and after pembrolizumab treatment. These scores quantify the intensity of gene expression identities at the single-cell level, revealing shifts in cellular identities induced by treatment. Boxplot center line indicates median; box bounds represent the interquartile range (IQR); whiskers extend to 1.5×IQR. (D) GSEA results for F1 and F2 gene sets after pembrolizumab treatment, highlighting the enrichment or depletion of these identity-specific gene sets in the treated cancer cell population. (E) Expression scores for three MsigDB hallmark gene sets in BIOKEY_9 (B9) cancer single cells before and after treatment. The hallmark gene sets analyzed include estrogen response (early and late combined), TNFα signaling via NF-κB, and epithelial-mesenchymal transition (EMT), providing insights into treatment-induced changes in key biological pathways. Violin plots show the distribution of expression scores. (F) Volcano plots of differentially expressed genes (DEGs) in BIOKEY_9 (B9) cancer single cells after pembrolizumab treatment. Pie charts accompanying the plots display the percentage of DEGs associated with each identity in the CCLE model. The right pie chart represents upregulated genes, while the left represents downregulated genes, linking these changes to specific identities. (G) Analysis of identity composition changes in three cancer cell clusters from BIOKEY_9, B9 (cluster_0, cluster_6, and cluster_3), before and after pembrolizumab treatment. Pie charts illustrate the percentage of single cells belonging to each identity within each cluster, revealing differential responses across clusters. (H) Global characterization of identity enrichment or depletion for cancer cells within 11 TNBC tumors following anti-PD1 treatment. Chi-squared test results assess the significance of identity changes across tumors, with stacked bar plots depicting residual scores for each identity. Positive residual scores indicate identity expansion, while negative scores indicate depletion. (I) Analysis of cancer cell clusters contributing to the largest identity expansions in cancer cells for each tumor. Chi-squared test *p*-values assess whether specific clusters are more associated with identity expansion compared to other clusters within the same tumor. Stacked bar plots represent the residual scores for each cluster, highlighting their contributions to identity changes. ∗*p* < 0.05, ∗∗*p* < 0.01, ∗∗∗*p* < 0.001, and ∗∗∗∗*p* < 0.0001 by the Wilcoxon rank-sum test (C and E) or by chi-squared test (H and I).

We applied our framework to single-cell transcriptomics data from 13 TNBC patient samples treated with the anti-PD1 immune checkpoint inhibitor pembrolizumab.^57^ Of these, 11 were treatment-naive prior to pembrolizumab, while 2 (BIOKEY_41, B41, and BIOKEY_35, B35) received neoadjuvant chemotherapy before anti-PD1 treatment. Single-cell analyses grouped cells into distinct clusters based on GE, corresponding to distinct cell types, with drug treatment inducing noticeable shifts within these clusters (Figure 4A). Using our bulk-derived identity mapping framework, we mapped each cancer cell to its corresponding identity across all treatment-naive and treated patient samples. This enabled us to characterize the composition of tumor subpopulations within each sample and track how these compositions changed after treatment. By comparing pre- and post-treatment compositions, we identified subpopulations with unique identity profiles that were either enriched, depleted, or exhibited adaptive responses to pembrolizumab.

As a proof-of-concept, we applied our approach to a representative patient sample (BIOKEY_9) with tumor samples collected before and after pembrolizumab monotherapy (Figure 4A). We identified six distinct subpopulations within the tumor cells, each composed of different identities (Figure 4B). Prior to treatment, the majority of cells were part of the F3 subpopulation; however, following treatment, the F2 subpopulation became predominant. The most notable shifts involved the expansion of F2 and the depletion of F1 and F3 identities (Figure 4C). GSEA^58^ further validated the significance of the shifts in F1 and F2 identities (Figure 4D).

These identity shifts correspond to biologically relevant changes in MsigDB hallmark gene sets associated with each identity. For example, the F2 identity exhibited a significant increase in estrogen response (early and late), the F3 identity showed a decrease in TNFα signaling via NF-κB, and the F1 identity displayed the depletion of EMT-related processes (Figure 4E). These findings align with the known biological roles of these subpopulations: F2 is linked to luminal-like characteristics, while F3 is associated with TNFα signaling via NF-κB and EGFR pathways. Differential GE analysis further revealed that most genes enriched after treatment mapped to the F2 identity, while the majority of depleted genes were associated with the F1 identity (Figure 4F).

We sought to determine whether distinct clusters of cells, initially grouped by GE, exhibited unique identity shifts in response to drug treatment. By analyzing these clusters independently, we uncovered striking differences in their behavior following treatment (Figure 4G). In the first cluster (cluster_6), predominantly composed of cells with an F2 identity, the identity composition remained stable, but the total number of cells increased after treatment, suggesting a selection mechanism favoring this subpopulation. In contrast, the second cluster (cluster_0), a mix of six identities dominated by F3, experienced a dramatic decline in cell numbers post-treatment, indicating possible elimination or selection against this subpopulation. The third cluster (cluster_3), another mix of six identities, displayed substantial shifts in composition without a significant change in total cell numbers, pointing to an adaptive subpopulation capable of dynamically reconfiguring under drug pressure. These findings highlight the power of our framework in dissecting drug responsiveness, revealing how distinct cell clusters, defined by their unique identity compositions, contribute differently to treatment outcomes.

### Global patterns of adaptation across patient samples

Building on these findings, we expanded our analysis to multiple patient samples^57^ (Figures S17 and S18, and Table S2) to determine whether global trends in identity shifts could be identified. This analysis revealed an intriguing pattern: Certain identities consistently played a disproportionate role in driving changes in subpopulation composition during treatment (Figure 4H). To quantify these contributions, we calculated a residual score for each identity, measuring the extent to which they influenced the overall shifts observed (Figure 4H). To investigate if disproportionate contribution to identity shifts from certain cancer cell clusters is a global trend, we focused on the identity with the most significant expansion during treatment for each patient and calculated a residual score for each cancer cell cluster regarding the identity expansion (Figure 4I). This approach enabled us to capture both positive and negative contributions from each cluster to the identity expansion, with identities with the most significant expansion during treatment highlighted in the figure (Figure 4I). Globally, we found that distinct cell clusters seemed to contribute differently to molecular treatment outcomes.

Notably, the F2 and F3 identities consistently emerged as key drivers of the largest shifts in subpopulation composition across patient samples (Figure 4H). Subpopulations, grouped by GE and defined by their identity composition, revealed that those dominated by F2 and F3 identities exhibited the most pronounced changes in response to drug treatment. These shifts, characterized by significant expansion or decline of these identities, highlight their pivotal role in driving treatment-induced adaptation. These findings emphasize that the composition of subpopulations—particularly their enrichment in F2 and F3 identities—is a critical determinant of therapy responsiveness, underscoring the value of identity-level analysis in understanding treatment outcomes.

These findings underscore the importance of identifying and targeting adaptive subpopulations at the single-cell level. Building on this, future studies may explore whether tumors enriched in F2 and F3 identities possess an enhanced capacity to adapt and evade therapy, driven by their distinct biological characteristics. The F2 identity is linked to luminal-like traits and PI3K-Akt signaling, while F3 is associated with NF-κB and EGFR pathways—hallmarks of treatment resistance and adaptability. The disproportionate influence of these identities highlights how tumors leverage specific adaptive programs under therapeutic pressure. By targeting these dominant subpopulations, it may be possible to disrupt critical survival and adaptive mechanisms, offering a promising strategy to mitigate therapy resistance and improve long-term treatment outcomes.

### Modeling complexity: Identifying drug-responsive identities in a TNBC organoid model

Our findings reveal that certain identities are inherently more adaptive, driving significant shifts that shape therapeutic adaptation. To investigate the mechanisms underlying these shifts, we developed an organoid model system that captures the complexity of tumor heterogeneity while providing a testable framework for assessing drug sensitivity. Unlike traditional cell lines, organoid models offer a more realistic representation of tumor diversity, enabling us to integrate single-cell RNA sequencing with single-cell epigenetic profiling to study treatment responses. Applying our framework to these organoid models allowed us to validate the identity shifts observed during treatment and uncover the molecular and epigenetic mechanisms driving these adaptations.

**Figure 5:**
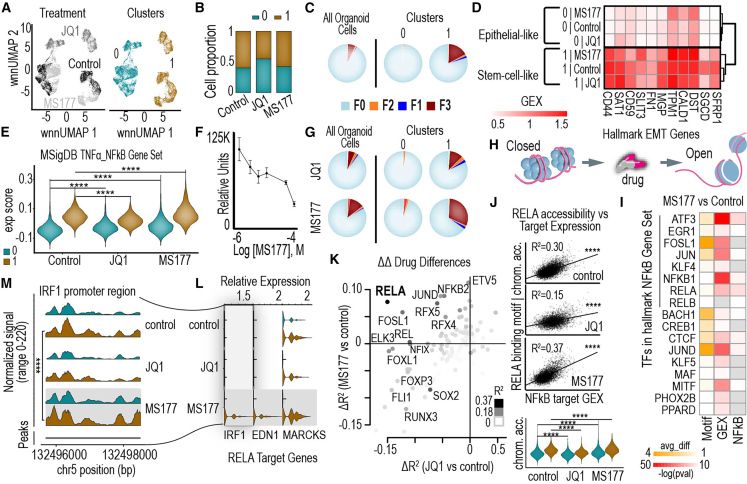
Investigating identity changes after drug treatment in a TNBC organoid model (A) UMAP plots show treatment conditions and cluster identifications of single cells within the TNBC organoid, providing an overview of the cellular landscape under different treatment conditions. (B) Bar plots depict the proportions of single cells belonging to each cluster across the three treatment conditions, highlighting shifts in cluster composition following drug treatment. (C) Pie charts illustrat the percentage of single cells belonging to each identity in the organoid as a whole and within each of the two distinct clusters under control conditions. (D) Comparison of gene expression levels for stem-cell/EMT markers between the epithelial-like and stem-cell-like clusters within the organoid across the three treatment conditions, revealing identity-specific transcriptional profiles. (E) Expression scores for the MsigDB hallmark gene set “TNFα signaling via NF-κB” for the two clusters within the organoid across the three treatment conditions, demonstrating differential pathway activation. Violin plots show the distribution of expression scores. (F) Drug treatment curve shows the response of the organoid to MS177, indicating the relative efficacy of the treatment on the organoid population. (G) Pie charts show the percentage of single cells belonging to each identity in the organoid as a whole and within the two distinct clusters under JQ1 and MS177 treatment conditions. (H) Schematic illustration depicts how drug treatments, such as MS177, open chromatin regions, enabling potential gene transcription and altering regulatory dynamics. (I) Analysis of transcription factors (TFs) relevant to the response to MS177 treatment by the Wilcoxon rank-sum test. The first column shows the average difference in TF binding motif accessibility after MS177 treatment. The second column highlights the significance of TFs in upregulating gene expression broadly after MS177 treatment. The third column focuses on TFs that significantly upregulate gene expression specifically in NF-κB pathway genes after MS177 treatment. (J) Scatterplots show R^2^ between RELA binding motif accessibility and the expression scores of RELA target genes in the NF-κB pathway under control, JQ1-treated, and MS177-treated conditions. Violin plots below display RELA binding motif chromatin accessibility levels in the two clusters (clusters 0 and 1) across the three treatment conditions. (K) Scatterplot compares changes in R^2^ values between TF binding motif accessibility and the expression scores of their target genes in the NF-κB pathway following JQ1 (x axis) and MS177 (y axis) treatments for all TFs. Dot colors indicate the R^2^ value under MS177-treated conditions. (L) Gene expression levels of RELA targets MARCKS, EDN1, and IRF1 in the two clusters across the three treatment conditions. Violin plots show the distribution of expression levels. Chromatin accessibility in the promoter regions of these genes, where RELA binds, increases after MS177 treatment, linking chromatin remodeling to transcriptional changes. (M) Chromatin accessibility of the IRF1 promoter region in the two clusters across the three treatment conditions, demonstrating treatment-induced epigenetic remodeling. ∗*p* < 0.05, ∗∗*p* < 0.01, ∗∗∗*p* < 0.001, and ∗∗∗∗*p* < 0.0001 by the Wilcoxon rank-sum test (E and J bottom panel) or *t* test (J top panel) or likelihood ratio test (M).

We developed an organoid model derived from a primary human TNBC tumor obtained from a UNC Lineberger Comprehensive Cancer Center patient (Figure S19) and treated it with the epigenetic remodeling agents JQ1 (a BRD4 inhibitor) and MS177 (an EZH2 degrader). These agents were selected based on prior findings (Figure 2F) showing that epigenetic remodeling drugs elicited the most significant differences in drug response between TNBC cell lines and other cancer types. The organoid model demonstrated sensitivity to both drugs (Figures 5F and S21A). To further investigate these responses, we generated matched single-cell multiomics data before and after treatment with each drug and applied our multimodal framework to analyze subpopulation identity shifts and their role in treatment adaptation.

Both scRNA and scATAC sequencing analyses revealed distinct drug-treatment clusters and corresponding GE and accessibility clusters, labeled 0 and 1 (Figure 5A). Across all three treatment conditions, these clusters maintained a consistent 3:2 cell number ratio (Figure 5B). Mapping cells to bulk-derived CCLE subpopulation identities showed that the organoid was predominantly represented by the F0 state, characterized by partial EMT and Notch signaling. Notably, the identity compositions of the two clusters differed significantly: Cluster 0 was almost entirely dominated by the F0 identity, while nearly one-quarter of the cells in Cluster 1 belonged to F1, F2, and F3 subpopulations, highlighting a greater degree of identity heterogeneity (Figure 5C).

Given the presence of the F1 identity in cluster 1, we analyzed hallmark EMT gene sets under control and treatment conditions, revealing that cluster 1 exhibited a stem-cell-like phenotype, while cluster 0 was more epithelial-like (Figure 5D). Similarly, the F3 identity in cluster 1 prompted an analysis of hallmark TNFα via NF-κB signaling gene sets, which showed significant differences between the epithelial (low NF-κB) and stem-like (high NF-κB) clusters. Notably, MS177 treatment specifically increased NF-κB signaling in cluster 1 cells, further reinforcing its stem-like characteristics^59^^,^^60^ (Figure 5E). Remarkably, our analysis uncovered the adaptive nature of cluster 1 identities during drug treatment. For example, the F3 identity expanded to nearly one-third of the total cell population following MS177 treatment, emphasizing its adaptive capacity (Figure 5G).

### Drug-responsive identities are defined by distinct epigenetic regulation

We hypothesized that the large-scale identity shifts observed in cluster 1 during drug treatment are driven by significant changes in chromatin accessibility. Chromatin accessibility, which determines how “open” or “closed” DNA is at specific loci, directly regulates the binding of transcription factors (TFs) and other regulatory proteins, thereby influencing GE (Figure 5H). With epigenetic remodeling drugs, we anticipate substantial changes in chromatin accessibility that ultimately drive the identity composition shifts within this cluster. For instance, in cluster 1 cells, altered chromatin accessibility at key regulatory regions likely corresponds to the GE shifts observed in identities such as F1, F2, and F3, which significantly expand following MS177 treatment.

To test this hypothesis, we analyzed chromatin accessibility changes at TF binding motifs associated with regulators of the NF-κB pathway. Specifically, we examined TF binding motif accessibility changes after MS177 treatment (Figure 5I, first column), assessed the general involvement of these TFs in upregulating GE (Figure 5I, second column), and evaluated their specific roles in the NF-κB signaling pathway (Figure 5I, third column). Several TFs, including ATF3 and RELA, exhibited increased binding motif accessibility alongside upregulated GE in the NF-κB signaling pathway, implicating these TFs as key mediators of MS177-induced transcriptional changes. Notably, RELA, a critical NF-κB subunit required for nuclear translocation and activation, emerged as a compelling candidate, with its binding motif accessibility strongly correlating with the expression of its target genes in the NF-κB signaling pathway (Figure 5J).

We further examined the relationship between chromatin accessibility and GE by systematically analyzing the correlation between accessibility at enriched TF binding motifs and the expression of the TF’s target genes within the NF-κB pathway. Consistent with expectations, RELA demonstrated the strongest correlation (R^2^) in the MS177-treated condition (Figures 5J, 5K, S20, and S21B–S21D), with this relationship significantly enhanced under MS177 treatment (Figures 5J and 5K). Furthermore, RELA binding motif accessibility was specifically induced by MS177, with a higher level observed in cluster 1 cells (Figure 5J, bottom panel). These findings establish a mechanistic link between RELA activity, the expansion of NF-κB signaling, and the adaptive responses exhibited by cluster 1 cells during MS177 treatment.

To investigate how these changes correlate with specific subpopulation identities, we focused on TFs with selective increases in R^2^ under MS177 treatment but not JQ1. RELA again emerged as a key regulator (Figure 5K), aligning with the observed expansion of the F3 identity, which occurs predominantly under MS177 treatment. Within this framework, three RELA target genes in the NF-κB pathway—IRF1, EDN1, and MARCKS—exhibited distinct increases in GE, consistent with the F3 identity expansion under MS177 treatment (Figure 5L). These genes, along with other genes in the NF-κB hallmark pathway (Figure S22), displayed higher expression in cluster 1 compared to cluster 0, specifically in response to MS177. Additionally, chromatin accessibility at the promoter regions of these three genes increased following MS177 treatment (Table S3). These findings further support the role of RELA in mediating NF-κB signaling and the adaptive expansion of the F3 subpopulation during treatment.

We investigated the promoter regions of these genes for chromatin accessibility differences. For IRF1, we observed significant accessibility differences between cluster 0 and cluster 1, particularly after MS177 treatment (Figure 5M). This observation suggests that epigenetic regulation plays a significant role in shaping the F3 identity during MS177 treatment. While IRF1 promoter accessibility provides an illustrative example of these changes, it likely reflects broader epigenetic differences between clusters that contribute to the dynamic remodeling of subpopulation identities during drug treatment.

Our analyses reveal that the adaptive changes in cluster 1 may be explained by increased NF-κB signaling and chromatin remodeling, which collectively support the expansion of the F3 identity. These findings highlight that drug-responsive identities, such as F3, are likely shaped not only by transcriptional changes but also by dynamic shifts in epigenetic regulation. The observed differences in chromatin accessibility between clusters, alongside TF activity, uncover key adaptive mechanisms driving subpopulation shifts under therapeutic pressure. This work underscores the critical interplay between epigenetic and transcriptional regulation in defining drug-responsive identities and emphasizes the potential of targeting these mechanisms to disrupt tumor adaptation. By linking changes in GE with chromatin accessibility, we gain valuable insights into how cancer subpopulations adapt, paving the way for more effective therapies that exploit the vulnerabilities of cells driven by adaptive identities.

In summary, we first leveraged large-scale bulk RNA-seq datasets to construct TNBC cellular state models that are predictive of drug response. We then validated the biological relevance of these states by integrating CRISPR gene-dependency screens and public drug-response datasets. Next, by incorporating scRNA-seq profiles from tumors collected before and after treatment, we demonstrated that specific subpopulations with defined identities predominantly drive adaptive responses to therapy. Finally, through the integration of chromatin accessibility and single-cell multiome data, we uncovered the epigenetic regulatory programs that underlie these transcriptional adaptations.

## Discussion

This study introduces a multimodal framework that integrates bulk and single-cell transcriptomics to define and characterize drug-responsive subpopulation identities. By bridging large-scale population data with single-cell resolution, this approach uncovers meaningful biological signals within the complexity of single-cell data. It enables the systematic mapping of adaptive cell identities under therapeutic pressure, addressing a key challenge in cancer research and advancing the development of precise strategies to overcome treatment resistance.

Through our analyses of cell lines, organoid models, and patient samples, we discovered global patterns of drug response that consistently point to the adaptive response of select subpopulation identities. Across these systems, certain identities emerge as more malleable, driving significant shifts in subpopulation identity composition during drug treatment. These findings emphasize the importance of understanding how tumors evolve at the level of their cellular diversity, providing key insights into the mechanisms that enable therapeutic evasion. To develop clinically actionable treatment strategies, future studies will need to 1) prospectively validate the impact of identities and shifting identities on therapeutic response, and 2) determine the efficacy of targeting identity-specific therapeutic vulnerabilities to enhance response and improve outcomes.

Our work also highlights the utility of single-cell multiomics in explaining the observed shifts in GE with changes in epigenetic regulation. The ability to link these shifts underscores the utility of single-cell multiomics in revealing the molecular underpinnings of adaptive subpopulations. Here, we discover that broader epigenetic differences exist between clusters and contribute to the dynamic remodeling of subpopulation identities during epigenetic remodeling drug treatment. Understanding regulatory networks underlying identity-plasticity on treatment could accelerate the prediction of identity shifts and be used to identify patient-specific co-treatment vulnerabilities.

This framework contributes to the growing body of work that seeks to unravel the complexities of cancer heterogeneity and treatment response. Enabling the identification of adaptive subpopulations and their underlying mechanisms provides a critical step toward precision medicine. Ultimately, this work lays the foundation for therapeutic strategies that not only target tumors at their current state but also anticipate and disrupt their future adaptive trajectories, offering hope for more durable and effective cancer treatments.

While our study focuses primarily on tumor-intrinsic transcriptional programs underlying drug responsiveness in TNBC, the tumor microenvironment (TME) also plays a critical role in shaping therapeutic outcomes. The patient scRNA-seq dataset we utilized was originally analyzed with an emphasis on TME dynamics, revealing molecular adaptations in T cells, macrophages, and dendritic cells during anti-PD1 treatment.^57^ These findings, together with other studies that have characterized TME remodeling in response to therapy^61^^,^^62^^,^^63^ in TNBC, provide an important biological context for our observations. Integrating tumor-intrinsic and microenvironmental programs in future analyses will be key to building a more comprehensive understanding of how cells in TME contribute to drug response and resistance in TNBC.

### Limitations of the study

Cancer cell pre-treatment identity composition and/or the specific identity shifts could serve as a biomarker for anti-PD1 therapy response and clinical outcomes. In our current study, further development of the identity-based biomarker is limited by the small TNBC sample size and the lack of published patient clinical outcome data for robust validation of effects on treatment response and outcomes. To develop such biomarkers, future studies will need a large cohort of patients with TNBC with longitudinal scRNA-seq data over the course of drug treatment to determine the impact of identity composition and shifts in composition on patient outcomes.

## Resource availability

### Lead contact

Requests for further information and resources should be directed to and will be fulfilled by the lead contact, Elizabeth Brunk (elizabeth_brunk@med.unc.edu).

### Materials availability

The TNBC organoid model system was generated in this study. Requests for this material can be directed to the lead contact and will be considered on a case-by-case basis.

### Data and code availability

• Data: This paper analyzes existing, publicly available data at cBioPortal, CCLE (https://depmap.org/portal/), Lambrechts lab website (http://biokey.lambrechtslab.org), and GEO. Data acquired from GEO has accession numbers of GEO:GSE288798, GEO: GSE182694, GEO: GSE174391, GEO: GSE152315, GEO: GSE173634, GEO: GSE202771, GEO: GSE139129, GEO: GSE164715, GEO: GSE228154, and GEO: GSE176078. Single-cell multiomics data generated from the TNBC organoid model system are available on Gene Expression Omnibus (GEO accession number GSE288798).
• Code: All codes are available at GitHub (https://github.com/Brunk-Lab/TNBC_Identities).
• All other items: Any additional information required to reanalyze the data reported in this paper is available from the lead contact upon request.

## Acknowledgments

This work was supported in part by the 10.13039/100000002National Institutes of Health grants K08CA280388, R37CA292075, R01CA280482, P30CA016086, and the UNC Lineberger Center for Triple Negative Breast Cancer.

## Author contributions

Conceptualization, E.B. and P.S.; methodology, E.B., and Y.W.; formal analysis, Y.W., S.H., and E.B.; funding acquisition, E.B. and P.S.; investigation, E.B. and P.S.; resources, E.B., P.S., A.W., and A.B.; supervision, E.B. and P.S.; validation, Y.W.; visualization, Y.W. and E.B.; writing – original draft, E.B. and Y.W.; writing – review and editing, All authors. All authors have read and agreed to the published version of the manuscript.

## Declaration of interests

The authors declare no competing interests.

## STAR★Methods

### Key resources table

**Table:** 

REAGENT or RESOURCE	SOURCE	IDENTIFIER
**Biological samples**		

TNBC organoid model system	this paper	–

**Critical commercial assays**		

Chromium Next GEM Single Cell Multiome ATAC + Gene Expression Reagent Bundle	10X genomics	PN-1000285
Chromium Next GEM Chip J Single Cell Kit	10X genomics	PN-1000230
Single Index Kit N Set A	10X genomics	PN-1000212
Dual Index Kit TT Set A	10X genomics	PN-1000215

**Deposited data**		

TNBC cell line RNA-seq data	CCLE (https://depmap.org/portal/)	v22Q2
TNBC cell line CRISPR gene effect data	CCLE (https://depmap.org/portal/)	v22Q2
TNBC cell line CTRP drug response data	CCLE (https://depmap.org/portal/)	v2.0
TCGA TNBC tumor RNA-seq data	cBioPortal	brca_tcga_pan_can_atlas_2018
10X Single-cell multiome data from a TNBC organoid in control, JQ1-treated, and MS177-treated conditions	This paper	GEO:GSE288798
Treatment-naive TNBC cell line scRNA-seq datasets	Dave et al.^64^	GEO: GSE182694
Treatment-naive TNBC cell line scRNA-seq datasets	Rashid et al.^36^	GEO: GSE174391
Treatment-naive MDAMB453 scRNA-seq	https://www.ncbi.nlm.nih.gov/geo/query/acc.cgi	GEO: GSE152315
Treatment-naive TNBC cell line scRNA-seq datasets	Gambardella et al.^34^	GEO: GSE173634
Treatment-naive TNBC cell line scRNA-seq datasets	Jovanovic et al.^35^	GEO: GSE202771
HCC1143 scRNA-seq data paclitaxel treated	https://www.ncbi.nlm.nih.gov/geo/query/acc.cgi	GEO: GSE139129
MDAMB468 scRNA-seq data 5-fluorouracil treated	Raimundo et al.^65^	GEO: GSE164715
MDAMB468 scRNA-seq data afatinib-treated	Pellecchia et al.^39^	GEO: GSE228154
TNBC patient tumor scRNA-seq data (treatment-naive)	Wu et al.^66^	GEO: GSE176078
TNBC patient tumor scRNA-seq data (anti-PD1 treatment)	Bassez et al.^57^	http://biokey.lambrechtslab.org

**Software and algorithms**		

Cell Ranger ARC(v2.0.1)	10X genomics	https://www.10xgenomics.com/
Lifelines (v0.27.4)	Pilon et al.^67^	https://pypi.org/project/lifelines/
ccal (v0.9.4)	Kim et al.^68^	https://github.com/UCSD-CCAL/onco_gps_paper_analysis
scipy (v1.8.0)	Virtanen et al.^69^	https://scipy.org/
sklearn (v1.2.2)	Pedregosa et al.^70^	https://scikit-learn.org/stable/
R (v4.2.1/v4.3.1)	https://www.r-project.org/	https://www.r-project.org/
Python(v3.8.12)	https://www.python.org/downloads/release/python-3812/	https://www.python.org/downloads/release/python-3812/
ggplot2	Wickham et al.^71^	https://ggplot2.tidyverse.org/
nnls	Mullen et al.^56^	https://cran.r-project.org/web/packages/nnls/index.html
clusterProfiler	Yu et al.^72^	https://bioconductor.org/packages/release/bioc/html/clusterProfiler.html
enrichplot (v1.18.4)	Yu et al.^73^	https://bioconductor.org/packages/release/bioc/html/enrichplot.html
DoubletFinder (v2.0.4)	McGinnis et al.^74^	https://github.com/chris-mcginnis-ucsf/DoubletFinder
Seurat (v4.4.0)	Hao et al.^75^	https://satijalab.org/seurat/
Signac (v1.10.0)	Stuart et al.^76^	https://stuartlab.org/signac/1.2.0/
infercnv (v1.21.0)	inferCNV of the Trinity CTAT Project.	https://github.com/broadinstitute/inferCNV
SingleR (v2.4.1)	Aran et al.^77^	https://github.com/dviraran/SingleR
celldex (v1.12.0)	Aran et al.^77^	https://bioconductor.org/packages/devel/data/experiment/html/celldex.html
JASPAR2020 (v0.99.10)	Fornes et al.^78^	http://jaspar.genereg.net
ChromVAR (v1.20.2)	Schep et al.^79^	https://bioconductor.org/packages/release/bioc/html/chromVAR.html
ChIPseeker(v1.34.1)	Yu et al.^80^ and Wang et al.^81^	https://www.bioconductor.org/packages/release/bioc/html/ChIPseeker.html
TxDb.Hsapiens.UCSC.hg38. knownGene (v.3.16.0)	Bioconductor Core Team	https://bioconductor.org/packages/release/data/annotation/html/TxDb.Hsapiens.UCSC.hg38.knownGene.html
org.Hs.eg.db (v3.16.0)	Marc Carlson.^82^	https://bioconductor.org/packages/release/data/annotation/html/org.Hs.eg.db.html
GenomicRange (v1.50.2)	Lawrence et al.^83^	https://bioconductor.org/packages/release/bioc/html/GenomicRanges.html
Enrichr database	Chen et al.^84^, Kuleshov et al.^85^, and Xie et al.^86^	https://maayanlab.cloud/Enrichr/
Accession codes	–	https://github.com/Brunk-Lab/TNBC_Identities

### Experimental model and study participant details

#### Human subjects

Human data analyzed in this study were obtained from publicly available, de-identified databases.

#### Organoid model systems

Tumor samples were obtained from a female patient with grade 3 invasive ductal carcinoma at the University of North Carolina (UNC) under IRB-approved protocols with patients providing written informed consent. The tumor was estrogen receptor negative (0%), progesterone receptor negative (0%) and HER2 negative (0%). To ensure cell viability, the tumor tissue was processed within 1–2 hours of surgical resection. The tissue was finely minced and enzymatically digested using a collagenase-hyaluronidase mix (StemCell Technologies) for 1–2 hours at 37°C. Following digestion, red blood cells were removed using ammonium chloride solution, and the remaining cell clusters were resuspended in growth factor-reduced Matrigel (Corning). Cell clusters were seeded as 20 μL Matrigel domes in 6-well plates and cultured in breast organoid medium. This medium consisted of Advanced DMEM:F12 (Gibco) supplemented with B27, N2, Glutamax, HEPES, Penicillin/Streptomycin, Primocin, Nicotinamide, N-Acetylcysteine, R-spondin 3, Heregulin β-1, Noggin, FGF-10, FGF-7, EGF, A83-01, SB202190, and Y-27632 (added for the first three days to enhance cell survival). Media were refreshed every 3–4 days. Upon reaching confluency, typically once per week, organoids were manually dissociated and passaged by enzymatic digestion with TrypLE (Gibco) for 8 minutes at 37°C.

### Method details

For basal-like breast cancer (BLBC) analysis, PAM50 intrinsic subtypes were assigned to each patient sample using an ER/HER2 subgroup-specific gene-centering method.^87^ Basal subtype samples were selected for further analysis.

#### Defining gene expression landscapes and identity profiles in TNBC cell lines and tumors

##### Gene selection with high variance across samples

To identify genes with high expression variance, we analyzed TNBC cell lines (CCLE) and basal-like breast cancer patient tumors (TCGA) separately. To further explore phenotypes associated with the MYC oncogene, DBSCAN clustering (Python sklearn package v1.2.2) was applied to two-dimensional expression data consisting of each gene and MYC. The eps parameter was systematically varied from 0.1 to 1.21 at intervals of 0.01 to optimize clustering. Genes were filtered based on DBSCAN^88^ cluster results. To ensure that genes input into downstream NMF algorithm were those most responsible for driving the formation of distinct identities, we systematically evaluated centroid distance thresholds between TNBC sample clusters identified by DBSCAN and selected the highest possible centroid distance to prioritize genes contributing most strongly to inter-cluster separation, while ensuring a sufficient number of genes remained to provide adequate representation for NMF decomposition. The final selected parameters for CCLE cell lines and TCGA patient samples are listed below.

For CCLE cell lines, genes with maximum expression values among TNBC cell lines lower than 3 were filtered out. Genes with expression ranges among TNBC cell lines lower than 3 were filtered out. Genes were selected if there was at least one cluster pair containing more than 3 TNBC cell lines in each cluster with cluster centroid distance above 3 and cluster separation above 1. Representative genes that substantially contribute to the differentiation of distinct identities are shown in Figure S23 where their expression patterns clearly separate TNBC cell lines into distinct groups.

For TCGA patient samples, a first round of DBSCAN was run using all breast cancer samples. Then genes were selected for further filtering if cluster pair distances were more than 4 (higher than 95% of the quantile) and there were no less than 10 basal-like breast cancer tumor samples in each cluster of the pair. 753 genes were selected after the first-round filtering.

These 753 genes were sent for another round of DBSCAN running. In this round, only basal-like breast cancer tumor samples were used. Then genes were selected with cluster pairs that had centroid distance more than 4, and there were at least 8 basal-like breast cancer tumor samples in each cluster of the pair.

This process yielded 524 salient genes (including MYC) for TNBC cell lines and 489 salient genes (including MYC) for basal-like breast tumors. All salient genes selected in the CCLE and TCGA models are shown separately in the Document S3 and Document S4.

##### Non-negative matrix factorization (NMF) and identity mapping

The selected genes were used to perform NMF (Python ccal^68^ package v0.9.4) on both TNBC cell lines and tumor samples (with k = 4–9). The elbow method determined the optimal k, after which hierarchical clustering (HC) was applied to the H and W matrices to group TNBC samples and genes, respectively. HC on the H matrix grouped TNBC samples into transcriptionally distinct identities, while the W matrix provided gene weights for each identity. TNBC CCLE cell line NMF H matrix results for k values ranging from 5 to 8 were provided in Figure S24.

To annotate the biological relevance of each identity, top-expressed genes were identified, and an in-depth literature review was conducted (Tables S1 and S4). Common pathways or gene expression programs explaining these genes were assigned to define the biological characteristics of each identity. Using the ccal^65^ package, transcriptomic identity maps were generated for TNBC cell lines and basal-like breast tumor samples.

##### Multi-omics evidence supporting identity biology

To validate the biological relevance of each identity, MsigDB hallmark gene sets were analyzed to identify genes supporting specific TNBC cell line subpopulations. For both hallmark and selected genes, expression levels between subpopulations and other samples were compared using Wilcoxon rank-sum tests, with adjusted p-values.

Essential genes for each subpopulation were identified using the CCLE CRISPR gene effect dataset. Gene effect scores were compared using Wilcoxon rank-sum tests.

##### Kaplan-meier survival analysis

Kaplan-Meier survival curves were constructed for each identity of TCGA basal-like breast cancer patients using the KaplanMeierFitter() function from the Python lifelines package^67^ (v0.27.4). Survival comparisons between identities were performed using the Python logrank_test() function, with the number of subjects at risk displayed using add_at_risk_counts() from lifelines.plotting.

##### Linking high-variance genes and subpopulation identities to drug response

To evaluate whether the selected high-variance genes effectively capture molecular drug responses, we collected publicly available RNA-seq and scRNA-seq datasets from TNBC cell lines and tumors under various drug treatments (Table S5). Differential gene expression (DGE) analysis was performed on these datasets. For scRNA-seq datasets, we focused on cancer cells to ensure tumor-specific insights. Enrichment of the selected high-variance genes within the differentially expressed genes (DEGs) was assessed using chi-squared tests, with results provided in Table S5.

Additionally, we explored relationships between subpopulation identities and drug sensitivity by leveraging drug concentration-viability area under the curve (AUC) data from CTRP and GDSC. Pearson correlations were calculated between AUC values and the H matrix z-scores representing subpopulation-specific contributions. These analyses, conducted using pearsonr() from the scipy package^69^ (v1.8.0), highlight the degree to which certain identities influence drug response. Linear regression lines were computed with stats.linregress(). For drugs/compounds in these databases, AUC levels between subpopulations and other samples were compared using Wilcoxon rank-sum tests.

#### Treating a TNBC organoid model for multiomics analysis

For drug treatment experiments, organoids were dissociated into single cells, counted, and 10,000 cells were resuspended in 10 μL of 70% Matrigel. These were seeded into 96-well plates as domes, solidified for 30 minutes, and overlaid with 100 μL of complete media. Organoids were allowed to form over 78 hours before drug treatment. Organoids were treated with various concentrations of JQ1 (a BRD4 inhibitor) and MS177 (an EZH2 degrader) for 48 hours, with DMSO serving as the diluent control, in triplicate. Viability was assessed using the CellTiter-Glo 3D Cell Viability Assay (Promega), which measures ATP levels as an indicator of viable cells. Luminescence was recorded using an Asteran microplate reader. For single-dose experiments, JQ1 and MS177 were administered at final concentrations of 500 nM and 250 nM, respectively, for 24 hours, after which organoids were harvested for downstream analyses.

Post-treatment, organoids were dissociated into single-cell suspensions using Accutase (Sigma) and gentle pipetting. Cells were filtered through a 40-μm strainer, washed with PBS, and counted with a Countess II Automated Cell Counter (ThermoFisher) to ensure >85% viability. Approximately 10,000 viable cells per condition were processed using the Chromium Single Cell Multiome ATAC + Gene Expression platform (10X Genomics). GEM generation, cDNA synthesis, and ATAC library preparation were conducted according to the manufacturer’s protocols, enabling downstream multiomic analysis of transcriptomic and chromatin accessibility changes.

#### Organoid 10X multiome library generation and sequencing

To characterize the transcriptional and chromatin accessibility landscapes of TNBC organoids treated with JQ1 and MS177, we performed multiomics single-cell sequencing using the Chromium Single Cell Multiome ATAC + Gene Expression platform (10X Genomics). Organoids were harvested after 24 hours of drug treatment to capture cellular state changes while minimizing artifacts associated with extended culture. Single-cell suspensions were prepared following the 10X Genomics protocol. The cell concentration and quality were assessed using a Countess II Automated Cell Counter (ThermoFisher). Approximately 10,000 cells per sample were loaded into the Chromium Controller to partition individual cells into Gel Bead-In Emulsions (GEMs), enabling simultaneous profiling of RNA transcripts and chromatin accessibility within the same cells. Library preparation followed the manufacturer’s guidelines, including reverse transcription for gene expression, transposition for chromatin accessibility, and amplification of both cDNA and ATAC libraries. Libraries were quantified using a Qubit dsDNA High Sensitivity Assay (ThermoFisher) and assessed for fragment size distribution using an Agilent 4200 TapeStation. Sequencing was performed using the NextSeq2000 (Illumina) with paired-end reads to ensure high-resolution data.

#### Multiomics data analysis

We conducted comprehensive analyses integrating scRNA-seq and single-cell multiome data to investigate transcriptional and chromatin accessibility changes in TNBC cell lines, organoids, and patient samples.

##### scRNA-seq analysis

For scRNA-seq data from TNBC cell lines under drug treatment, we focused on HCC1143 treated with paclitaxel for 24 hours, MDAMB468 treated with 5-FU for 50 days, and MDAMB468 treated with Afatinib for 3 days. For scRNA-seq datasets of TNBC patients treated with anti-PD1 therapy, we included samples with sufficient cancer cell counts (n=13) (IDs: BIOKEY_1 (B1), BIOKEY_2 (B2), BIOKEY_9 (B9), BIOKEY_10 (B10), BIOKEY_11 (B11), BIOKEY_14 (B14), BIOKEY_15 (B15), BIOKEY_16 (B16), BIOKEY_19 (B19), BIOKEY_26 (B26), BIOKEY_31 (B31), BIOKEY_35 (B35), BIOKEY_41 (B41)). For patient scRNA-seq data, cancer cells were annotated in the processed data provided by the authors from the source publication.^58^ Analyses were performed using R (v4.2.1/v4.3.1) and the Seurat package (v4.4.0). Data were normalized using the SCTransform() function, and differentially expressed genes (DEGs) were identified with the FindMarkers() function (Wilcoxon rank-sum test). Gene set expression scores were computed using the AddModuleScore() function. UMAP visualizations were generated using DimPlot(), and pie charts, volcano plots, box plots, and violin plots were created with ggplot2 (v3.4.4).

To map single cells to gene expression identities from the CCLE bulk data model, we employed the nnls package (v1.5). Identity enrichment and depletion analyses were performed using GSEA (clusterProfiler v4.6.2) and visualized with enrichplot (v1.18.4). To assess identity expansion or shrinkage, we calculated cell counts for each identity pre- and post-treatment, performing chi-squared tests using chisq.test(). Residual scores from these tests quantified the magnitude of expansion or shrinkage. For cancer cell clusters contributing to identity changes, chi-squared tests were used to assess cluster-specific contributions to the most expanded identity.

##### Single-cell multiome analysis

Raw FASTQ files from single-cell multiome sequencing were aligned to the GRCh38 genome using Cell Ranger ARC (v2.0.1). Downstream analysis was performed in R (v4.2.1/v4.3.1) with the Seurat (v4.4.0) and Signac (v1.10.0) packages to process RNA and ATAC data simultaneously. Datasets for 3 treatment conditions were merged together following the standard pipeline outlined in the Signac tutorial. To ensure high-quality data, single-cell barcodes meeting specific thresholds were retained, including ATAC read counts between 1,000 and 70,000, RNA read counts between 1,000 and 25,000, detection of more than 500 genes per cell, nucleosome signal less than 2, TSS.enrichment more than 1, and mitochondrial transcript proportions below 20%. Doublets were identified and removed using DoubletFinder (v2.0.4).

To validate that all cells within the organoid samples were cancerous, infercnv (v1.21.0), SingleR (v2.4.1) and celldex (v1.12.0) were employed. Reference cells, including T cells, NK cells, macrophages, monocytes, and B cells, from the GEO study GSE176078 were used for infercnv, ensuring that non-cancerous cells were excluded from further analysis. RNA data were normalized using the SCTransform() function in Seurat, with results stored in the “SCT” assay. For ATAC data, The function CallPeaks() was used for peak calling, and the resulting peaks were saved in the “peaks” assay. Gene activity scores, representing transcriptional activity across promoter and gene body regions, were calculated with GeneActivity() from Signac and stored in the “gene_activity” assay.

Transcription factor analysis included identifying chromatin binding motifs with JASPAR2020 (v0.99.10) and quantifying chromatin accessibility associated with these motifs using ChromVAR (v1.20.2). Differential motif accessibility, gene expression changes, and ATAC peaks were identified using the FindMarkers() function in Seurat for each drug treatment condition. Visualization of the data included violin plots created with the VlnPlot() function, while heatmaps, scatter plots, stacked bar plots, and pie charts were generated using ggplot2 (v3.4.4). Chromatin accessibility in genomic regions was visualized with the CoveragePlot() function from Signac.

To systematically identify transcription factors involved in CCLE F3 identity changes after MS177 and JQ1 treatments, upregulated genes—including those in the NF-κB pathway—were input into the Enrichr database^84^^,^^85^^,^^86^ (https://maayanlab.cloud/Enrichr/). Potential transcription factors were identified under the “Transcription” section of the results. Gene targets of transcription factors were curated from ChIP-seq databases such as ChEA 2022, ENCODE, and ChEA Consensus TFs from ChIP-X. The intersection of these targets with genes in the MsigDB TNFA signaling hallmark gene set was taken for further analysis.

To evaluate the relationship between transcription factor motif chromatin accessibility and target gene expression, linear models were constructed using the lm() function in R. R-squared values were extracted to quantify the correlation between TF binding motif accessibility and the expression of NF-κB pathway target genes. Differences in R-squared values between treated (JQ1 or MS177) and untreated conditions were calculated and visualized in scatterplots. This analysis highlighted transcription factors whose chromatin accessibility and target gene expression were most affected by drug treatments, revealing molecular drivers of adaptive responses.

For the differential ATAC peaks after drug treatment in the organoid as a whole and in each of the two distinct clusters within the organoid, to link ATAC peaks with genes, the package ChIPseeker(v1.34.1) was employed. Genomic positions for mRNA transcripts were acquired from package TxDb.Hsapiens.UCSC.hg38.knownGene (v3.16.0). Human genome annotation data was acquired from package org.Hs.eg.db (v3.16.0). GRanges objects were created using the package GenomicRange (v1.50.2). The results are stored in Tables S3 and S6.

This integrated analysis pipeline provided a comprehensive view of transcriptional and chromatin accessibility landscapes, enabling the identification of key transcriptional regulators and adaptive mechanisms in TNBC organoid models treated with epigenetic drugs.

### Quantification and statistical analysis

R (v4.2.1/v4.3.1) and Python (v.3.8.12) were used to perform all the statistical analyses. Wilcoxon rank-sum tests were performed to compare gene expression, CRISPR gene dependency score, identity score, gene set expression score, and chromatin accessibility between two groups. Chi-squared tests were performed to evaluate the enrichment or depletion of identities upon drug treatments. Log-rank tests were performed to evaluate patient survival between different identities. Two-sided t-tests were performed to assess the statistical significance of the regression coefficient. Likelihood ratio tests were performed to evaluate chromatin accessibility between two groups. Pearson correlation was computed for correlation analyses.

## Supplemental information

Document S1. Figures S1–S24

Table S1. Results from the literature search supporting the biological meanings of states in the CCLE model, related to Figure 1 and Figure 2 (A) Gene literature annotations for CCLE F0 identity. (B) Gene literature annotations for CCLE F1 identity. (C) Gene literature annotations for CCLE F2 identity. (D) Gene literature annotations for CCLE F3 identity. (E) Gene literature annotations for CCLE F5 identity. (F) Gene literature annotations for CCLE F6 identity.

Table S2. TNBC tumor cancer cell cluster drug response analysis, related to Figure 4 (A) Cluster drug response signatures across TNBC tumor samples.

Table S3. Differential peaks annotated to nearby genes after MS177 treatment in the TNBC organoid model, related to Figure 5 (A) Differential peaks after MS177 on the bulk level. (B) Differential peaks after MS177 in the epithelial-like cluster of the organoid. (C) Differential peaks after MS177 in the stem-cell-like cluster of the organoid.

Table S4. Results from the literature search supporting the biological meanings of states in the TCGA model, related to Figure 1 (A) Gene literature annotations for TCGA F0 identity. (B) Gene literature annotations for TCGA F1 identity. (C) Gene literature annotations for TCGA F2 identity. (D) Gene literature annotations for TCGA F3 identity. (E) Gene literature annotations for TCGA F5 identity.

Table S5. Enrichment analysis results of CCLE model identity-defining genes among differentially expressed genes following drug treatment across all TNBC samples, related to Figures 3 and 4 (A) Enrichment analysis results of CCLE model identity-defining genes among differentially expressed genes following drug treatment across all TNBC samples.

Table S6. Differential peaks annotated to nearby genes after JQ1 treatment in the TNBC organoid model, related to Figure 5 (A) Differential peaks after JQ1 on the bulk level. (B) Differential peaks after JQ1 in the epithelial-like cluster of the organoid. (C) Differential peaks after JQ1 in the stem-cell-like cluster of the organoid.

Data S1. All 523 selected salient genes in the CCLE TNBC model, related to Figures 1 and 2 For each representative gene, its log-transformed TPM expression and MYC expression are shown on scatterplots across all CCLE pan-cancer cell lines. Each dot represents a CCLE cell line, with TNBC cell lines highlighted in red. Colored clusters (yellow, green, orange, and so forth) indicate DBSCAN-identified cell line groups.

Data S2. All 488 selected salient genes in the TCGA TNBC model, related to Figure 1 For each representative gene, its log-transformed TPM expression and MYC expression are shown on scatterplots across all TCGA breast cancer samples. Each dot represents a TCGA breast cancer sample, with basal-like breast cancer samples highlighted in red.
