# Supplementary material for: Decoding drug-responsive cell subpopulations in triple-negative breast cancer using single-cell multiomics
Source: iScience. 2026 Mar 21;29(5):115445. doi: 10.1016/j.isci.2026.115445 (PMC13138057; doi:10.1016/j.isci.2026.115445)
Supplement: Document S1. Figures S1–S24 [file mmc1.pdf]

iScience, Volume *29*

Supplemental information

Decoding drug-responsive cell subpopulations in triple-negative breast cancer using single-cell multiomics

Yue Wang, Santiago Haase, Austin Whitman, Adriana S. Beltran, Philip M. Spanheimer, and Elizabeth Brunk

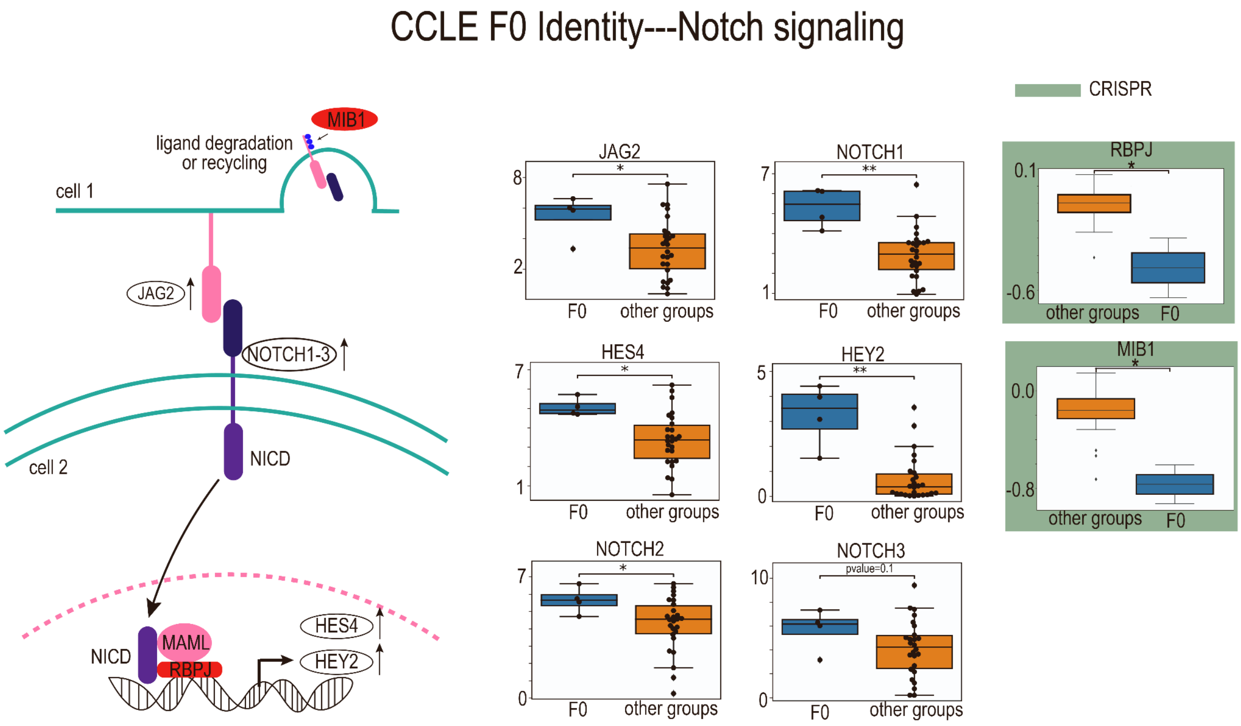

cartoon figure adapted from Zhao F, He Y, Zhao Z, et al. The Notch signaling-regulated angiogenesis in rheumatoid arthritis: pathogenic mechanisms and therapeutic potentials. Front Immunol. 2023;14:1272133. Published 2023 Oct 26. doi:10.3389/fimmu.2023.1272133

Figure S1. Cartoon illustration demonstrating the involvement of CCLE F0 identity-defining genes in Notch signaling, Related to Figure 1 and Figure 2

NICD: Notch intracellular domain, MAML: mastermind-like proteins. Boxplots show the gene expressions and CRISPR scores. Boxplot center line indicates median; box bounds represent the interquartile range (IQR); whiskers extend to 1.5×IQR. *$p<0.05$, **$p<0.01$ by Wilcoxon rank-sum test

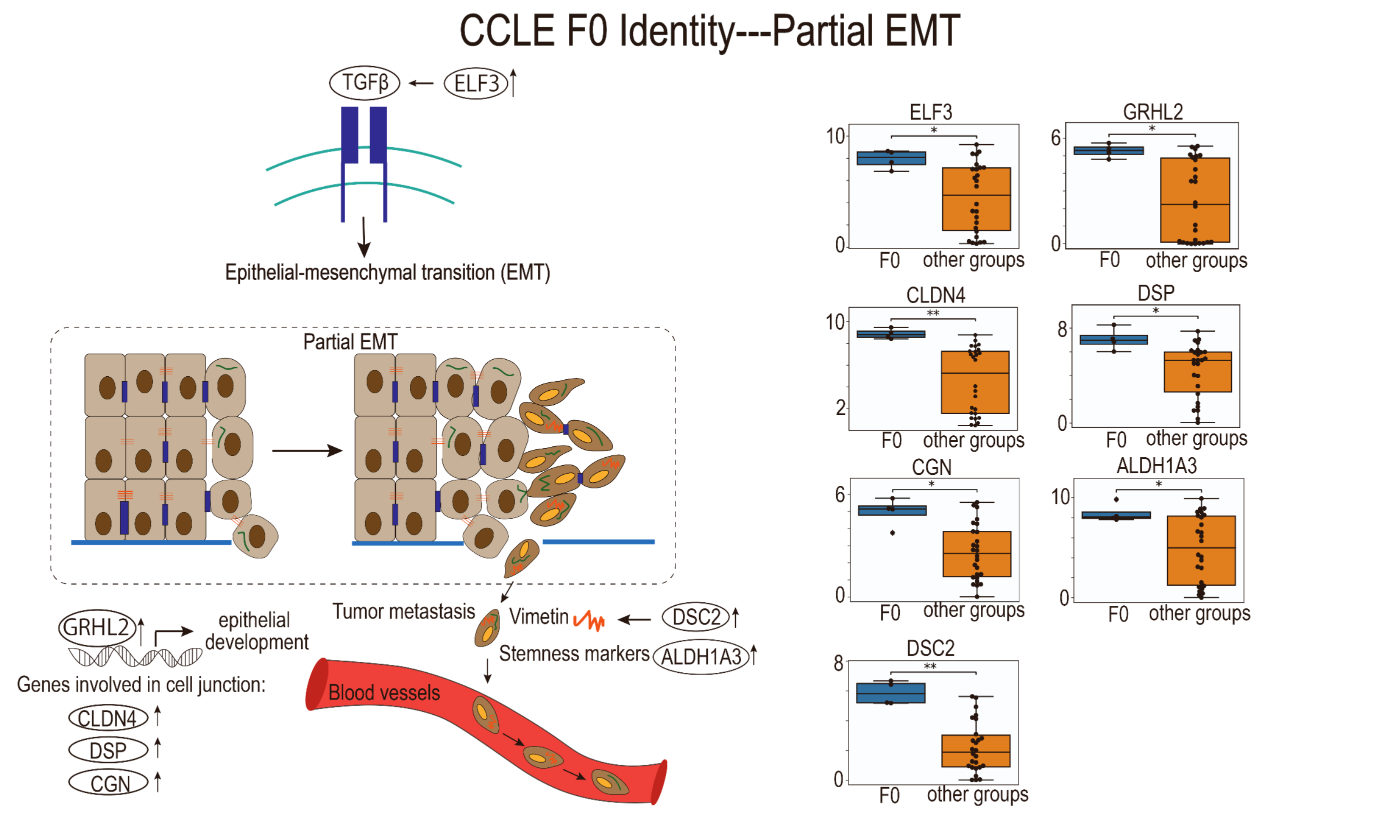

cartoon figure adapted from Sheng L, Zhuang S. New Insights Into the Role and Mechanism of Partial Epithelial-Mesenchymal Transition in Kidney Fibrosis. Front Physiol. 2020;11:569322. Published 2020 Sep 15. doi:10.3389/fphys.2020.569322

Figure S2. Cartoon illustration demonstrating the involvement of CCLE F0 identity-defining genes in partial-EMT, Related to Figure 1 and Figure 2 Boxplots show the gene expressions of salient genes. Boxplot center line indicates median; box bounds represent the interquartile range (IQR); whiskers extend to 1.5×IQR. $^*p<0.05$, $^{**}p<0.01$ by Wilcoxon rank-sum test

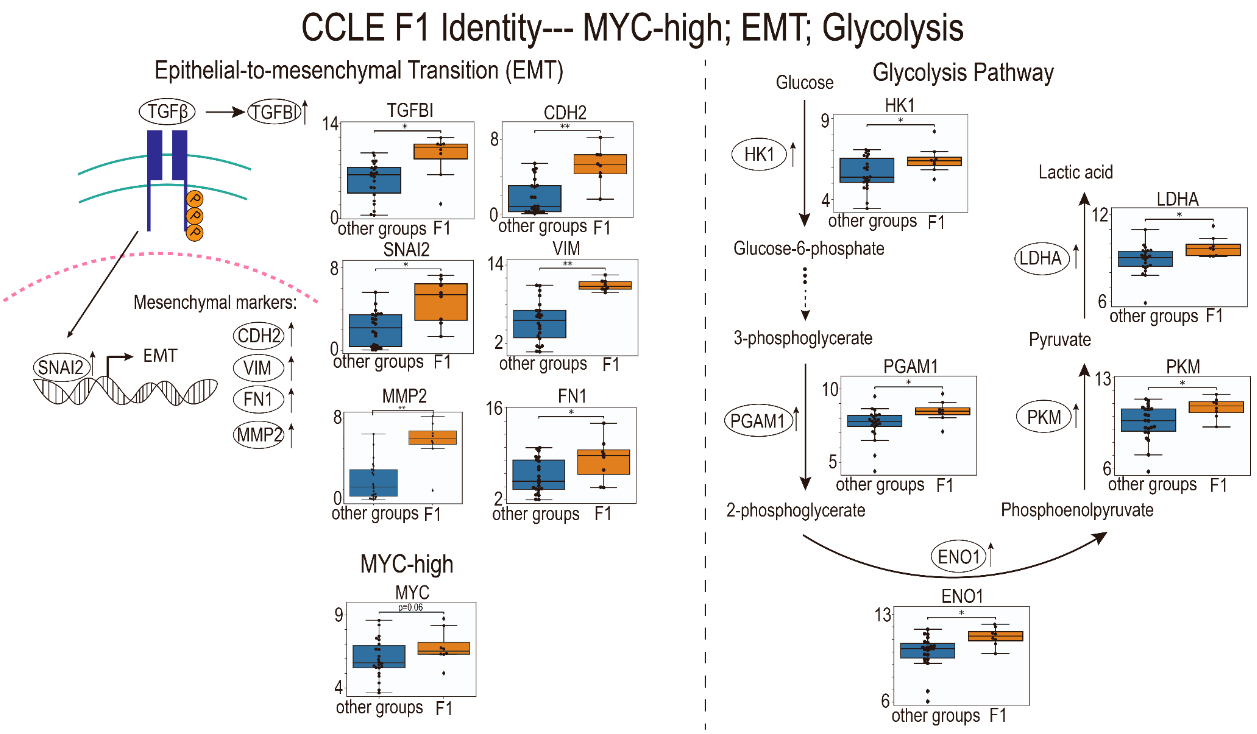

Figure S3. Cartoon illustration demonstrating the involvement of CCLE F1 identity-defining genes in MYC-high, EMT and glycolysis, Related to Figure 1 and Figure 2 Boxplots show the gene expressions of salient genes. Boxplot center line indicates median; box bounds represent the interquartile range (IQR); whiskers extend to 1.5×IQR. *p<0.05, **p<0.01 by Wilcoxon rank-sum test

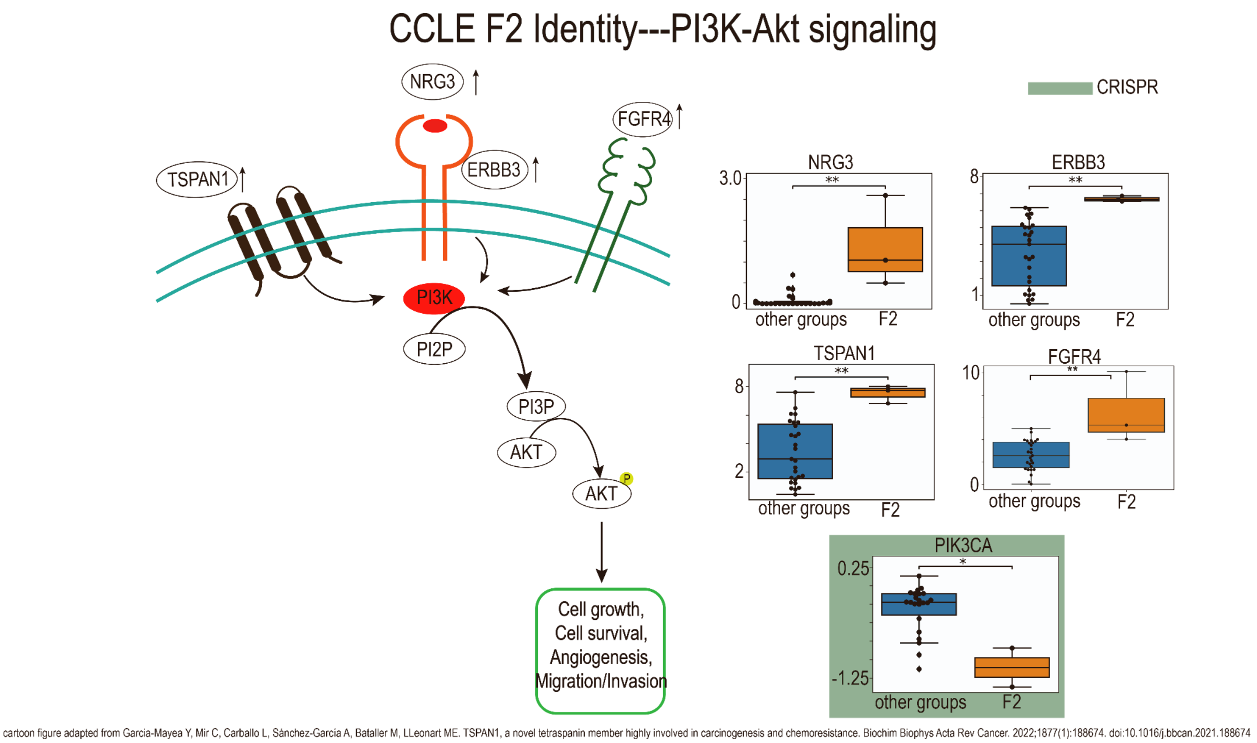

Figure S4. Cartoon illustration demonstrating the involvement of CCLE F2 identity-defining genes in PI3K-Akt signaling, Related to Figure 1 and Figure 2 Boxplots show the gene expressions and CRISPR scores. Boxplot center line indicates median; box bounds represent the interquartile range (IQR); whiskers extend to 1.5×IQR. *p<0.05, **p<0.01 by Wilcoxon rank-sum test

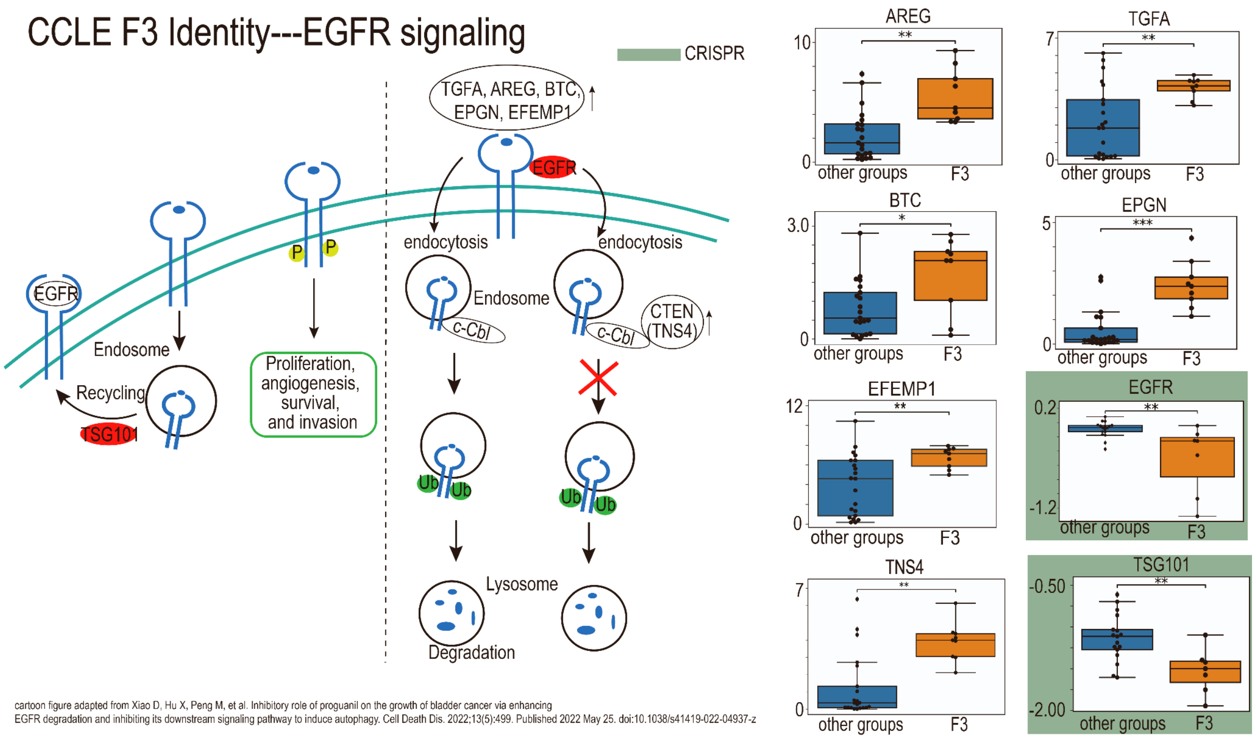

Figure S5. Cartoon illustration demonstrating the involvement of CCLE F3 identity-defining genes in EGFR signaling, Related to Figure 1 and Figure 2 c-Cbl: Casitas B lineage lymphoma, an E3 ubiquitin ligase. Boxplots show the gene expressions and CRISPR scores. Boxplot center line indicates median; box bounds represent the interquartile range (IQR); whiskers extend to 1.5×IQR. *p<0.05, **p<0.01 by Wilcoxon rank-sum test

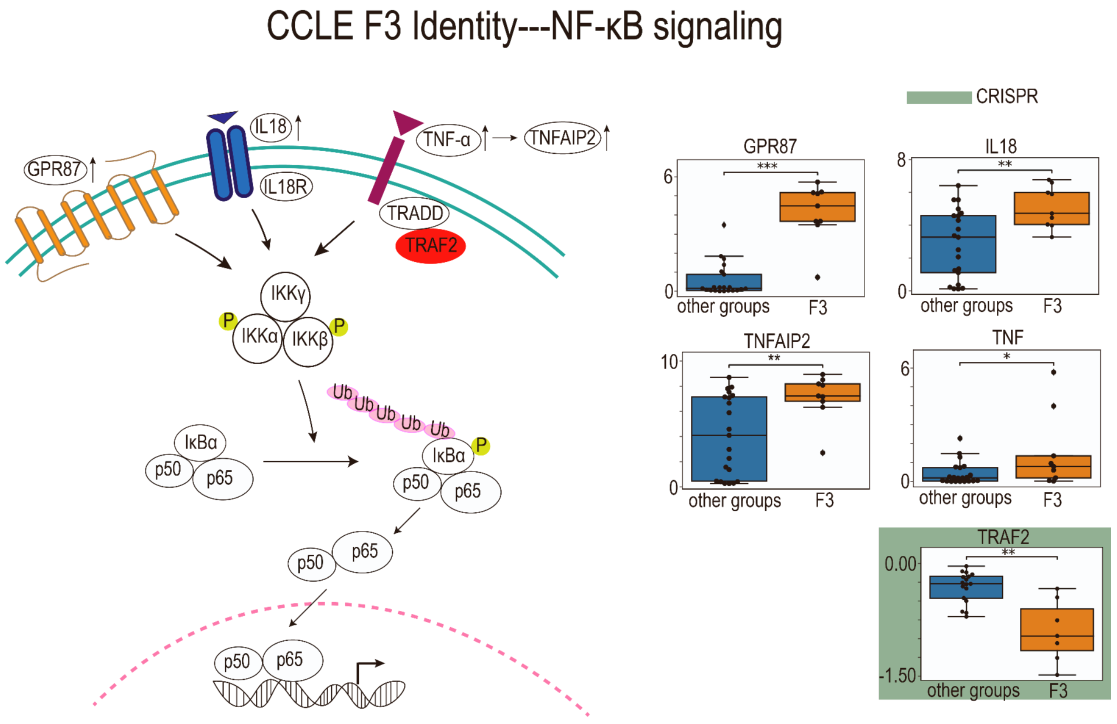

cartoon figure adapted from Peng C, Ouyang Y, Lu N, Li N. The NF-κB Signaling Pathway, the Microbiota, and Gastrointestinal Tumorigenesis: Recent Advances. Front Immunol. 2020;11:1387. Published 2020 Jun 30. doi:10.3389/fimmu.2020.01387

Figure S6. Cartoon illustration demonstrating the involvement of CCLE F3 identity-defining genes in NF-κB signaling, Related to Figure 1 and Figure 2 Boxplots show the gene expressions and CRISPR scores. Boxplot center line indicates median; box bounds represent the interquartile range (IQR); whiskers extend to 1.5×IQR. *p<0.05, **p<0.01 by Wilcoxon rank-sum test

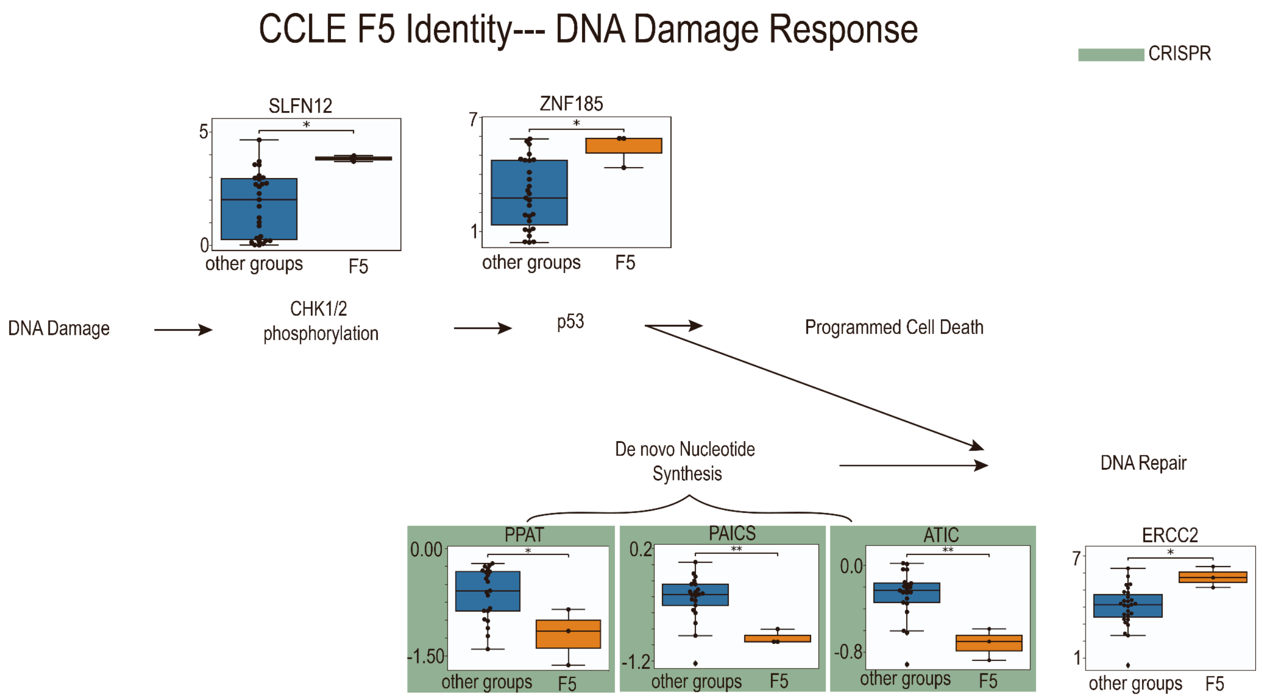

Figure S7. Cartoon illustration demonstrating how identity-defining genes point to DNA damage response in CCLE F5 identity, Related to Figure 1 and Figure 2 Boxplots show the gene expressions and CRISPR scores. Boxplot center line indicates median; box bounds represent the interquartile range (IQR); whiskers extend to 1.5×IQR. *p<0.05, **p<0.01 by Wilcoxon rank-sum test

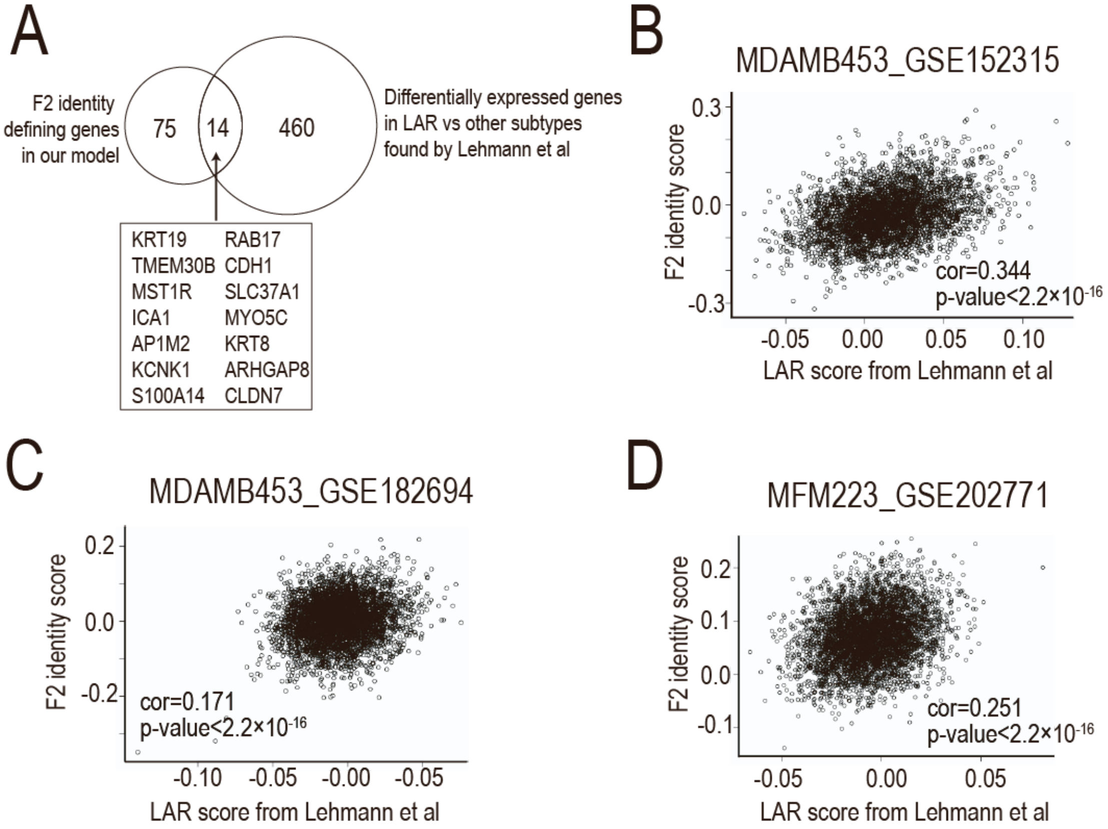

Figure S8. Comparison between CCLE F2-identity-defining genes and LAR-defining genes, Related to Figure 1 and Figure 2

A. Venn diagram shows the overlap between CCLE F2-identity-defining genes and LAR-defining genes. **B.** The correlation between F2-identity-defining gene expression and LAR-defining gene expression across single cells in cell line MDAMB453 from the GEO dataset GSE152315. **C.** The correlation between F2-identity-defining gene expression and LAR-defining gene expression across single cells in cell line MDAMB453 from the GEO dataset GSE182694. **D.** The correlation between F2-identity-defining gene expression and LAR-defining gene expression across single cells in cell line MFM223 from the GEO dataset GSE202771. Pearson correlation was computed between F2 identity score and LAR score.

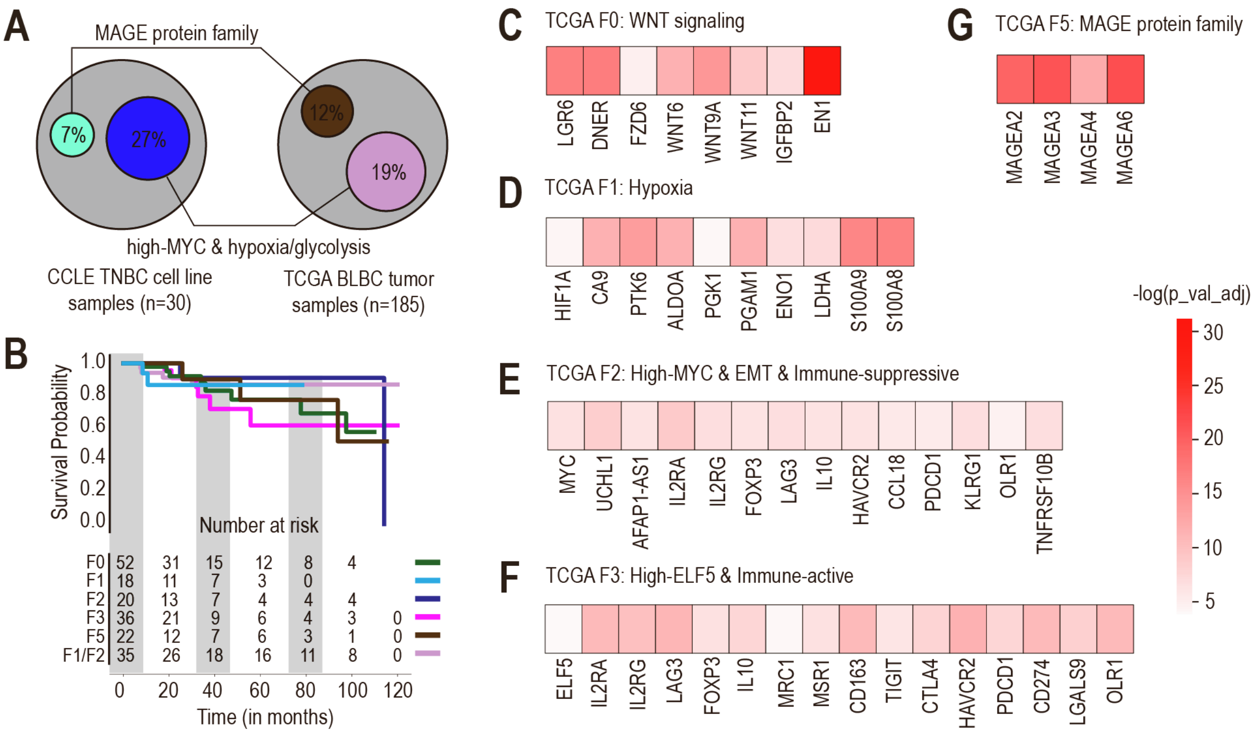

Figure S9. TCGA sample identity characterization, Related to Figure 1

A. Common gene expression identities between cell line and tumor sample models. **B**. Kaplan-Meier survival curves for TCGA basal-like breast tumor samples, stratified by identity. The risk table indicates the number of patients at risk over time for each group, including the F1/F2 Mixture identity, denoted as F1/F2. Log-rank tests were performed to evaluate survival between different groups. **C-E & G**. Gene expression evidence supporting the biology of identities F0, F1, F2 and F5. The heatmaps present the adjusted p-values from Wilcoxon rank-sum tests comparing gene expression levels in tumor samples belonging to a specific identity versus all others. Genes representative of each identity show significantly higher expressions within the associated tumor samples. **F.** Gene expression evidence supporting the biology of the F3 identity. The heatmap presents the adjusted p-values from Wilcoxon rank-sum tests comparing gene expression levels in tumor samples belonging to the F3 identity versus all others. Gene ELF5 has higher expression in samples belonging to the F3 identity. The rest of genes, which are immune-suppressive genes, have lower expressions in samples belonging to the F3 identity.

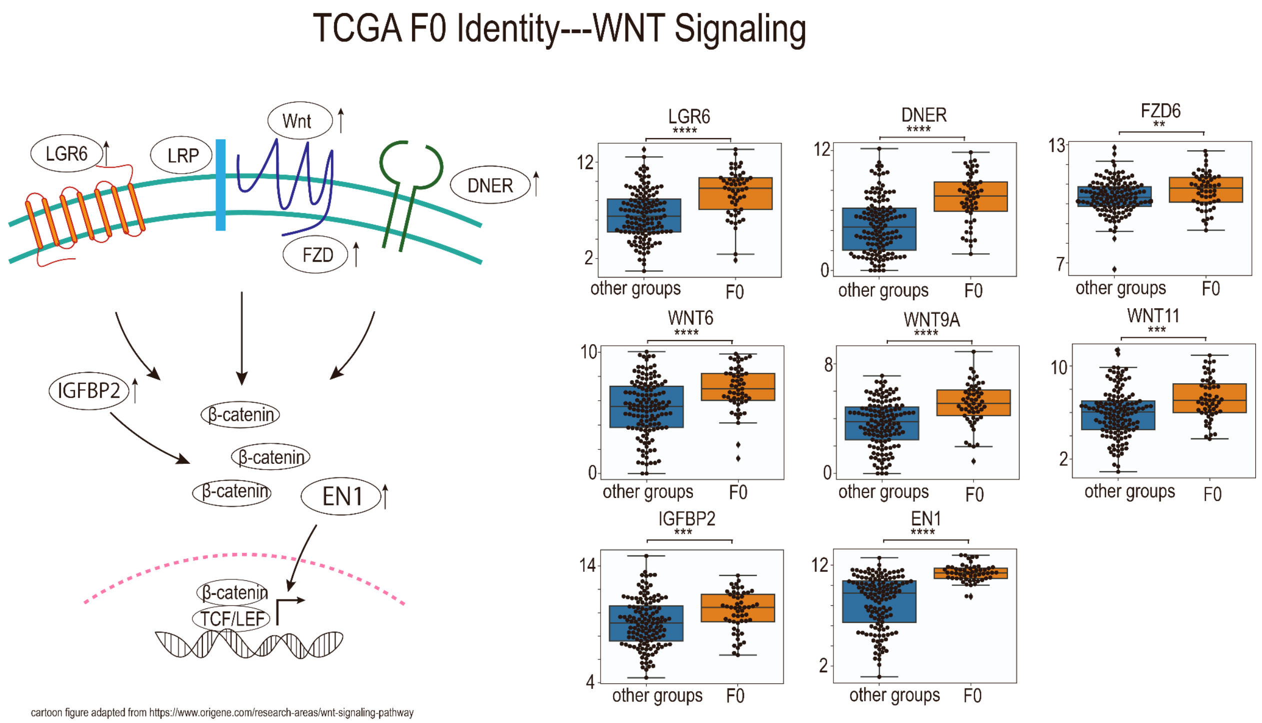

Figure S10. Cartoon illustration demonstrating how identity-defining genes point to WNT signaling in TCGA F0 identity, Related to Figure 1

LRP: low-density lipoprotein receptor-related protein, TCF/LEF: T-cell factor/lymphoid enhancer factor. Boxplots show the gene expressions of salient genes. Boxplot center line indicates median; box bounds represent the interquartile range (IQR); whiskers extend to 1.5×IQR. *p<0.05, **p<0.01 by Wilcoxon rank-sum test

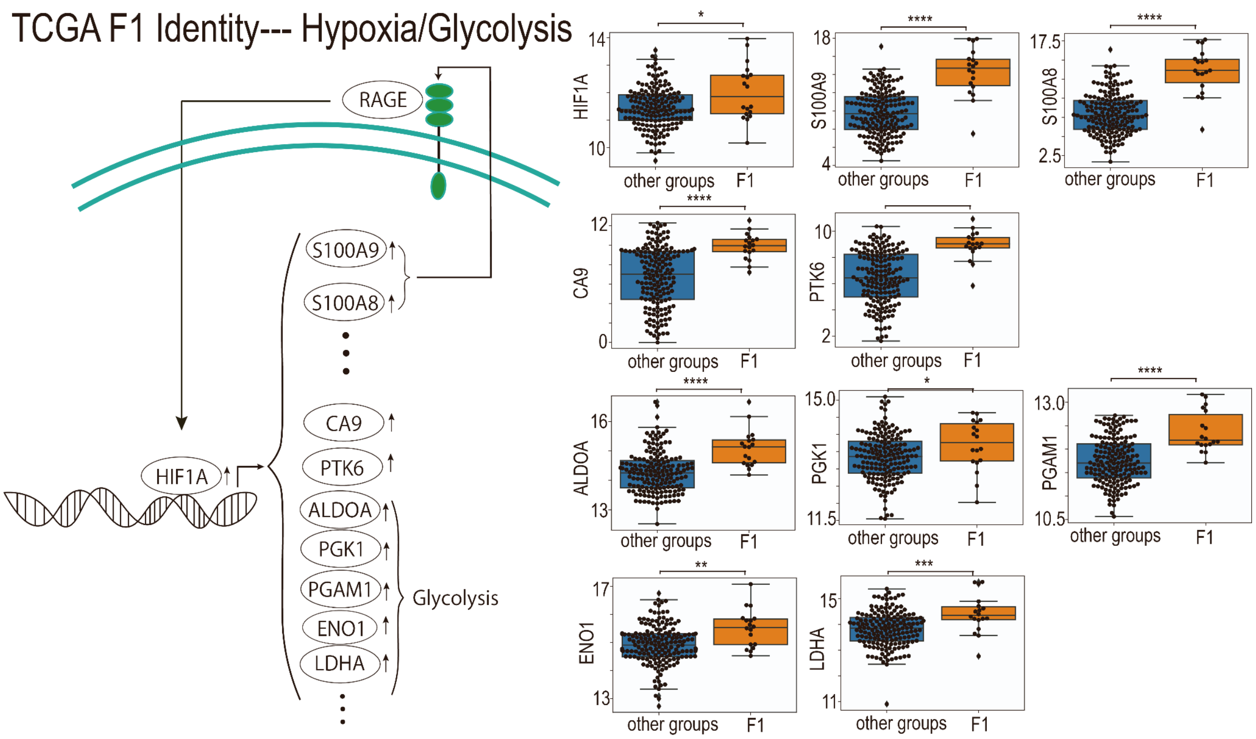

Figure S11. Cartoon illustration demonstrating how identity-defining genes point to hypoxia/glycolysis in TCGA F1 identity, Related to Figure 1

RAGE: receptor for advanced glycation end products. Boxplots show the gene expressions of salient genes. Boxplot center line indicates median; box bounds represent the interquartile range (IQR); whiskers extend to 1.5×IQR. *p<0.05, **p<0.01 by Wilcoxon rank-sum test

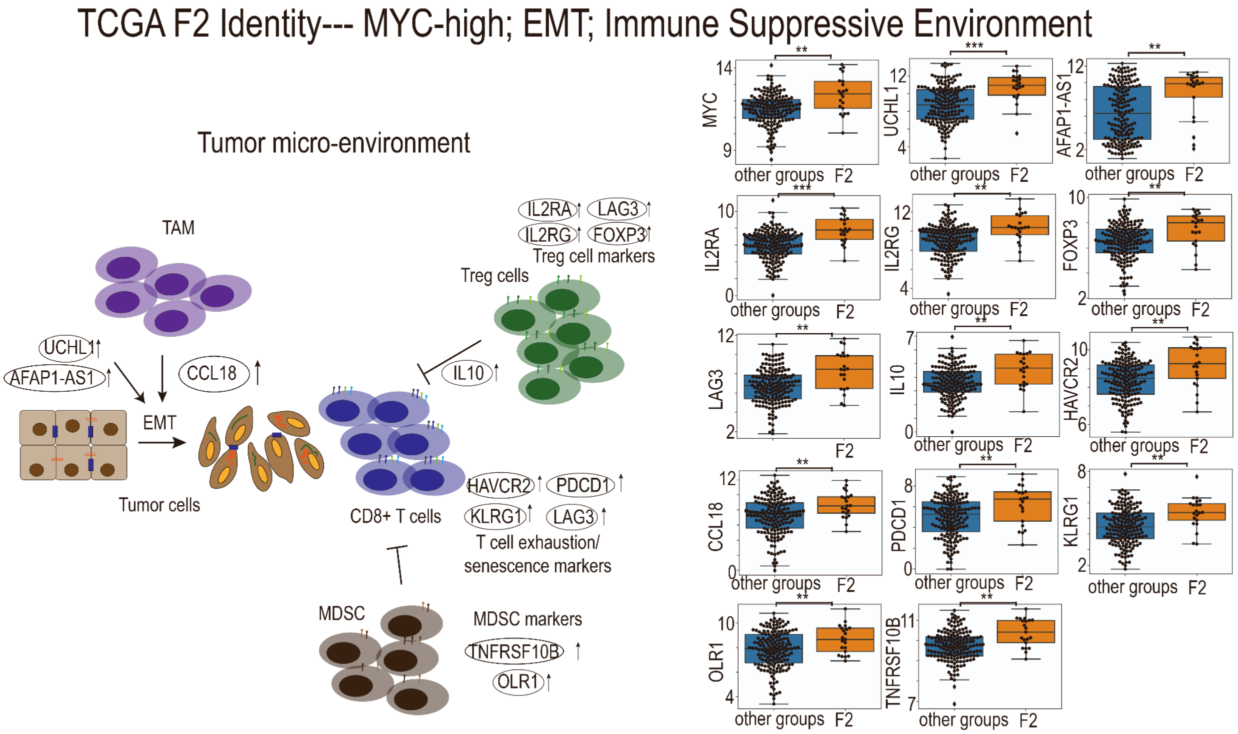

Figure S12. Cartoon illustration demonstrating the involvement of TCGA F2 identity-defining genes in "MYC"-high, EMT and immune suppressive environment, Related to Figure 1

TAM: tumor-associated macrophage, MDSC: myeloid-derived suppressor cell. Boxplots show the gene expressions of salient genes. Boxplot center line indicates median; box bounds represent the interquartile range (IQR); whiskers extend to 1.5×IQR. *p<0.05, **p<0.01 by Wilcoxon rank-sum test

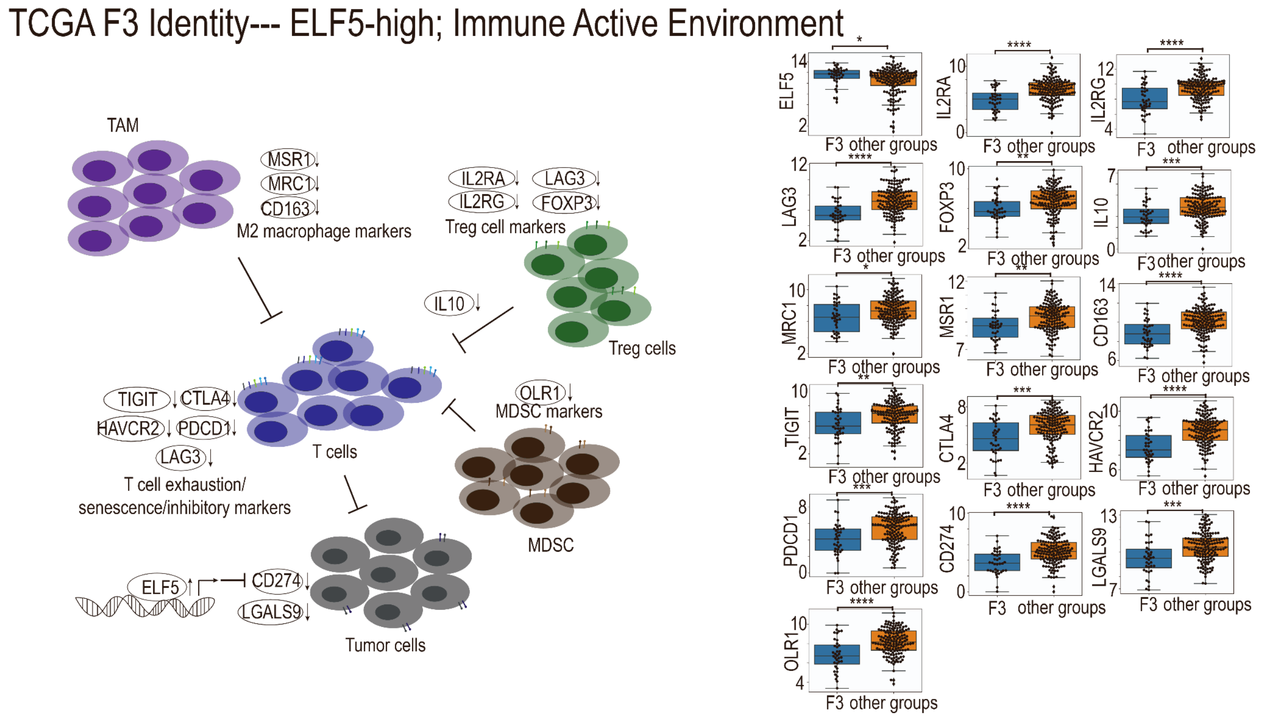

Figure S13. Cartoon illustration demonstrating the involvement of TCGA F3 identity-defining genes in "ELF5"-high and immune active environment, Related to Figure 1

TAM: tumor-associated macrophage, MDSC: myeloid-derived suppressor cell. Boxplots show the gene expressions of salient genes. Boxplot center line indicates median; box bounds represent the interquartile range (IQR); whiskers extend to 1.5×IQR. *p<0.05, **p<0.01 by Wilcoxon rank-sum test

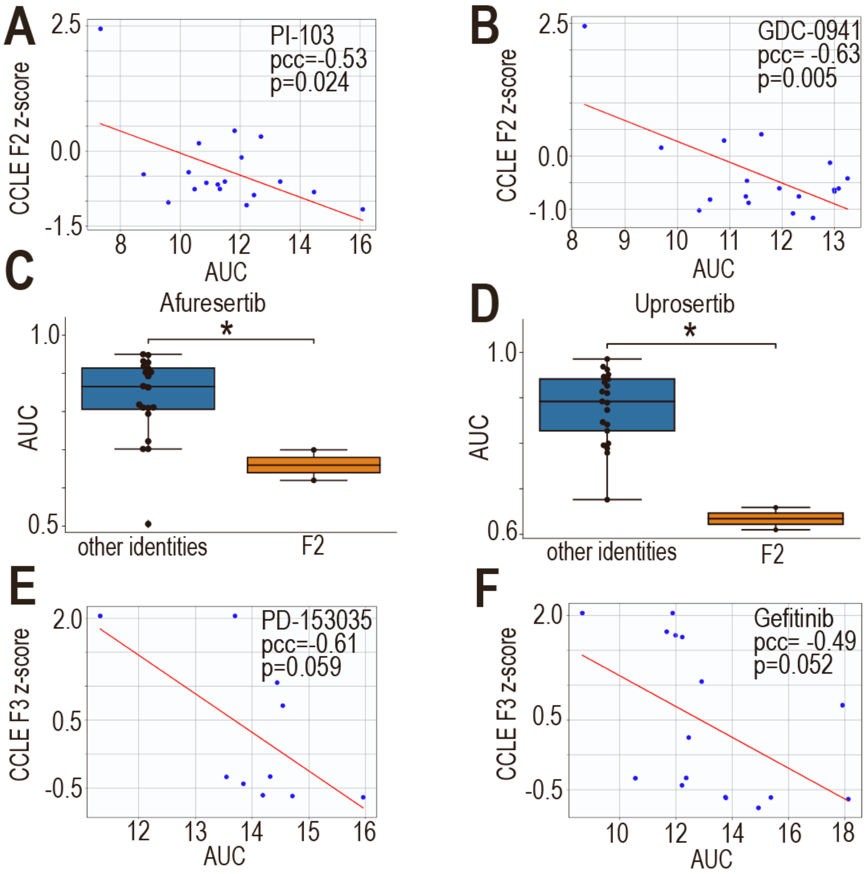

Figure S14. Extra evidence linking drug sensitivity to gene expression identities across TNBC cell lines, Related to Figure 2

A-B Drug efficacies for two PI3K inhibitors PI-103 (**A**) and GDC-0941 (**B**) are positively correlated with the CCLE F2 identity z-score across TNBC cell lines. The Pearson correlation coefficient and p-value are shown in the figures. **C-D** Cell lines belonging to F2 identity are more sensitive to afuresertib (**C**) and uprosertib (**D**), two Akt inhibitors. Boxplots show the AUC values of the two compounds. Boxplot center line indicates median; box bounds represent the interquartile range (IQR); whiskers extend to 1.5×IQR. **E-F** Drug efficacies for two EGFR inhibitors PD-153035 (**E**) and gefitinib (**F**) are positively correlated with CCLE F3 identity z-score across TNBC cell lines. The Pearson correlation coefficient and p-value are shown in the figures. *$p<0.05$, **$p<0.01$ by Wilcoxon rank-sum test; Pearson correlation was computed between identity expression score and drug/compound AUC value.

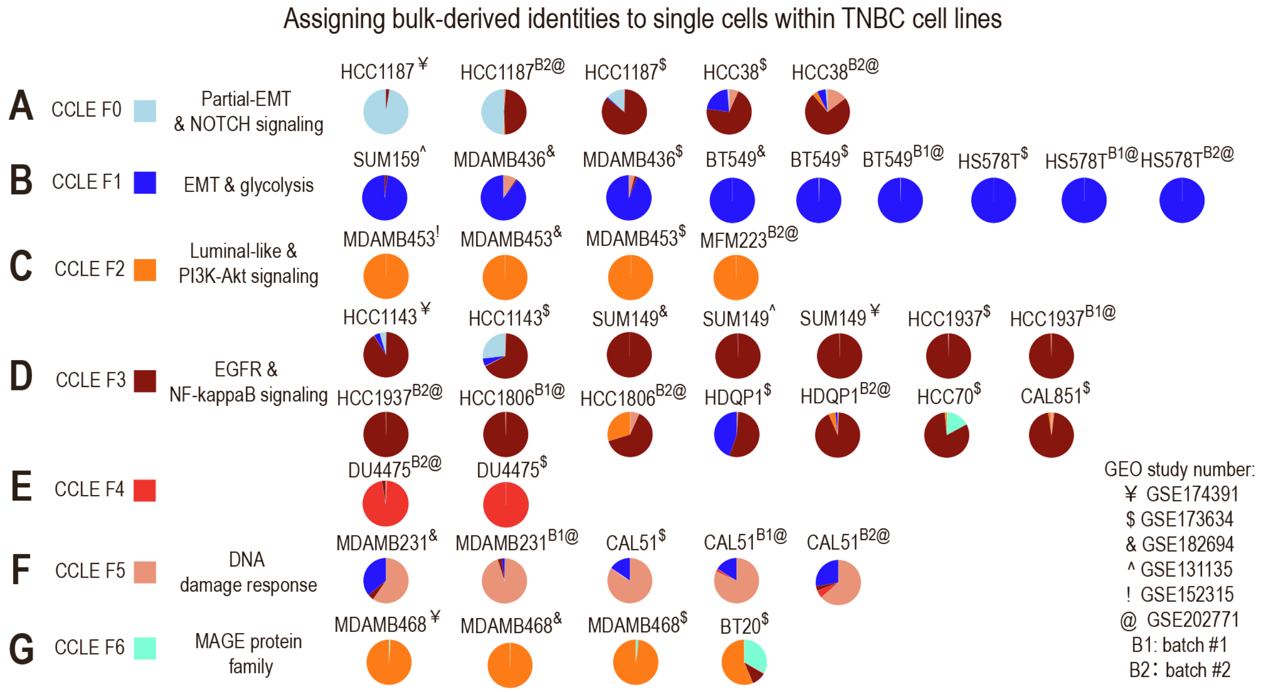

Figure S15. The result of mapping single cells in TNBC cell lines to bulk-derived identities, Related to Figure 3

Each pie chart shows the percentage of single cells belonging to each bulk-level identity in a specific TNBC cell line scRNA-seq dataset. The markers on the upper-right corner of cell line names indicate the GEO study number that the scRNA-seq data is from. Cell line scRNA-seq data is organized based on the bulk-identity it belongs to. The mapping result of single cells in TNBC cell lines from CCLE F0-F6 identities are shown in **A-G** correspondingly.

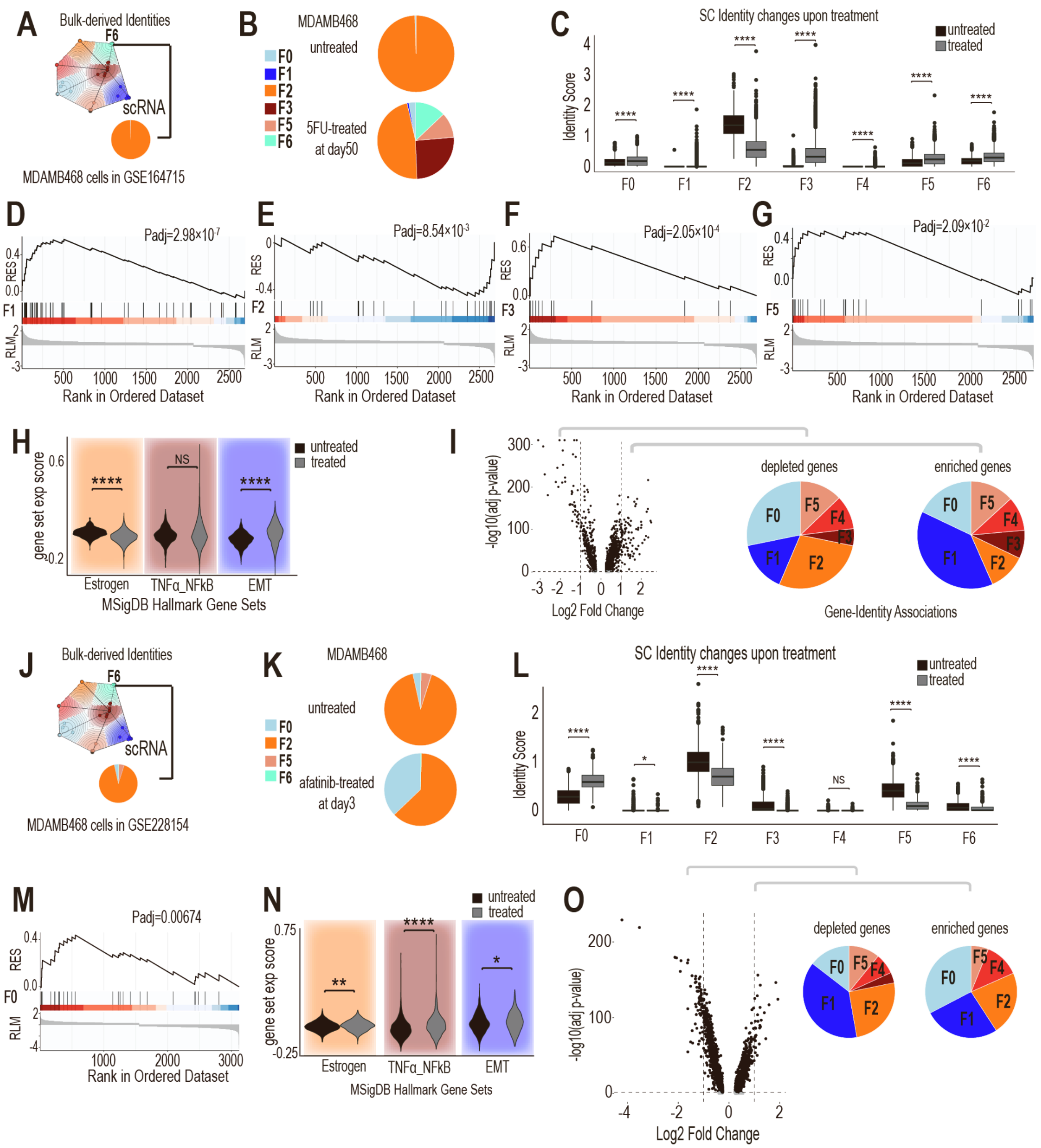

Figure S16. Single-cell identity changes in MDAMB468 after treatments by 5-FU and afatinib, Related to Figure 4

A. Single-cell identity composition of MDAMB468 from GSE164715, a TNBC cell line associated with the F6 identity in the CCLE model. **B**. Single-cell identity shifts in MDAMB468 from GSE164715 after 5-FU treatment. **C**. Identity scores for single cells in MDAMB468 from GSE164715 before and after 5-FU treatment. Boxplot center line indicates median; box bounds represent the interquartile range (IQR); whiskers extend to 1.5×IQR. **D-G** Gene Set Enrichment Analysis (GSEA) results for F1 (**D**), F2 (**E**), F3 (**F**), and F5 (**G**) gene sets after 5-FU treatment.

H. Expression scores for three MsigDB hallmark gene sets in MDAMB468 single cells from GSE164715 before and after 5-FU treatment. Violin plots show the distribution of expression scores. **I**. Volcano plots of differentially expressed genes (DEGs) in MDAMB468 single cells from GSE164715 after 5-FU treatment. For upregulated genes, the pie chart on the right illustrates the proportion of DEGs belonging to each identity as defined in the CCLE model. Similarly, for downregulated genes, the pie chart on the left shows the identity distribution of DEGs. **J.** Single-cell identity composition of MDAMB468 from GSE228154, a TNBC cell line associated with the F6 identity in the CCLE model. **K**. Single-cell identity shifts in MDAMB468 from GSE228154 after afatinib treatment. **L**. Identity scores for MDAMB468 single cells from GSE228154 before and after afatinib treatment. Boxplot center line indicates median; box bounds represent the interquartile range (IQR); whiskers extend to 1.5×IQR. **M**. Gene Set Enrichment Analysis (GSEA) results for the F0 gene set after afatinib treatment. **N**. Expression scores for three MsigDB hallmark gene sets in MDAMB468 single cells from GSE228154 before and after afatinib treatment. Violin plots show the distribution of expression scores. **O.** Volcano plots of differentially expressed genes (DEGs) in MDAMB468 single cells after afatinib treatment. For upregulated genes, the pie chart on the right illustrates the proportion of DEGs belonging to each identity as defined in the CCLE model. Similarly, for downregulated genes, the pie chart on the left shows the identity distribution of DEGs. *p<0.05, **p<0.01 by Wilcoxon rank-sum test

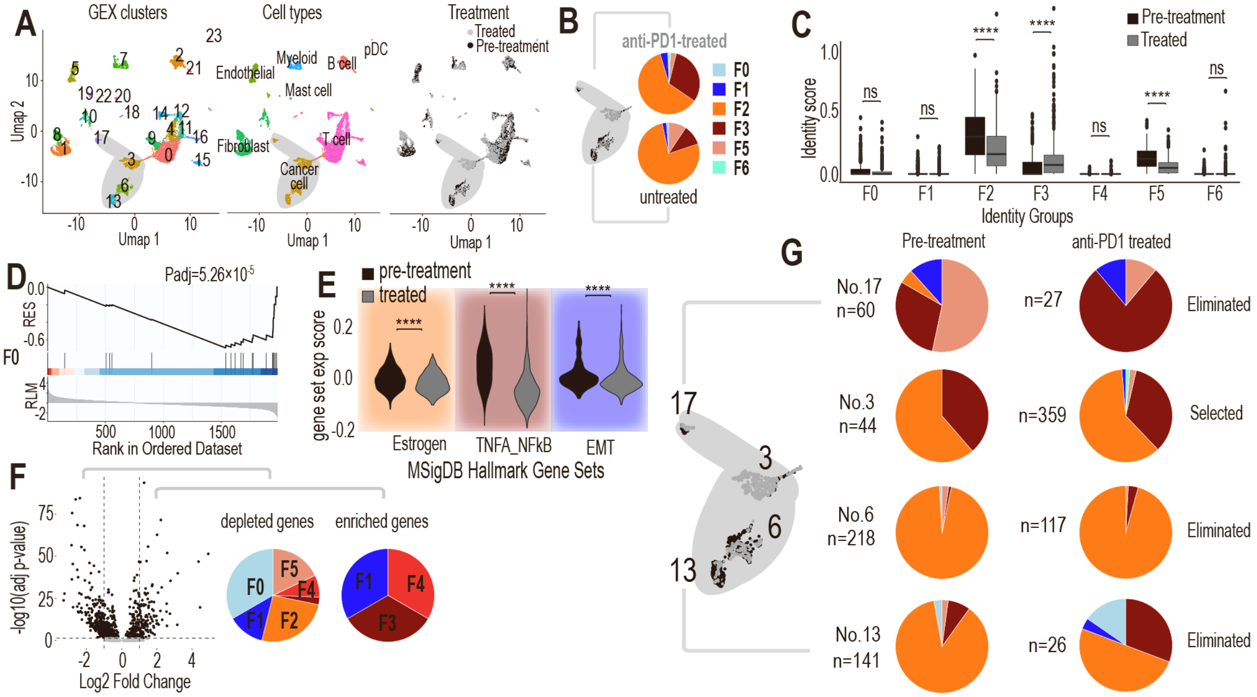

Figure S17. Single-cell identity changes of cancer cells in patient sample BIOKEY_19 after anti-PD1 treatment, Related to Figure 4

A. UMAP plots showing cluster identifications, cell types, and treatment conditions for all single cells in the BIOKEY_19 tumor. These plots provide an overview of the cellular landscape within the tumor, highlighting distinct clusters and their associations with treatment conditions. **B.** Single-cell identity changes in the cancer cells of TNBC patient BIOKEY_19 after

pembrolizumab treatment. Pie charts illustrate the distribution of single cells across different identities within the cancer cell population before and after treatment. **C.** Identity scores for single cells in the BIOKEY_19 cancer cell population before and after pembrolizumab treatment. Boxplot center line indicates median; box bounds represent the interquartile range (IQR); whiskers extend to 1.5×IQR. **D.** GSEA results for the F0 gene set after pembrolizumab treatment, highlighting the enrichment or depletion of these identity-specific gene sets in the treated cancer cell population. **E.** Expression scores for three MsigDB hallmark gene sets in BIOKEY_19 cancer single cells before and after treatment. The hallmark gene sets analyzed include estrogen response (early and late combined), TNFα signaling via NF-κB, and epithelial-mesenchymal transition (EMT), providing insights into treatment-induced changes in key biological pathways. Violin plots show the distribution of expression scores. **F.** Volcano plots of differentially expressed genes (DEGs) in BIOKEY_19 cancer single cells after pembrolizumab treatment. Pie charts accompanying the plots display the percentage of DEGs associated with each identity in the CCLE model. The right pie chart represents upregulated genes, while the left represents downregulated genes, linking these changes to specific identities. **G.** Analysis of identity composition changes in four cancer cell clusters from BIOKEY_19 (cluster_3, cluster_6, cluster_13, and cluster_17) before and after pembrolizumab treatment. Pie charts illustrate the percentage of single cells belonging to each identity within each cluster, revealing differential responses across clusters. *p<0.05, **p<0.01 by Wilcoxon rank-sum test

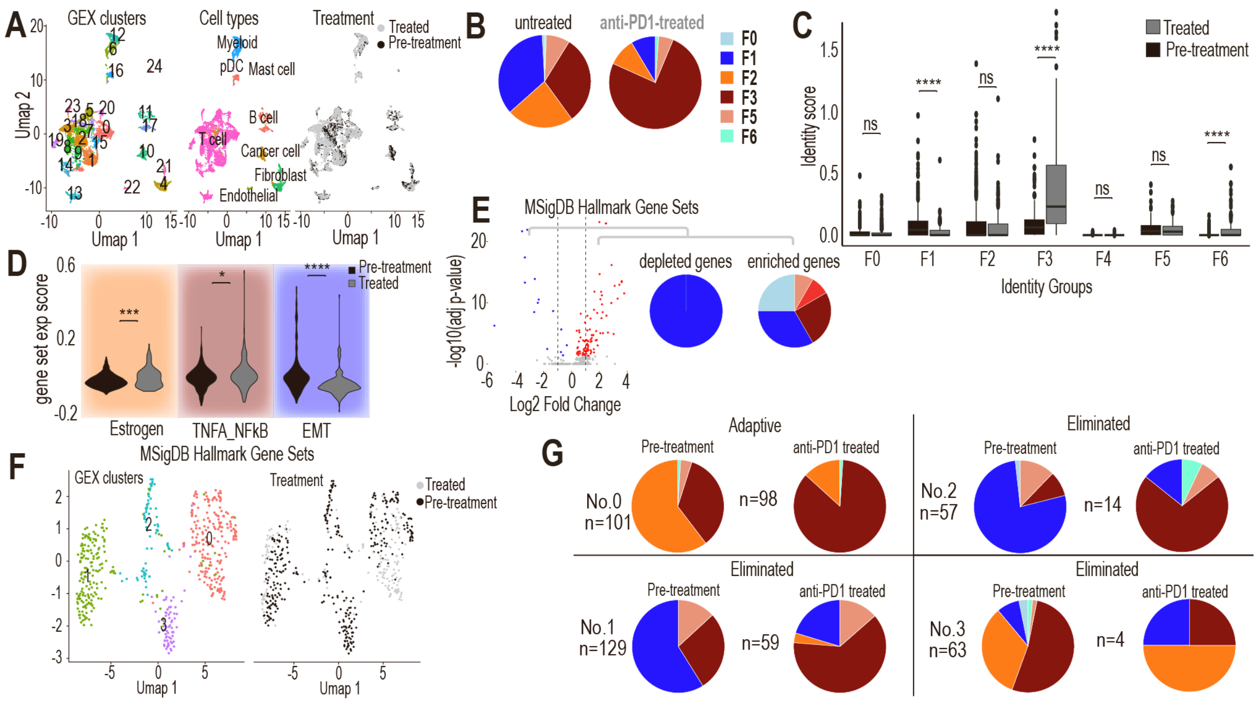

Figure S18. Single-cell identity changes of cancer cells in patient sample BIOKEY_10 after anti-PD1 treatment, Related to Figure 4

A. UMAP plots showing cluster identifications, cell types, and treatment conditions for all single cells in the BIOKEY_10 tumor. These plots provide an overview of the cellular landscape within the tumor, highlighting distinct clusters and their associations with treatment conditions. **B.**

Single-cell identity changes in the cancer cells of TNBC patient BIOKEY_10 after pembrolizumab treatment. Pie charts illustrate the distribution of single cells across different identities within the cancer cell population before and after treatment. **C.** Identity scores for single cells in the BIOKEY_10 cancer cell population before and after pembrolizumab treatment. Boxplot center line indicates median; box bounds represent the interquartile range (IQR); whiskers extend to 1.5×IQR. **D.** Expression scores for three MsigDB hallmark gene sets in BIOKEY_10 cancer single cells before and after treatment. The hallmark gene sets analyzed include estrogen response (early and late combined), TNFα signaling via NF-κB, and epithelial-mesenchymal transition (EMT), providing insights into treatment-induced changes in key biological pathways. Violin plots show the distribution of expression scores. **E.** Volcano plots of differentially expressed genes (DEGs) in BIOKEY_10 cancer single cells after pembrolizumab treatment. Pie charts accompanying the plots display the percentage of DEGs associated with each identity in the CCLE model. The right pie chart represents upregulated genes, while the left represents downregulated genes, linking these changes to specific identities. **F**. UMAP plots showing cluster identifications and treatment conditions for cancer cells in the BIOKEY_10 tumor. **G.** Analysis of identity composition changes in four cancer cell clusters from **F** (cluster_0, cluster_1, cluster_2, and cluster_3) before and after pembrolizumab treatment. Pie charts illustrate the percentage of single cells belonging to each identity within each cluster, revealing differential responses across clusters. *$p<0.05$, **$p<0.01$ by Wilcoxon rank-sum test.

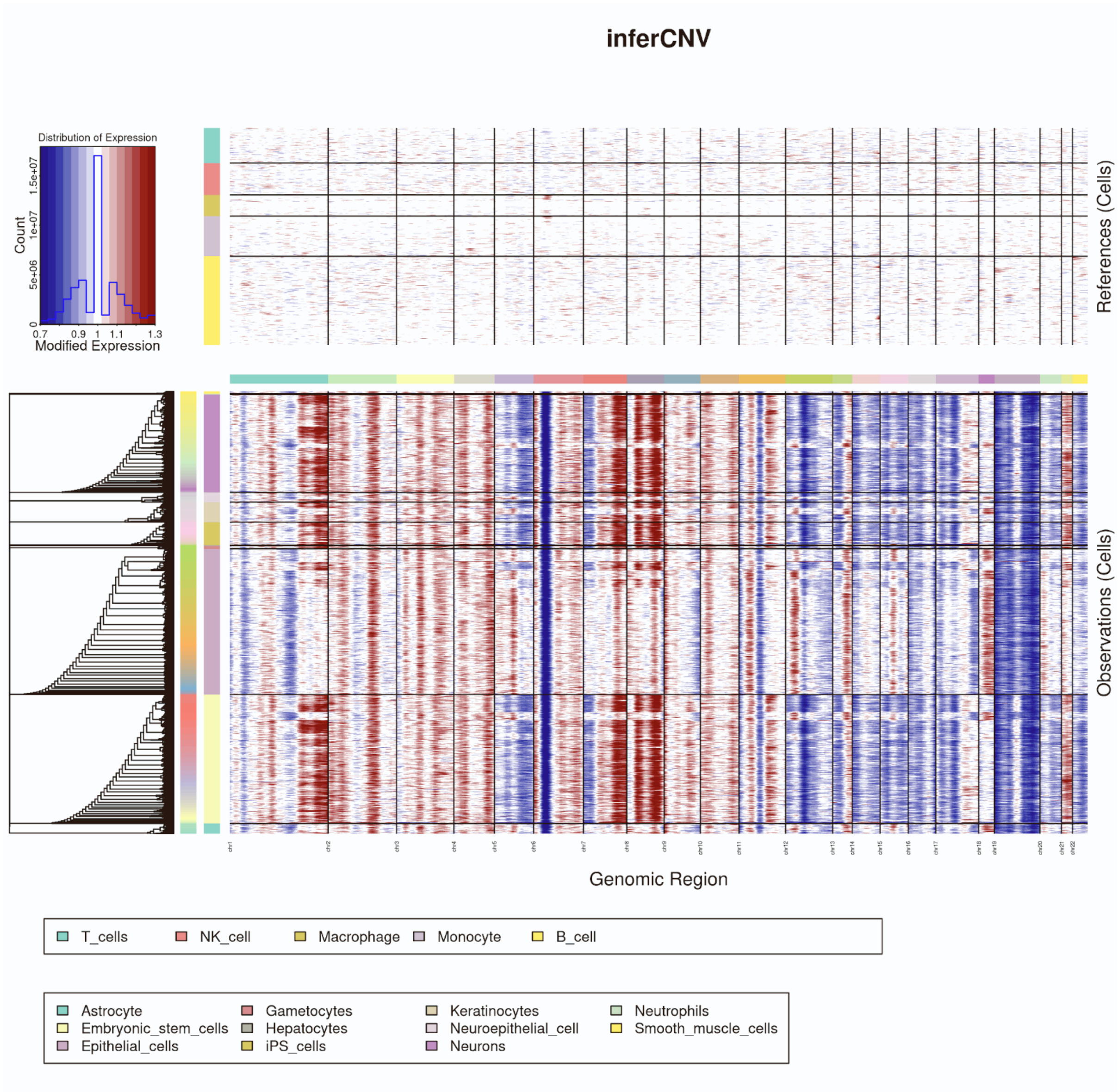

Figure S19. Infercnv result for cells in the organoid, Related to Figure 5

The top heatmap shows the chromosomal copy number alteration profiles in the reference cells. The bottom heatmap shows the chromosomal copy number alteration profiles of the single cells in the organoid. In the heatmap, each row stands for a cell. Each column stands for a gene. Genes are arranged based on their chromosomal locations.

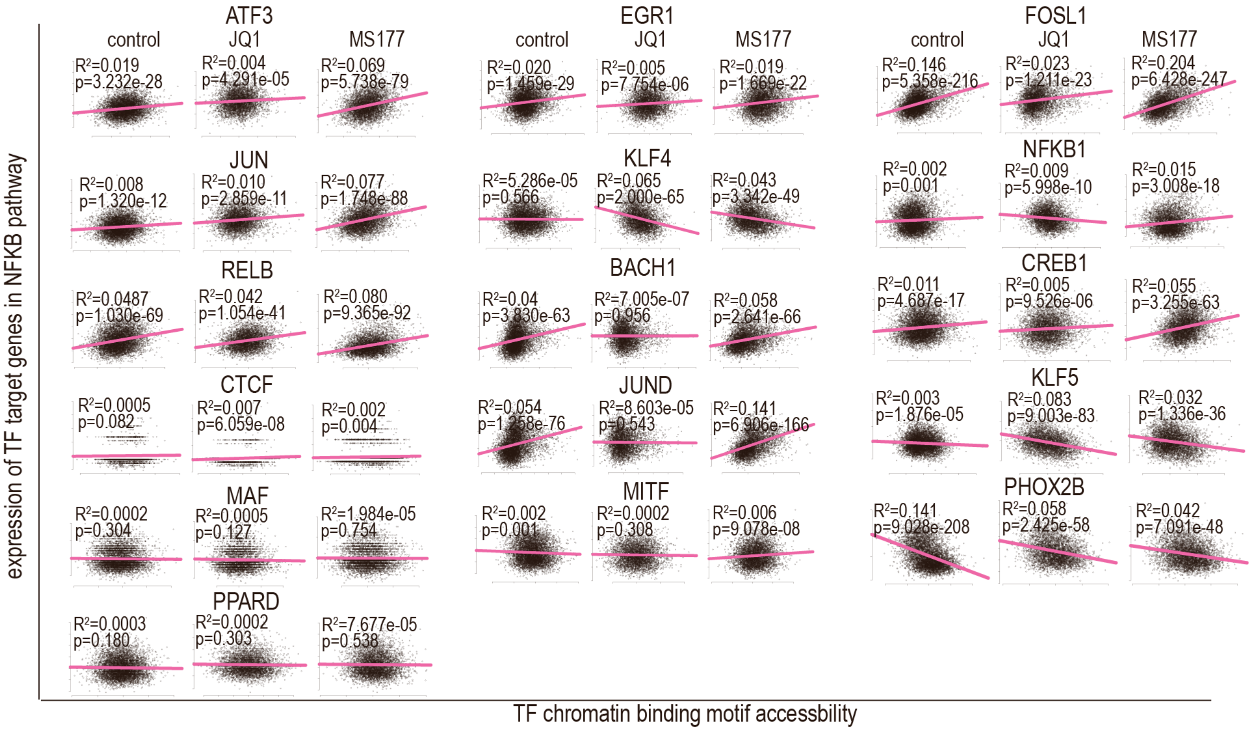

Figure S20. The relationship between TF chromatin binding motif accessibility and the expression of its target genes in NF-κB signaling pathway for all the TFs shown in main Fig. 5I in control, JQ1-treated, and MS177-treated conditions, Related to Figure 5

Comparing all of the TFs shown here with RELA, RELA has the strongest regulation of NF-κB pathway gene expression in the MS177-treated condition, and it is the most involved TF in response to MS177 treatment by comparing R^2 difference between MS177 and control conditions. Two-sided t-tests were performed to assess the statistical significance of the regression coefficient.

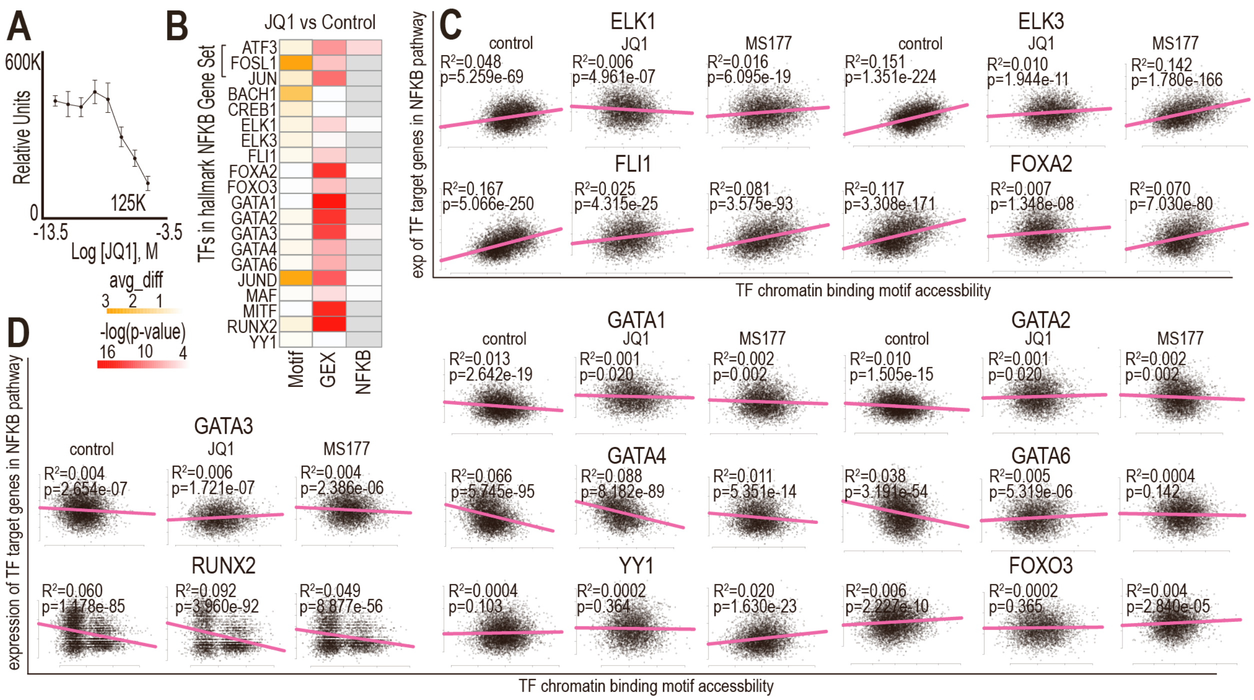

Figure S21. TFs potentially involved in JQ1 drug response, Related to Figure 5

A. Drug treatment curve showing the response of the organoid to JQ1, indicating the relative efficacy of the treatment on the organoid population **B.** Analysis of transcription factors (TFs) relevant to the response to JQ1 treatment. The first column shows the average difference in TF binding motif accessibility after JQ1 treatment. The second column highlights the significance of TFs in upregulating gene expression broadly after JQ1 treatment. The third column focuses on TFs that significantly upregulate gene expression specifically in NF-κB pathway genes after JQ1 treatment. **C-D.** The relationship between TF chromatin binding motif accessibility and the expression of its target genes in NF-κB signaling pathway for all the TFs shown in **B** in the control, JQ1-treated, and MS177-treated conditions. Comparing all of the TFs shown here with RELA, RELA has the strongest regulation of NF-κB pathway gene expression in the MS177-treated condition, and it is the most involved TF in response to MS177 treatment by comparing R^2 difference between MS177 and control conditions. Two-sided t-tests were performed to assess the statistical significance of the regression coefficient.

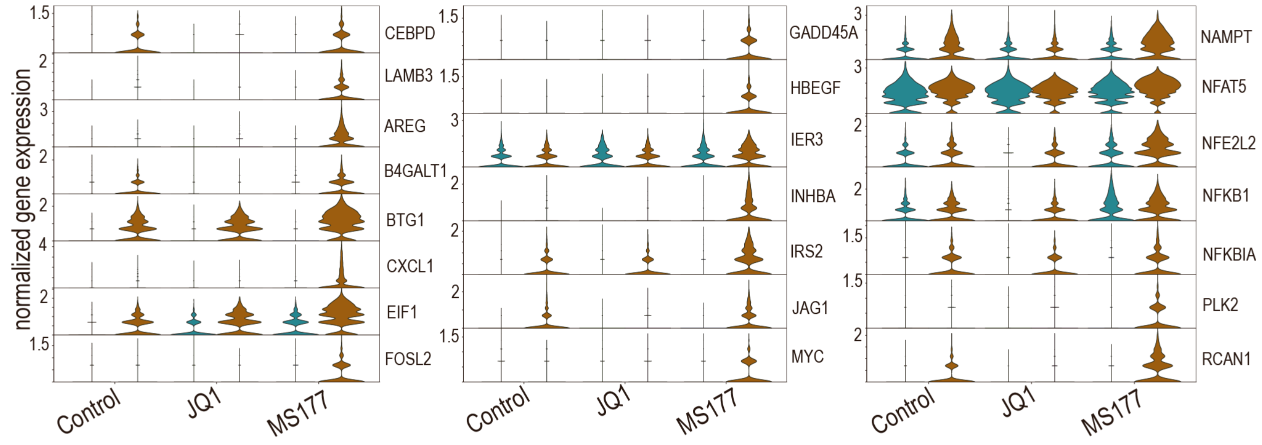

Figure S22. Genes in the NF-κB hallmark gene set whose expressions are specifically induced by MS177 in the stem-cell-like cluster, Related to Figure 5

This further confirms that distinct clusters of cancer cells have different pathway activities in response to MS177 treatment. Violin plots show the distribution of expression scores.

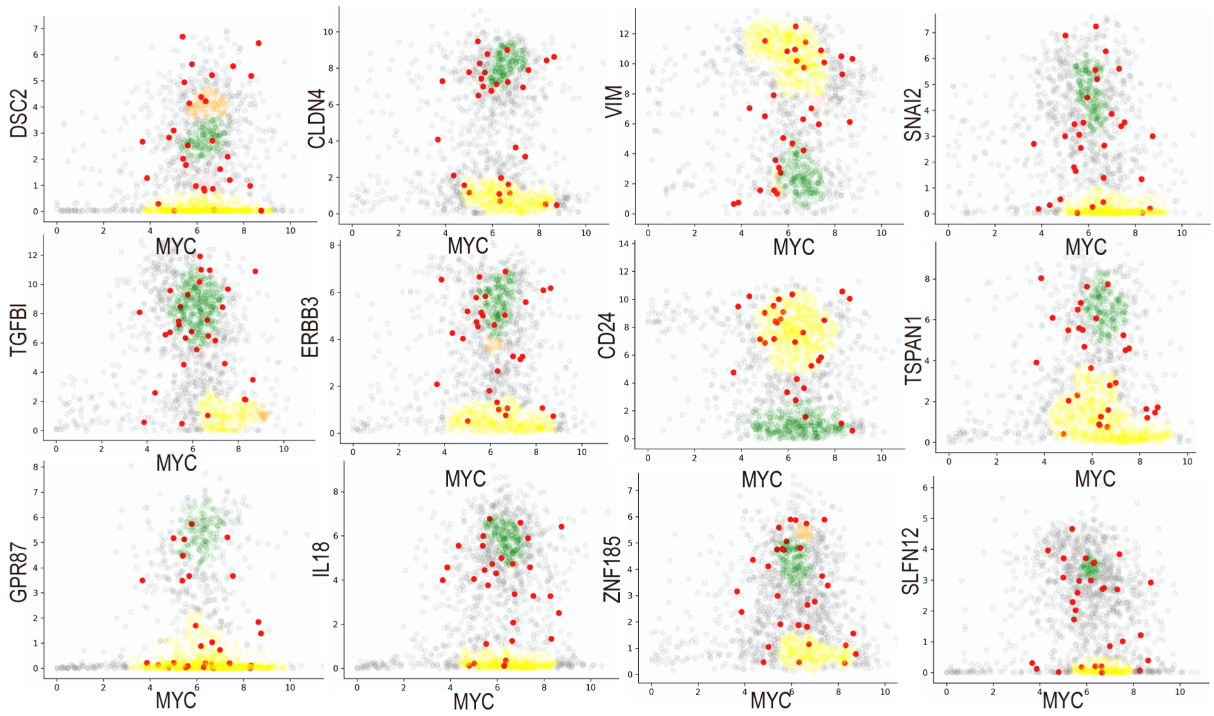

Figure S23. Twelve representative salient genes that substantially contribute to the differentiation of distinct identities in TNBC cell lines, as identified by DBSCAN, Related to Figure 1 and Figure 2

For each representative gene, its log-transformed TPM expression and MYC expression are shown on scatterplots across all CCLE pan-cancer cell lines. Each dot represents a CCLE cell line, with TNBC cell lines highlighted in red. The yellow, green and orange clusters on the scatterplots denote cell line groups identified by the DBSCAN algorithm.

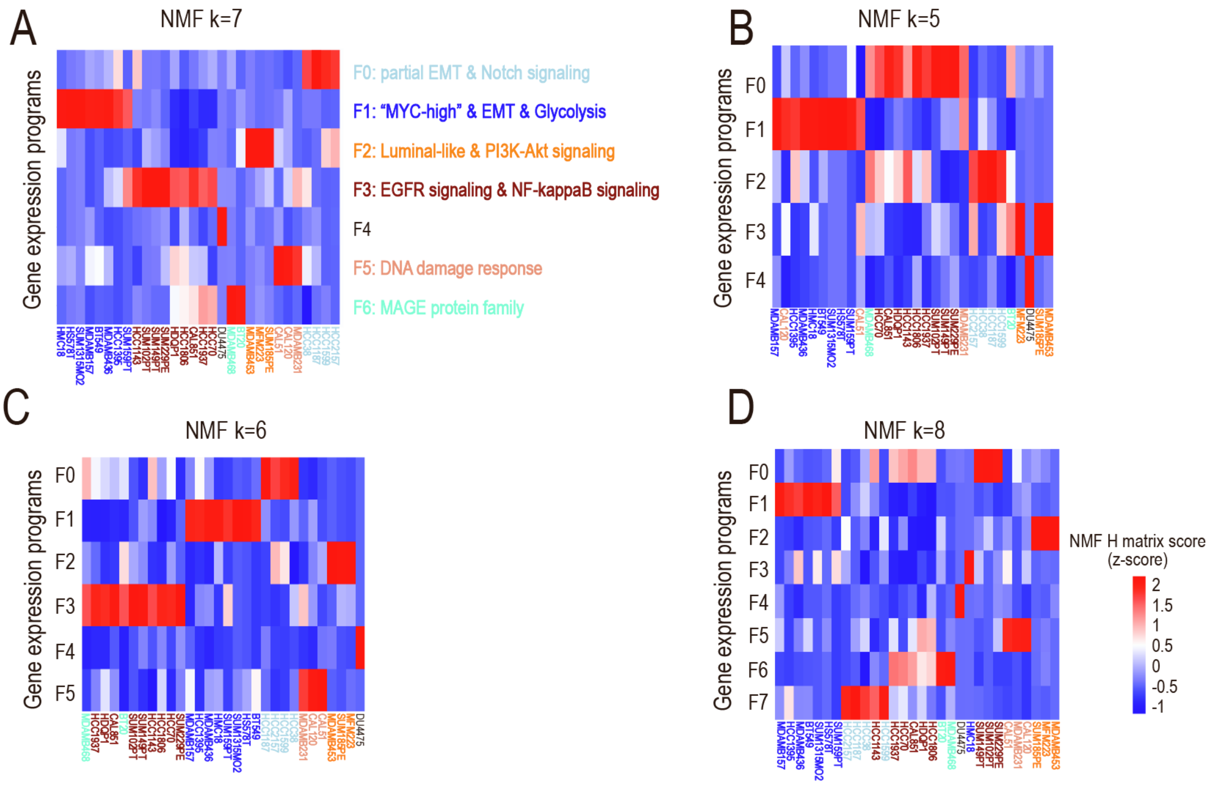

Figure S24. NMF H matrix results for TNBC CCLE cell line models across k values ranging from 5 to 8, Related to Figure 1 and Figure 2

Each heatmap displays the grouping of TNBC cell lines under a specific k value, with color intensity representing the contributing score across different identities. Cell lines are colored according to their assigned identities under k=7. For k=5,6, and 8, cell lines with the same identity color consistently cluster together, indicating that cell line identities are relatively stable across varying k values.
