# Supplementary material for: Decoding drug-responsive cell subpopulations in triple-negative breast cancer using single-cell multiomics
Source: iScience. 2026 Mar 21;29(5):115445. doi: 10.1016/j.isci.2026.115445 (PMC13138057; doi:10.1016/j.isci.2026.115445)
Supplement: Data S1. All 523 selected salient genes in the CCLE TNBC model, related to Figures 1 and 2 — For each representative gene, its log-transformed TPM expression and MYC expression are shown on scatterplots across all CCLE pan-cancer cell lines. Each dot represents a CCLE cell line, with TNBC cell lines highlighted in red. Colored clusters (yellow, green, orange, and so forth) indicate DBSCAN-identified cell line groups. [file mmc8.pdf]

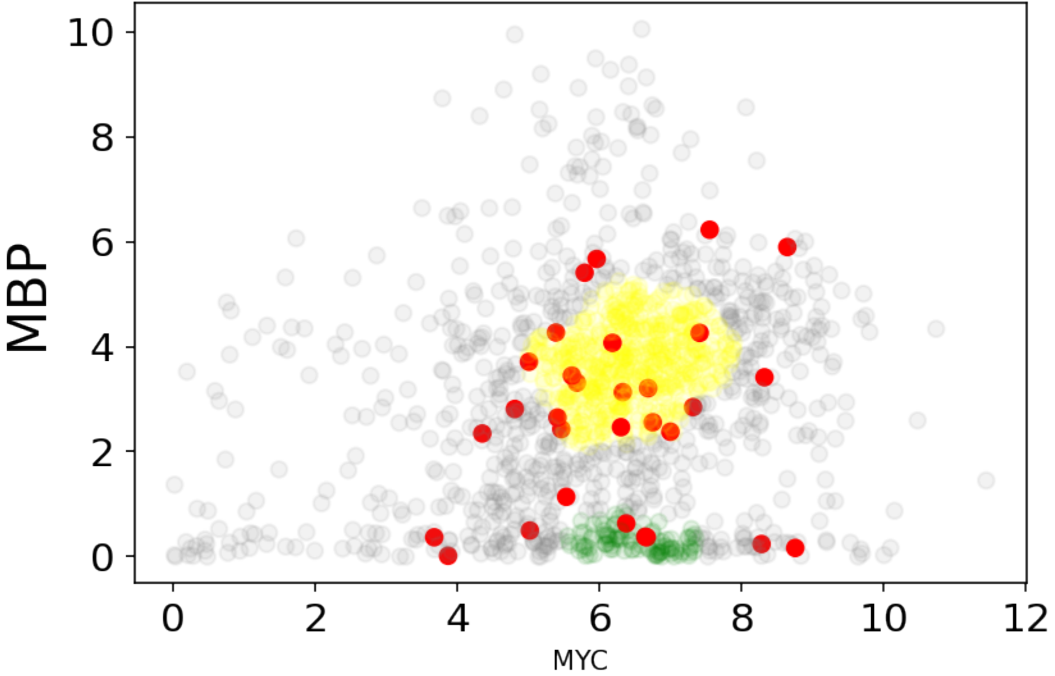

MBP
MYC
0
2
4
6
8
10
12

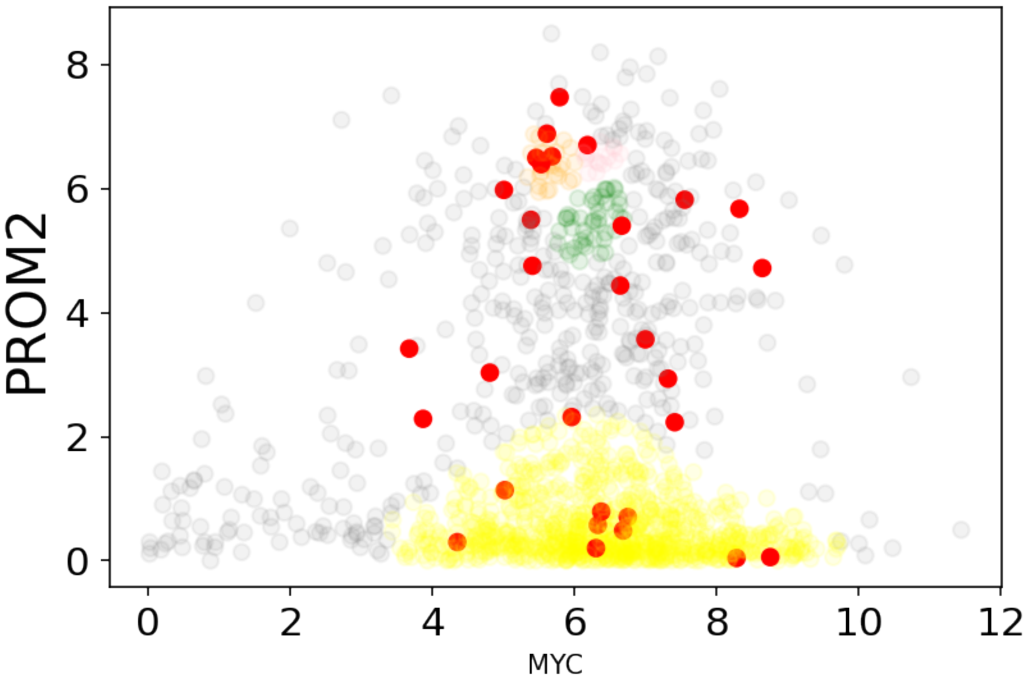
PROM2
MYC
0
2
4
6
8
10
12

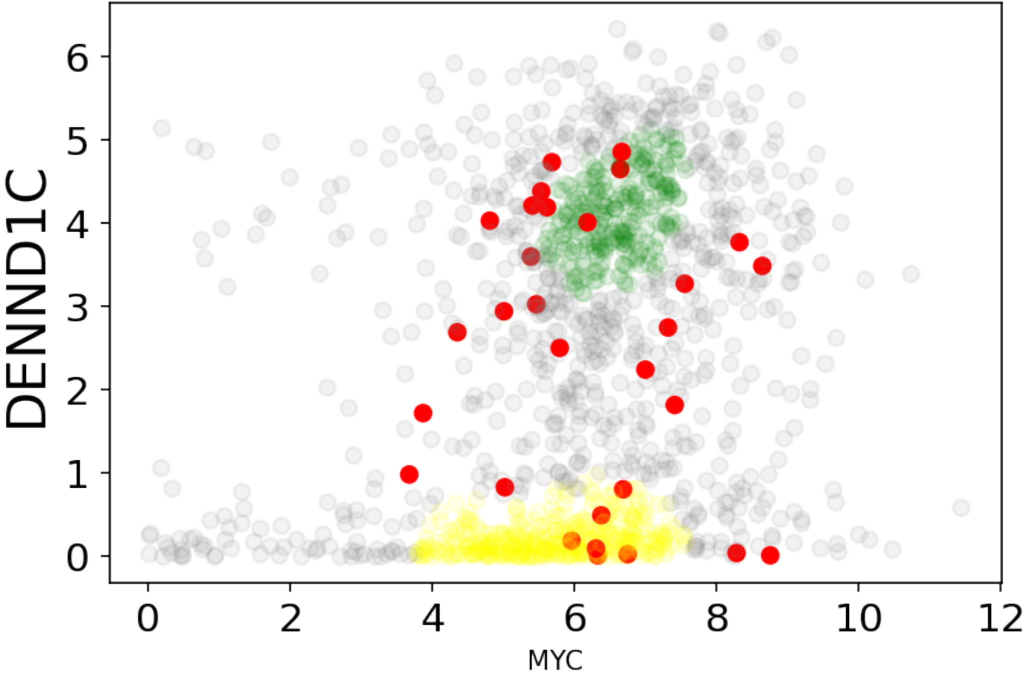

DENND1C
MYC
0
1
2
3
4
5
6
0
2
4
6
8
10
12

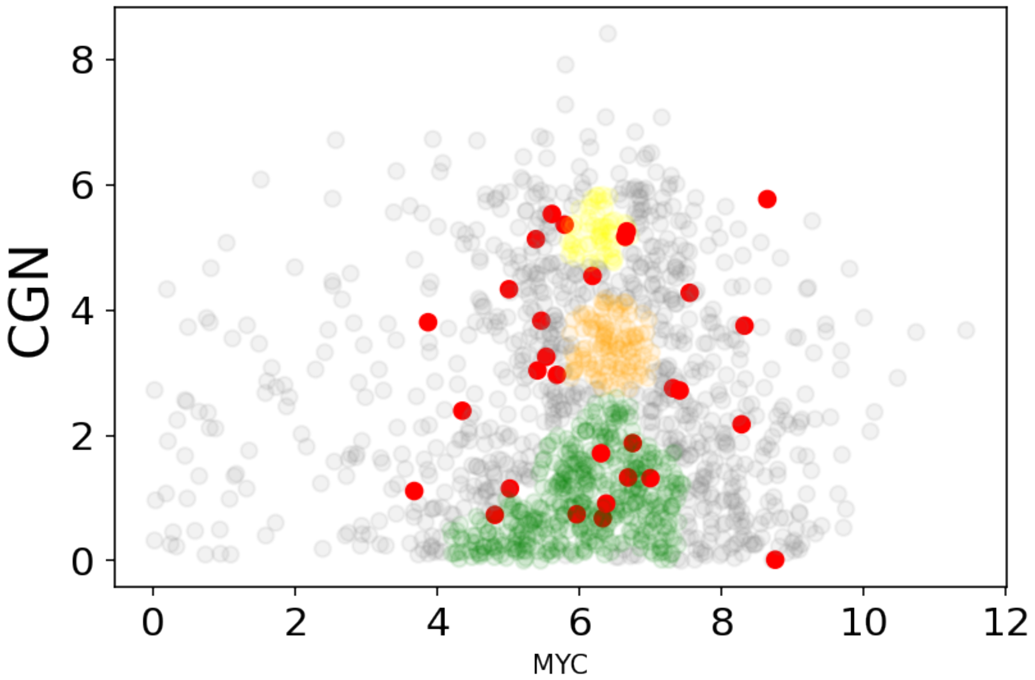

CGN
0
2
4
6
8
0
2
4
6
8
10
12
MYC

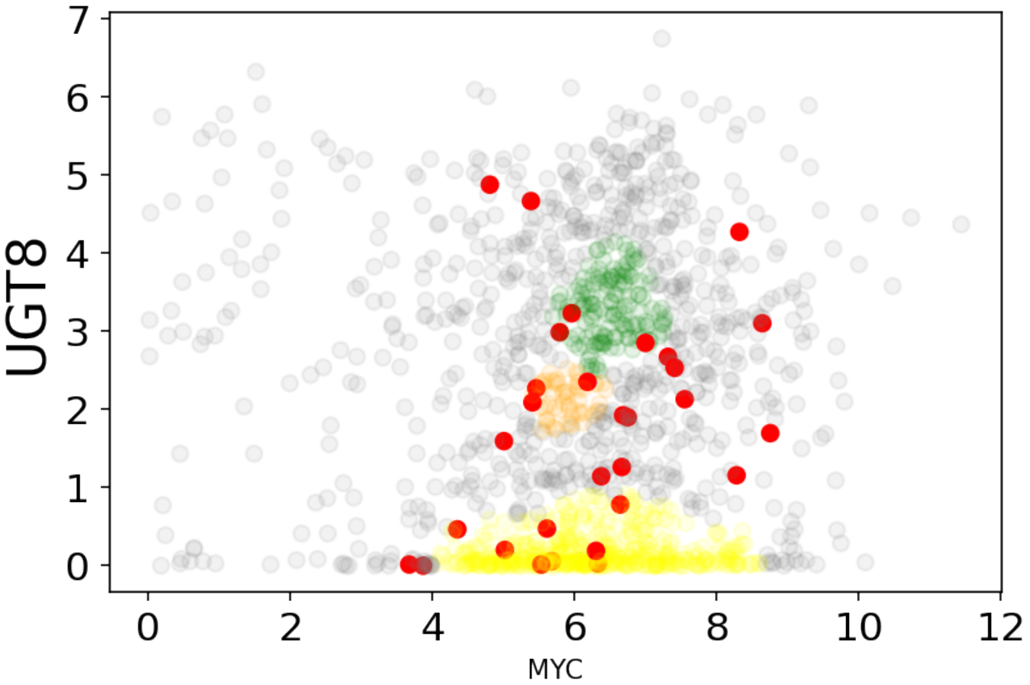
UGT8
0
1
2
3
4
5
6
7
0
2
4
6
8
10
12
MYC

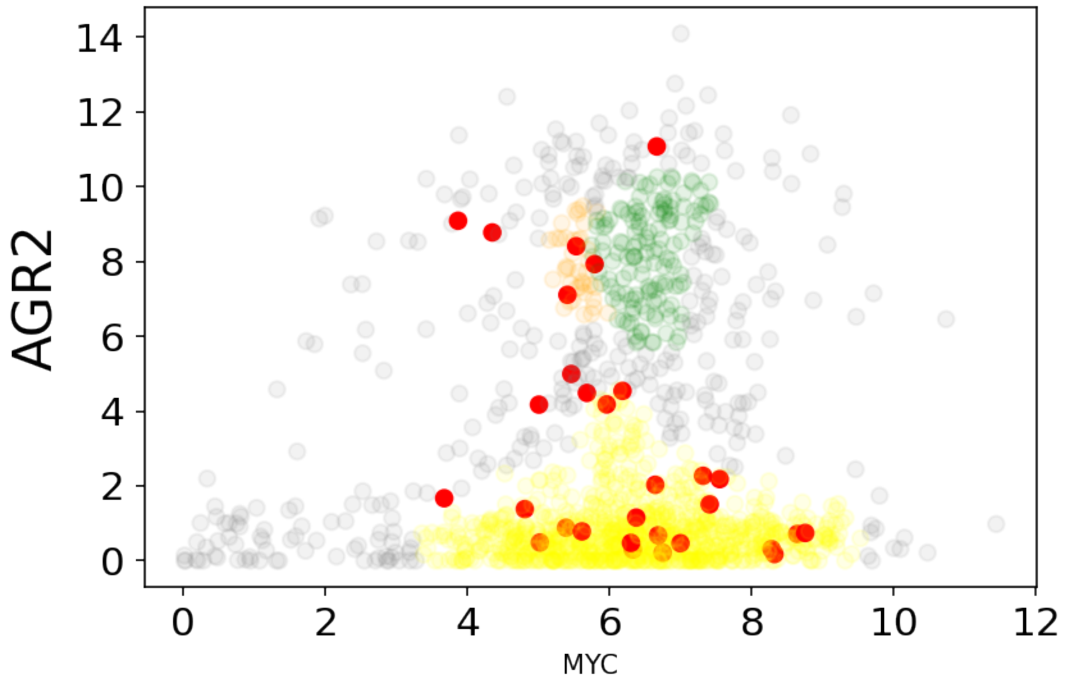
AGR2
14
12
10
8
6
4
2
0
0
2
4
6
8
10
12
MYC

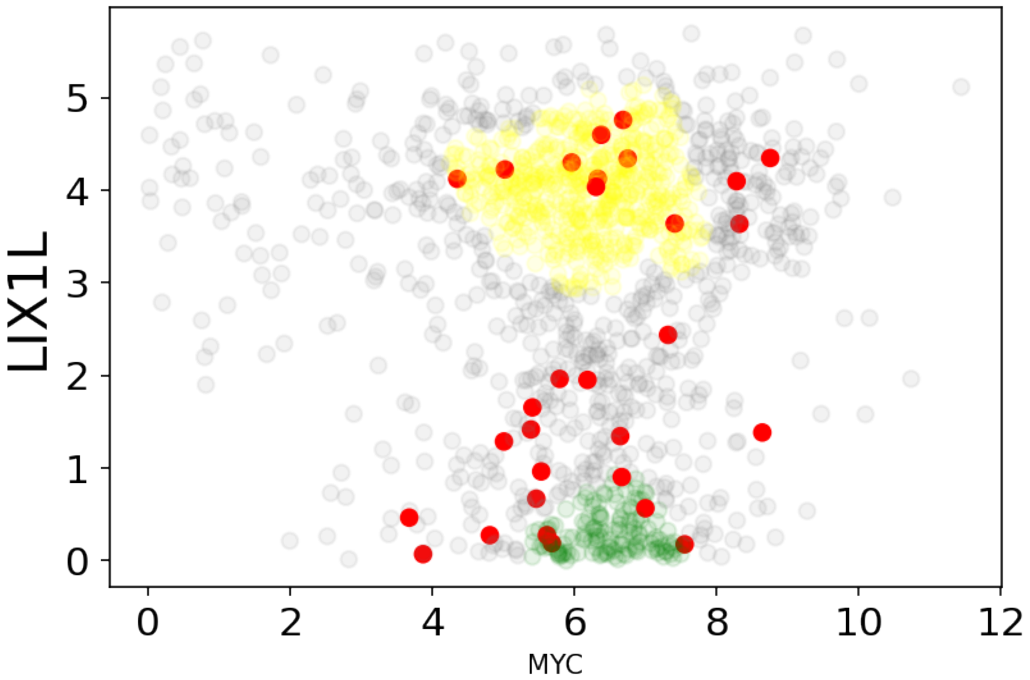
LIX1L
MYC
0
1
2
3
4
5
0
2
4
6
8
10
12

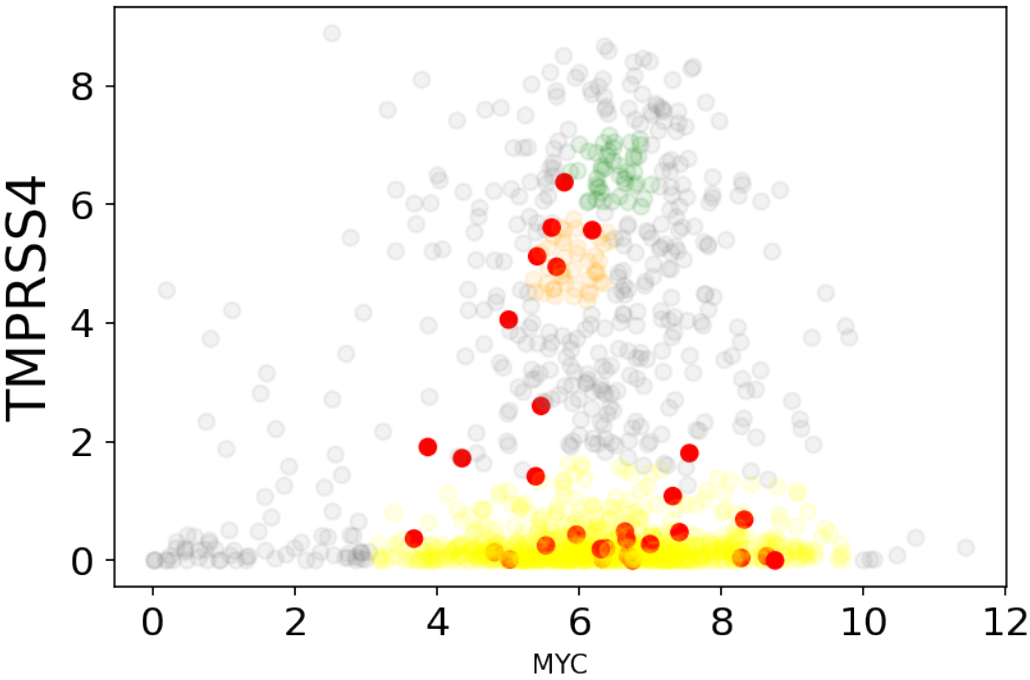
TMPRSS4
0
2
4
6
8
0
2
4
6
8
10
12
MYC

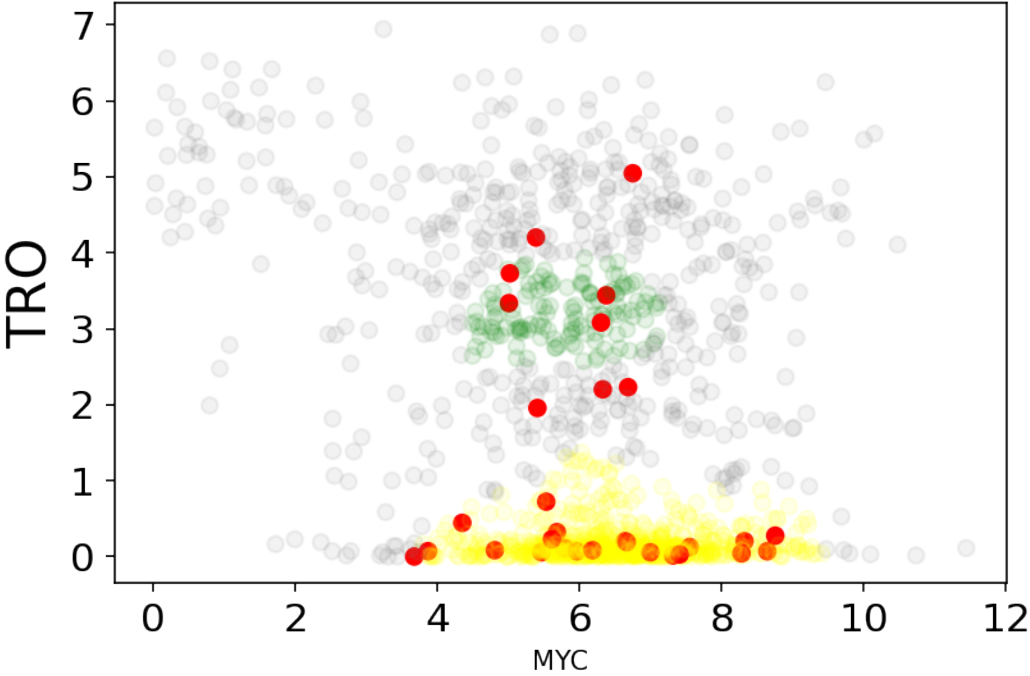

TRO
7
6
5
4
3
2
1
0
0
2
4
6
8
10
12
MYC

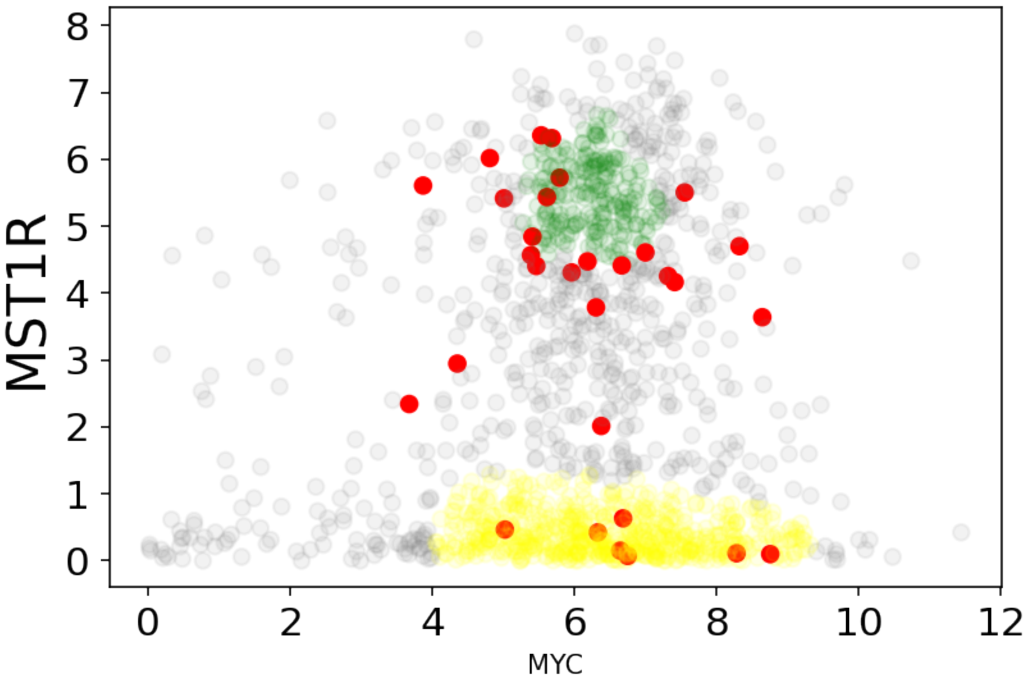

MST1R
MYC
0
1
2
3
4
5
6
7
8
0
2
4
6
8
10
12

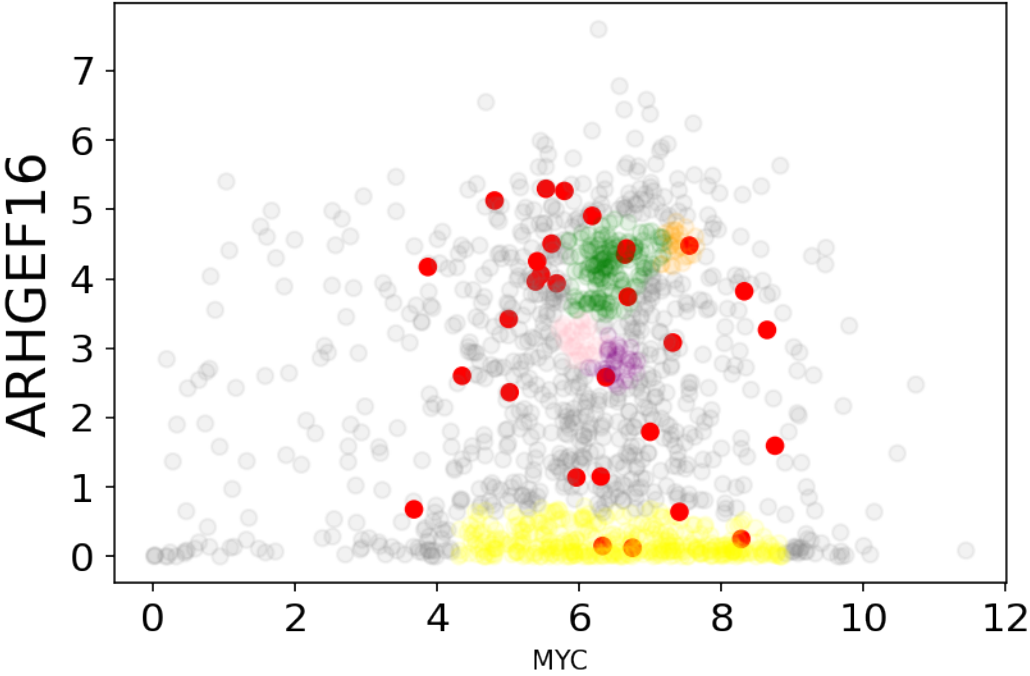

ARHGEF16
7
6
5
4
3
2
1
0
0
2
4
6
8
10
12
MYC

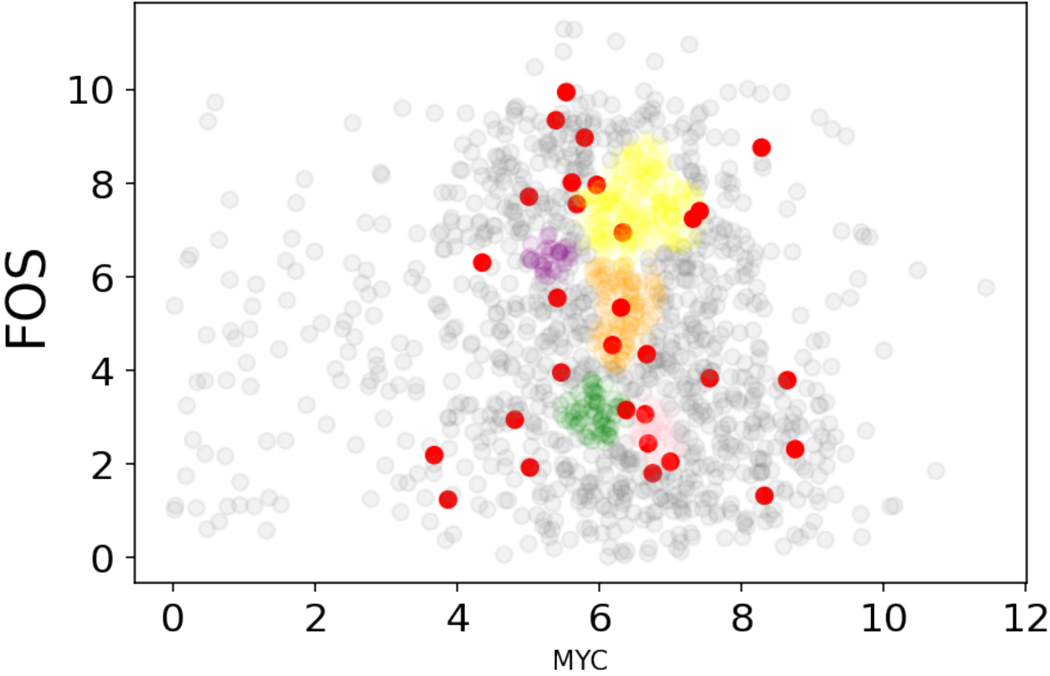

FOS
MYC
0
2
4
6
8
10
12

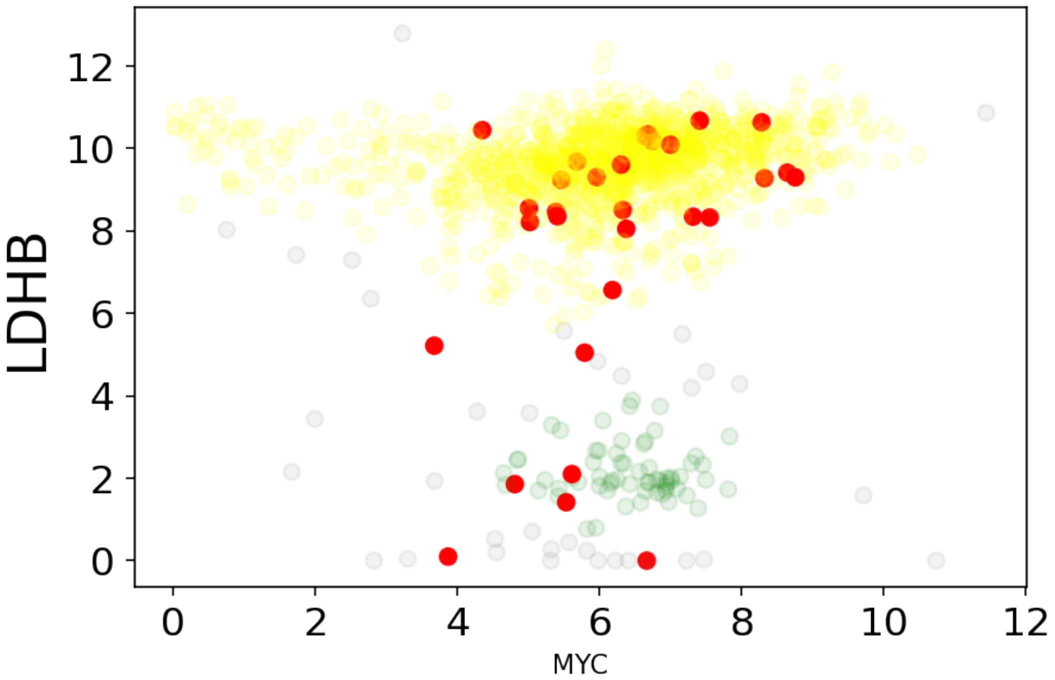

LDHB
MYC
0
2
4
6
8
10
12

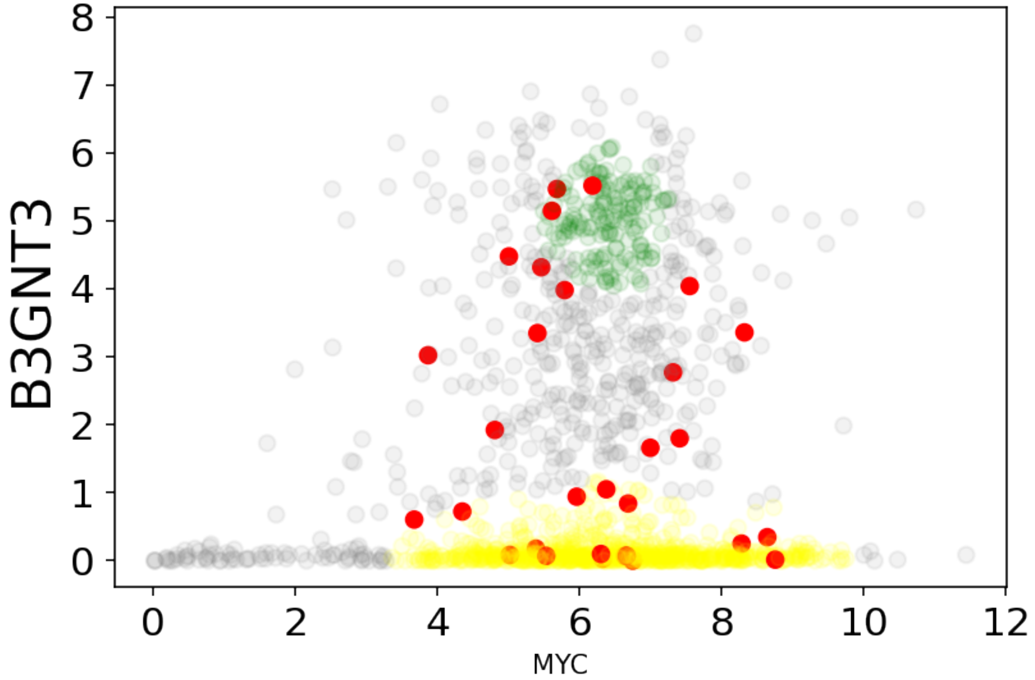

B3GNT3
MYC
0
1
2
3
4
5
6
7
8
0
2
4
6
8
10
12

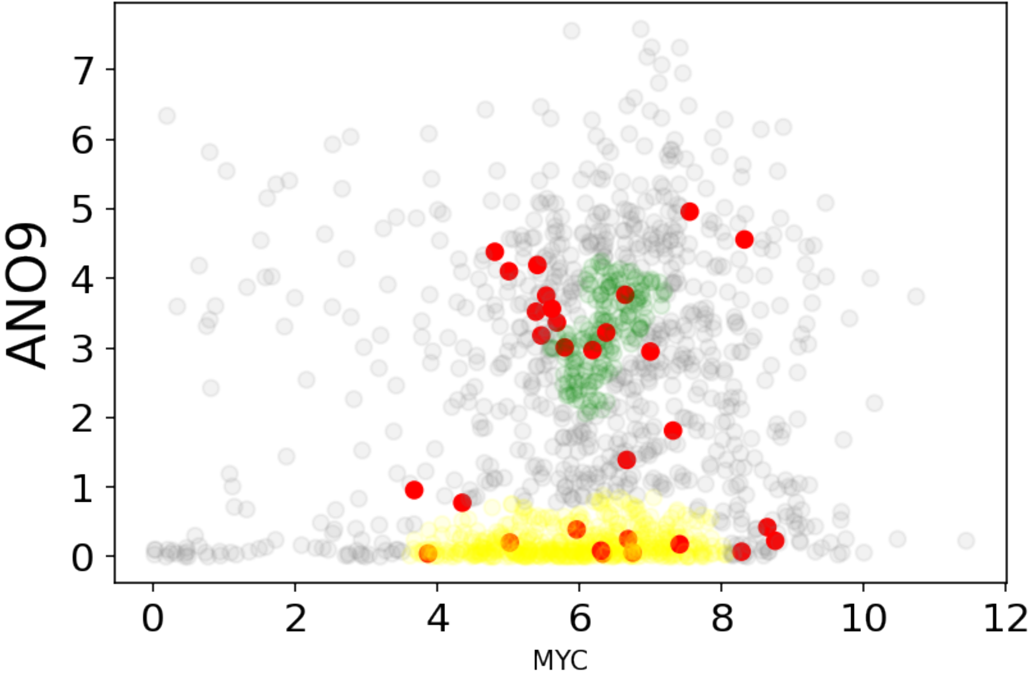
ANO9
0
1
2
3
4
5
6
7
0
2
4
6
8
10
12
MYC

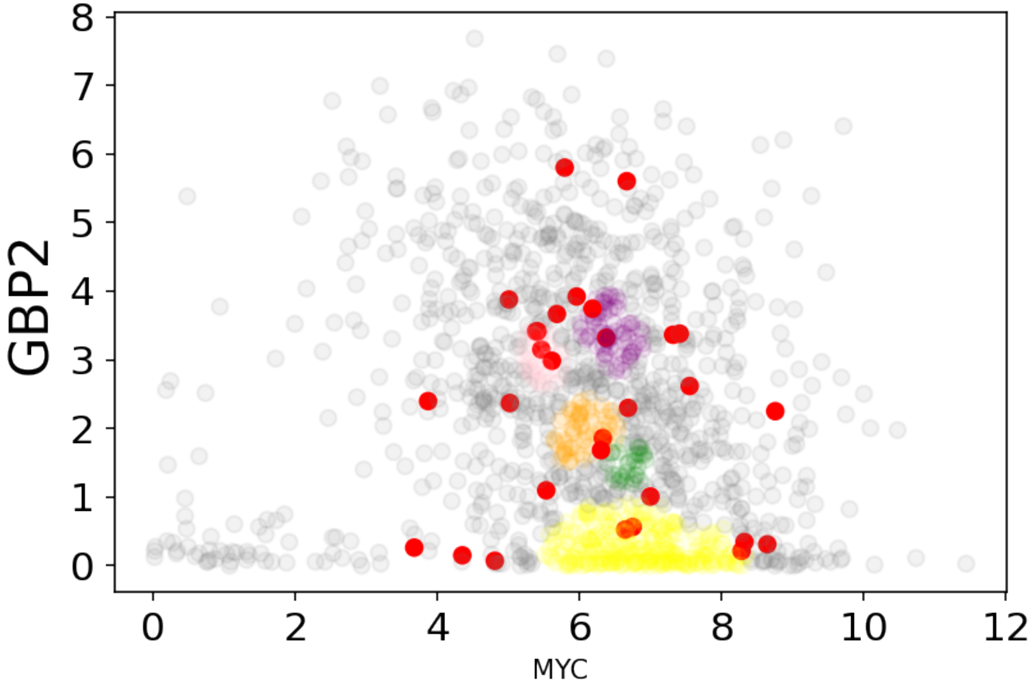
GBP2
0
1
2
3
4
5
6
7
8
0
2
4
6
8
10
12
MYC

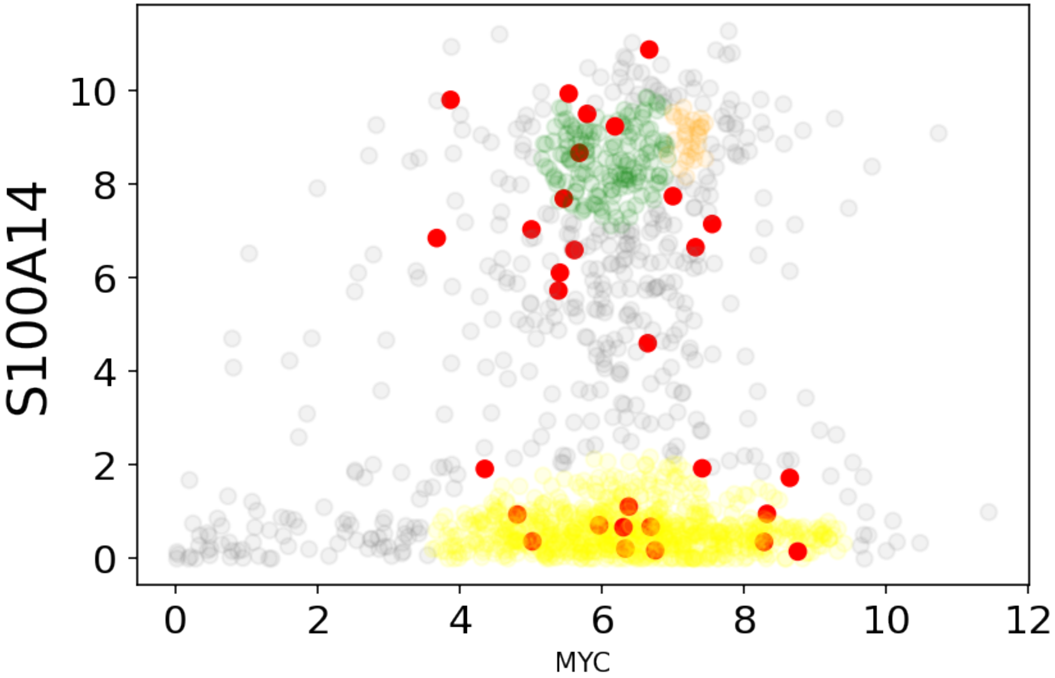
S100A14
MYC
0
2
4
6
8
10
12

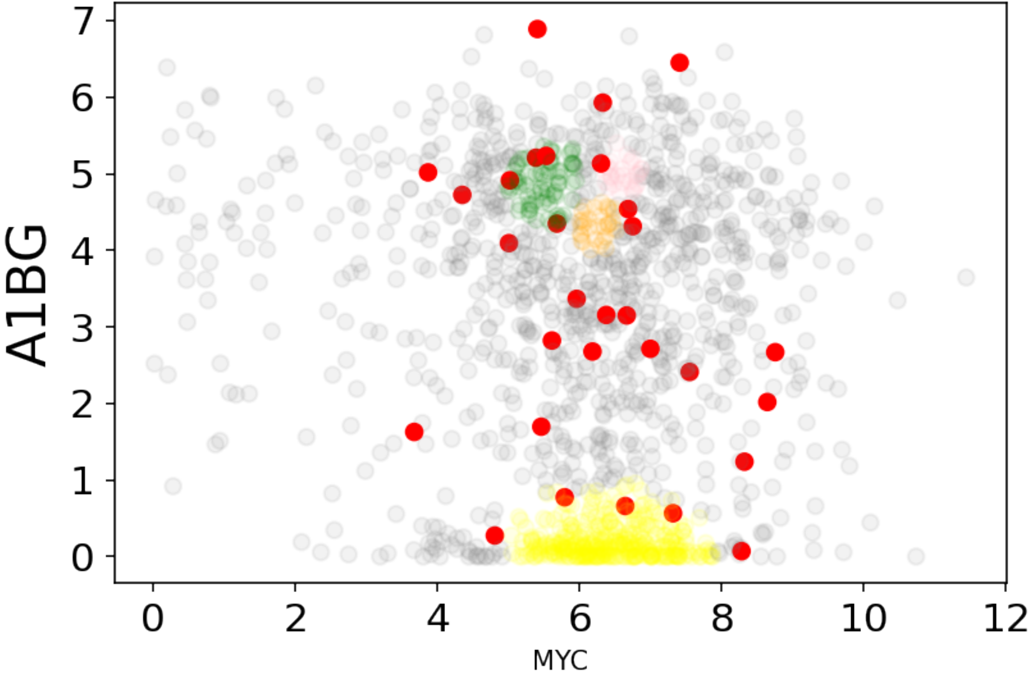

A1BG
MYC
0
1
2
3
4
5
6
7
0
2
4
6
8
10
12

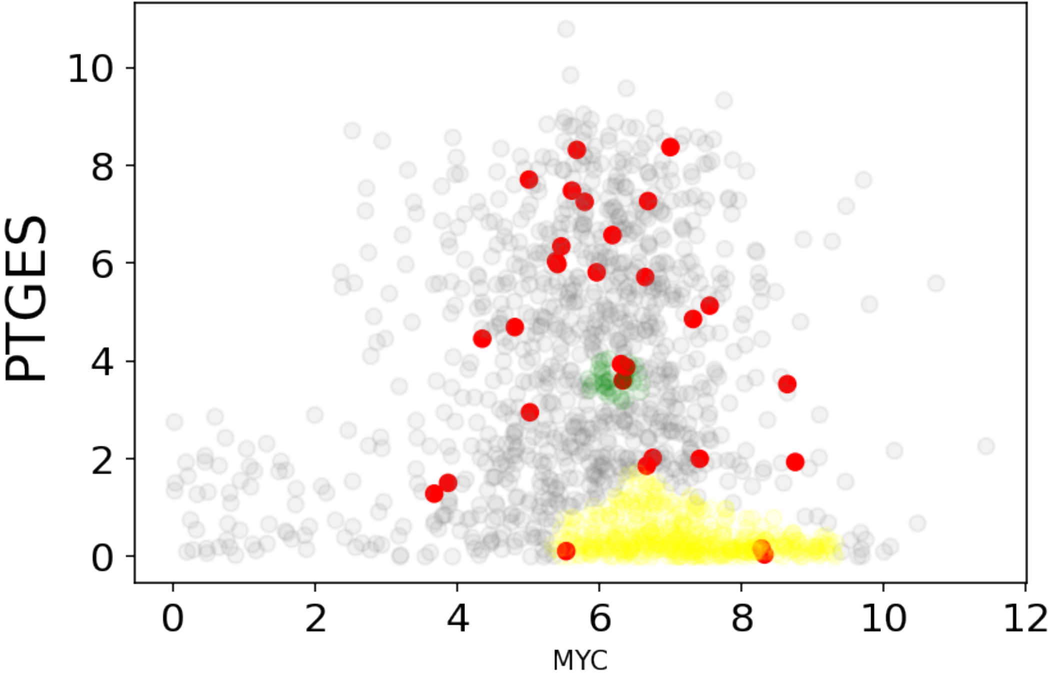
PTGES
MYC
0
2
4
6
8
10
12

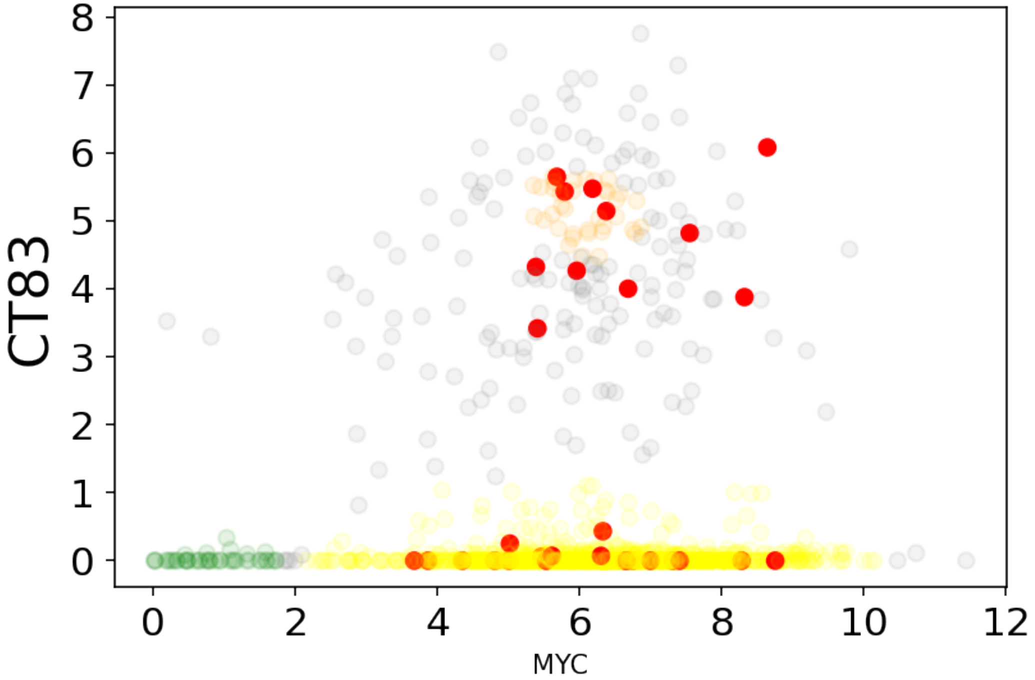
CT83
MYC
0
1
2
3
4
5
6
7
8
0
2
4
6
8
10
12

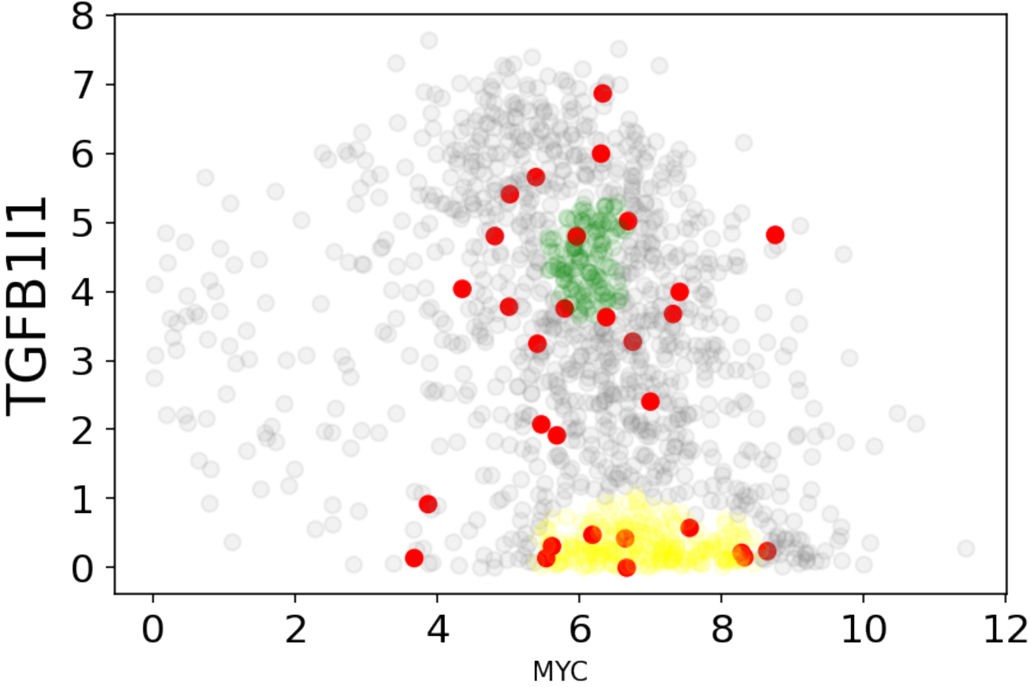
TGFB1I1
MYC
0
1
2
3
4
5
6
7
8
0
2
4
6
8
10
12

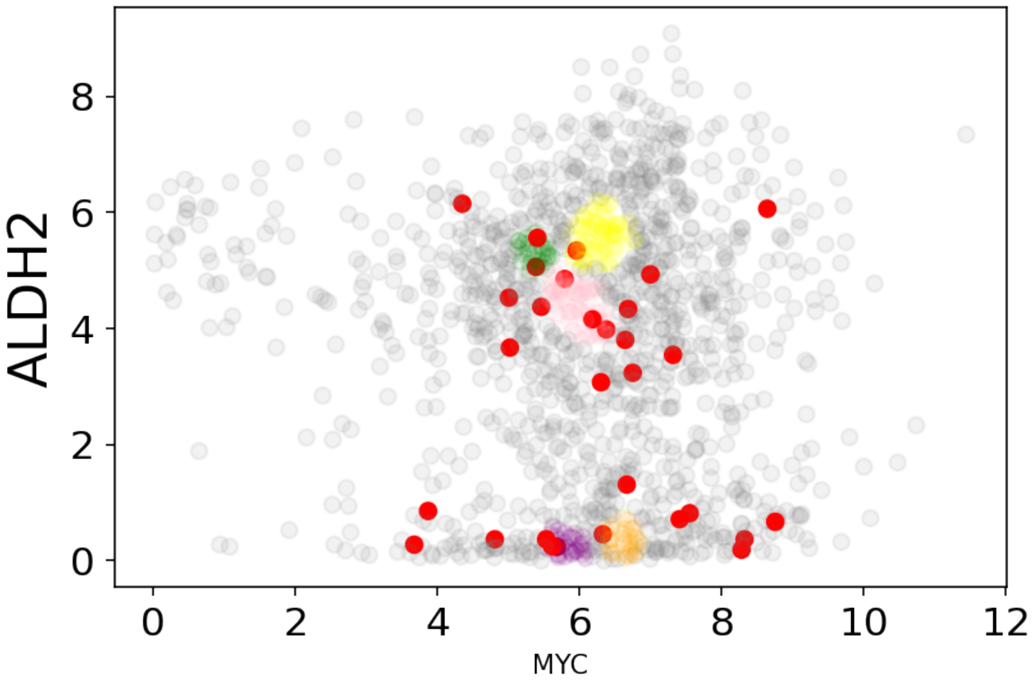

ALDH2
MYC
0
2
4
6
8
10
12

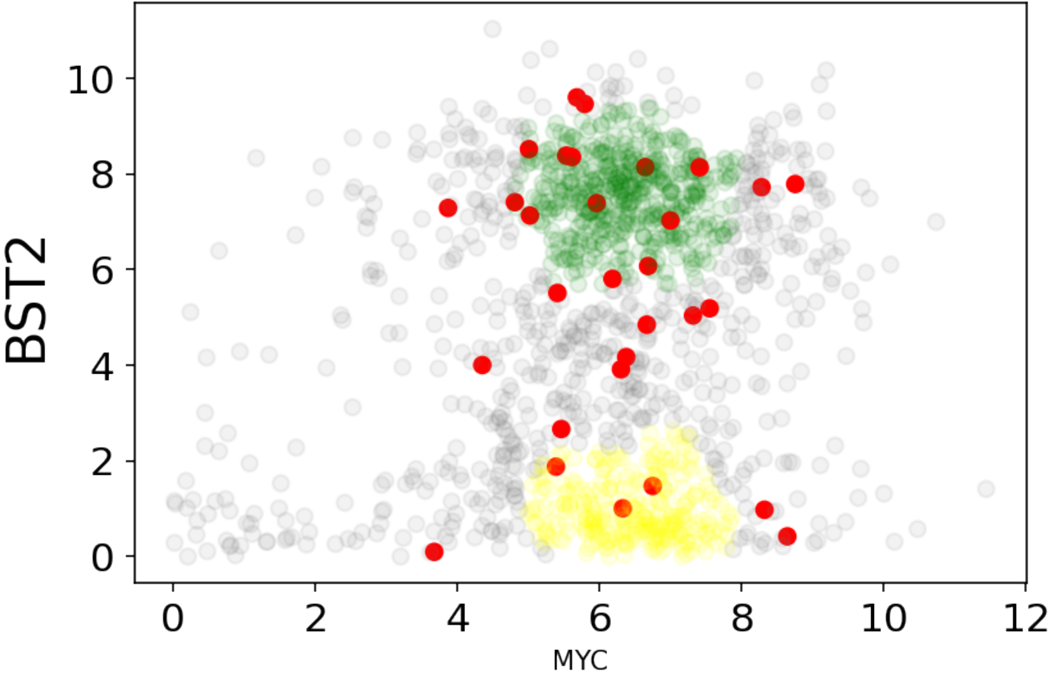

BST2
MYC
0
2
4
6
8
10
12

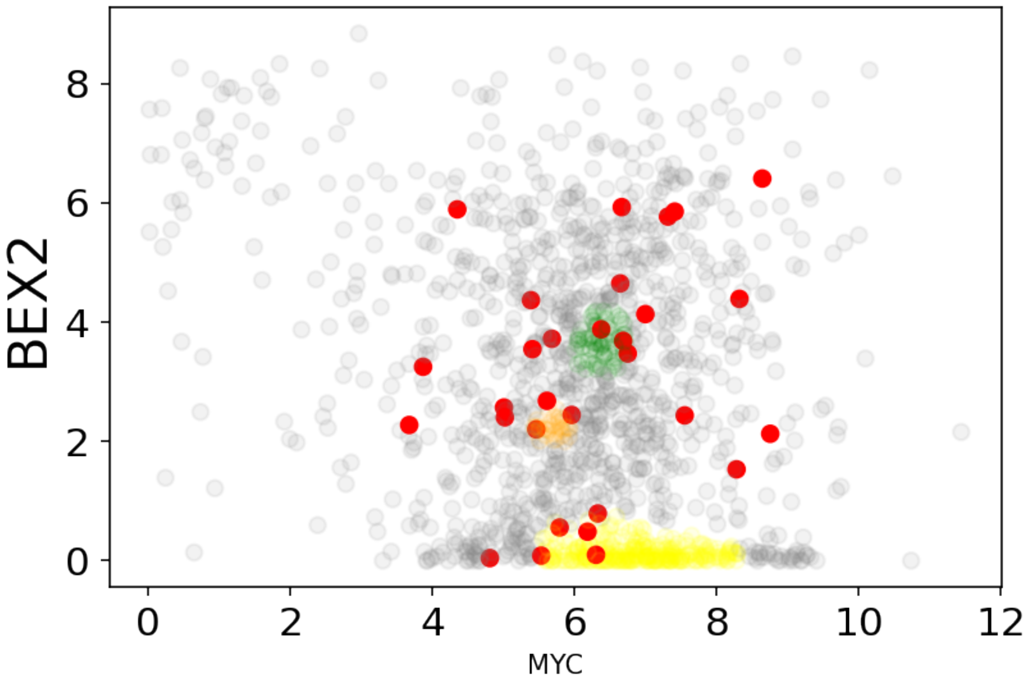

BEX2
MYC
0
2
4
6
8
10
12

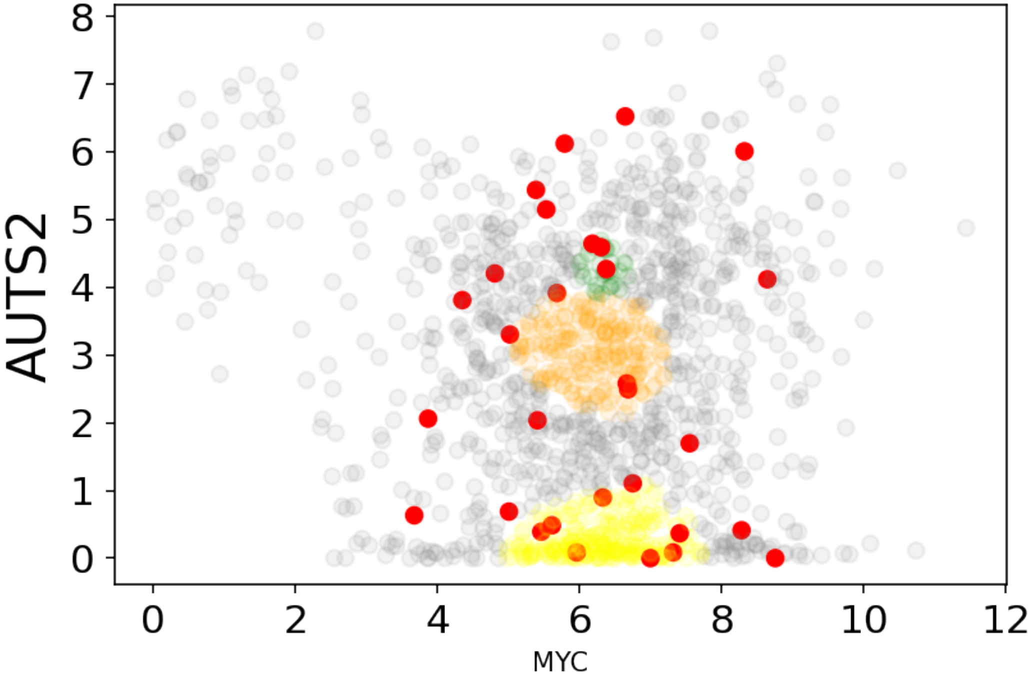

AUTS2
MYC
0
1
2
3
4
5
6
7
8
0
2
4
6
8
10
12

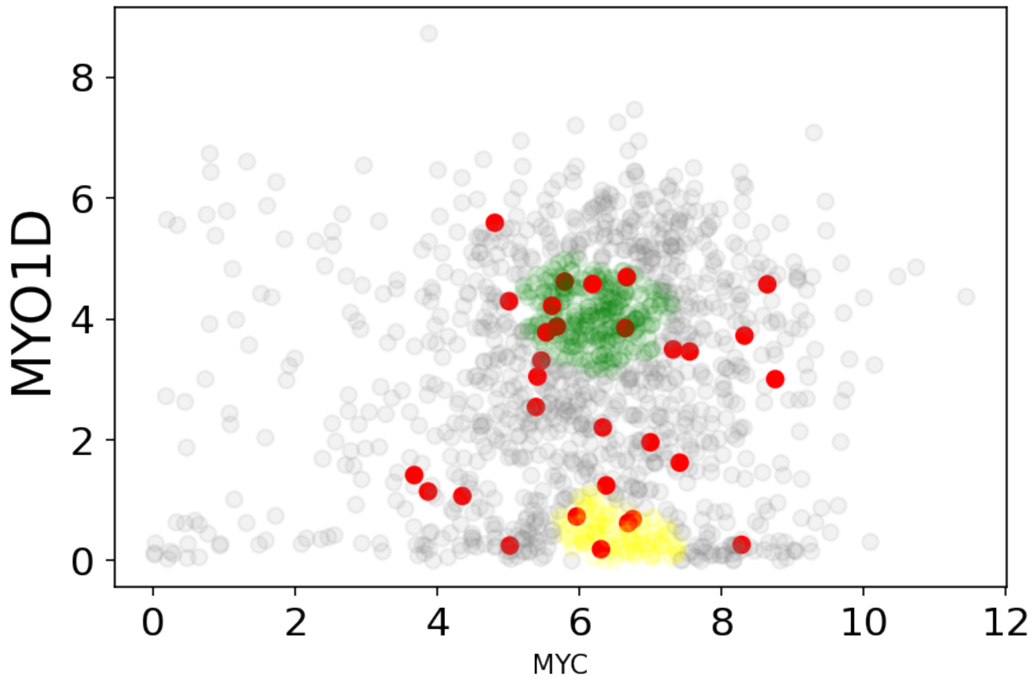
MYO1D
MYC
0
2
4
6
8
10
12

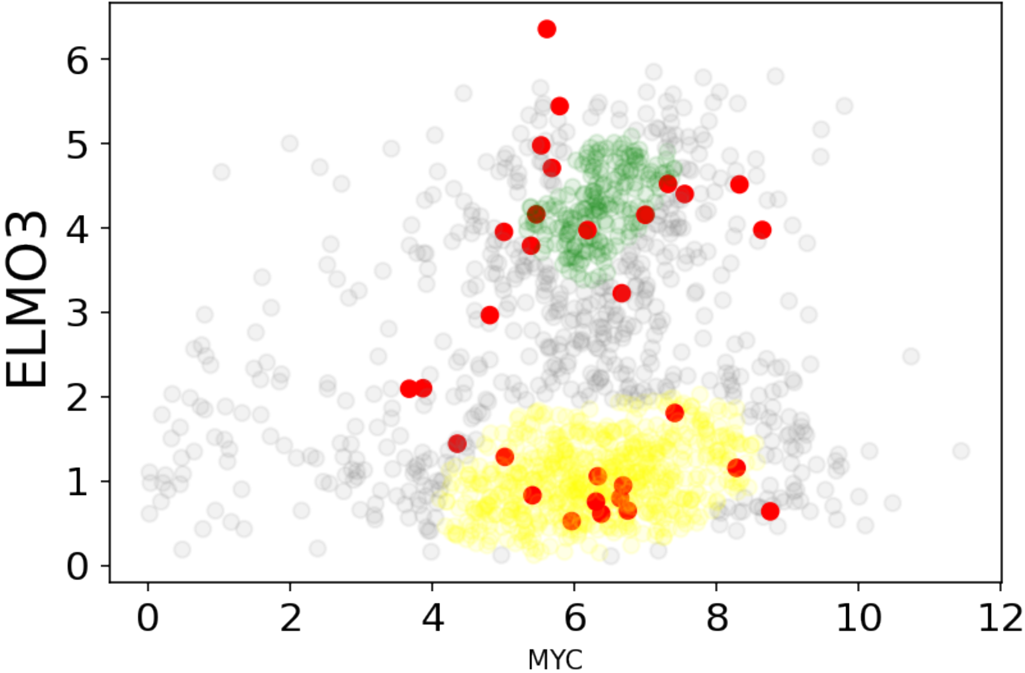

ELMO3
MYC
0
1
2
3
4
5
6
0
2
4
6
8
10
12

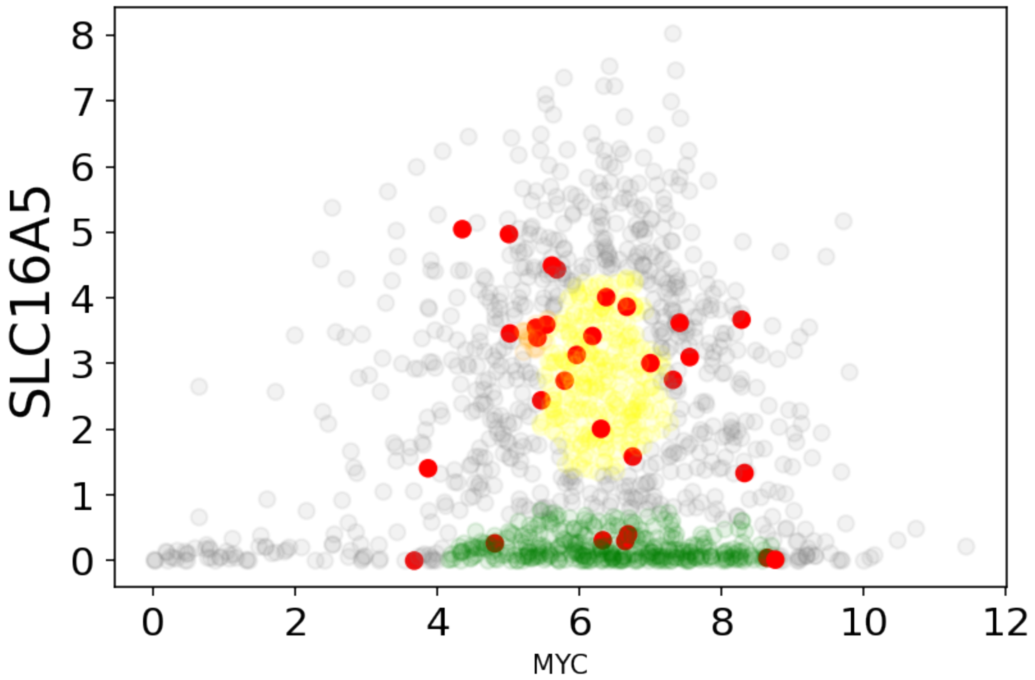
SLC16A5
MYC
0
1
2
3
4
5
6
7
8
0
2
4
6
8
10
12

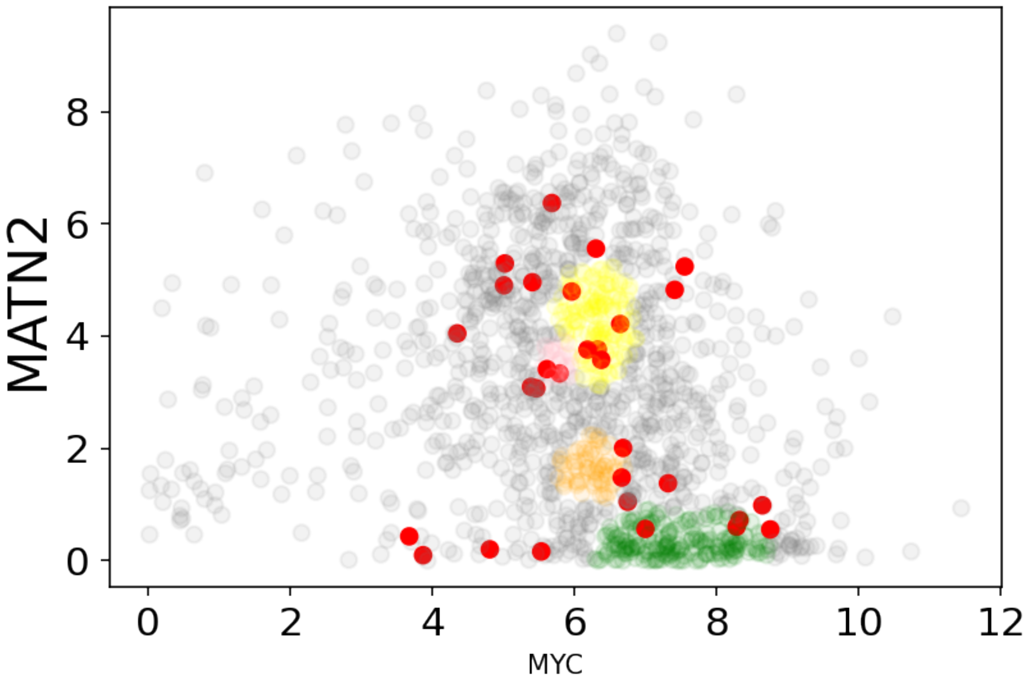

MATN2
8
6
4
2
0
0
2
4
6
8
10
12
MYC

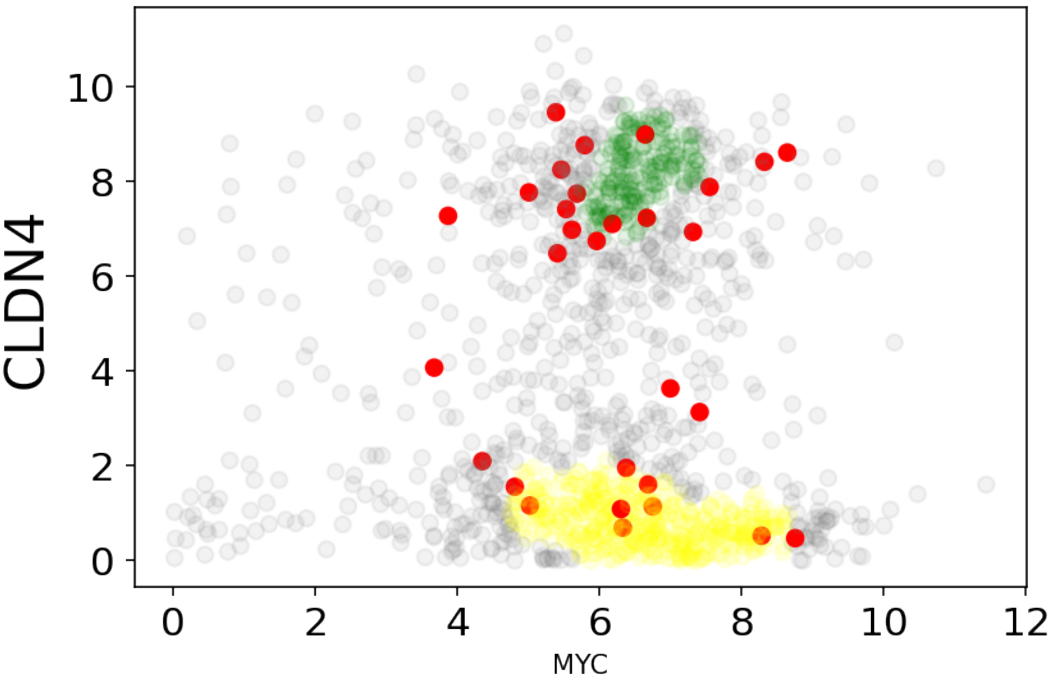

CLDN4
MYC
0
2
4
6
8
10
12

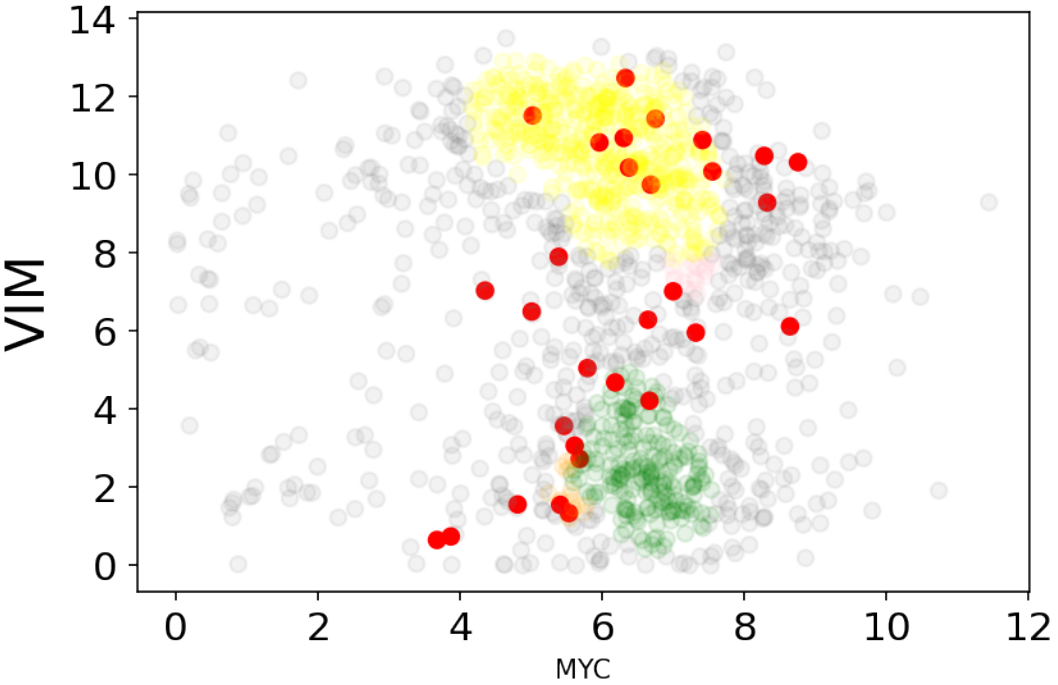
VIM
MYC
0
2
4
6
8
10
12
14

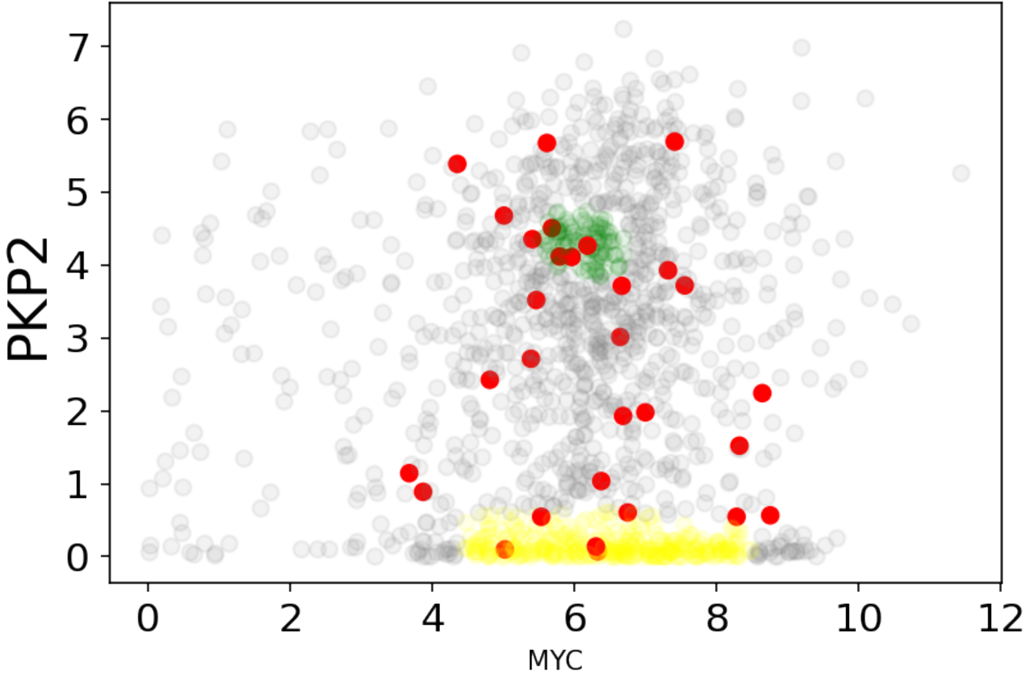
PKP2
MYC
0
1
2
3
4
5
6
7
0
2
4
6
8
10
12

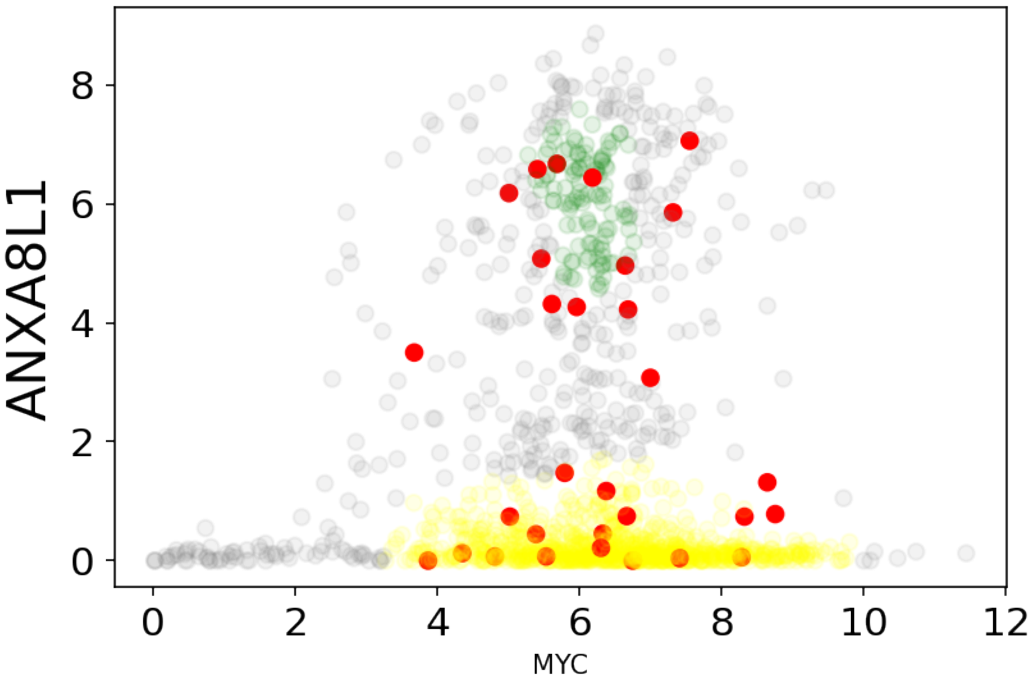
ANXA8L1
MYC
0
2
4
6
8
0
2
4
6
8
10
12

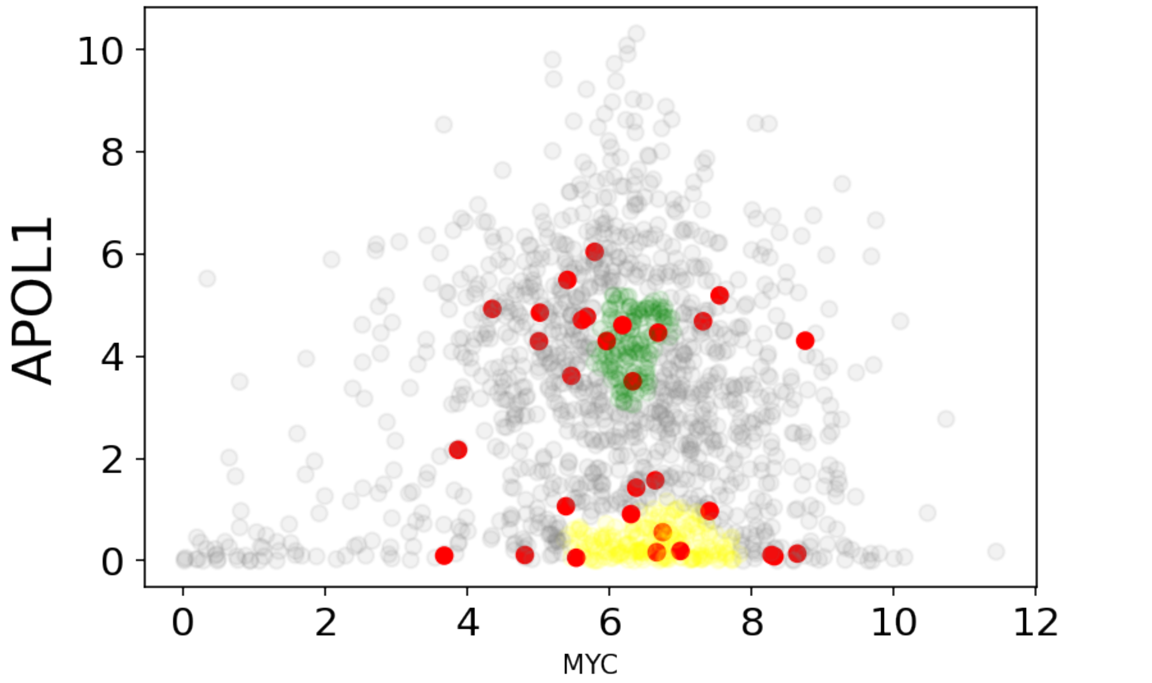
APOL1
10
8
6
4
2
0
0
2
4
6
8
10
12
MYC

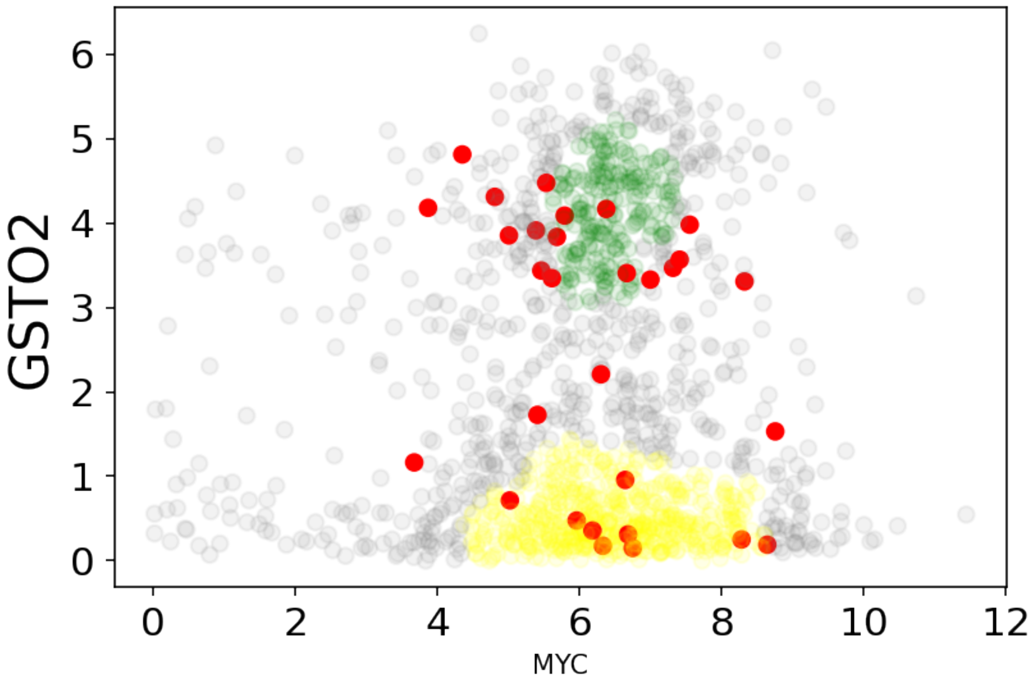
GSTO2
MYC
0
1
2
3
4
5
6
0
2
4
6
8
10
12

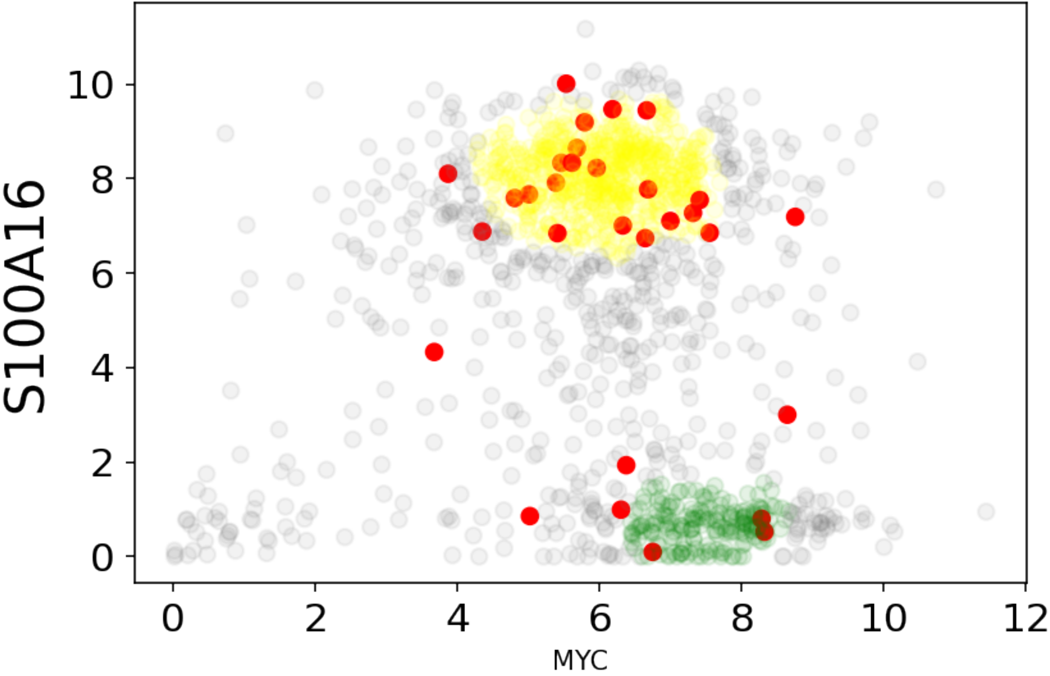

S100A16
10
8
6
4
2
0
0
2
4
6
8
10
12
MYC

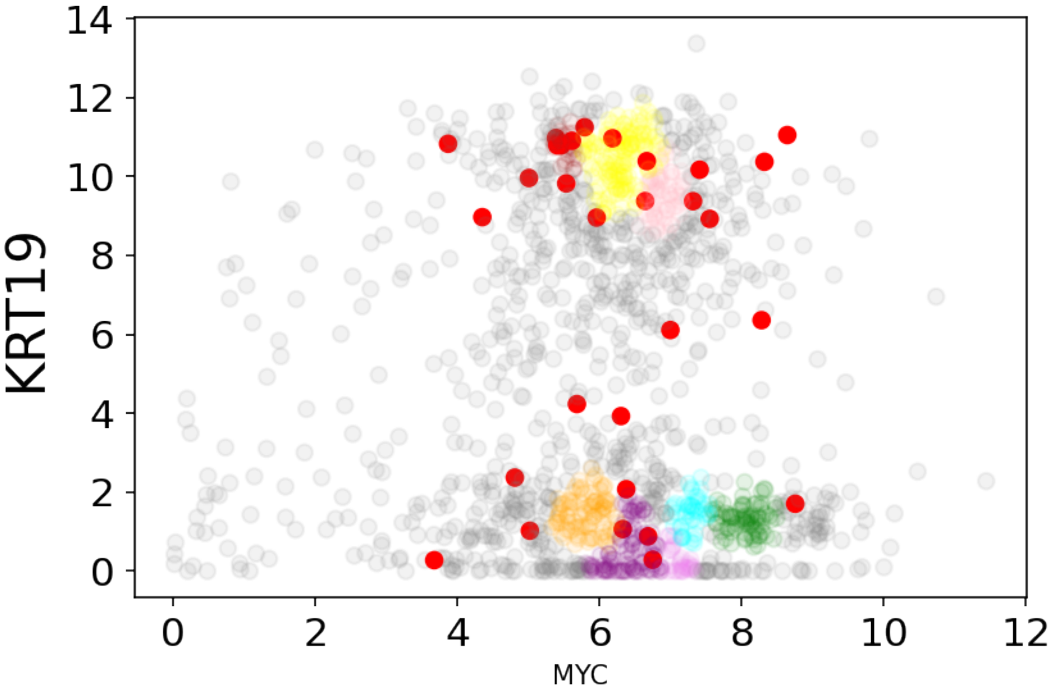

KRT19
MYC
0
2
4
6
8
10
12
14

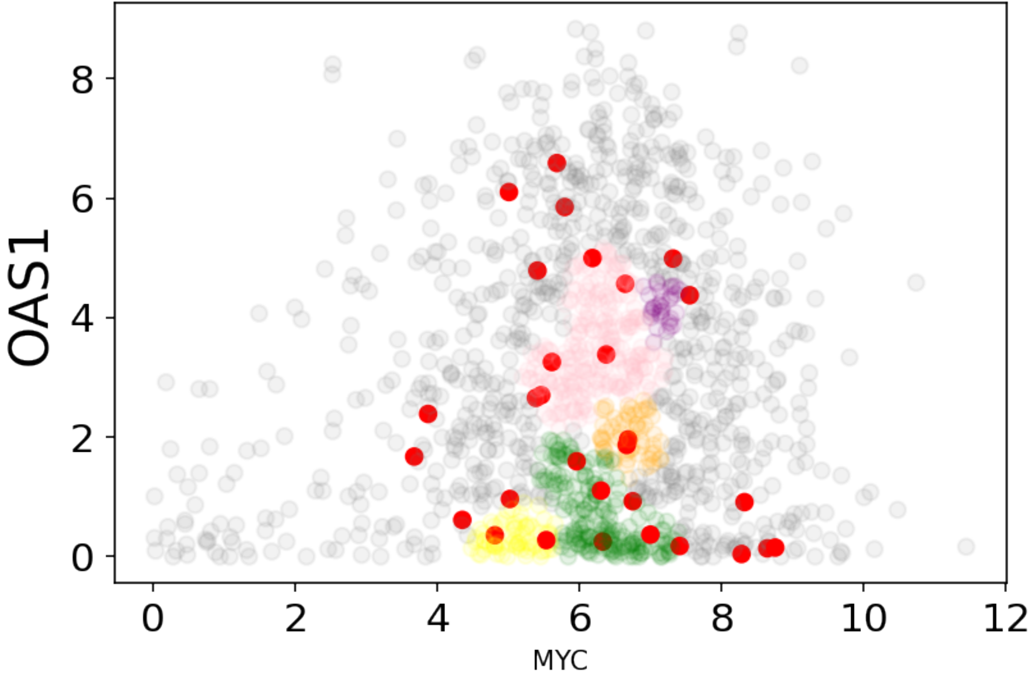

OAS1
MYC
0
2
4
6
8
10
12

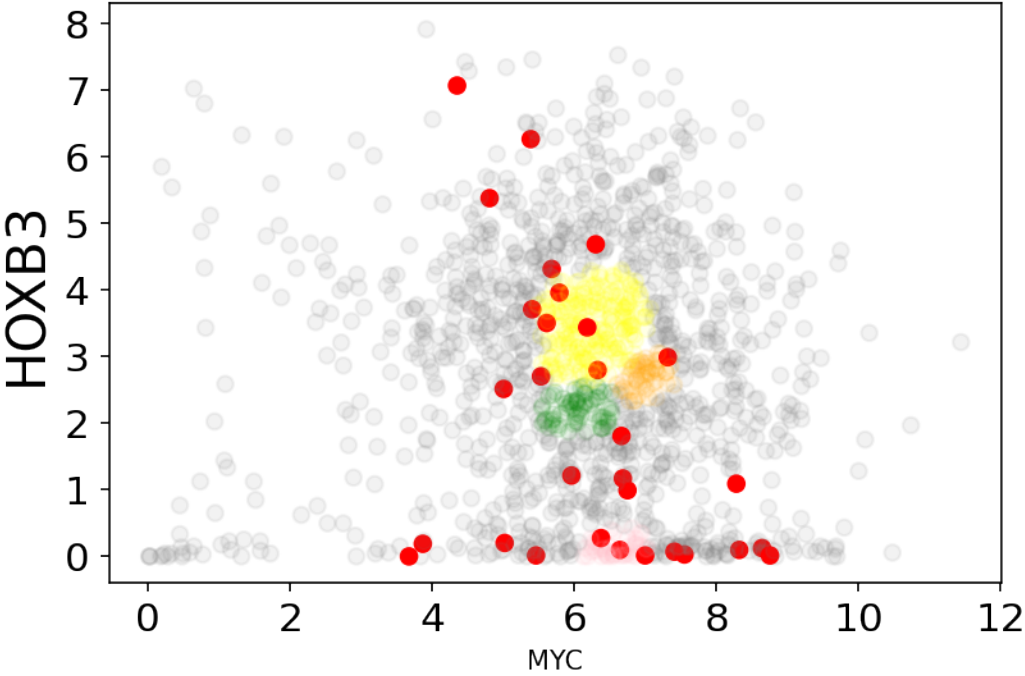

HOXB3
MYC
0
1
2
3
4
5
6
7
8
0
2
4
6
8
10
12

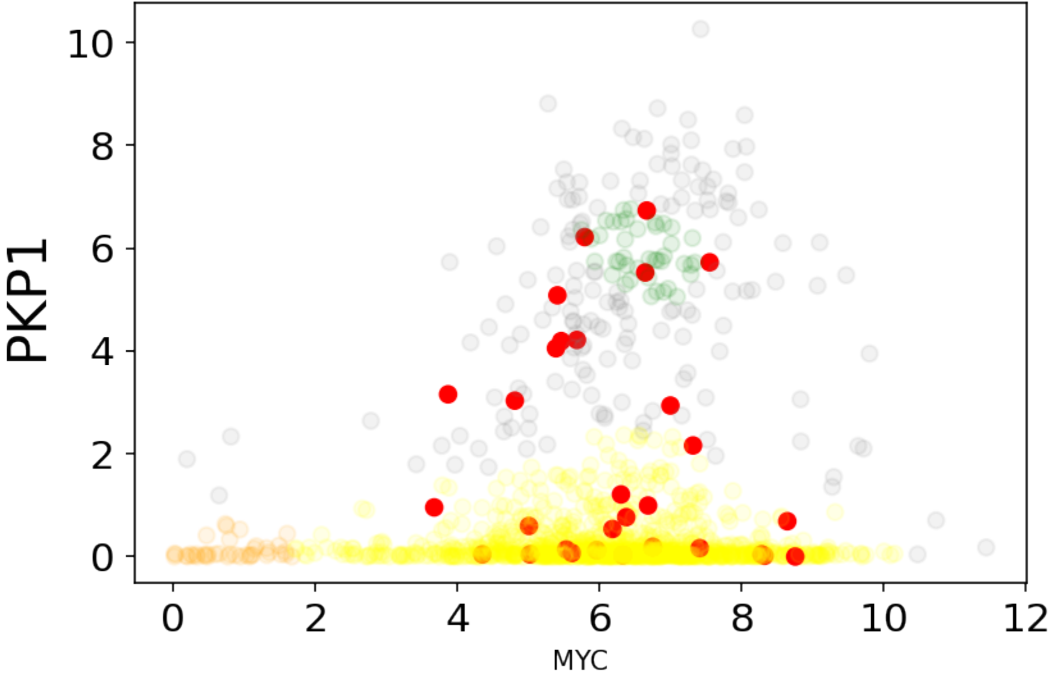
PKP1
MYC
0
2
4
6
8
10
12

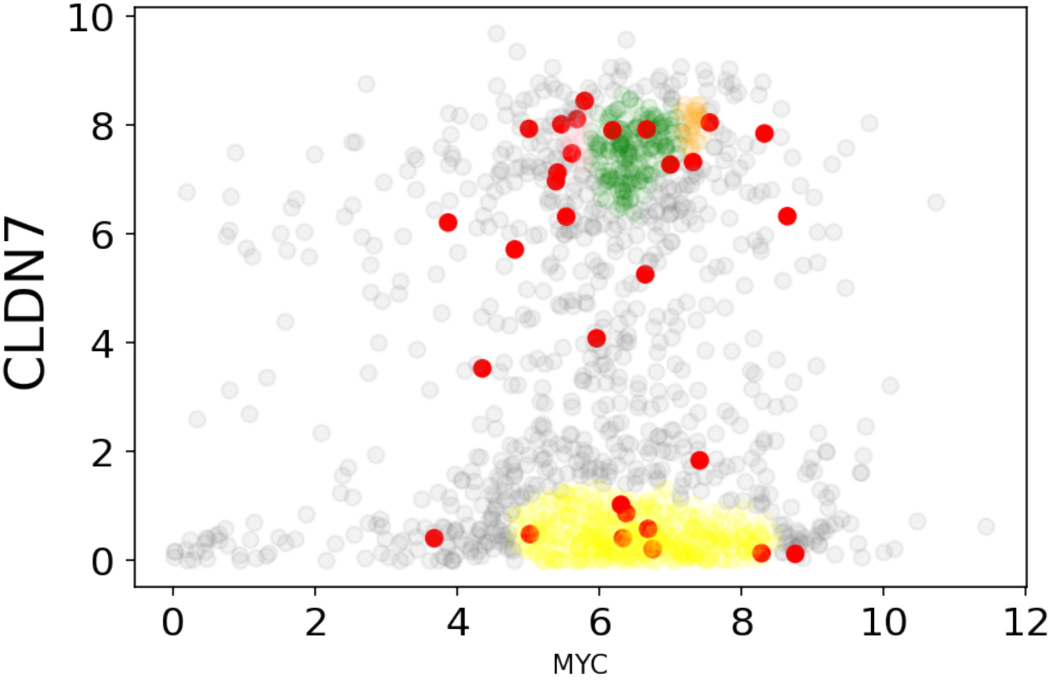

CLDN7
MYC
10
8
6
4
2
0
0
2
4
6
8
10
12

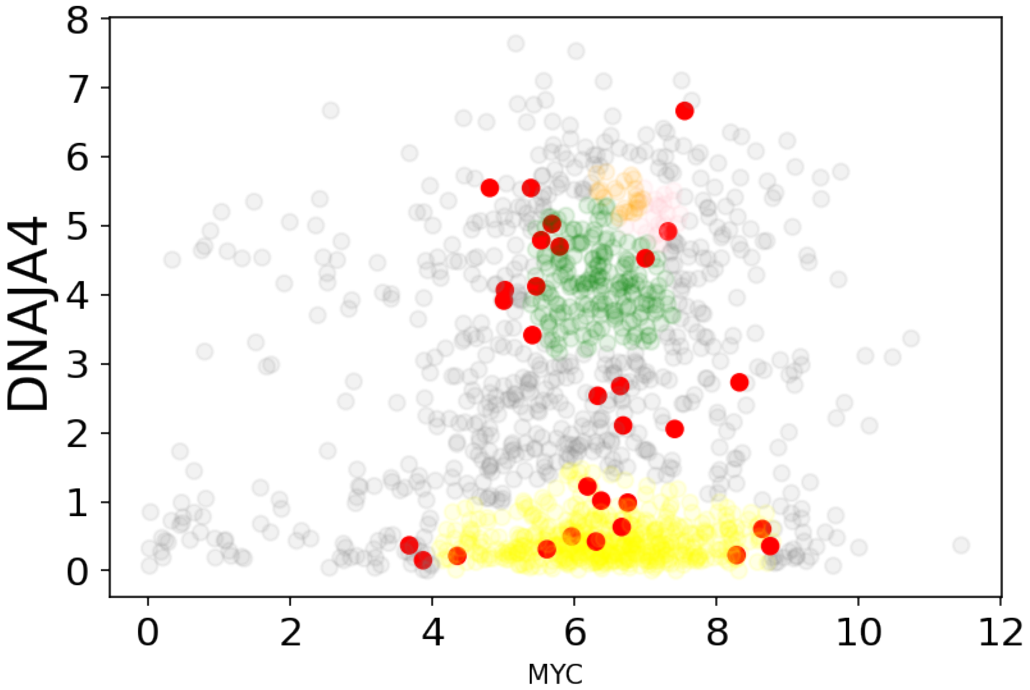

DNAJA4
MYC
0
1
2
3
4
5
6
7
8
0
2
4
6
8
10
12

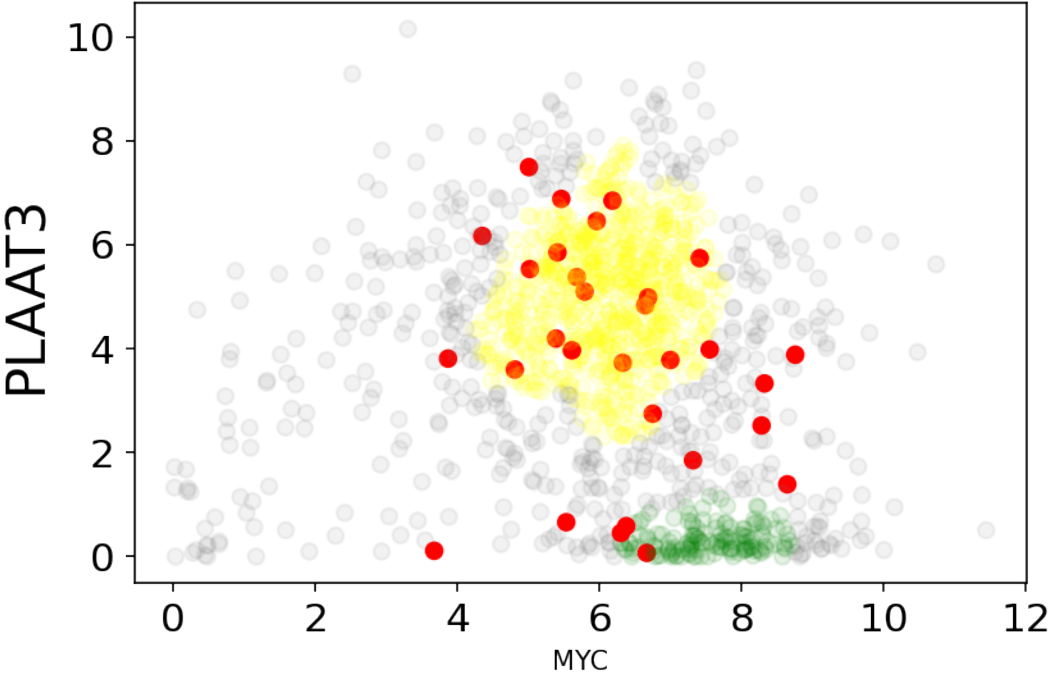
PLAAT3
10
8
6
4
2
0
0
2
4
6
8
10
12
MYC

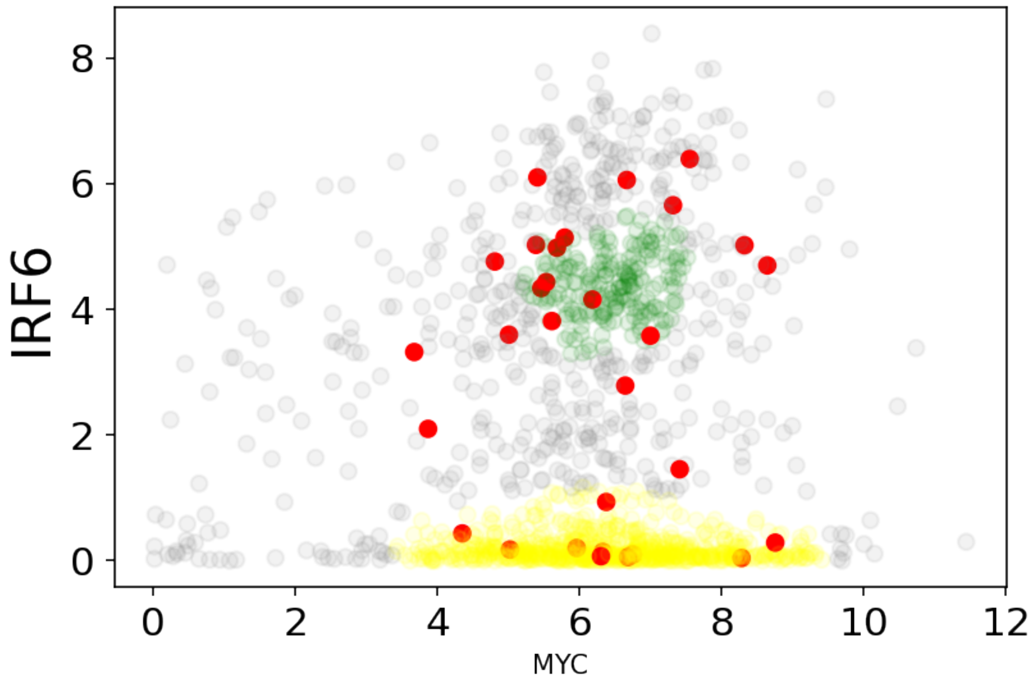
IRF6
MYC
0
2
4
6
8
10
12

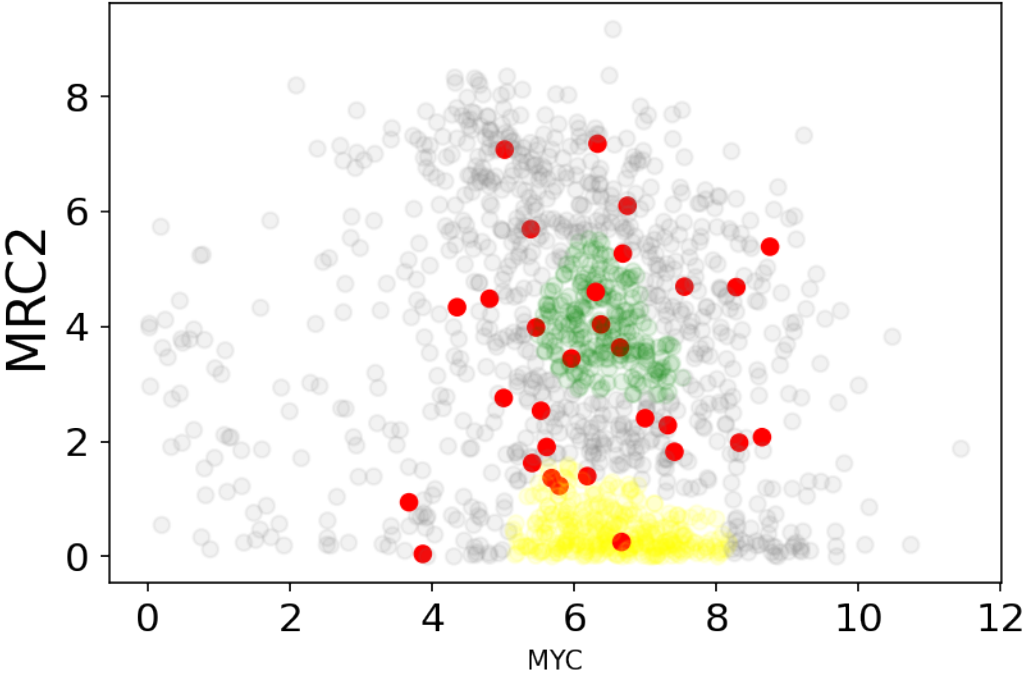

MRC2
8
6
4
2
0
0
2
4
6
8
10
12
MYC

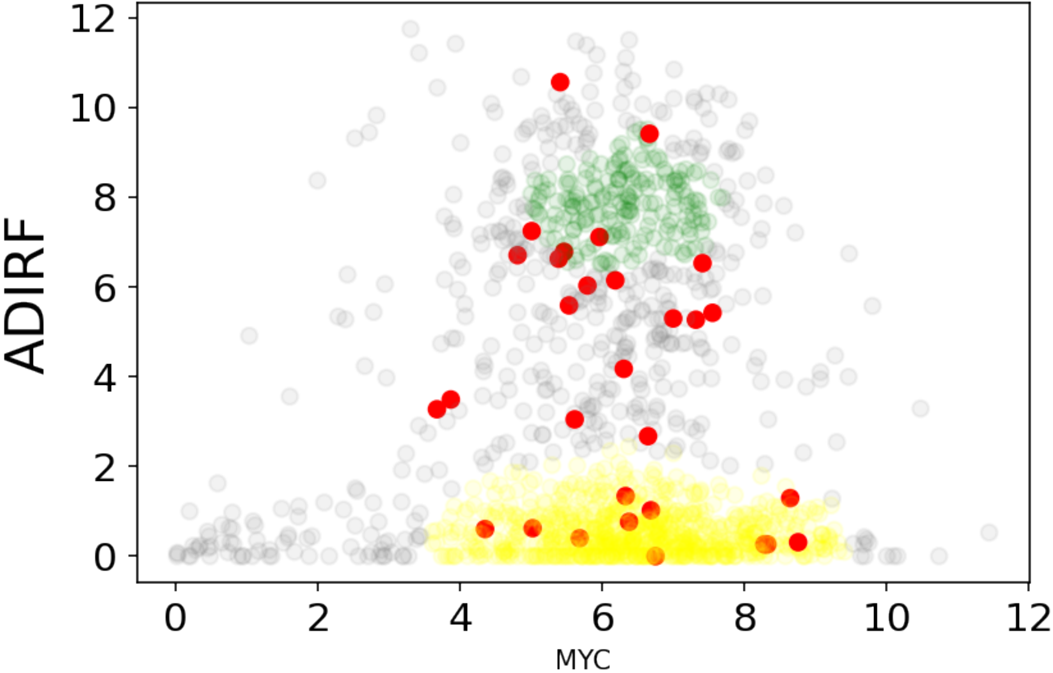

ADIRF
MYC
0
2
4
6
8
10
12

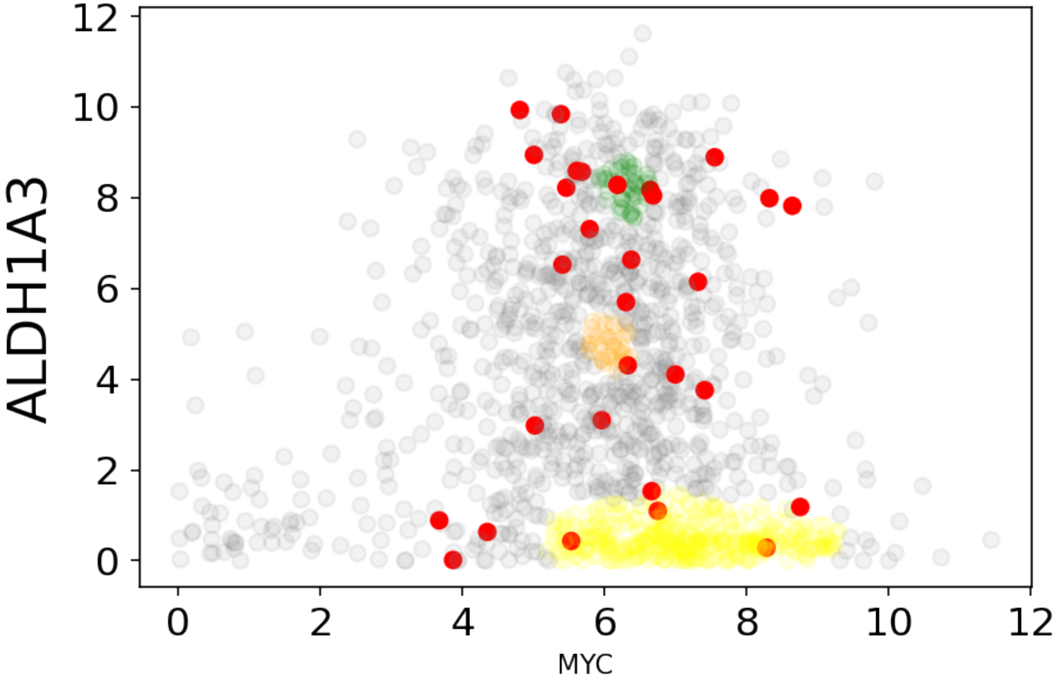

ALDH1A3
MYC
0
2
4
6
8
10
12

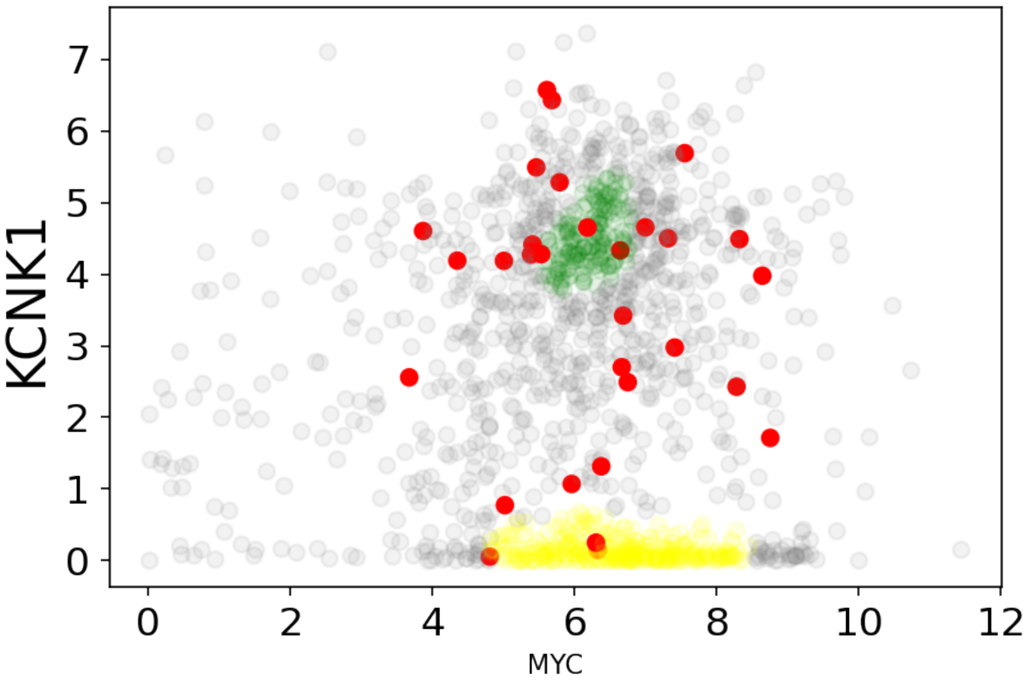

KCNK1
MYC
0
1
2
3
4
5
6
7
0
2
4
6
8
10
12

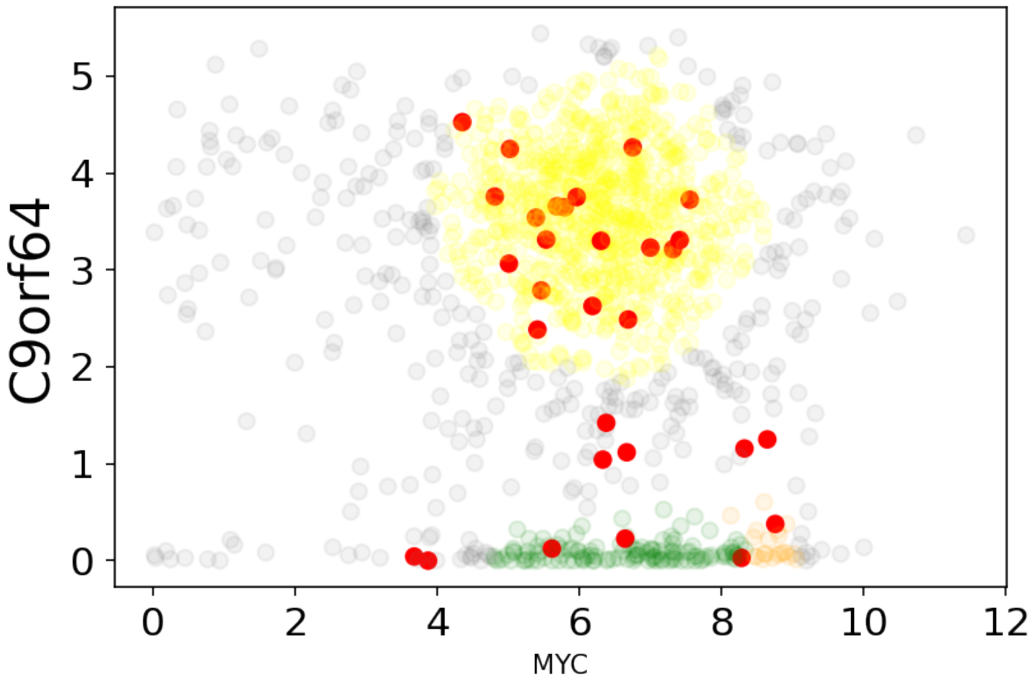
C9orf64
MYC
0
1
2
3
4
5
0
2
4
6
8
10
12

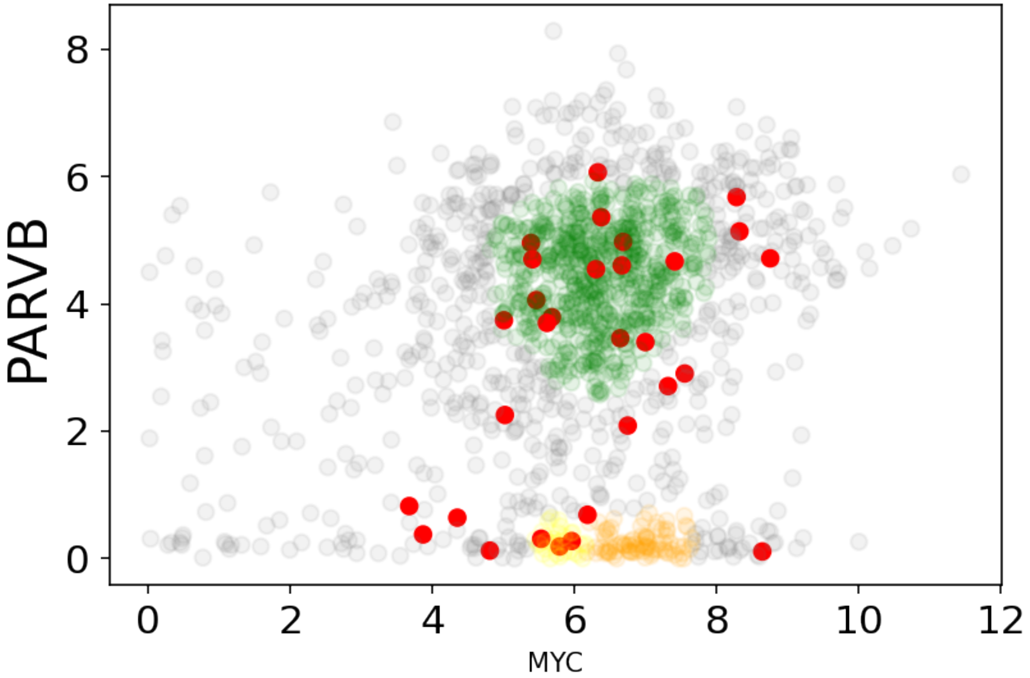

PARVB
MYC
0
2
4
6
8
0
2
4
6
8
10
12

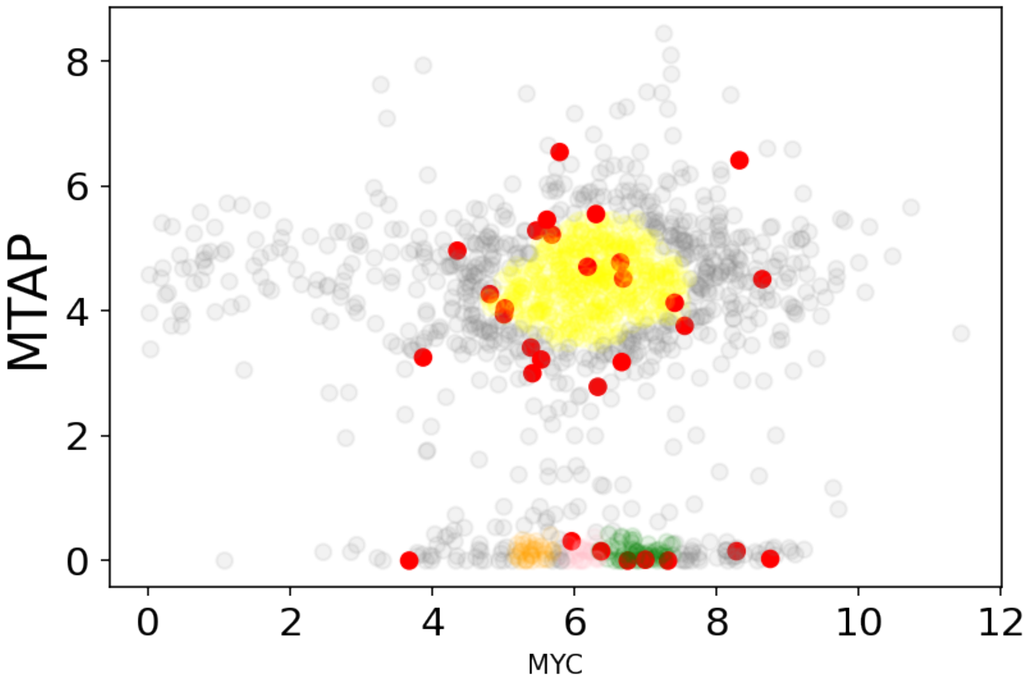

MTAP
8
6
4
2
0
0
2
4
6
8
10
12
MYC

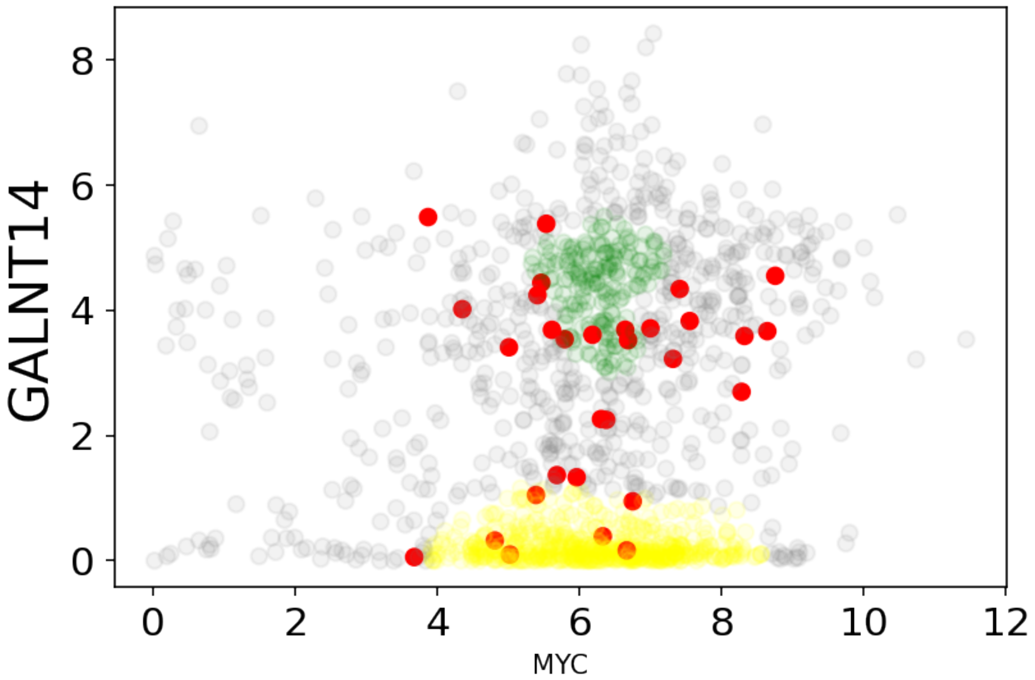

GALNT14
MYC
0
2
4
6
8
0
2
4
6
8
10
12

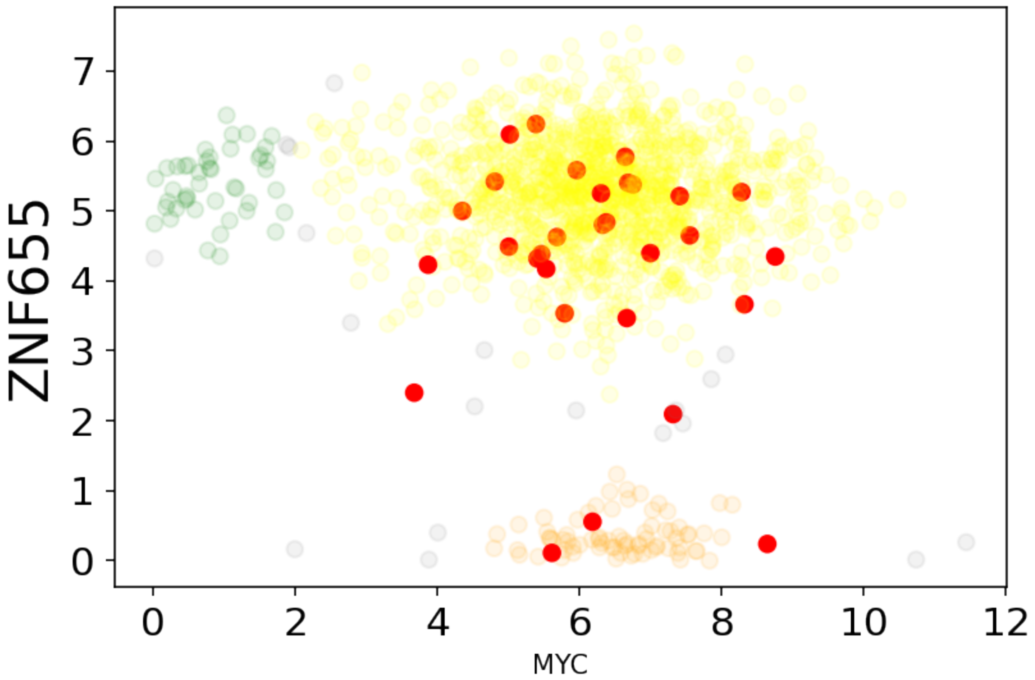

ZNF655
MYC
0
1
2
3
4
5
6
7
0
2
4
6
8
10
12

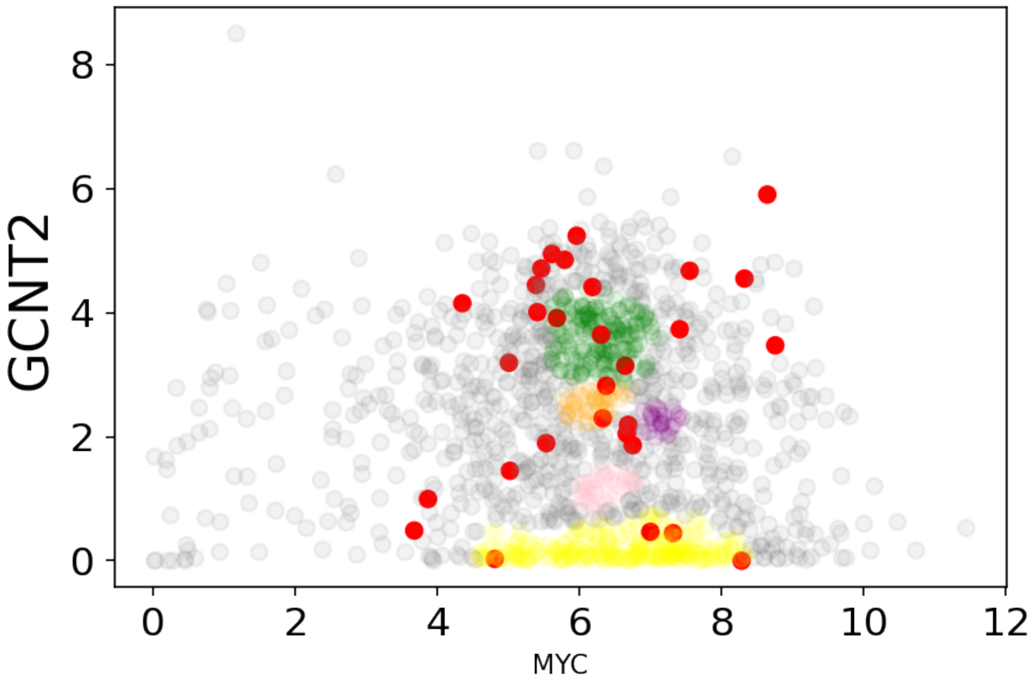

GCNT2
MYC
0
2
4
6
8
10
12

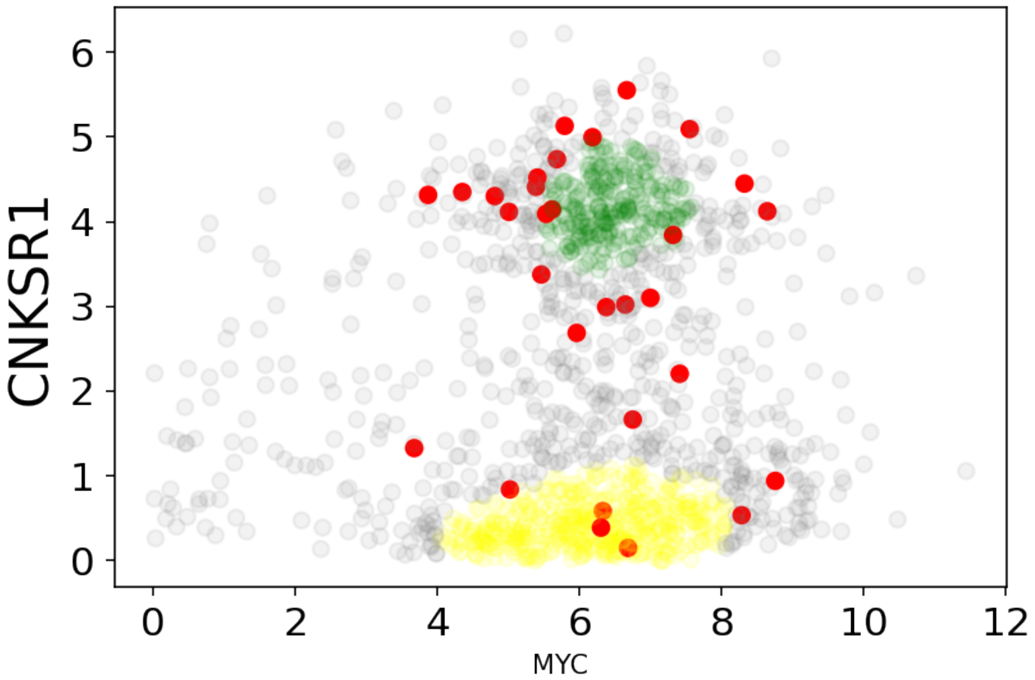

CNKSR1
MYC
0
1
2
3
4
5
6
0
2
4
6
8
10
12

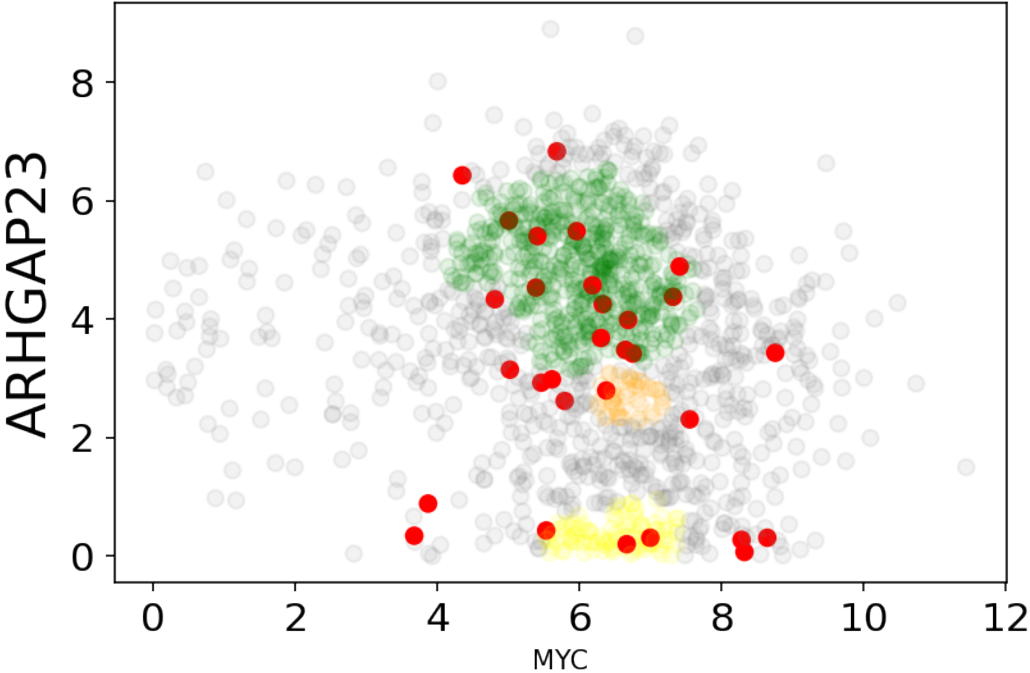
ARHGAP23
0
2
4
6
8
0
2
4
6
8
10
12
MYC

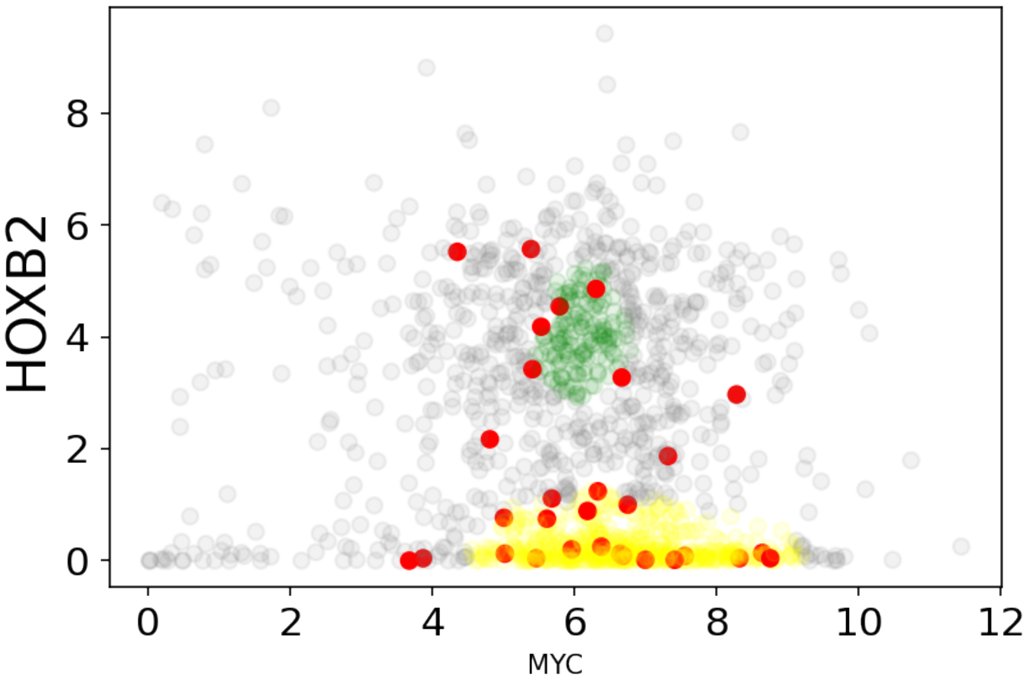

HOXB2
8
6
4
2
0
0
2
4
6
8
10
12
MYC

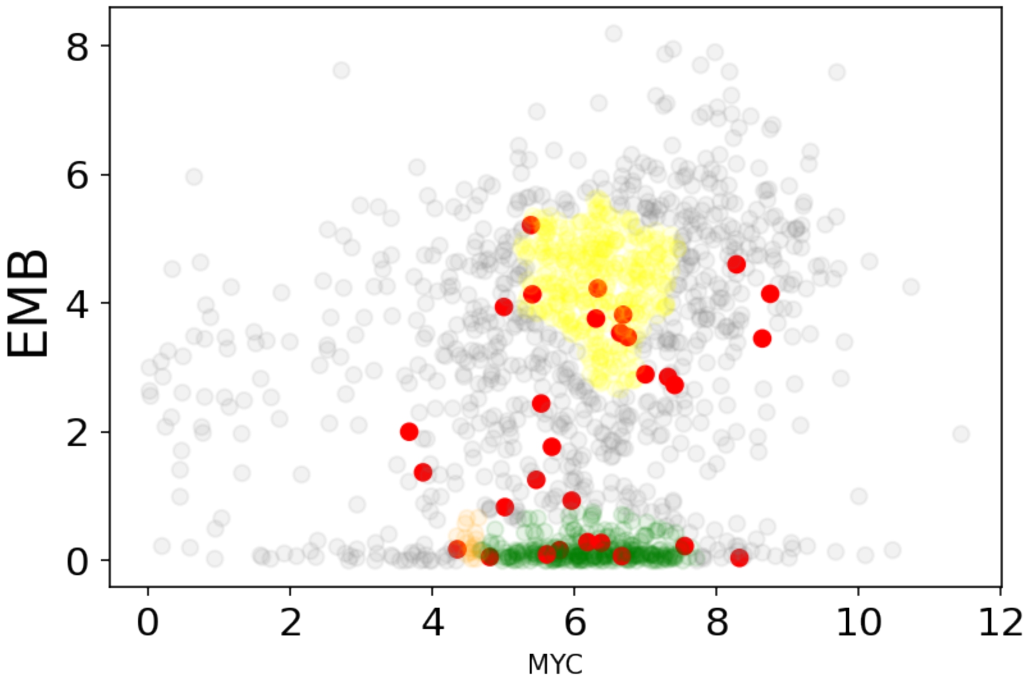
EMB
8
6
4
2
0
0
2
4
6
8
10
12
MYC

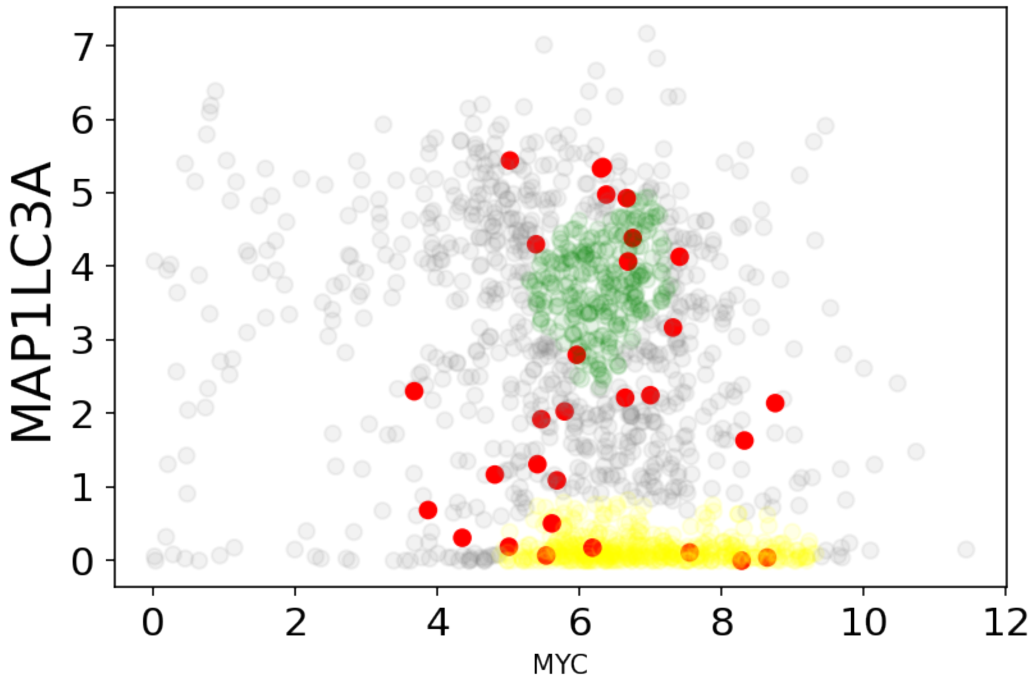

MAP1LC3A
0
1
2
3
4
5
6
7
0
2
4
6
8
10
12
MYC

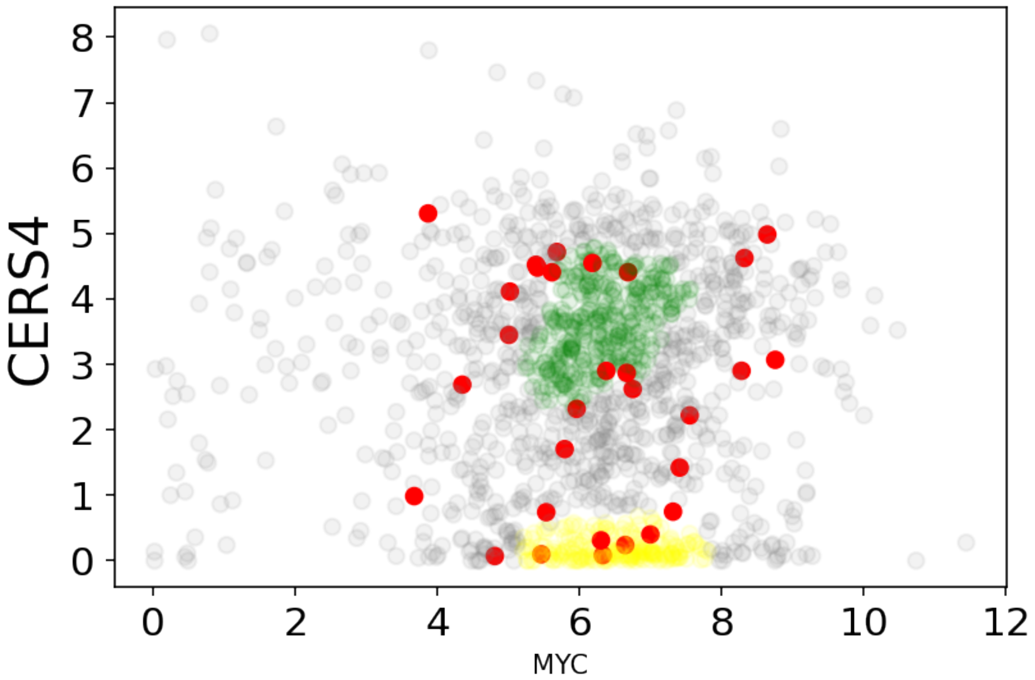
CERS4
MYC
0
1
2
3
4
5
6
7
8
0
2
4
6
8
10
12

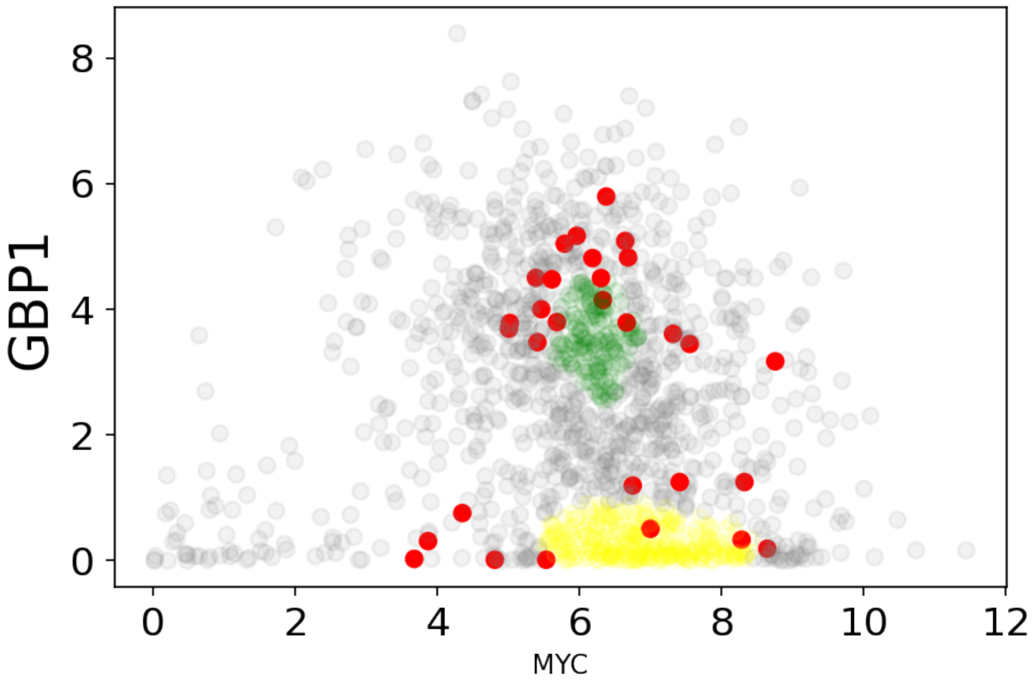

GBP1
MYC
0
2
4
6
8
0
2
4
6
8
10
12

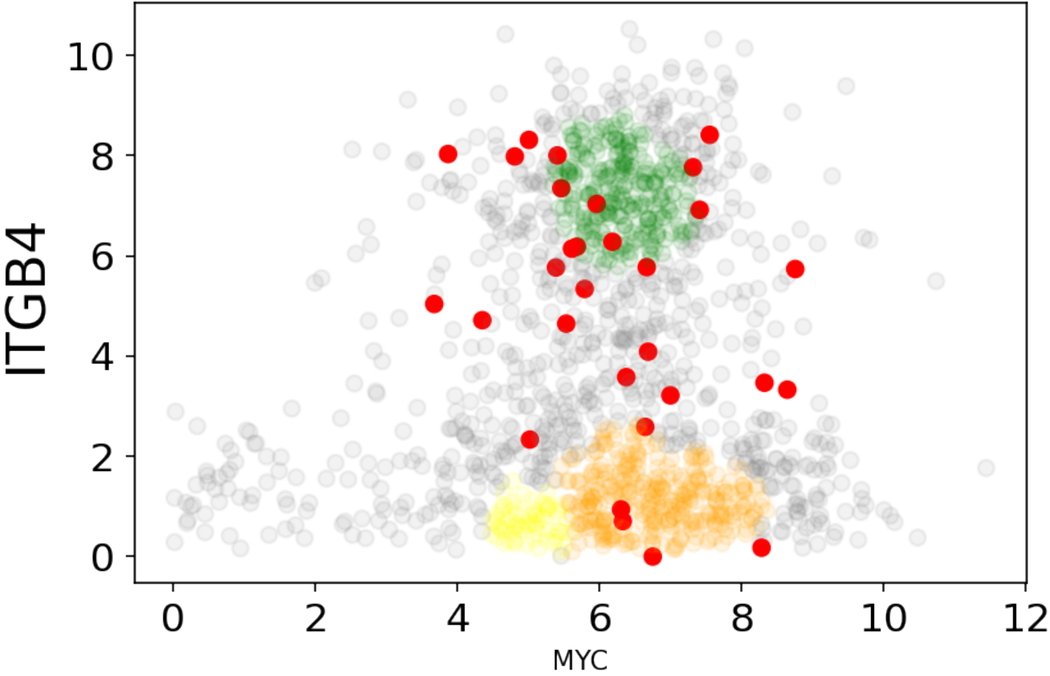

ITGB4
MYC
10
8
6
4
2
0
0
2
4
6
8
10
12

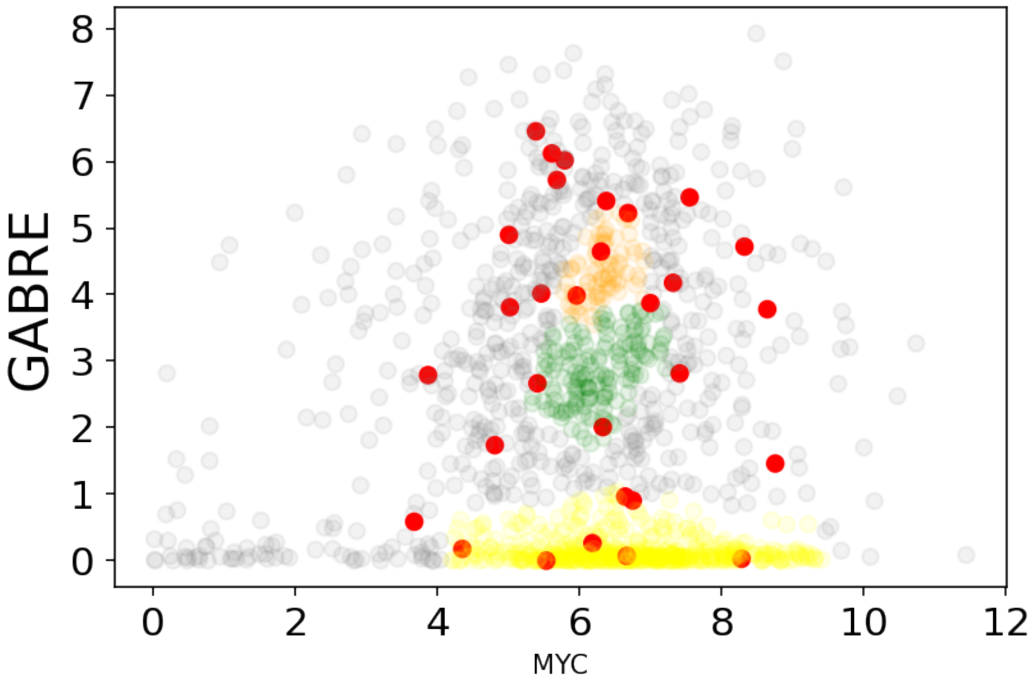

GABRE
MYC
0
1
2
3
4
5
6
7
8
0
2
4
6
8
10
12

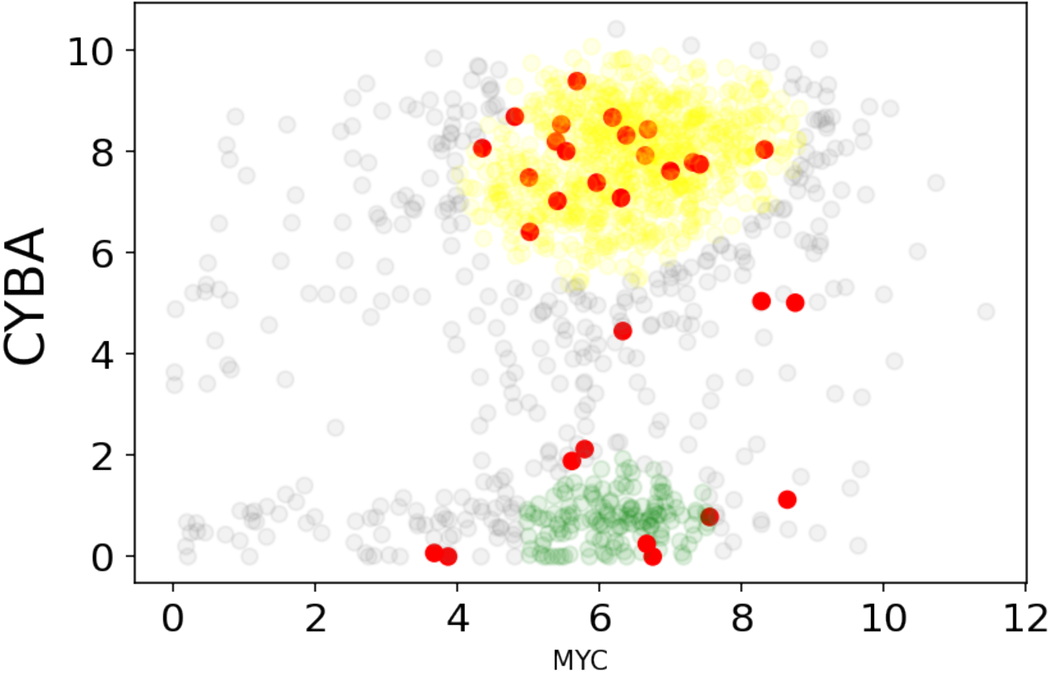

CYBA
MYC
0
2
4
6
8
10
12

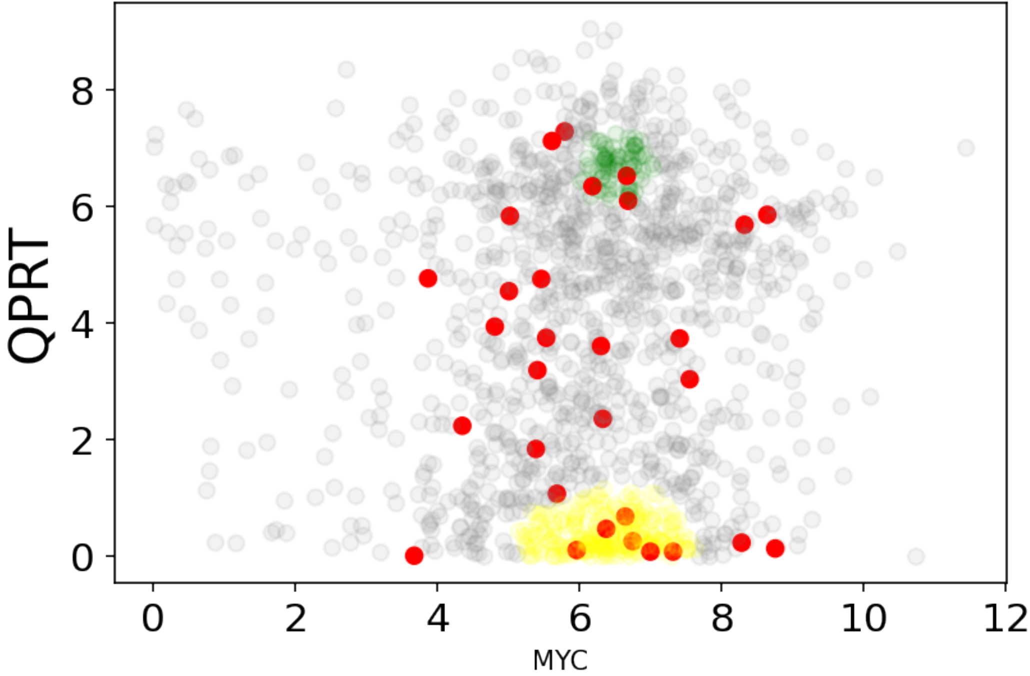
QPRT
MYC
0
2
4
6
8
0
2
4
6
8
10
12

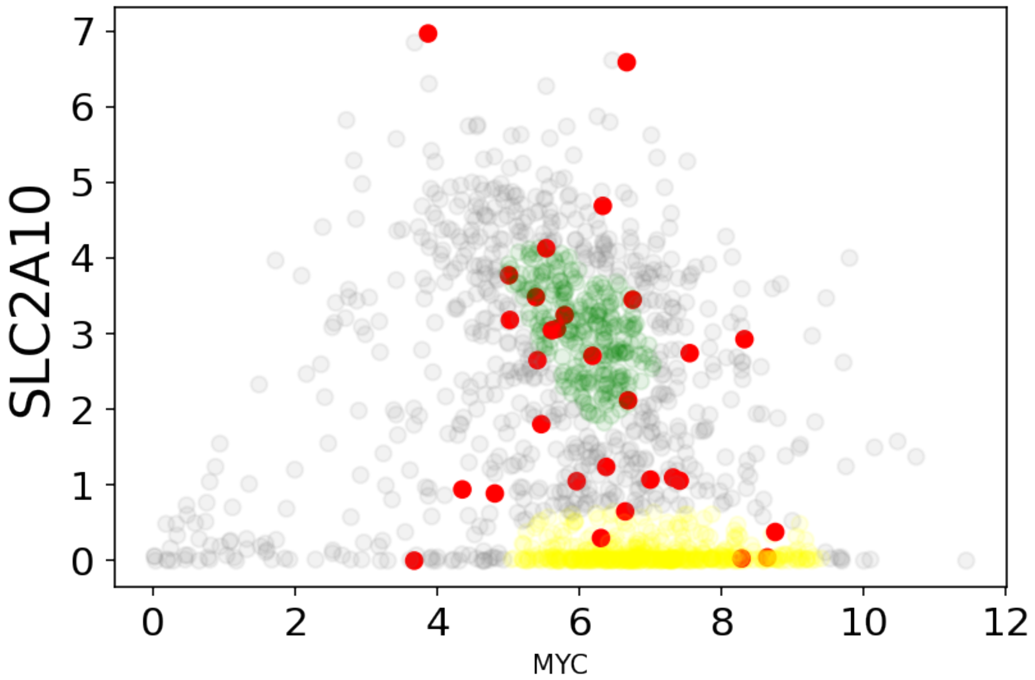

SLC2A10
MYC
0
1
2
3
4
5
6
7
0
2
4
6
8
10
12

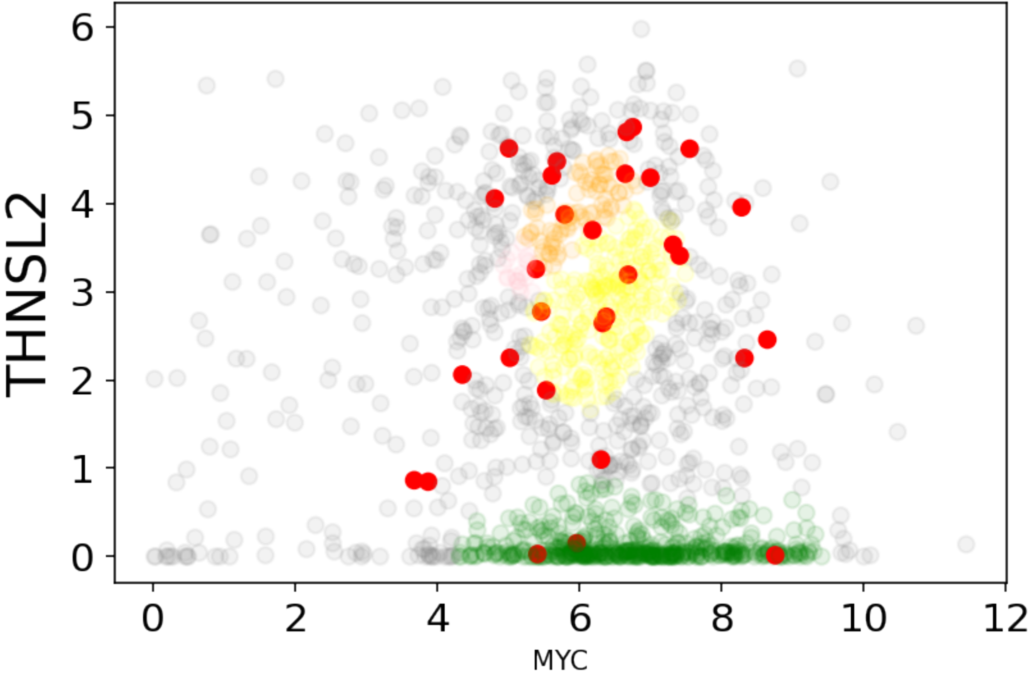

THNSL2
MYC
0
1
2
3
4
5
6
0
2
4
6
8
10
12

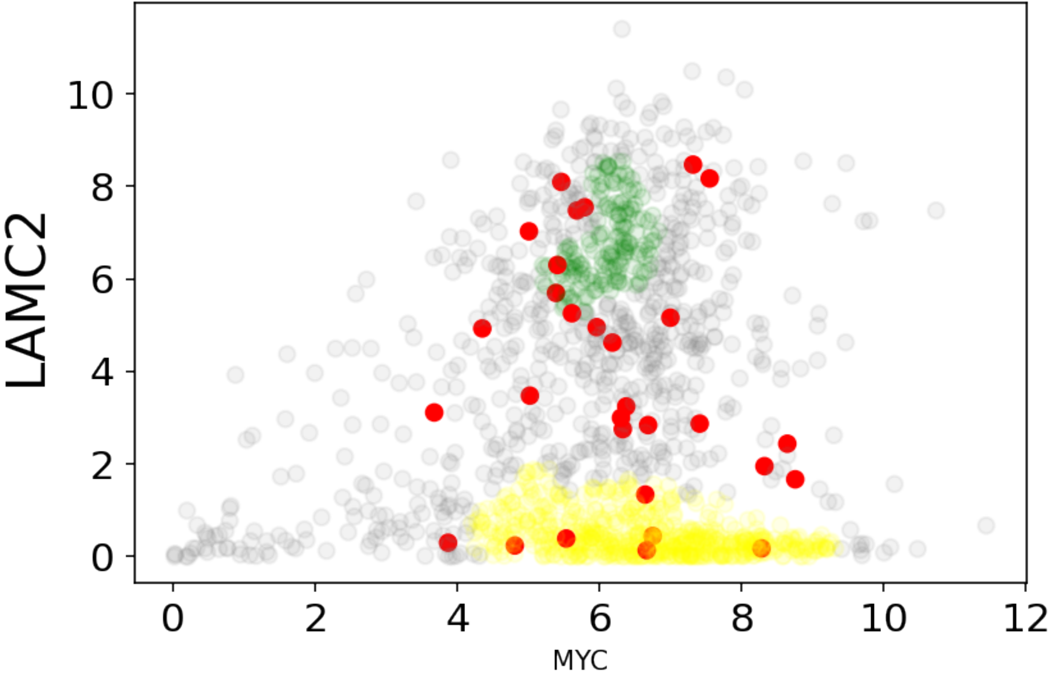

LAMC2
10
8
6
4
2
0
0
2
4
6
8
10
12
MYC

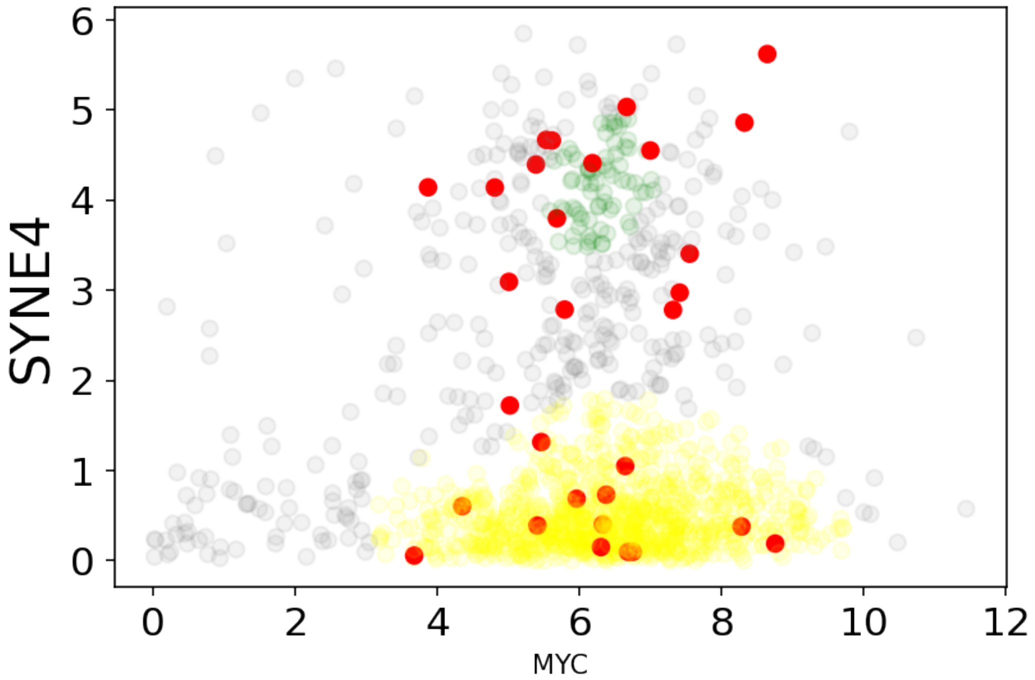

SYNE4
MYC
0
1
2
3
4
5
6
0
2
4
6
8
10
12

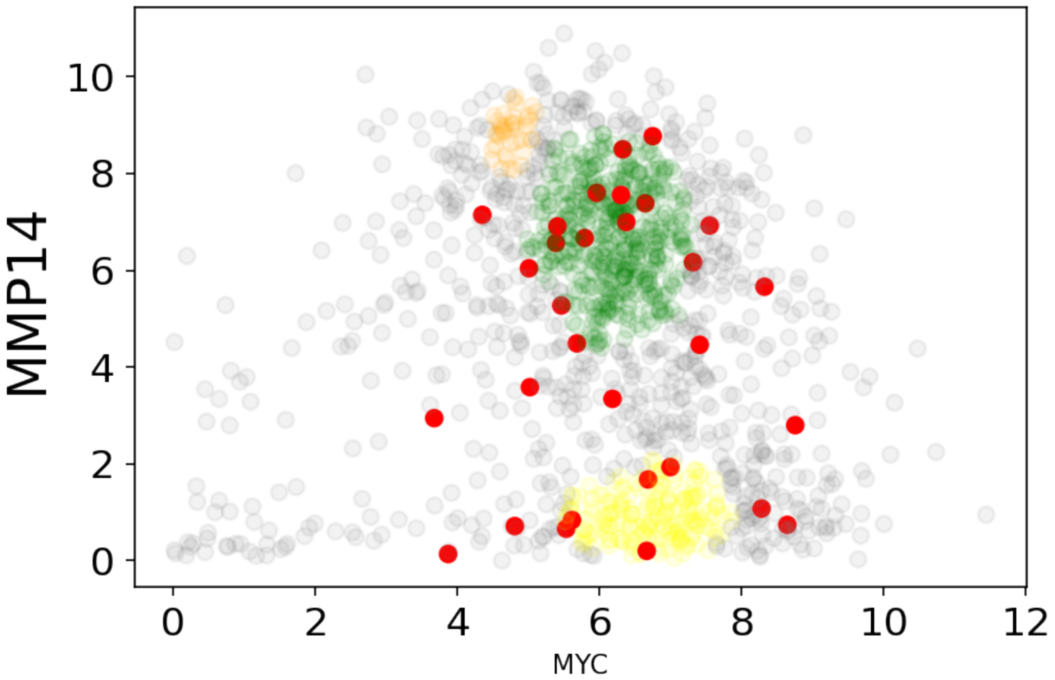
MMP14
MYC
0
2
4
6
8
10
12

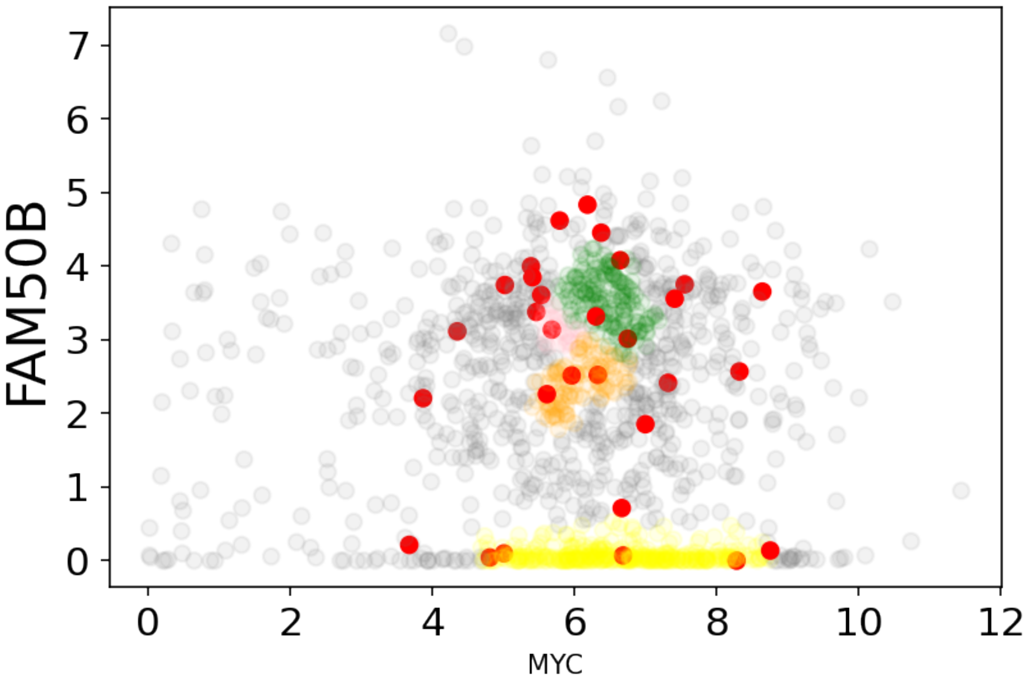

FAM50B
0
1
2
3
4
5
6
7
0
2
4
6
8
10
12
MYC

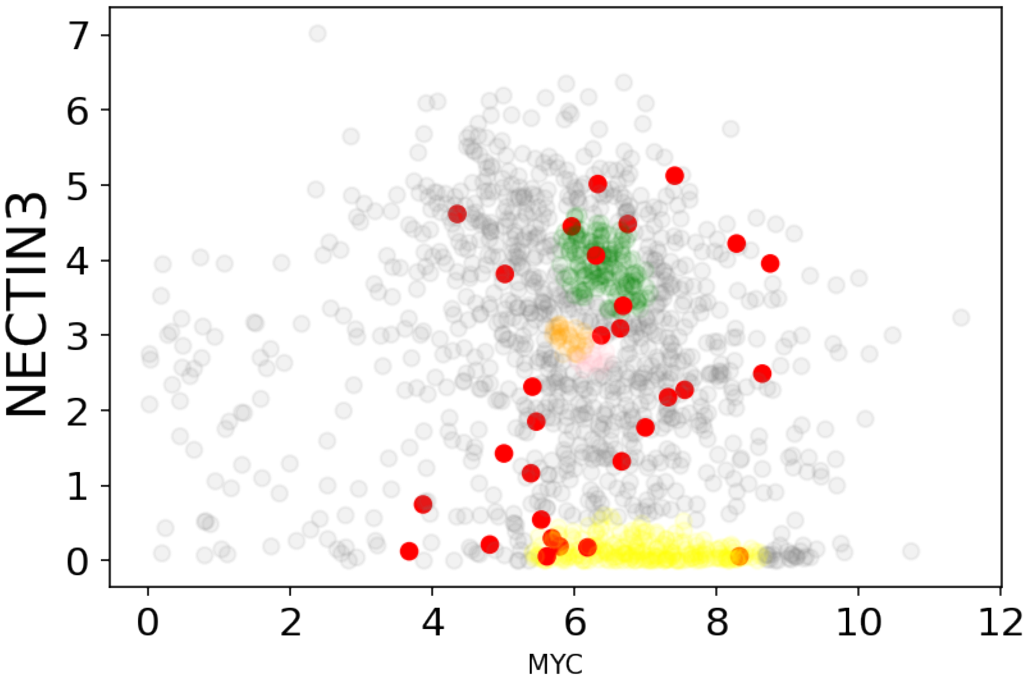
NECTIN3
7
6
5
4
3
2
1
0
0
2
4
6
8
10
12
MYC

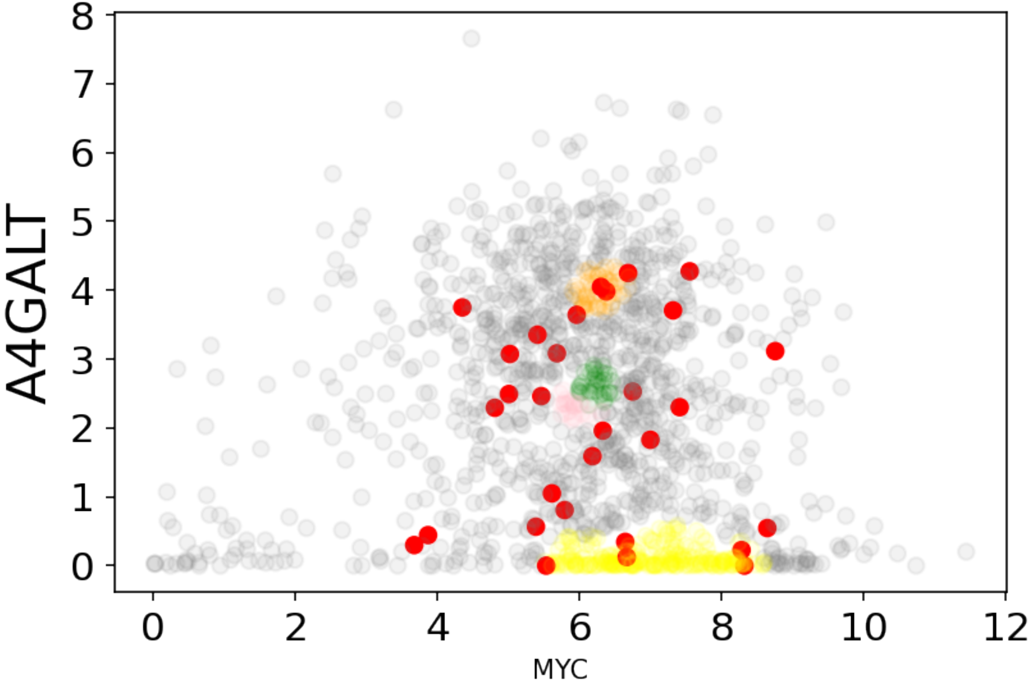

A4GALT
MYC
0
1
2
3
4
5
6
7
8
0
2
4
6
8
10
12

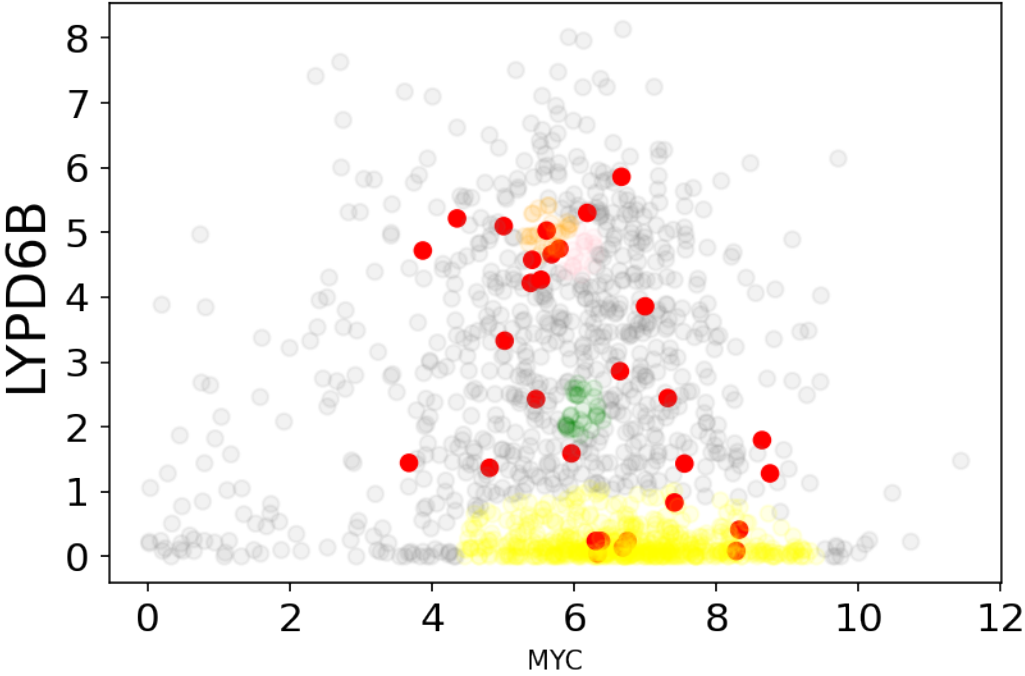

LYPD6B
0
1
2
3
4
5
6
7
8
0
2
4
6
8
10
12
MYC

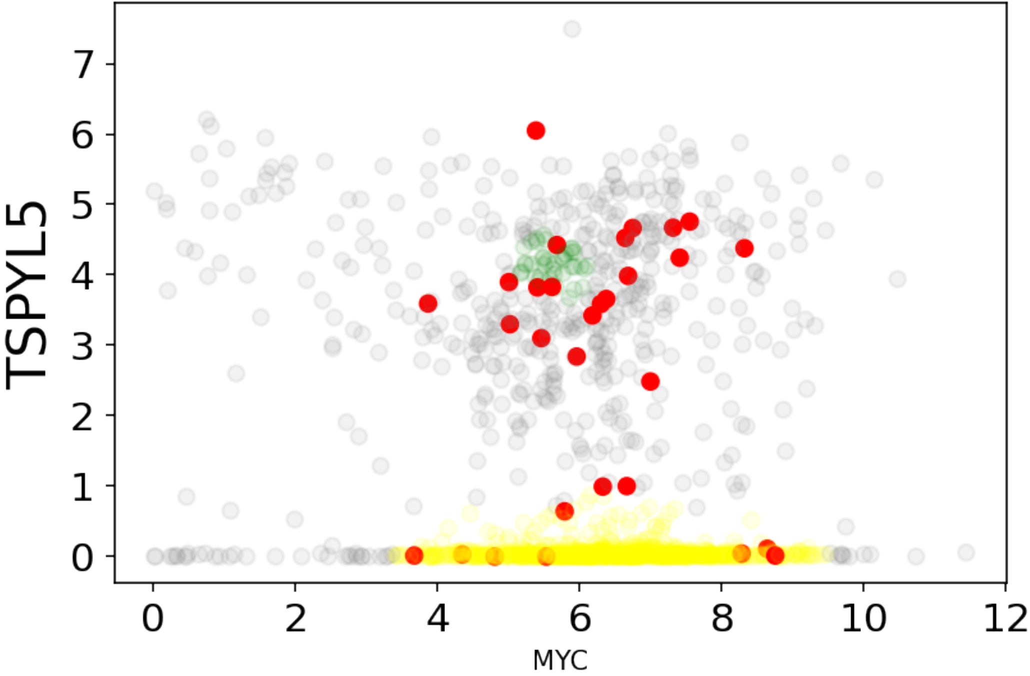

TSPYL5
MYC
0
1
2
3
4
5
6
7
0
2
4
6
8
10
12

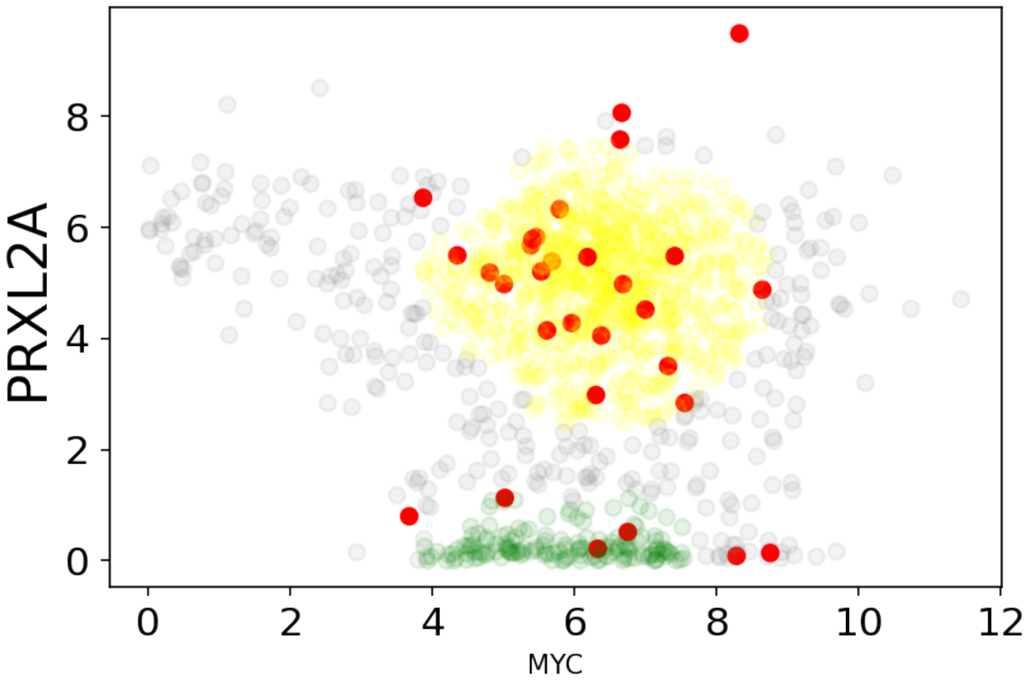
PRXL2A
MYC
0
2
4
6
8
0
2
4
6
8
10
12

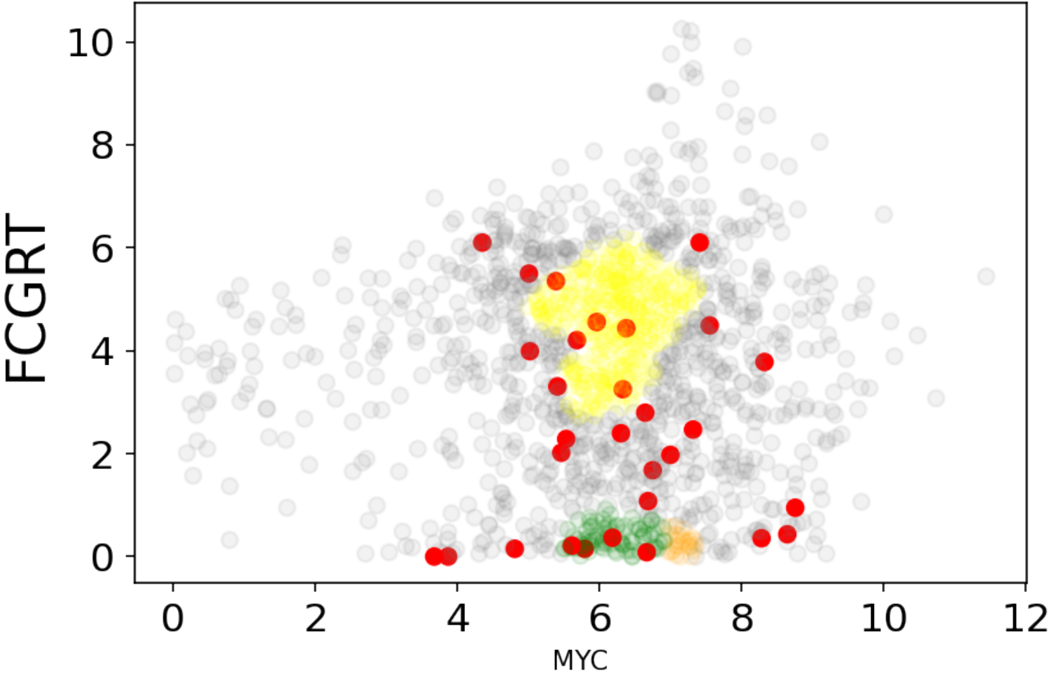
FCGRT
MYC
0
2
4
6
8
10
12

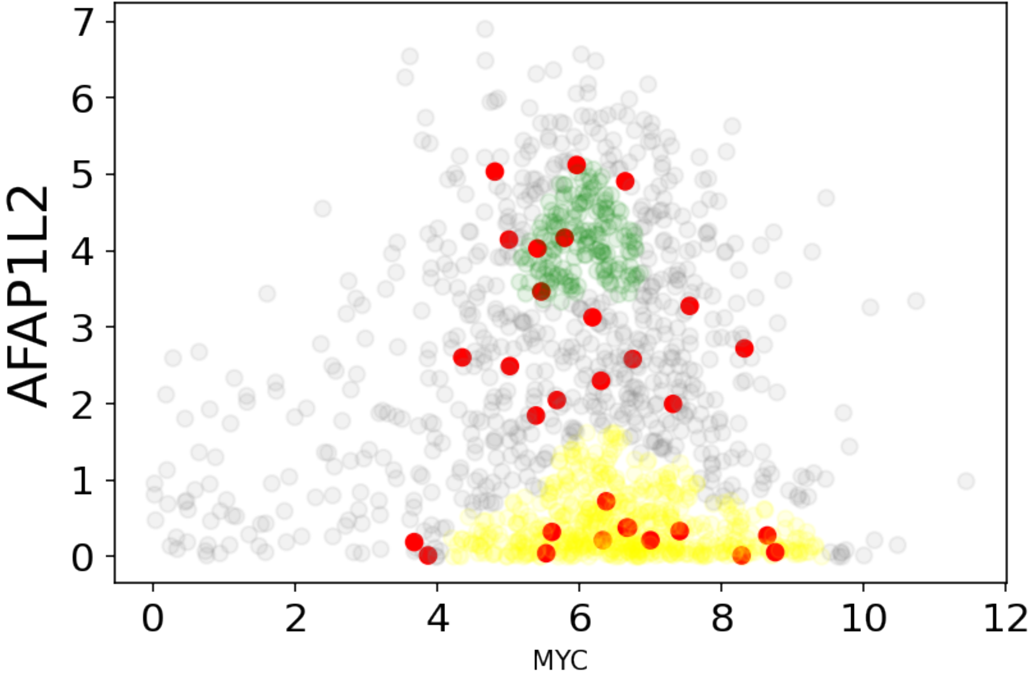

AFAP1L2
MYC
0
1
2
3
4
5
6
7
0
2
4
6
8
10
12

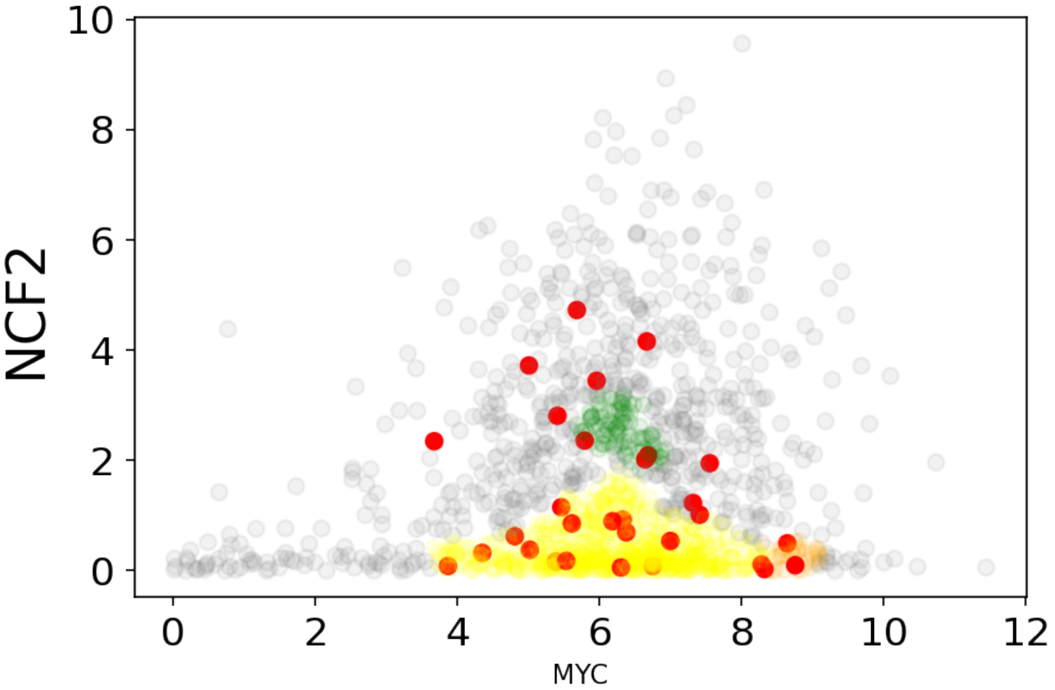
NCF2
0
2
4
6
8
10
0
2
4
6
8
10
12
MYC

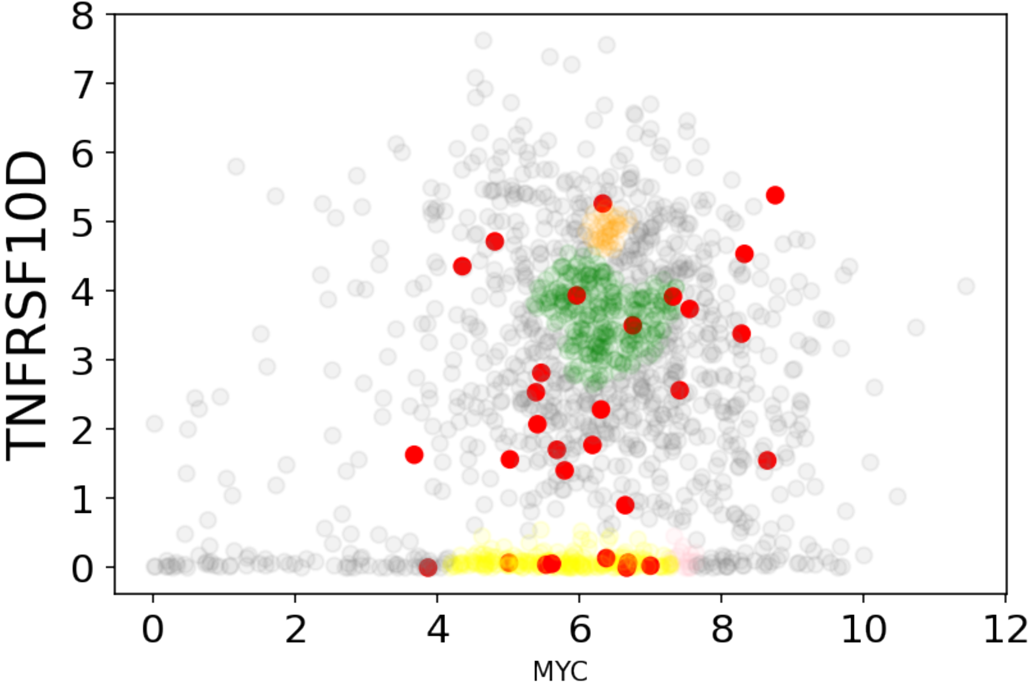

TNFRSF10D
MYC
0
1
2
3
4
5
6
7
8
0
2
4
6
8
10
12

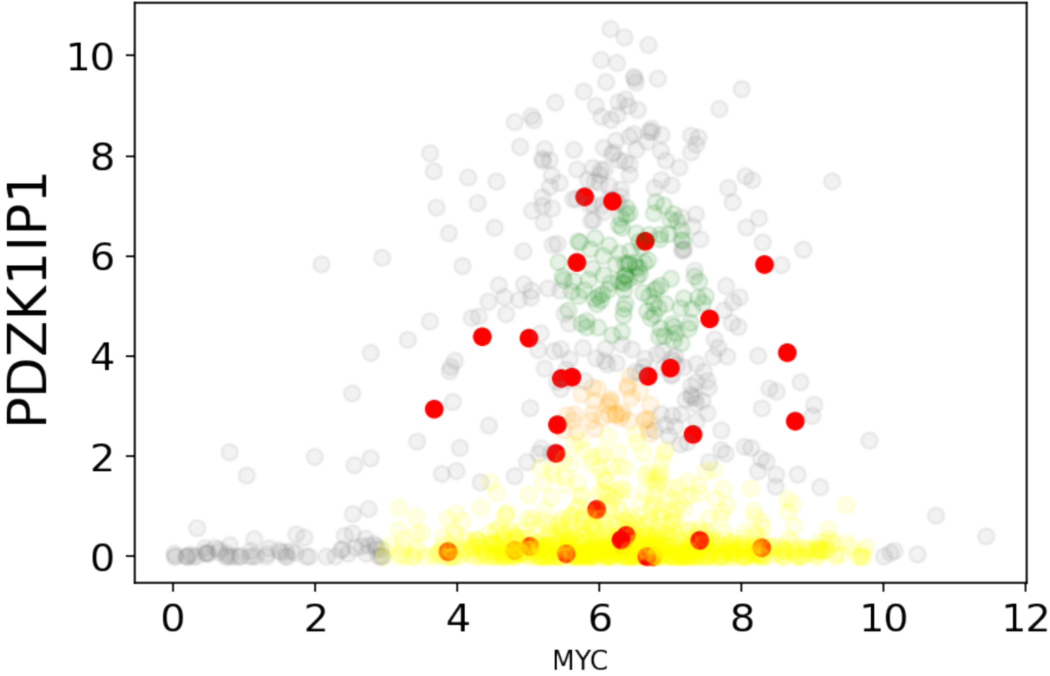

PDZK1IP1
MYC
0
2
4
6
8
10
0
2
4
6
8
10
12

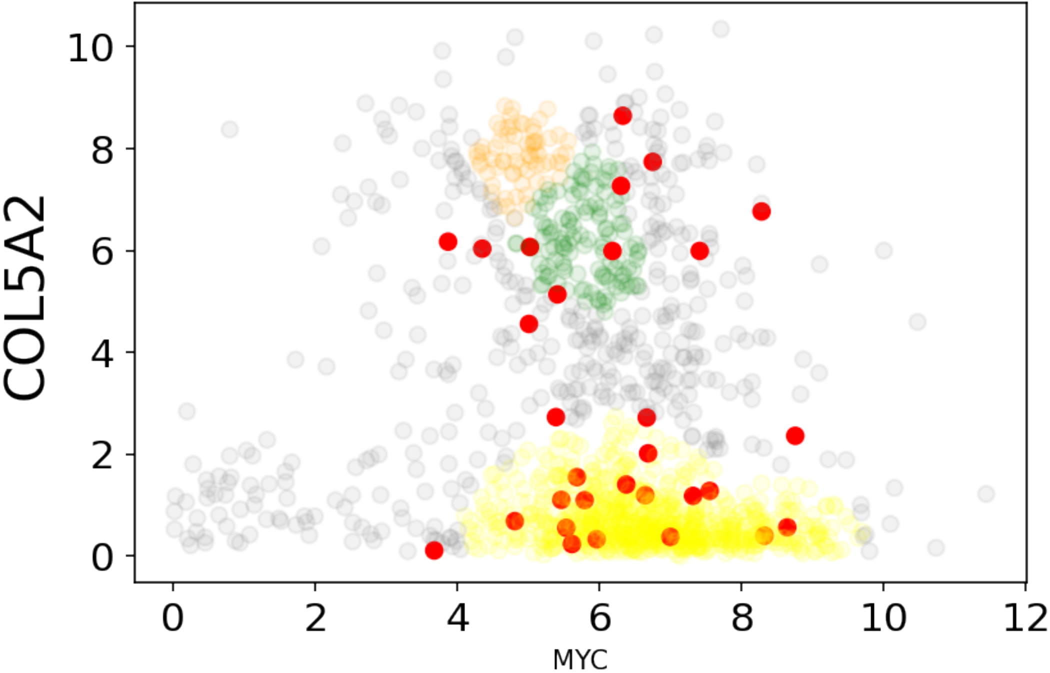

COL5A2
MYC
0
2
4
6
8
10
12

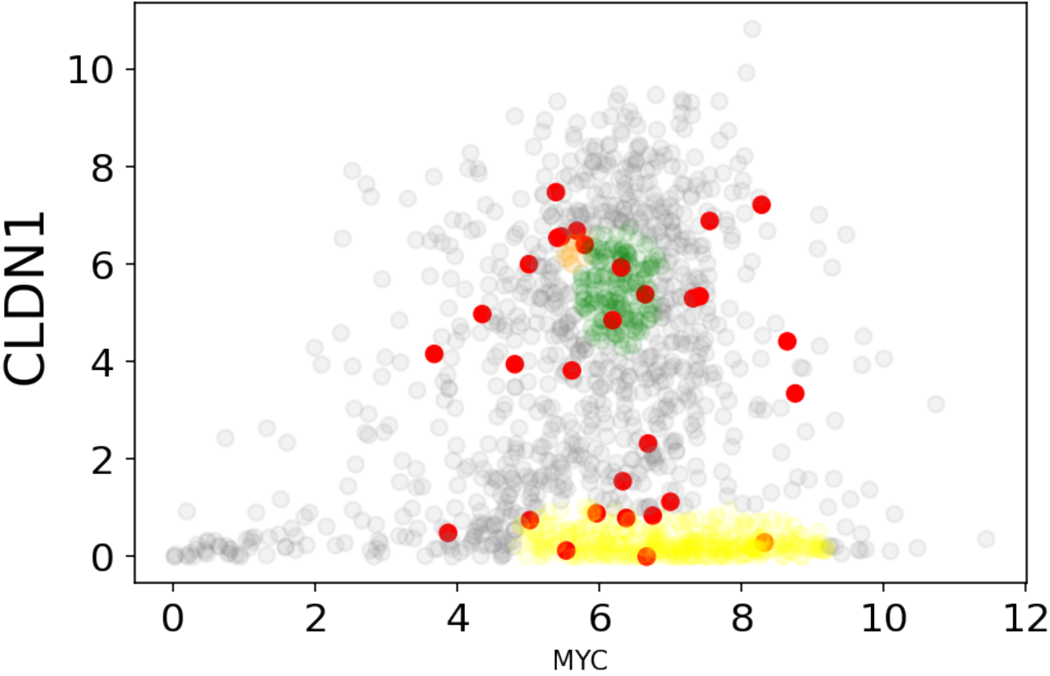

CLDN1
MYC
0
2
4
6
8
10
12

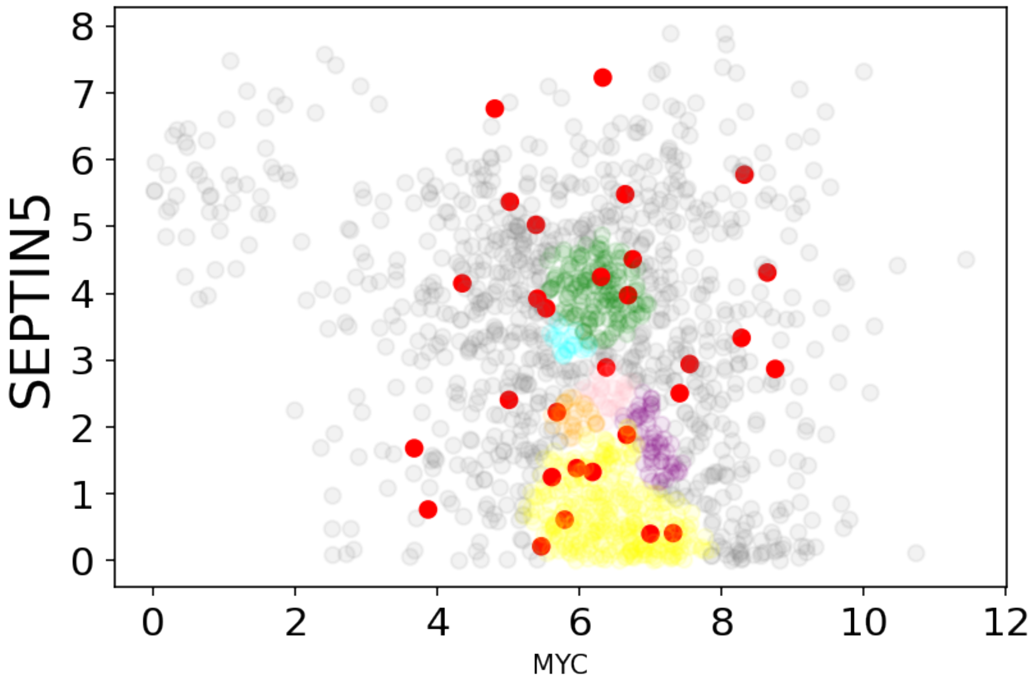

SEPTIN5
MYC
0
1
2
3
4
5
6
7
8
0
2
4
6
8
10
12

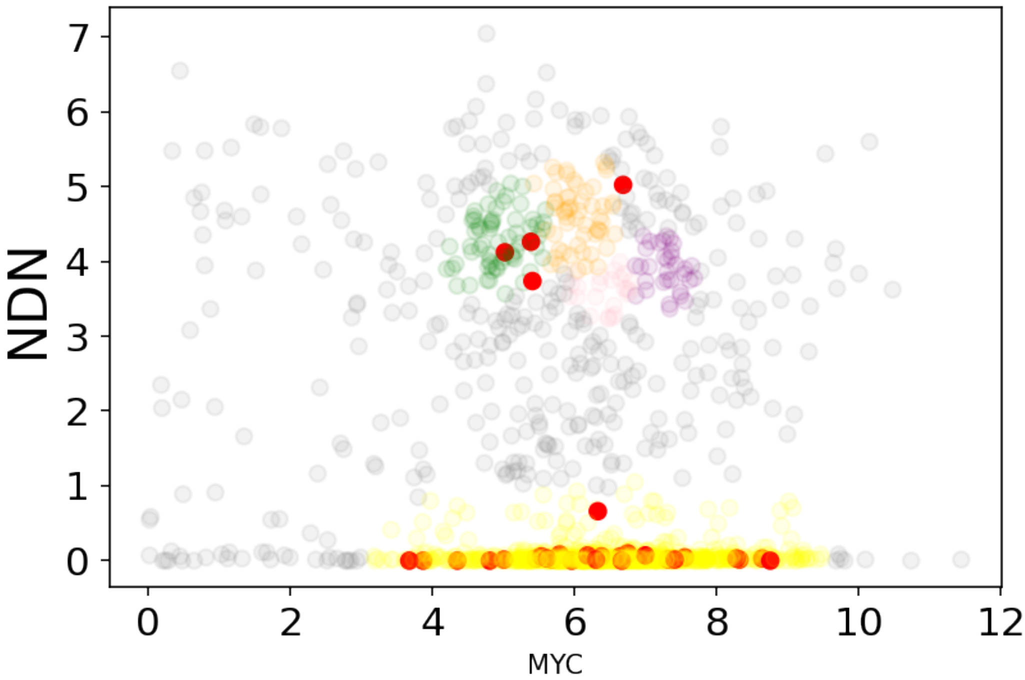

NDN
MYC
0
1
2
3
4
5
6
7
0
2
4
6
8
10
12

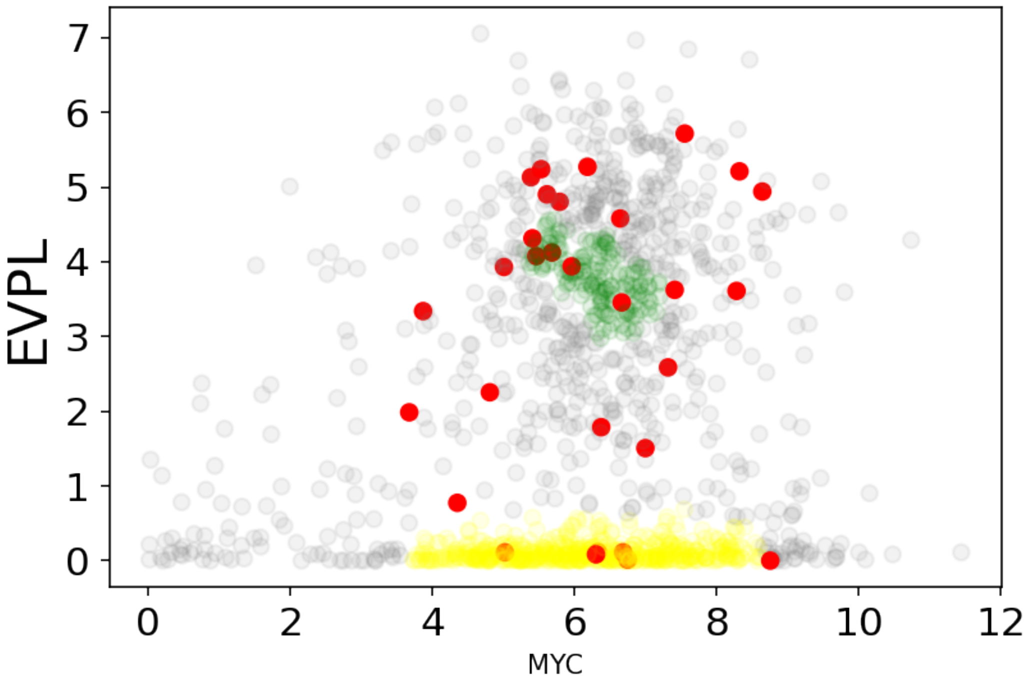

EVPL
7
6
5
4
3
2
1
0
0
2
4
6
8
10
12
MYC

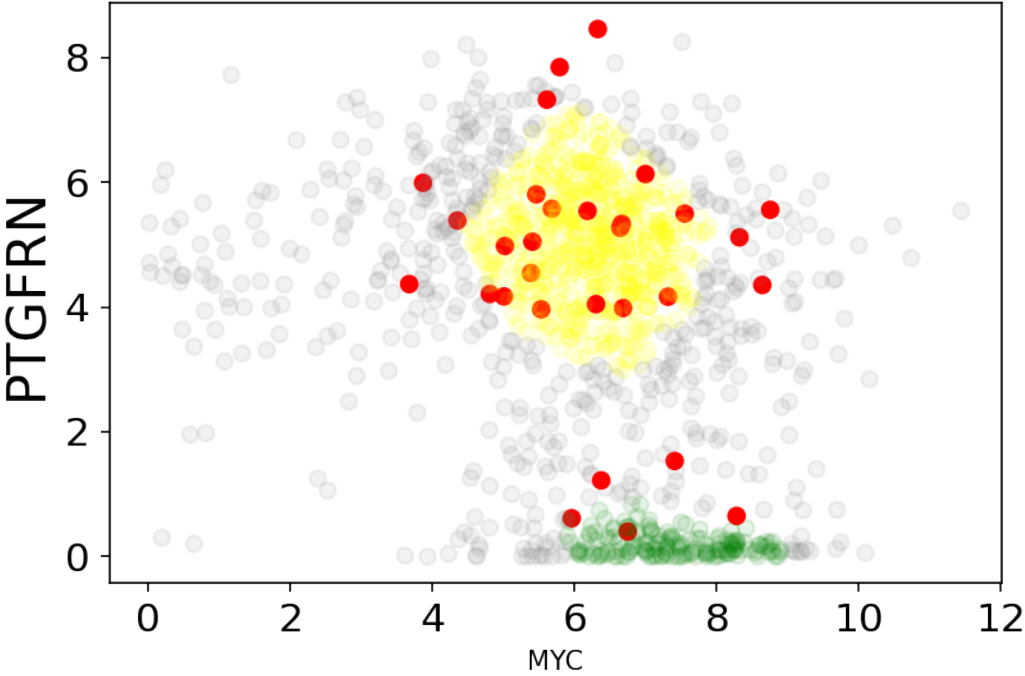
PTGFRN
MYC
0
2
4
6
8
10
12

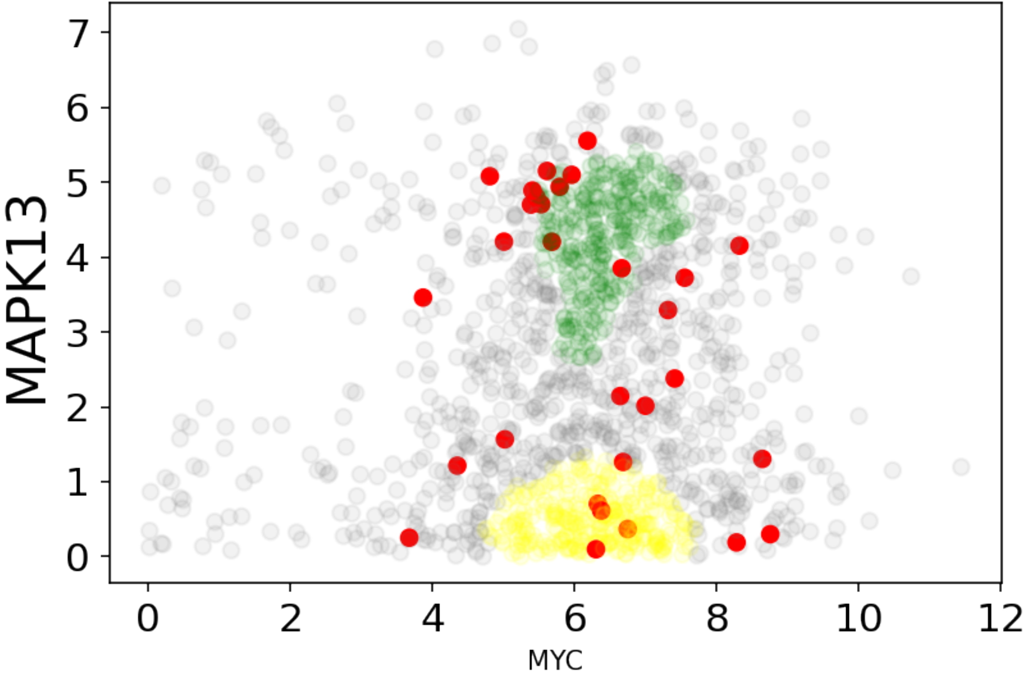

MAPK13
MYC
0
1
2
3
4
5
6
7
0
2
4
6
8
10
12

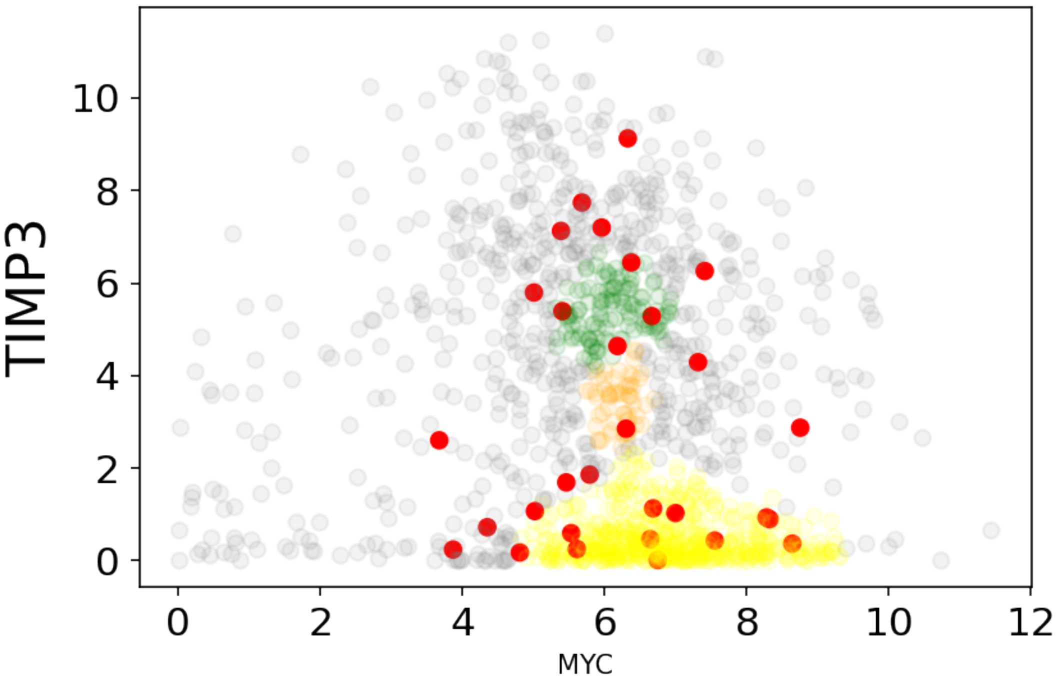

TIMP3
MYC
0
2
4
6
8
10
12

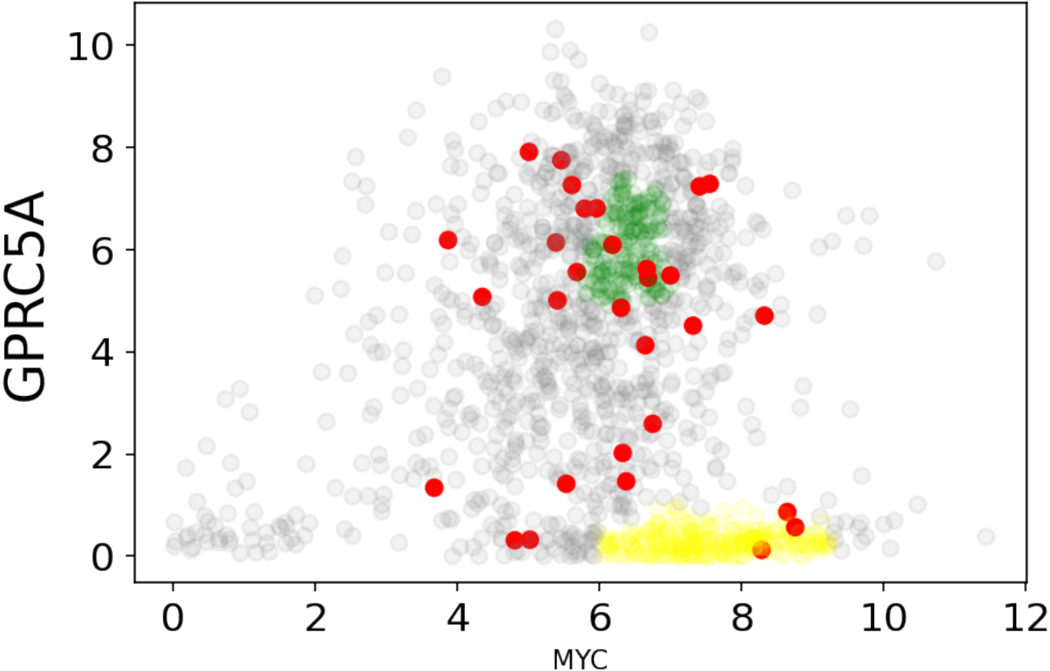
GPRC5A
10
8
6
4
2
0
0
2
4
6
8
10
12
MYC

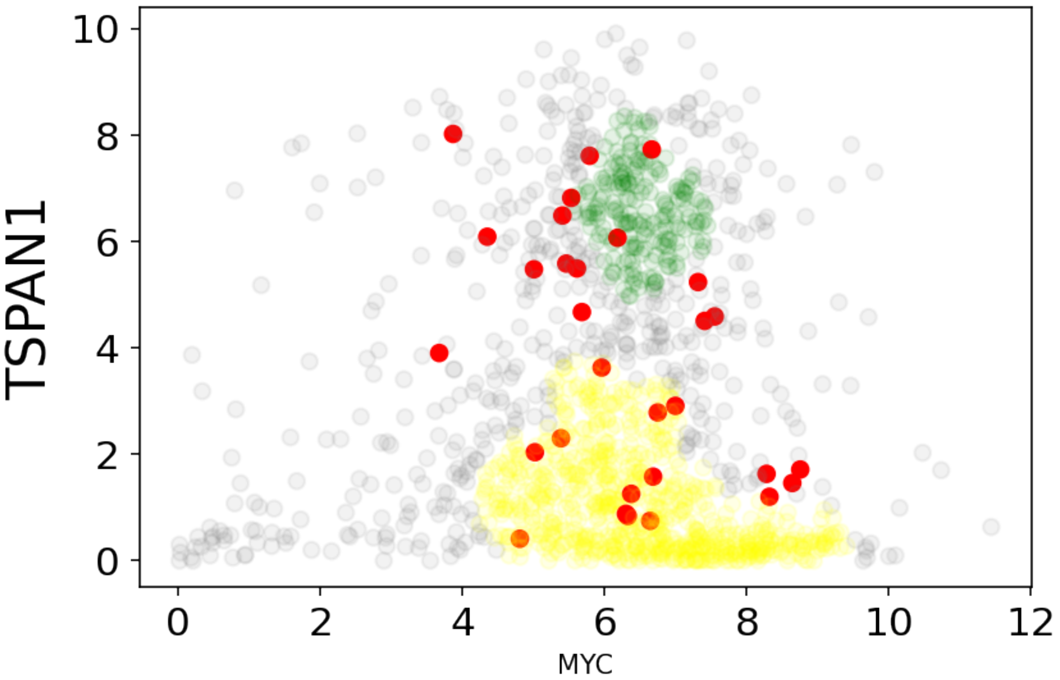
TSPAN1
10
8
6
4
2
0
0
2
4
6
8
10
12
MYC

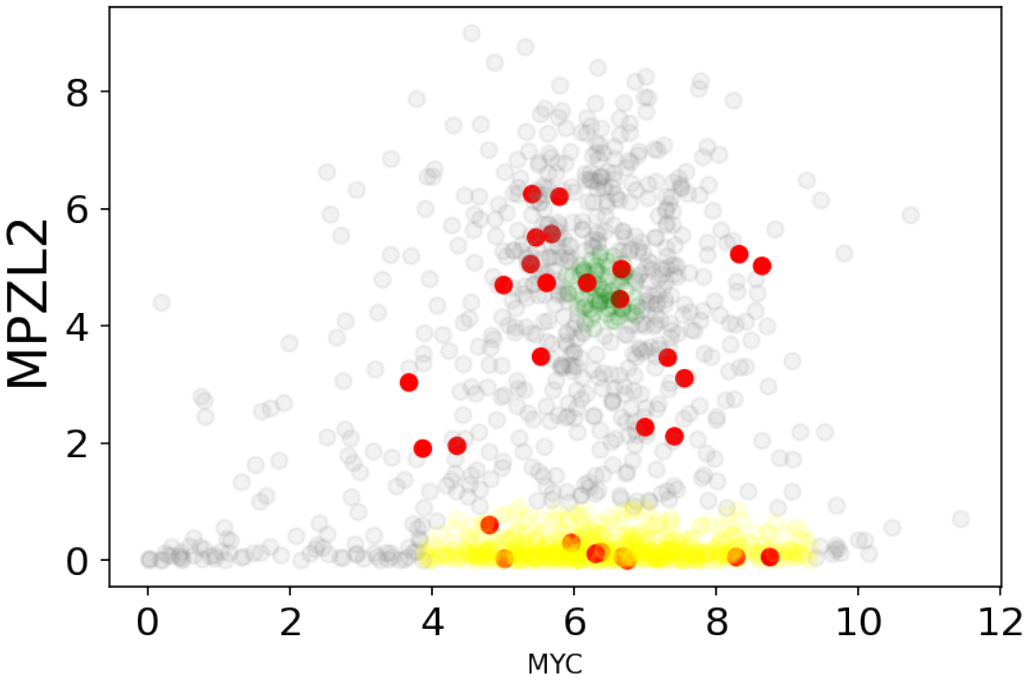

MPZL2
MYC
0
2
4
6
8
10
12

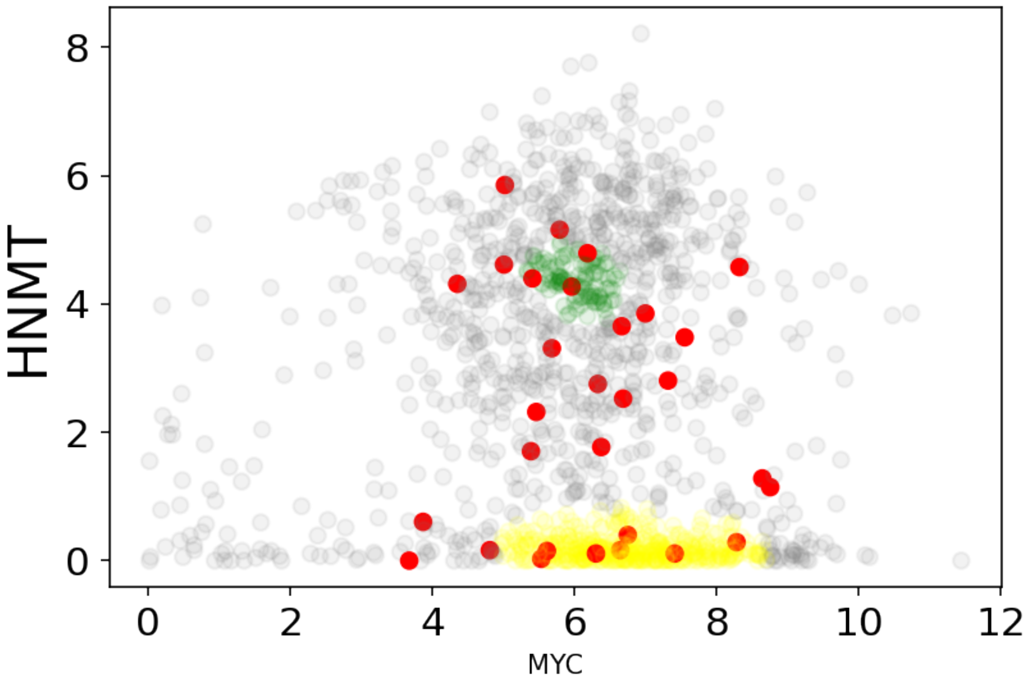
HNMT
MYC
0
2
4
6
8
10
12

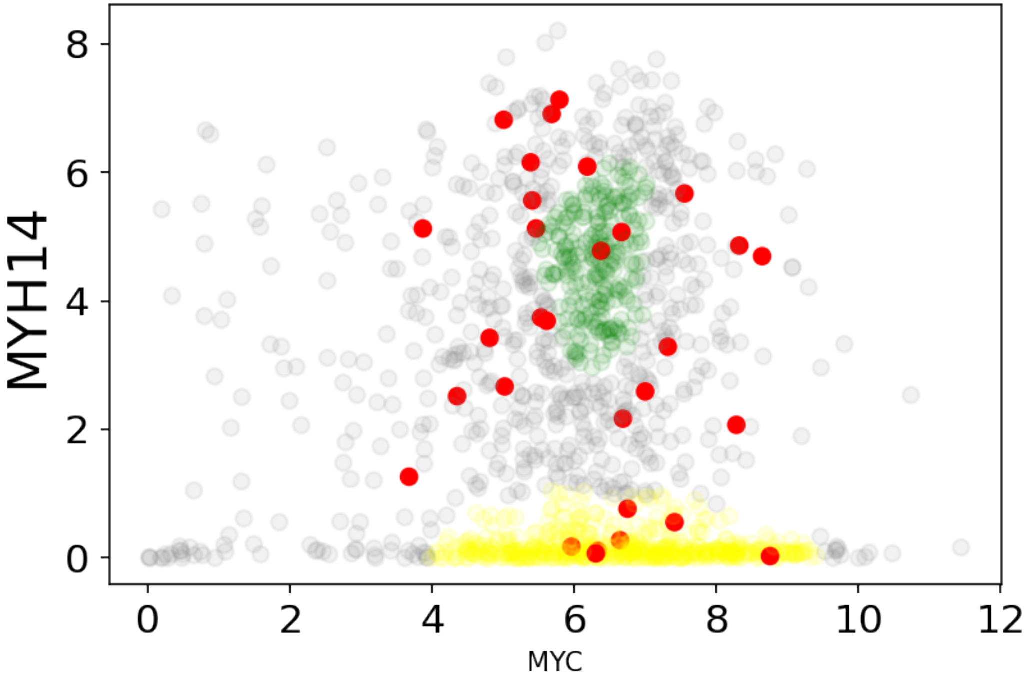

MYH14
8
6
4
2
0
0
2
4
6
8
10
12
MYC

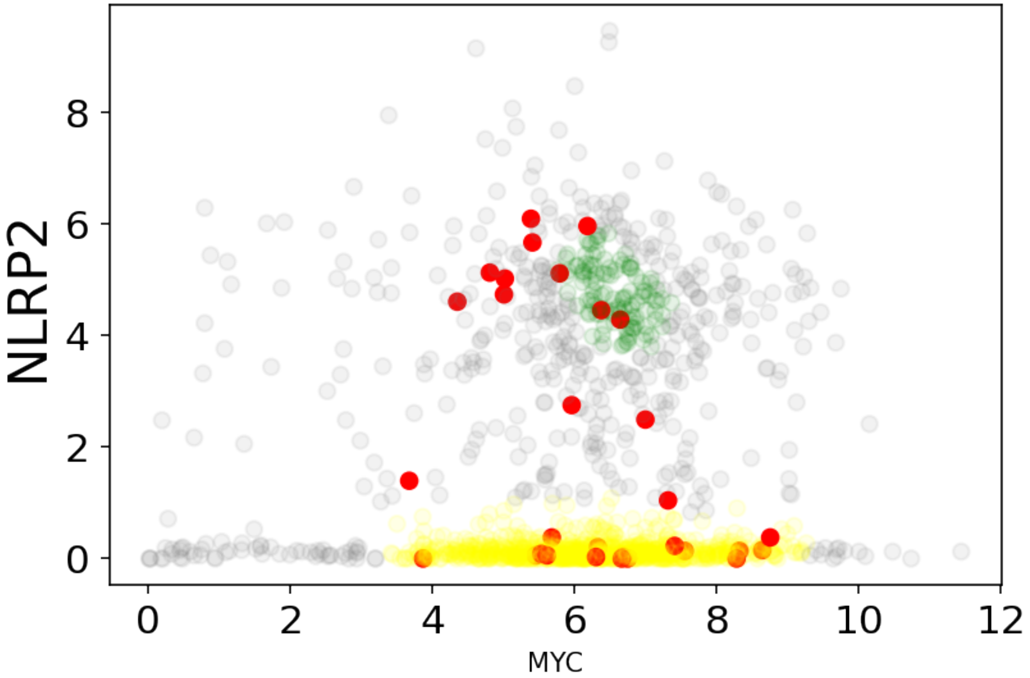

NLRP2
0
2
4
6
8
0
2
4
6
8
10
12
MYC

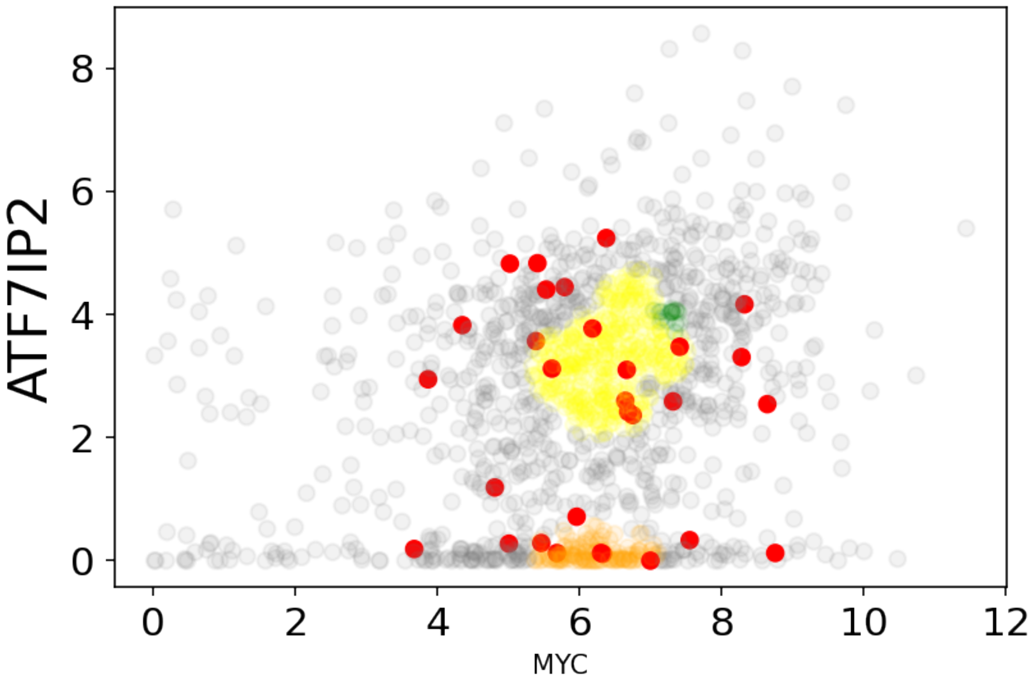

ATF7IP2
8
6
4
2
0
0
2
4
6
8
10
12
MYC

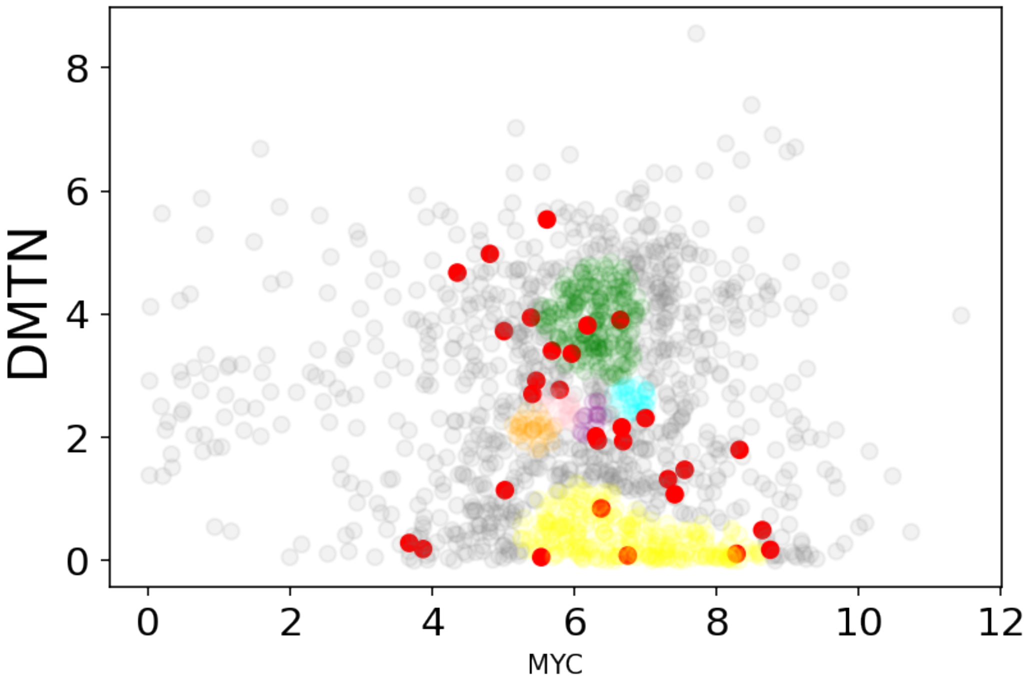
DMTN
MYC
0
2
4
6
8
10
12

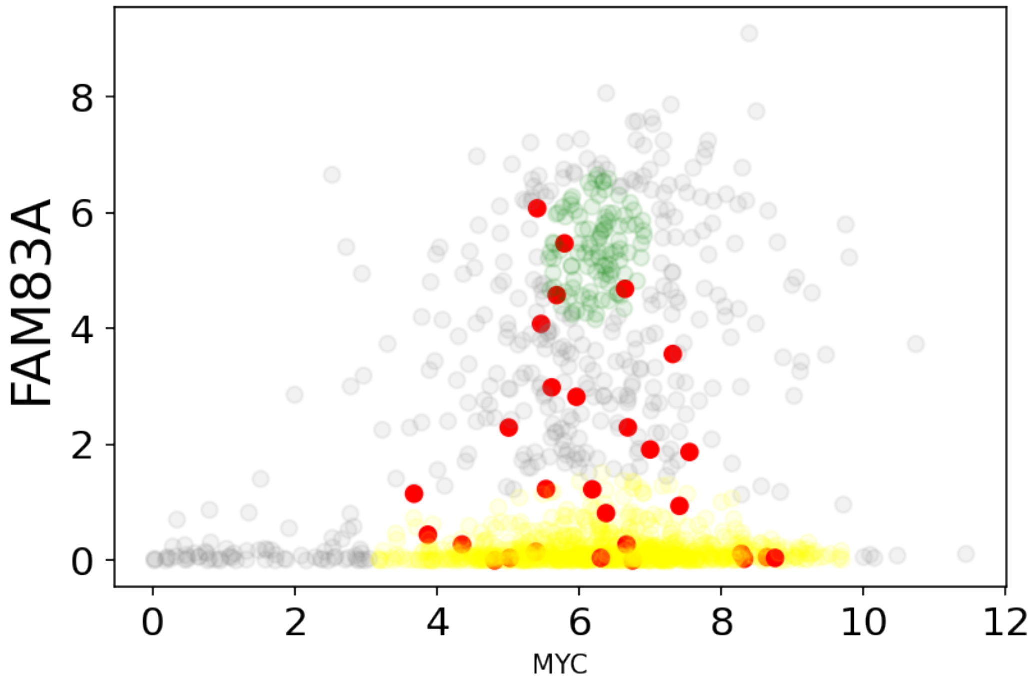
FAM83A
MYC
0
2
4
6
8
10
12

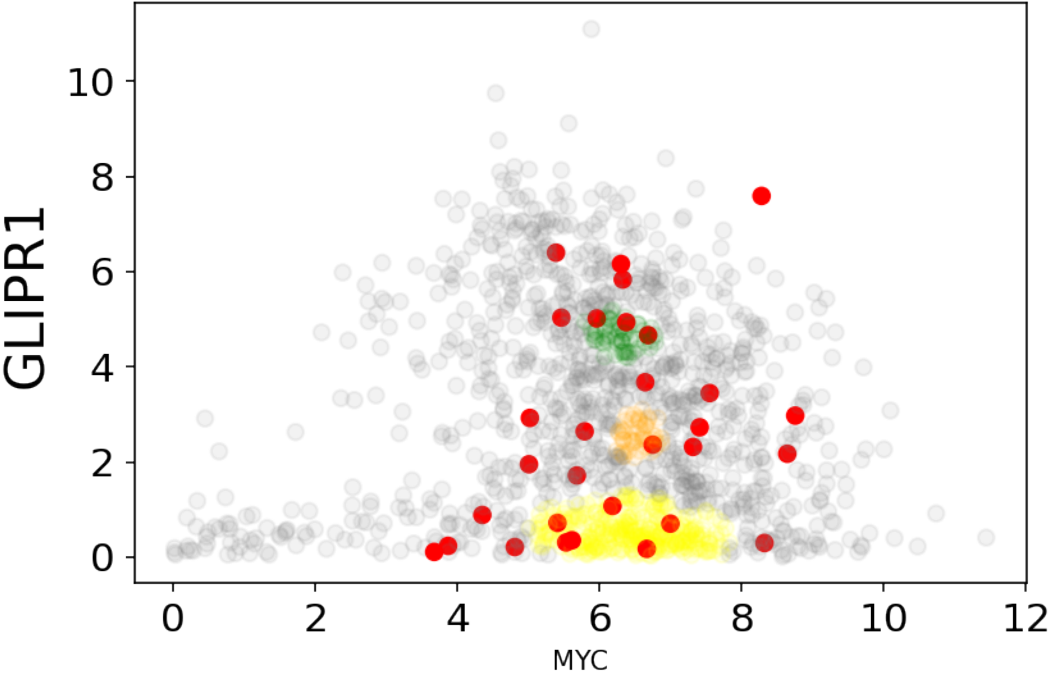
GLIPR1
MYC
0
2
4
6
8
10
12

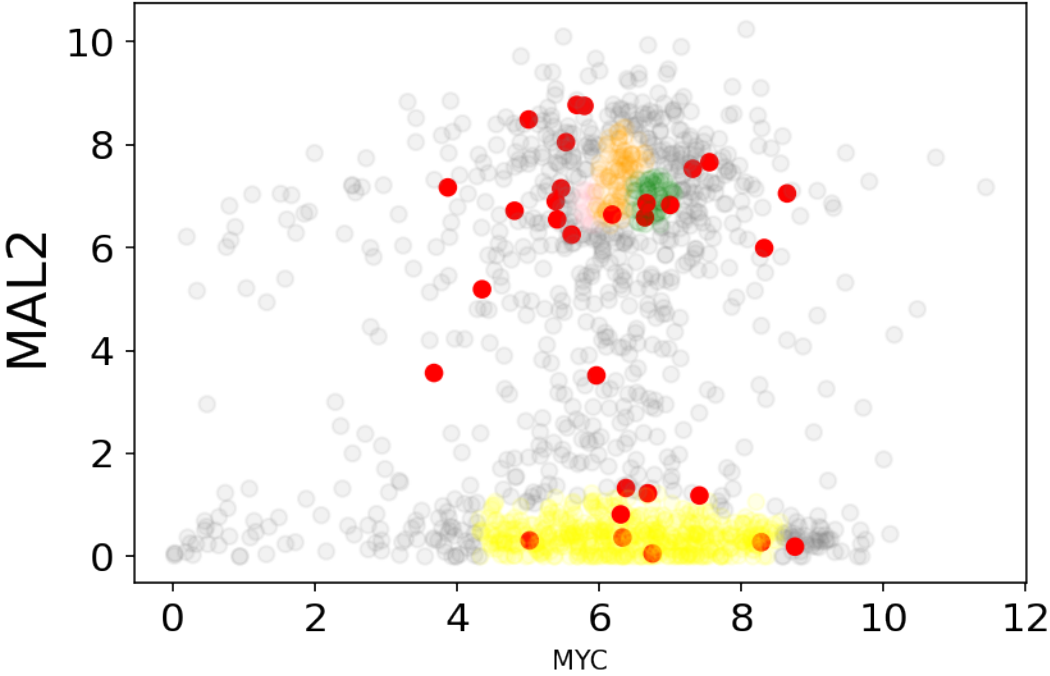

MAL2
MYC
0
2
4
6
8
10
12

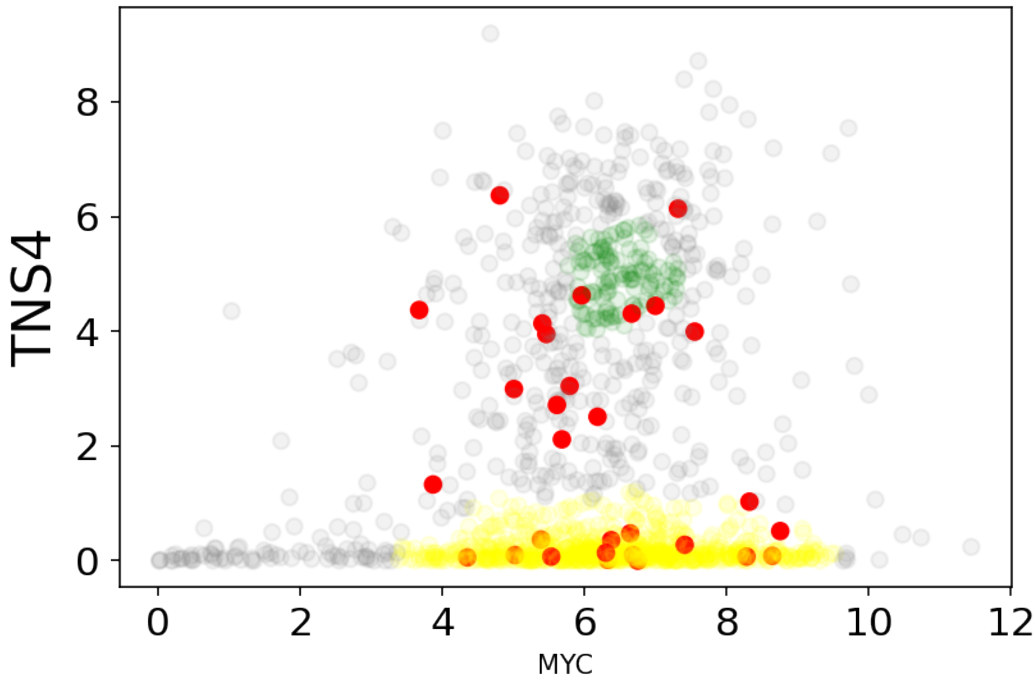

TNS4
0
2
4
6
8
0
2
4
6
8
10
12
MYC

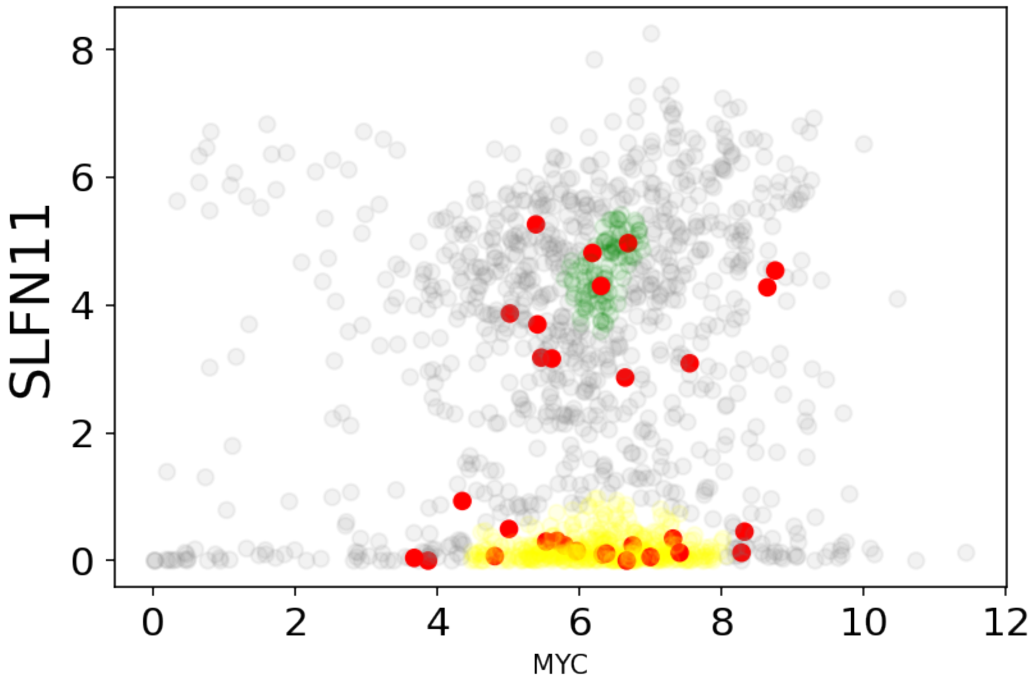
SLFN11
MYC
0
2
4
6
8
10
12

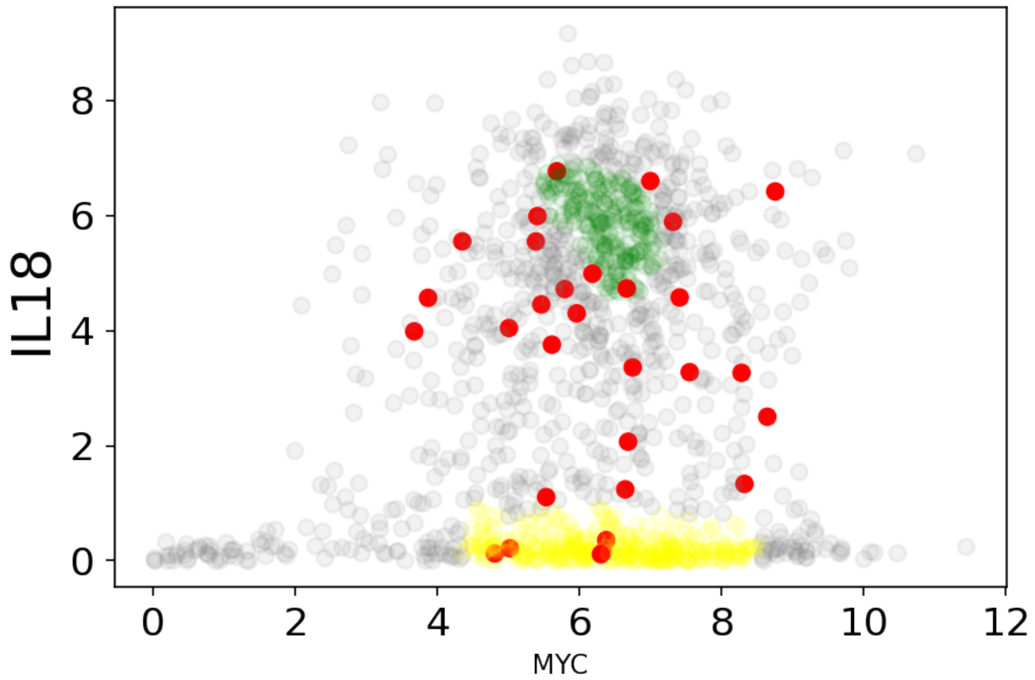
IL18
8
6
4
2
0
0
2
4
6
8
10
12
MYC

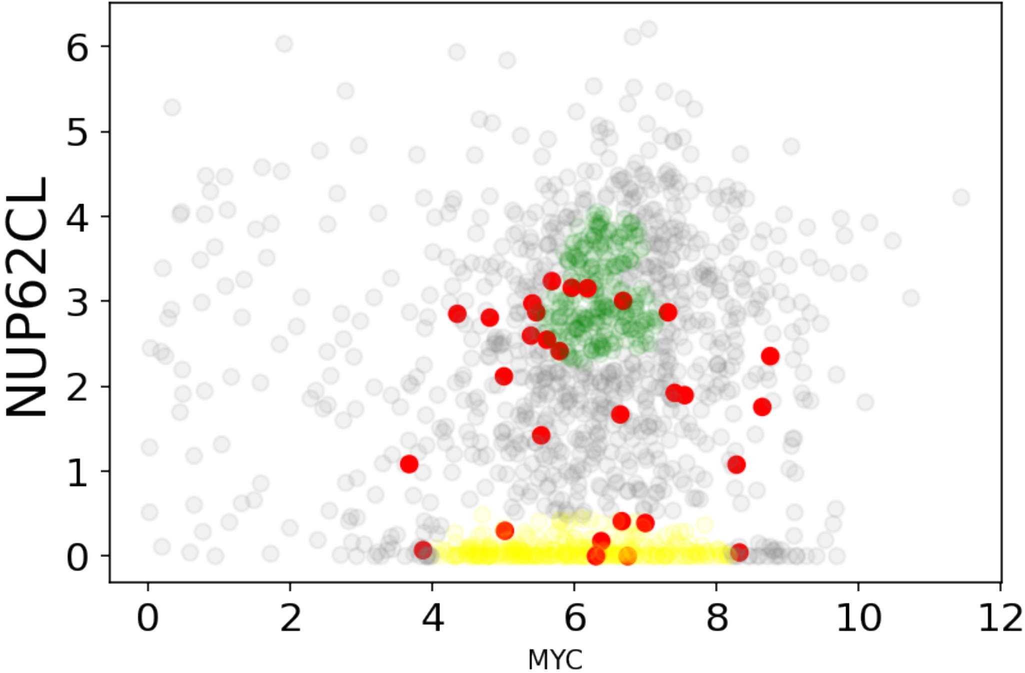
NUP62CL
MYC
0
1
2
3
4
5
6
0
2
4
6
8
10
12

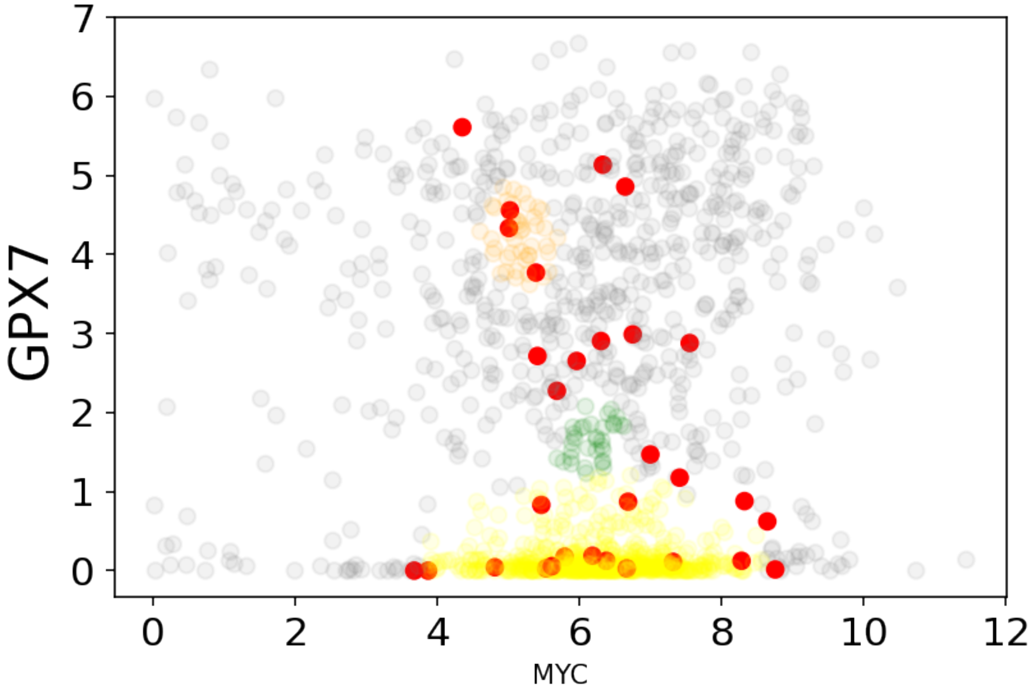

GPX7
0
1
2
3
4
5
6
7
0
2
4
6
8
10
12
MYC

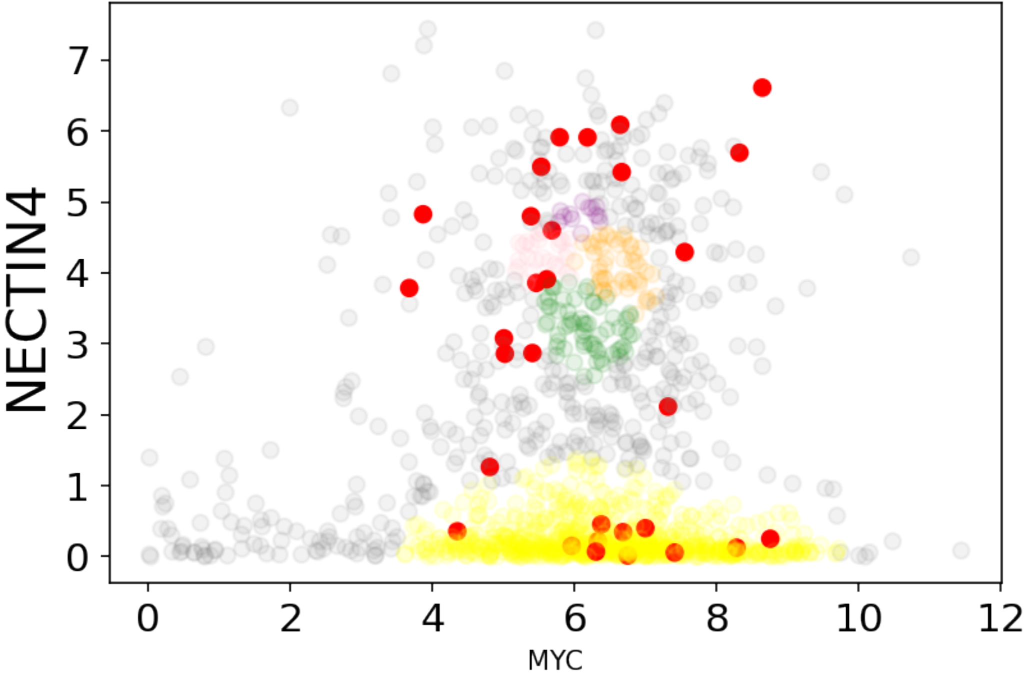

NECTIN4
MYC
0
1
2
3
4
5
6
7
0
2
4
6
8
10
12

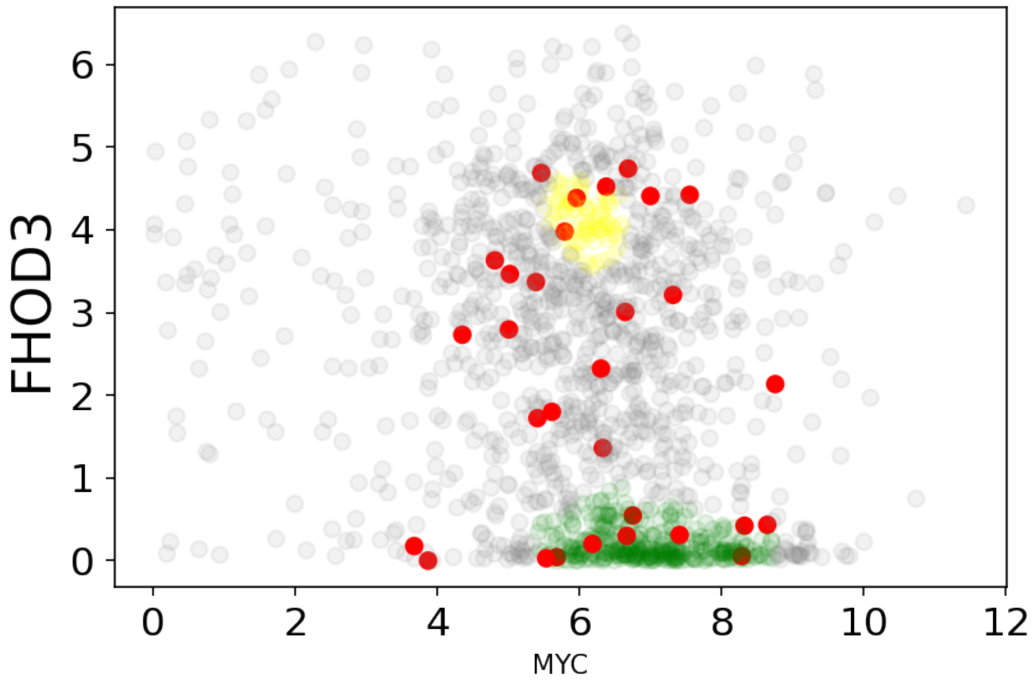

FHOD3
MYC
0
1
2
3
4
5
6
0
2
4
6
8
10
12

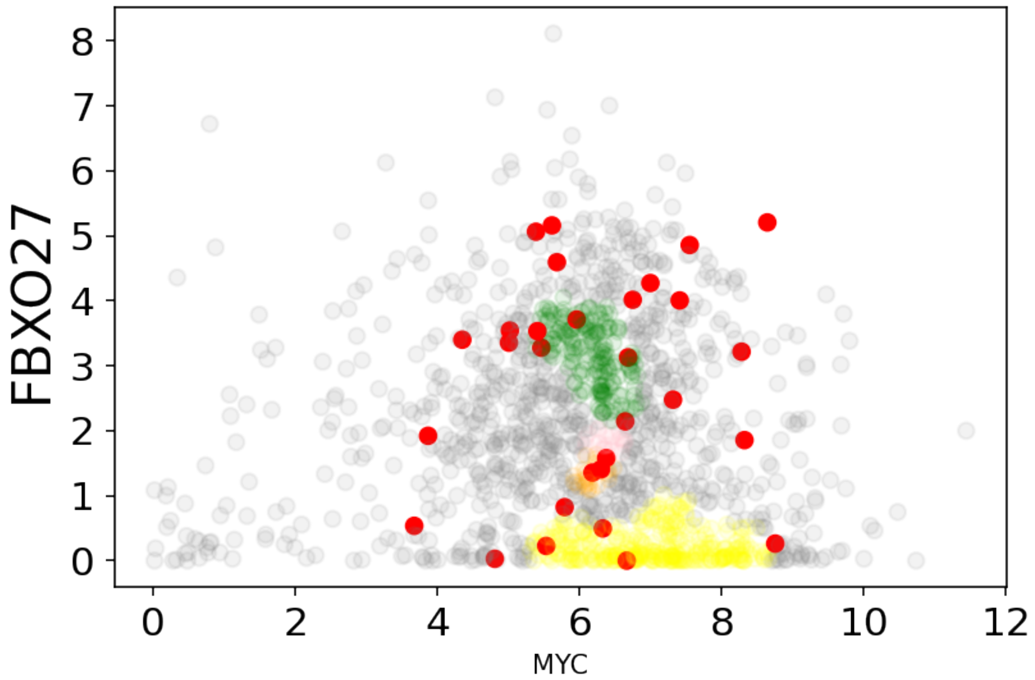
FBXO27
MYC
0
1
2
3
4
5
6
7
8
0
2
4
6
8
10
12

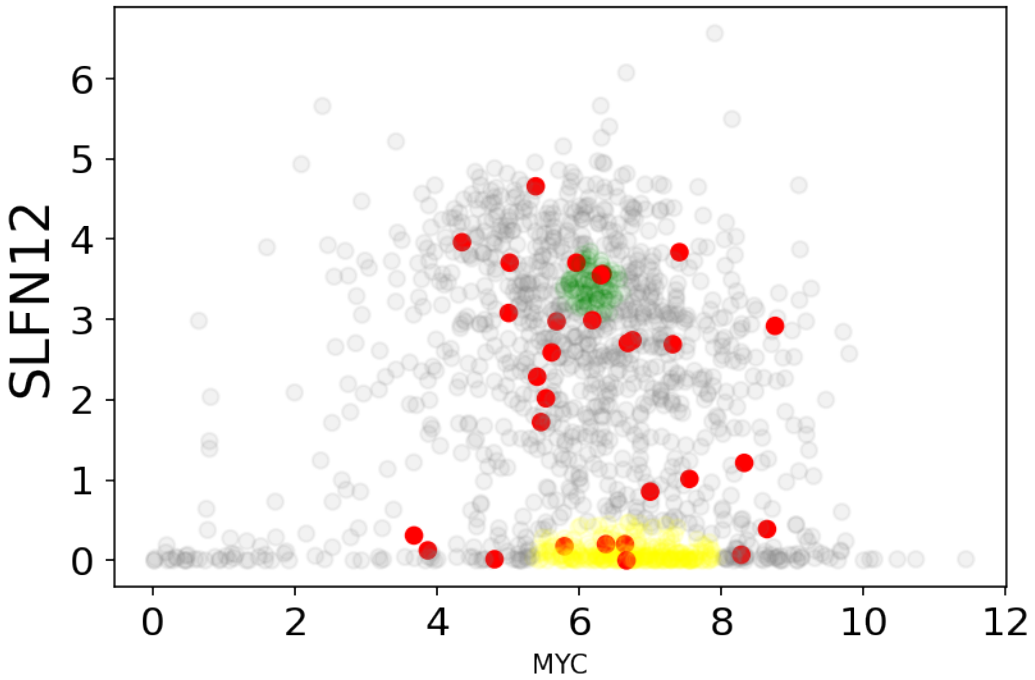
SLFN12
MYC
0
1
2
3
4
5
6
0
2
4
6
8
10
12

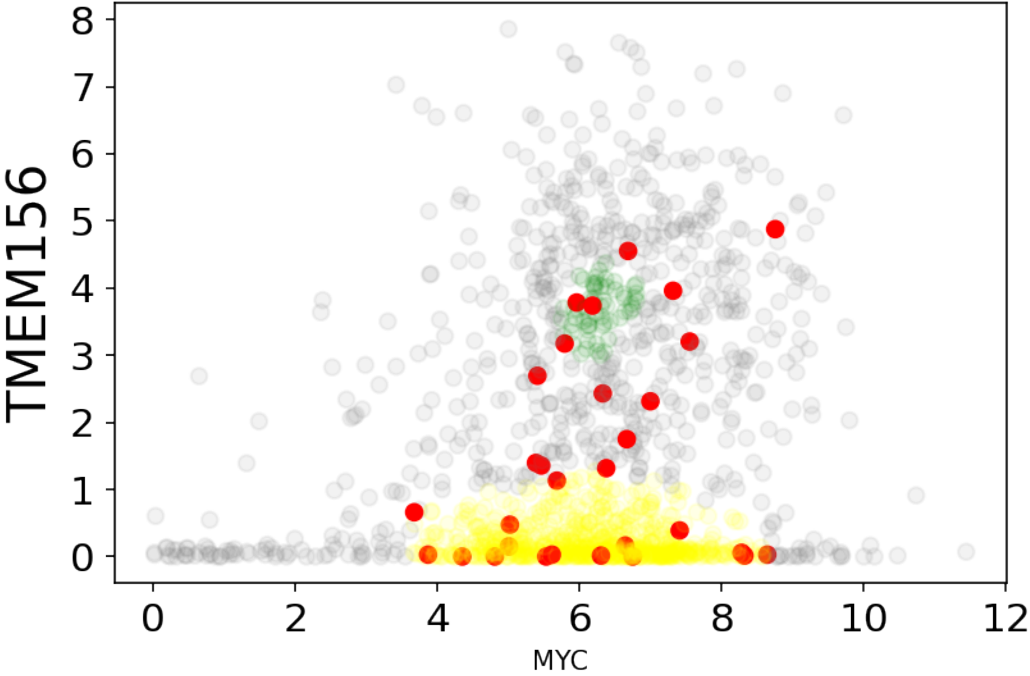
TMEM156
MYC
0
1
2
3
4
5
6
7
8
0
2
4
6
8
10
12

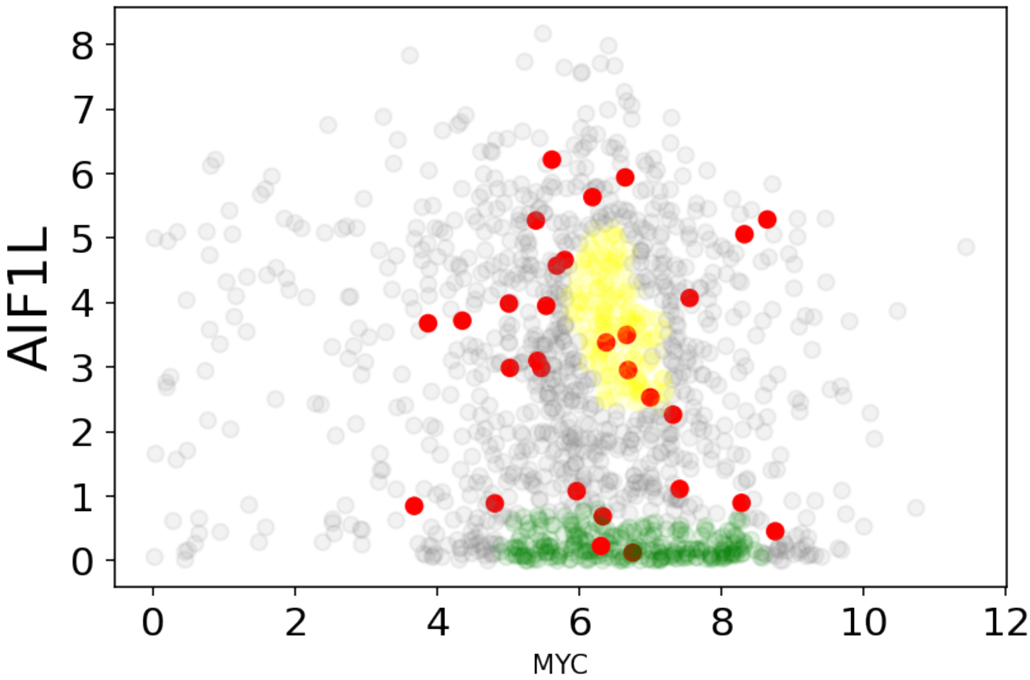

AIF1L
MYC
0
1
2
3
4
5
6
7
8
0
2
4
6
8
10
12

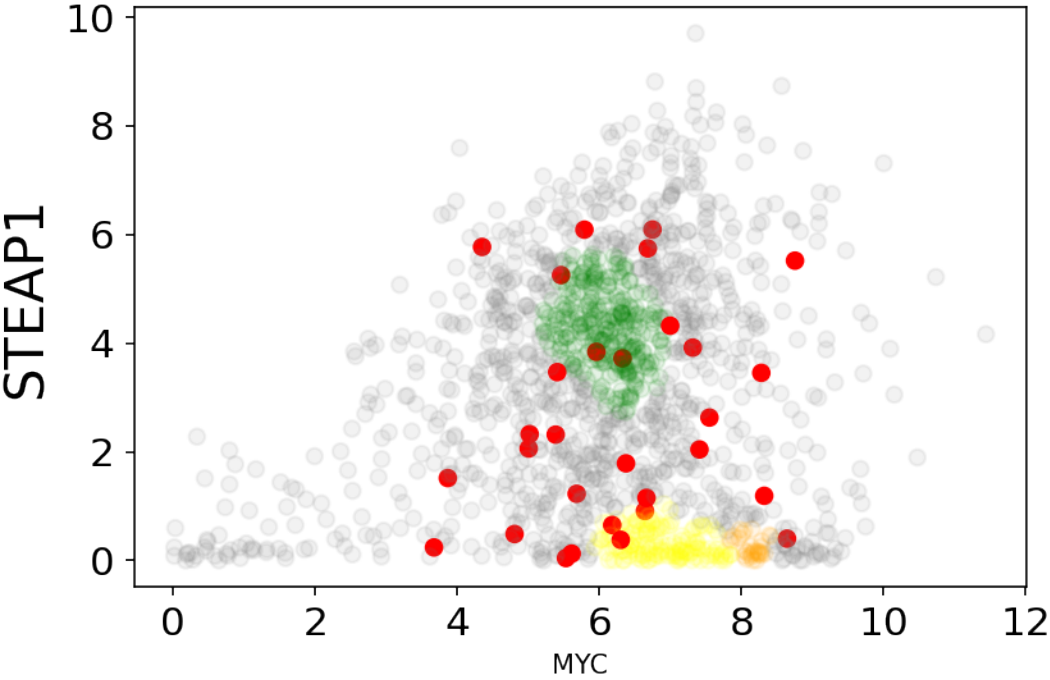

STEAP1
10
8
6
4
2
0
0
2
4
6
8
10
12
MYC

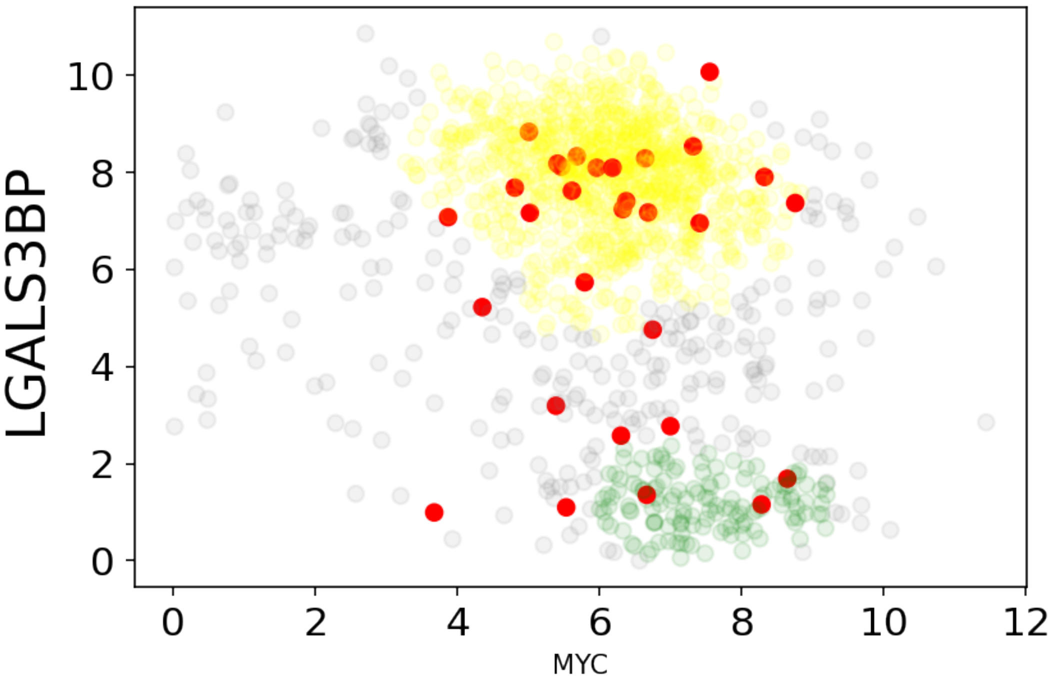

LGALS3BP
0
2
4
6
8
10
0
2
4
6
8
10
12
MYC

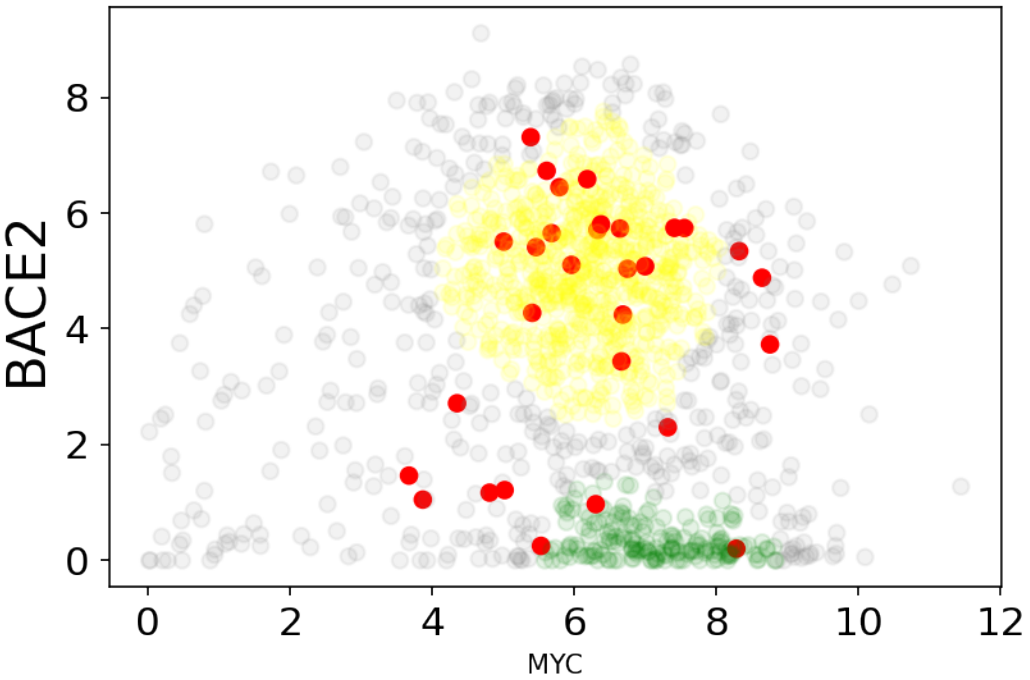
BACE2
MYC
0
2
4
6
8
0
2
4
6
8
10
12

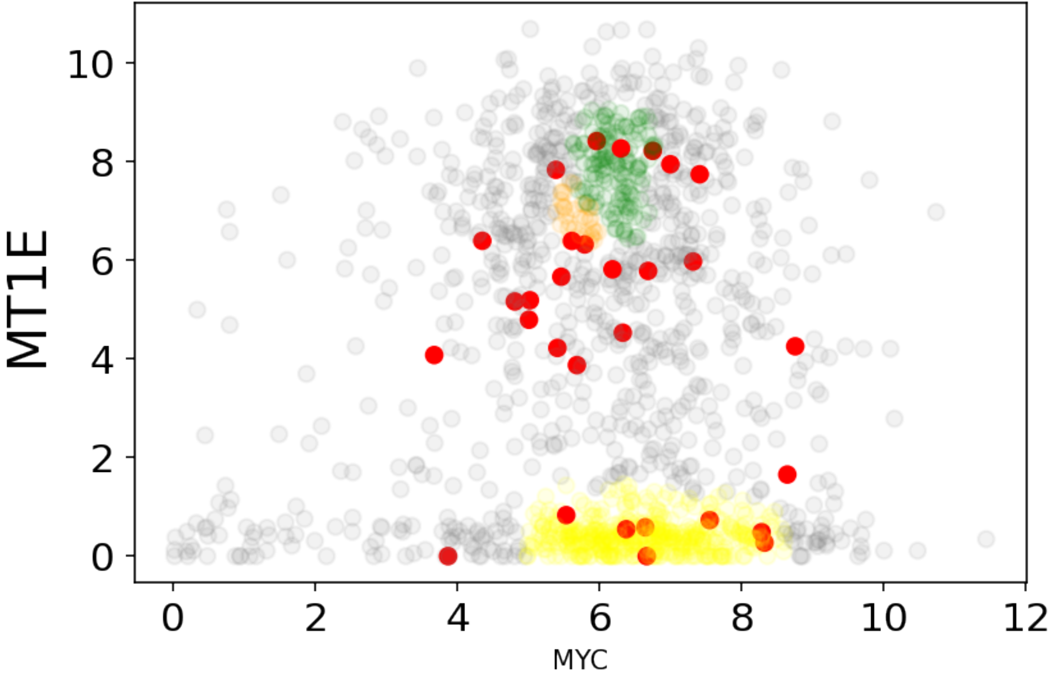

MT1E
0
2
4
6
8
10
0
2
4
6
8
10
12
MYC

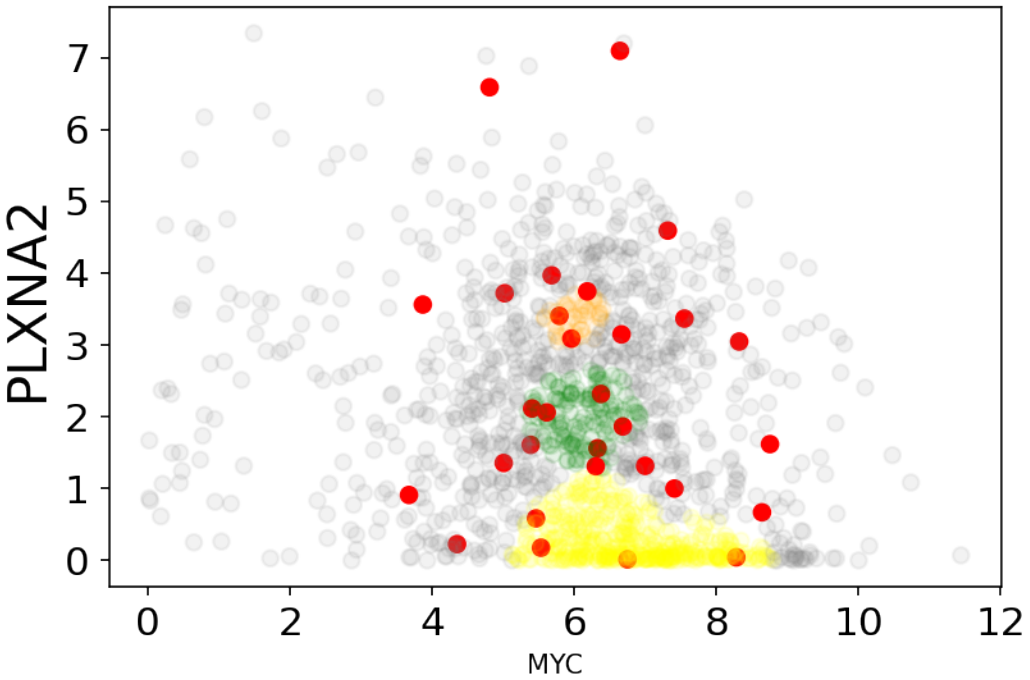
PLXNA2
MYC
0
1
2
3
4
5
6
7
0
2
4
6
8
10
12

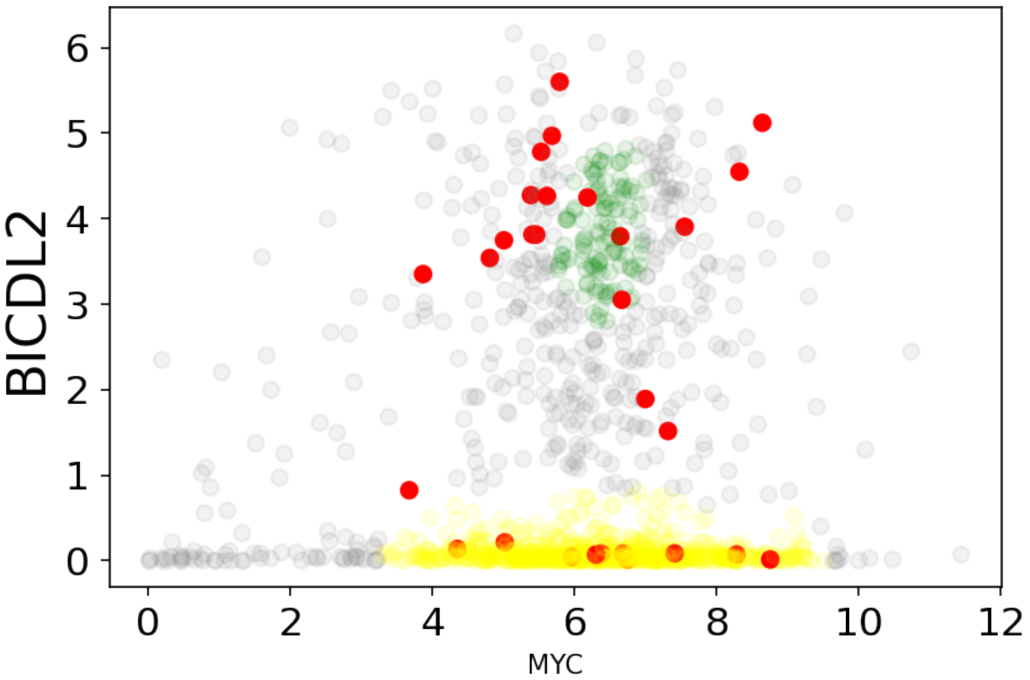

BICDL2
MYC
0
1
2
3
4
5
6
0
2
4
6
8
10
12

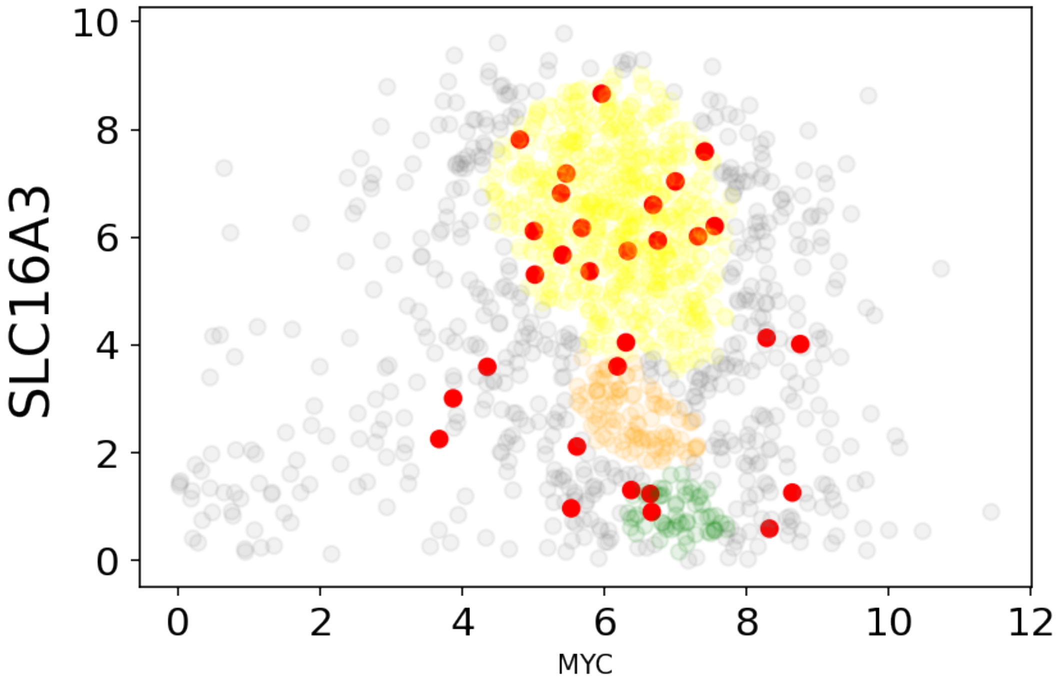
SLC16A3
MYC
0
2
4
6
8
10
12

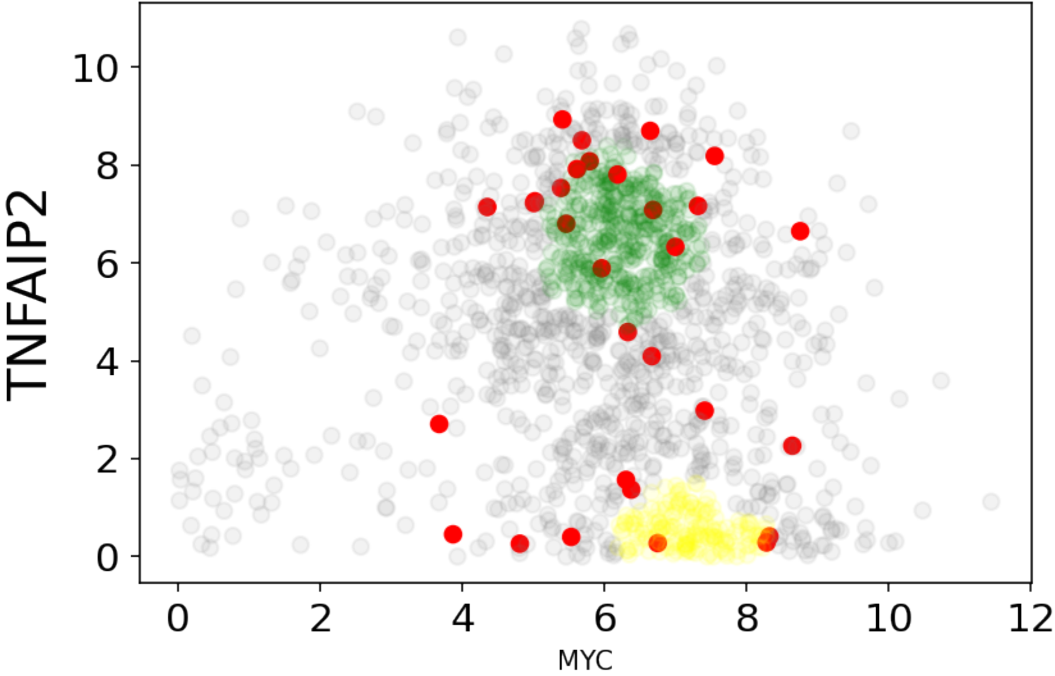
TNFAIP2
MYC
0
2
4
6
8
10
12

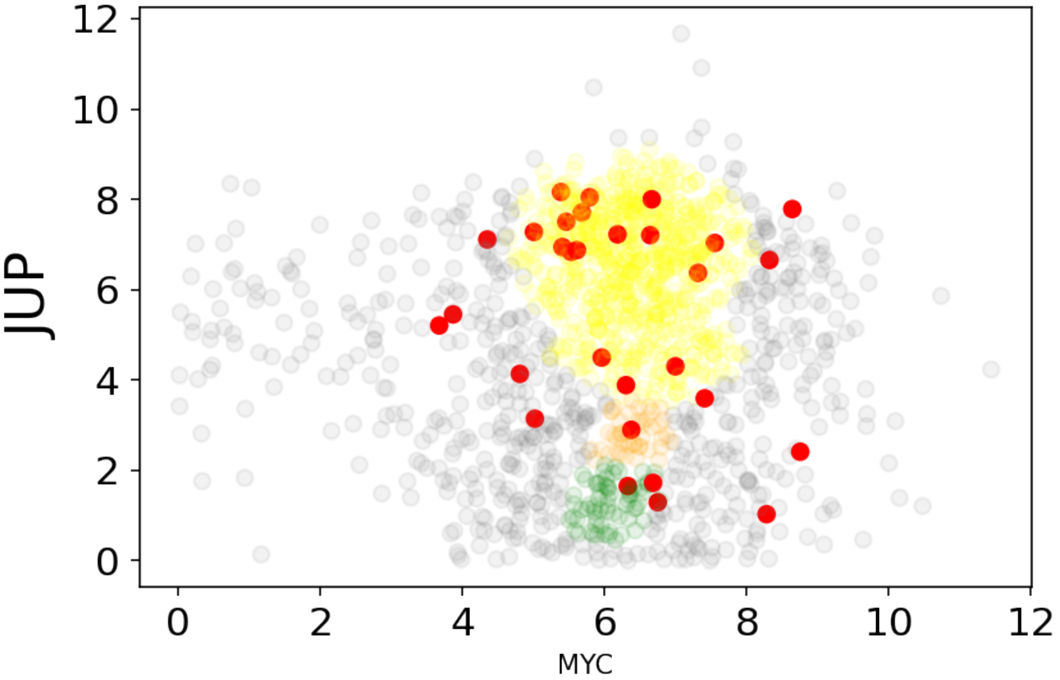

JUP
MYC
0
2
4
6
8
10
12

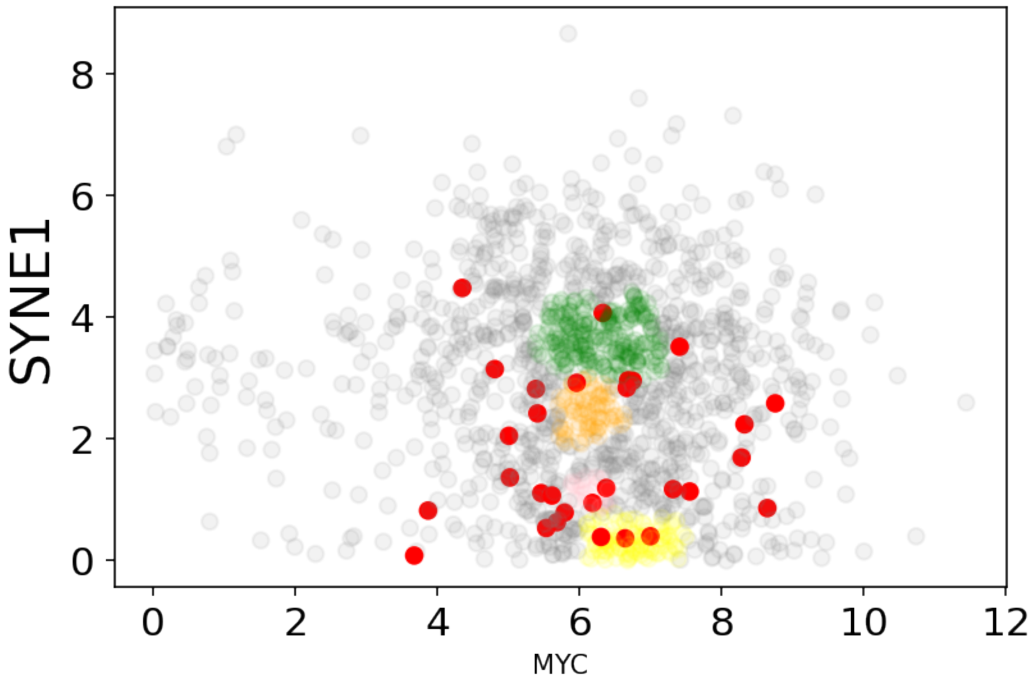
SYNE1
MYC
0
2
4
6
8
0
2
4
6
8
10
12

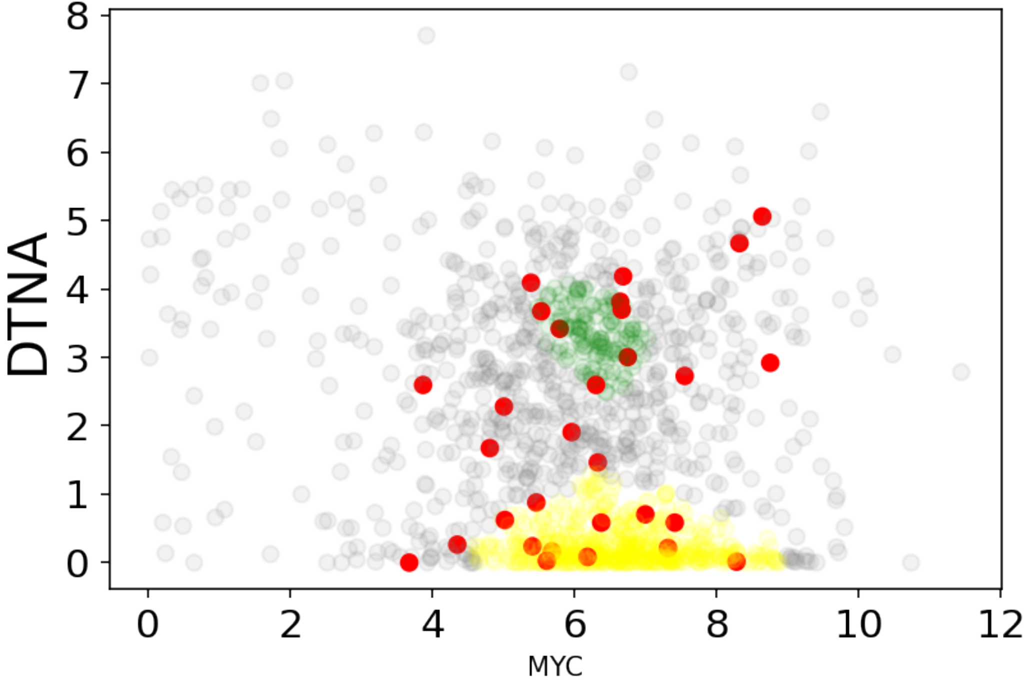

DTNA
MYC
0
1
2
3
4
5
6
7
8
0
2
4
6
8
10
12

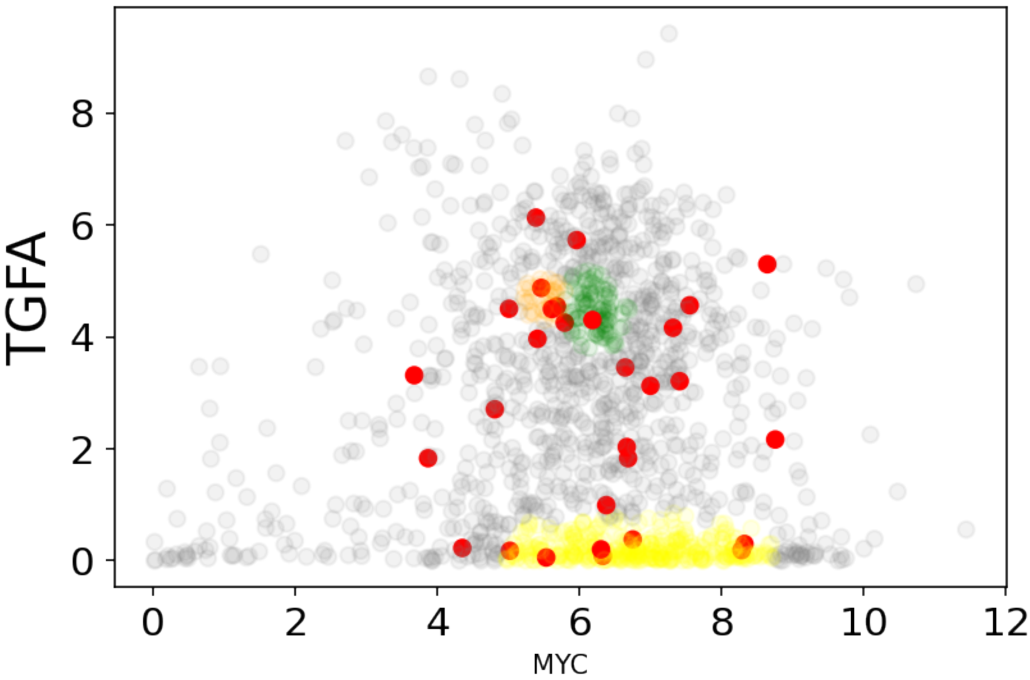
TGFA
8
6
4
2
0
0
2
4
6
8
10
12
MYC

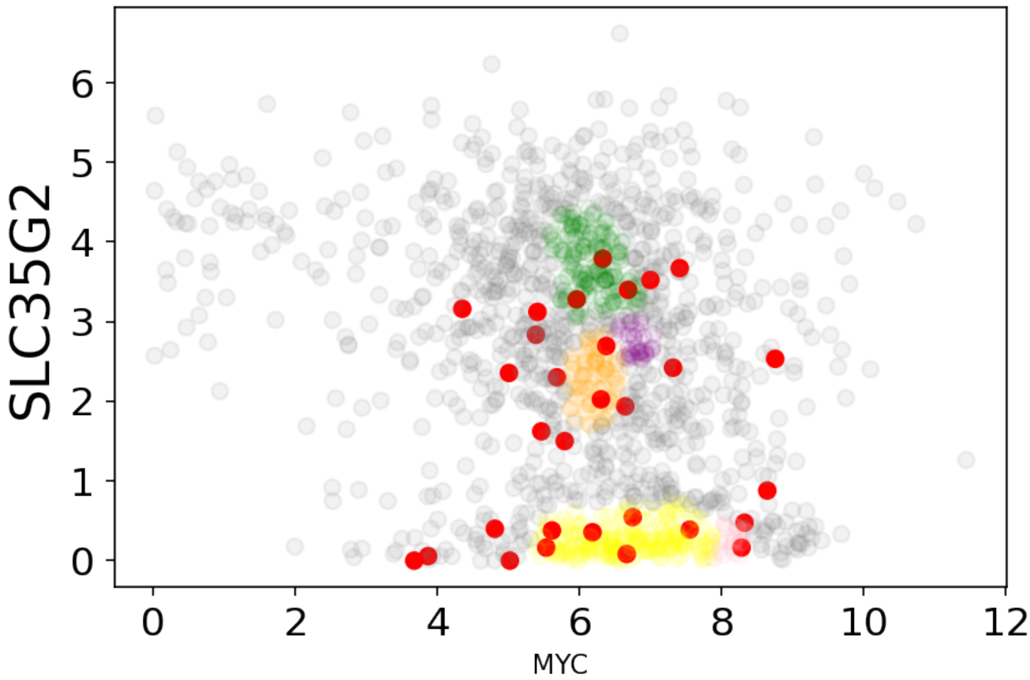

SLC35G2
6
5
4
3
2
1
0
0
2
4
6
8
10
12
MYC

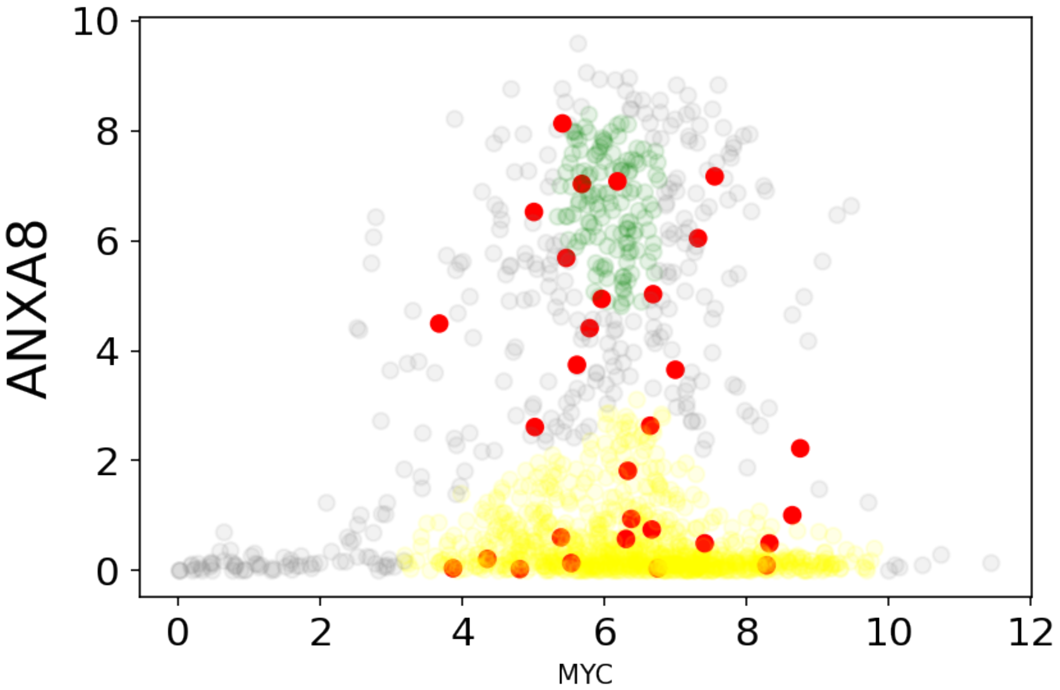

ANXA8
MYC
0
2
4
6
8
10
12

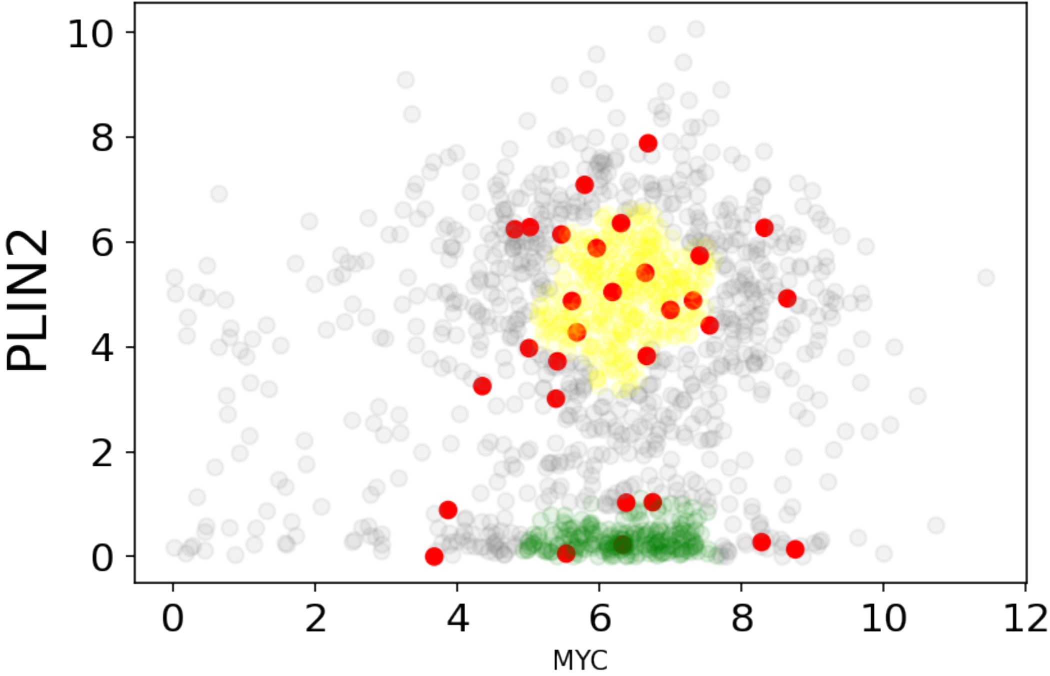

PLIN2
MYC
0
2
4
6
8
10
12

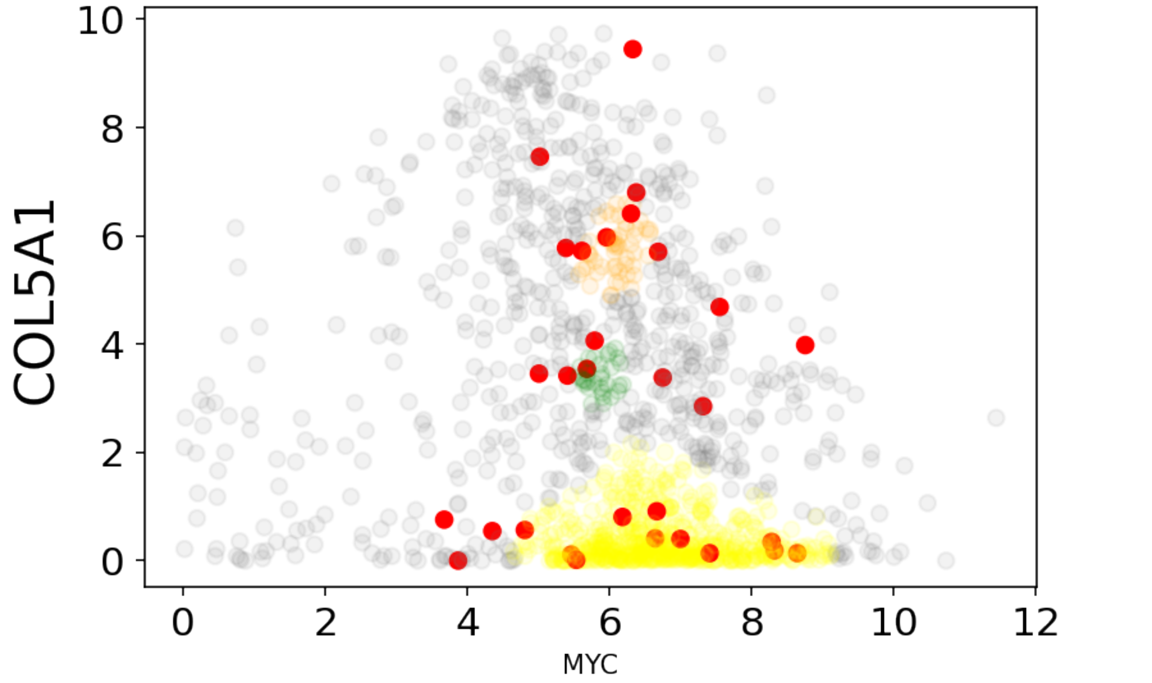
COL5A1
MYC
0
2
4
6
8
10
12

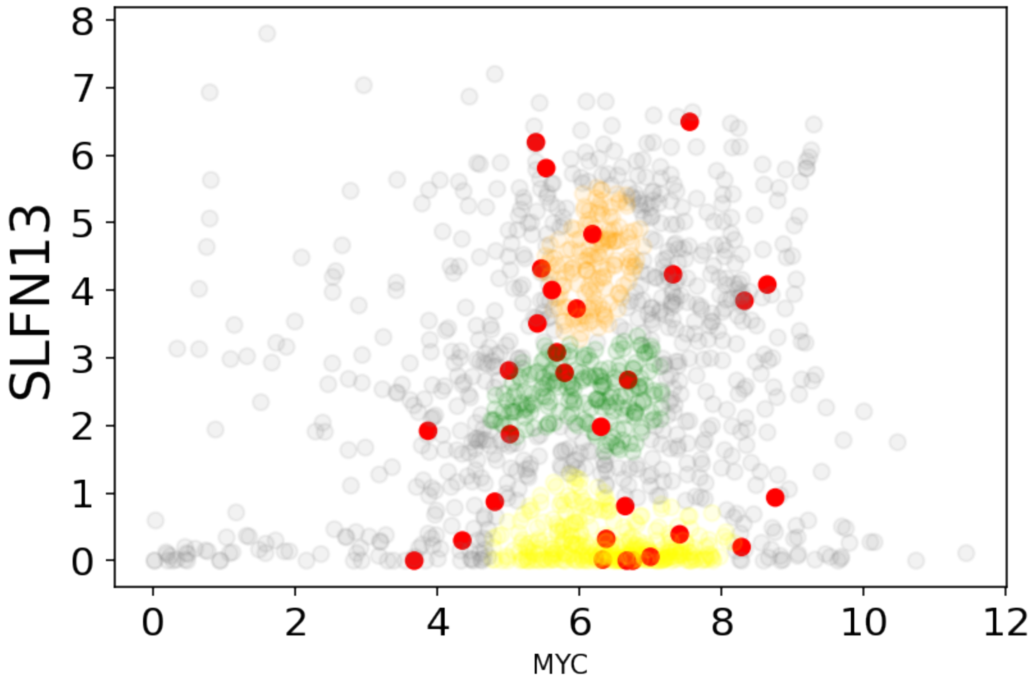

SLFN13
MYC
0
1
2
3
4
5
6
7
8
0
2
4
6
8
10
12

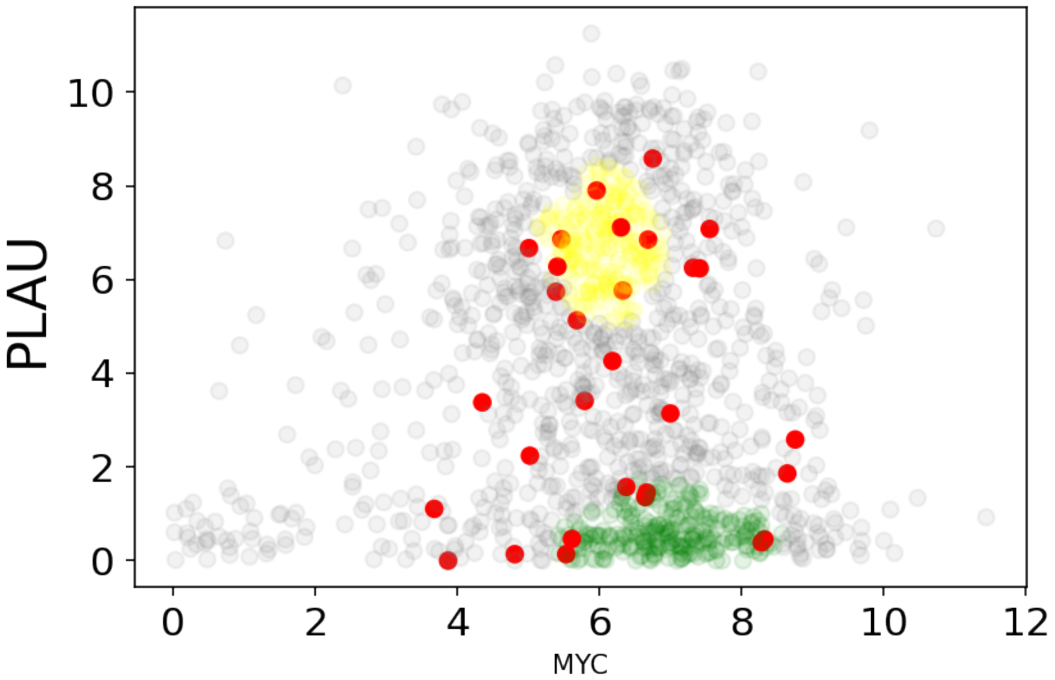

PLAU
MYC
0
2
4
6
8
10
12

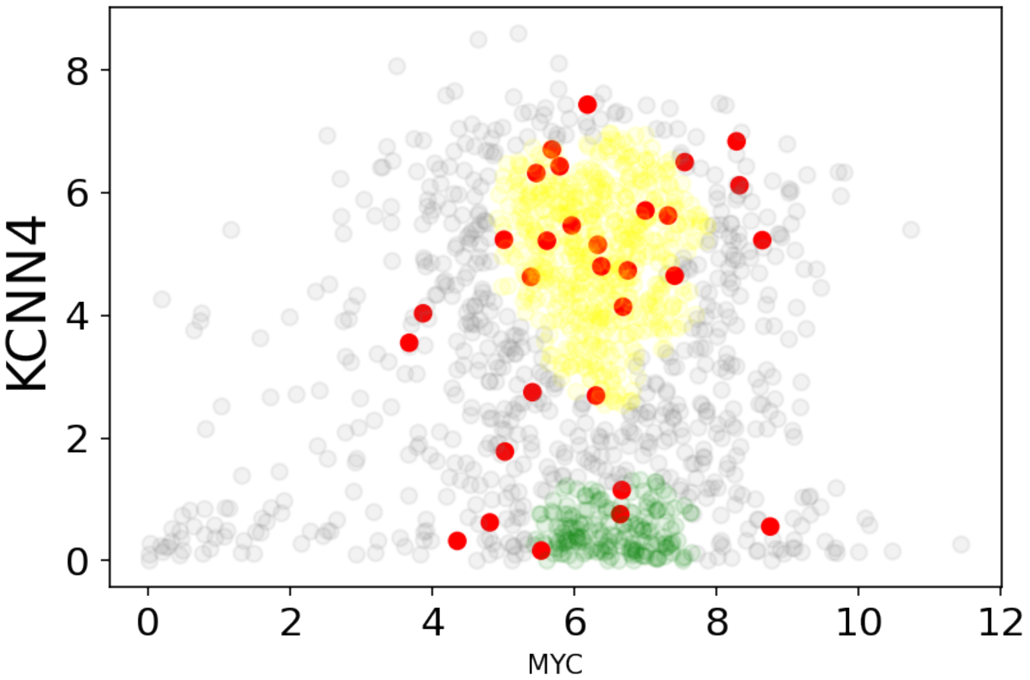

KCNN4
0
2
4
6
8
0
2
4
6
8
10
12
MYC

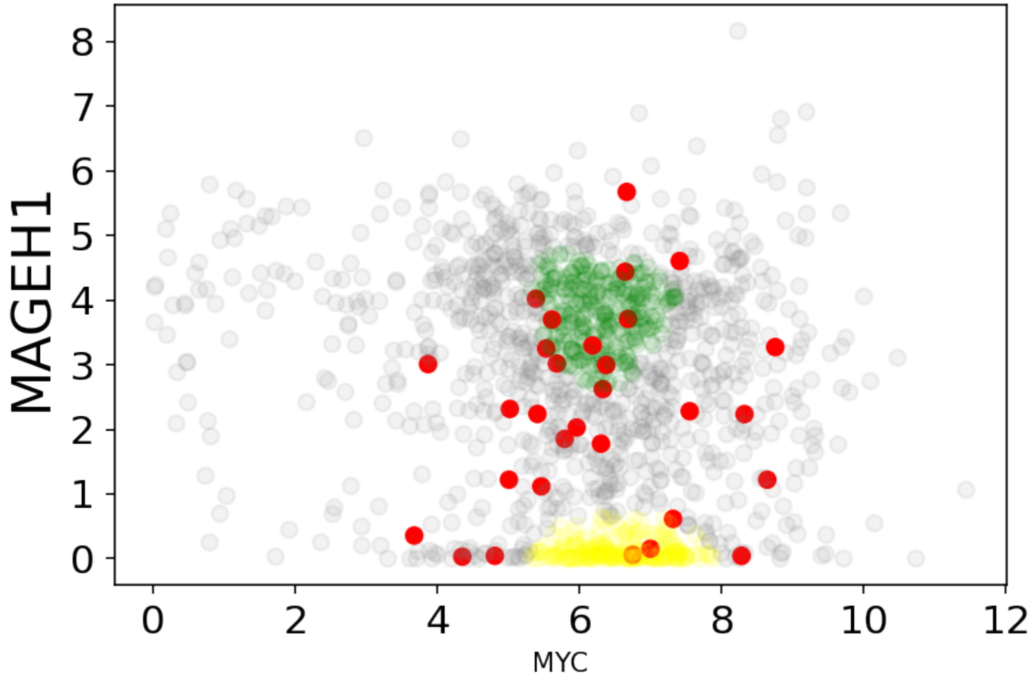

MAGEH1
MYC
0
1
2
3
4
5
6
7
8
0
2
4
6
8
10
12

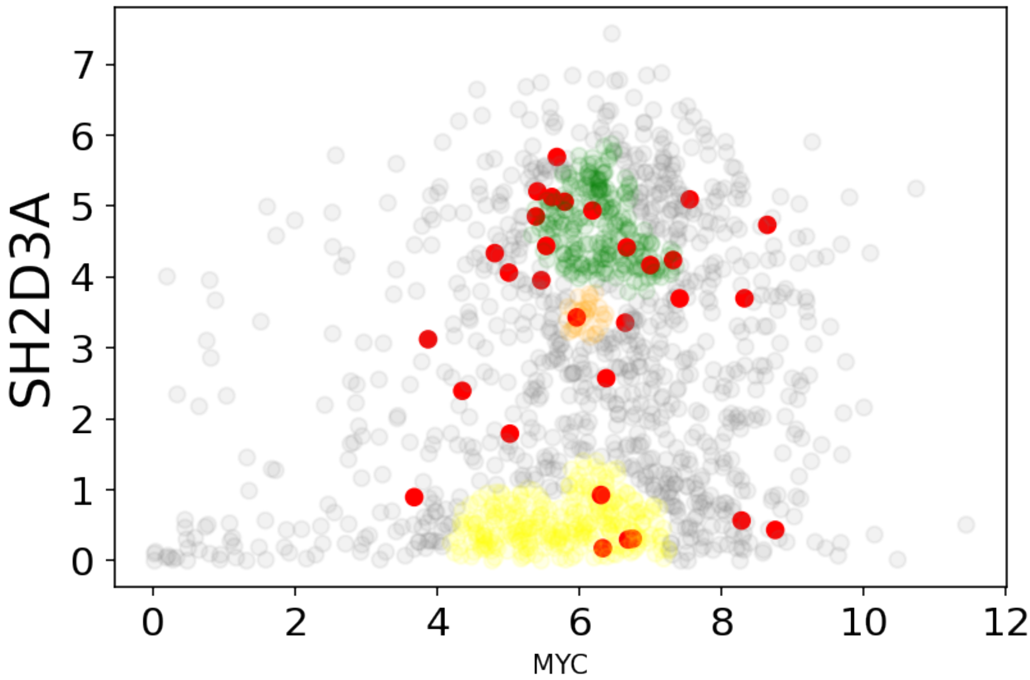

SH2D3A
MYC
0
1
2
3
4
5
6
7
0
2
4
6
8
10
12

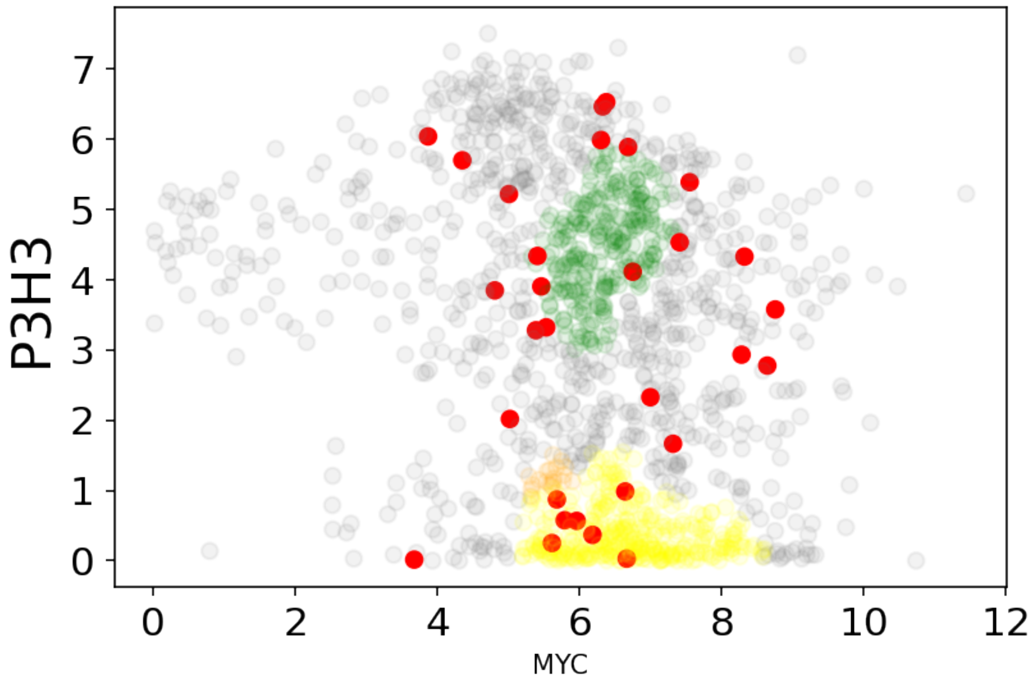

P3H3
0
1
2
3
4
5
6
7
0
2
4
6
8
10
12
MYC

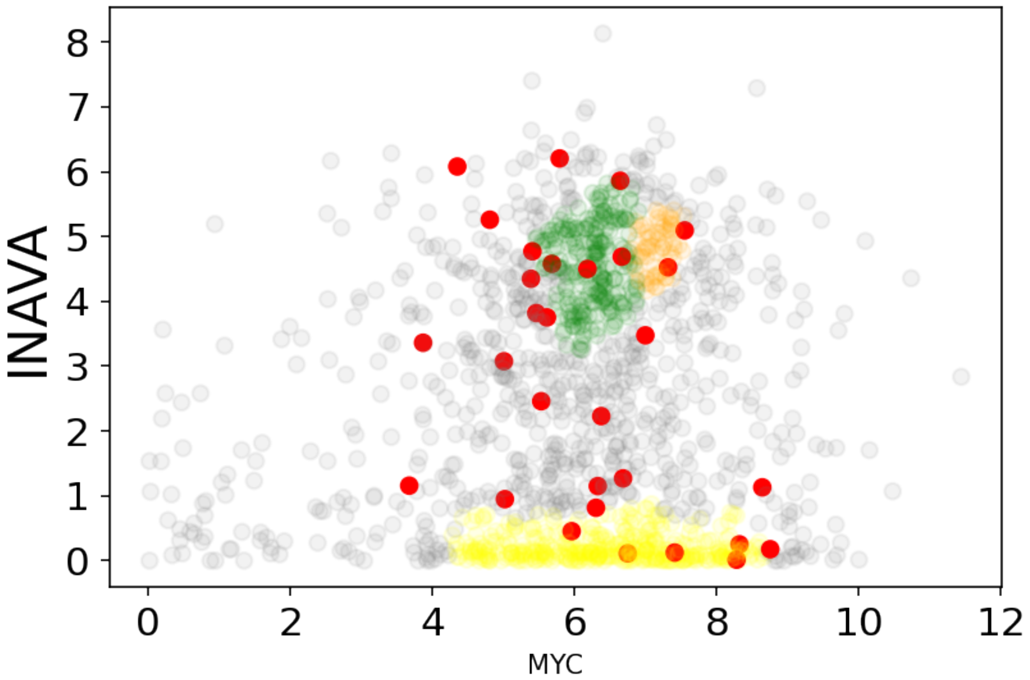

INAVA
MYC
0
1
2
3
4
5
6
7
8
0
2
4
6
8
10
12

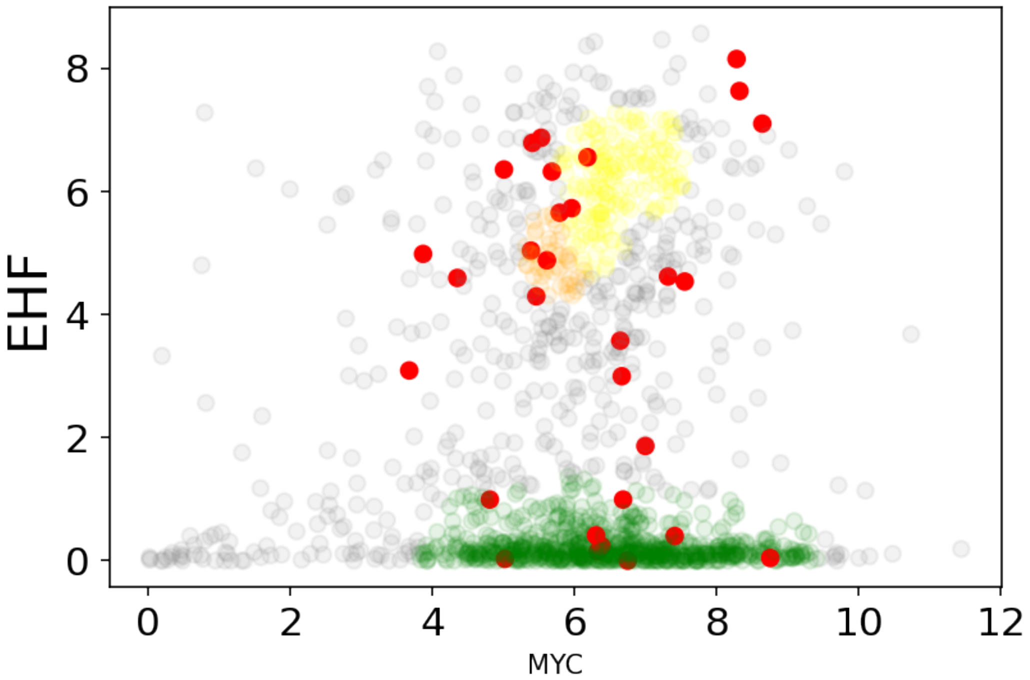
EHF
MYC
0
2
4
6
8
0
2
4
6
8
10
12

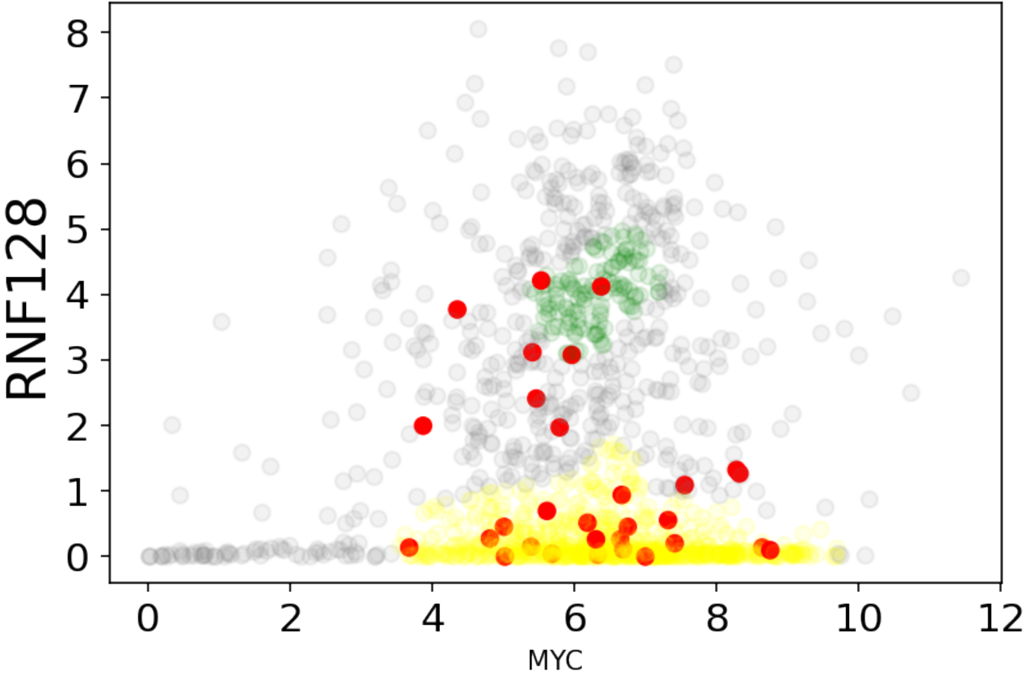
RNF128
8
7
6
5
4
3
2
1
0
0
2
4
6
8
10
12
MYC

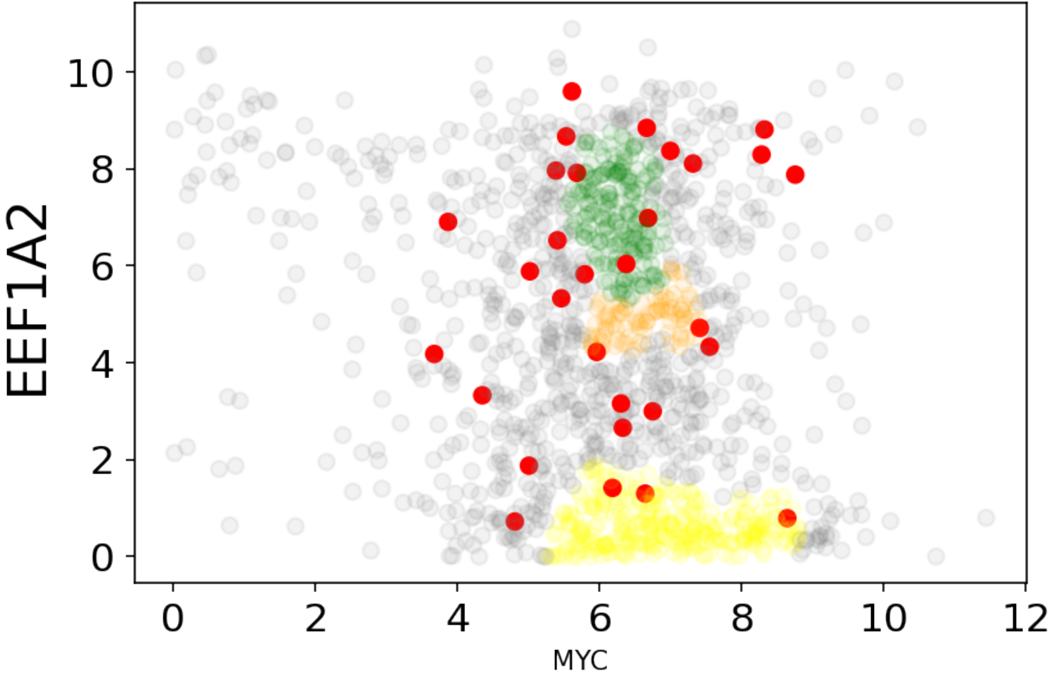
EEF1A2
0
2
4
6
8
10
0
2
4
6
8
10
12
MYC

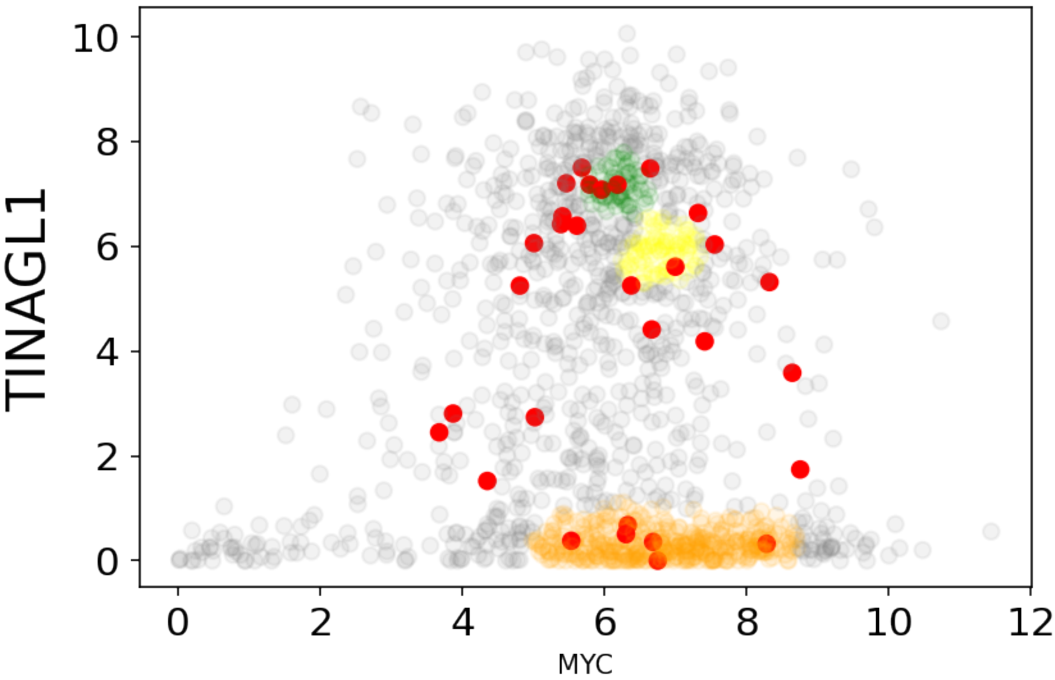

TINAGL1
MYC
0
2
4
6
8
10
12

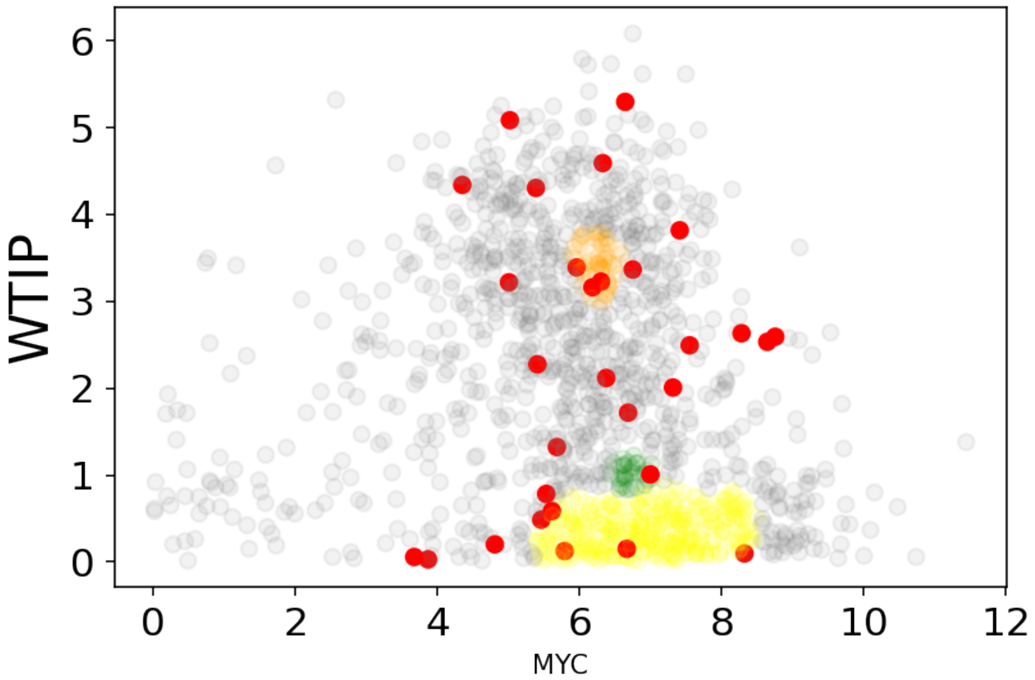

WTIP
0
1
2
3
4
5
6
0
2
4
6
8
10
12
MYC

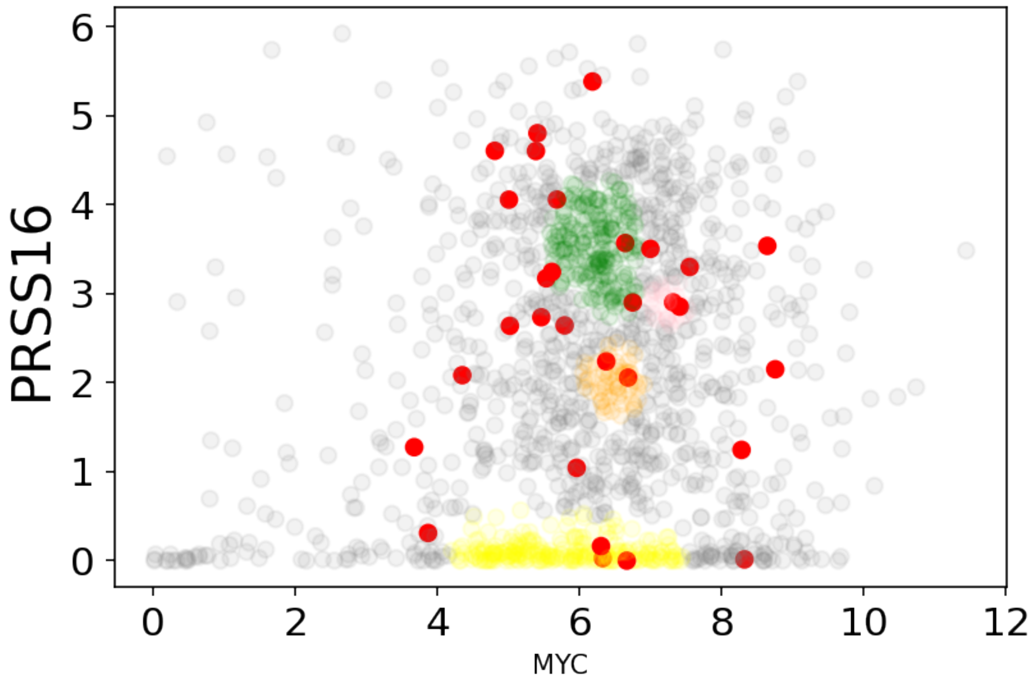
PRSS16
MYC
0
1
2
3
4
5
6
0
2
4
6
8
10
12

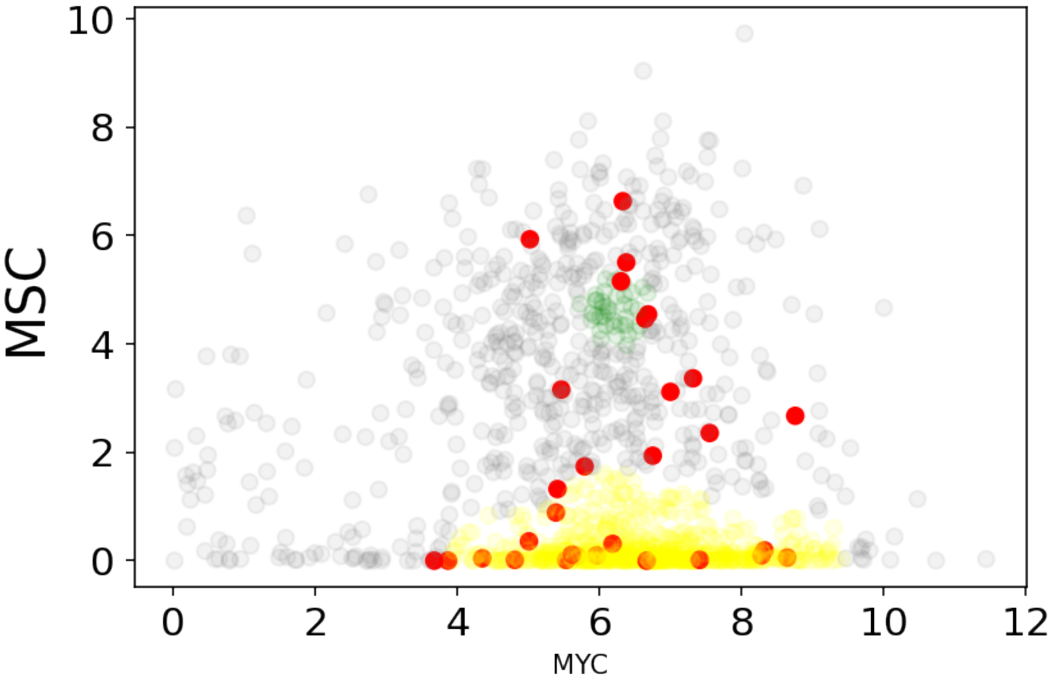

MSC
MYC
0
2
4
6
8
10
12

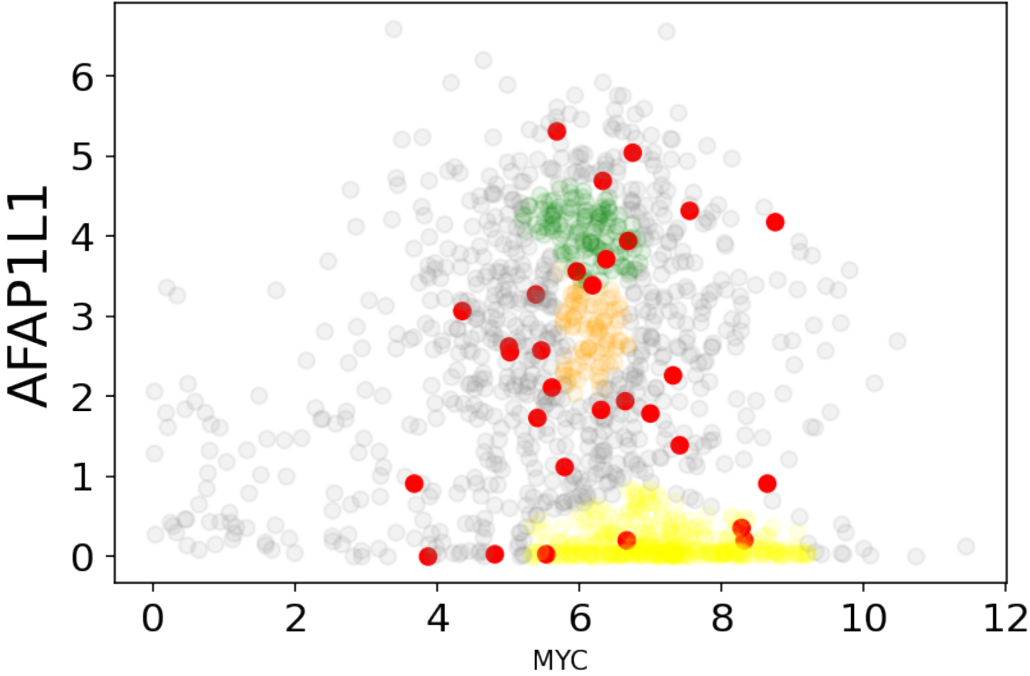

AFAP1L1
MYC
0
1
2
3
4
5
6
0
2
4
6
8
10
12

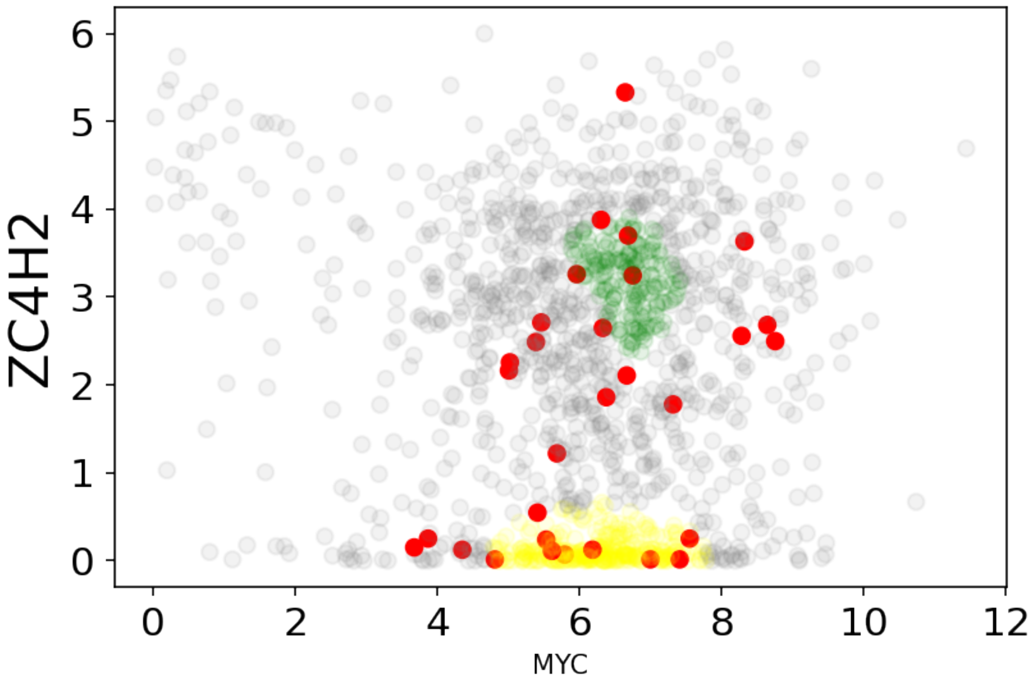
ZC4H2
6
5
4
3
2
1
0
0
2
4
6
8
10
12
MYC

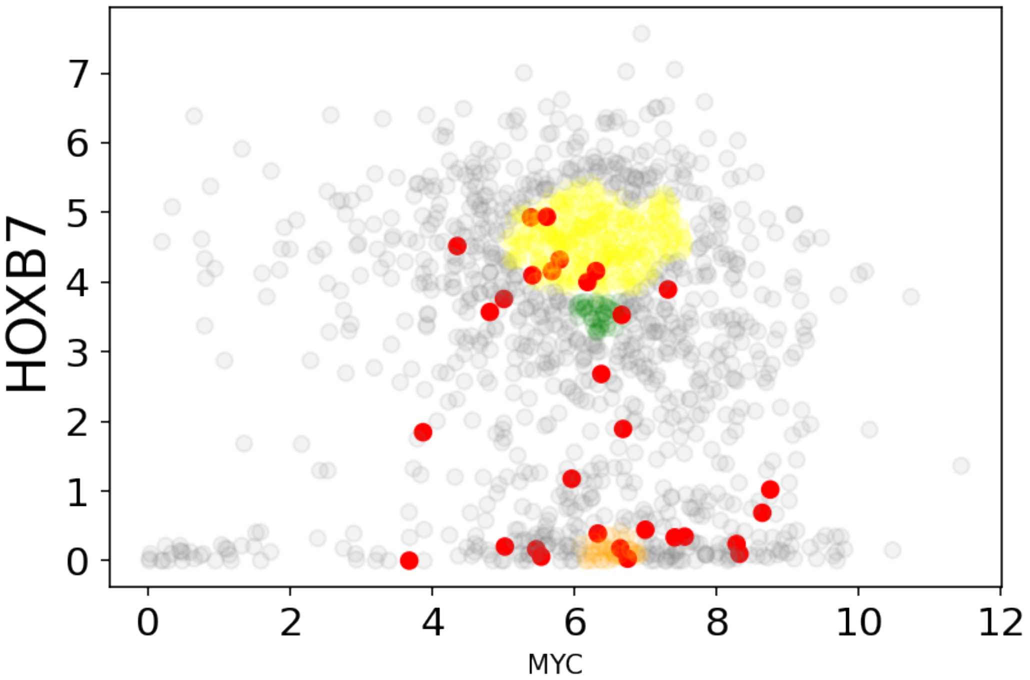

HOXB7
0
1
2
3
4
5
6
7
0
2
4
6
8
10
12
MYC

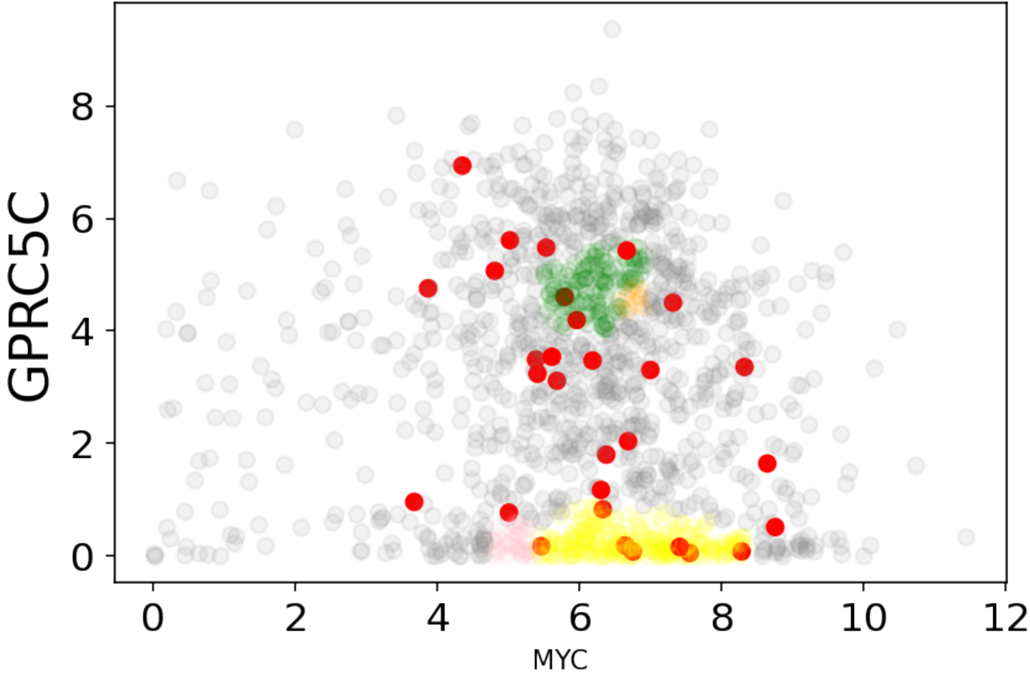
GPRC5C
0
2
4
6
8
0
2
4
6
8
10
12
MYC

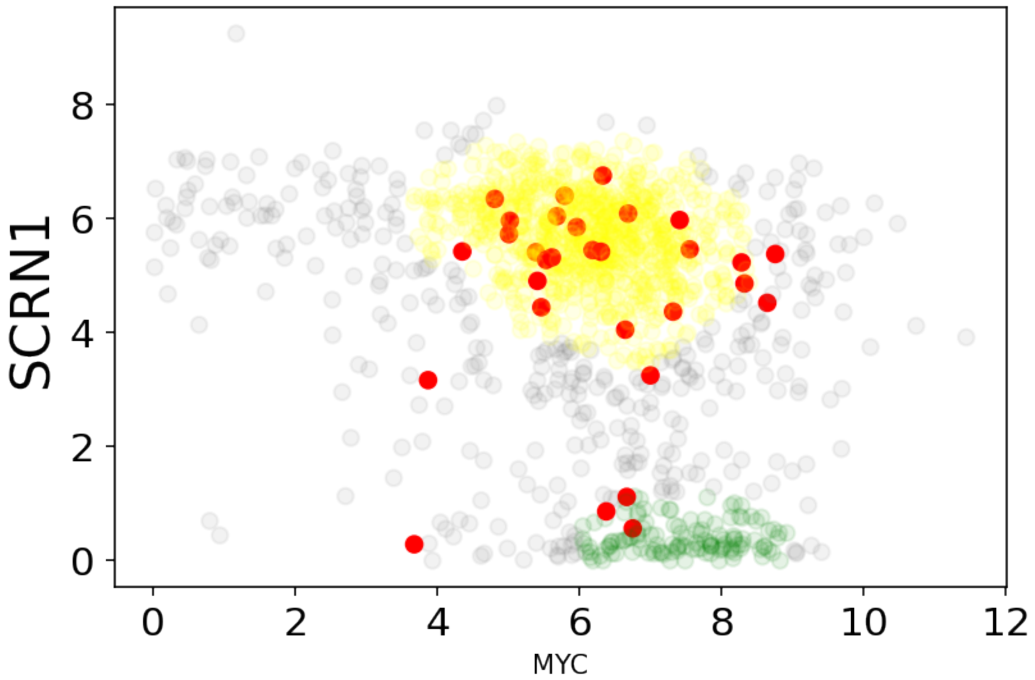

SCRN1
8
6
4
2
0
0
2
4
6
8
10
12
MYC

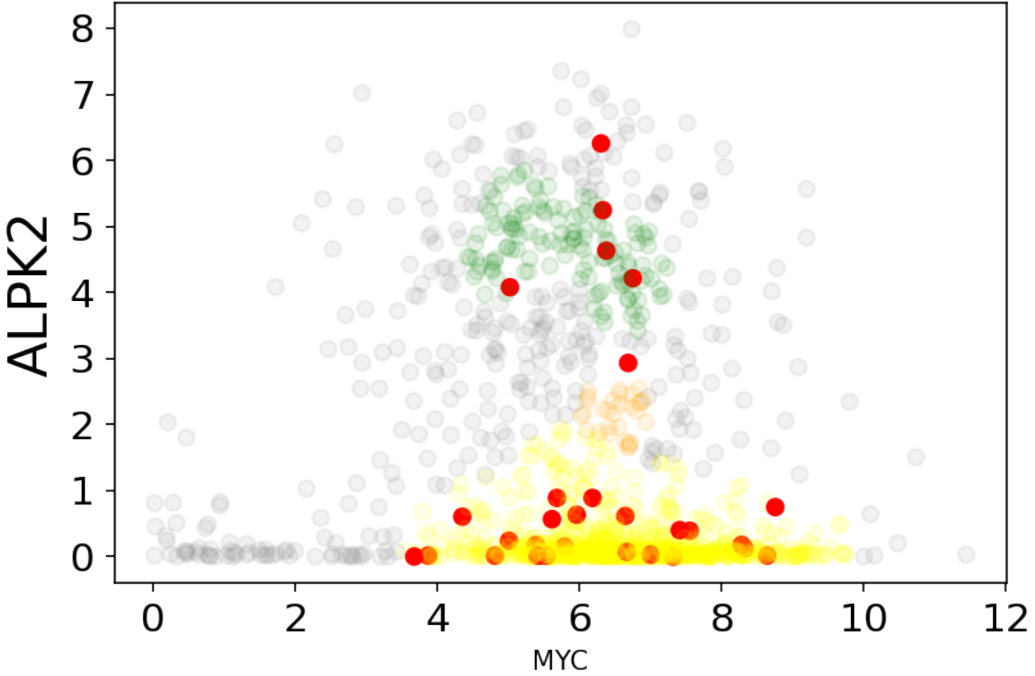

ALPK2
MYC
0
1
2
3
4
5
6
7
8
0
2
4
6
8
10
12

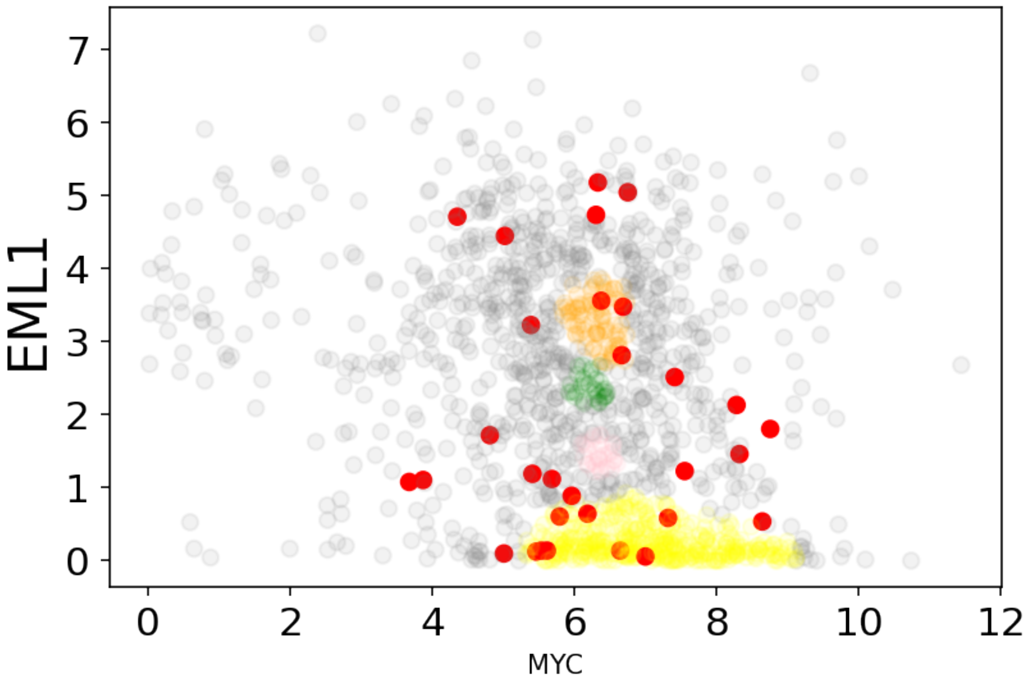

EML1
7
6
5
4
3
2
1
0
0
2
4
6
8
10
12
MYC

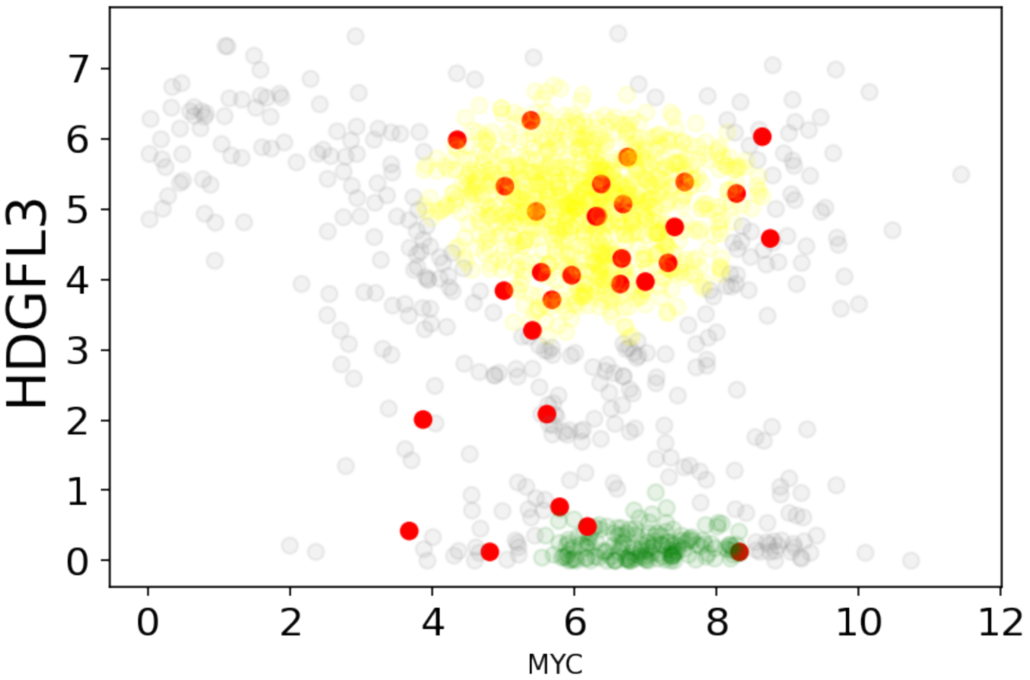
HDGFL3
MYC
0
1
2
3
4
5
6
7
0
2
4
6
8
10
12

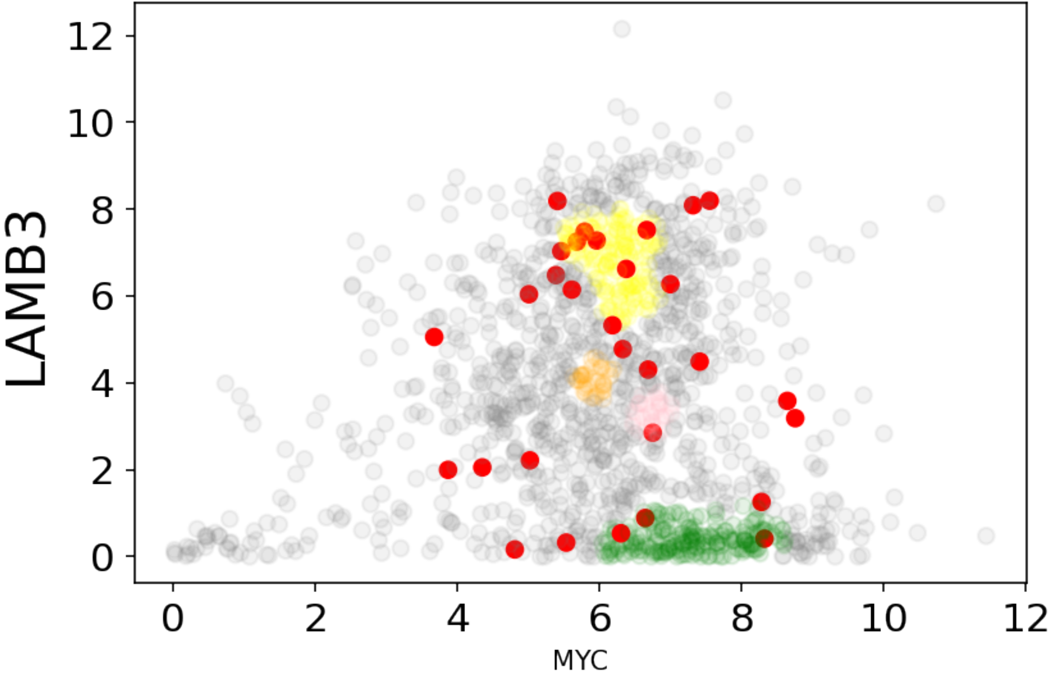

LAMB3
MYC
0
2
4
6
8
10
12

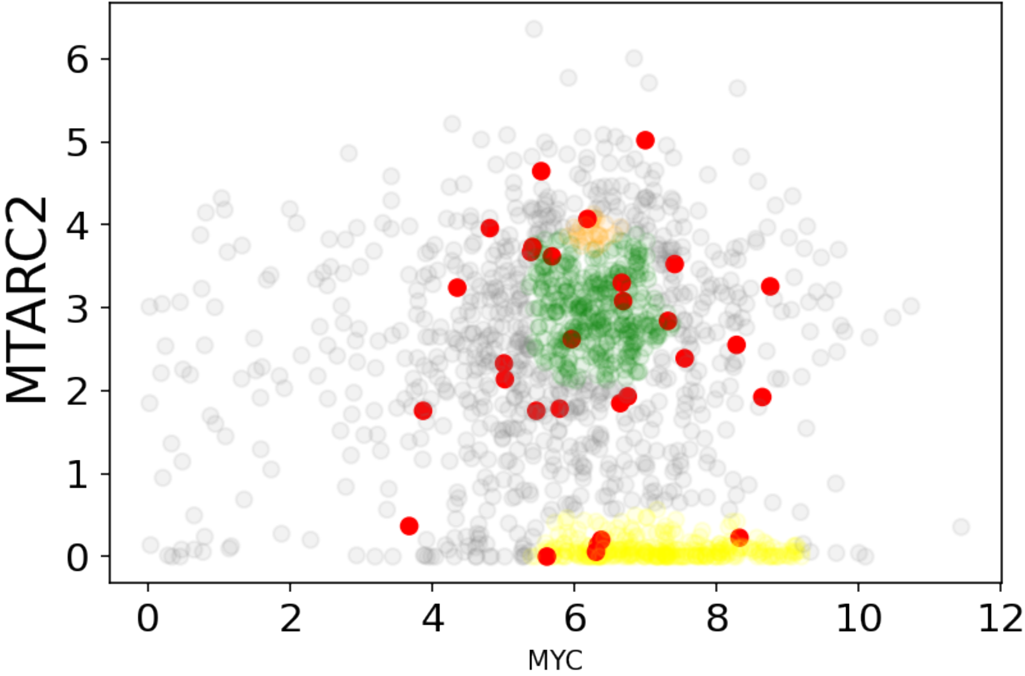

MTARC2
0
1
2
3
4
5
6
0
2
4
6
8
10
12
MYC

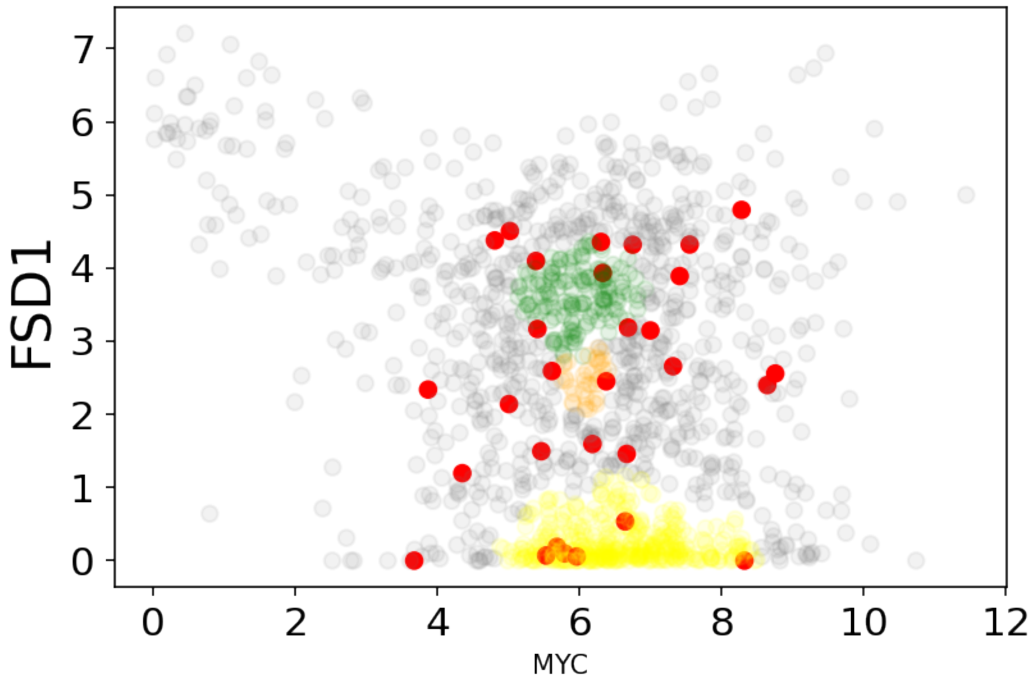
FSD1
MYC
0
1
2
3
4
5
6
7
0
2
4
6
8
10
12

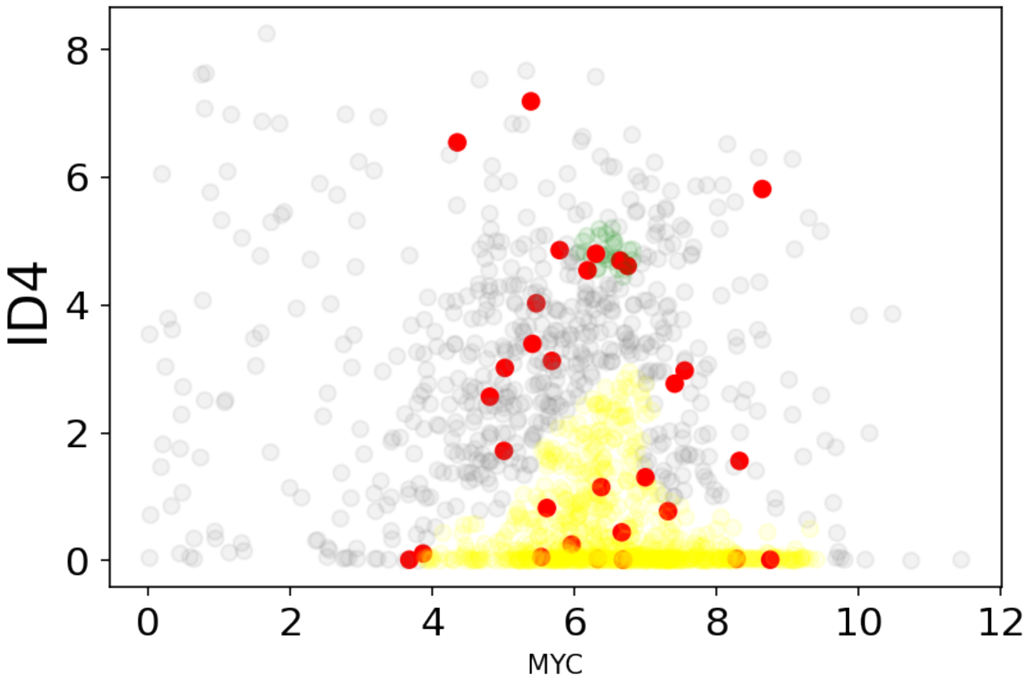

ID4
8
6
4
2
0
0
2
4
6
8
10
12
MYC

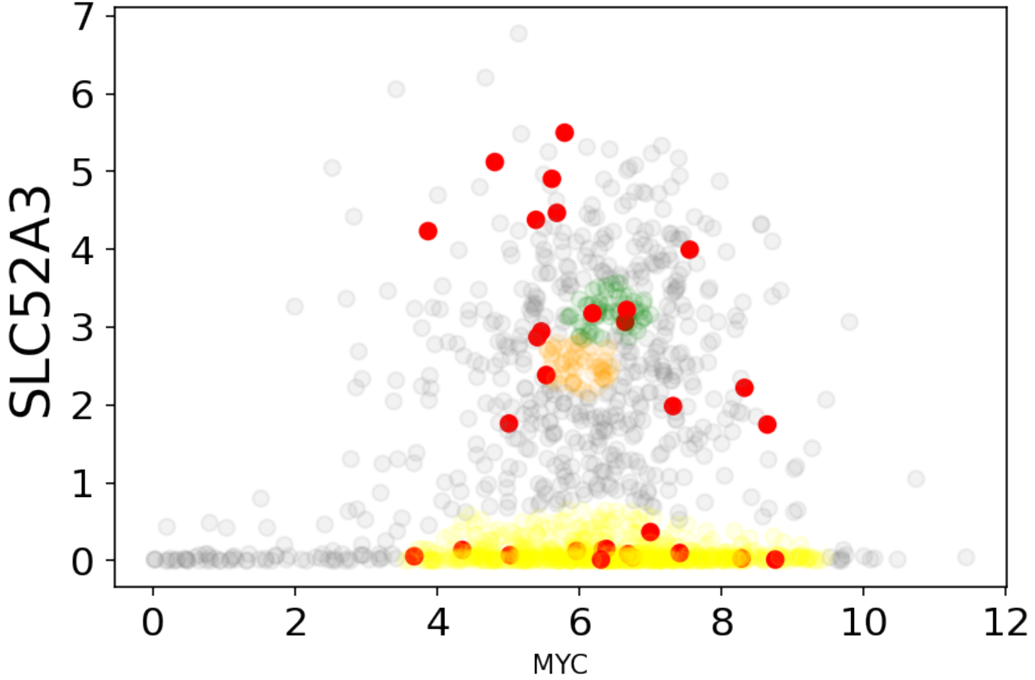

SLC52A3
MYC
0
1
2
3
4
5
6
7
0
2
4
6
8
10
12

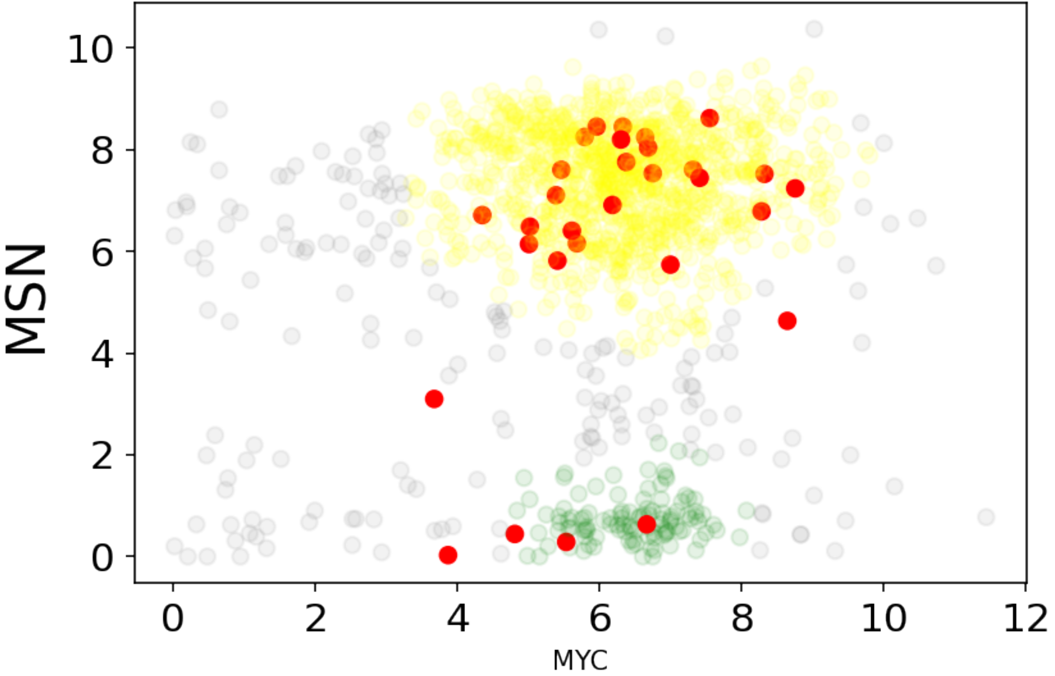

MSN
10
8
6
4
2
0
0
2
4
6
8
10
12
MYC

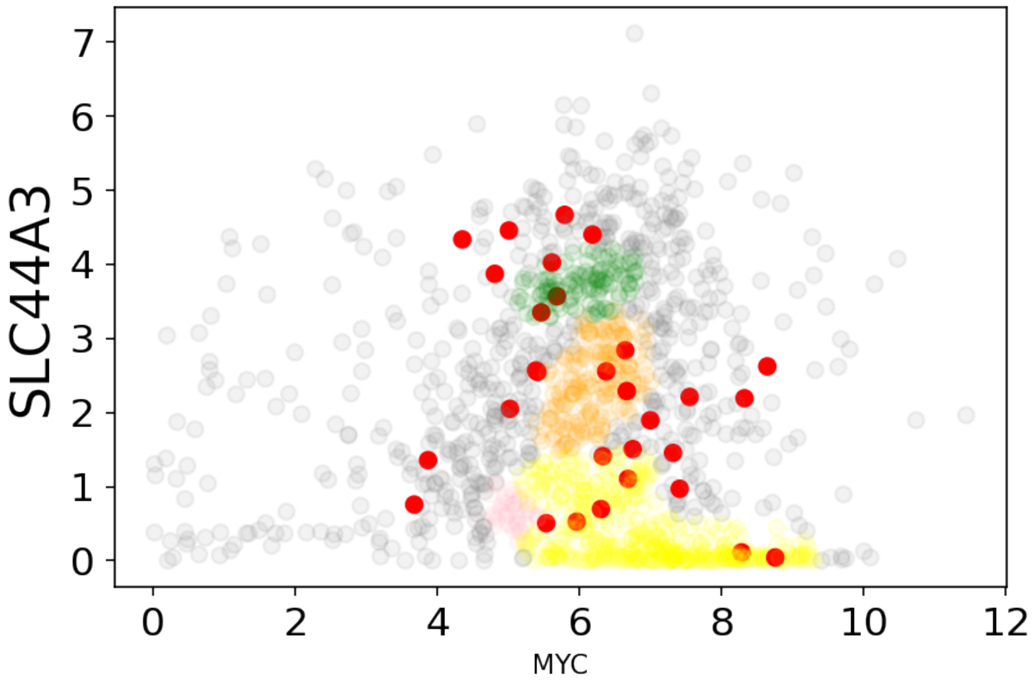
SLC44A3
7
6
5
4
3
2
1
0
0
2
4
6
8
10
12
MYC

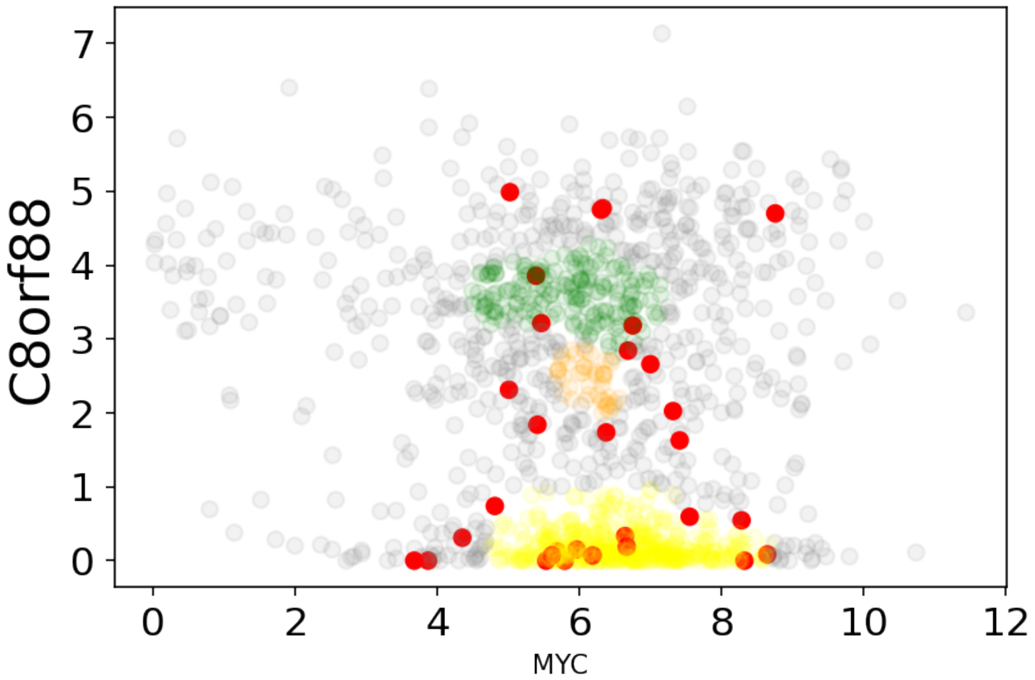
C8orf88
MYC
0
1
2
3
4
5
6
7
0
2
4
6
8
10
12

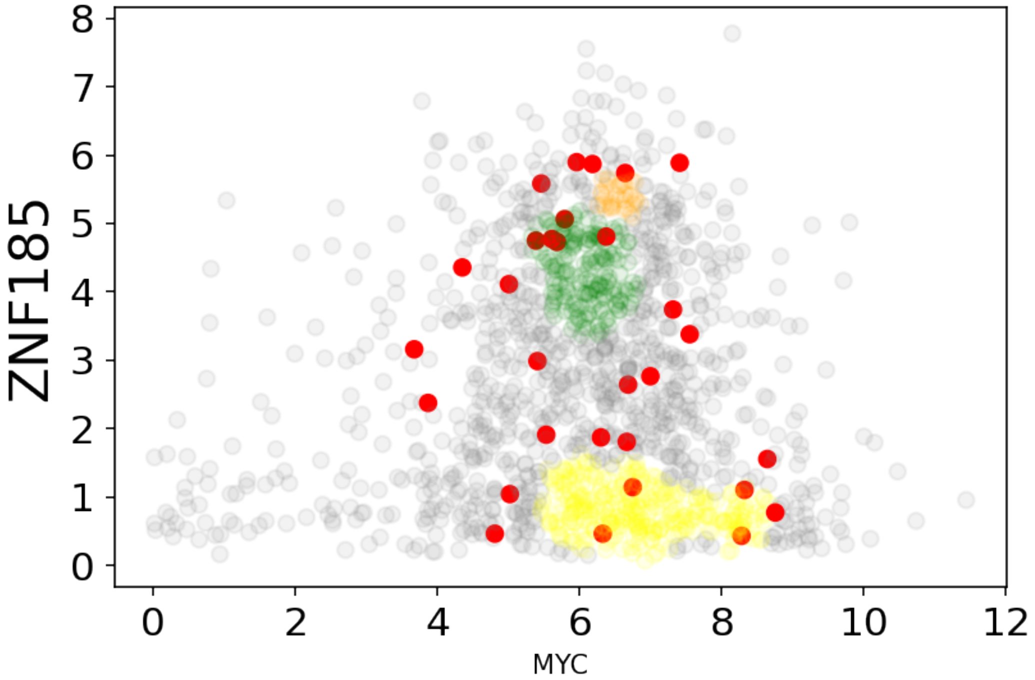

ZNF185
MYC
0
1
2
3
4
5
6
7
8
0
2
4
6
8
10
12

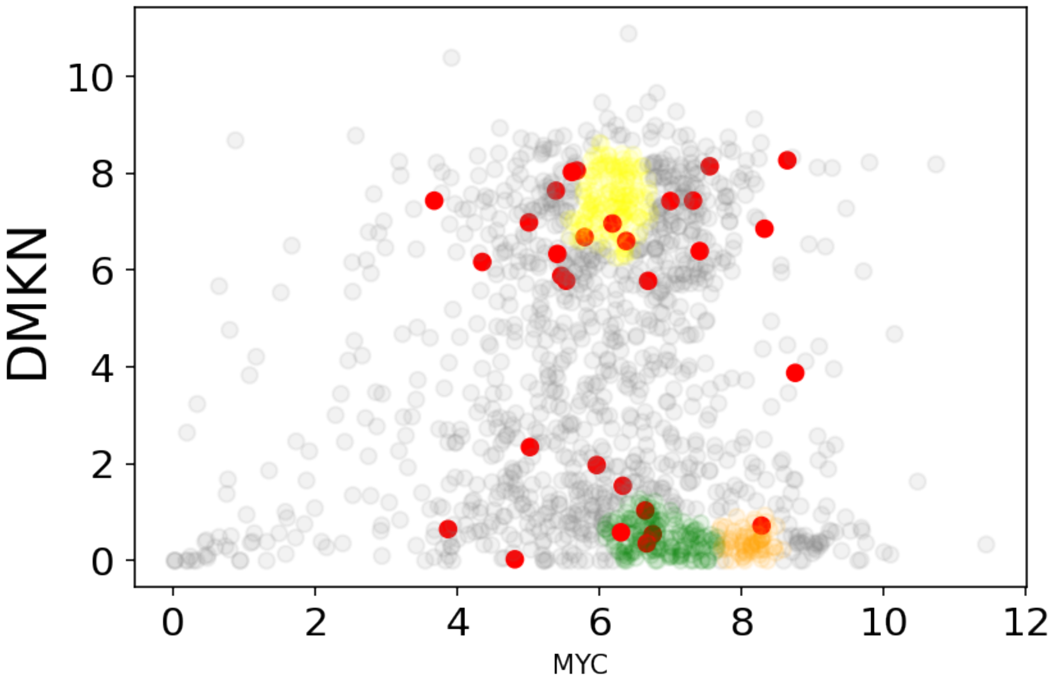

DMKN
10
8
6
4
2
0
0
2
4
6
8
10
12
MYC

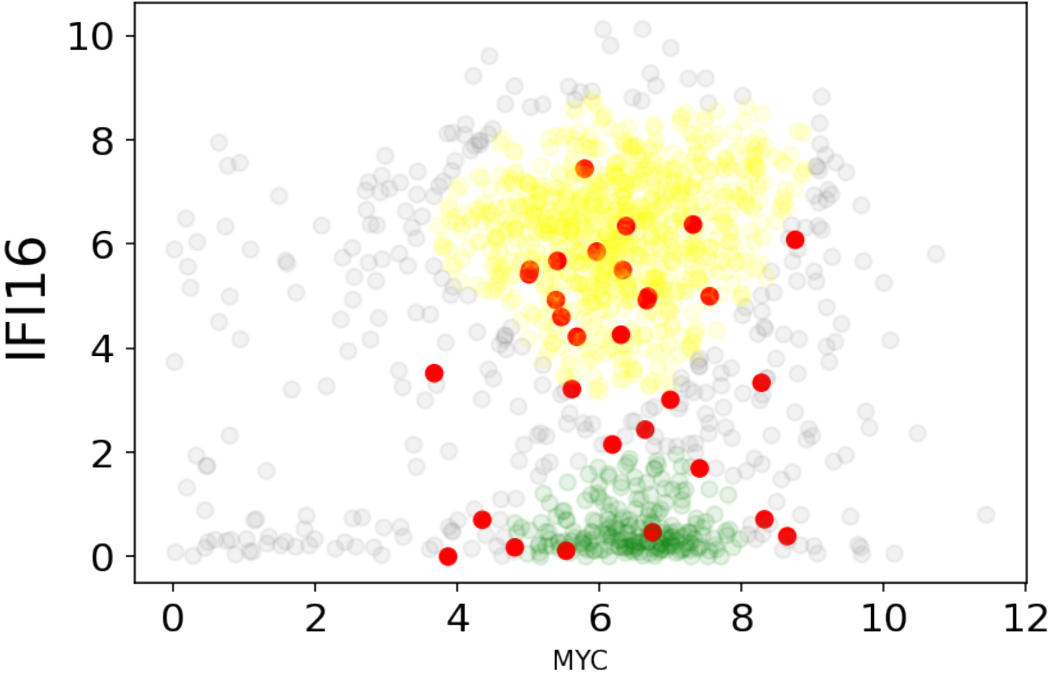
IFI16
MYC
0
2
4
6
8
10
12

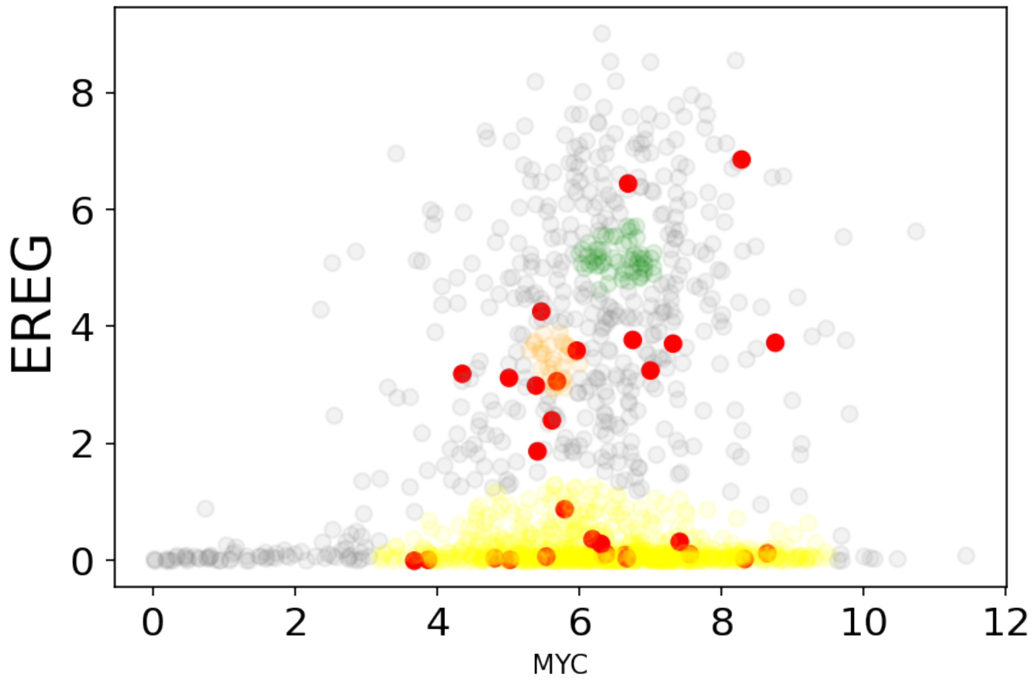

EREG
MYC
0
2
4
6
8
10
12

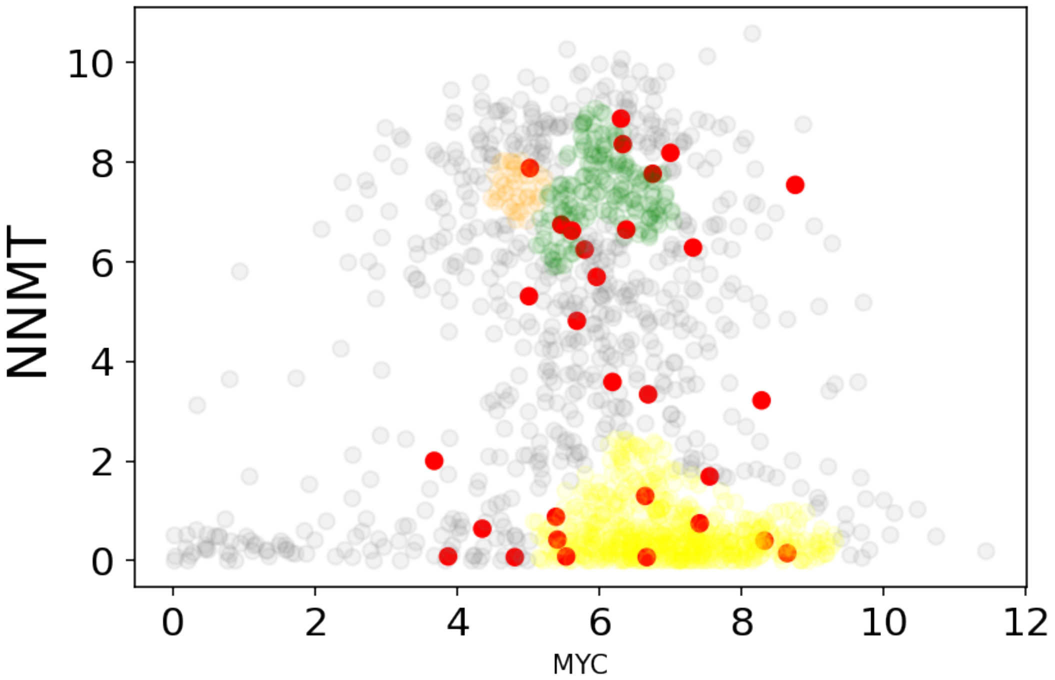

NNMT
0
2
4
6
8
10
0
2
4
6
8
10
12
MYC

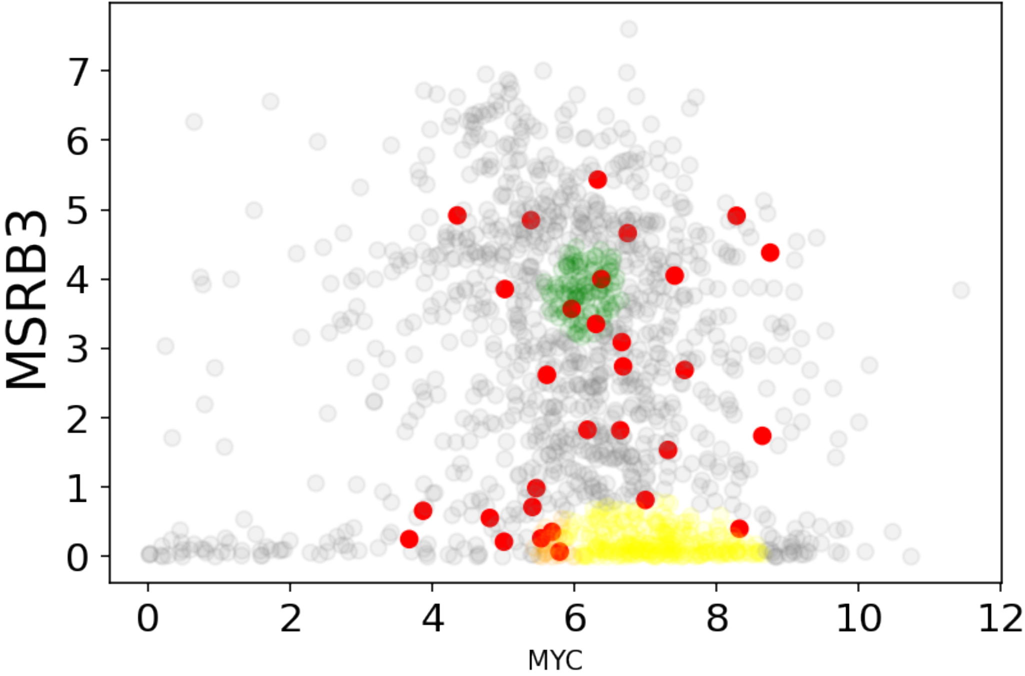

MSRB3
0
1
2
3
4
5
6
7
0
2
4
6
8
10
12
MYC

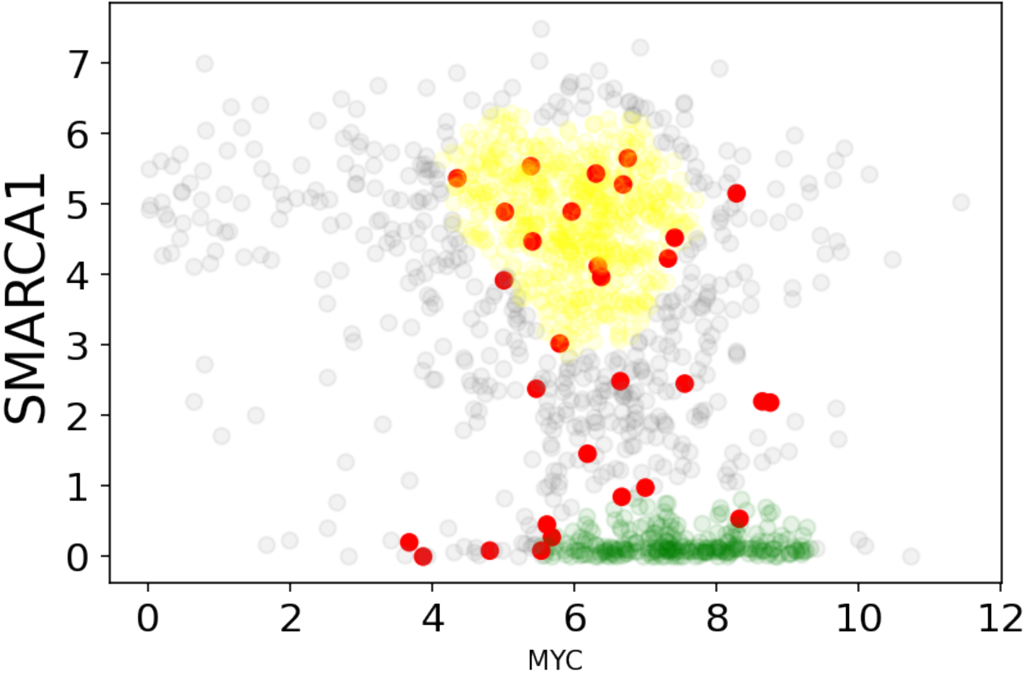
SMARCA1
MYC
0
1
2
3
4
5
6
7
0
2
4
6
8
10
12

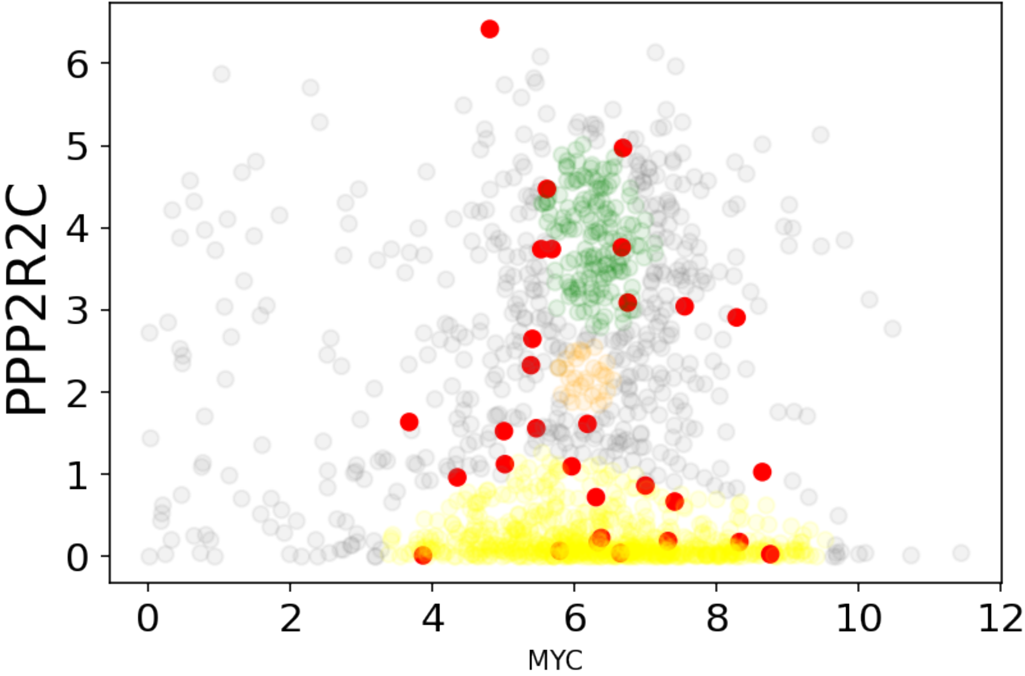

PPP2R2C
MYC
0
1
2
3
4
5
6
0
2
4
6
8
10
12

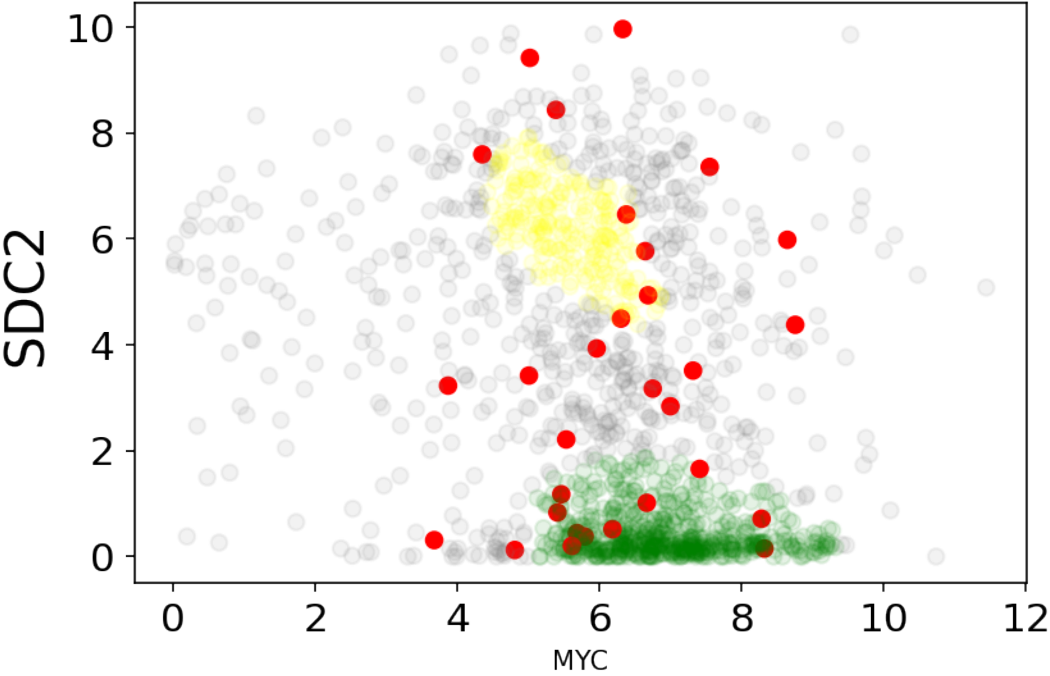

SDC2
MYC
10
8
6
4
2
0
0
2
4
6
8
10
12

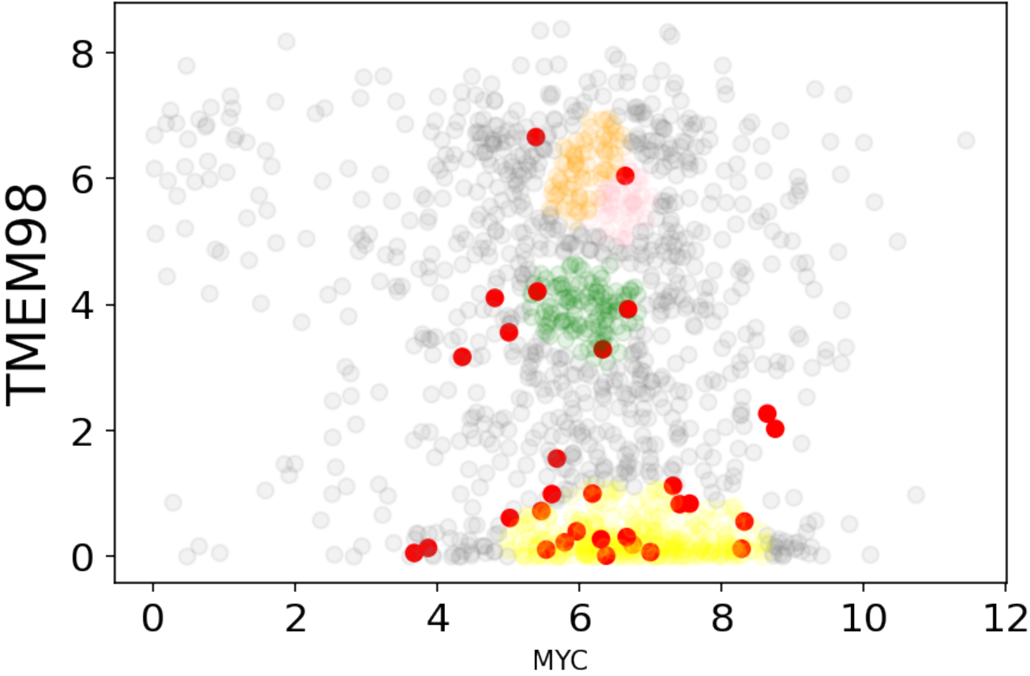

TMEM98
MYC
0
2
4
6
8
0
2
4
6
8
10
12

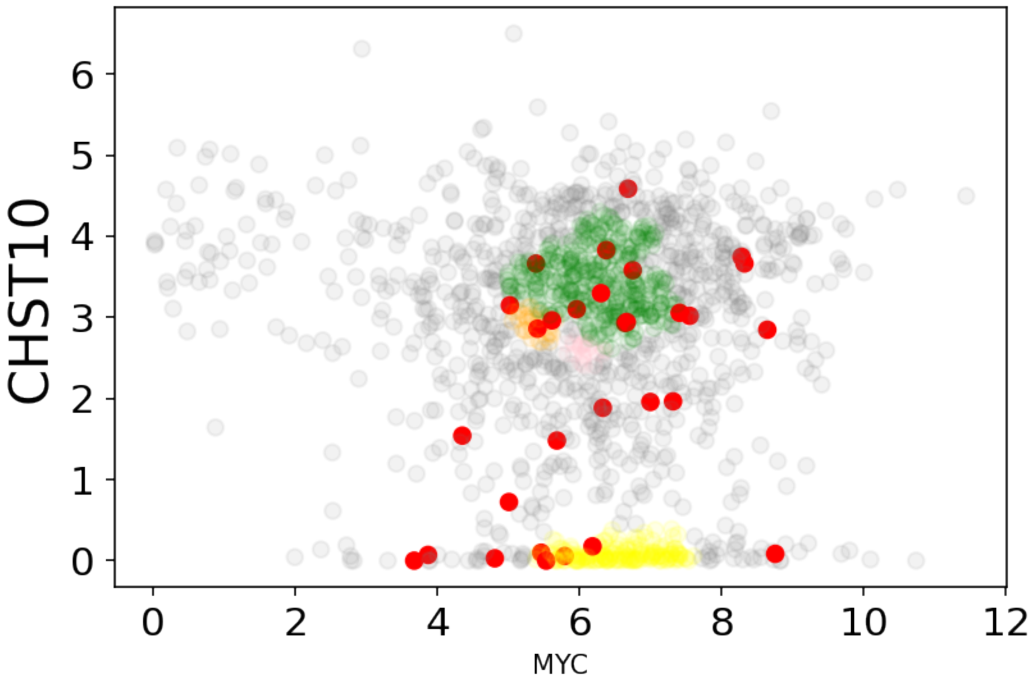
CHST10
MYC
0
1
2
3
4
5
6
0
2
4
6
8
10
12

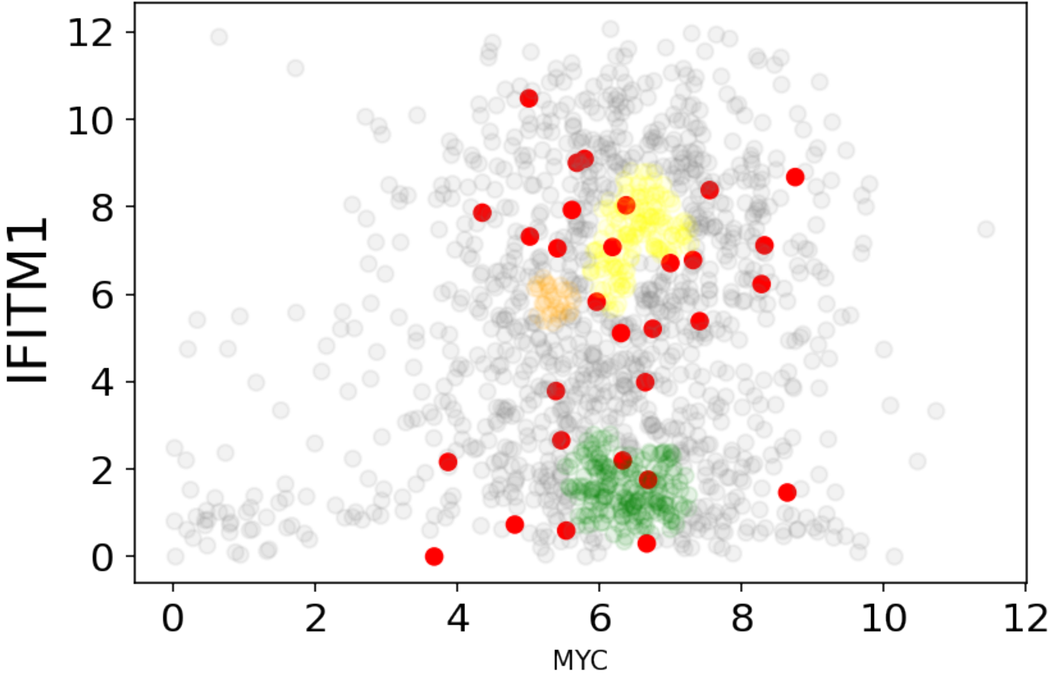

IFITM1
MYC
0
2
4
6
8
10
12

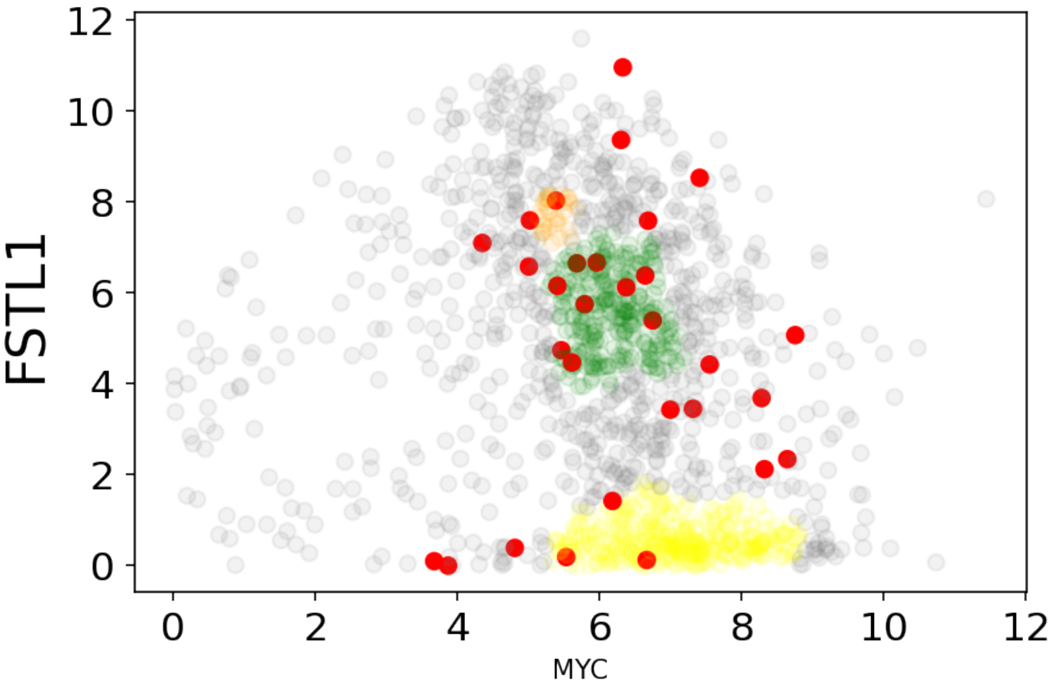

FSTL1
MYC
0
2
4
6
8
10
12

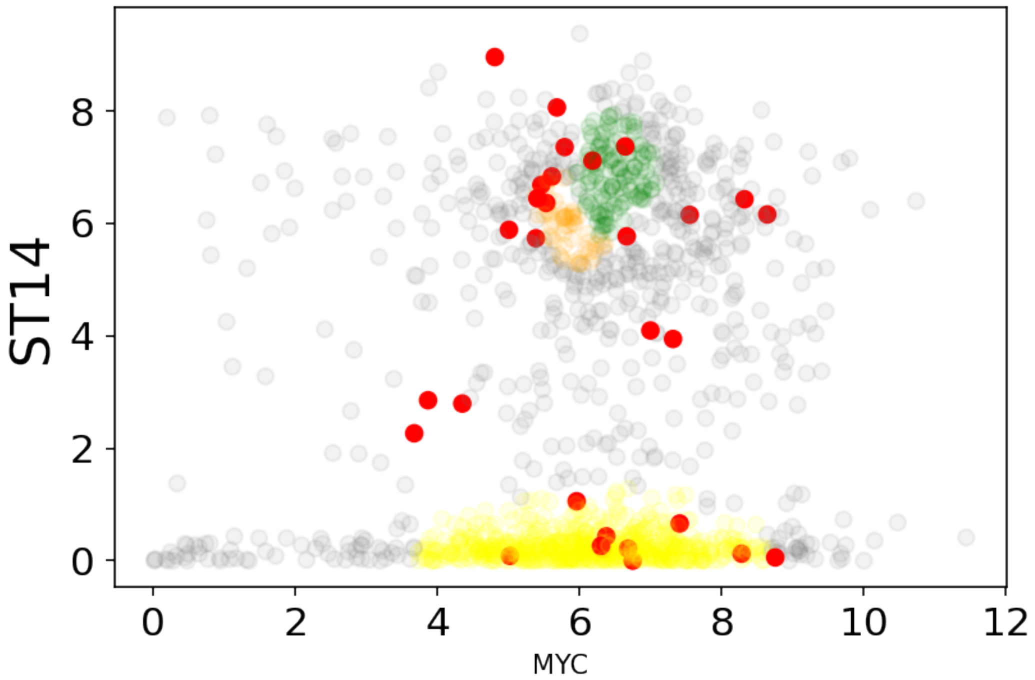
ST14
MYC
0
2
4
6
8
10
12

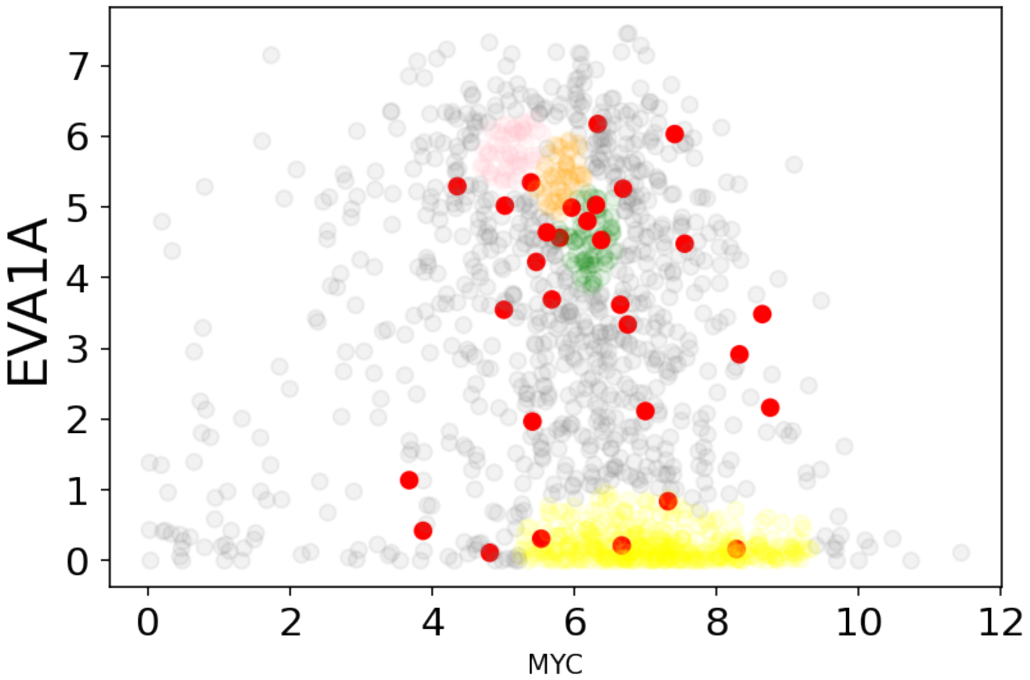
EVA1A
7
6
5
4
3
2
1
0
0
2
4
6
8
10
12
MYC

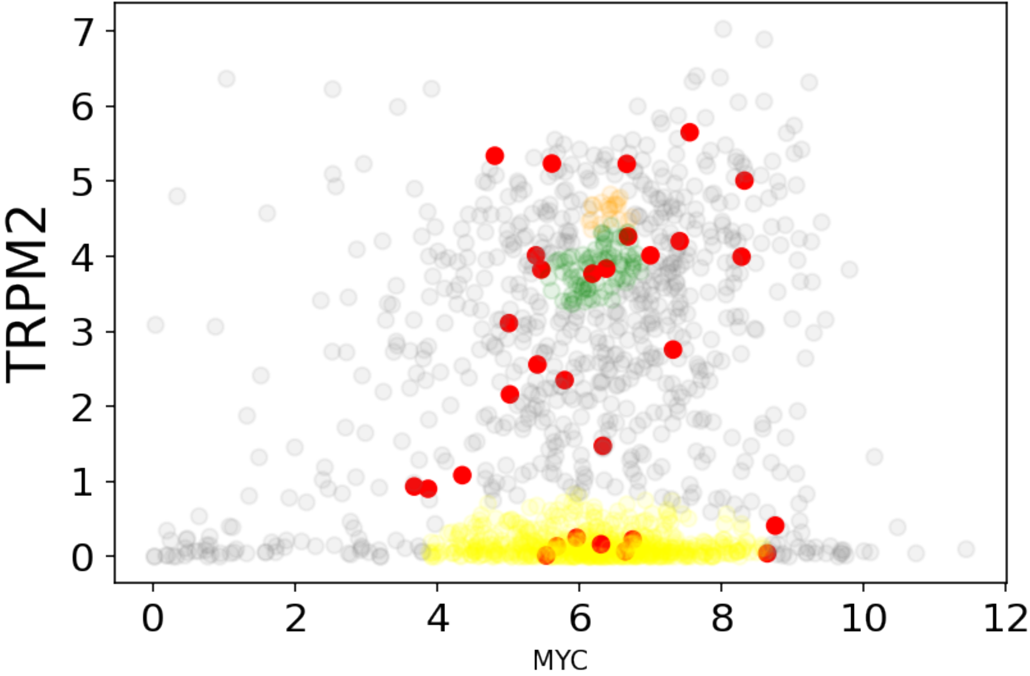

TRPM2
MYC
0
1
2
3
4
5
6
7
0
2
4
6
8
10
12

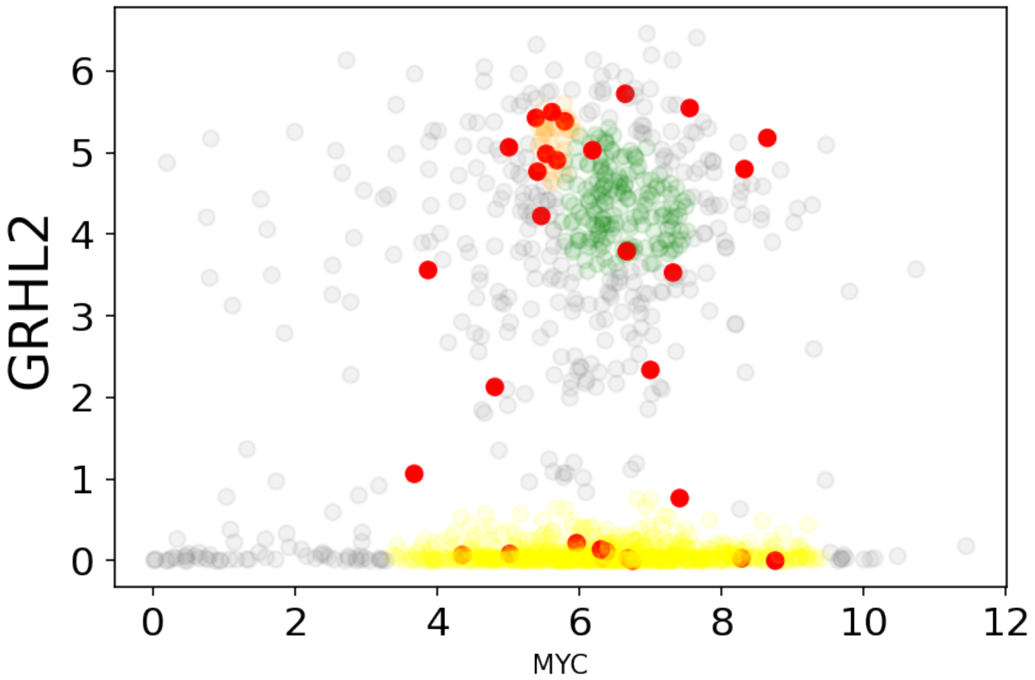

GRHL2
MYC
0
1
2
3
4
5
6
0
2
4
6
8
10
12

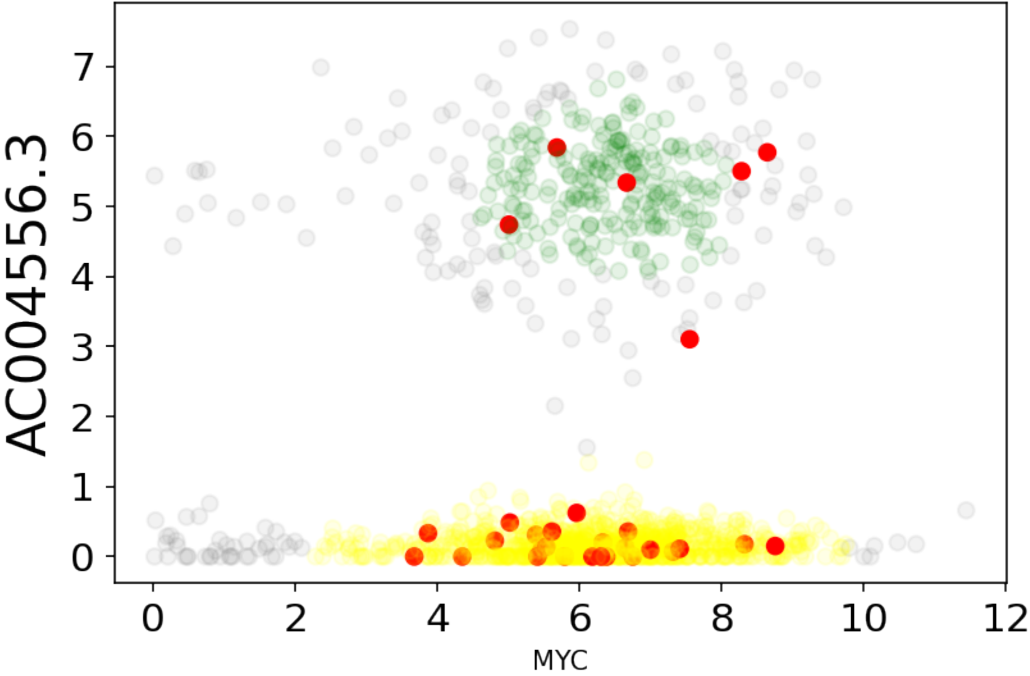
AC004556.3
MYC
0
1
2
3
4
5
6
7
0
2
4
6
8
10
12

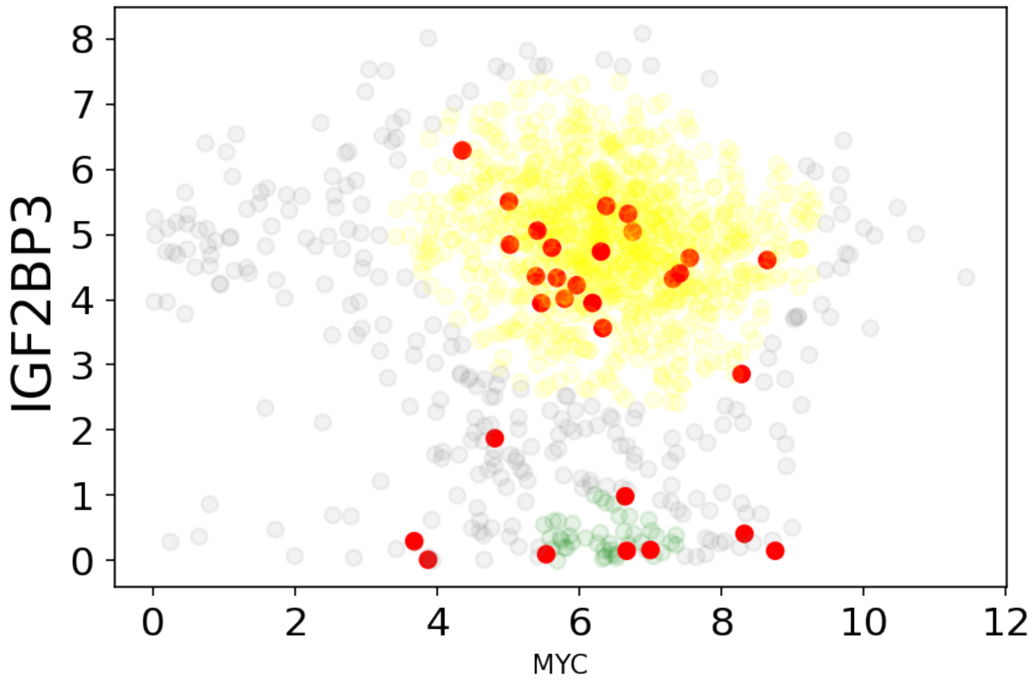

IGF2BP3
MYC
0
1
2
3
4
5
6
7
8
0
2
4
6
8
10
12

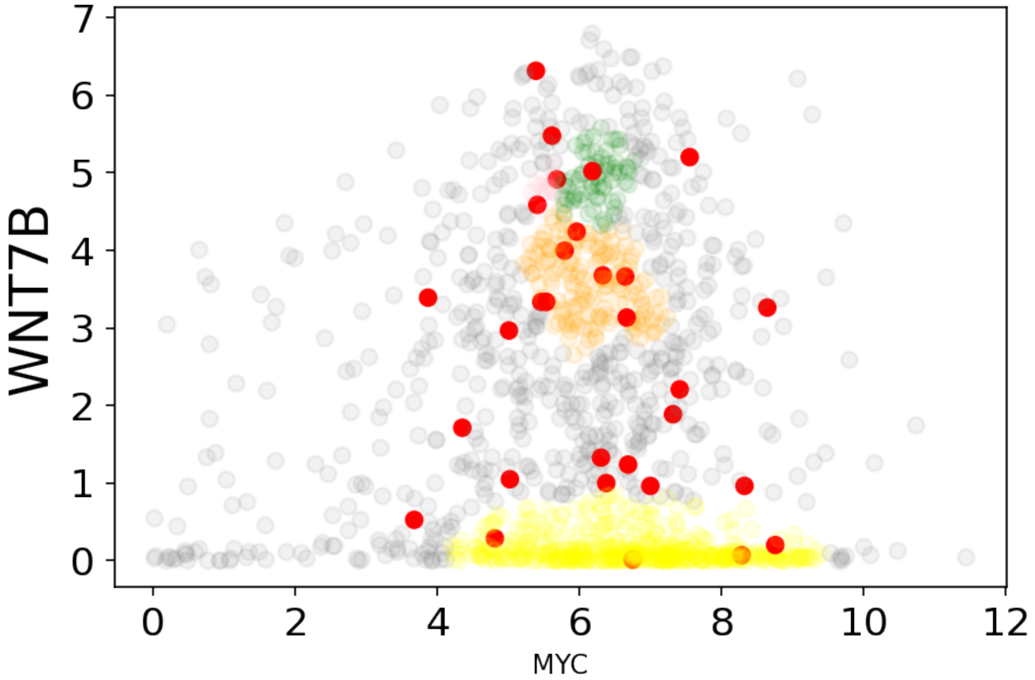
WNT7B
7
6
5
4
3
2
1
0
0
2
4
6
8
10
12
MYC

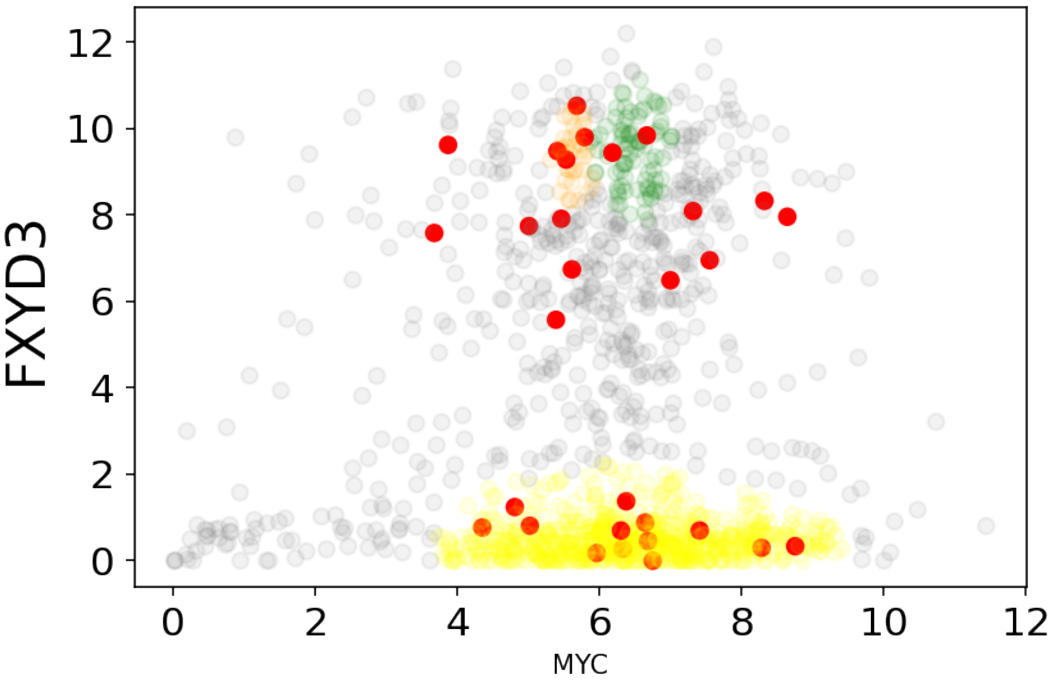

FXYD3
MYC
0
2
4
6
8
10
12

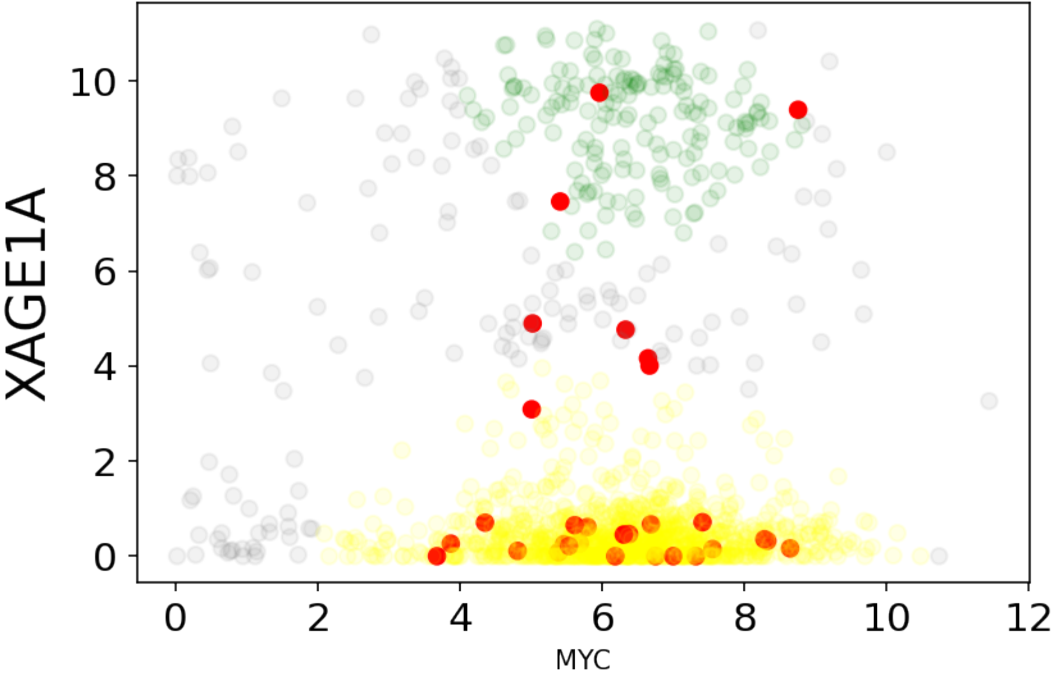

XAGE1A
0
2
4
6
8
10
0
2
4
6
8
10
12
MYC

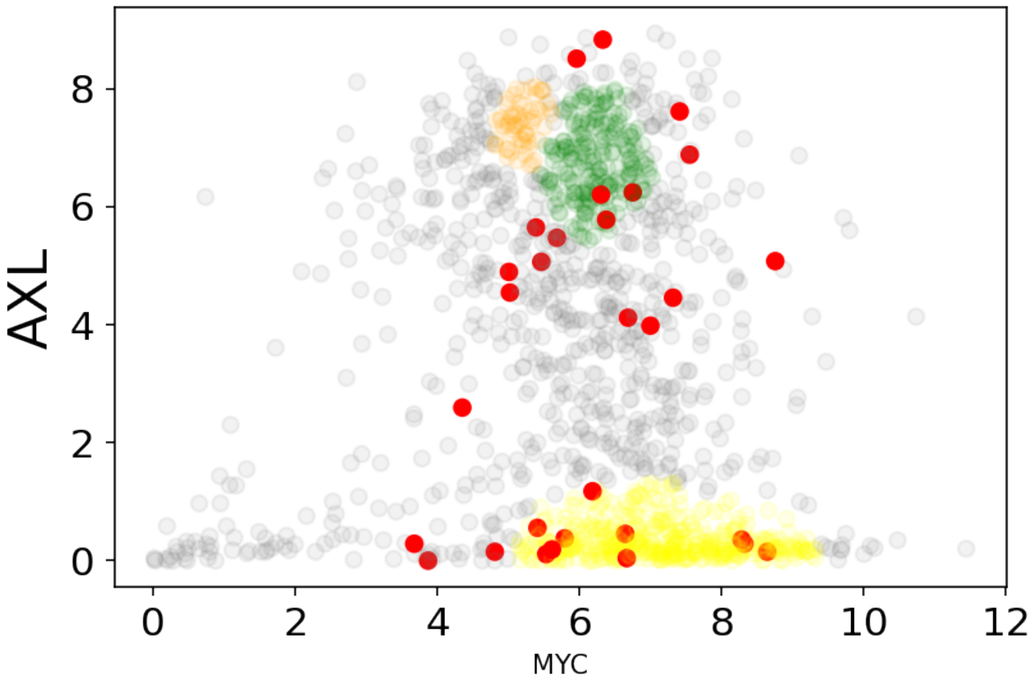

AXL
8
6
4
2
0
0
2
4
6
8
10
12
MYC

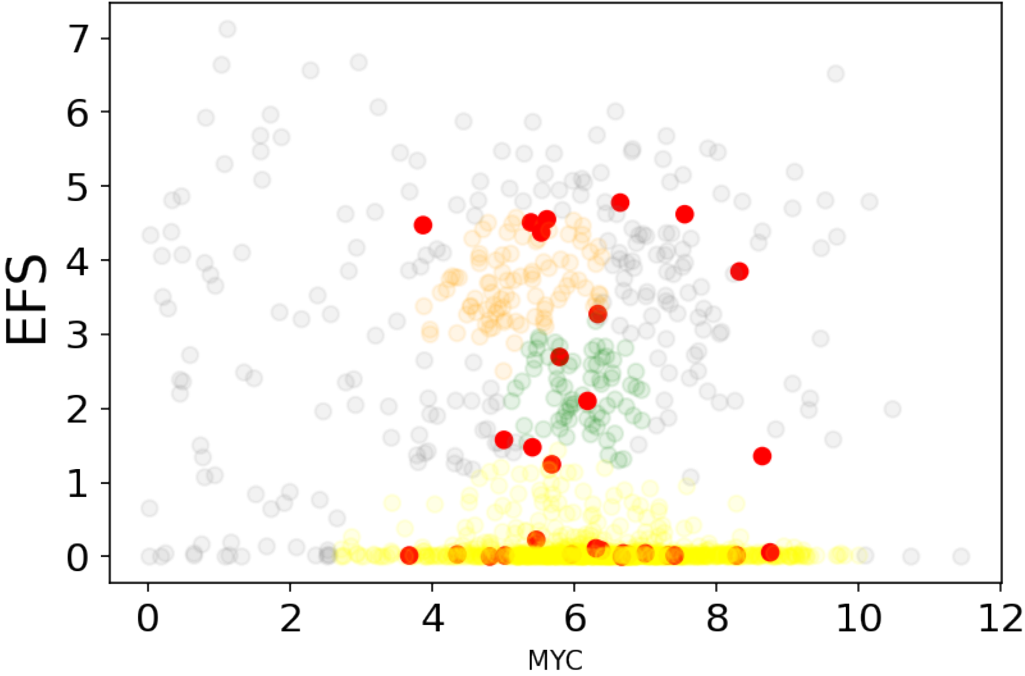
EFS
MYC
0
1
2
3
4
5
6
7
0
2
4
6
8
10
12

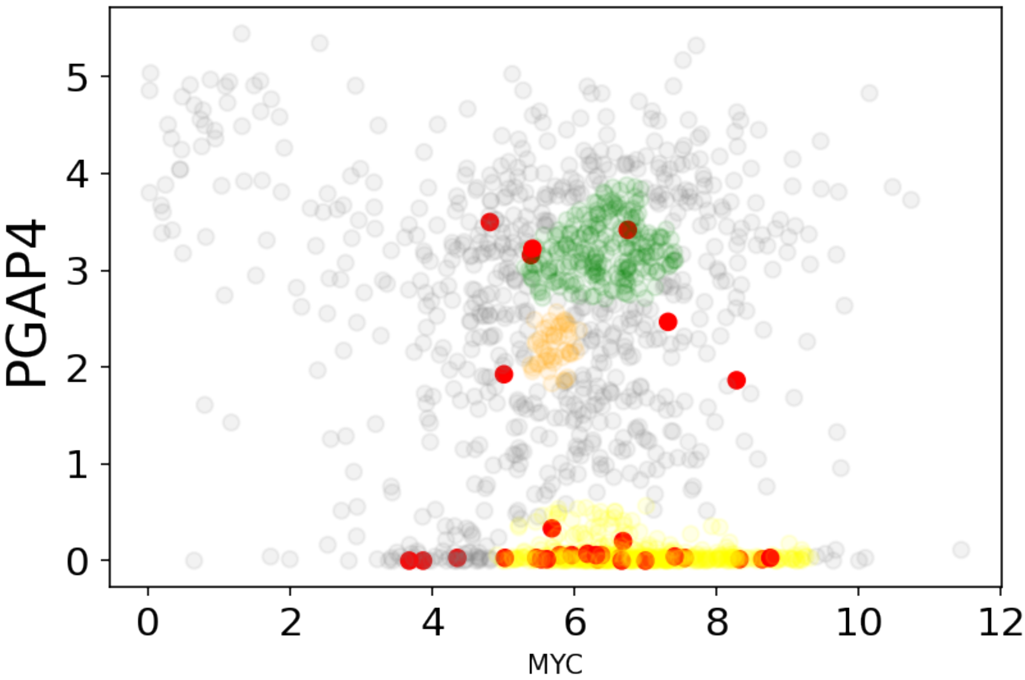
PGAP4
MYC
0
1
2
3
4
5
0
2
4
6
8
10
12

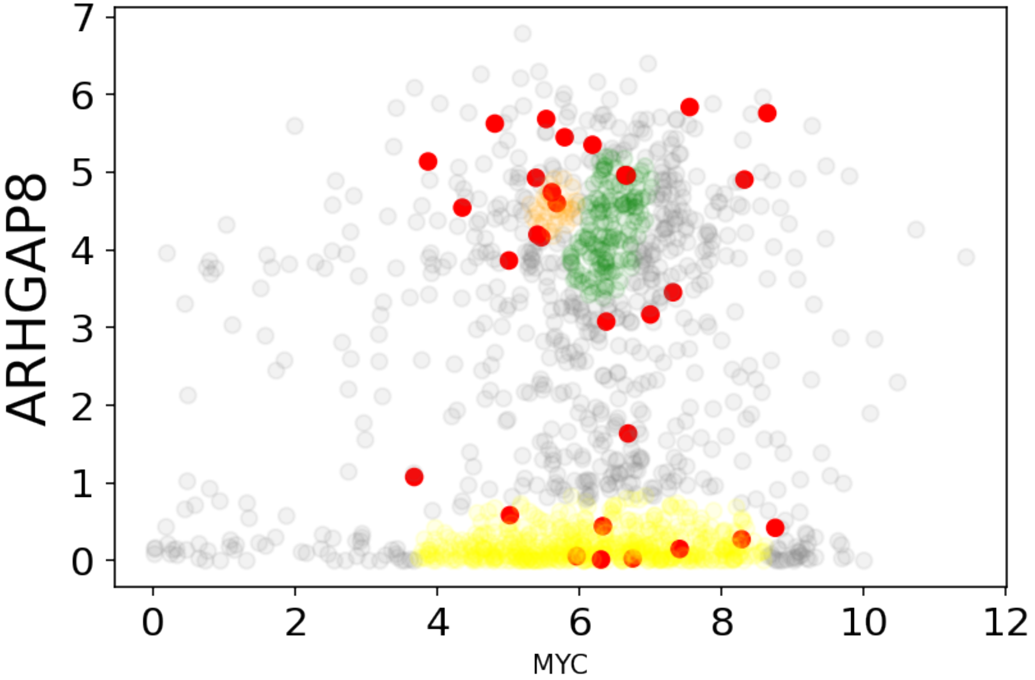

ARHGAP8
MYC
0
1
2
3
4
5
6
7
0
2
4
6
8
10
12

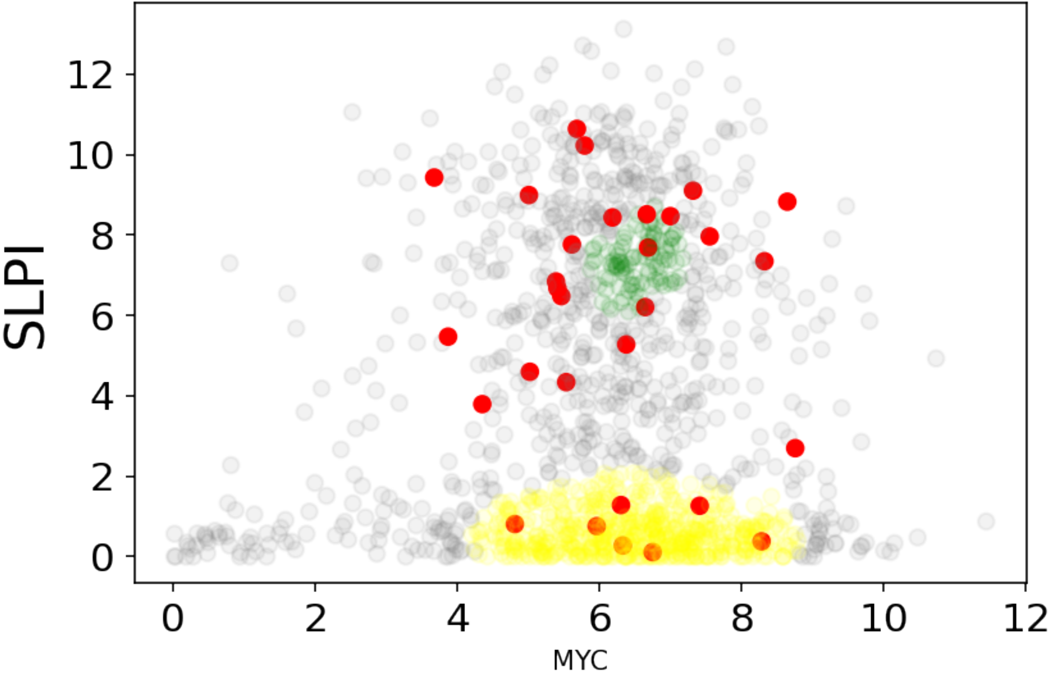
SLPI
0
2
4
6
8
10
12
MYC
0
2
4
6
8
10
12

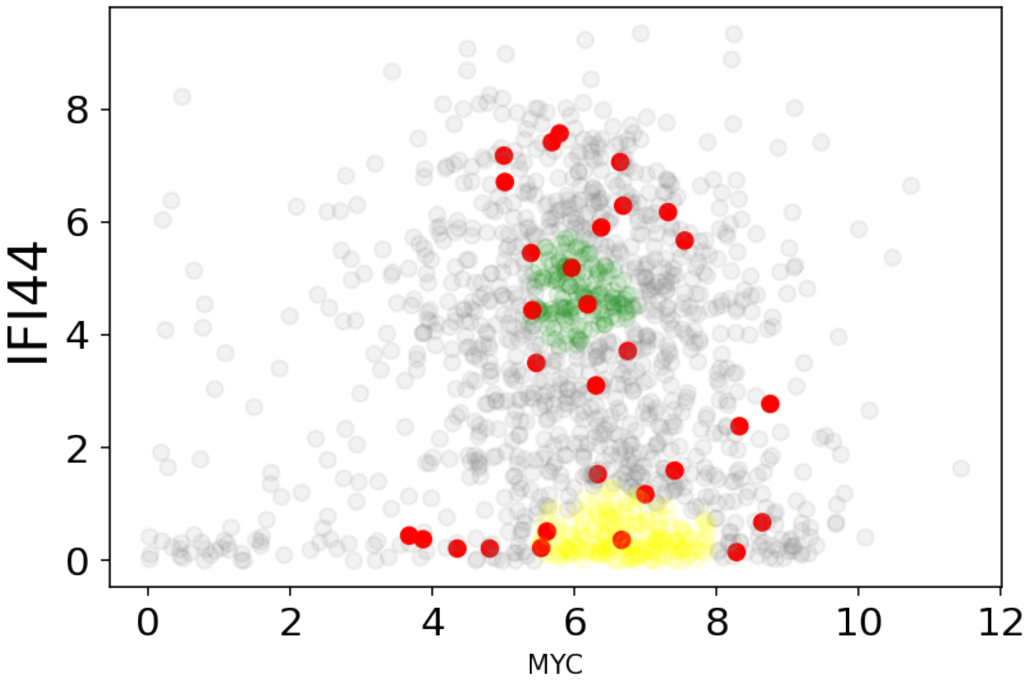

IFI44
MYC
0
2
4
6
8
0
2
4
6
8
10
12

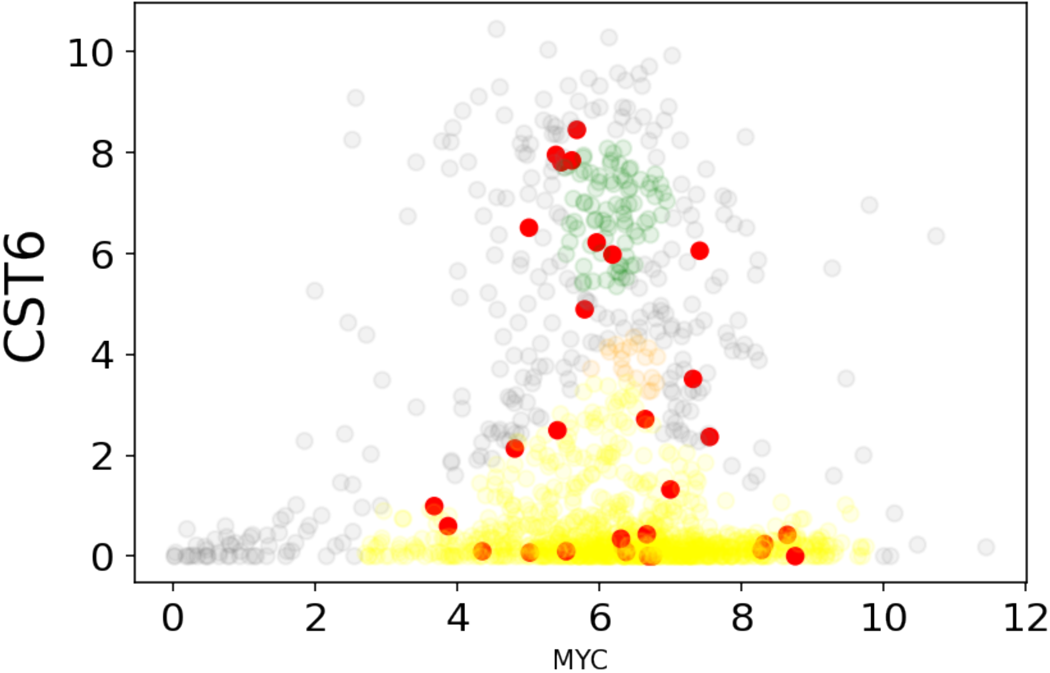

CST6
10
8
6
4
2
0
0
2
4
6
8
10
12
MYC

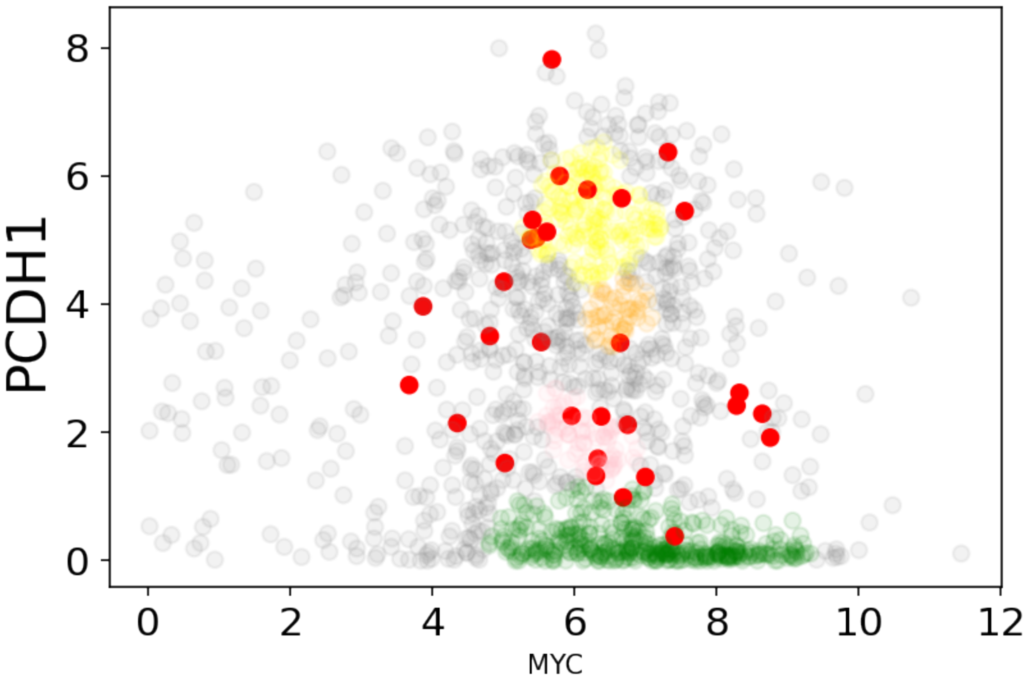
PCDH1
MYC
0
2
4
6
8
10
12

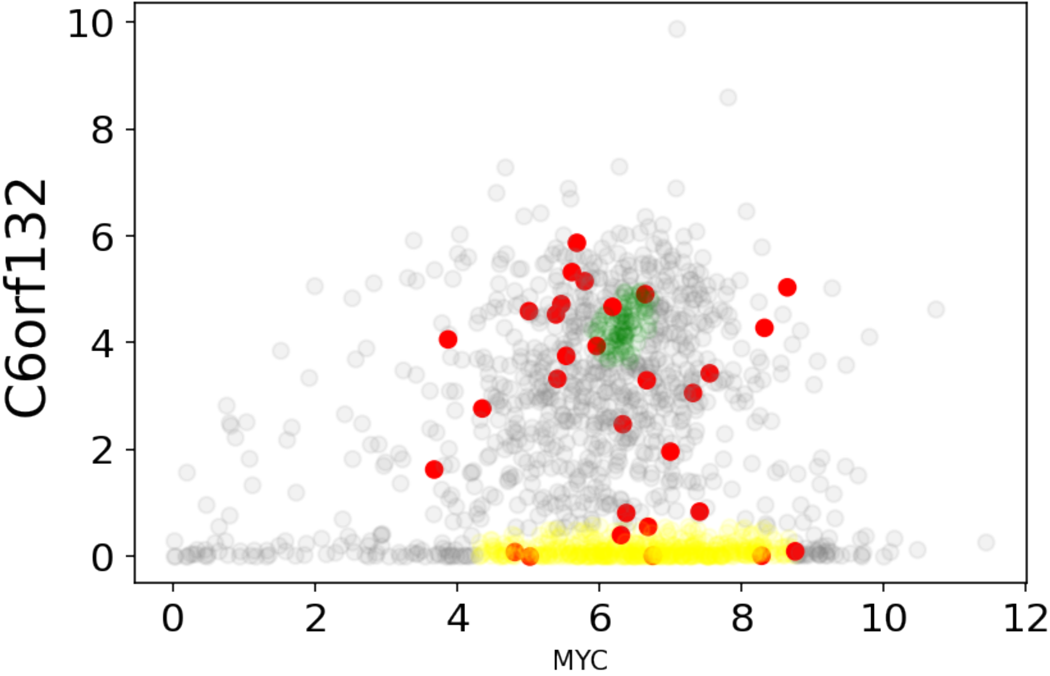

C6orf132
MYC
0
2
4
6
8
10
12

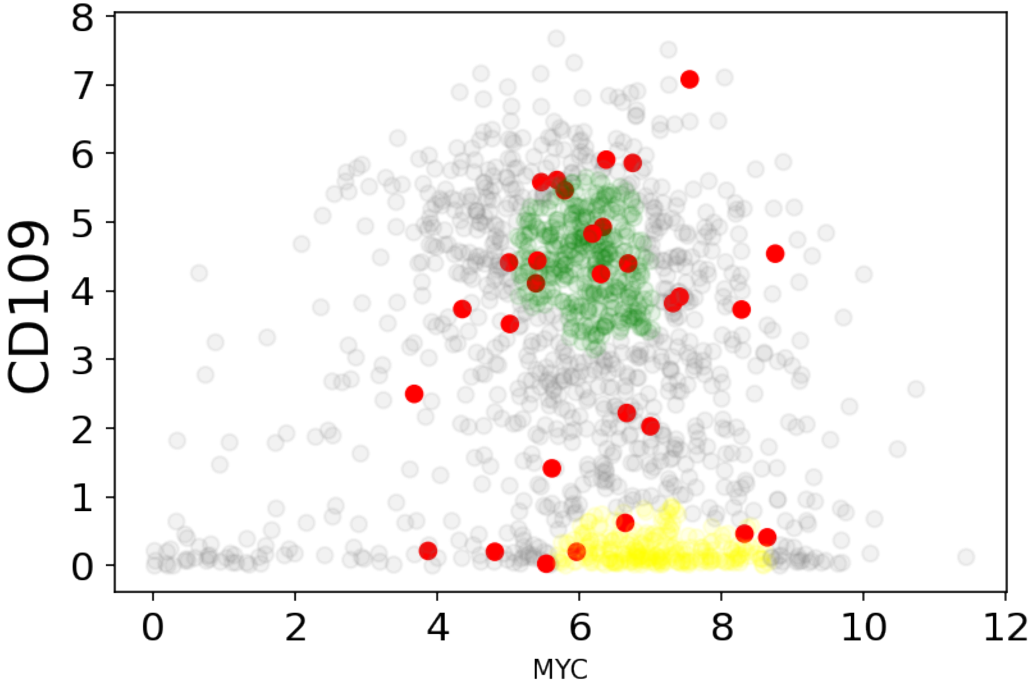
CD109
MYC
0
1
2
3
4
5
6
7
8
0
2
4
6
8
10
12

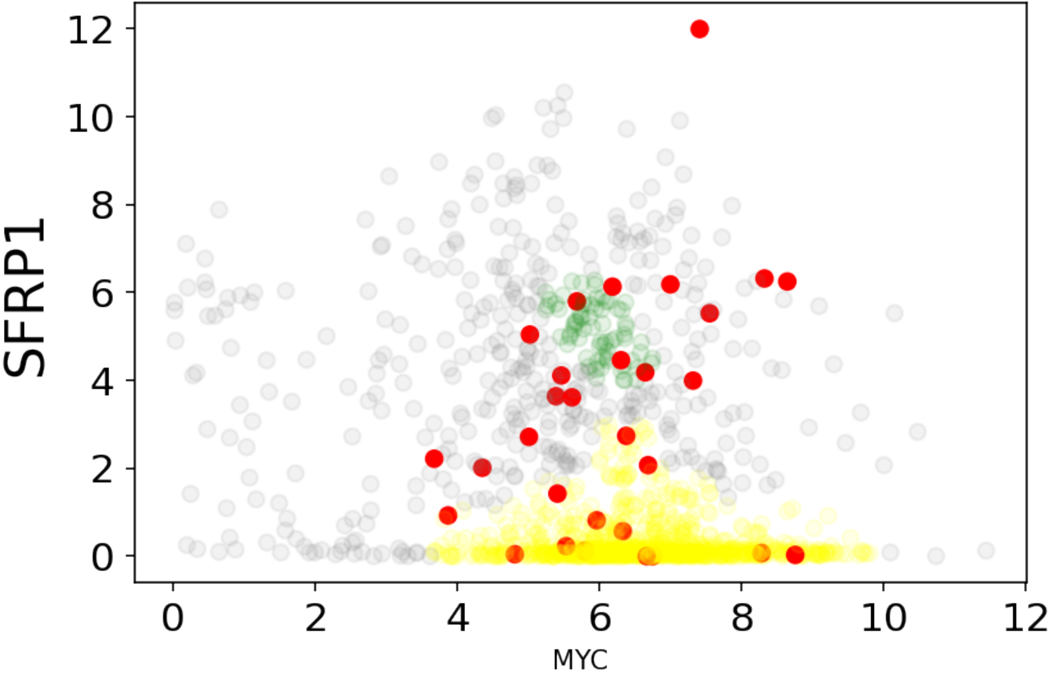

SFRP1
MYC
0
2
4
6
8
10
12

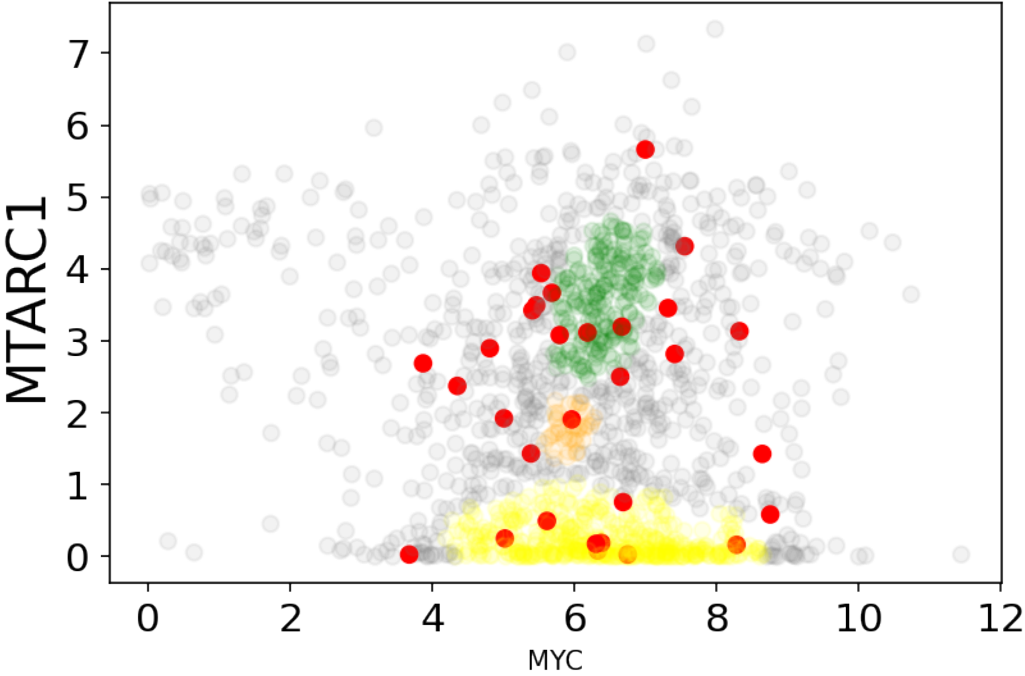
MTARC1
MYC
0
1
2
3
4
5
6
7
0
2
4
6
8
10
12

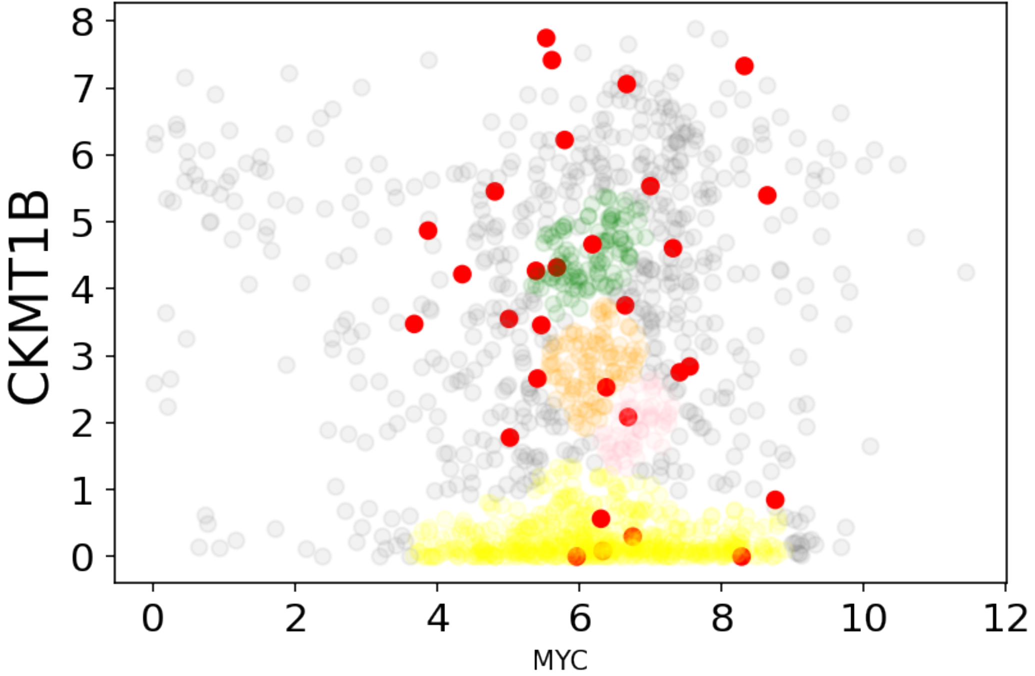
CKMT1B
MYC
0
1
2
3
4
5
6
7
8
0
2
4
6
8
10
12

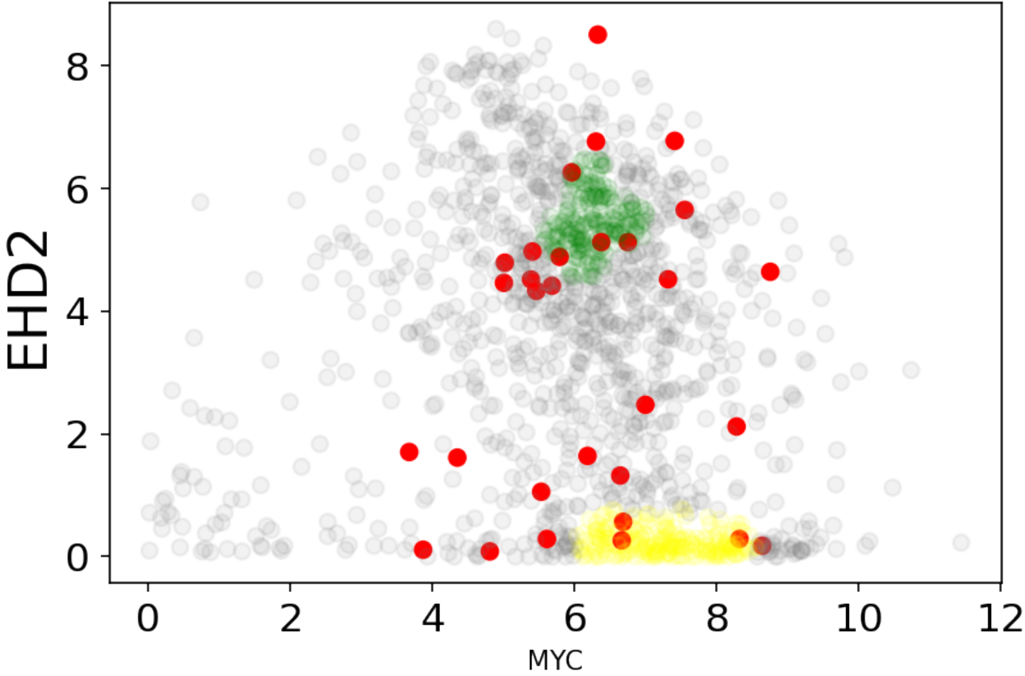

EHD2
MYC
0
2
4
6
8
10
12

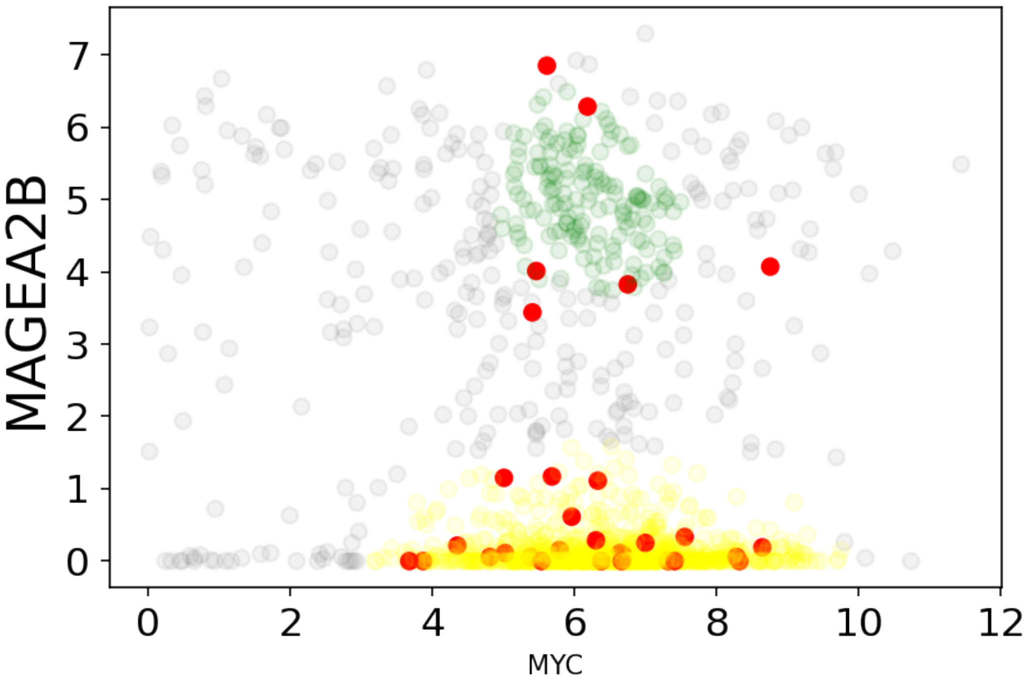

MAGEA2B
MYC
0
1
2
3
4
5
6
7
0
2
4
6
8
10
12

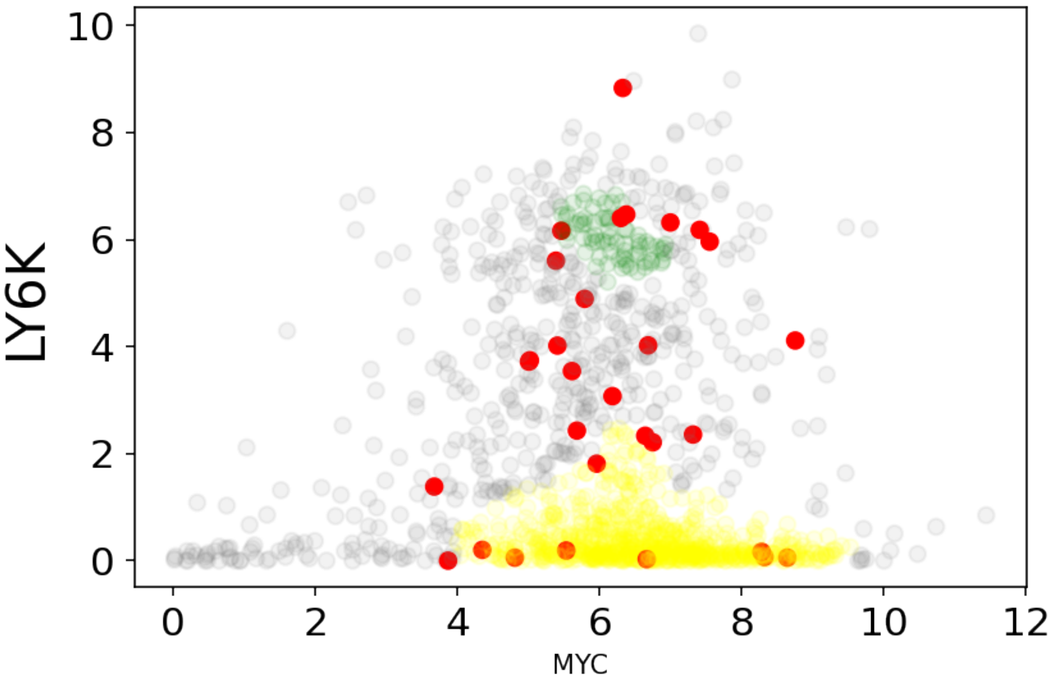

LY6K
MYC
0
2
4
6
8
10
12

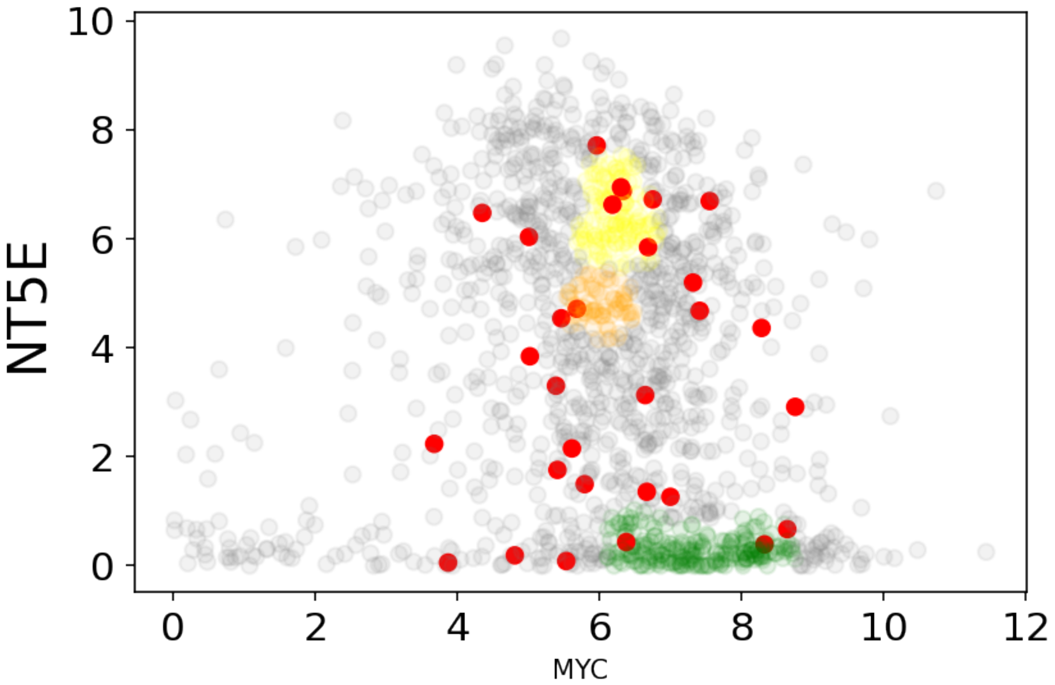
NT5E
MYC
0
2
4
6
8
10
12

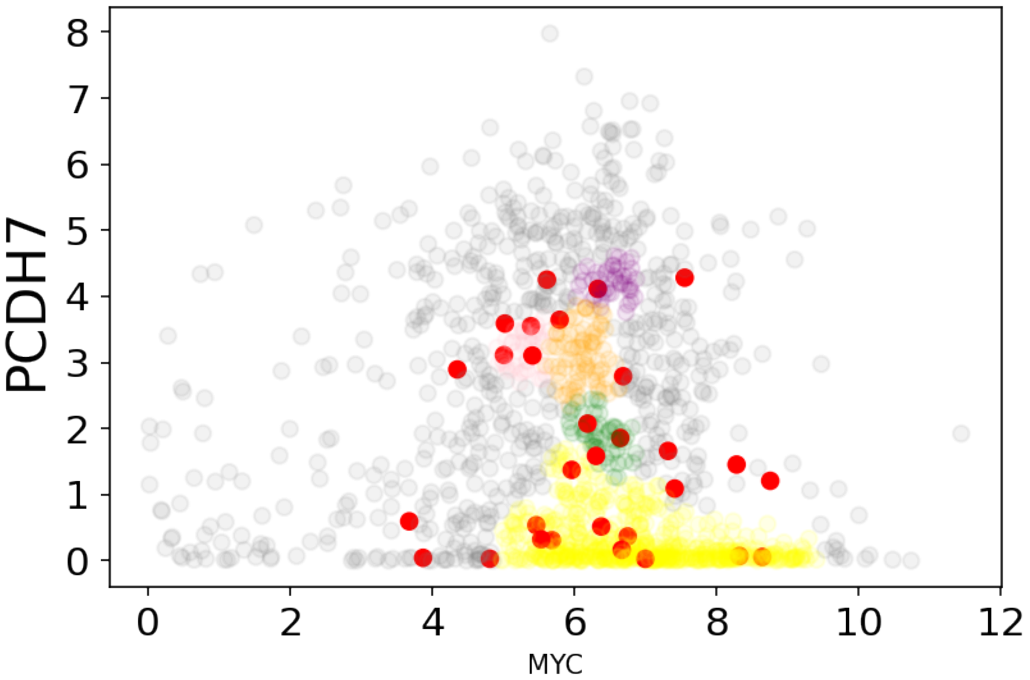

PCDH7
MYC
0
1
2
3
4
5
6
7
8
0
2
4
6
8
10
12

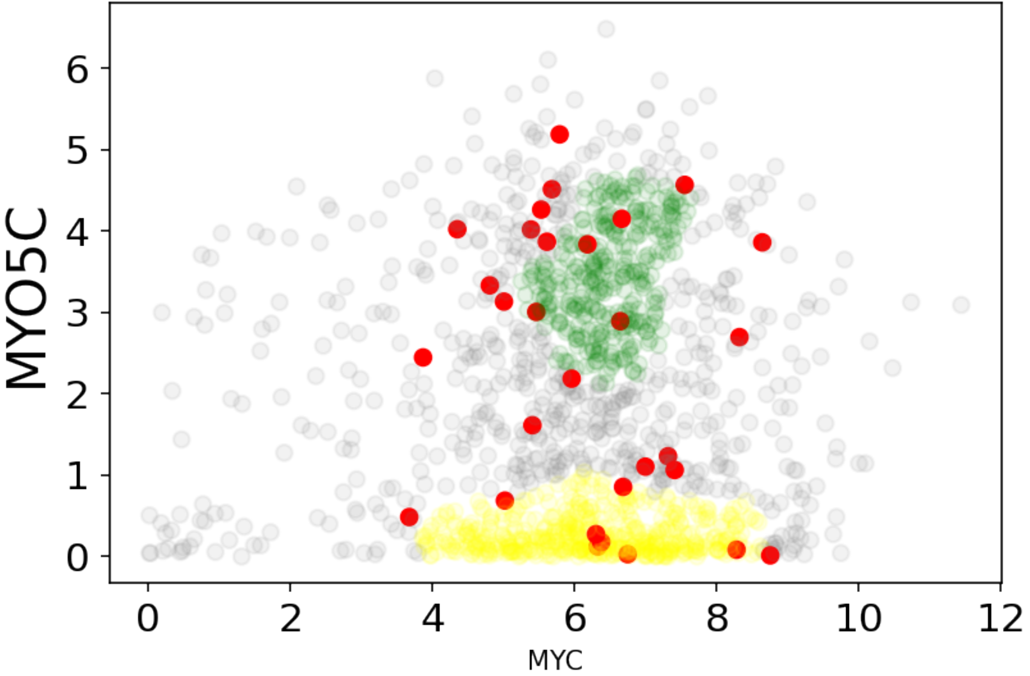

MYO5C
6
5
4
3
2
1
0
0
2
4
6
8
10
12
MYC

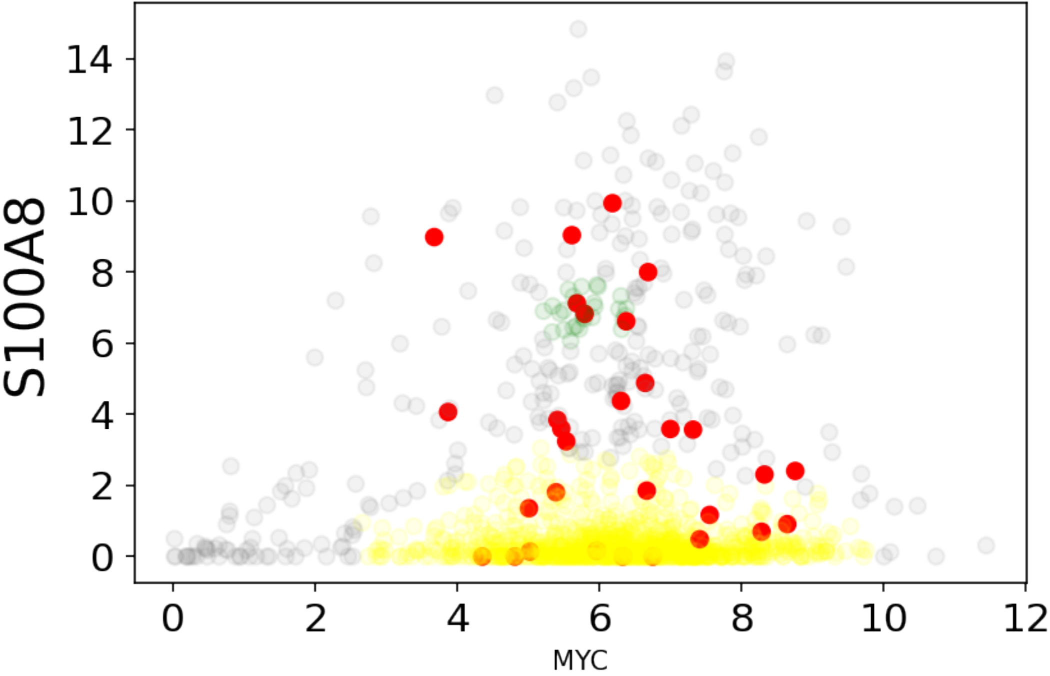

S100A8
MYC
0
2
4
6
8
10
12
14

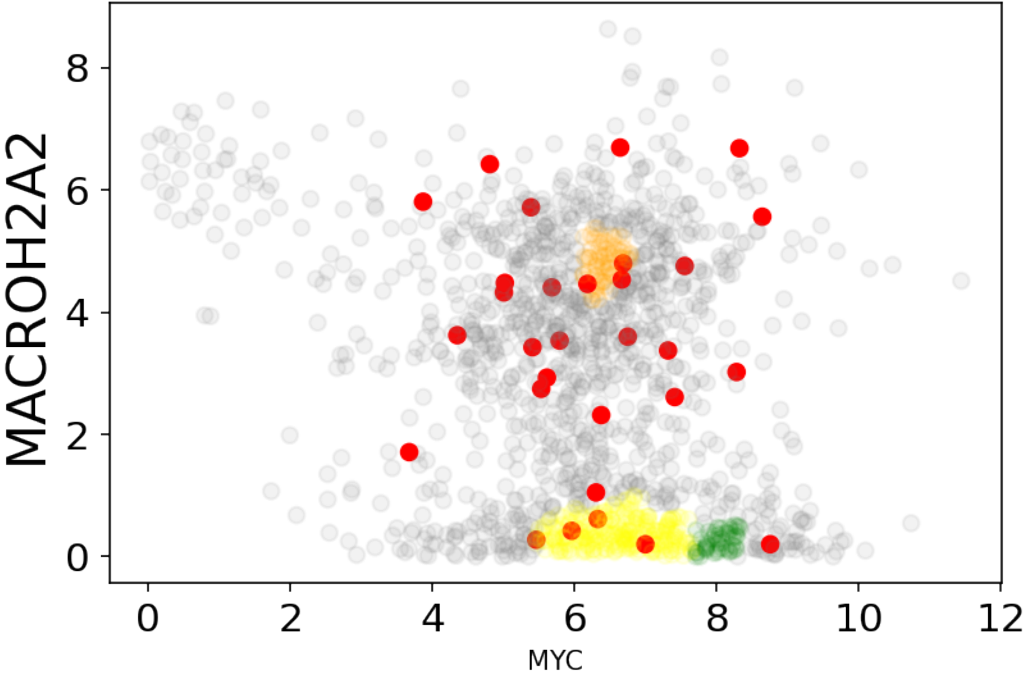

MACROH2A2
MYC
0
2
4
6
8
10
12

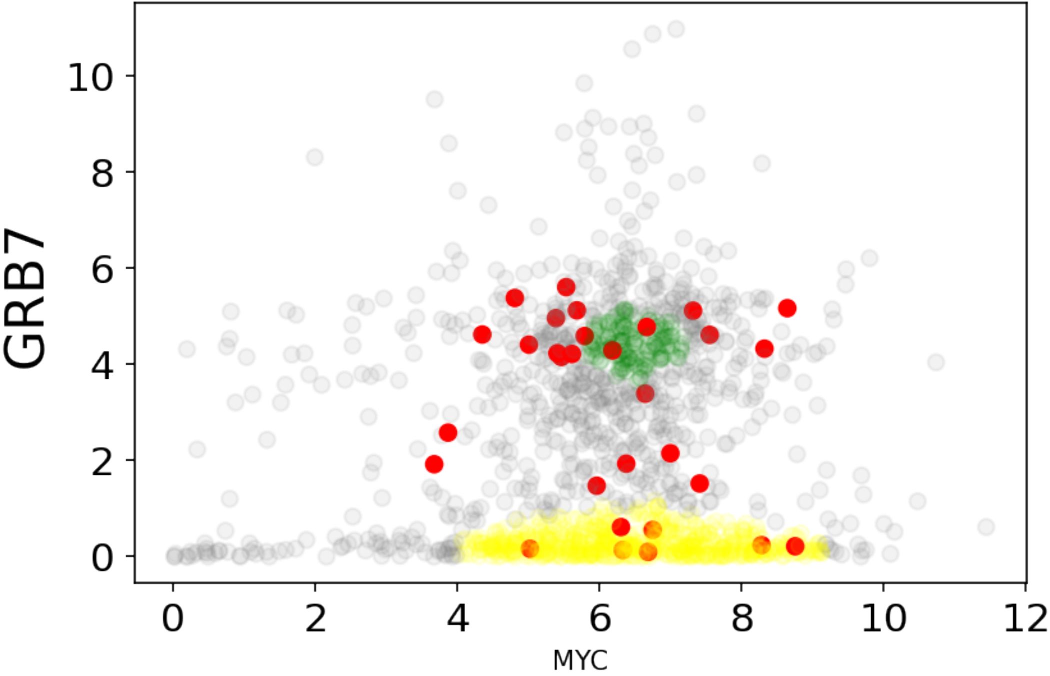
GRB7
10
8
6
4
2
0
0
2
4
6
8
10
12
MYC

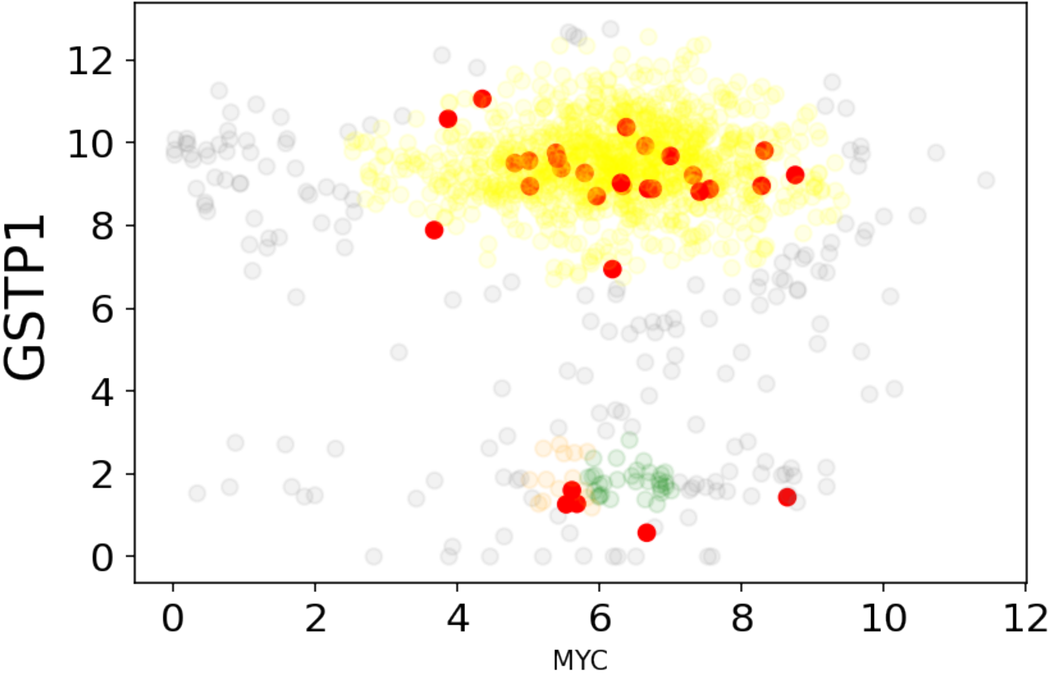

GSTP1
MYC
0
2
4
6
8
10
12

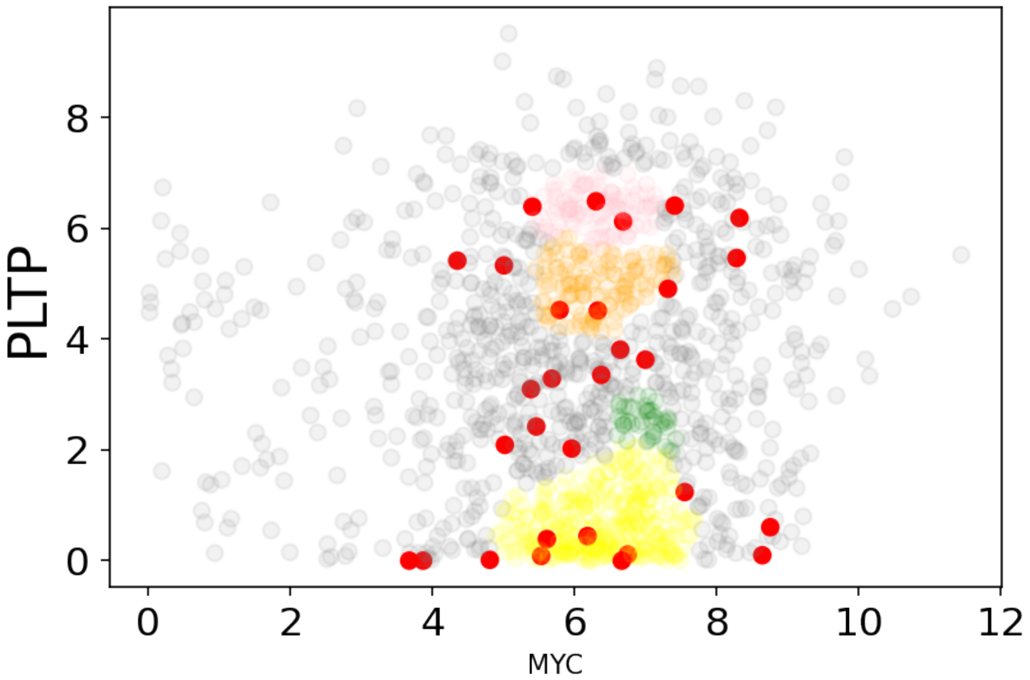

PLTP
0
2
4
6
8
0
2
4
6
8
10
12
MYC

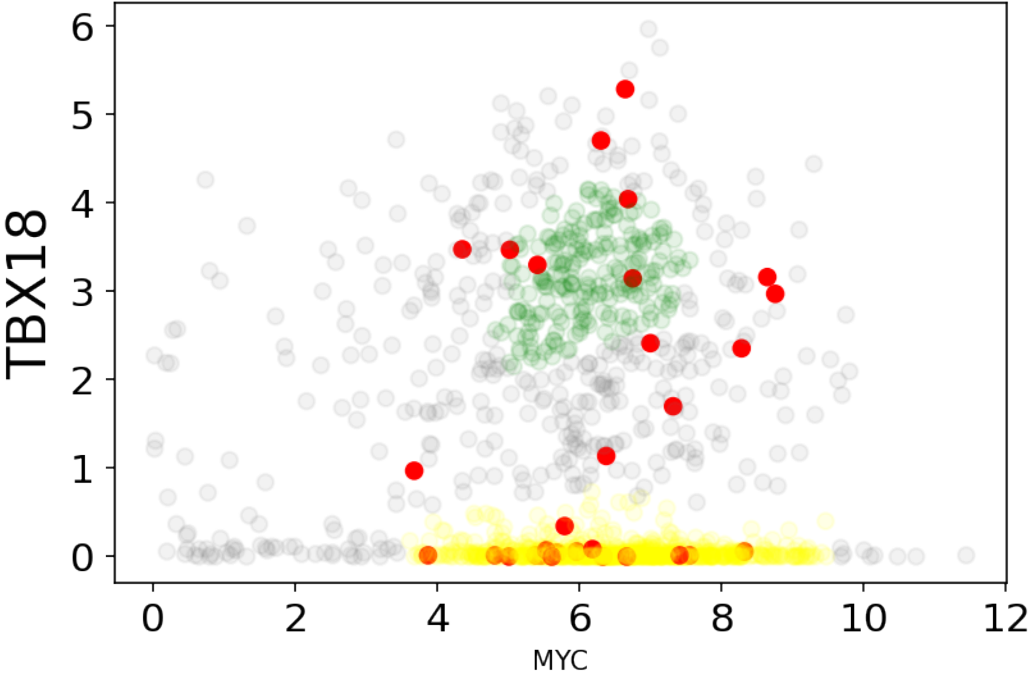
TBX18
MYC
0
1
2
3
4
5
6
0
2
4
6
8
10
12

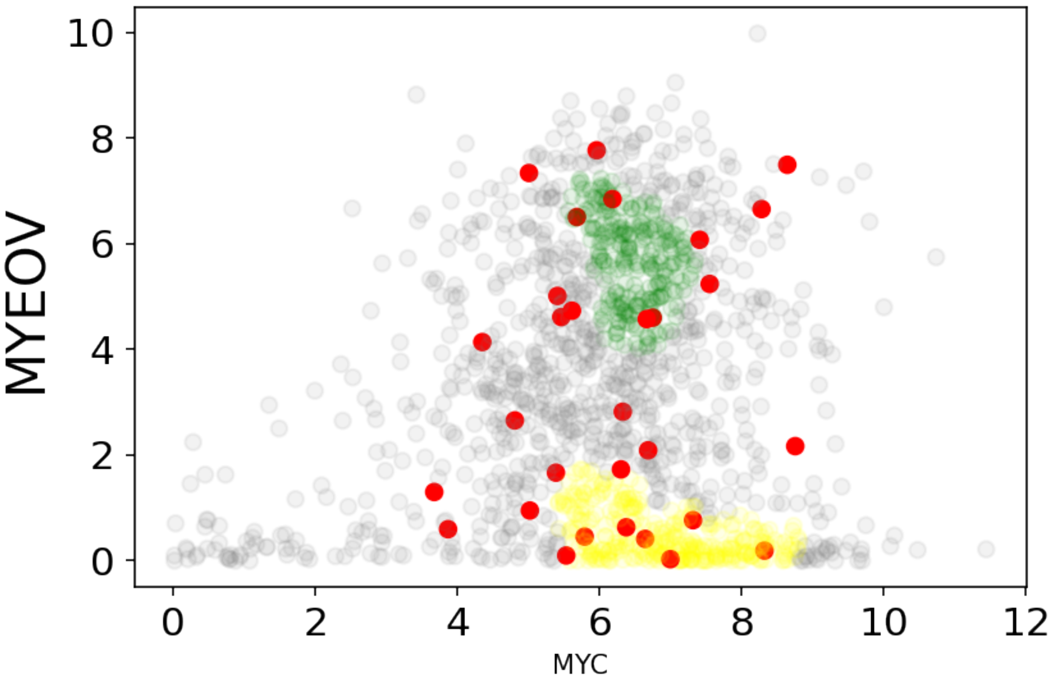
MYEOV
MYC
0
2
4
6
8
10
12

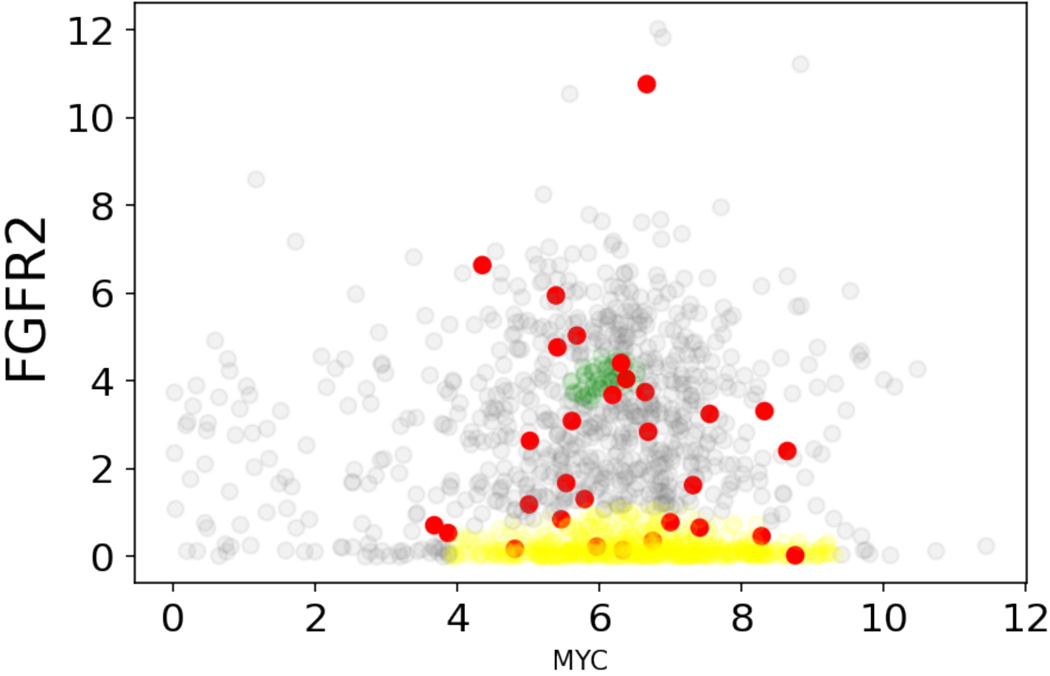

FGFR2
12
10
8
6
4
2
0
0
2
4
6
8
10
12
MYC

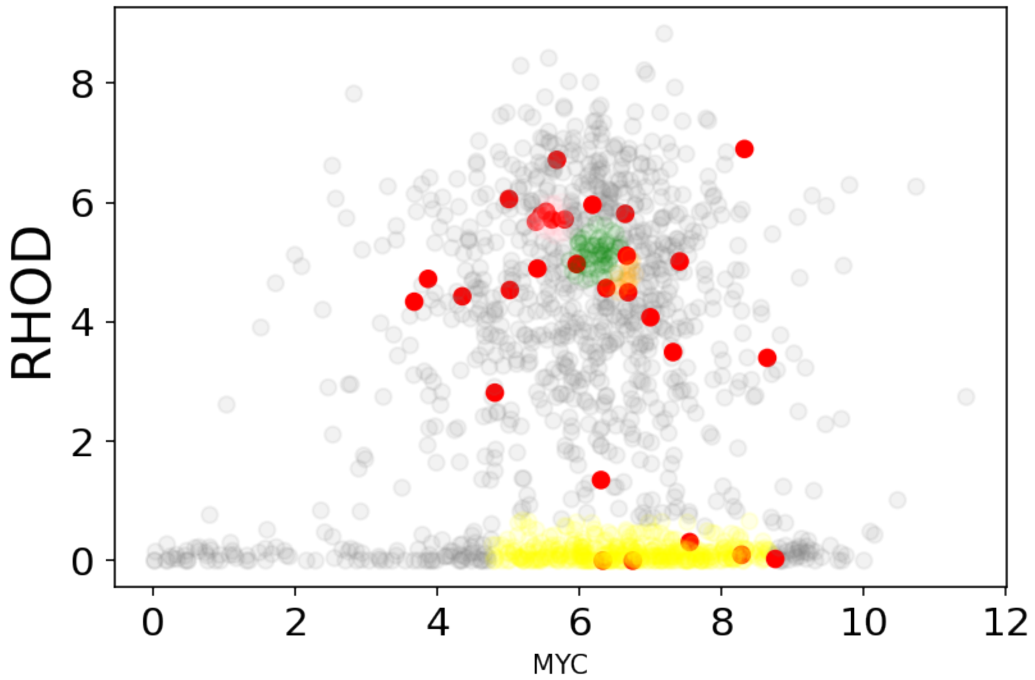

RHOD
MYC
0
2
4
6
8
10
12

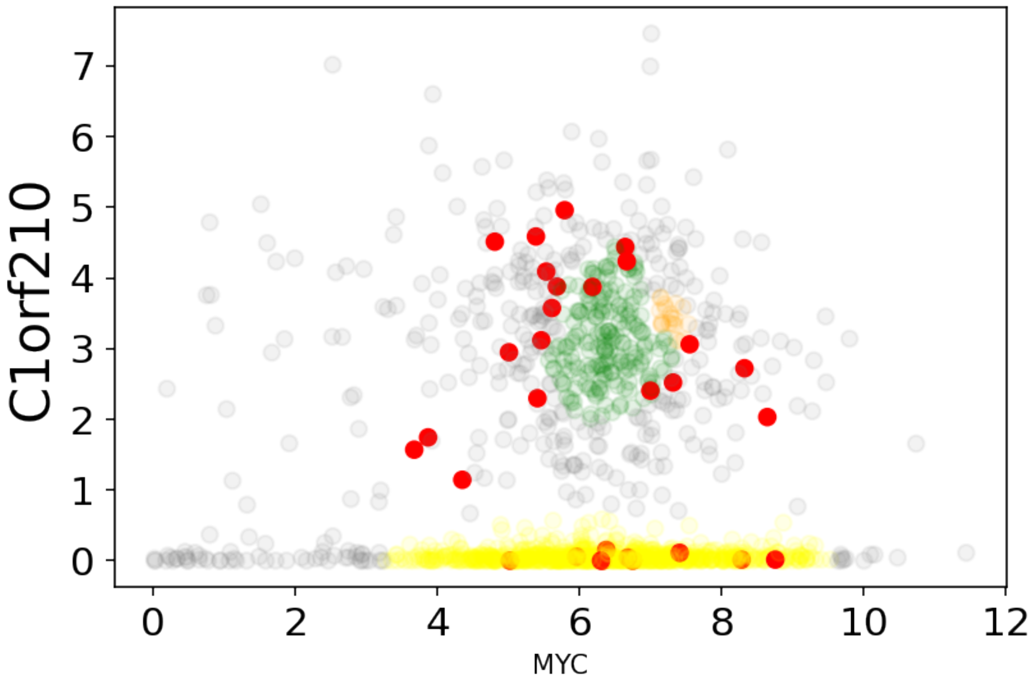

C1orf210
MYC
0
1
2
3
4
5
6
7
0
2
4
6
8
10
12

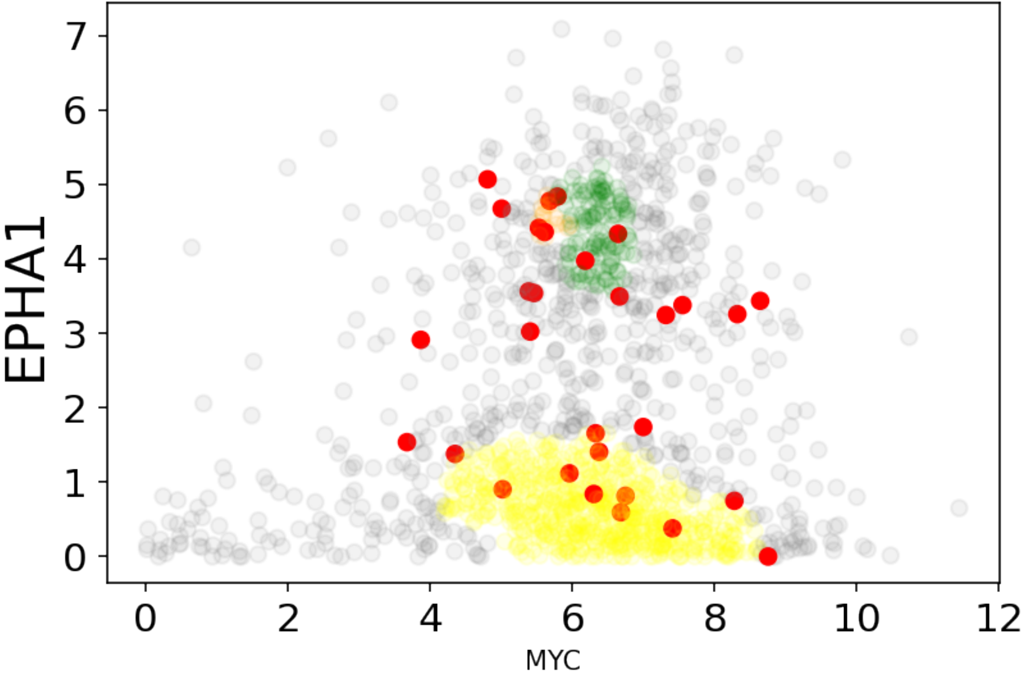

EPHA1
MYC
0
1
2
3
4
5
6
7
0
2
4
6
8
10
12

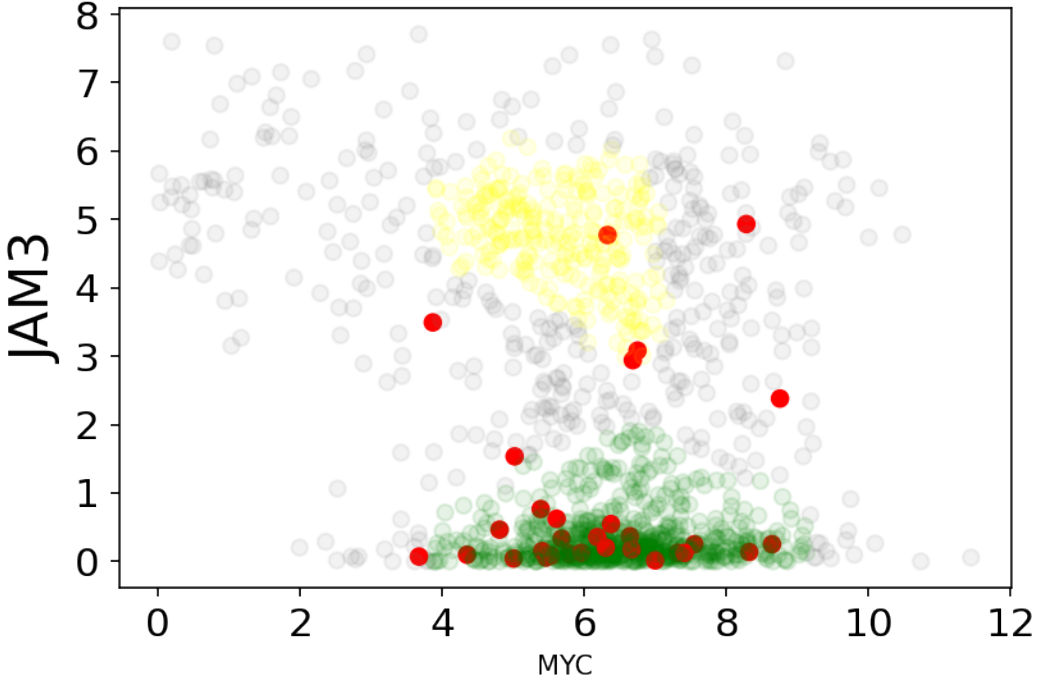

JAM3
MYC
0
1
2
3
4
5
6
7
8
0
2
4
6
8
10
12

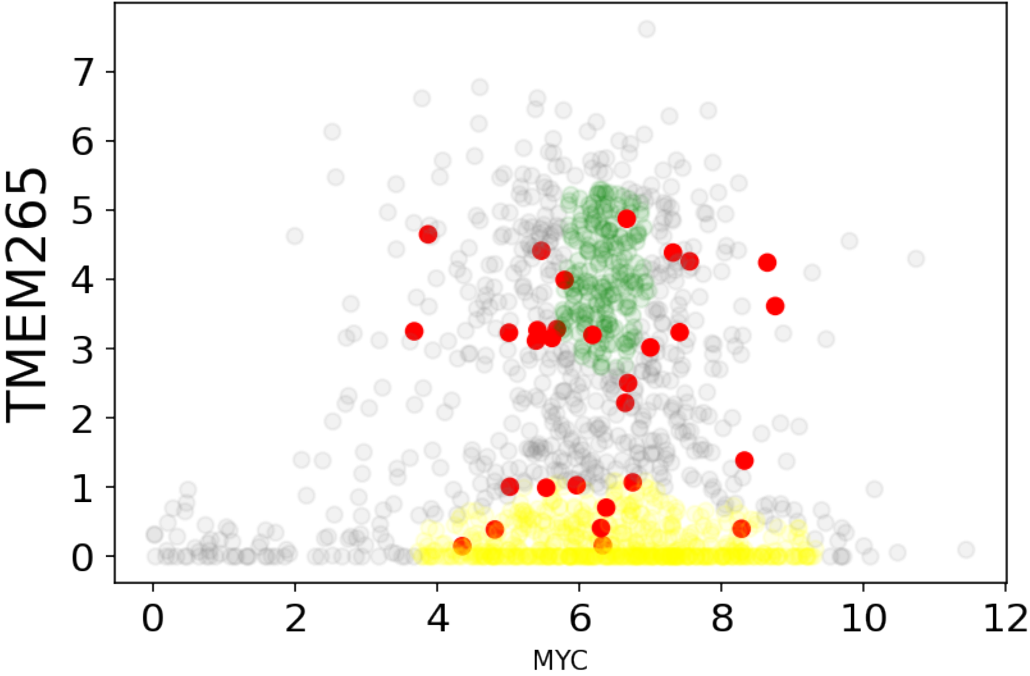
TMEM265
MYC
0
1
2
3
4
5
6
7
0
2
4
6
8
10
12

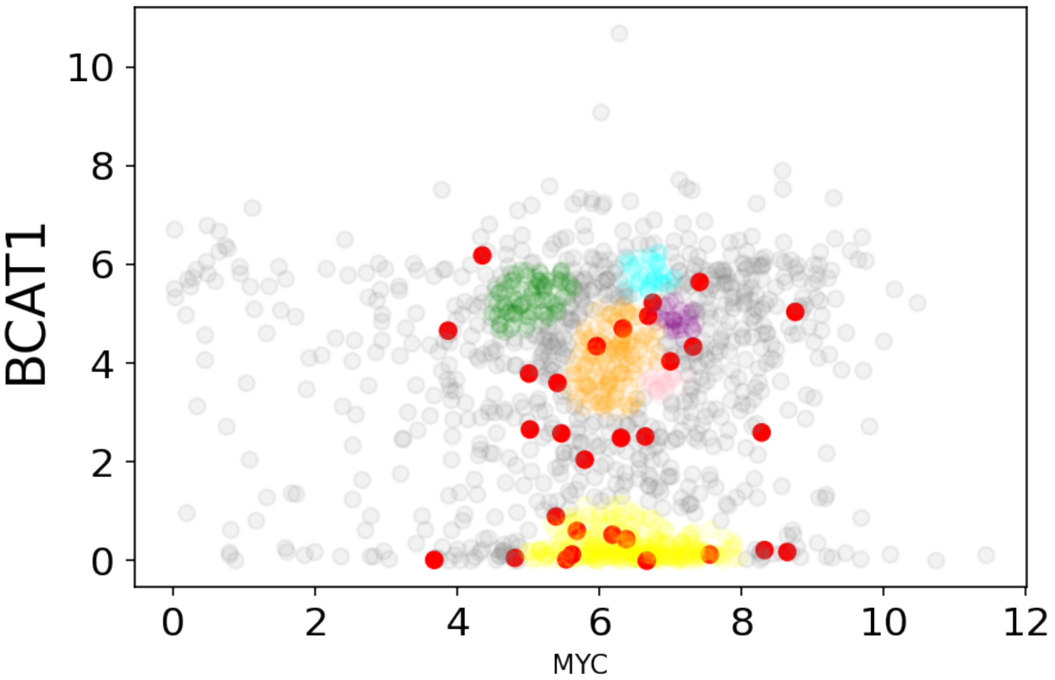
BCAT1
MYC
0
2
4
6
8
10
12

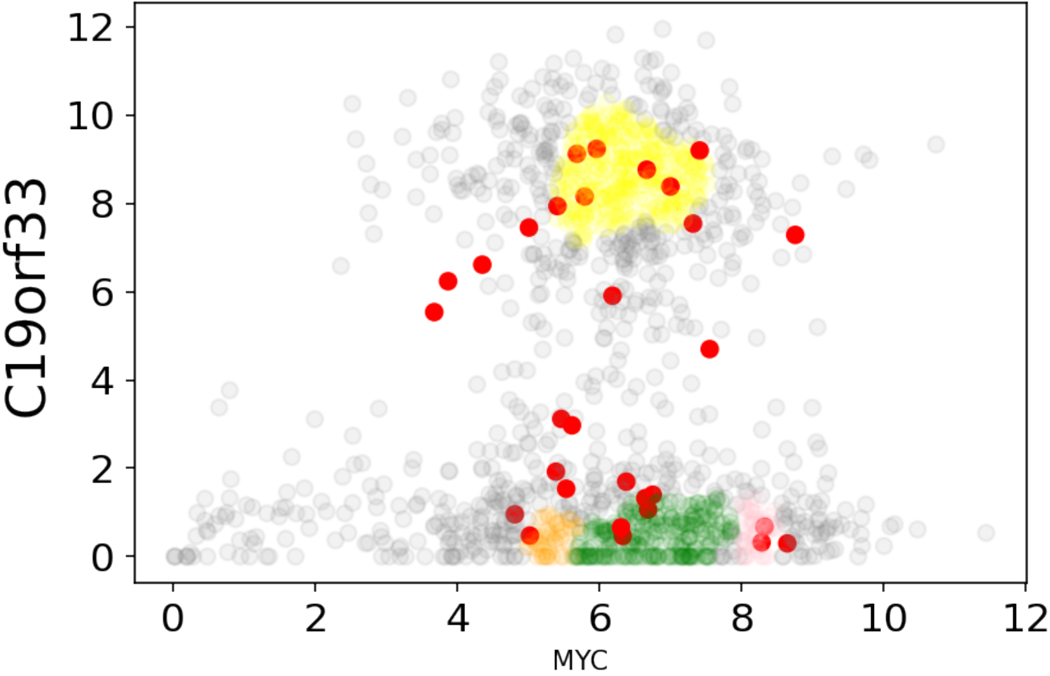

C19orf33
MYC
0
2
4
6
8
10
12

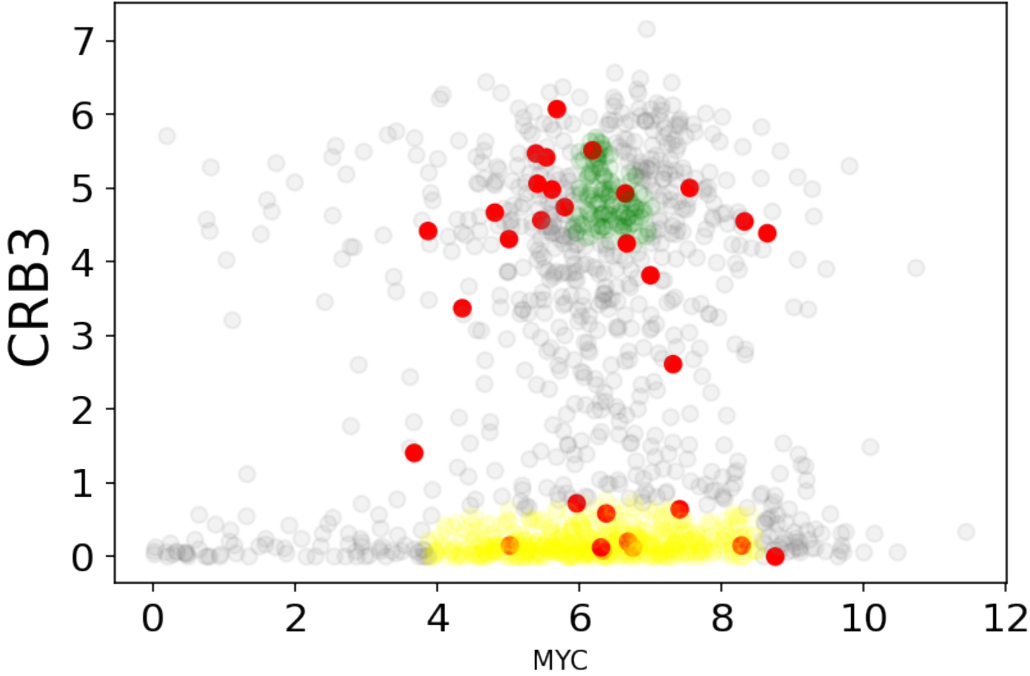

CRB3
MYC
0
1
2
3
4
5
6
7
0
2
4
6
8
10
12

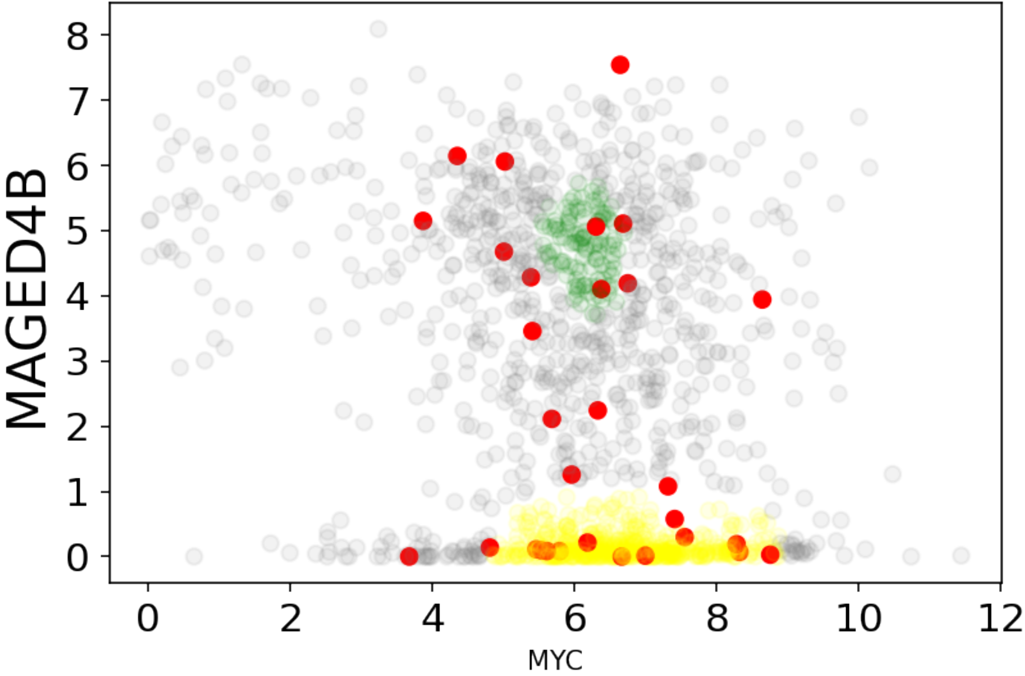
MAGED4B
MYC
0
1
2
3
4
5
6
7
8
0
2
4
6
8
10
12

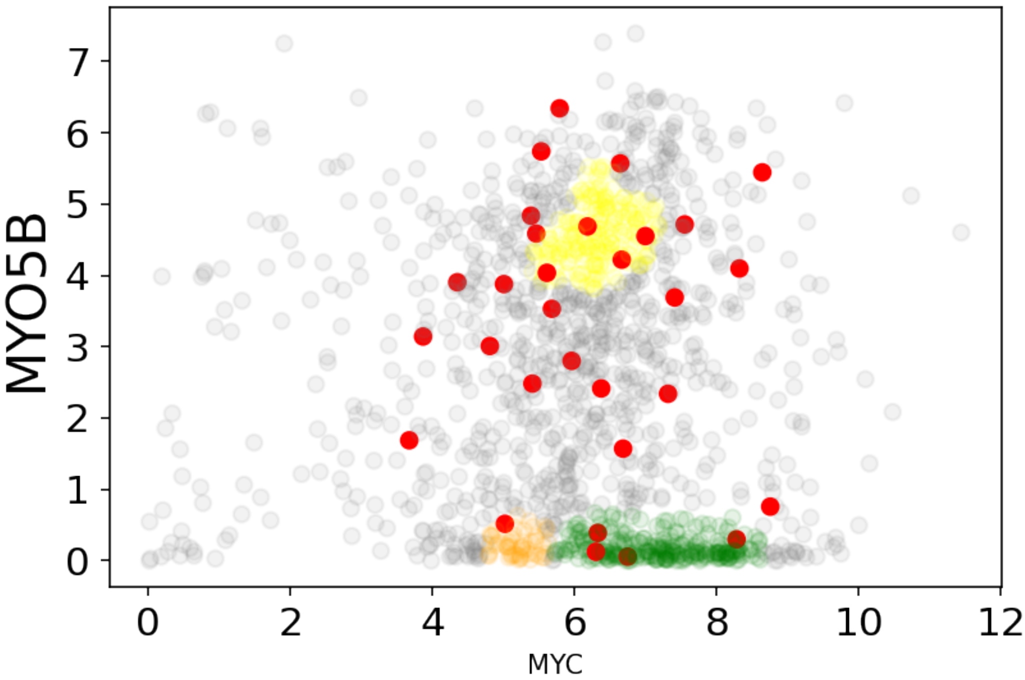

MYO5B
7
6
5
4
3
2
1
0
0
2
4
6
8
10
12
MYC

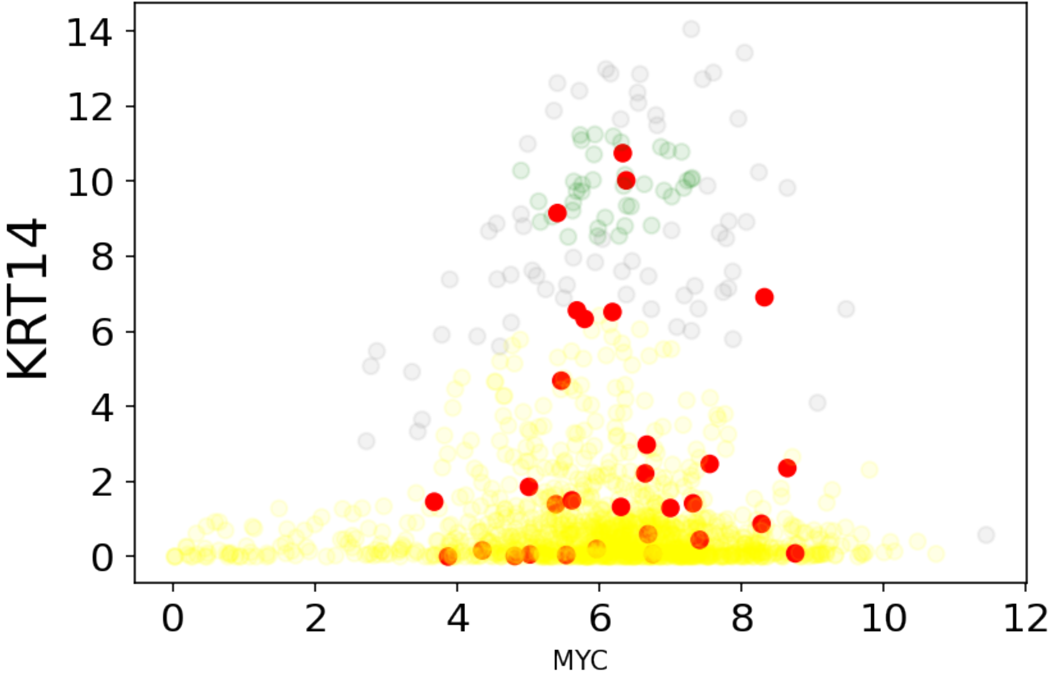

KRT14
14
12
10
8
6
4
2
0
0
2
4
6
8
10
12
MYC

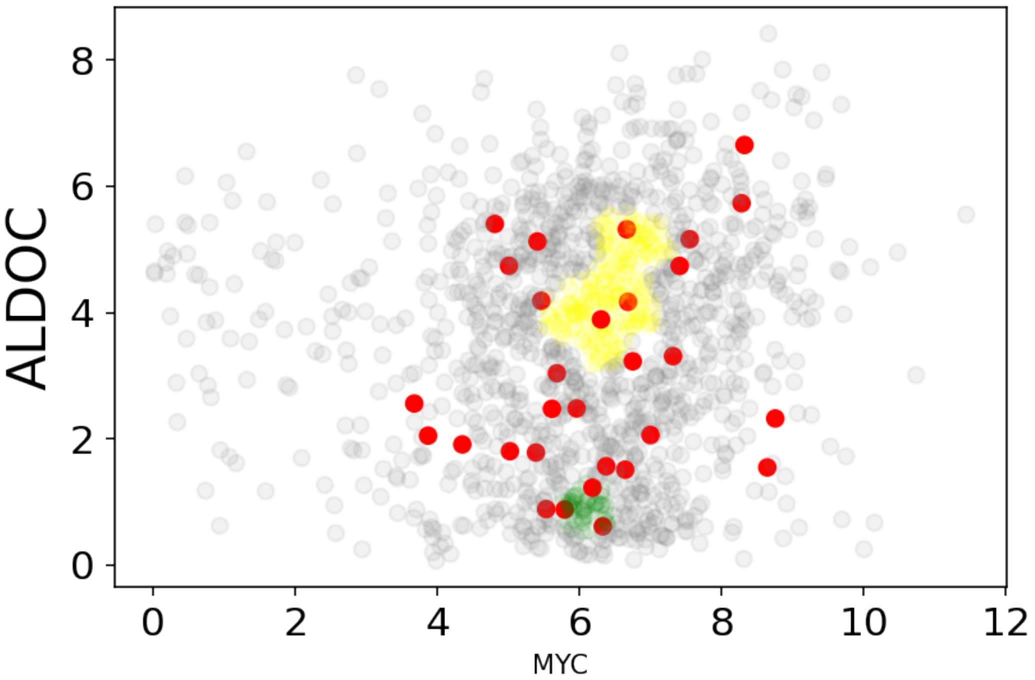
ALDOC
MYC
0
2
4
6
8
0
2
4
6
8
10
12

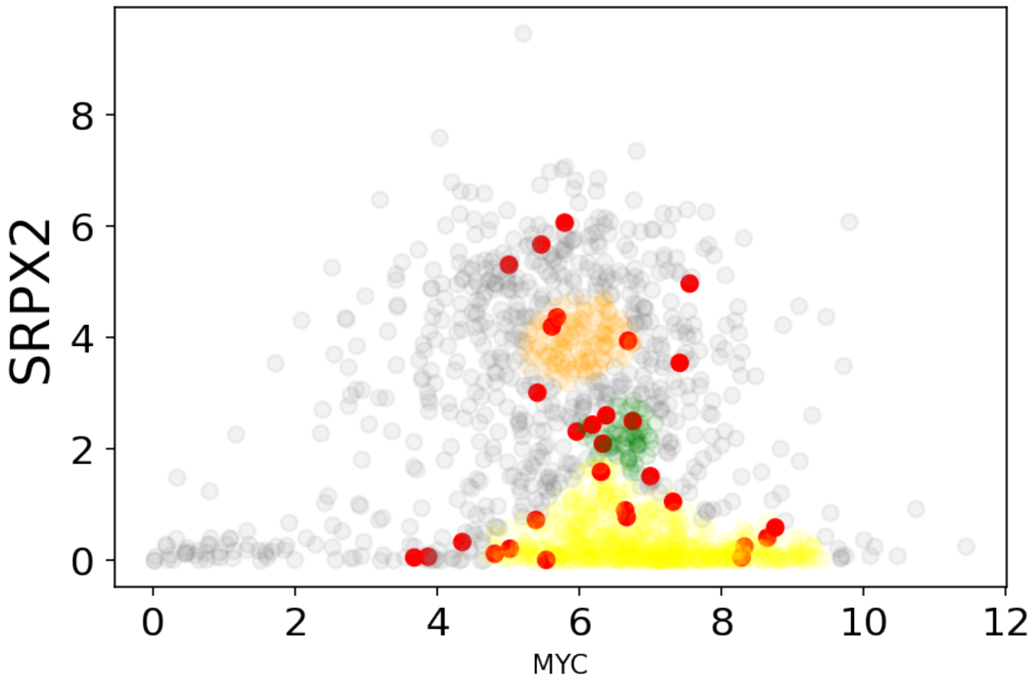

SRPX2
0
2
4
6
8
0
2
4
6
8
10
12
MYC

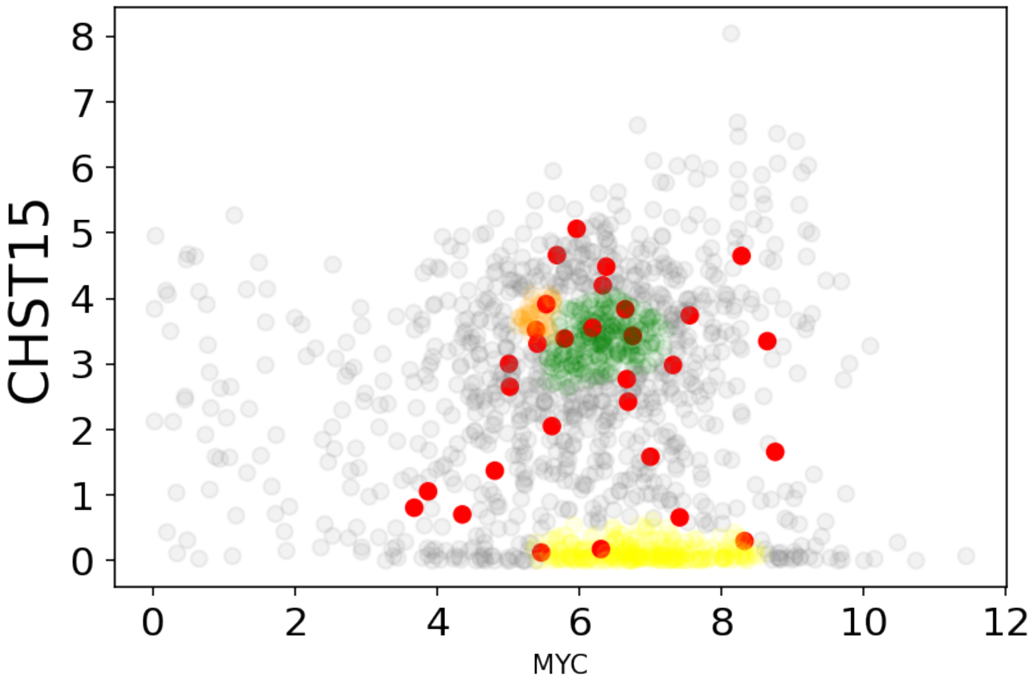

CHST15
MYC
0
1
2
3
4
5
6
7
8
0
2
4
6
8
10
12

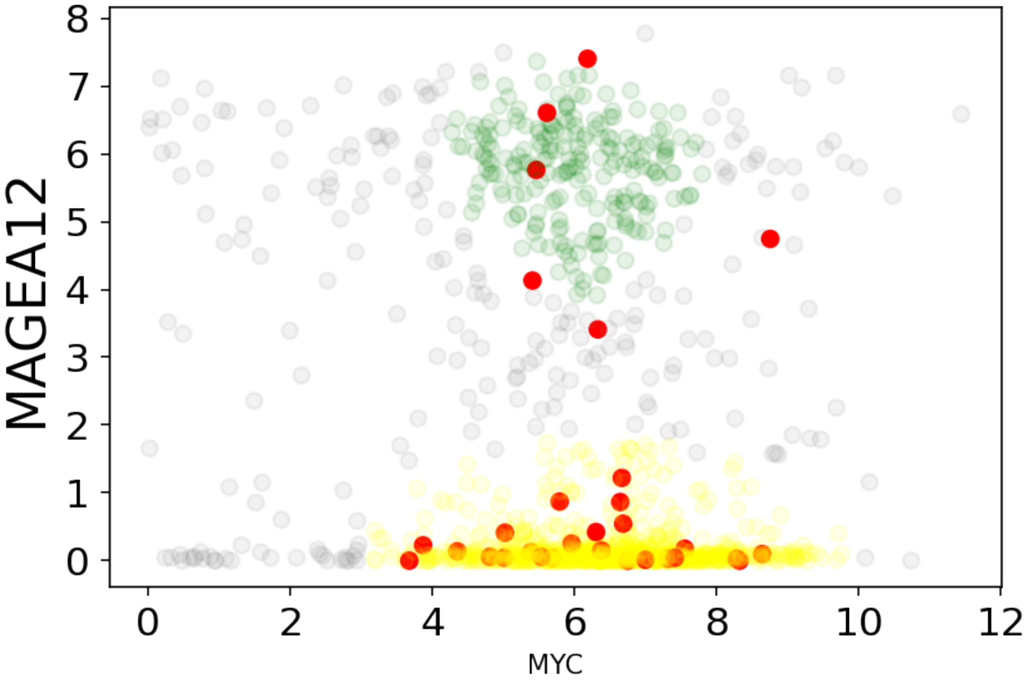

MAGEA12
MYC
0
1
2
3
4
5
6
7
8
0
2
4
6
8
10
12

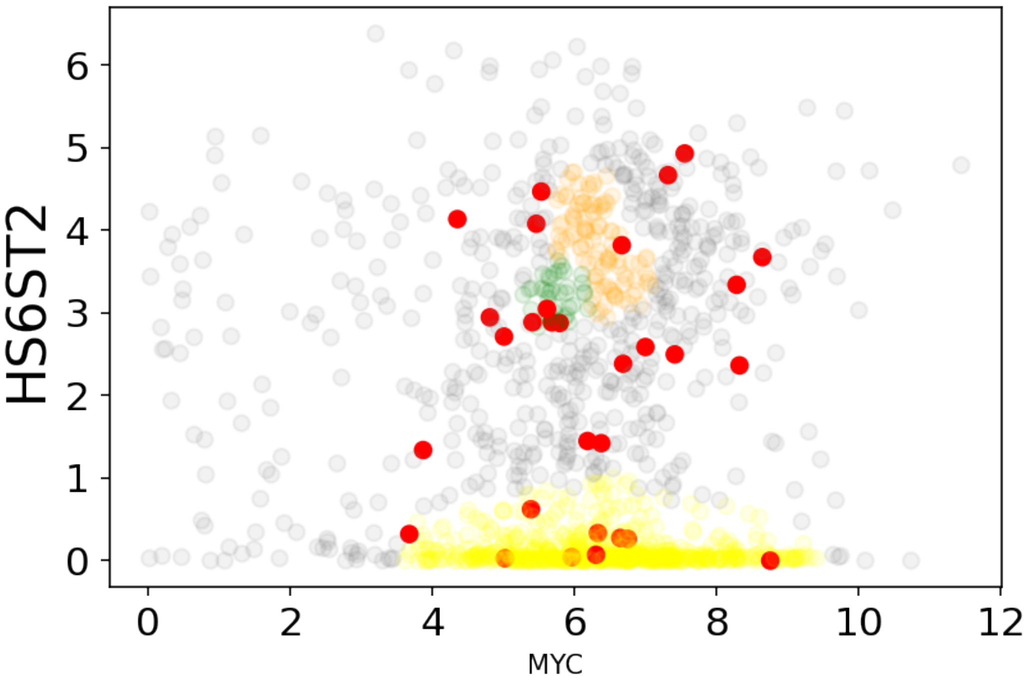
HS6ST2
6
5
4
3
2
1
0
0
2
4
6
8
10
12
MYC

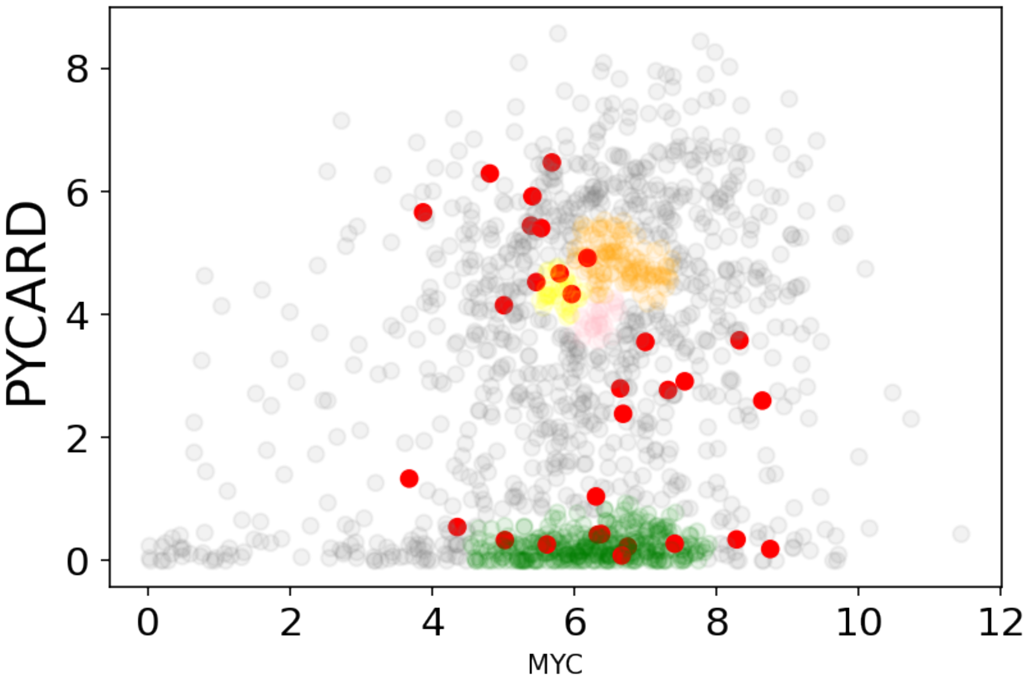

PYCARD
MYC
0
2
4
6
8
0
2
4
6
8
10
12

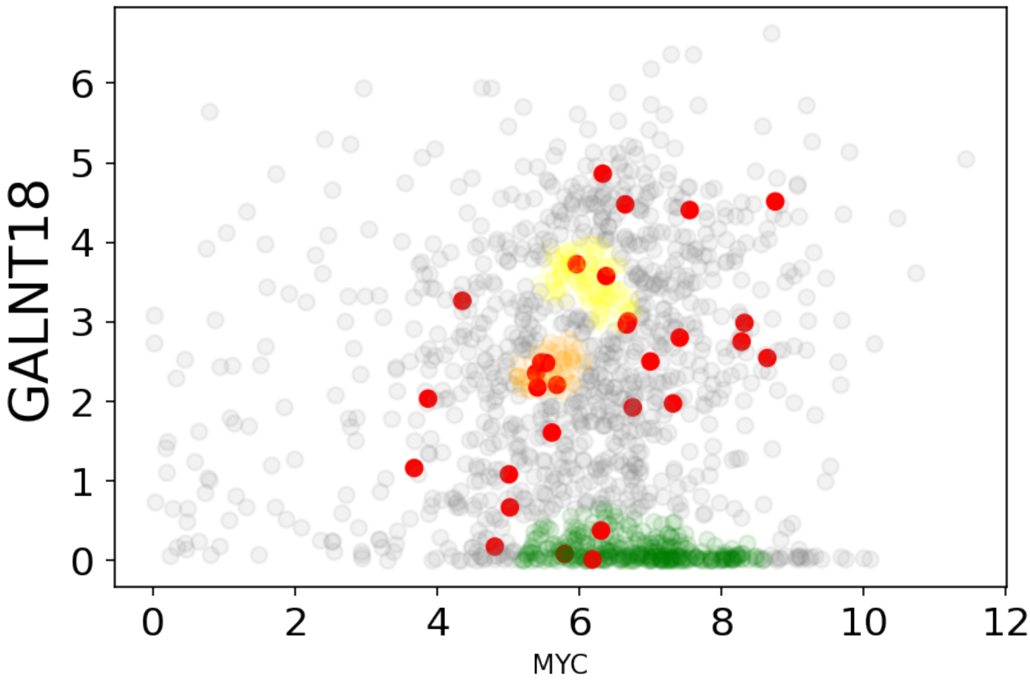

GALNT18
6
5
4
3
2
1
0
0
2
4
6
8
10
12
MYC

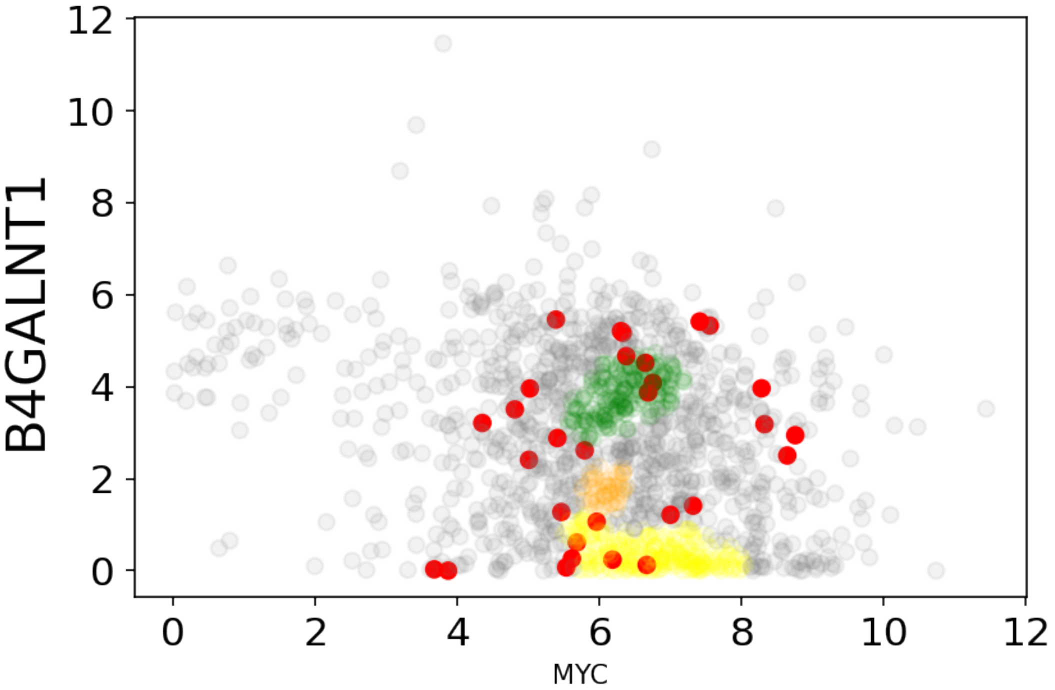

B4GALNT1
MYC
0
2
4
6
8
10
12

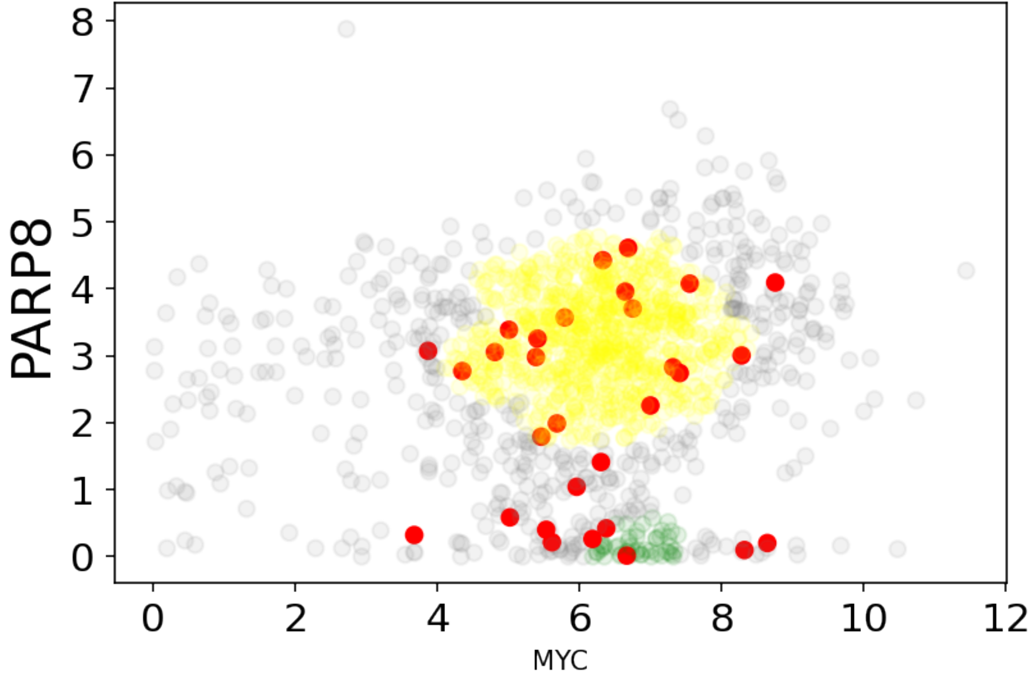
PARP8
MYC
0
1
2
3
4
5
6
7
8
0
2
4
6
8
10
12

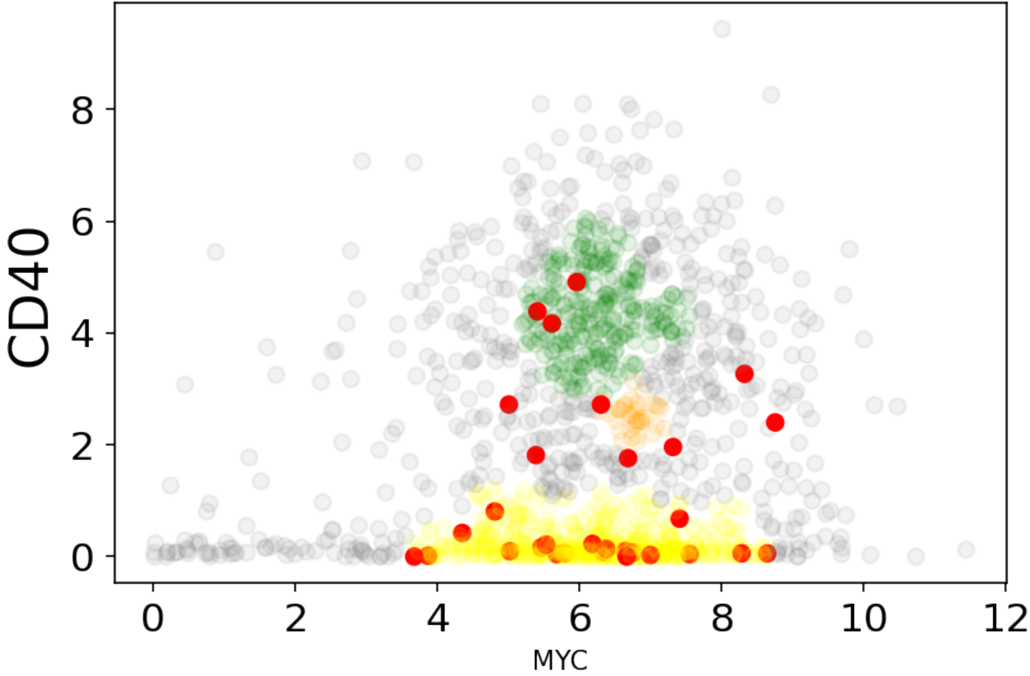
CD40
0
2
4
6
8
0
2
4
6
8
10
12
MYC

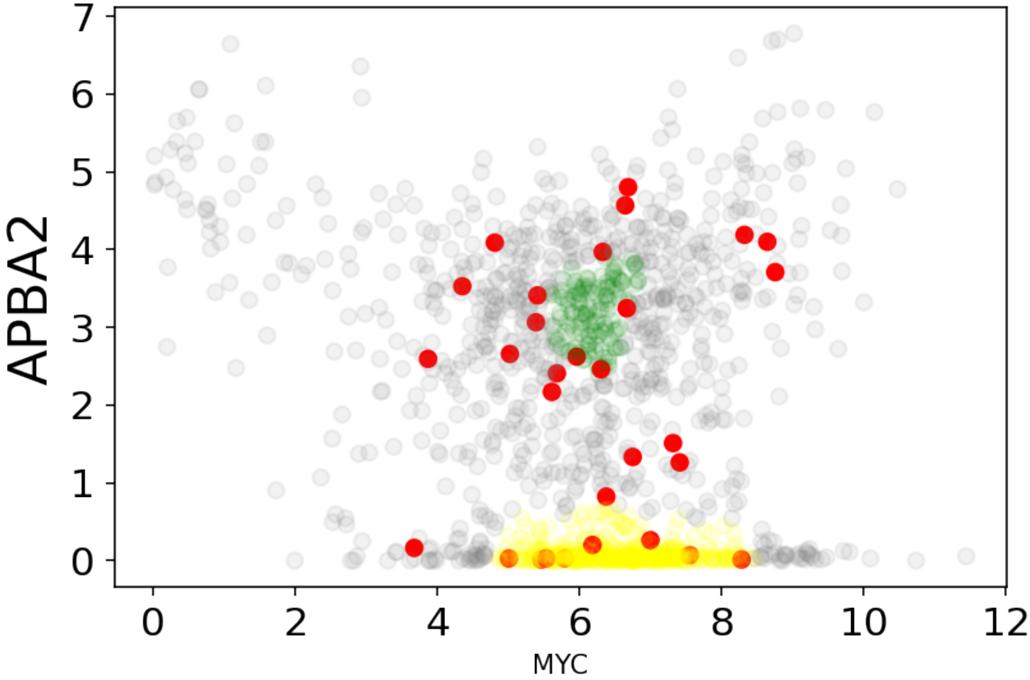
APBA2
MYC
0
1
2
3
4
5
6
7
0
2
4
6
8
10
12

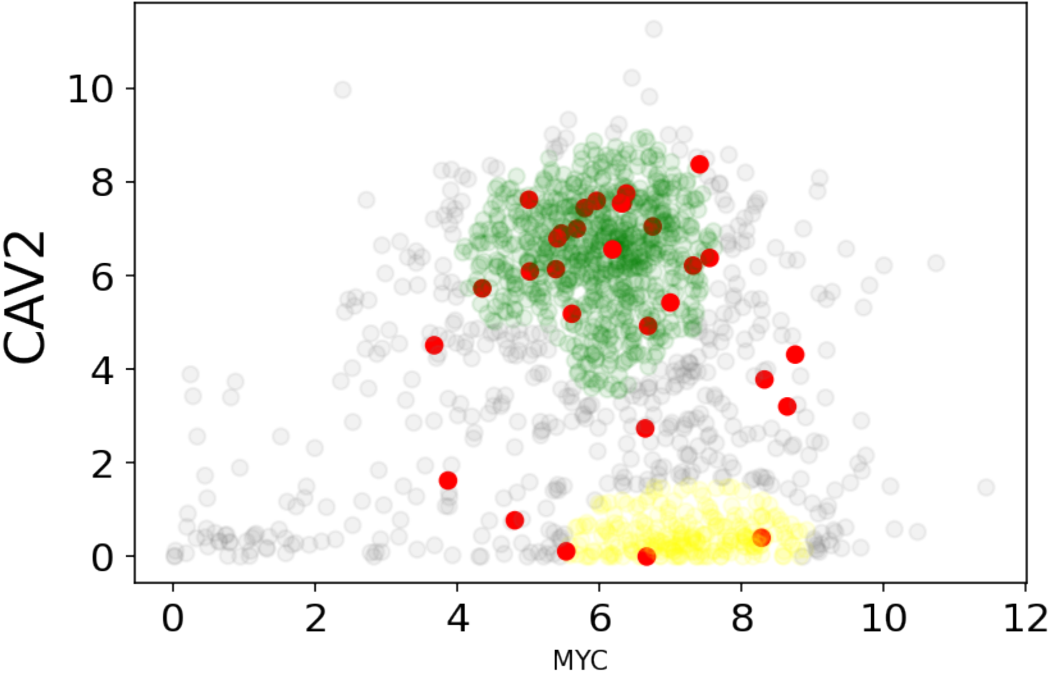

CAV2
MYC
10
8
6
4
2
0
0
2
4
6
8
10
12

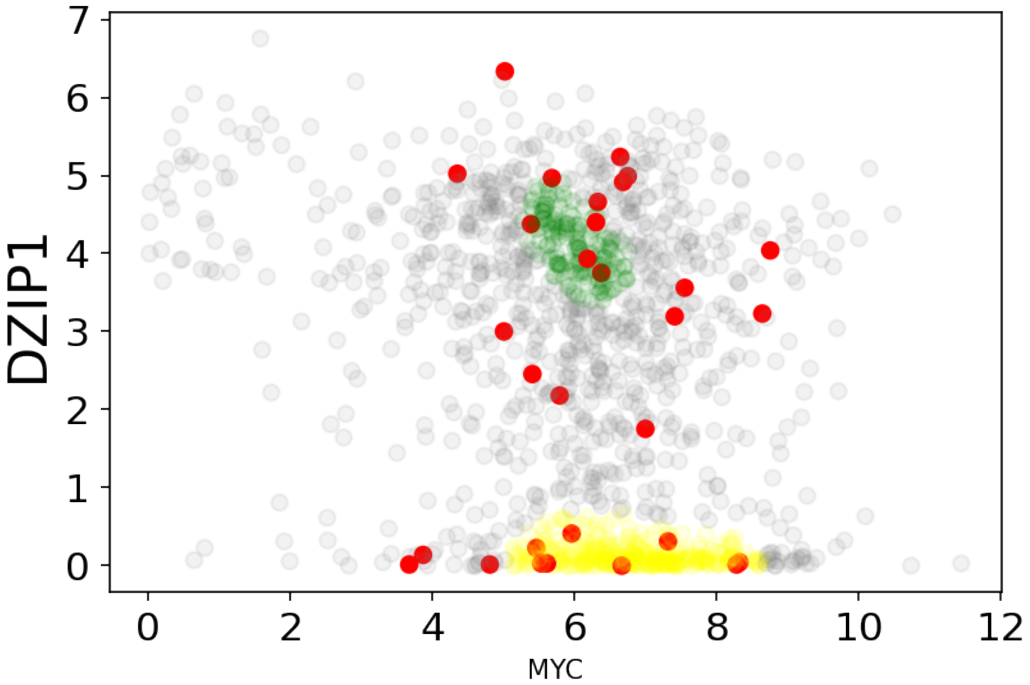
DZIP1
MYC
0
1
2
3
4
5
6
7
0
2
4
6
8
10
12

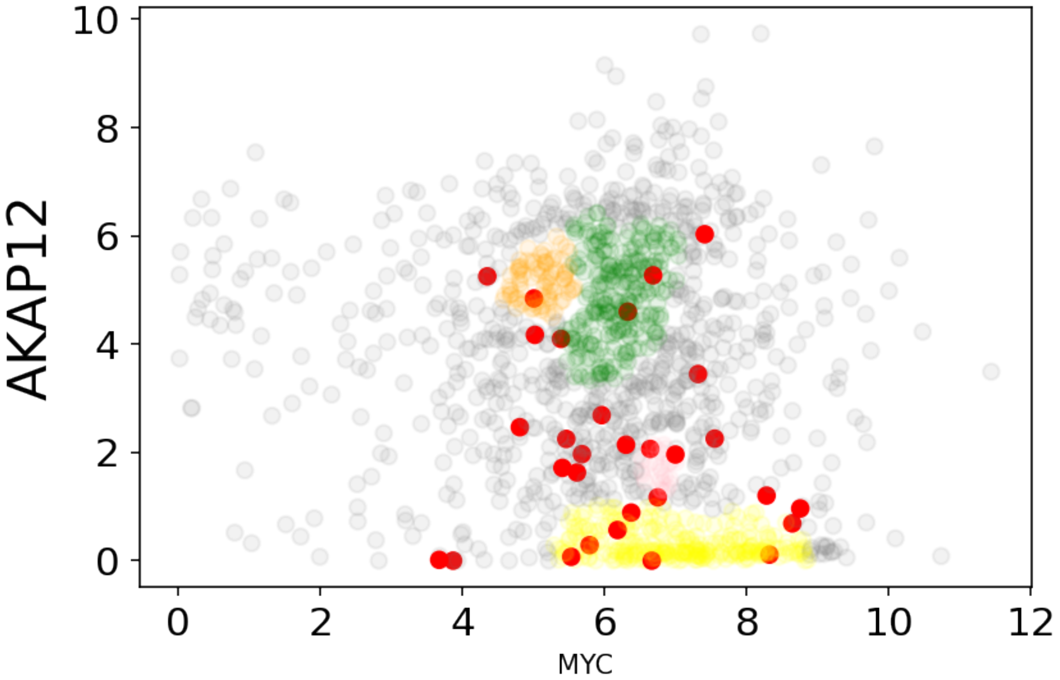
AKAP12
MYC
0
2
4
6
8
10
12

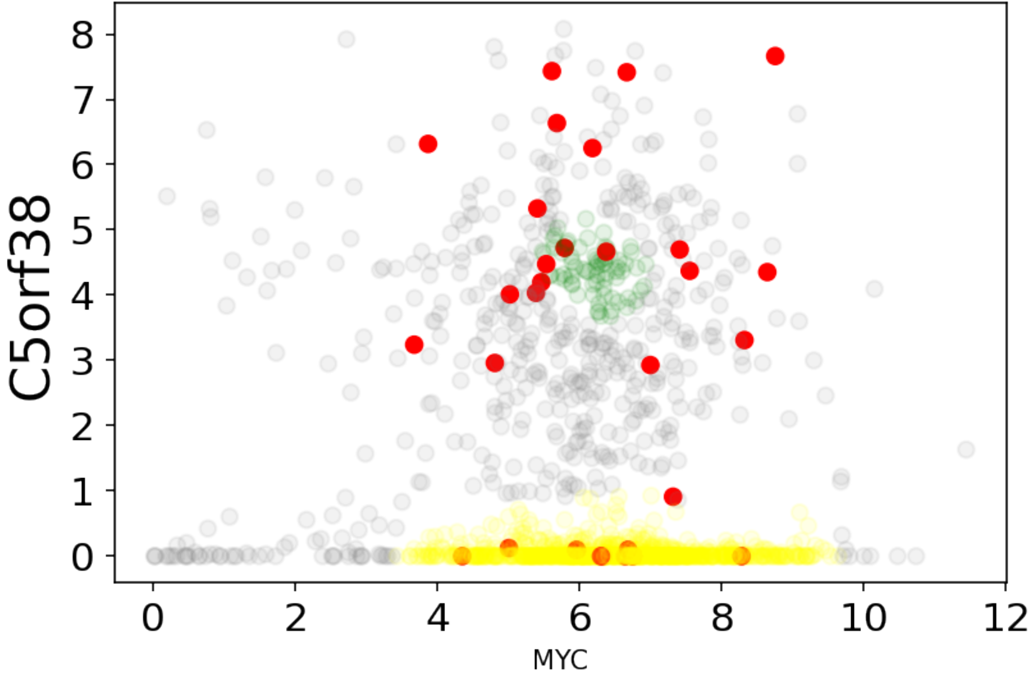

C5orf38
MYC
0
1
2
3
4
5
6
7
8
0
2
4
6
8
10
12

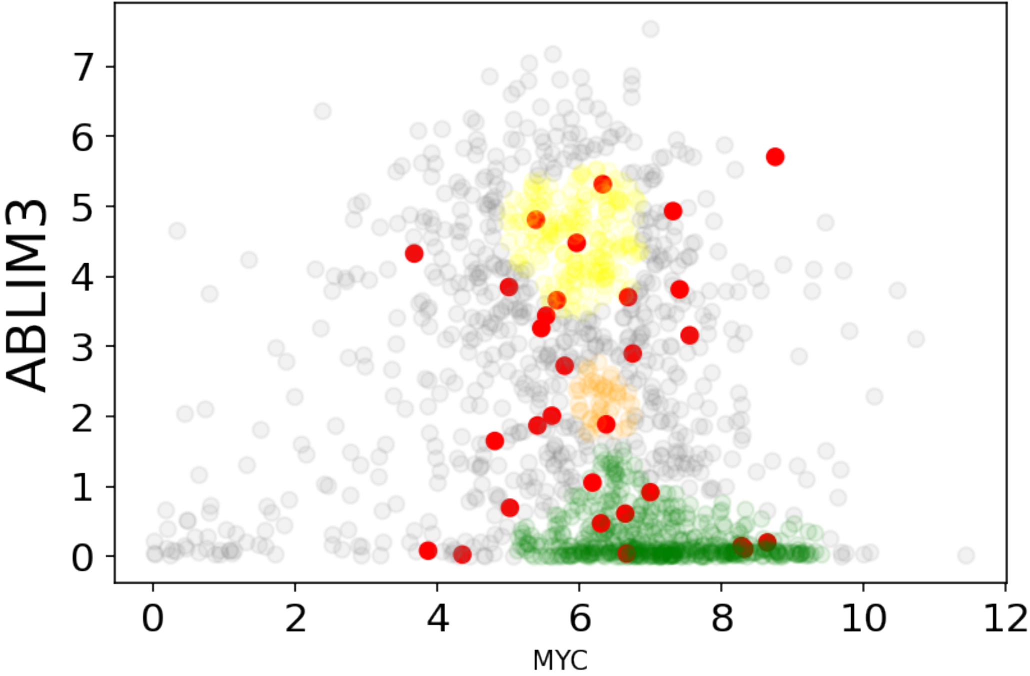

ABLIM3
MYC
0
1
2
3
4
5
6
7
0
2
4
6
8
10
12

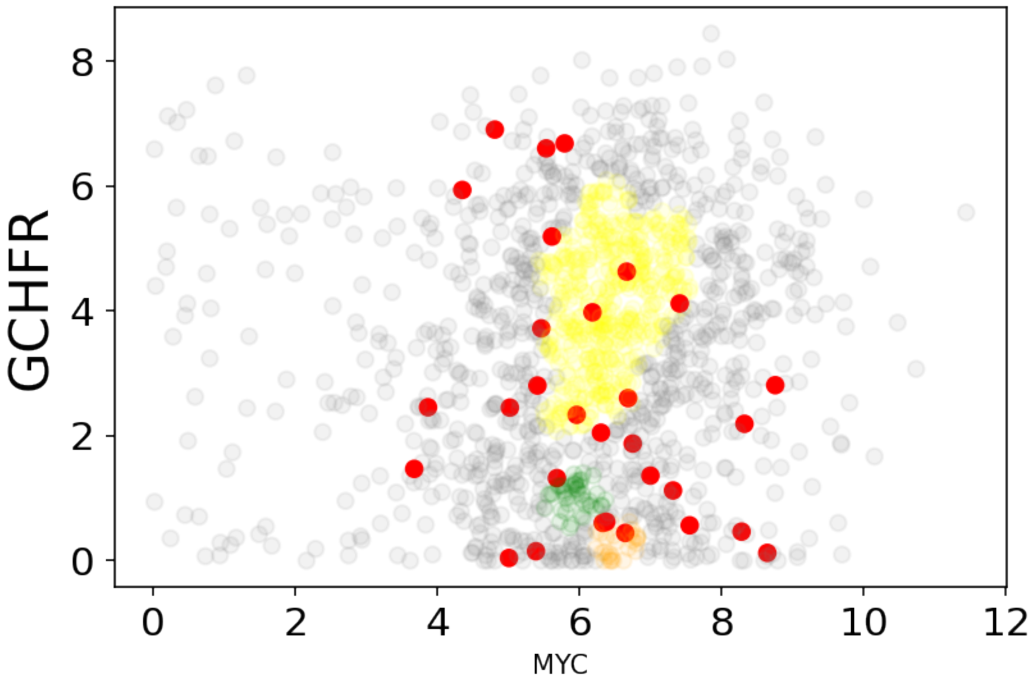

GCHFR
MYC
0
2
4
6
8
10
12

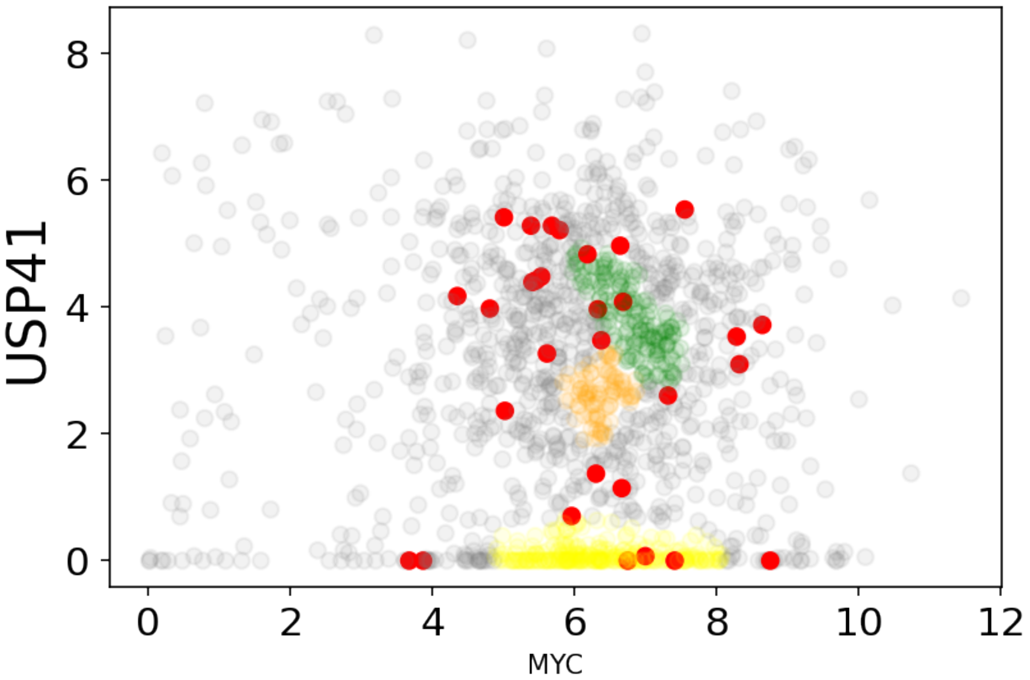
USP41
MYC
0
2
4
6
8
0
2
4
6
8
10
12

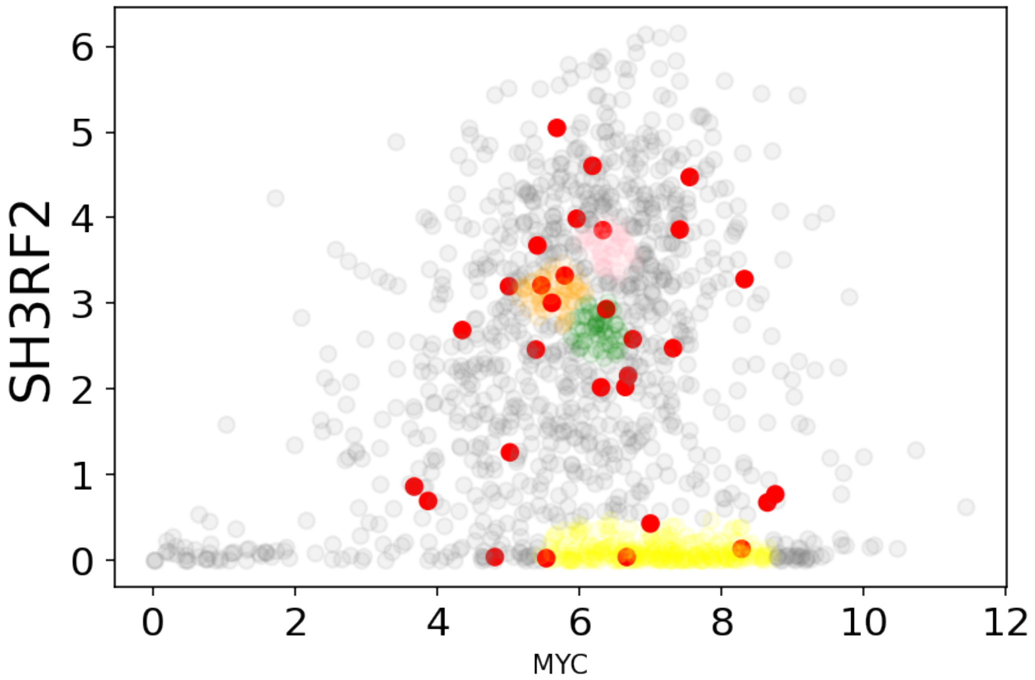
SH3RF2
0
1
2
3
4
5
6
0
2
4
6
8
10
12
MYC

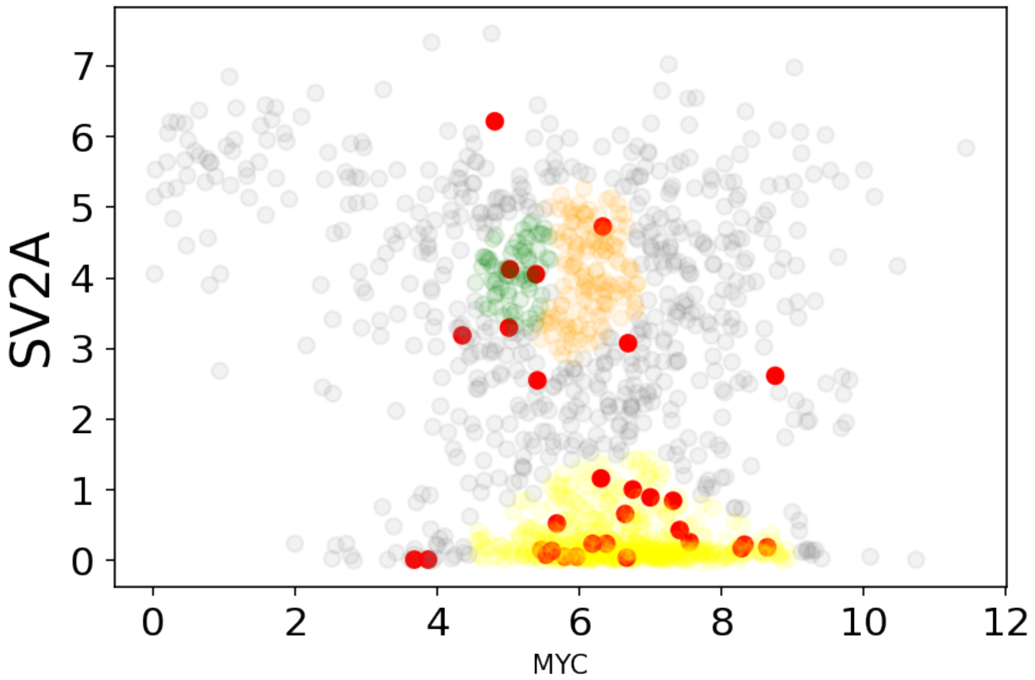
SV2A
MYC
0
1
2
3
4
5
6
7
0
2
4
6
8
10
12

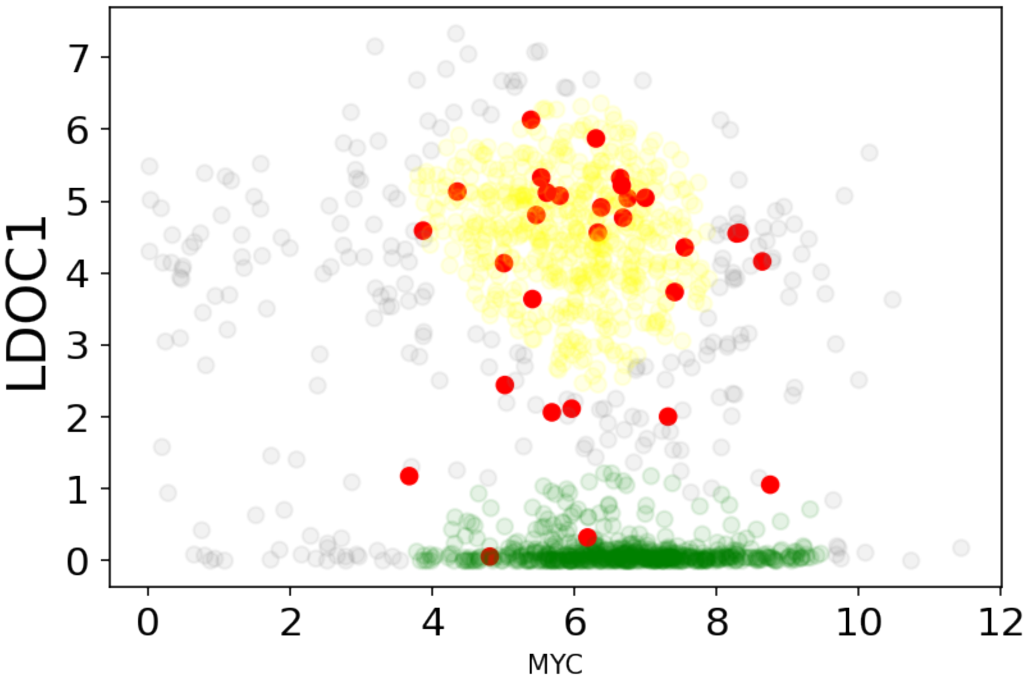

LDOC1
0
1
2
3
4
5
6
7
0
2
4
6
8
10
12
MYC

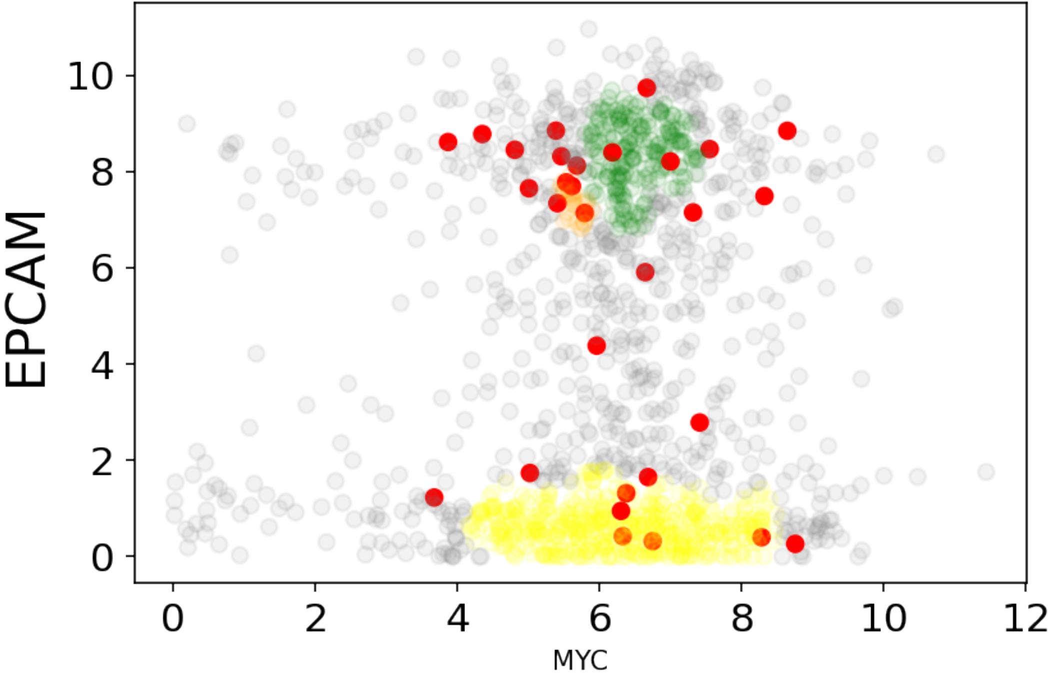

EPCAM
10
8
6
4
2
0
0
2
4
6
8
10
12
MYC

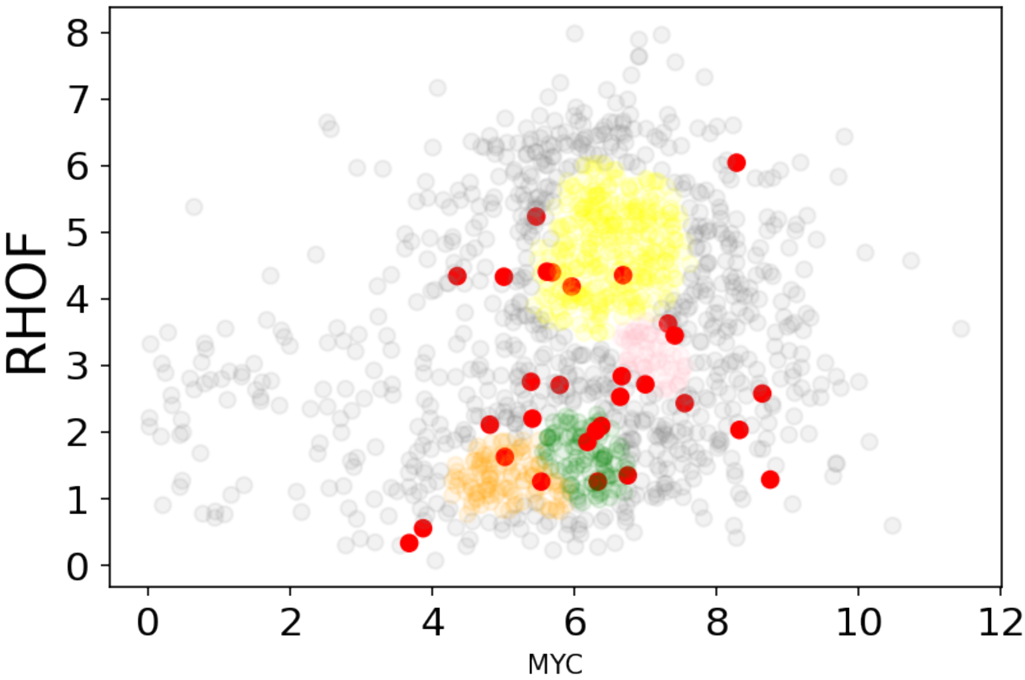

RHOF
MYC
0
1
2
3
4
5
6
7
8
0
2
4
6
8
10
12

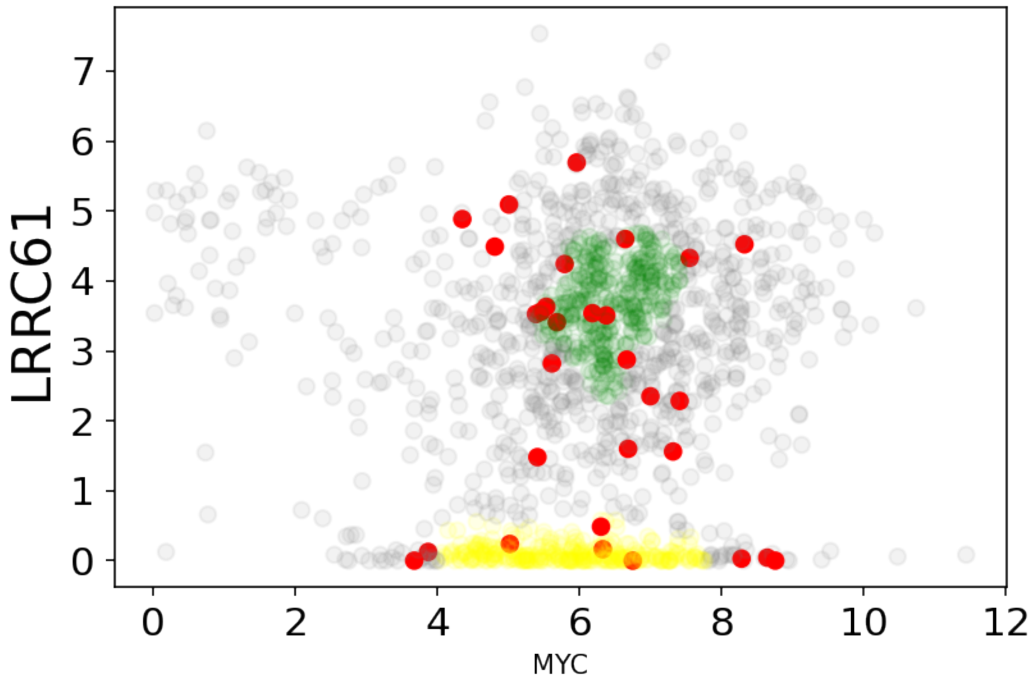

LRRC61
7
6
5
4
3
2
1
0
0
2
4
6
8
10
12
MYC

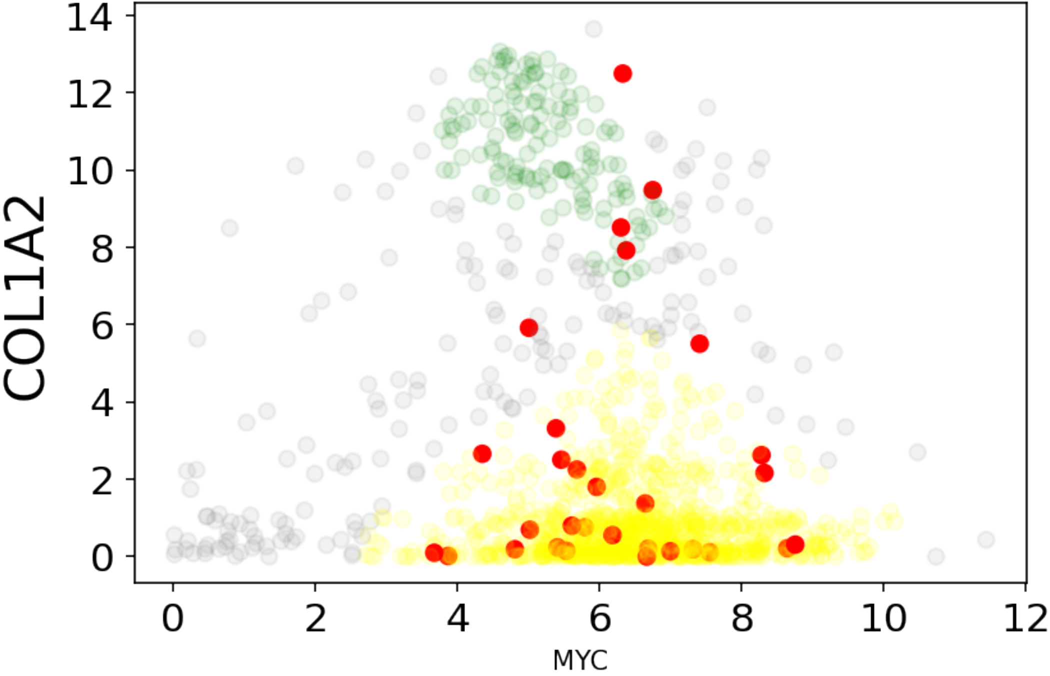
COL1A2
MYC
0
2
4
6
8
10
12
14
0
2
4
6
8
10
12

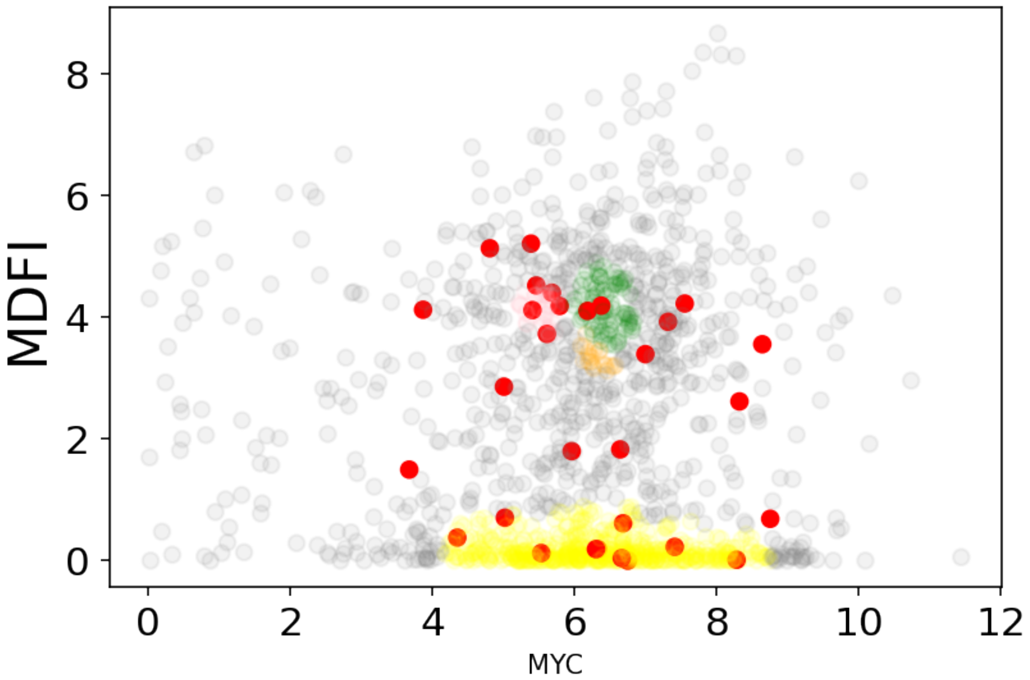

MDFI
MYC
0
2
4
6
8
10
12

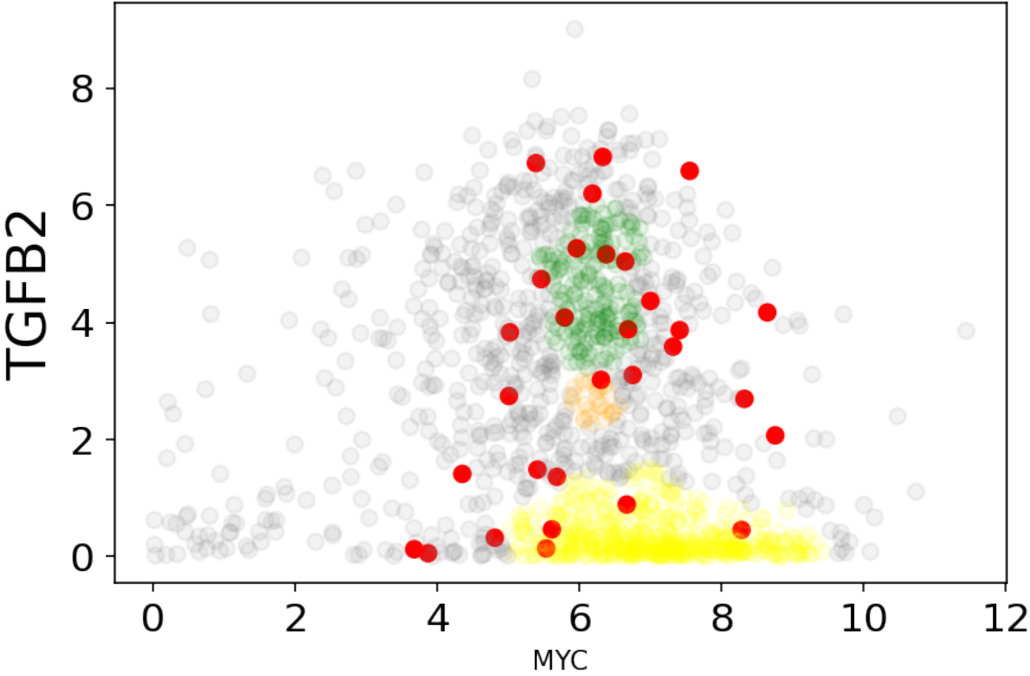

TGFB2
MYC
0
2
4
6
8
0
2
4
6
8
10
12

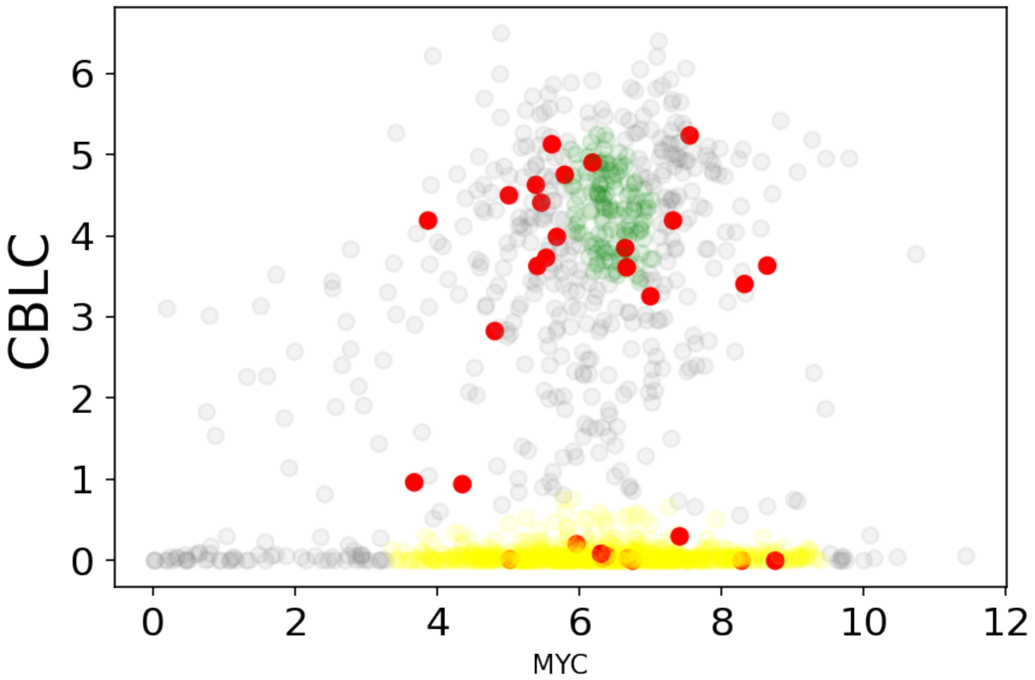
CBLC
6
5
4
3
2
1
0
0
2
4
6
8
10
12
MYC

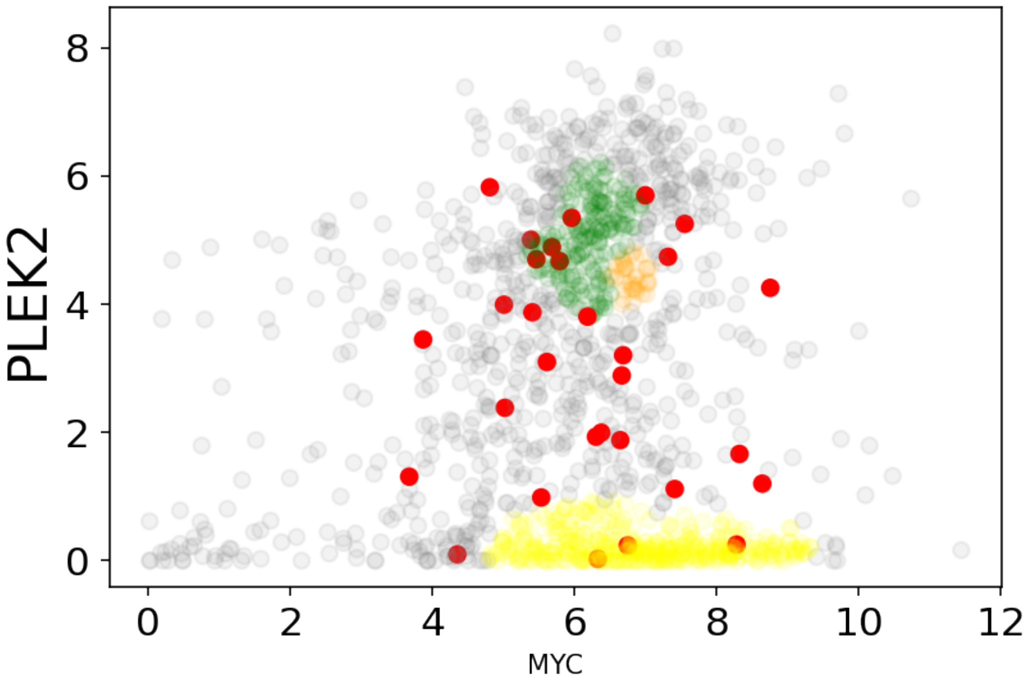
PLEK2
MYC
0
2
4
6
8
0
2
4
6
8
10
12

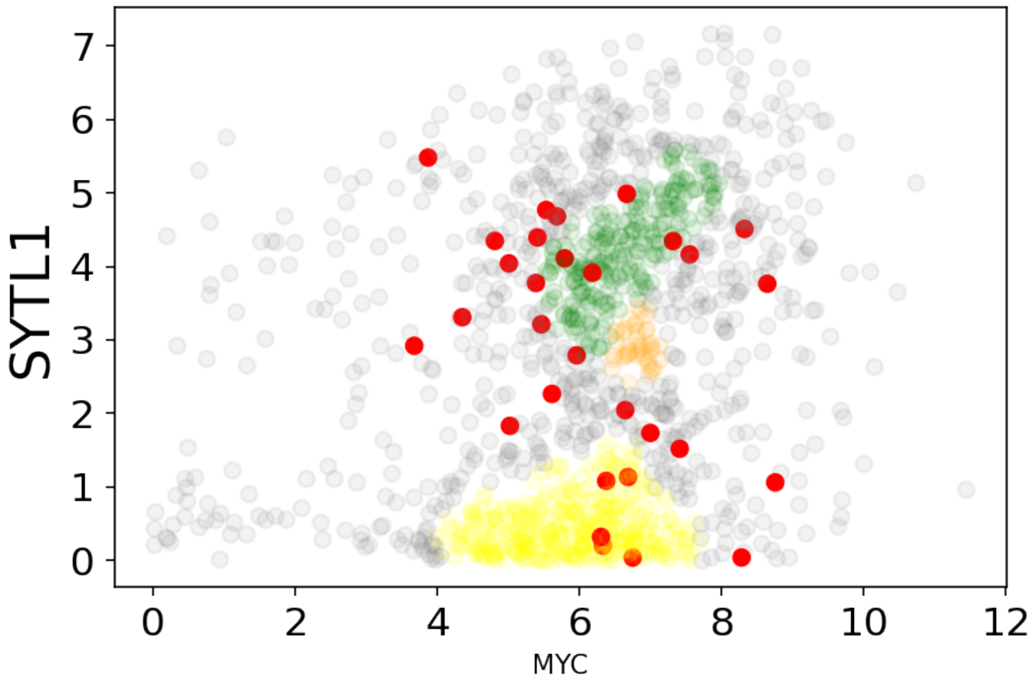

SYTL1
MYC
0
1
2
3
4
5
6
7
0
2
4
6
8
10
12

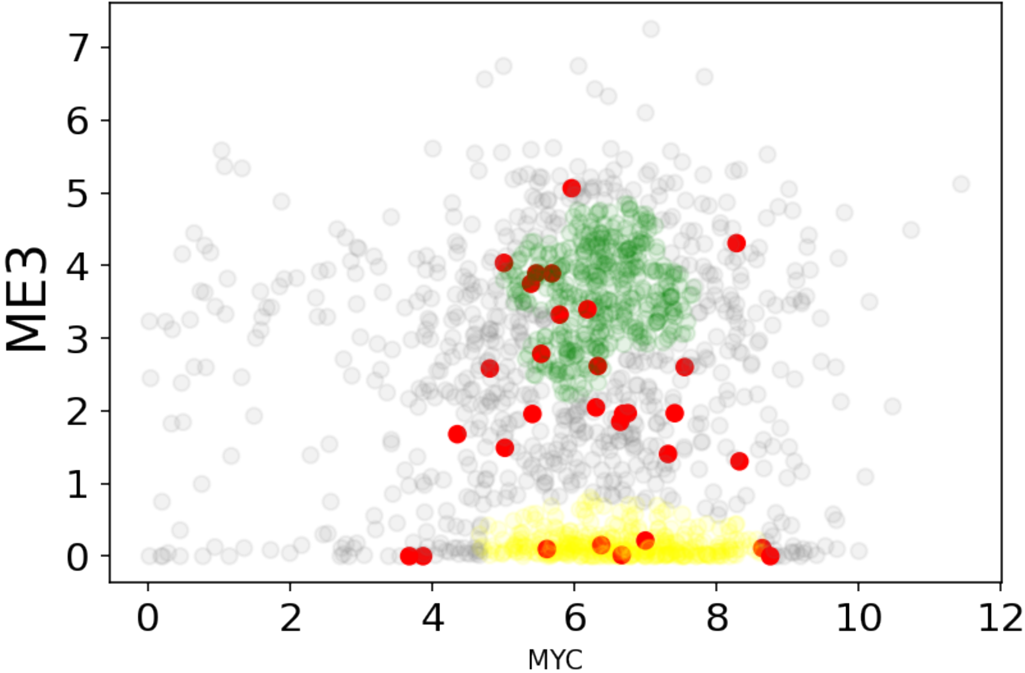
ME3
MYC
0
1
2
3
4
5
6
7
0
2
4
6
8
10
12

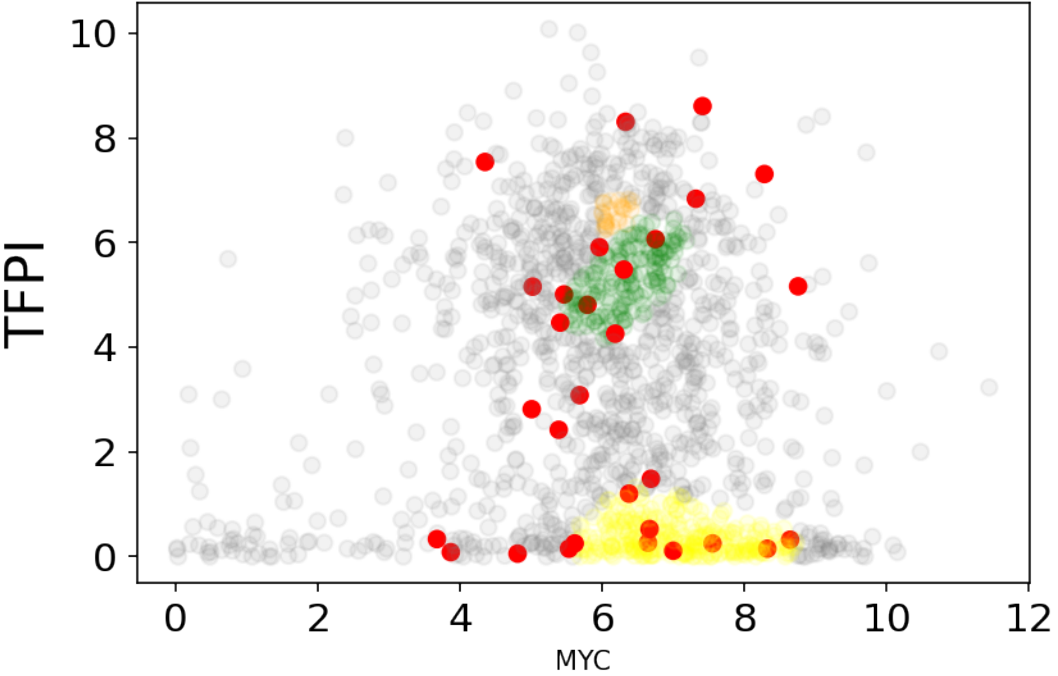

TFPI
10
8
6
4
2
0
0
2
4
6
8
10
12
MYC

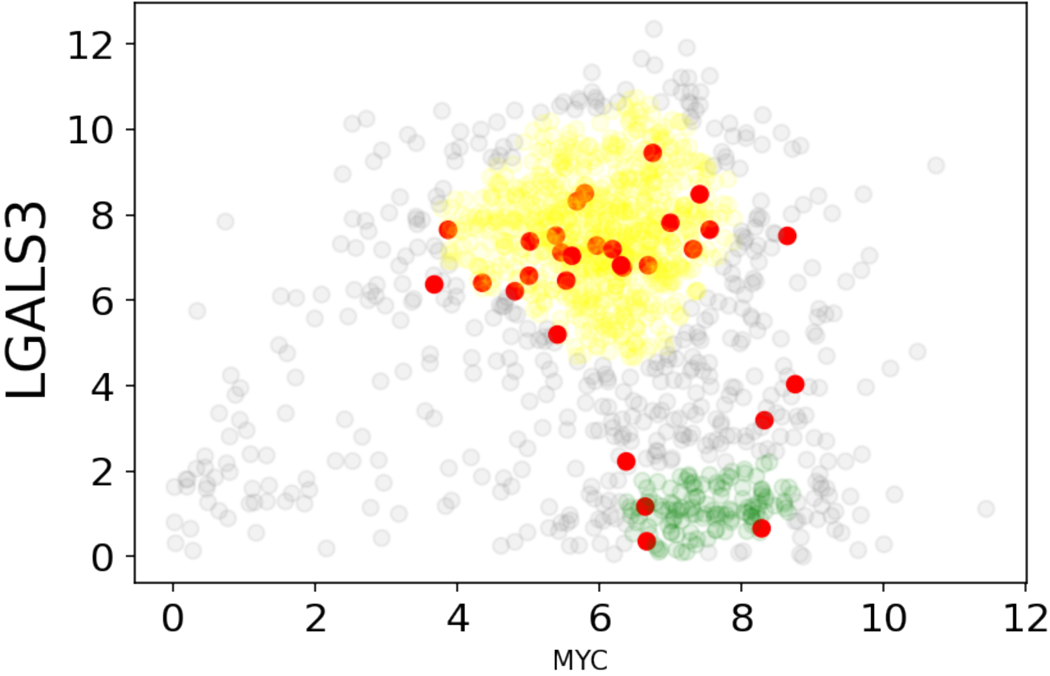
LGALS3
MYC
0
2
4
6
8
10
12

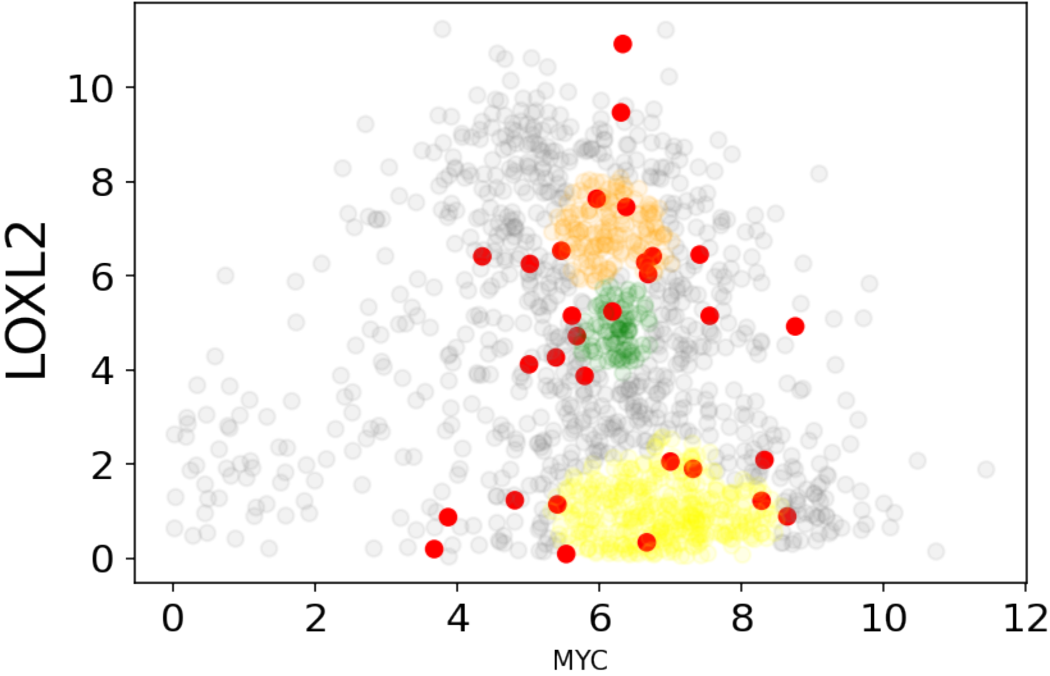

LOXL2
10
8
6
4
2
0
0
2
4
6
8
10
12
MYC

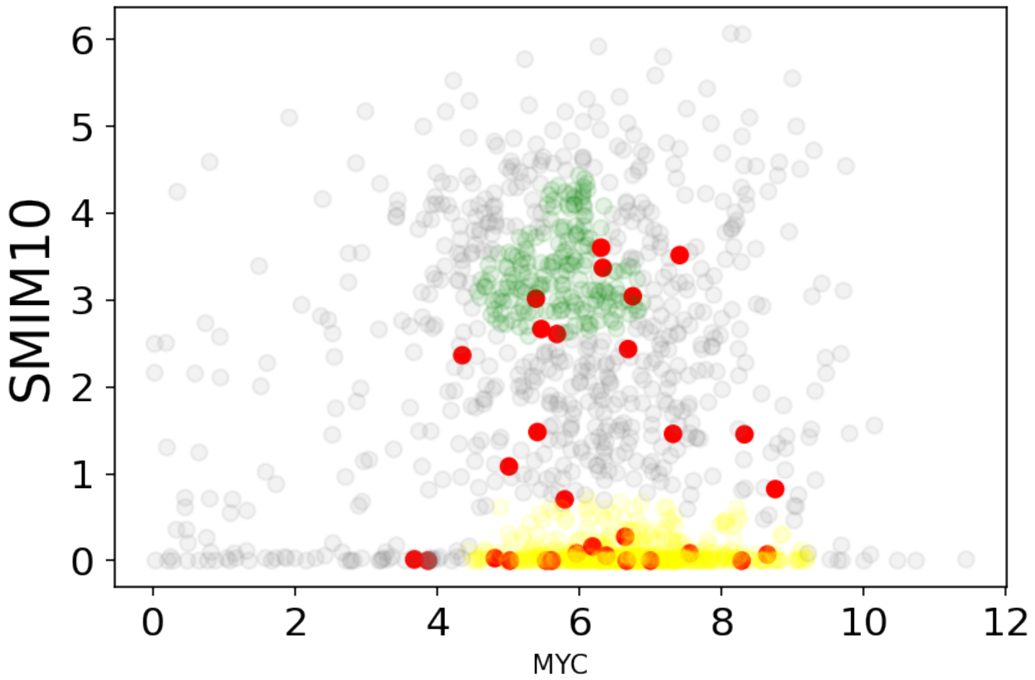

SMIM10
6
5
4
3
2
1
0
0
2
4
6
8
10
12
MYC

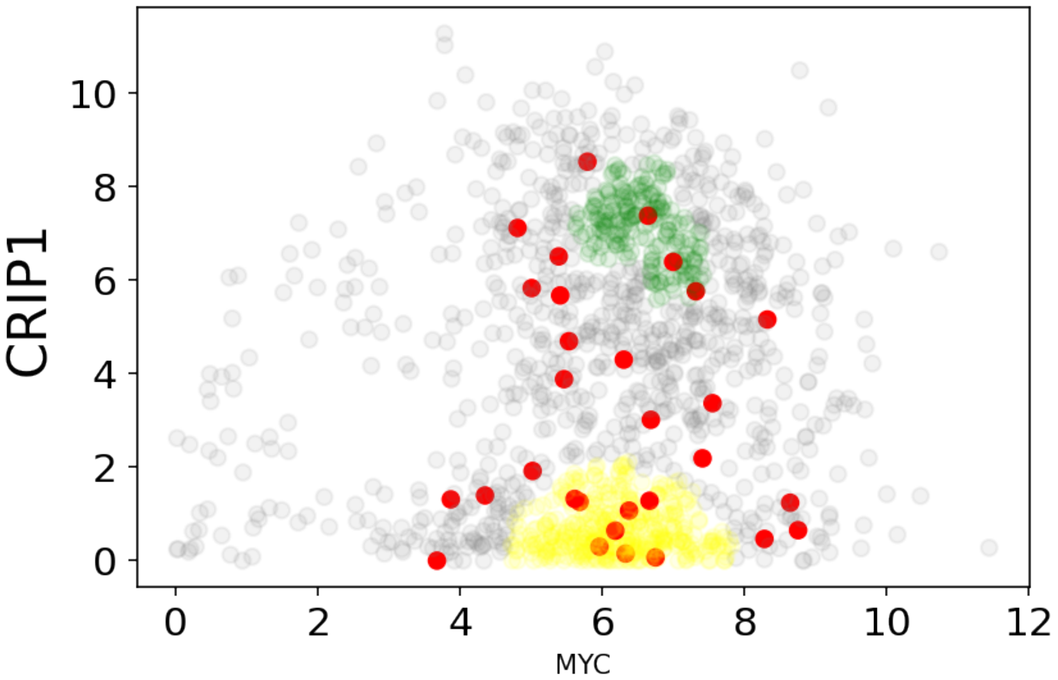

CRIP1
MYC
10
8
6
4
2
0
0
2
4
6
8
10
12

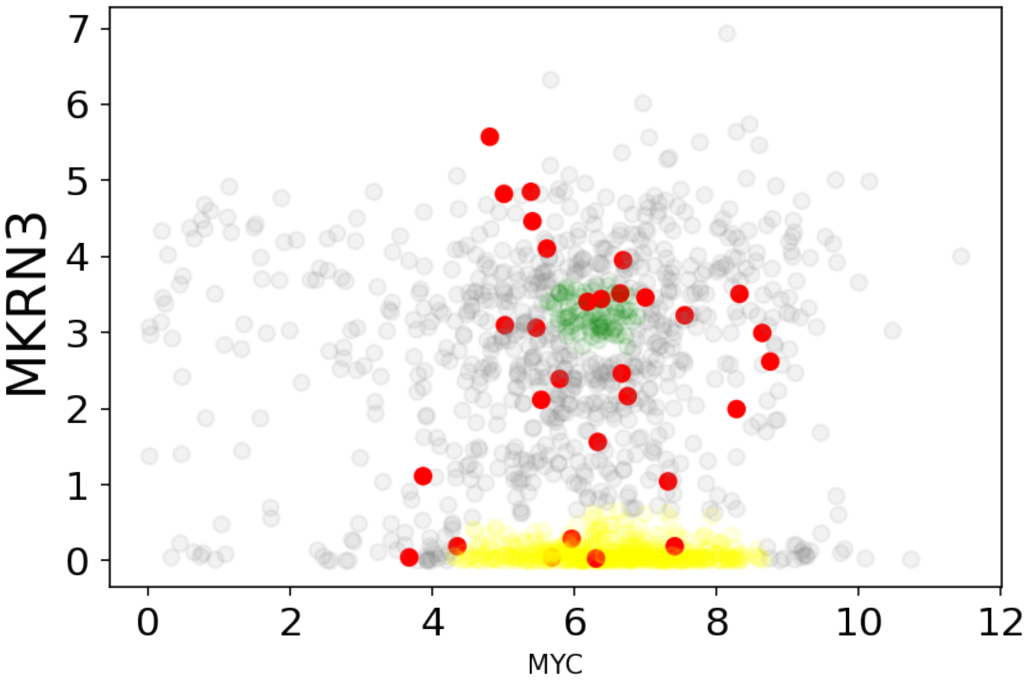

MKRN3
MYC
0
1
2
3
4
5
6
7
0
2
4
6
8
10
12

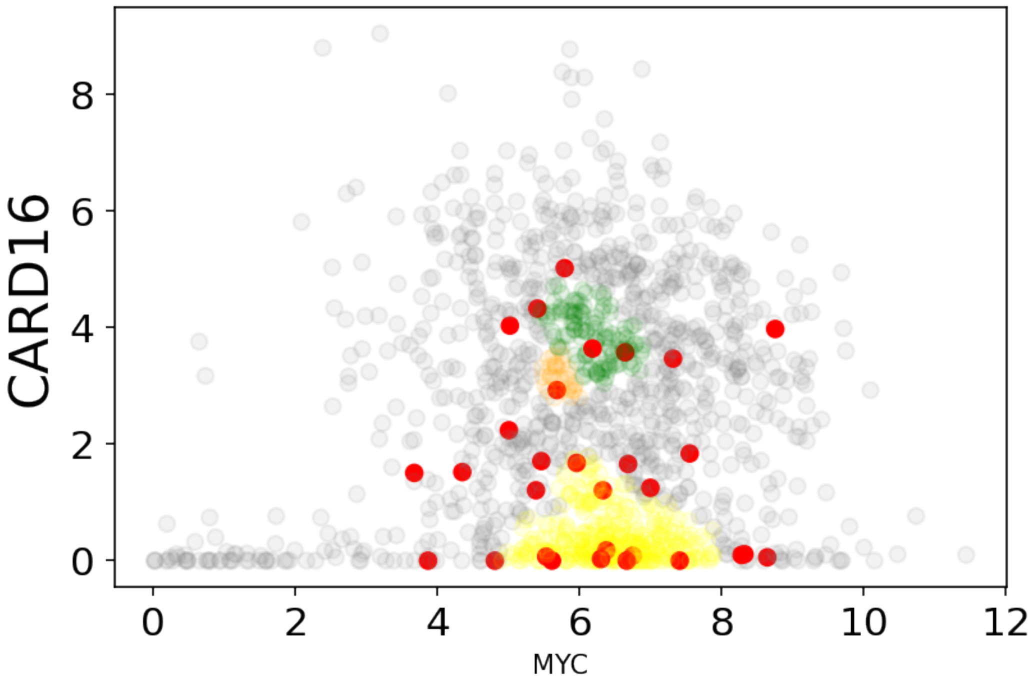

CARD16
MYC
0
2
4
6
8
10
12

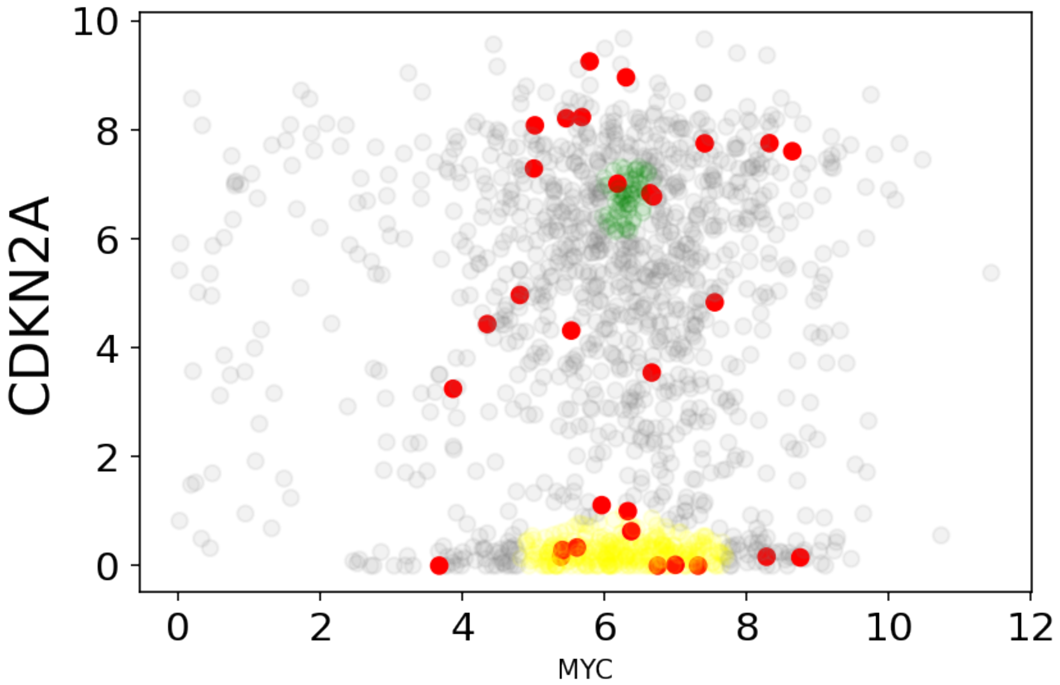

CDKN2A
MYC
0
2
4
6
8
10
12

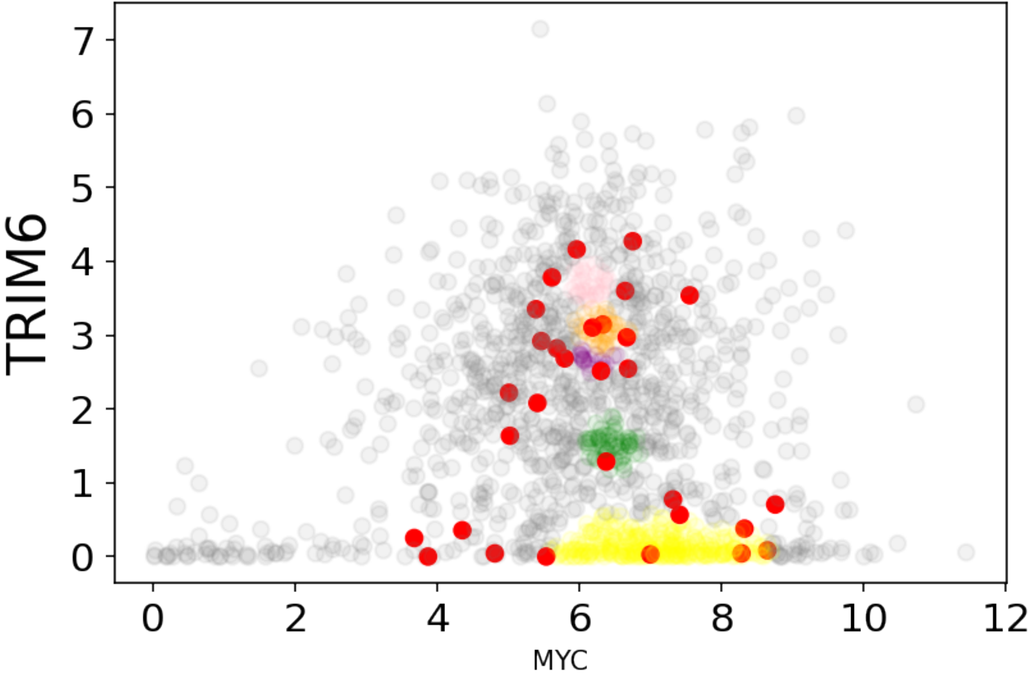
TRIM6
MYC
0
1
2
3
4
5
6
7
0
2
4
6
8
10
12

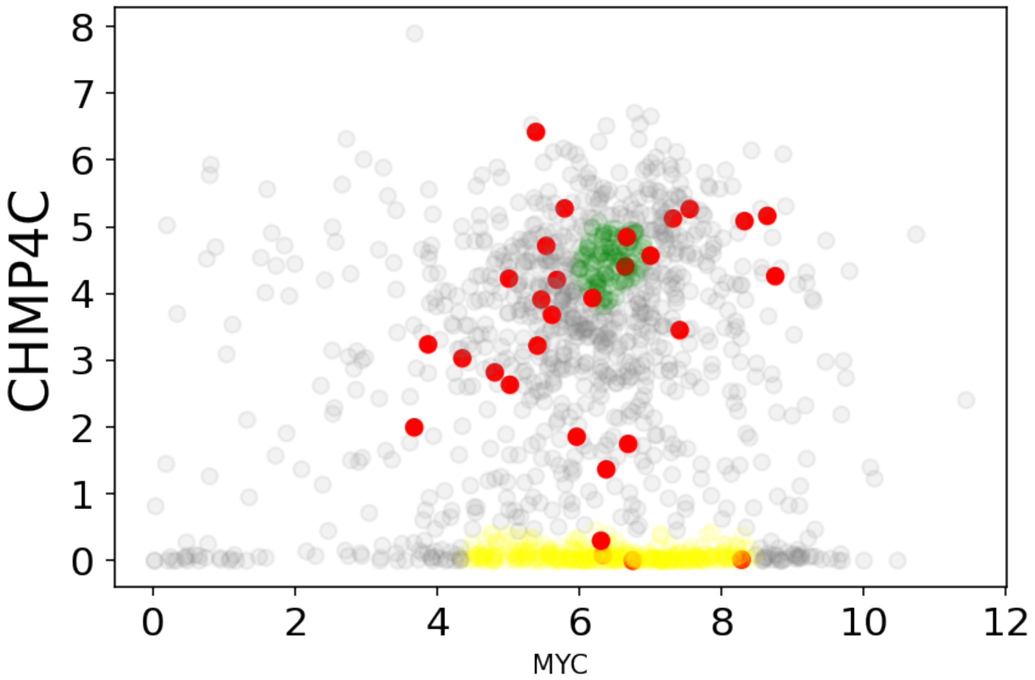
CHMP4C
MYC
0
1
2
3
4
5
6
7
8
0
2
4
6
8
10
12

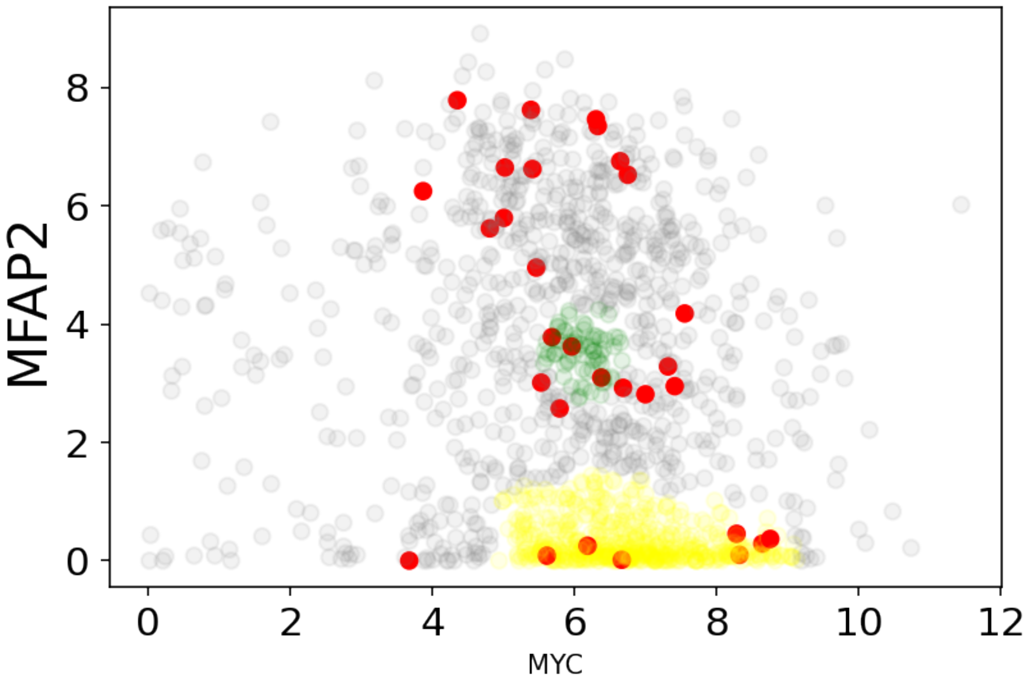
MFAP2
8
6
4
2
0
0
2
4
6
8
10
12
MYC

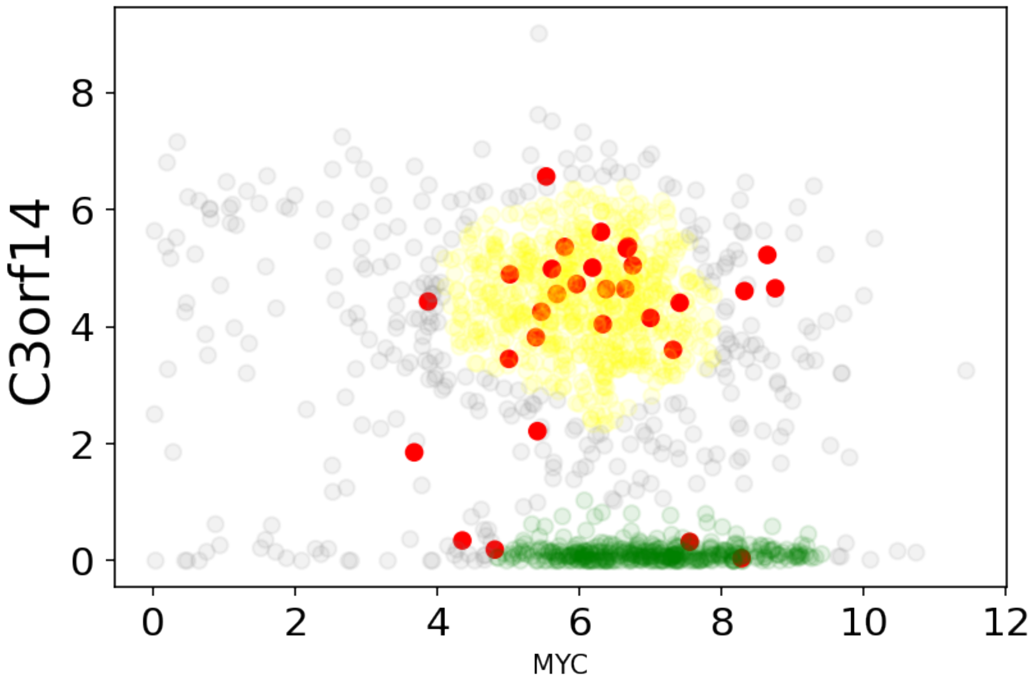

C3orf14
MYC
0
2
4
6
8
0
2
4
6
8
10
12

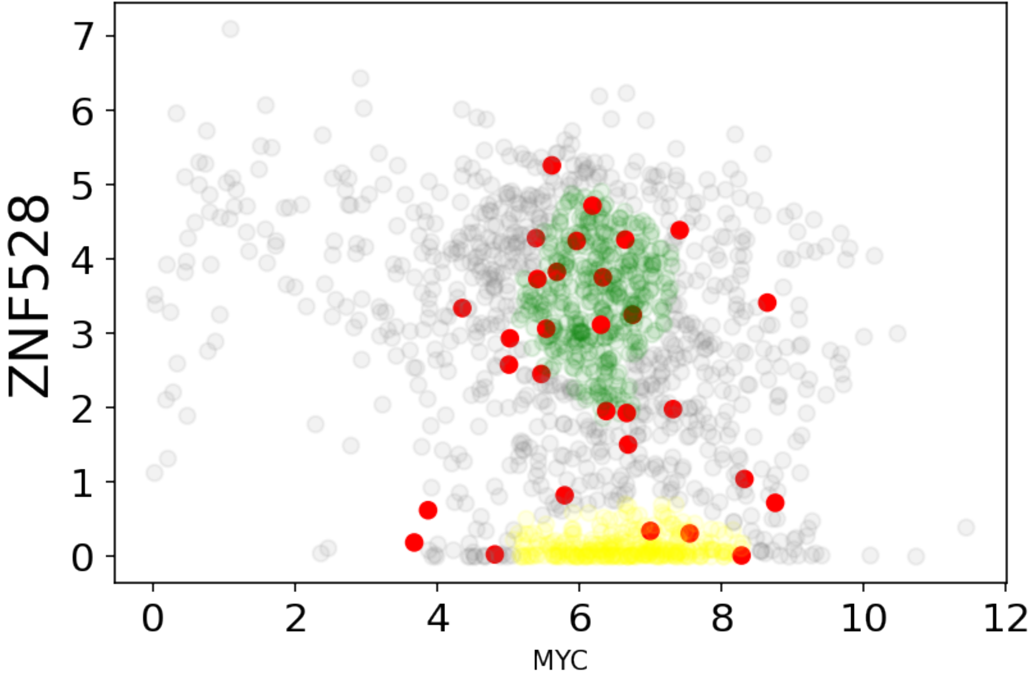

ZNF528
7
6
5
4
3
2
1
0
0
2
4
6
8
10
12
MYC

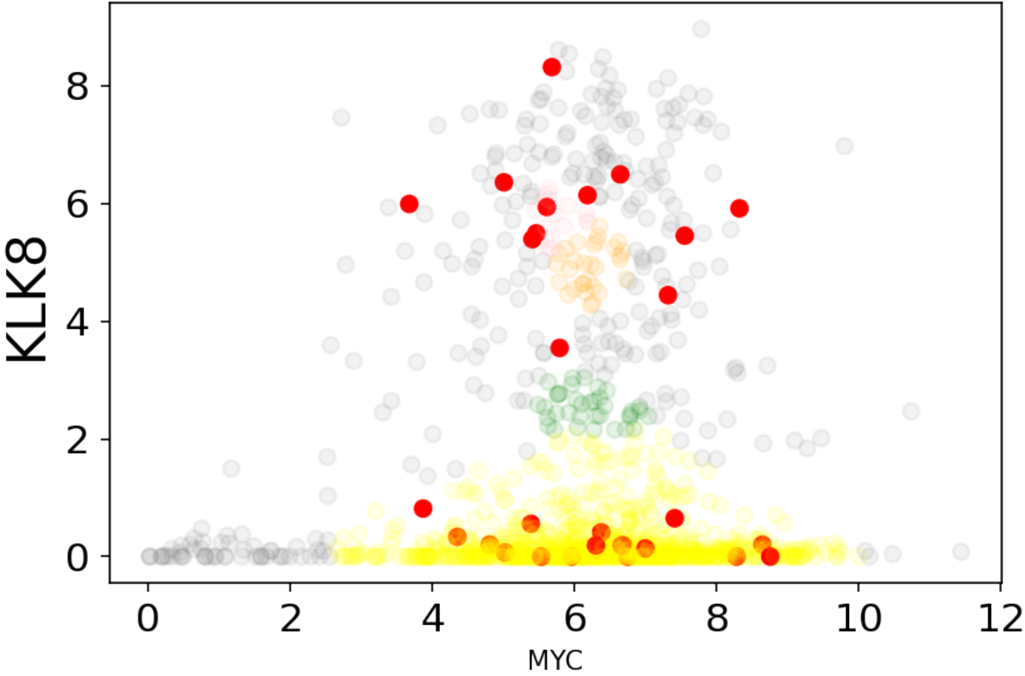

KLK8
0
2
4
6
8
0
2
4
6
8
10
12
MYC

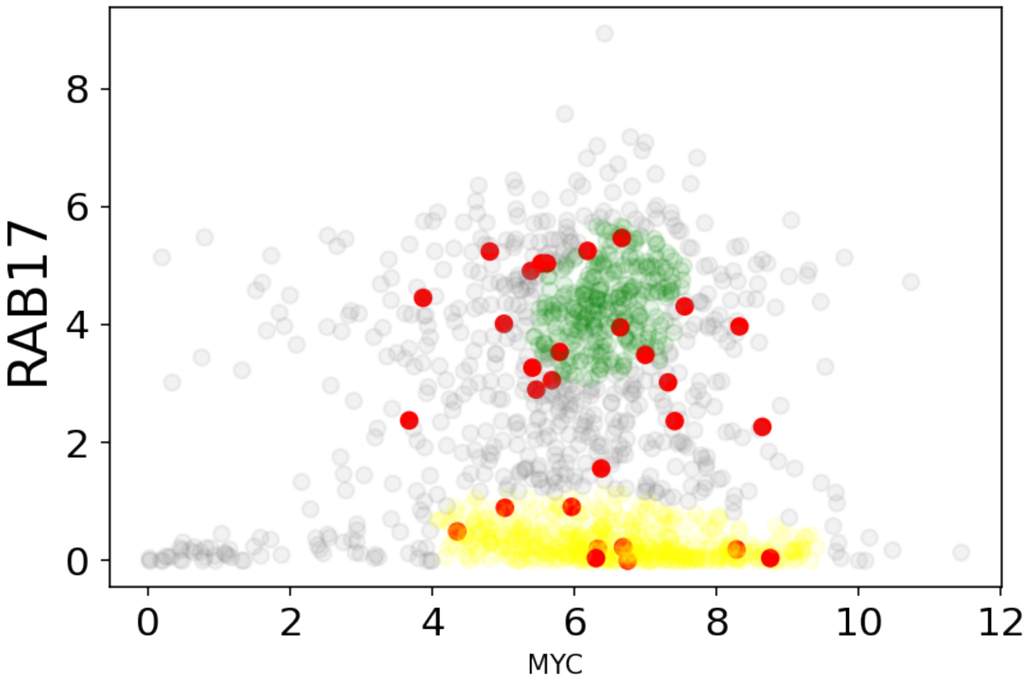

RAB17
8
6
4
2
0
0
2
4
6
8
10
12
MYC

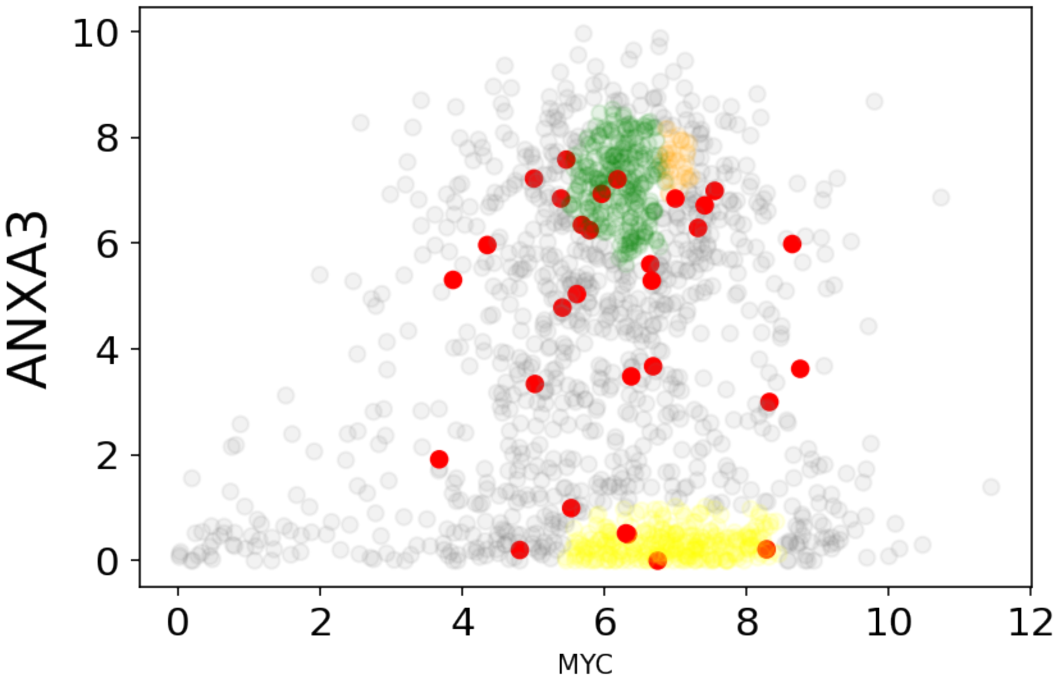

ANXA3
MYC
10
8
6
4
2
0
0
2
4
6
8
10
12

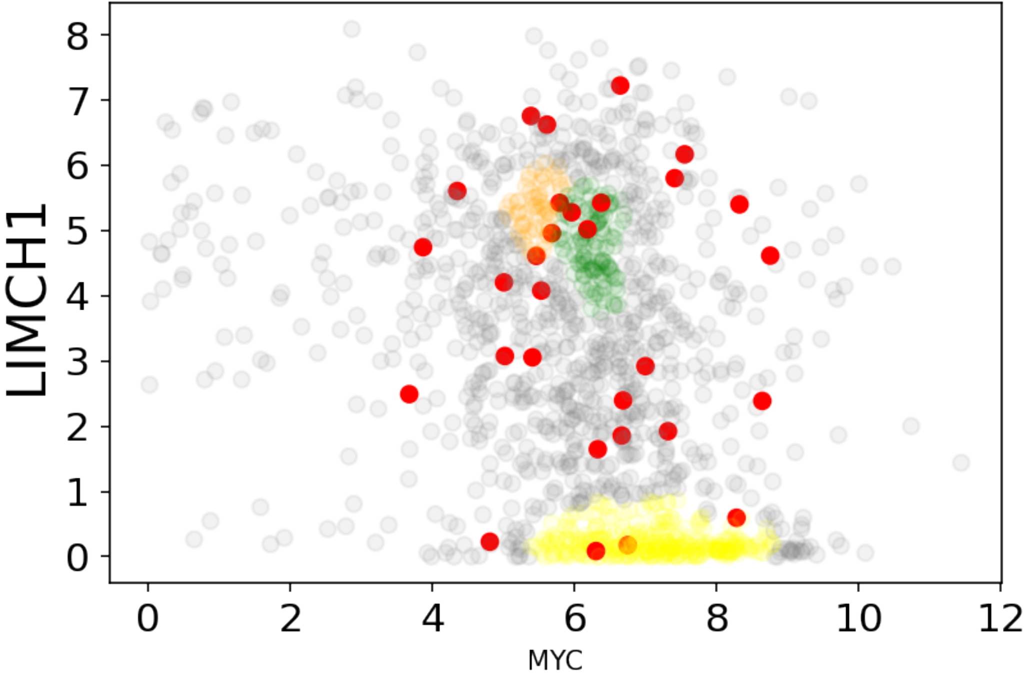

LIMCH1
MYC
0
1
2
3
4
5
6
7
8
0
2
4
6
8
10
12

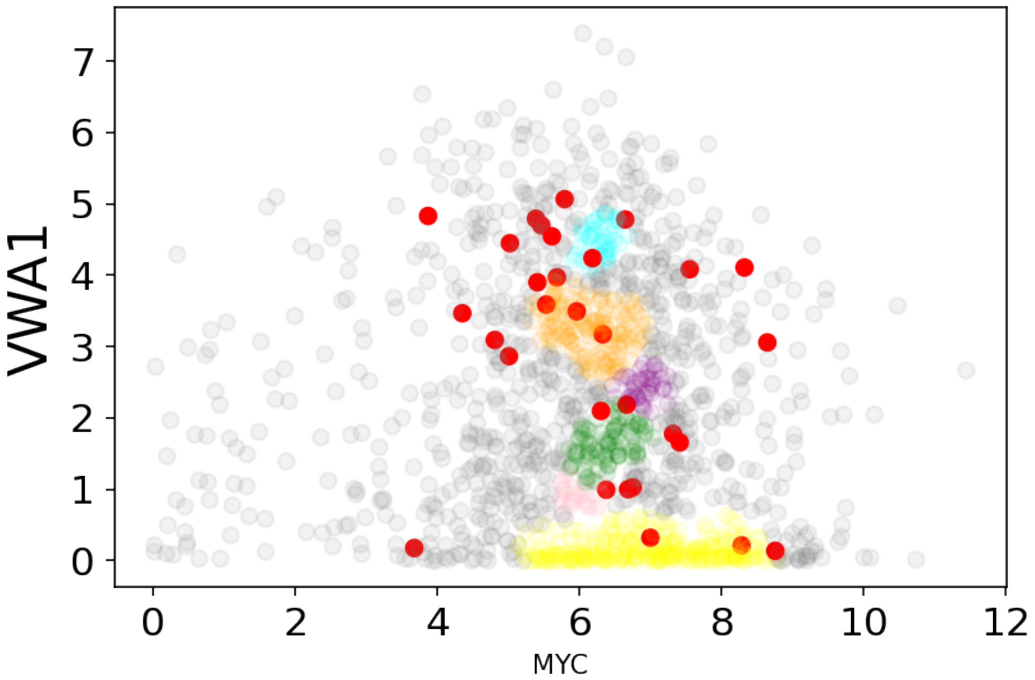

VWA1
MYC
0
1
2
3
4
5
6
7
0
2
4
6
8
10
12

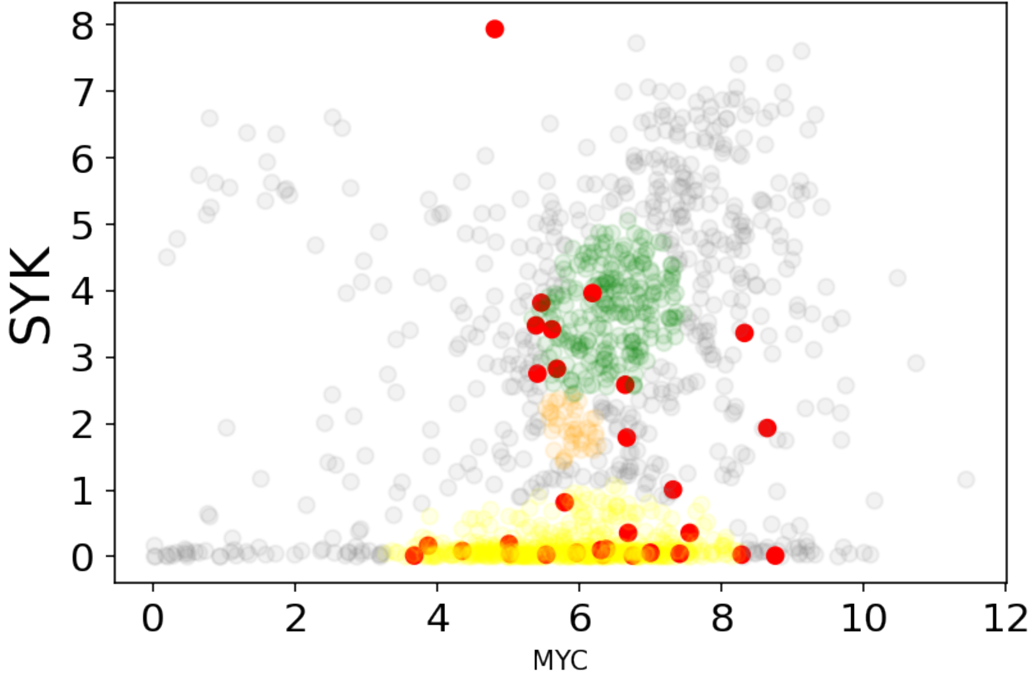

SYK
MYC
0
1
2
3
4
5
6
7
8
0
2
4
6
8
10
12

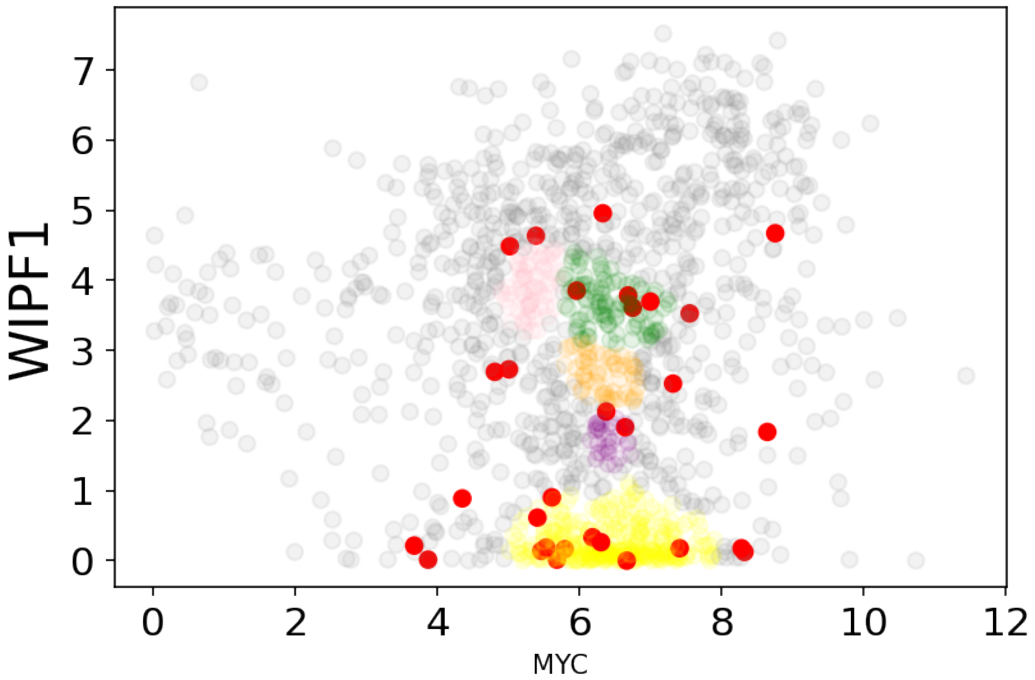
WIPF1
0
1
2
3
4
5
6
7
0
2
4
6
8
10
12
MYC

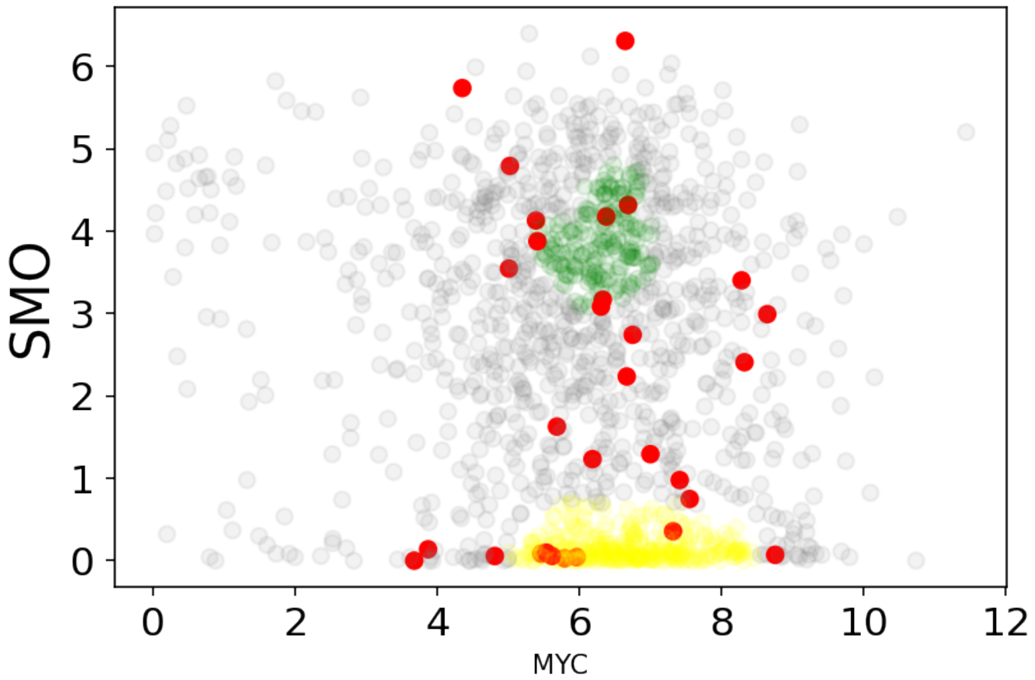
SMO
6
5
4
3
2
1
0
0
2
4
6
8
10
12
MYC

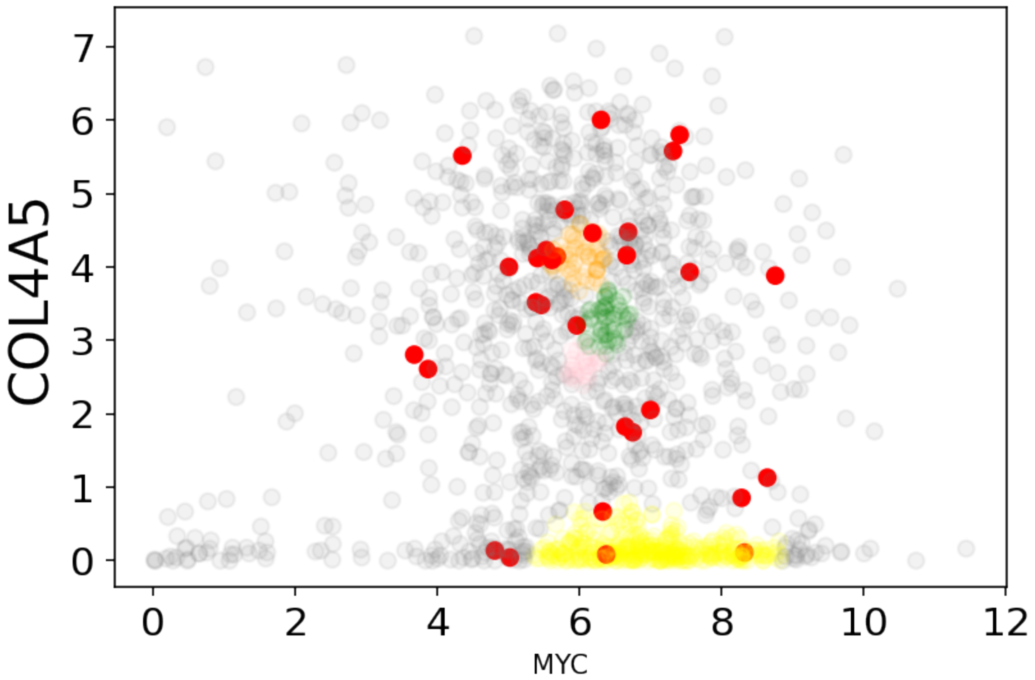

COL4A5
MYC
0
1
2
3
4
5
6
7
0
2
4
6
8
10
12

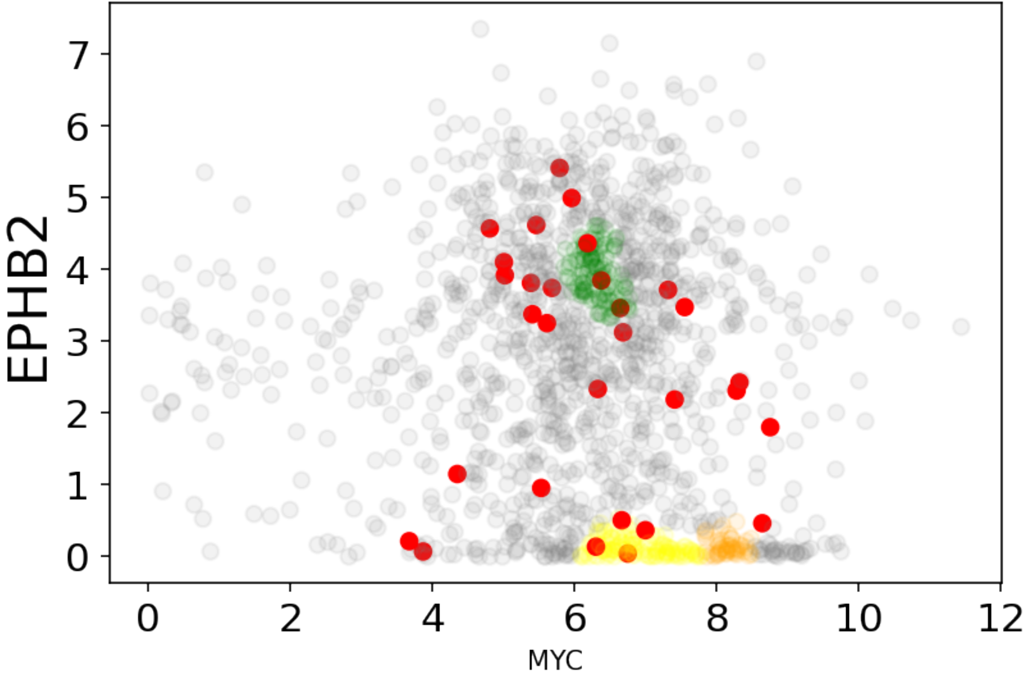
EPHB2
MYC
0
1
2
3
4
5
6
7
0
2
4
6
8
10
12

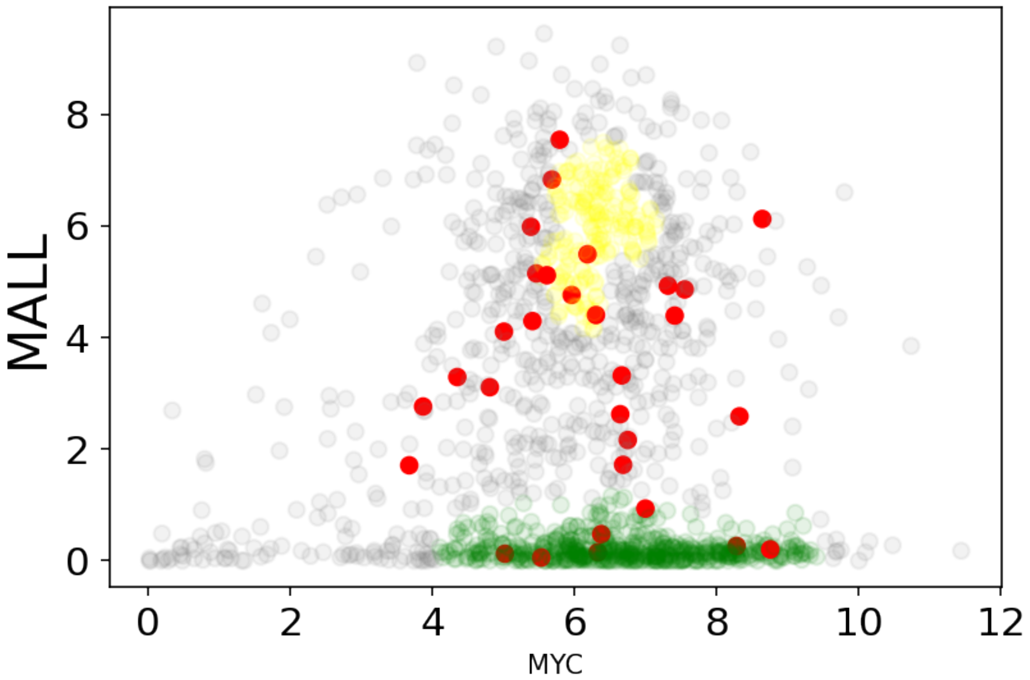

MALL
8
6
4
2
0
0
2
4
6
8
10
12
MYC

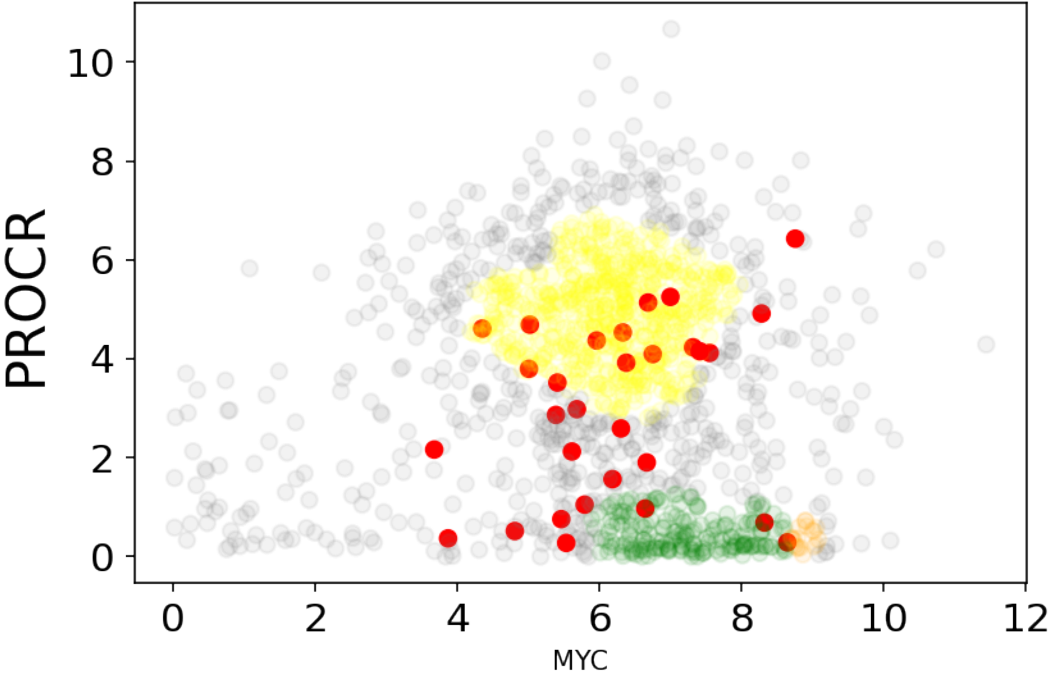

PROCR
MYC
0
2
4
6
8
10
12

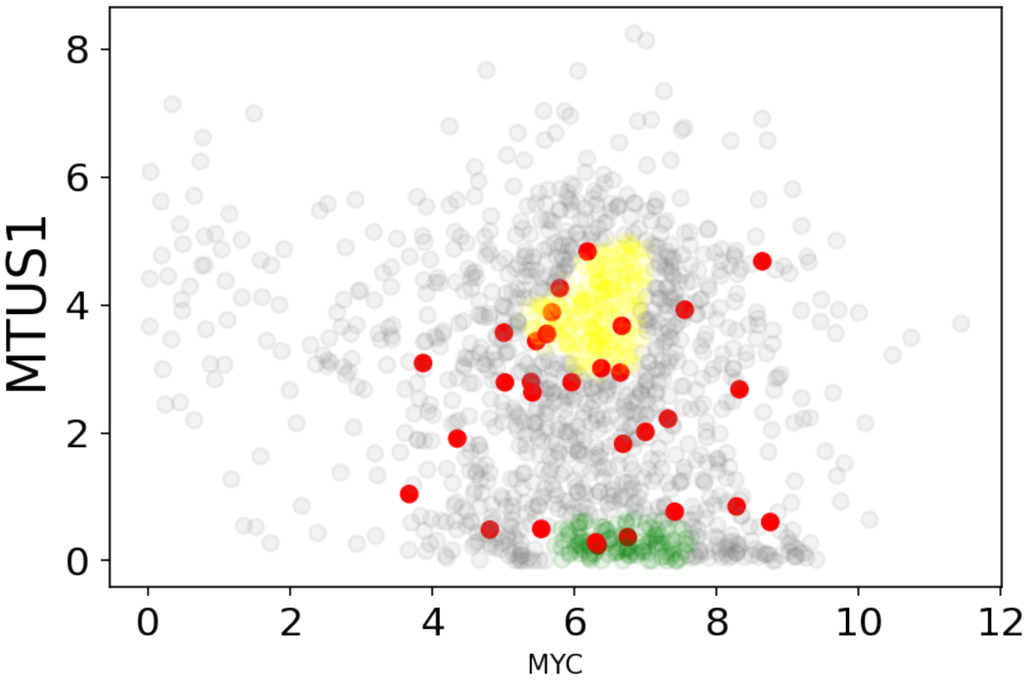

MTUS1
MYC
0
2
4
6
8
10
12

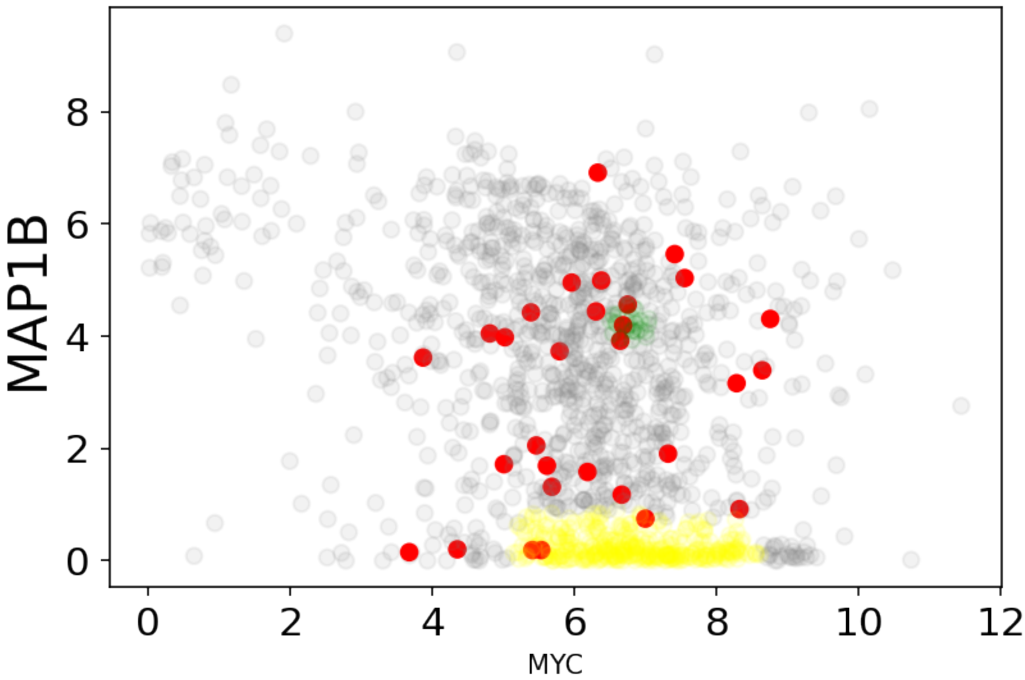

MAP1B
8
6
4
2
0
0
2
4
6
8
10
12
MYC

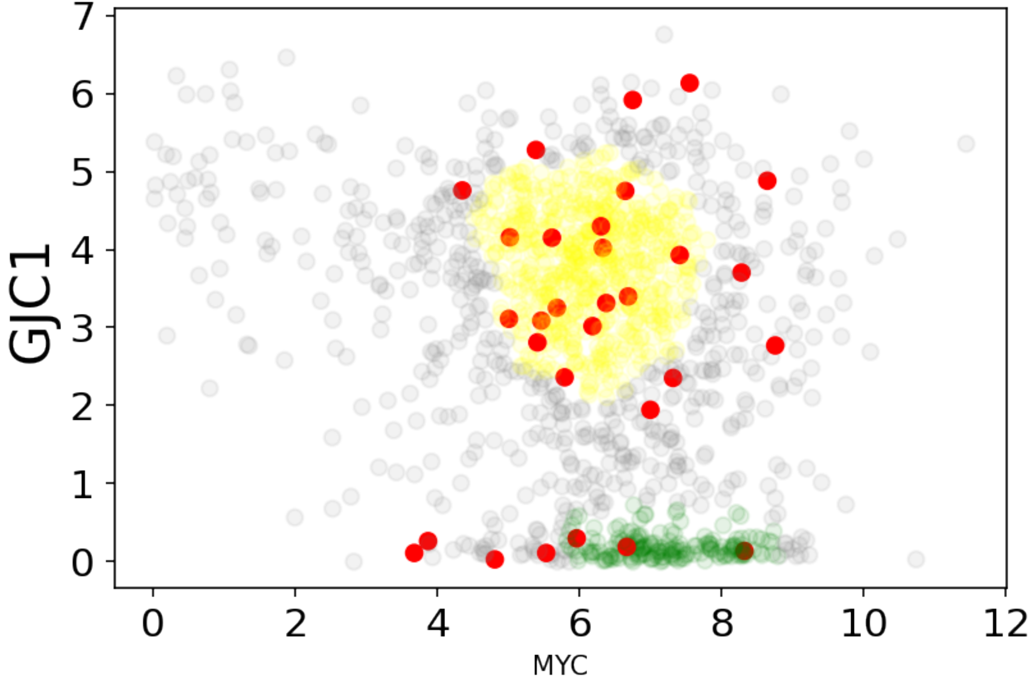

GJC1
MYC
0
1
2
3
4
5
6
7
0
2
4
6
8
10
12

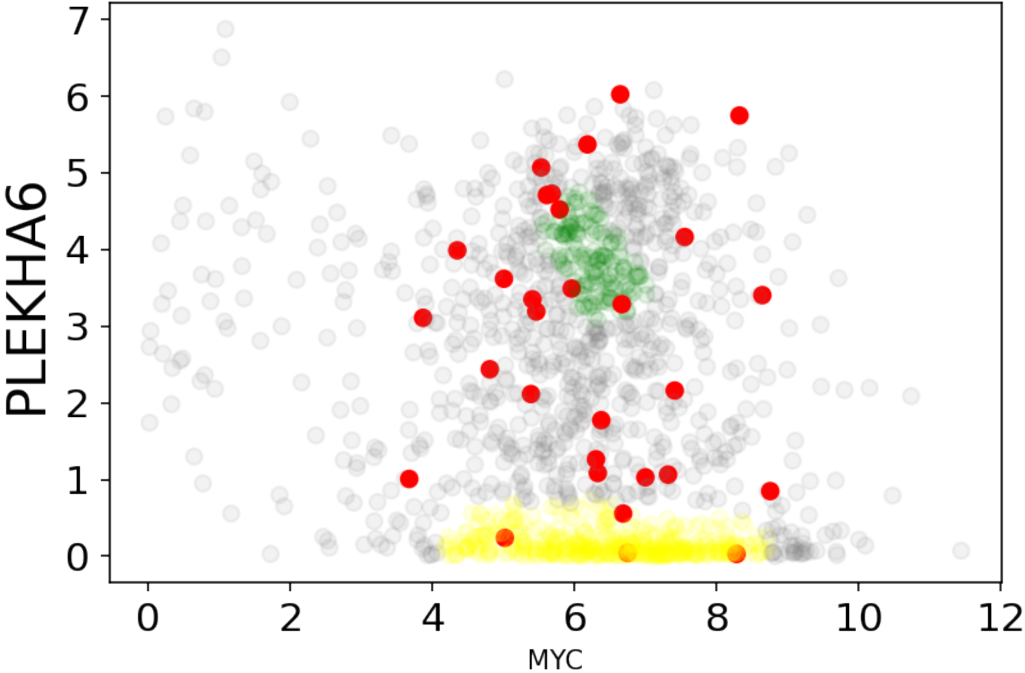

PLEKHA6
MYC
0
1
2
3
4
5
6
7
0
2
4
6
8
10
12

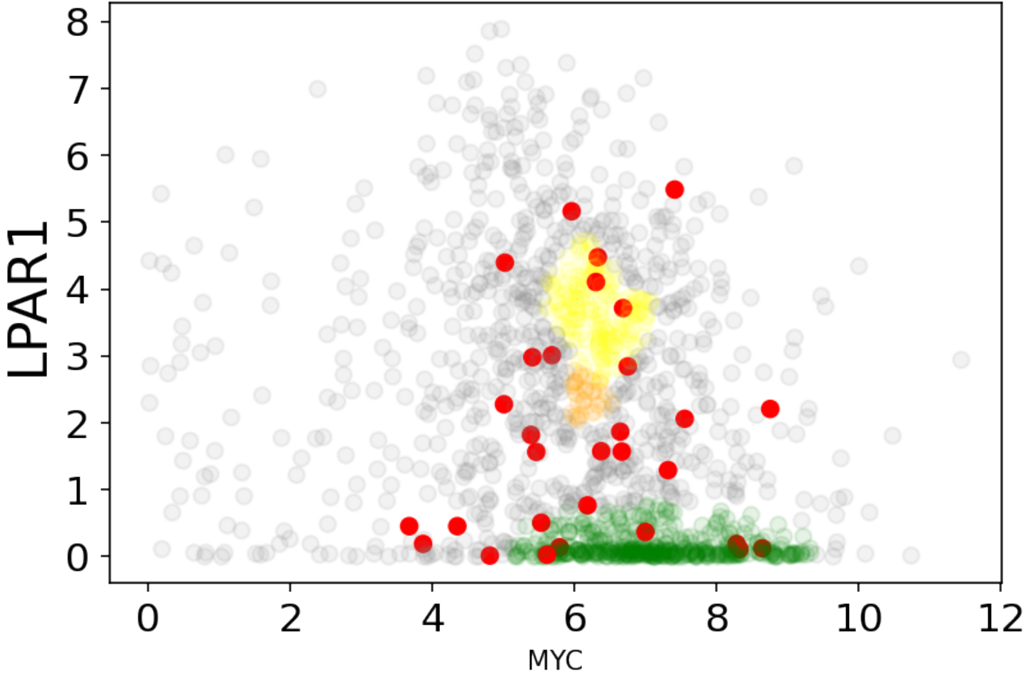

LPAR1
MYC
0
1
2
3
4
5
6
7
8
0
2
4
6
8
10
12

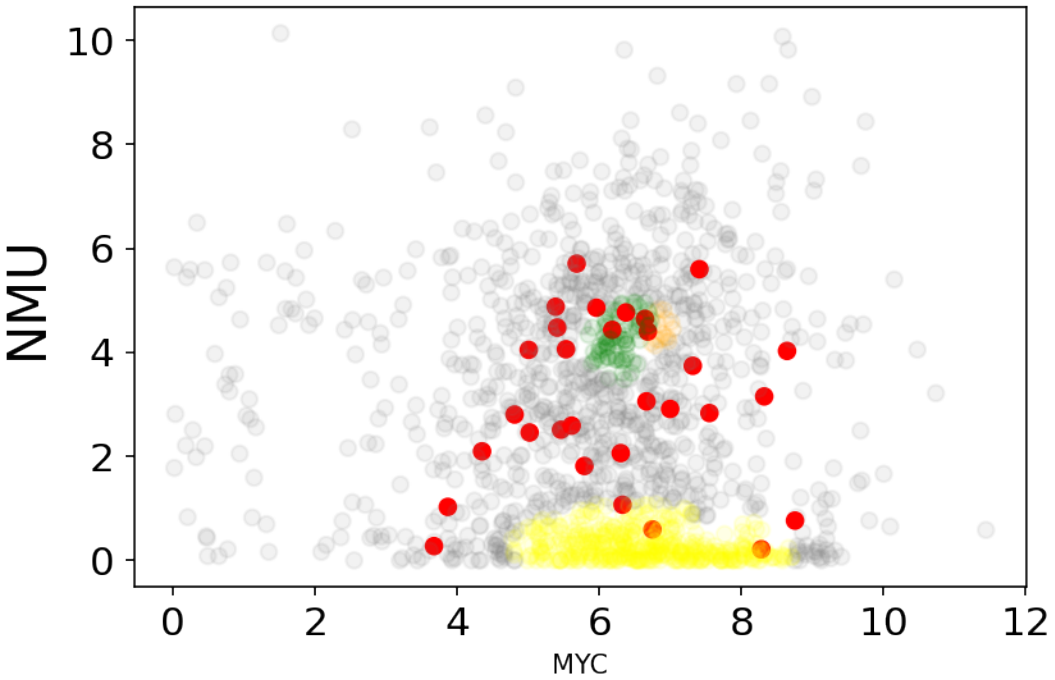
NMU
MYC
0
2
4
6
8
10
12

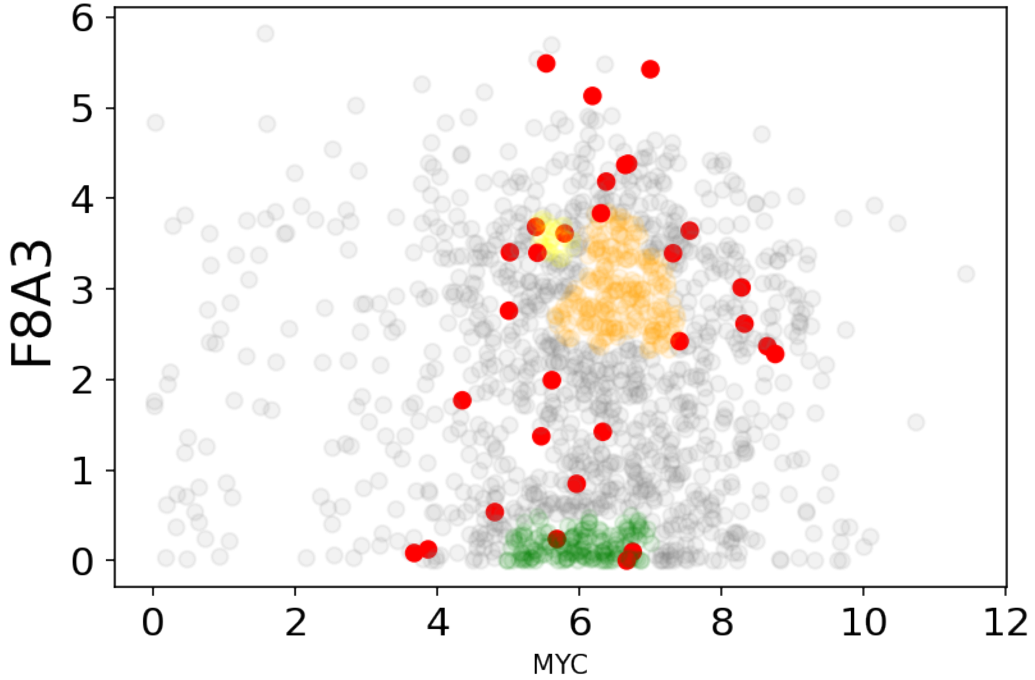

F8A3
MYC
0
1
2
3
4
5
6
0
2
4
6
8
10
12

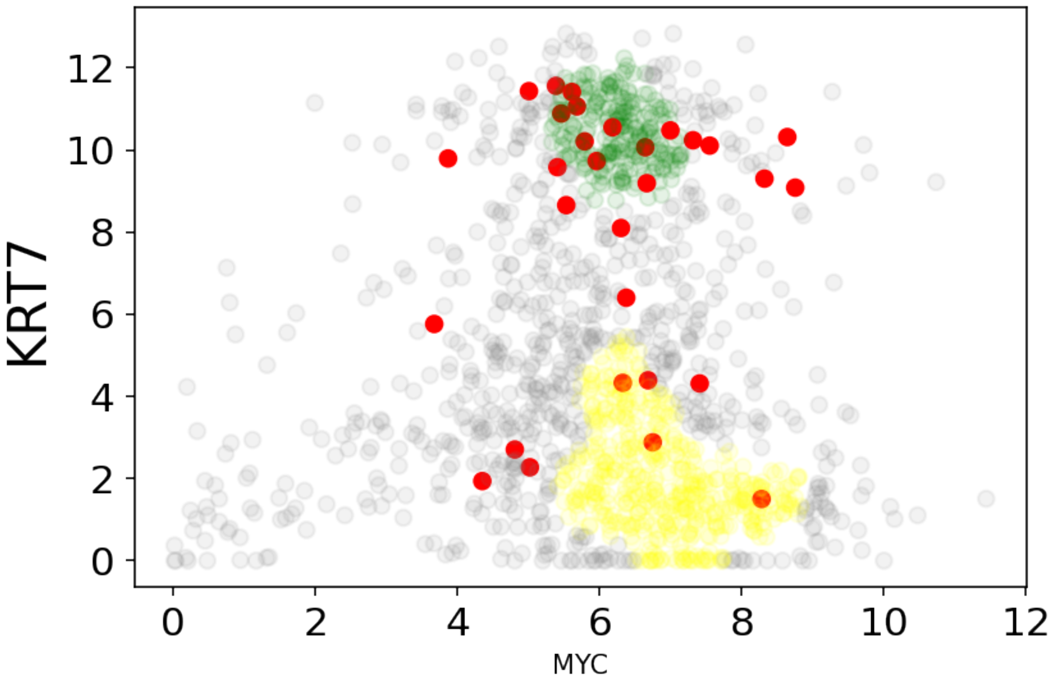

KRT7
MYC
0
2
4
6
8
10
12

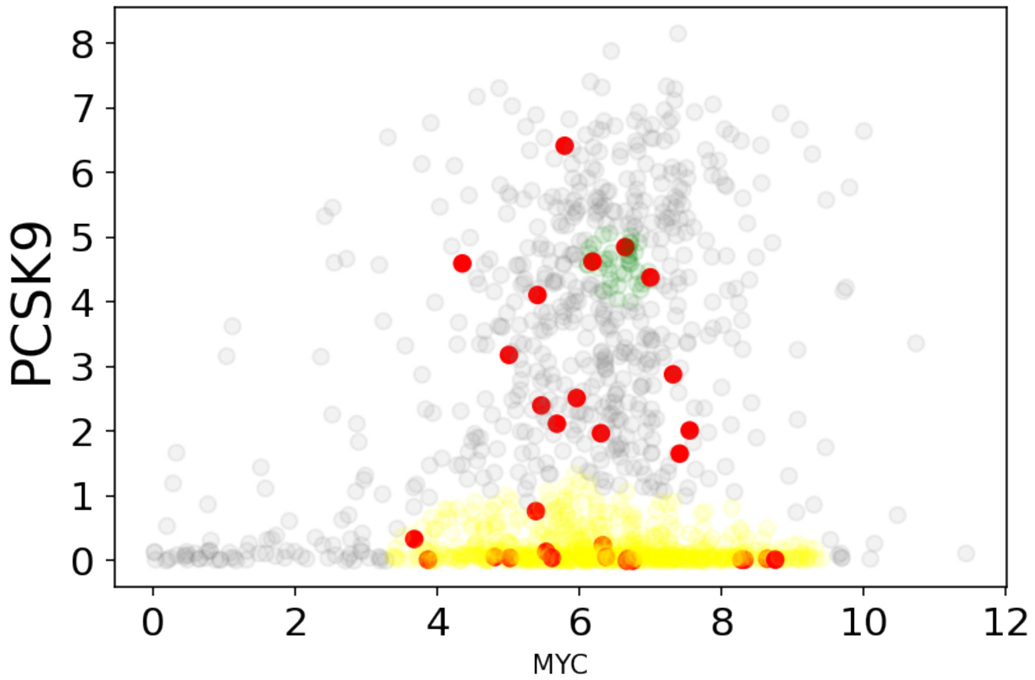
PCSK9
8
7
6
5
4
3
2
1
0
0
2
4
6
8
10
12
MYC

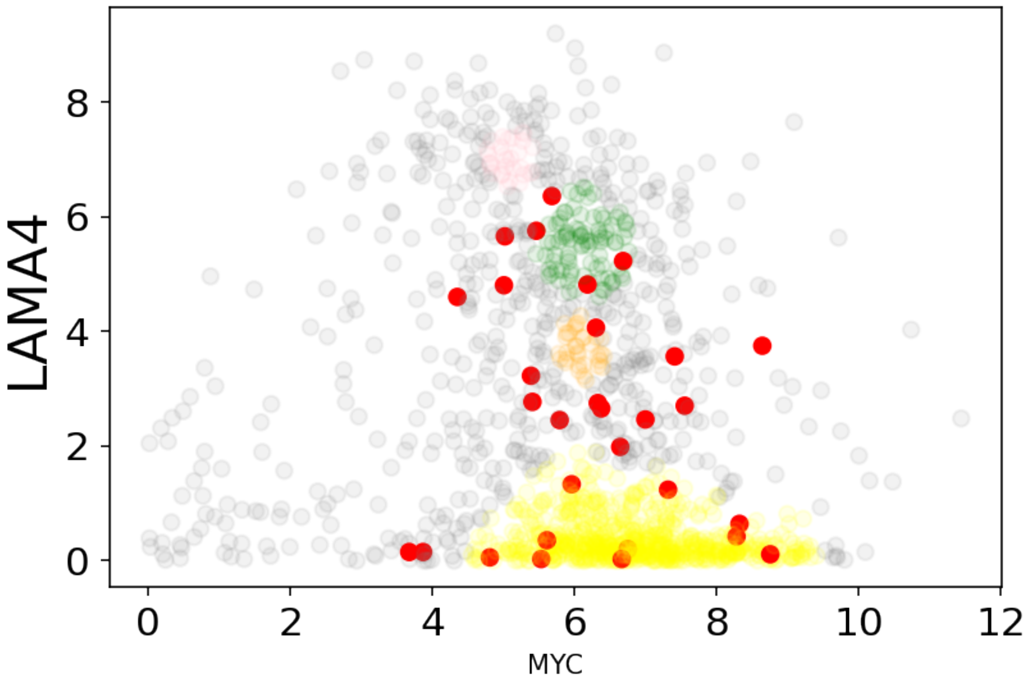

LAMA4
0
2
4
6
8
0
2
4
6
8
10
12
MYC

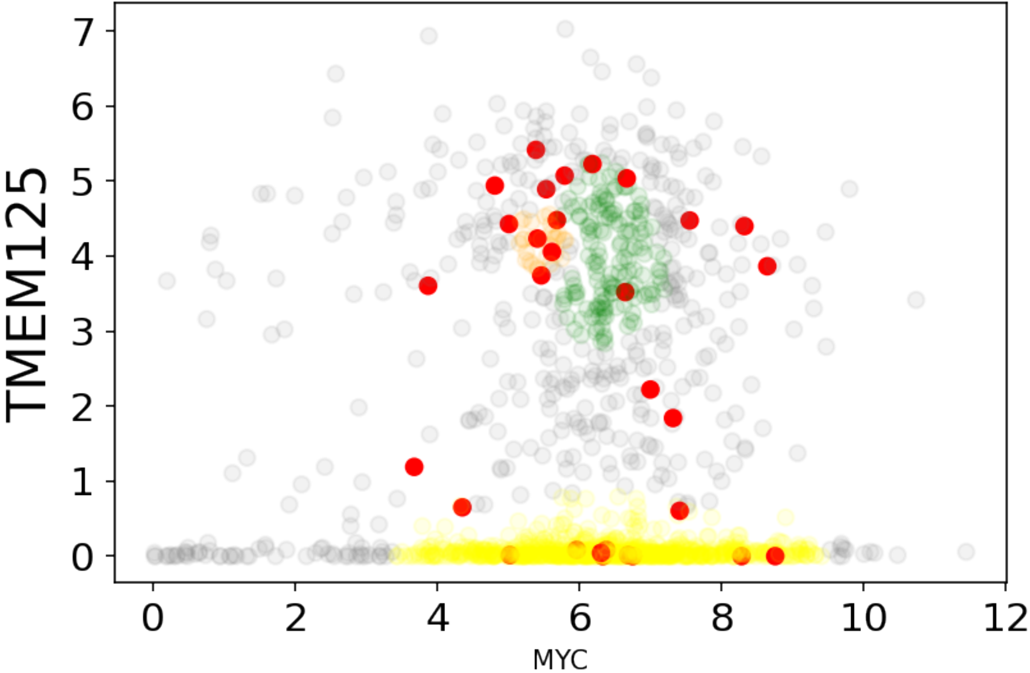

TMEM125
7
6
5
4
3
2
1
0
0
2
4
6
8
10
12
MYC

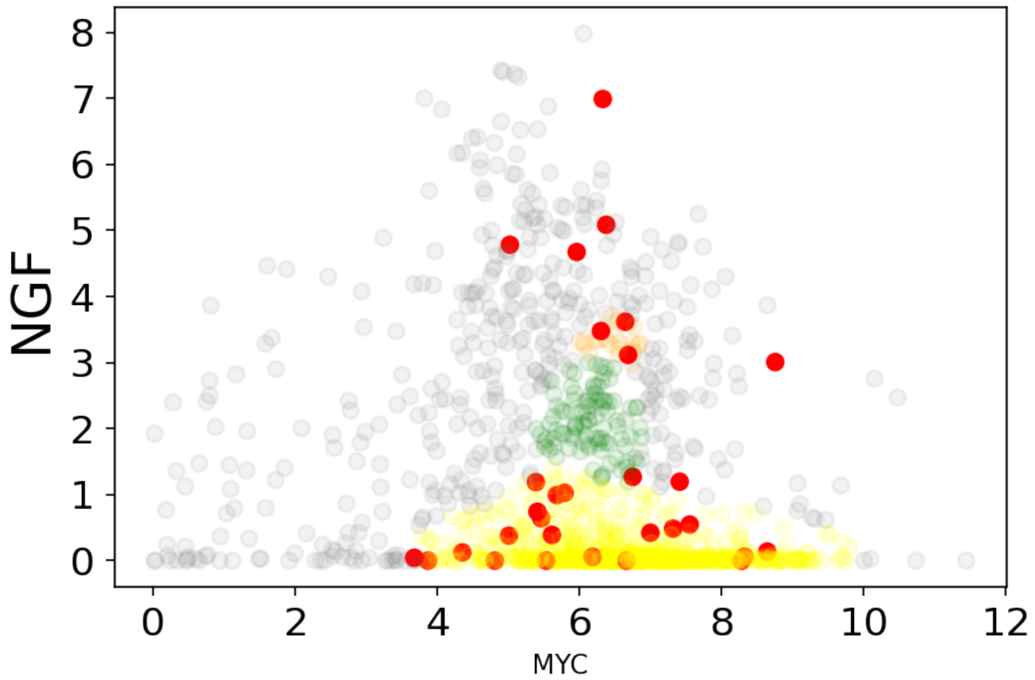
NGF
8
7
6
5
4
3
2
1
0
0
2
4
6
8
10
12
MYC

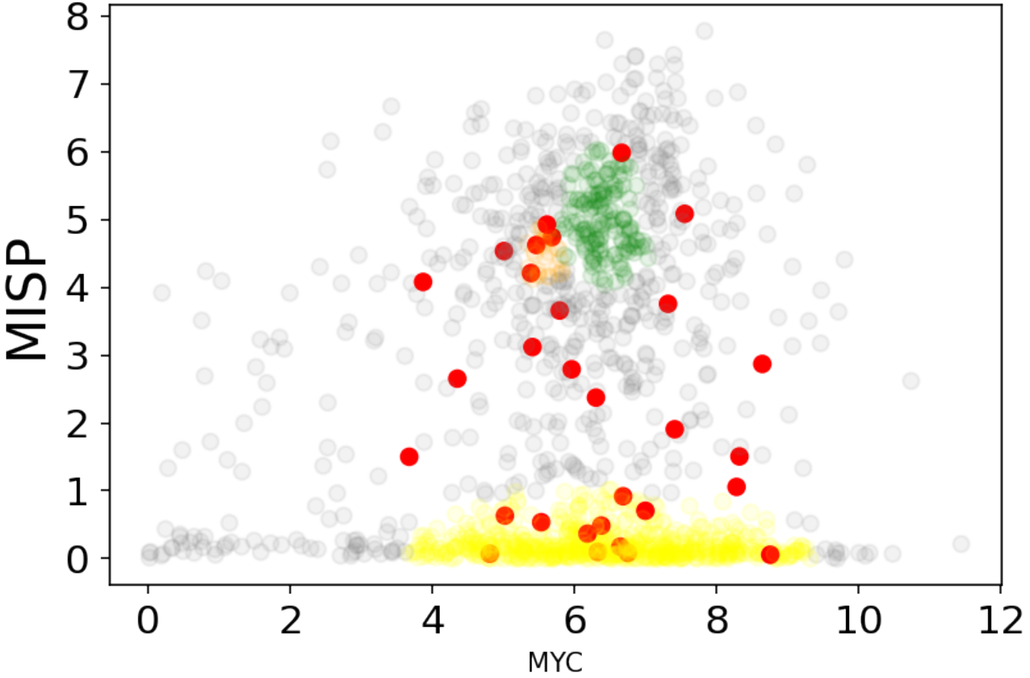

MISP
MYC
0
1
2
3
4
5
6
7
8
0
2
4
6
8
10
12

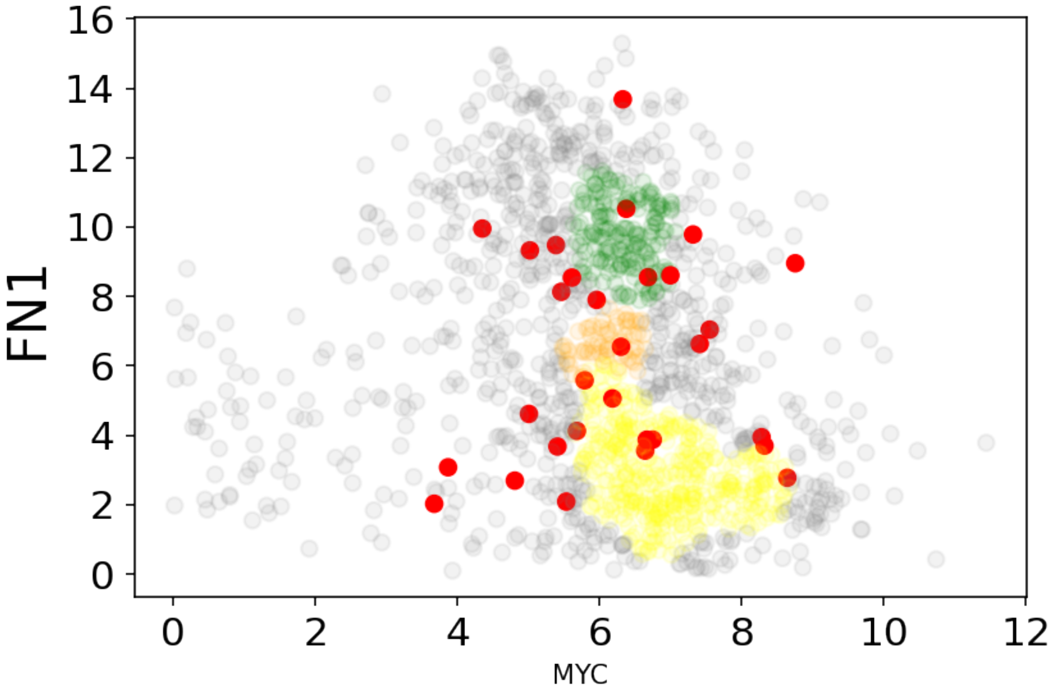
FN1
MYC
0
2
4
6
8
10
12
14
16
0
2
4
6
8
10
12

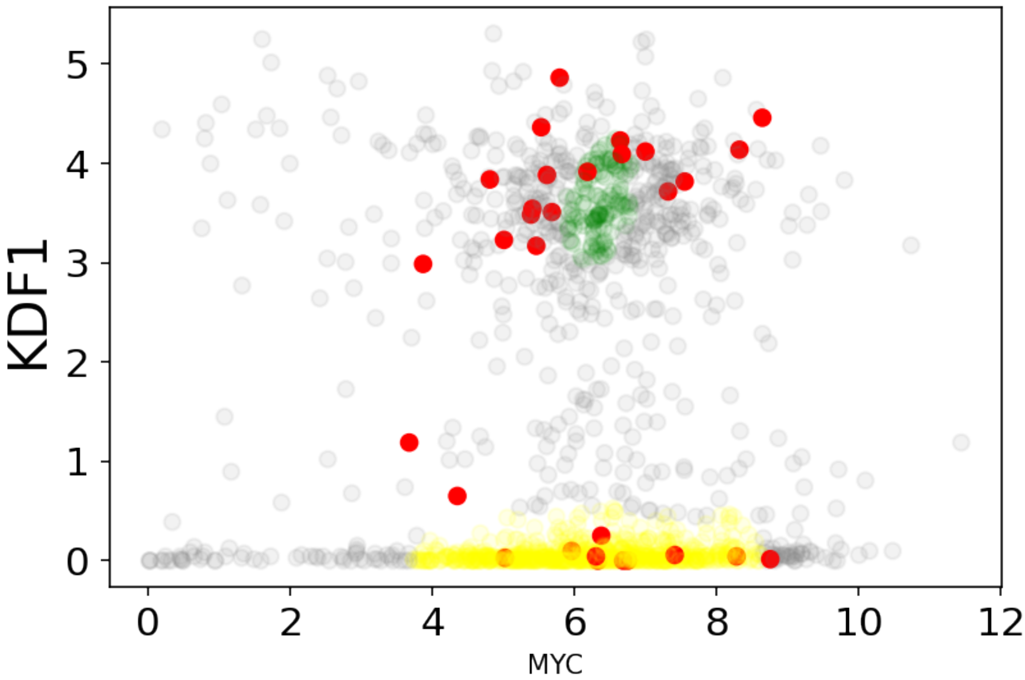

KDF1
5
4
3
2
1
0
0
2
4
6
8
10
12
MYC

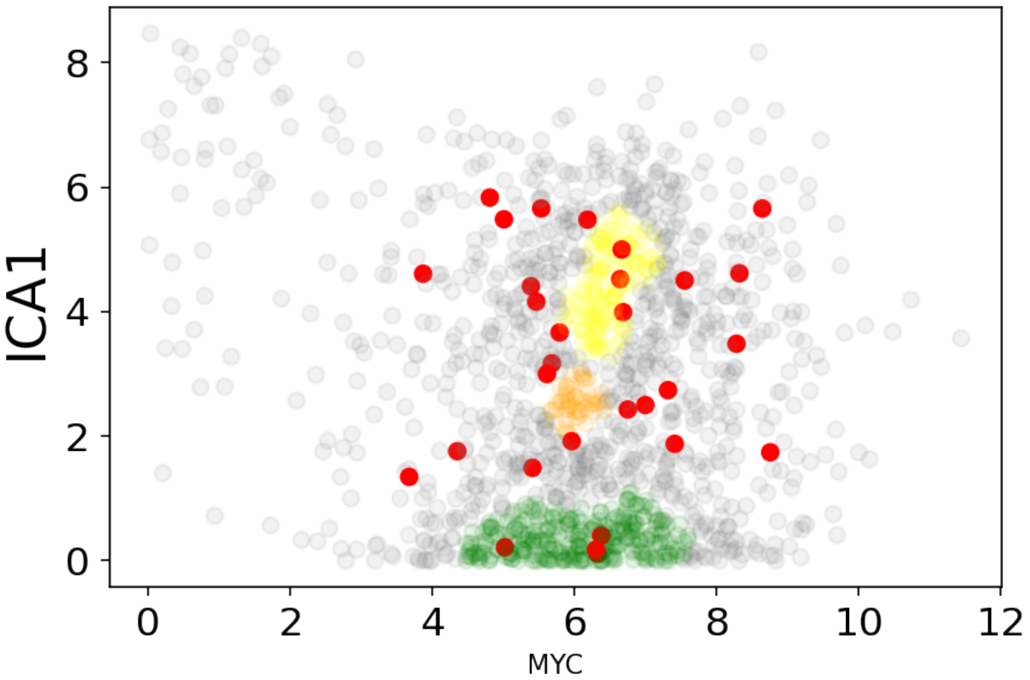

ICA1
8
6
4
2
0
0
2
4
6
8
10
12
MYC

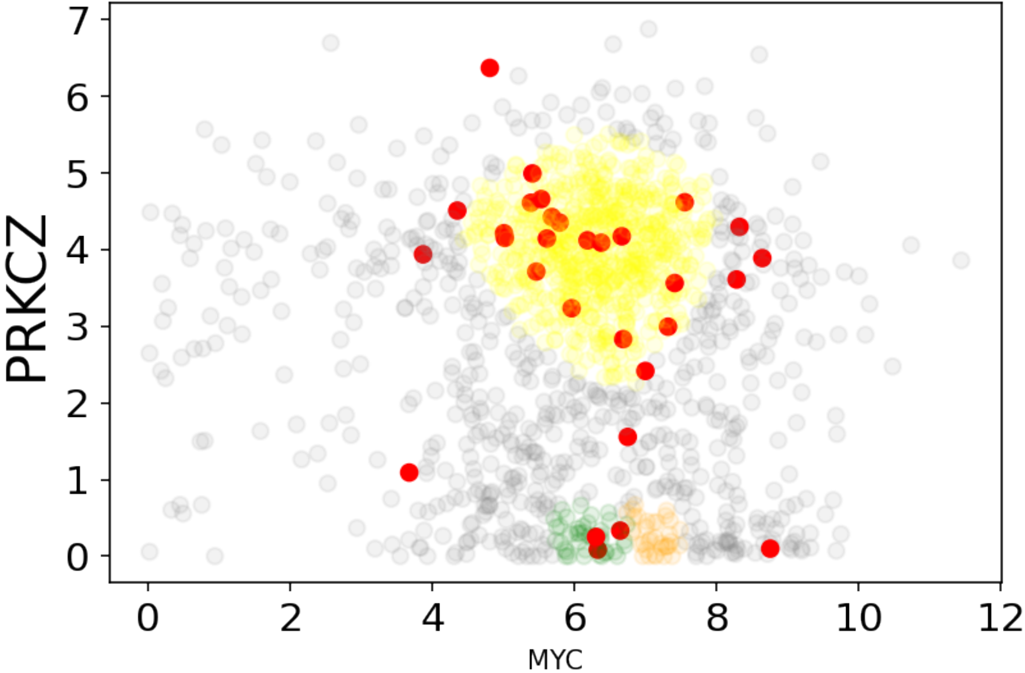

PRKCZ
7
6
5
4
3
2
1
0
0
2
4
6
8
10
12
MYC

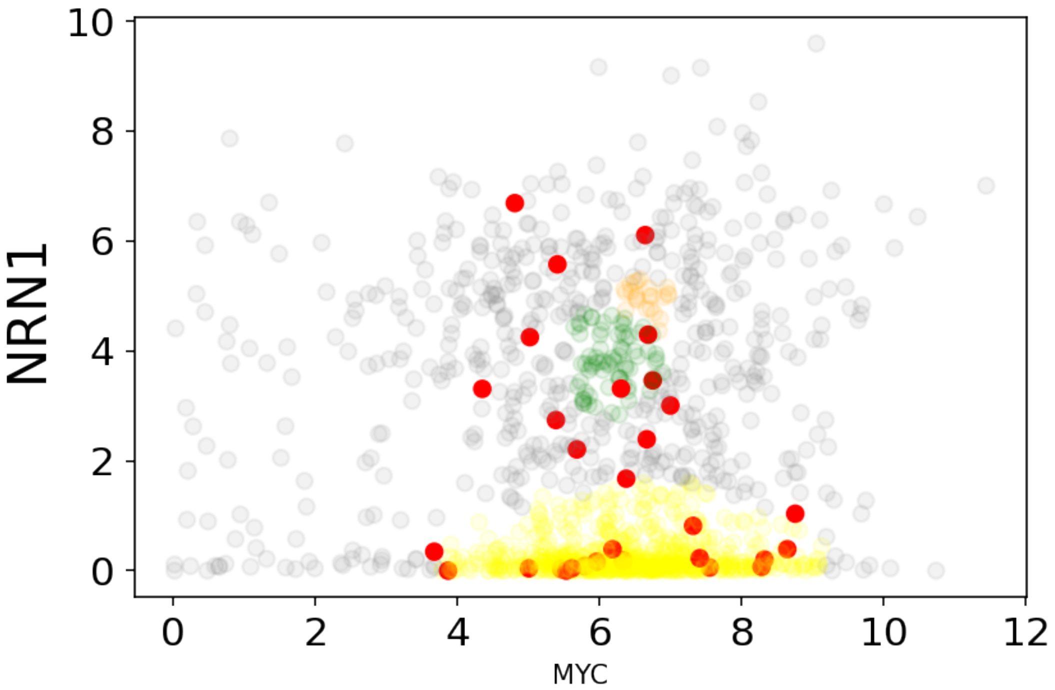

NRN1
MYC
0
2
4
6
8
10
12

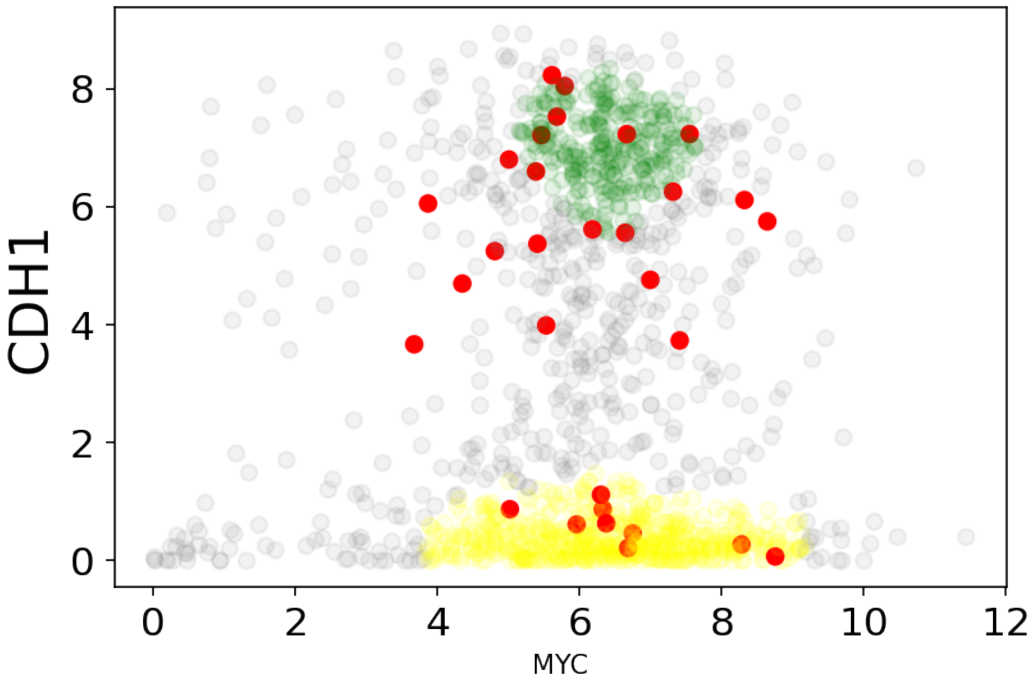

CDH1
MYC
0
2
4
6
8
10
12

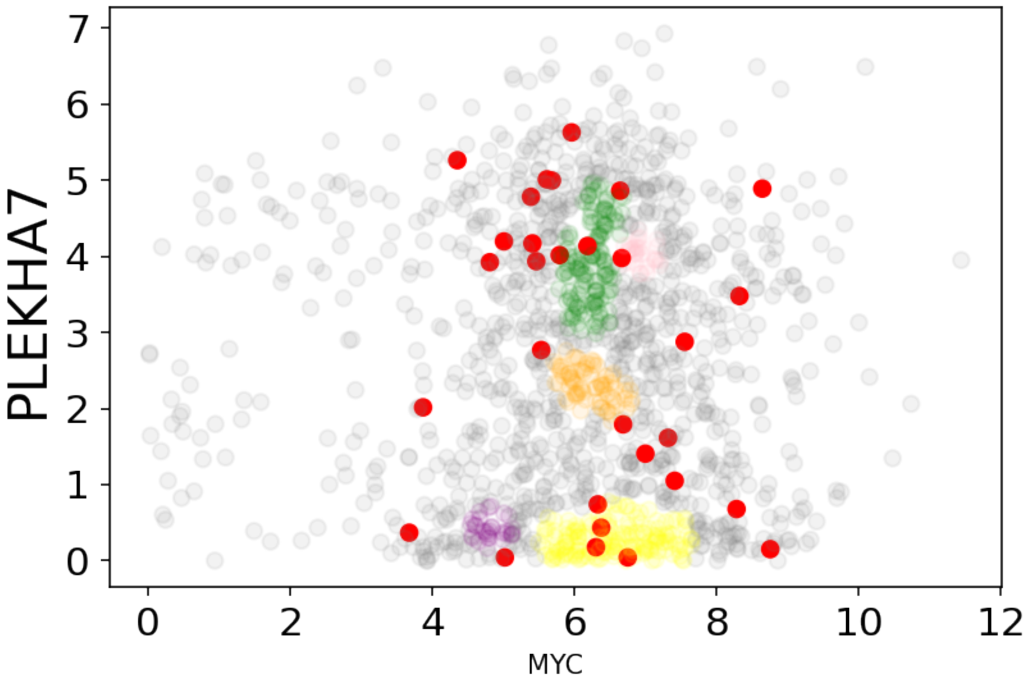

PLEKHA7
MYC
0
1
2
3
4
5
6
7
0
2
4
6
8
10
12

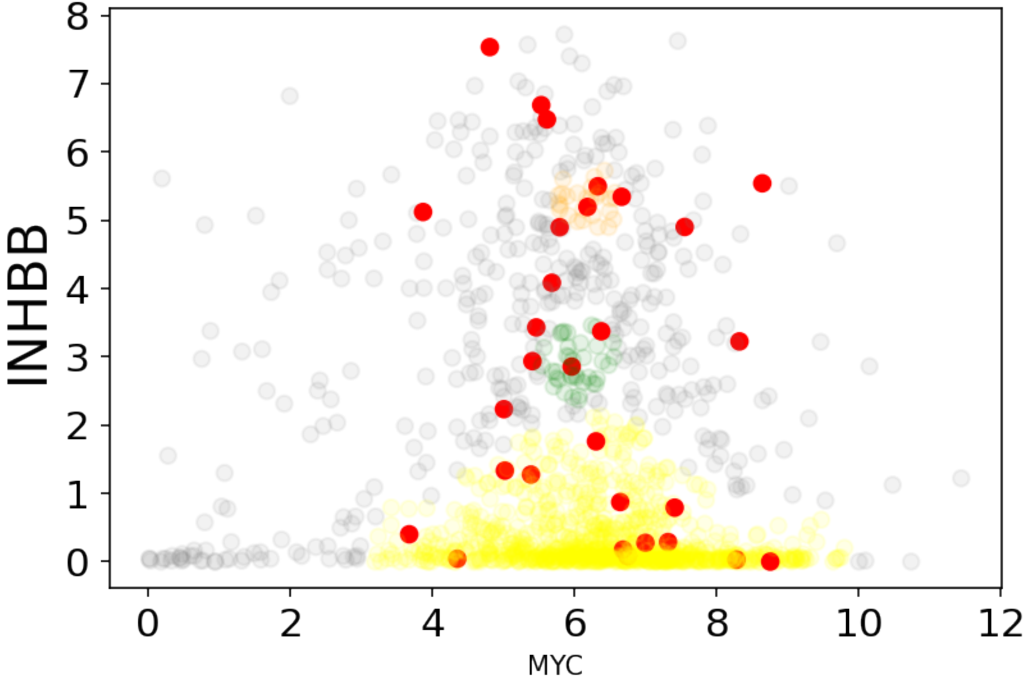
INHBB
8
7
6
5
4
3
2
1
0
0
2
4
6
8
10
12
MYC

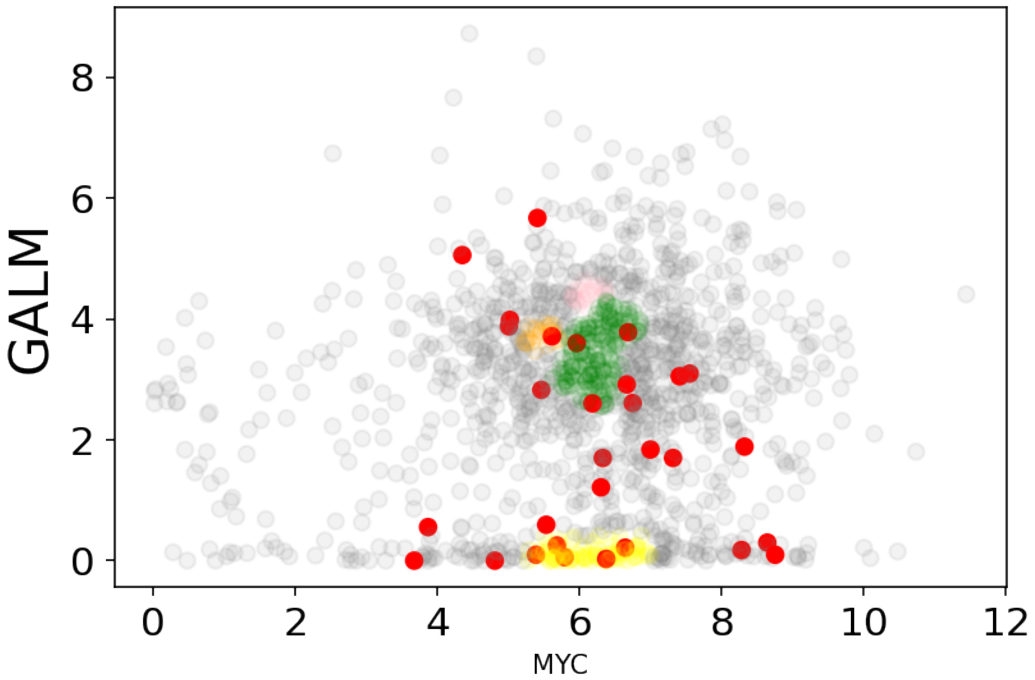

GALM
MYC
0
2
4
6
8
10
12

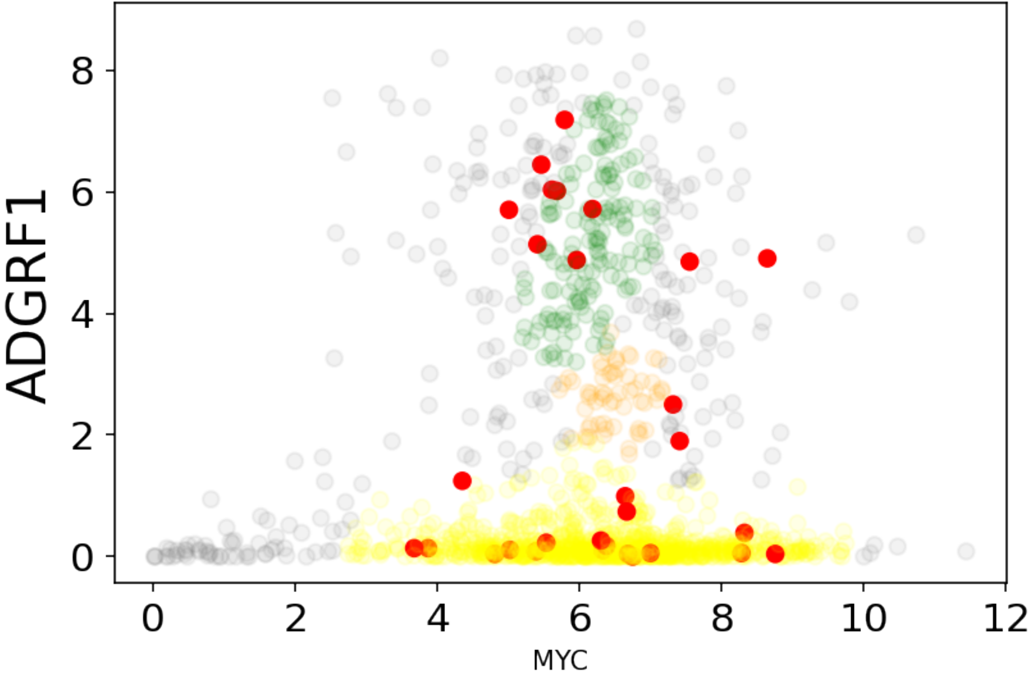
ADGRF1
MYC
0
2
4
6
8
10
12

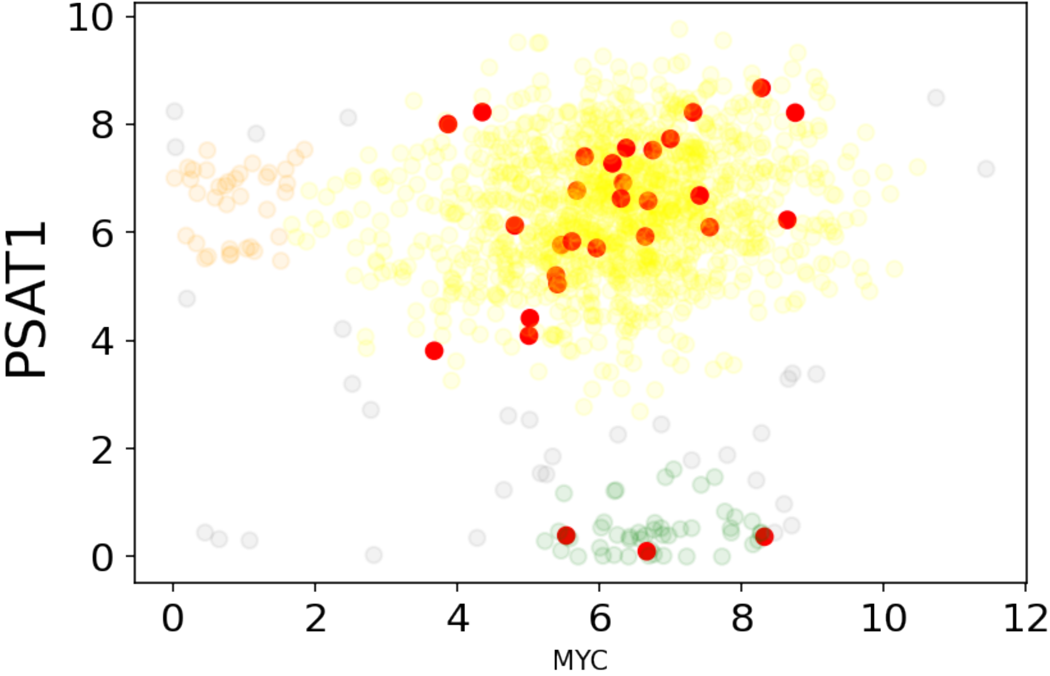

PSAT1
10
8
6
4
2
0
0
2
4
6
8
10
12
MYC

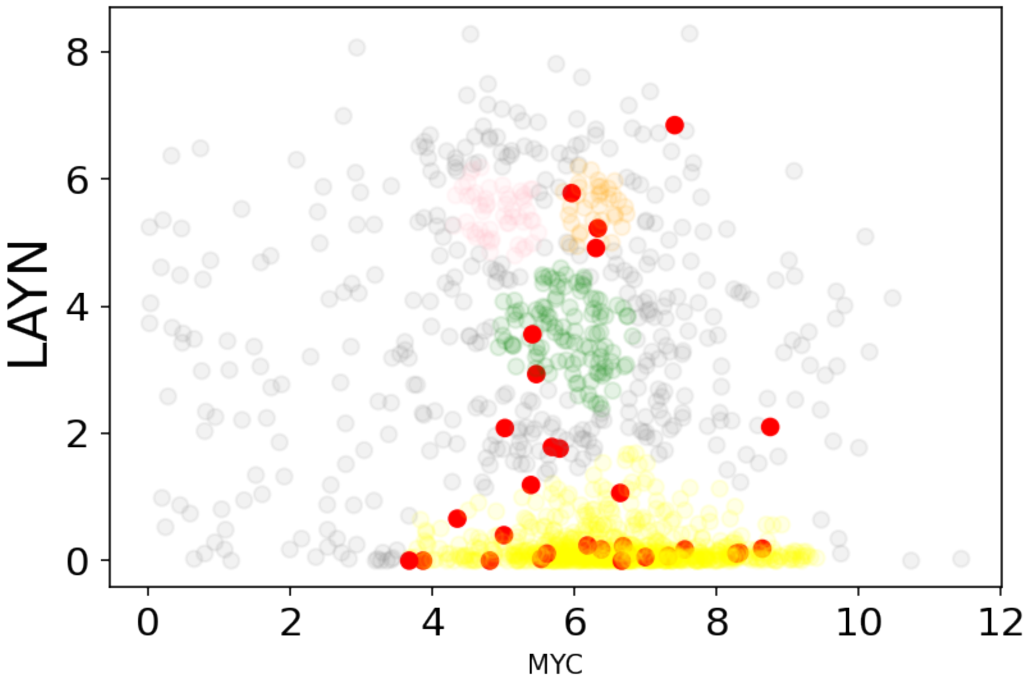
LAYN
8
6
4
2
0
0
2
4
6
8
10
12
MYC

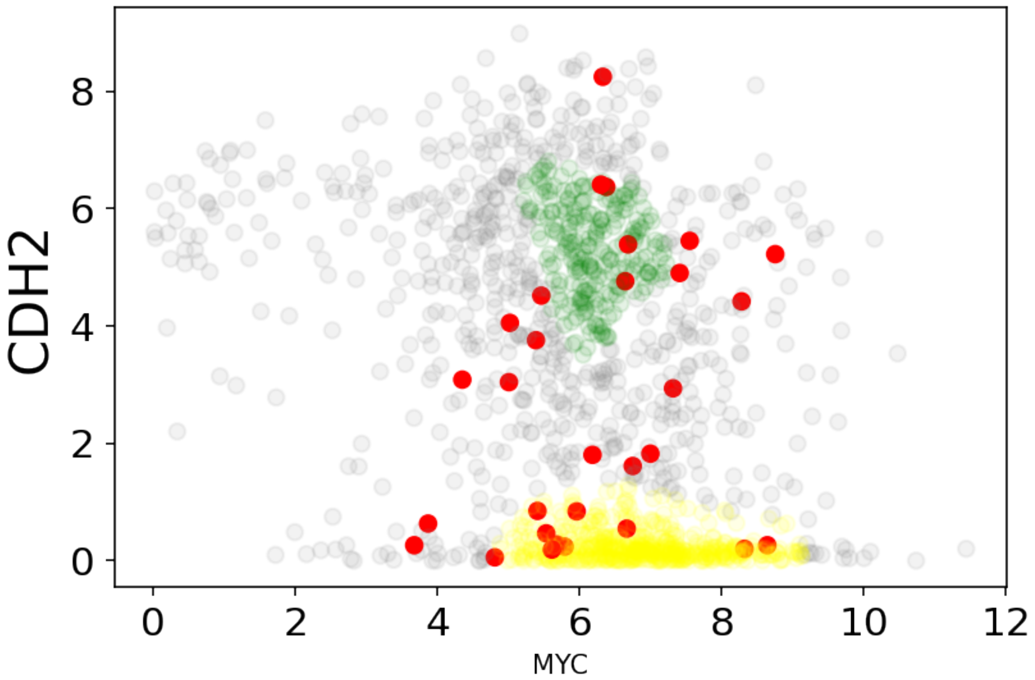

CDH2
MYC
0
2
4
6
8
10
12

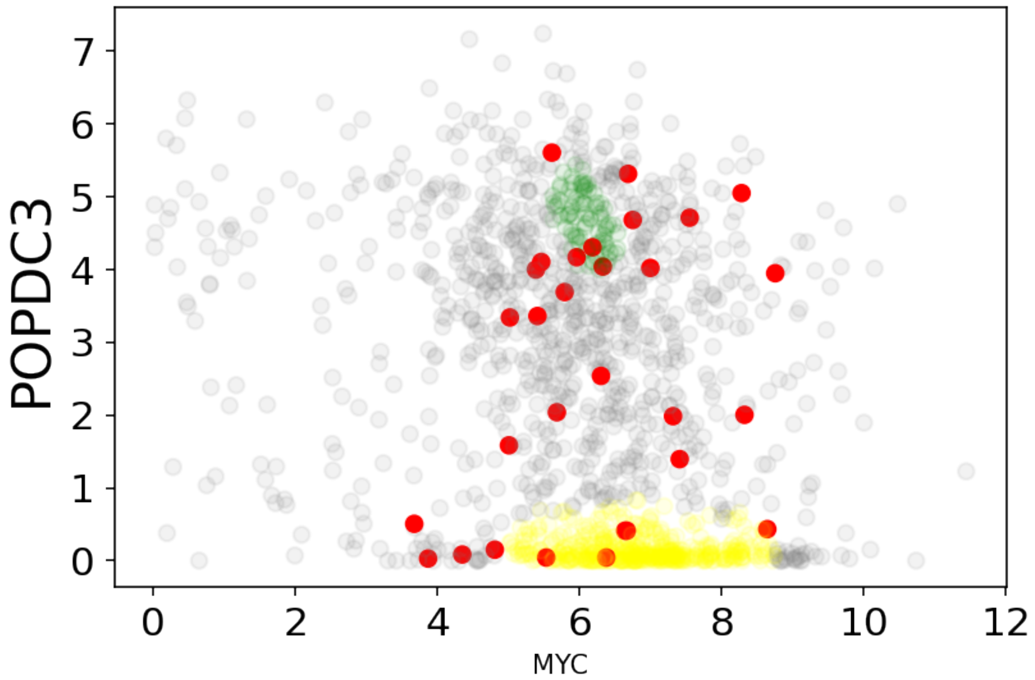

POPDC3
MYC
0
1
2
3
4
5
6
7
0
2
4
6
8
10
12

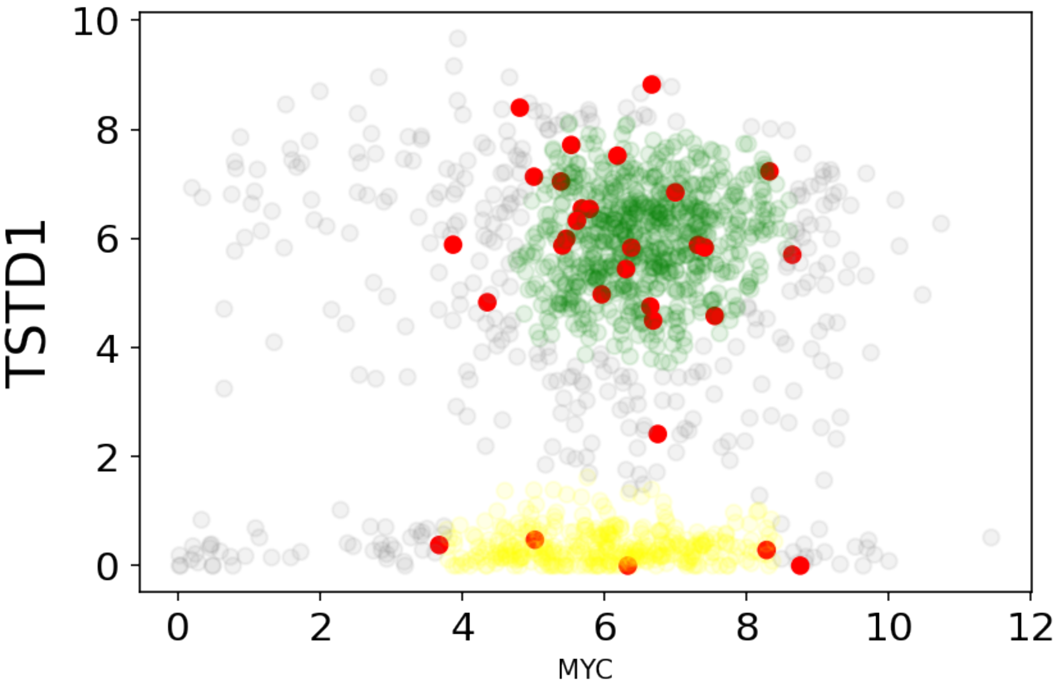
TSTD1
MYC
0
2
4
6
8
10
12
0
2
4
6
8
10

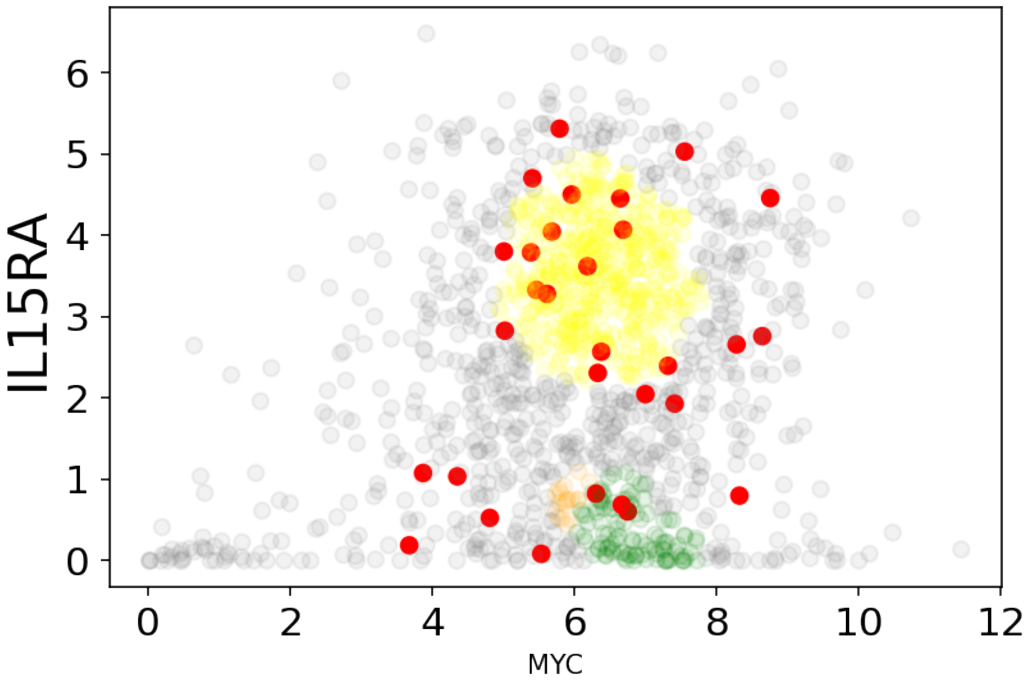
IL15RA
6
5
4
3
2
1
0
0
2
4
6
8
10
12
MYC

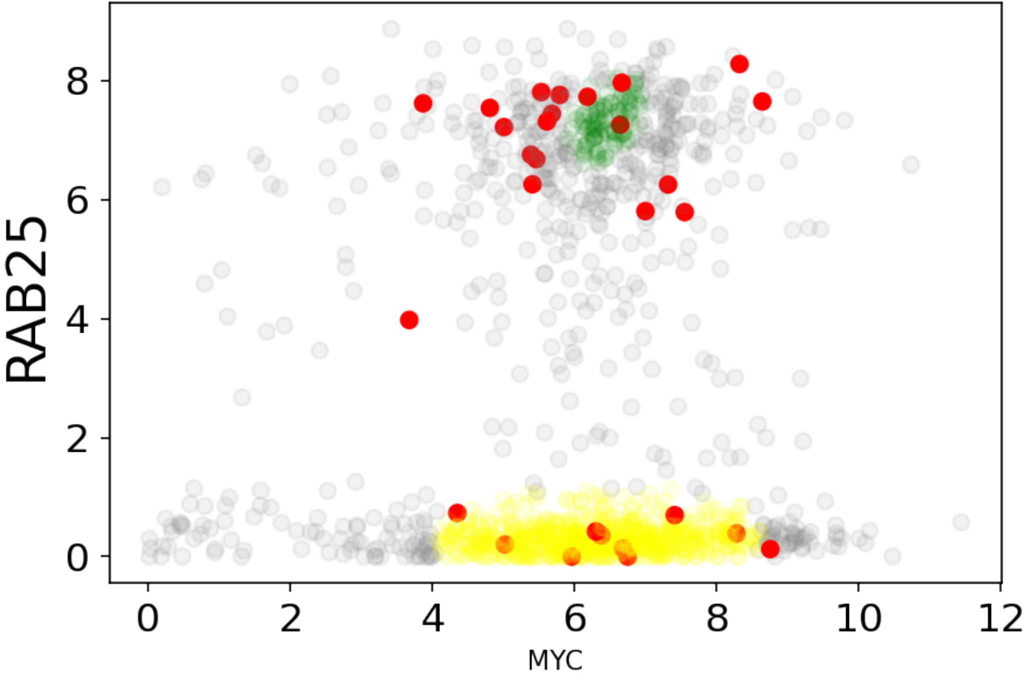

RAB25
0
2
4
6
8
0
2
4
6
8
10
12
MYC

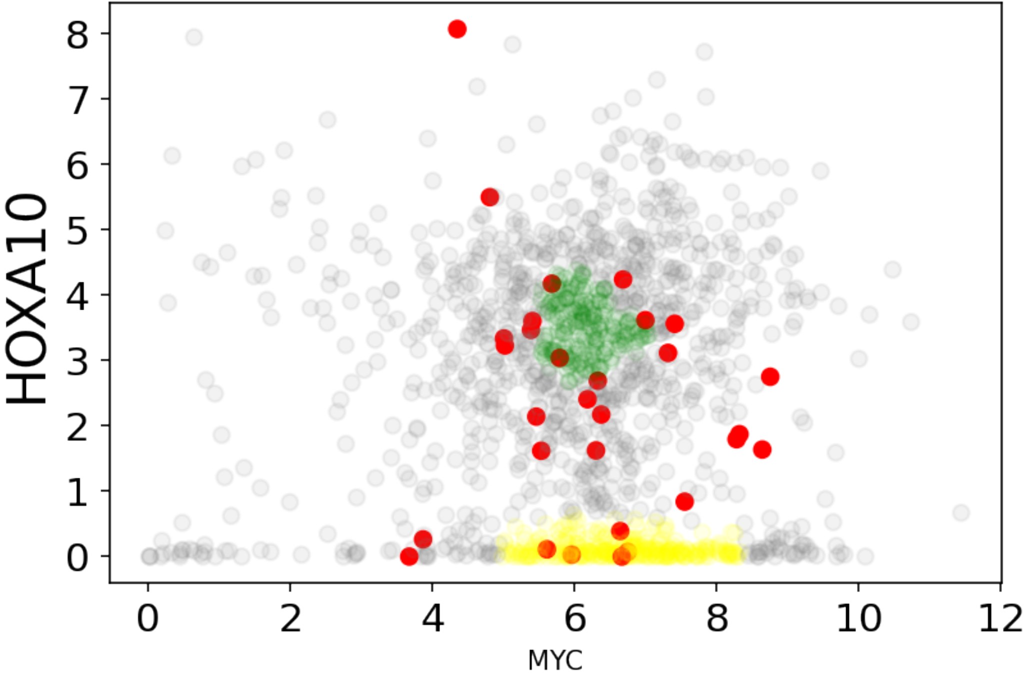

HOXA10
MYC
0
1
2
3
4
5
6
7
8
0
2
4
6
8
10
12

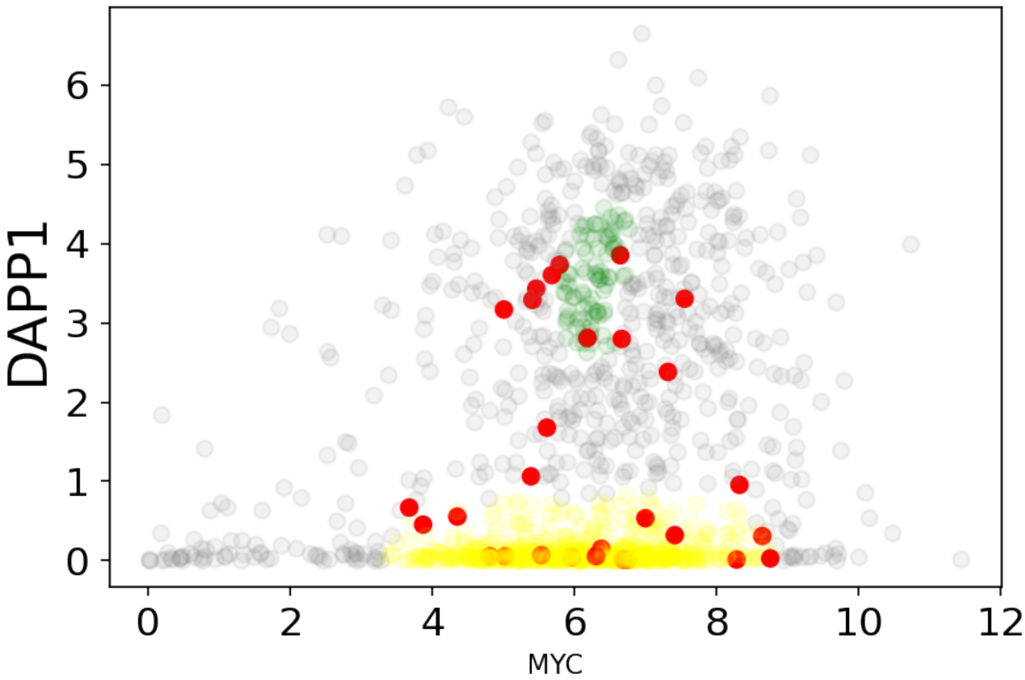
DAPP1
0
1
2
3
4
5
6
0
2
4
6
8
10
12
MYC

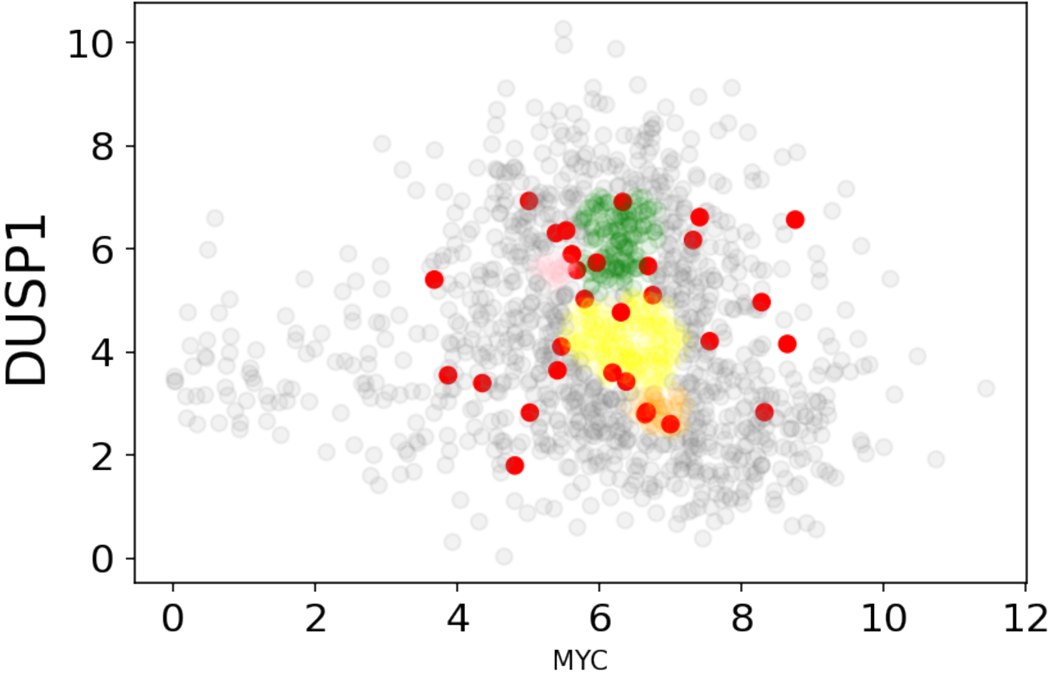

DUSP1
MYC
0
2
4
6
8
10
12

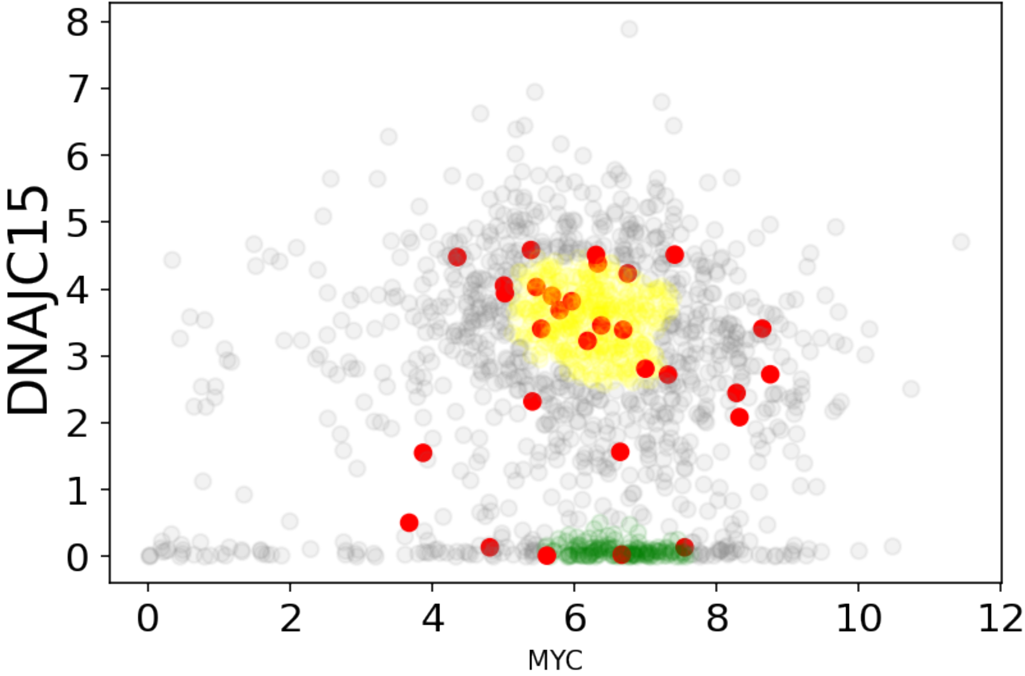

DNAJC15
MYC
0
1
2
3
4
5
6
7
8
0
2
4
6
8
10
12

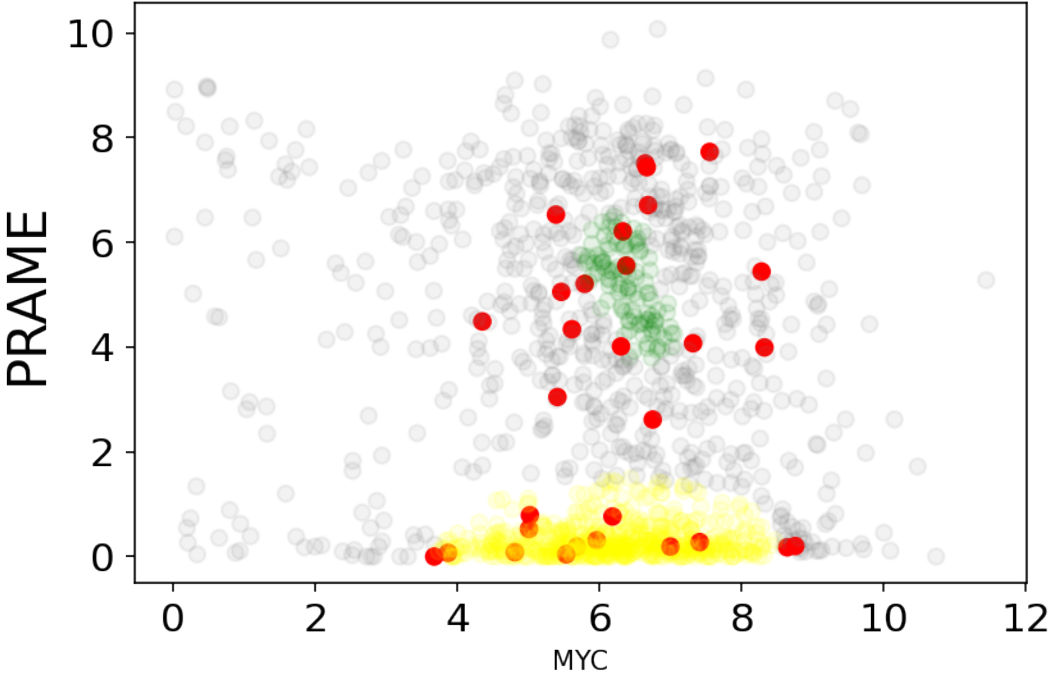

10
8
6
4
2
0
PRAME
0
2
4
6
8
10
12
MYC

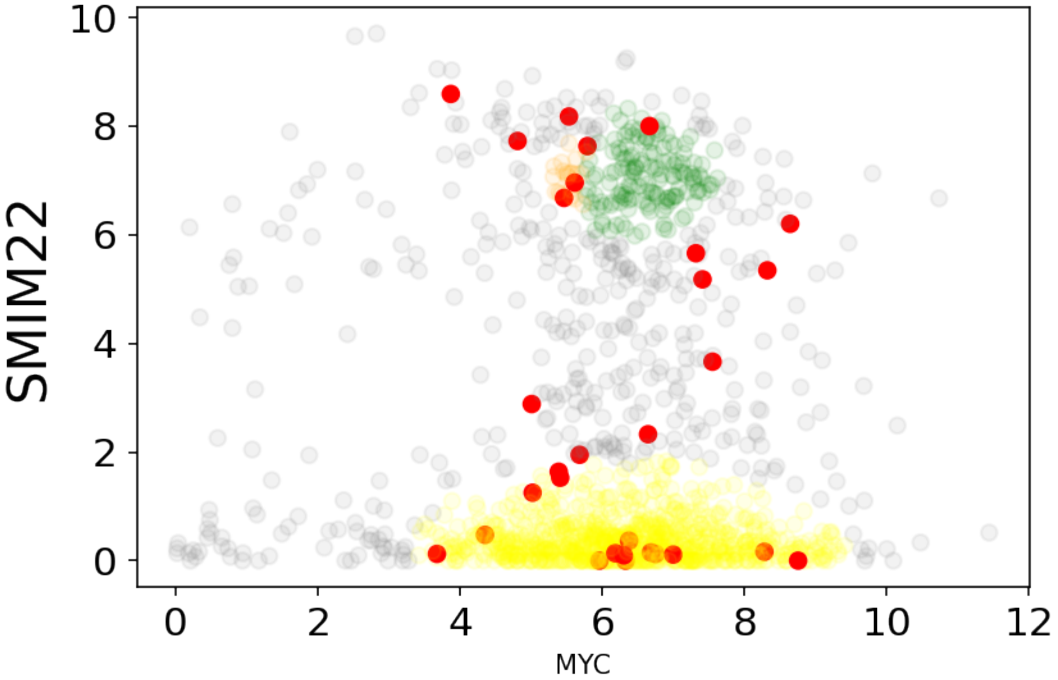

SMIM22
MYC
10
8
6
4
2
0
0
2
4
6
8
10
12

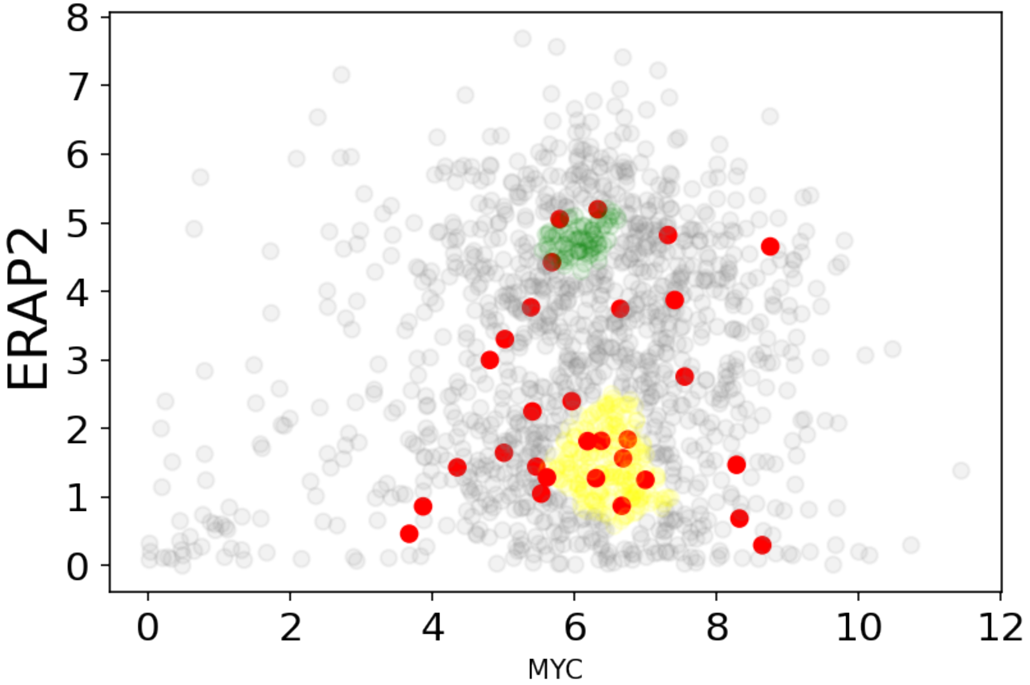

ERAP2
MYC
0
1
2
3
4
5
6
7
8
0
2
4
6
8
10
12

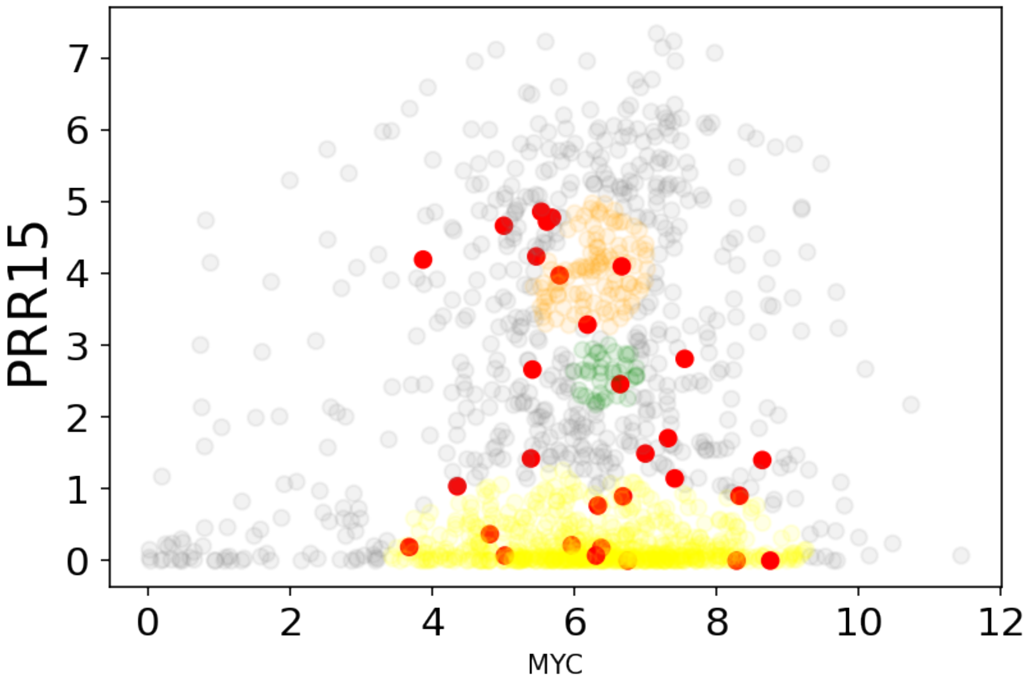
PRR15
7
6
5
4
3
2
1
0
0
2
4
6
8
10
12
MYC

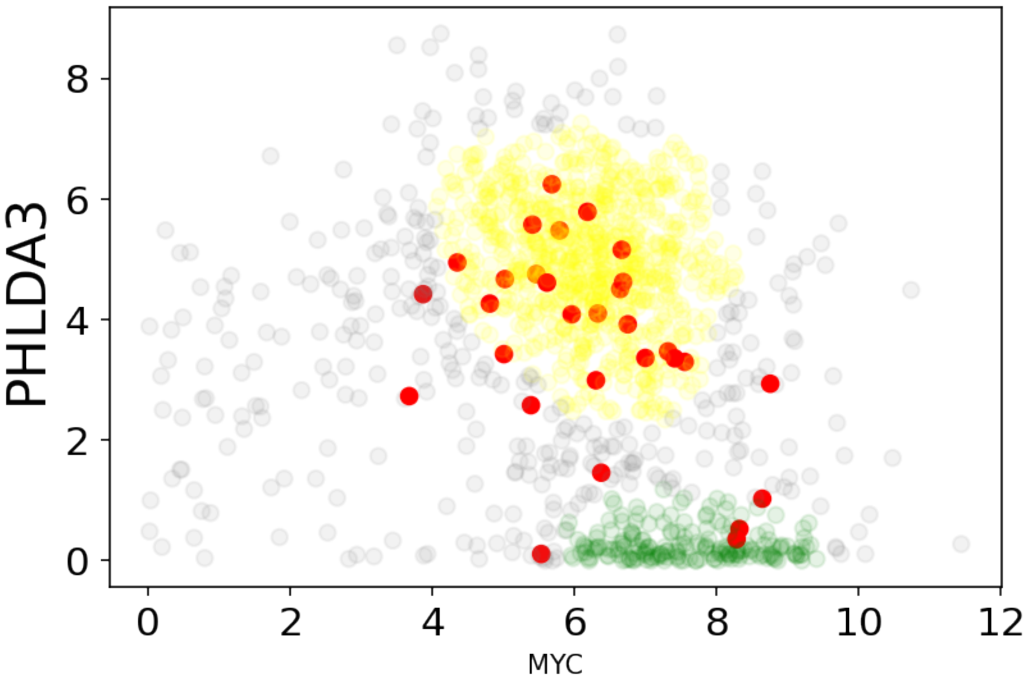

PHLDA3
MYC
0
2
4
6
8
10
12

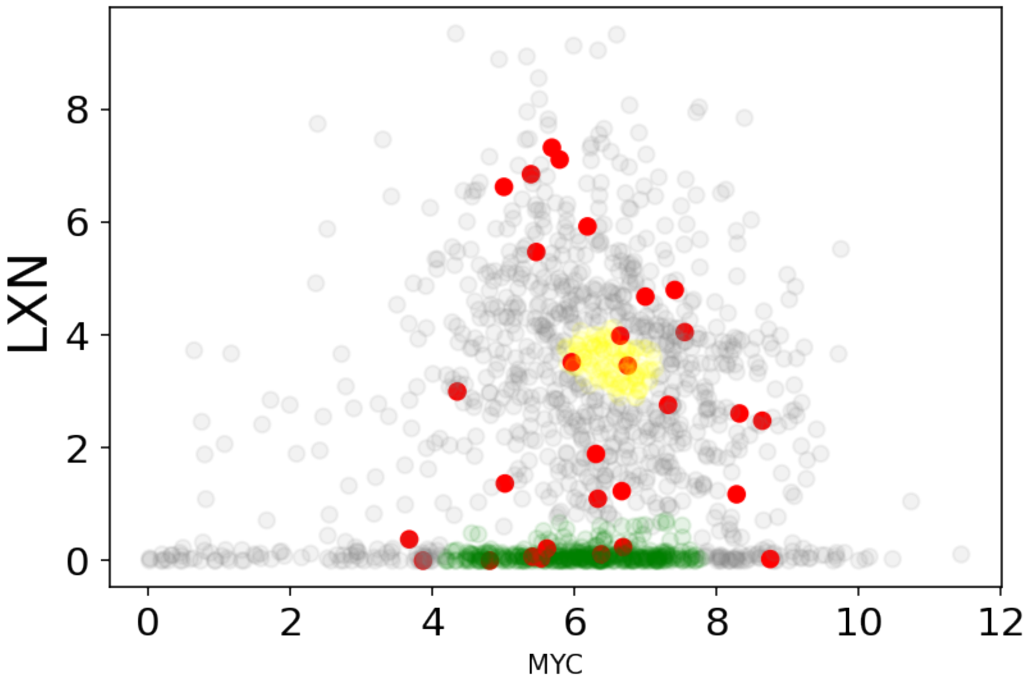

LXN
MYC
0
2
4
6
8
10
12

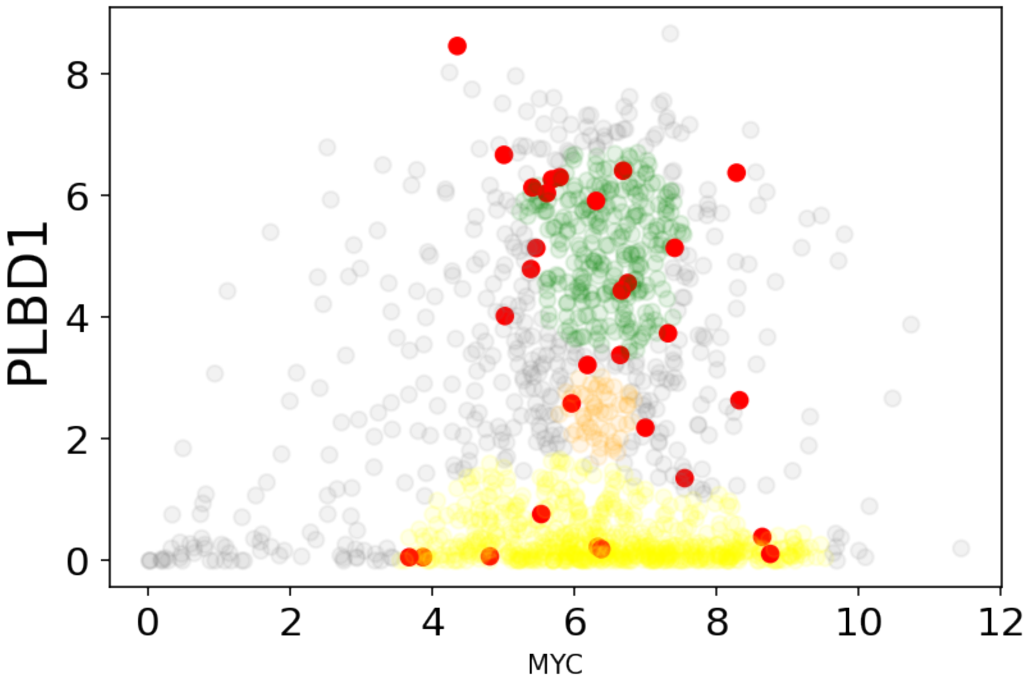
PLBD1
8
6
4
2
0
0
2
4
6
8
10
12
MYC

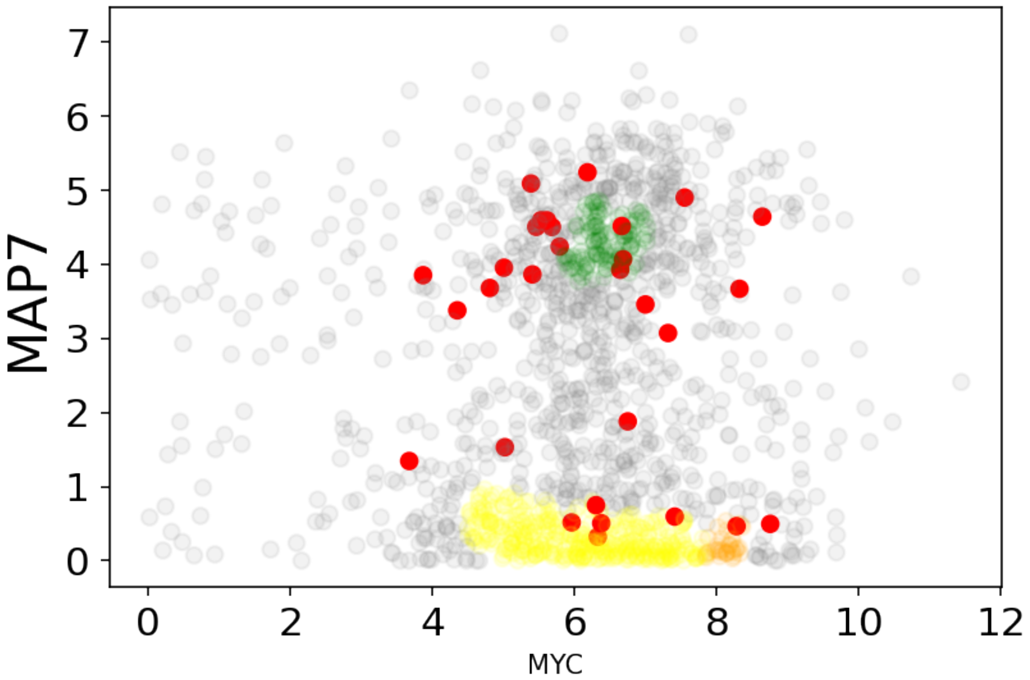

MAP7
MYC
0
1
2
3
4
5
6
7
0
2
4
6
8
10
12

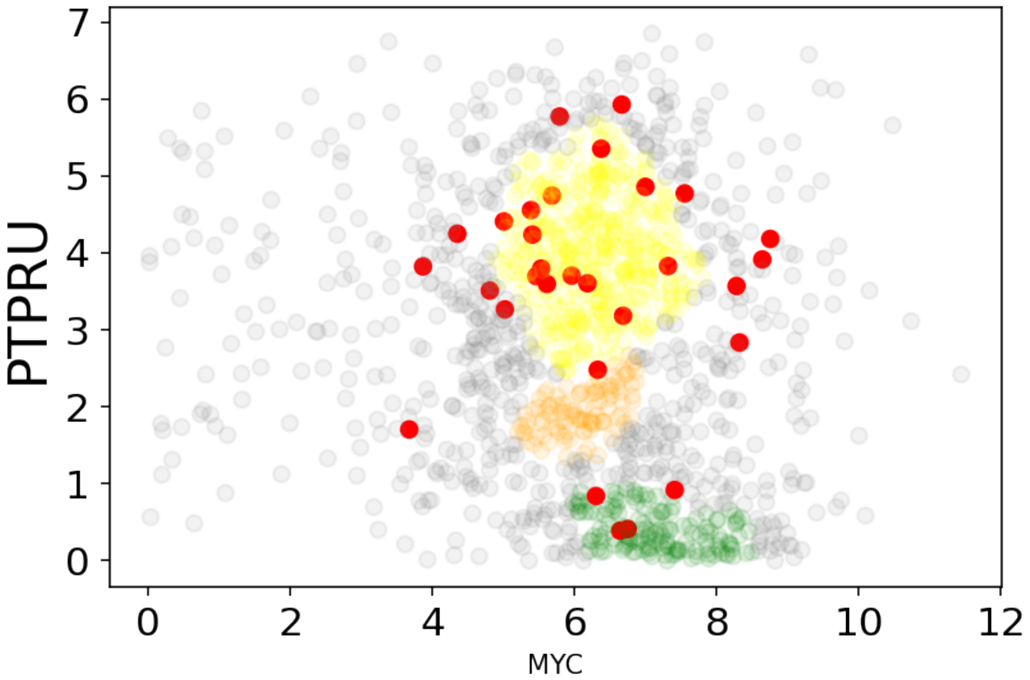
PTPRU
MYC
0
1
2
3
4
5
6
7
0
2
4
6
8
10
12

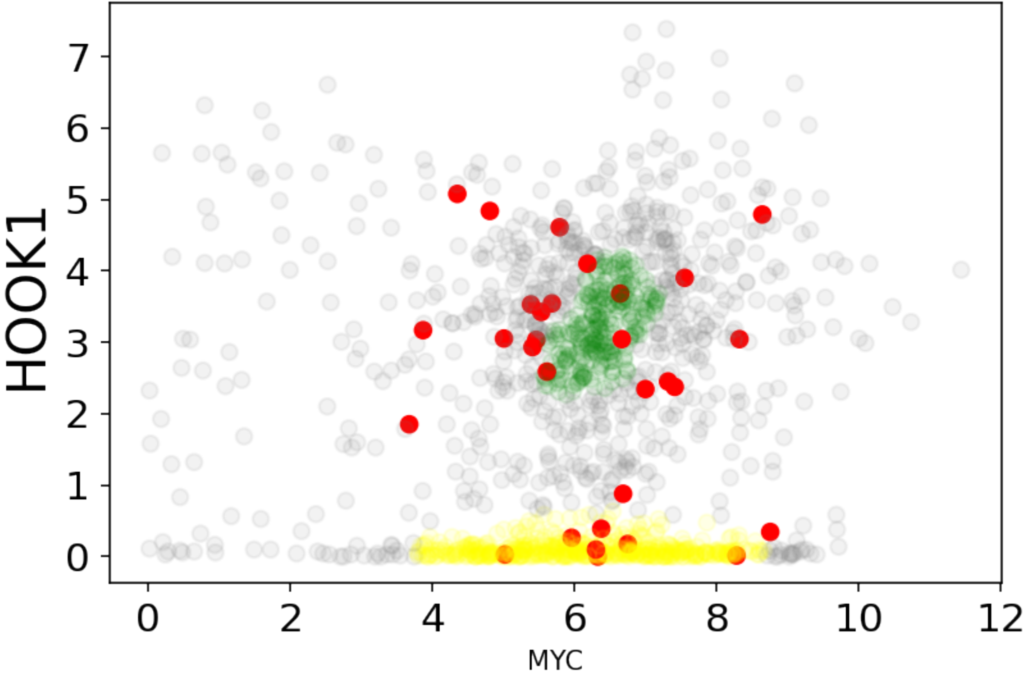

HOOK1
0
1
2
3
4
5
6
7
0
2
4
6
8
10
12
MYC

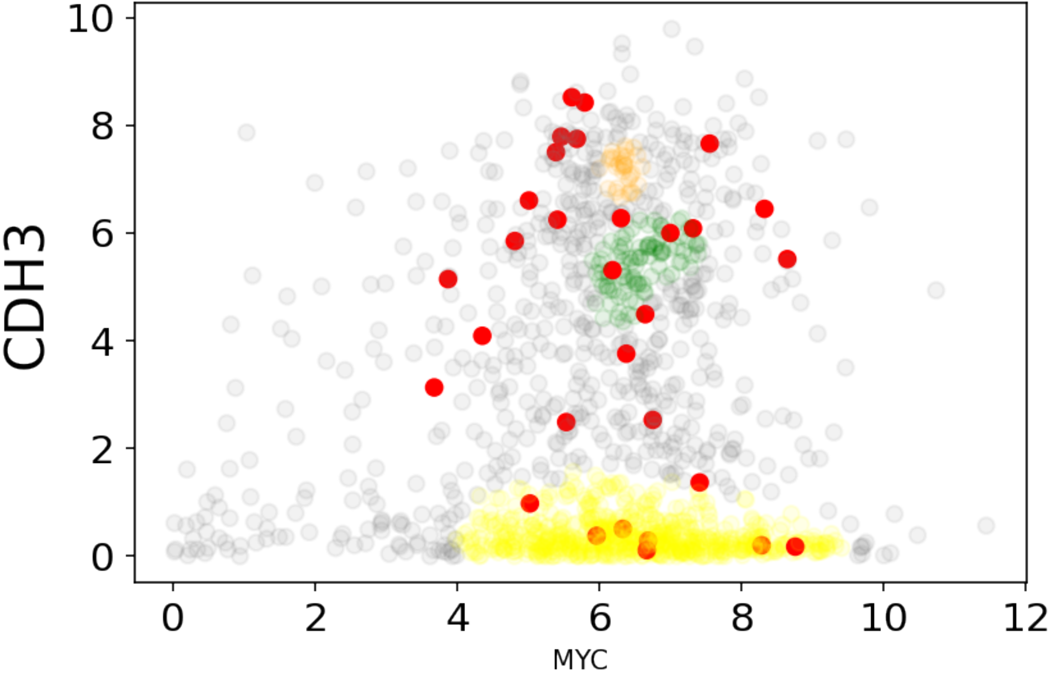

CDH3
MYC
0
2
4
6
8
10
12

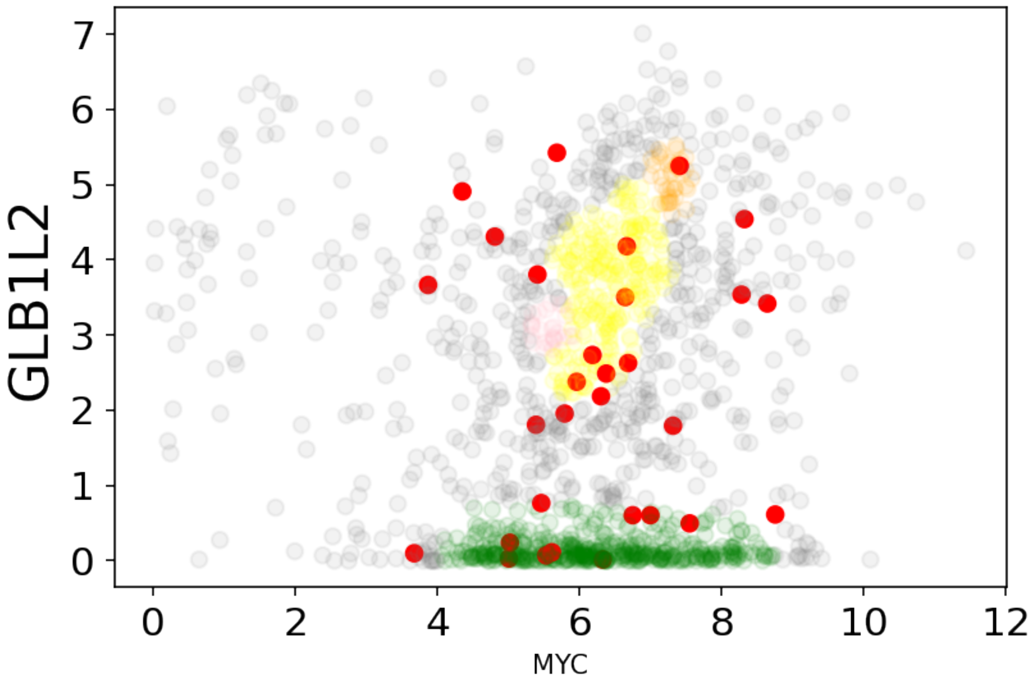
GLB1L2
MYC
0
1
2
3
4
5
6
7
0
2
4
6
8
10
12

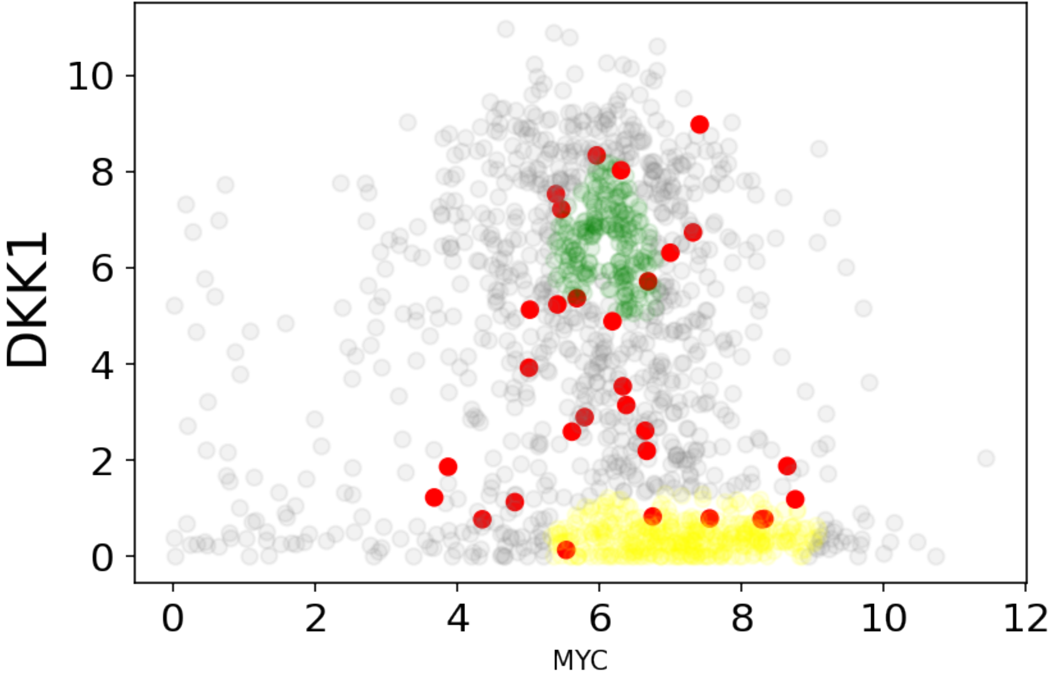

DKK1
10
8
6
4
2
0
0
2
4
6
8
10
12
MYC

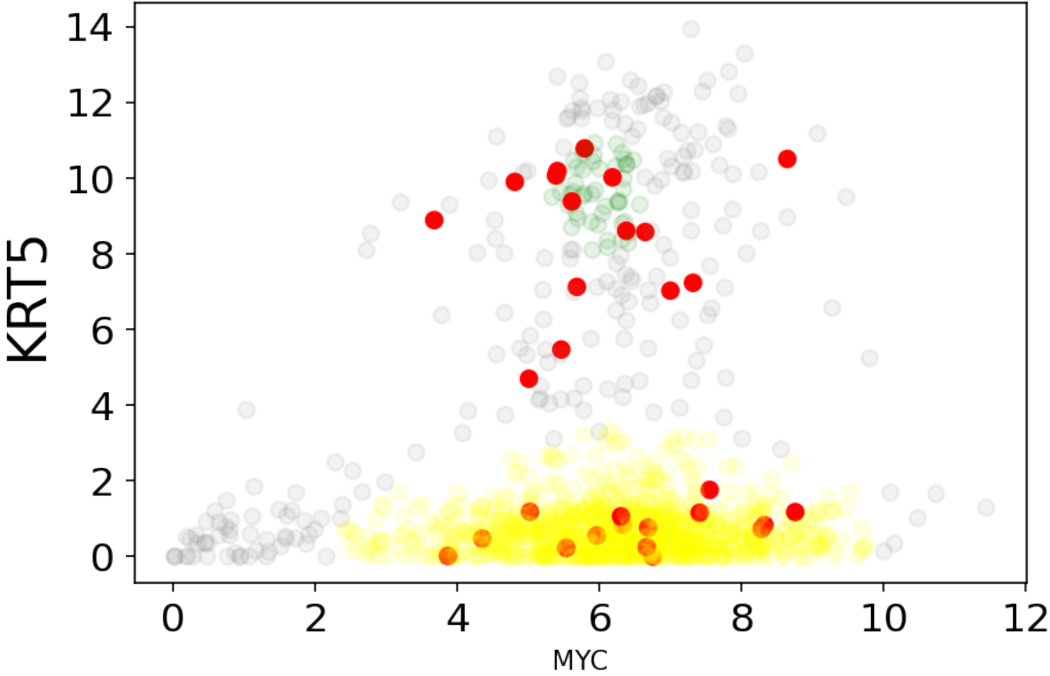

KRT5
MYC
0
2
4
6
8
10
12
14

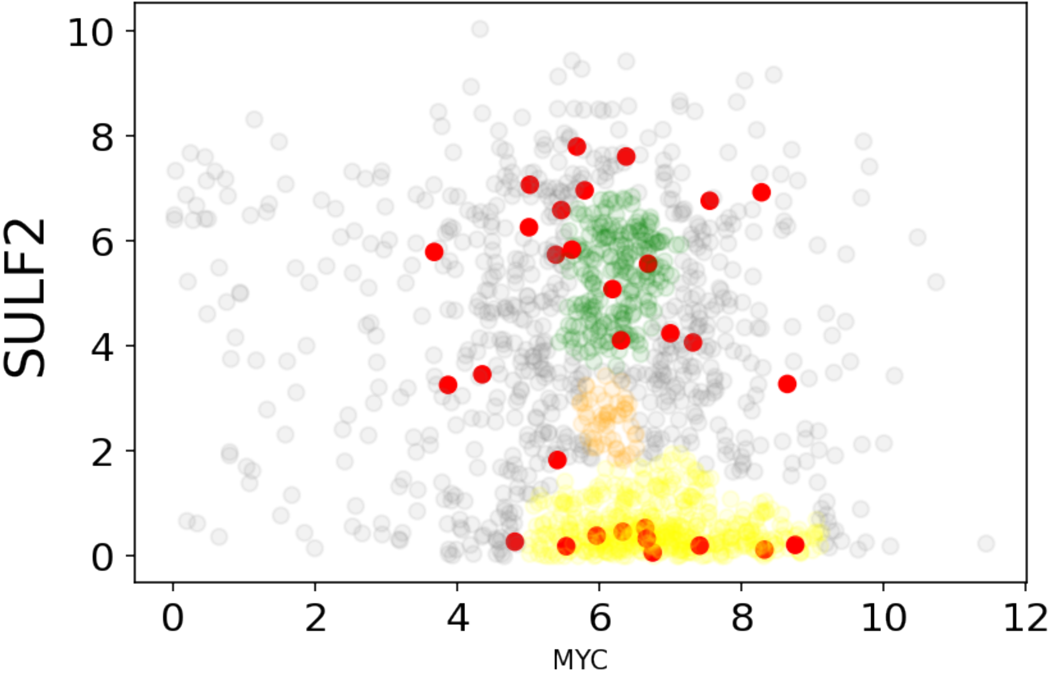
SULF2
MYC
0
2
4
6
8
10
12

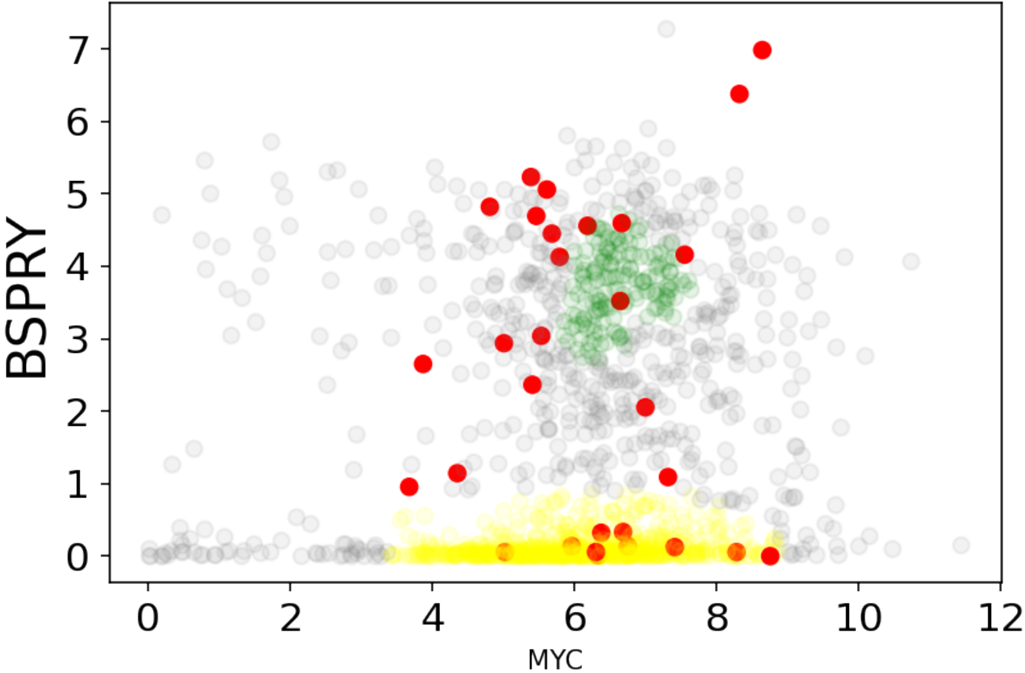
BSPRY
MYC
0
1
2
3
4
5
6
7
0
2
4
6
8
10
12

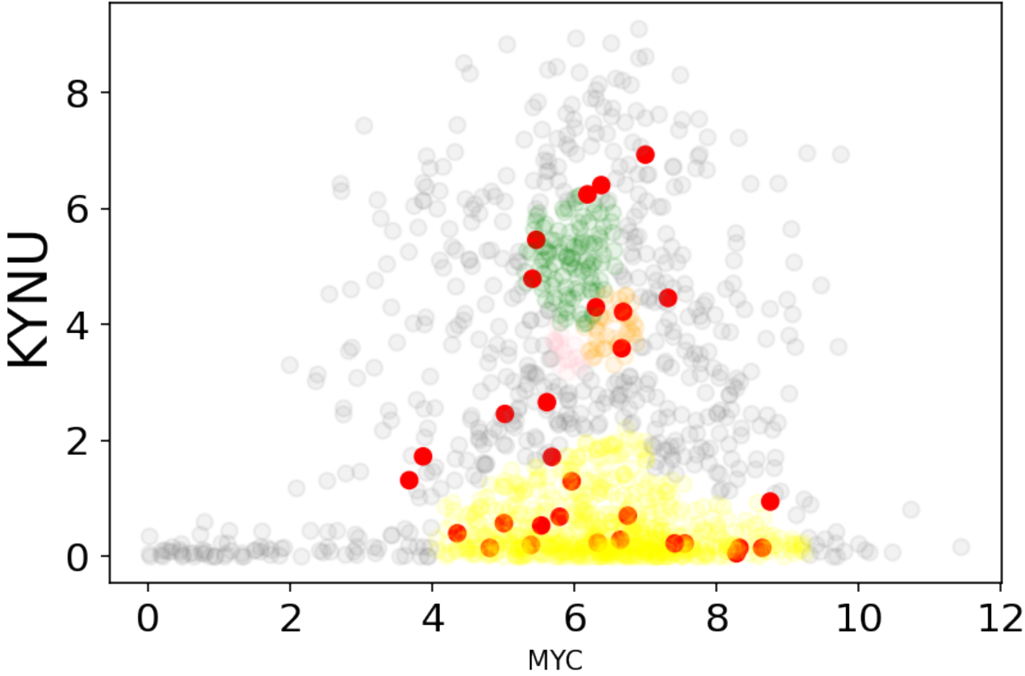

KYNU
MYC
0
2
4
6
8
0
2
4
6
8
10
12

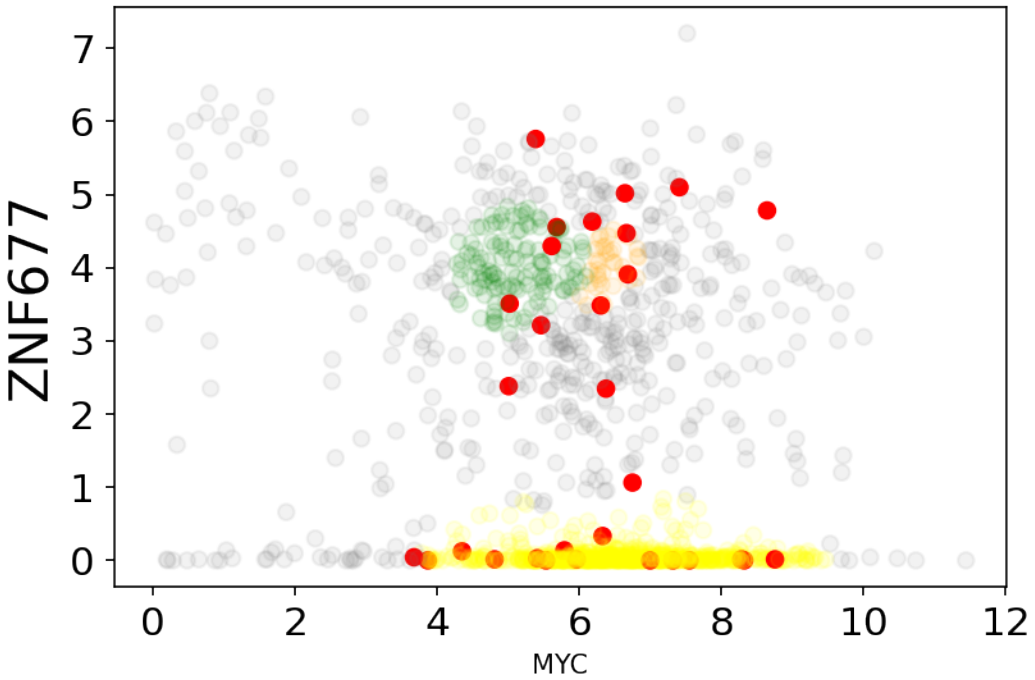

ZNF677
MYC
0
1
2
3
4
5
6
7
0
2
4
6
8
10
12

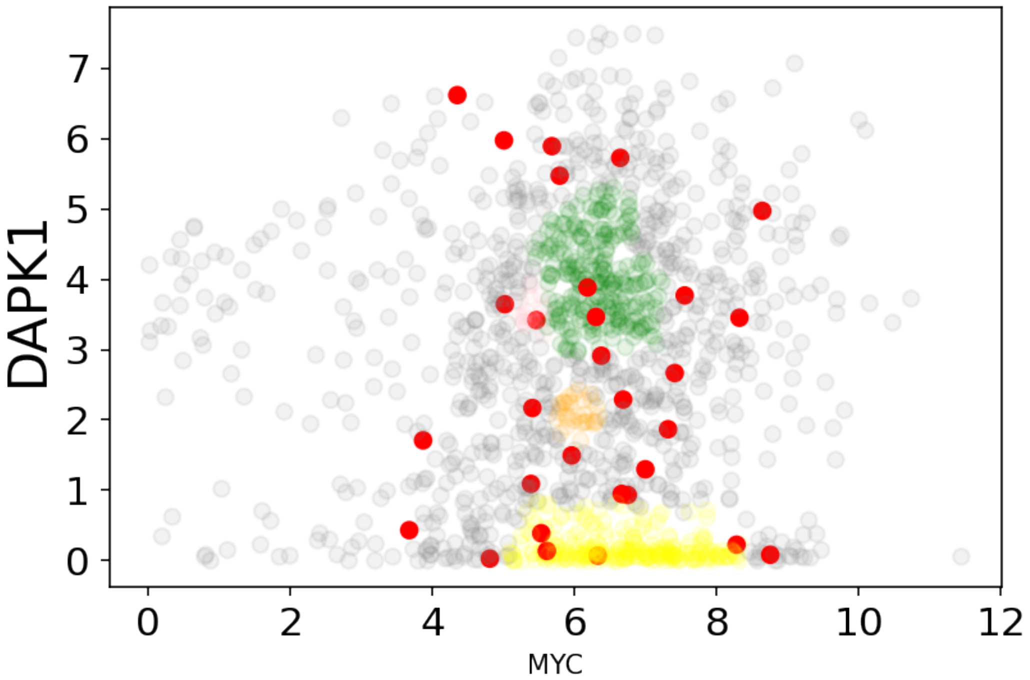
DAPK1
MYC
0
1
2
3
4
5
6
7
0
2
4
6
8
10
12

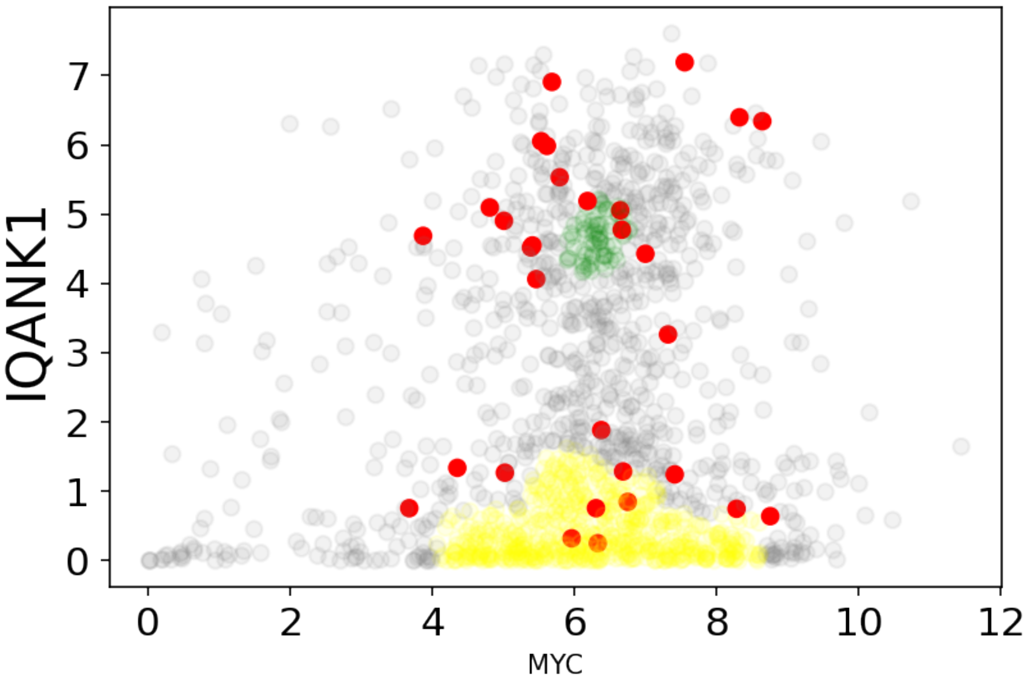
IQANK1
MYC
0
1
2
3
4
5
6
7
0
2
4
6
8
10
12

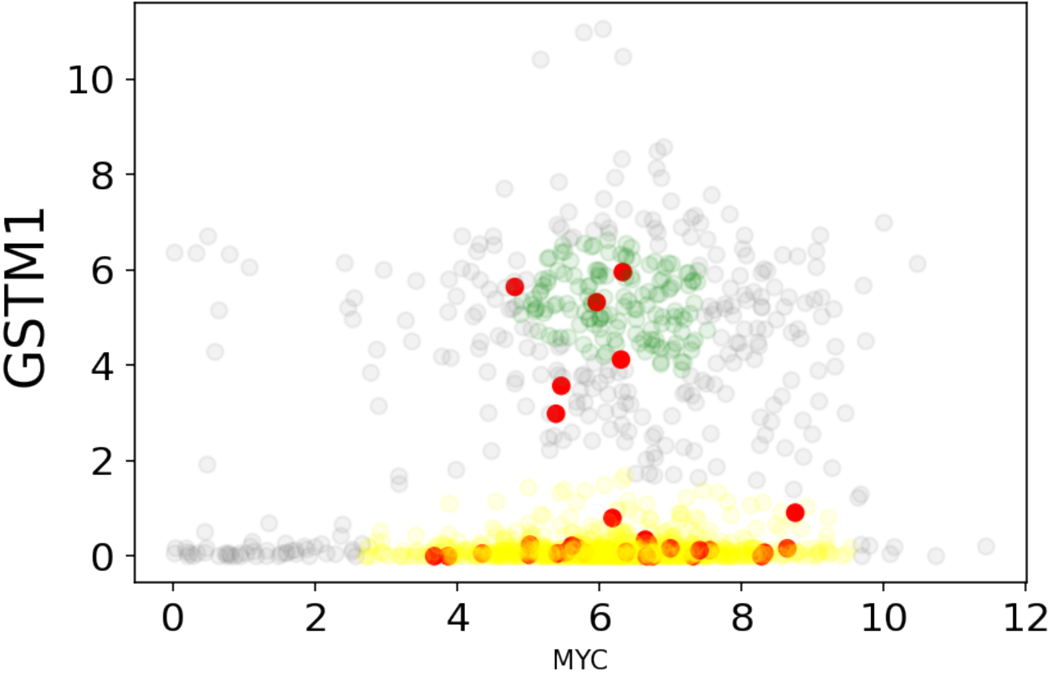
GSTM1
MYC
0
2
4
6
8
10
12

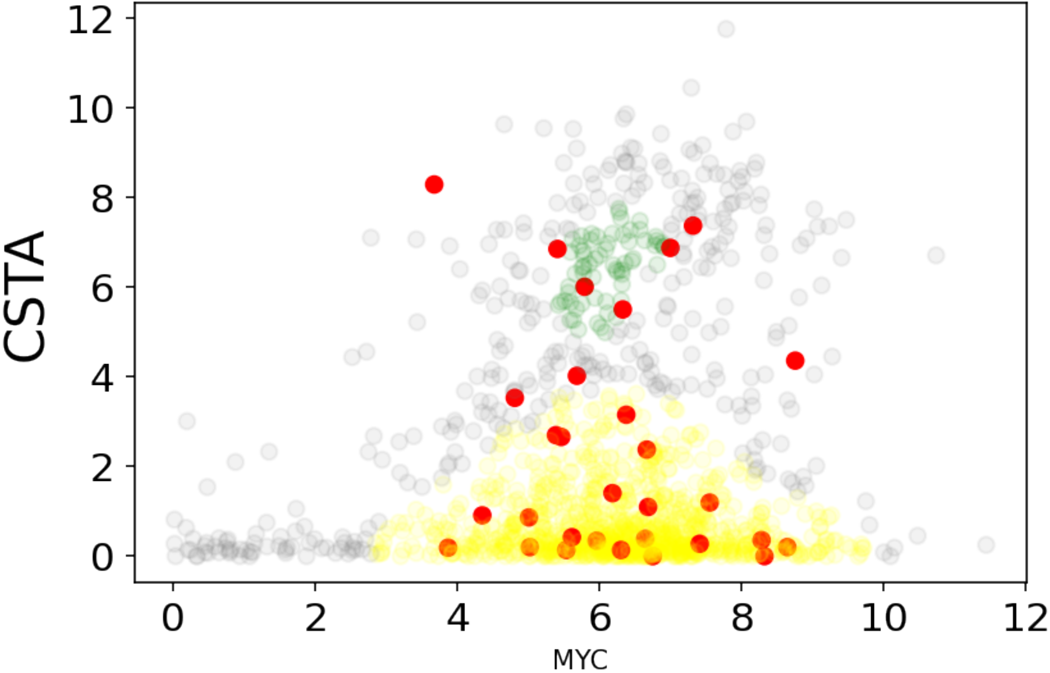
12
10
8
6
4
2
0
CSTA
0
2
4
6
8
10
12
MYC

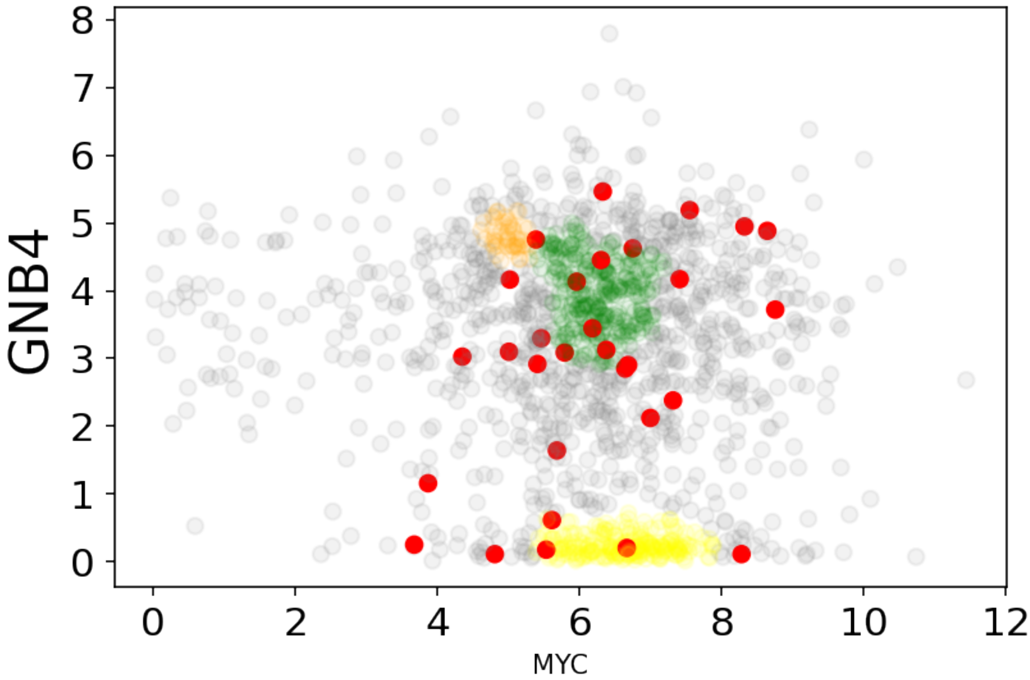
GNB4
MYC
0
1
2
3
4
5
6
7
8
0
2
4
6
8
10
12

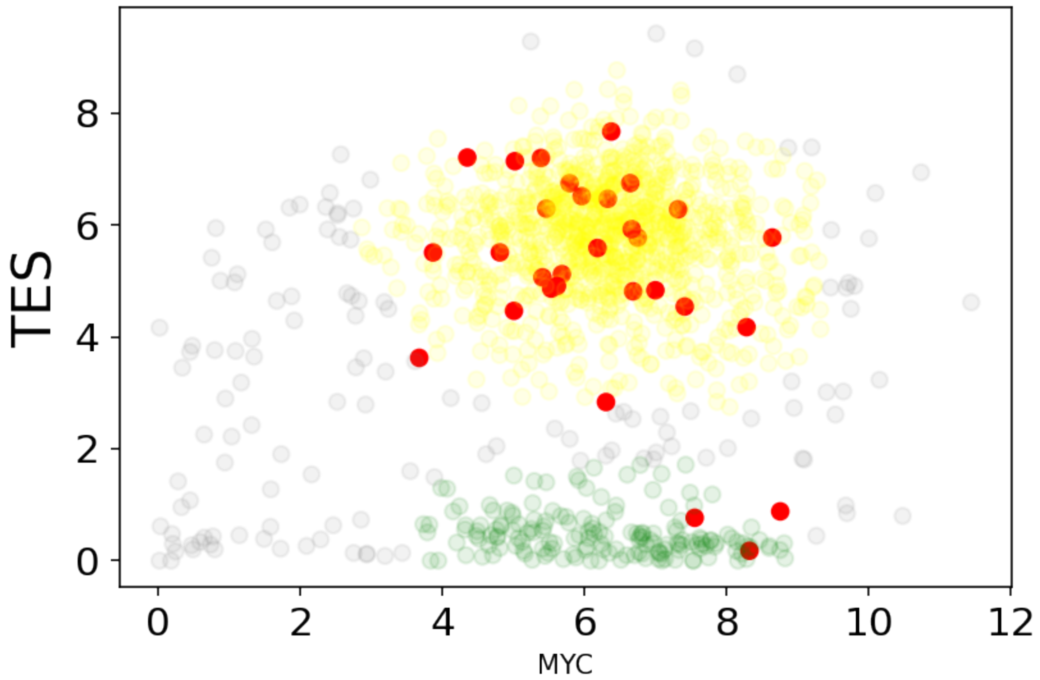

TES
0
2
4
6
8
0
2
4
6
8
10
12
MYC

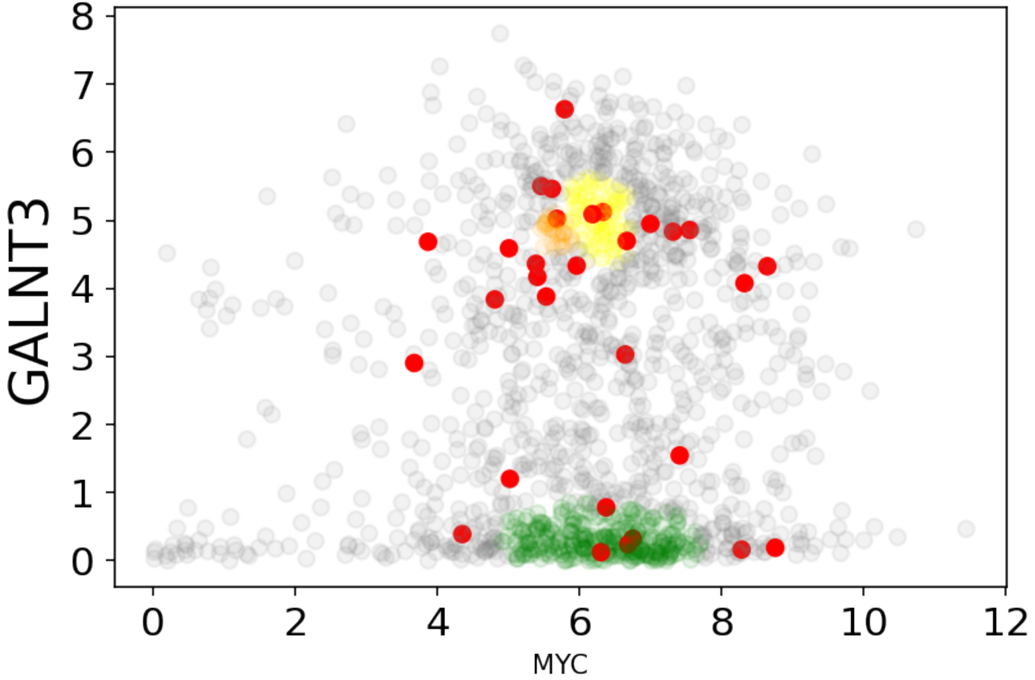

GALNT3
MYC
0
1
2
3
4
5
6
7
8
0
2
4
6
8
10
12

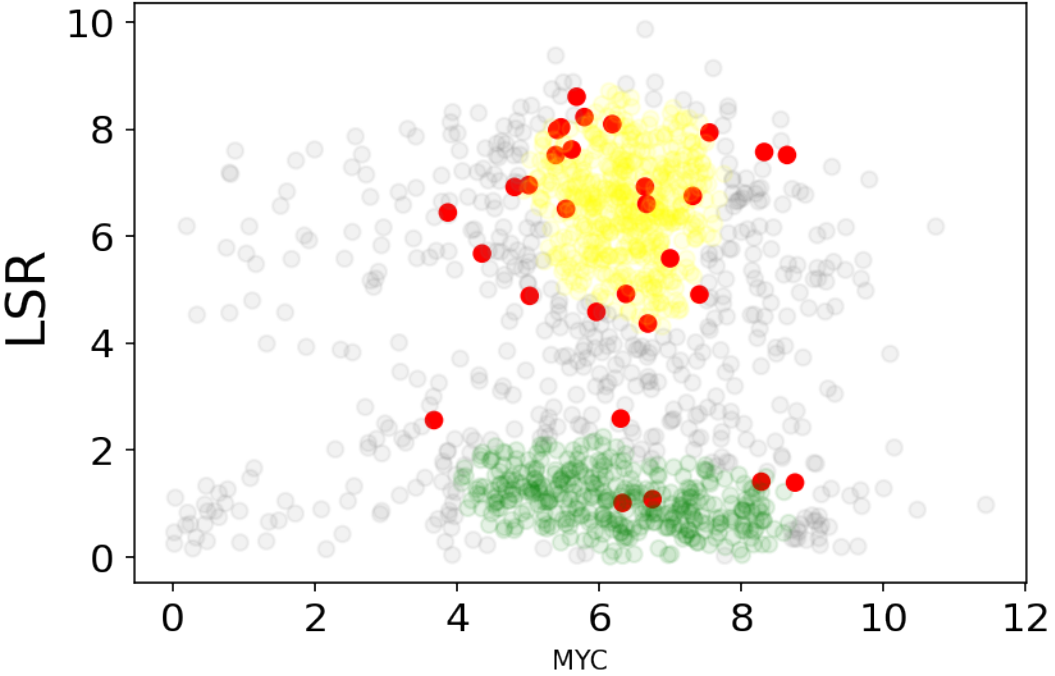
10
8
6
4
2
0
LSR
0
2
4
6
8
10
12
MYC

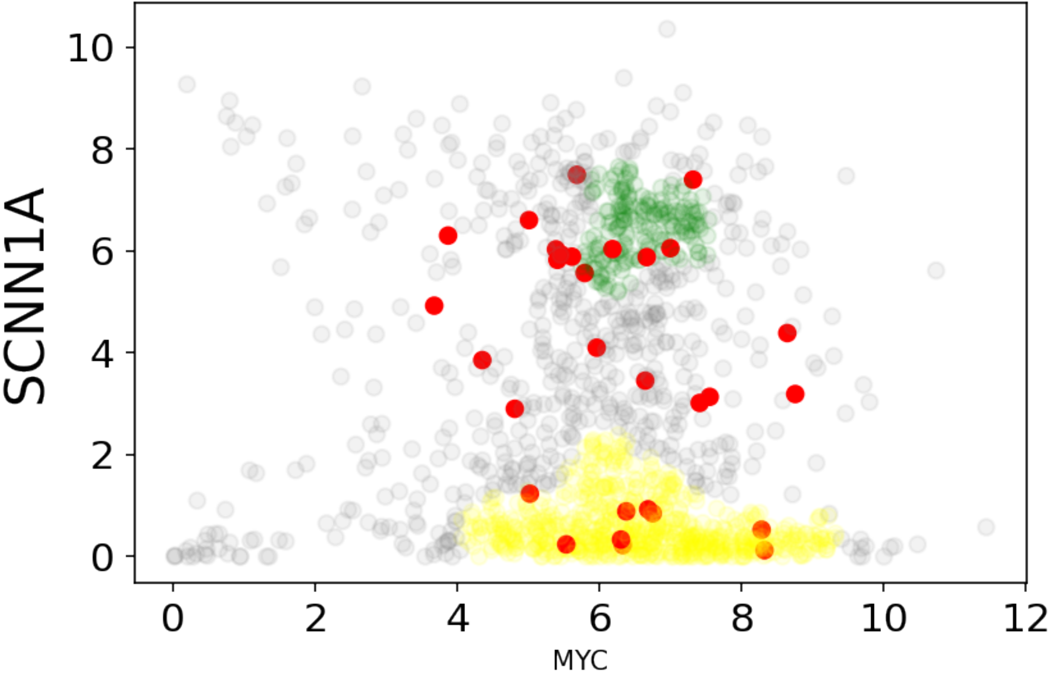
SCNN1A
MYC
0
2
4
6
8
10
12

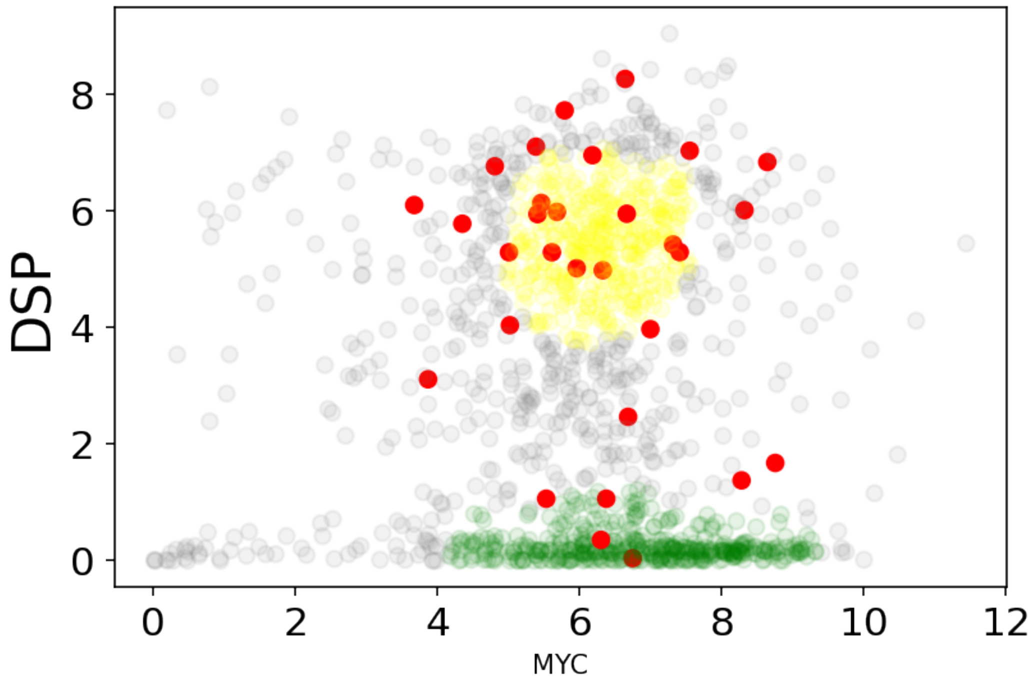

DSP
MYC
0
2
4
6
8
10
12

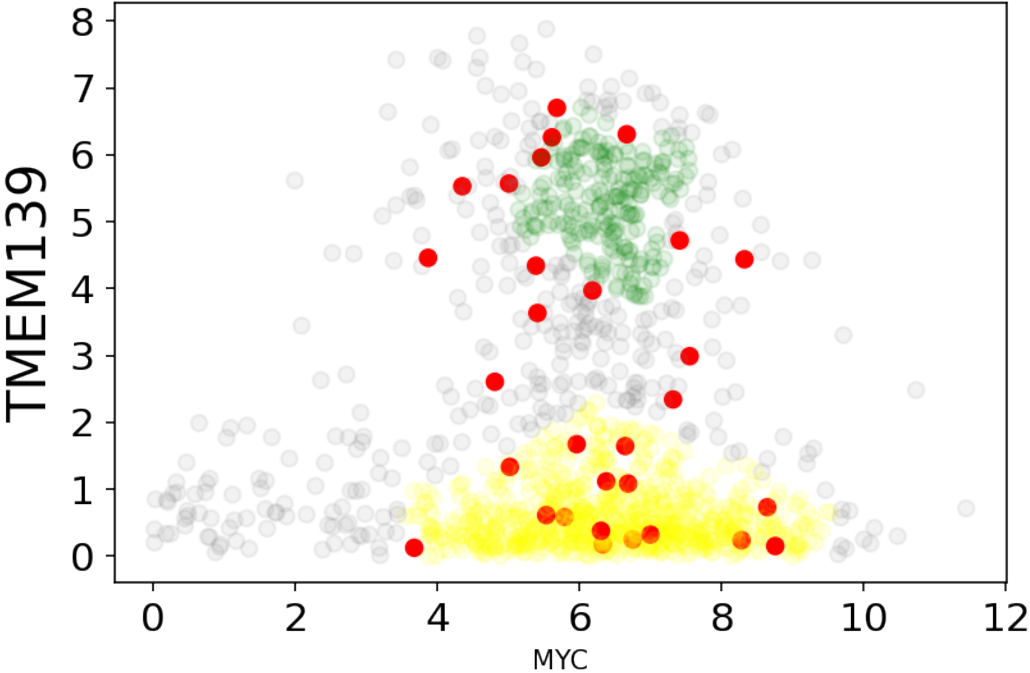
TMEM139
MYC
0
1
2
3
4
5
6
7
8
0
2
4
6
8
10
12

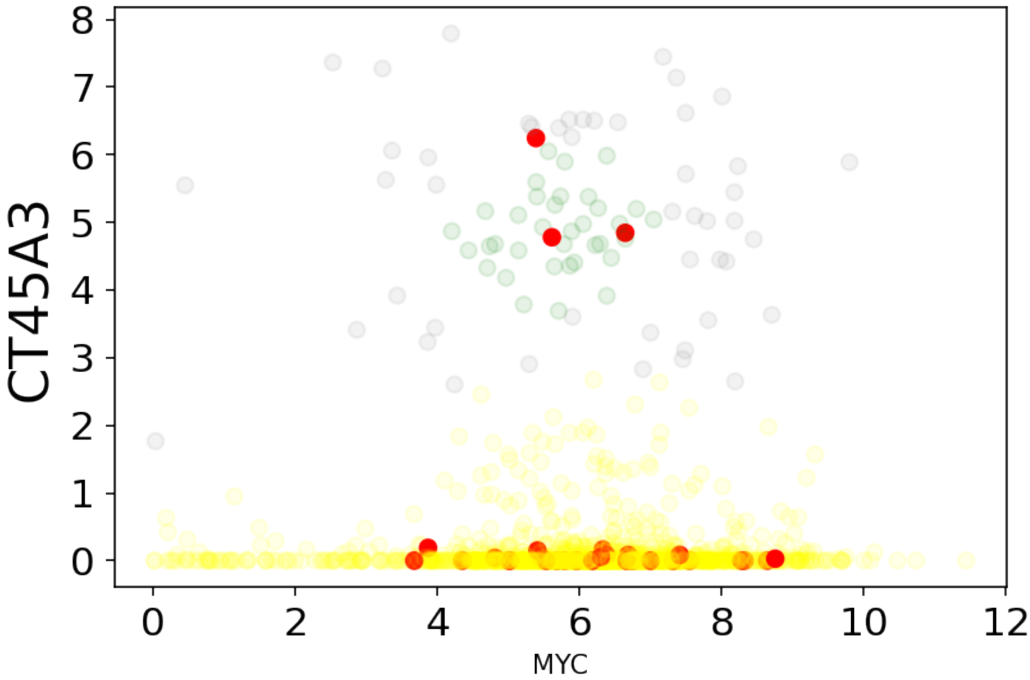

CT45A3
MYC
0
1
2
3
4
5
6
7
8
0
2
4
6
8
10
12

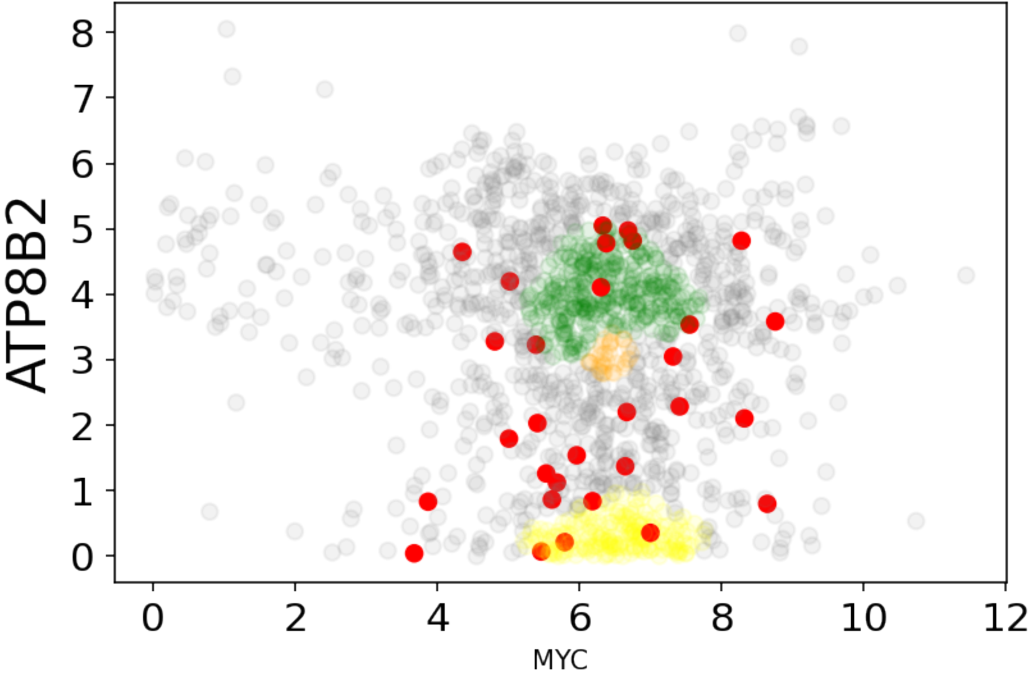

ATP8B2
MYC
0
1
2
3
4
5
6
7
8
0
2
4
6
8
10
12

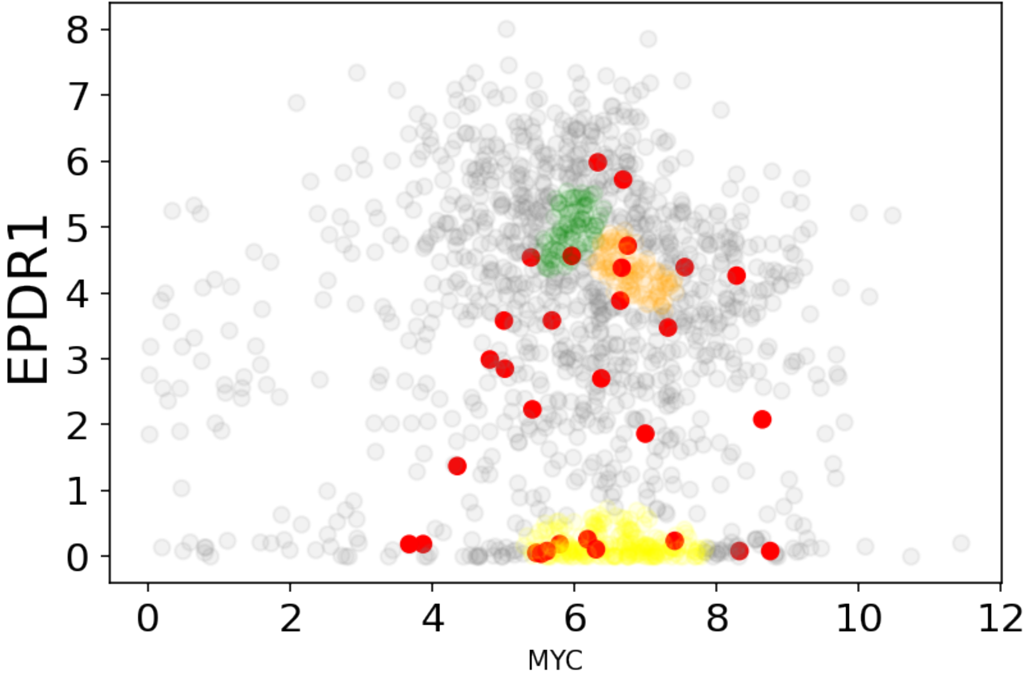
EPDR1
MYC
0
1
2
3
4
5
6
7
8
0
2
4
6
8
10
12

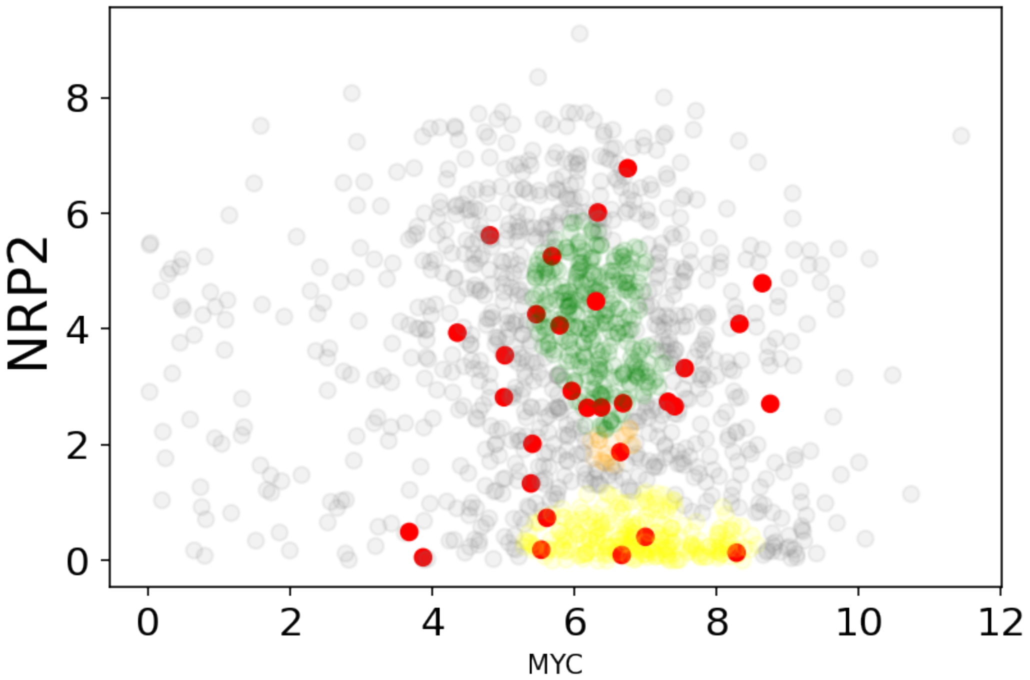

NRP2
8
6
4
2
0
0
2
4
6
8
10
12
MYC

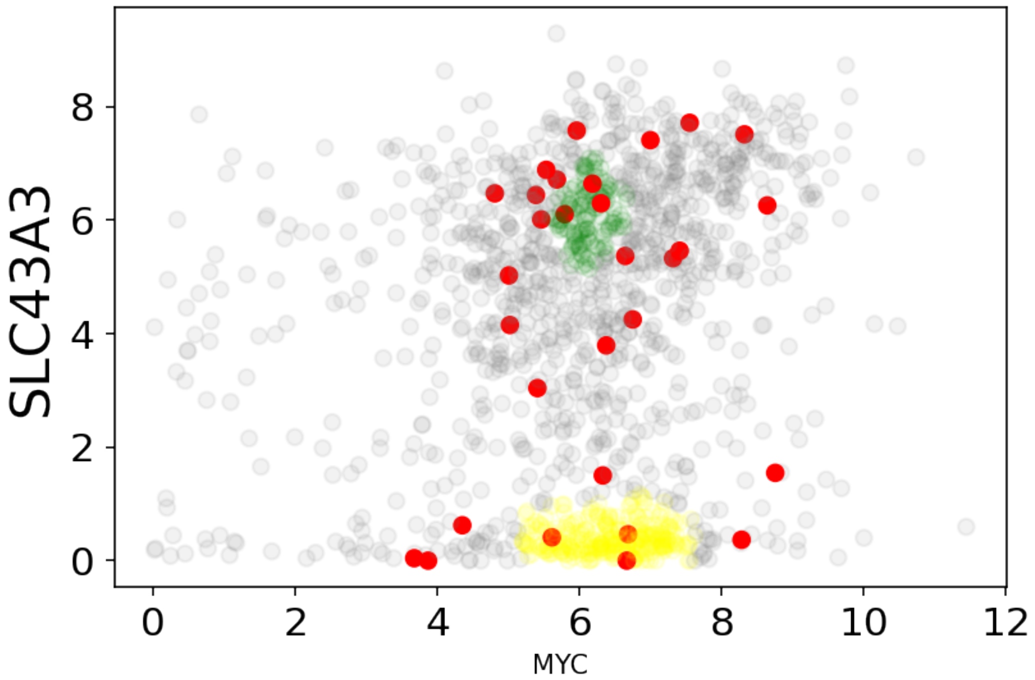
SLC43A3
8
6
4
2
0
0
2
4
6
8
10
12
MYC

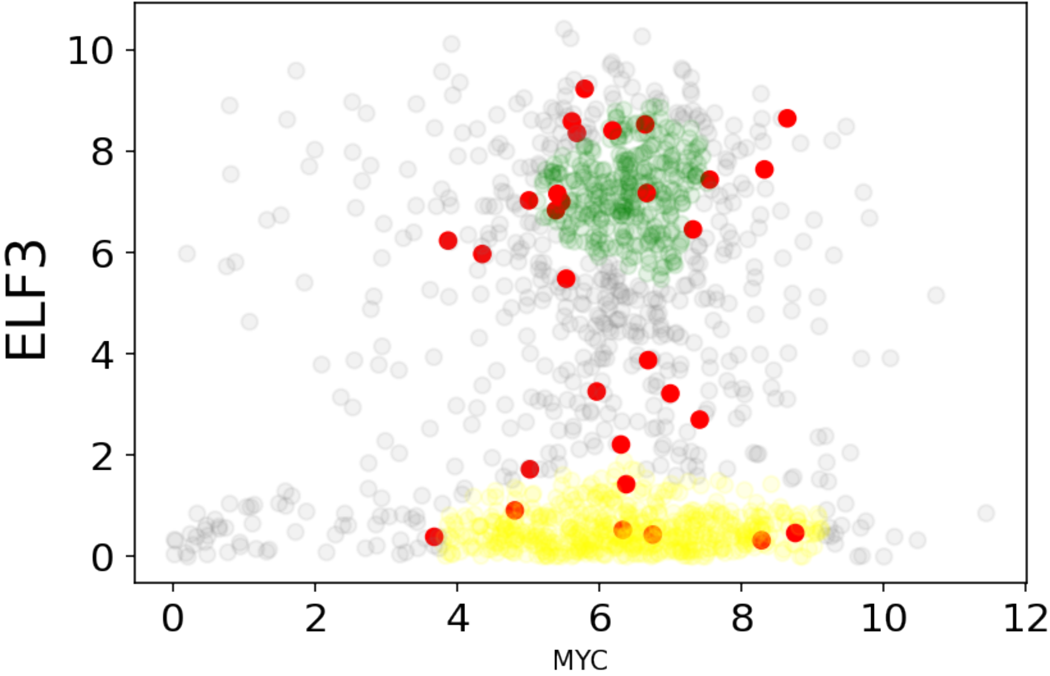
ELF3
MYC
0
2
4
6
8
10
12

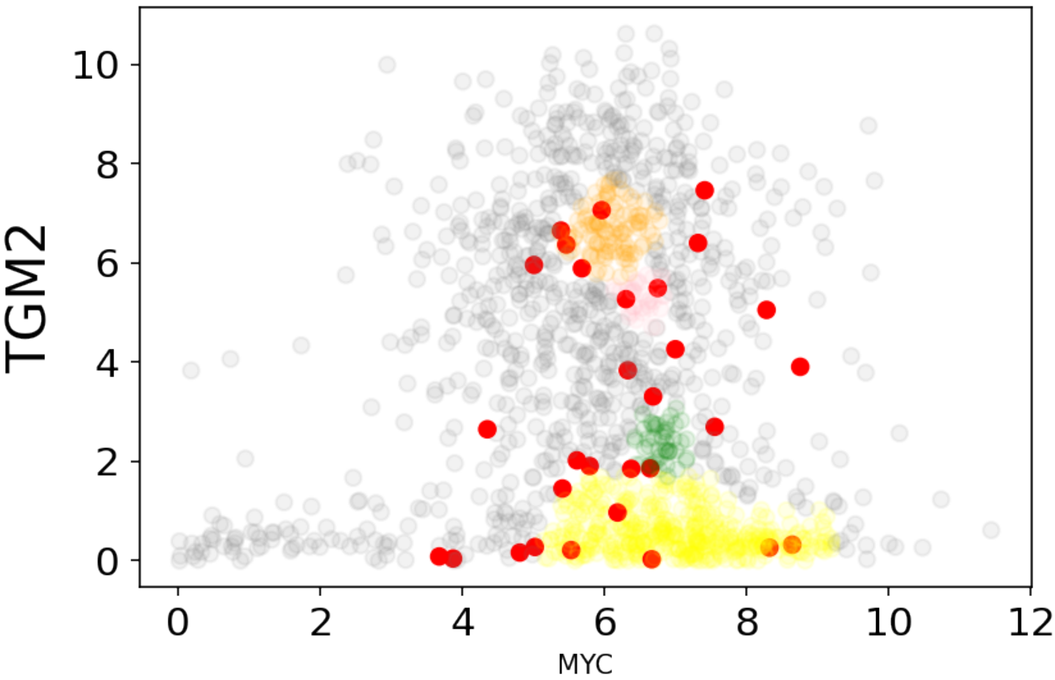

TGM2
10
8
6
4
2
0
0
2
4
6
8
10
12
MYC

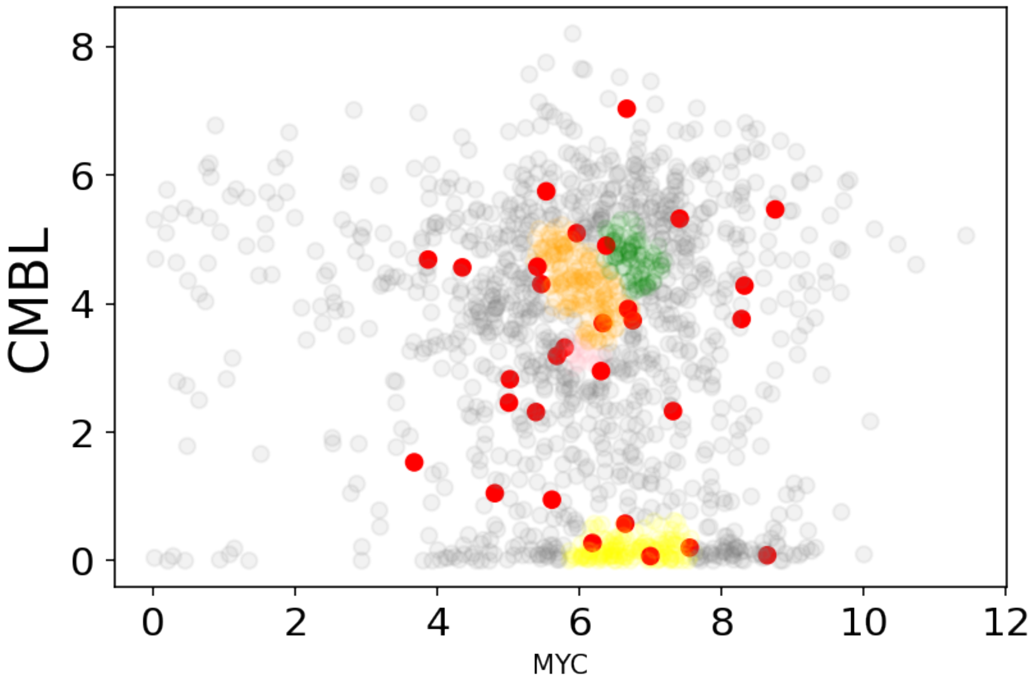

CMBL
8
6
4
2
0
0
2
4
6
8
10
12
MYC

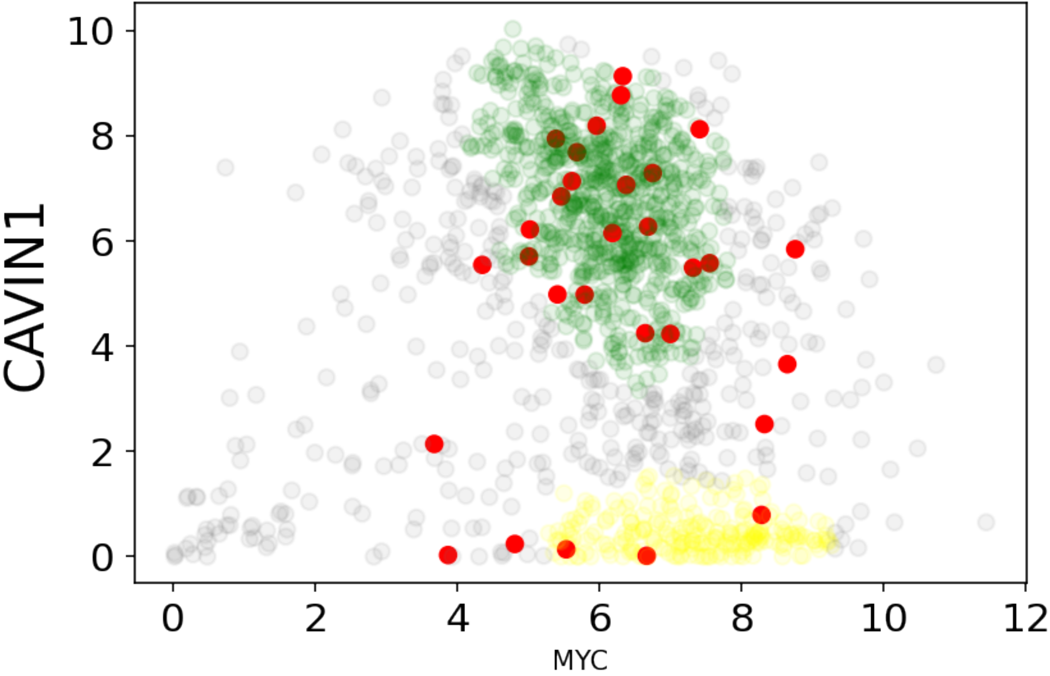

CAVIN1
MYC
0
2
4
6
8
10
12

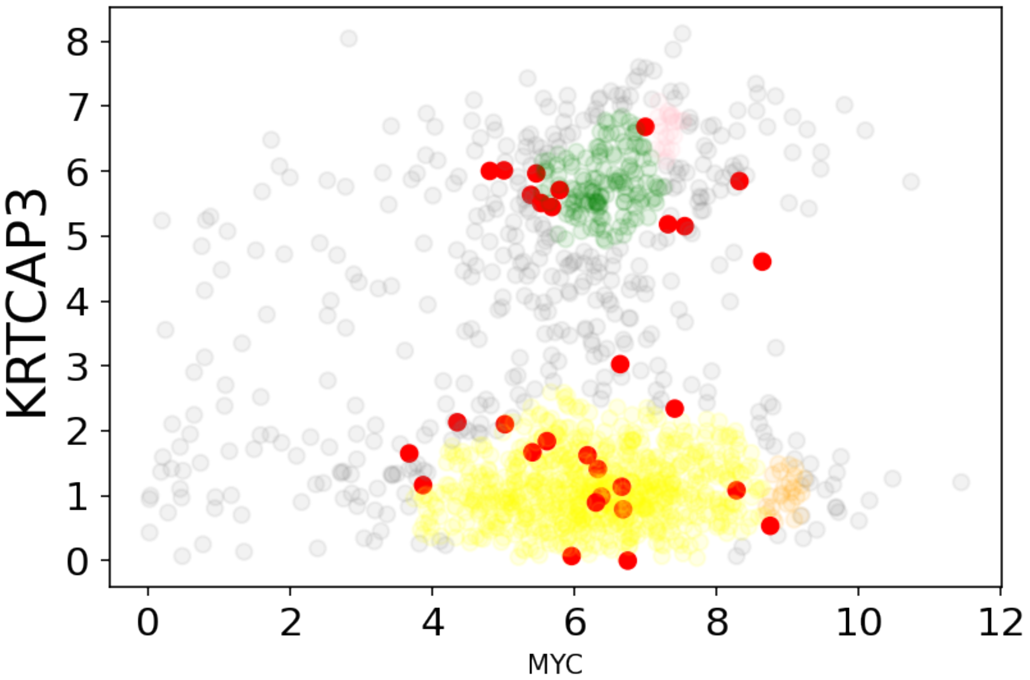

KRTCAP3
MYC
0
1
2
3
4
5
6
7
8
0
2
4
6
8
10
12

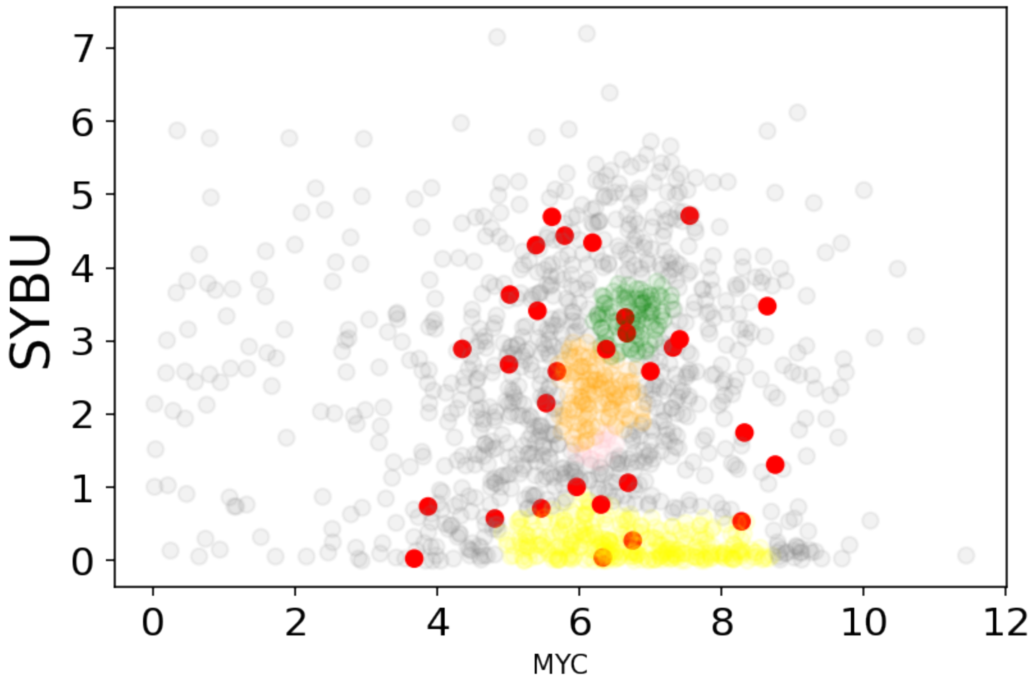

SYBU
7
6
5
4
3
2
1
0
0
2
4
6
8
10
12
MYC

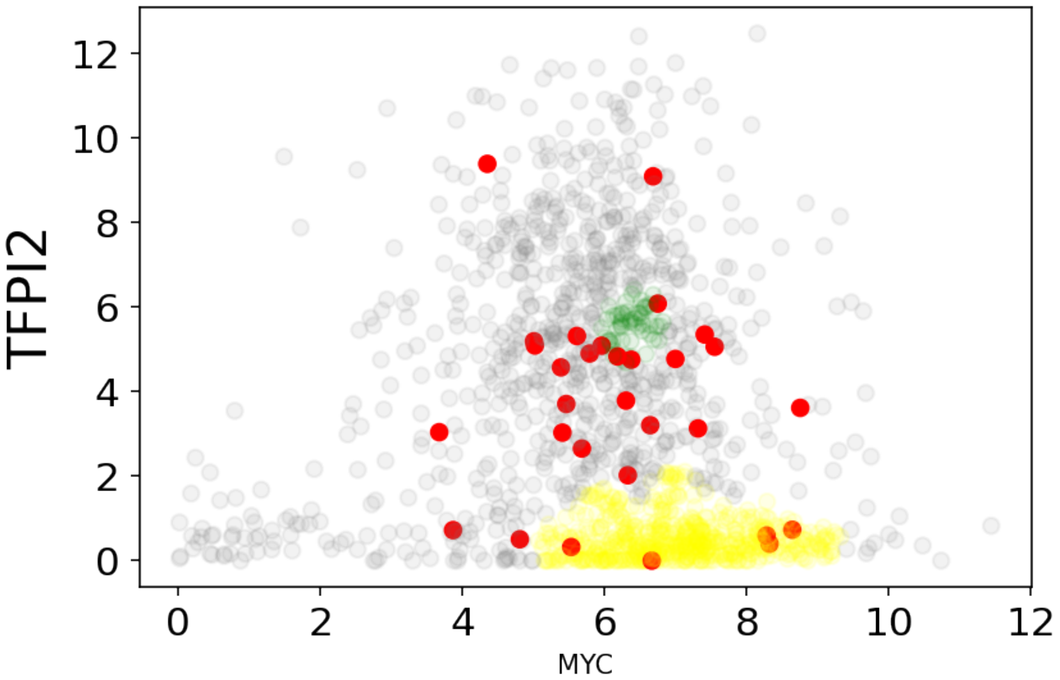

TFPI2
MYC
0
2
4
6
8
10
12

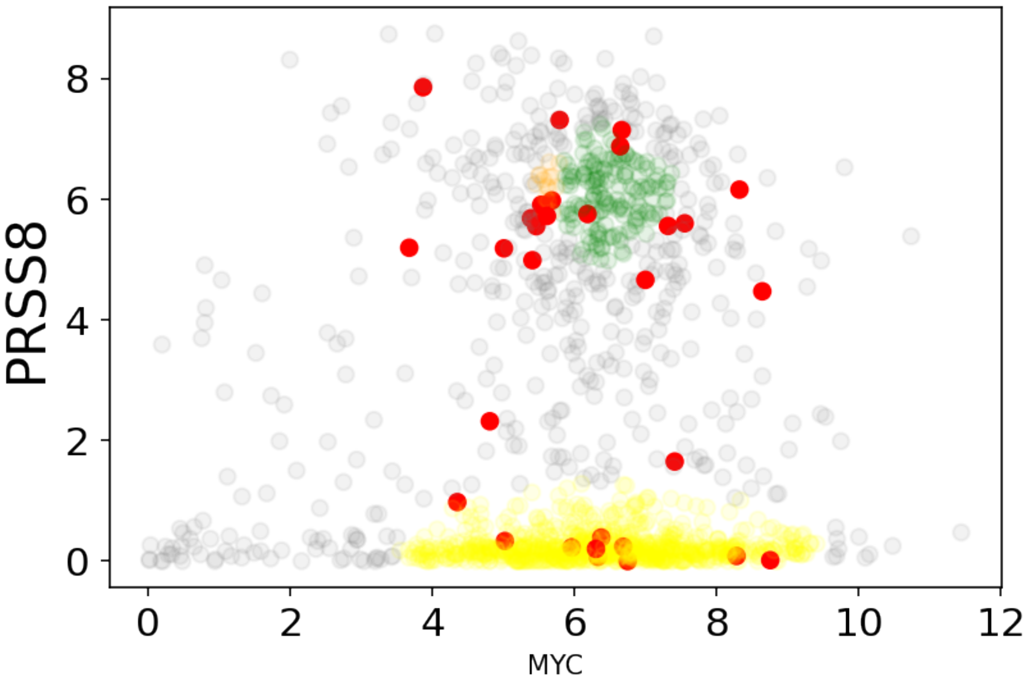

PRSS8
MYC
0
2
4
6
8
0
2
4
6
8
10
12

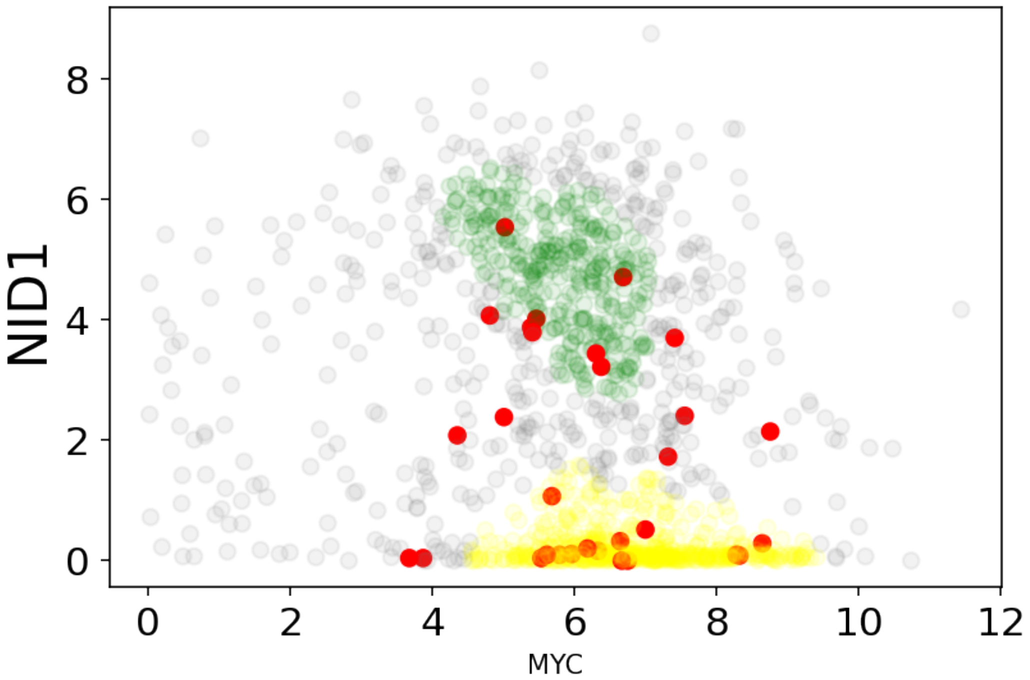

NID1
MYC
0
2
4
6
8
10
12

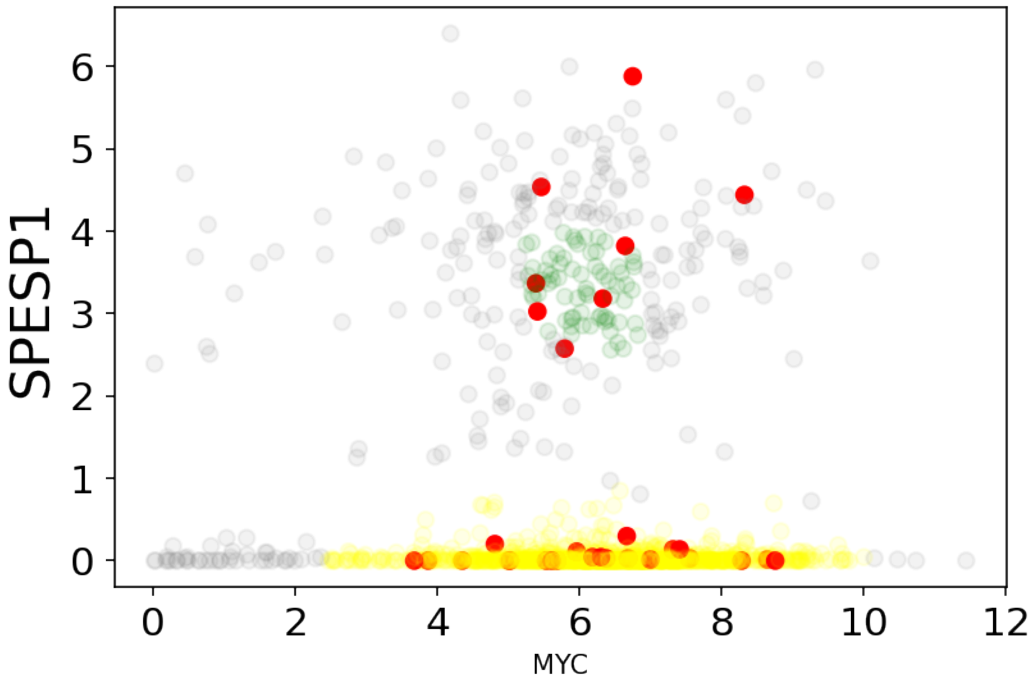
SPESP1
MYC
0
1
2
3
4
5
6
0
2
4
6
8
10
12

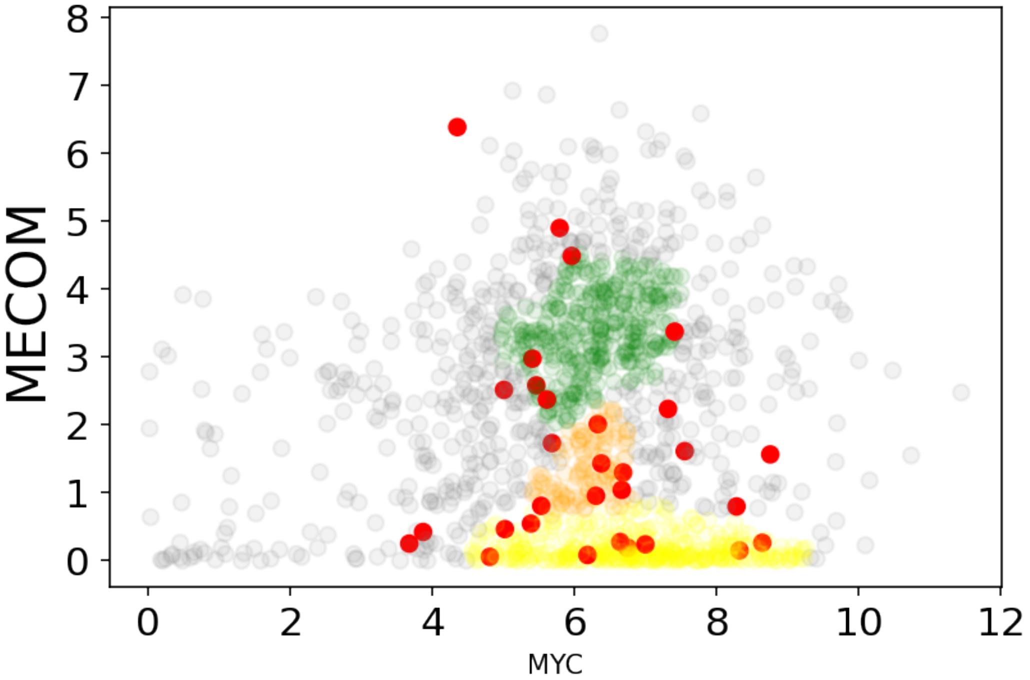
MECOM
MYC
0
1
2
3
4
5
6
7
8
0
2
4
6
8
10
12

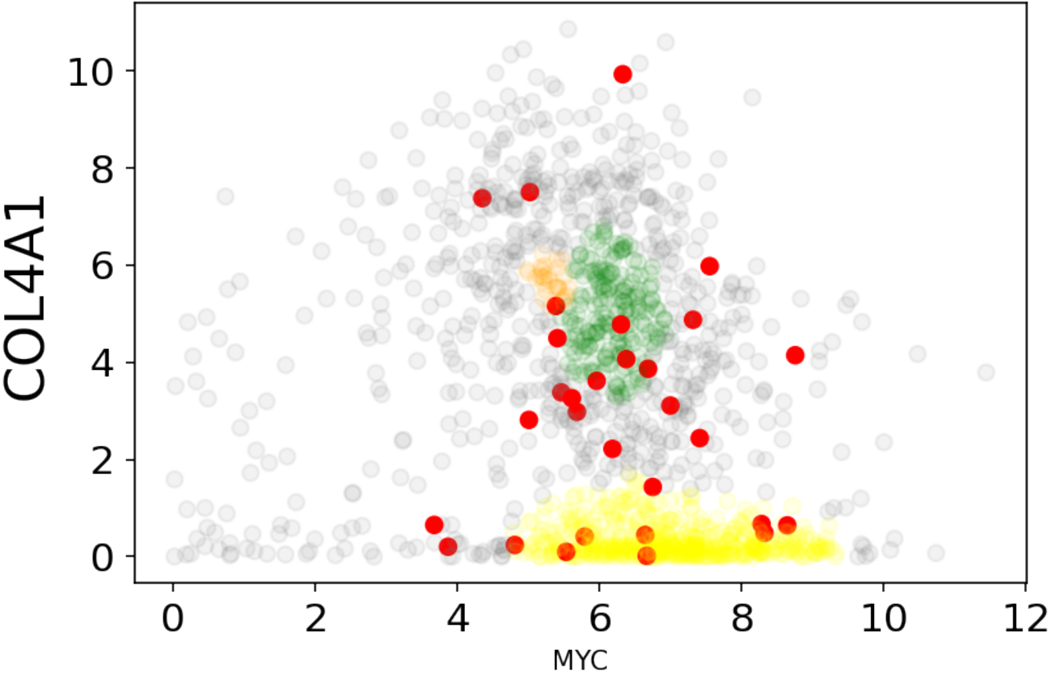

COL4A1
MYC
0
2
4
6
8
10
12

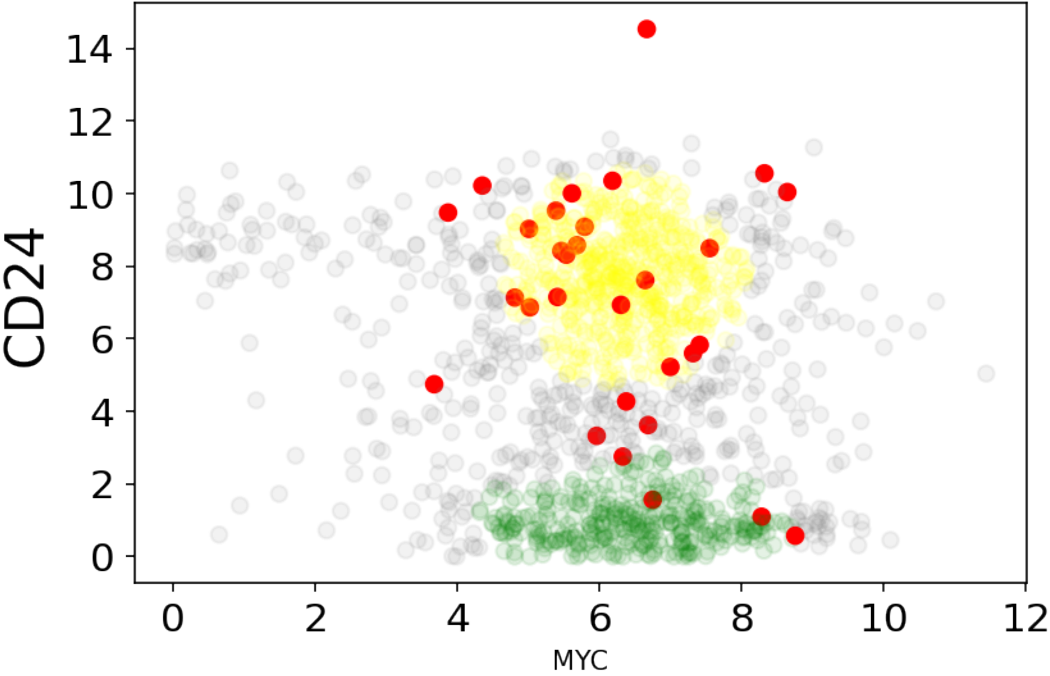

CD24
14
12
10
8
6
4
2
0
0
2
4
6
8
10
12
MYC

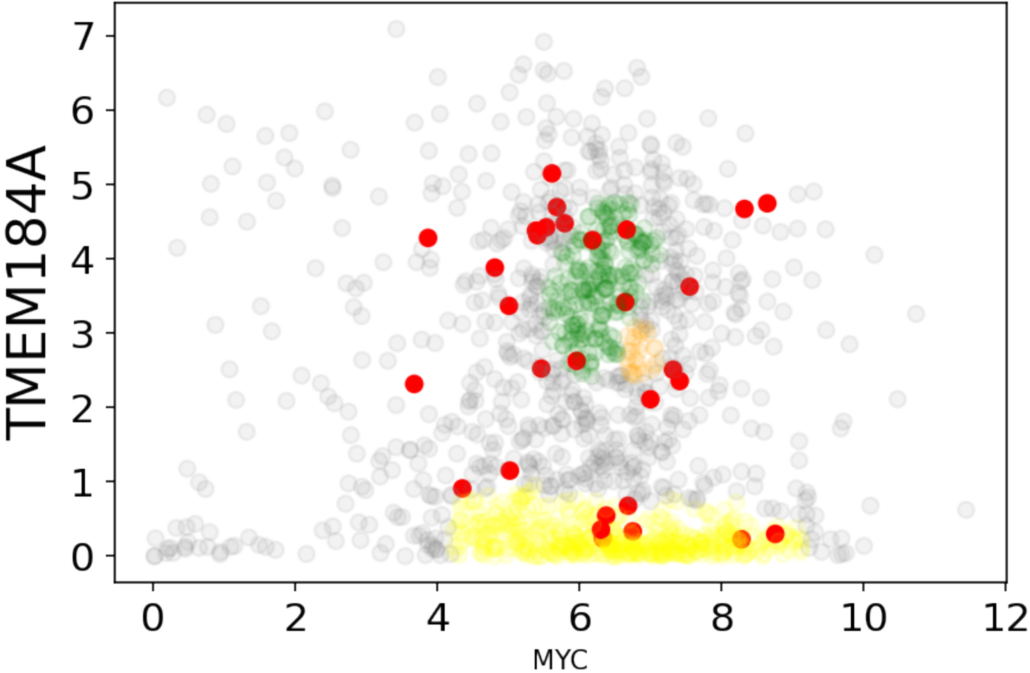

TMEM184A
MYC
0
1
2
3
4
5
6
7
0
2
4
6
8
10
12

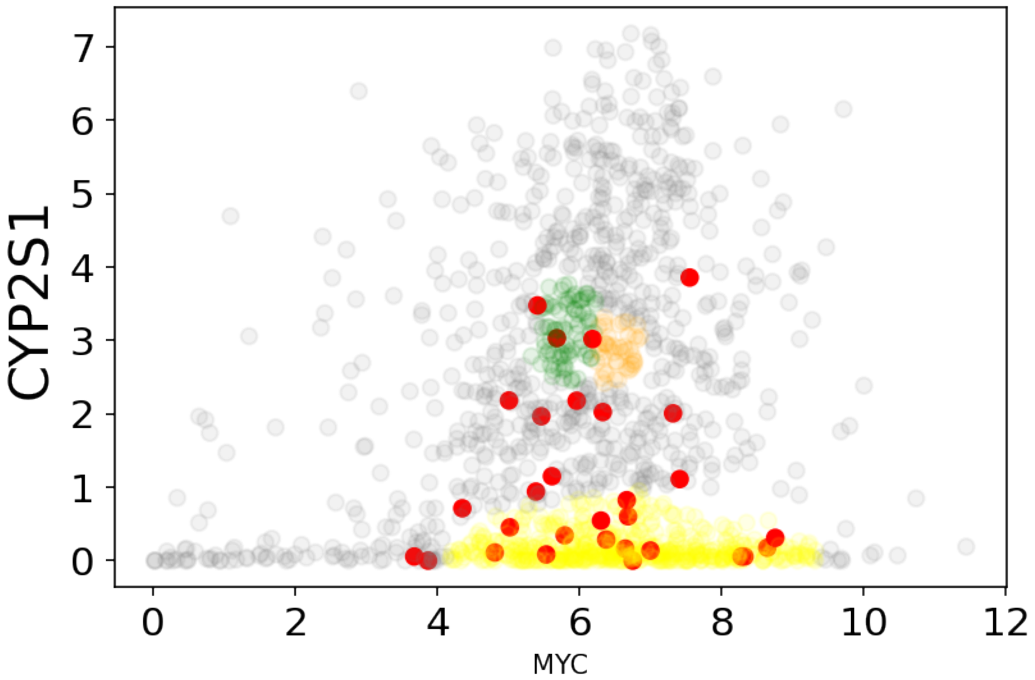

CYP2S1
0
1
2
3
4
5
6
7
0
2
4
6
8
10
12
MYC

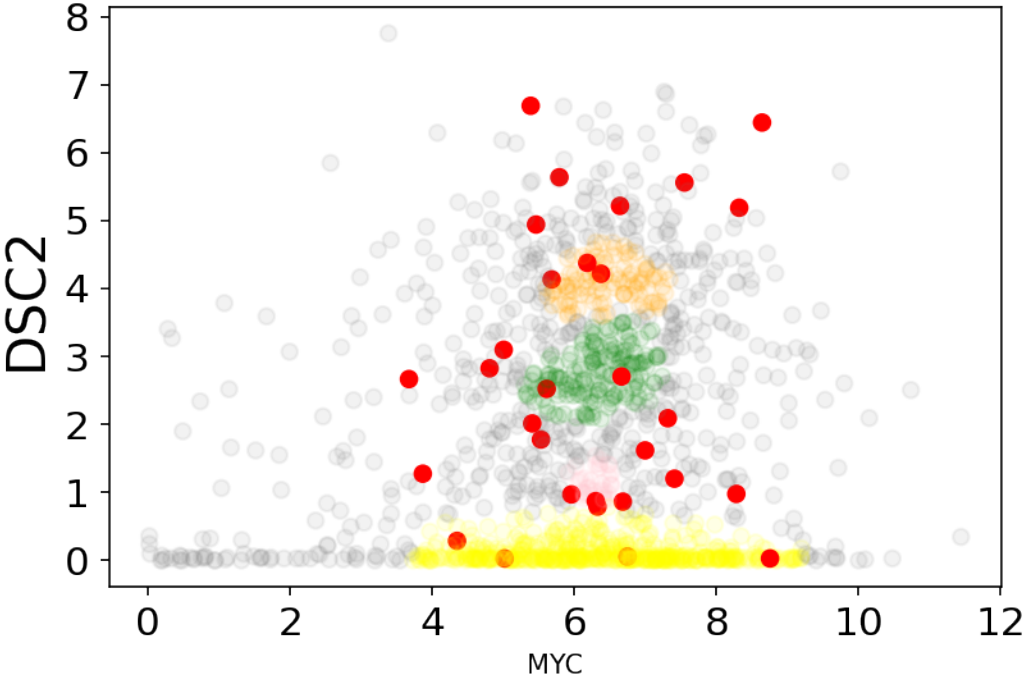
DSC2
MYC
0
2
4
6
8
10
12
0
1
2
3
4
5
6
7
8

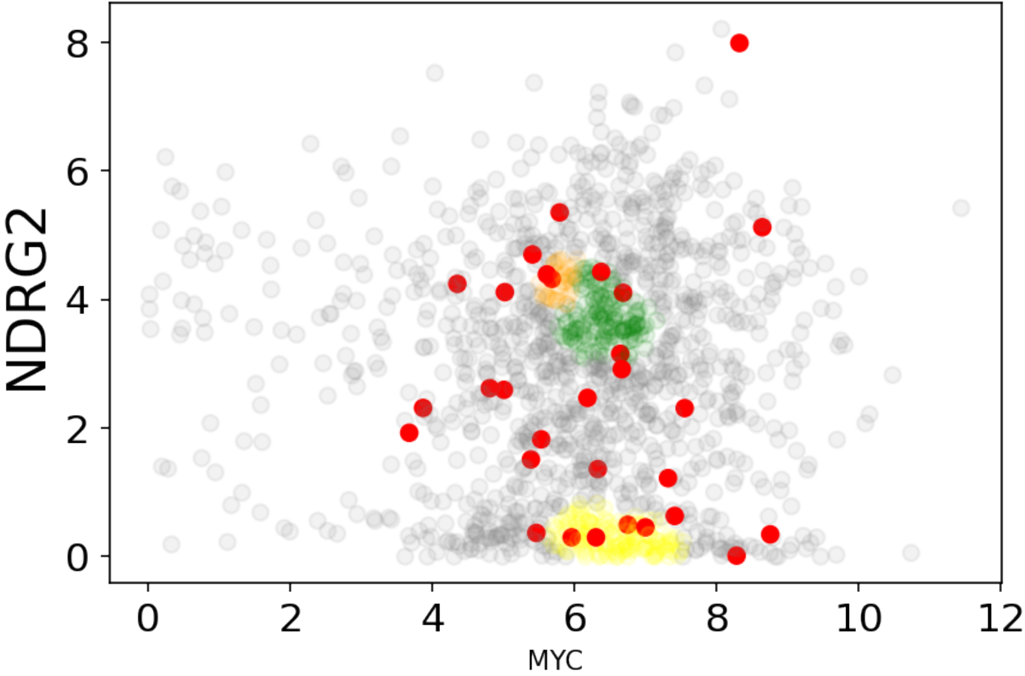
NDRG2
MYC
0
2
4
6
8
10
12

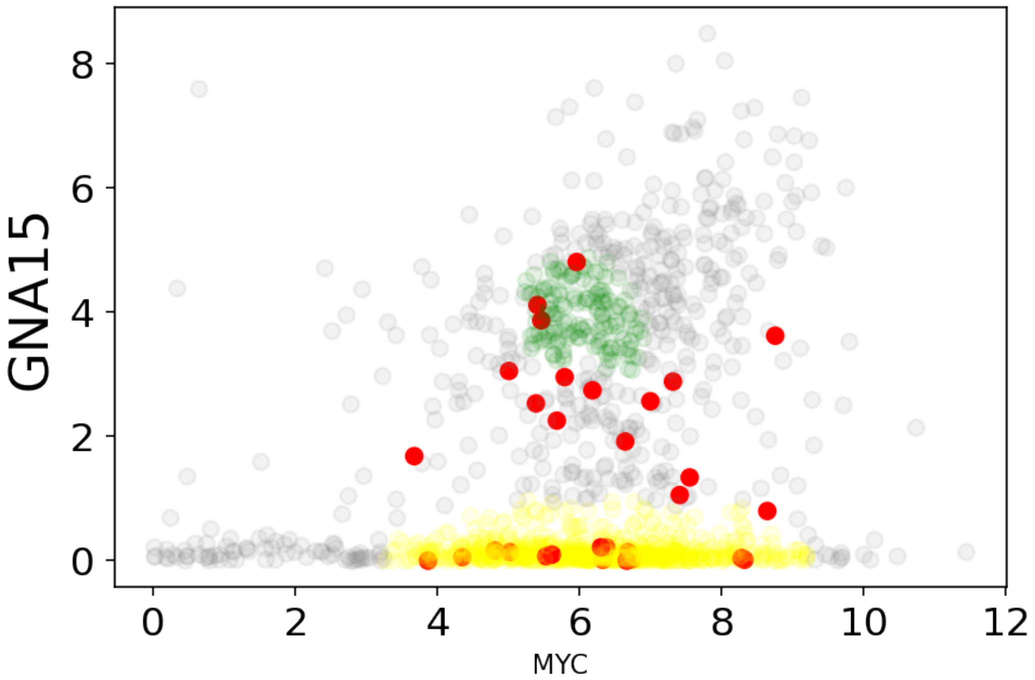

GNA15
8
6
4
2
0
0
2
4
6
8
10
12
MYC

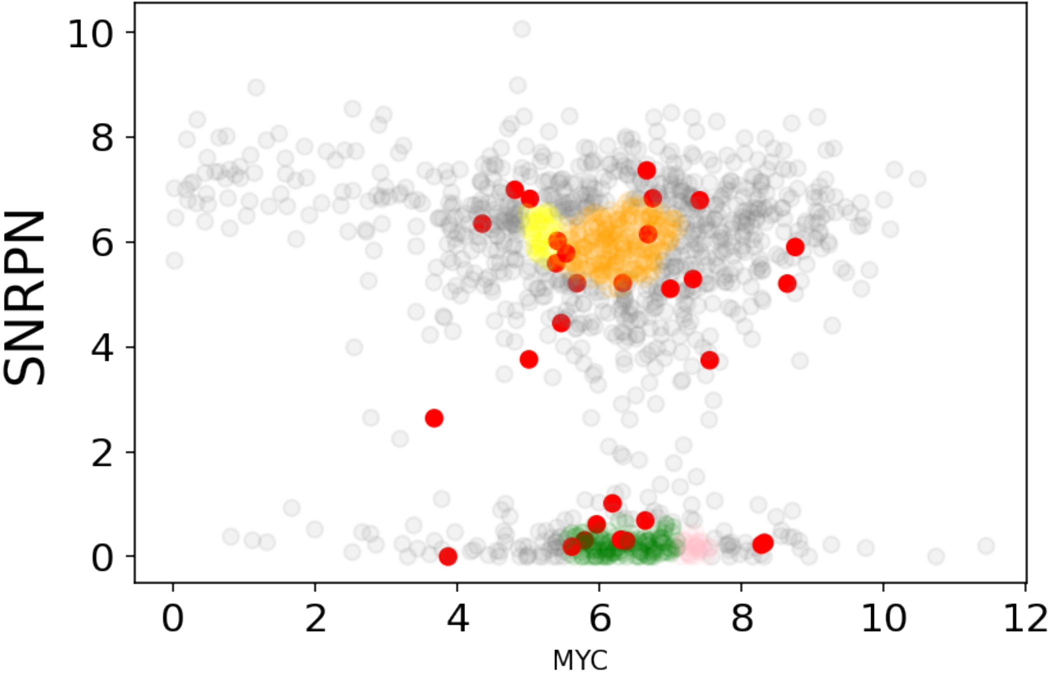

SNRPN
MYC
0
2
4
6
8
10
12

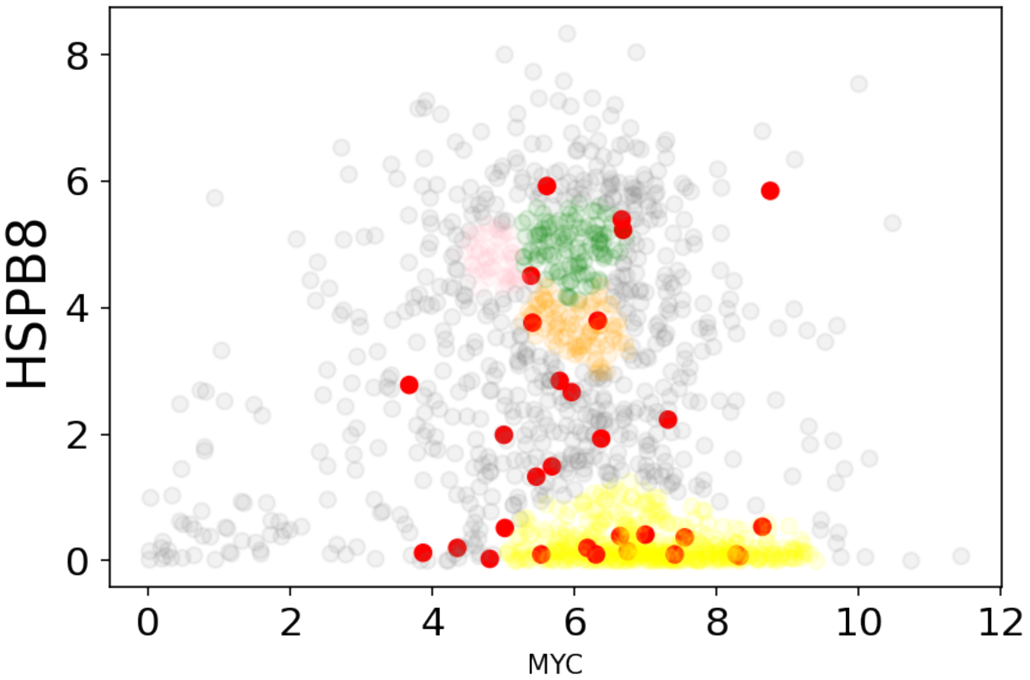
HSPB8
0
2
4
6
8
0
2
4
6
8
10
12
MYC

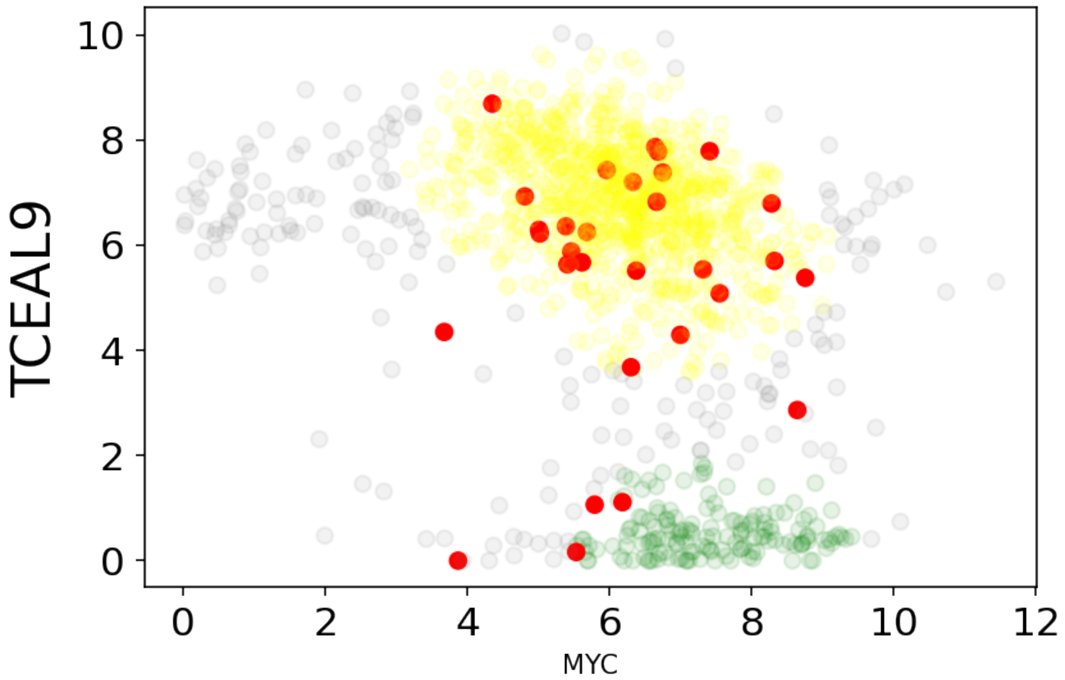
TCEAL9
10
8
6
4
2
0
0
2
4
6
8
10
12
MYC

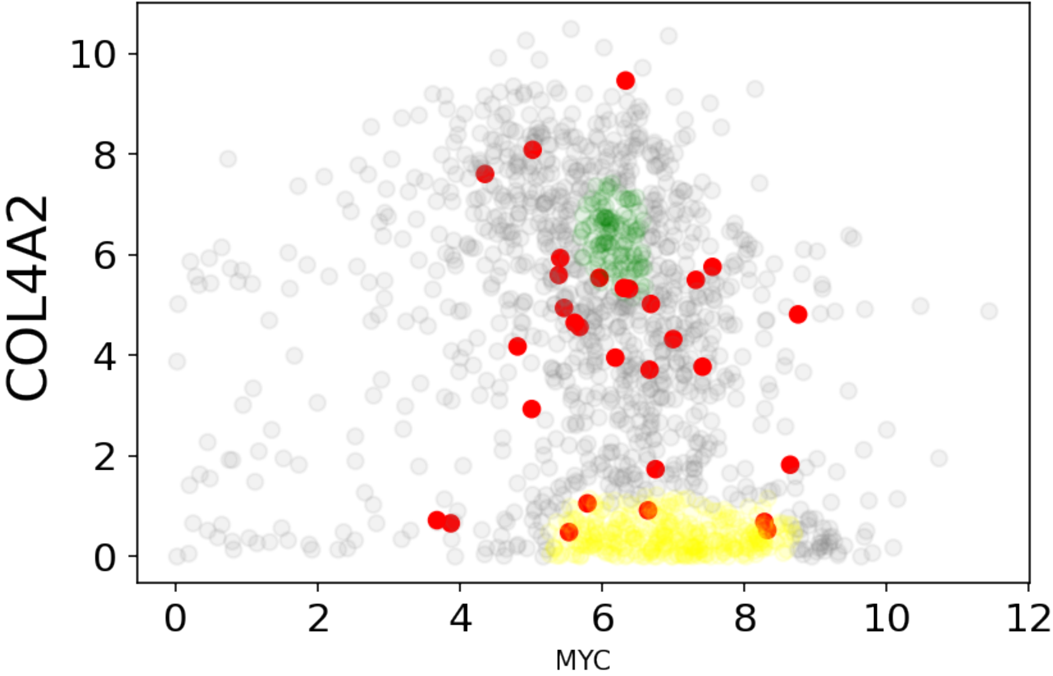

COL4A2
10
8
6
4
2
0
0
2
4
6
8
10
12
MYC

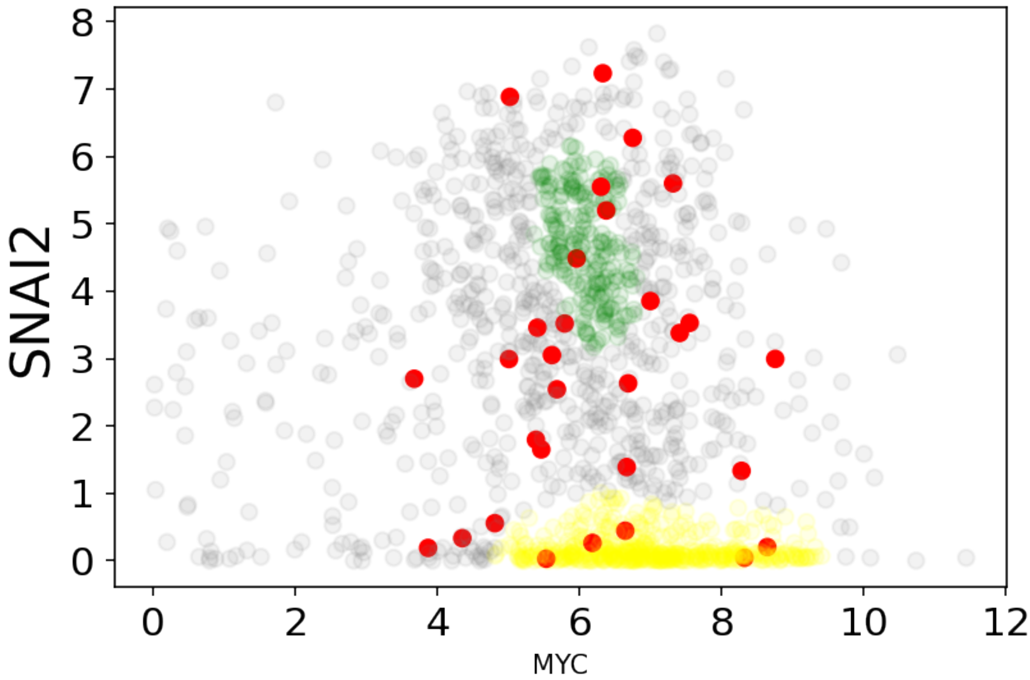

SNAI2
MYC
0
1
2
3
4
5
6
7
8
0
2
4
6
8
10
12

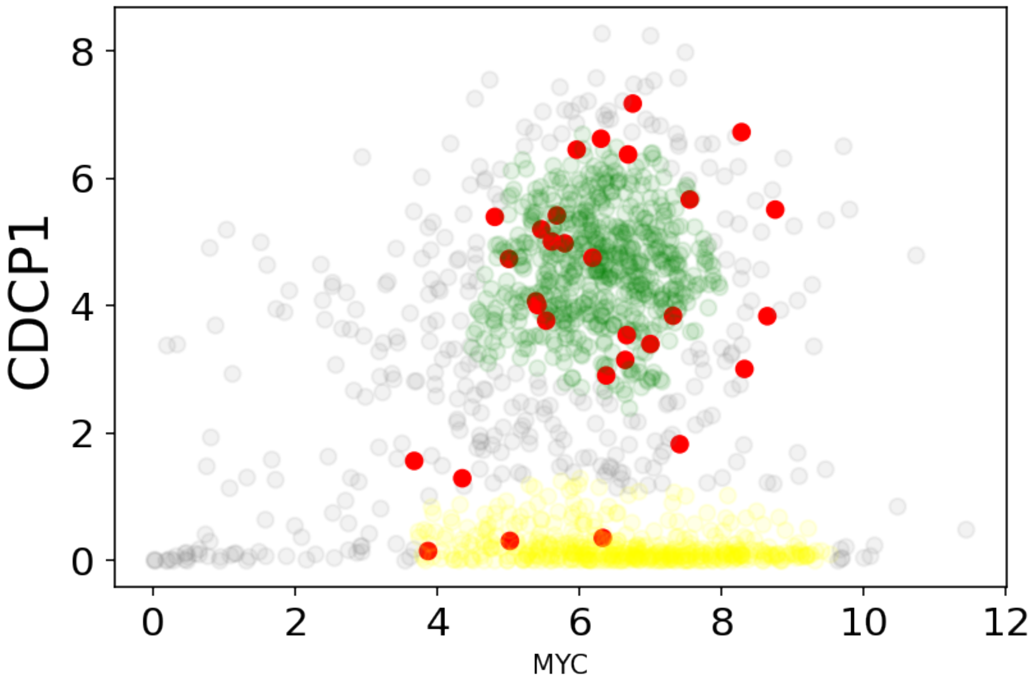
CDCP1
8
6
4
2
0
0
2
4
6
8
10
12
MYC

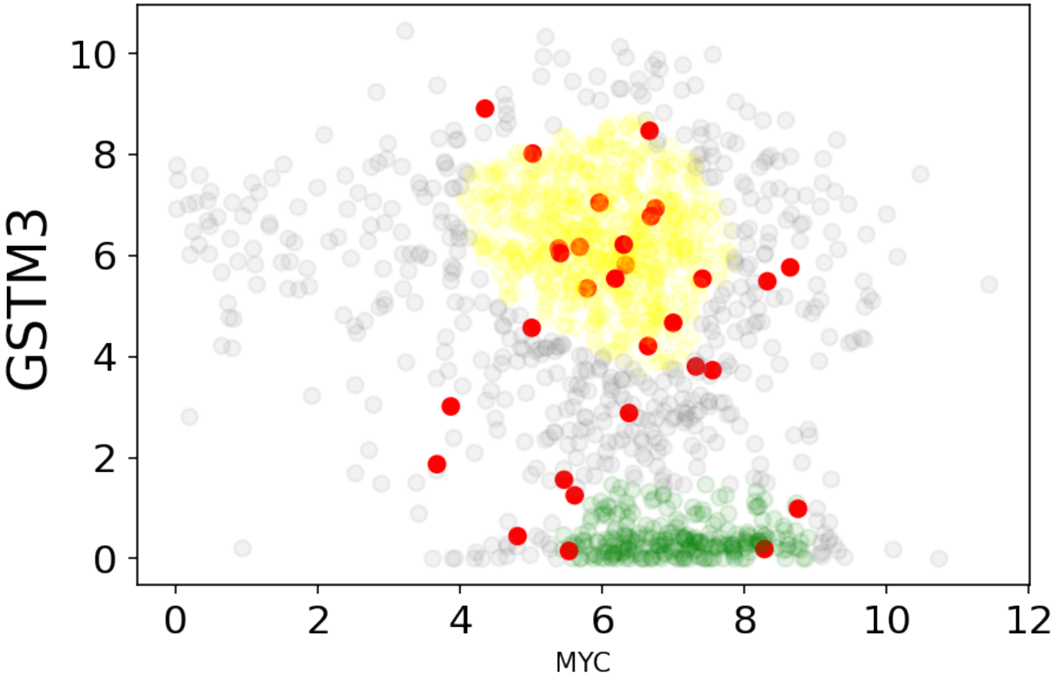
GSTM3
10
8
6
4
2
0
0
2
4
6
8
10
12
MYC

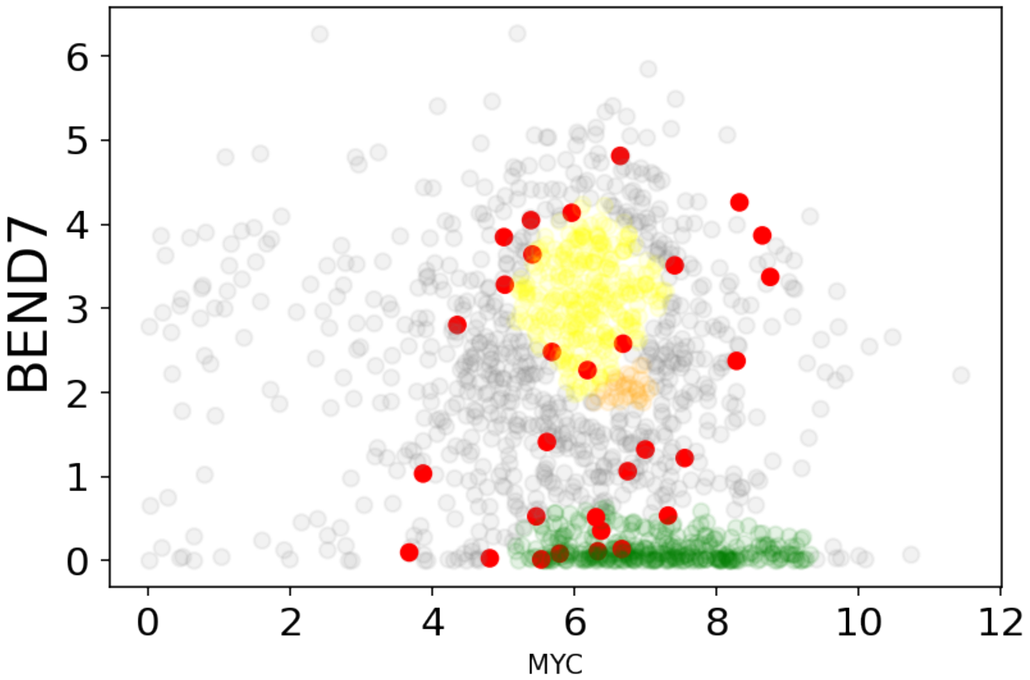

BEND7
MYC
0
1
2
3
4
5
6
0
2
4
6
8
10
12

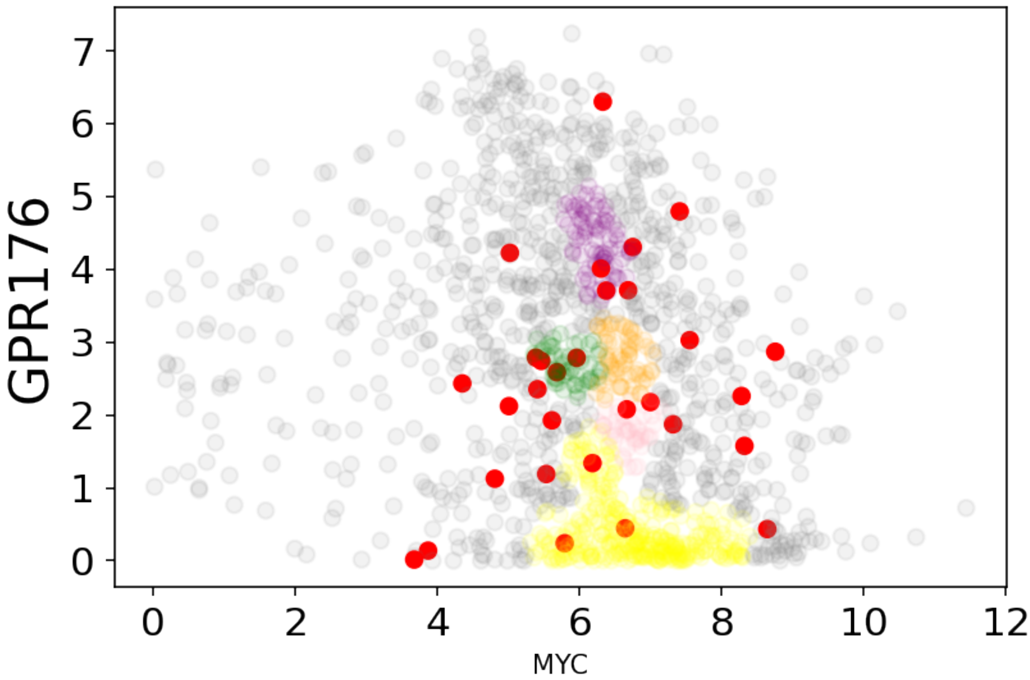
GPR176
MYC
0
1
2
3
4
5
6
7
0
2
4
6
8
10
12

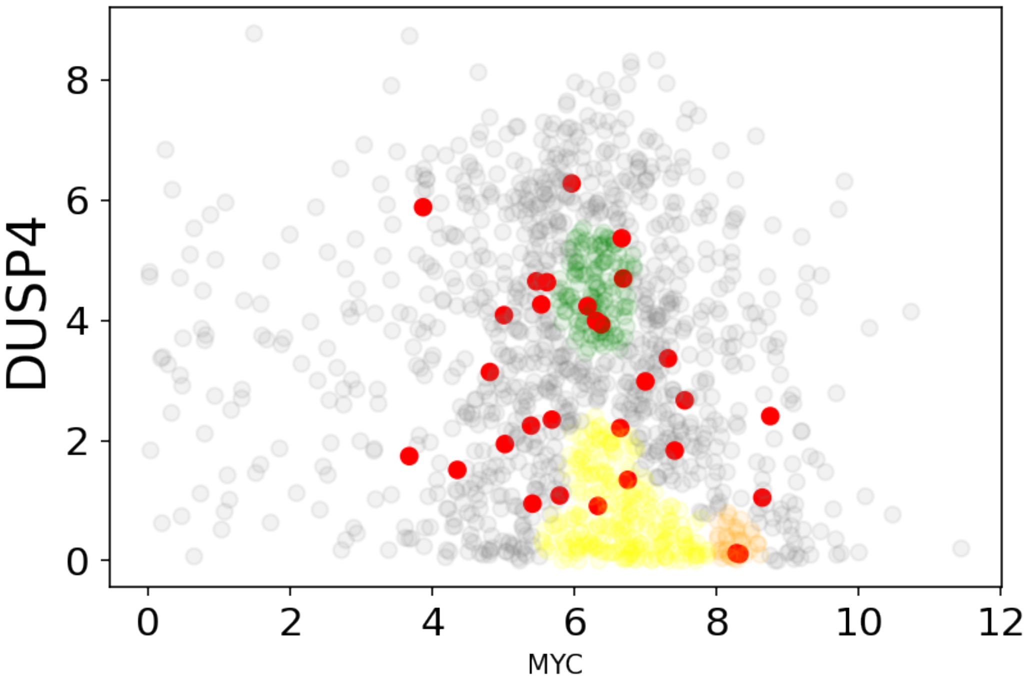

DUSP4
MYC
0
2
4
6
8
10
12

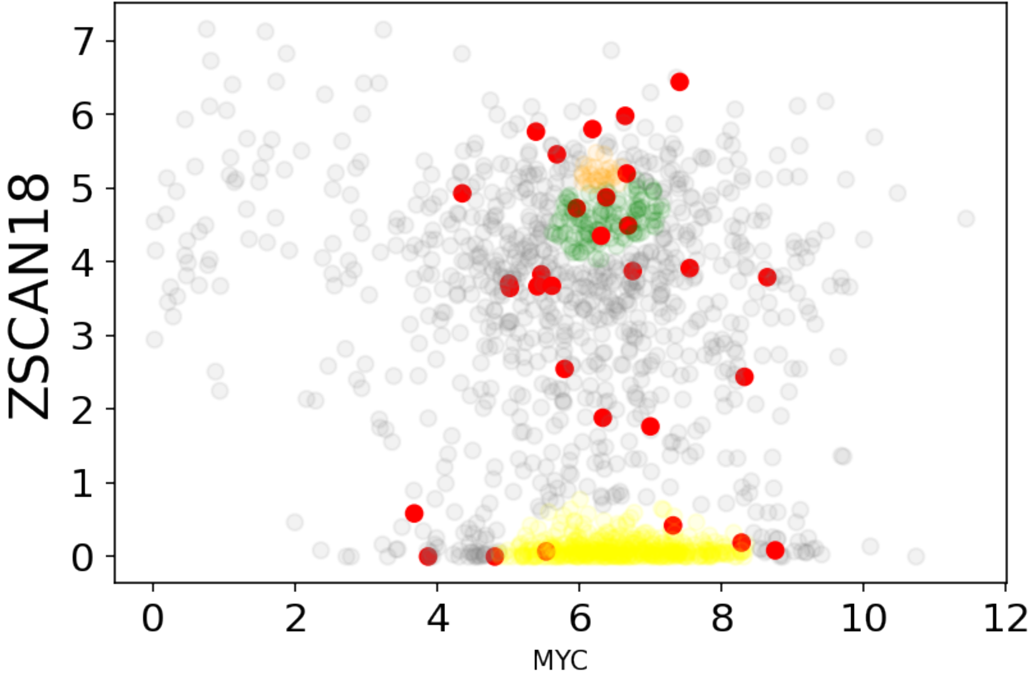

ZSCAN18
MYC
0
1
2
3
4
5
6
7
0
2
4
6
8
10
12

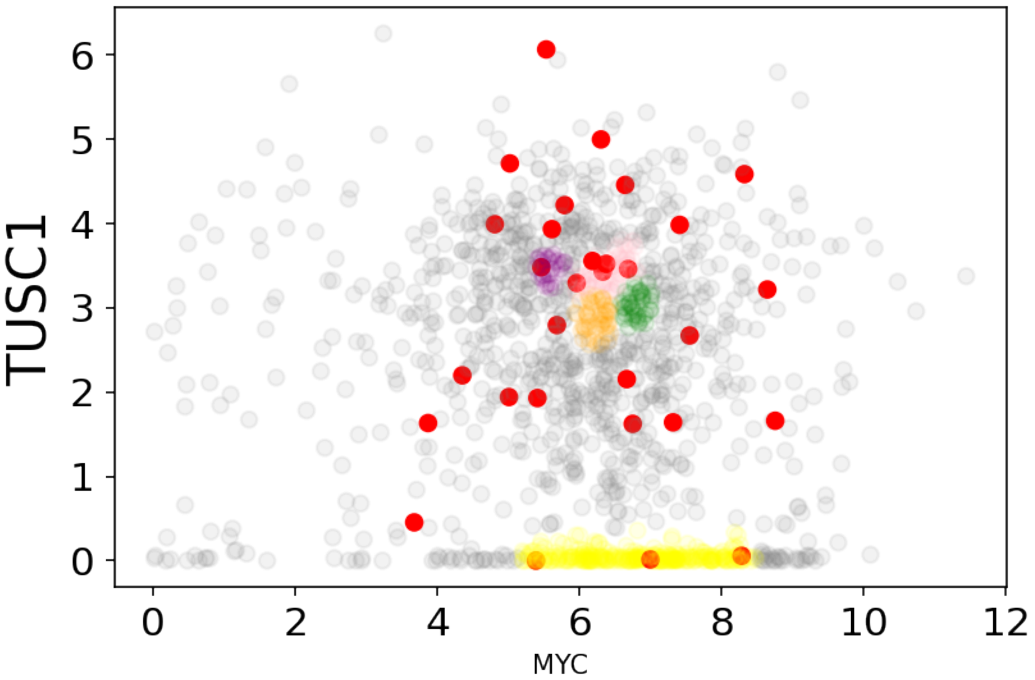

TUSC1
MYC
0
1
2
3
4
5
6
0
2
4
6
8
10
12

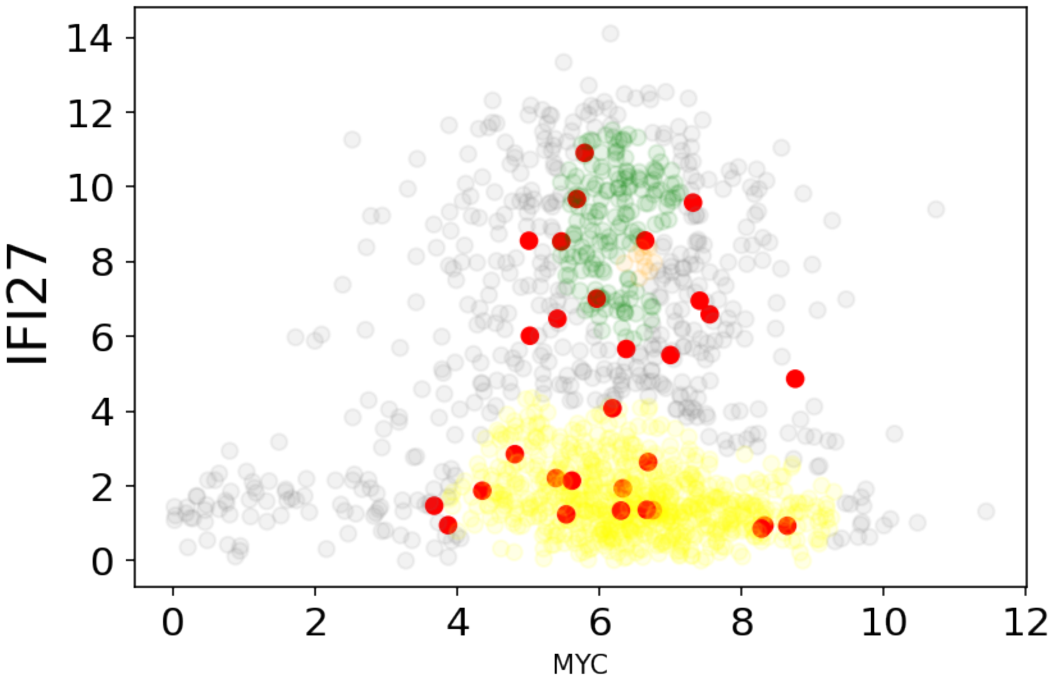

IFI27
14
12
10
8
6
4
2
0
0
2
4
6
8
10
12
MYC

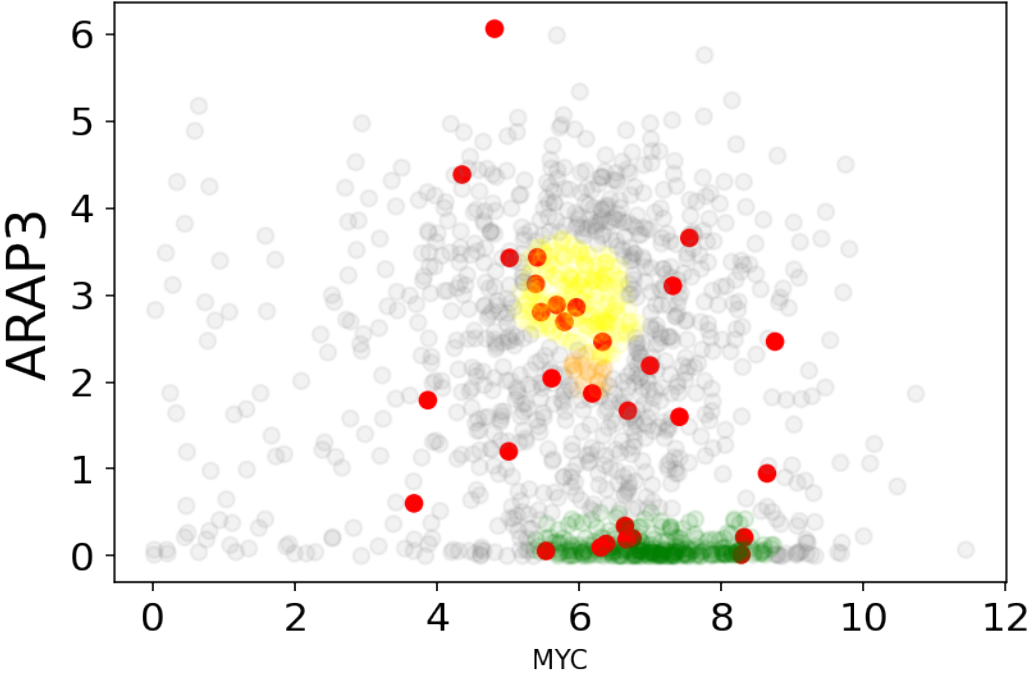
ARAP3
MYC
0
1
2
3
4
5
6
0
2
4
6
8
10
12

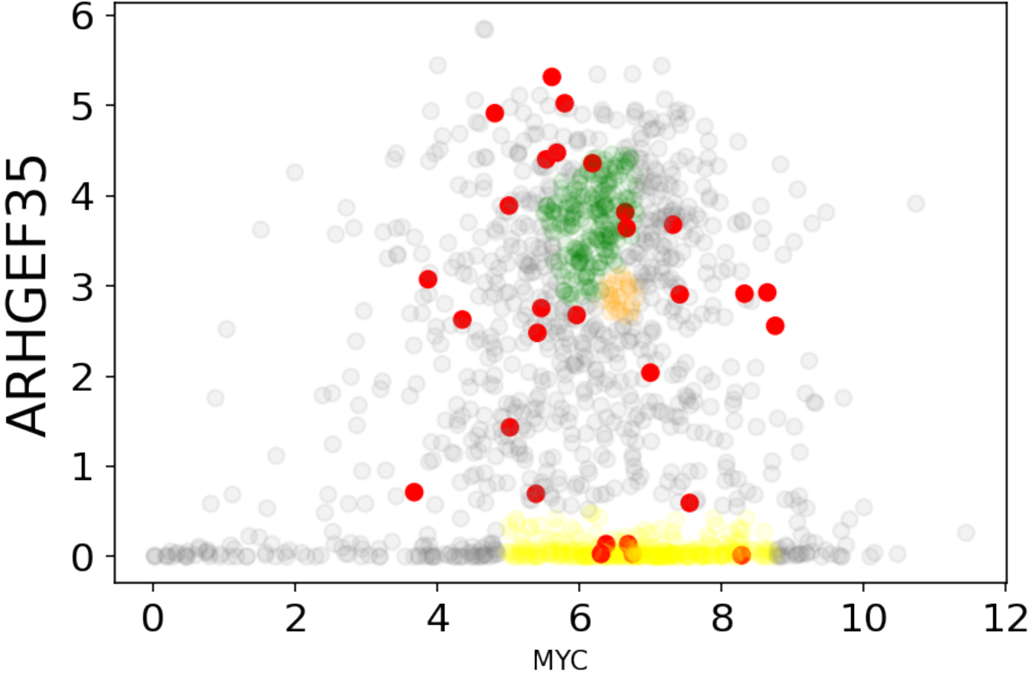

ARHGEF35
MYC
0
1
2
3
4
5
6
0
2
4
6
8
10
12

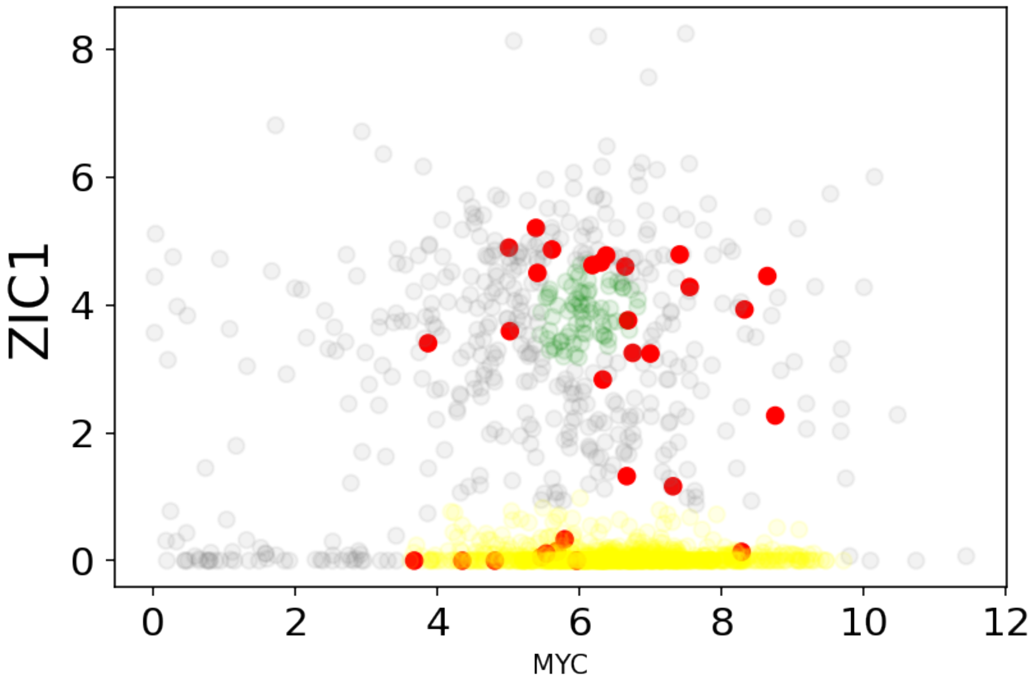

ZIC1
8
6
4
2
0
0
2
4
6
8
10
12
MYC

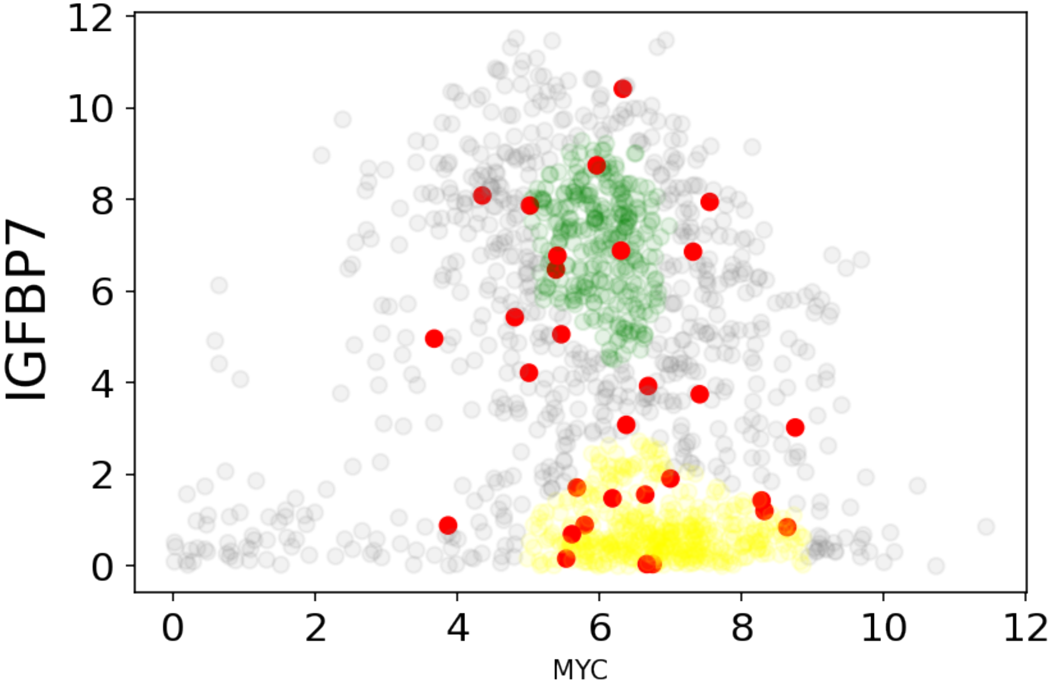

IGFBP7
MYC
0
2
4
6
8
10
12

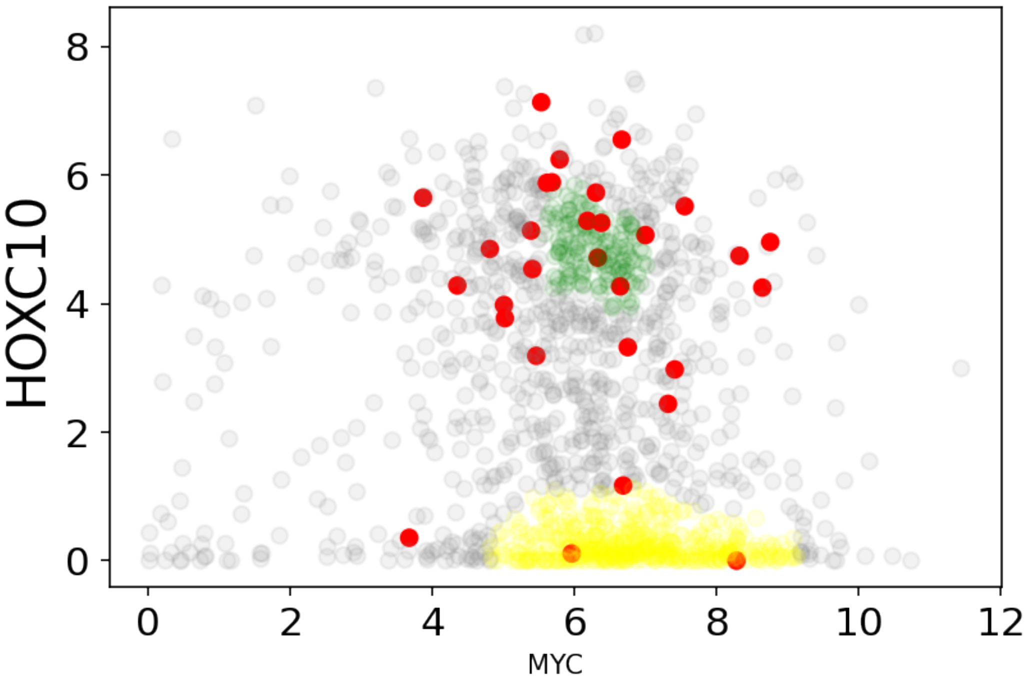

HOXC10
MYC
0
2
4
6
8
10
12

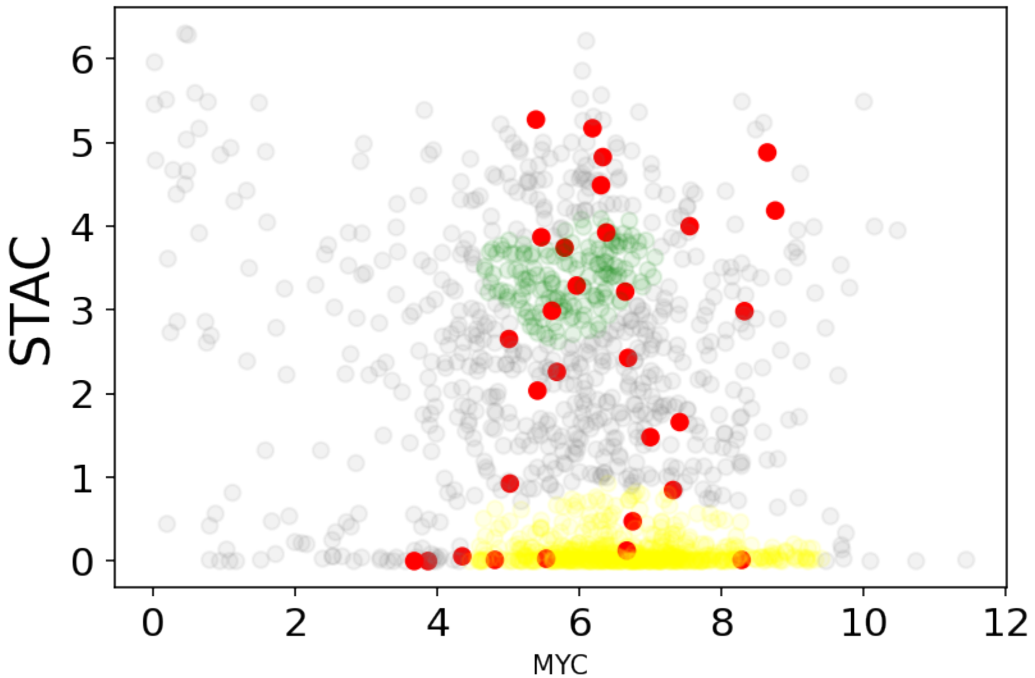

STAC
MYC
0
1
2
3
4
5
6
0
2
4
6
8
10
12

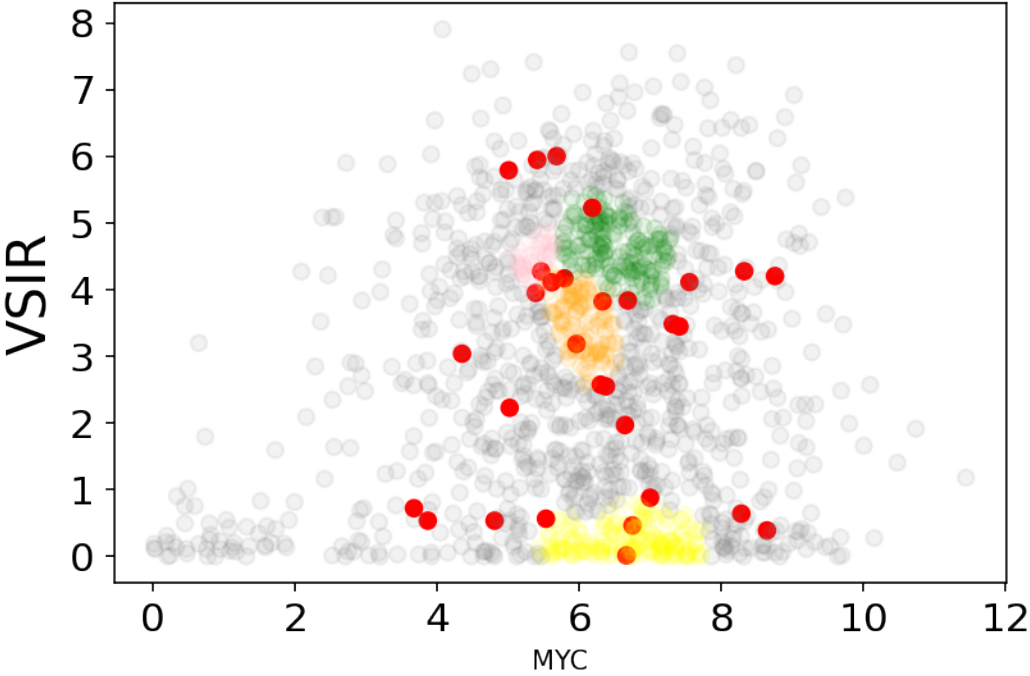

VSIR
MYC
0
1
2
3
4
5
6
7
8
0
2
4
6
8
10
12

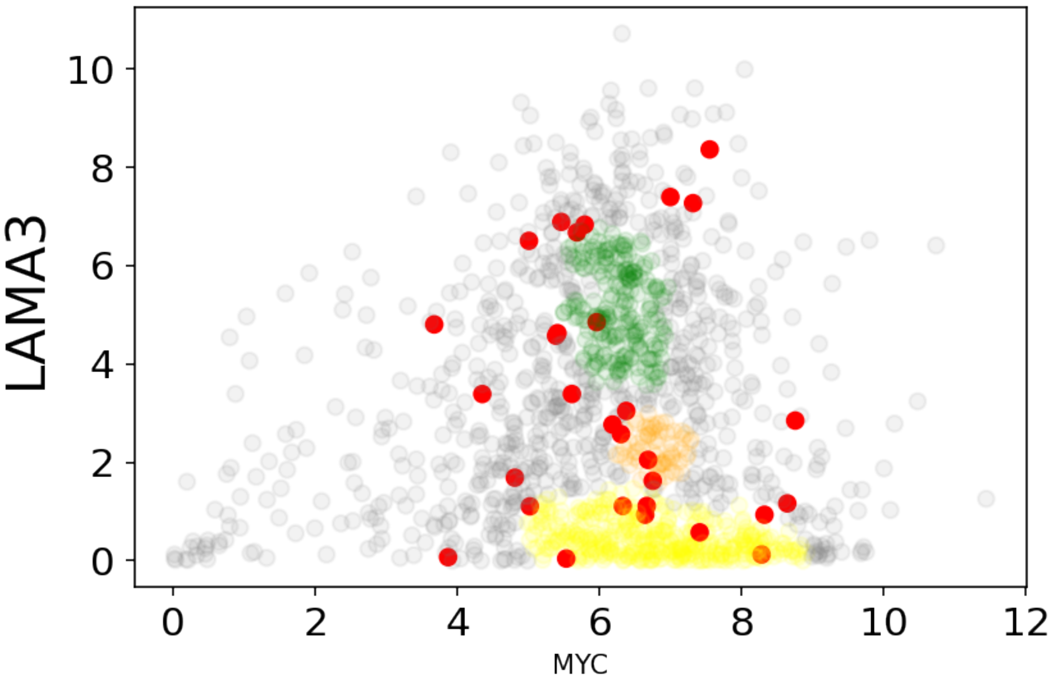

LAMA3
MYC
0
2
4
6
8
10
12

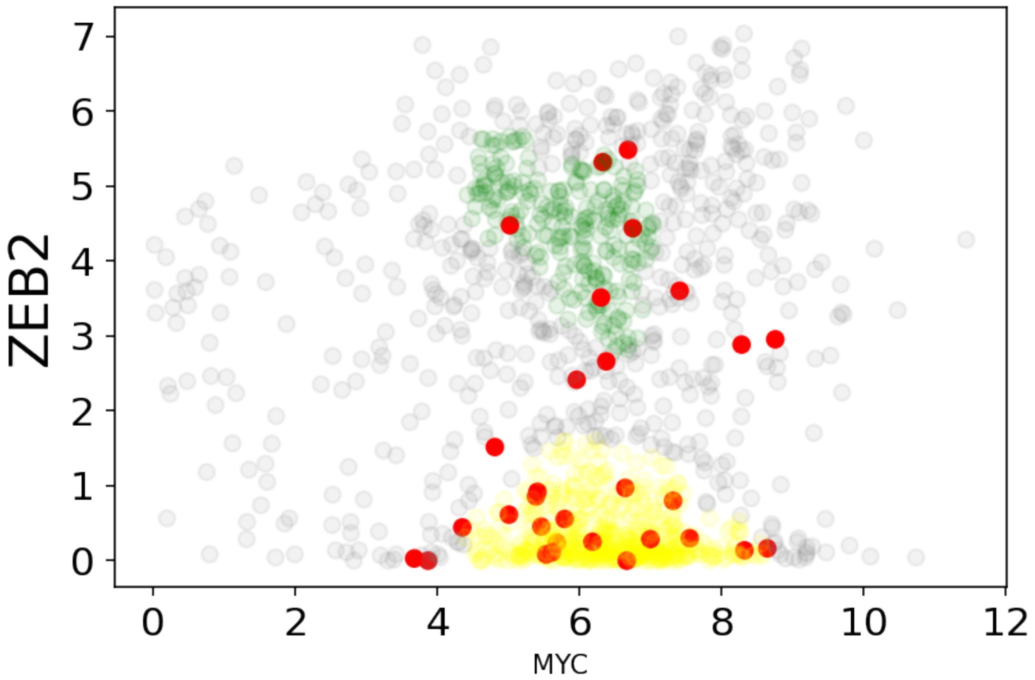
ZEB2
MYC
0
1
2
3
4
5
6
7
0
2
4
6
8
10
12

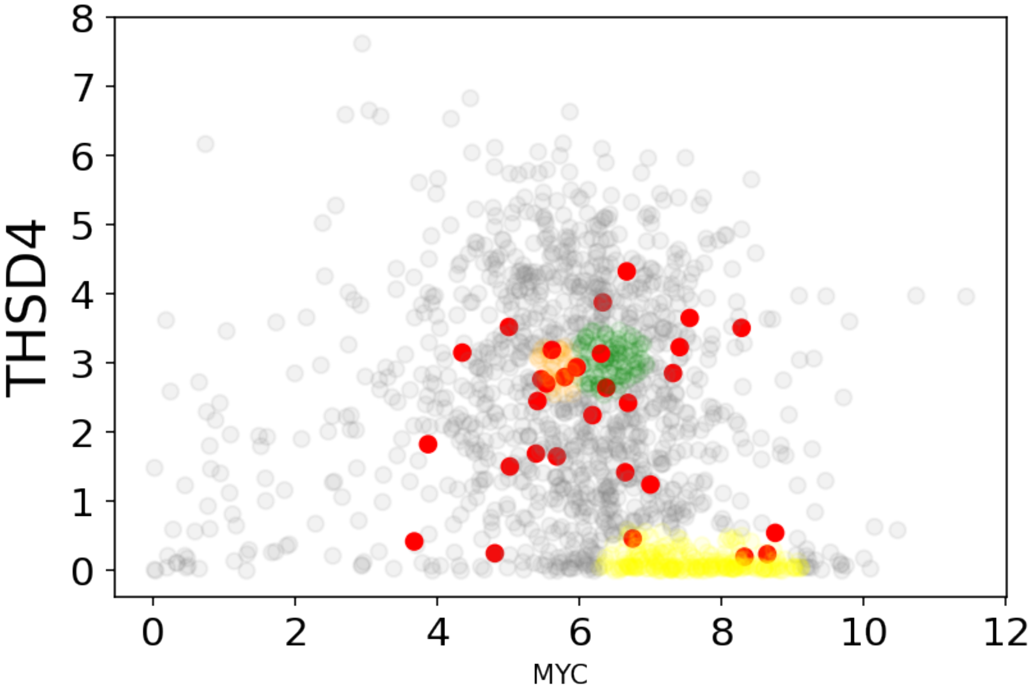
THSD4
MYC
0
1
2
3
4
5
6
7
8
0
2
4
6
8
10
12

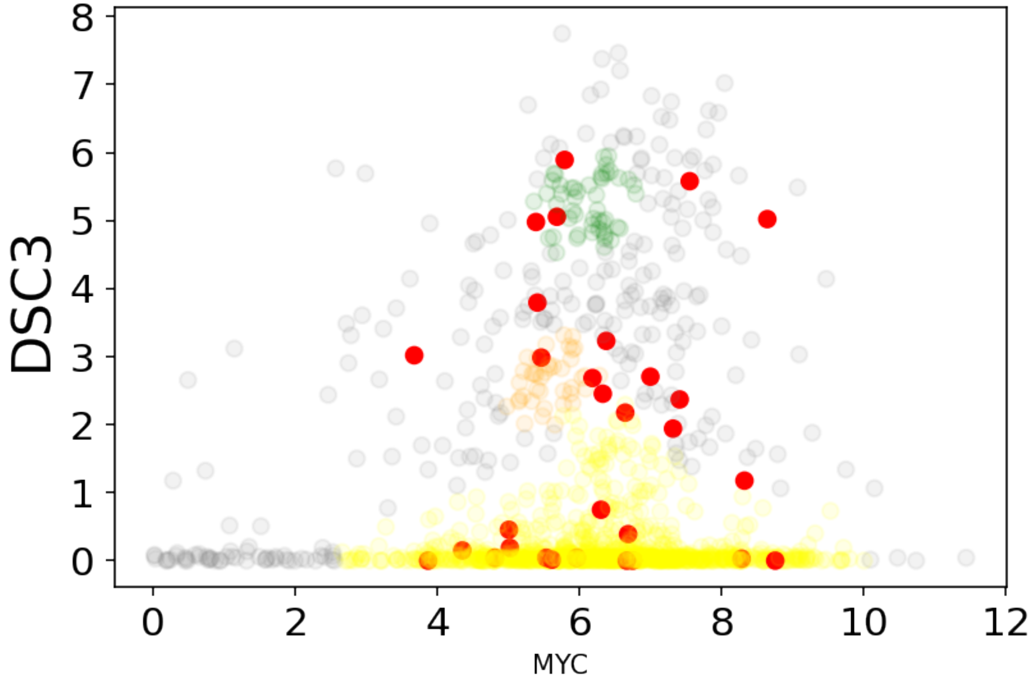

DSC3
MYC
0
1
2
3
4
5
6
7
8
0
2
4
6
8
10
12

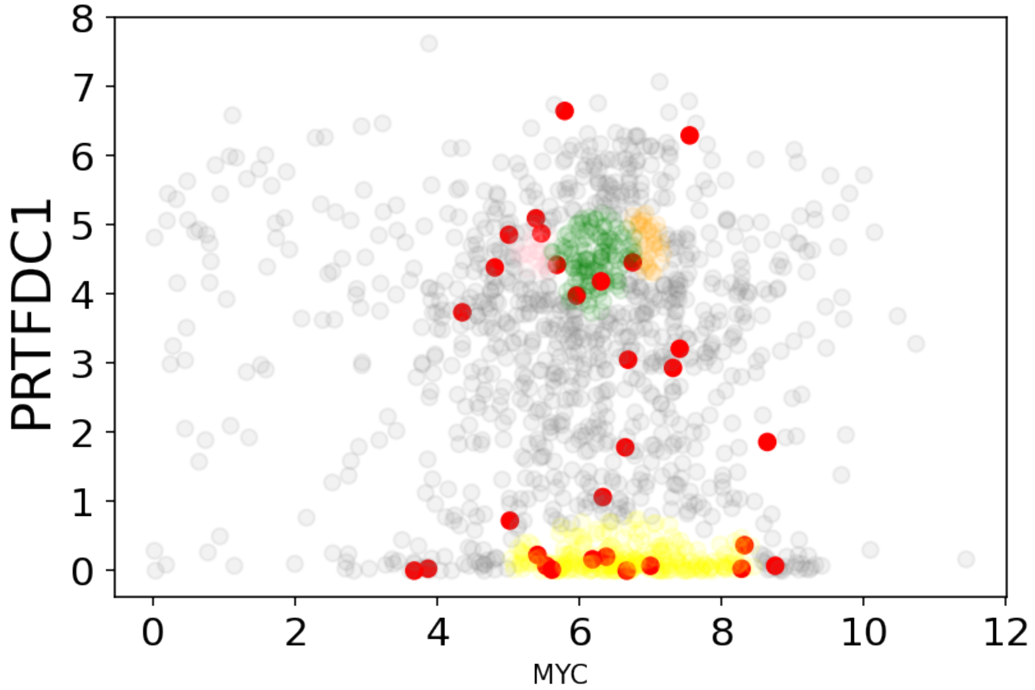
PRTFDC1
MYC
0
1
2
3
4
5
6
7
8
0
2
4
6
8
10
12

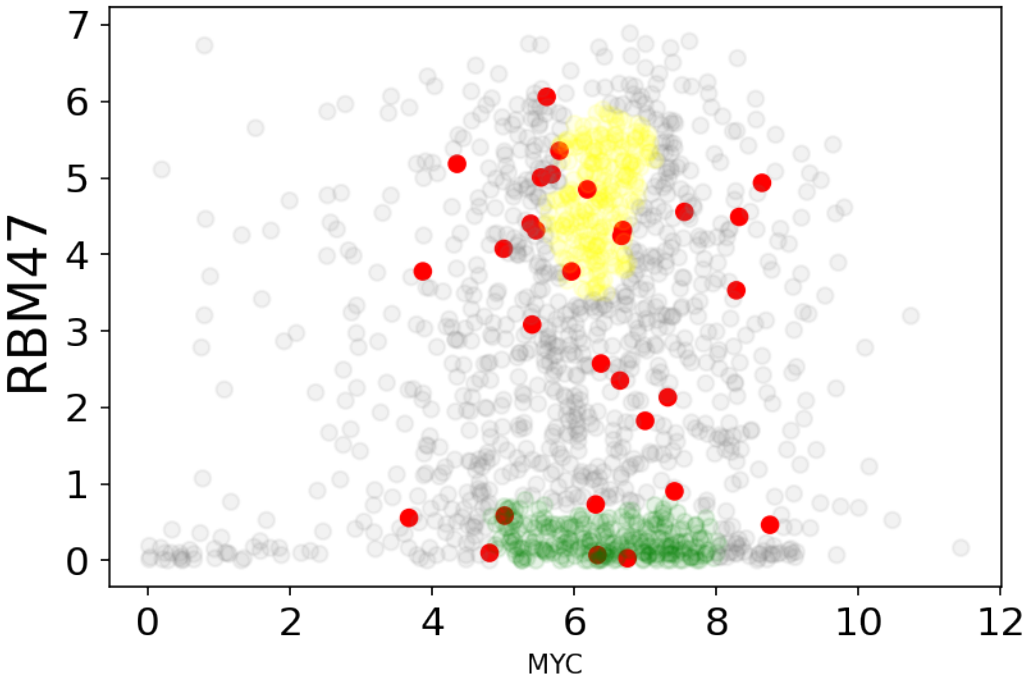

RBM47
MYC
0
1
2
3
4
5
6
7
0
2
4
6
8
10
12

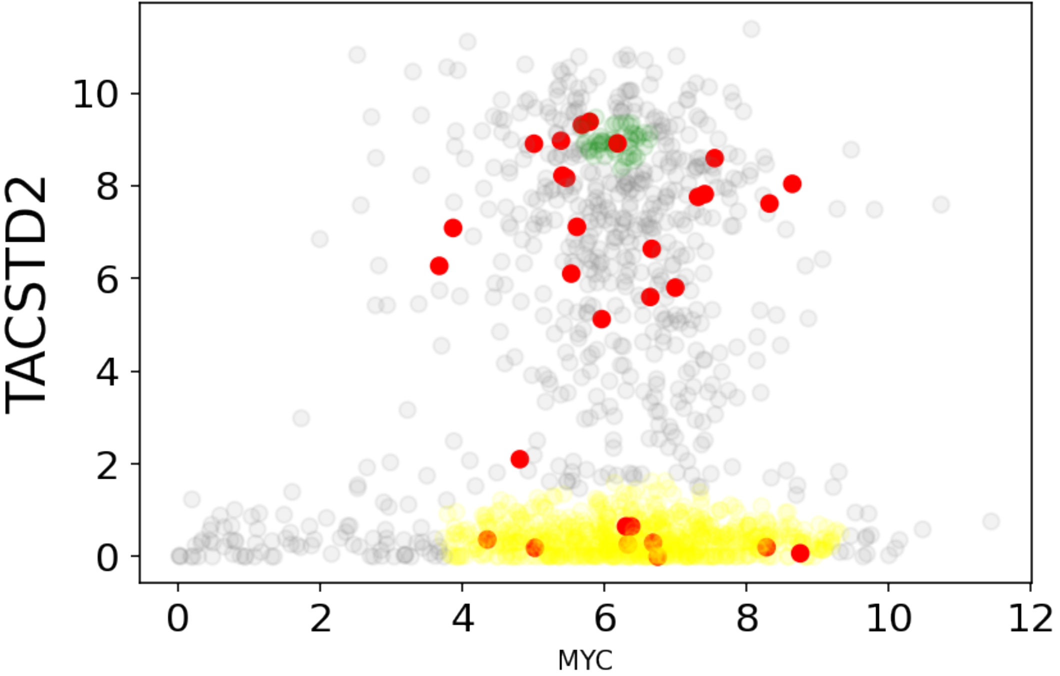
TACSTD2
10
8
6
4
2
0
0
2
4
6
8
10
12
MYC

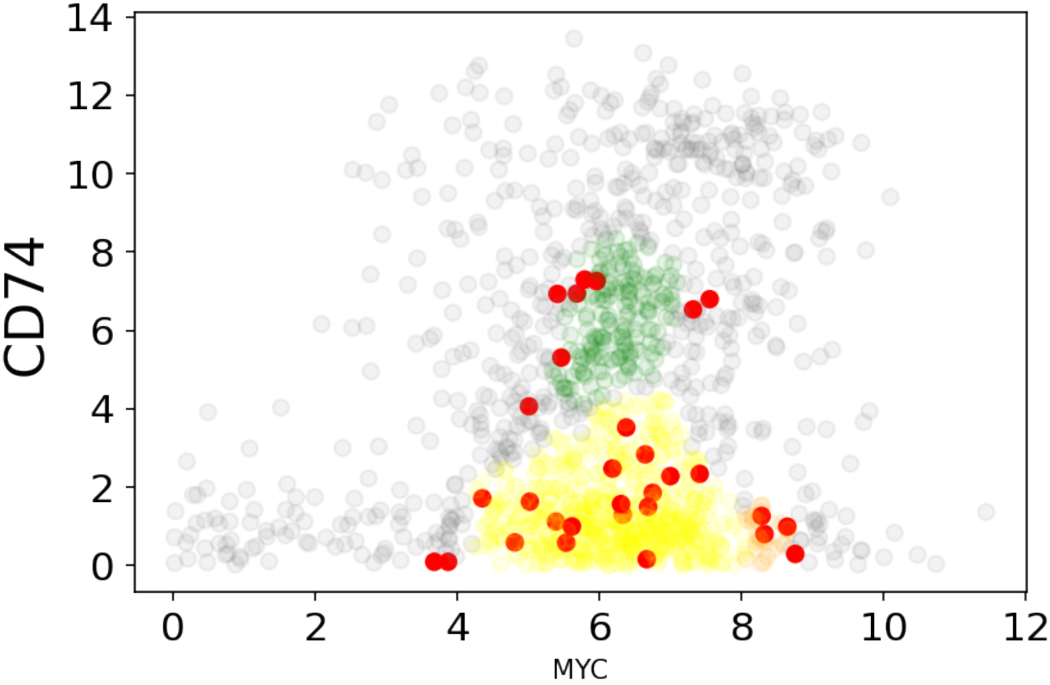

CD74
MYC
0
2
4
6
8
10
12
14

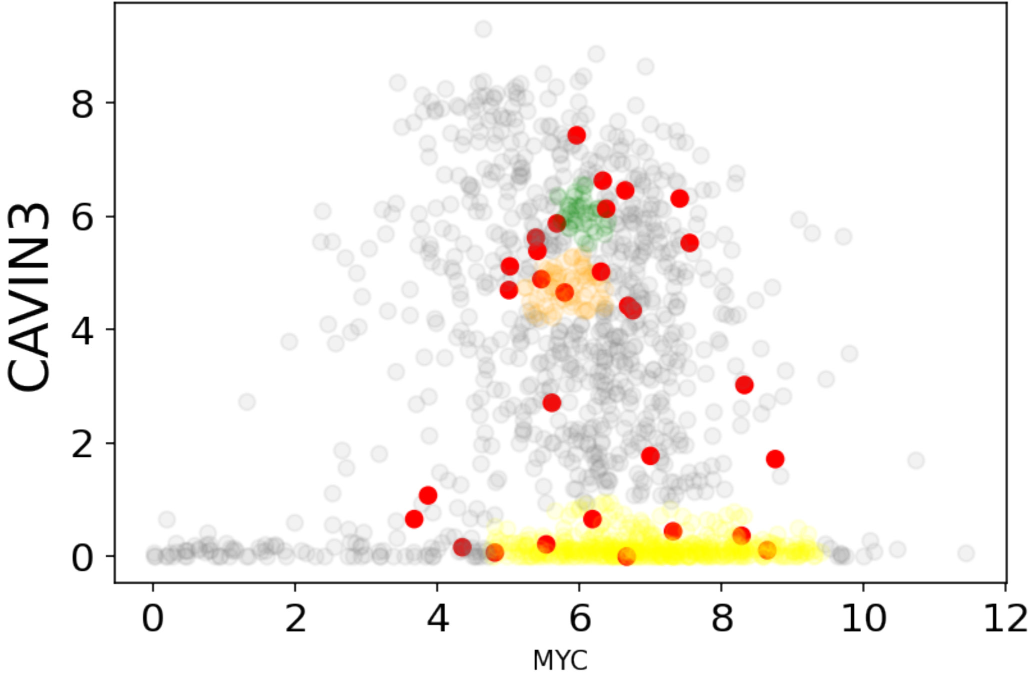
CAVIN3
MYC
0
2
4
6
8
0
2
4
6
8
10
12

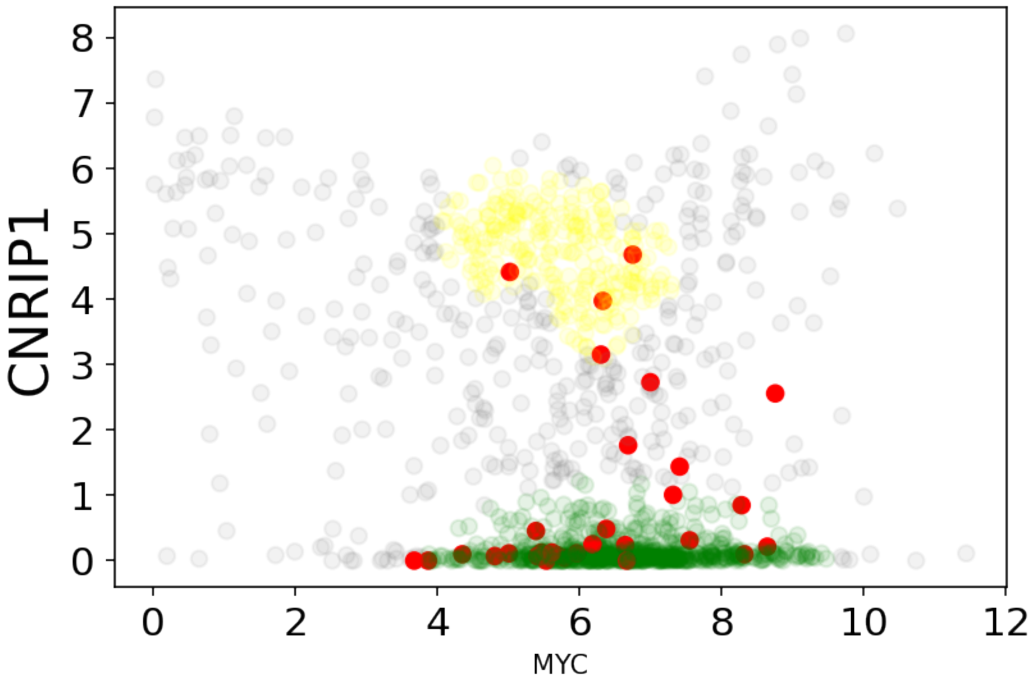
CNRIP1
MYC
0
1
2
3
4
5
6
7
8
0
2
4
6
8
10
12

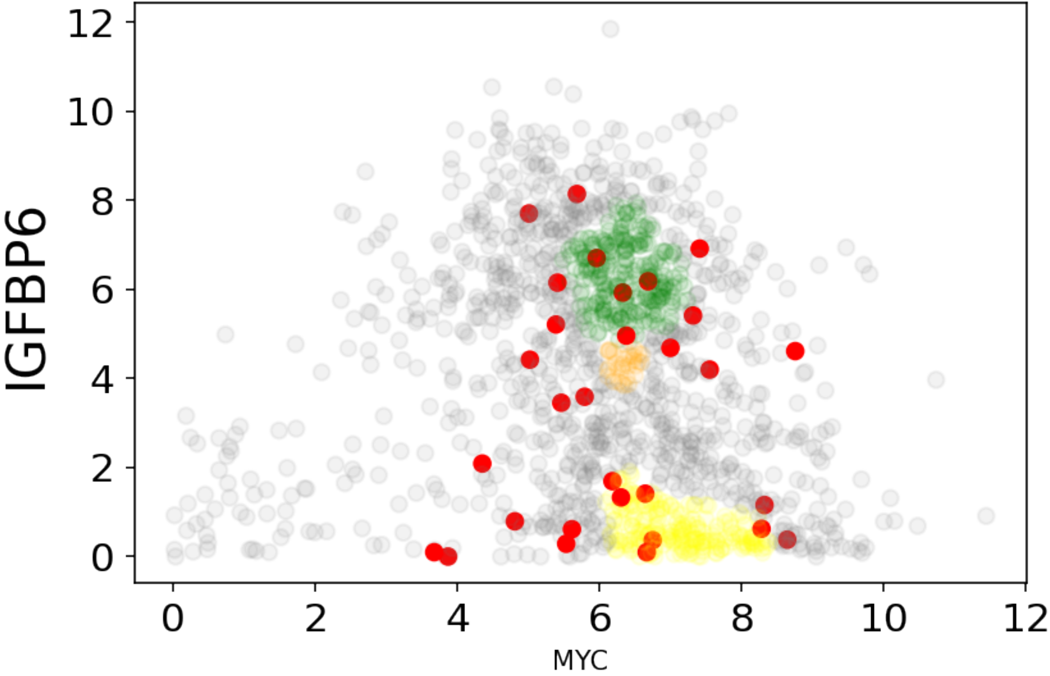

IGFBP6
MYC
0
2
4
6
8
10
12

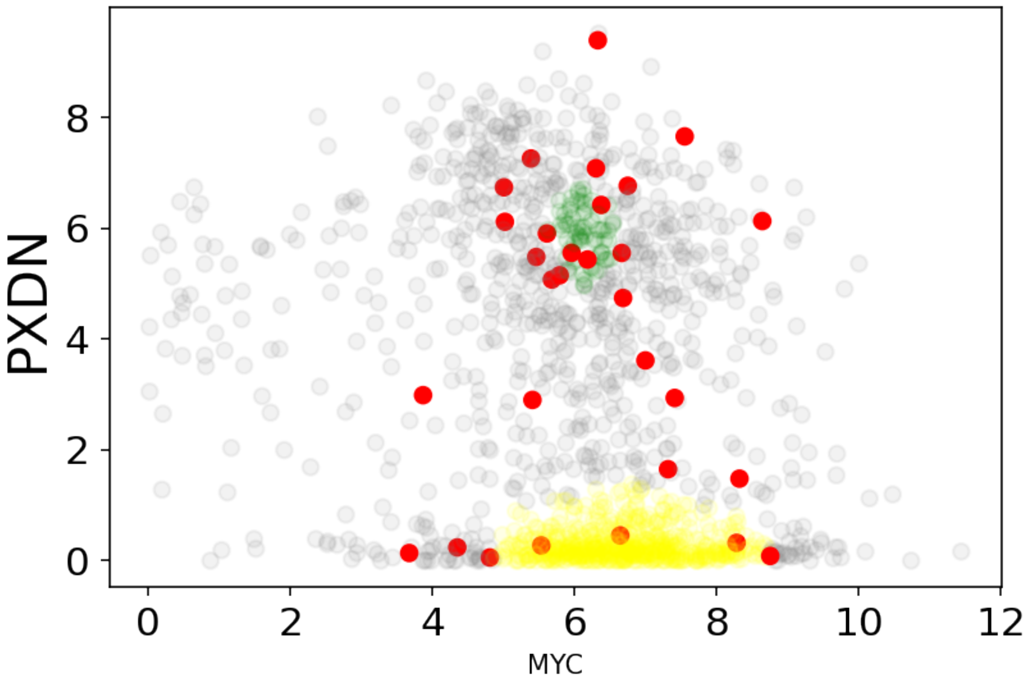

PXDN
MYC
0
2
4
6
8
0
2
4
6
8
10
12

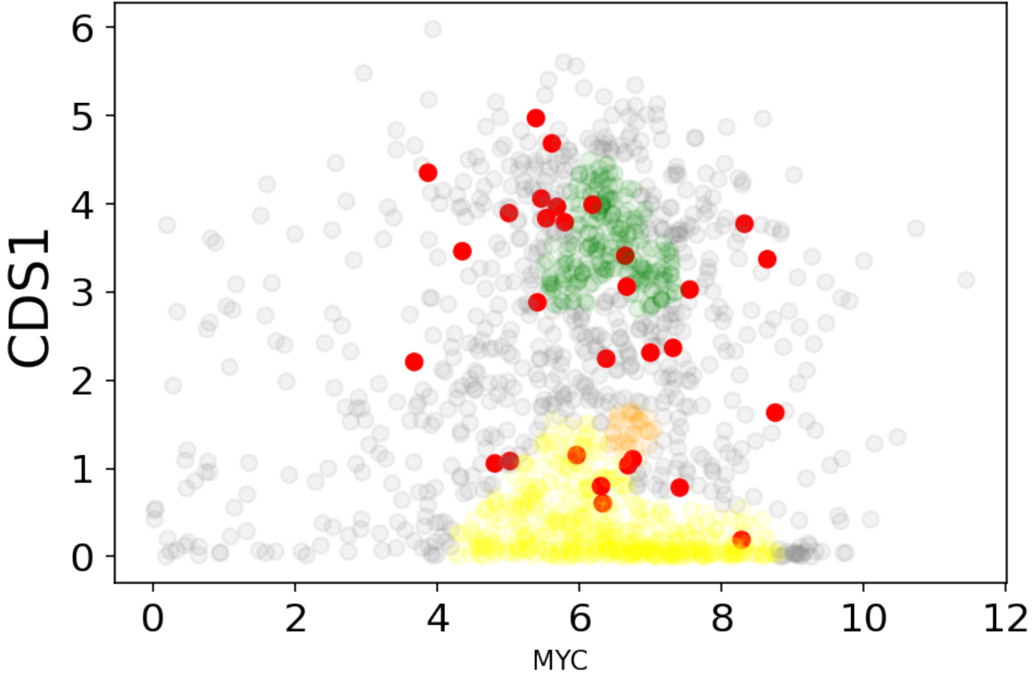

CDS1
MYC
0
1
2
3
4
5
6
0
2
4
6
8
10
12

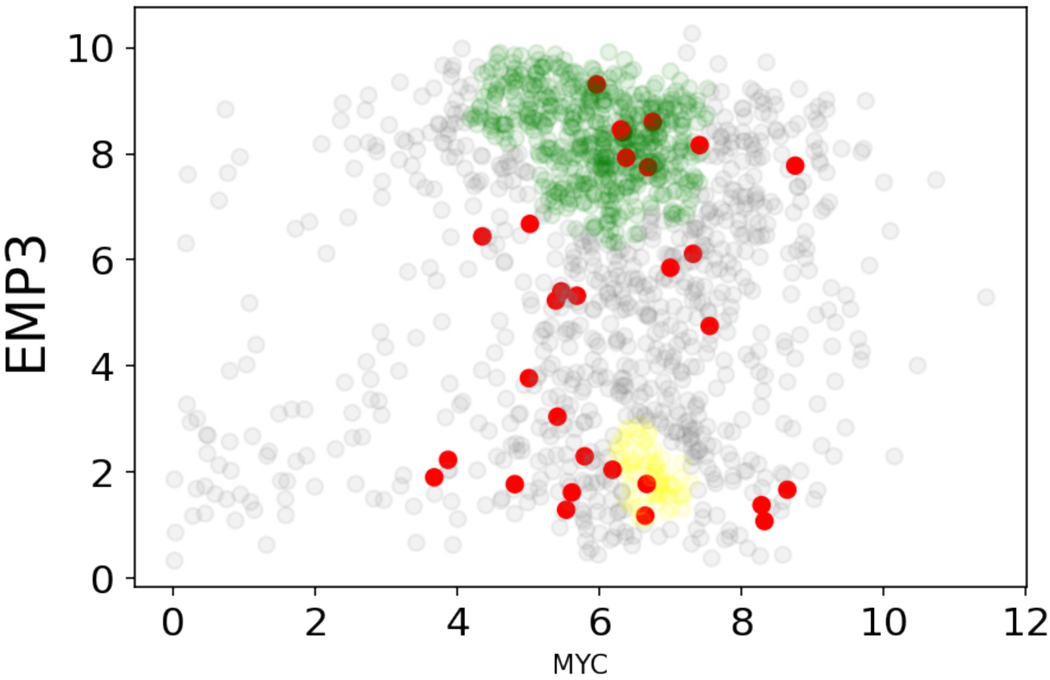

EMP3
MYC
0
2
4
6
8
10
12

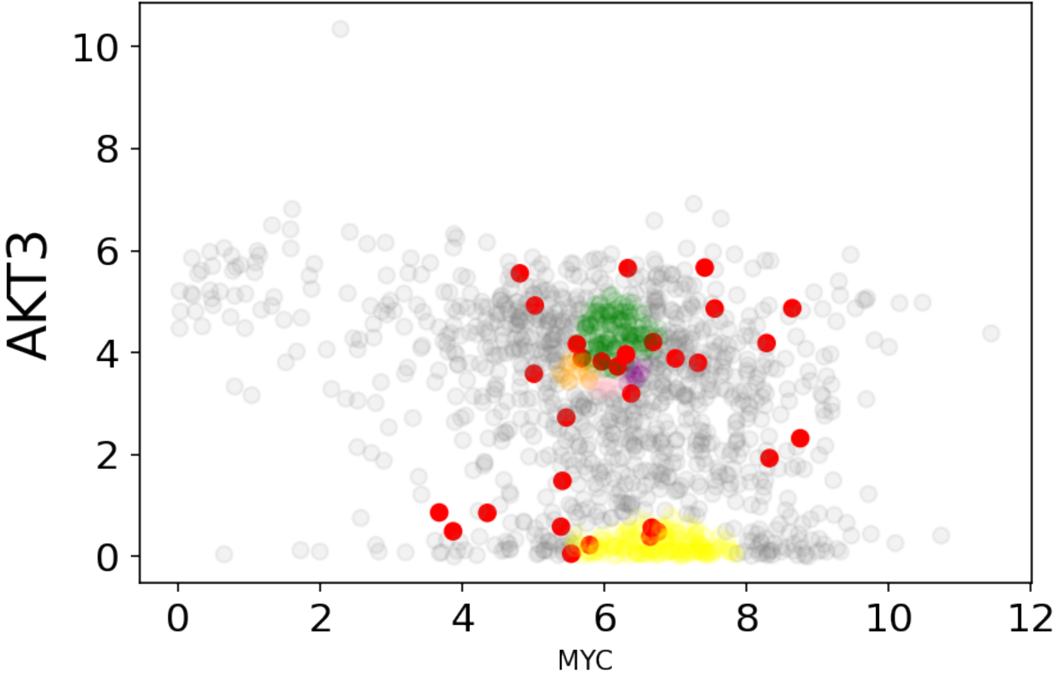
AKT3
MYC
0
2
4
6
8
10
12
0
2
4
6
8
10

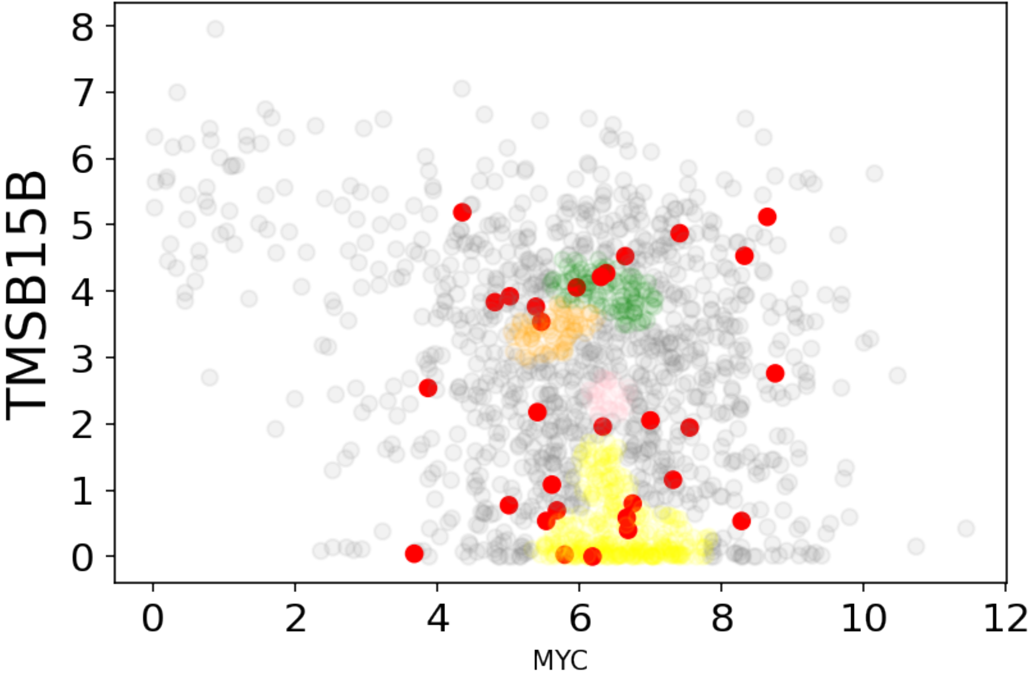

TMSB15B
MYC
0
1
2
3
4
5
6
7
8
0
2
4
6
8
10
12

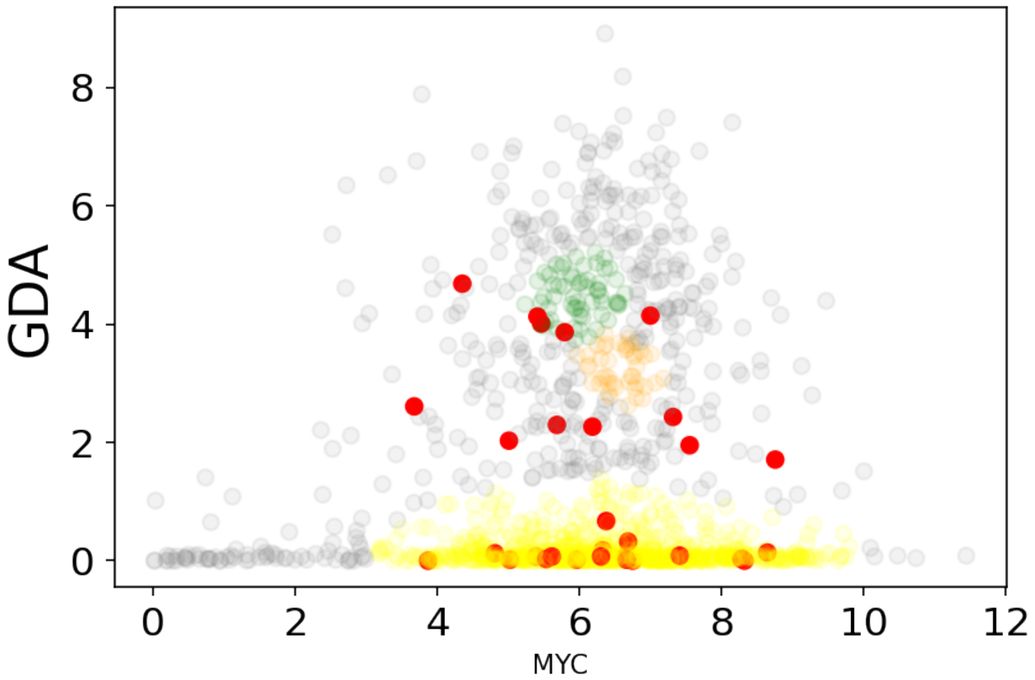
GDA
8
6
4
2
0
0
2
4
6
8
10
12
MYC

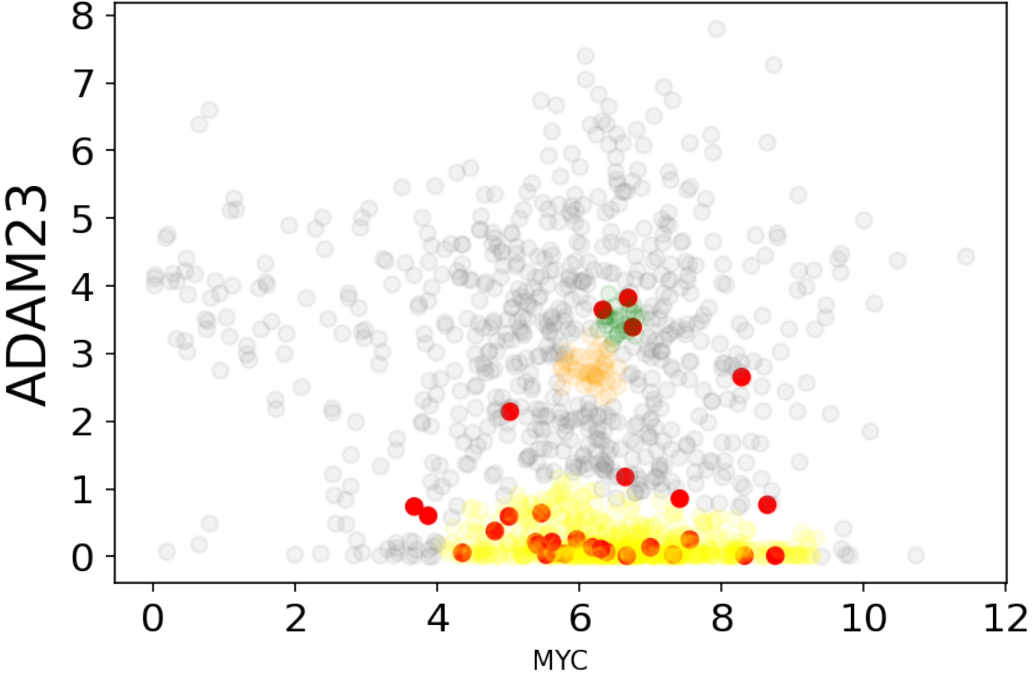
ADAM23
MYC
0
1
2
3
4
5
6
7
8
0
2
4
6
8
10
12

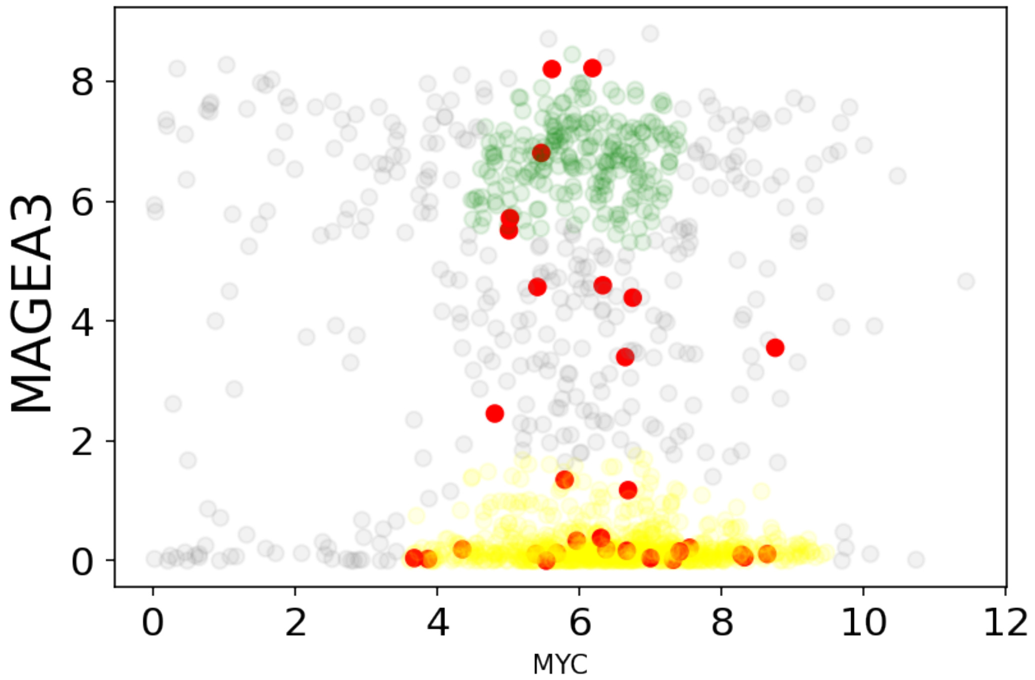

MAGEA3
0
2
4
6
8
0
2
4
6
8
10
12
MYC

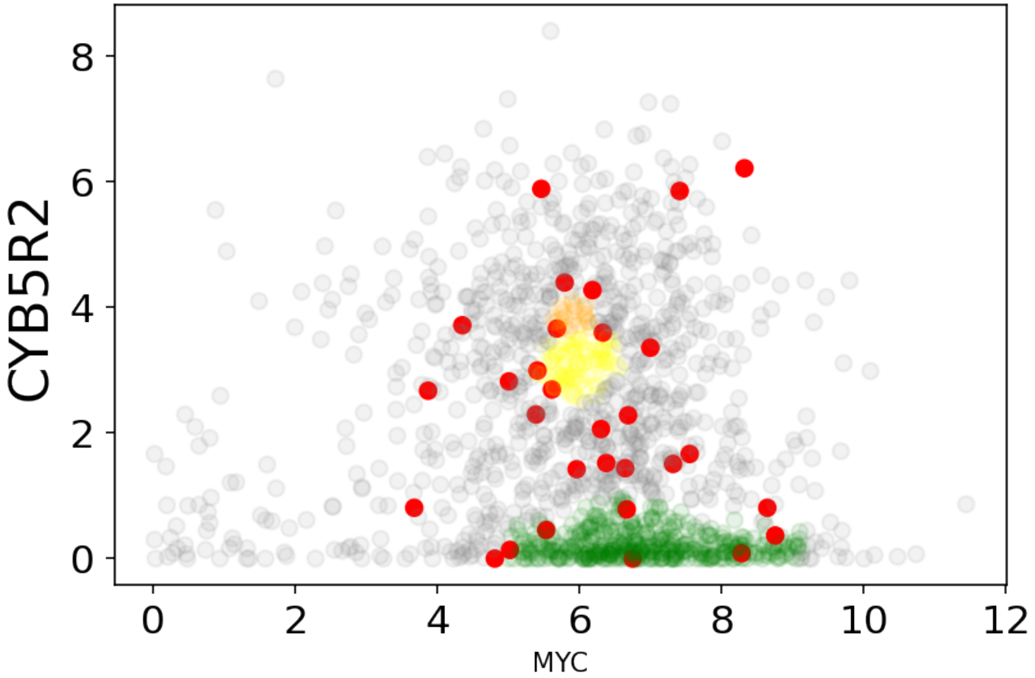

CYB5R2
0
2
4
6
8
0
2
4
6
8
10
12
MYC

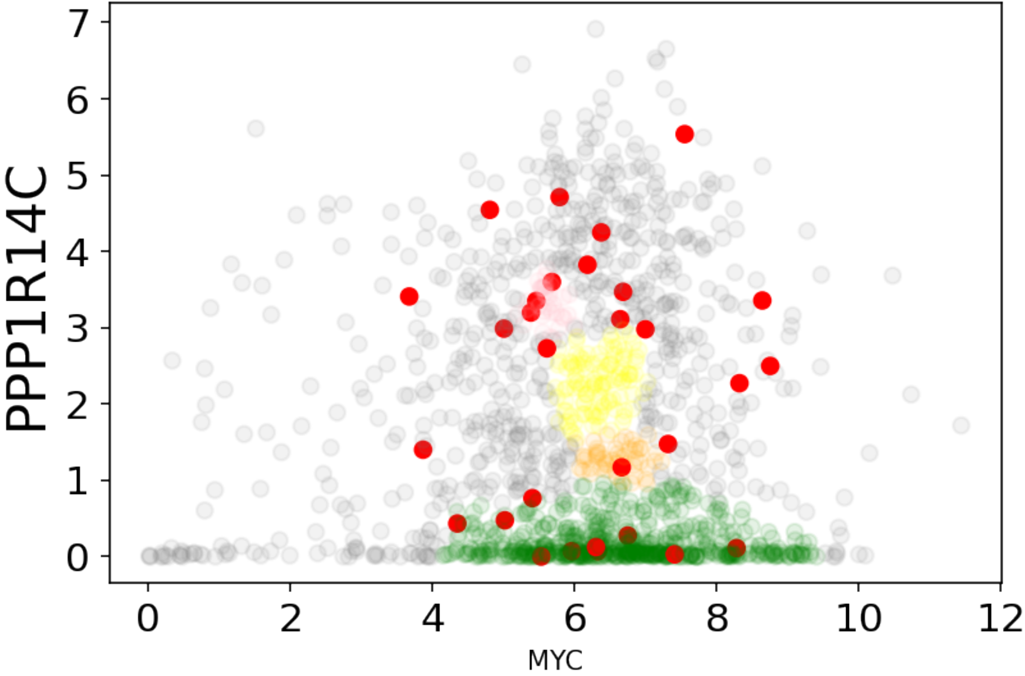

PPP1R14C
MYC
0
1
2
3
4
5
6
7
0
2
4
6
8
10
12

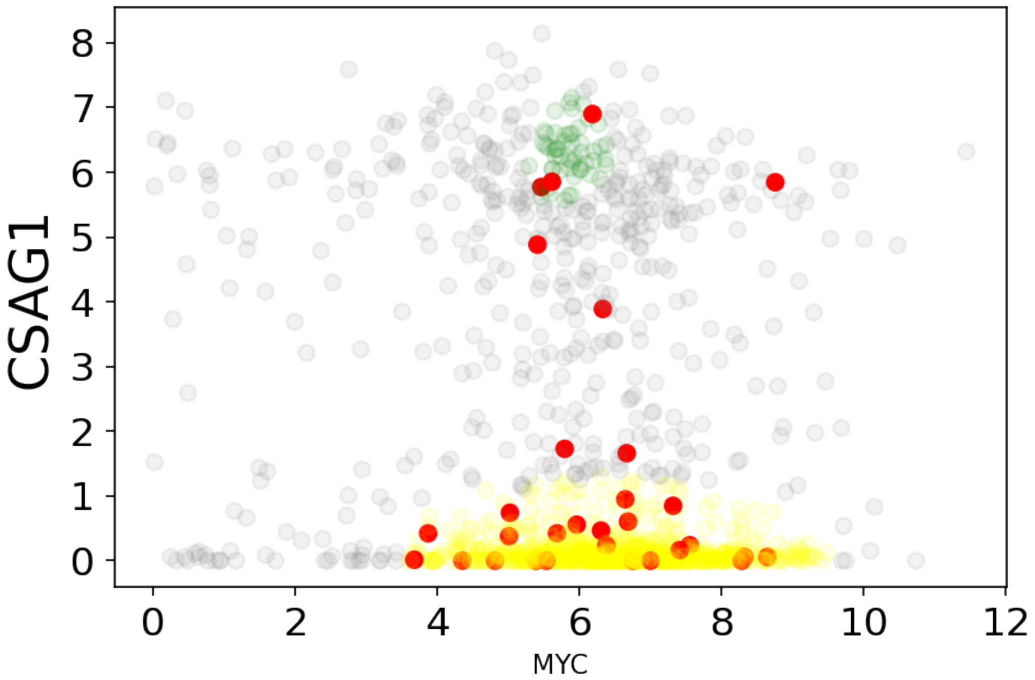

CSAG1
0
1
2
3
4
5
6
7
8
0
2
4
6
8
10
12
MYC

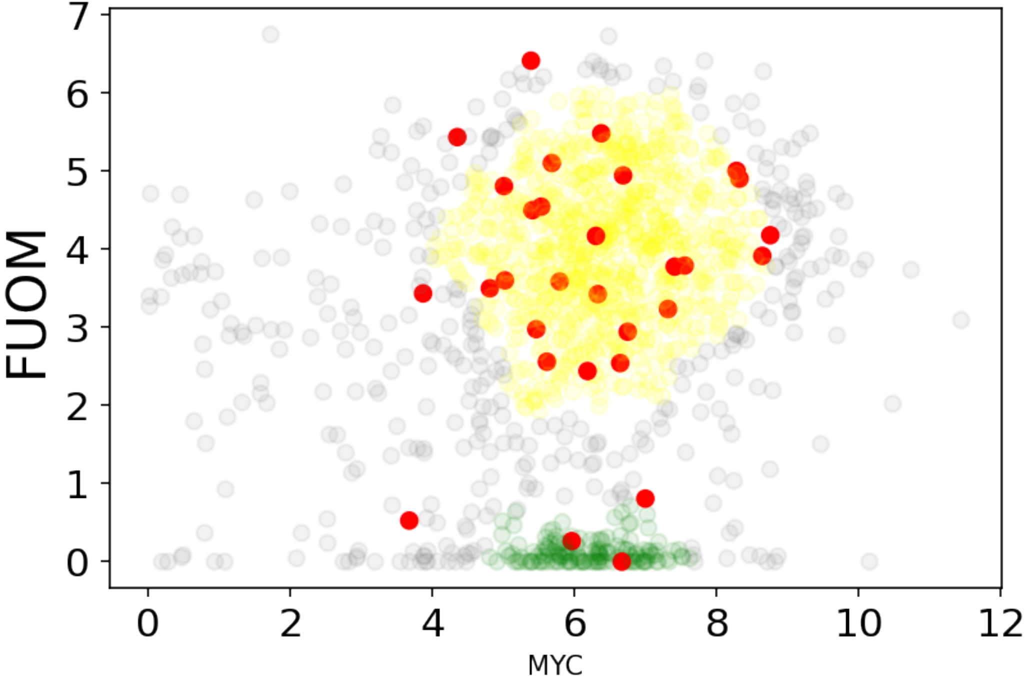
FUOM
MYC
0
1
2
3
4
5
6
7
0
2
4
6
8
10
12

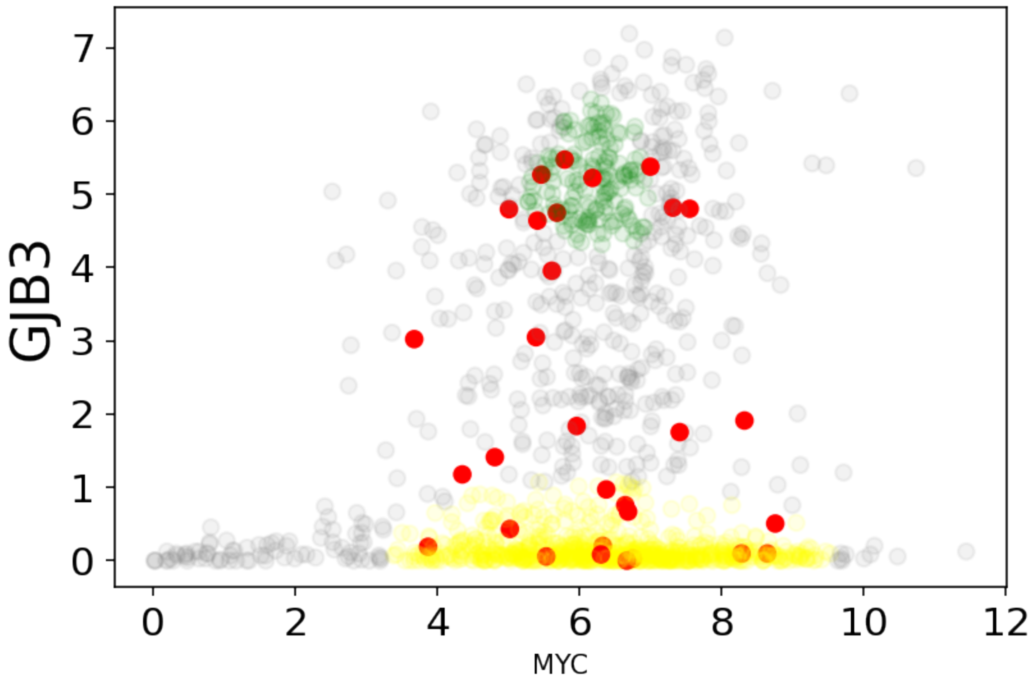
GJB3
MYC
0
1
2
3
4
5
6
7
0
2
4
6
8
10
12

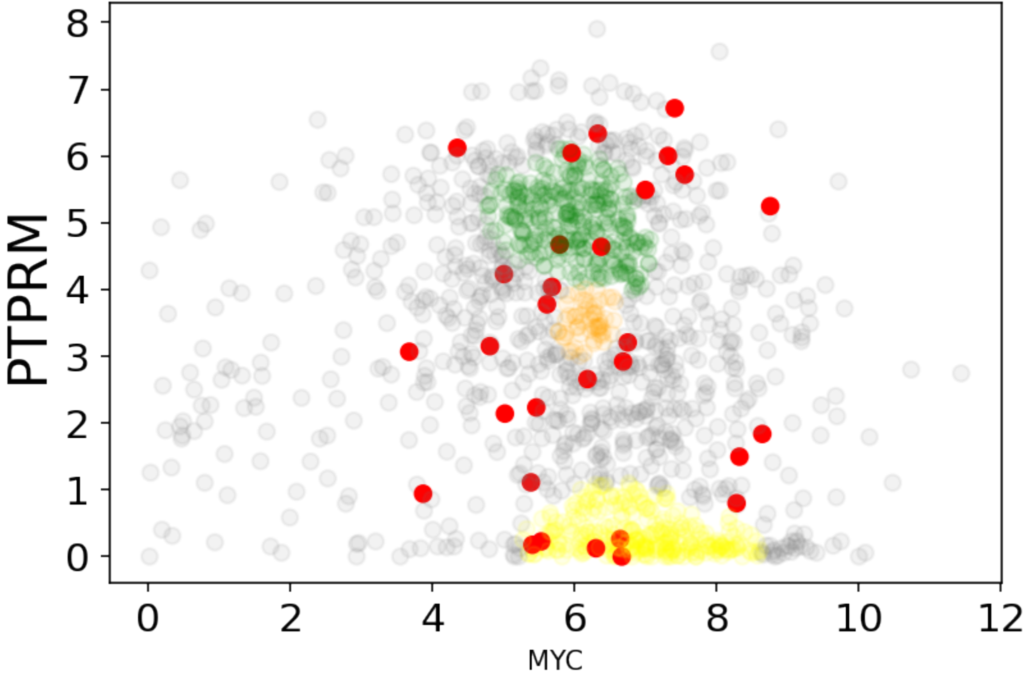
8
7
6
5
4
3
2
1
0
PTPRM
0
2
4
6
8
10
12
MYC

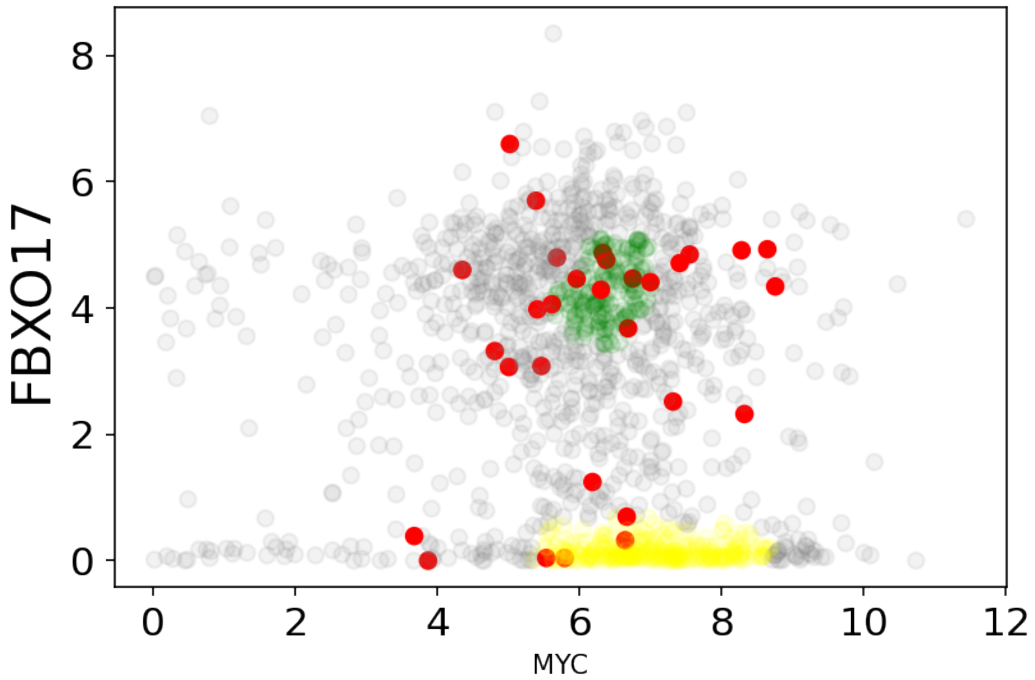

FBXO17
MYC
0
2
4
6
8
0
2
4
6
8
10
12

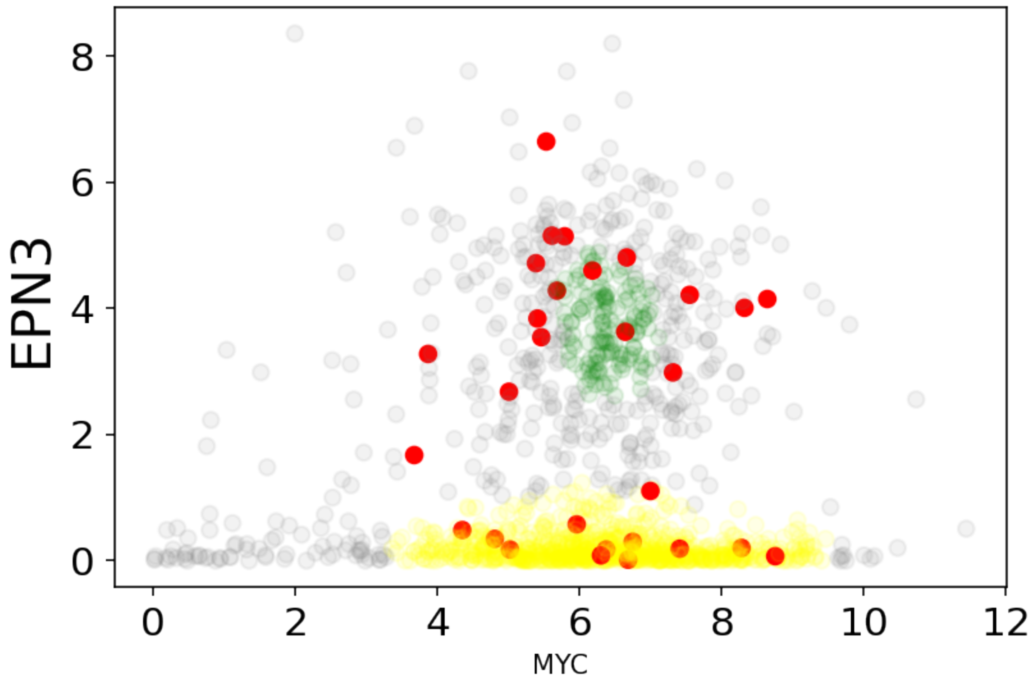

EPN3
8
6
4
2
0
0
2
4
6
8
10
12
MYC

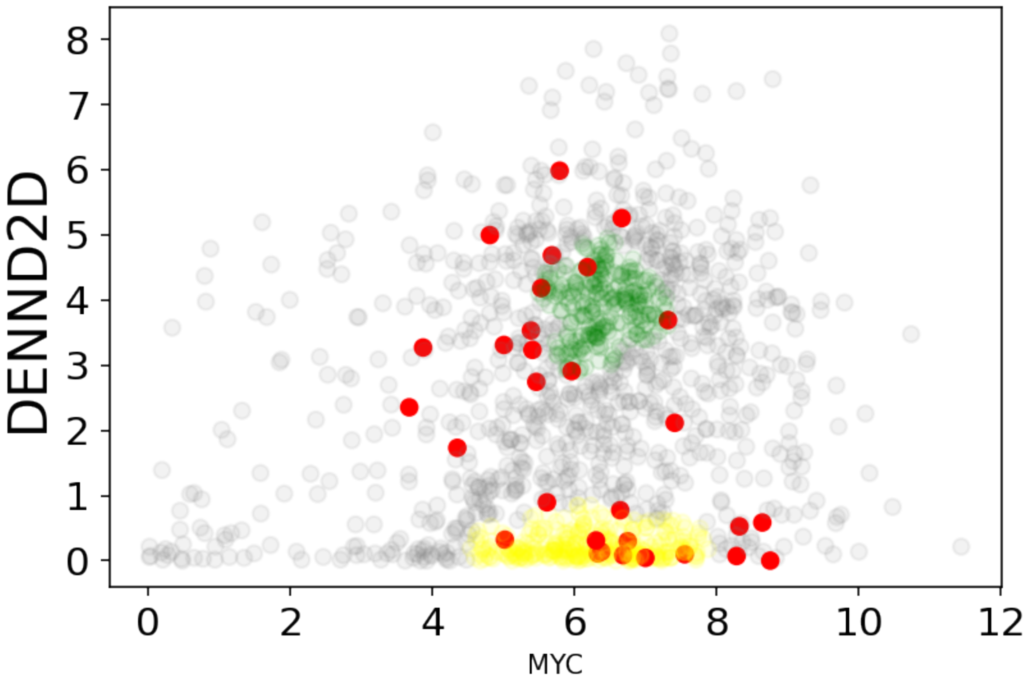

DENND2D
MYC
0
1
2
3
4
5
6
7
8
0
2
4
6
8
10
12

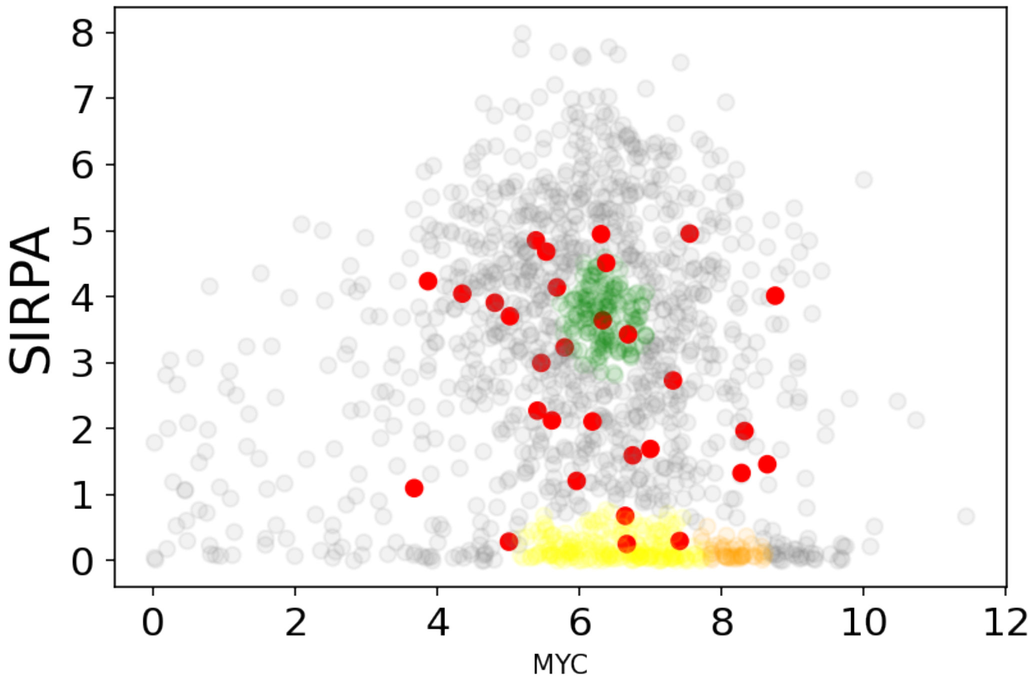
SIRPA
MYC
0
1
2
3
4
5
6
7
8
0
2
4
6
8
10
12

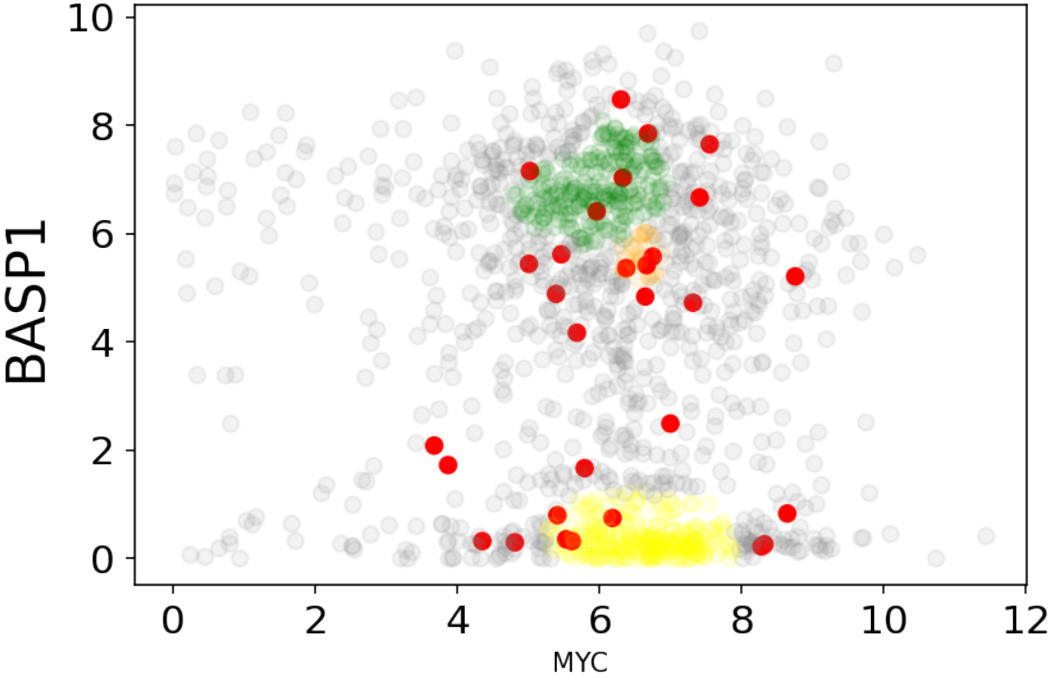

BASP1
MYC
0
2
4
6
8
10
12

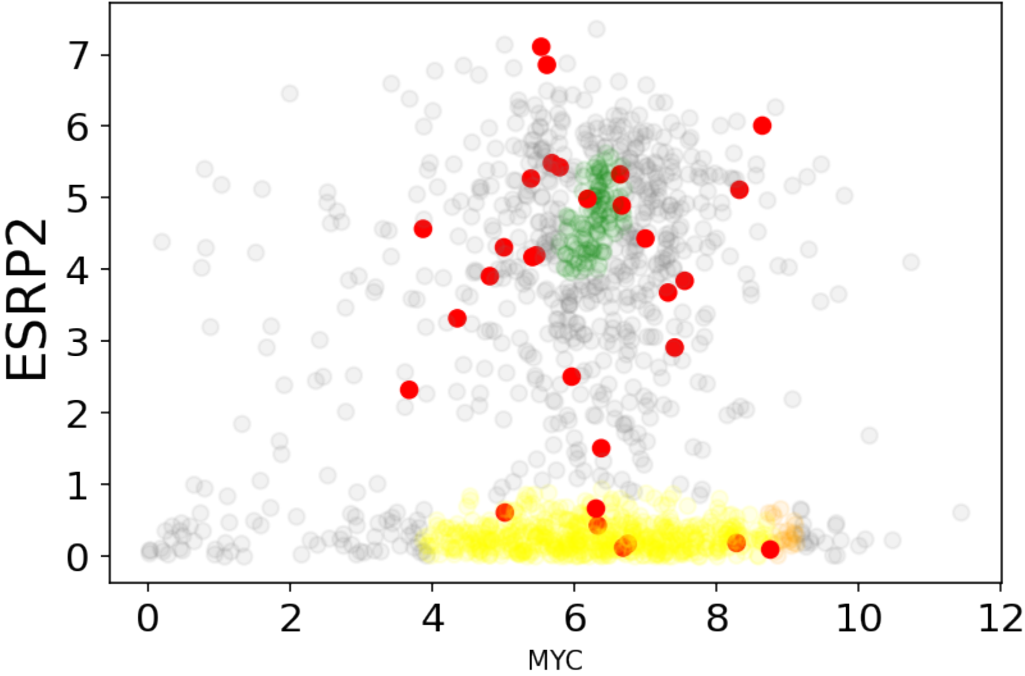
ESRP2
MYC
0
1
2
3
4
5
6
7
0
2
4
6
8
10
12

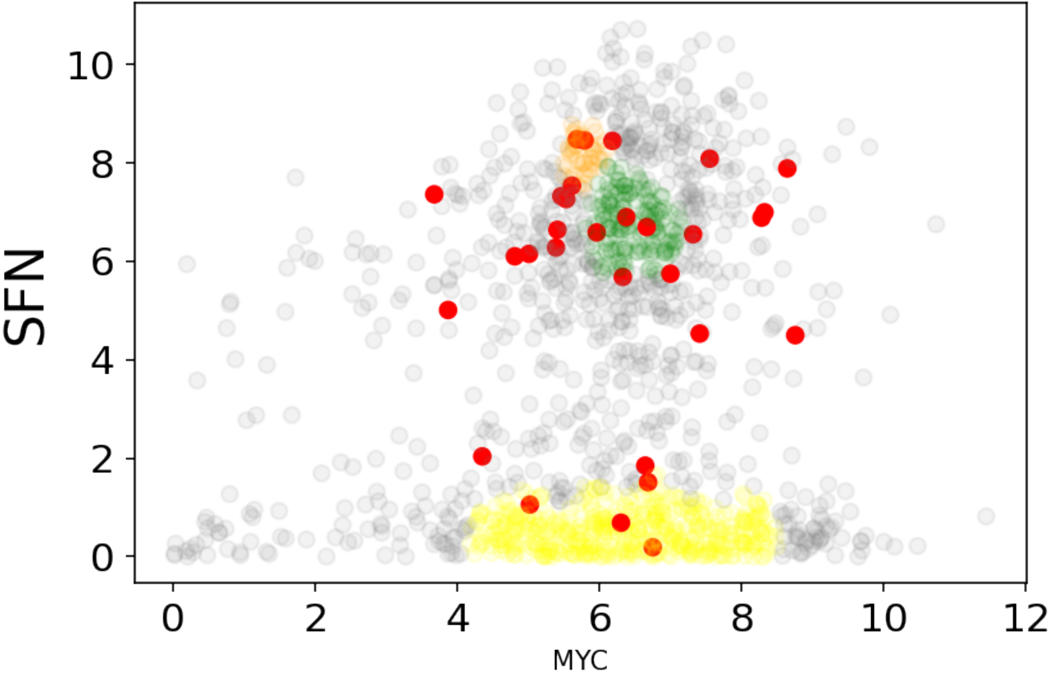

SFN
MYC
0
2
4
6
8
10
12

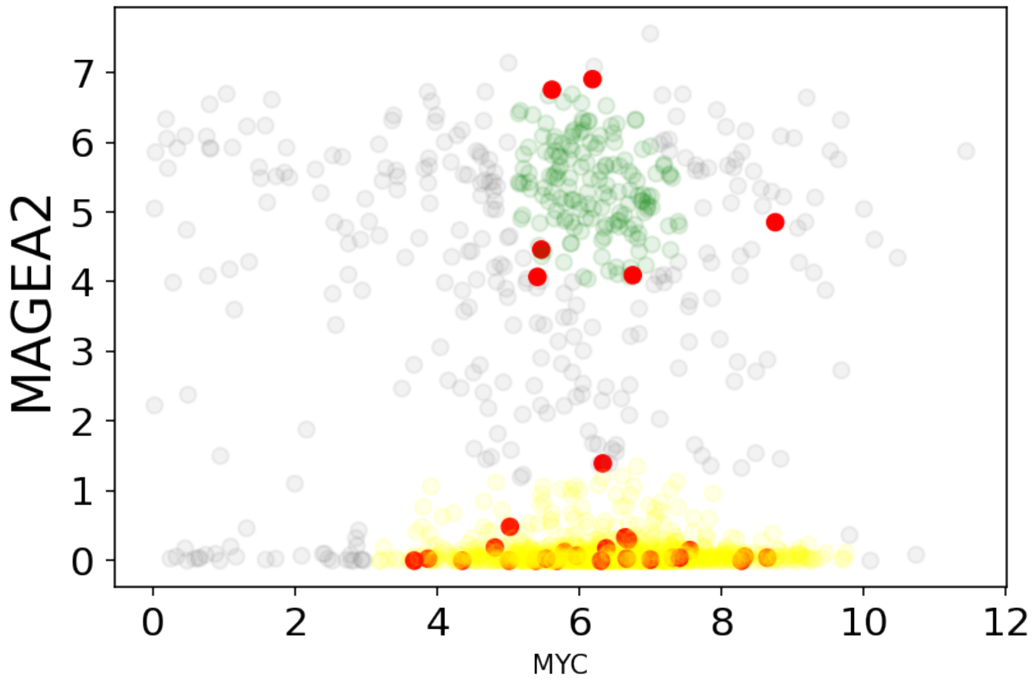
MAGEA2
MYC
0
1
2
3
4
5
6
7
0
2
4
6
8
10
12

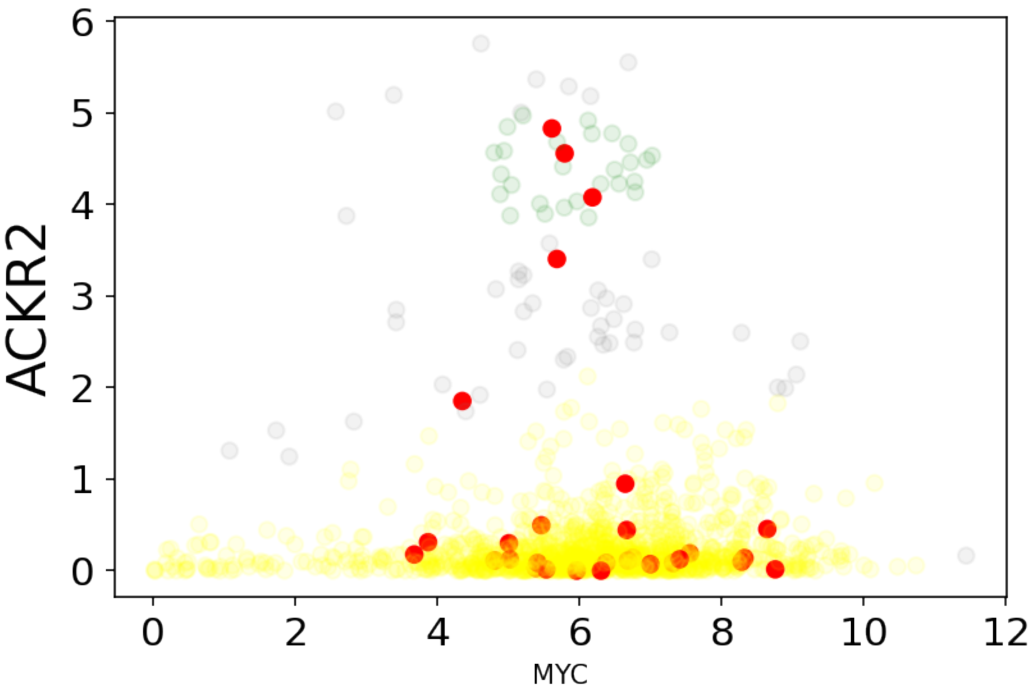
ACKR2
MYC
0
1
2
3
4
5
6
0
2
4
6
8
10
12

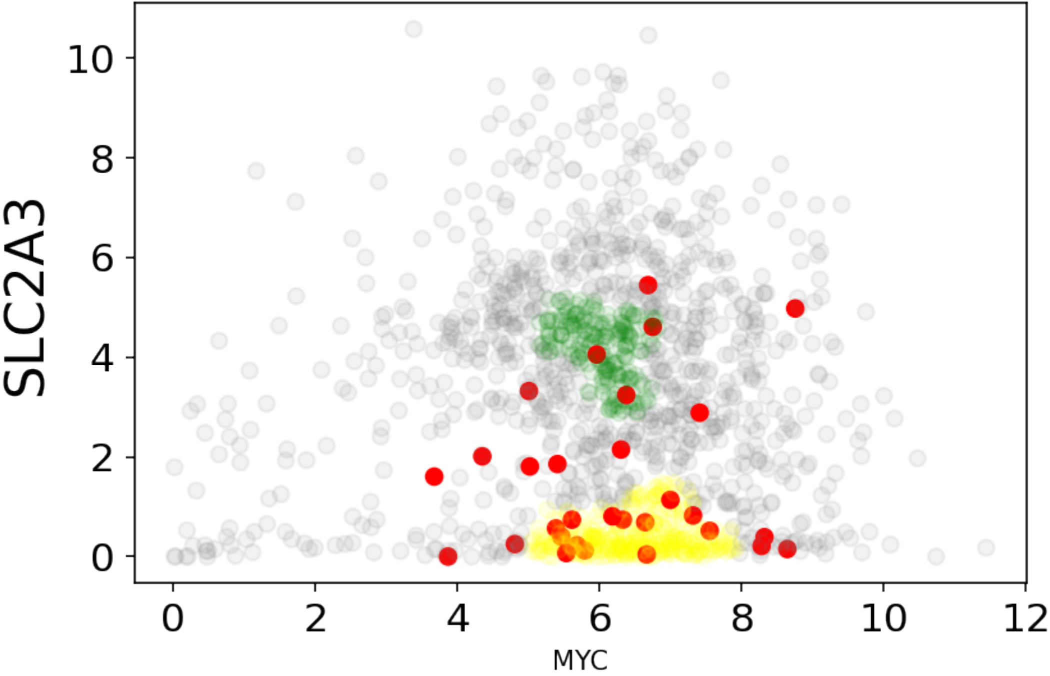

SLC2A3
MYC
10
8
6
4
2
0
0
2
4
6
8
10
12

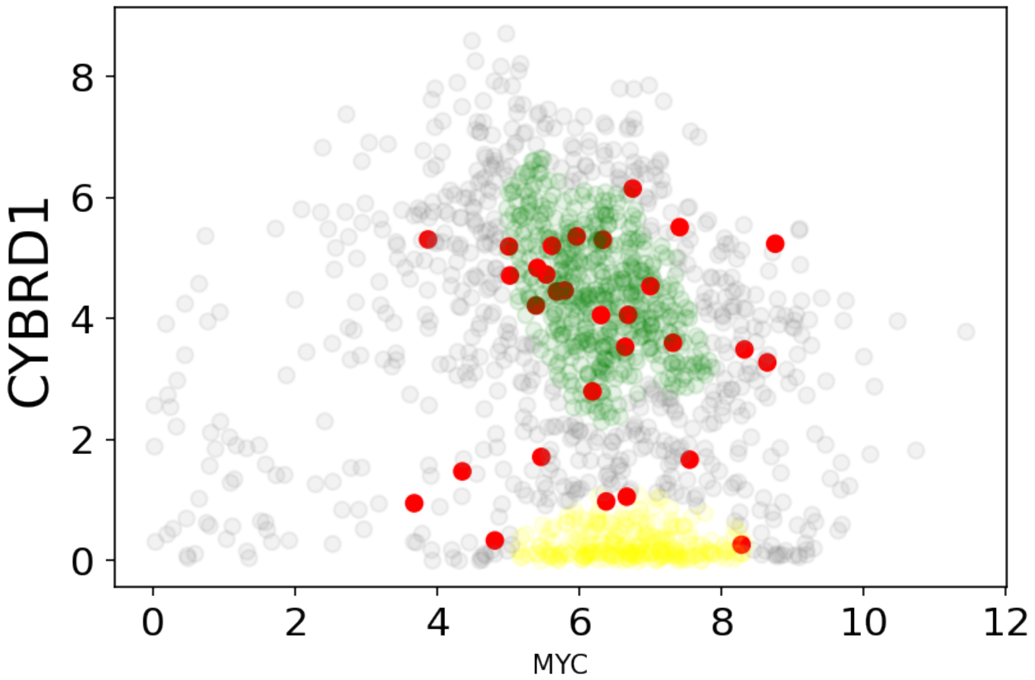
CYBRD1
MYC
0
2
4
6
8
10
12

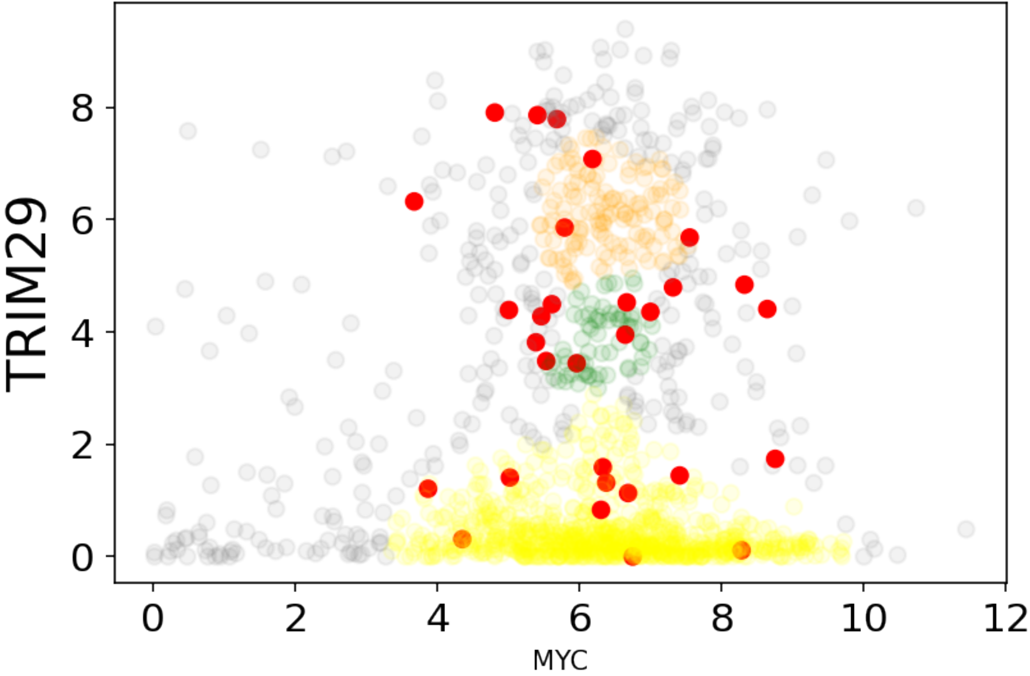
TRIM29
8
6
4
2
0
0
2
4
6
8
10
12
MYC

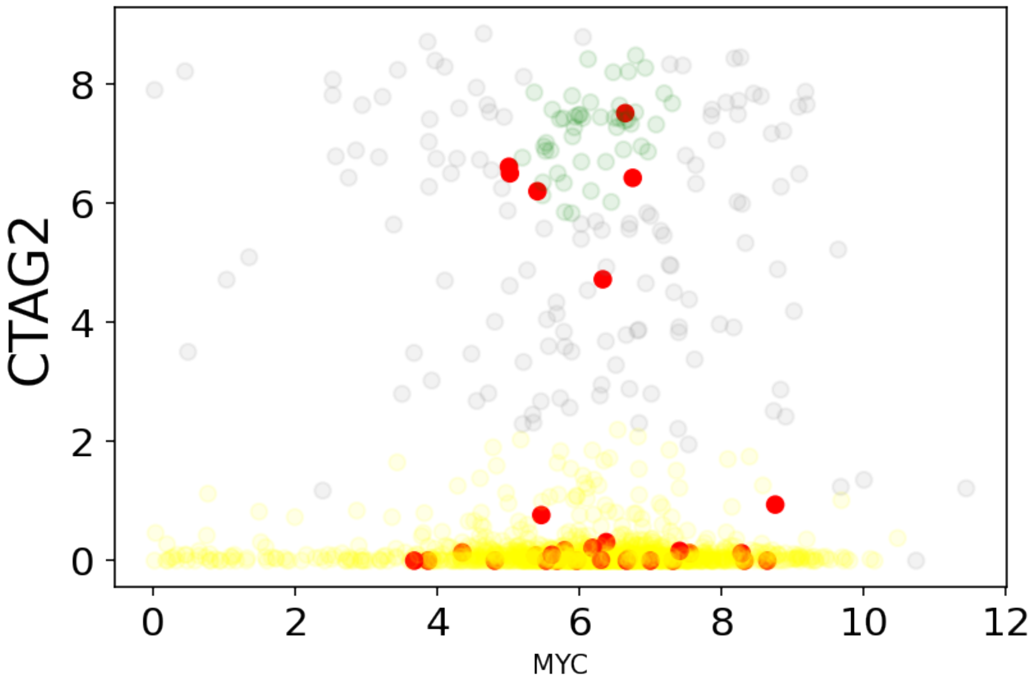

CTAG2
0
2
4
6
8
0
2
4
6
8
10
12
MYC

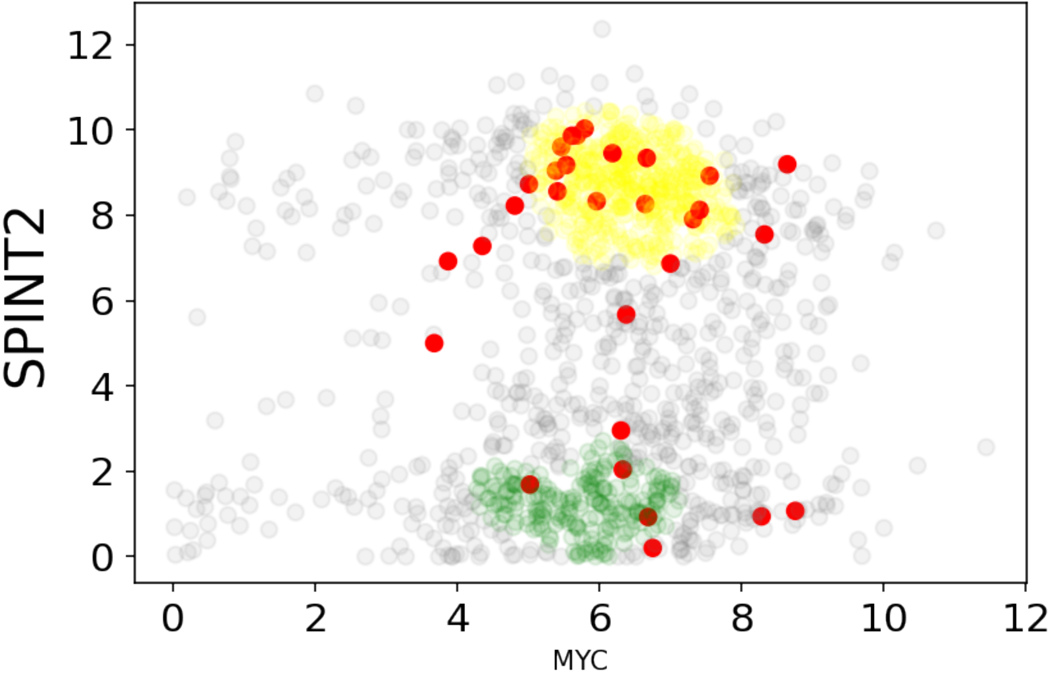
SPINT2
MYC
0
2
4
6
8
10
12
0
2
4
6
8
10
12

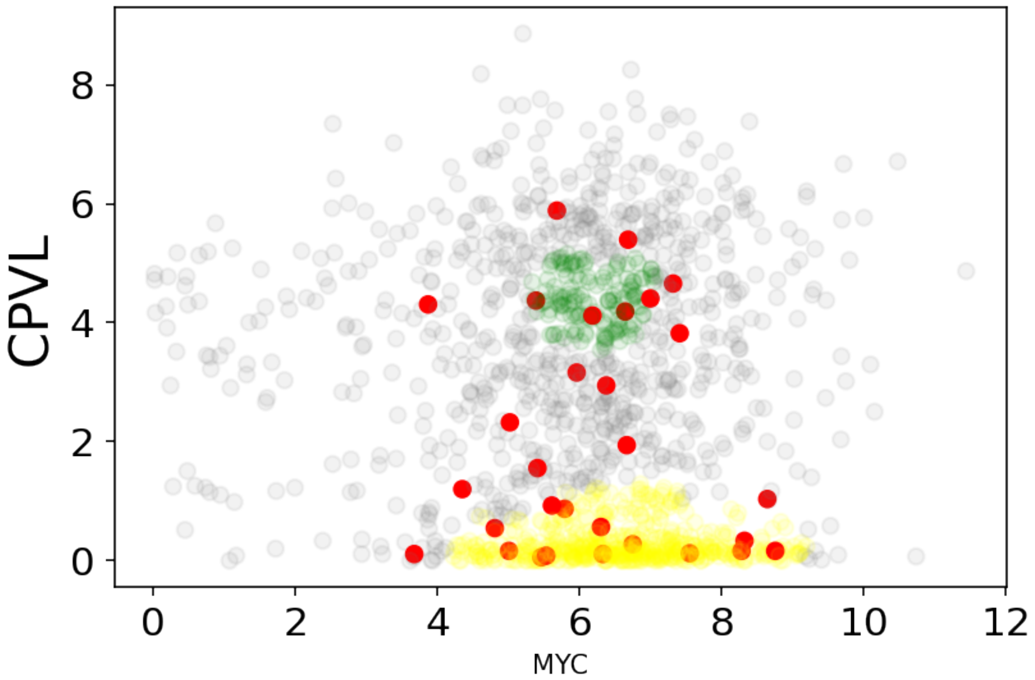

CPVL
0
2
4
6
8
0
2
4
6
8
10
12
MYC

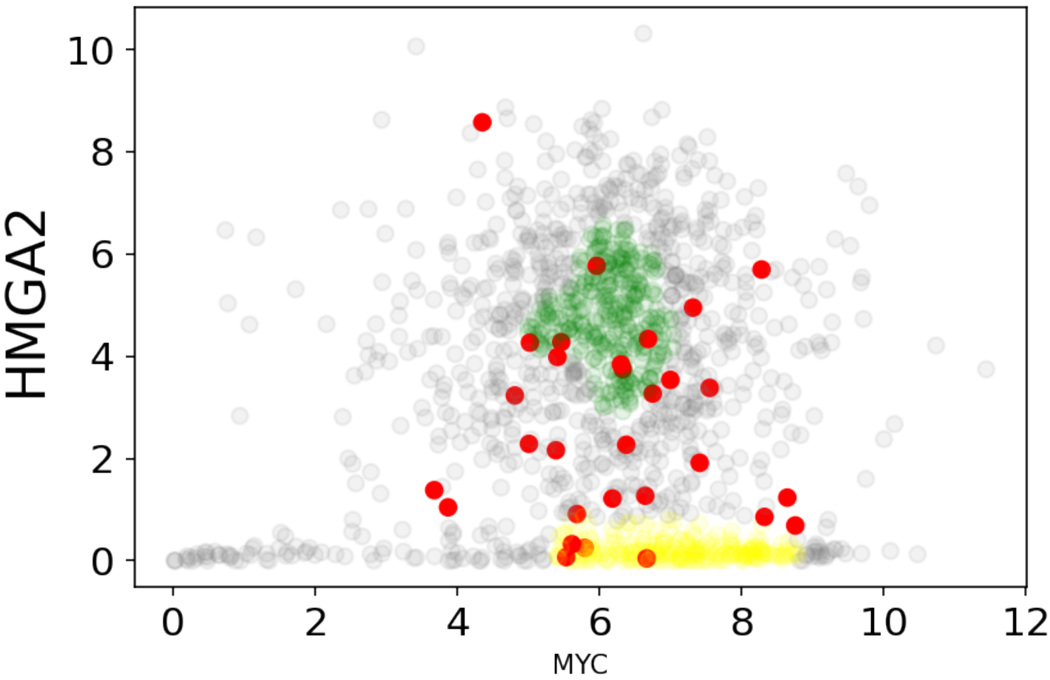
HMGA2
MYC
0
2
4
6
8
10
12

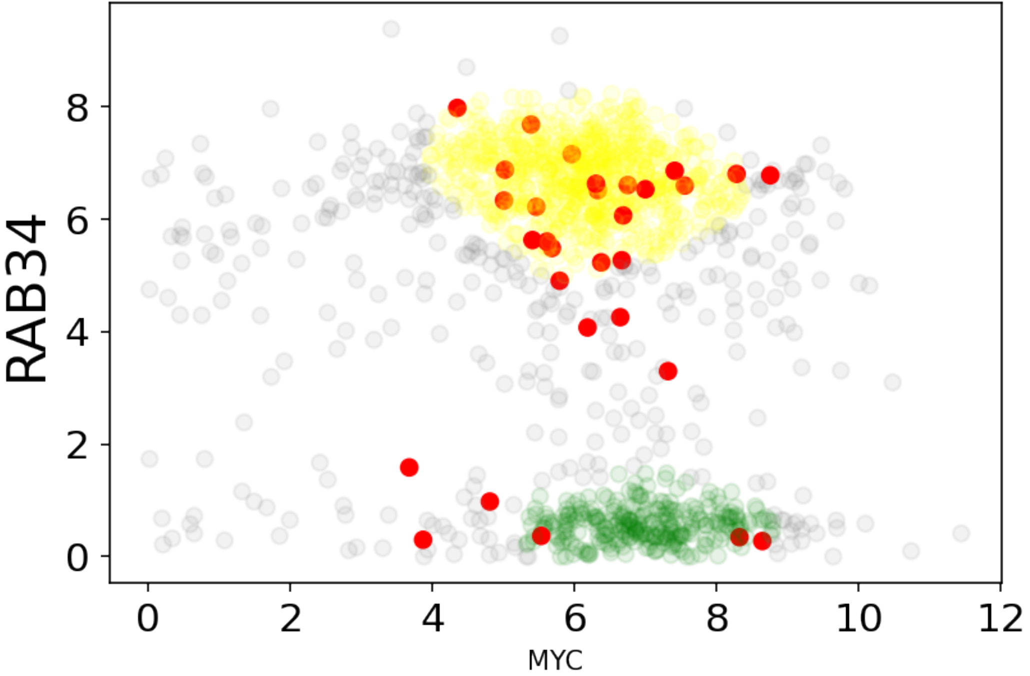

RAB34
8
6
4
2
0
0
2
4
6
8
10
12
MYC

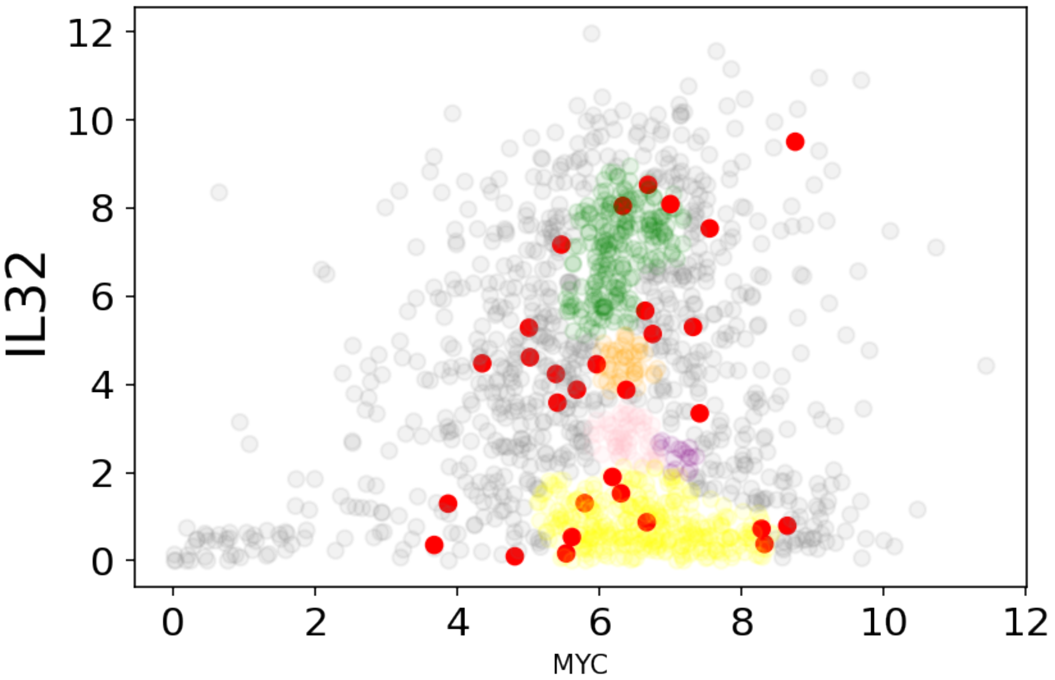
IL32
MYC
0
2
4
6
8
10
12

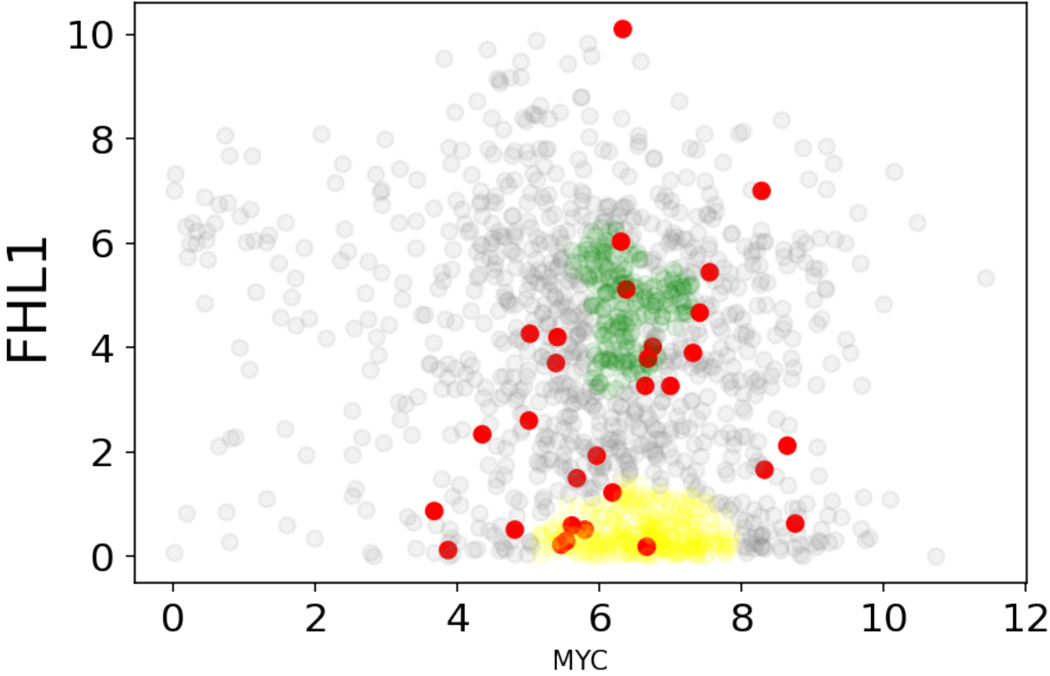

FHL1
10
8
6
4
2
0
0
2
4
6
8
10
12
MYC

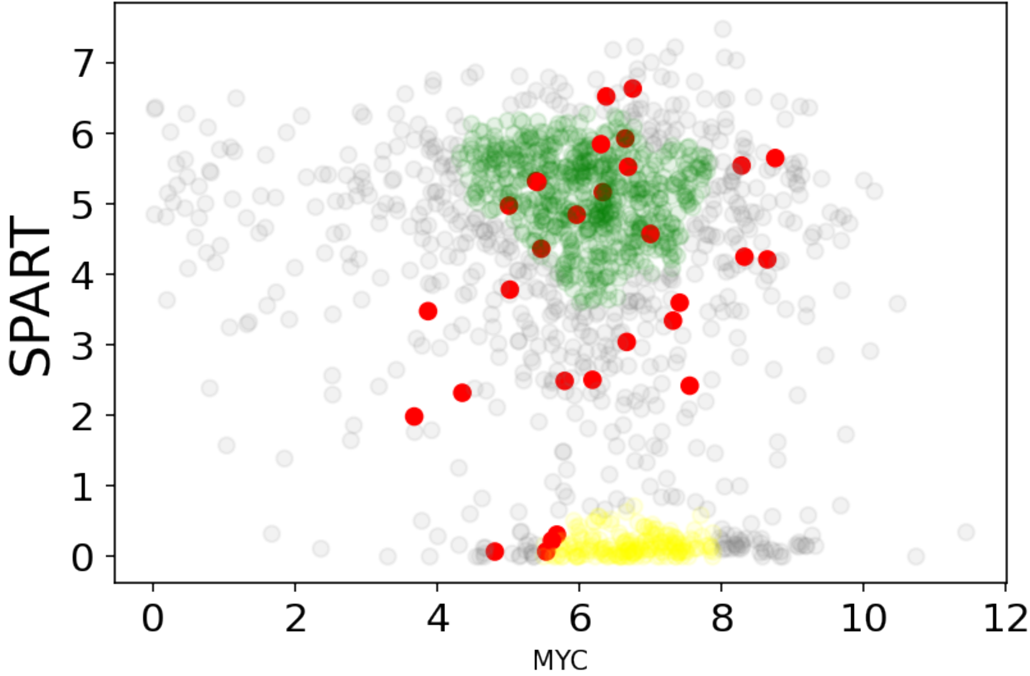

SPART
MYC
0
1
2
3
4
5
6
7
0
2
4
6
8
10
12

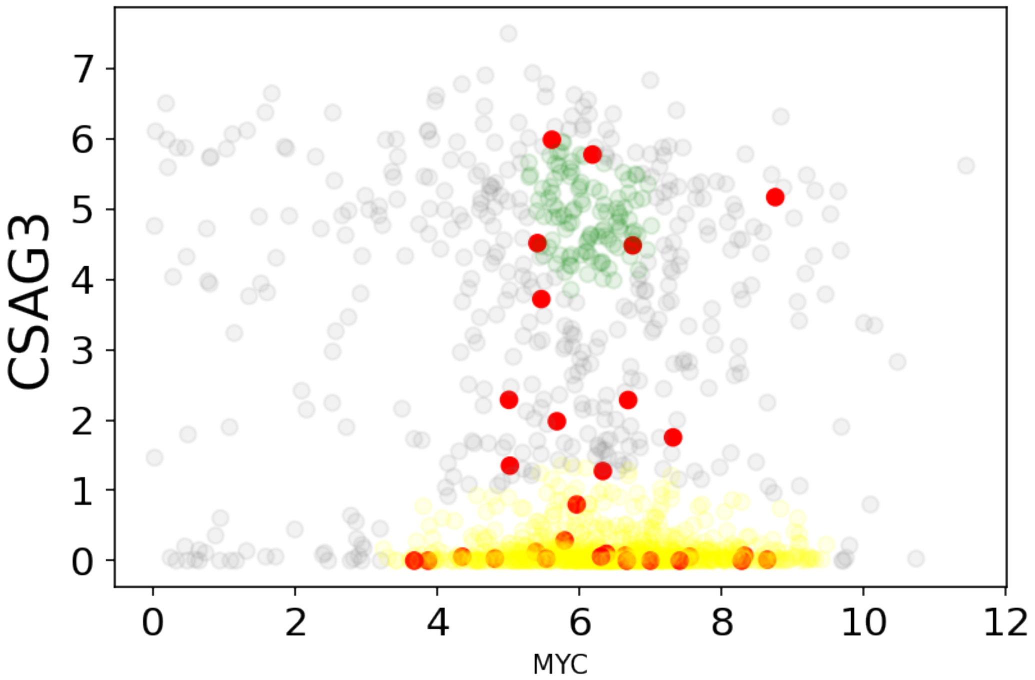

CSAG3
7
6
5
4
3
2
1
0
0
2
4
6
8
10
12
MYC

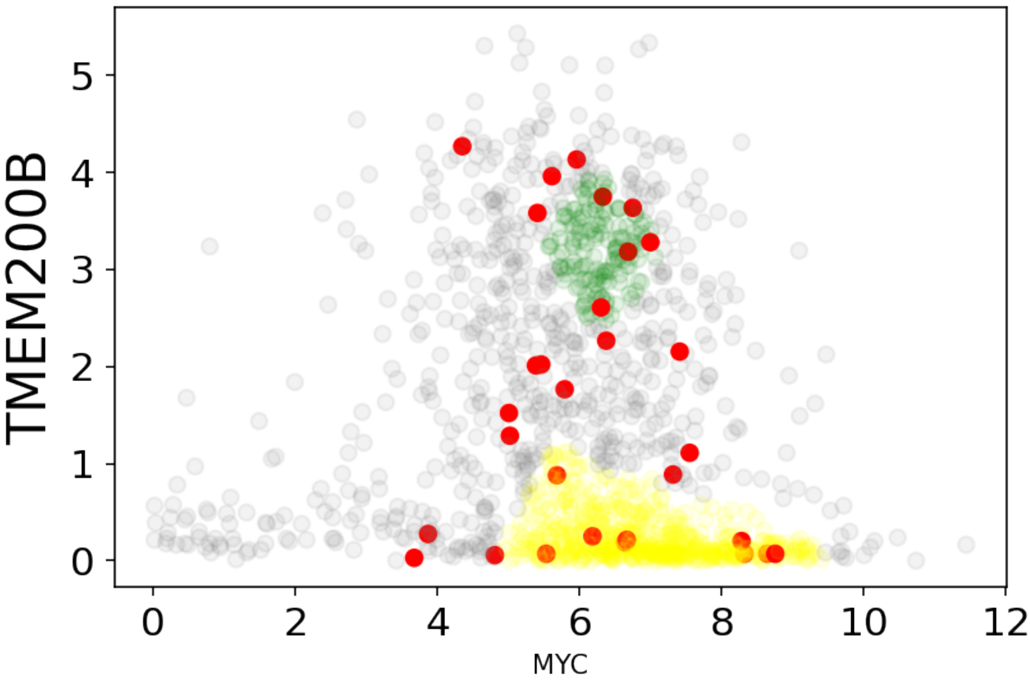

TMEM200B
0
1
2
3
4
5
0
2
4
6
8
10
12
MYC

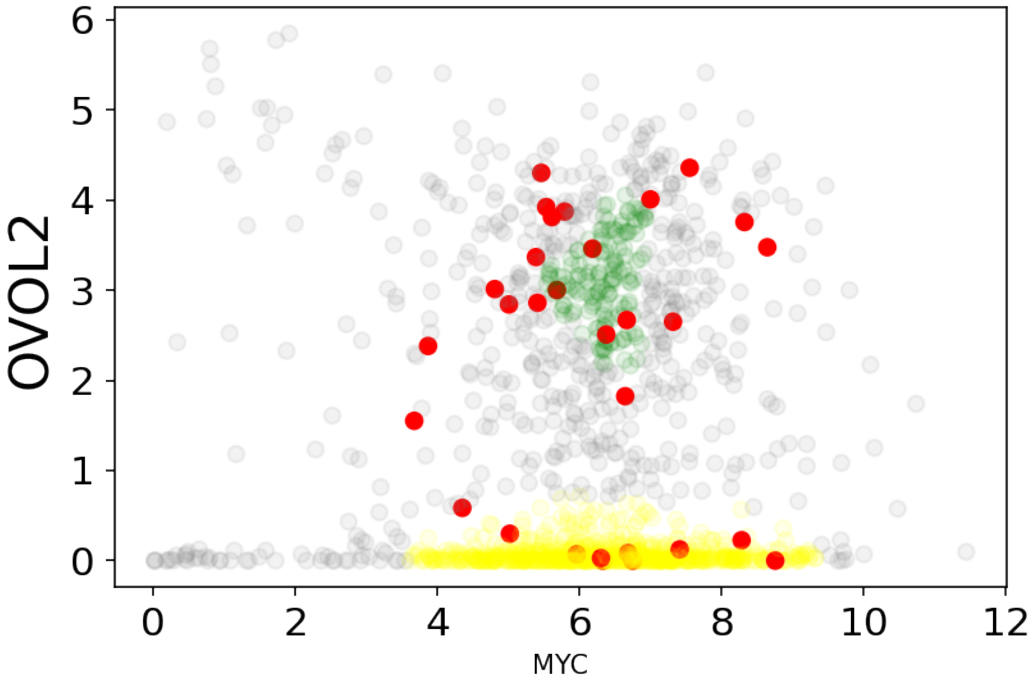

OVOL2
MYC
0
1
2
3
4
5
6
0
2
4
6
8
10
12

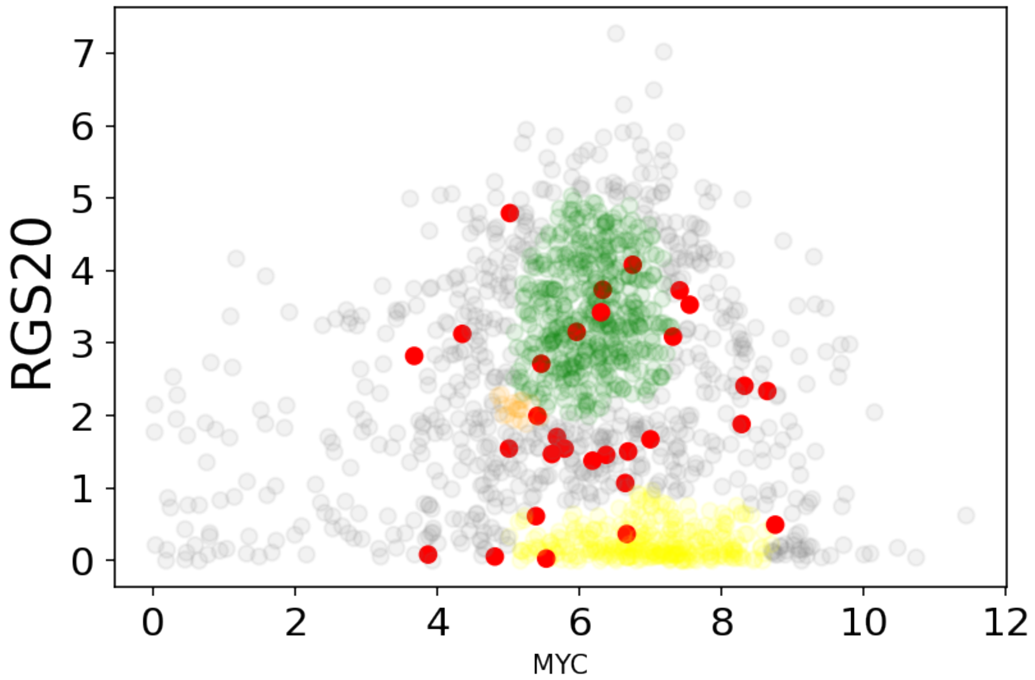
RGS20
7
6
5
4
3
2
1
0
0
2
4
6
8
10
12
MYC

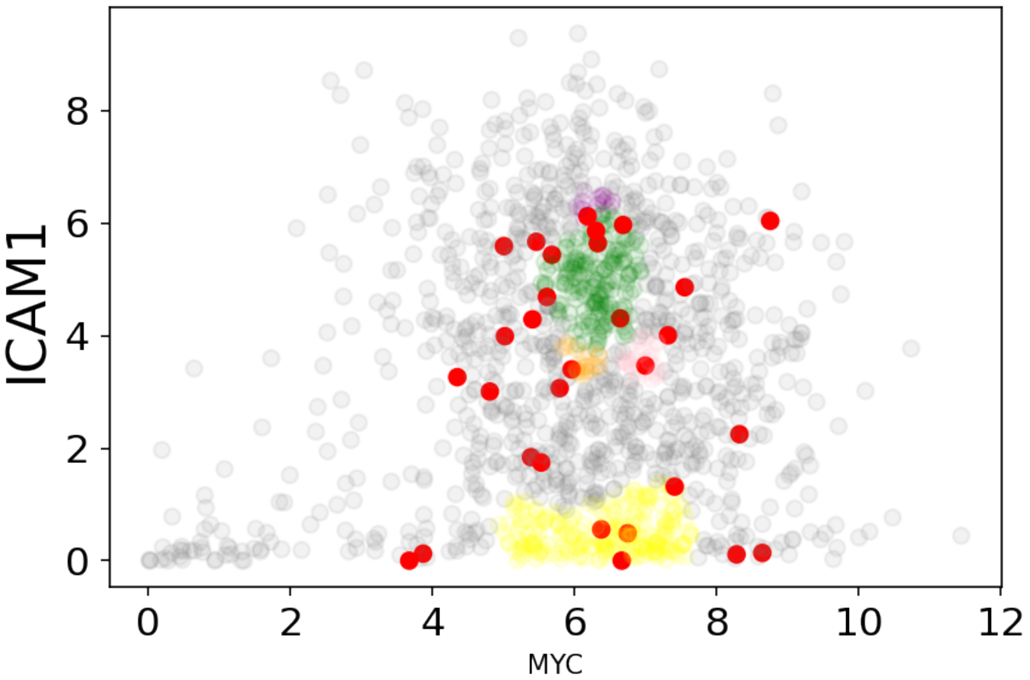

ICAM1
MYC
0
2
4
6
8
10
12

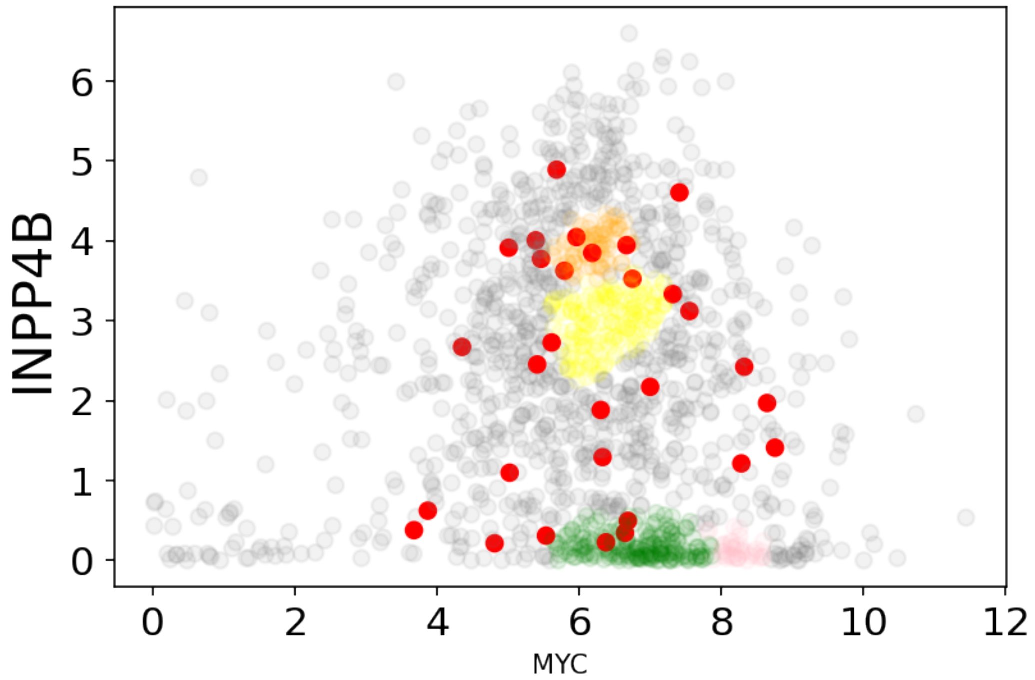

INPP4B
MYC
0
1
2
3
4
5
6
0
2
4
6
8
10
12

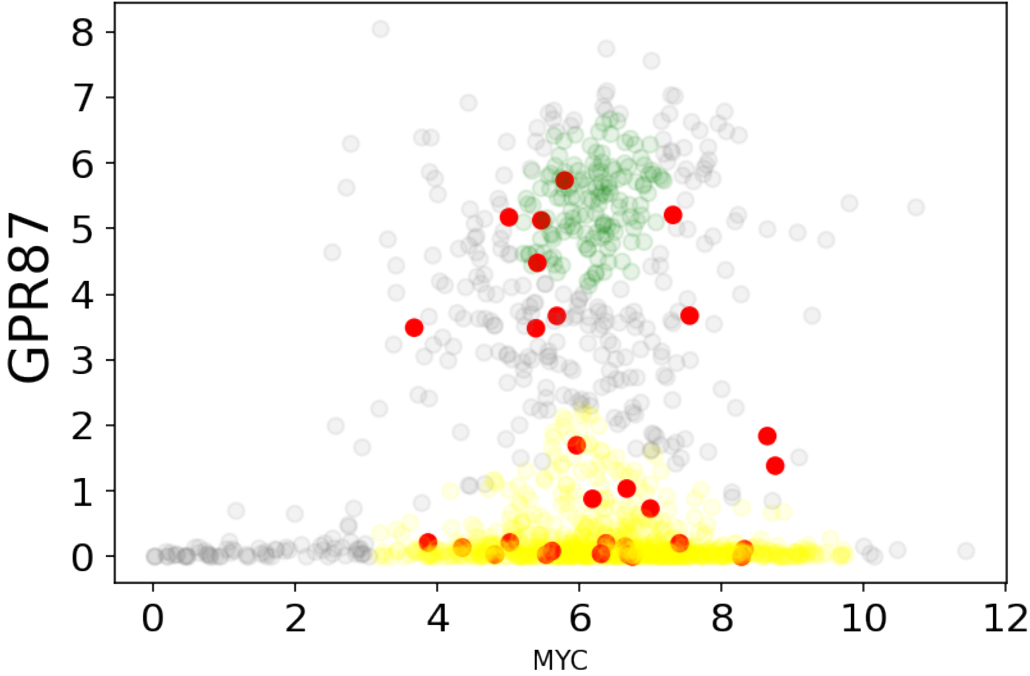

GPR87
MYC
0
1
2
3
4
5
6
7
8
0
2
4
6
8
10
12

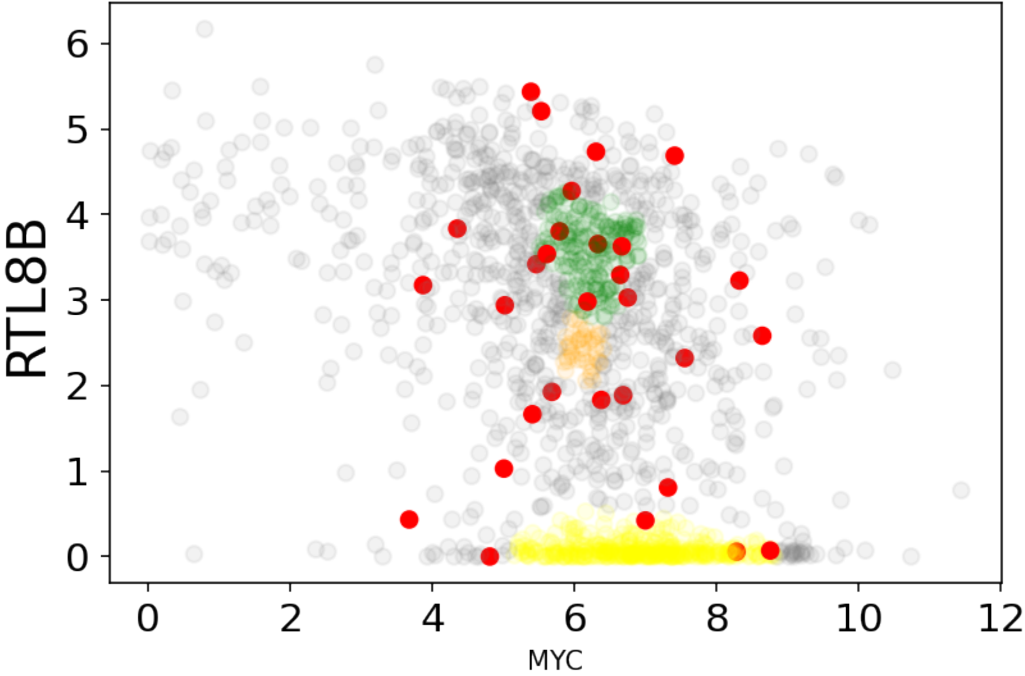

RTL8B
MYC
0
1
2
3
4
5
6
0
2
4
6
8
10
12

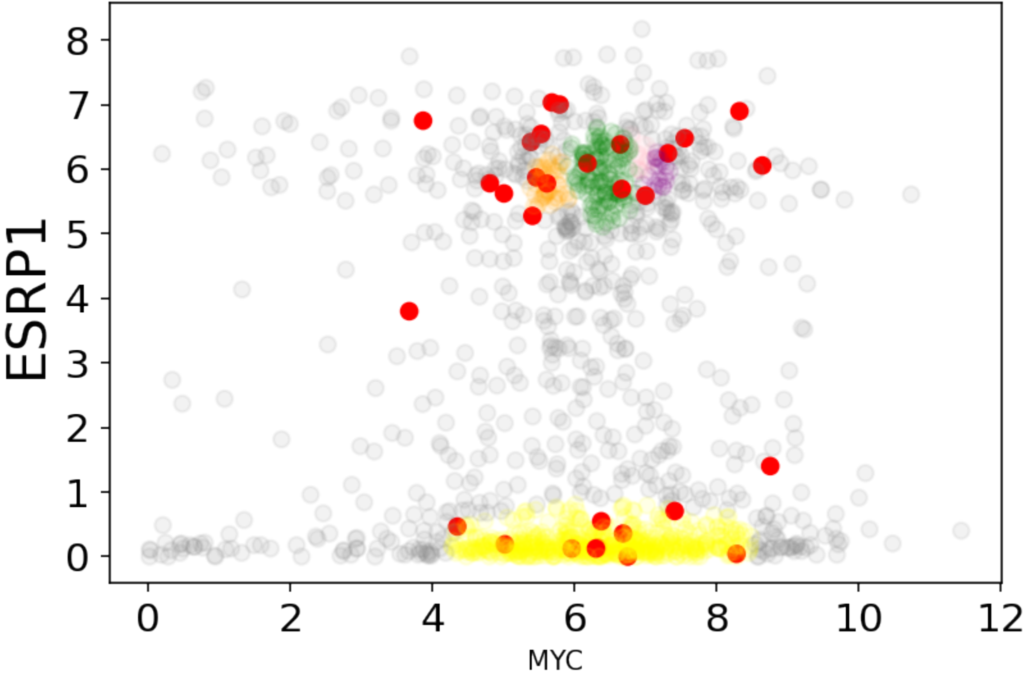

ESRP1
8
7
6
5
4
3
2
1
0
0
2
4
6
8
10
12
MYC

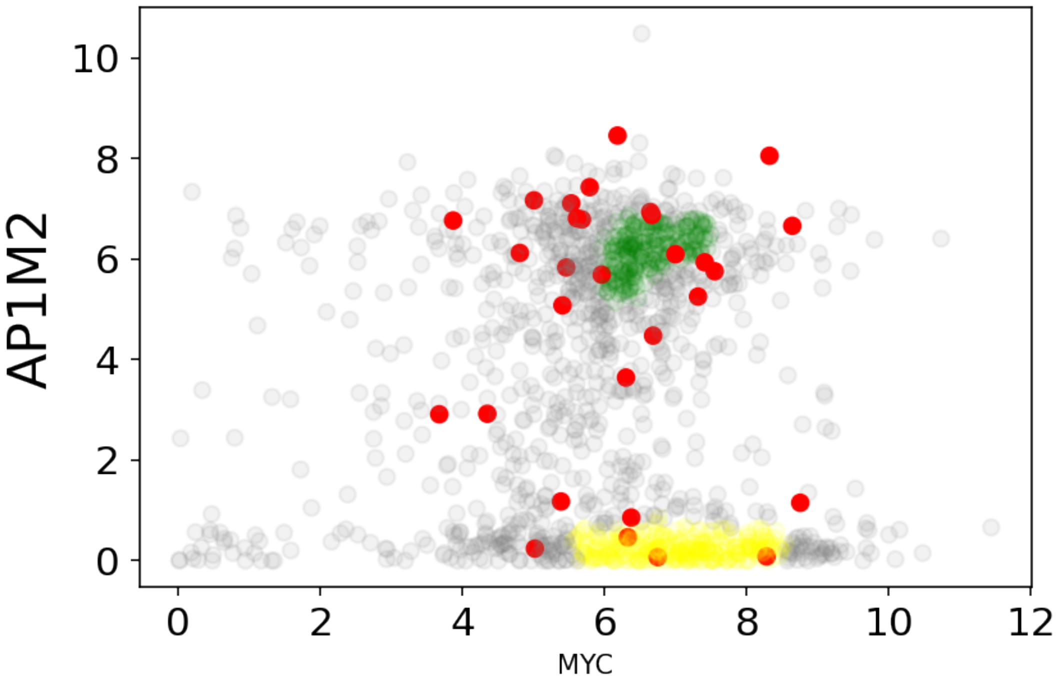

AP1M2
MYC
0
2
4
6
8
10
12

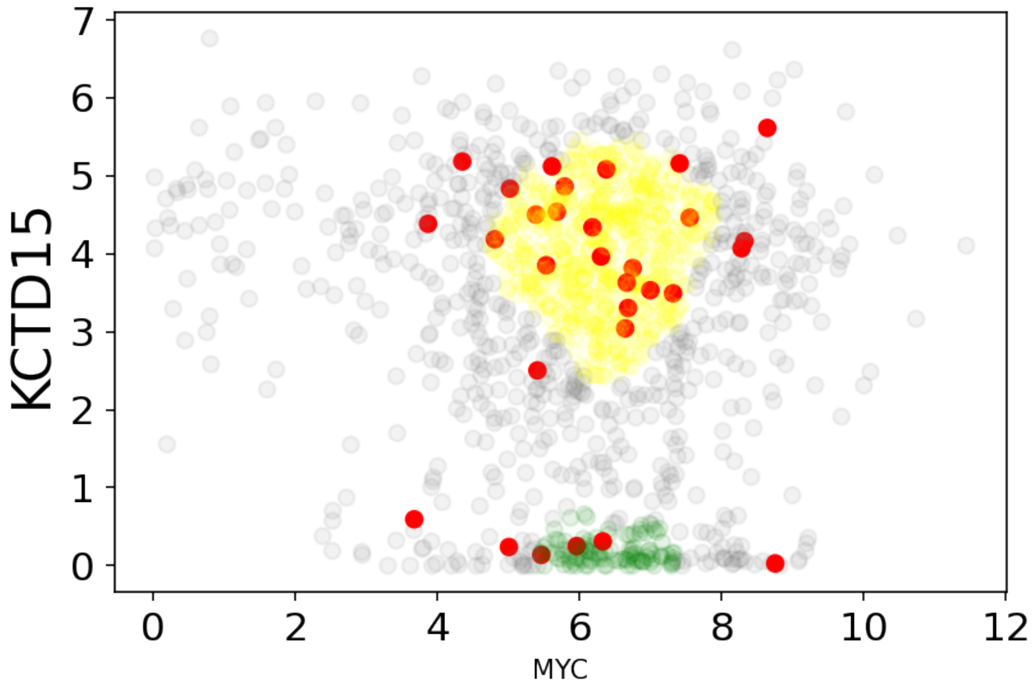
KCTD15
MYC
0
1
2
3
4
5
6
7
0
2
4
6
8
10
12

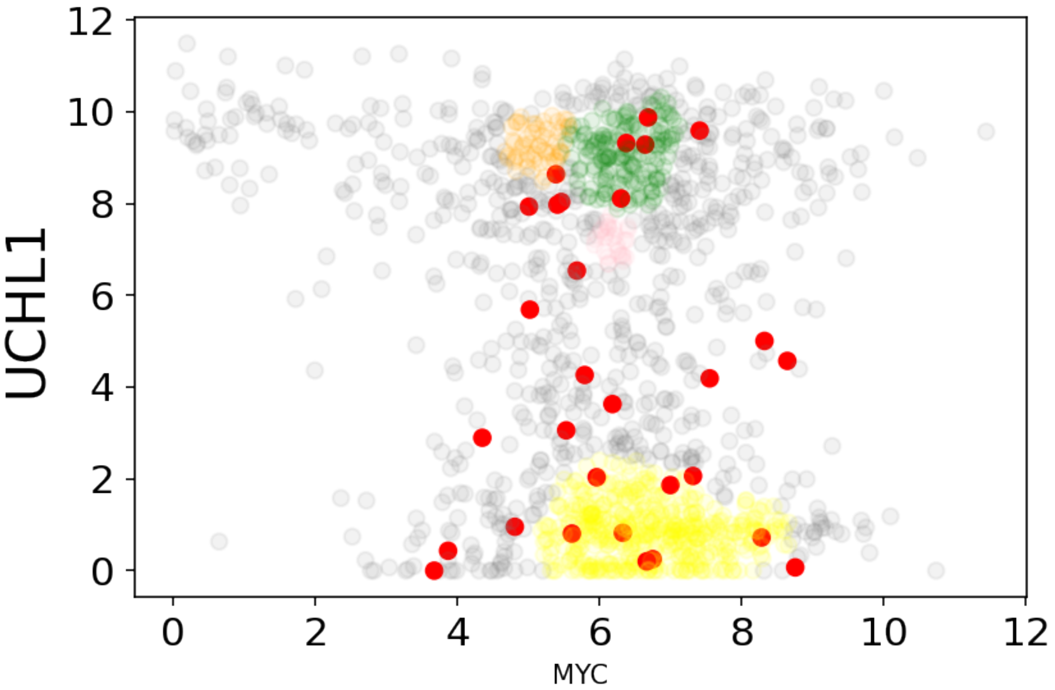

UCHL1
MYC
0
2
4
6
8
10
12

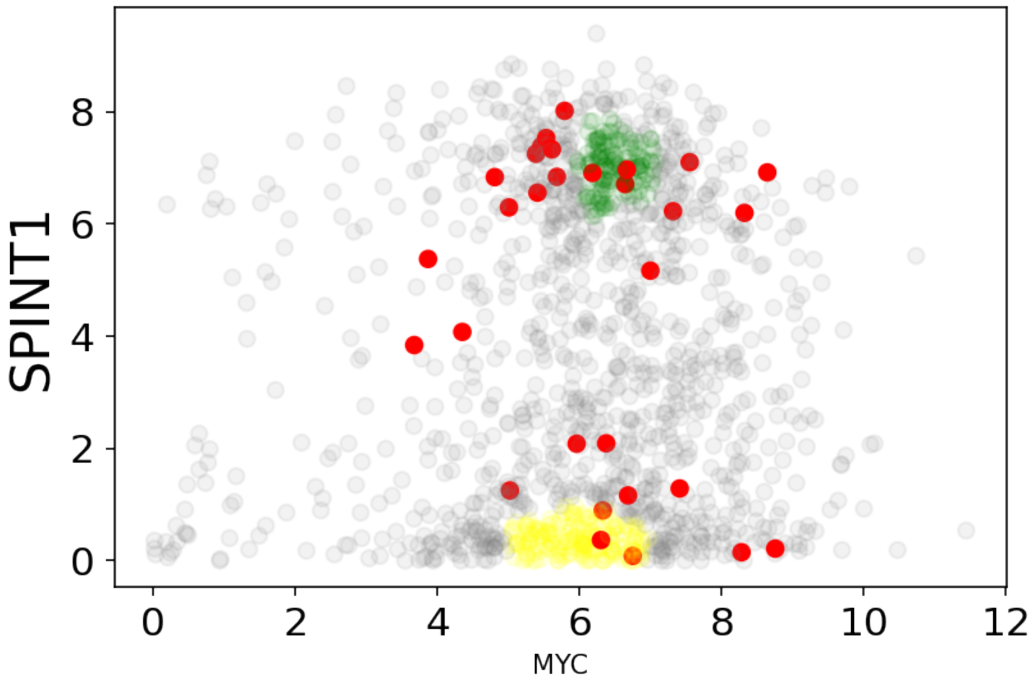

SPINT1
8
6
4
2
0
0
2
4
6
8
10
12
MYC

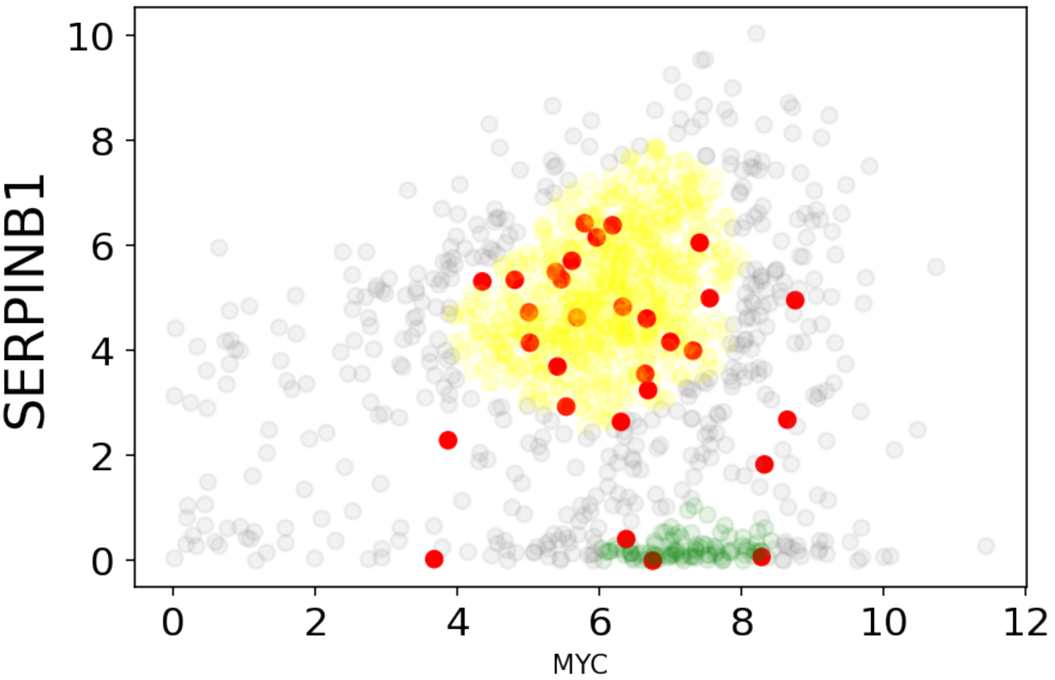

SERPINB1
MYC
0
2
4
6
8
10
12

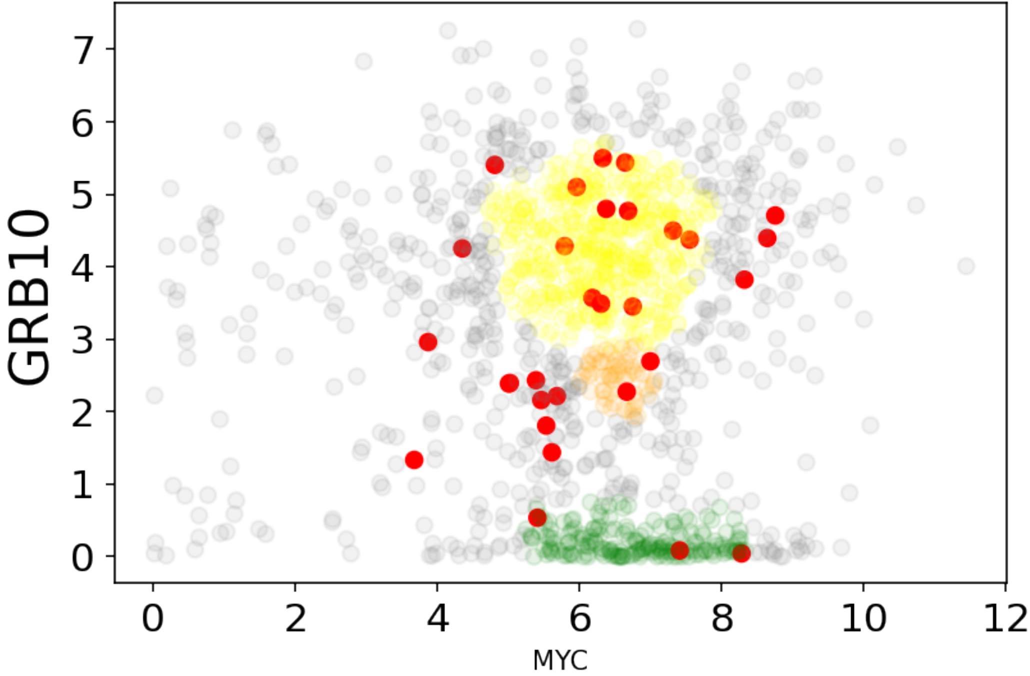
GRB10
MYC
0
1
2
3
4
5
6
7
0
2
4
6
8
10
12

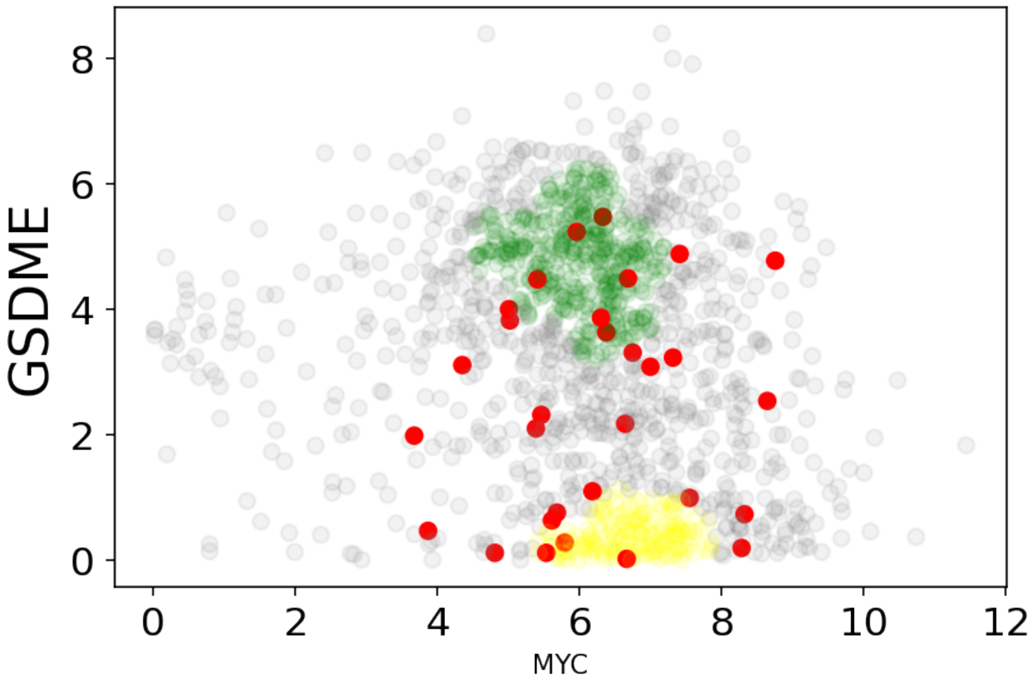

GSDME
MYC
0
2
4
6
8
0
2
4
6
8
10
12

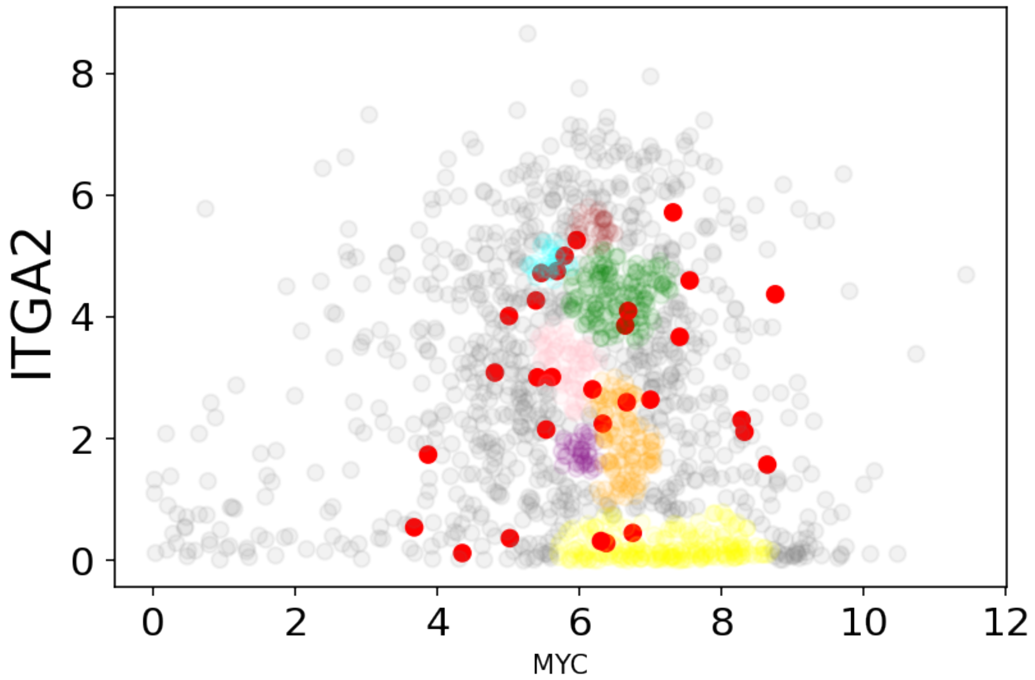
ITGA2
MYC
0
2
4
6
8
0
2
4
6
8
10
12

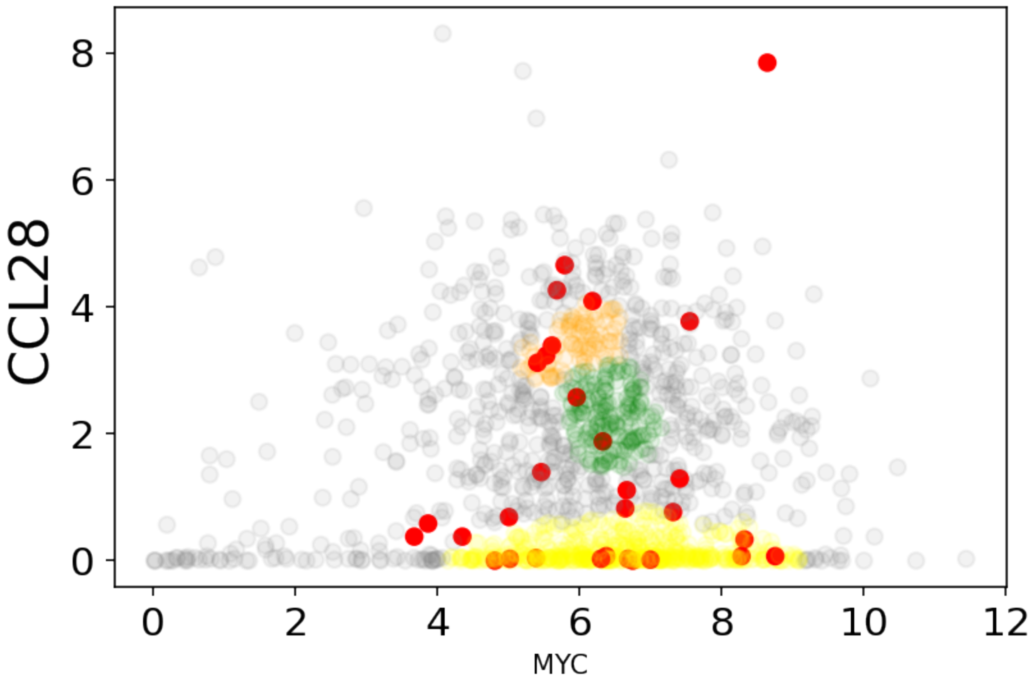
CCL28
0
2
4
6
8
0
2
4
6
8
10
12
MYC

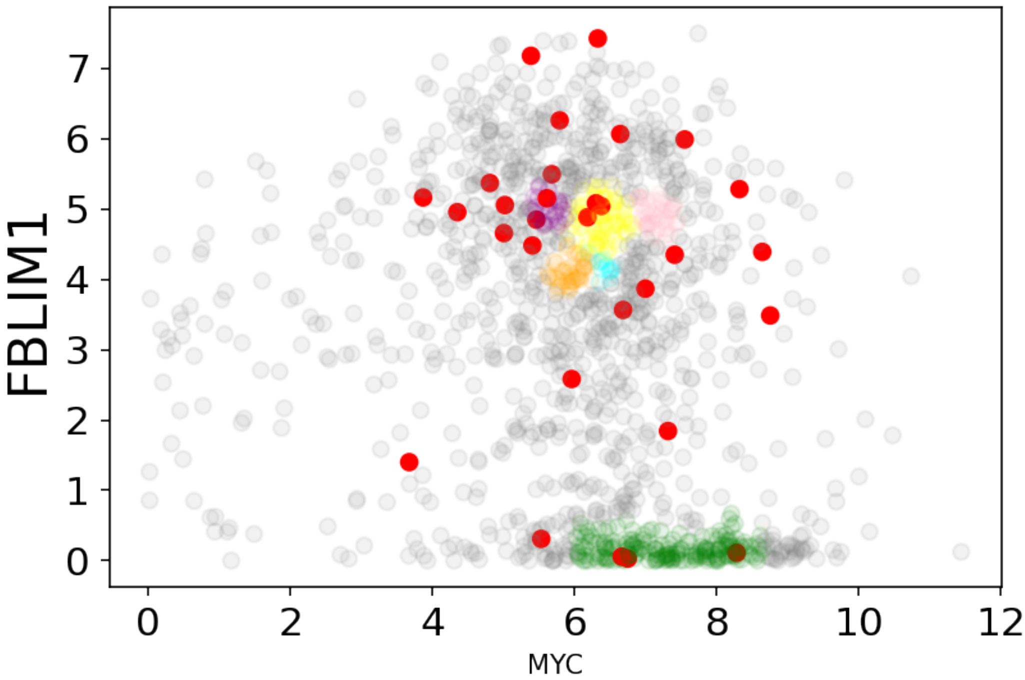

FBLIM1
MYC
0
1
2
3
4
5
6
7
0
2
4
6
8
10
12

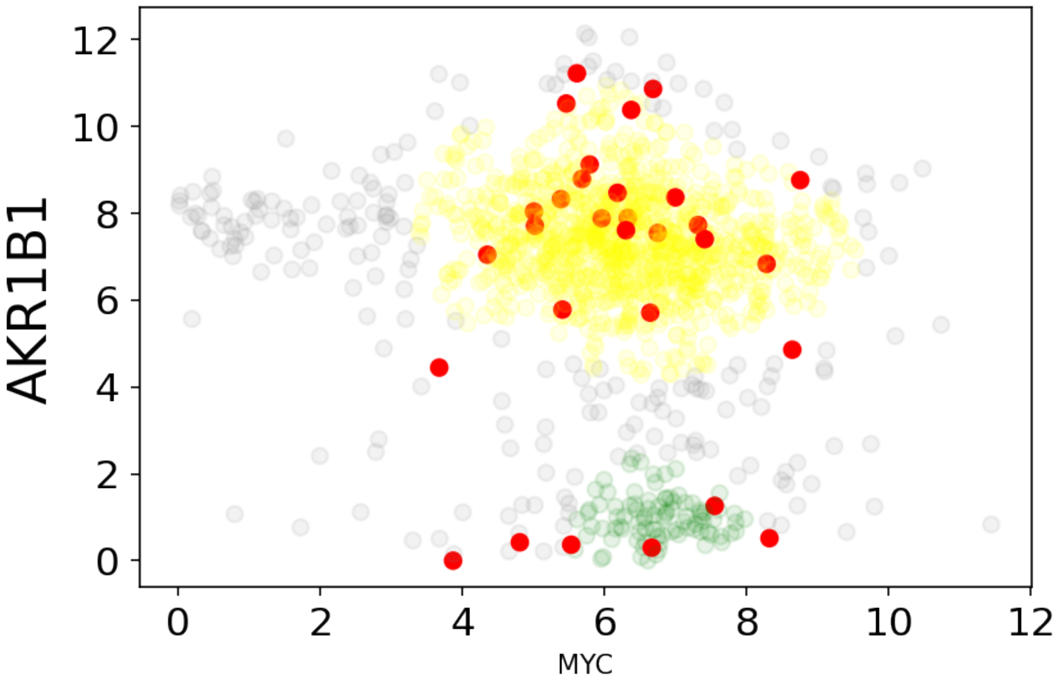

AKR1B1
MYC
0
2
4
6
8
10
12

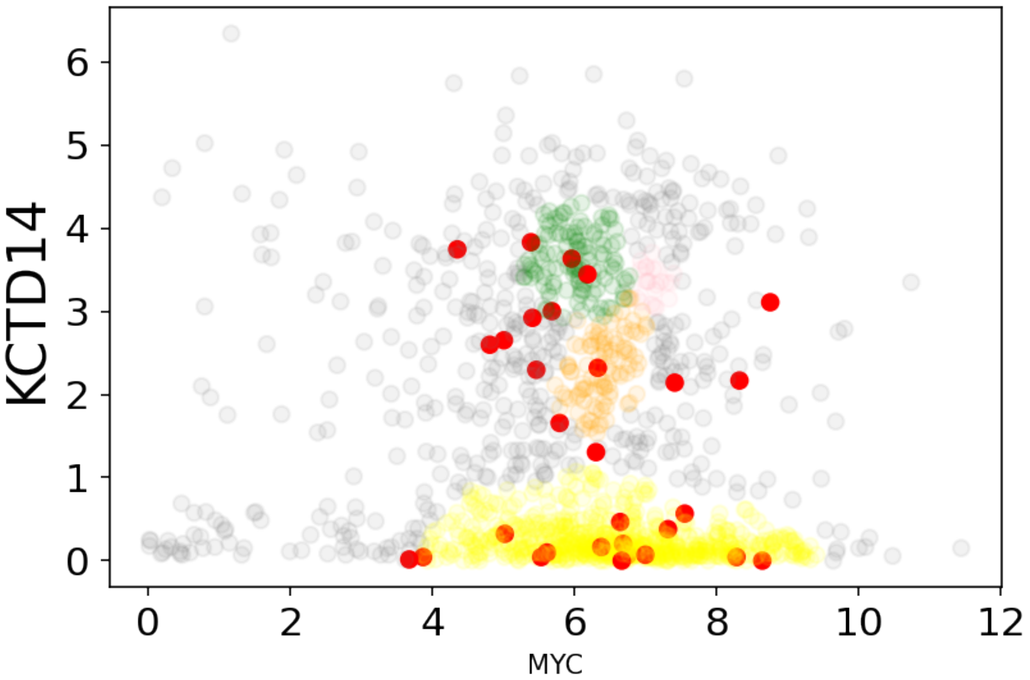

KCTD14
MYC
0
1
2
3
4
5
6
0
2
4
6
8
10
12

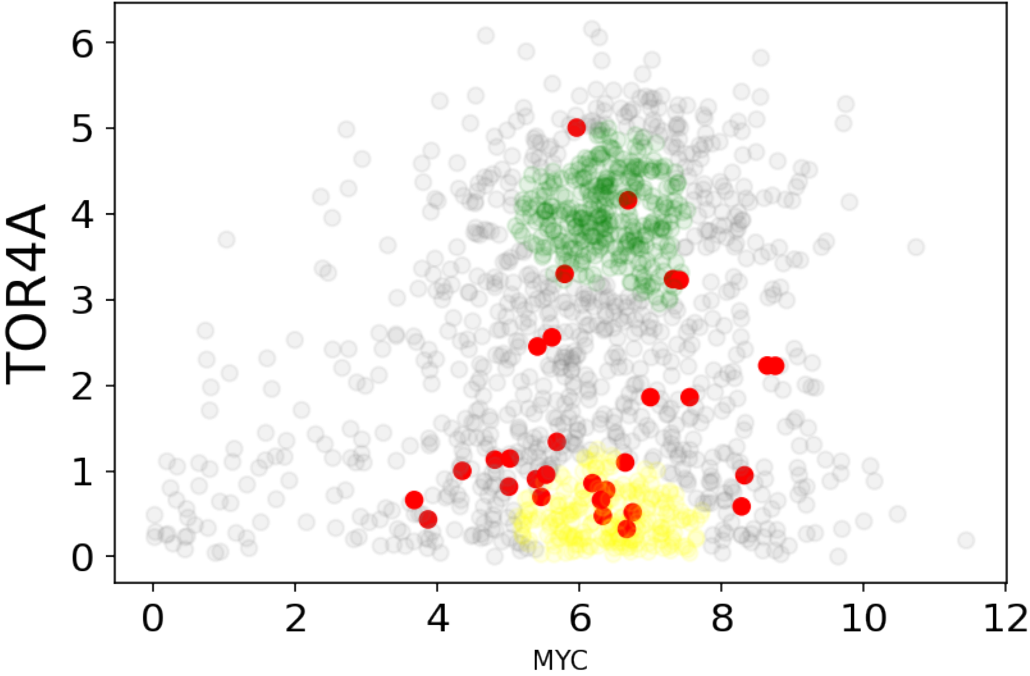
TOR4A
0
1
2
3
4
5
6
0
2
4
6
8
10
12
MYC

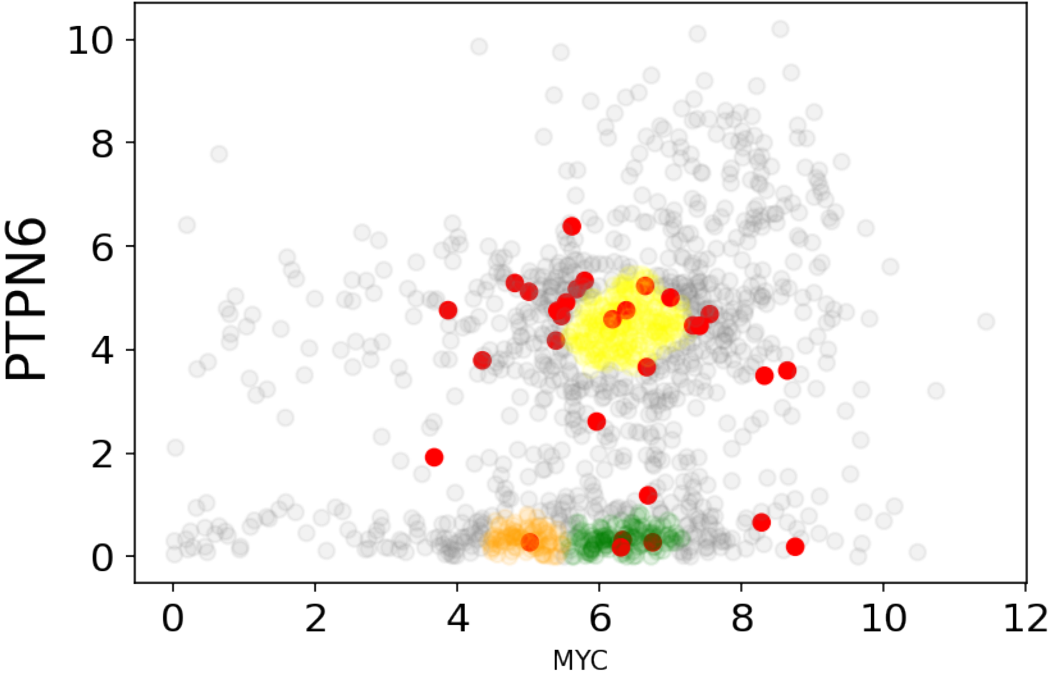

PTPN6
MYC
0
2
4
6
8
10
12

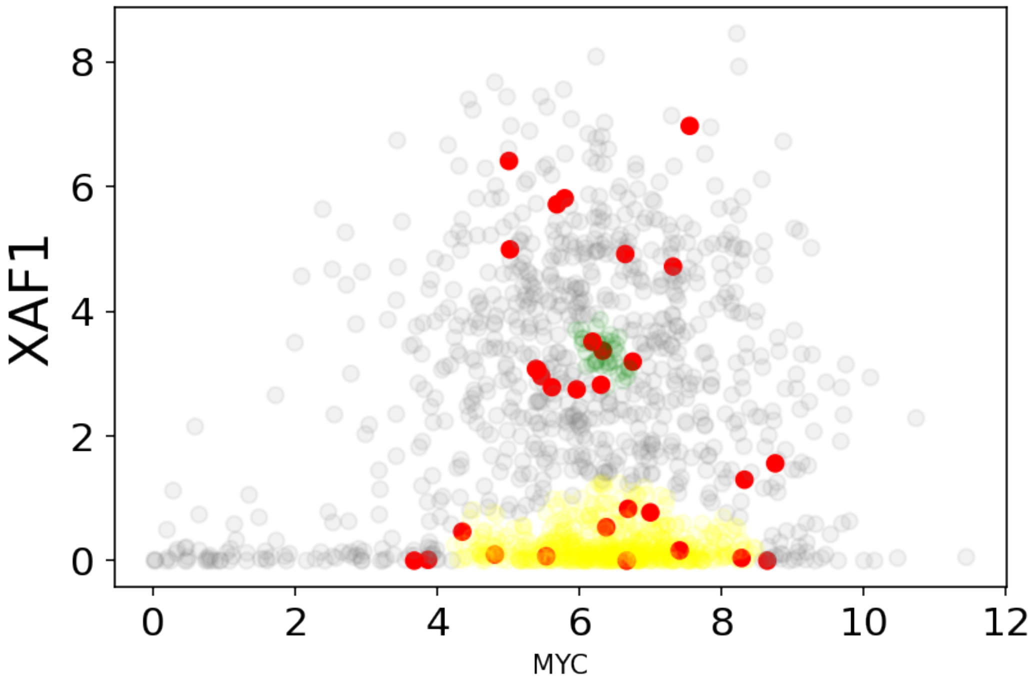

XAF1
MYC
0
2
4
6
8
10
12

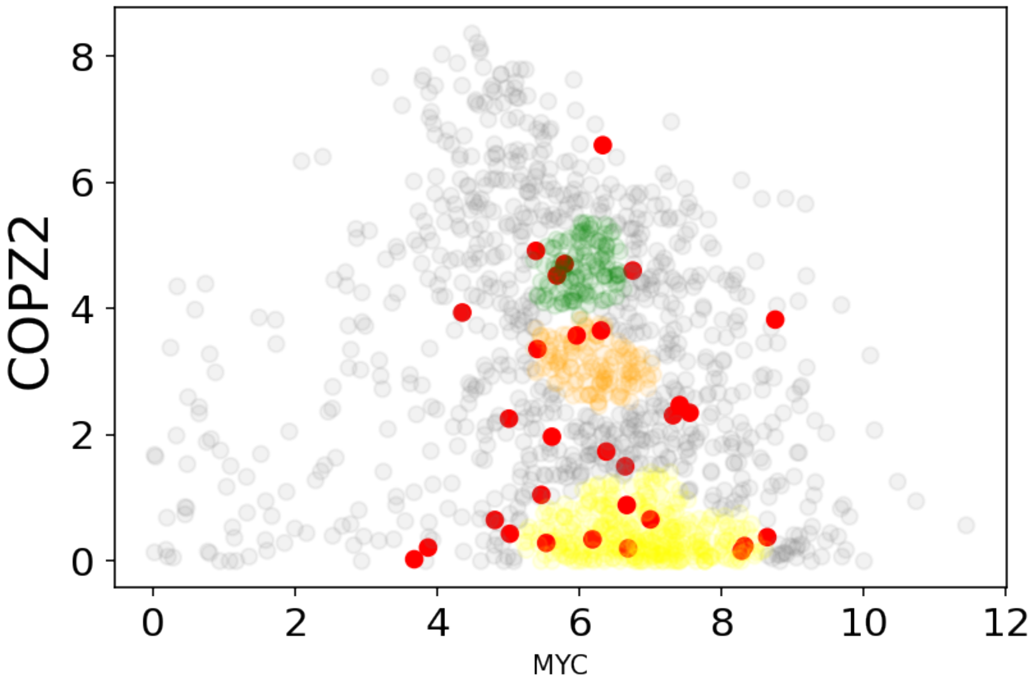
COPZ2
MYC
0
2
4
6
8
0
2
4
6
8
10
12

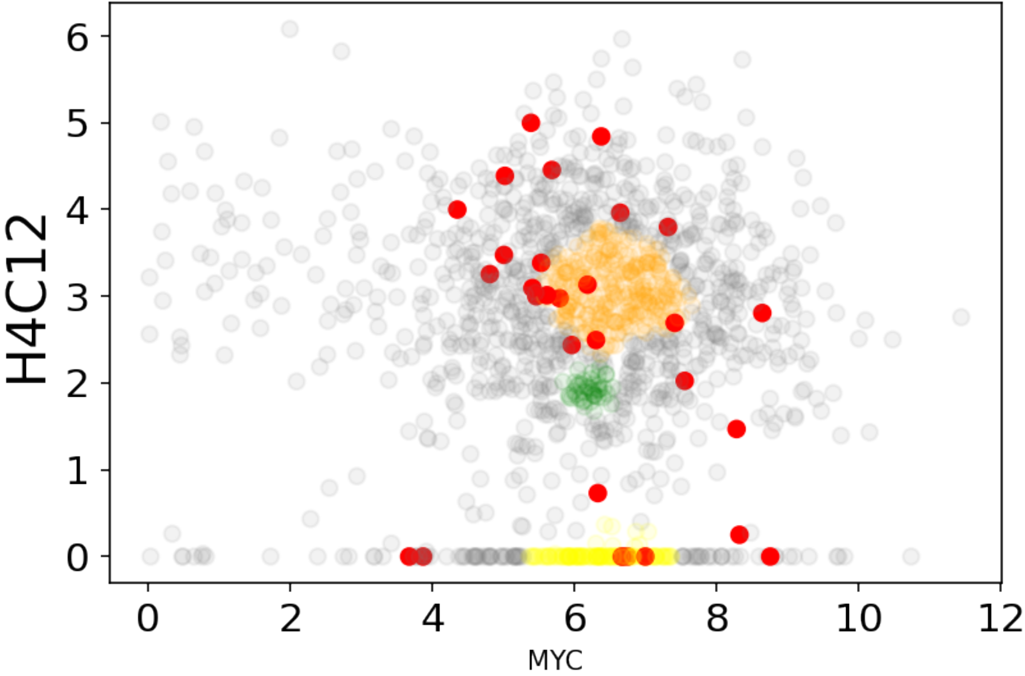

H4C12
MYC
0
1
2
3
4
5
6
0
2
4
6
8
10
12

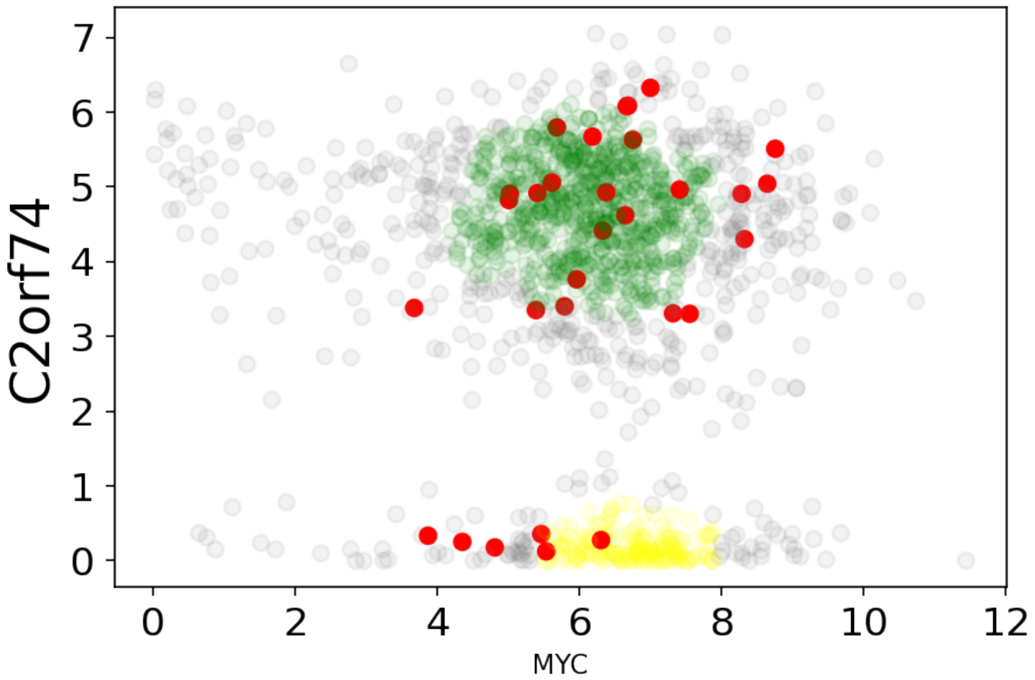

C2orf74
7
6
5
4
3
2
1
0
0
2
4
6
8
10
12
MYC

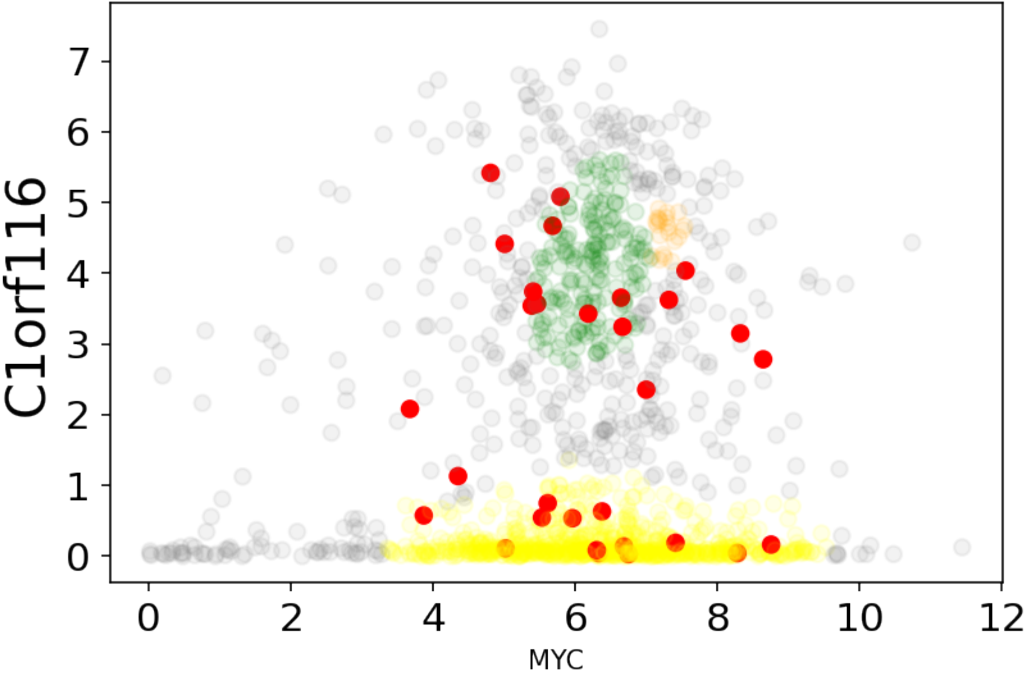
C1orf116
MYC
0
1
2
3
4
5
6
7
0
2
4
6
8
10
12

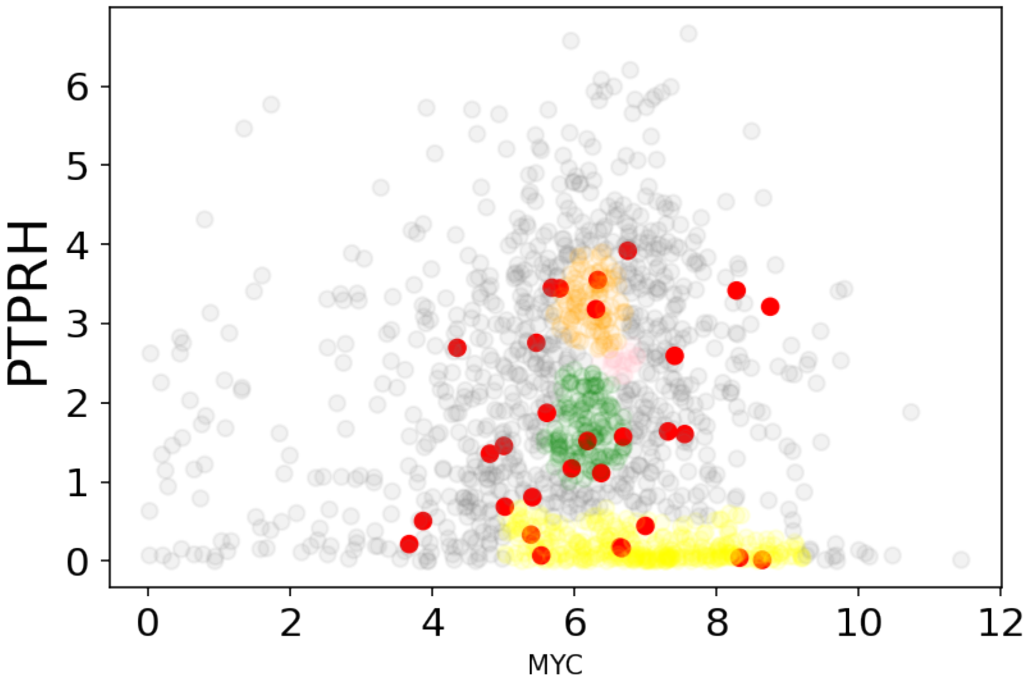

PTPRH
6
5
4
3
2
1
0
0
2
4
6
8
10
12
MYC

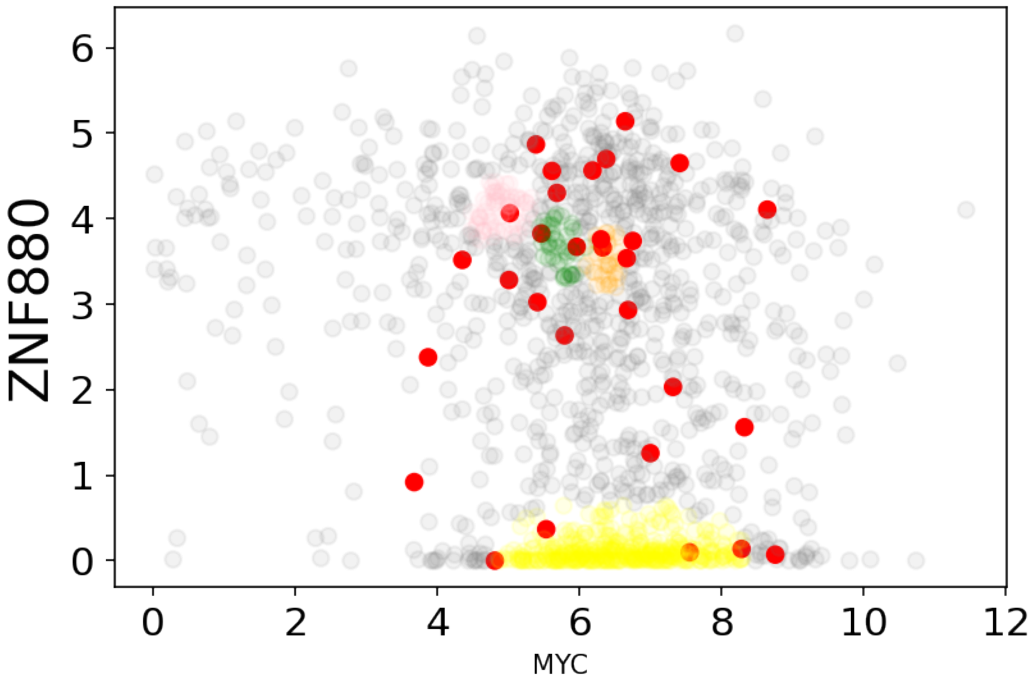

ZNF880
6
5
4
3
2
1
0
0
2
4
6
8
10
12
MYC

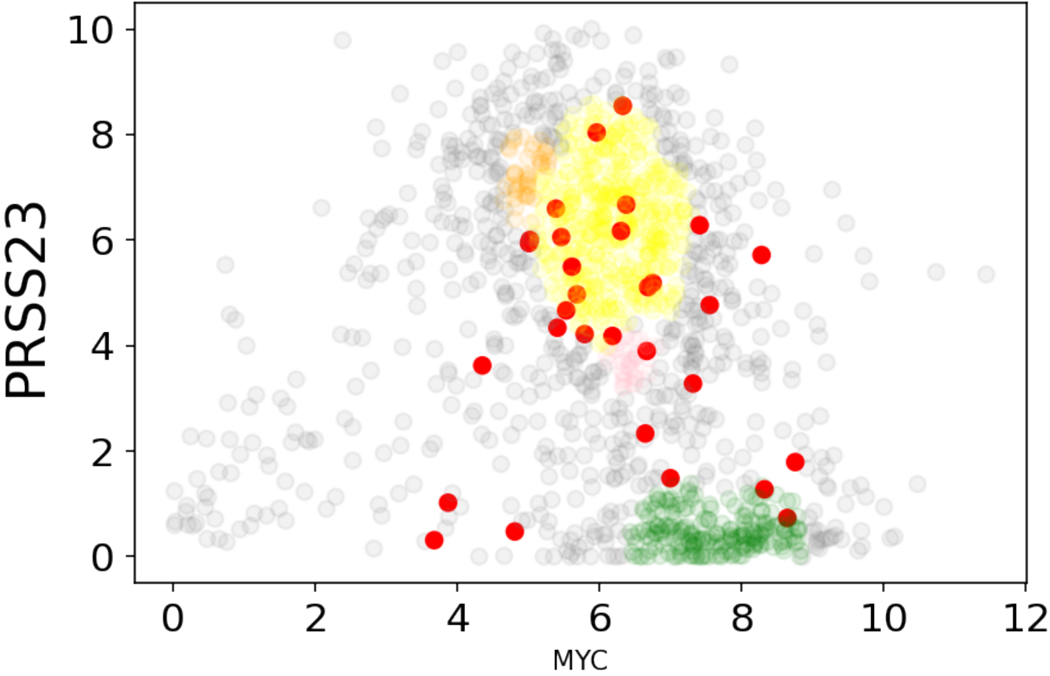

PRSS23
MYC
0
2
4
6
8
10
12

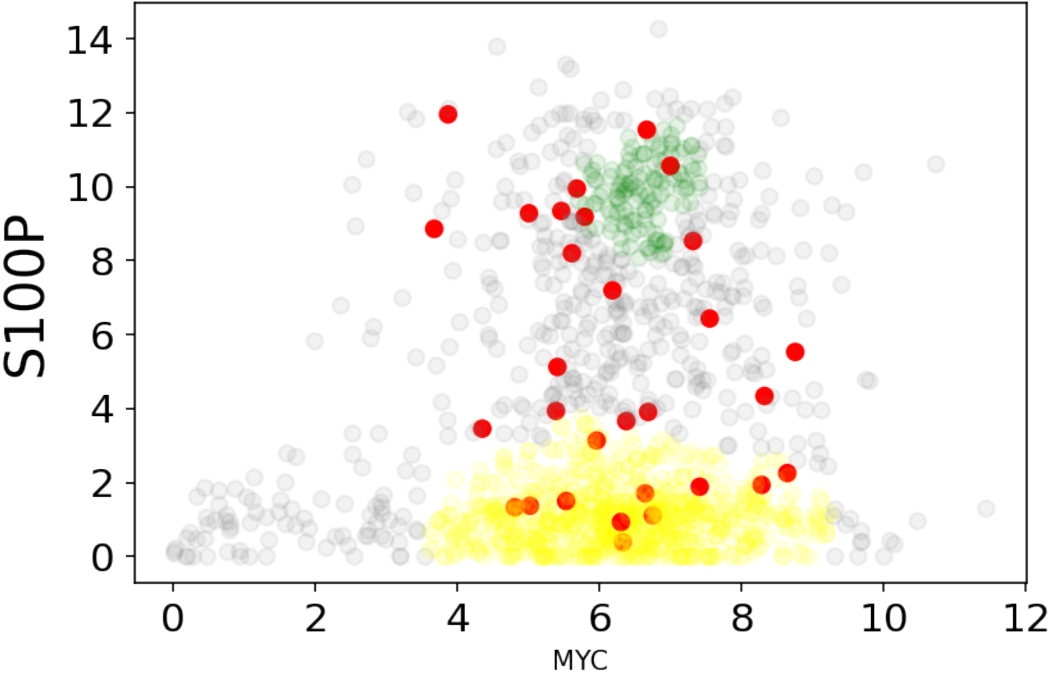

S100P
MYC
0
2
4
6
8
10
12
14
0
2
4
6
8
10
12

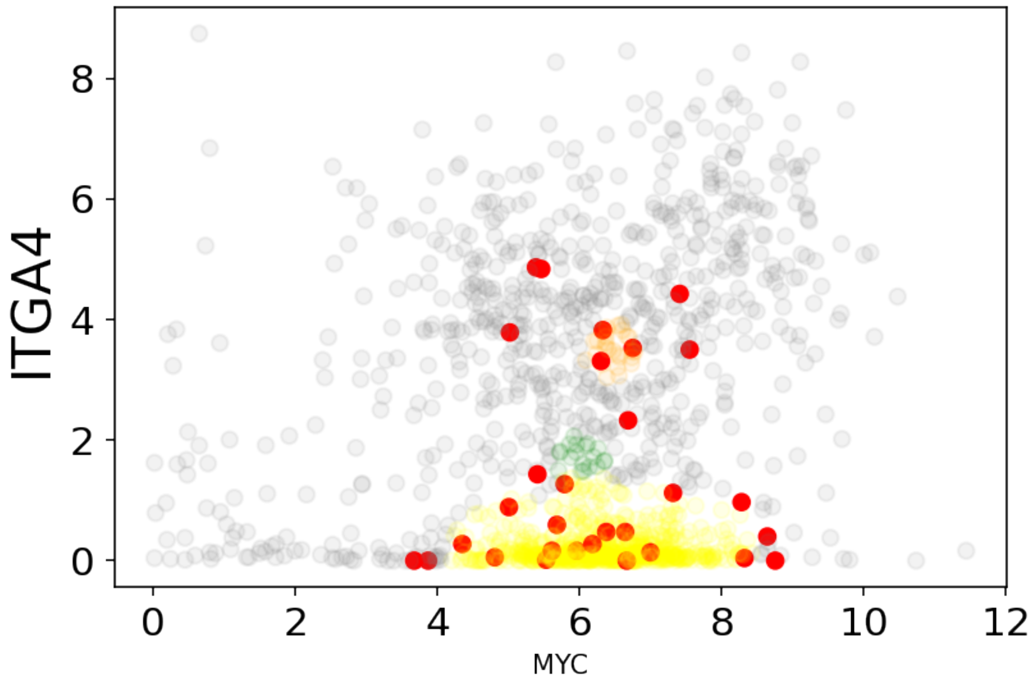
ITGA4
0
2
4
6
8
0
2
4
6
8
10
12
MYC

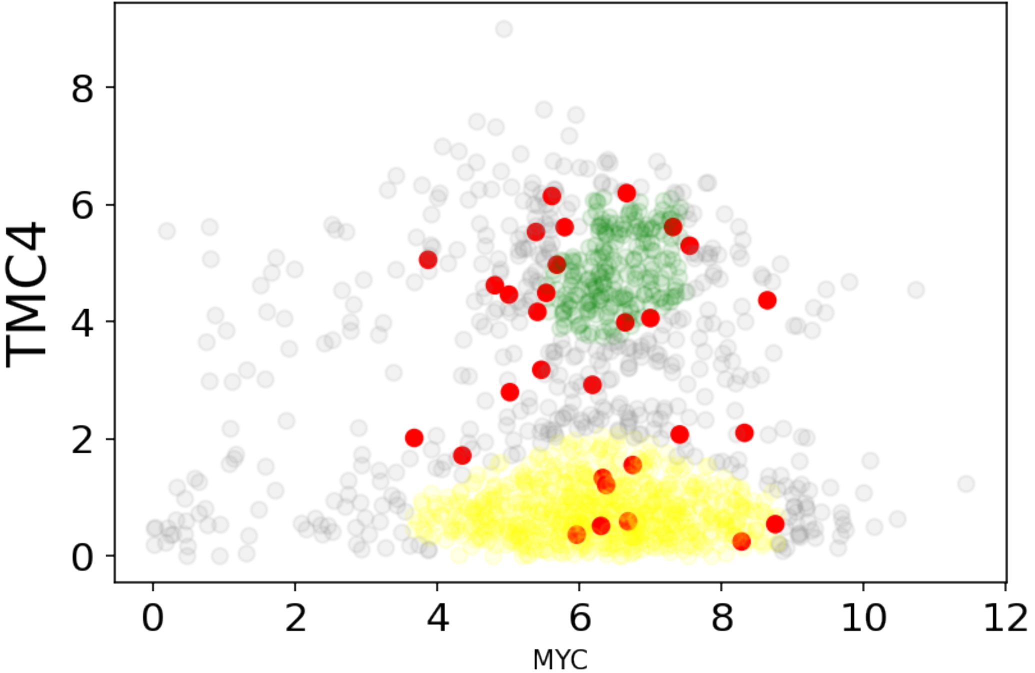

TMC4
0
2
4
6
8
MYC
0
2
4
6
8
10
12

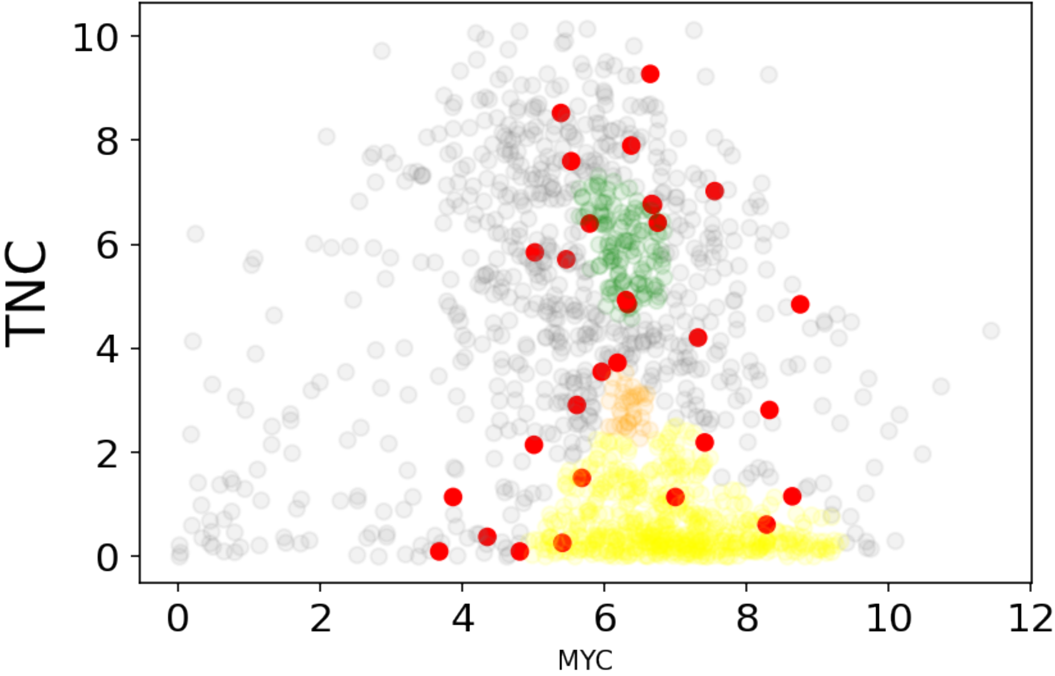
TNC
MYC
10
8
6
4
2
0
0
2
4
6
8
10
12

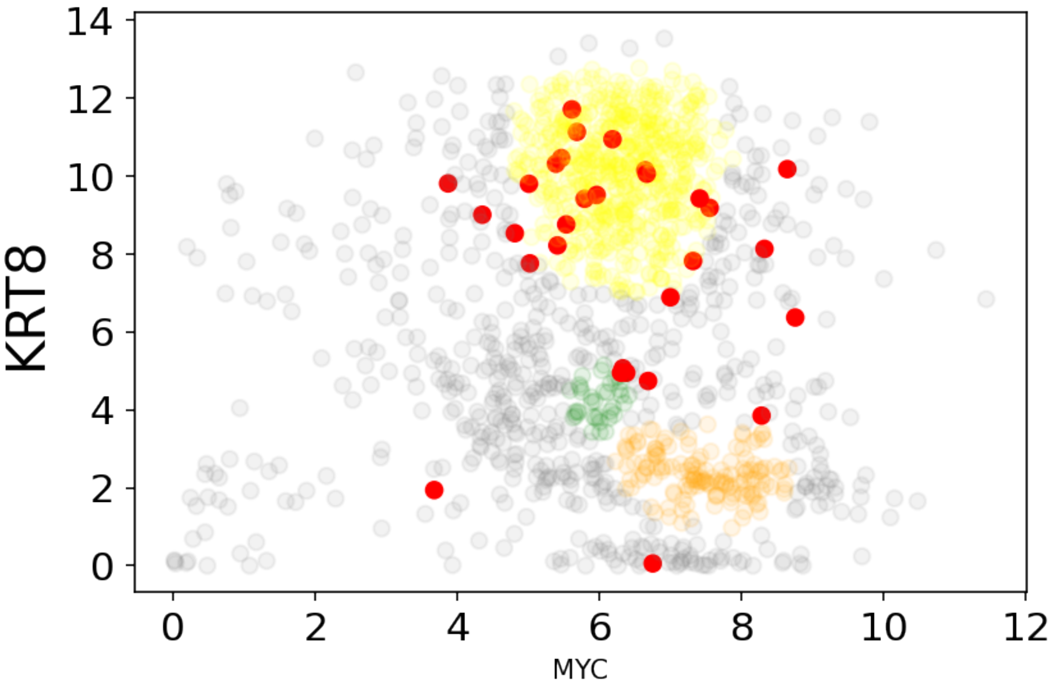

KRT8
MYC
0
2
4
6
8
10
12
14

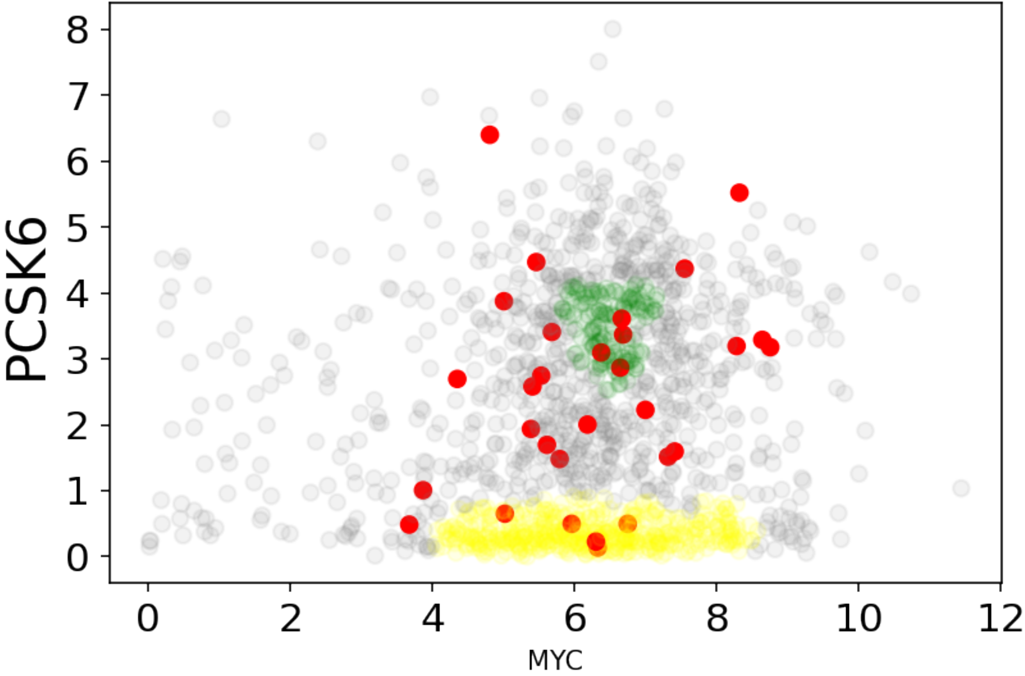

PCSK6
0
1
2
3
4
5
6
7
8
0
2
4
6
8
10
12
MYC

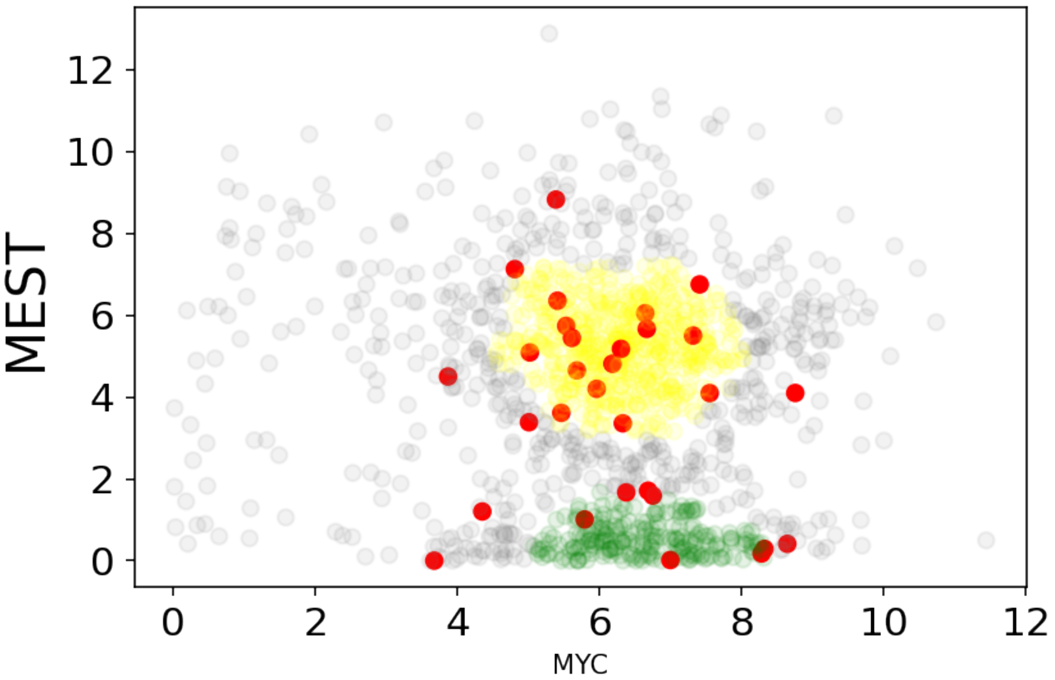

MEST
MYC
0
2
4
6
8
10
12

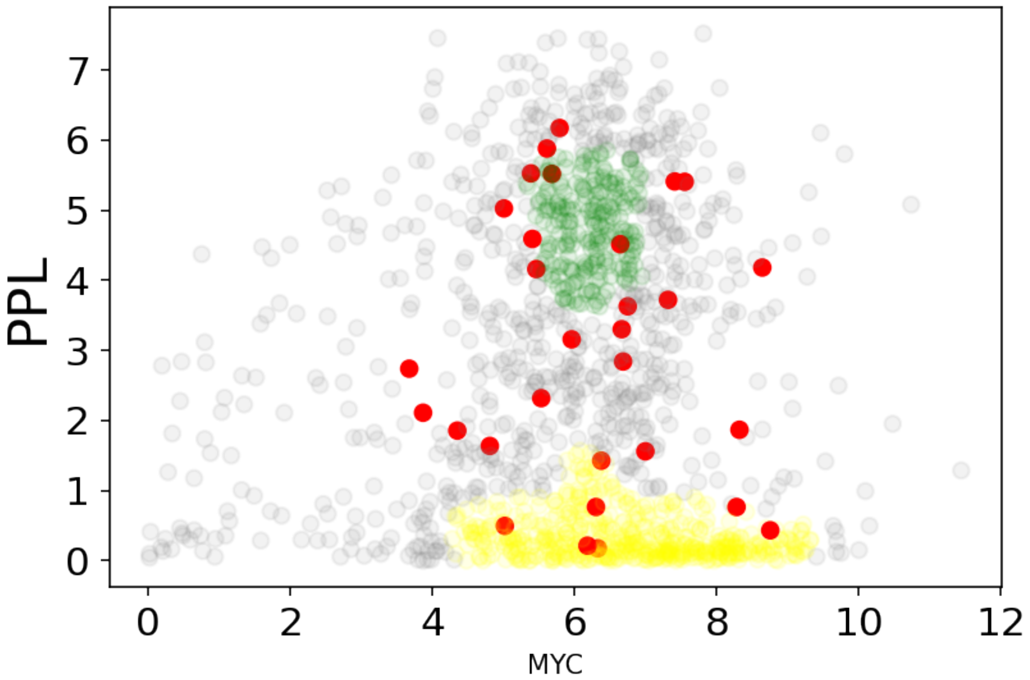

PPL
0
1
2
3
4
5
6
7
0
2
4
6
8
10
12
MYC

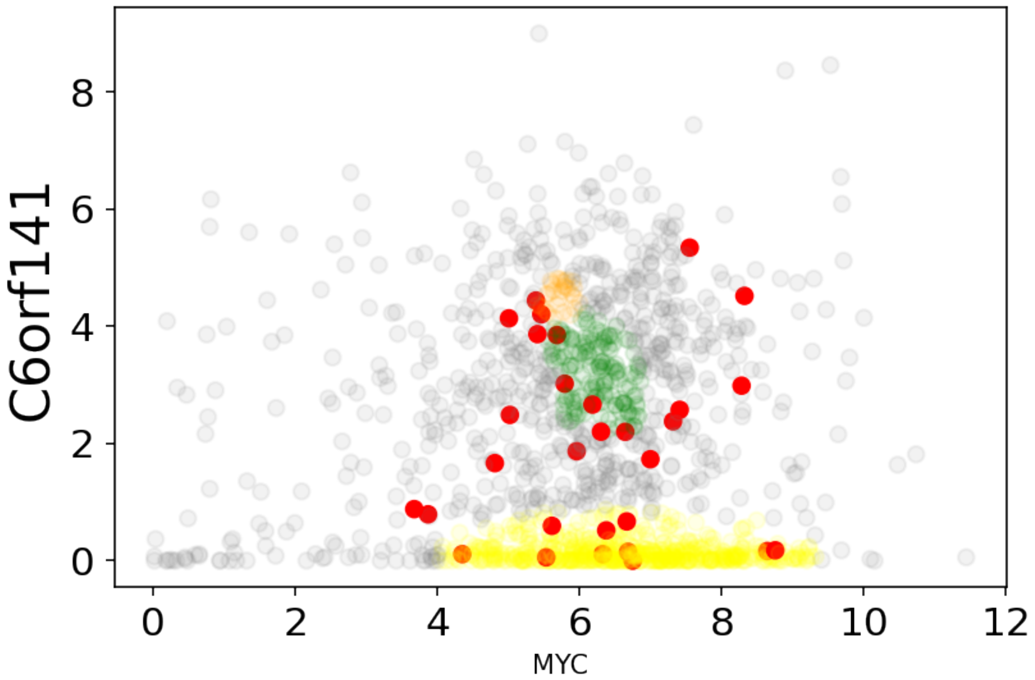
C6orf141
8
6
4
2
0
0
2
4
6
8
10
12
MYC

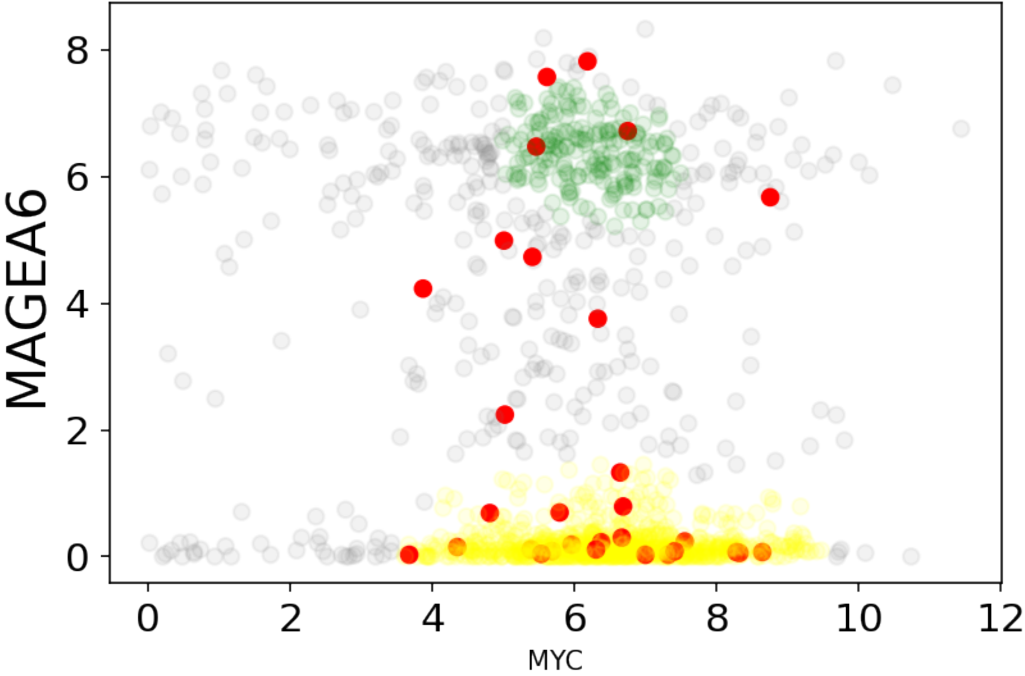

MAGEA6
MYC
0
2
4
6
8
10
12

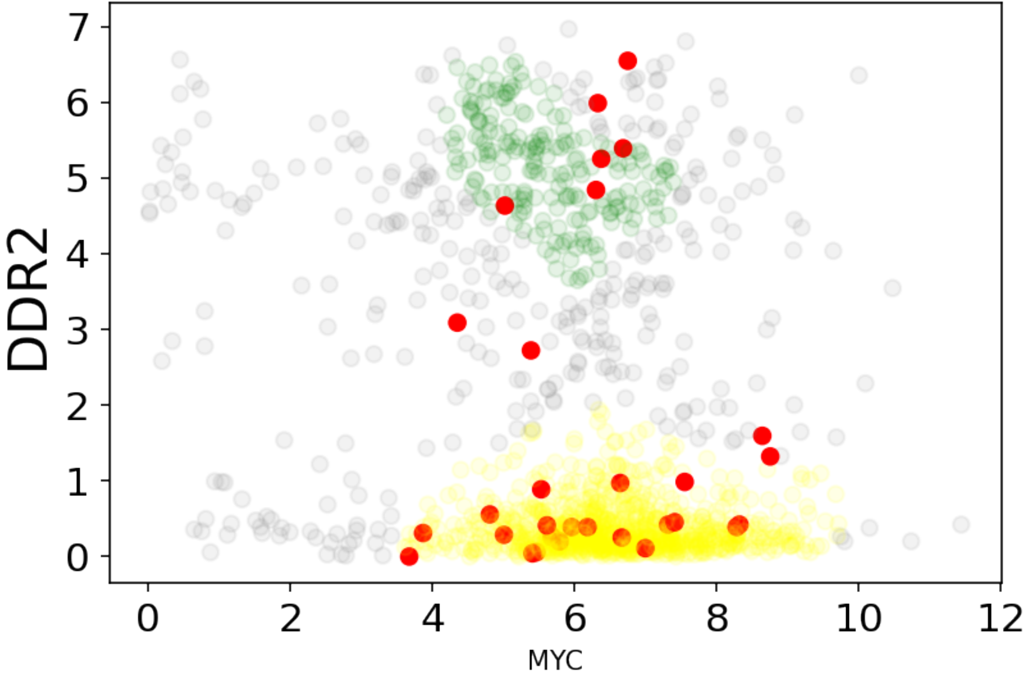

DDR2
MYC
0
1
2
3
4
5
6
7
0
2
4
6
8
10
12

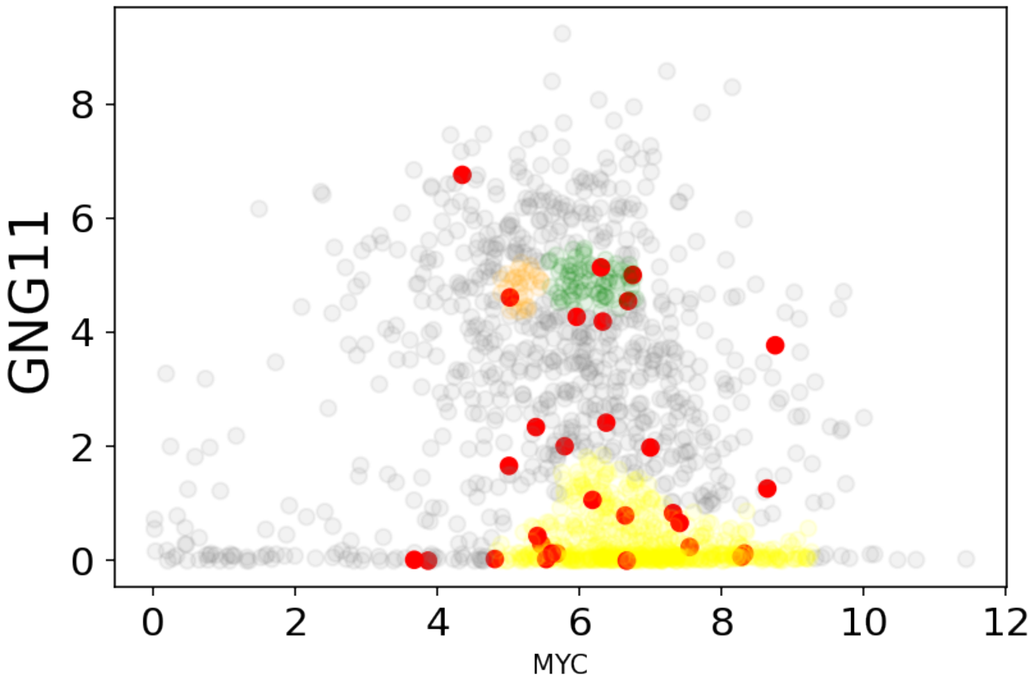

GNG11
8
6
4
2
0
0
2
4
6
8
10
12
MYC

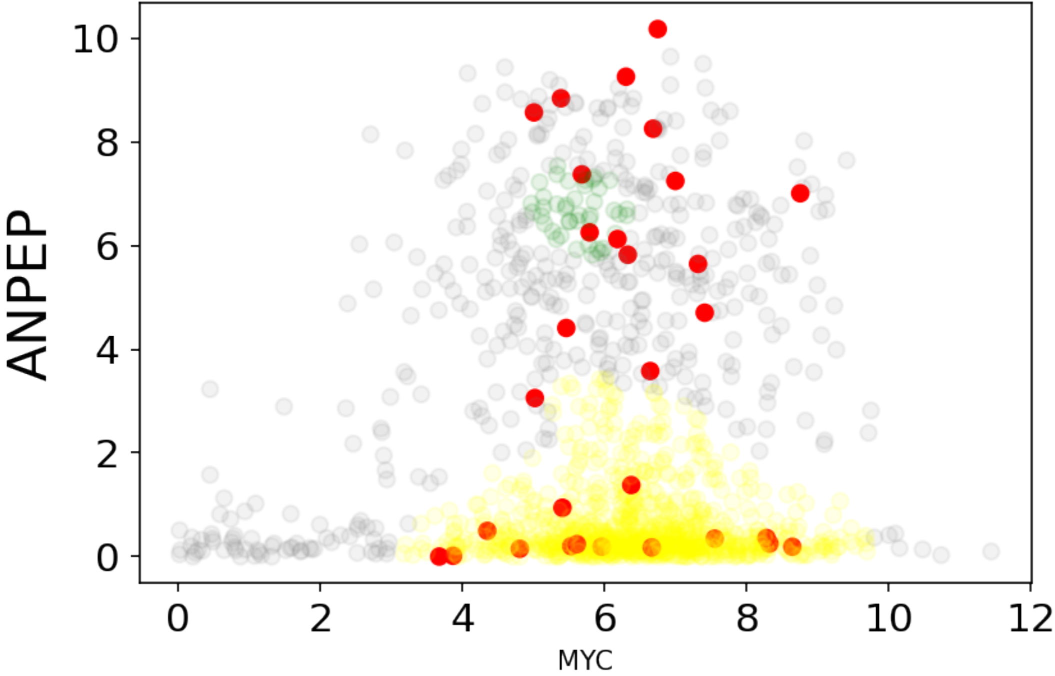

ANPEP
10
8
6
4
2
0
0
2
4
6
8
10
12
MYC

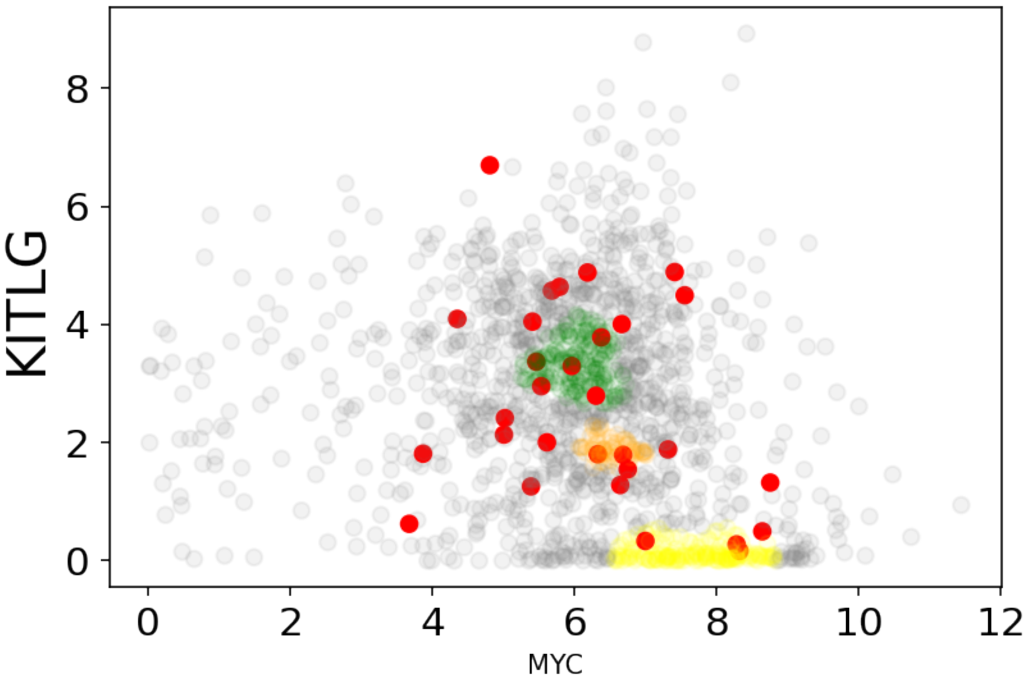

KITLG
8
6
4
2
0
0
2
4
6
8
10
12
MYC

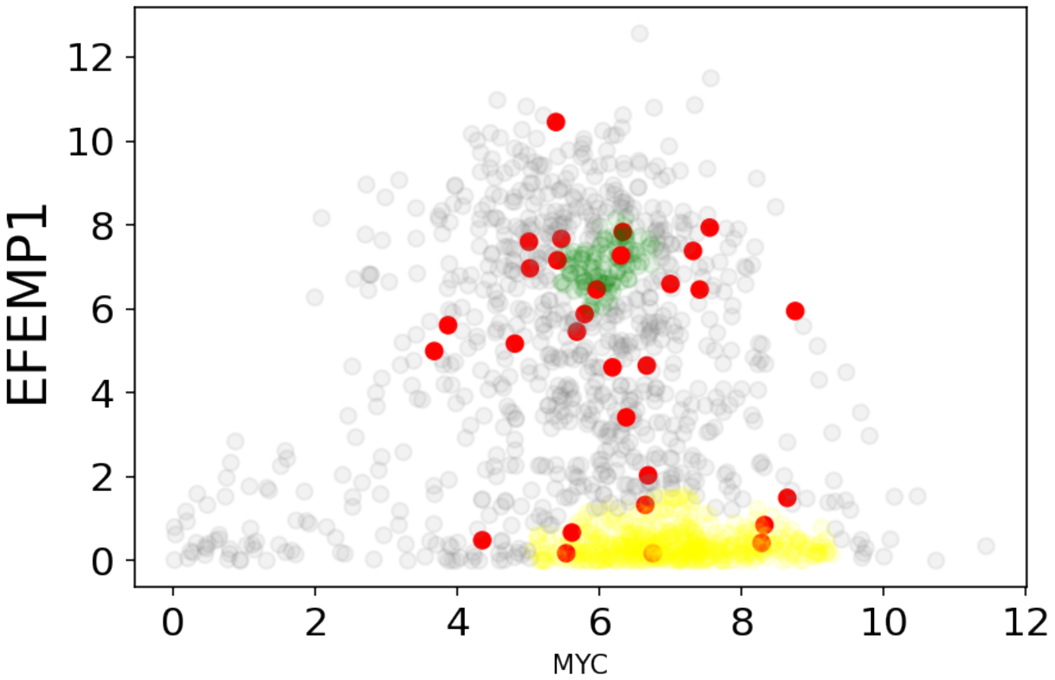

EFEMP1
0
2
4
6
8
10
12
MYC

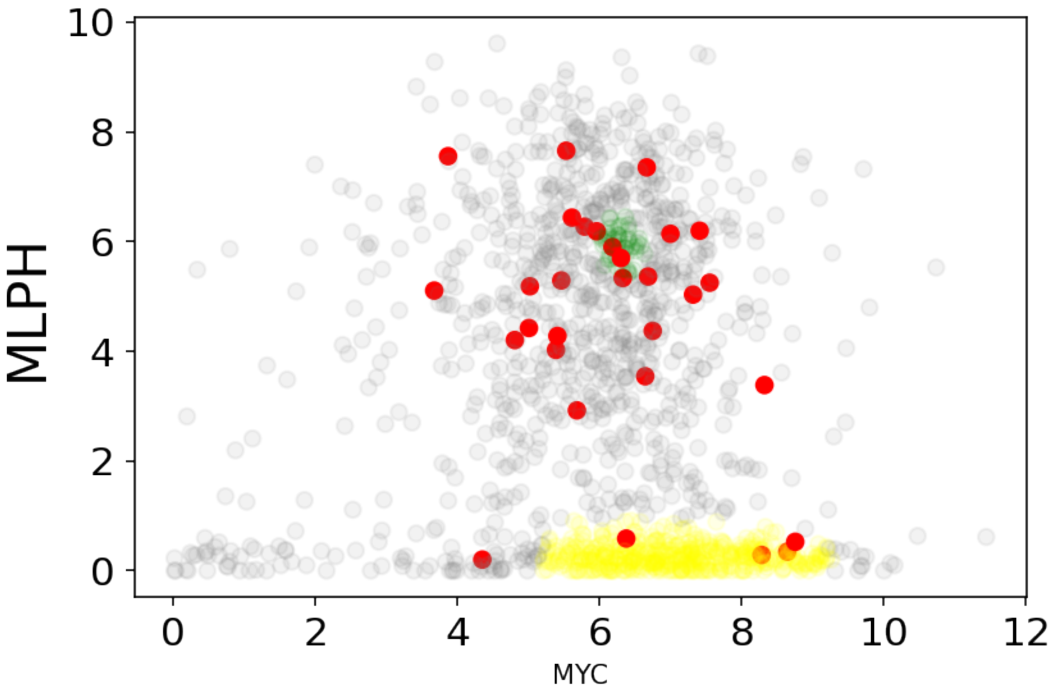
MLPH
MYC
0
2
4
6
8
10
12

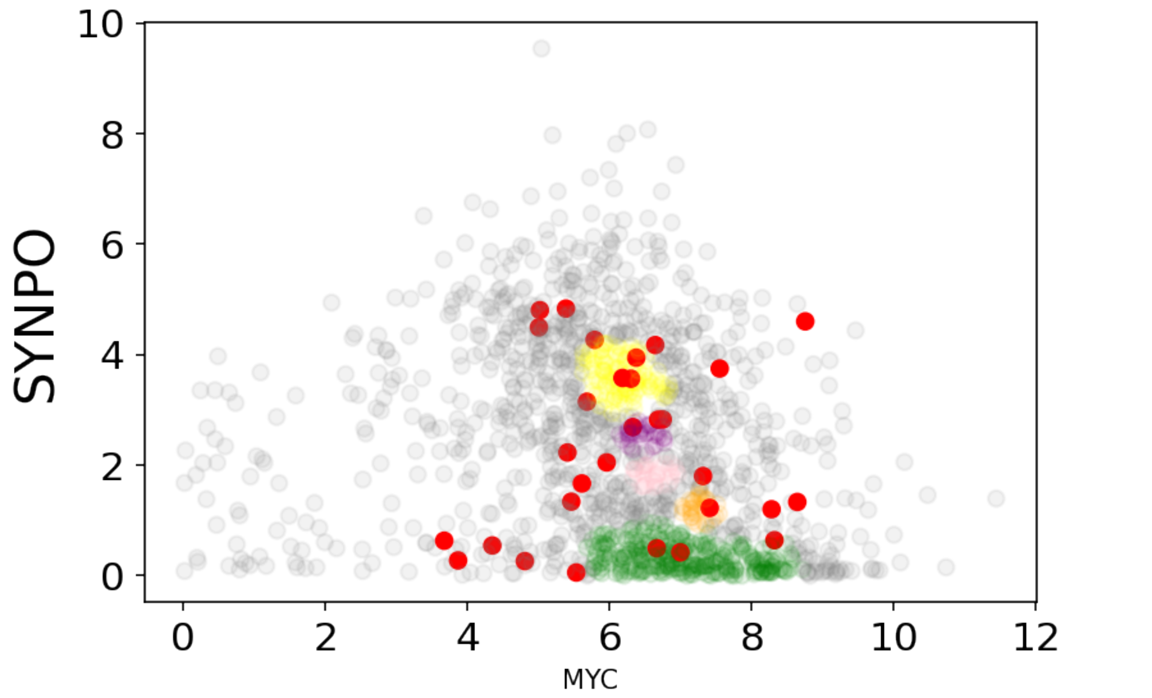

SYNPO
MYC
0
2
4
6
8
10
12

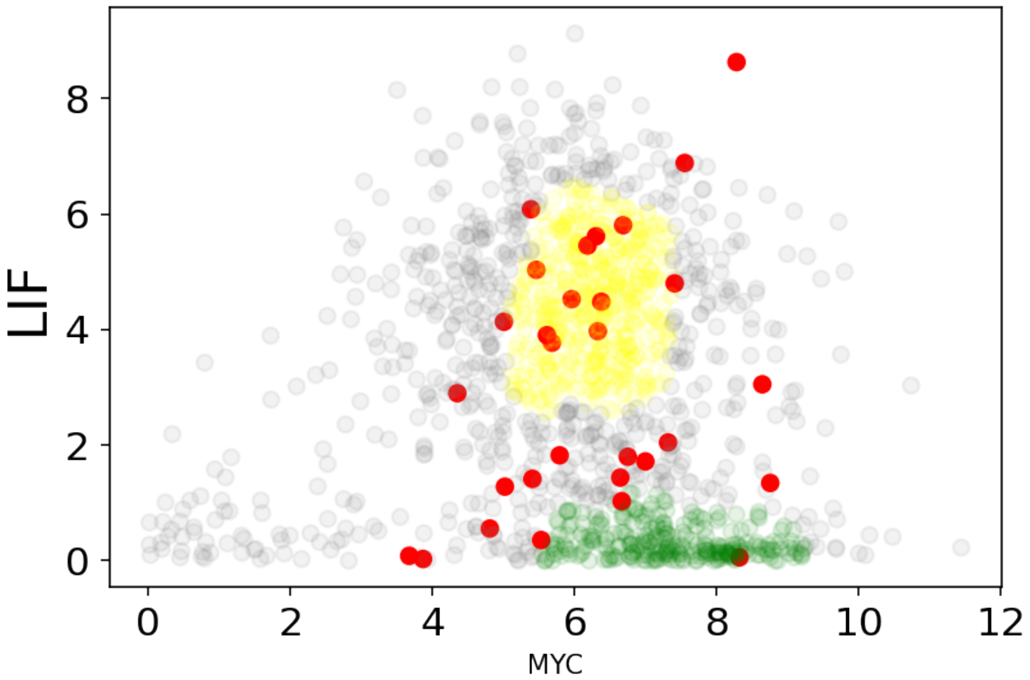
LIF
MYC
0
2
4
6
8
0
2
4
6
8
10
12

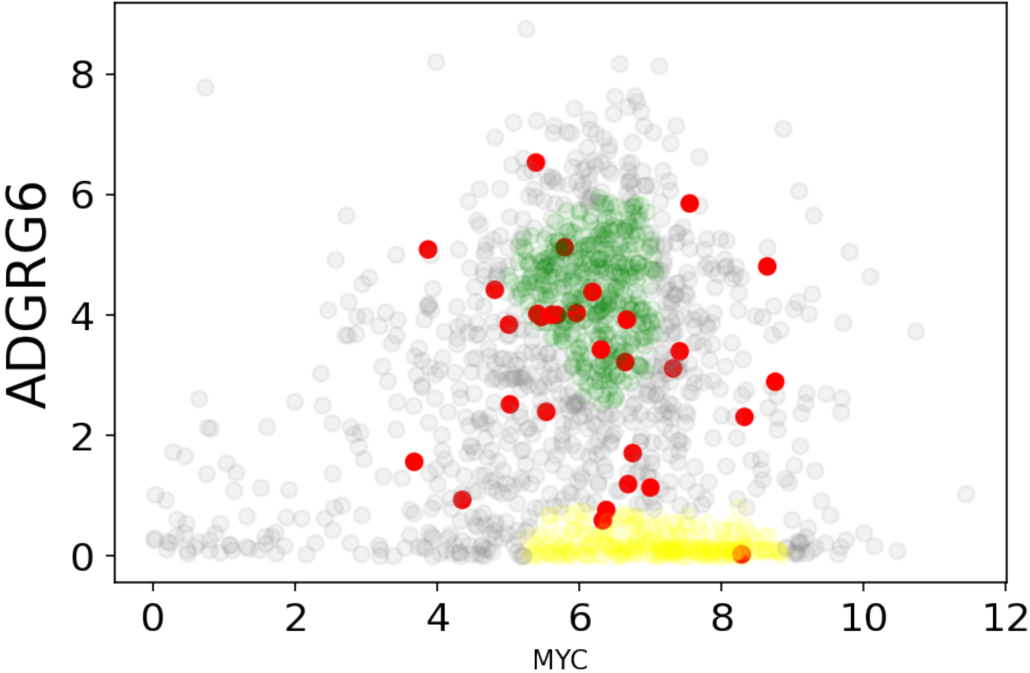
ADGRG6
MYC
0
2
4
6
8
0
2
4
6
8
10
12

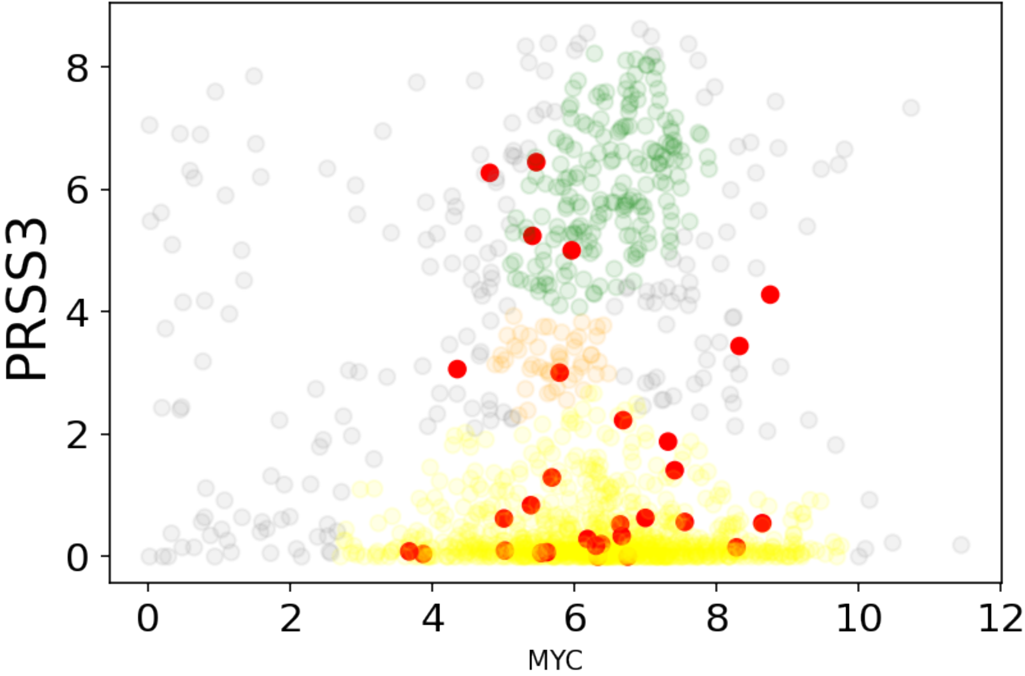

PRSS3
8
6
4
2
0
0
2
4
6
8
10
12
MYC

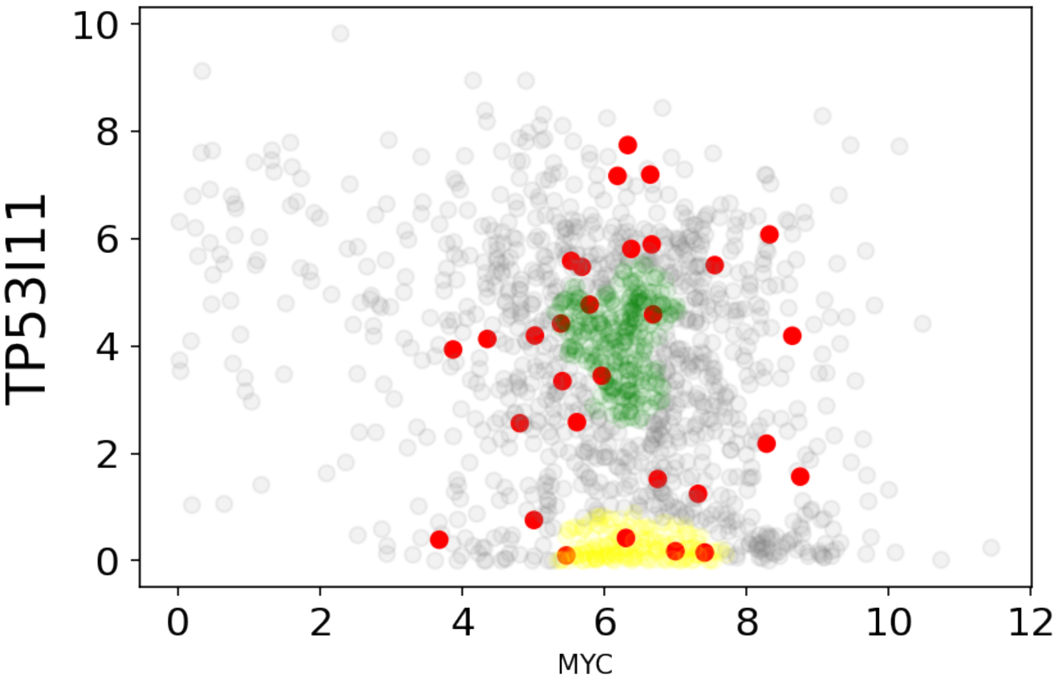
TP53I11
MYC
0
2
4
6
8
10
12

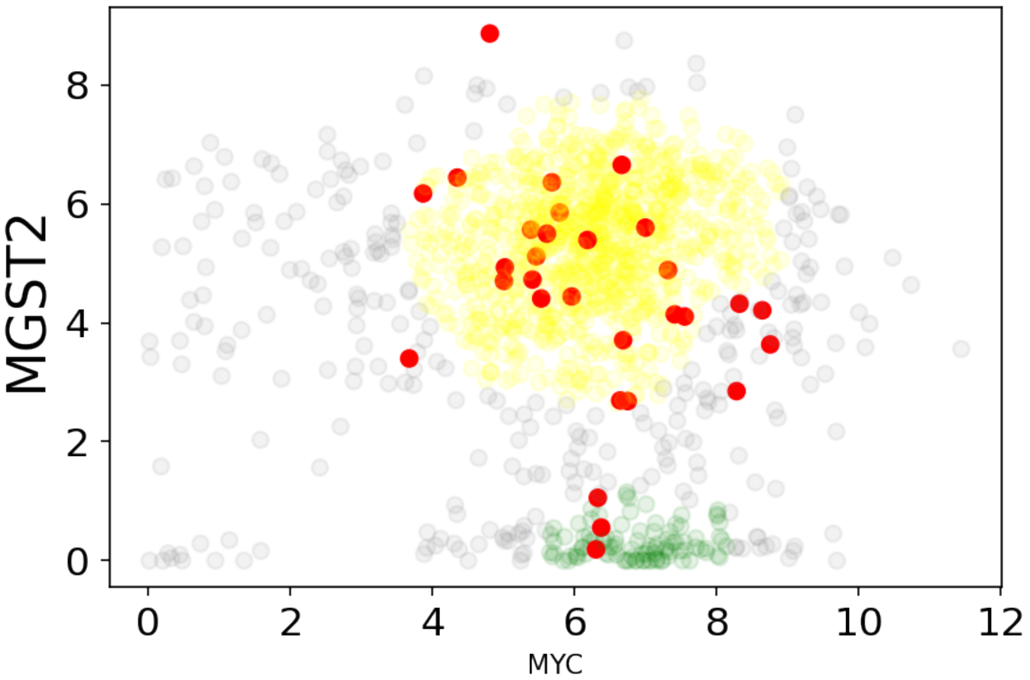

MGST2
0
2
4
6
8
0
2
4
6
8
10
12
MYC

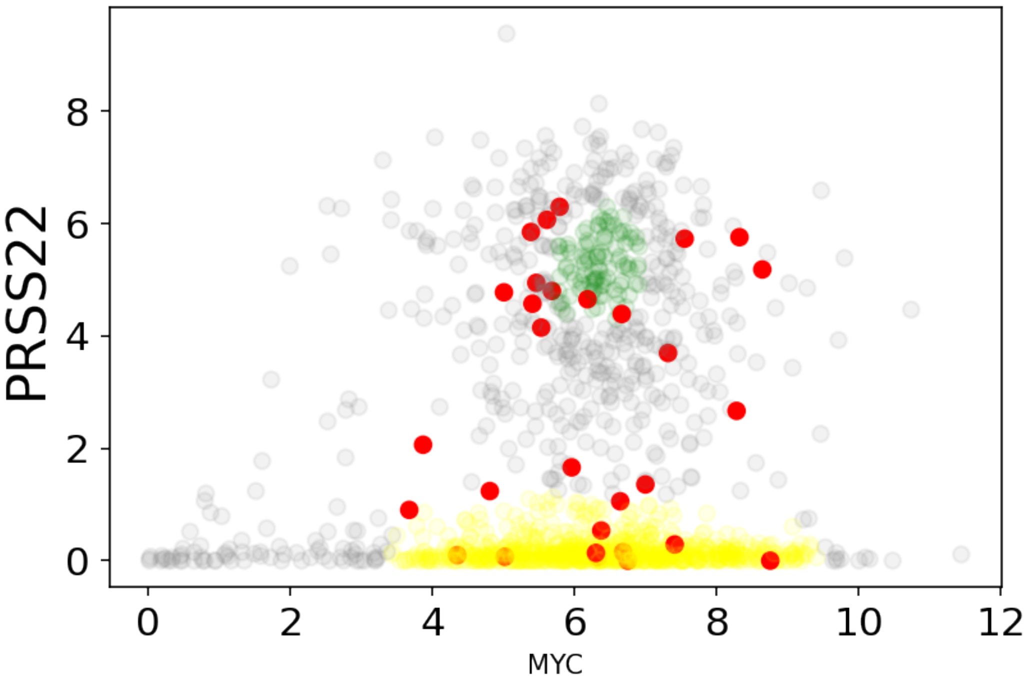
PRSS22
MYC
0
2
4
6
8
10
12

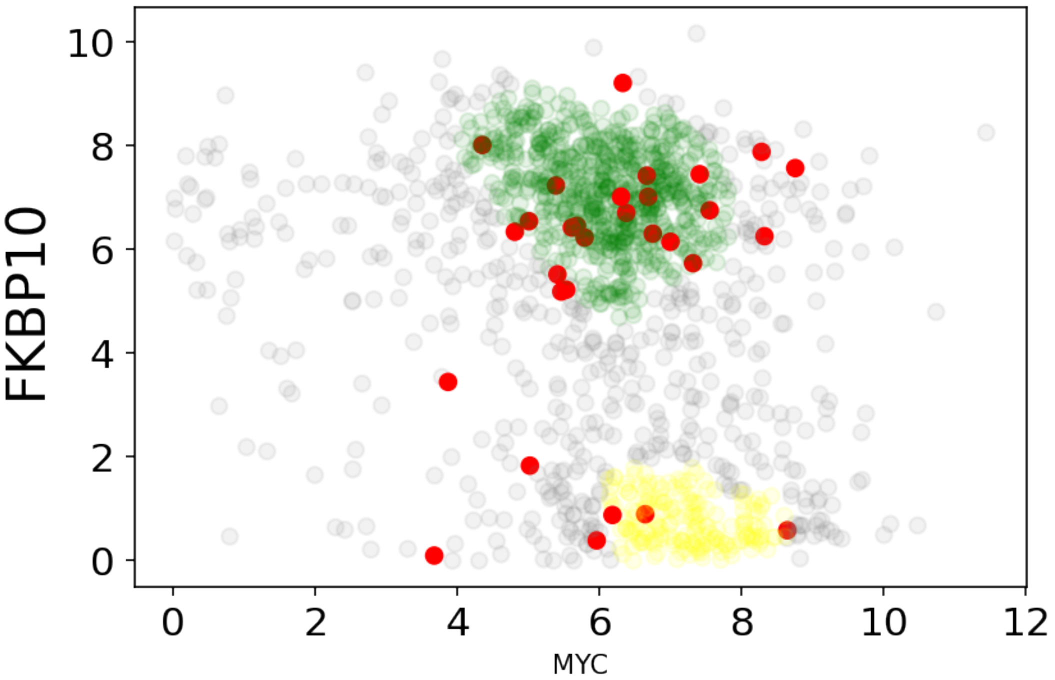

FKBP10
MYC
10
8
6
4
2
0
0
2
4
6
8
10
12

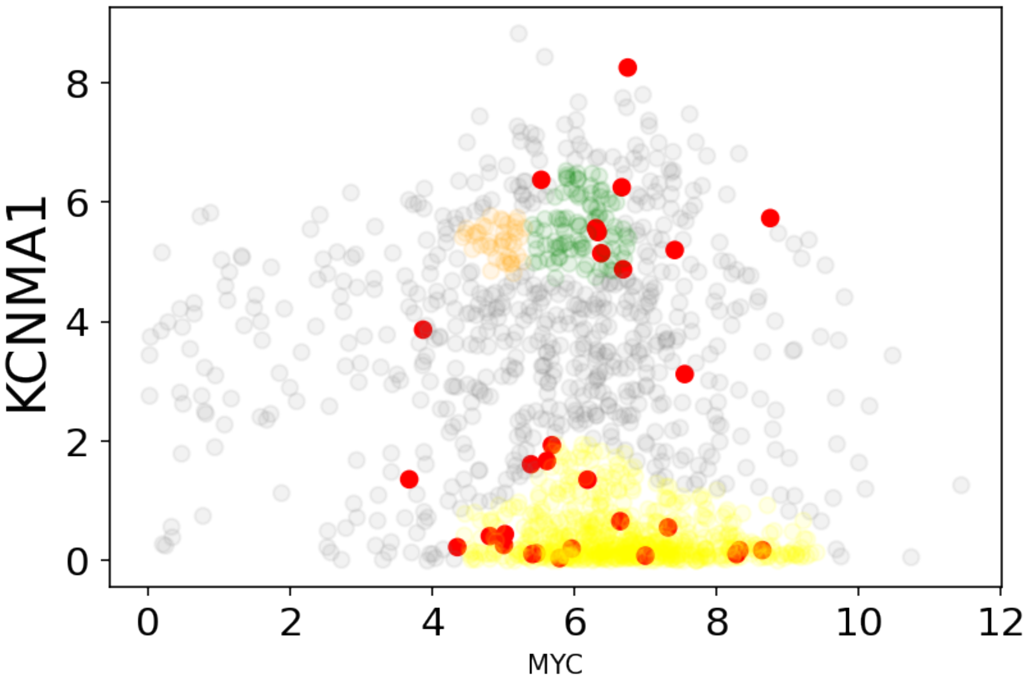
KCNMA1
0
2
4
6
8
0
2
4
6
8
10
12
MYC

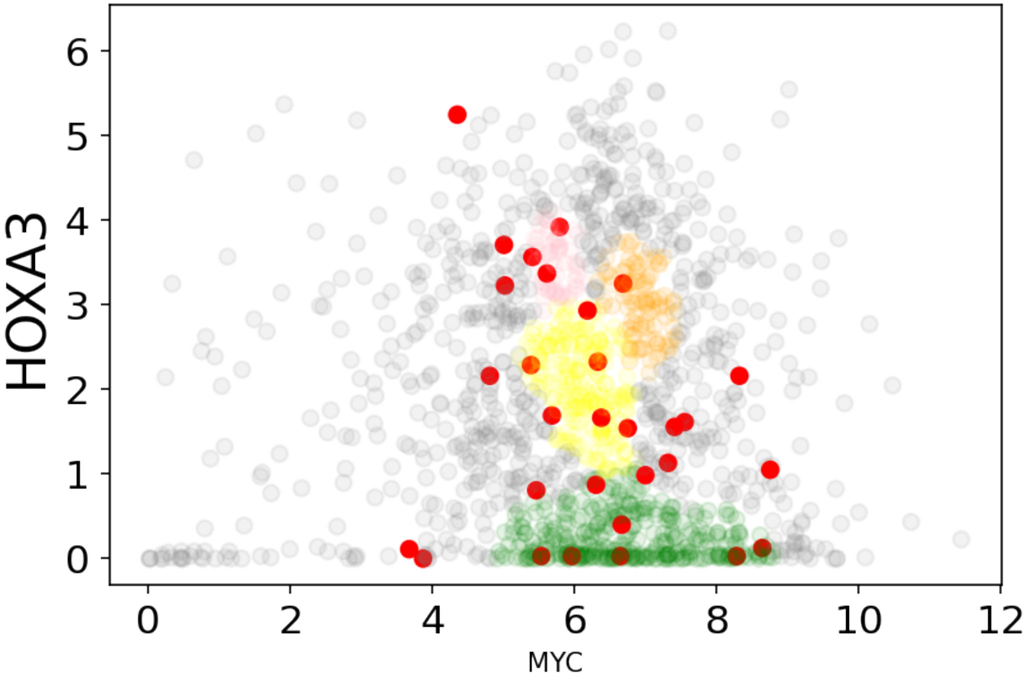

HOXA3
0
1
2
3
4
5
6
0
2
4
6
8
10
12
MYC

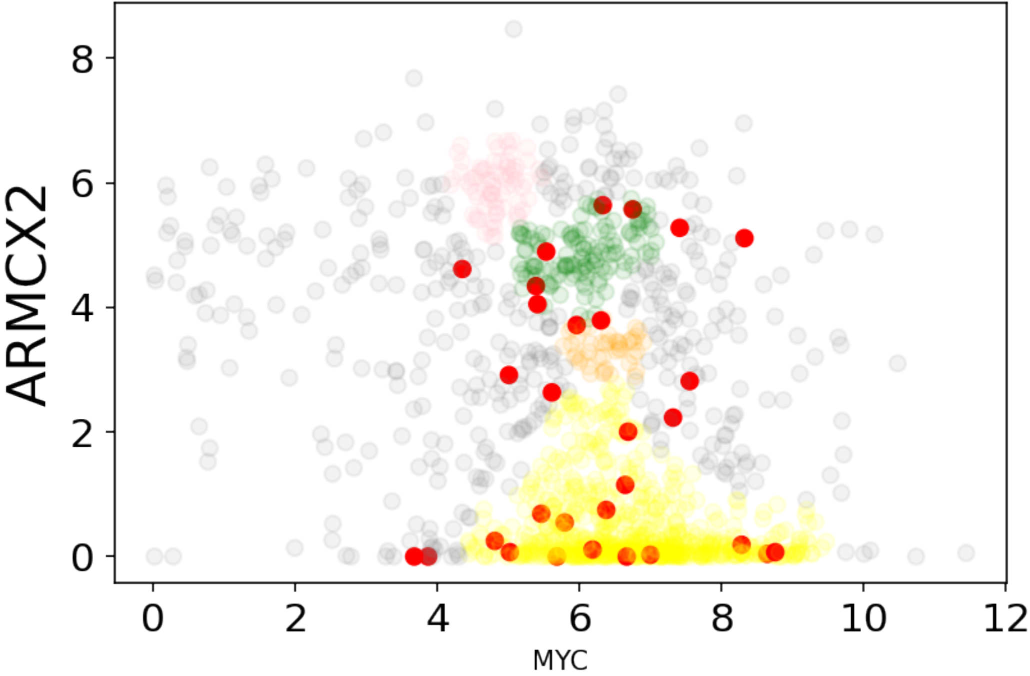
ARMCX2
MYC
0
2
4
6
8
10
12

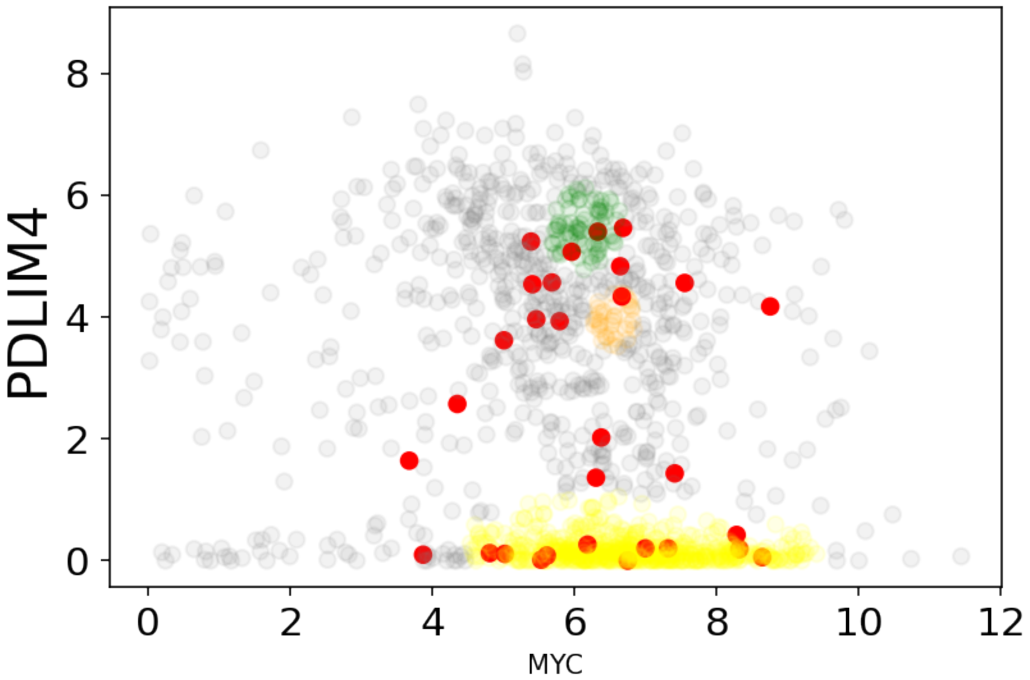

PDLIM4
MYC
0
2
4
6
8
10
12

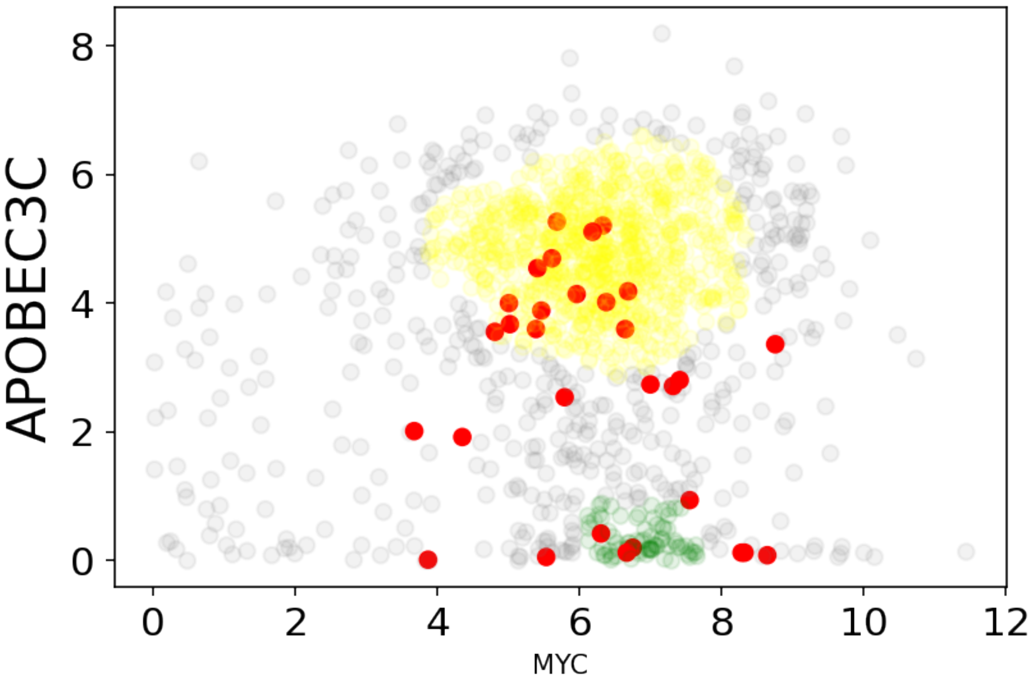

APOBEC3C
MYC
0
2
4
6
8
10
12

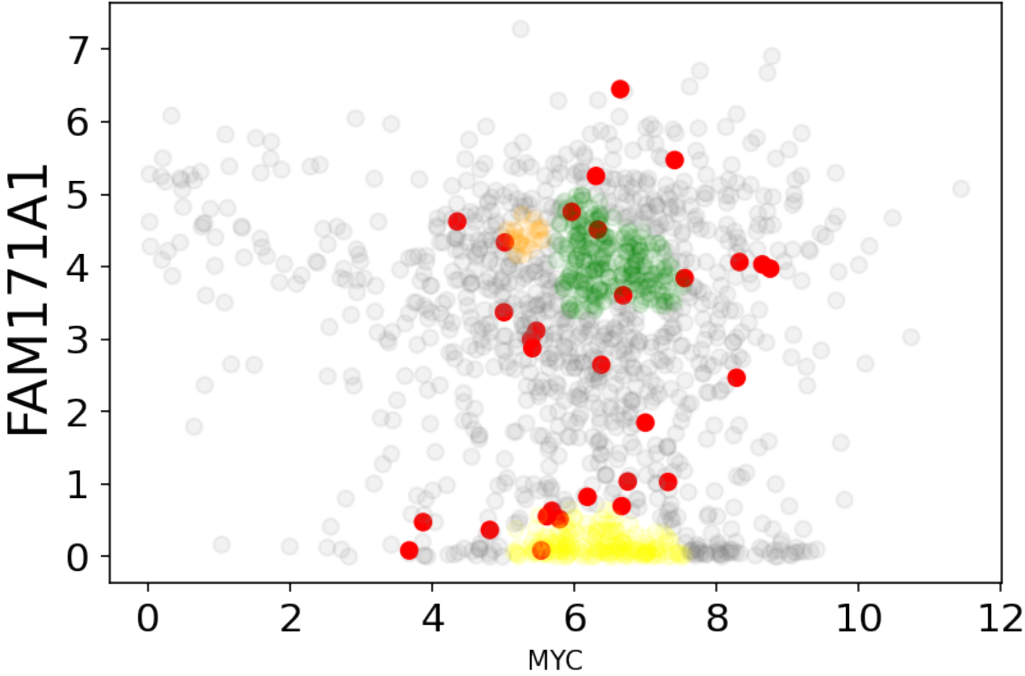

FAM171A1
MYC
0
1
2
3
4
5
6
7
0
2
4
6
8
10
12

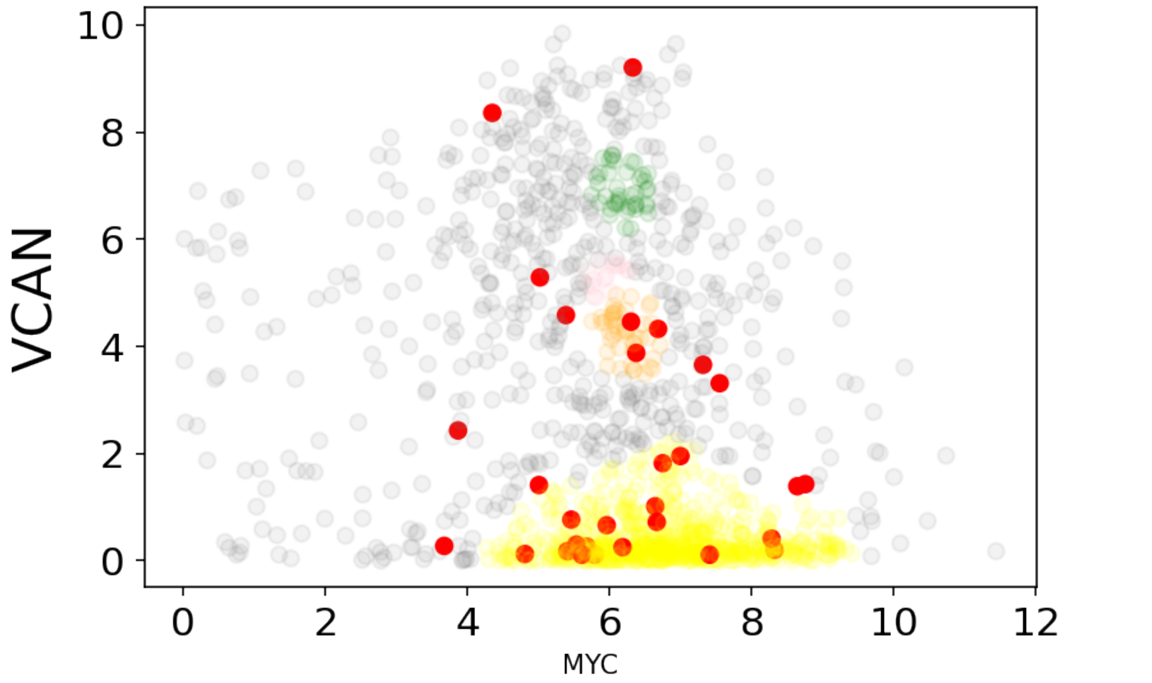

VCAN
MYC
10
8
6
4
2
0
0
2
4
6
8
10
12

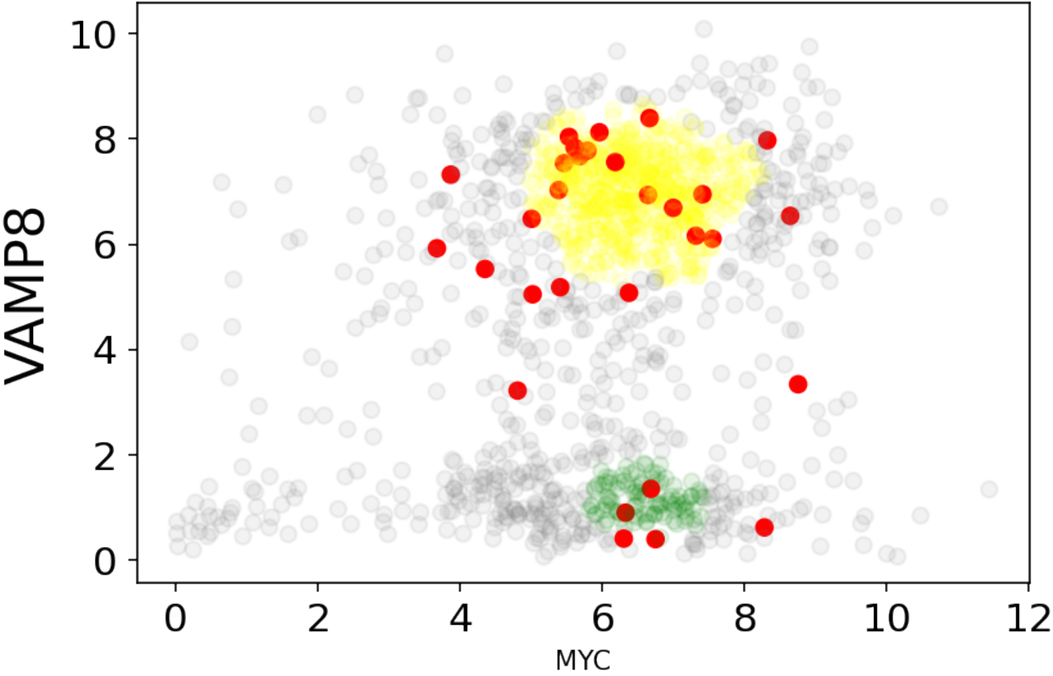
VAMP8
MYC
0
2
4
6
8
10
12

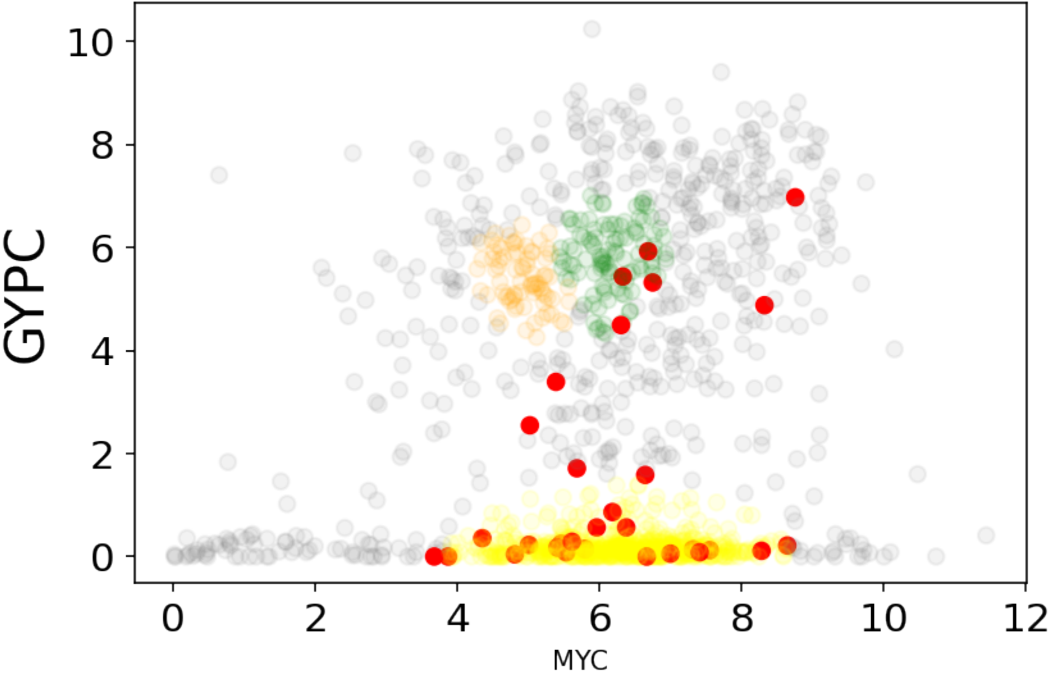
GYPC
MYC
0
2
4
6
8
10
12

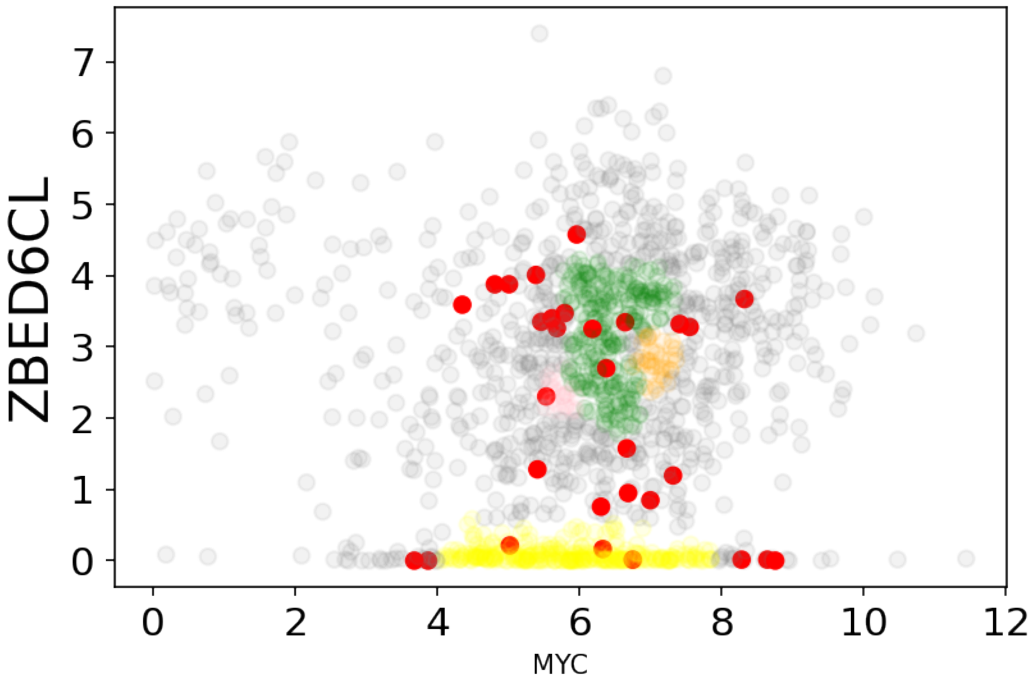
ZBED6CL
MYC
0
1
2
3
4
5
6
7
0
2
4
6
8
10
12

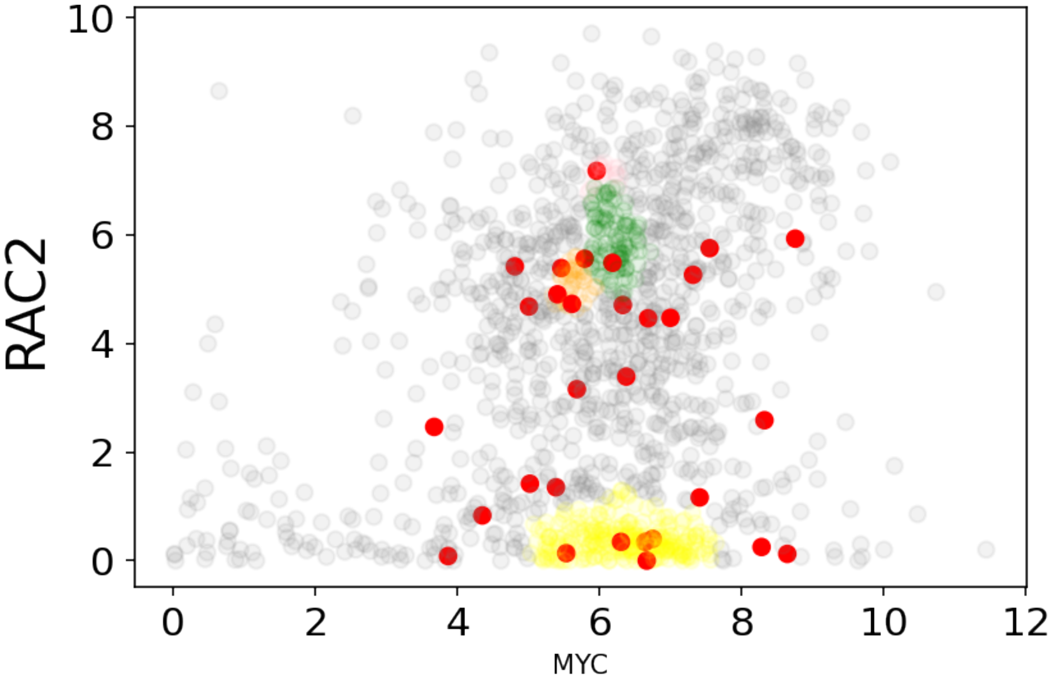

RAC2
MYC
10
8
6
4
2
0
0
2
4
6
8
10
12

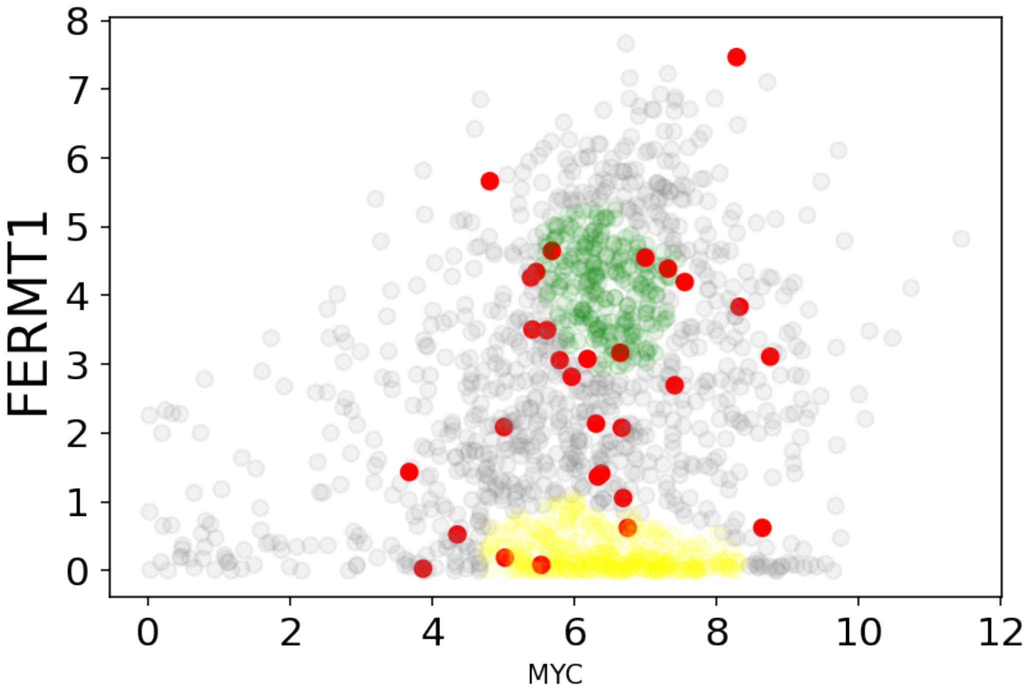

FERMT1
MYC
0
1
2
3
4
5
6
7
8
0
2
4
6
8
10
12

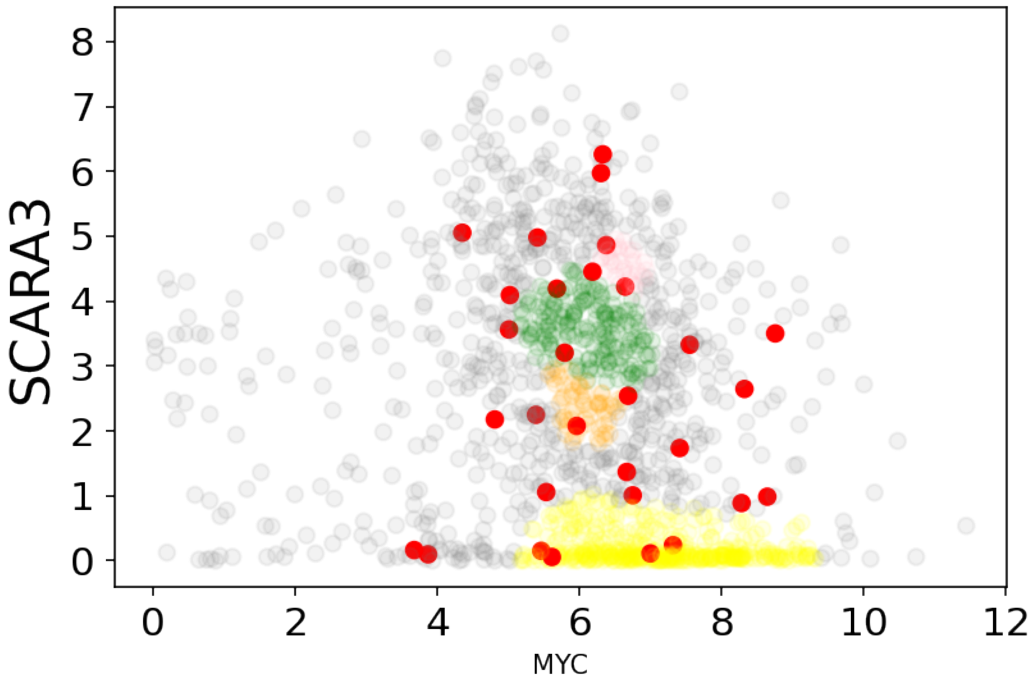

SCARA3
MYC
0
1
2
3
4
5
6
7
8
0
2
4
6
8
10
12

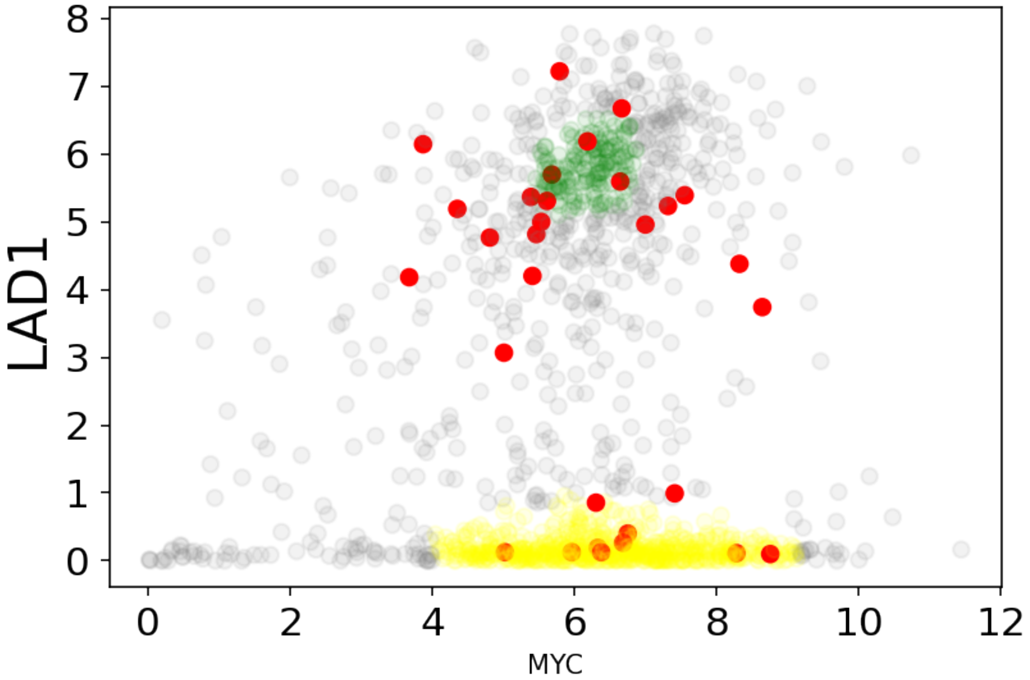

LAD1
MYC
0
1
2
3
4
5
6
7
8
0
2
4
6
8
10
12

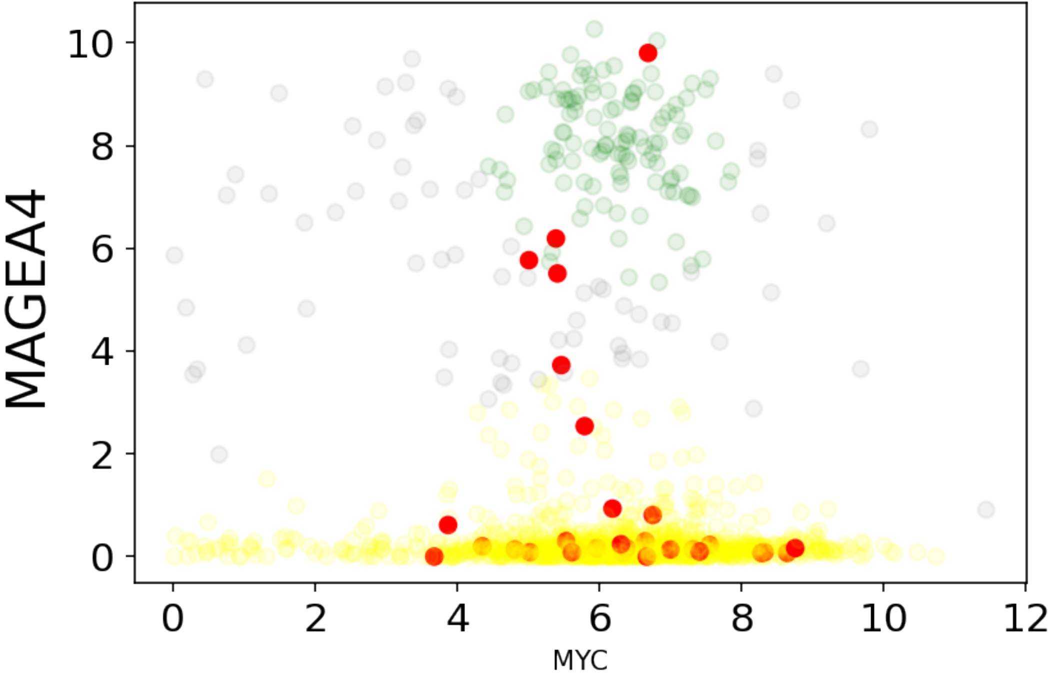
MAGEA4
MYC
0
2
4
6
8
10
12

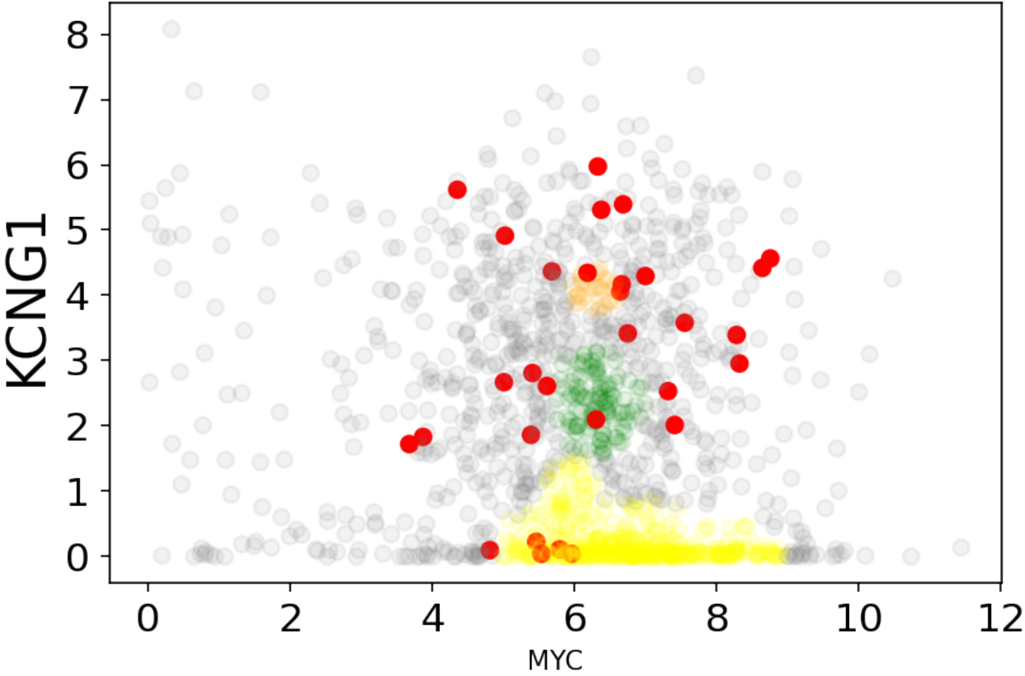

KCNG1
MYC
0
1
2
3
4
5
6
7
8
0
2
4
6
8
10
12

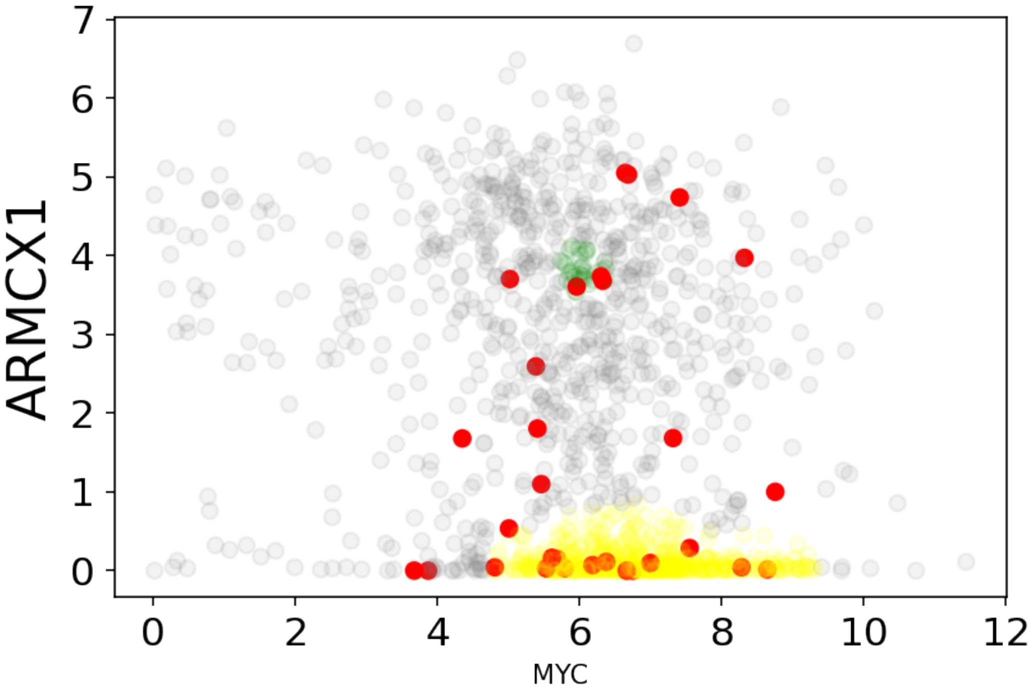

ARMCX1
MYC
0
1
2
3
4
5
6
7
0
2
4
6
8
10
12

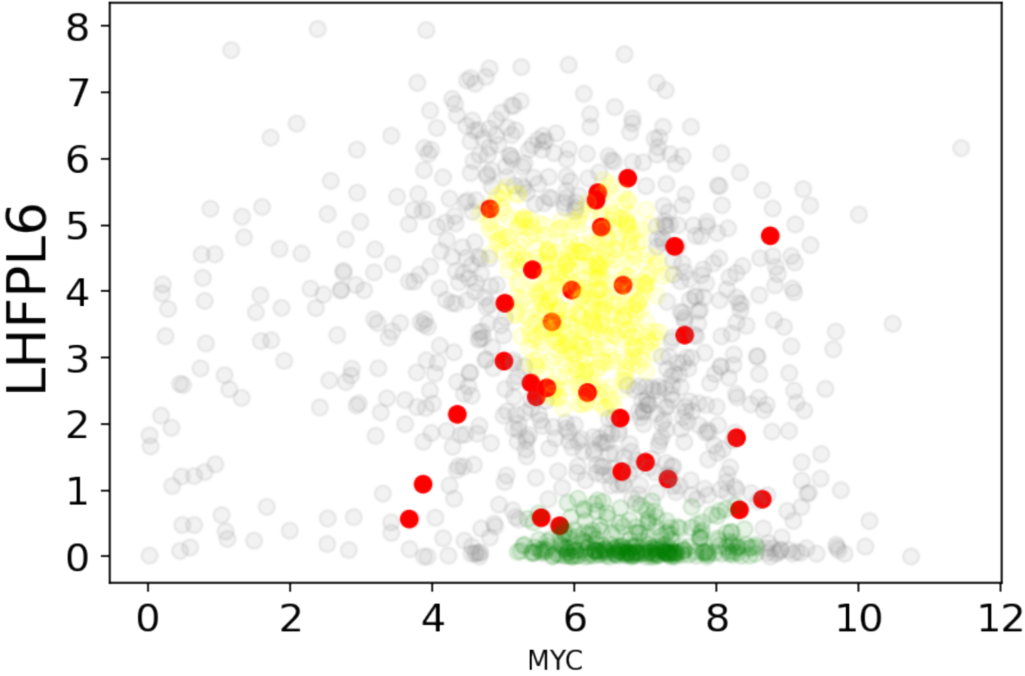

LHFPL6
MYC
0
1
2
3
4
5
6
7
8
0
2
4
6
8
10
12

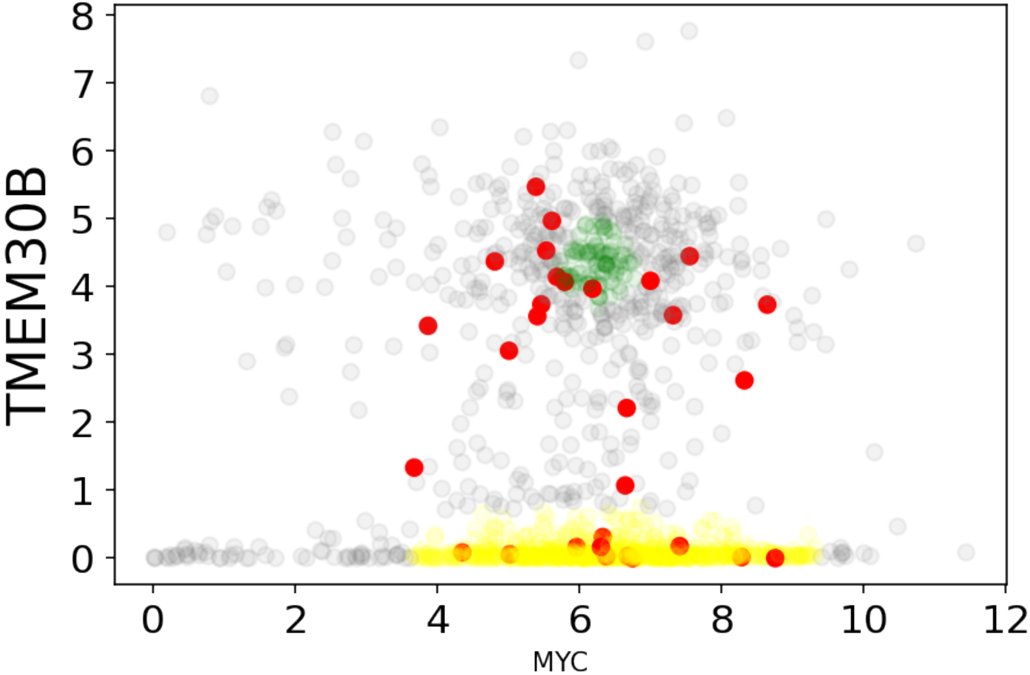
TMEM30B
MYC
0
1
2
3
4
5
6
7
8
0
2
4
6
8
10
12

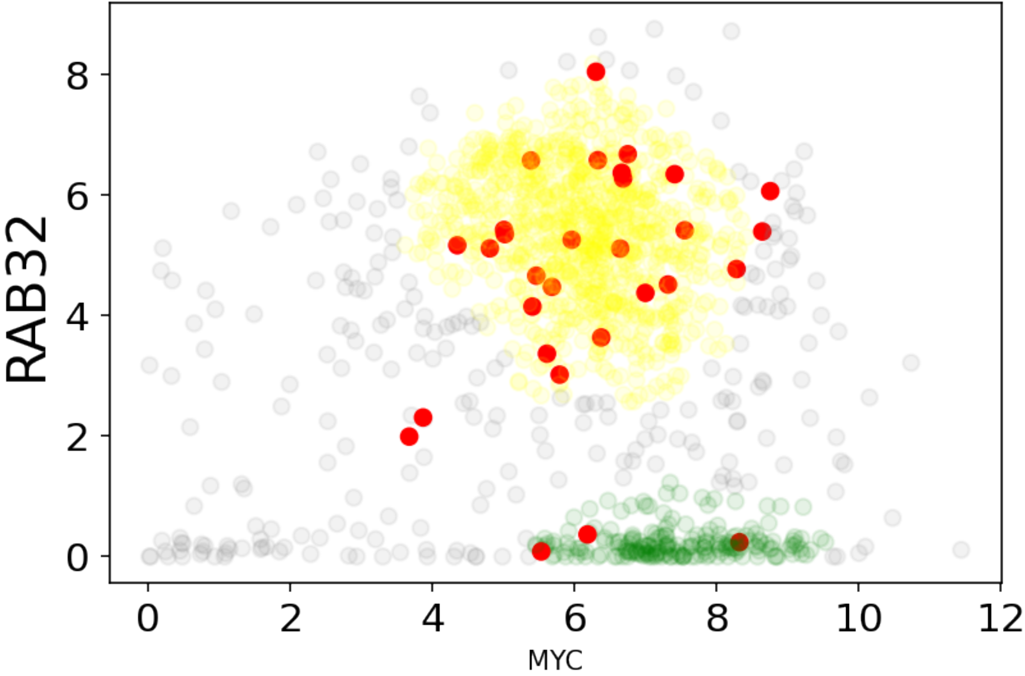
RAB32
8
6
4
2
0
0
2
4
6
8
10
12
MYC

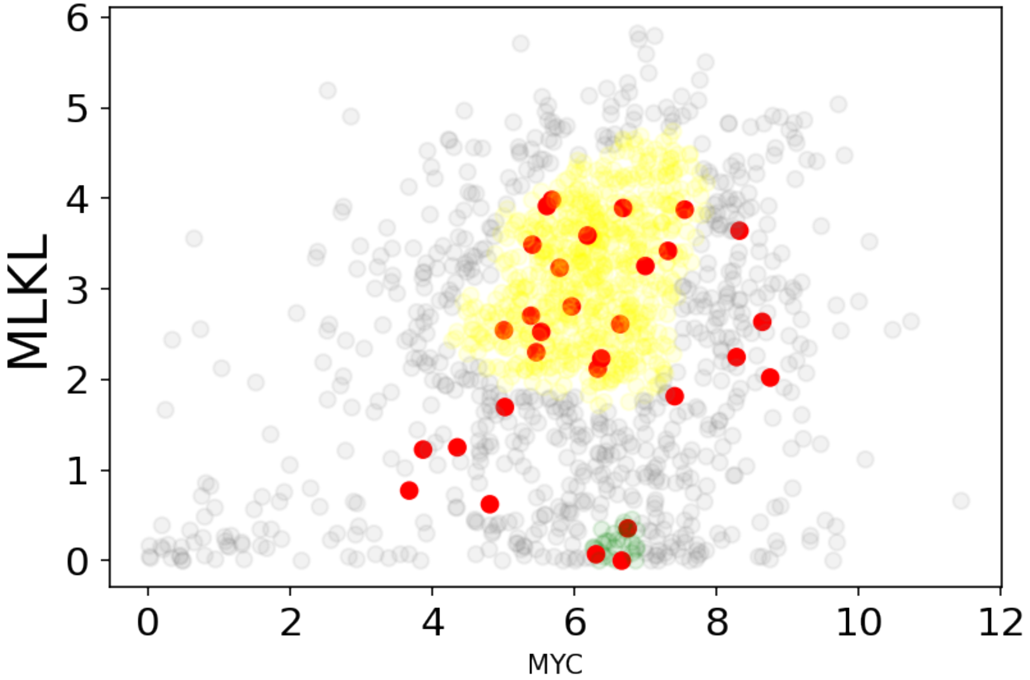

MLKL
MYC
0
1
2
3
4
5
6
0
2
4
6
8
10
12

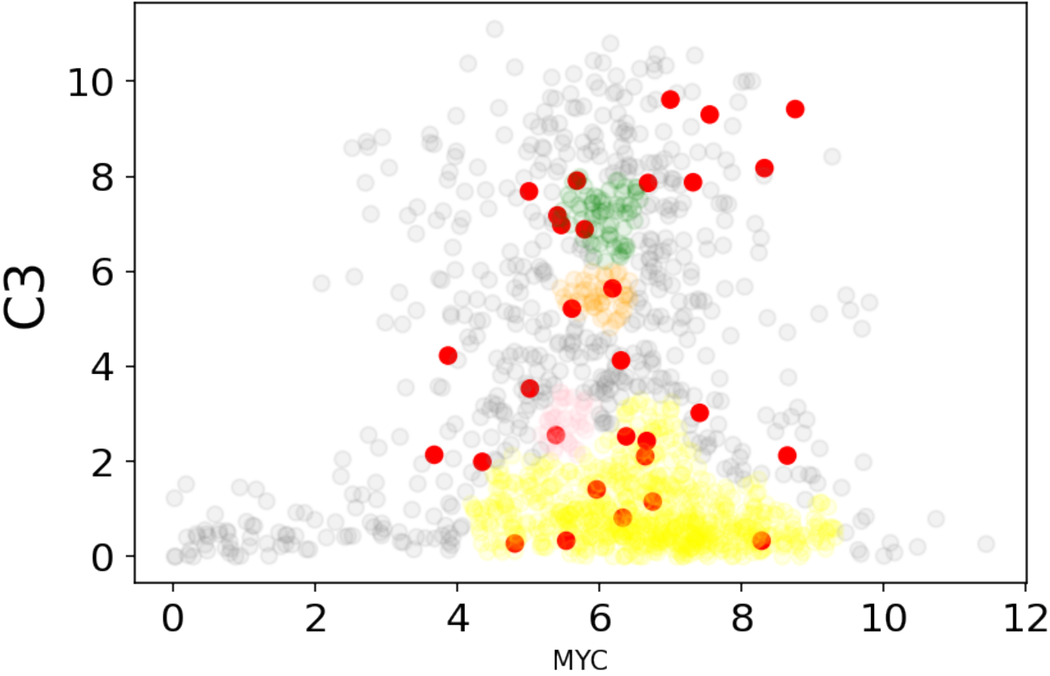

C3
10
8
6
4
2
0
0
2
4
6
8
10
12
MYC

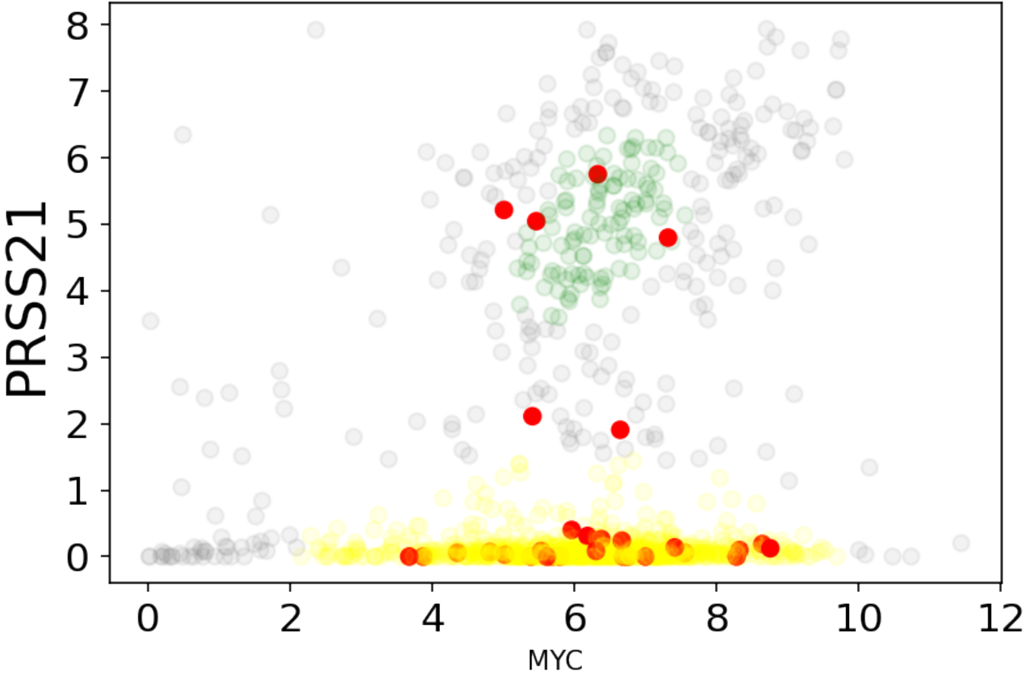

PRSS21
8
7
6
5
4
3
2
1
0
0
2
4
6
8
10
12
MYC

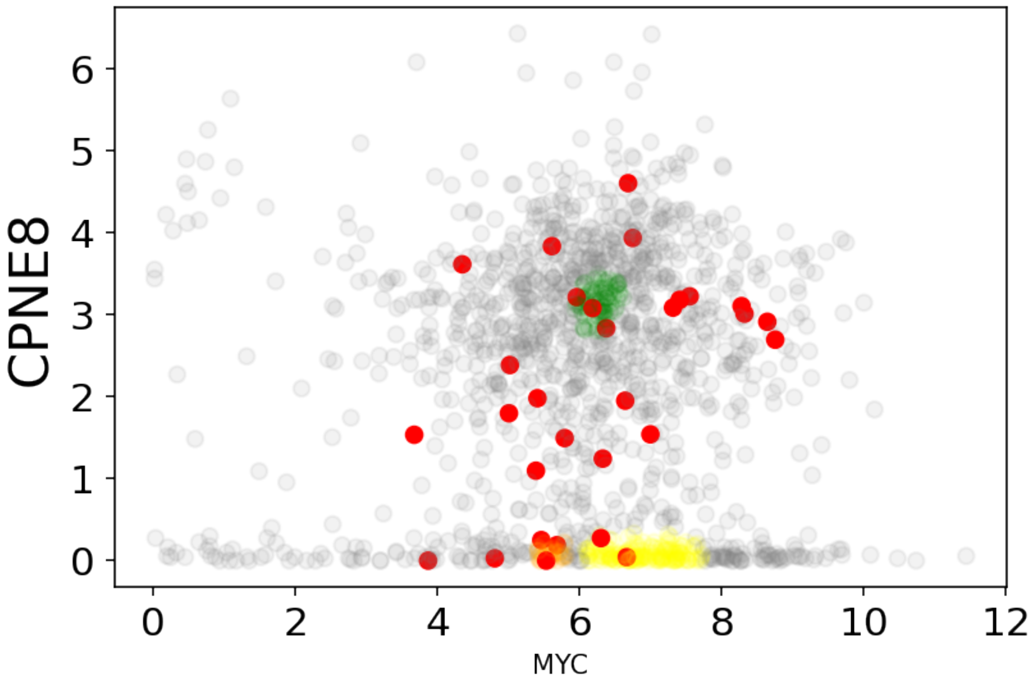

CPNE8
6
5
4
3
2
1
0
0
2
4
6
8
10
12
MYC

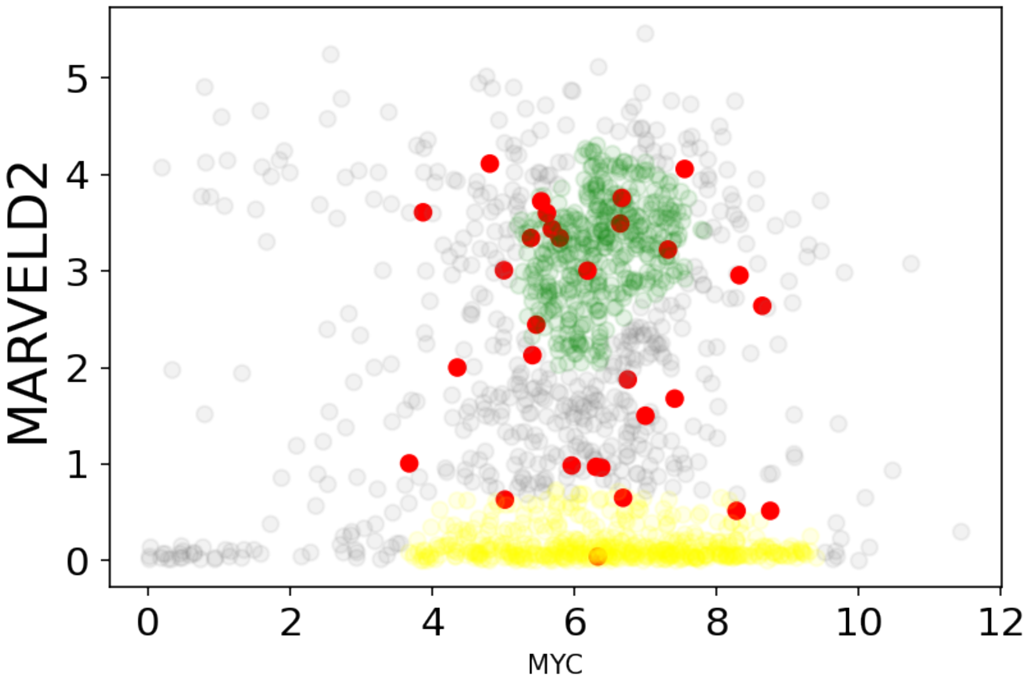

MARVELD2
5
4
3
2
1
0
0
2
4
6
8
10
12
MYC

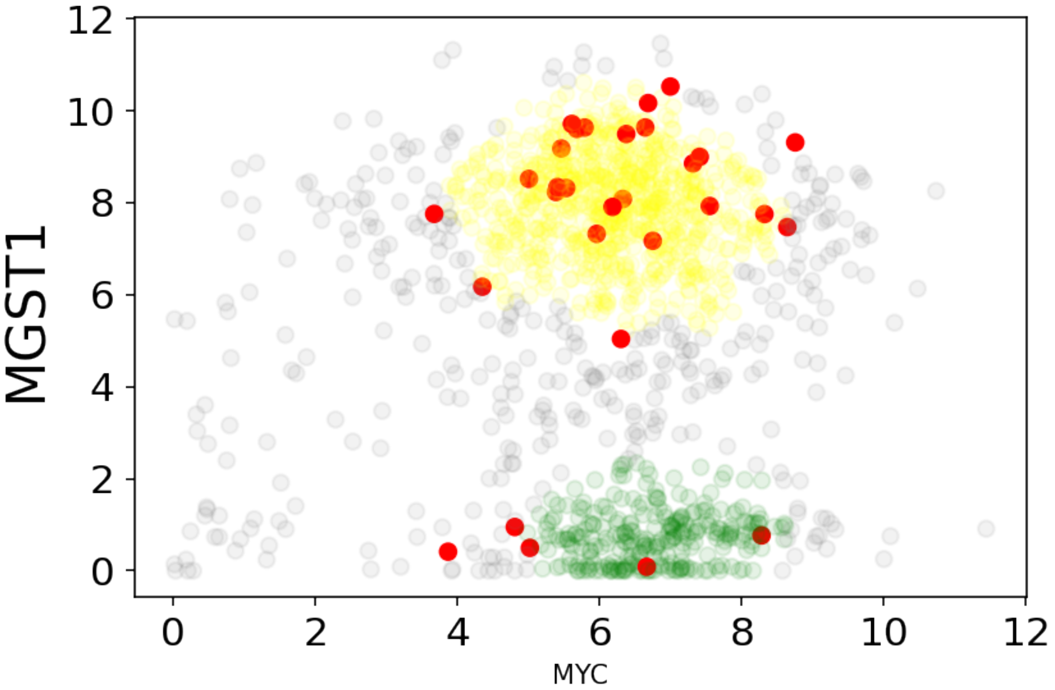
MGST1
MYC
0
2
4
6
8
10
12

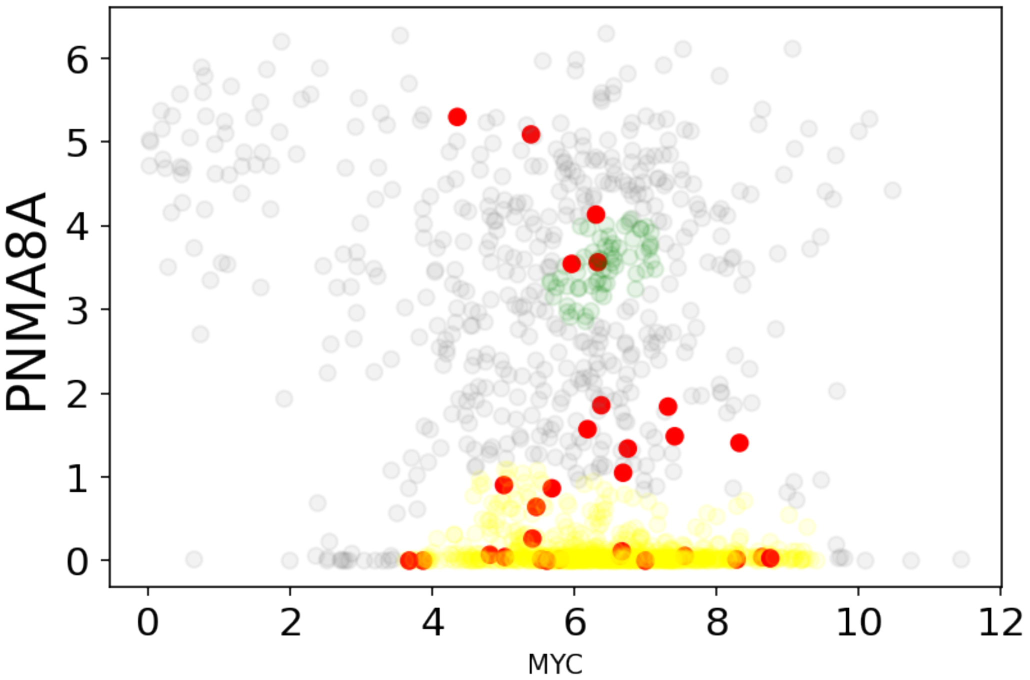
PNMA8A
MYC
0
1
2
3
4
5
6
0
2
4
6
8
10
12

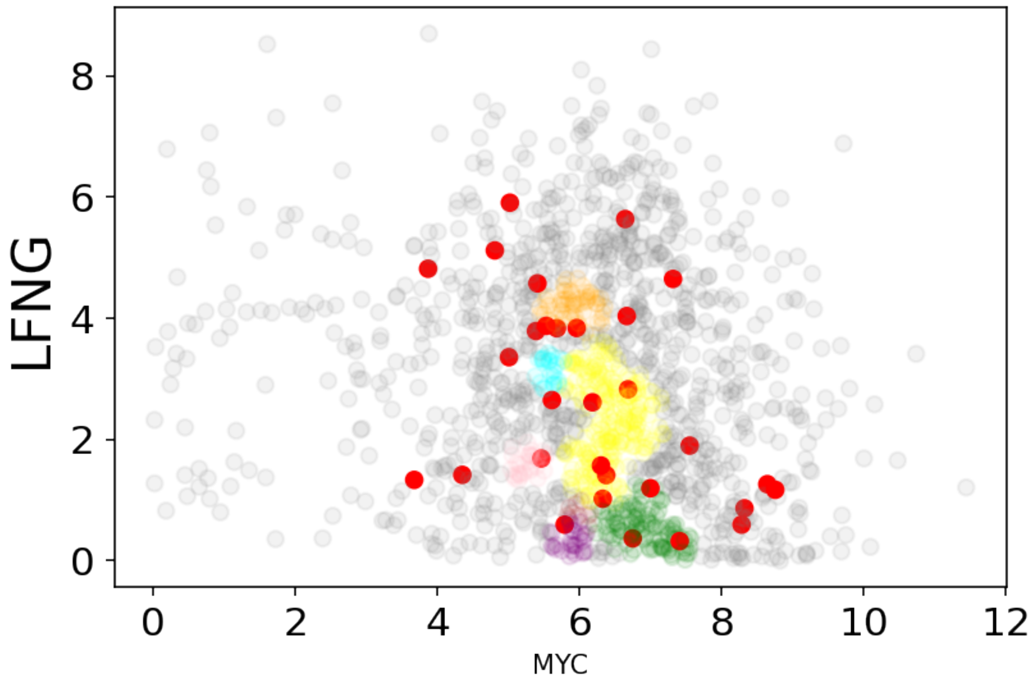

LFNG
8
6
4
2
0
0
2
4
6
8
10
12
MYC

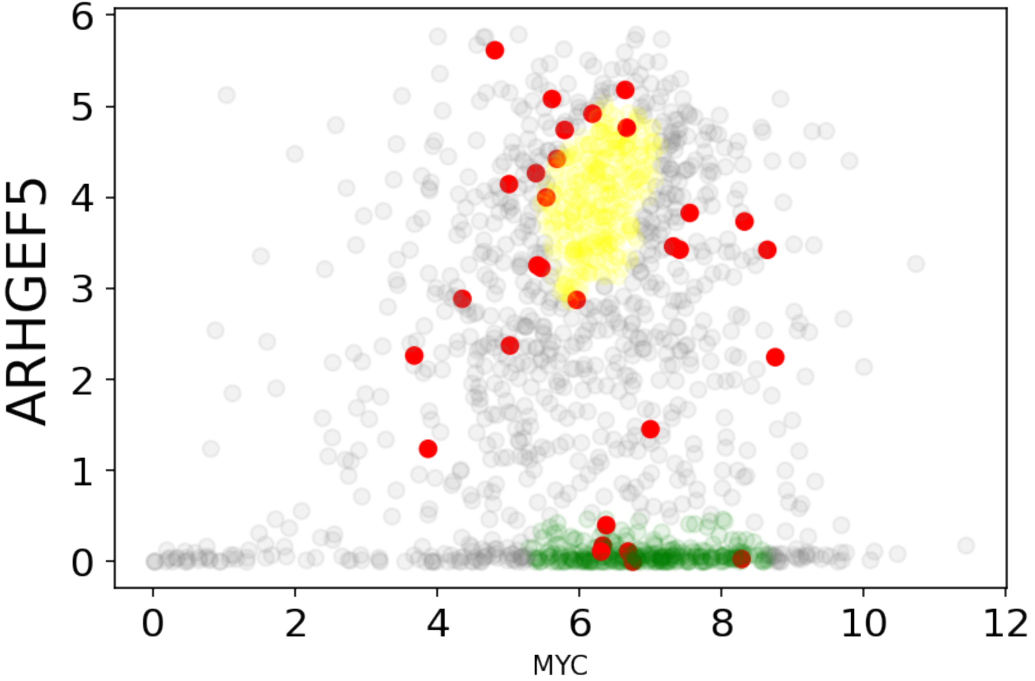

ARHGEF5
MYC
0
1
2
3
4
5
6
0
2
4
6
8
10
12

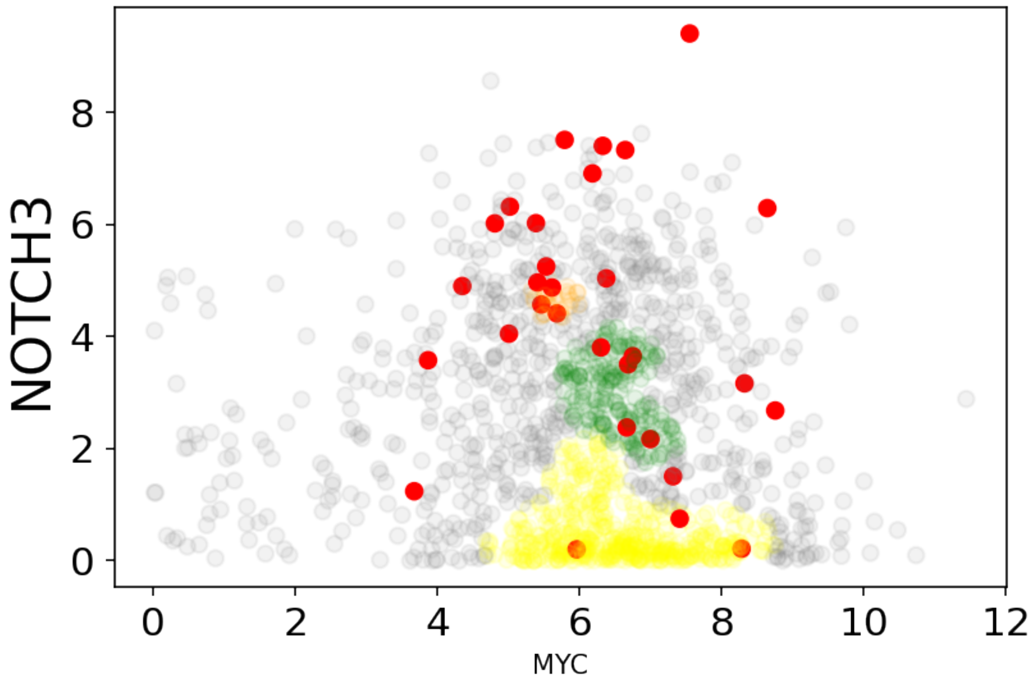

NOTCH3
8
6
4
2
0
0
2
4
6
8
10
12
MYC

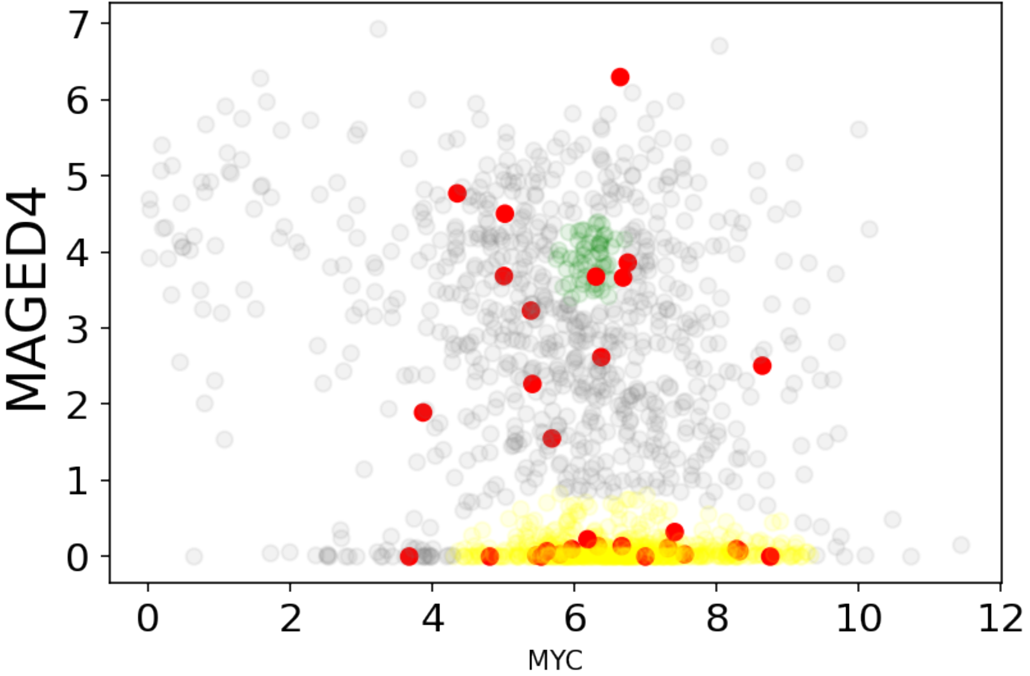

MAGED4
0
1
2
3
4
5
6
7
0
2
4
6
8
10
12
MYC

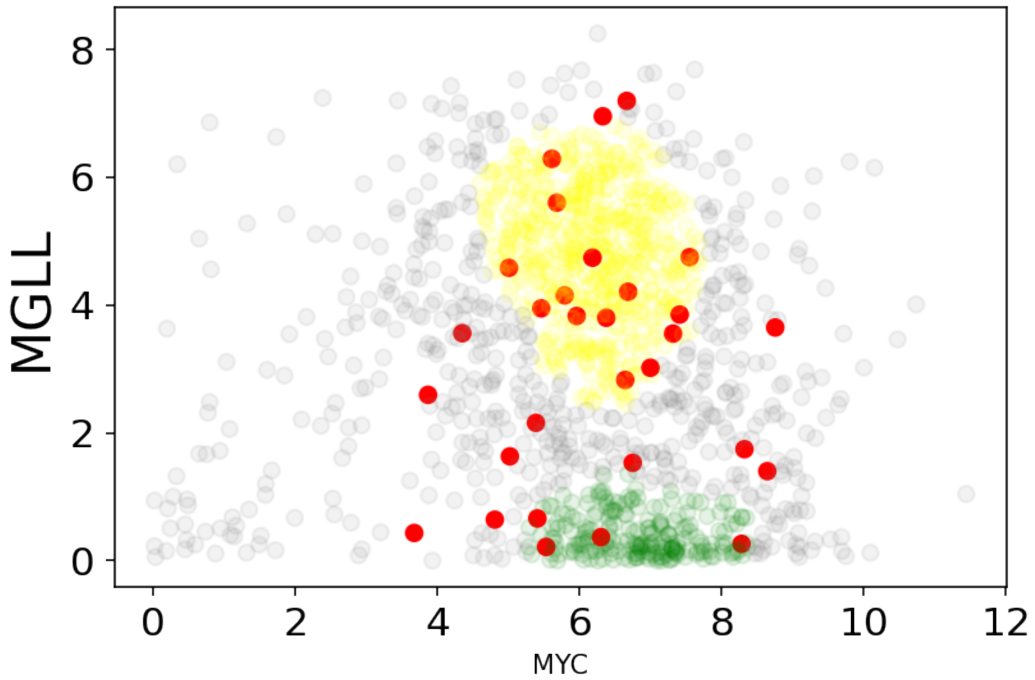

MGLL
8
6
4
2
0
0
2
4
6
8
10
12
MYC

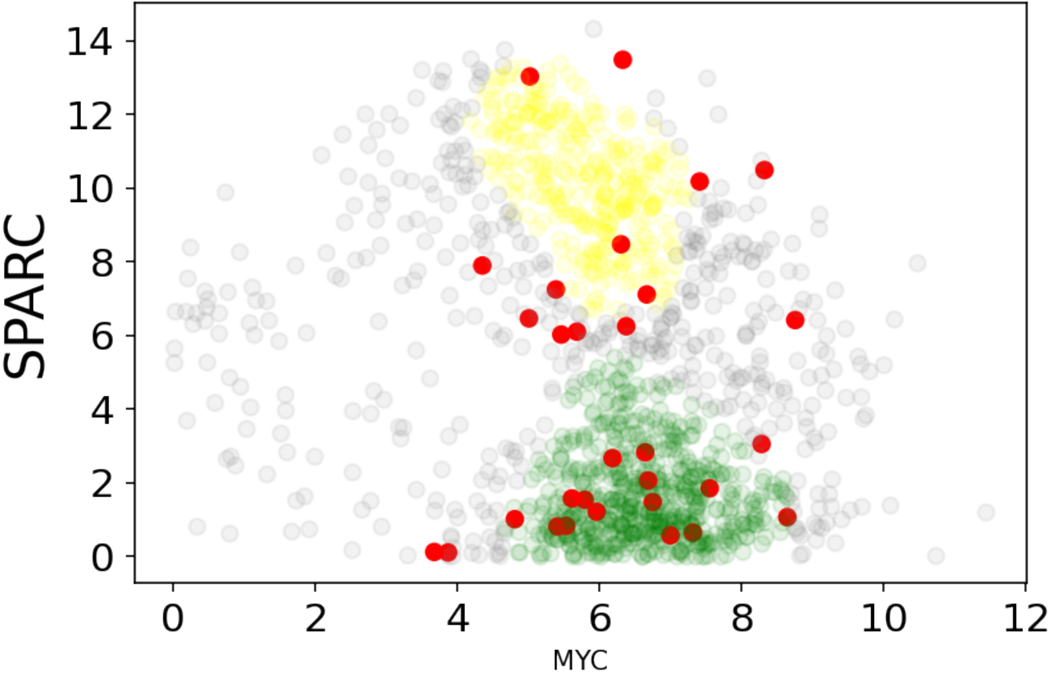

SPARC
MYC
0
2
4
6
8
10
12
14

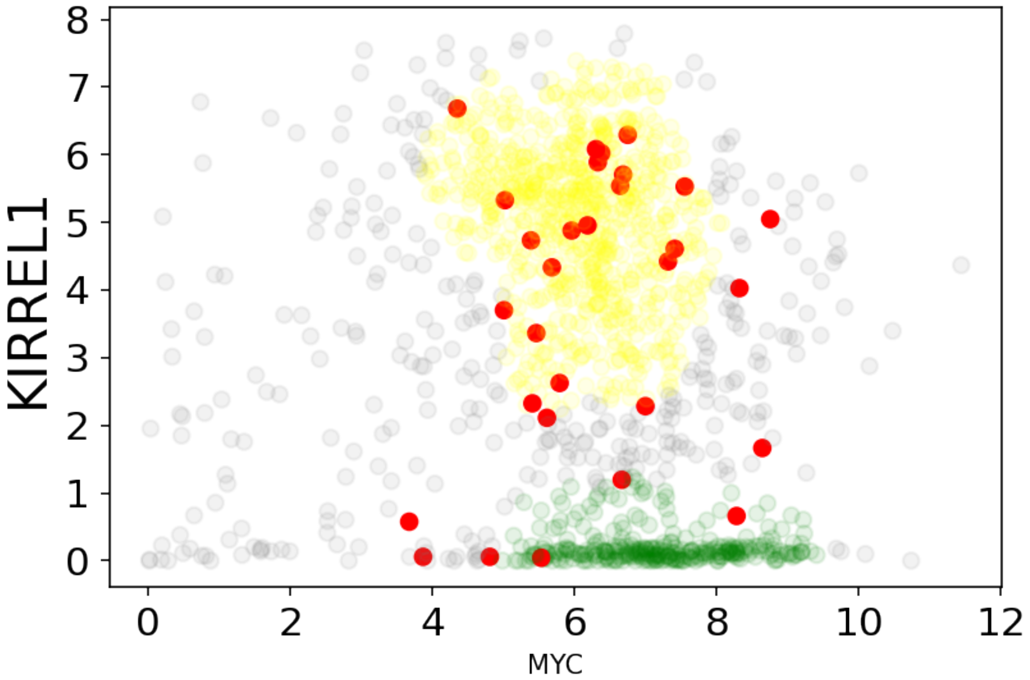

KIRREL1
MYC
0
1
2
3
4
5
6
7
8
0
2
4
6
8
10
12

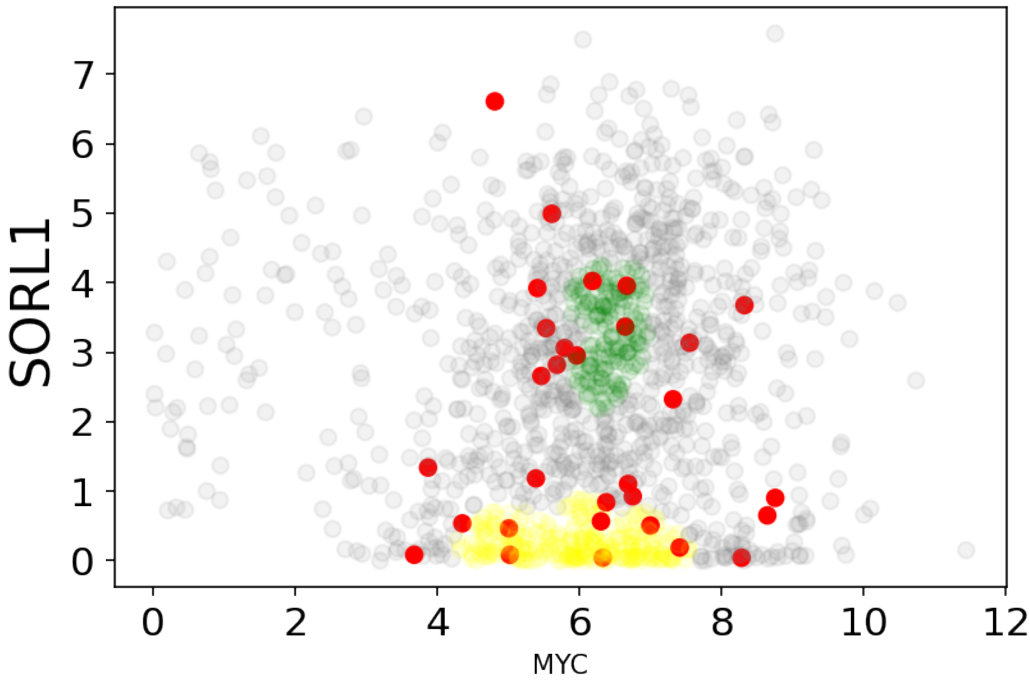

SORL1
0
1
2
3
4
5
6
7
0
2
4
6
8
10
12
MYC

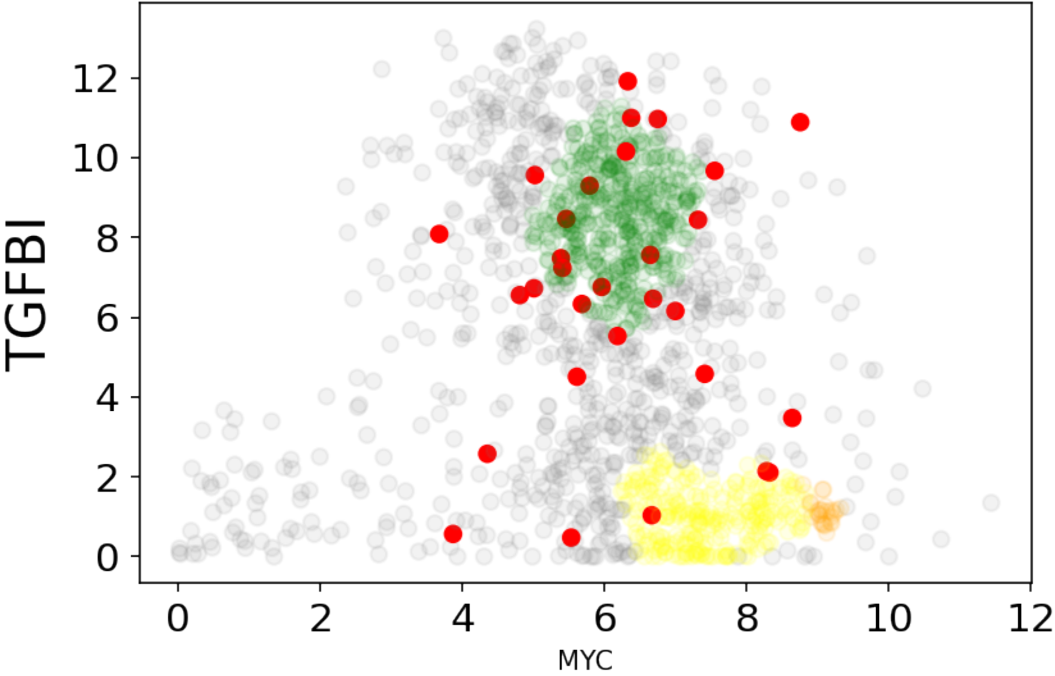

TGFBI
12
10
8
6
4
2
0
0
2
4
6
8
10
12
MYC

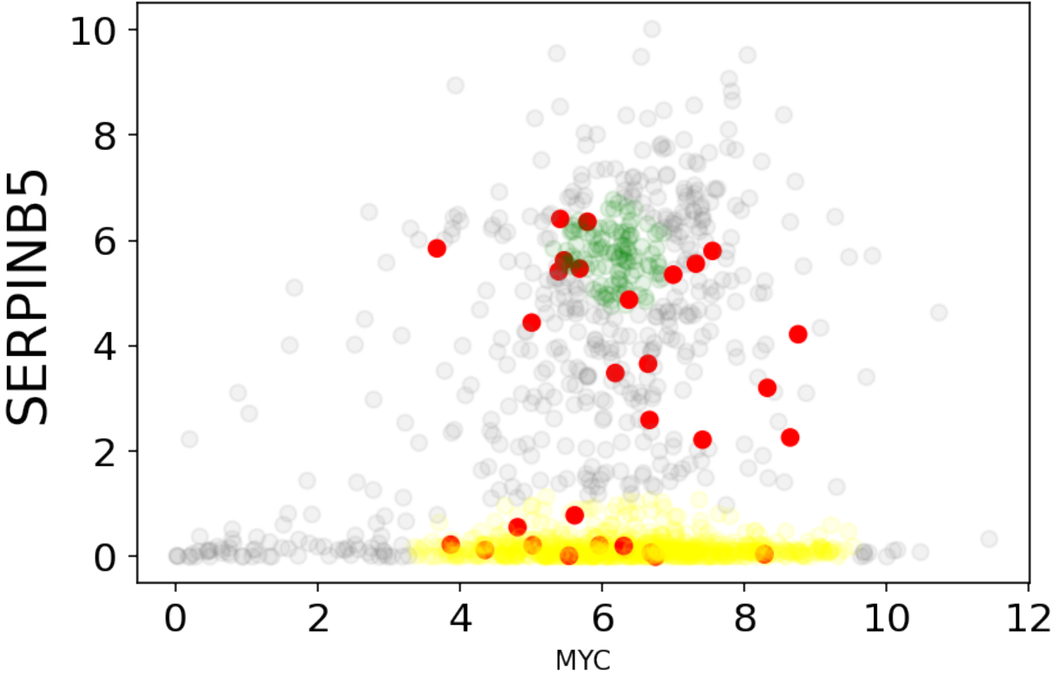
SERPINB5
MYC
0
2
4
6
8
10
12

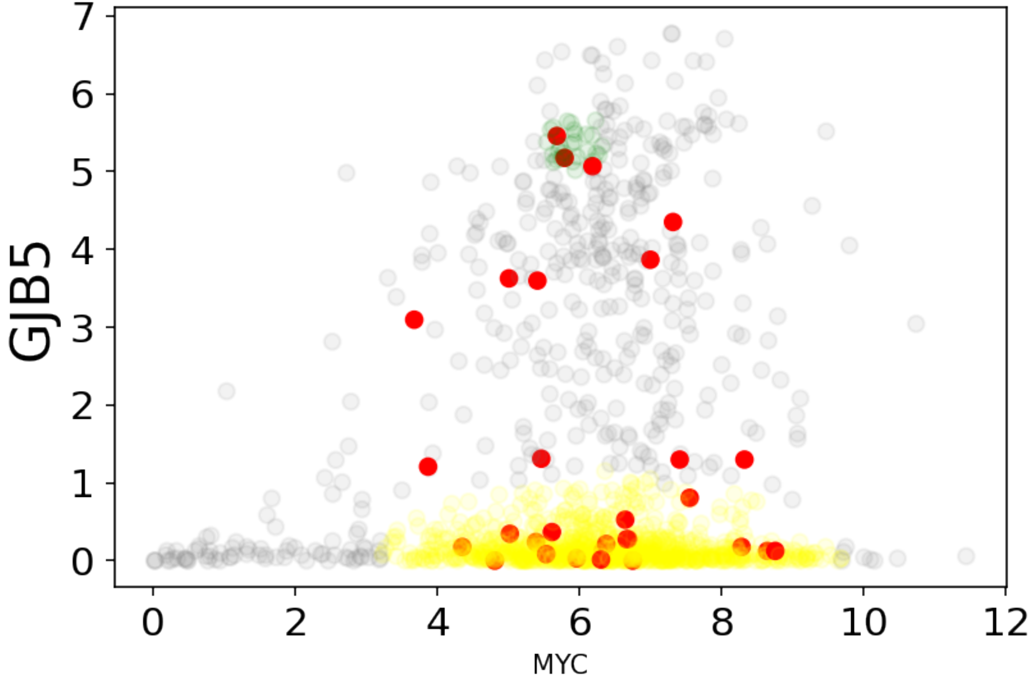

GJB5
MYC
0
1
2
3
4
5
6
7
0
2
4
6
8
10
12

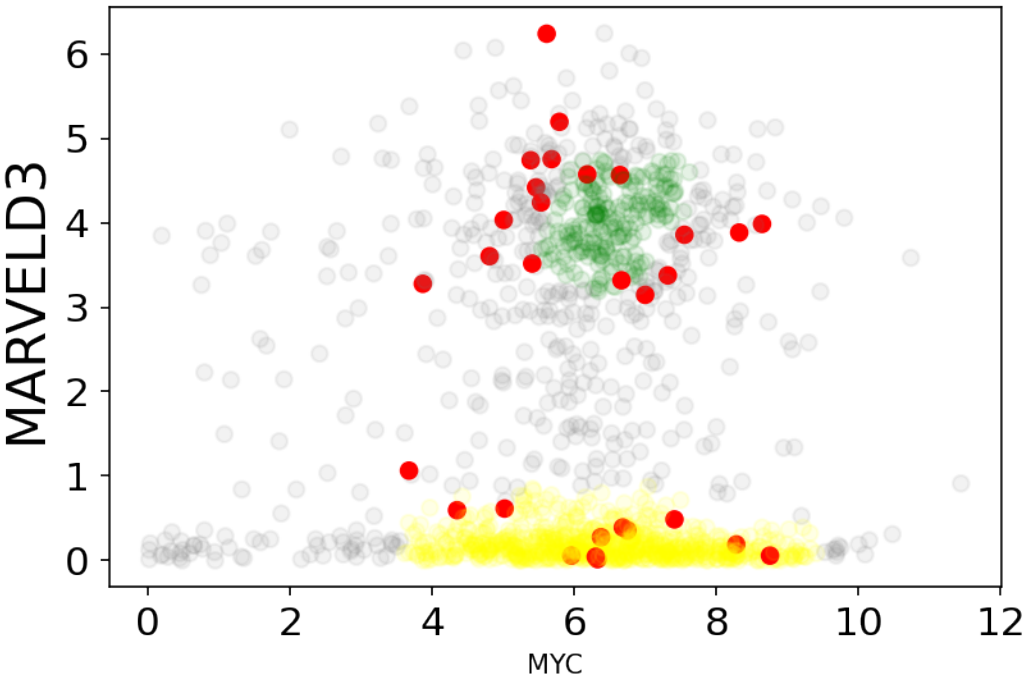

MARVELD3
0
1
2
3
4
5
6
0
2
4
6
8
10
12
MYC

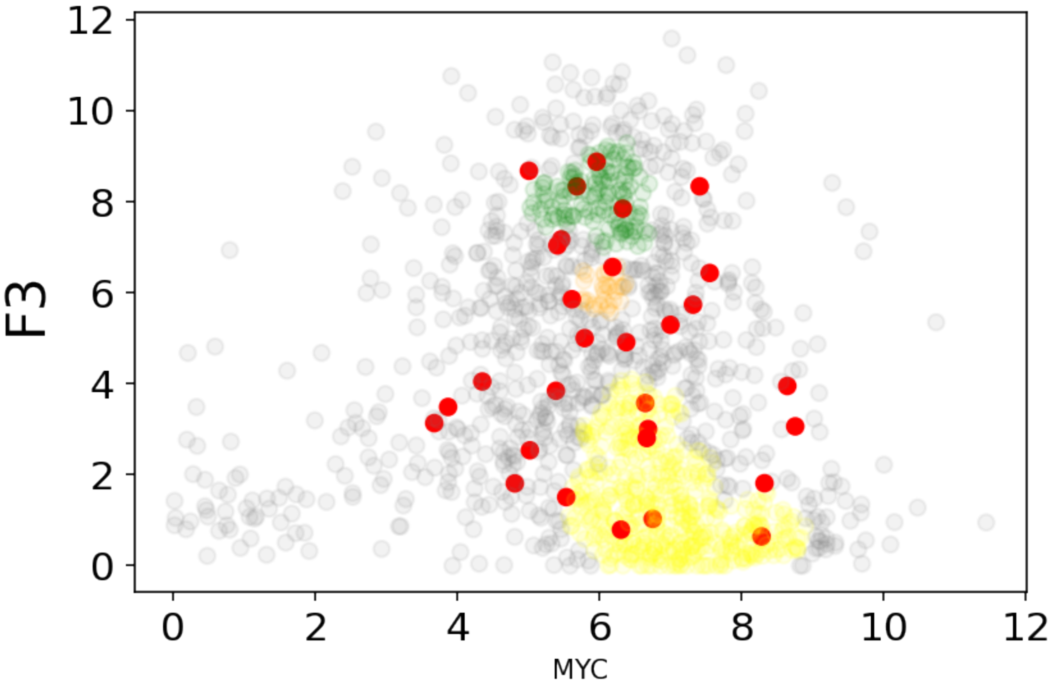

F3
MYC
0
2
4
6
8
10
12

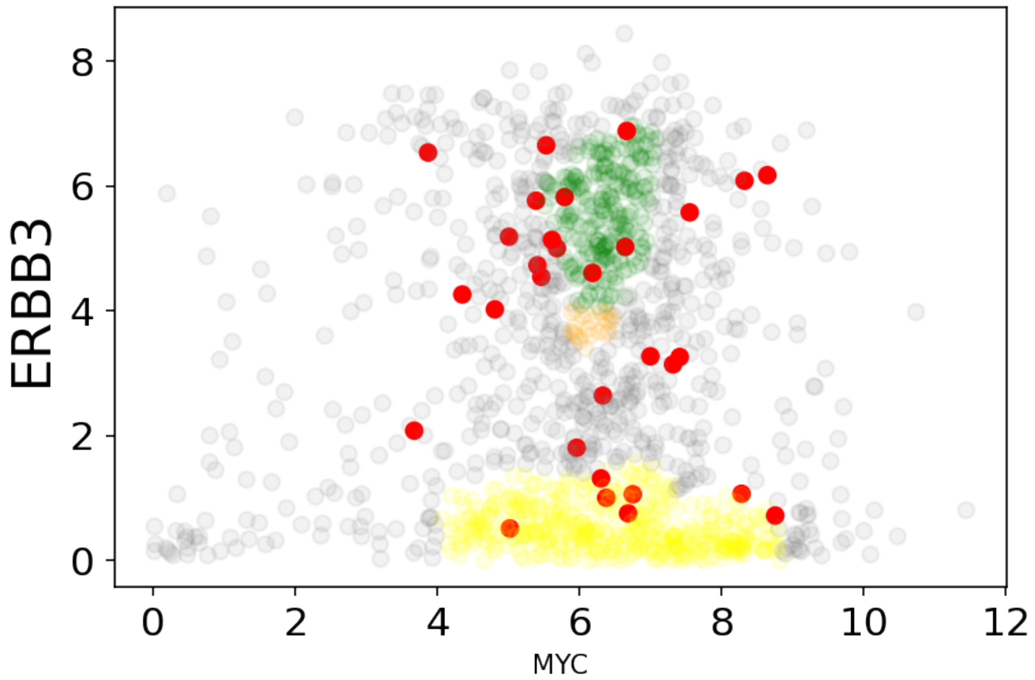
ERBB3
8
6
4
2
0
0
2
4
6
8
10
12
MYC

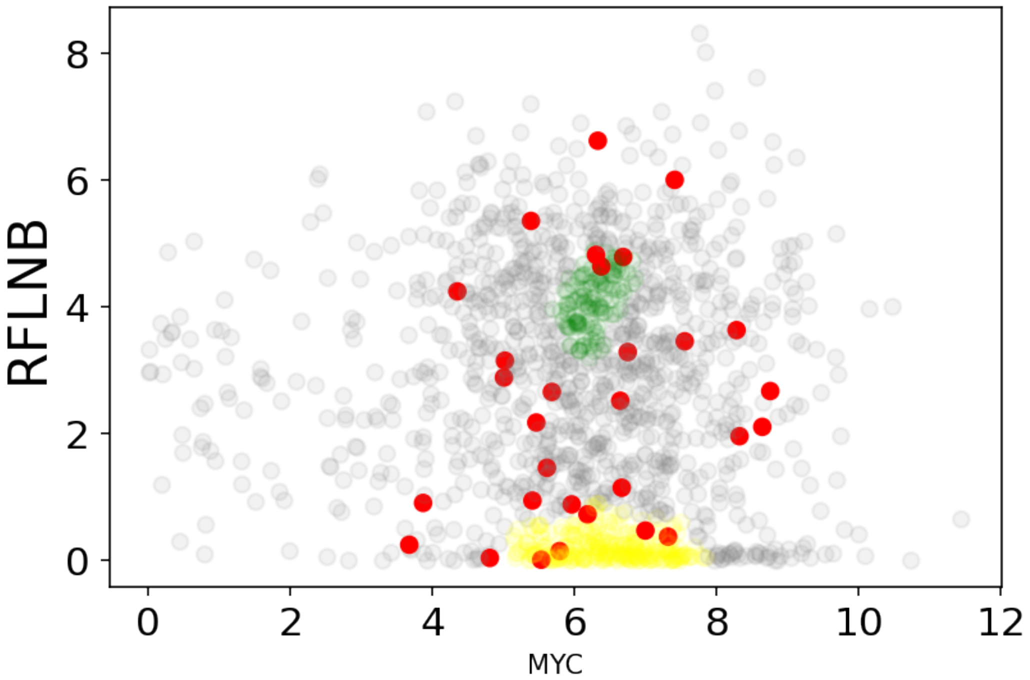
RFLNB
MYC
0
2
4
6
8
10
12

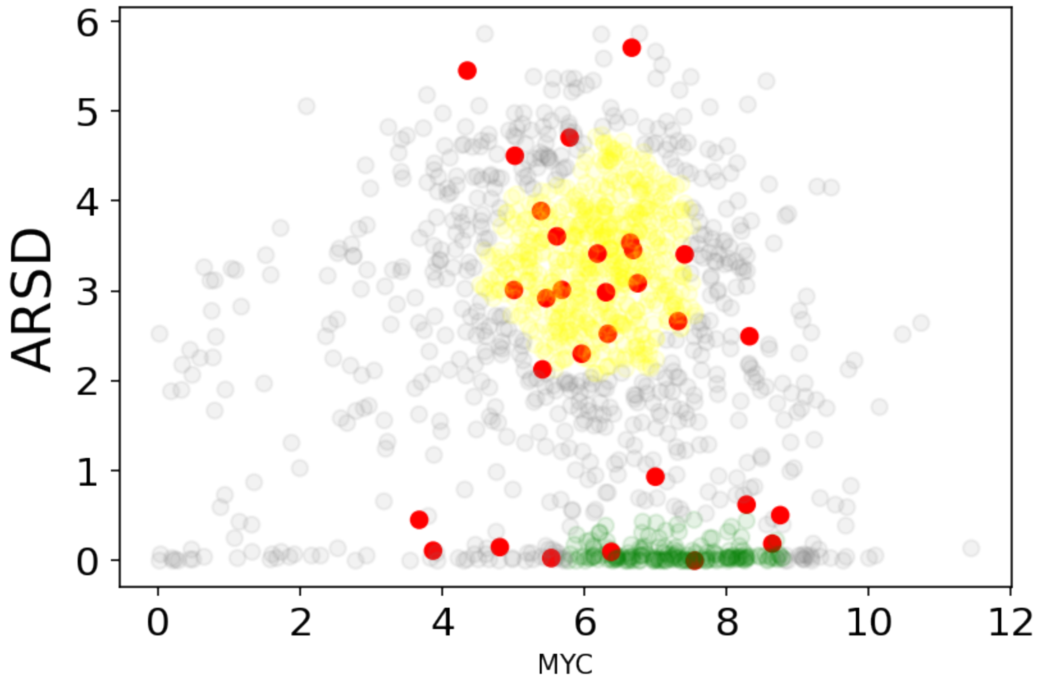

ARSD
6
5
4
3
2
1
0
0
2
4
6
8
10
12
MYC

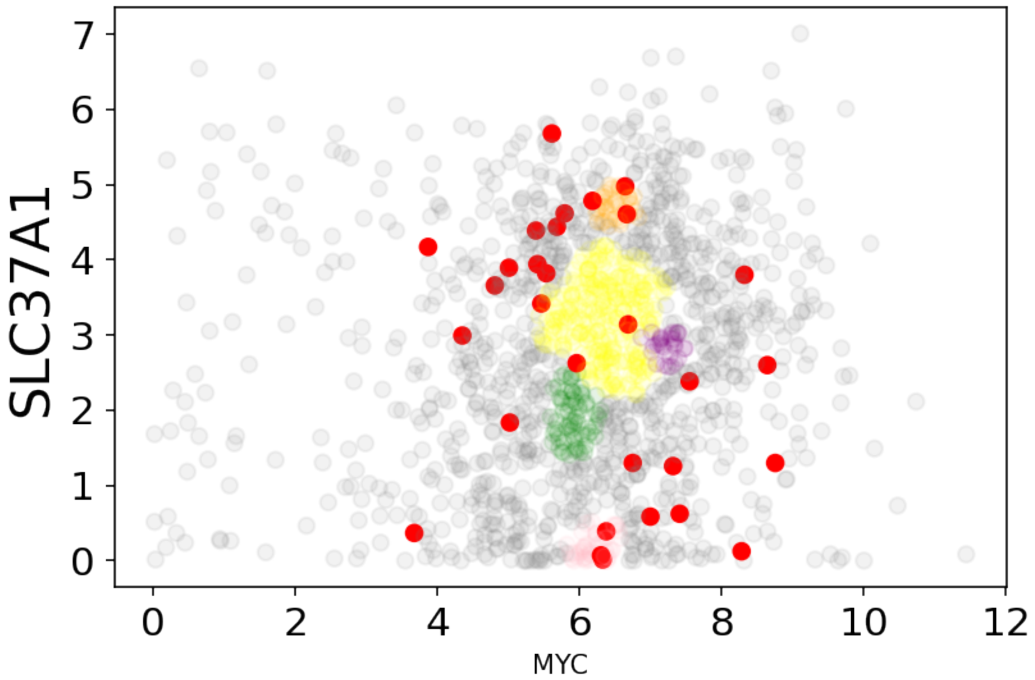
SLC37A1
7
6
5
4
3
2
1
0
0
2
4
6
8
10
12
MYC
