# Supplementary figures and images for: Decoding drug-responsive cell subpopulations in triple-negative breast cancer using single-cell multiomics
Source: iScience. 2026 Mar 21;29(5):115445. doi: 10.1016/j.isci.2026.115445 (PMC13138057; doi:10.1016/j.isci.2026.115445)

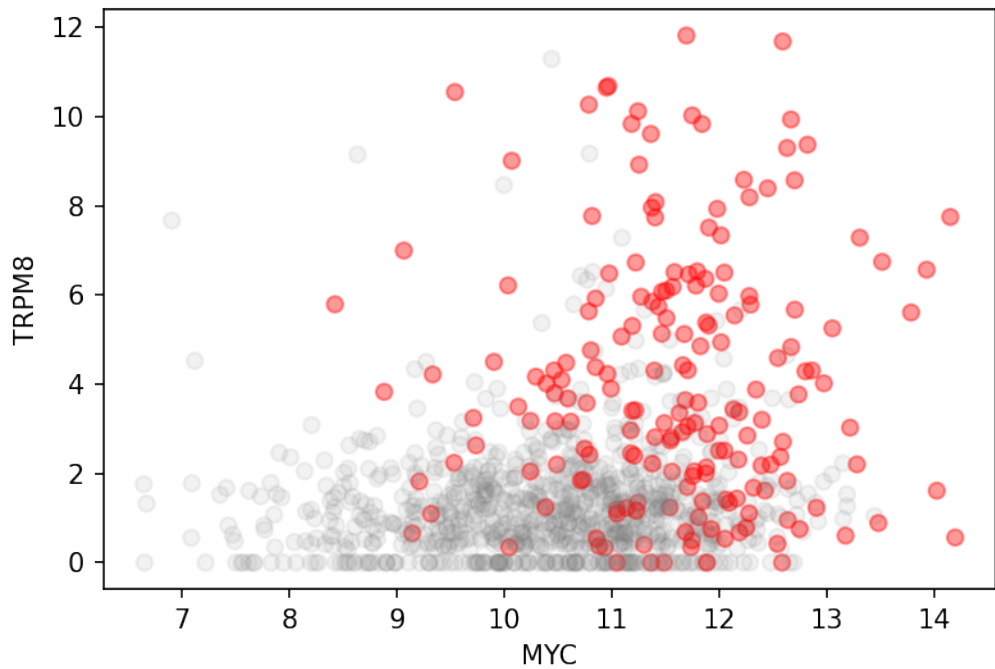

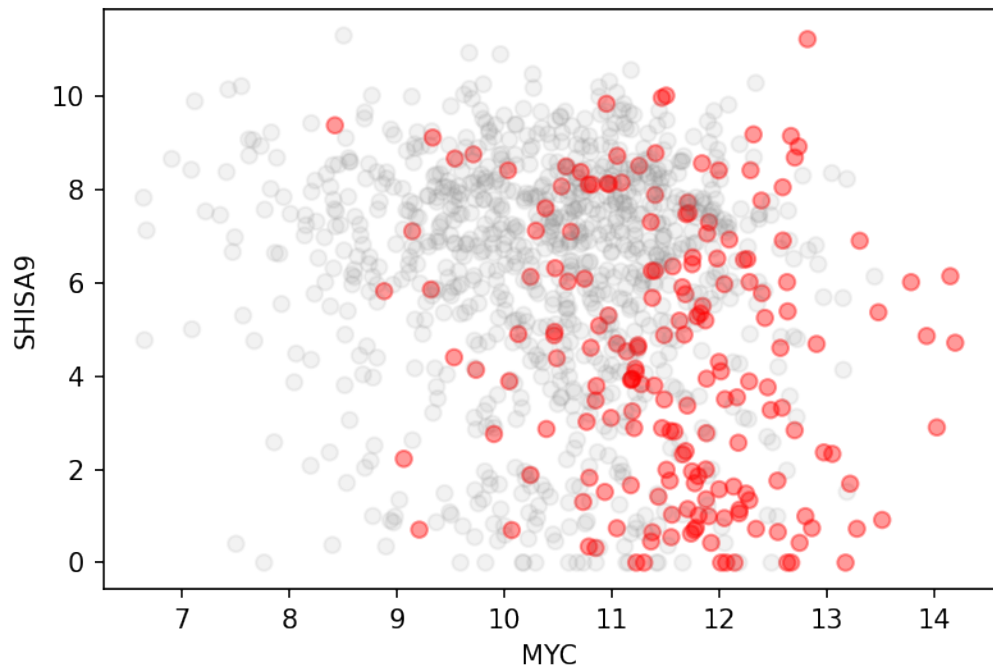

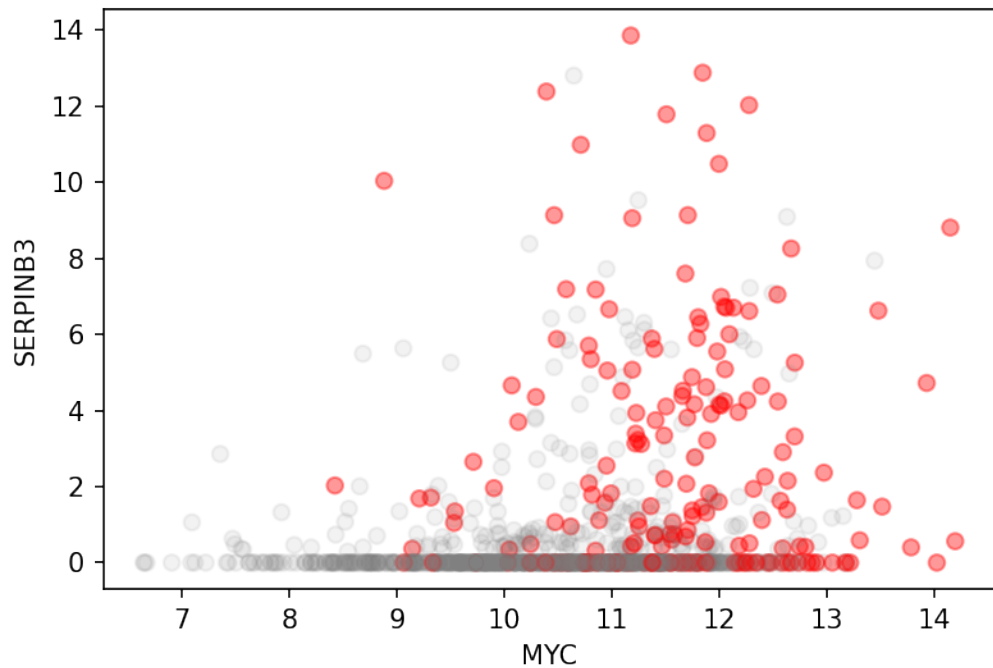

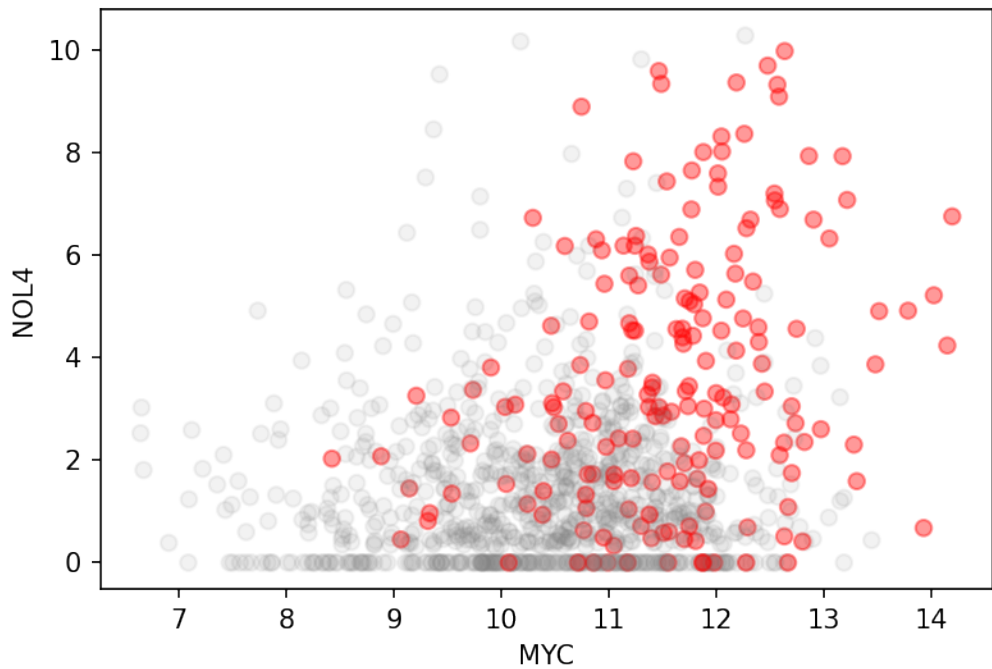

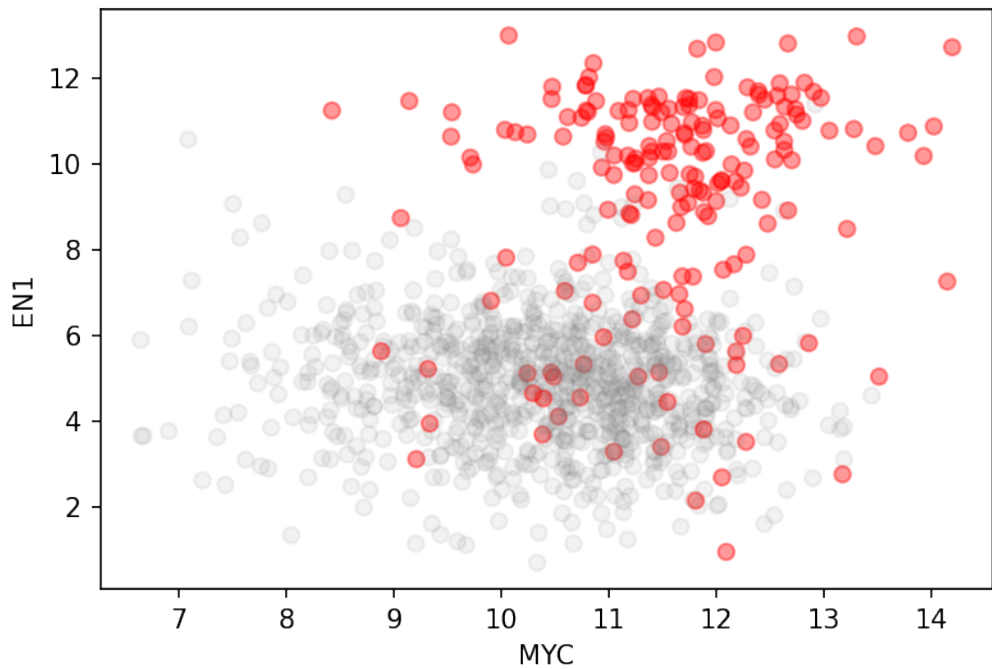

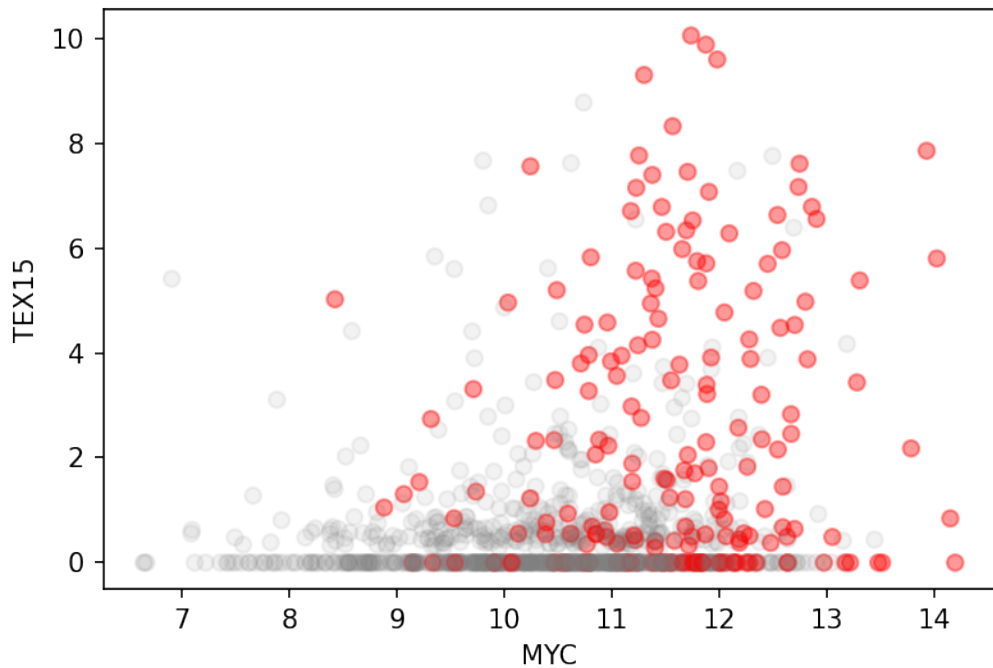

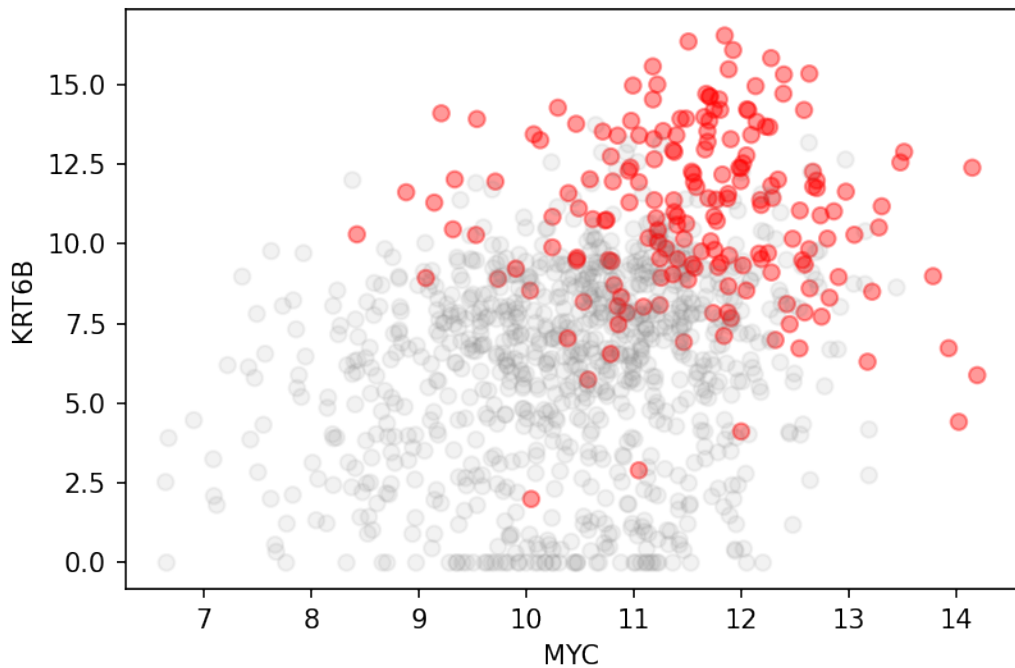

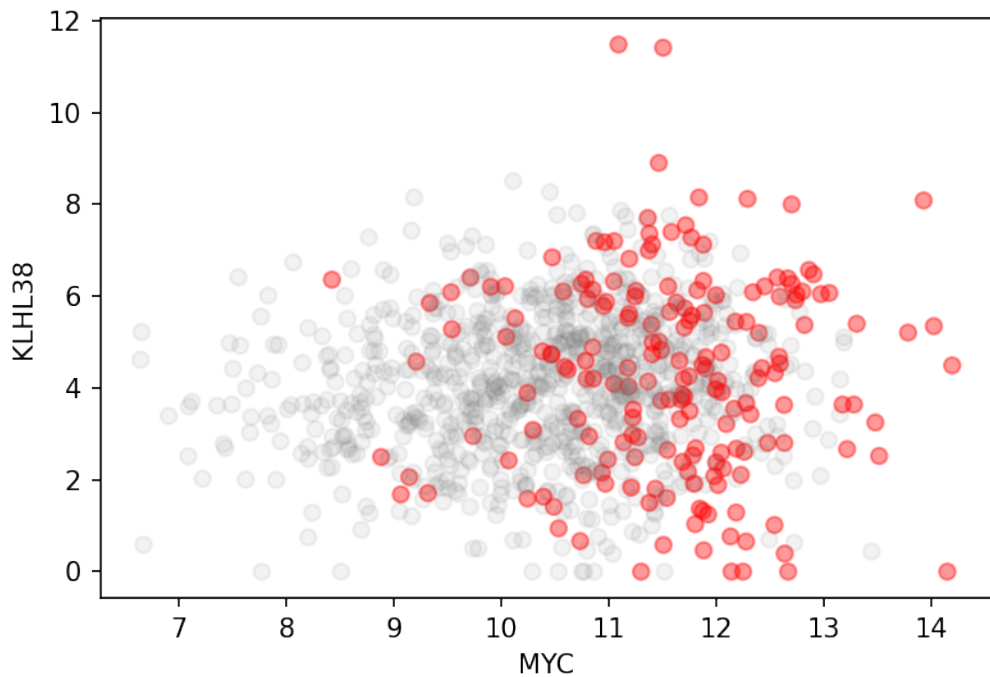

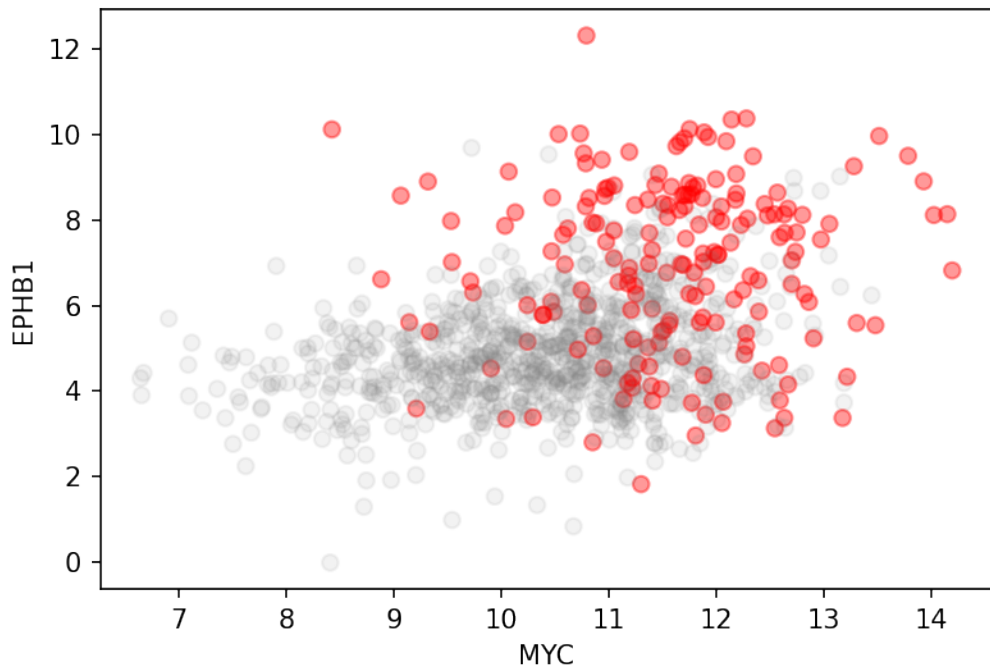

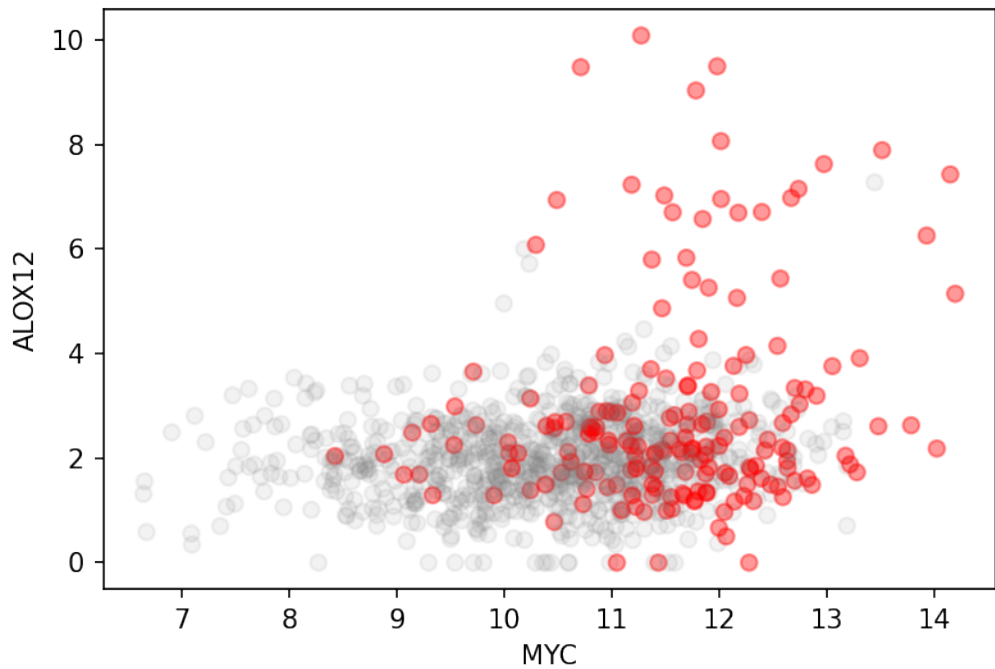

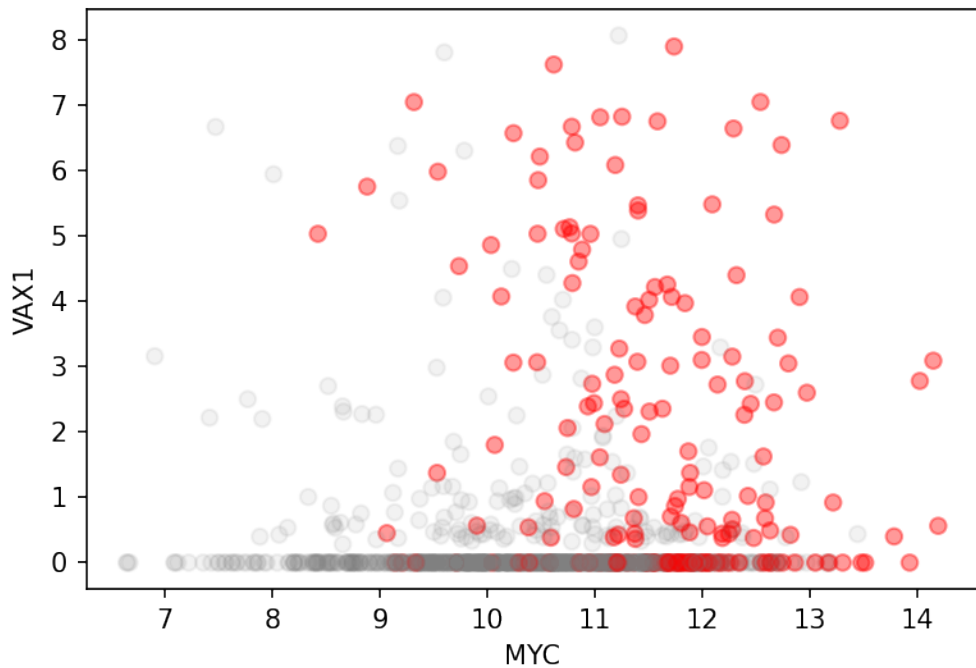

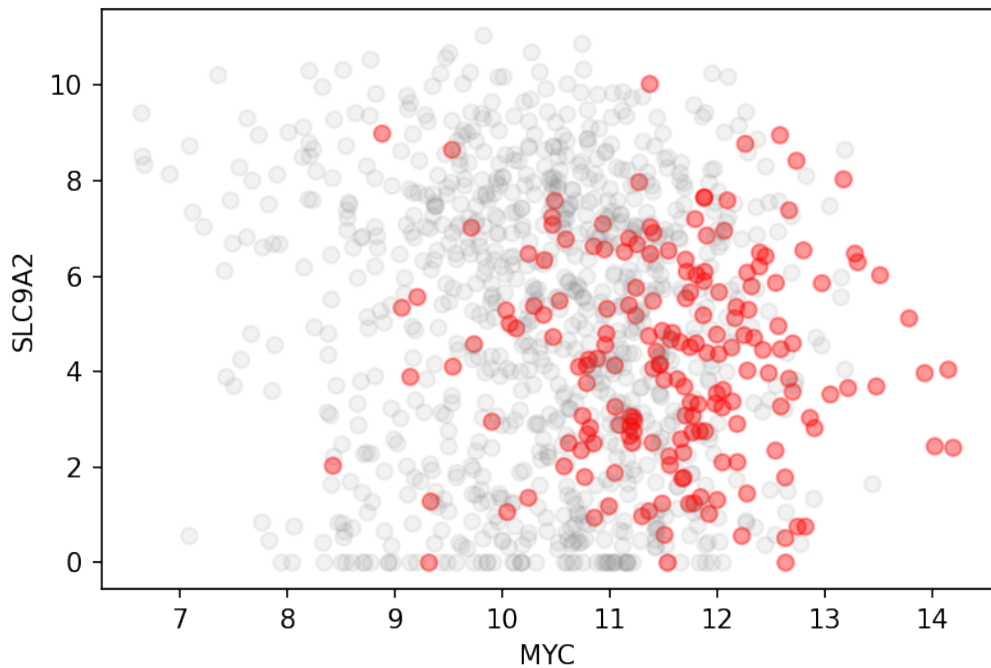

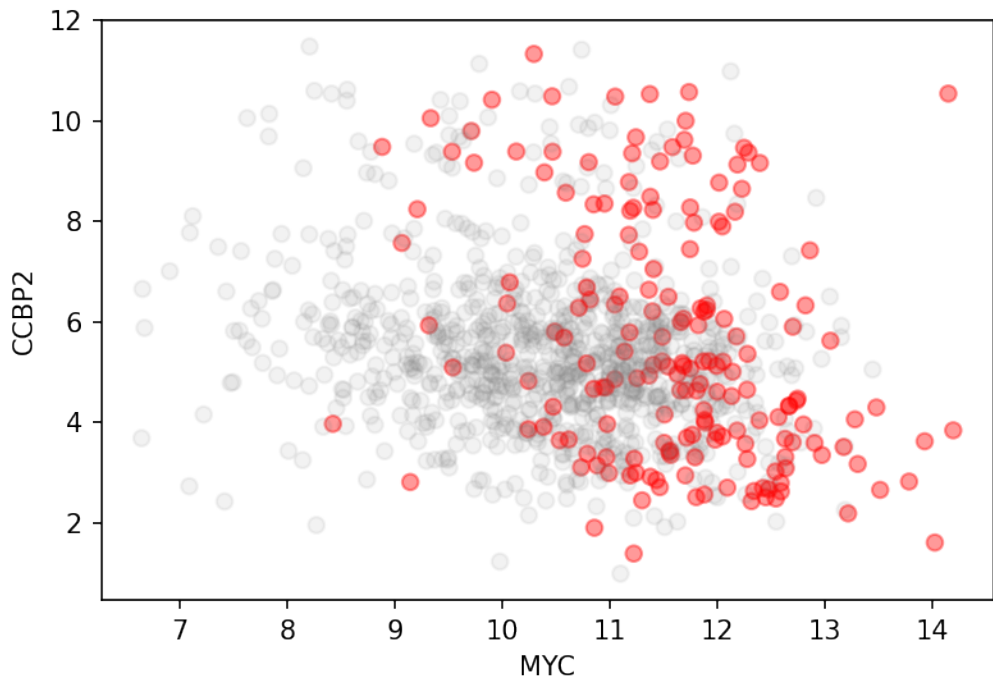

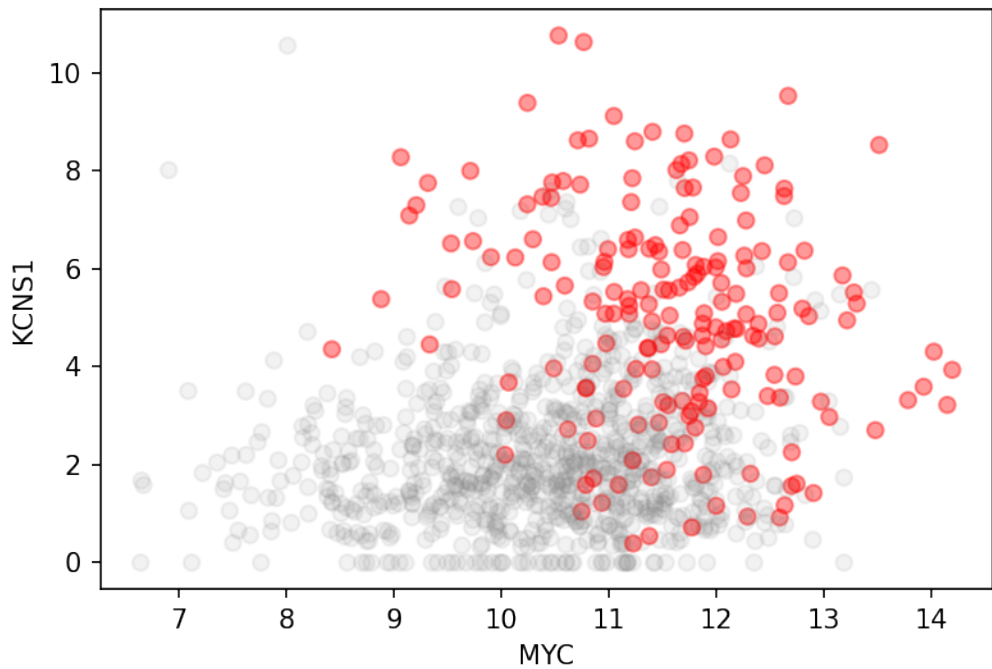

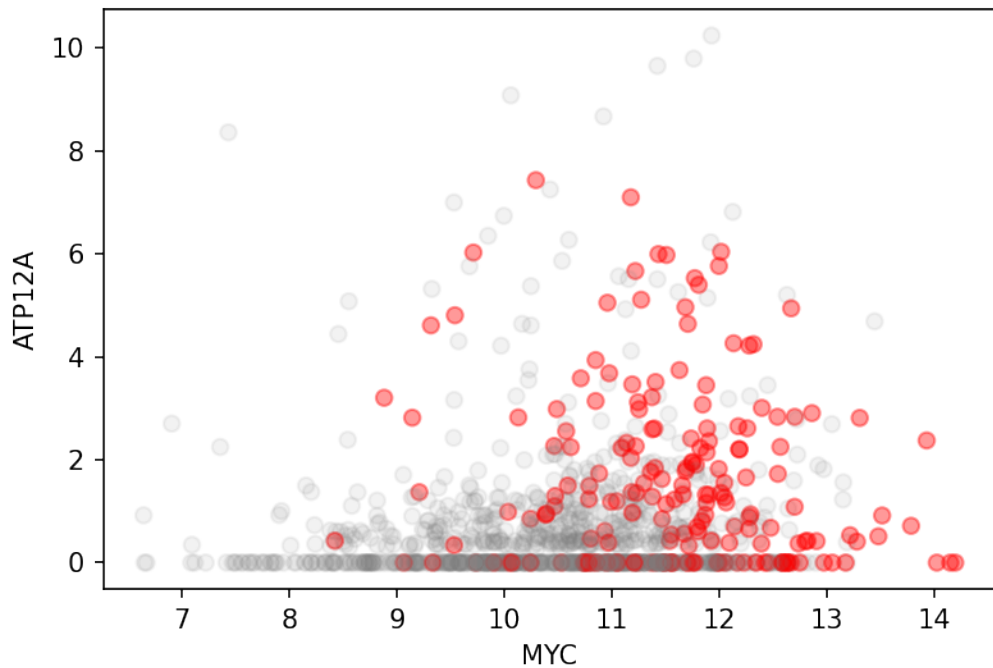

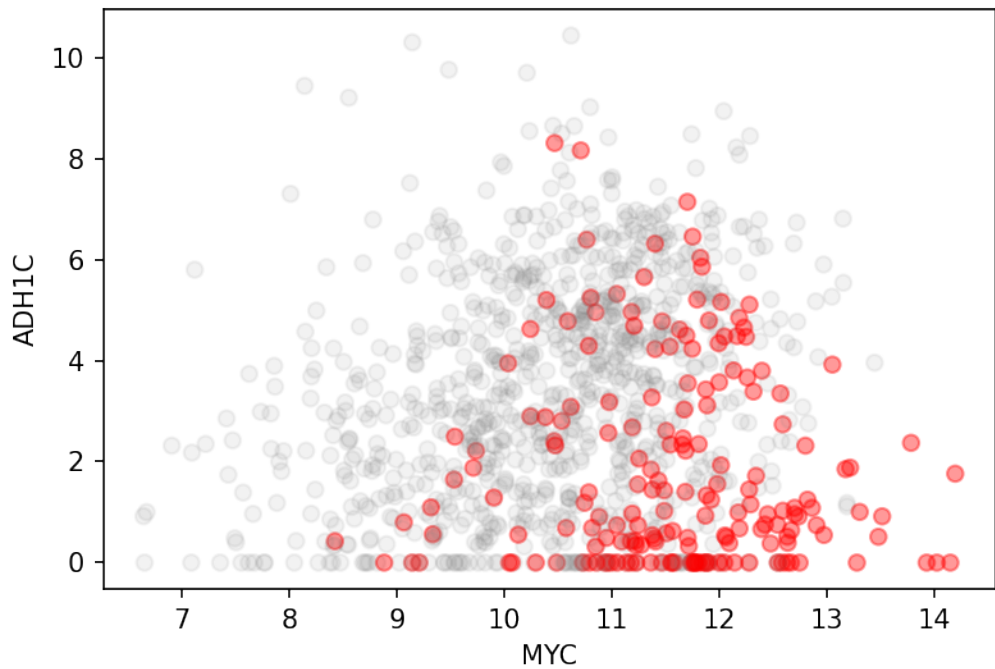

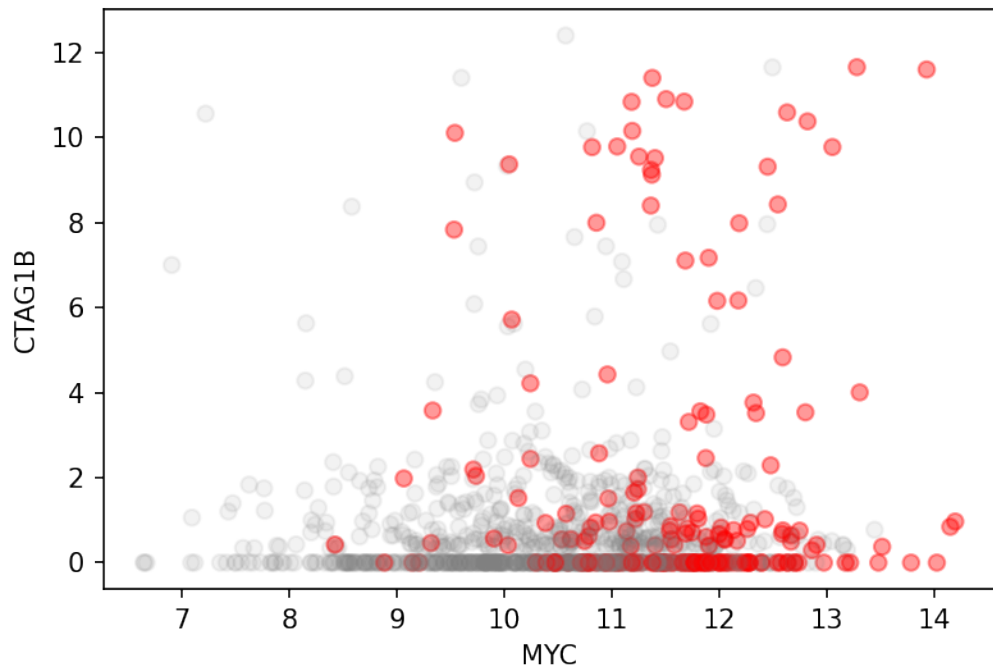

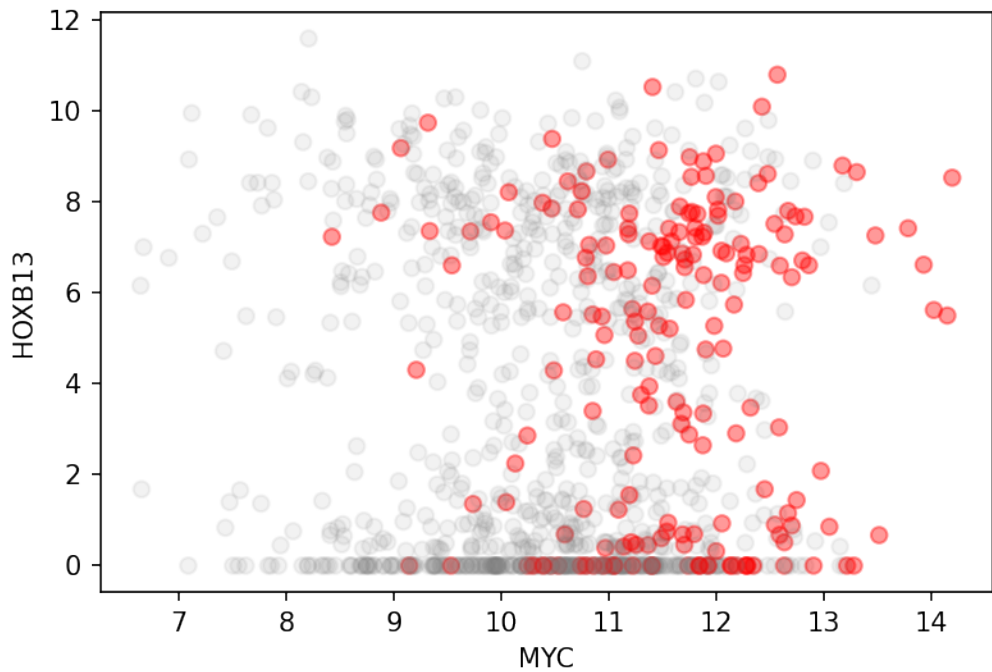

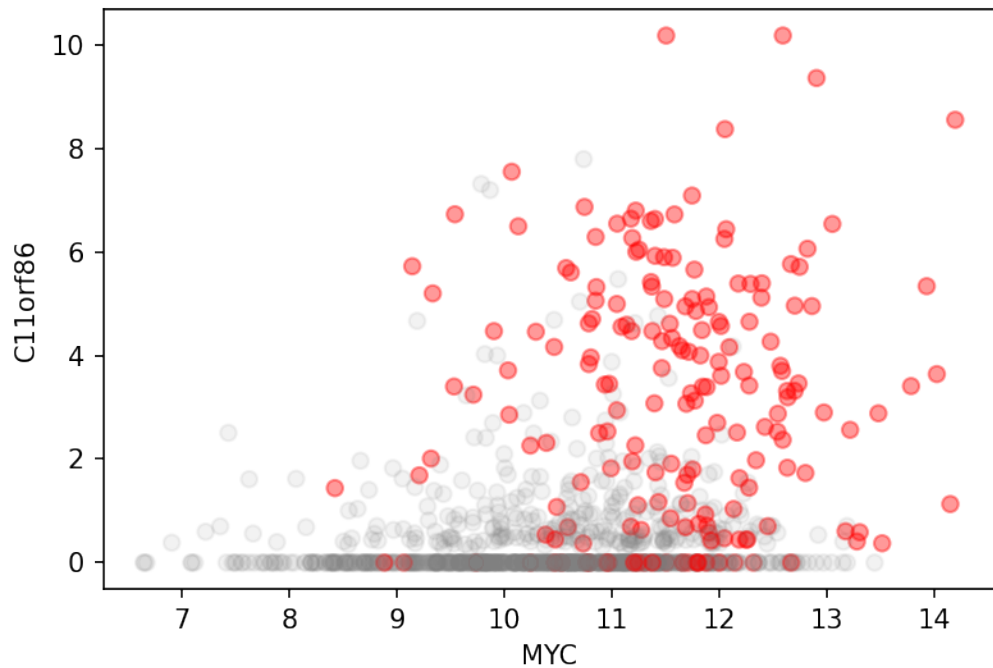

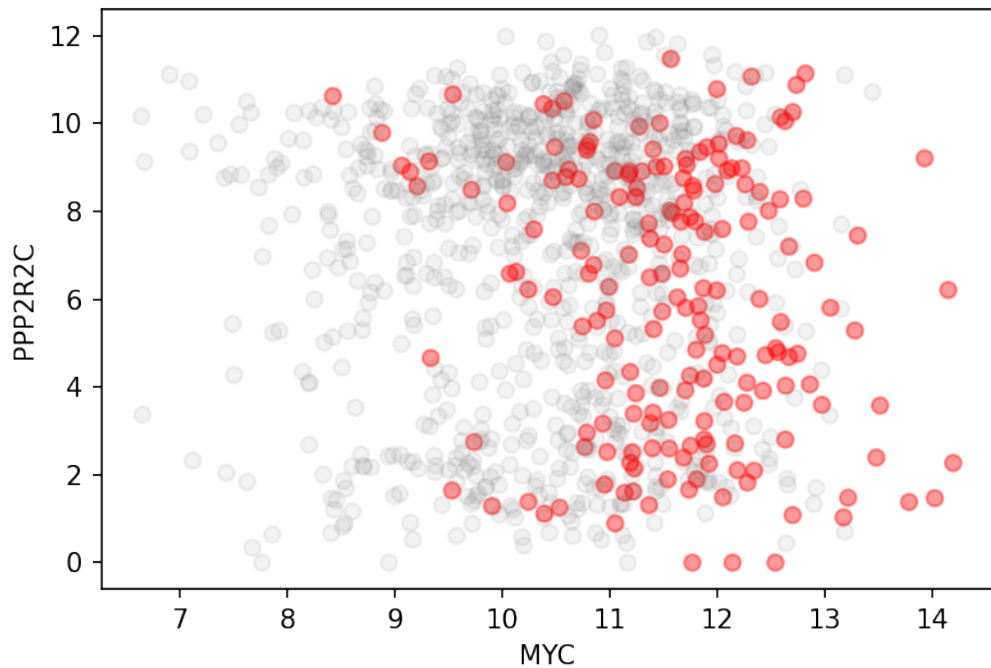

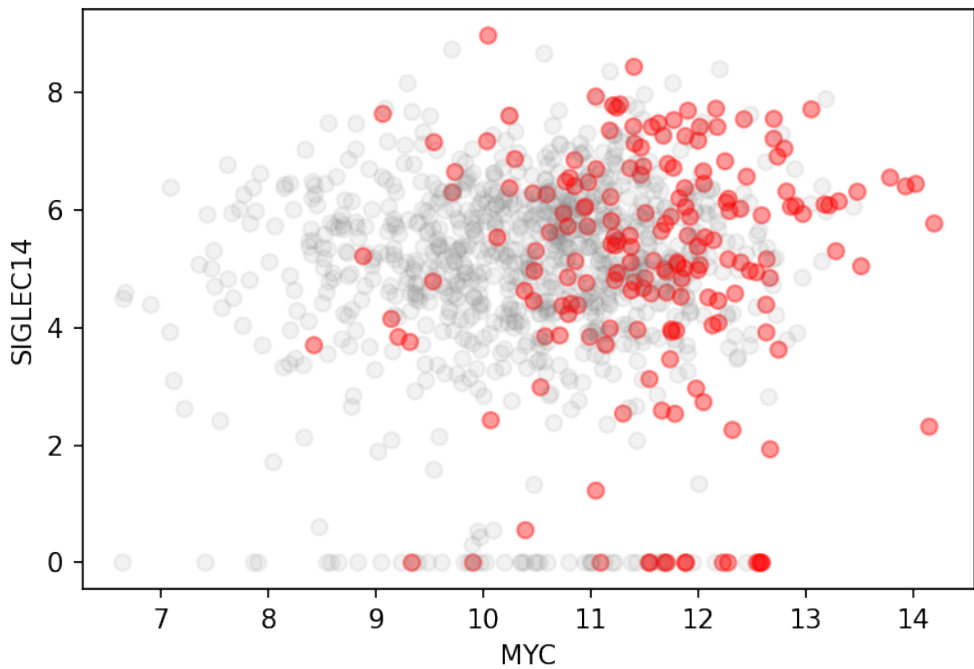

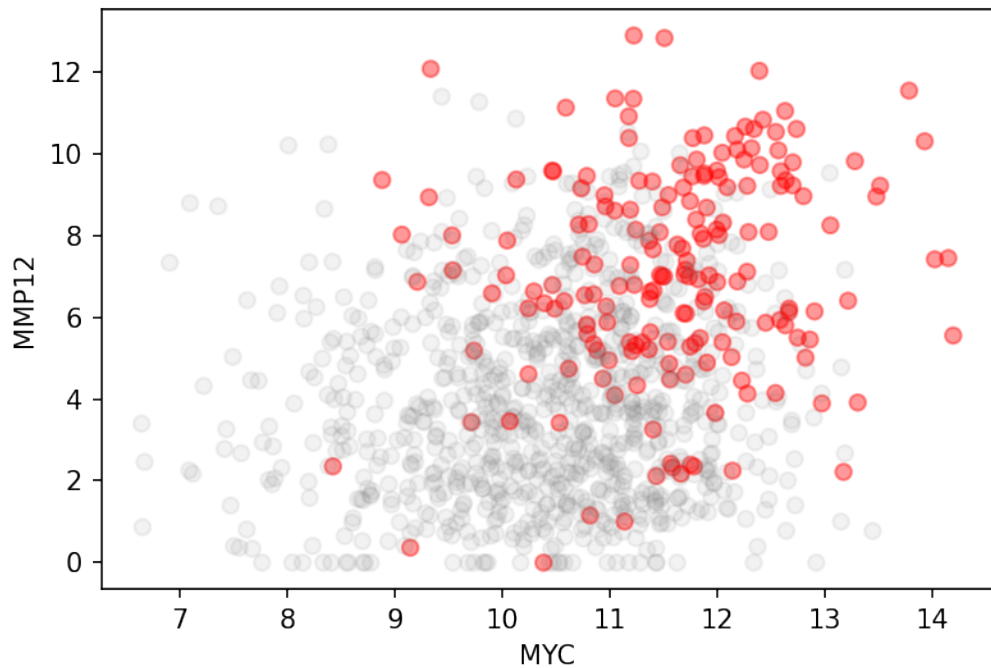

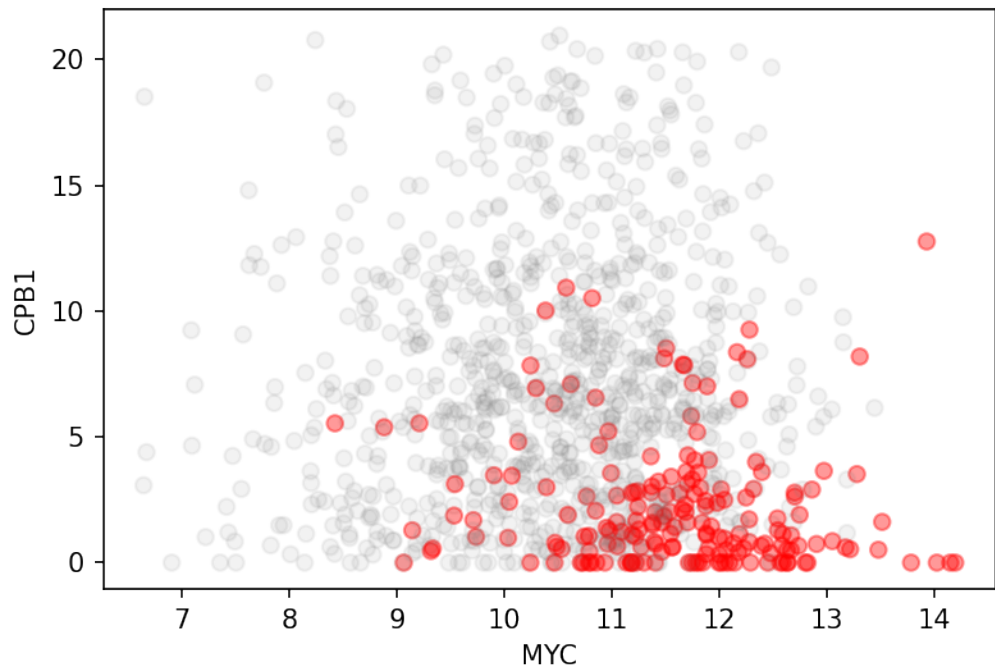

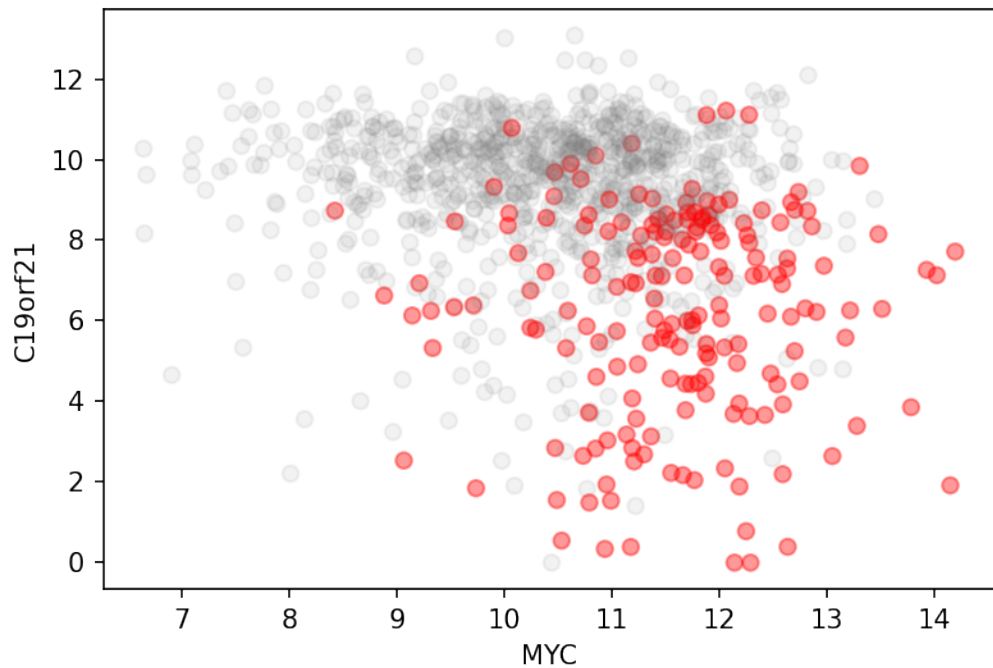

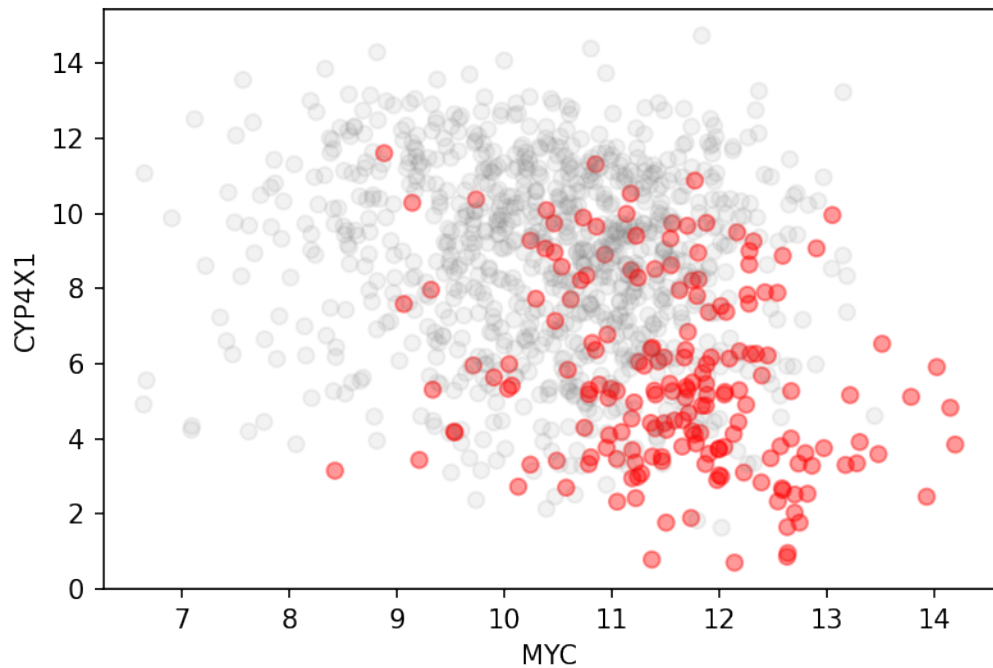

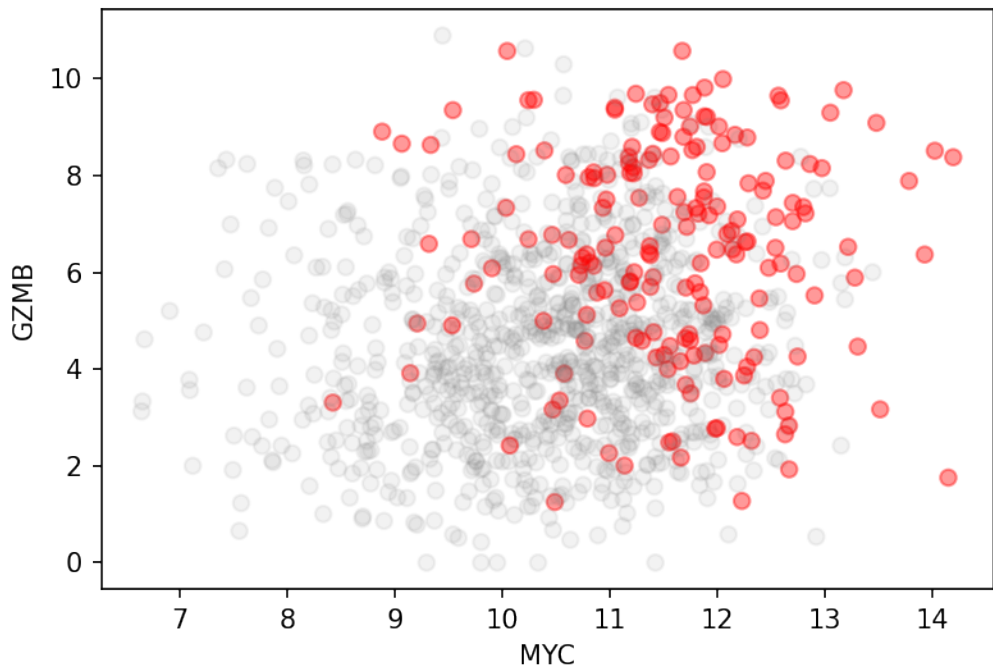

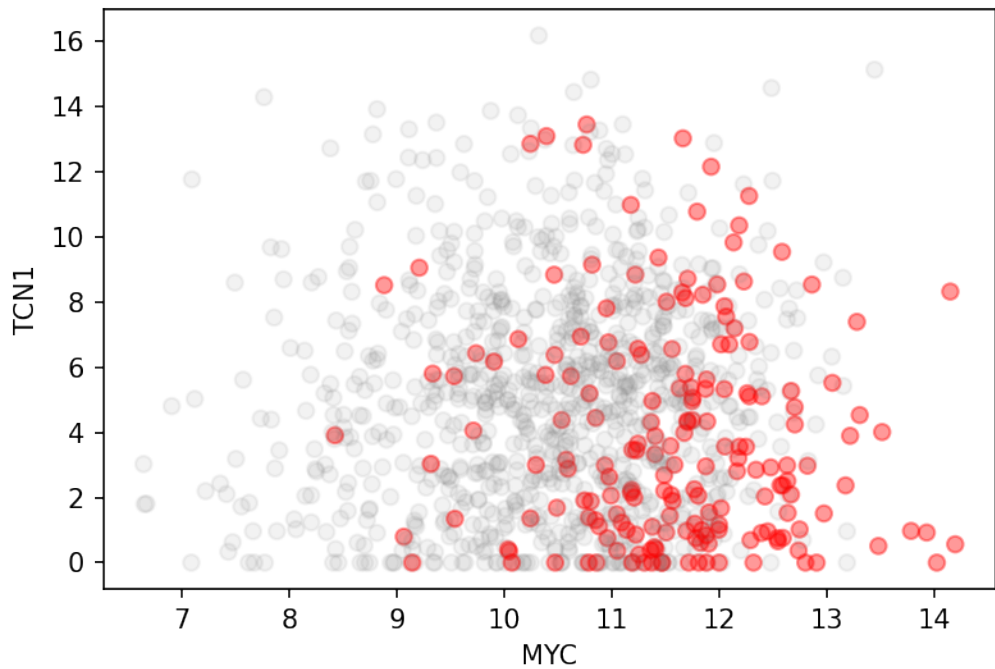

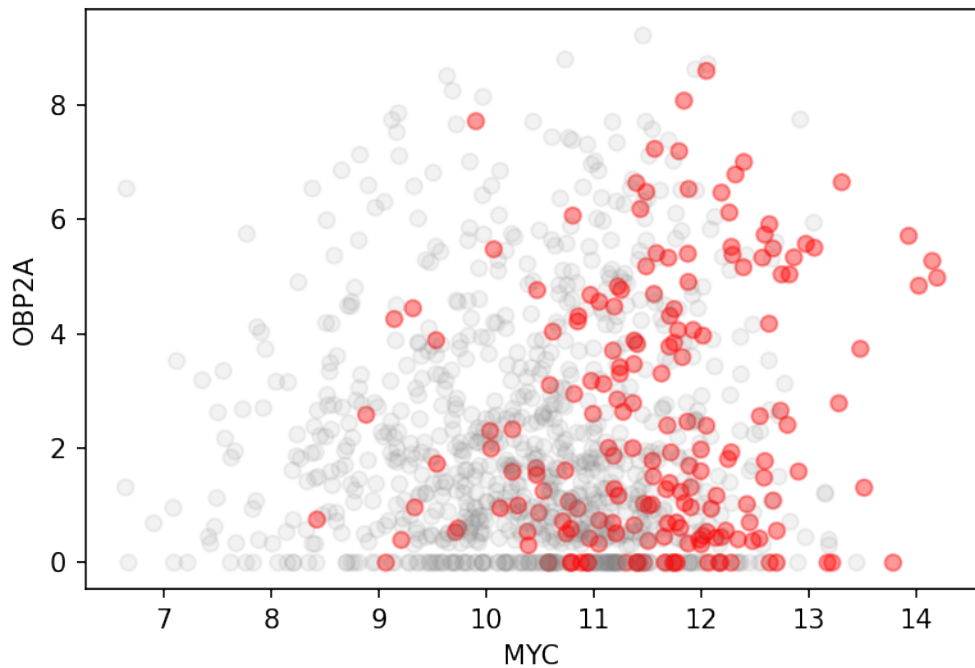

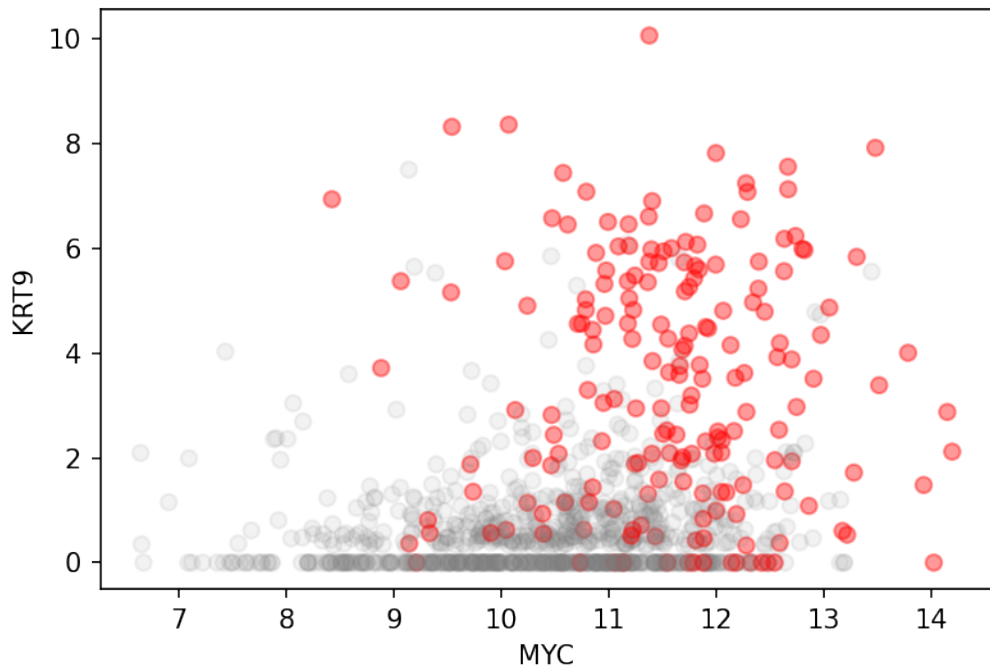

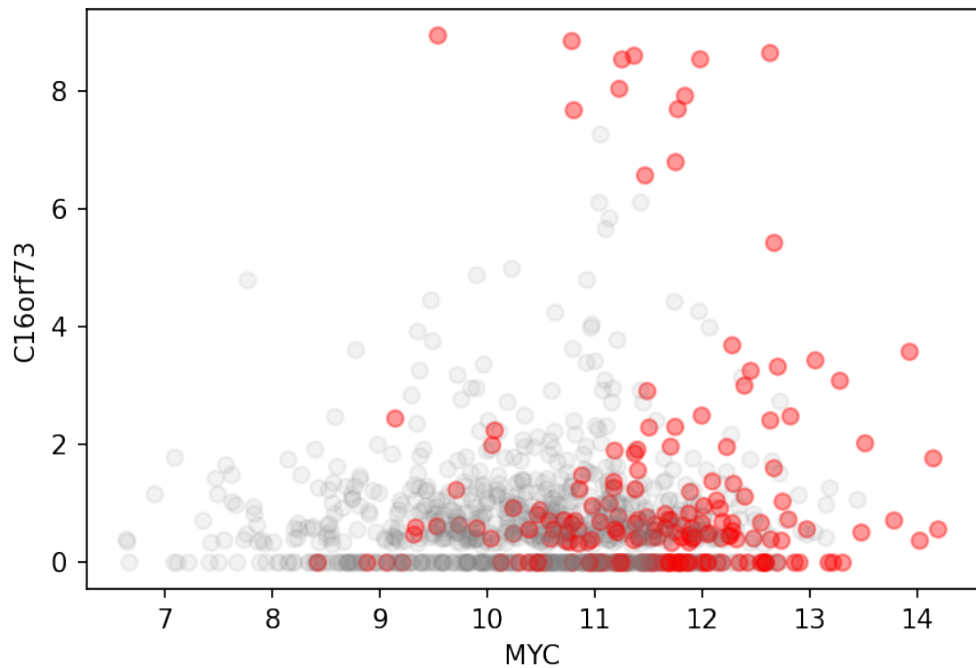

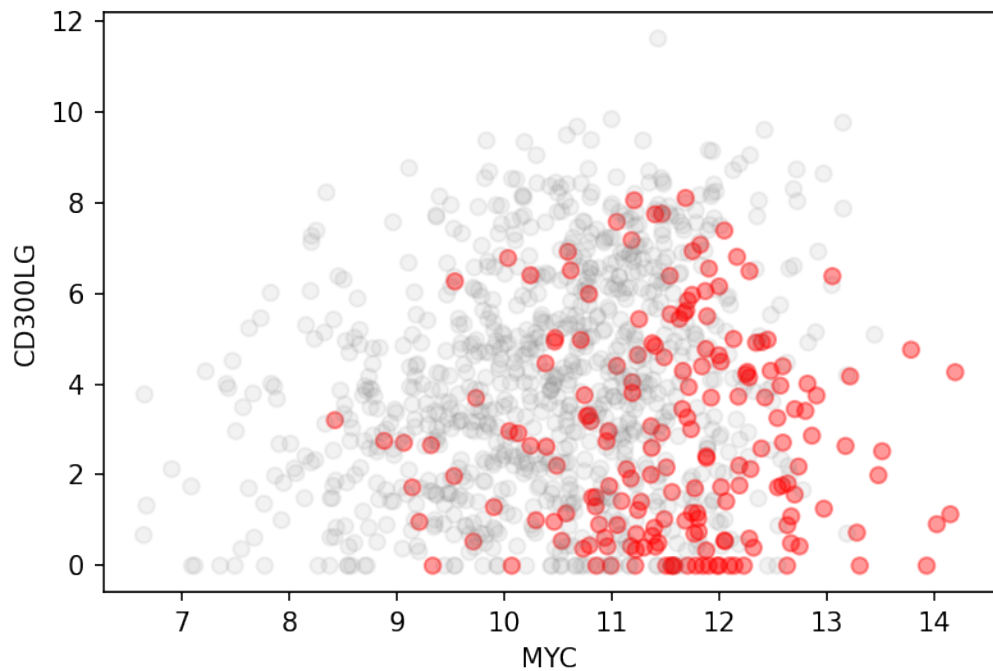

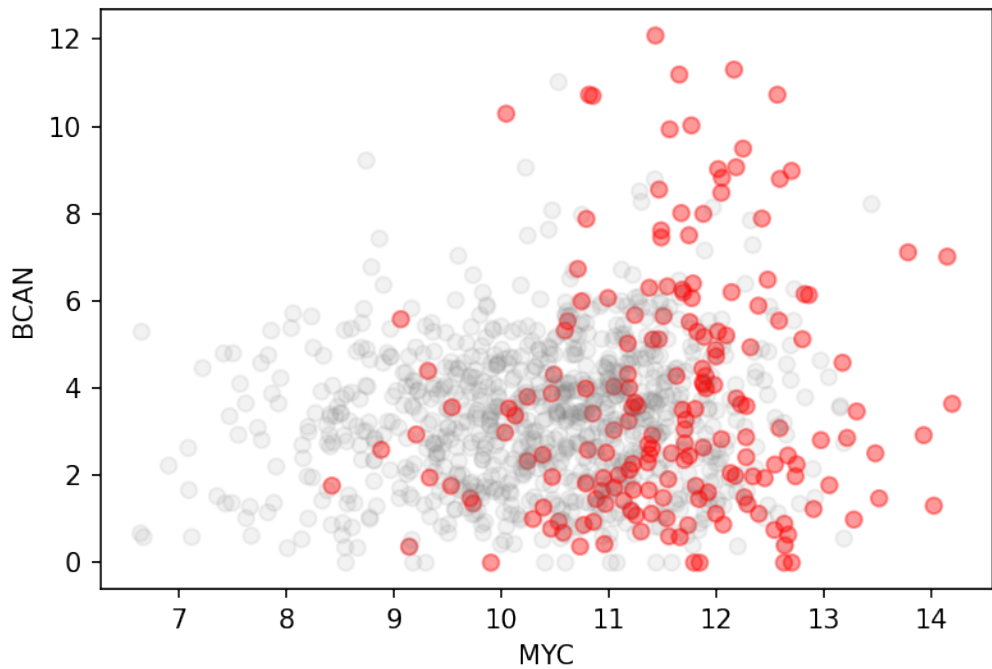

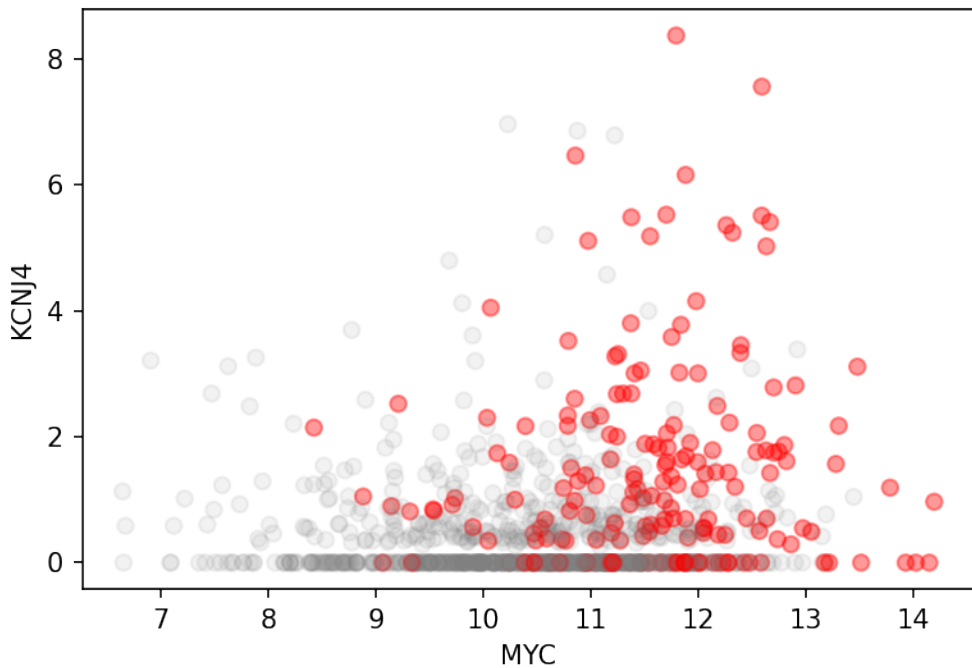

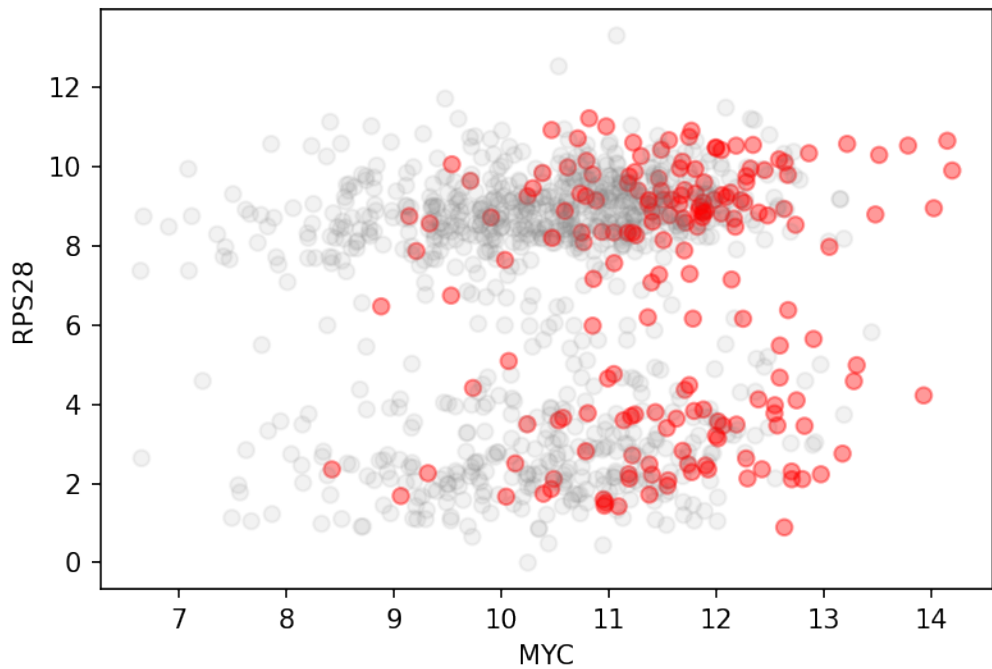

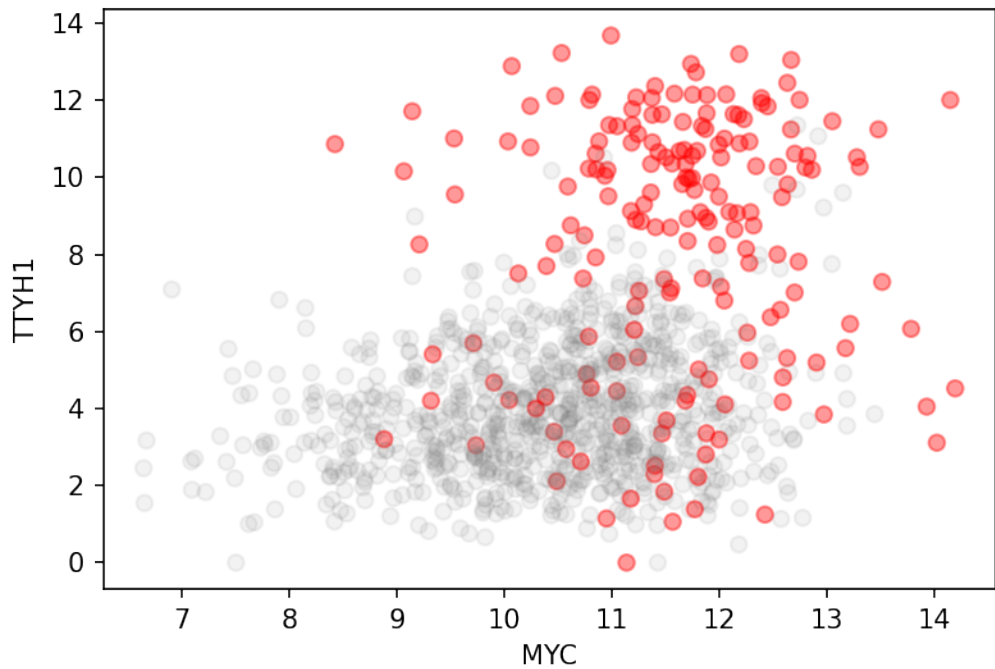

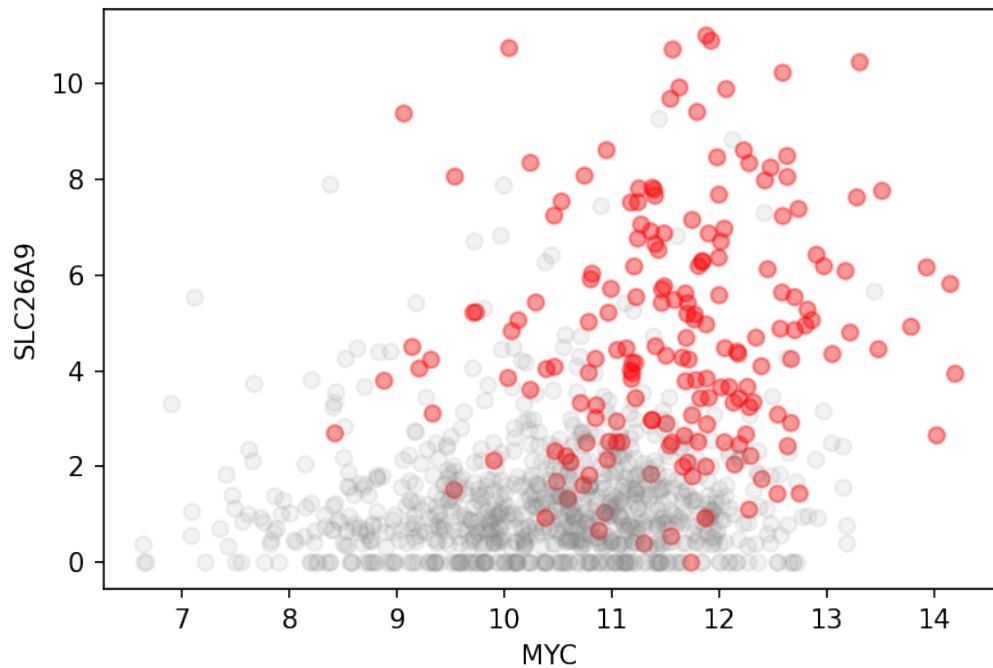

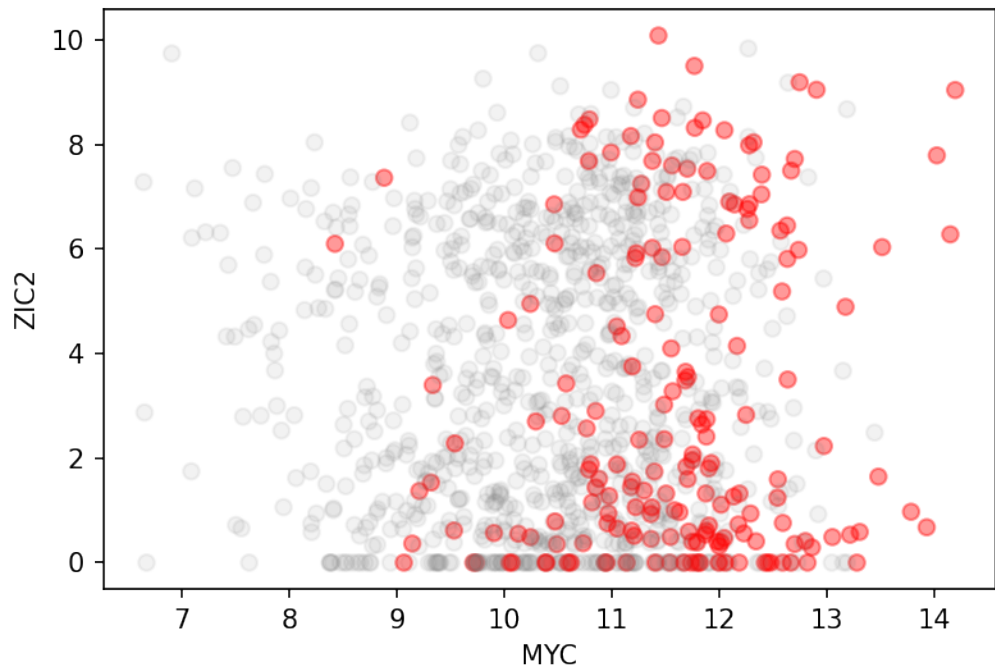

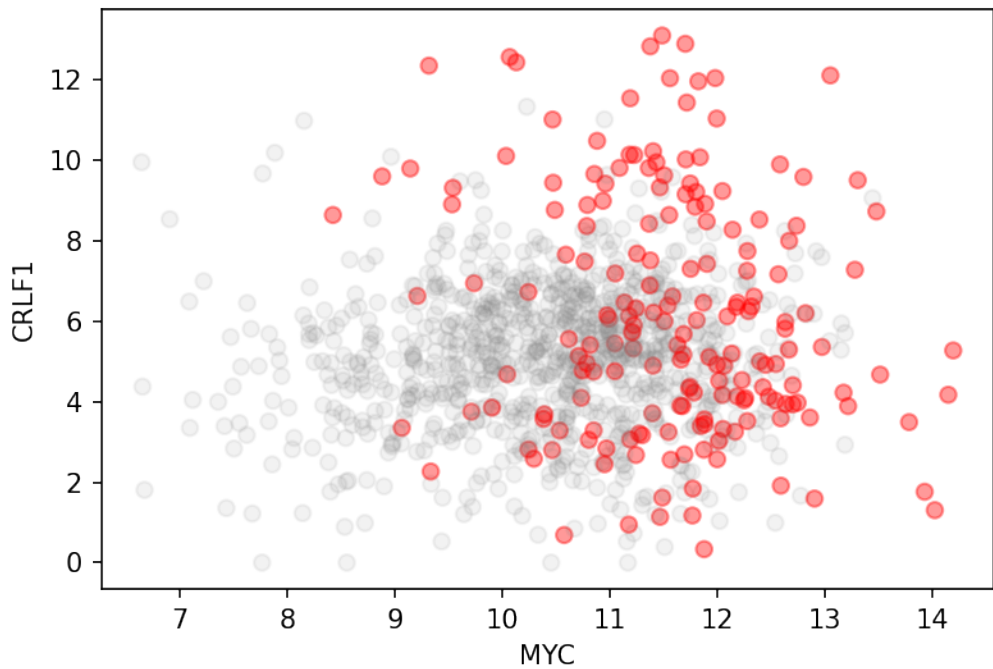

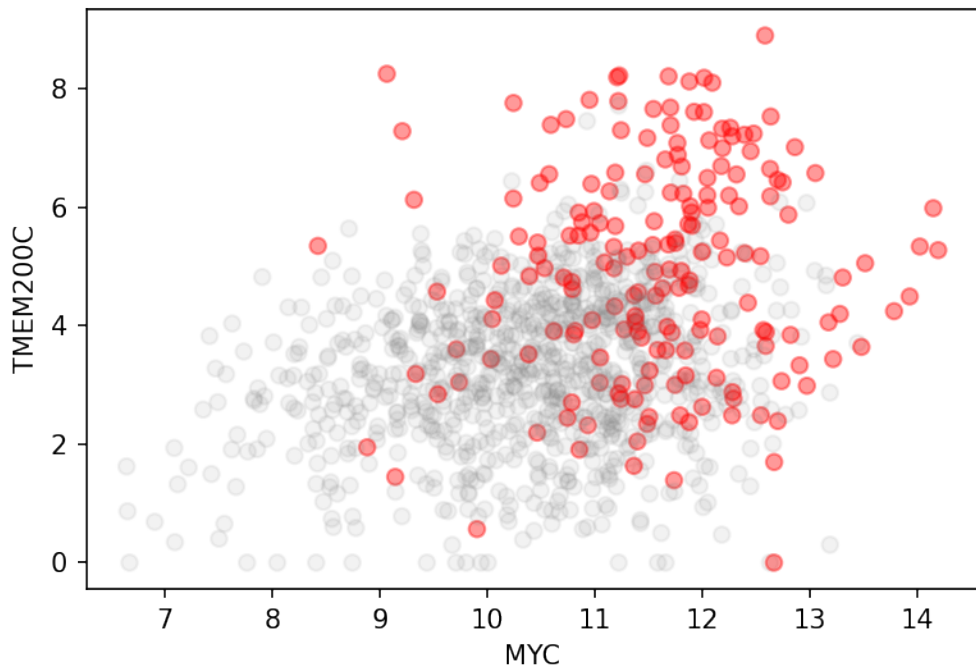

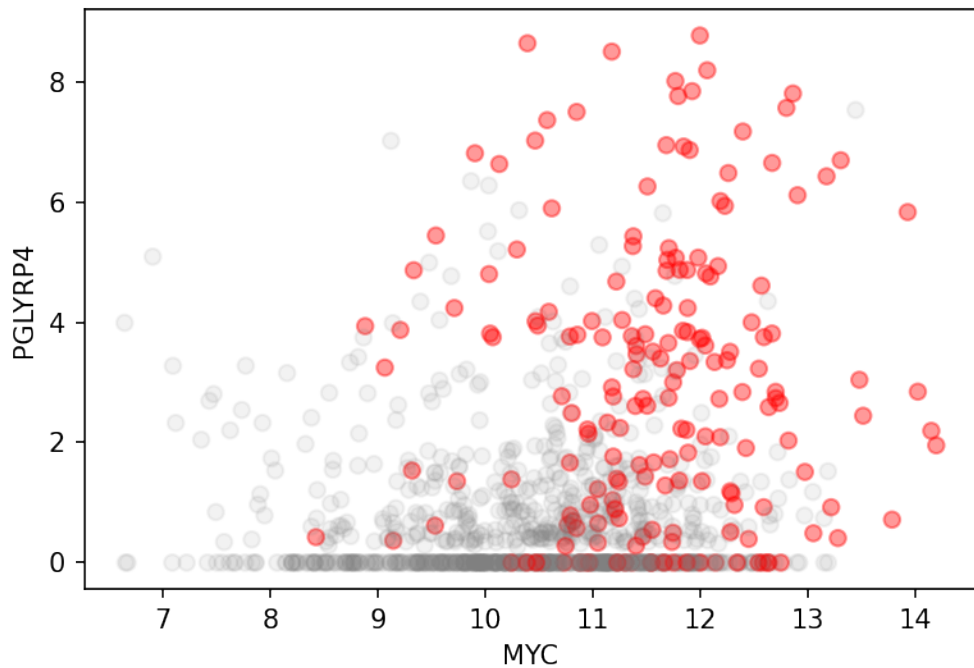

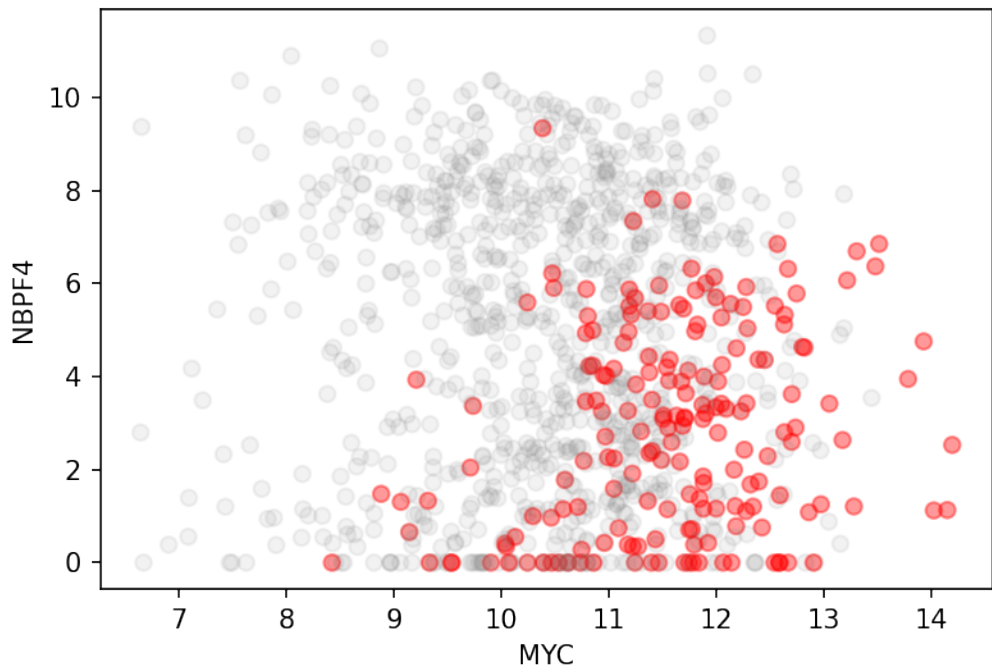

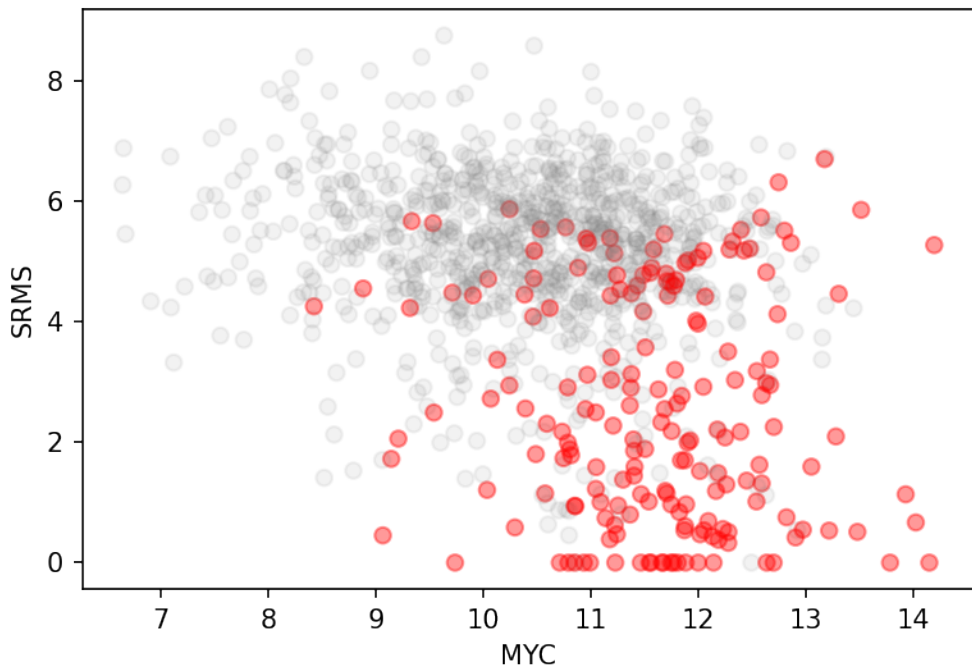

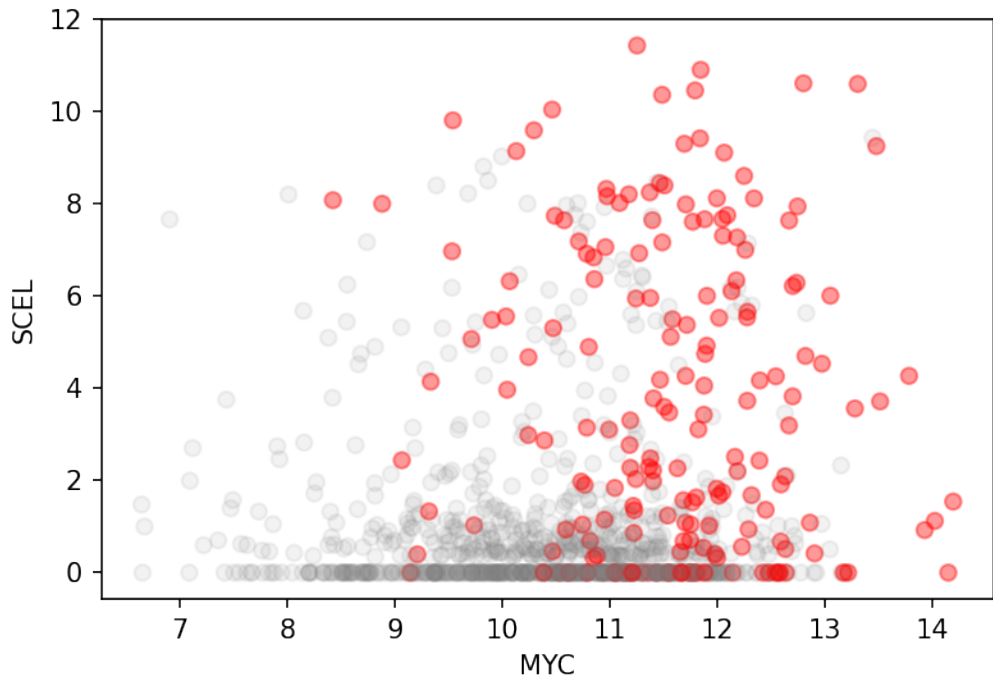

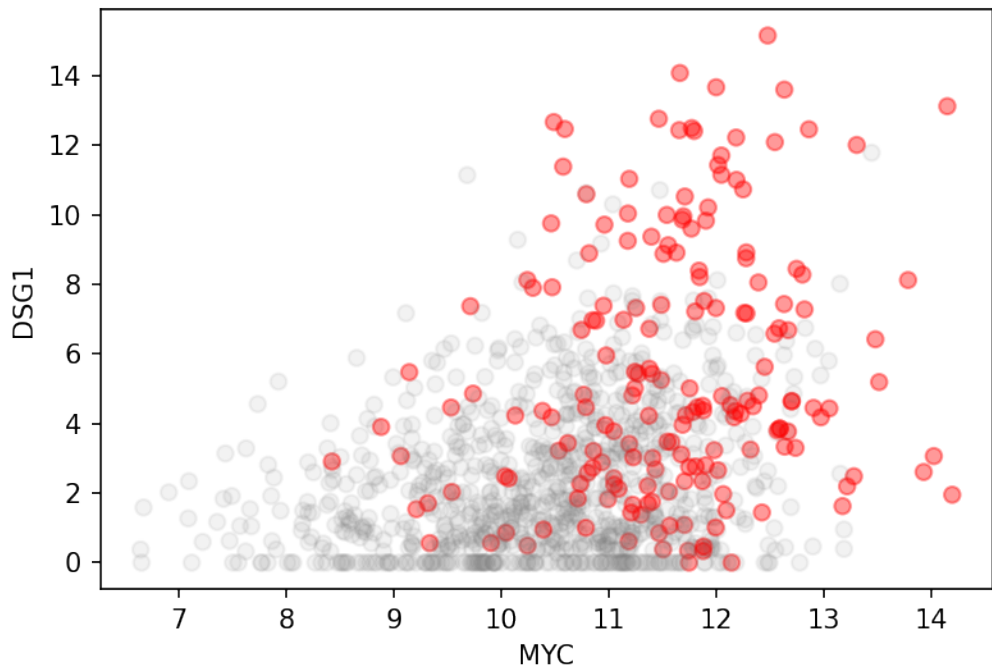

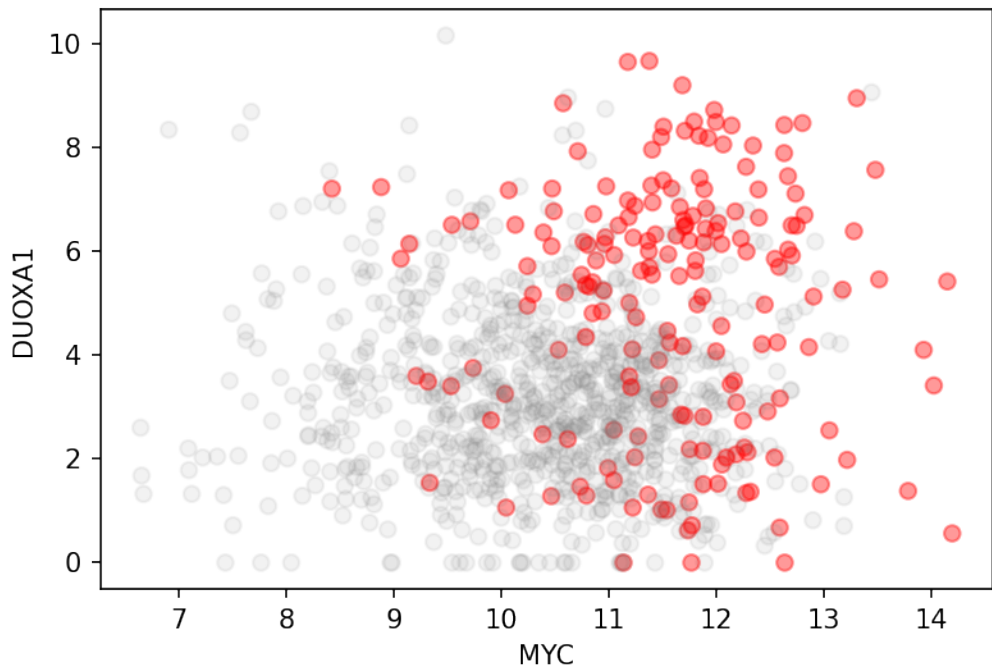

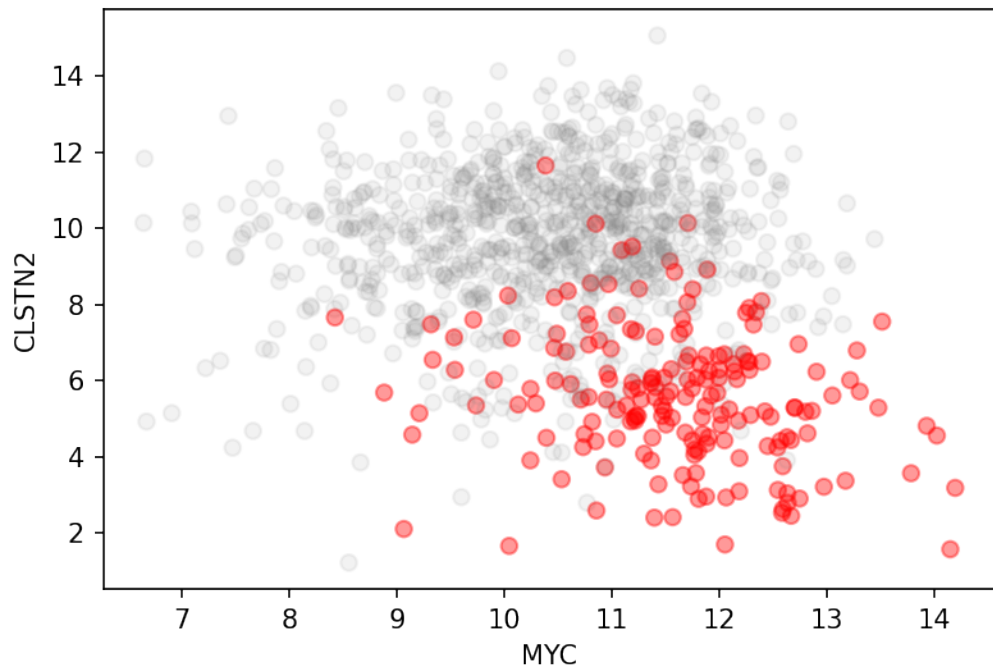

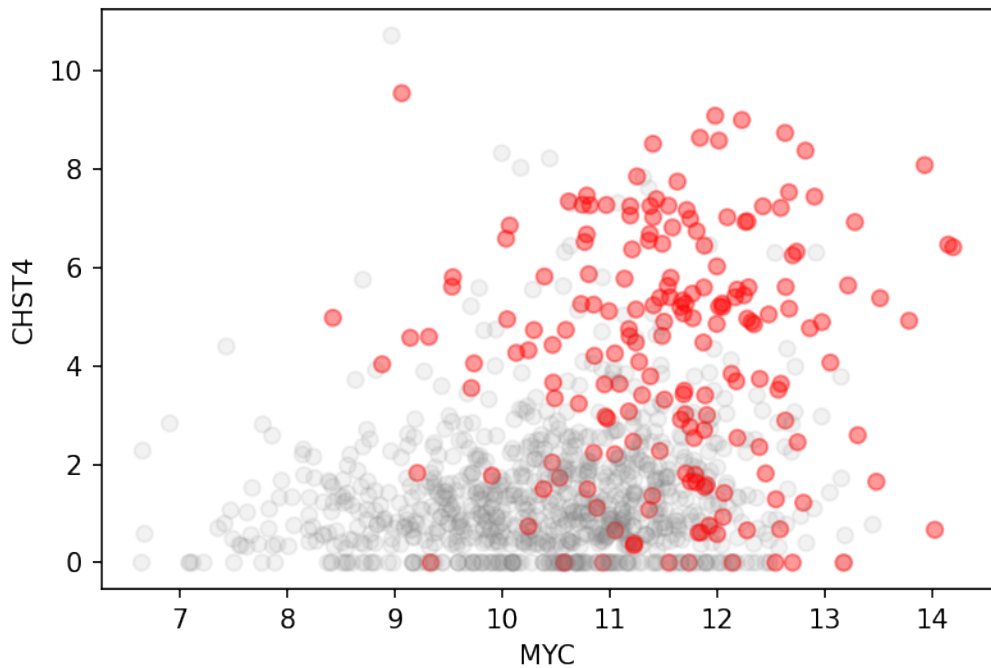

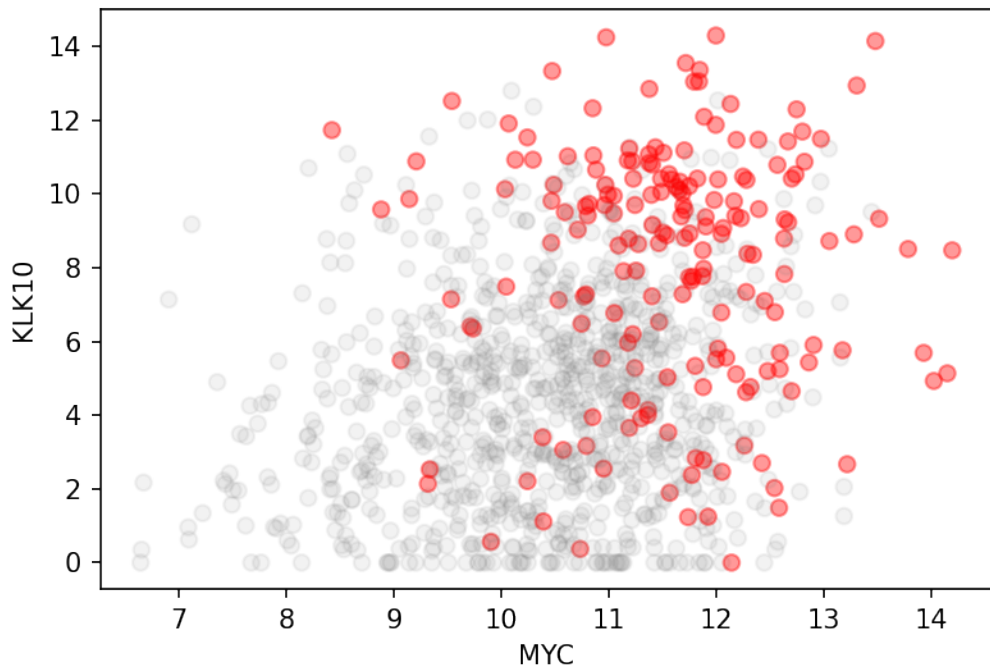

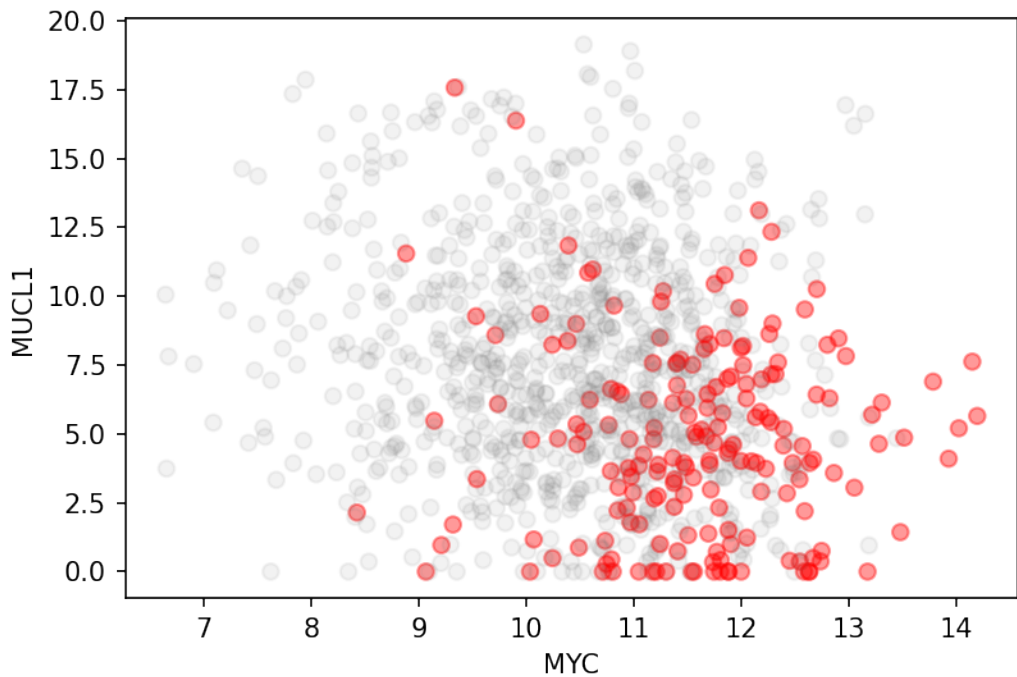

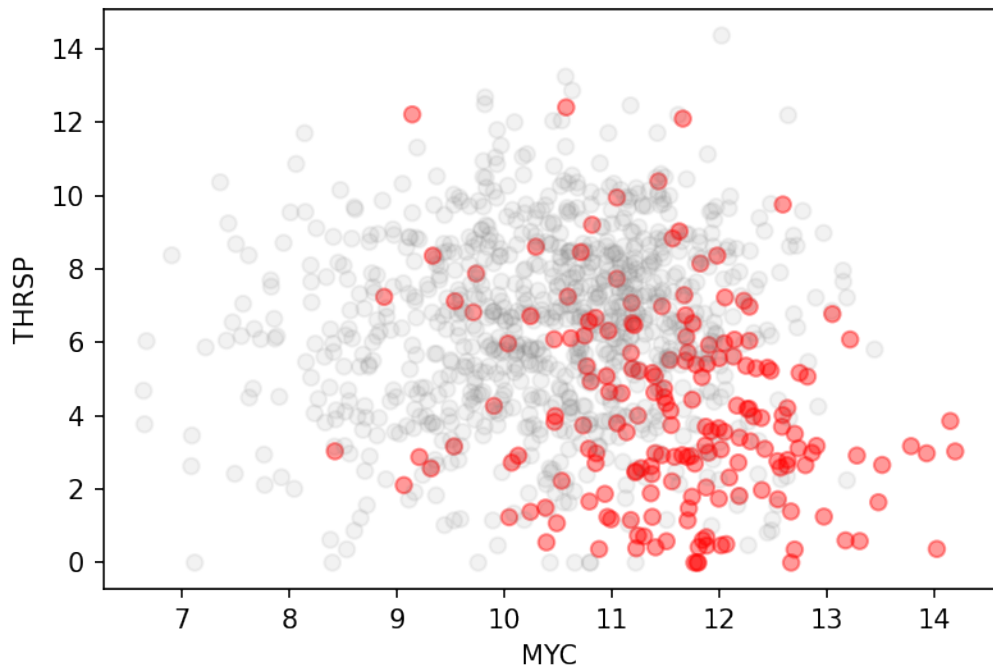

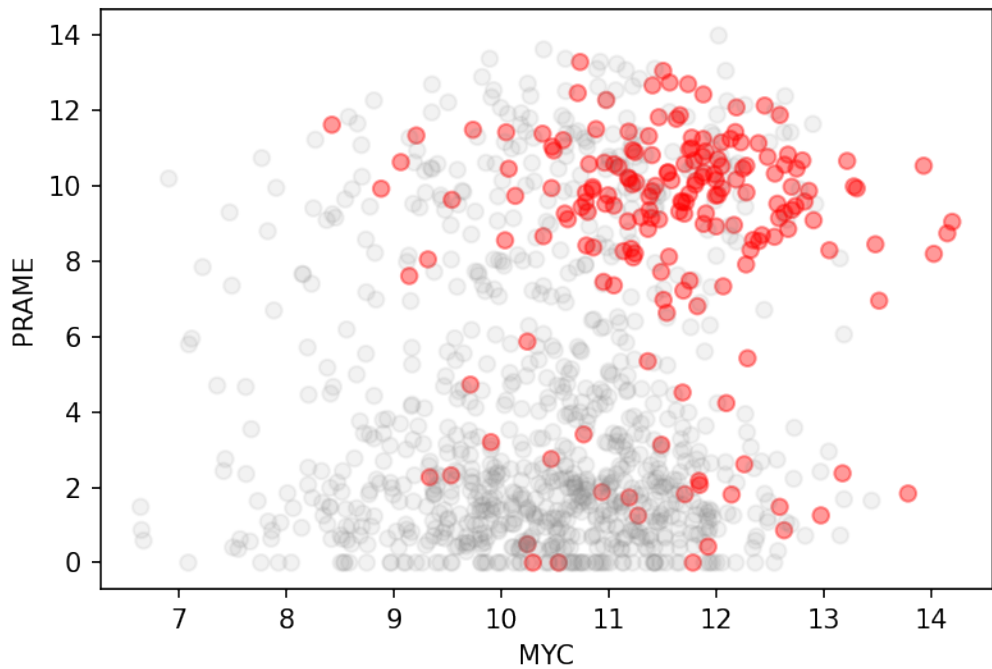

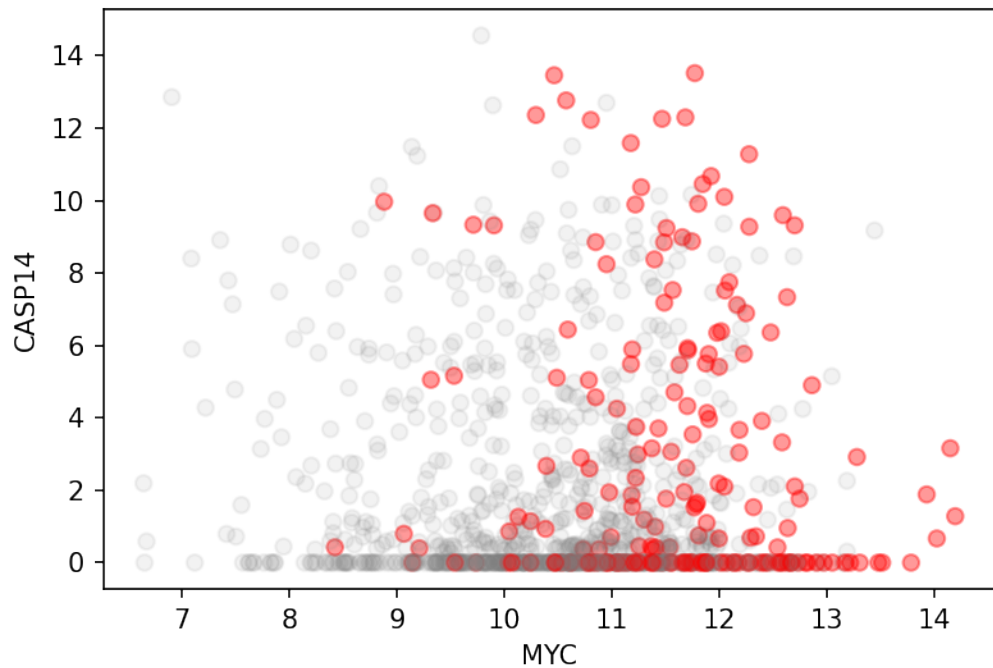

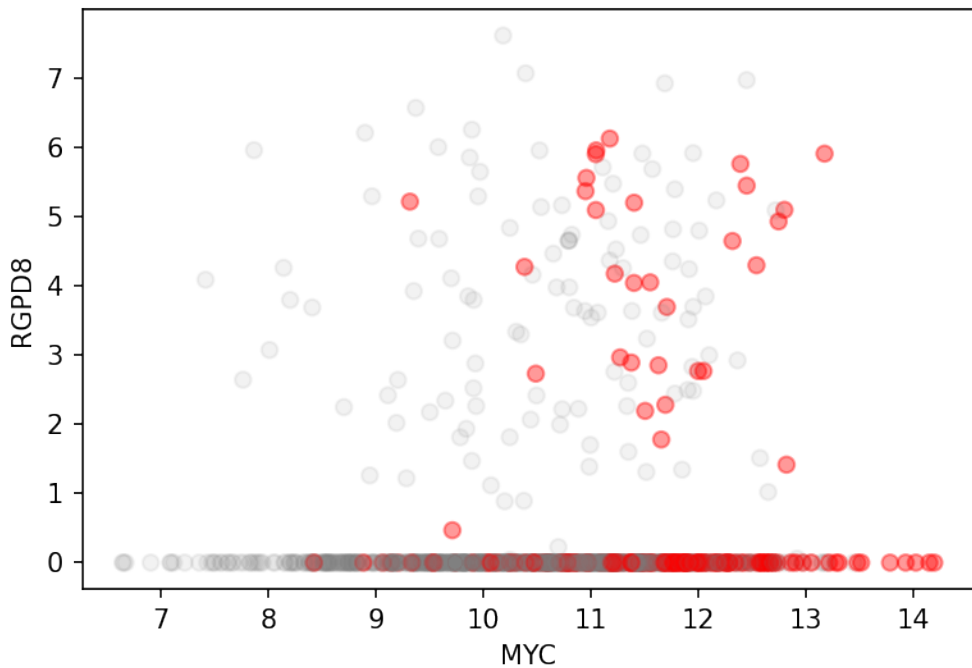

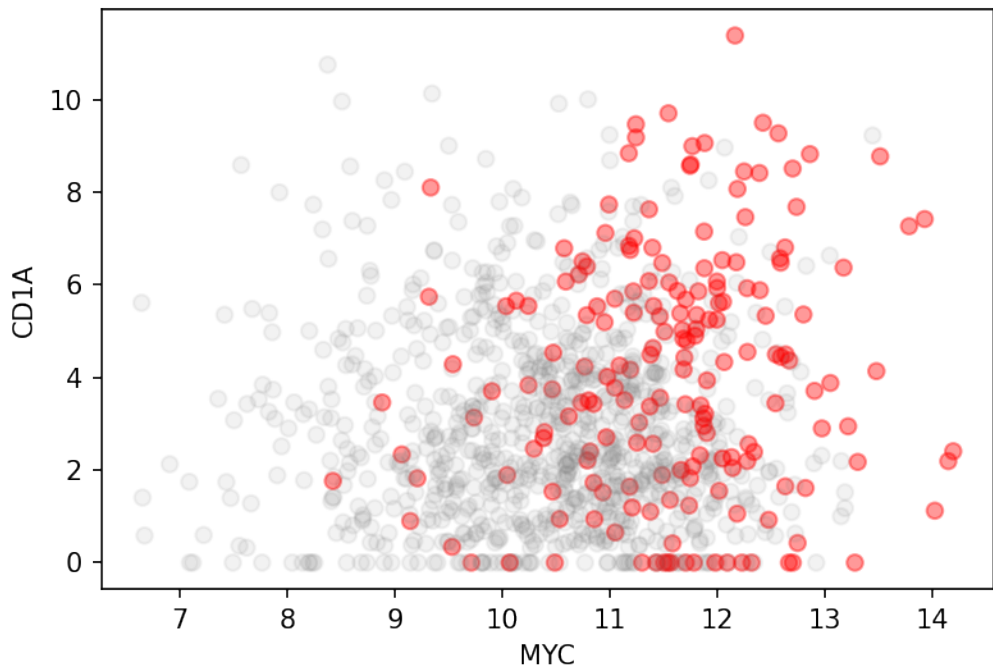

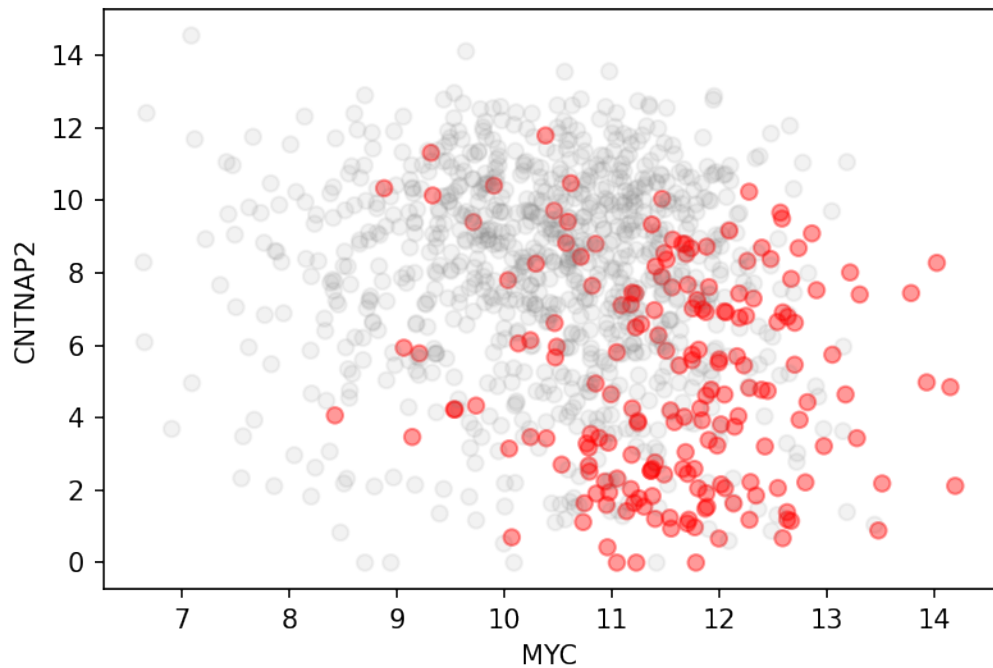

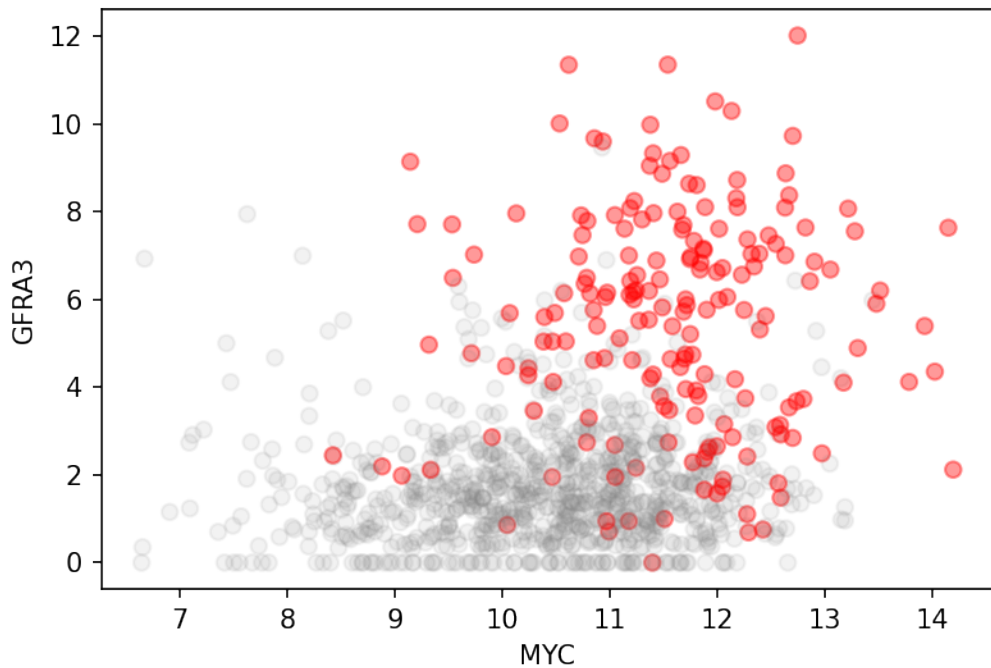

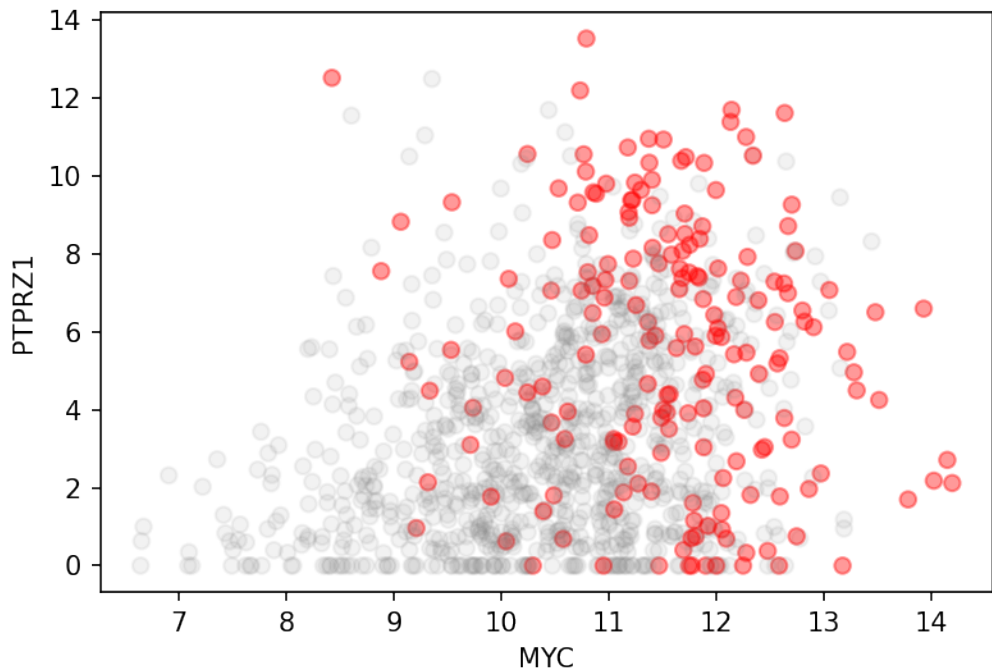

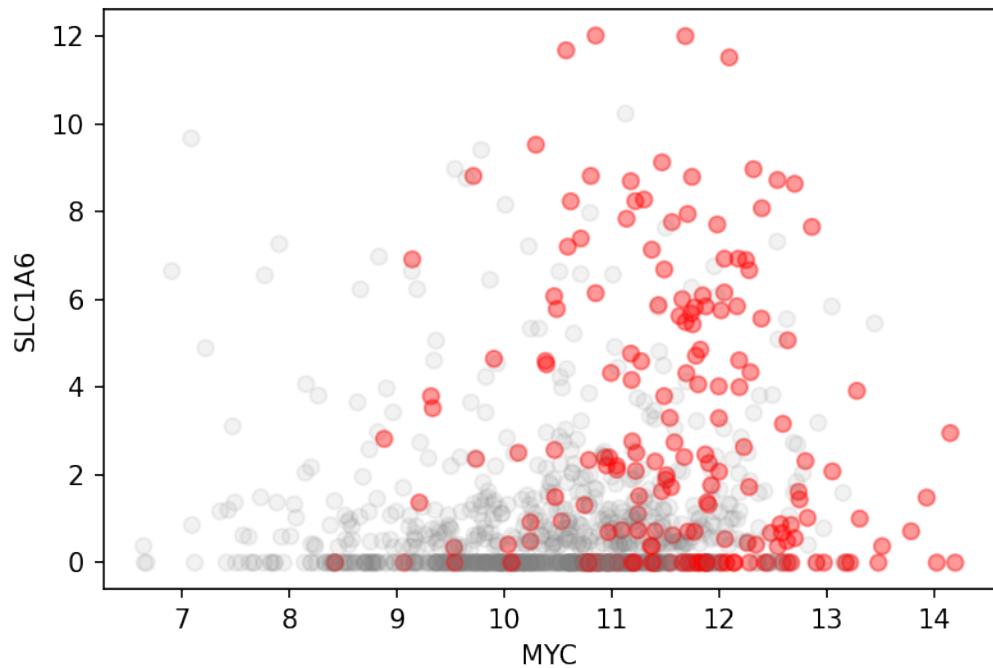

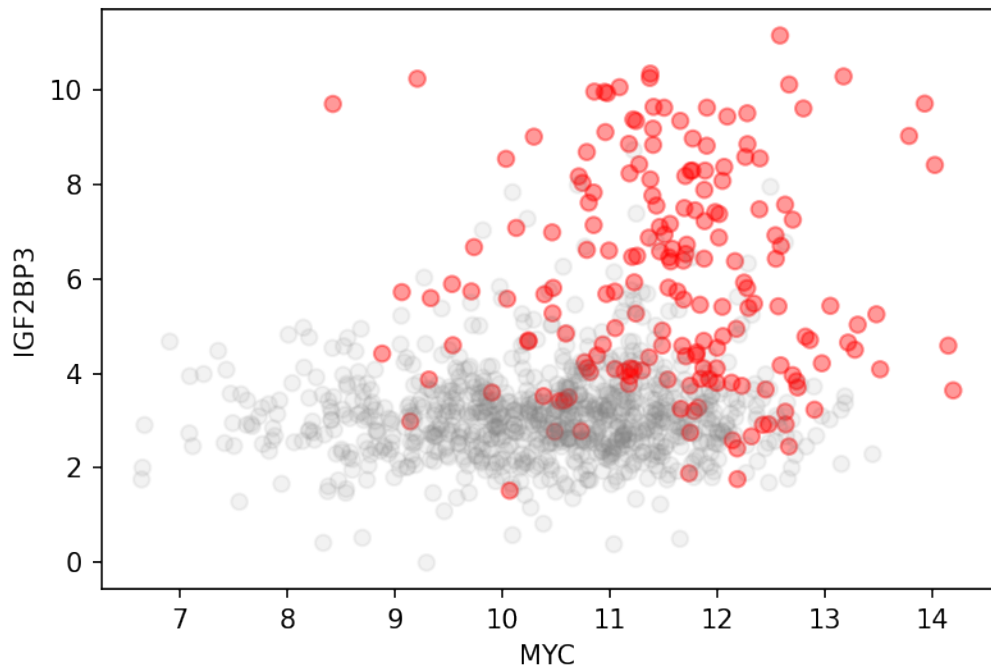

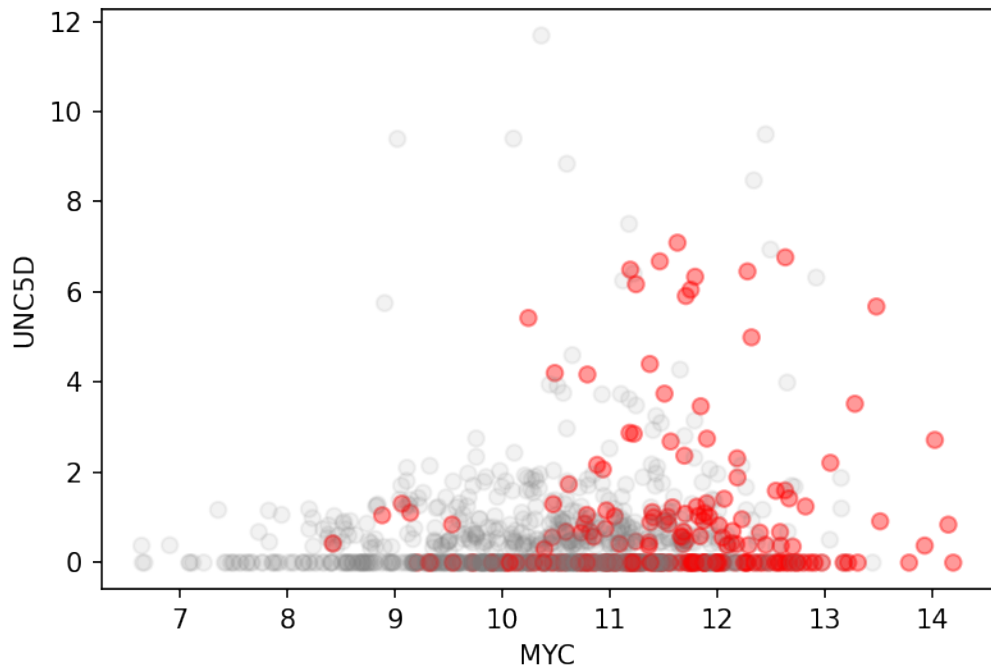

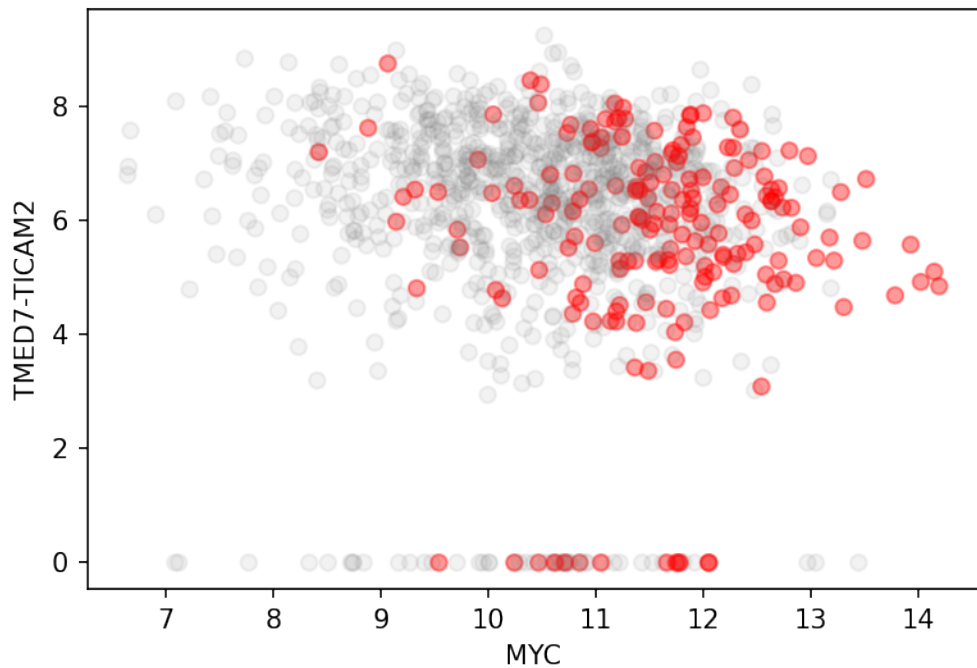

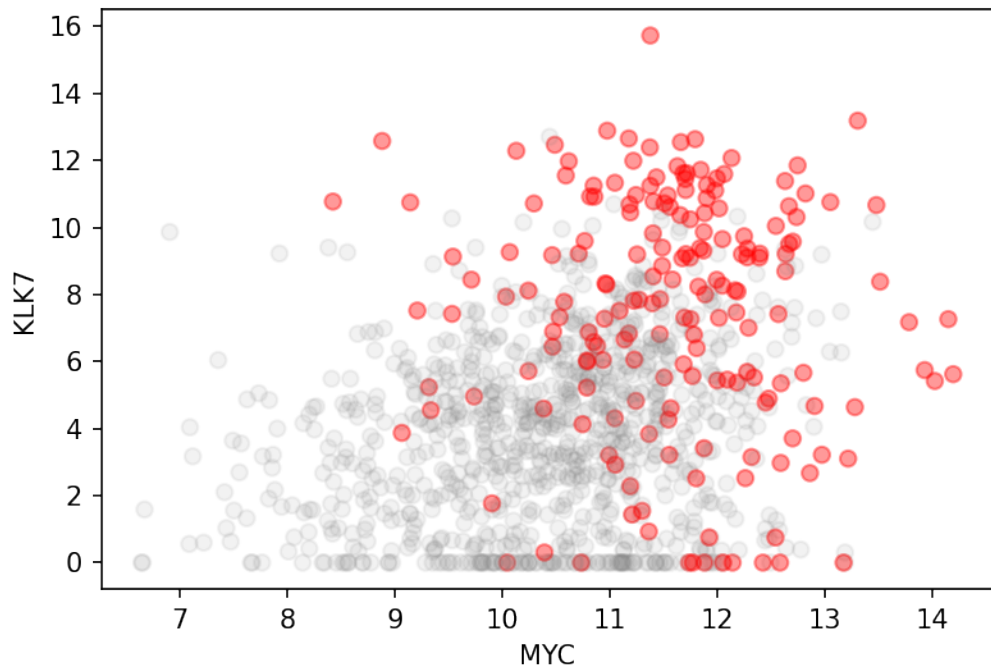

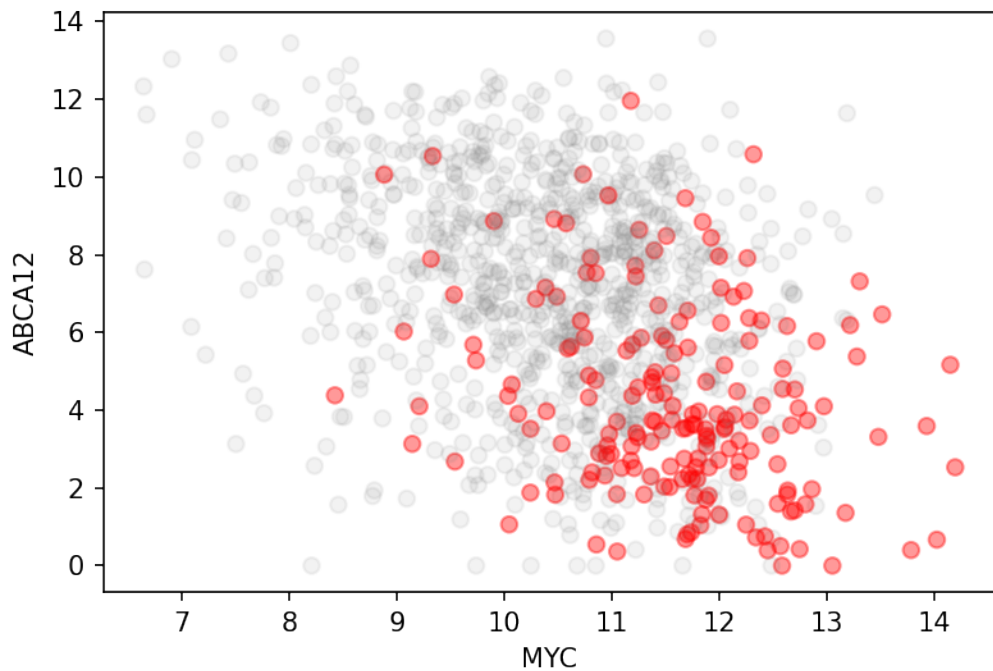

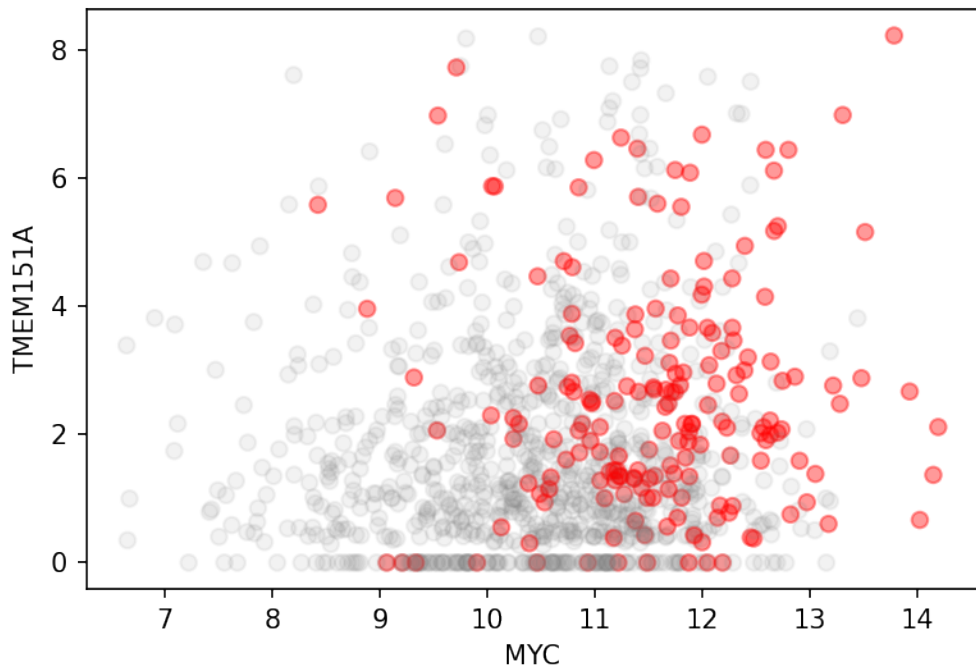

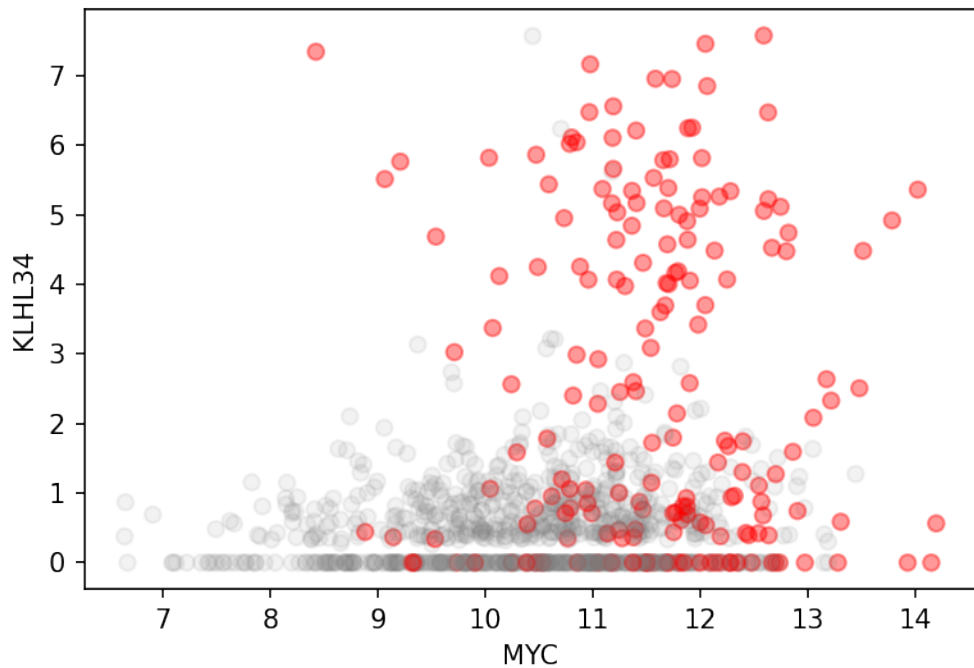

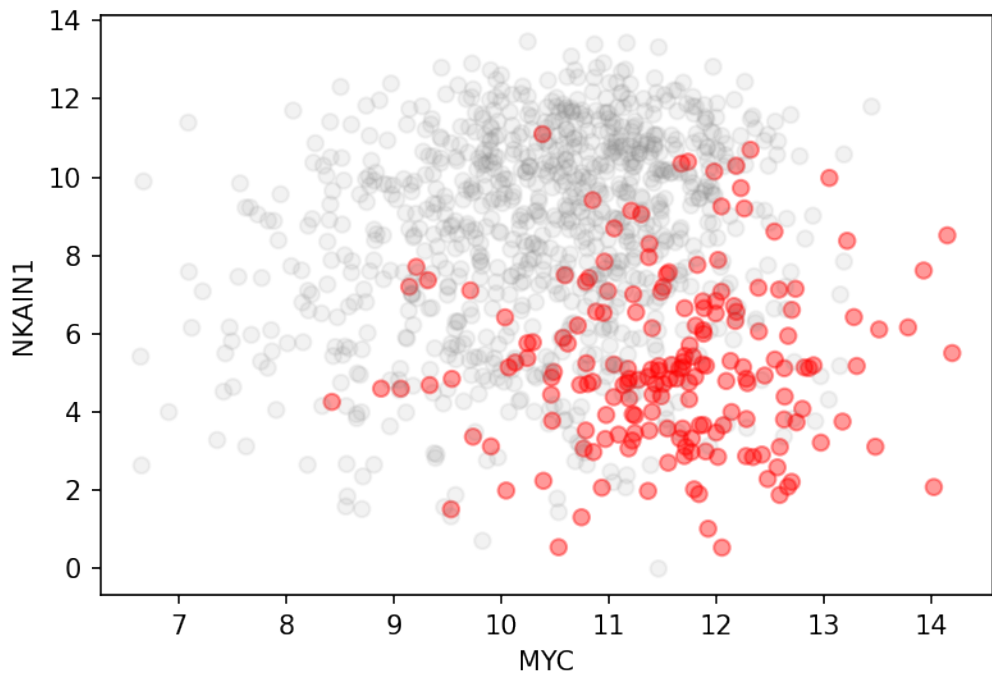

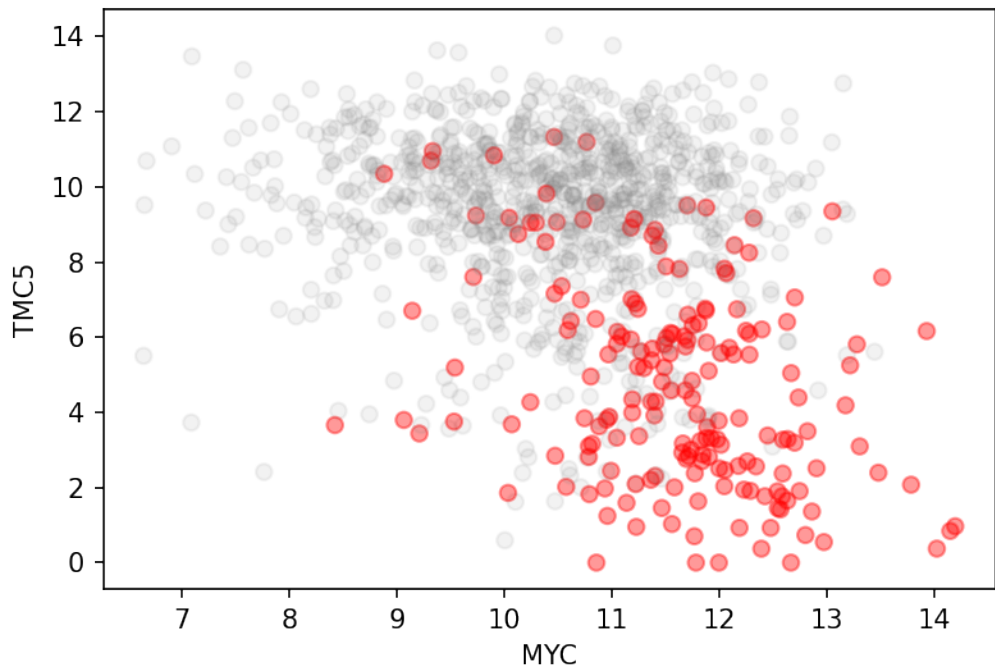

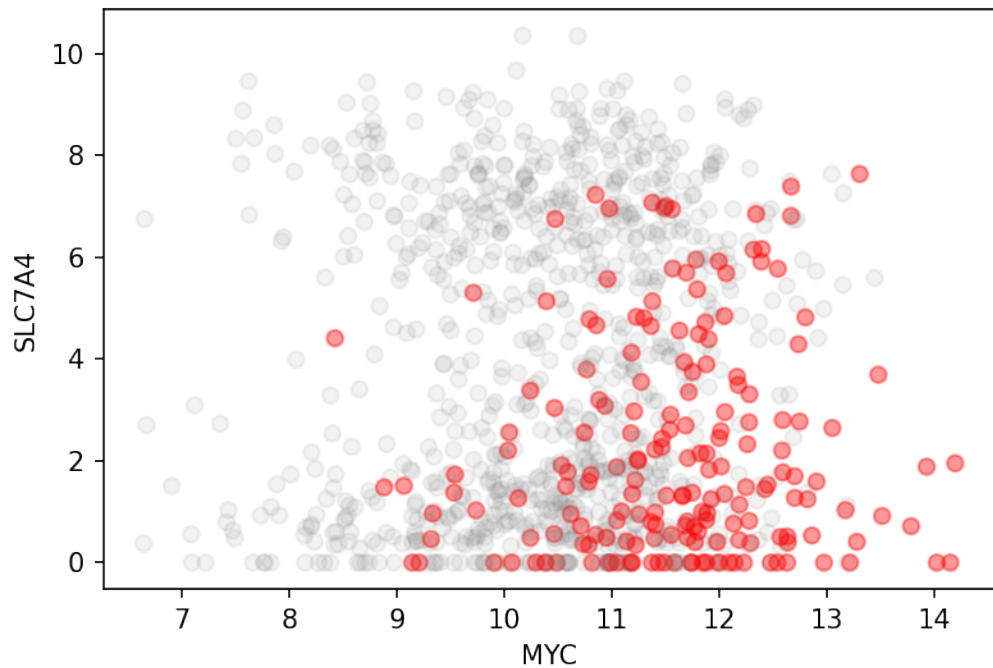

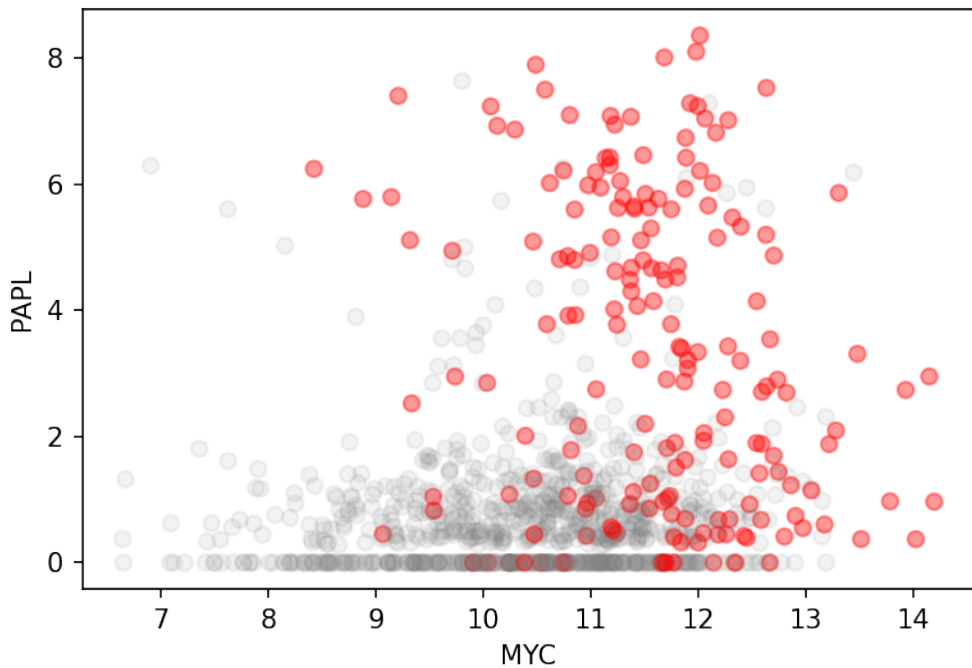

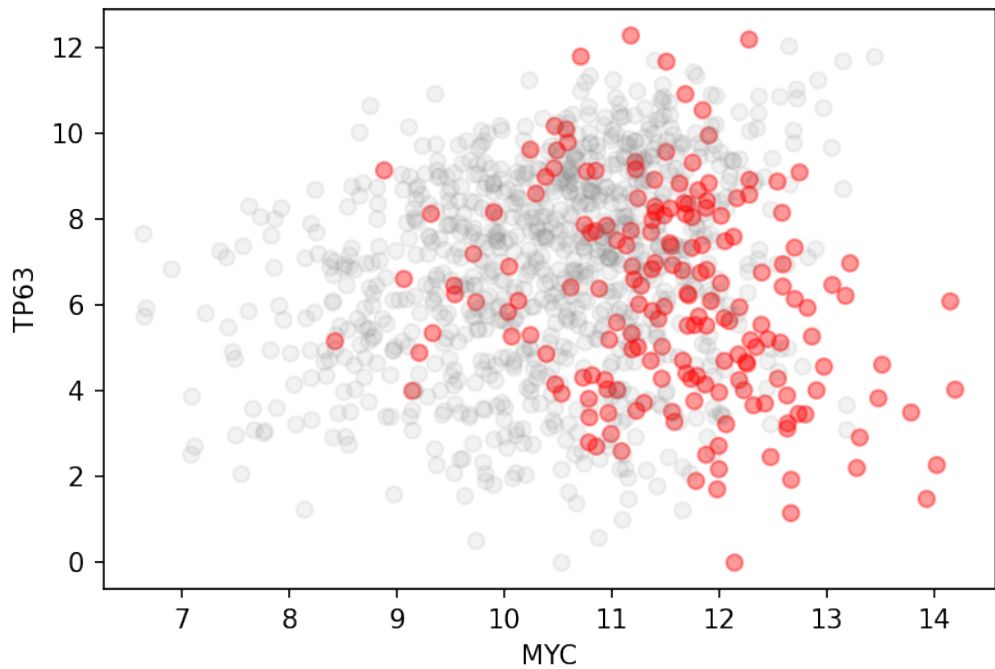

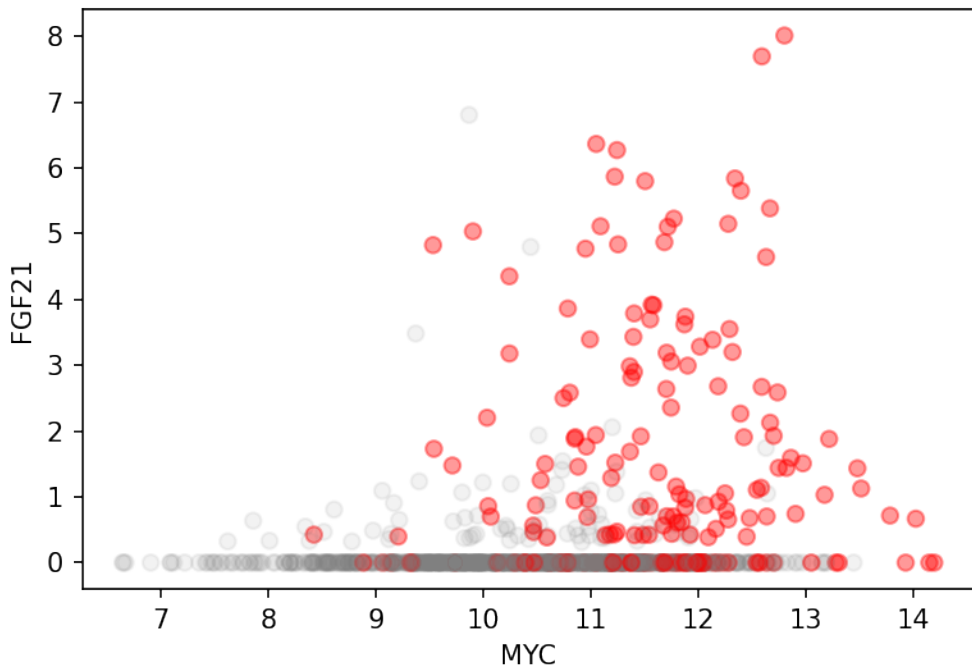

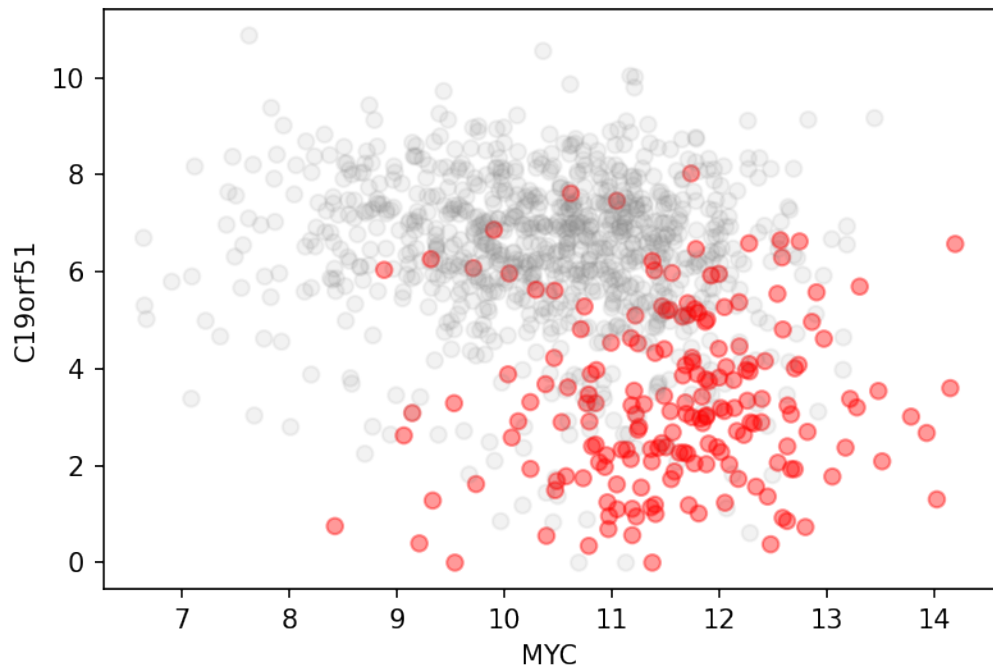

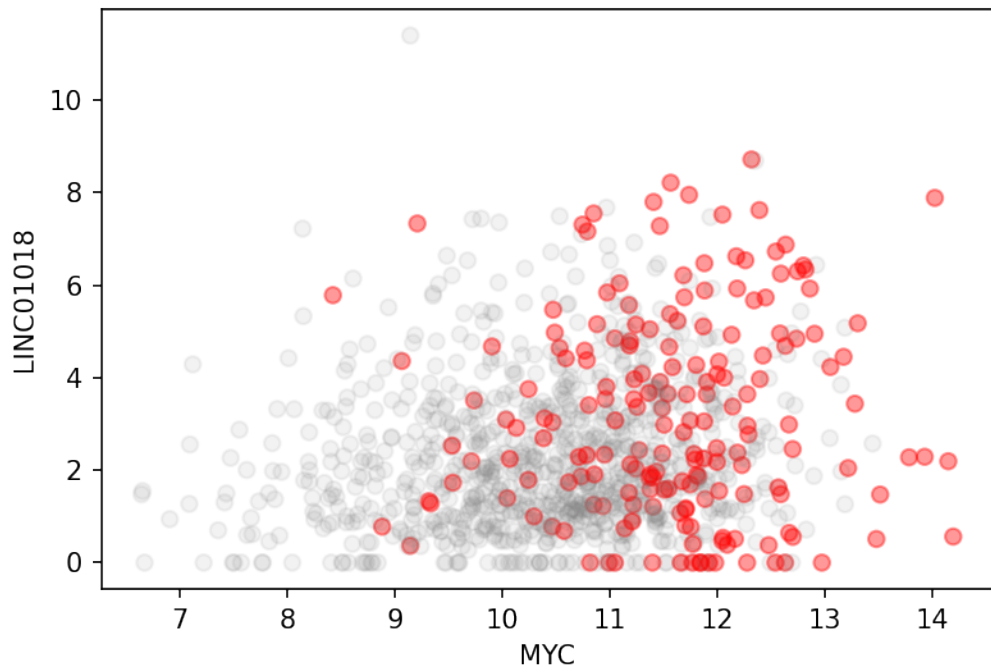

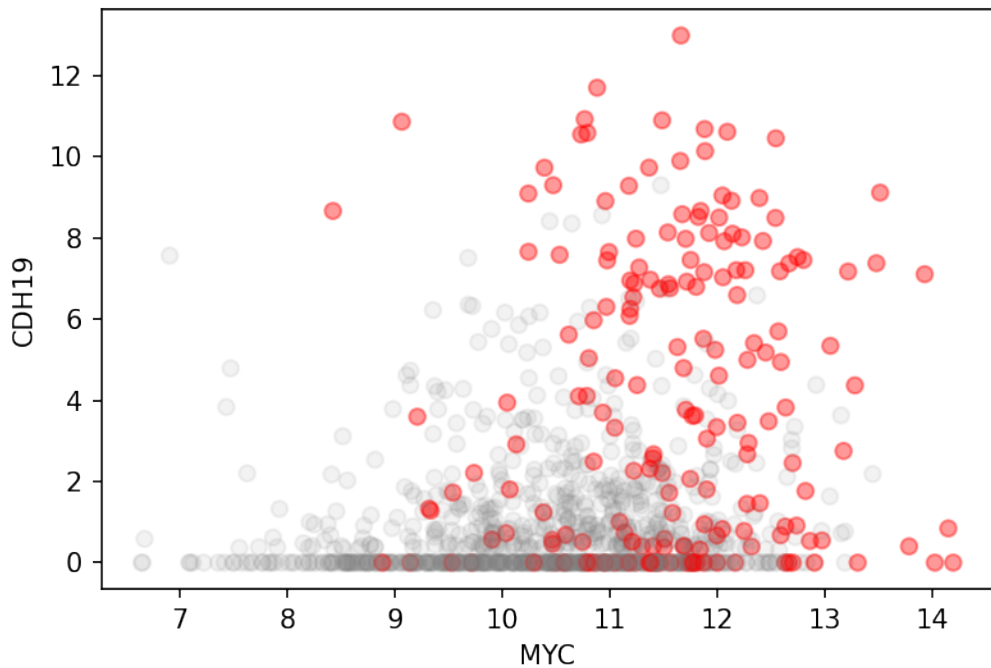

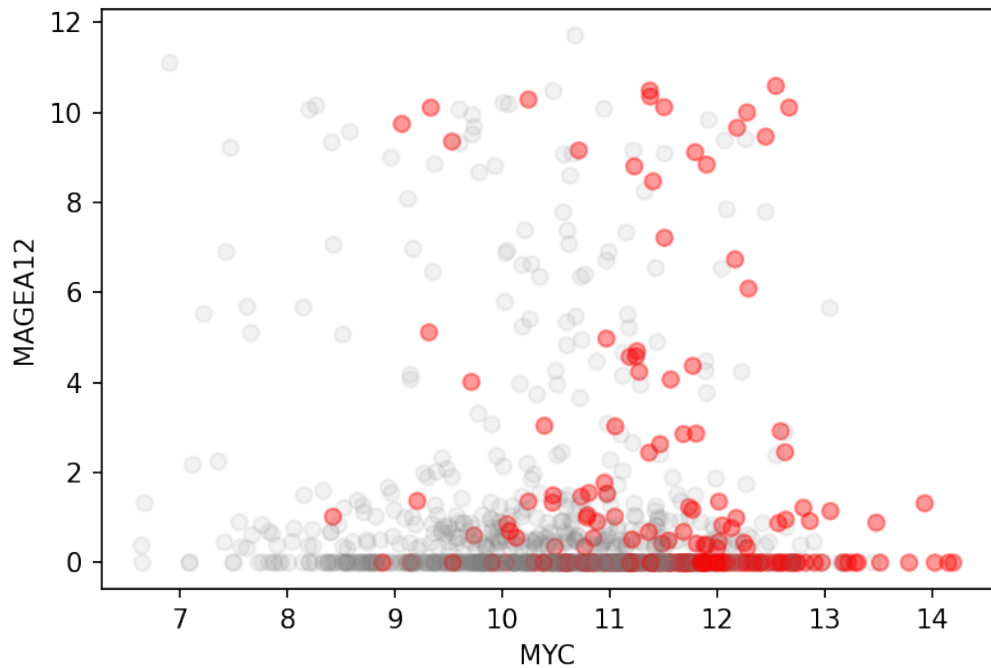

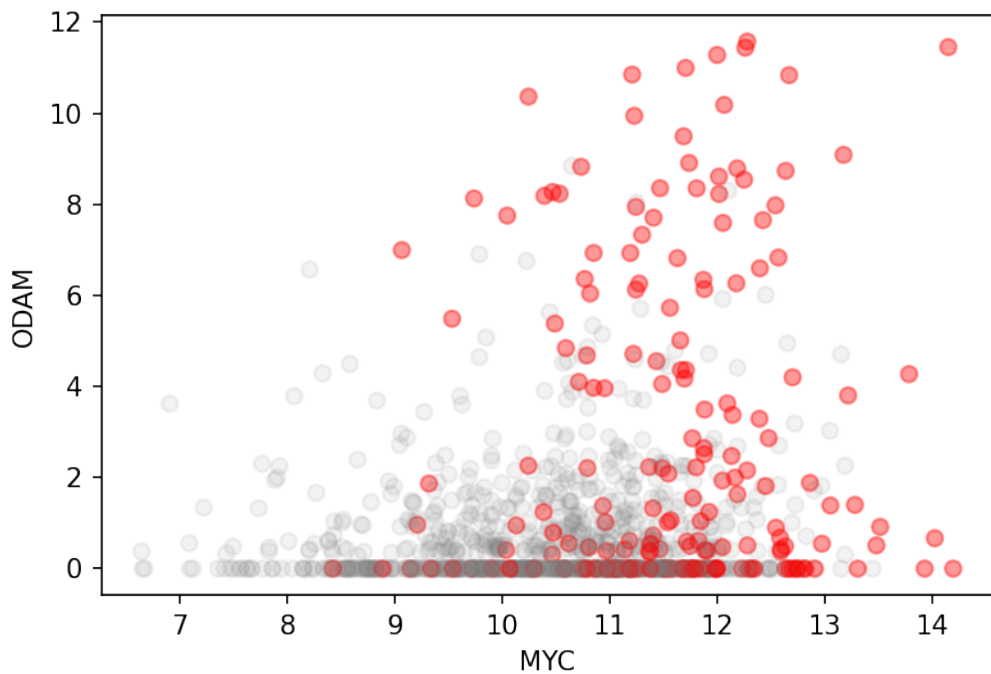

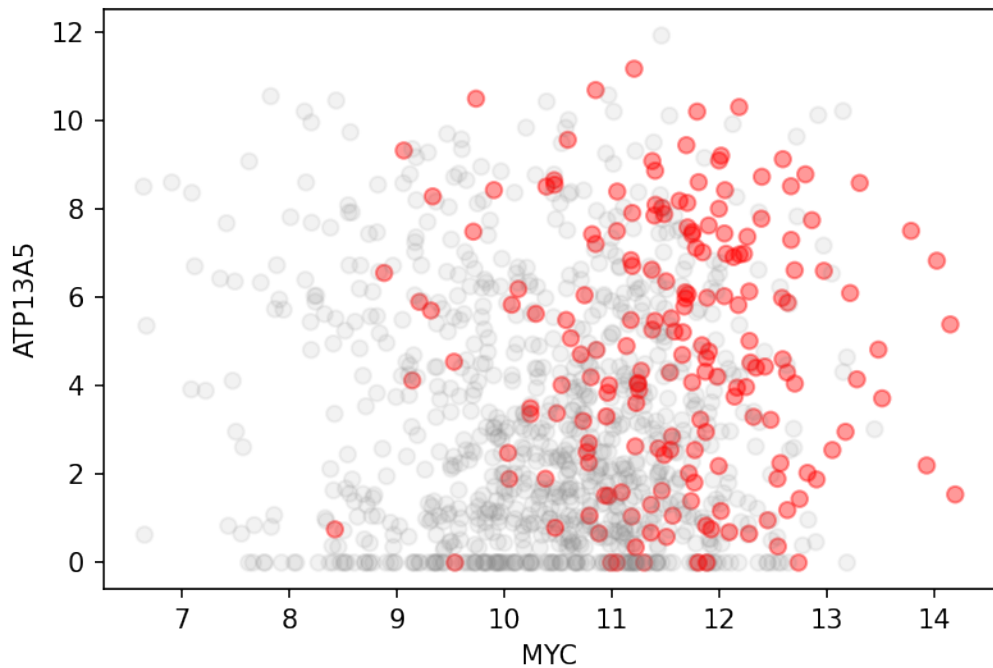

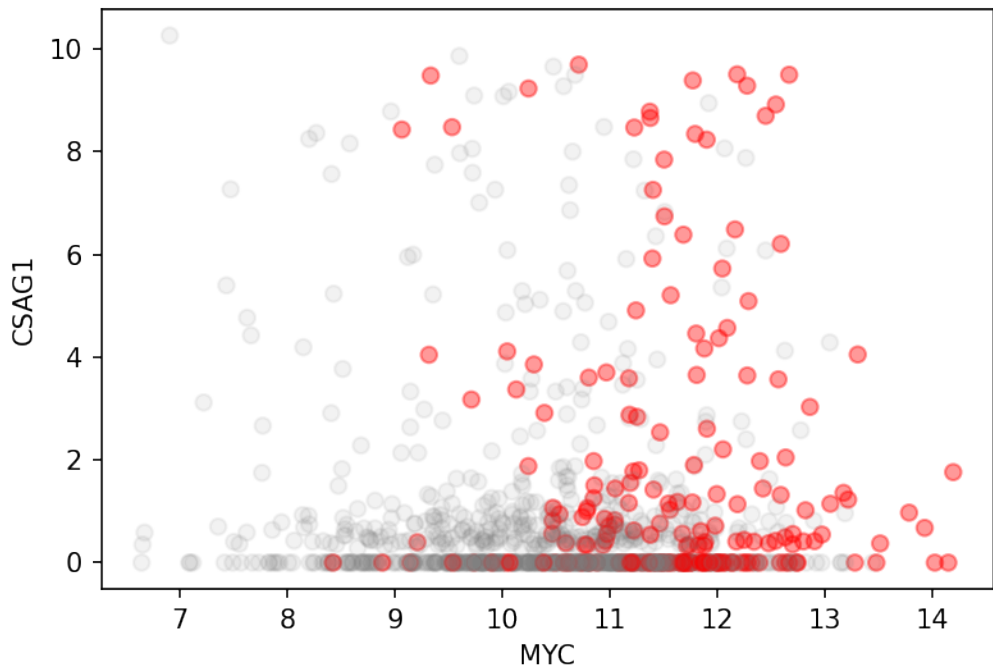

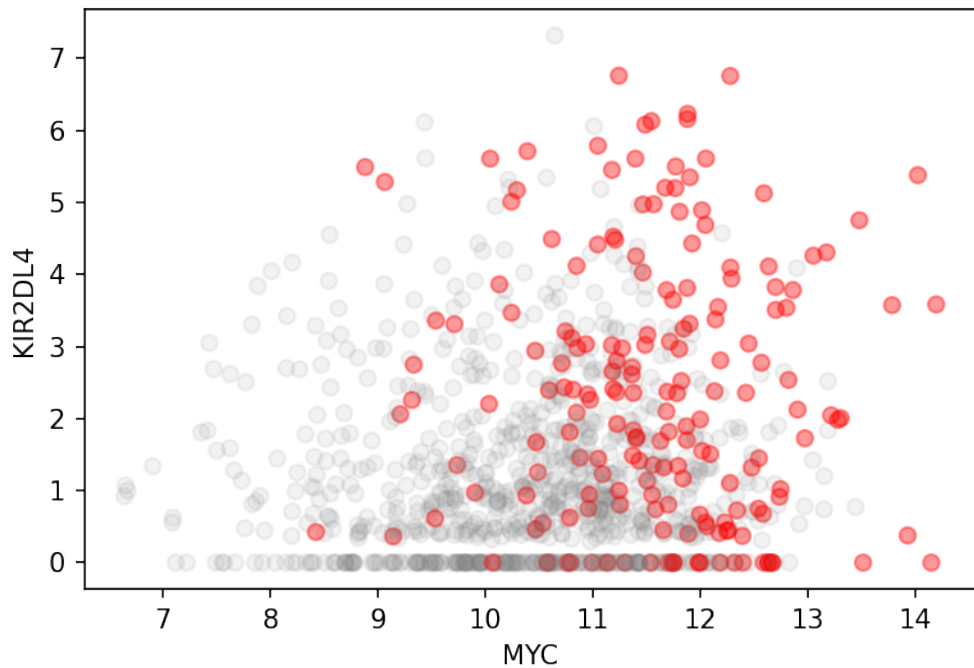

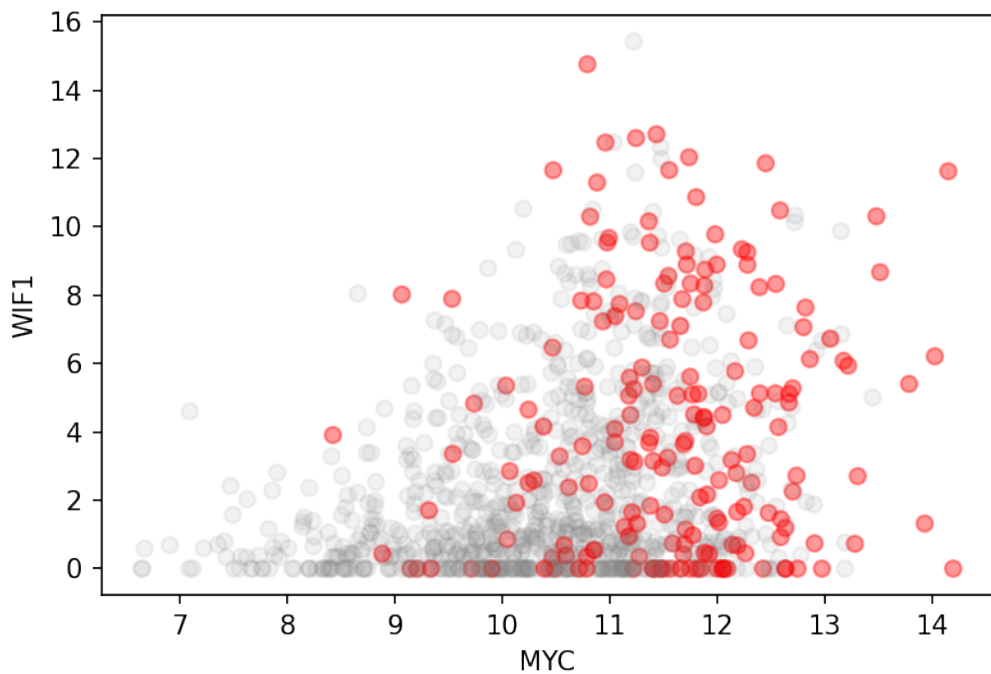

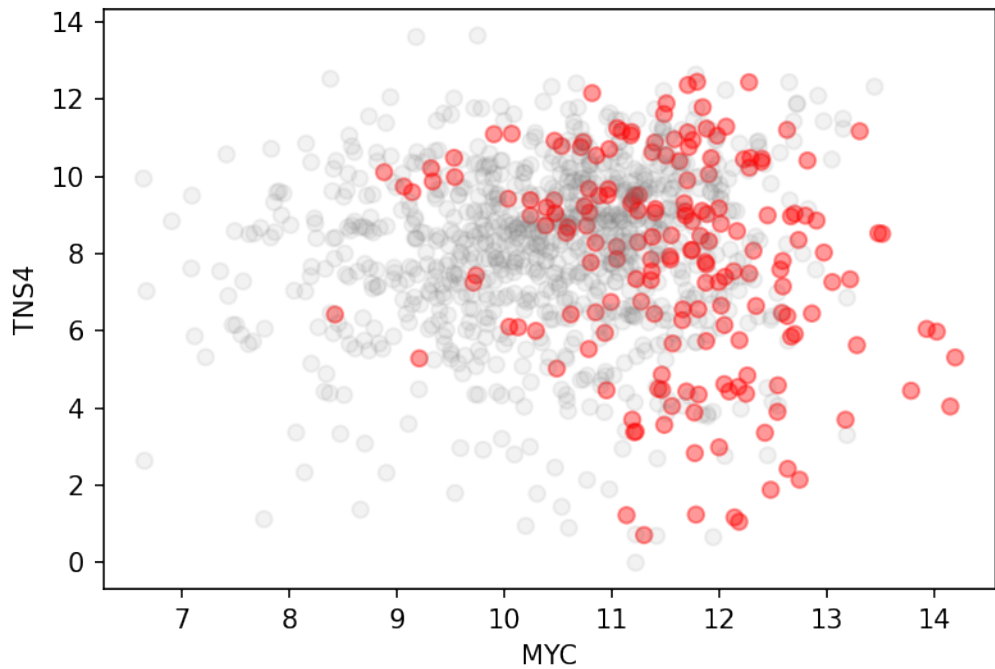

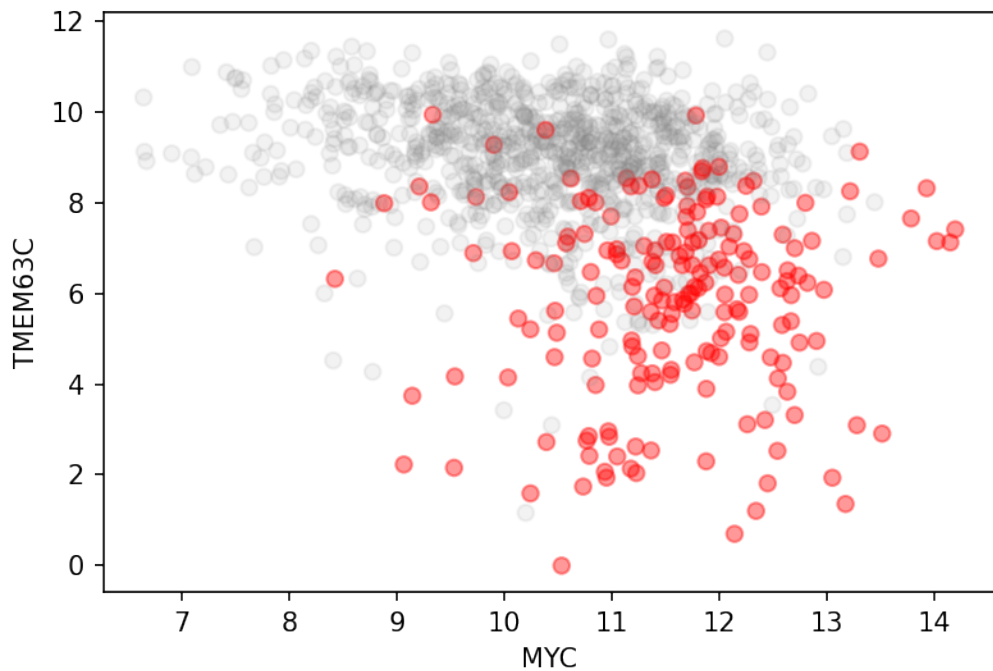

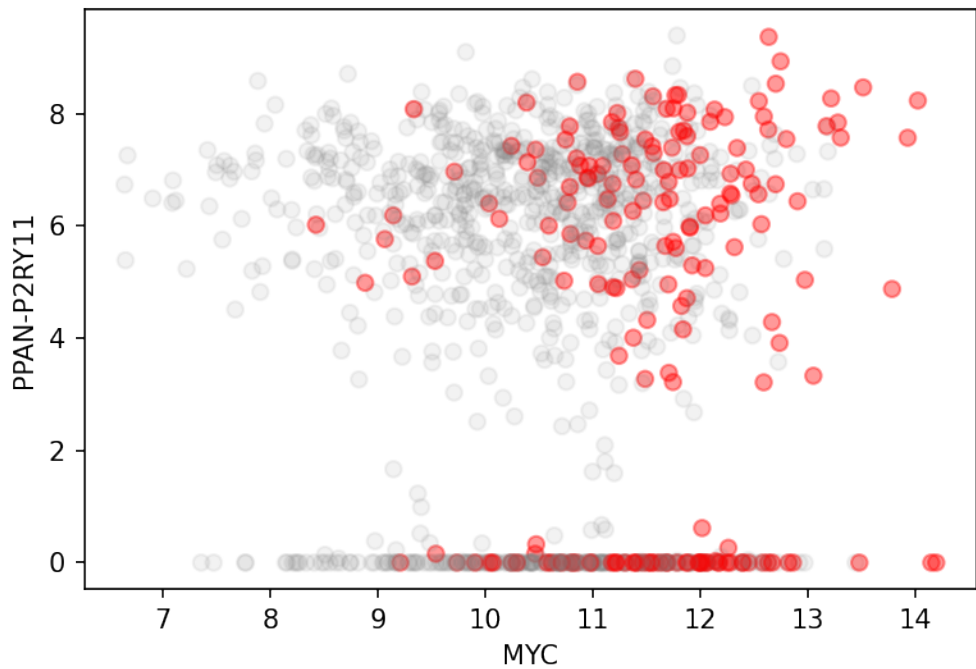

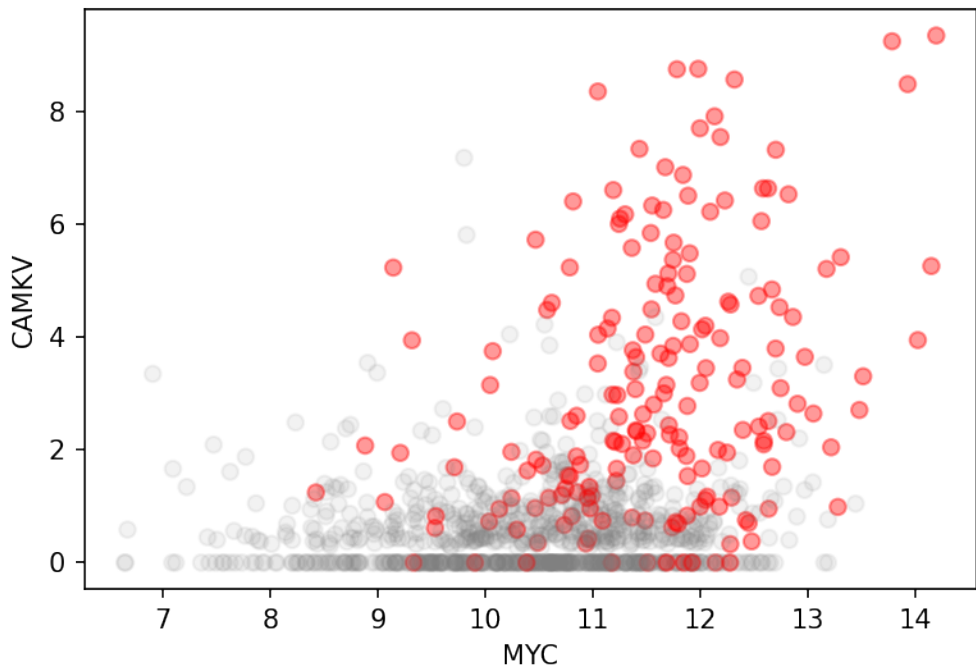

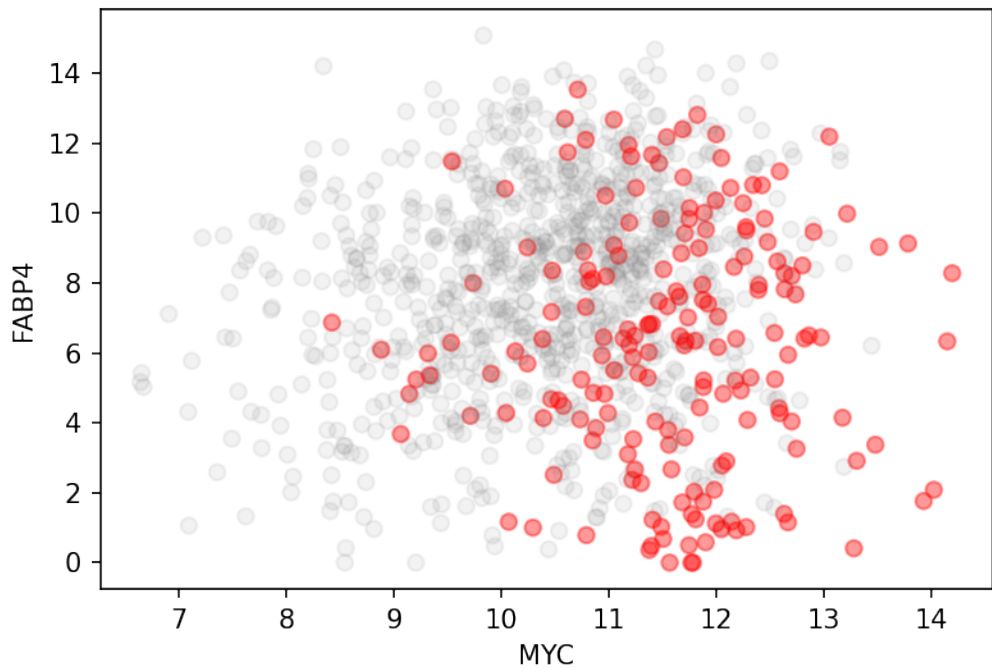

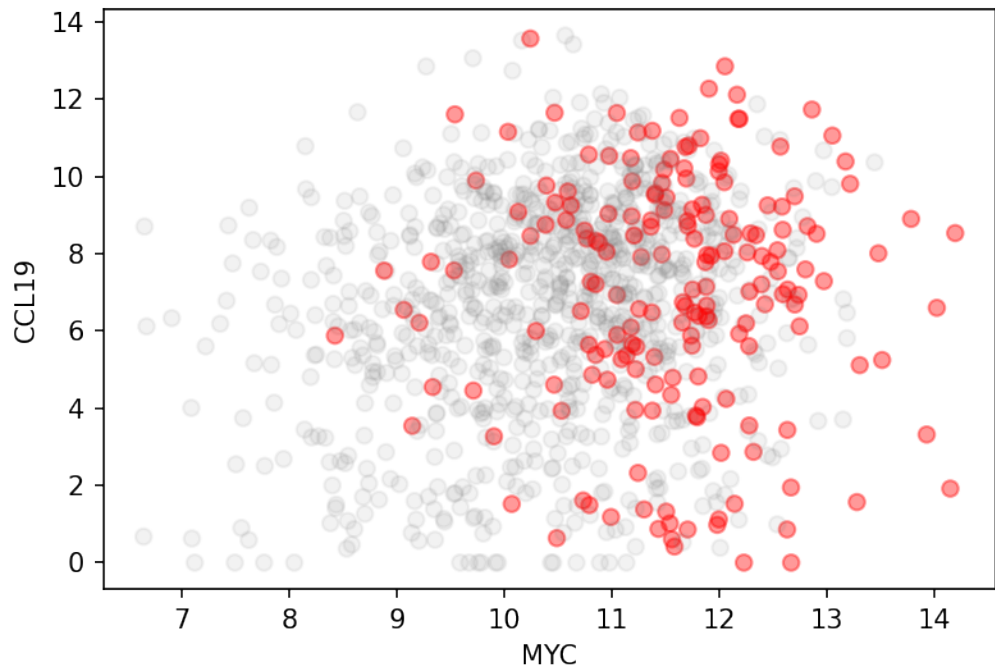

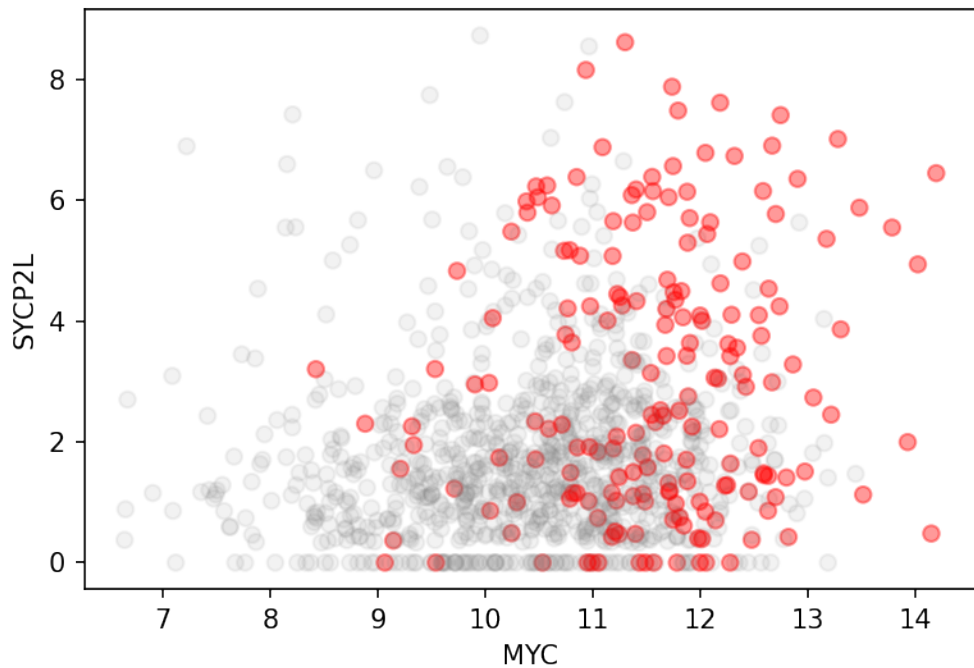

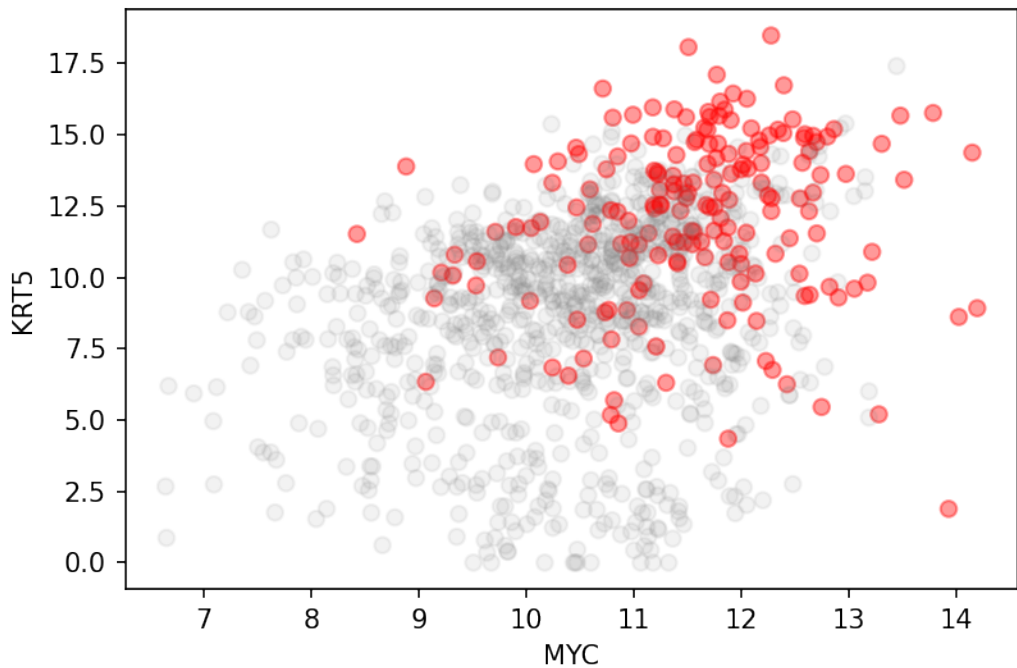

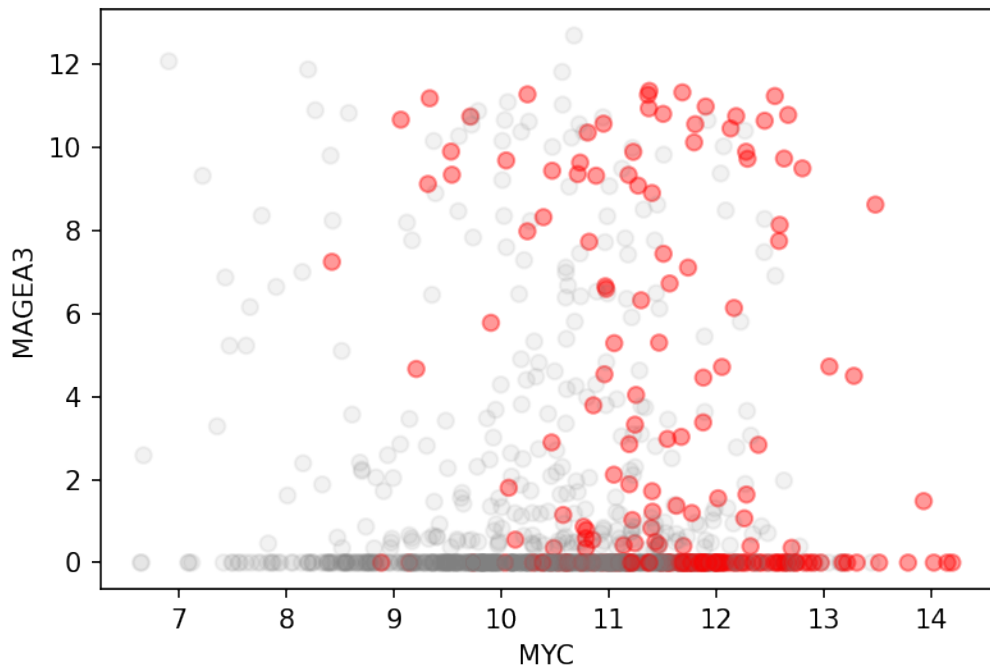

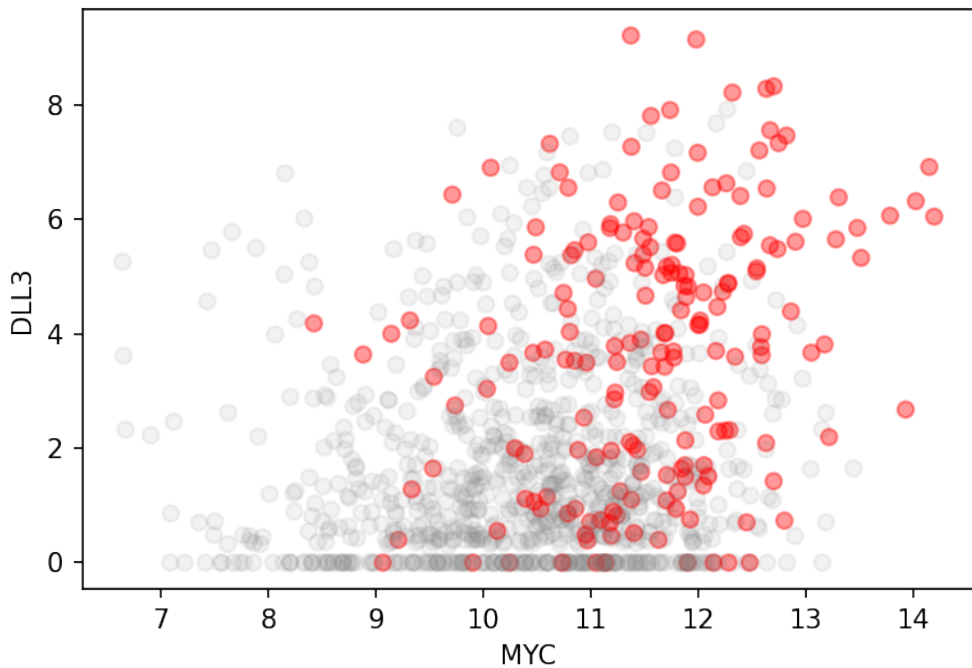

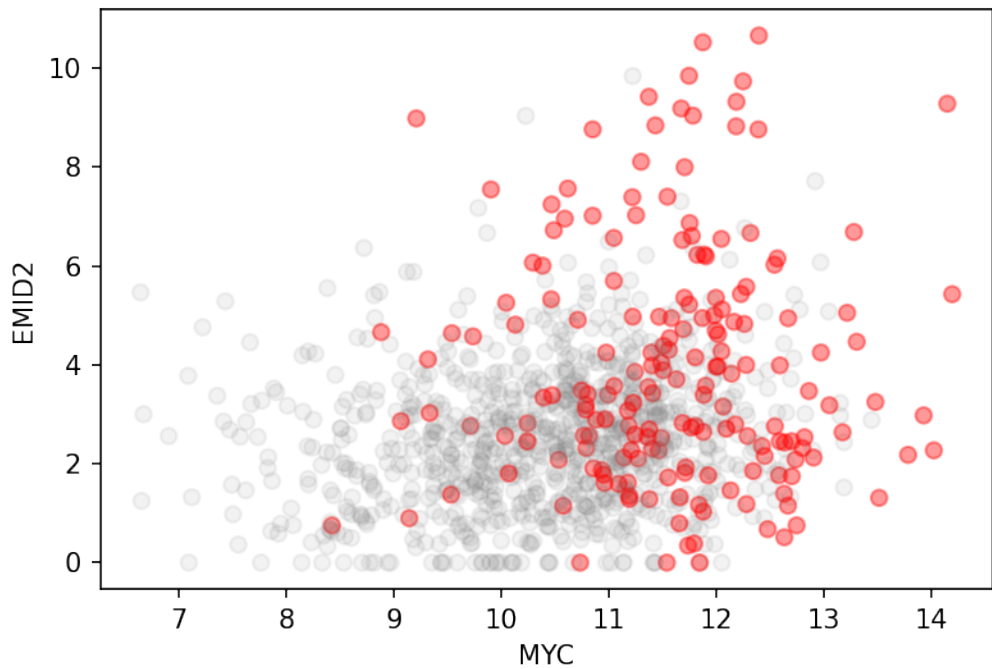

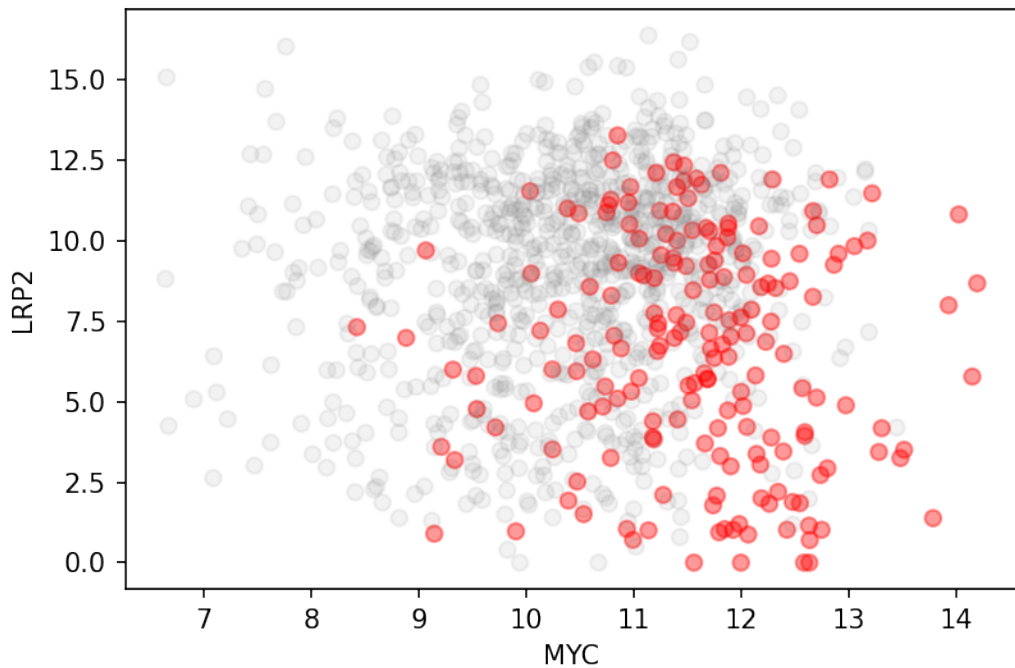

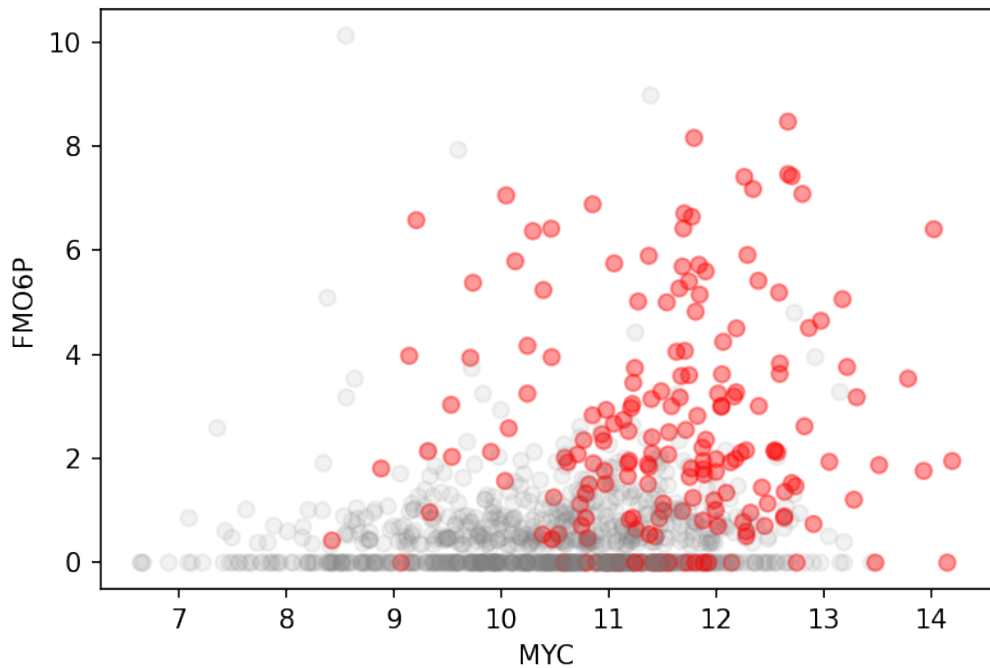

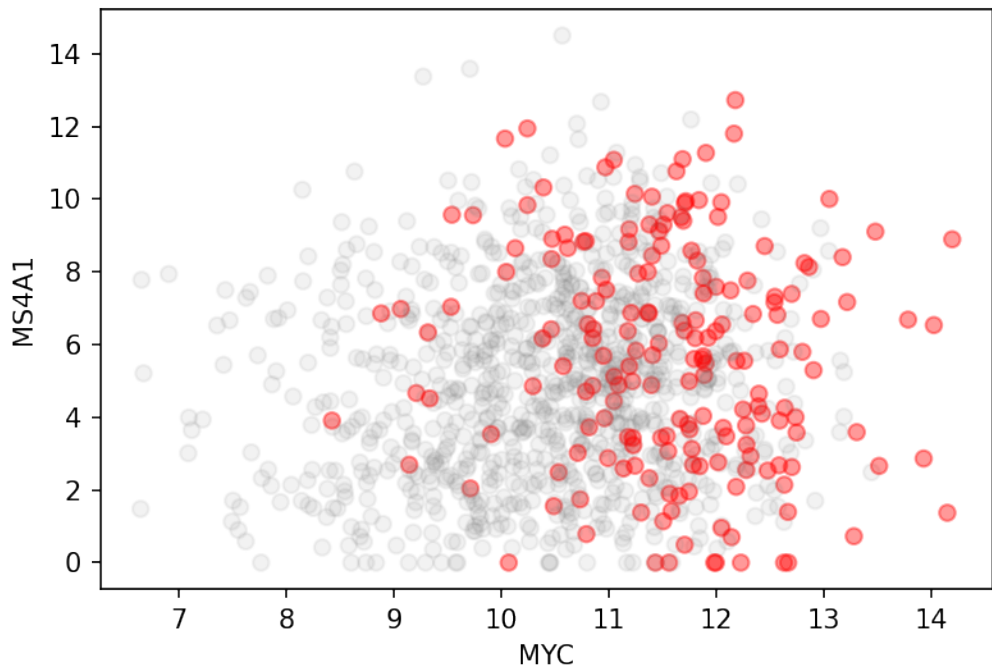

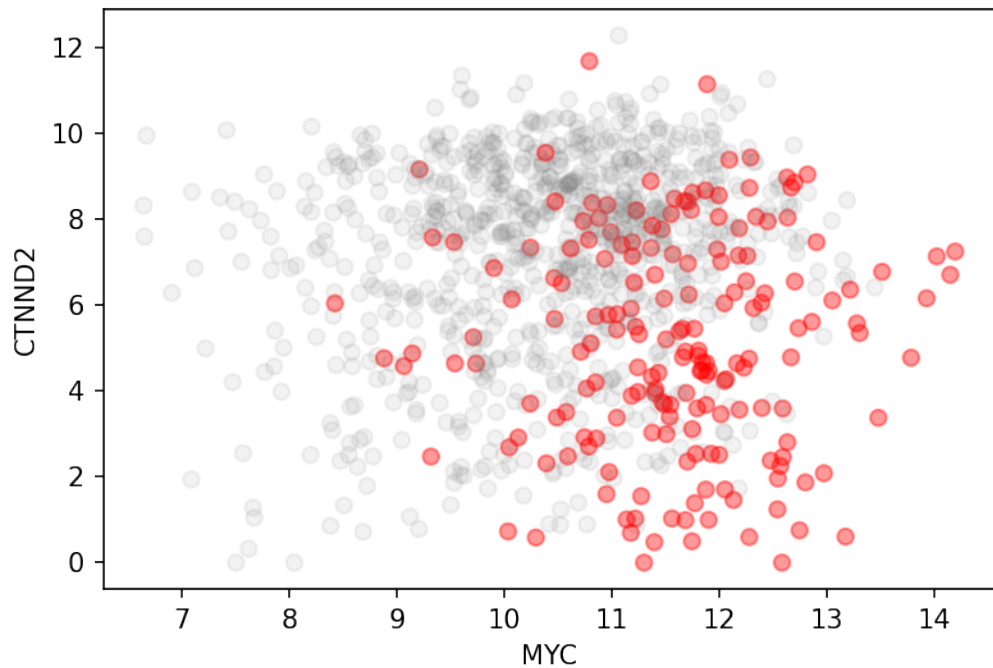

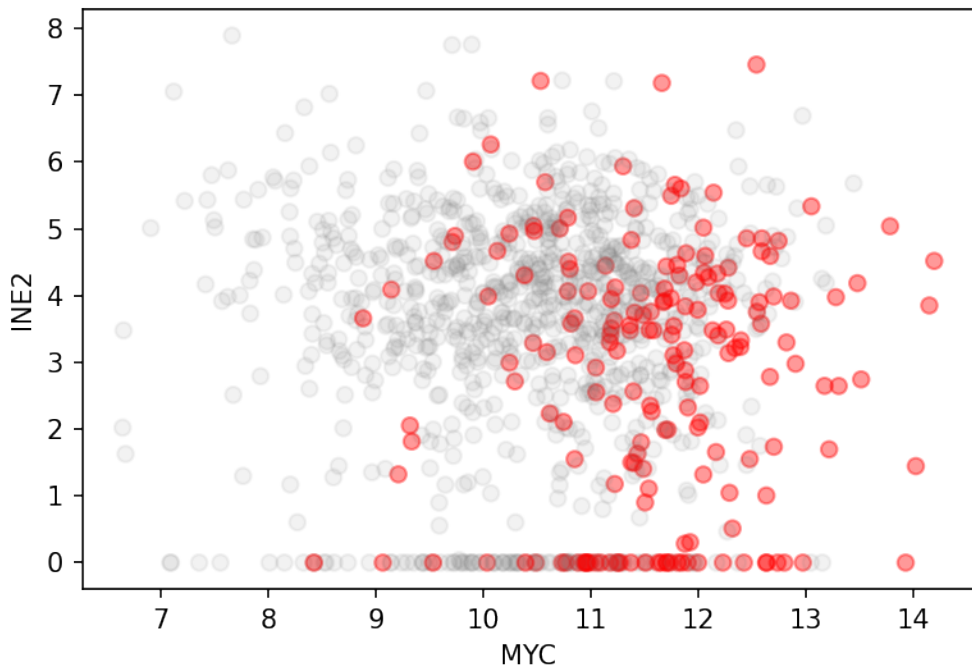

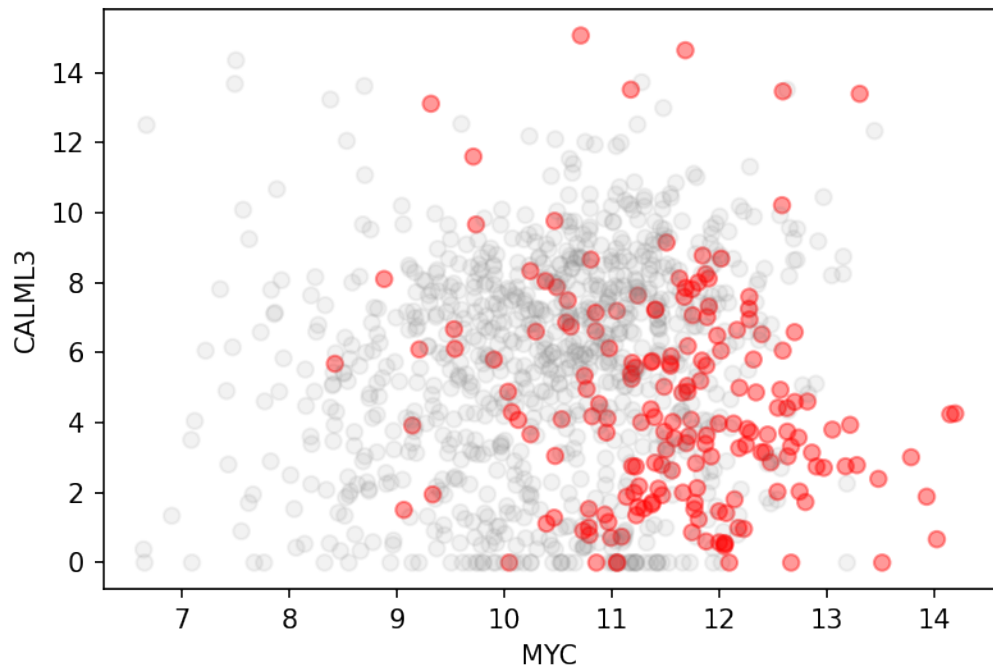

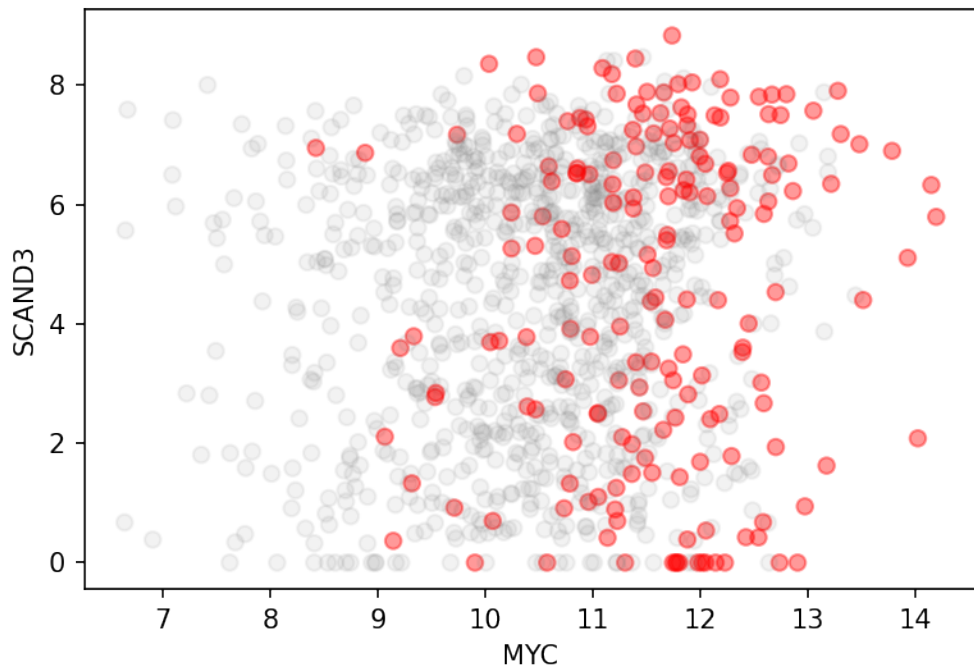

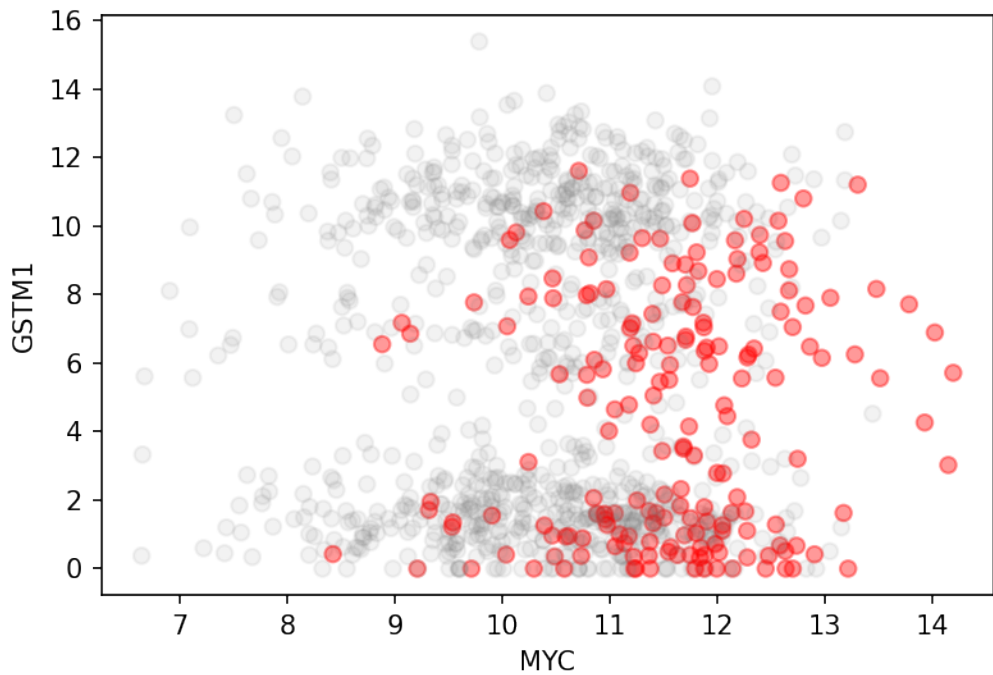

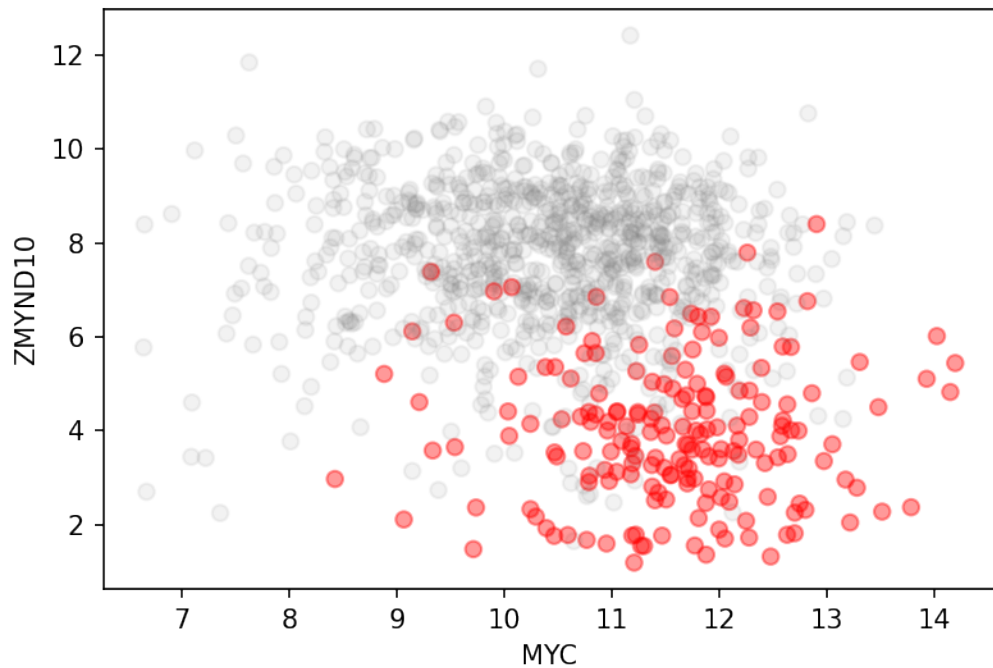

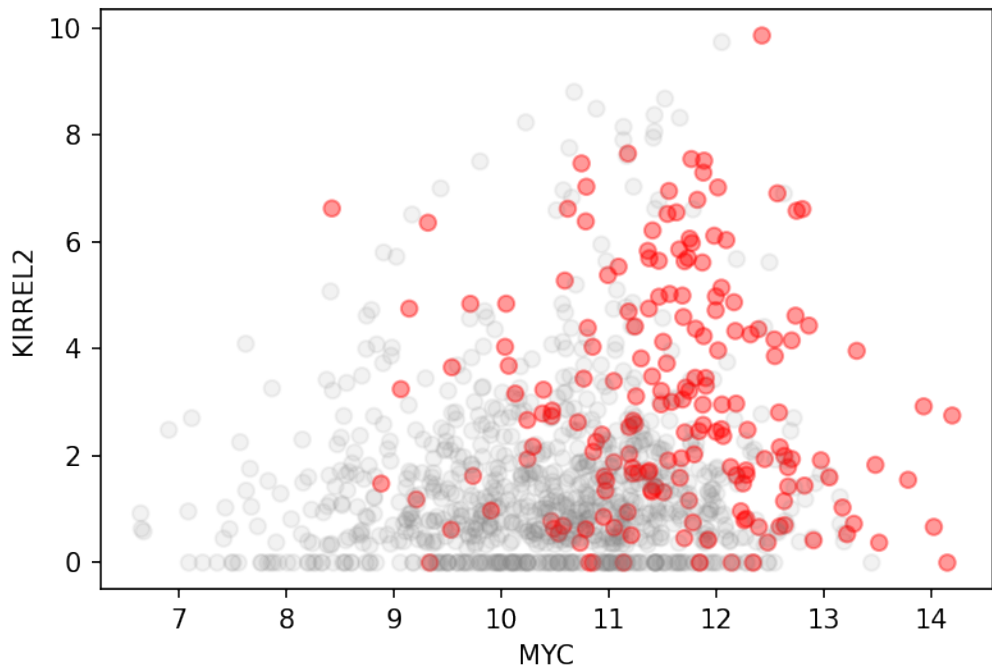

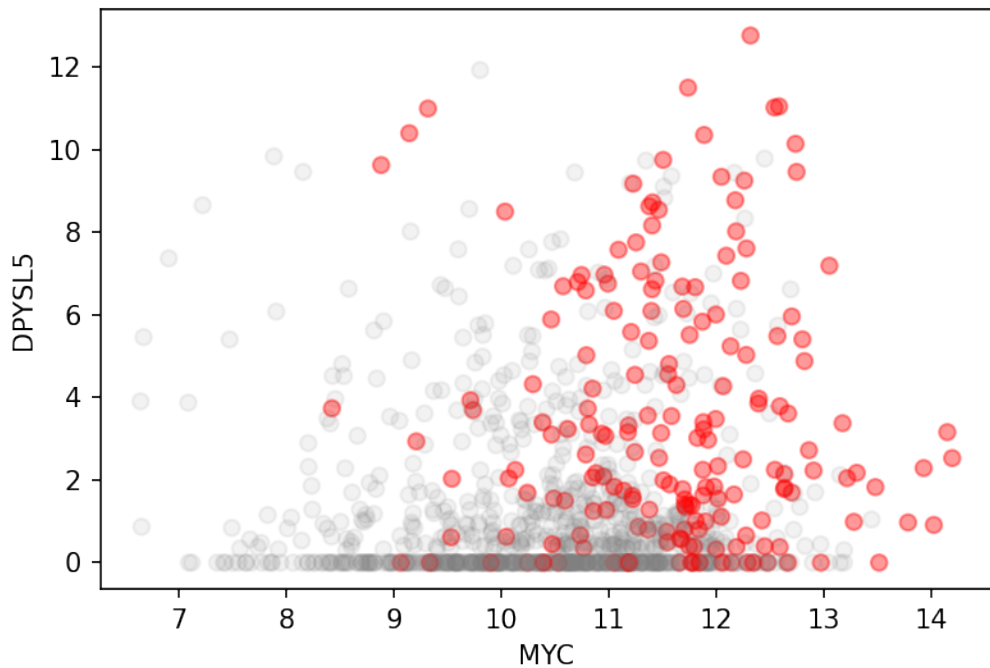

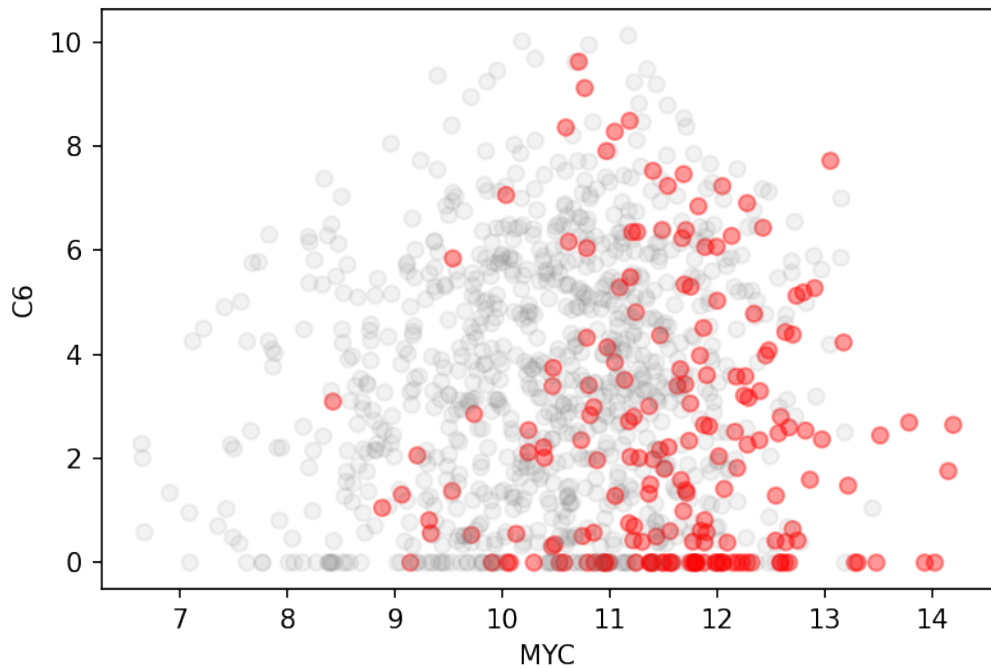

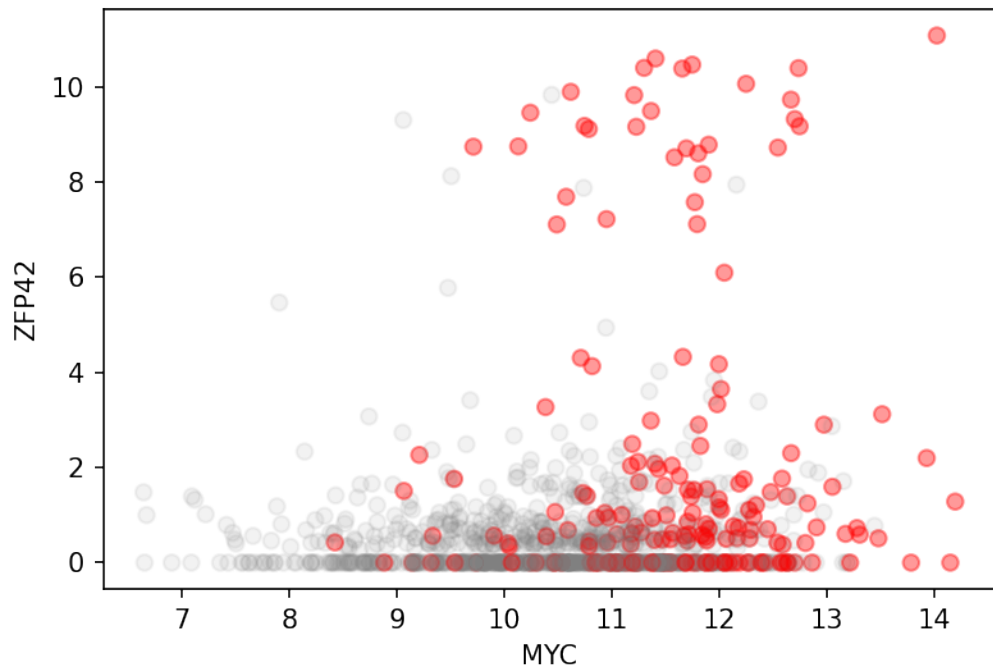

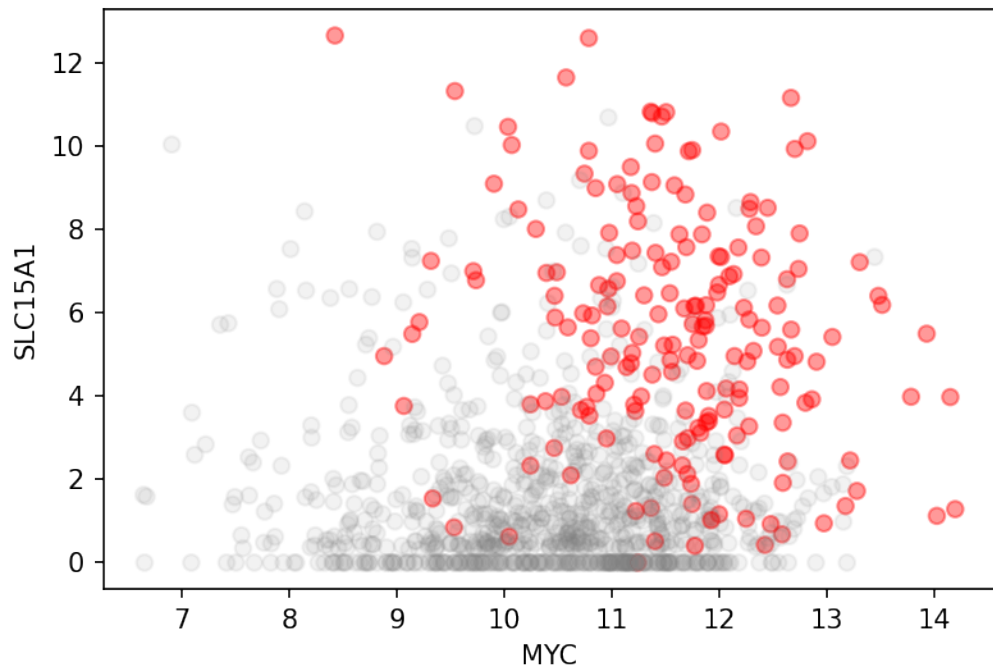

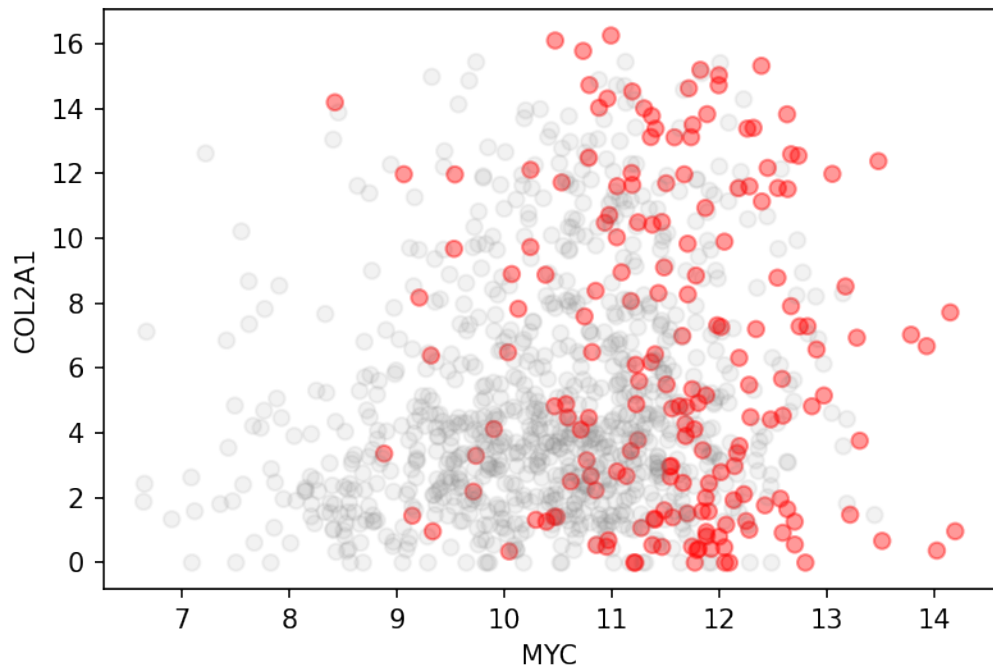

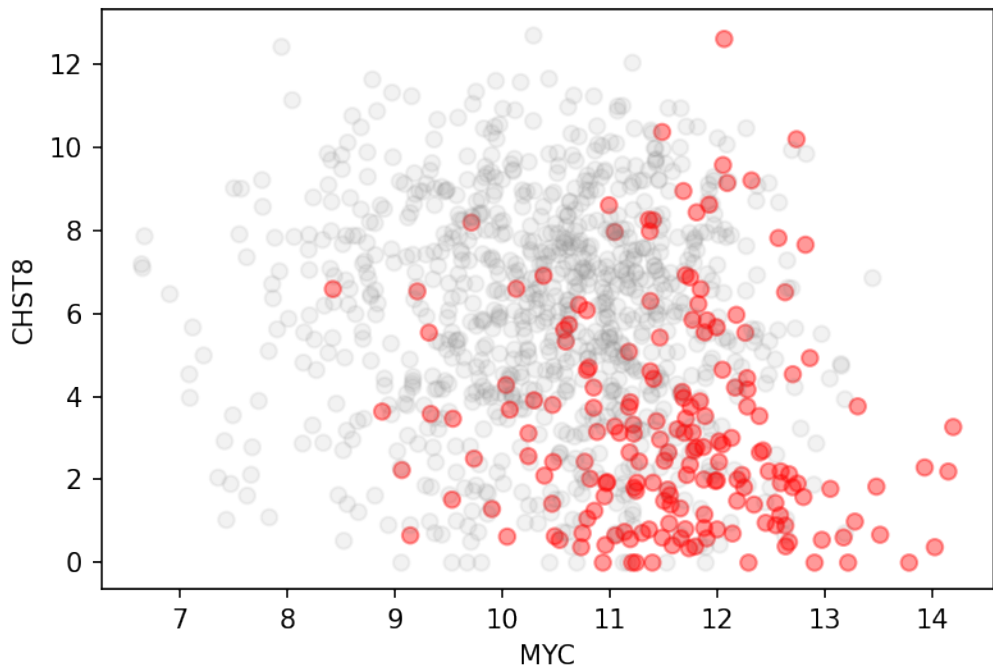

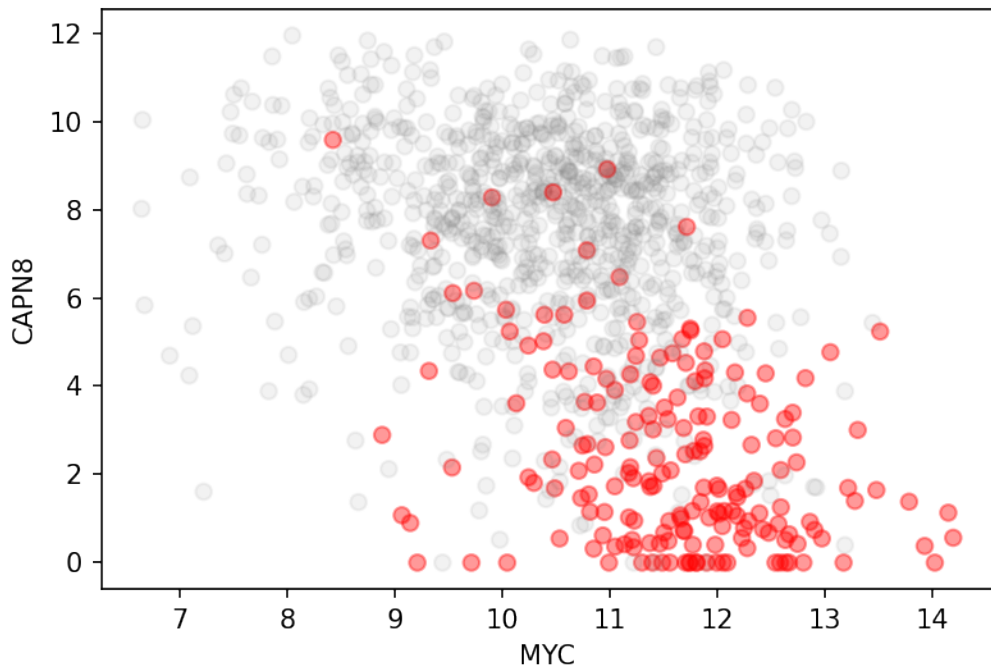

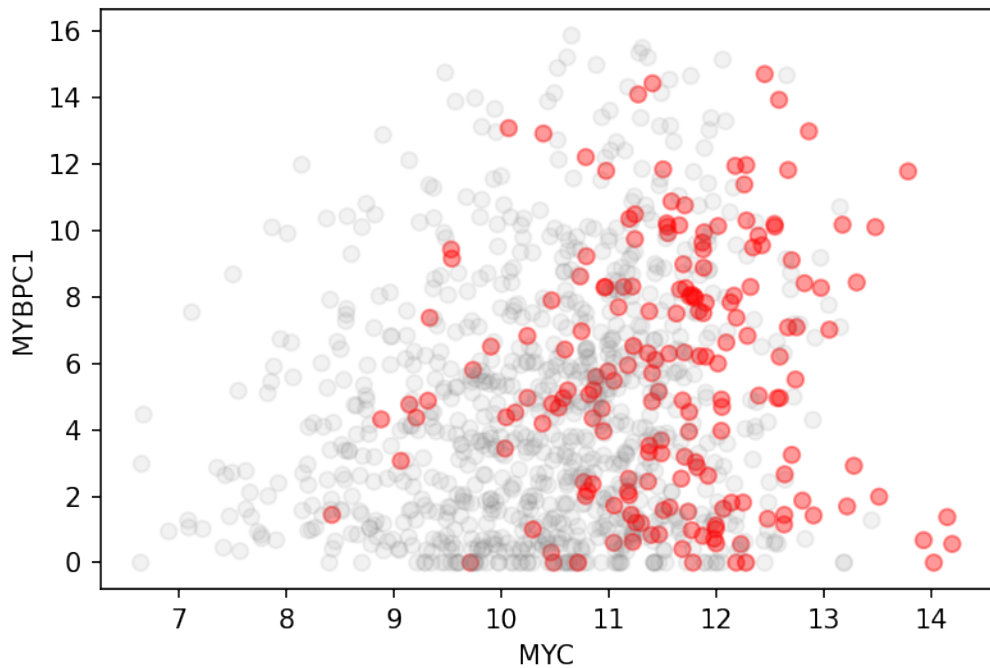

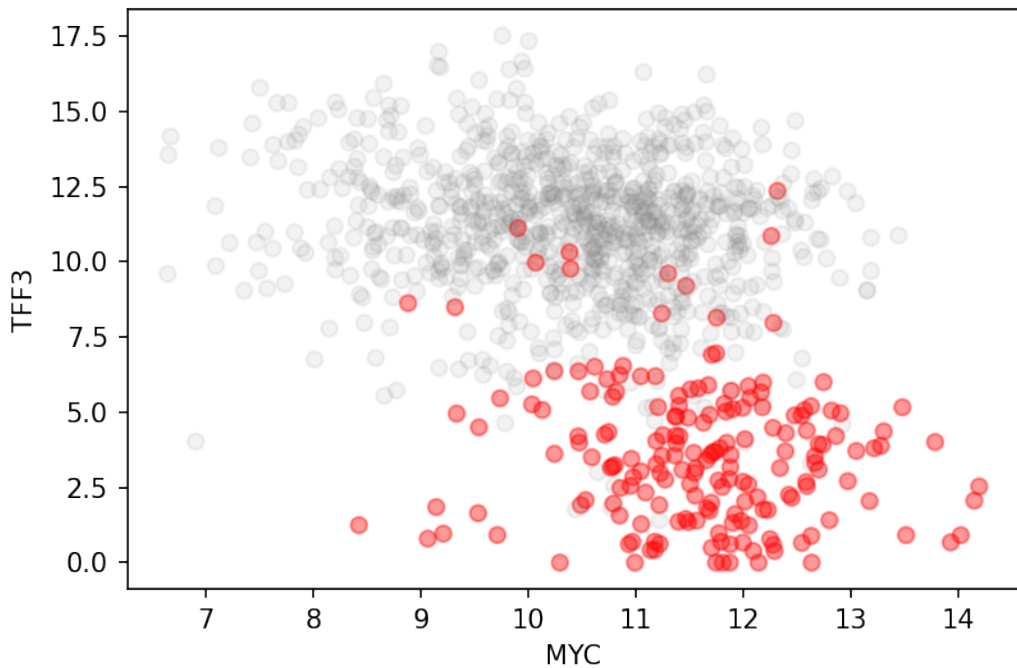

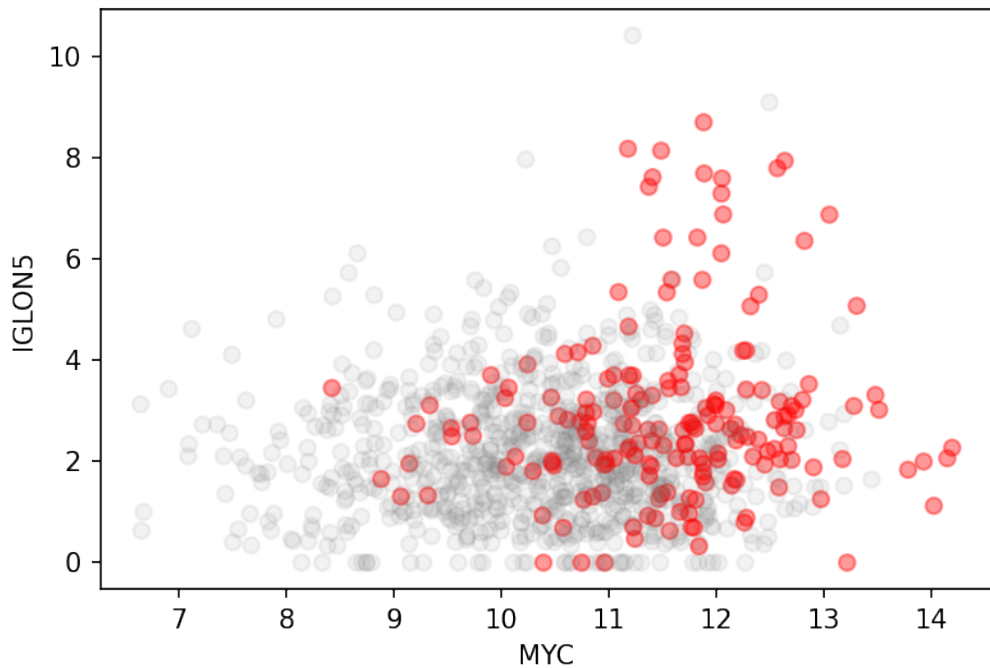

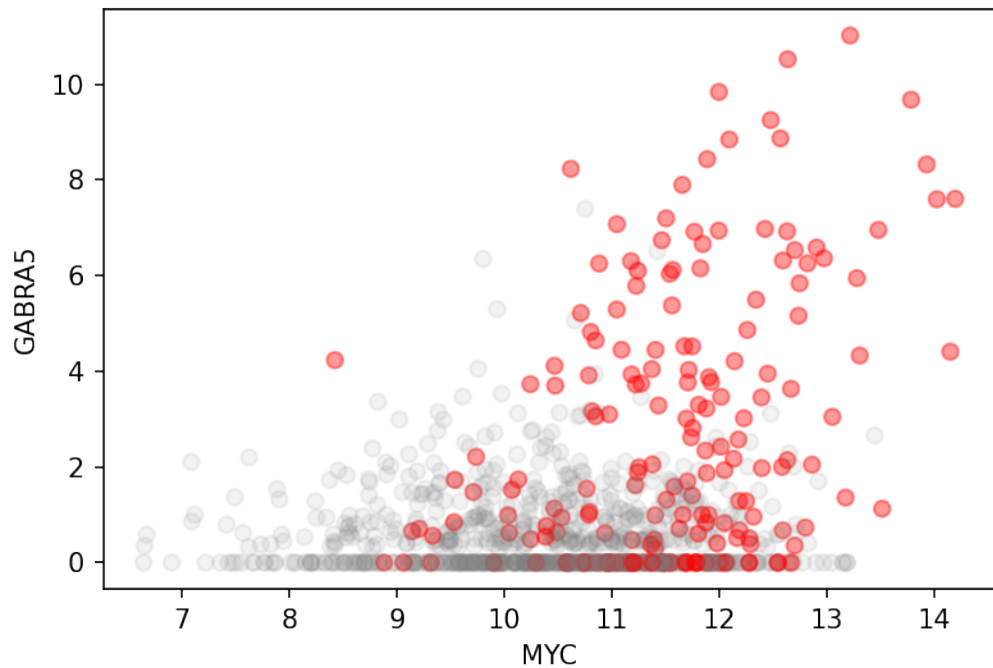

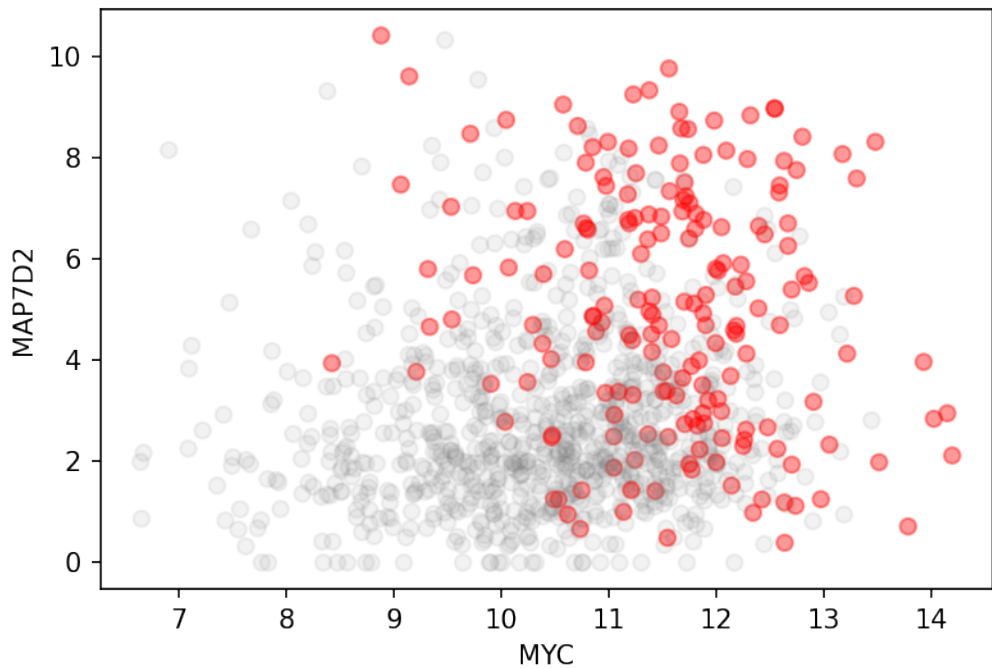

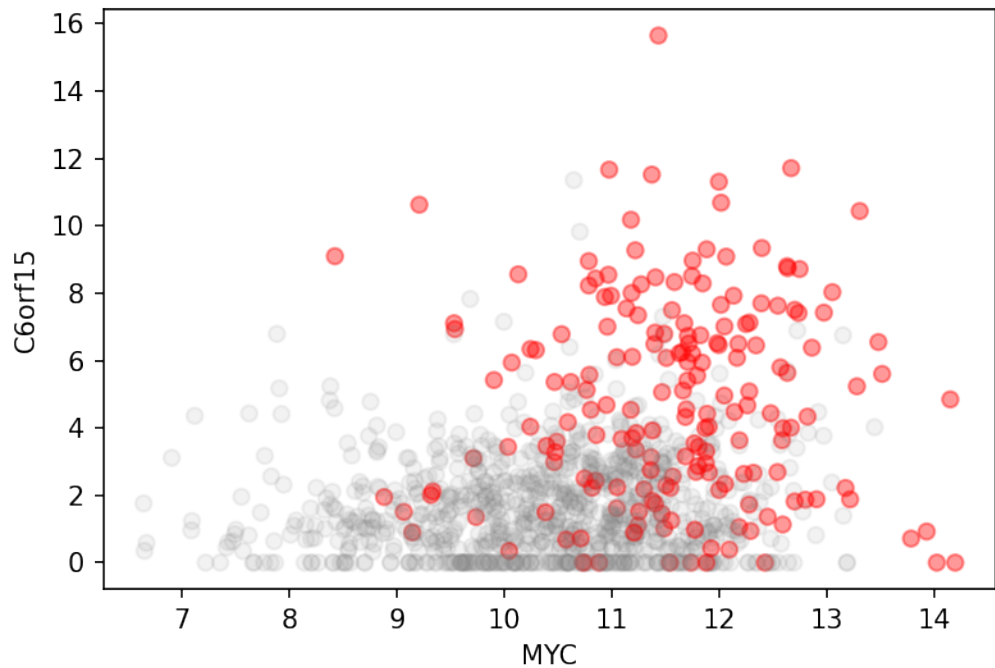

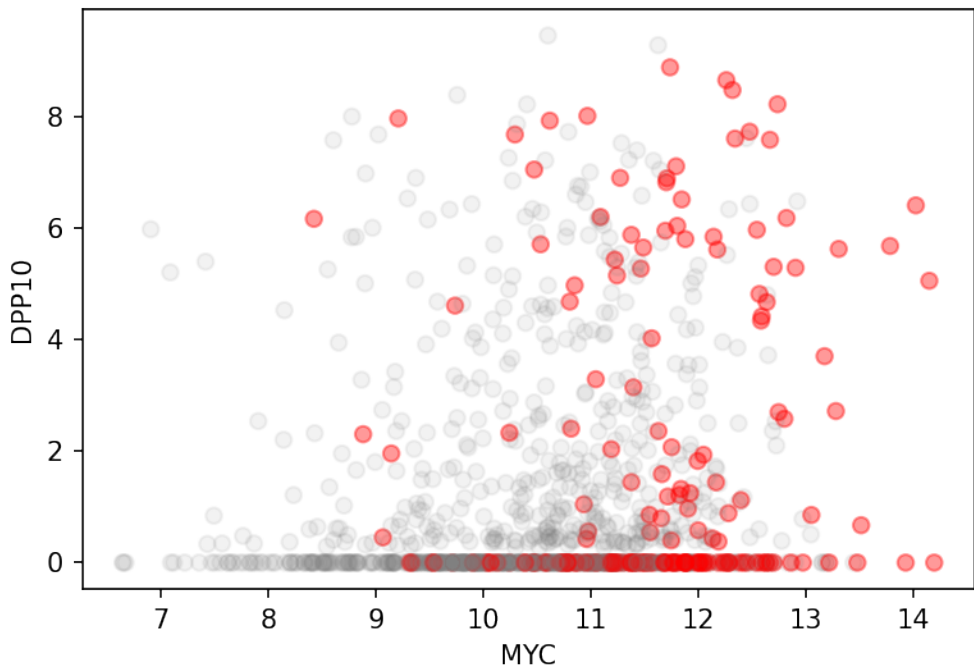

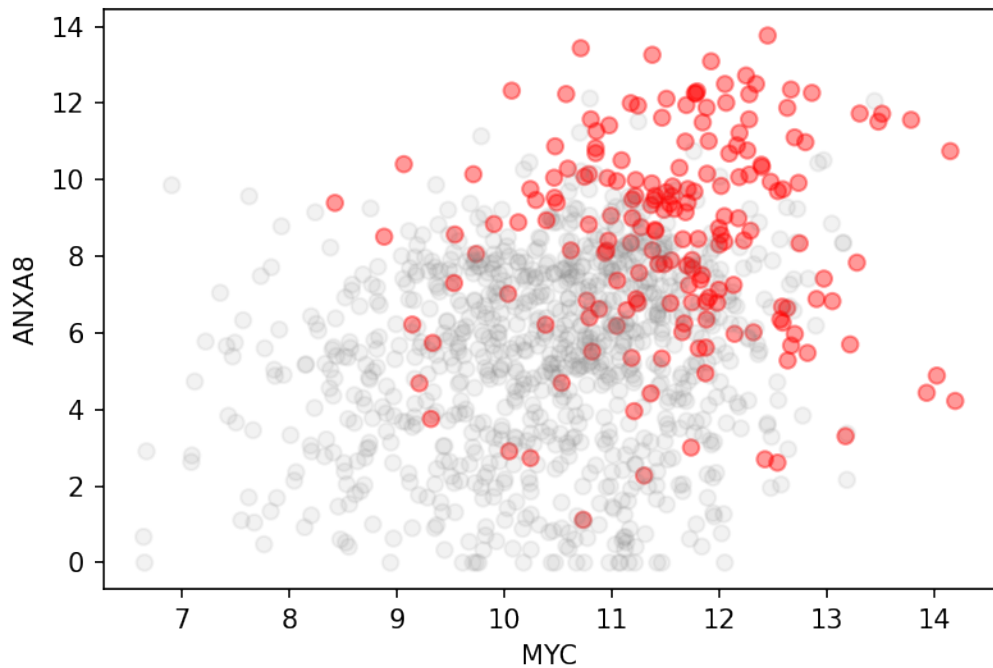

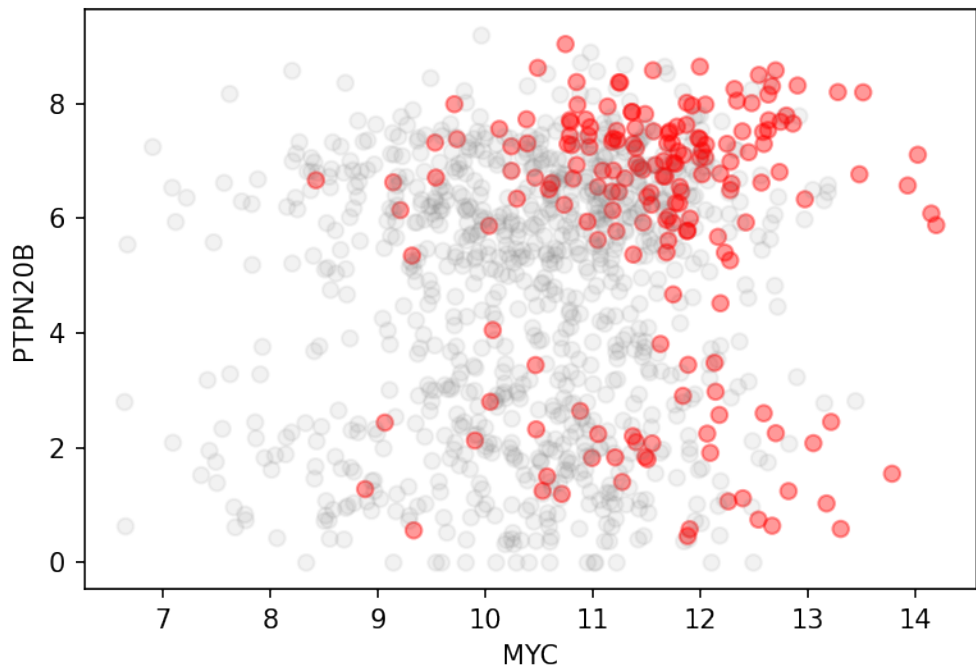

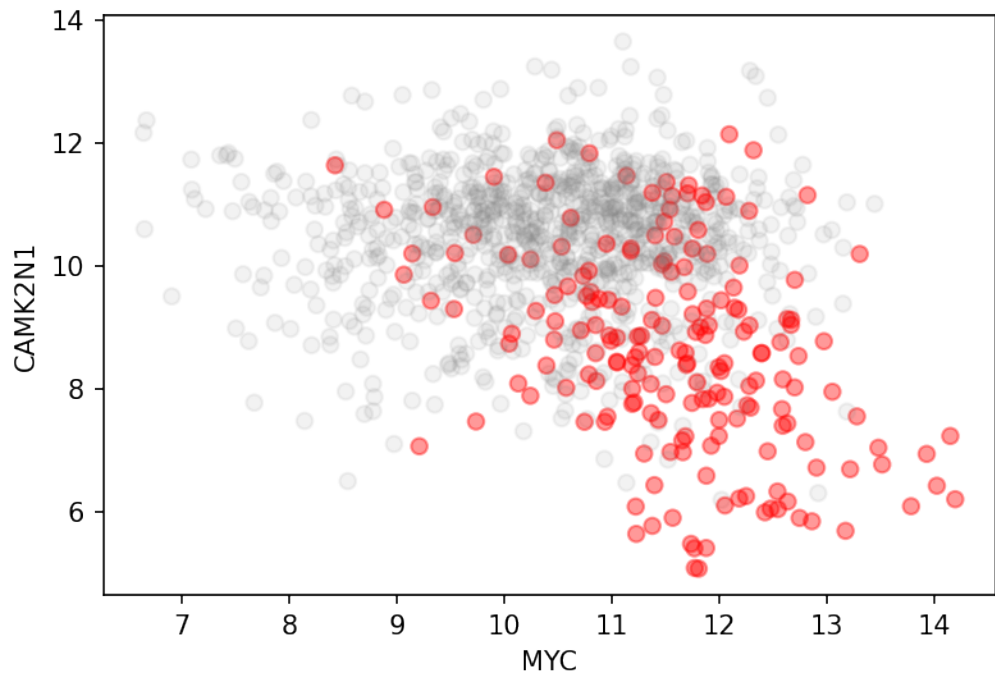

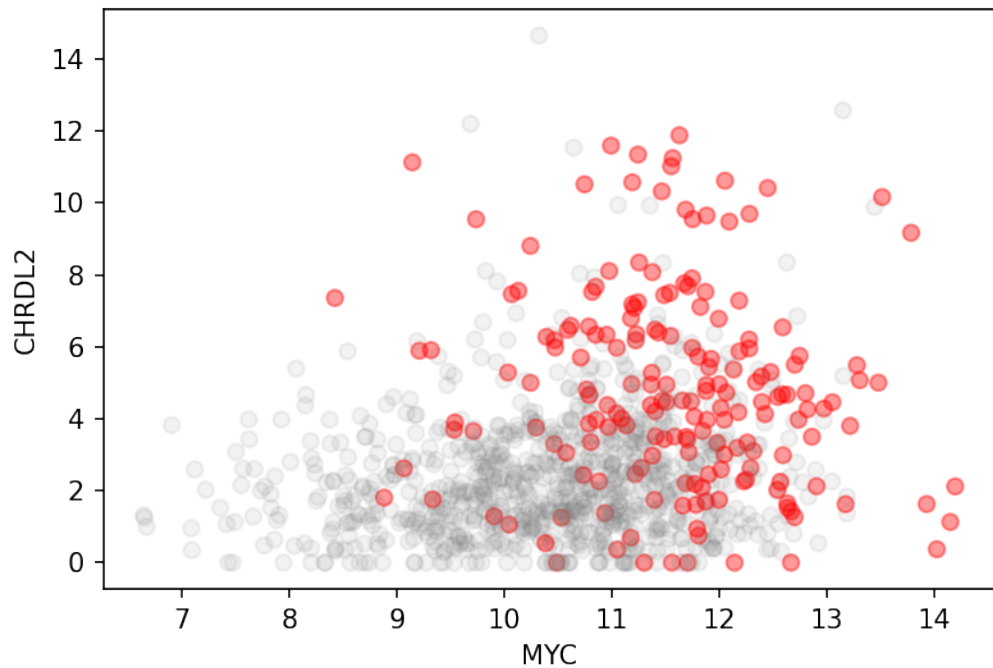

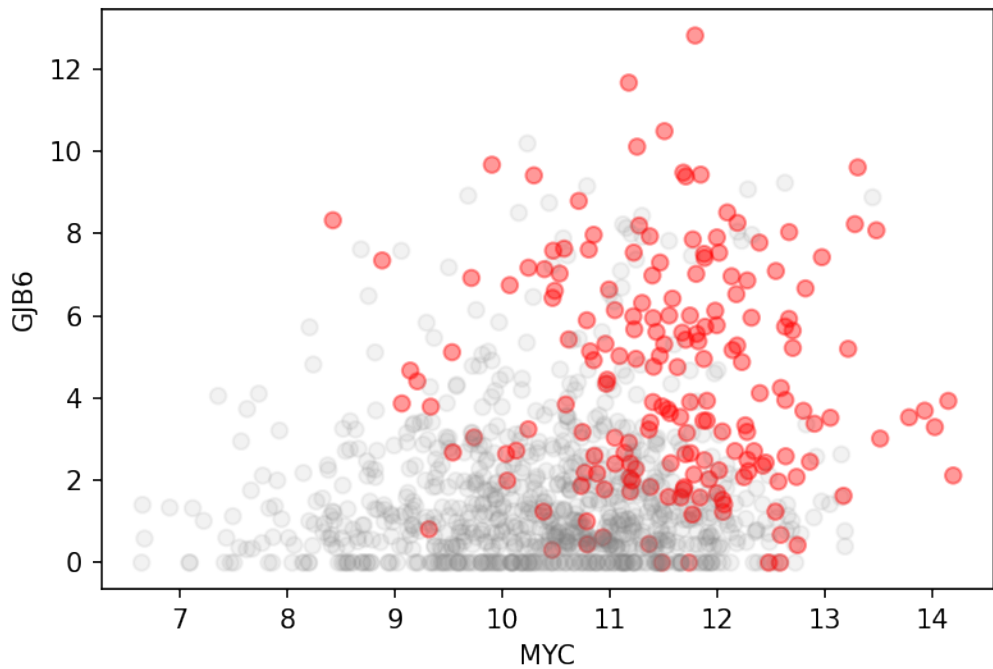

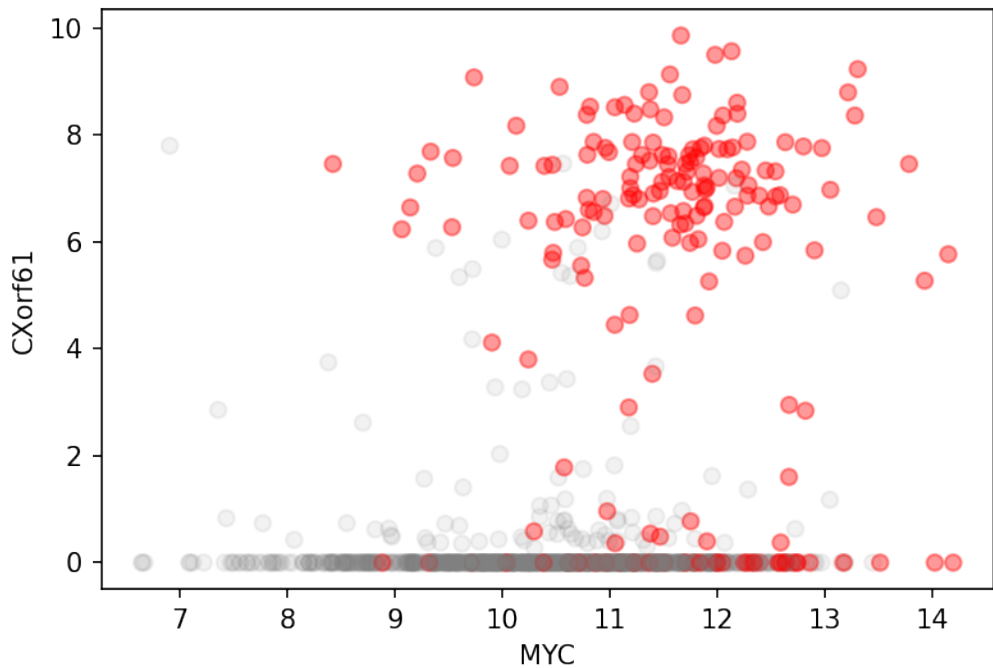

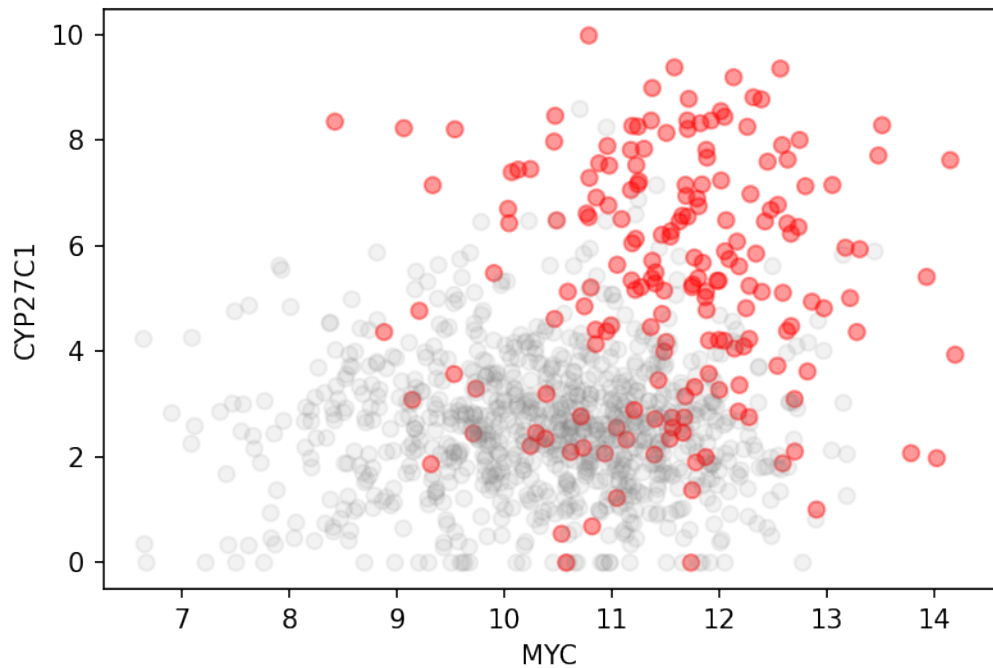

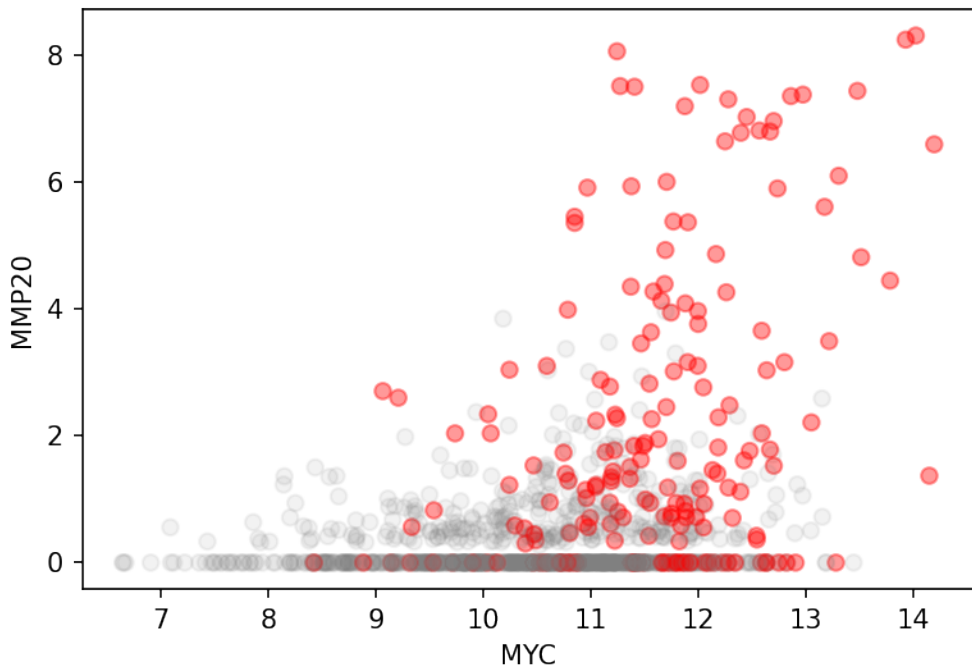

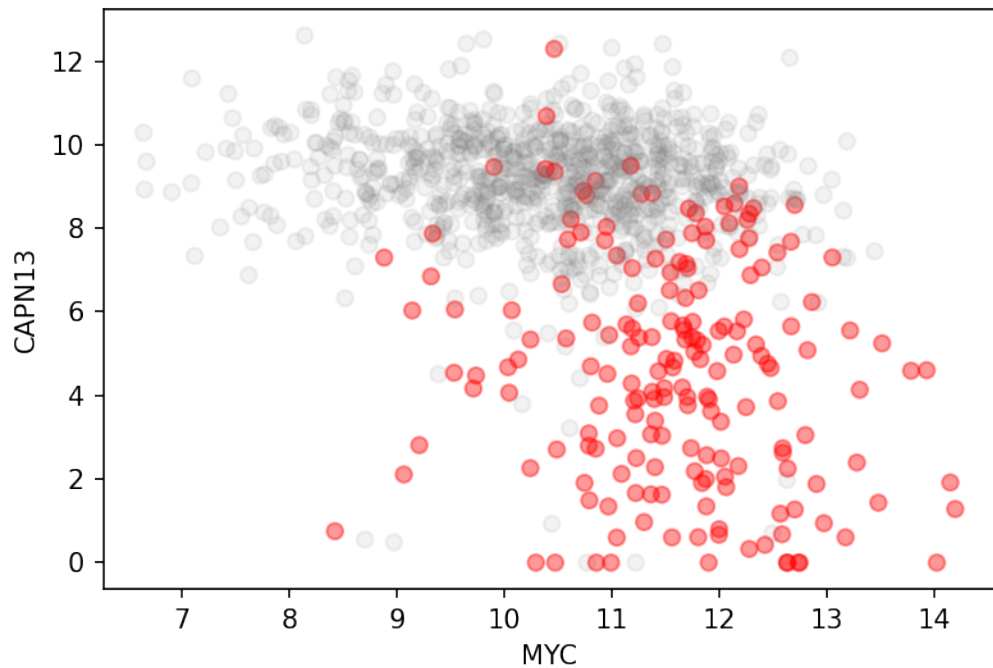

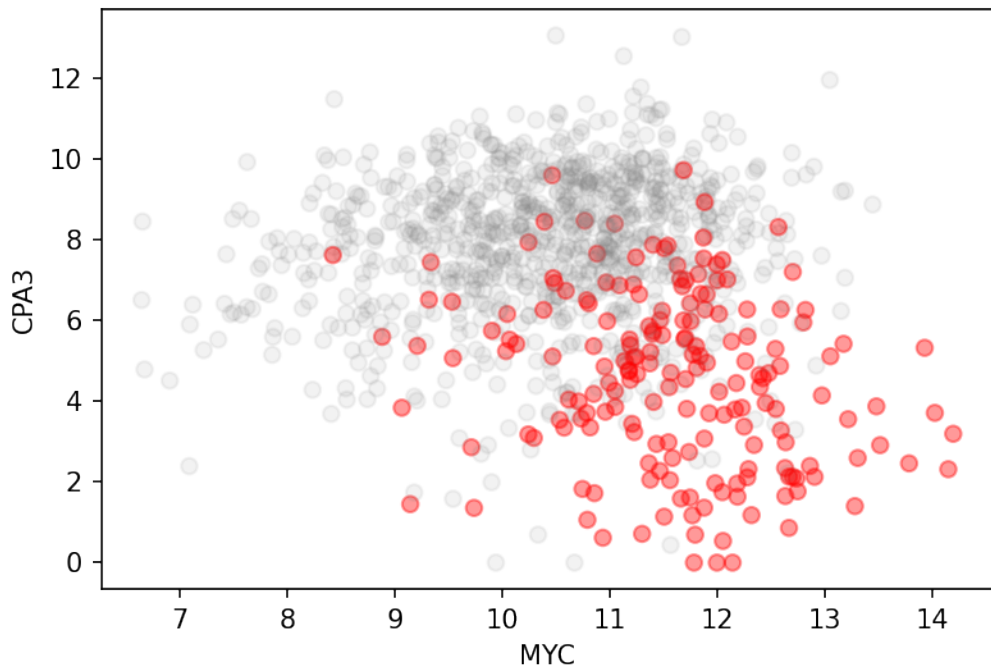

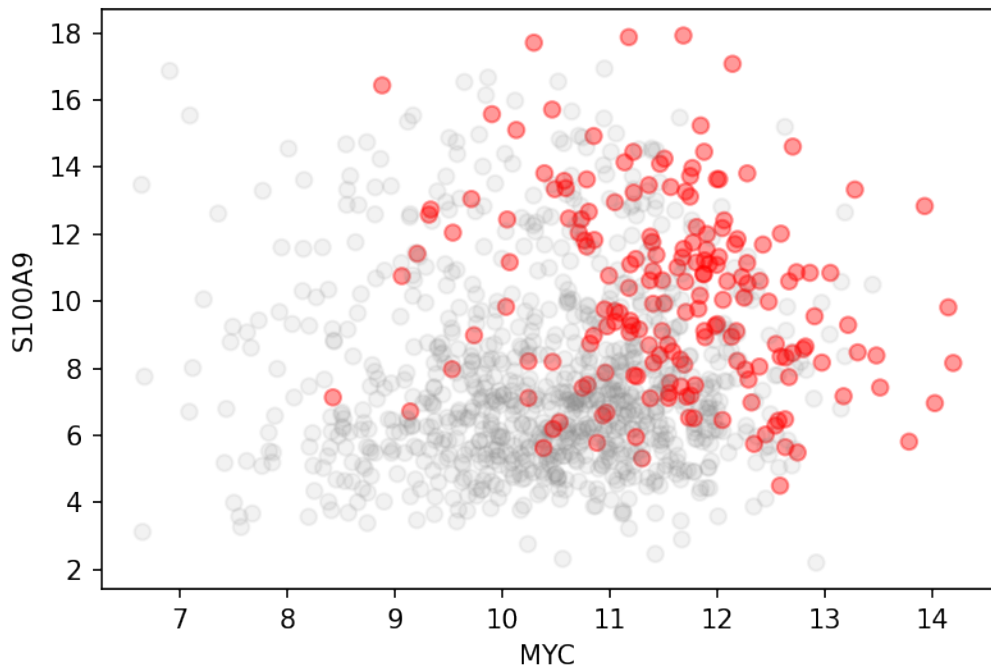

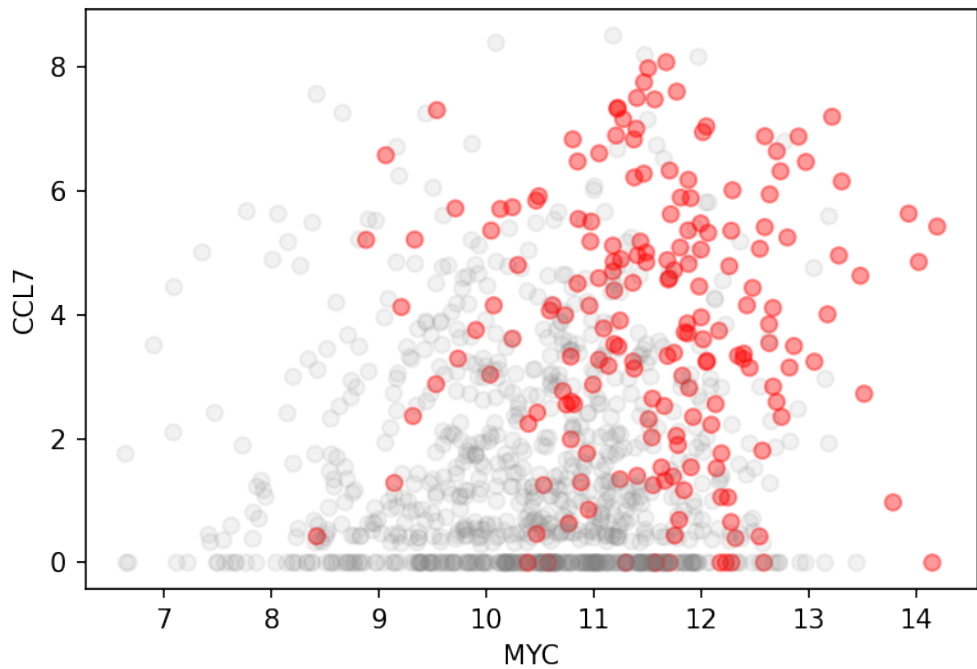

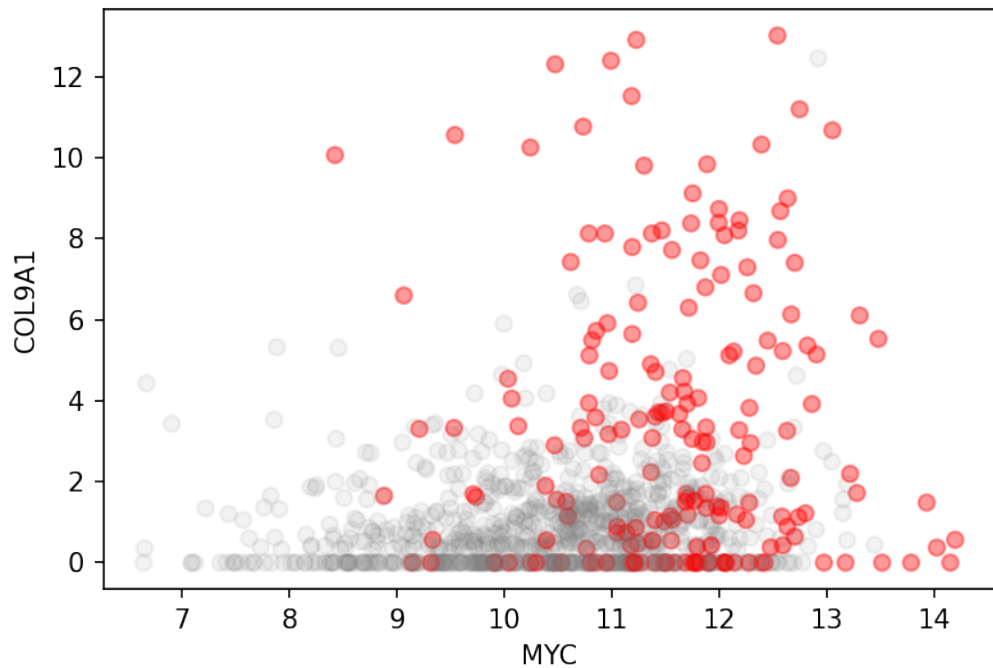

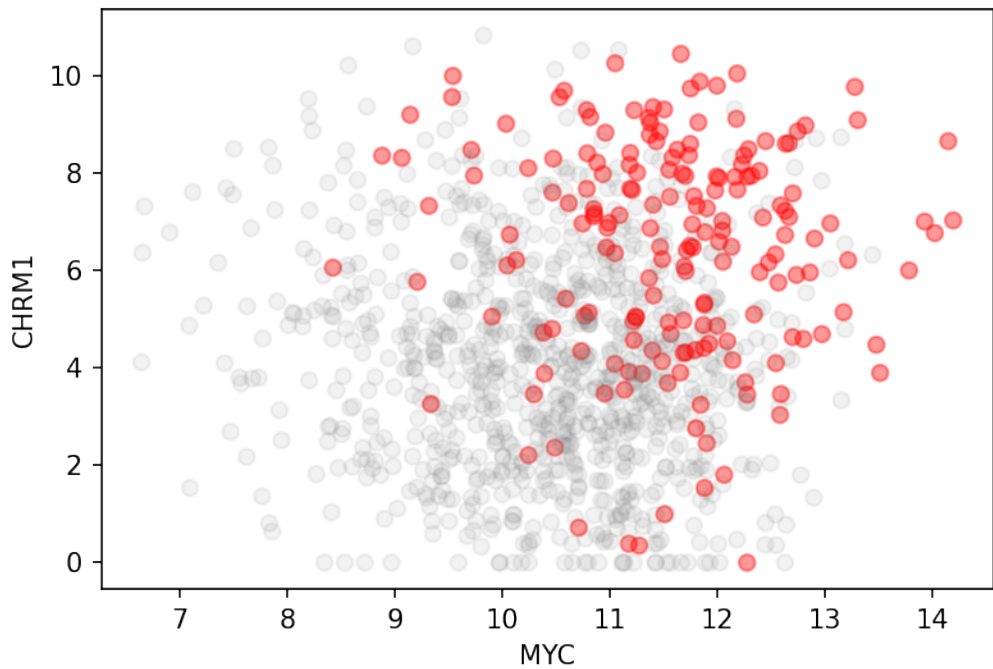

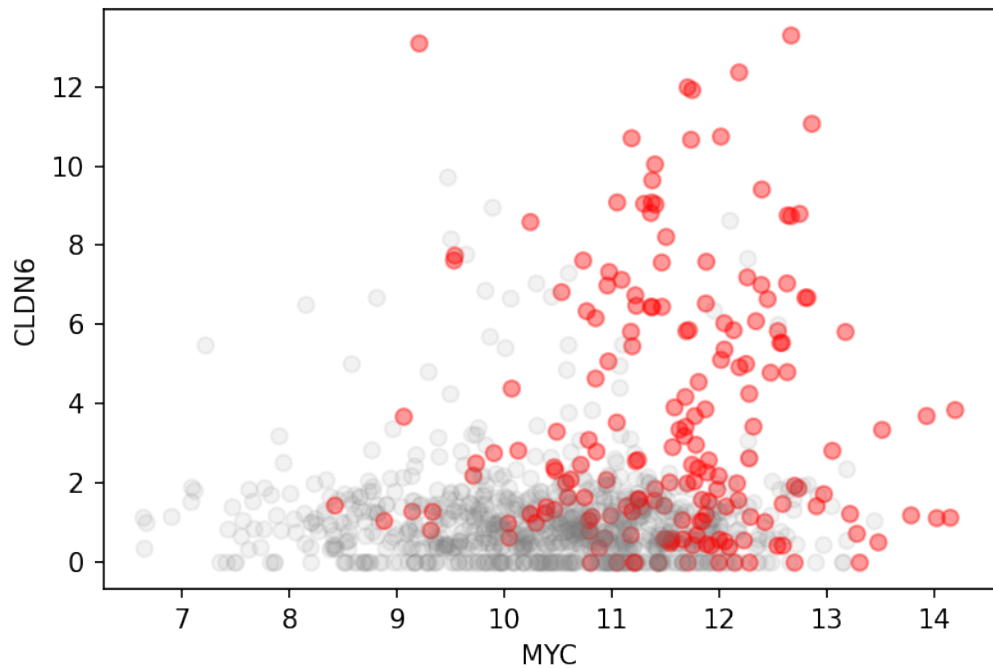

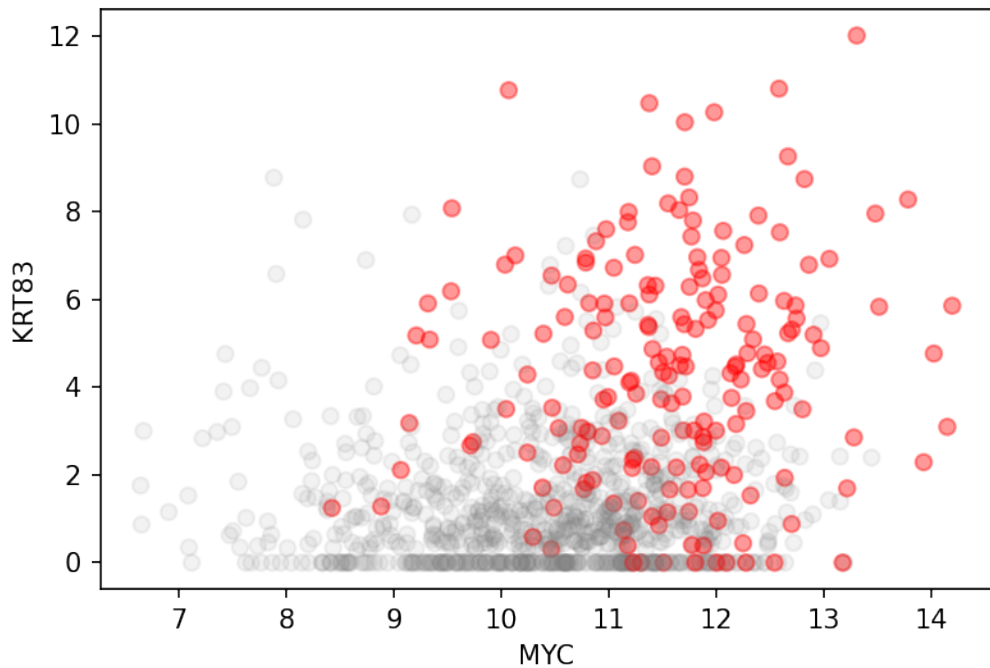

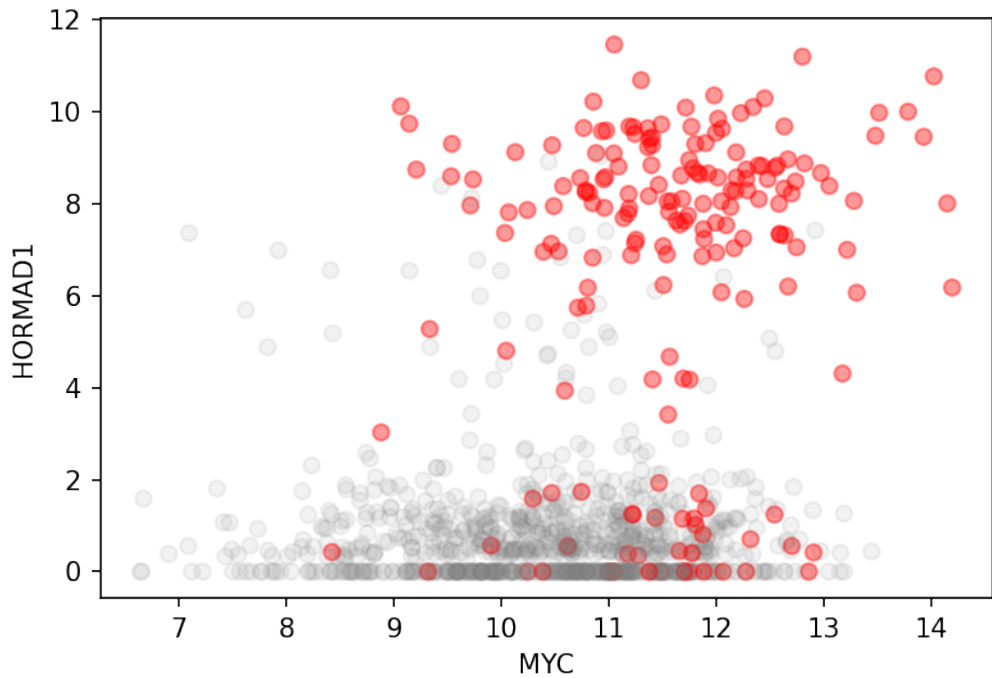

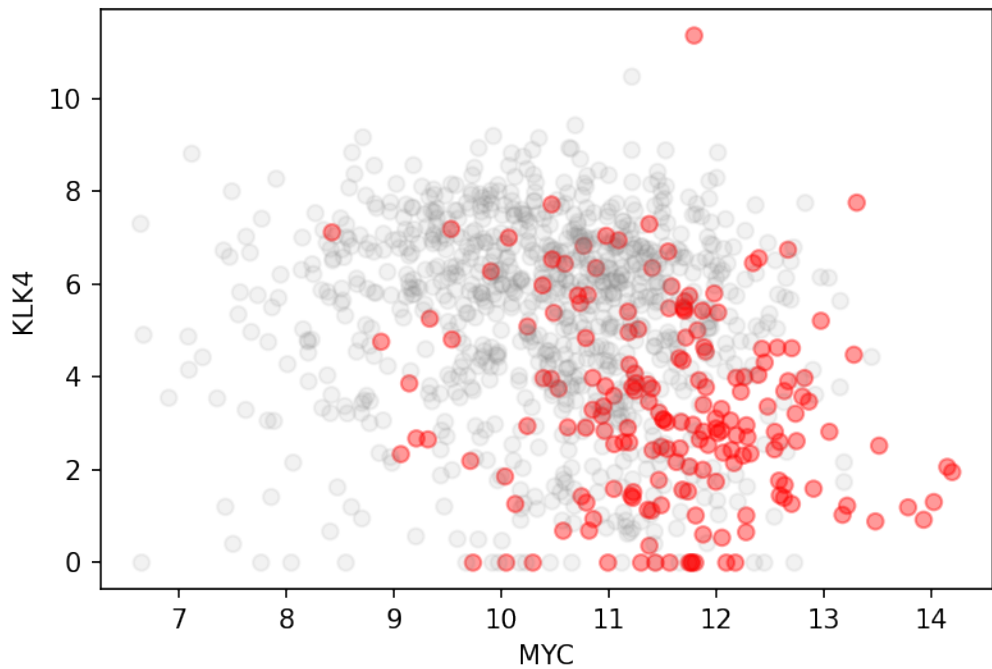

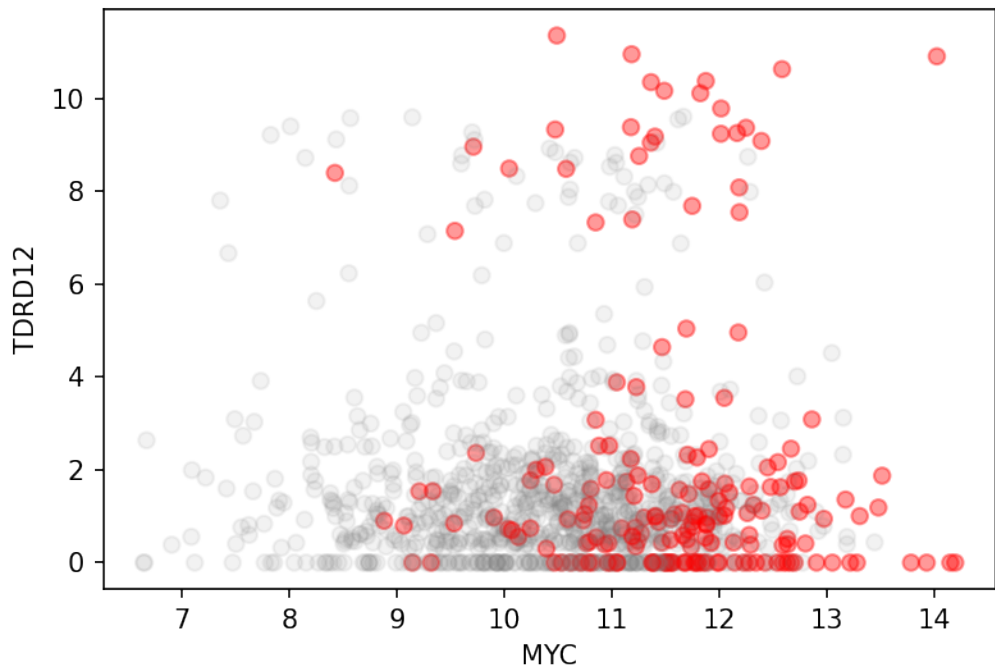

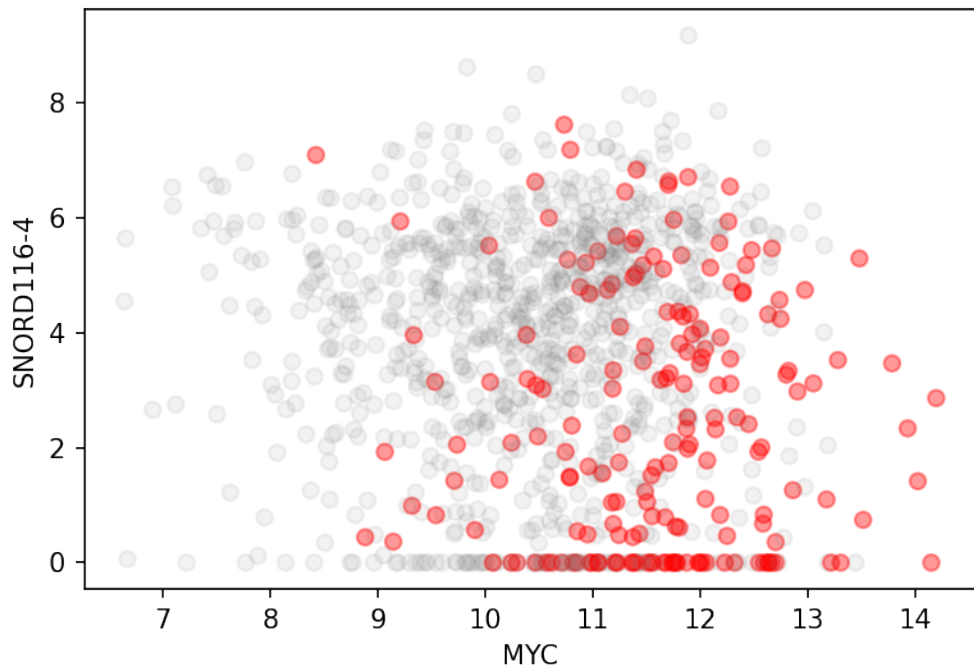

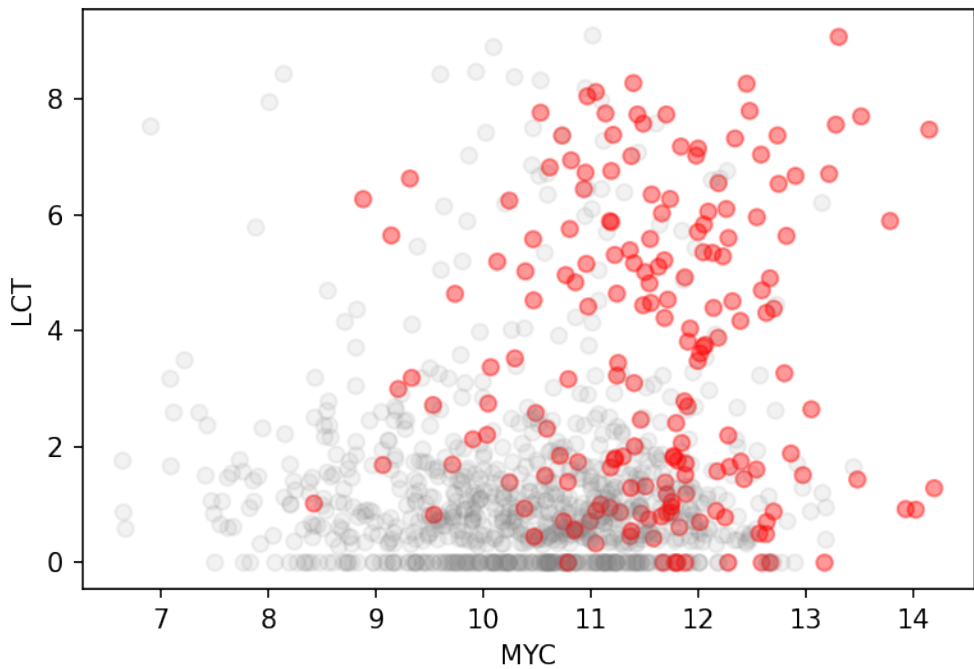

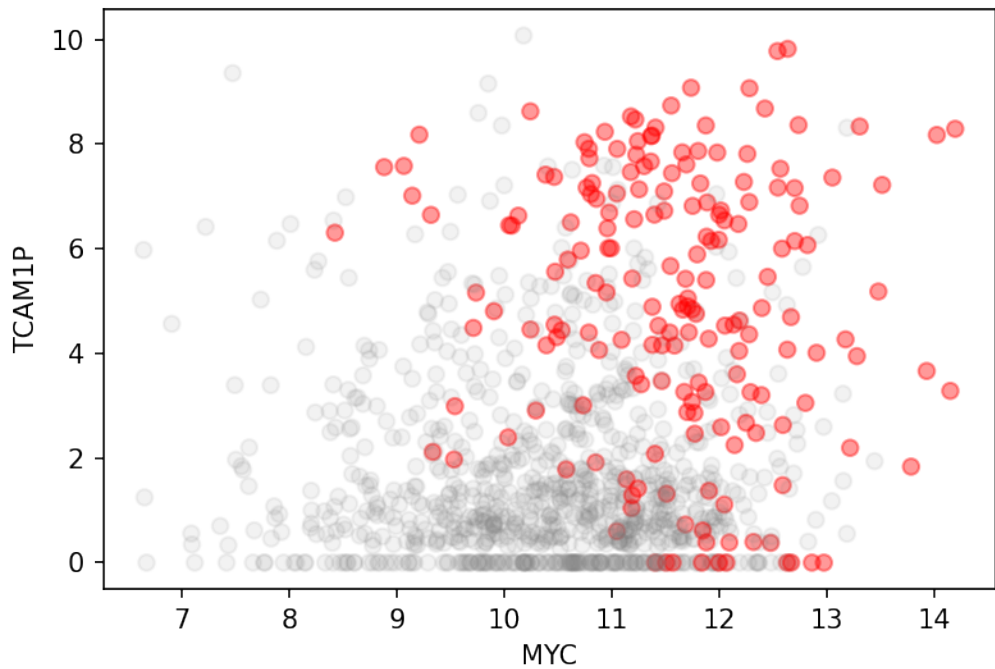

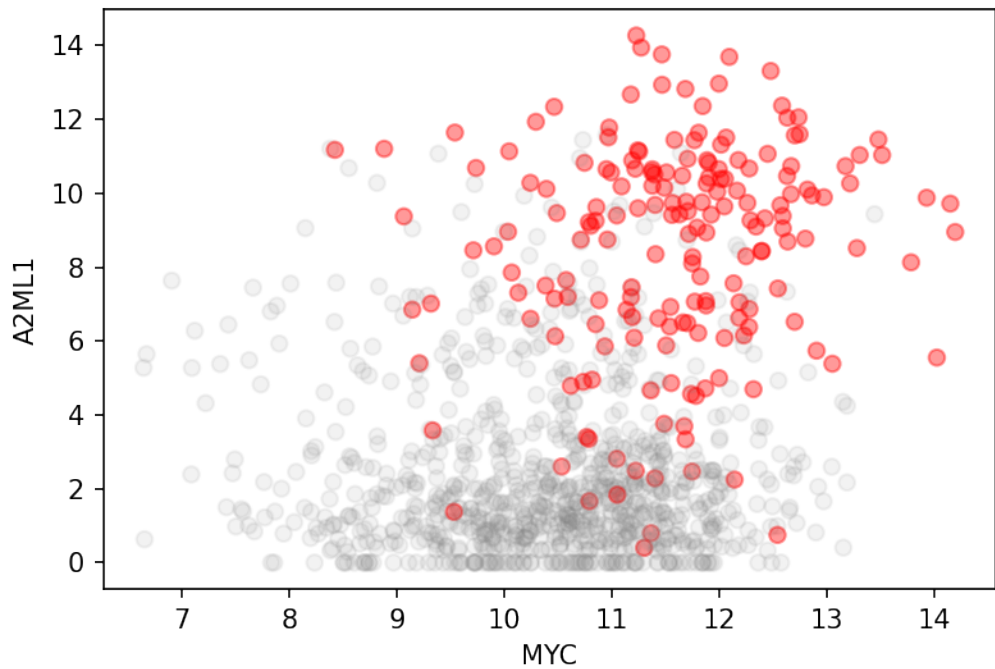

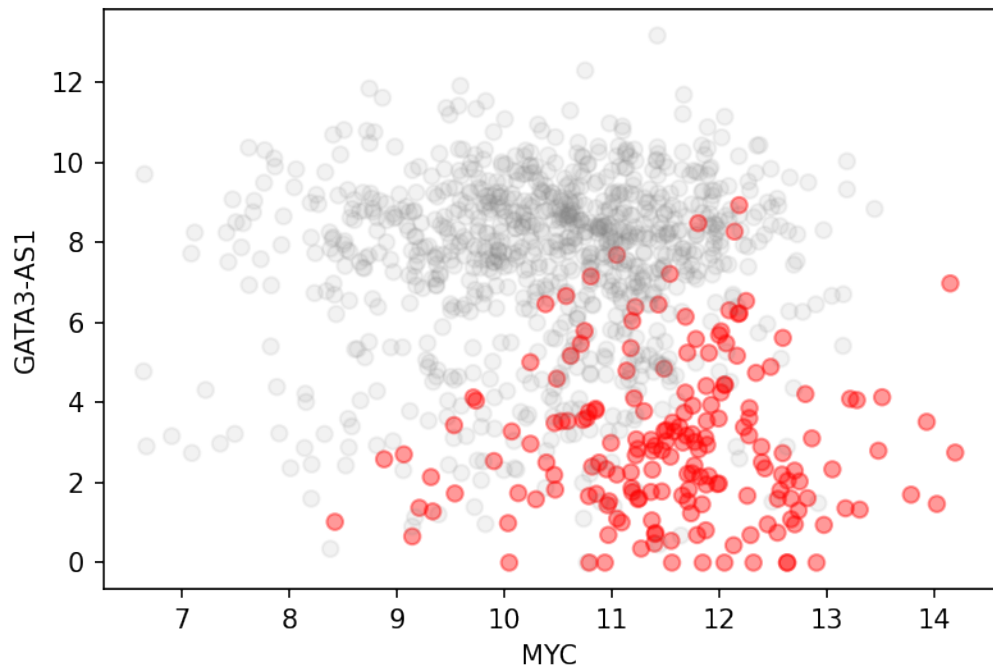

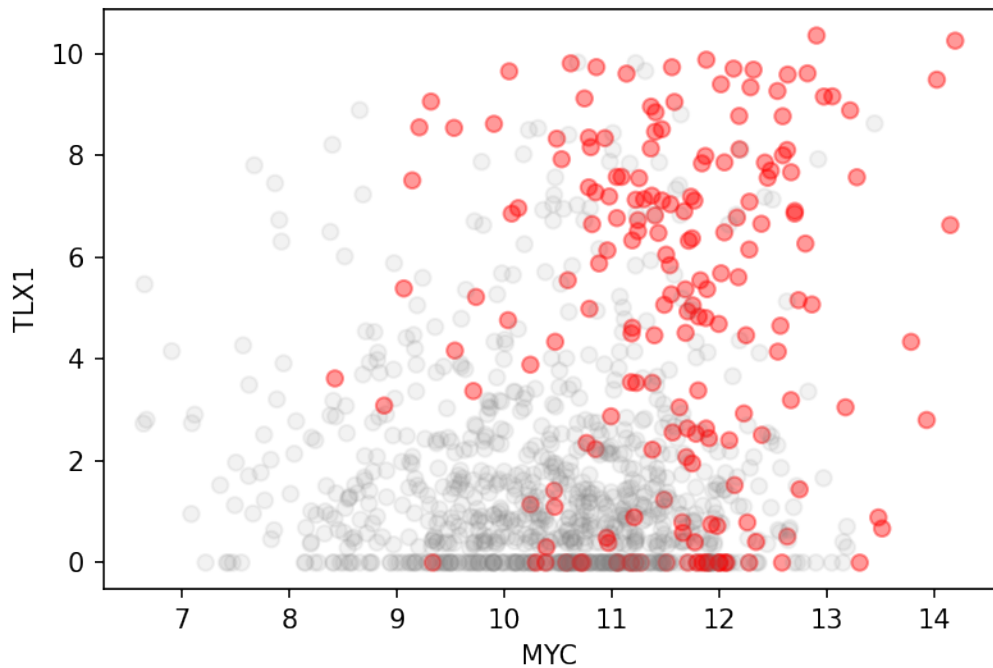

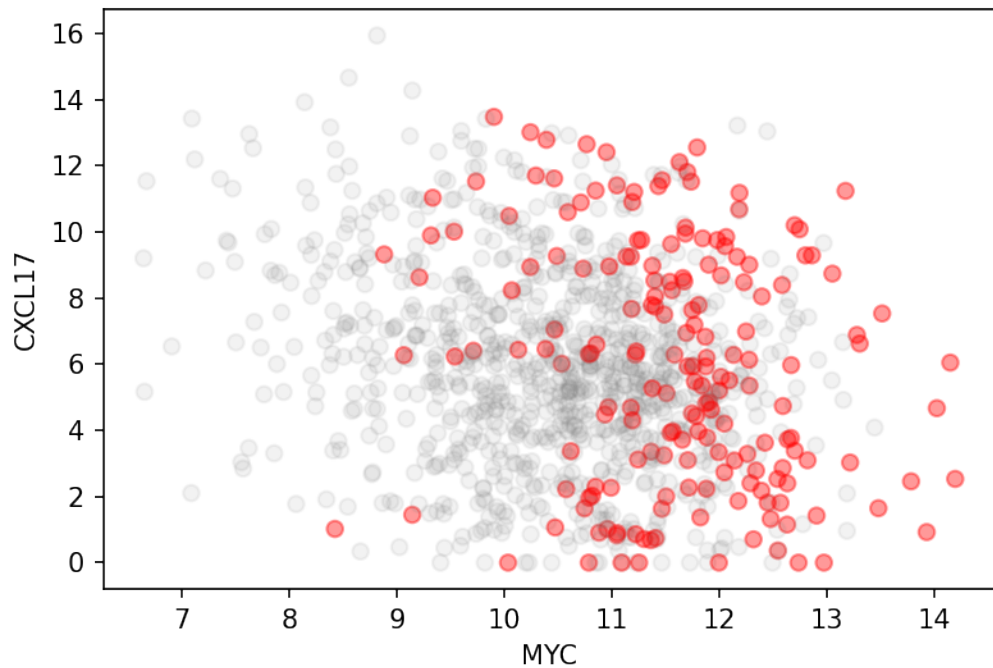

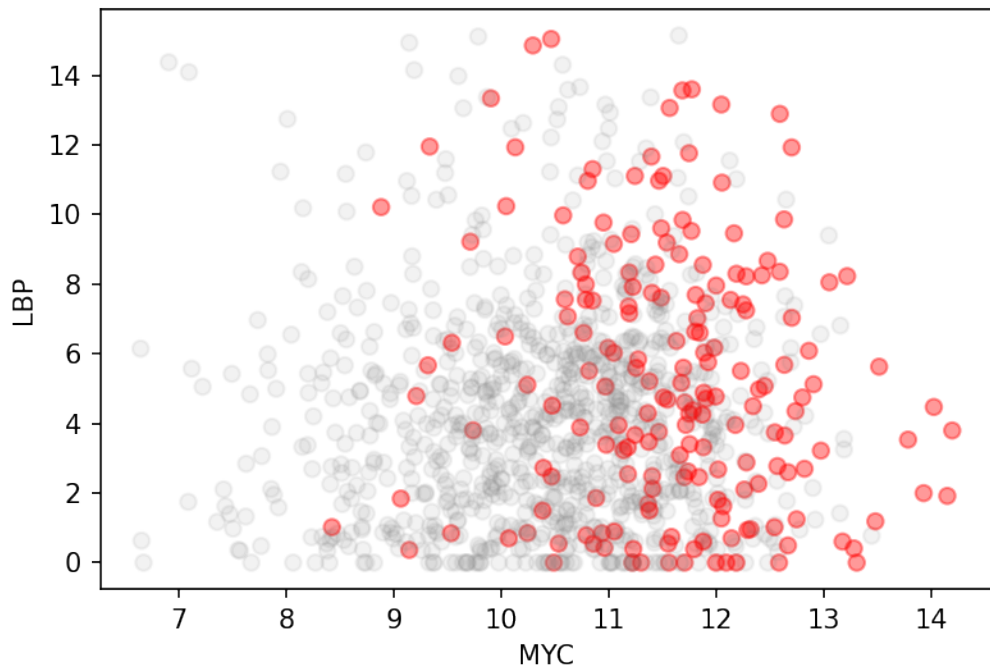

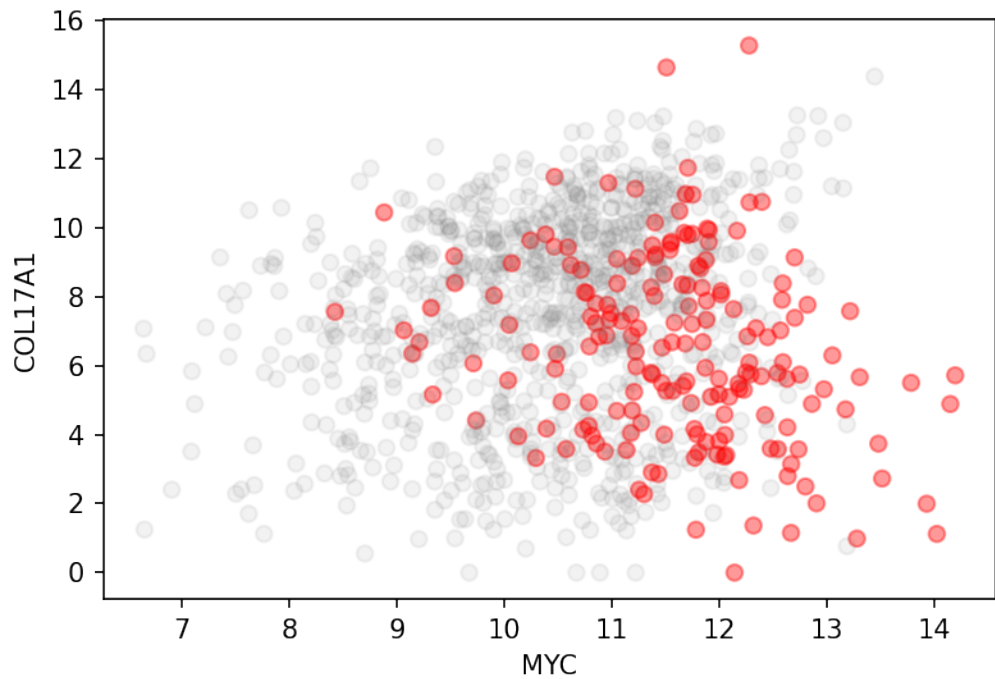

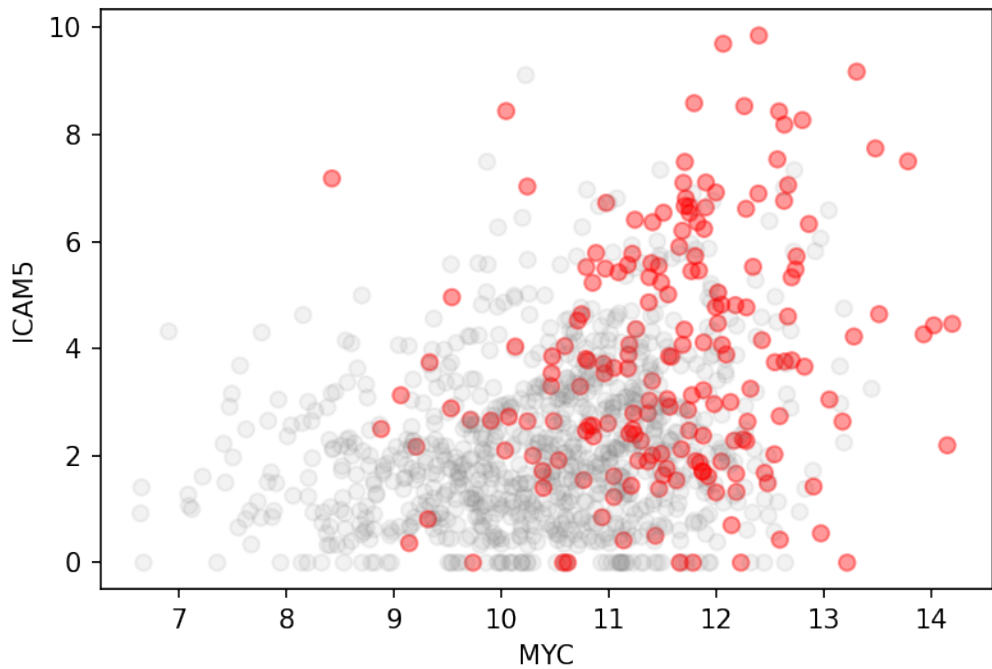

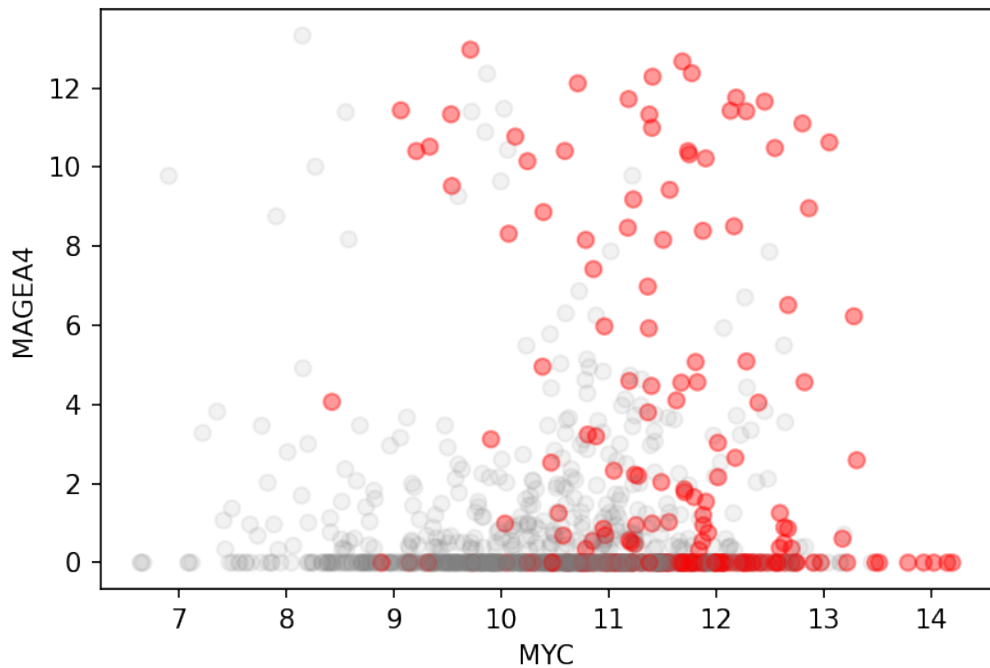

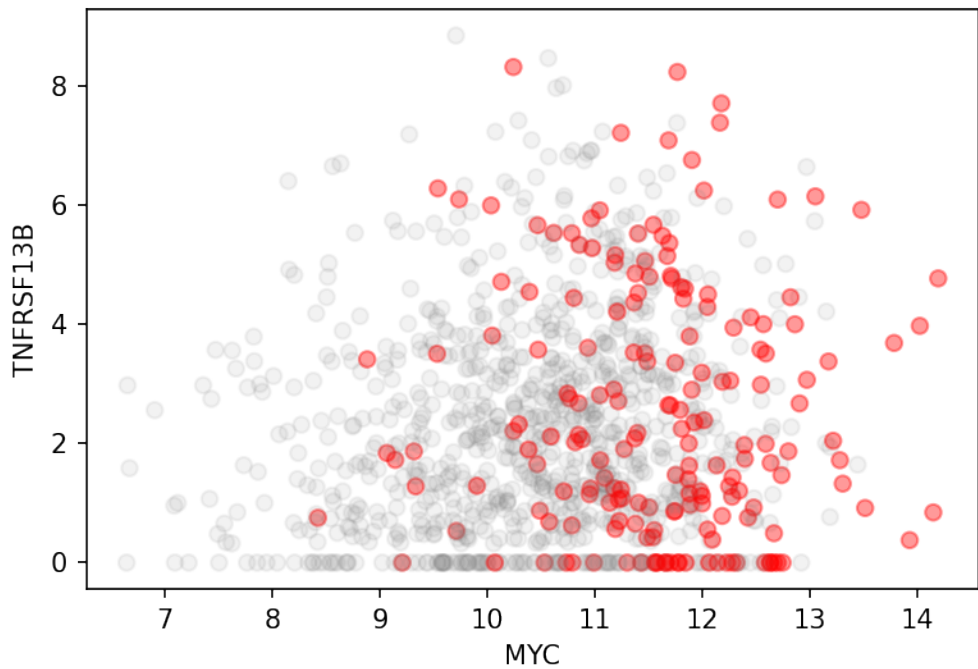

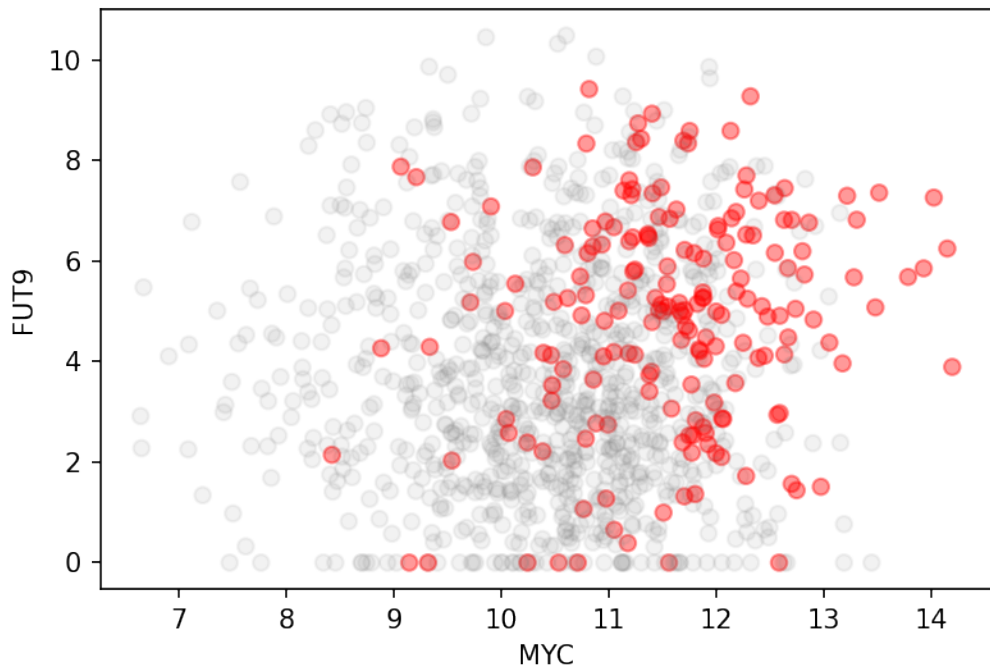

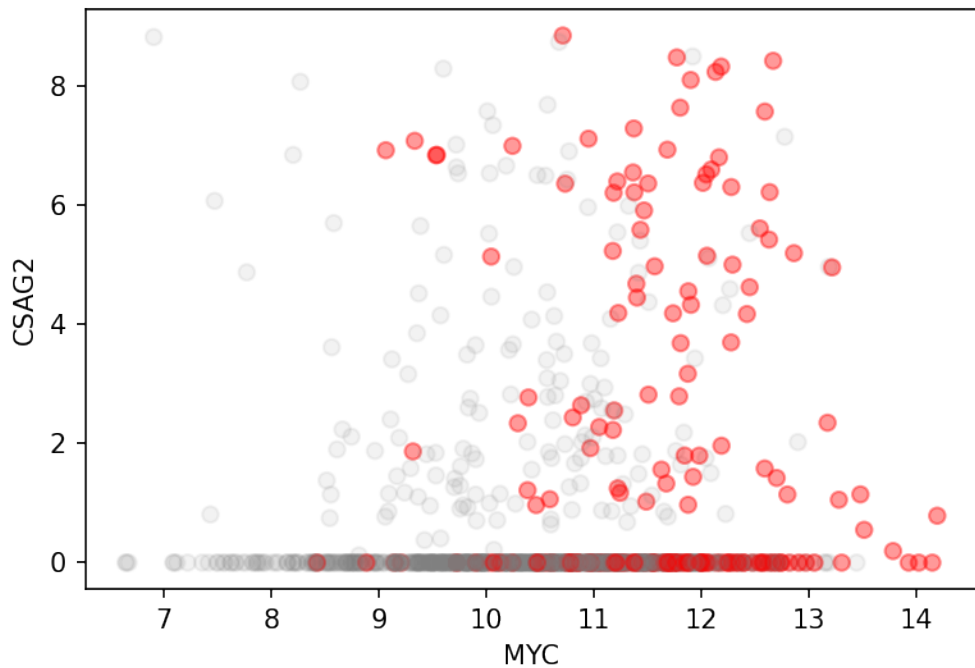

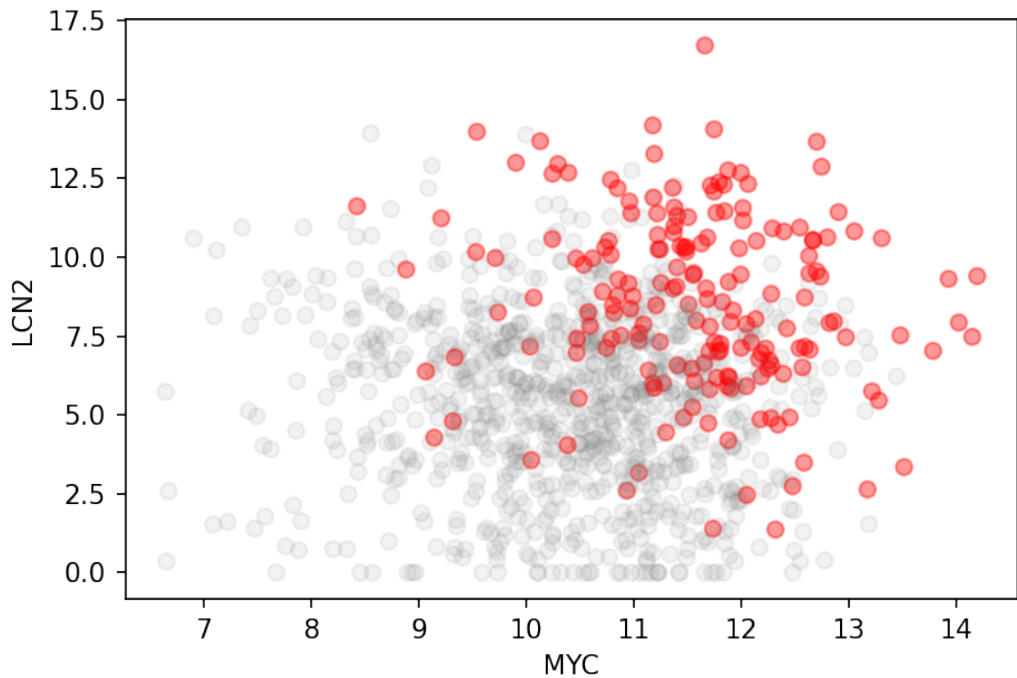

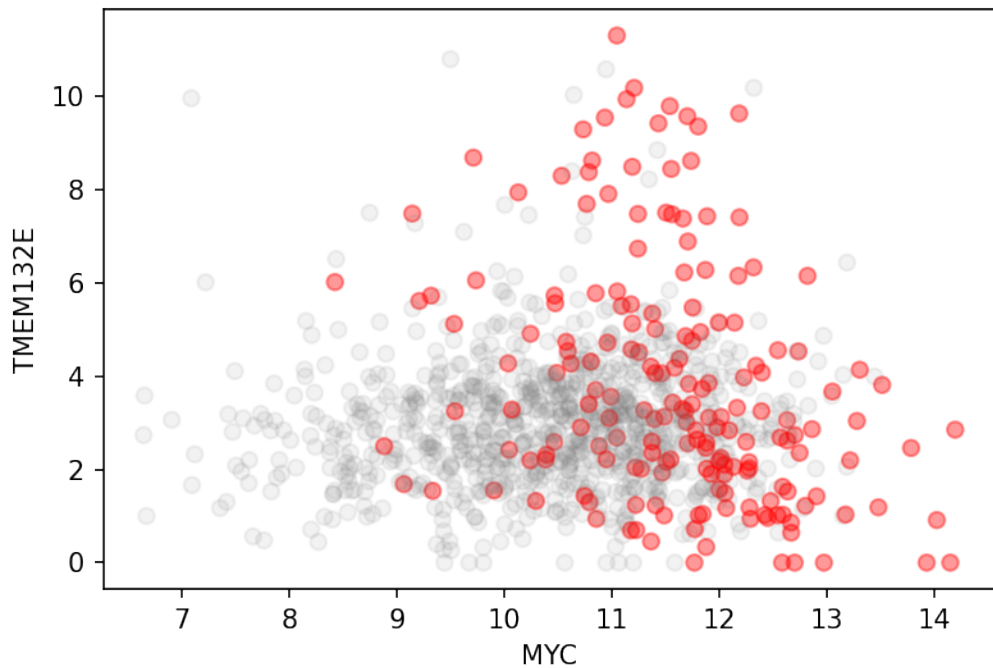

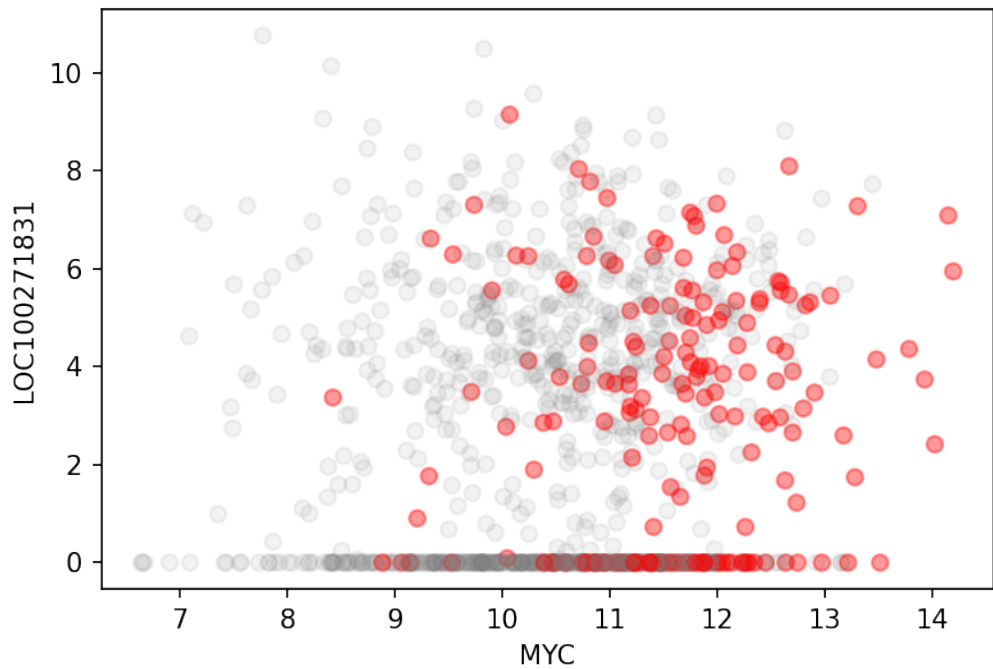

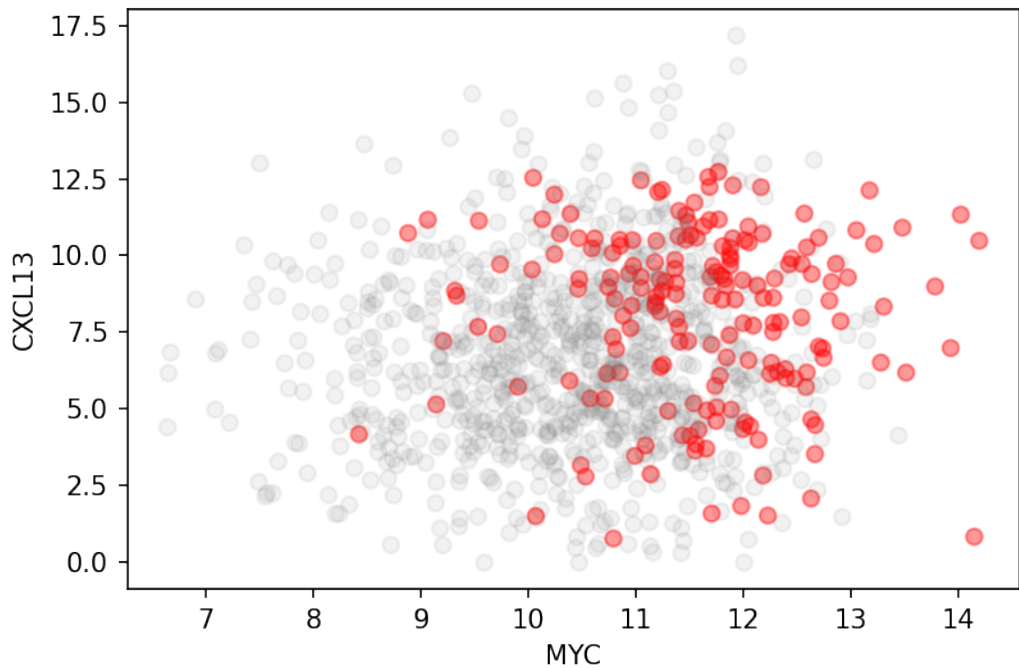

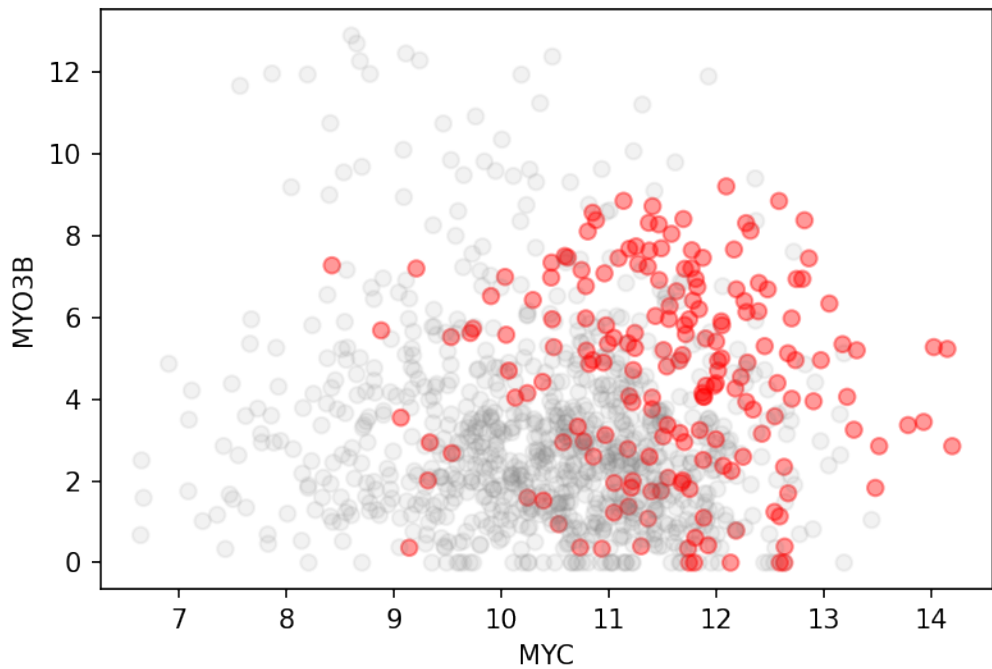

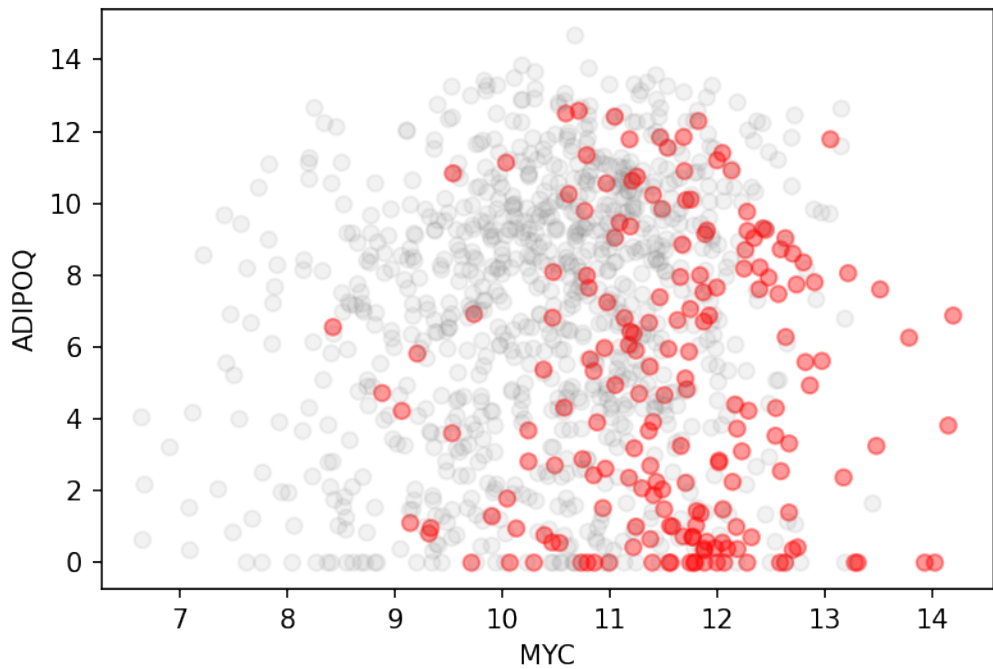

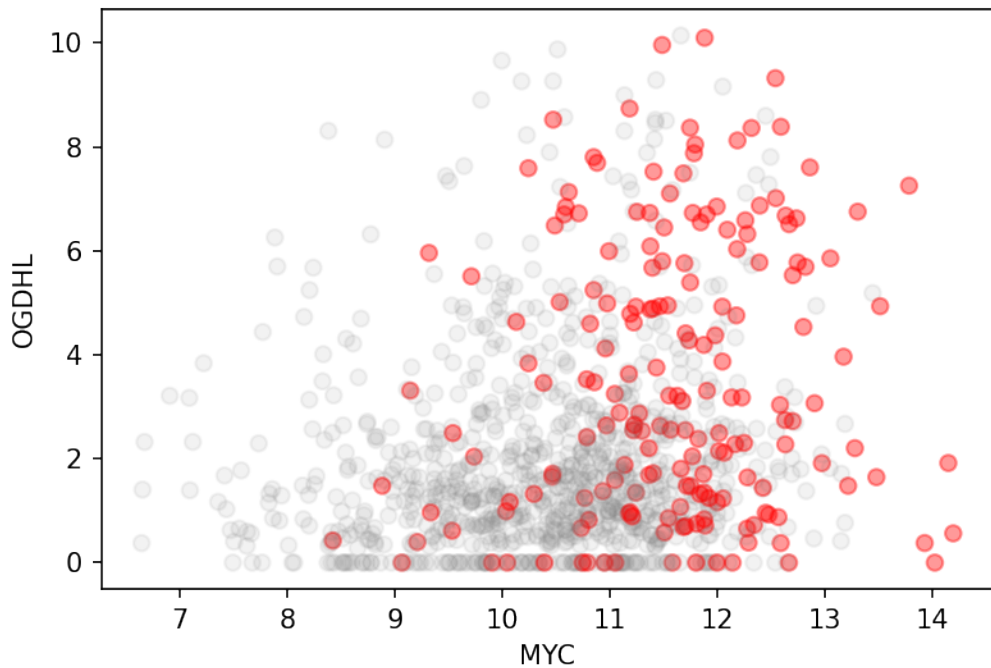

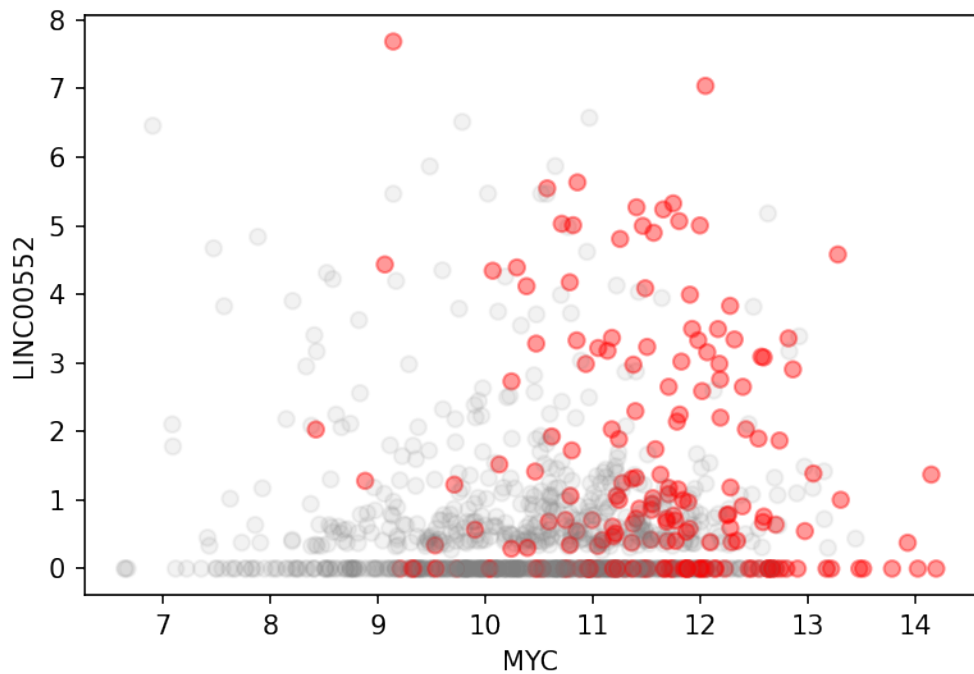

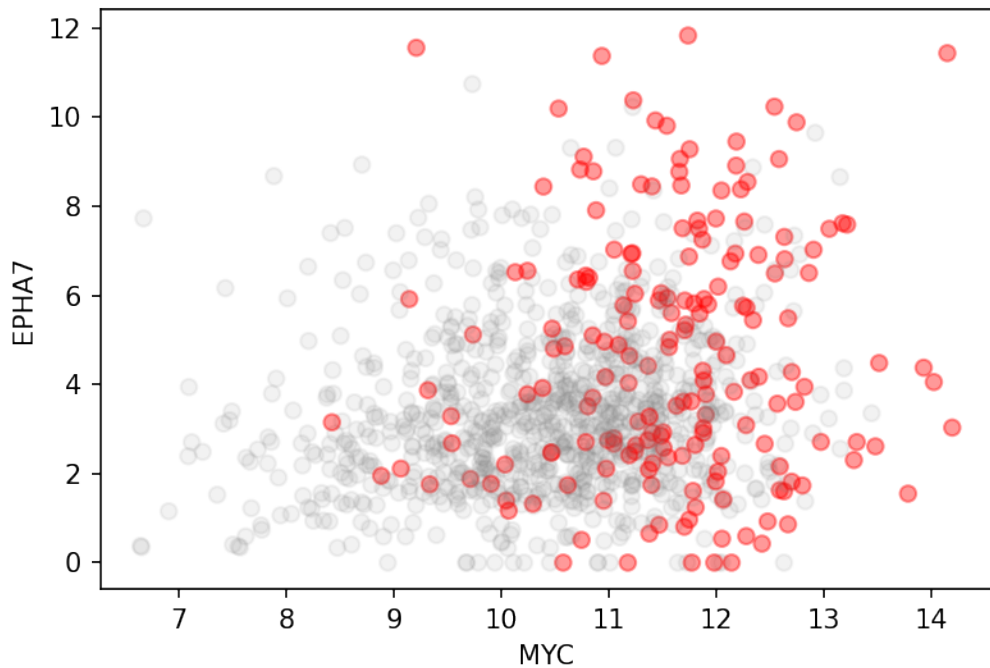

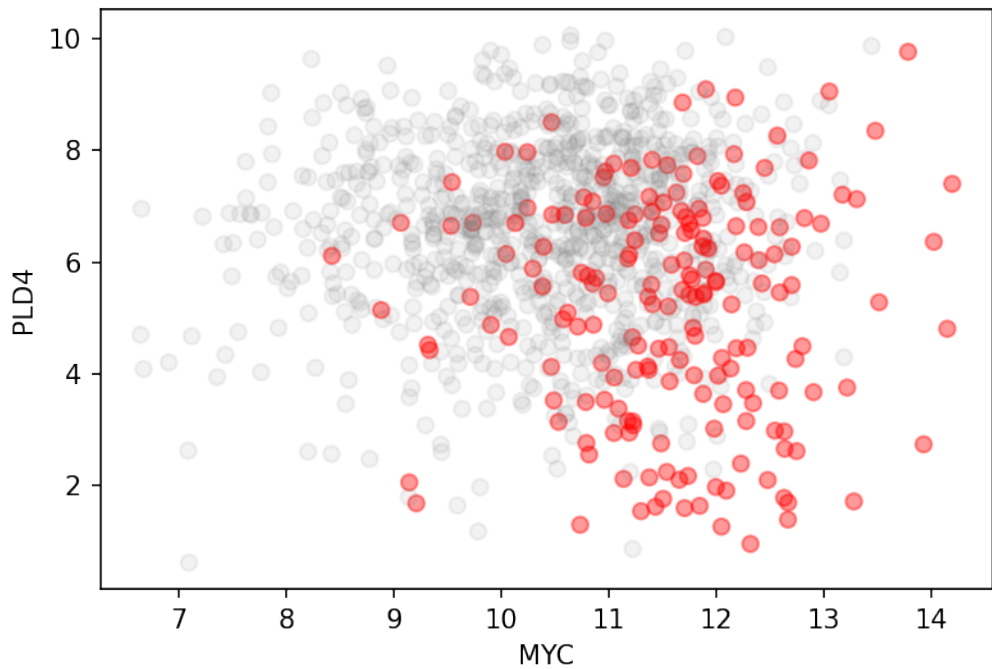

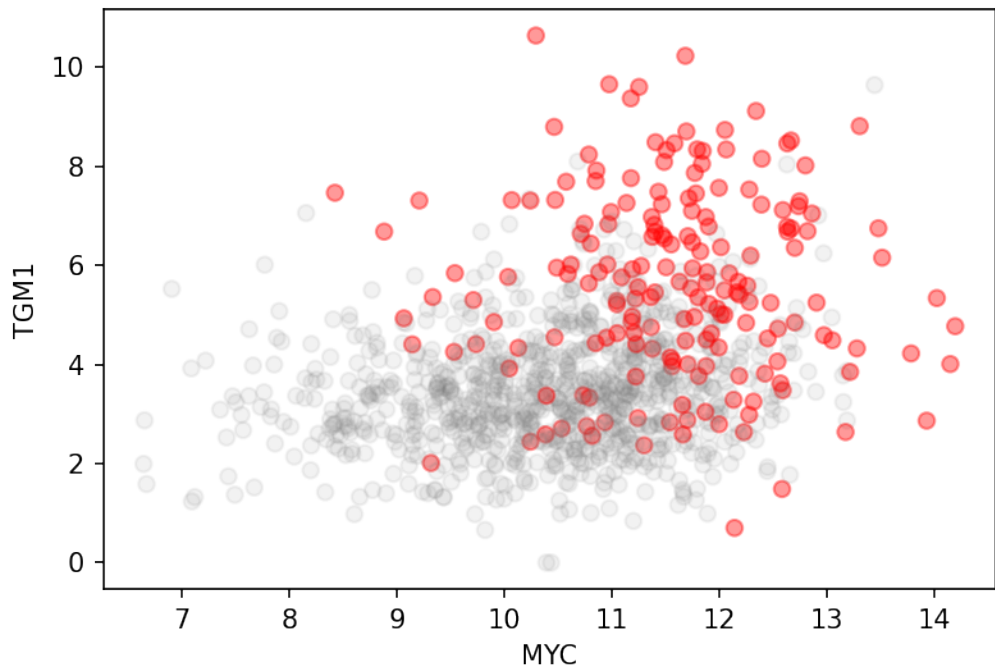

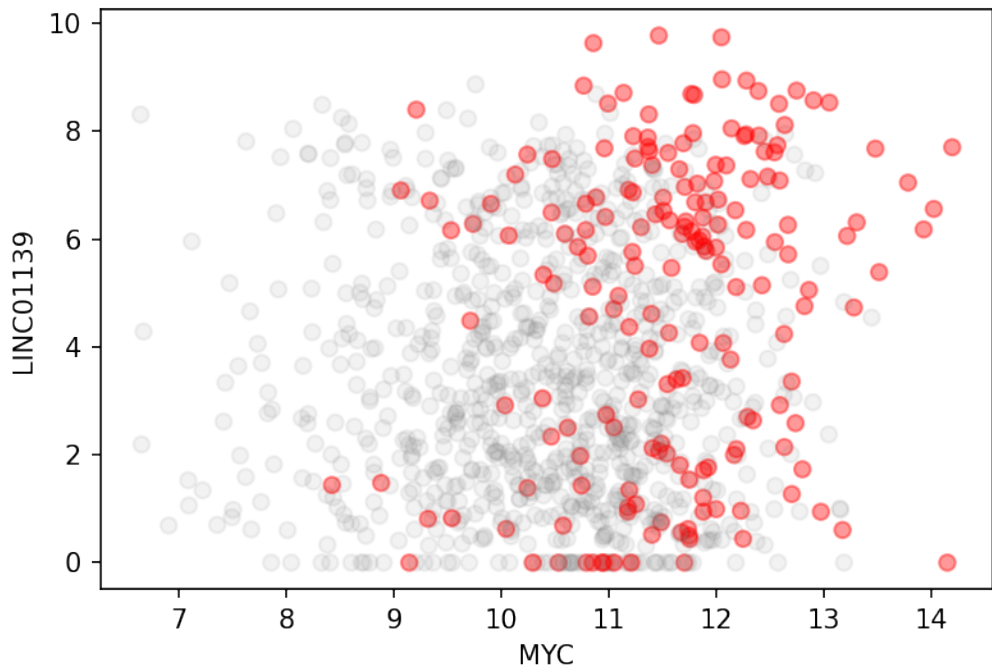

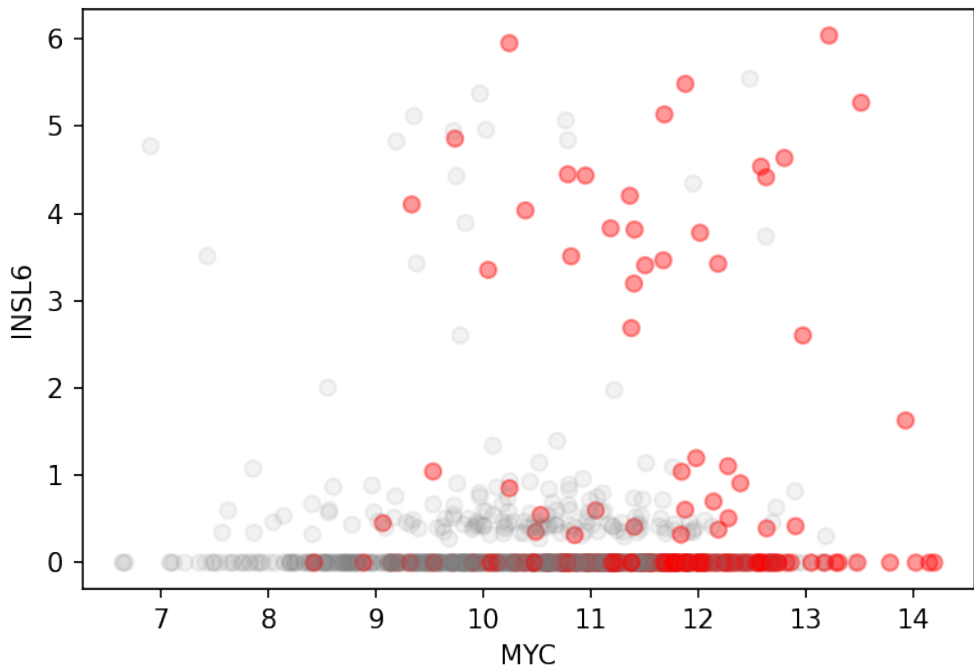

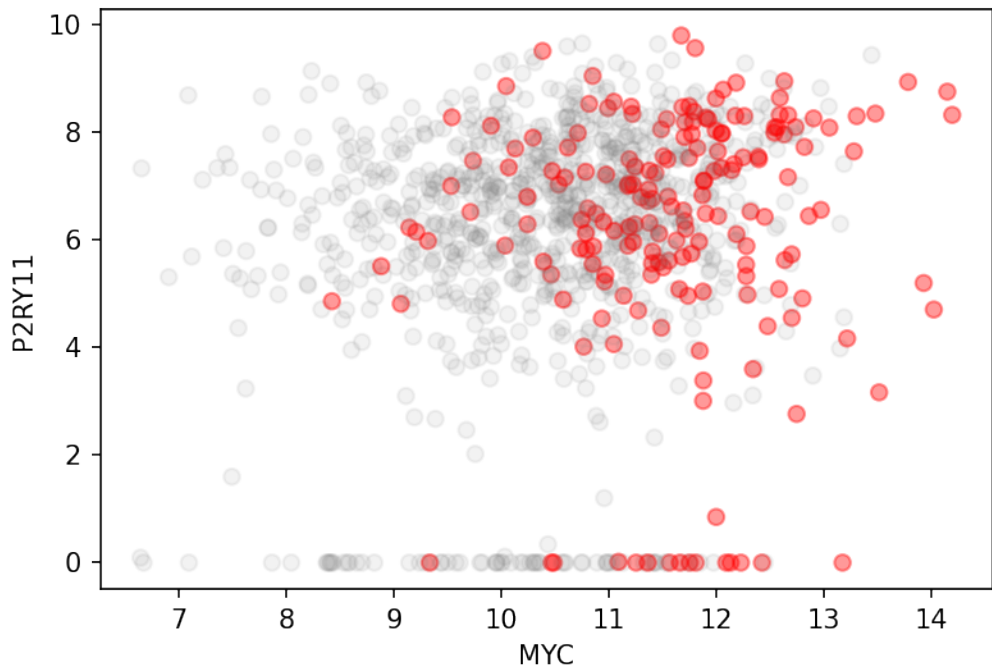

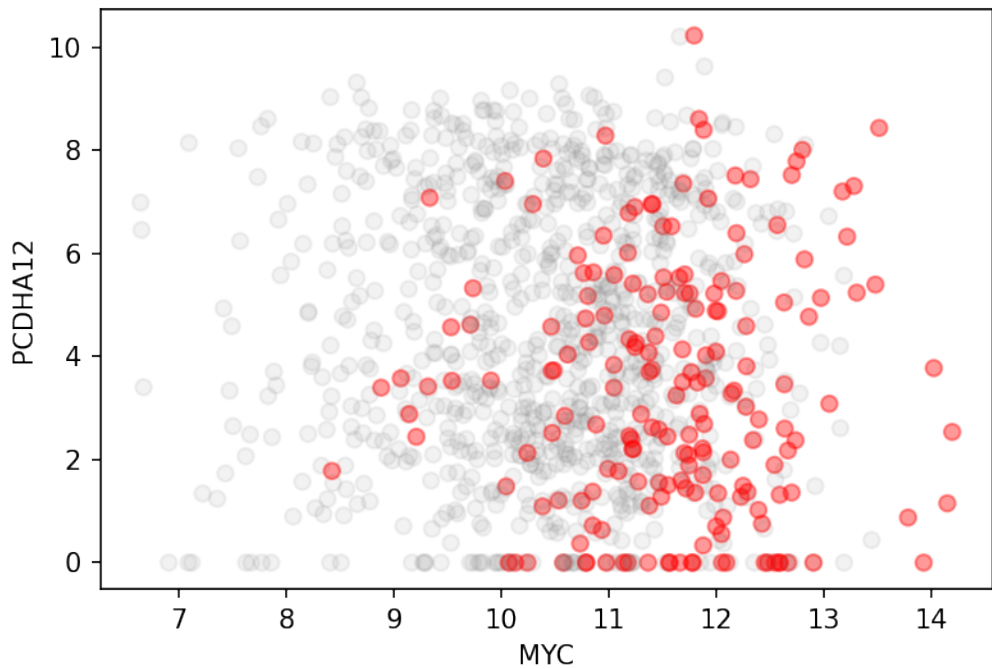

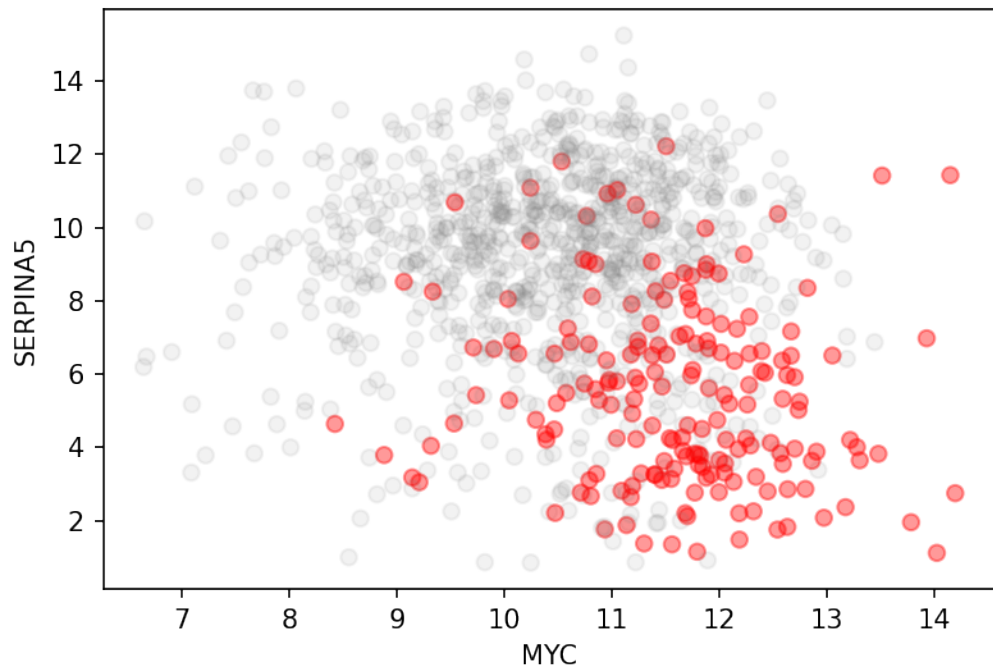

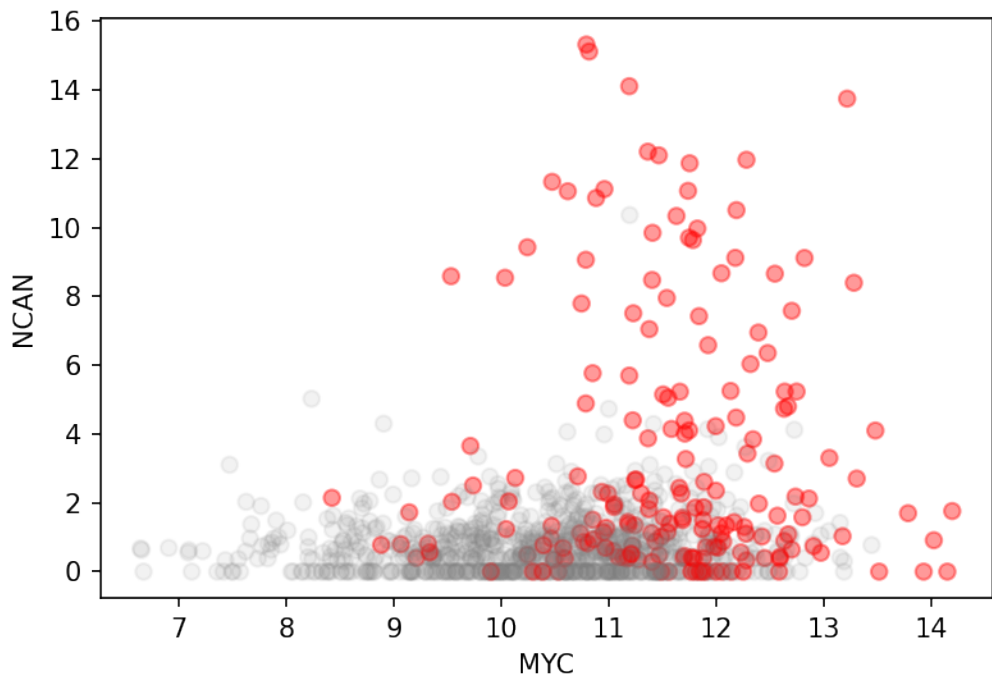

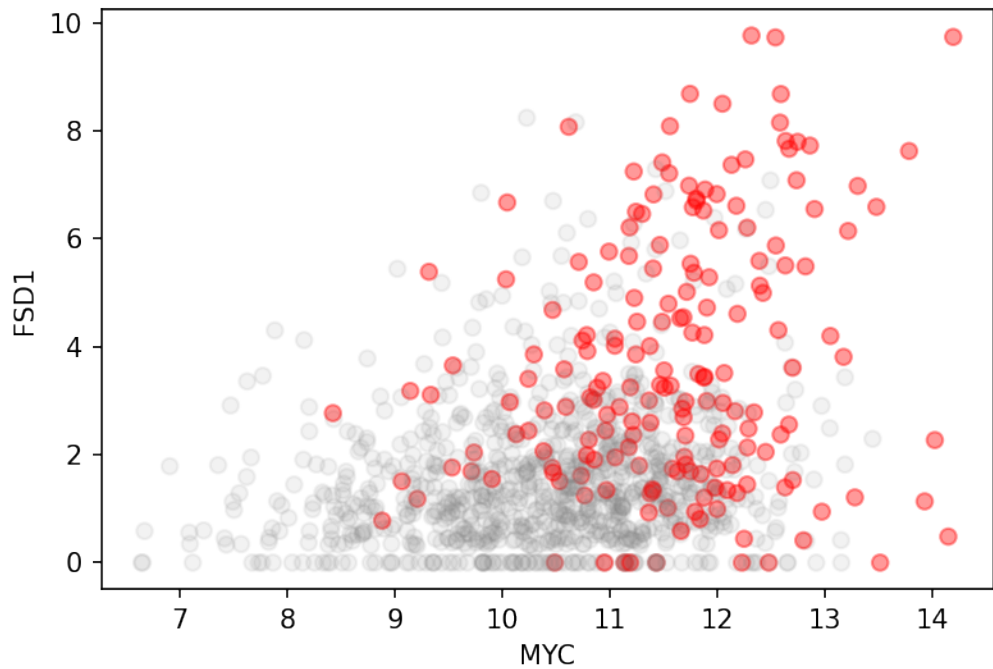

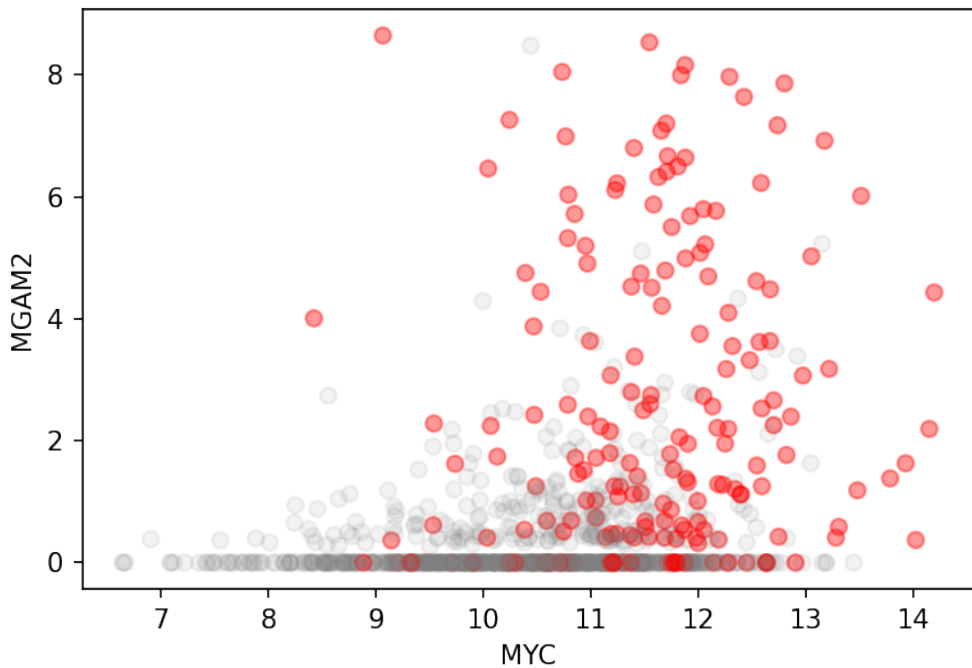

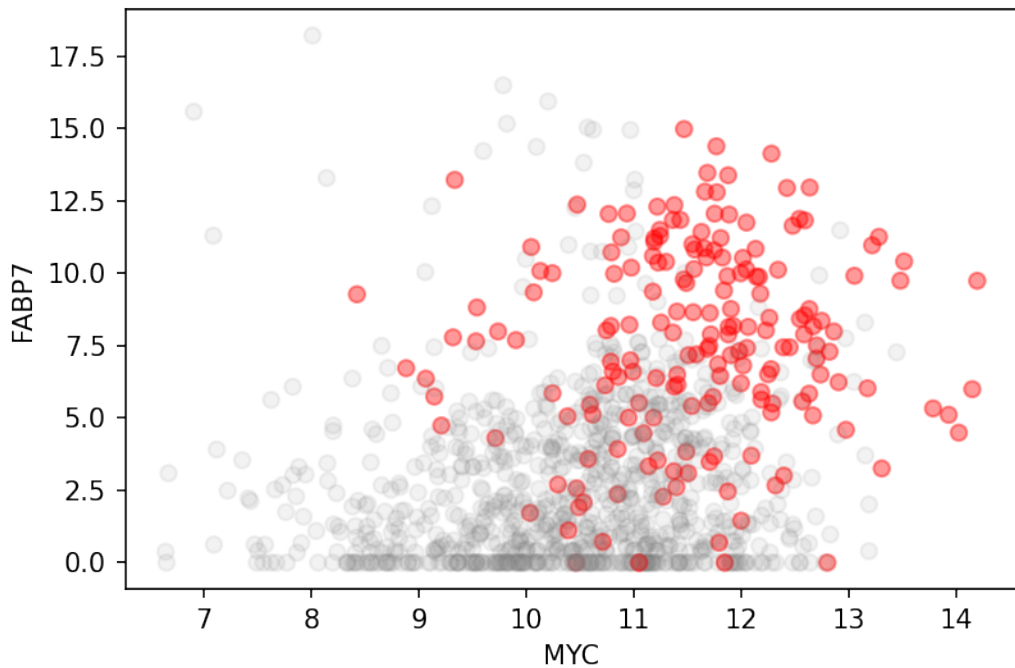

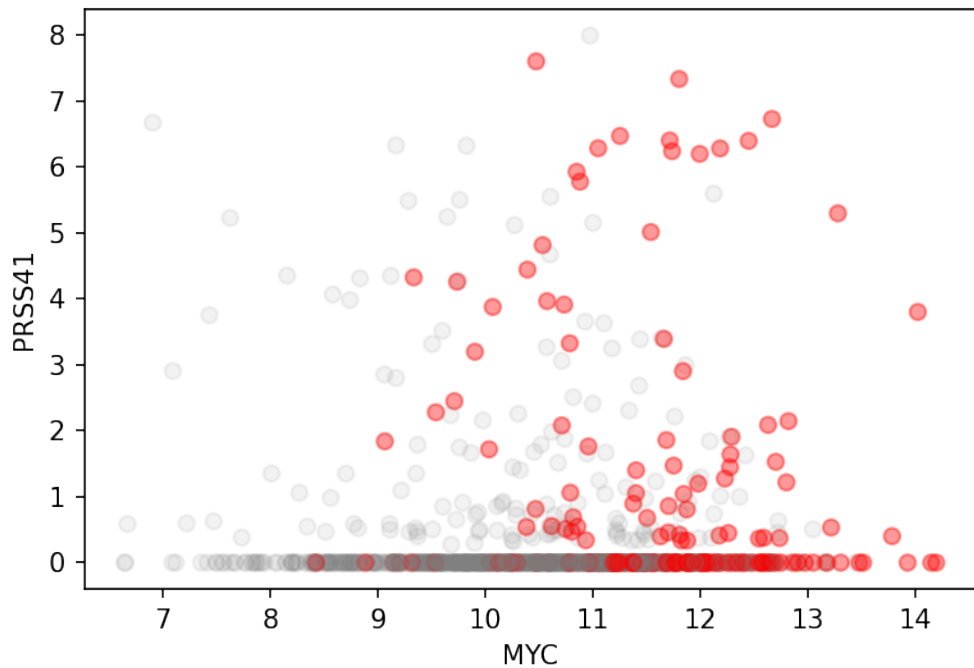

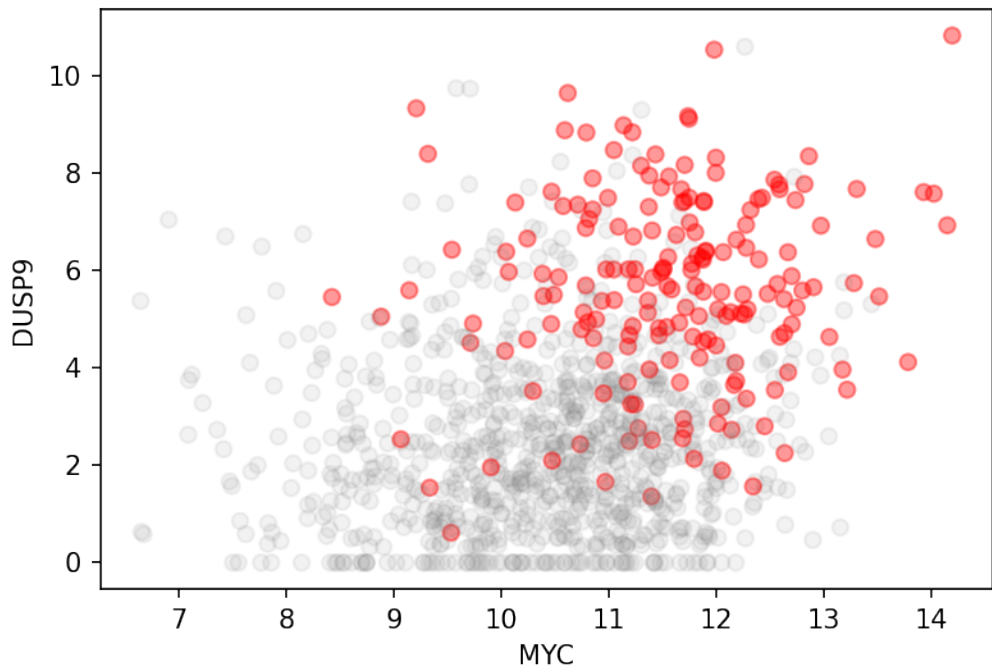

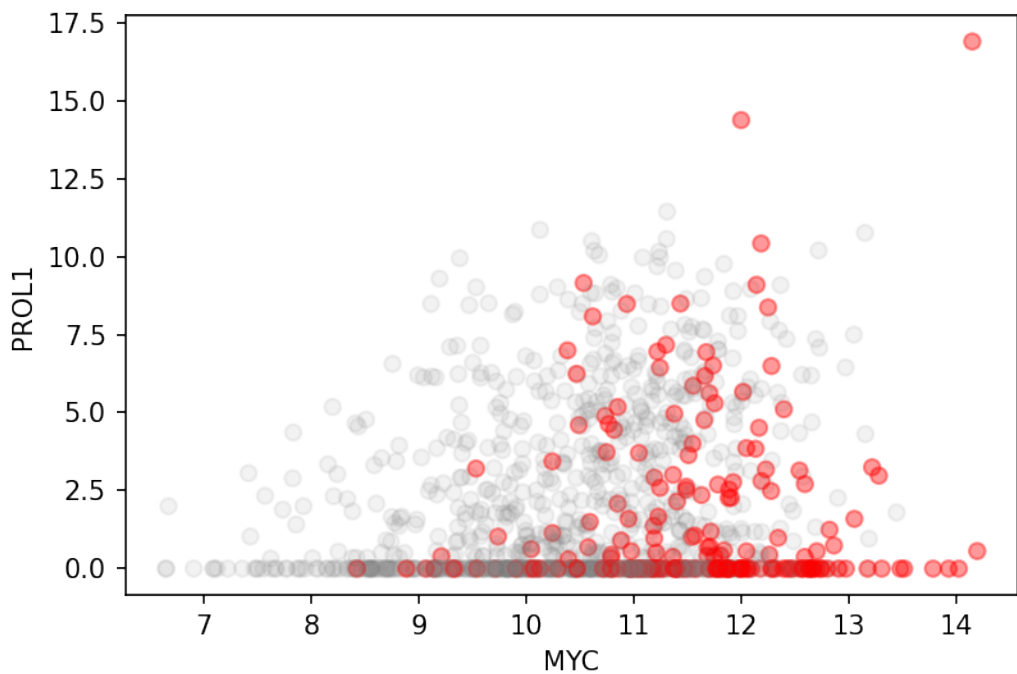

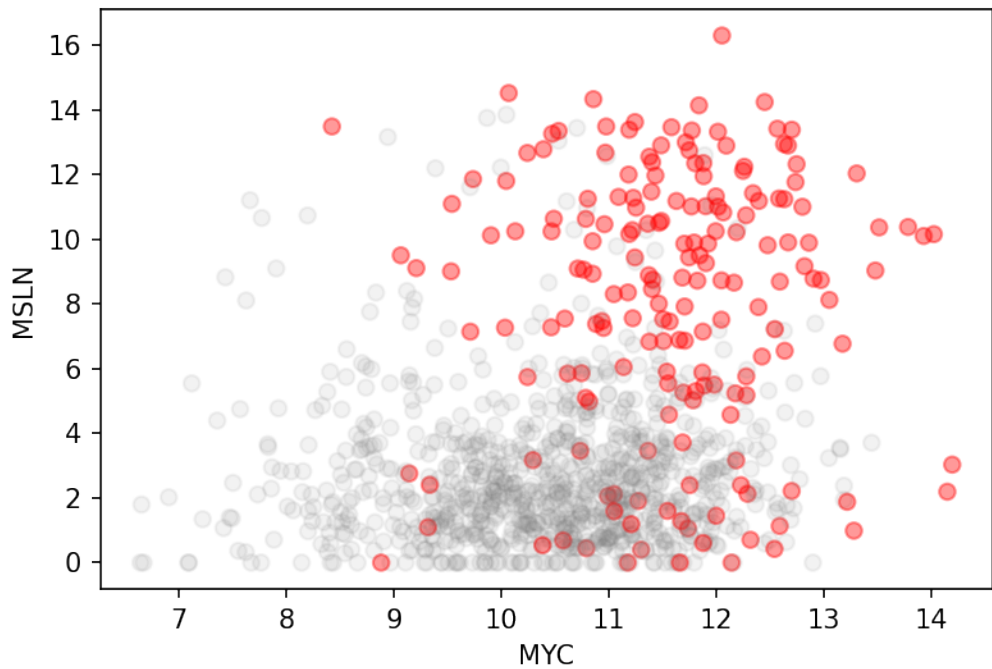

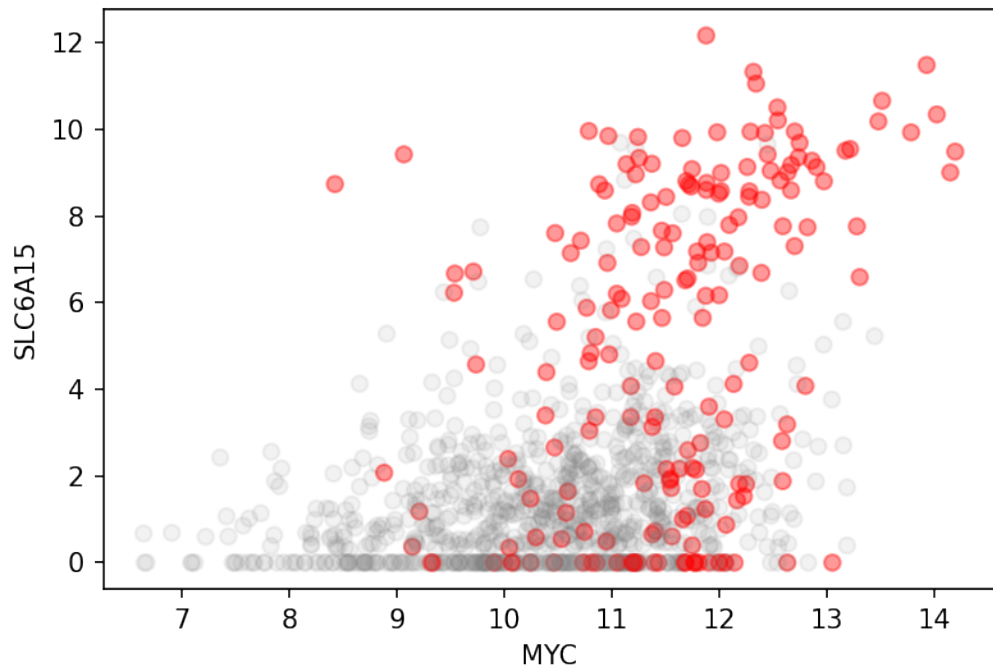

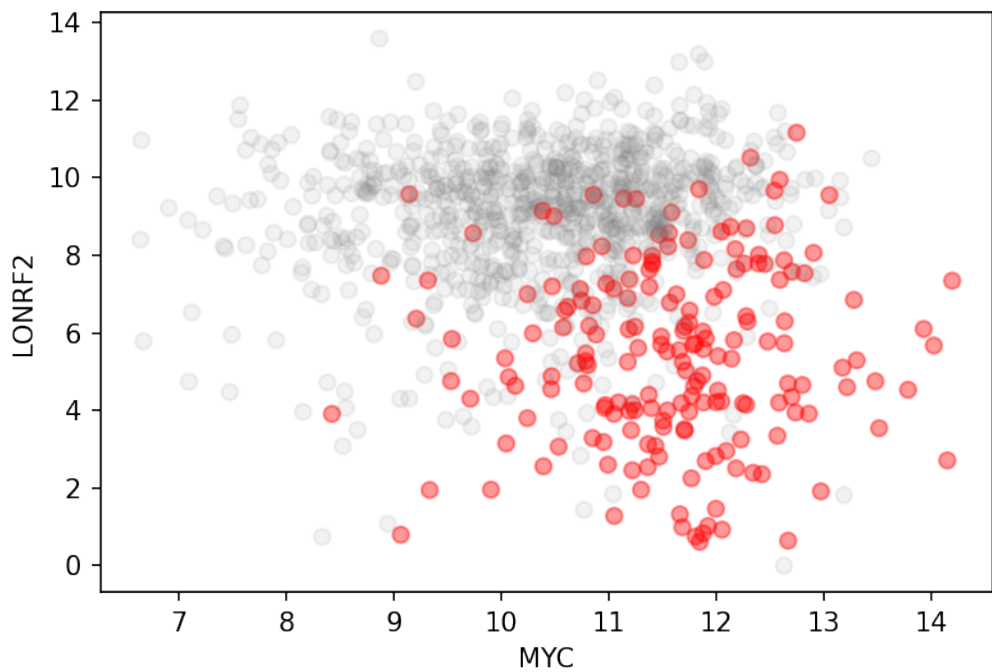

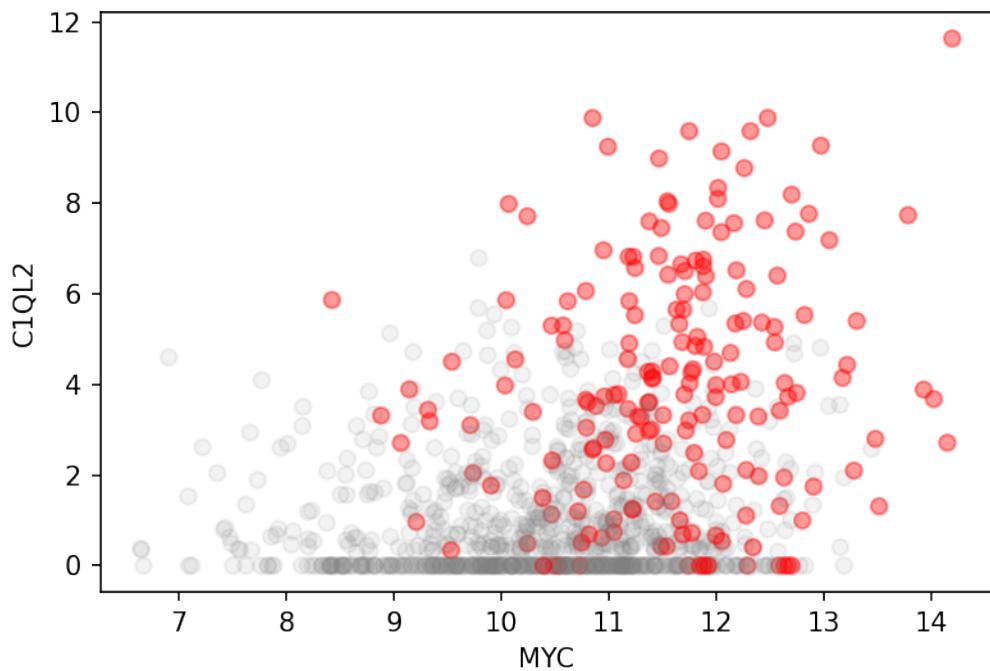

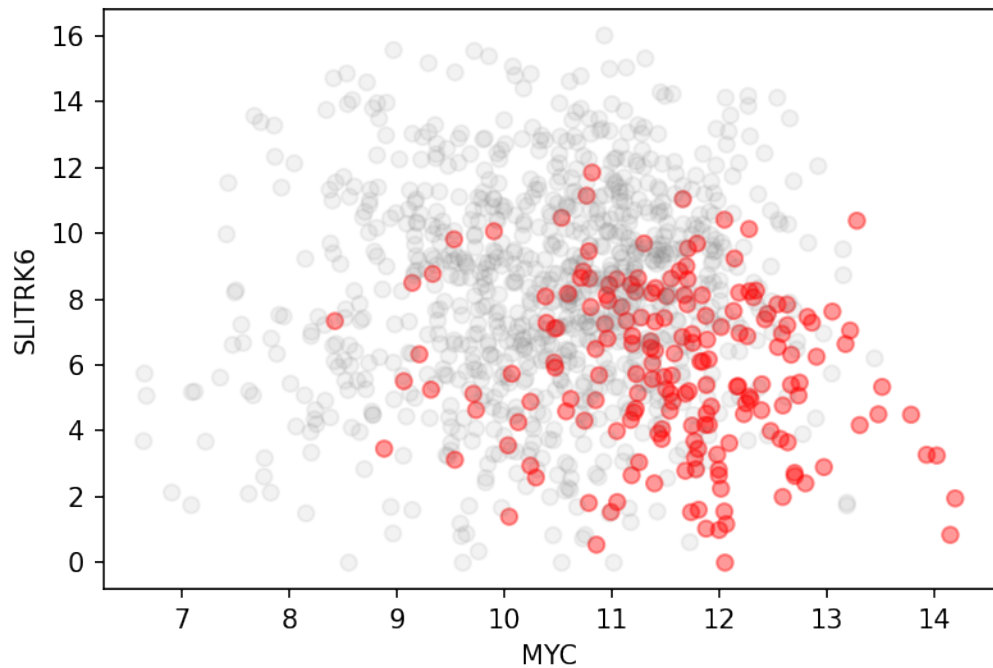

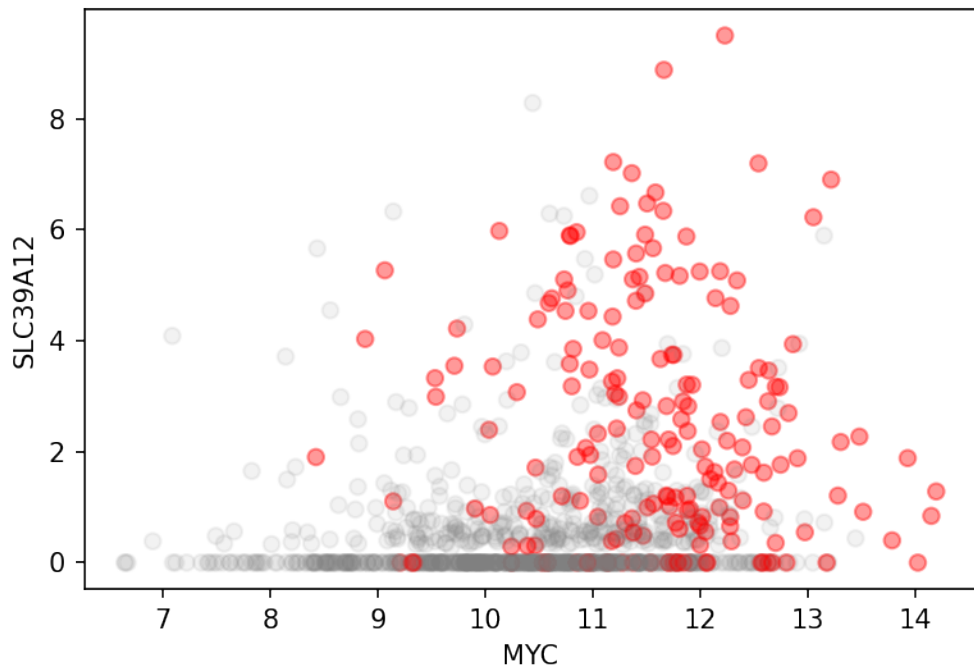

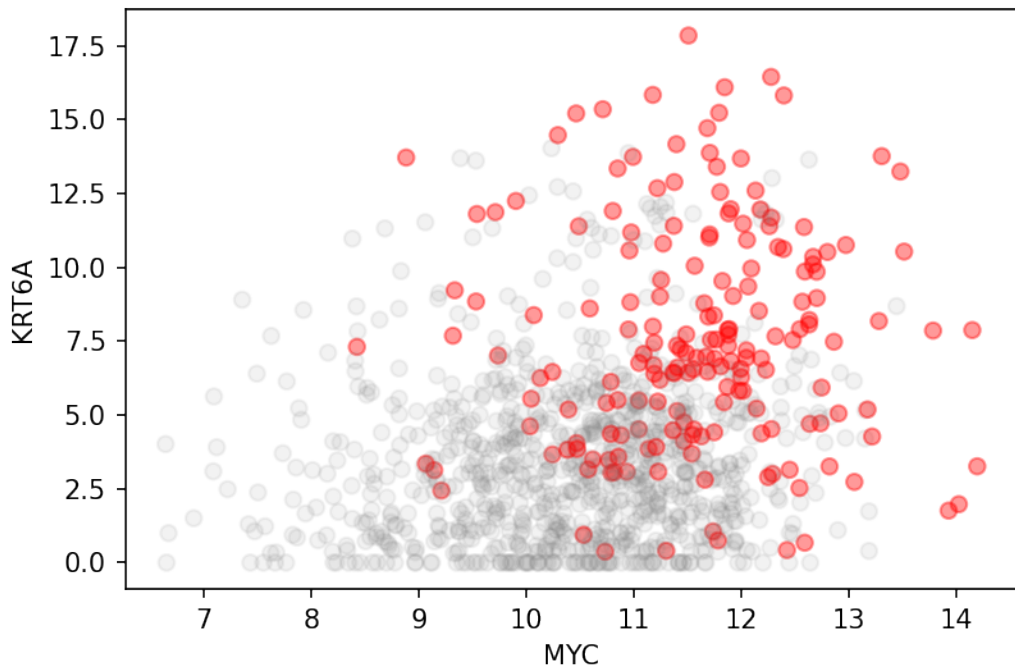

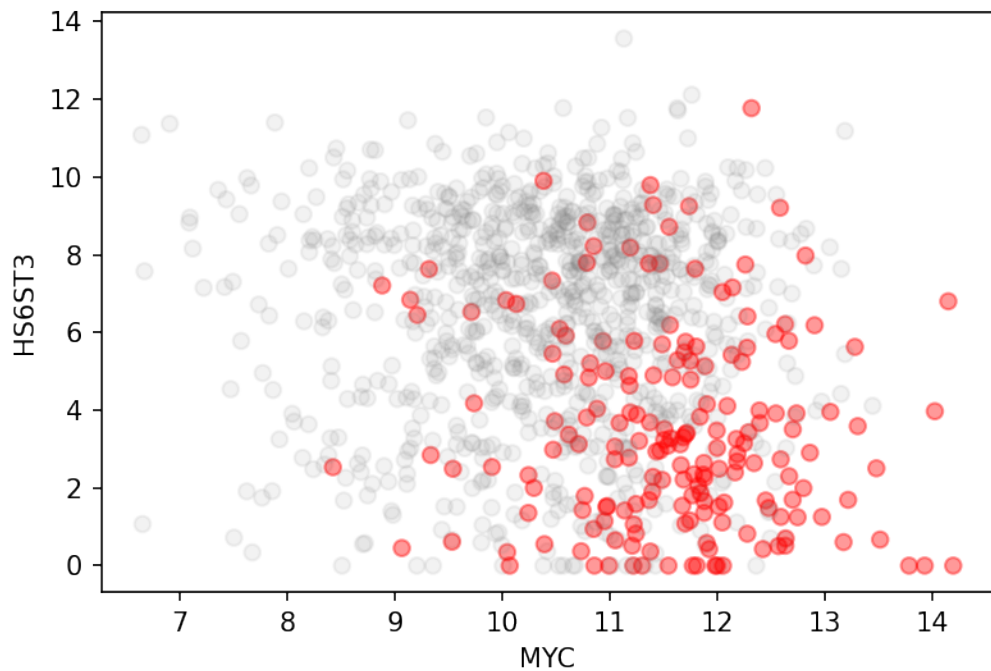

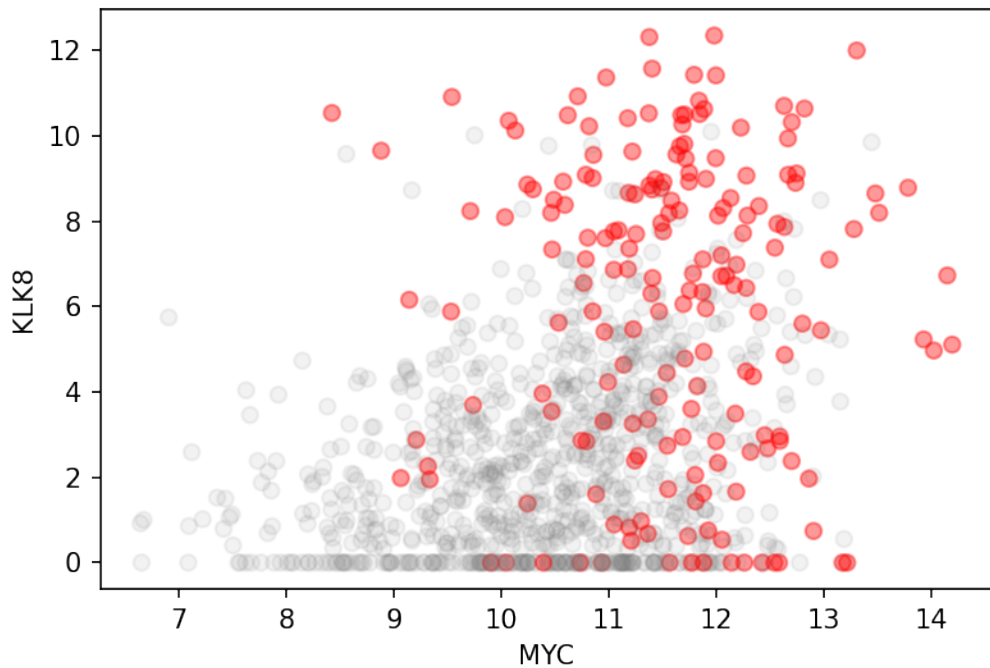

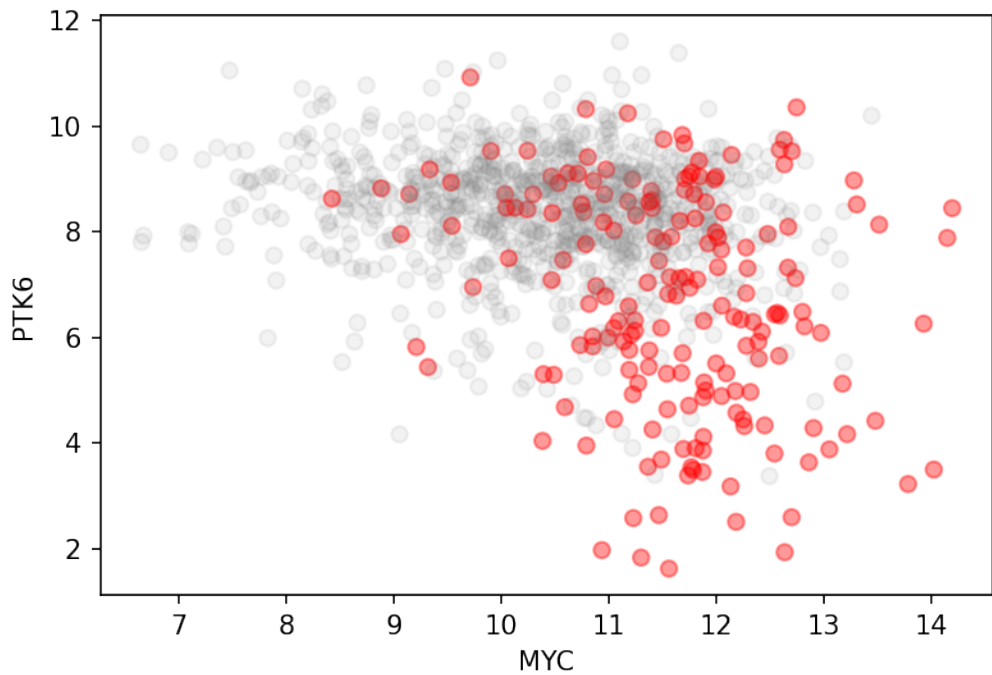

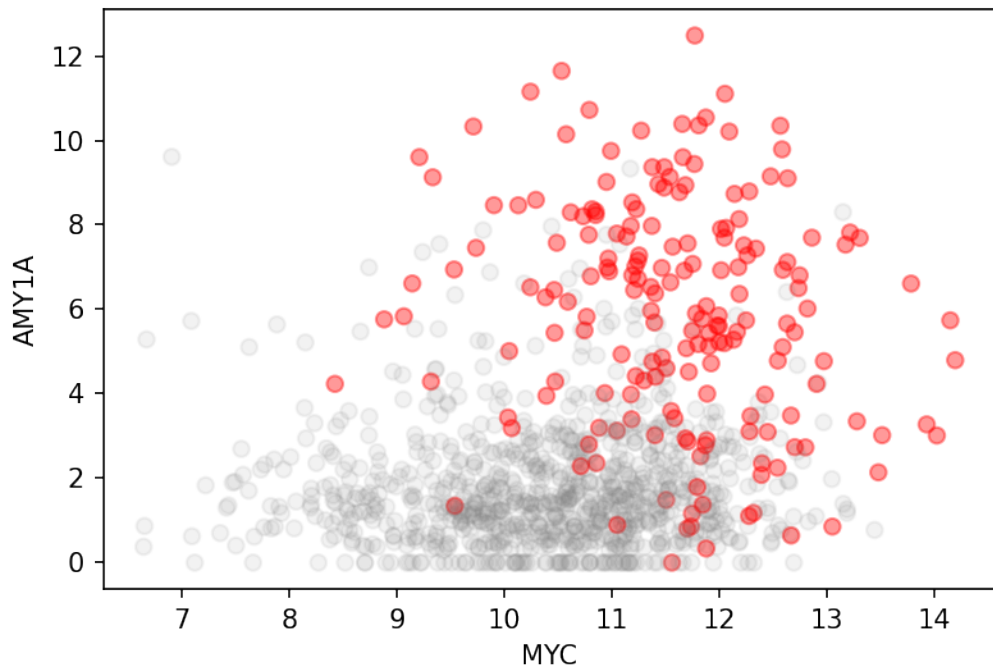

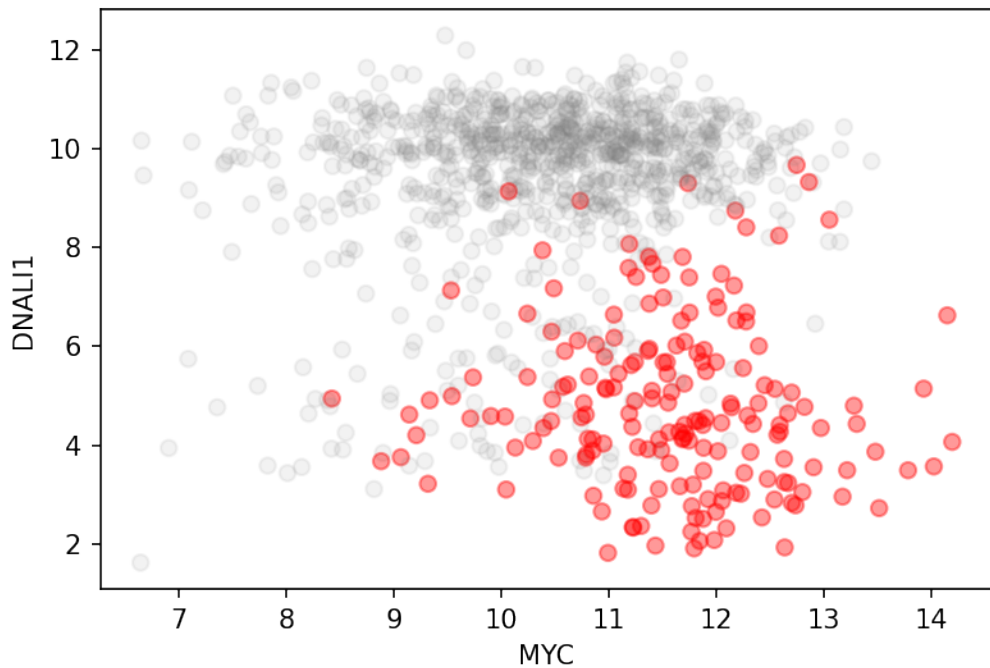

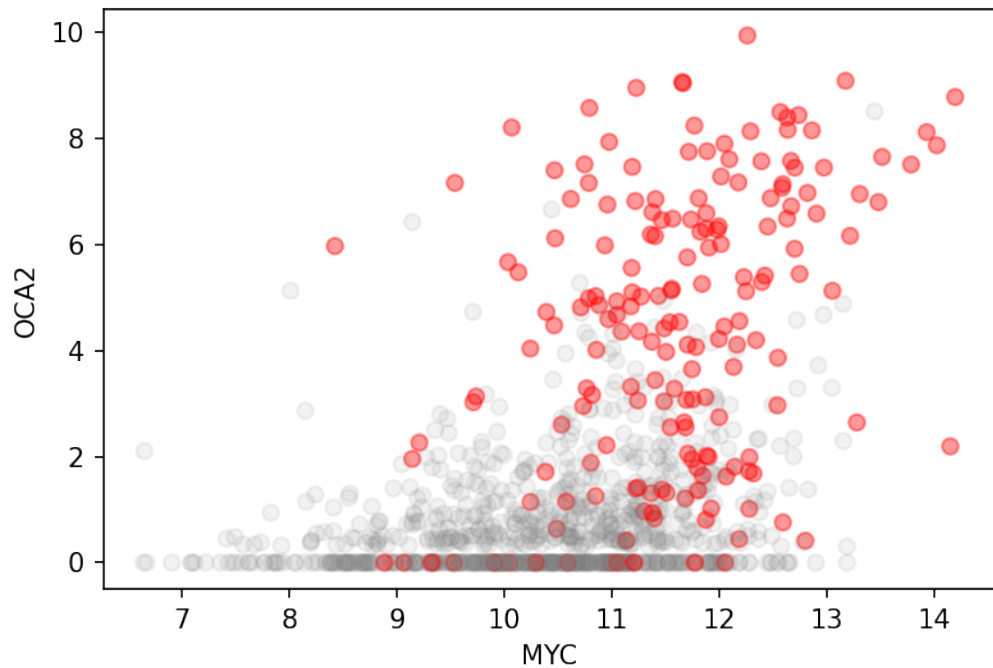

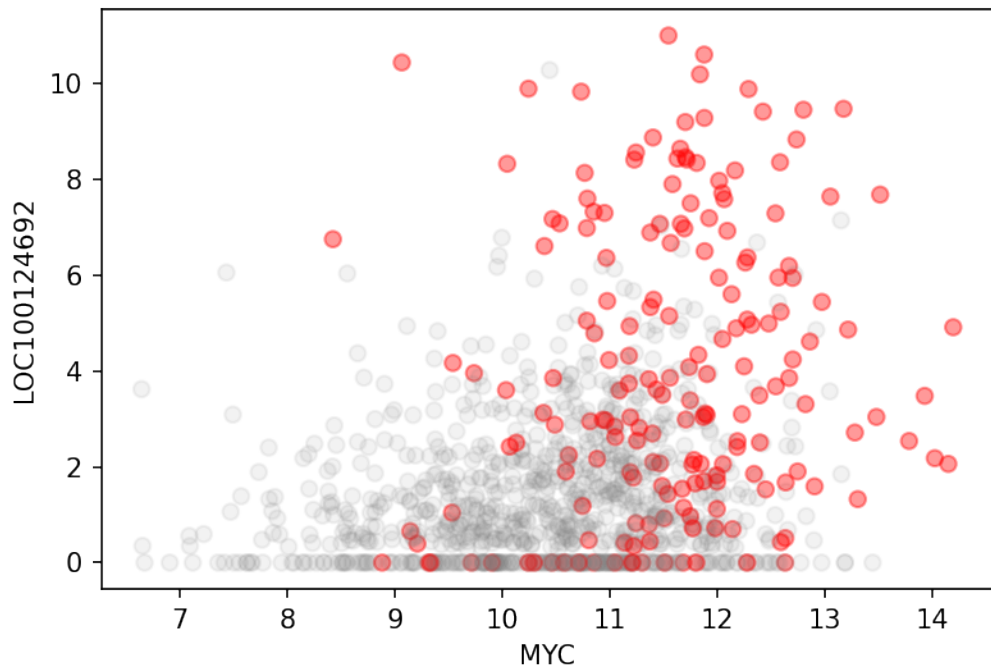

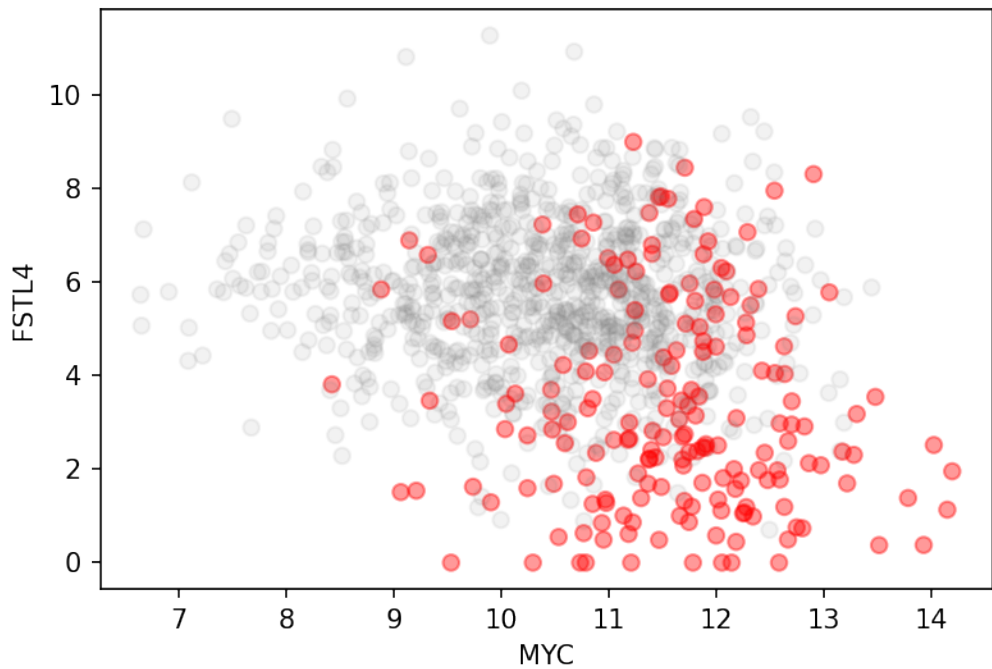

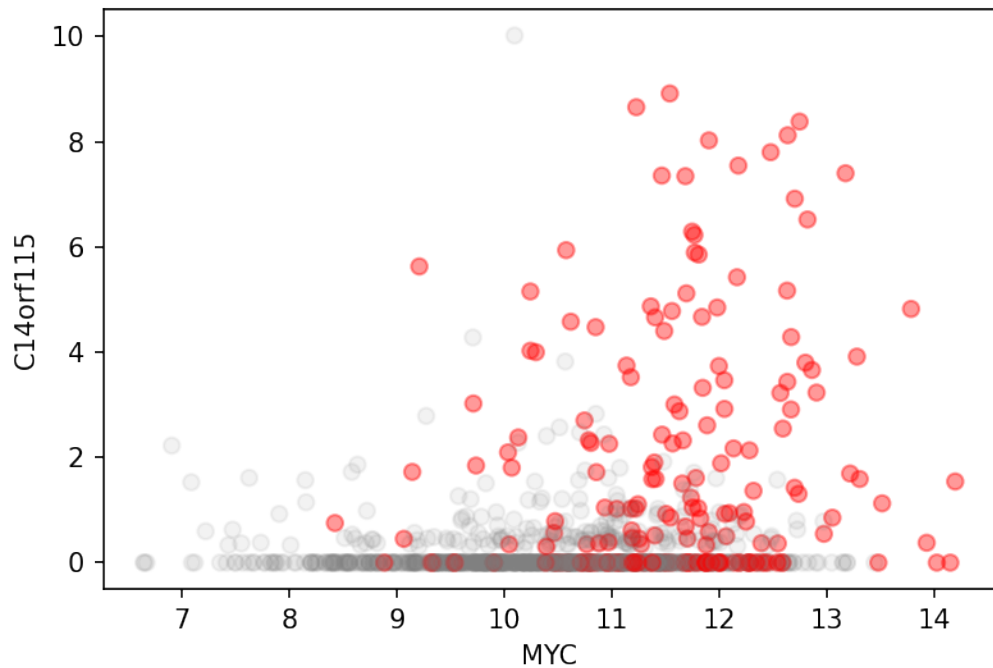

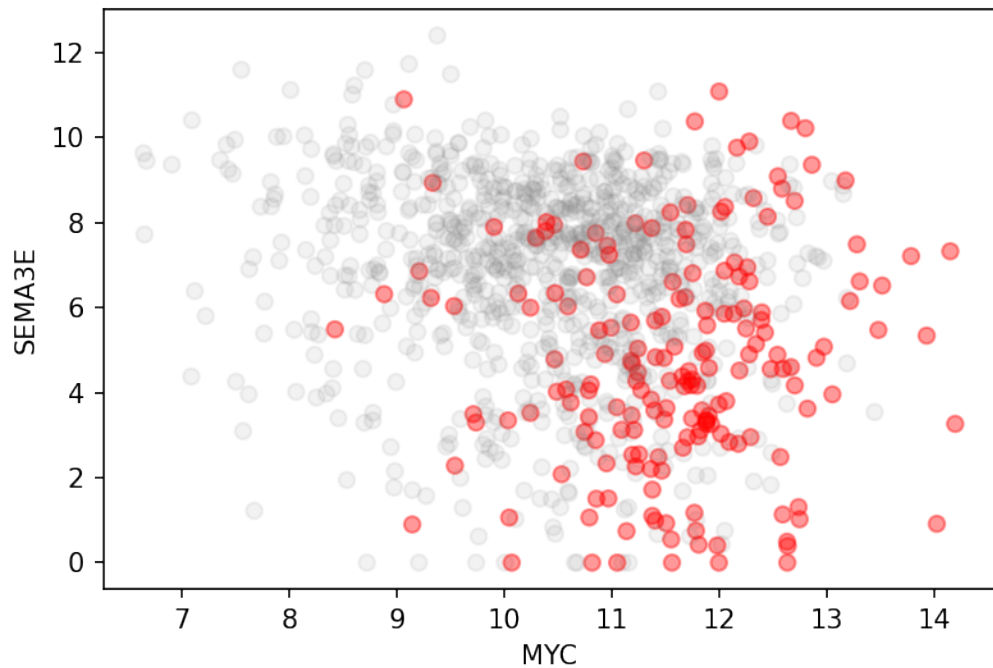

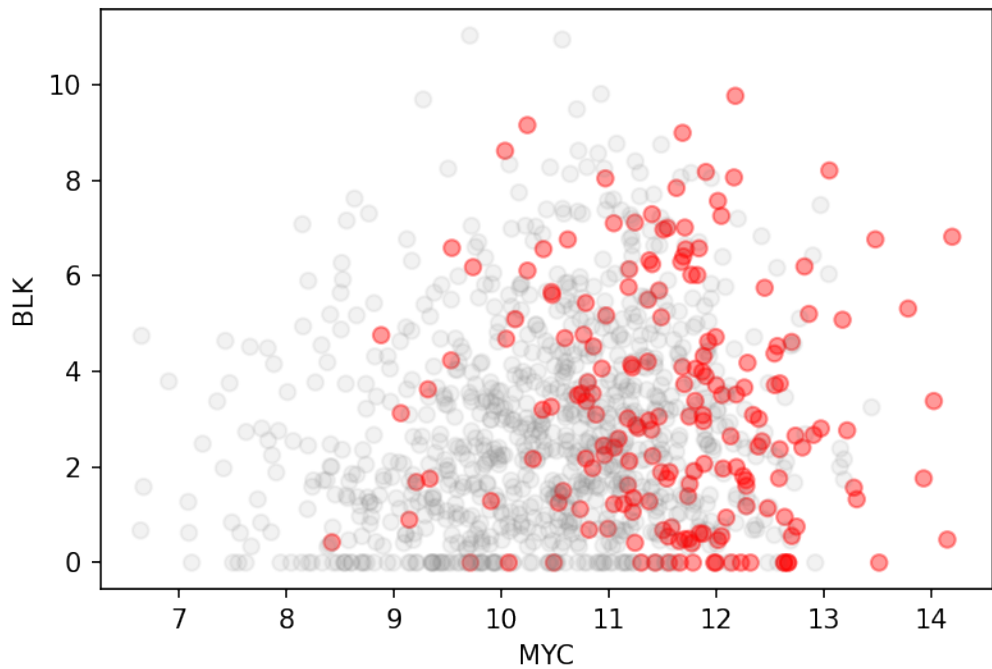

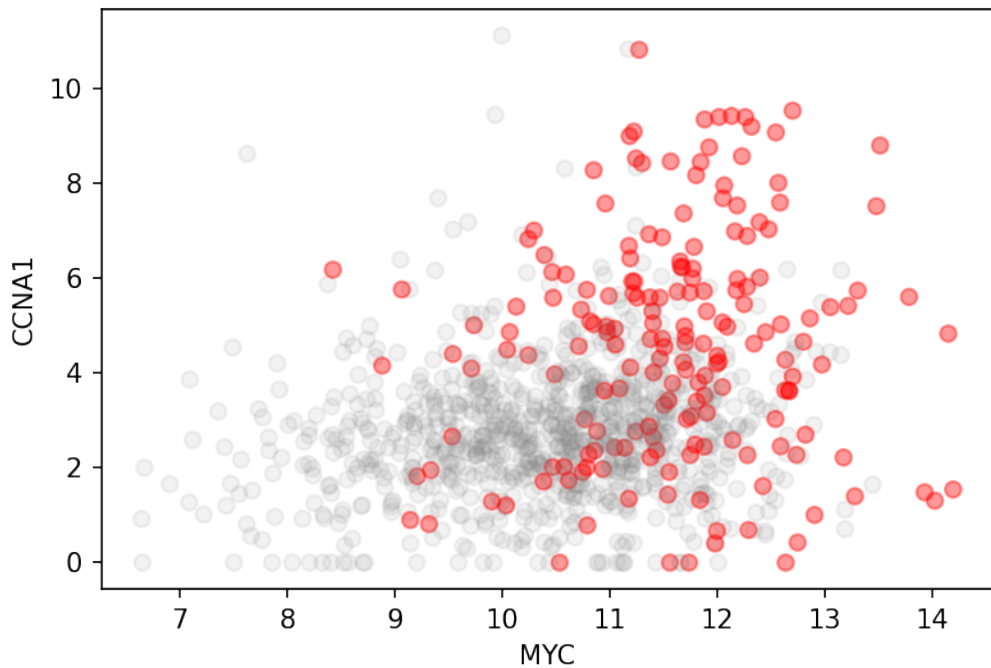

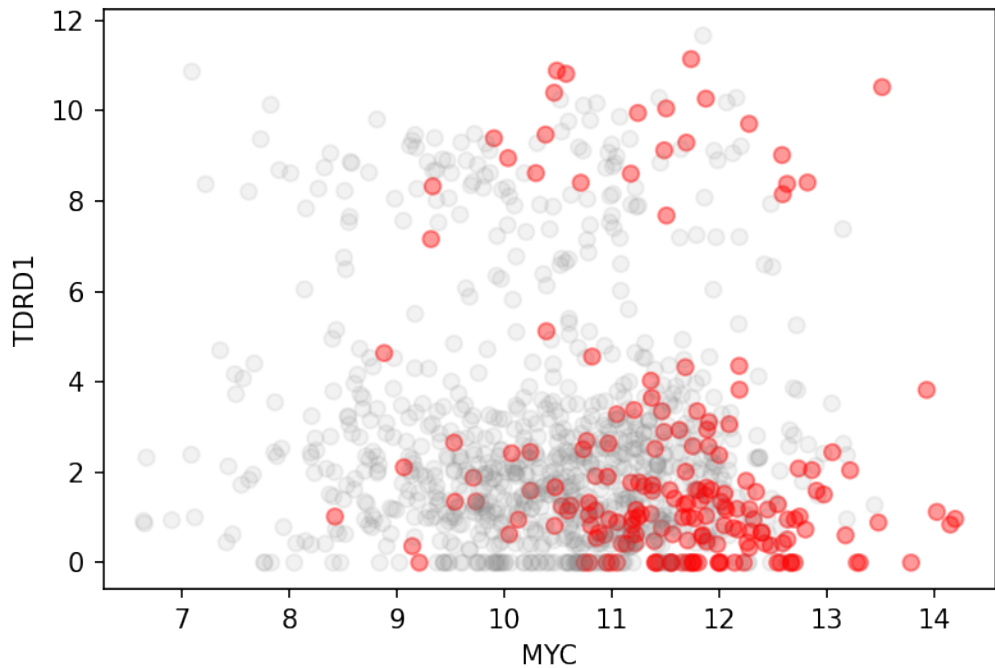

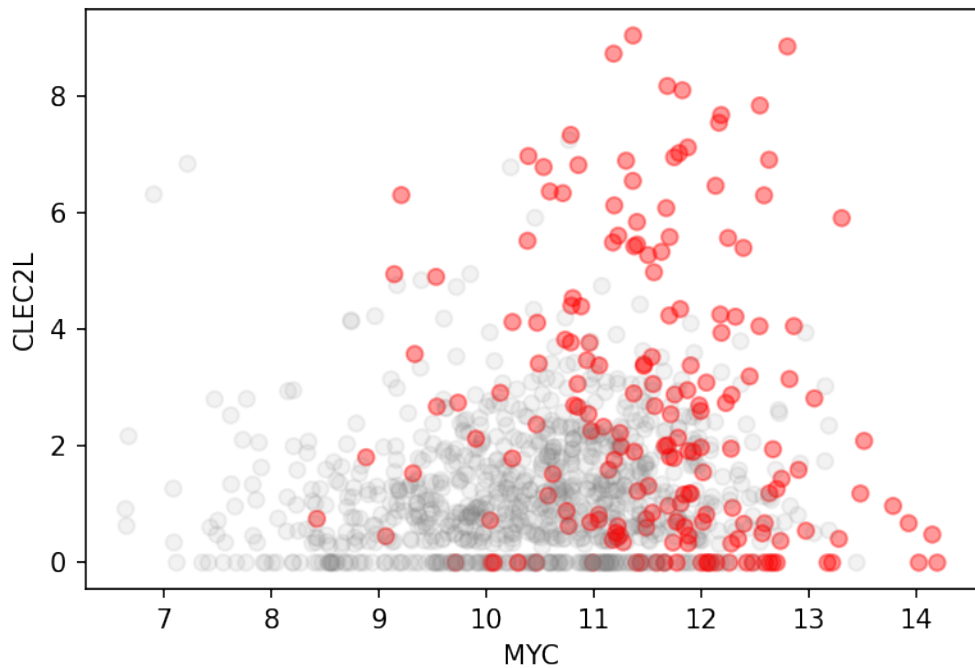

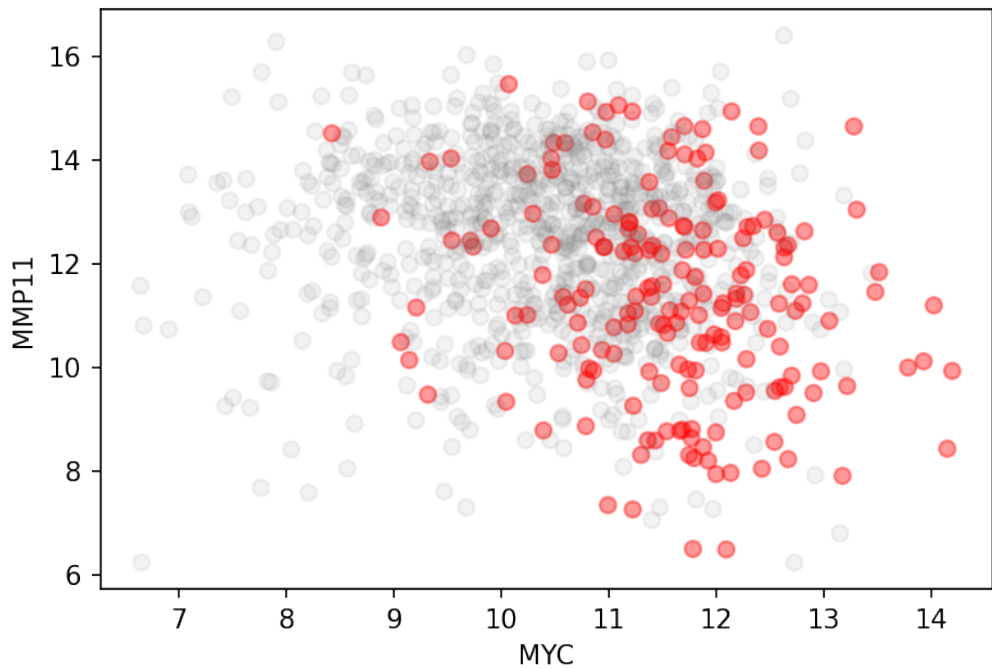

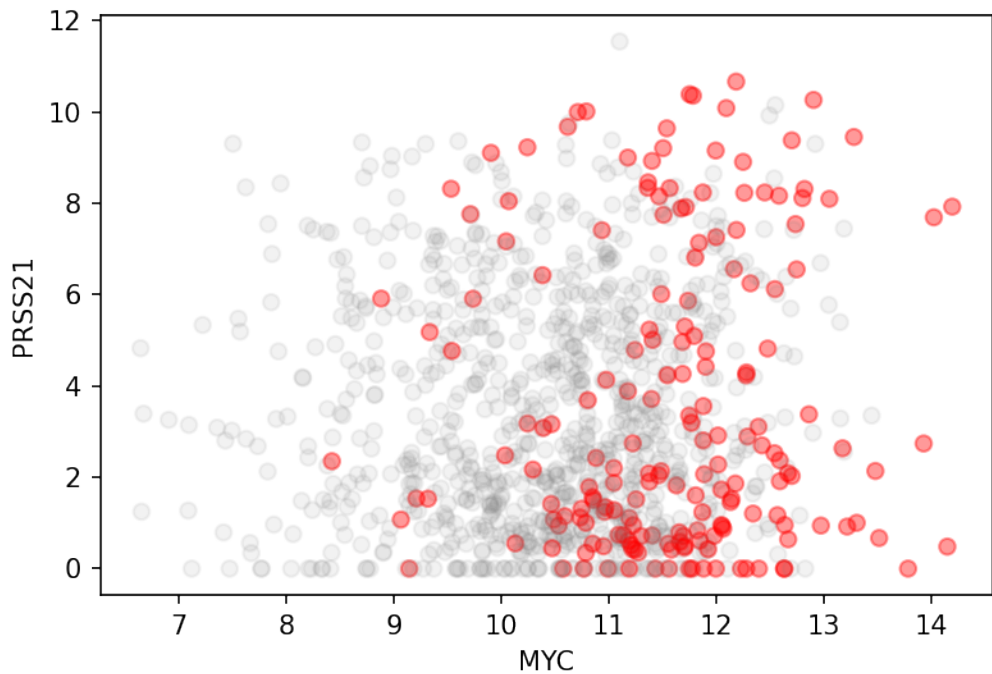

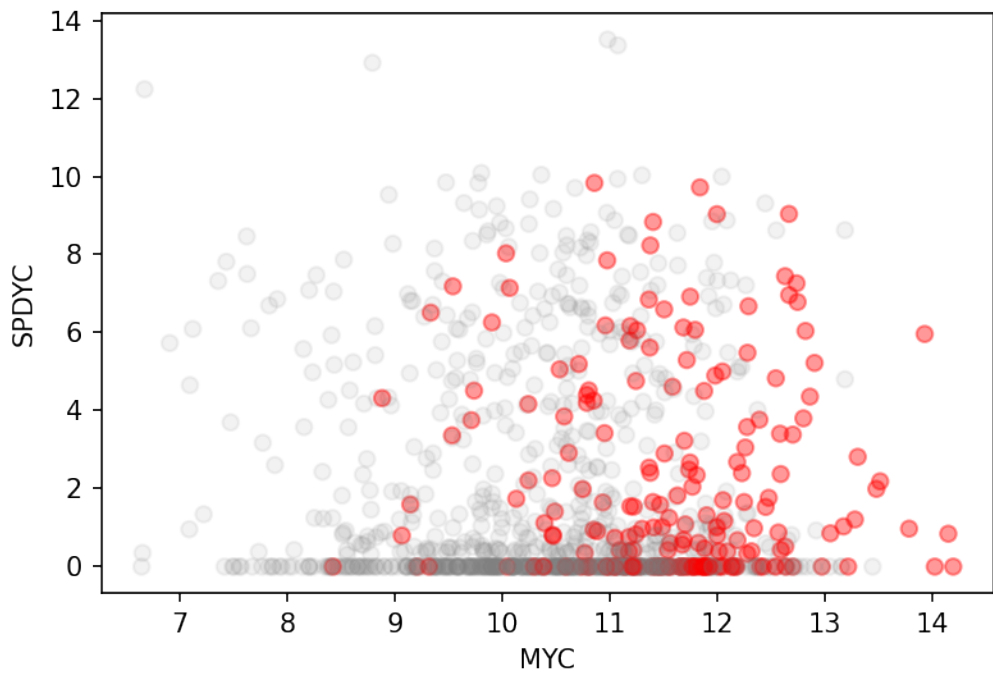

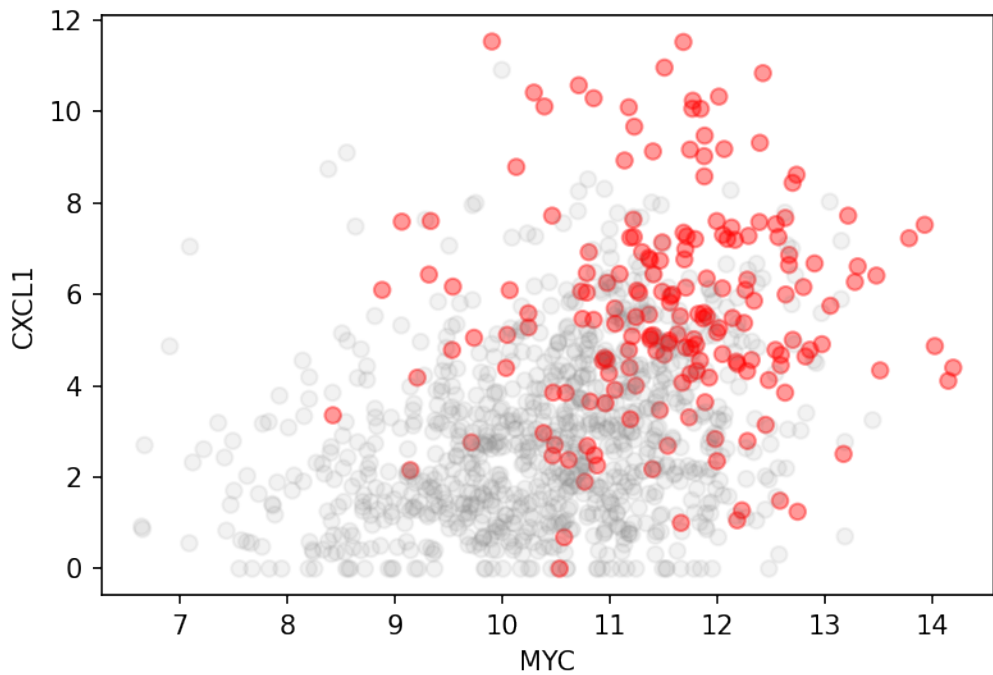

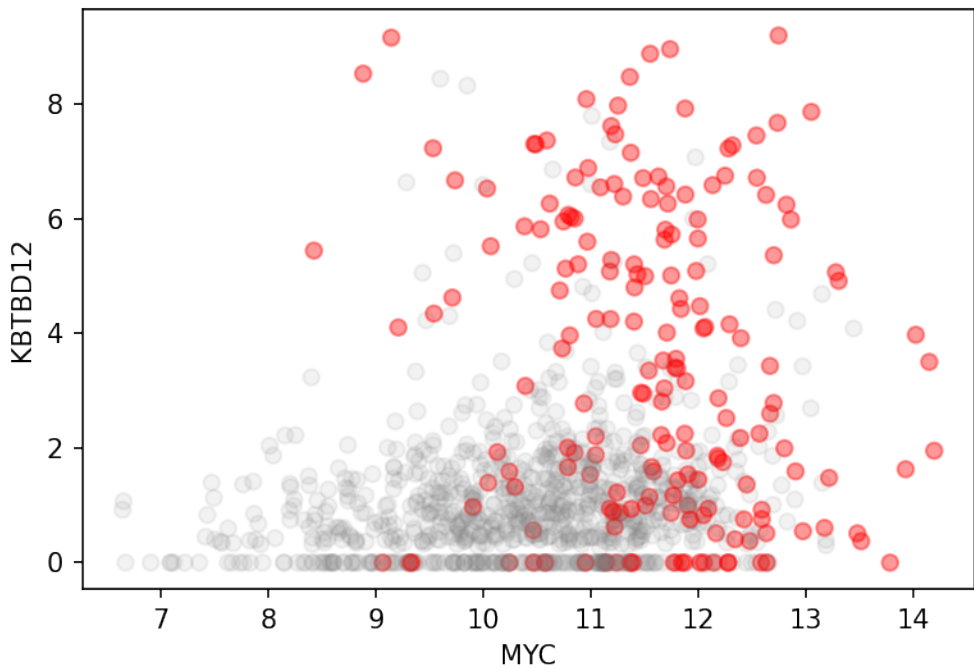

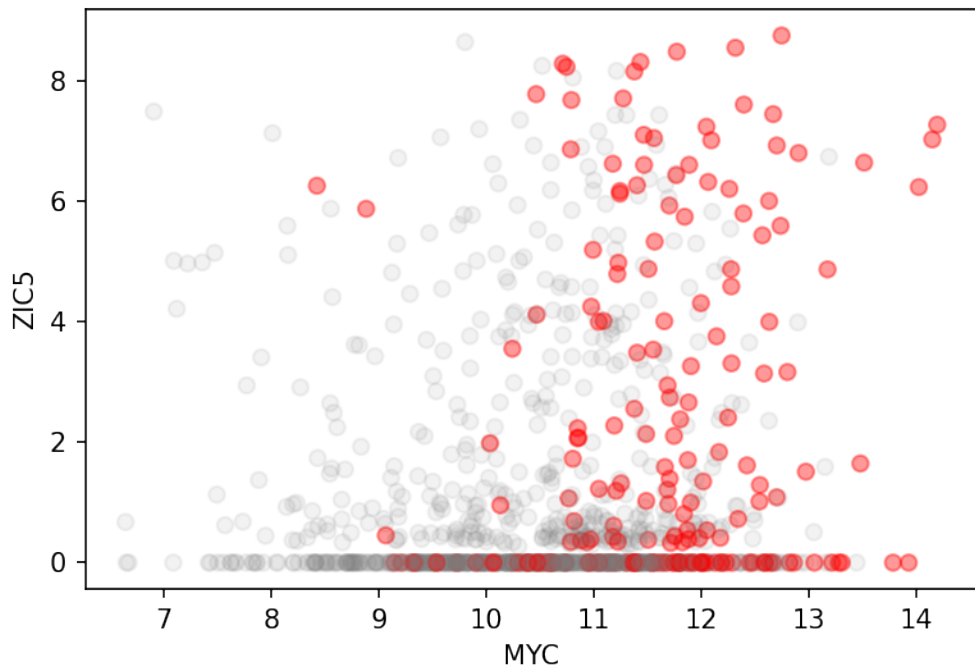

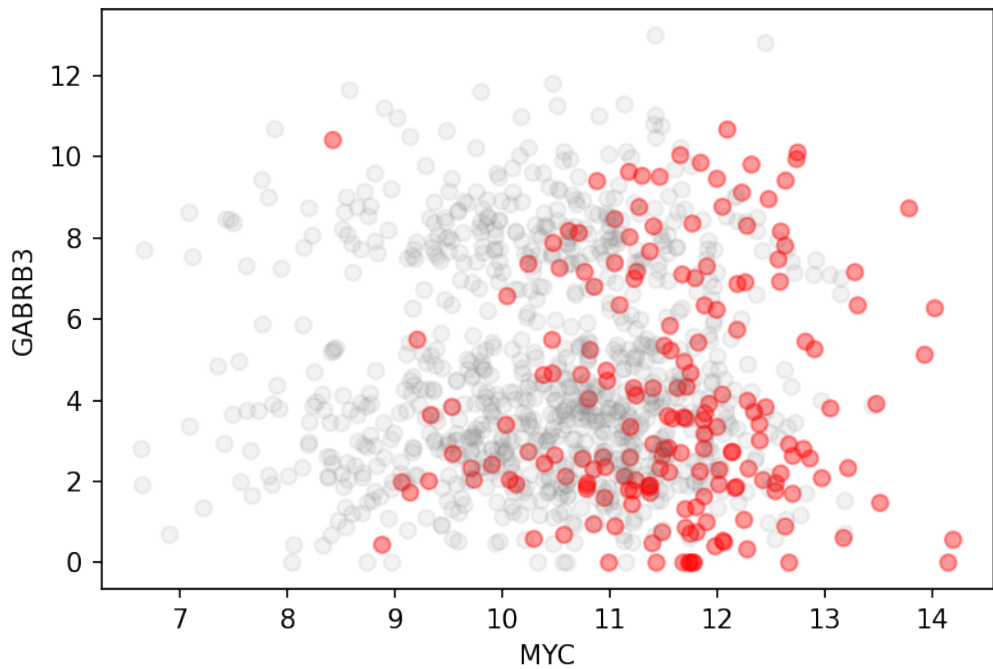

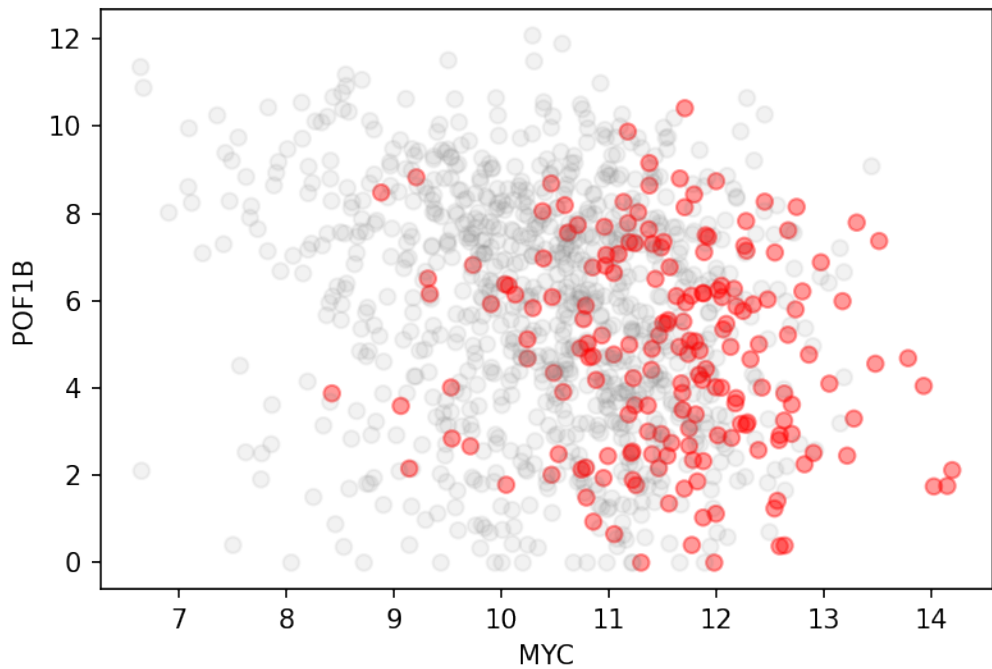

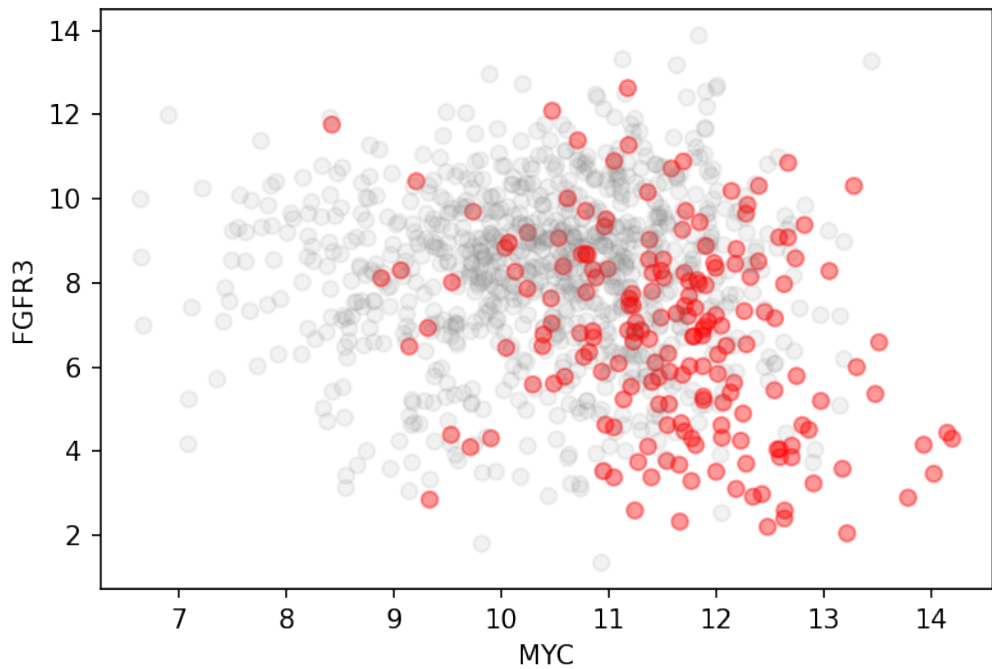

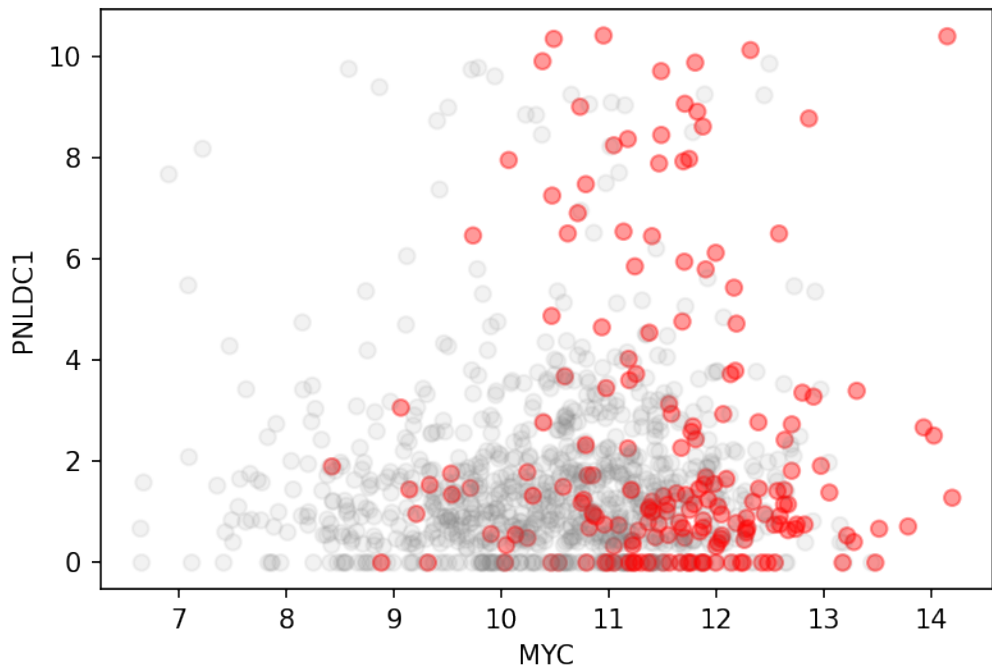

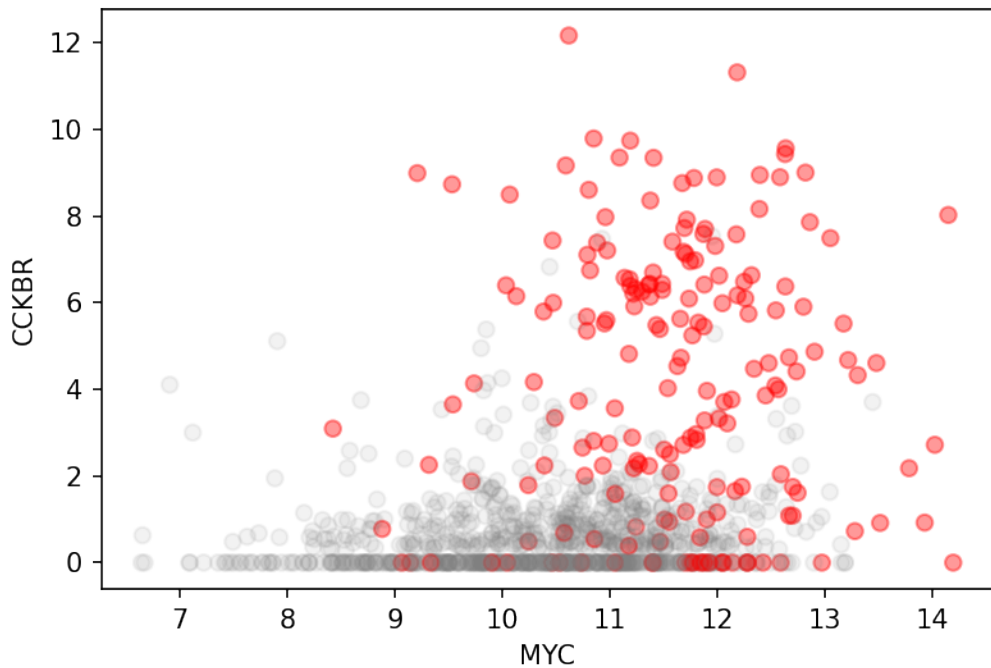

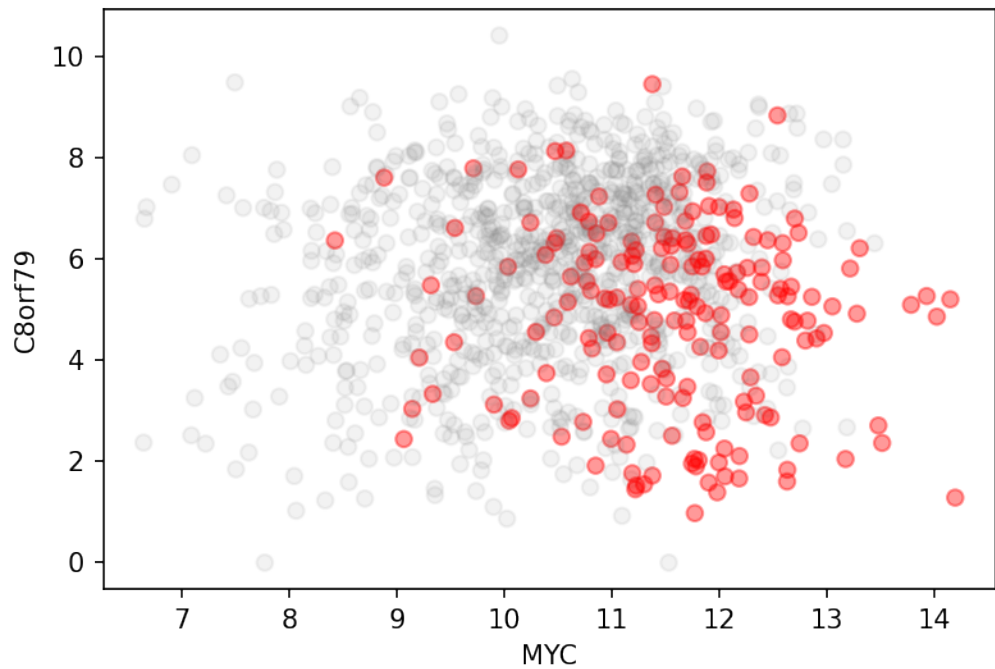

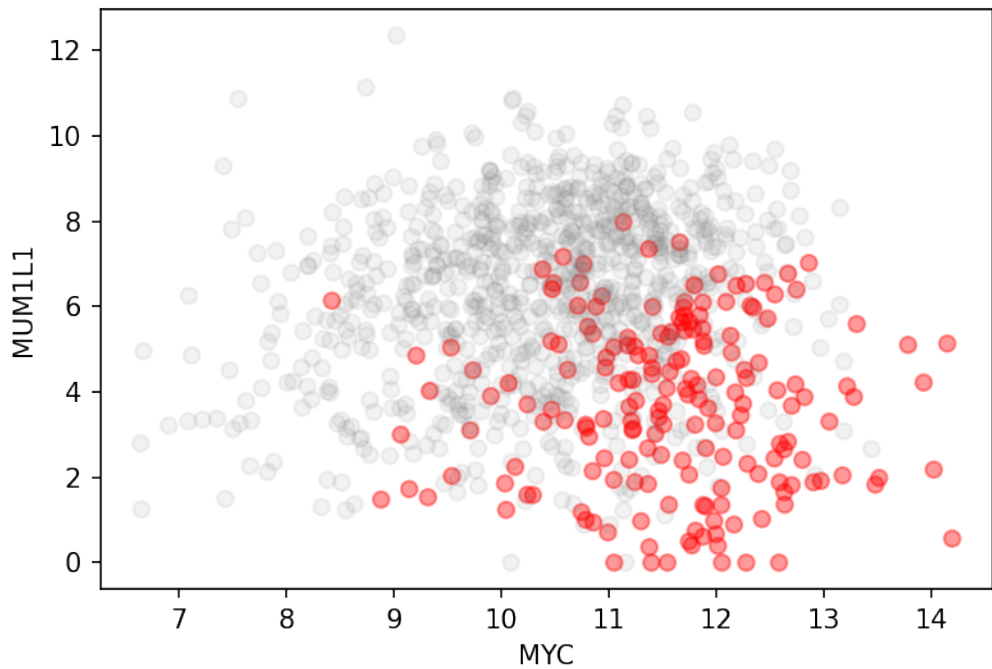

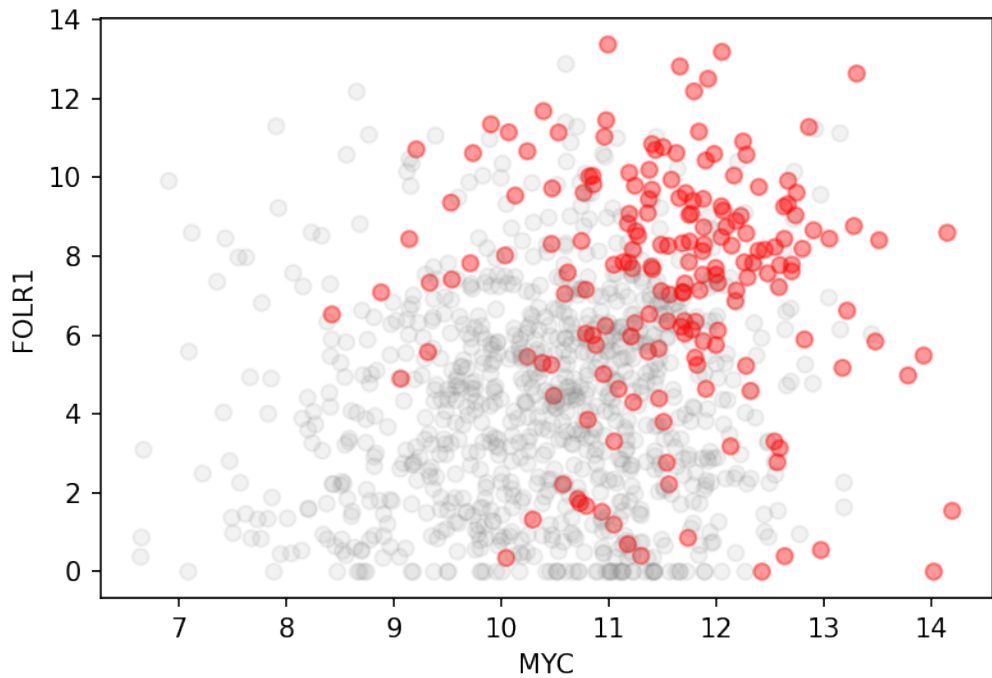

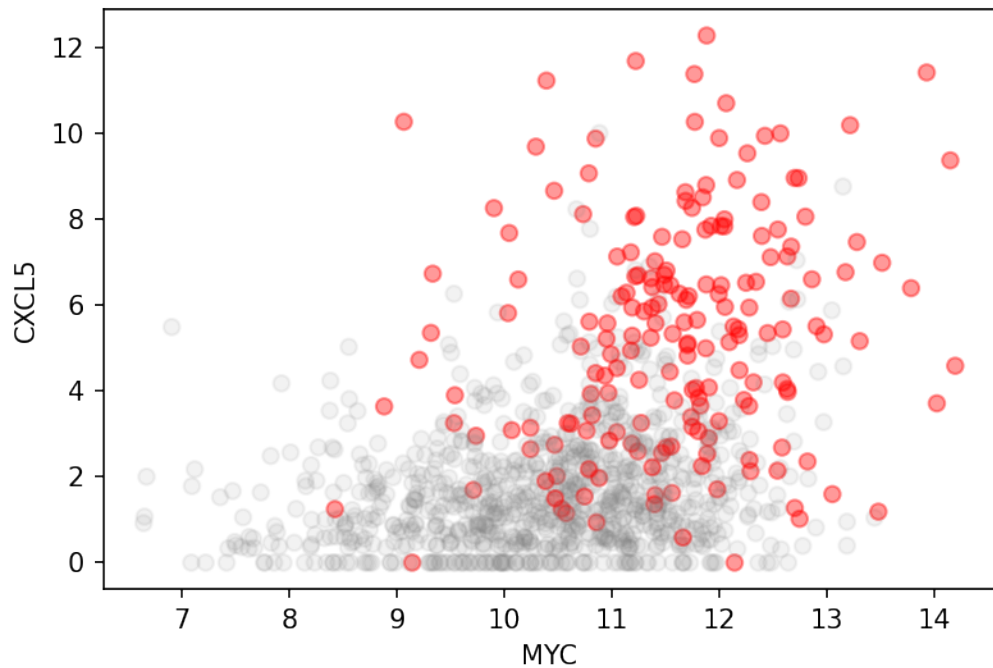

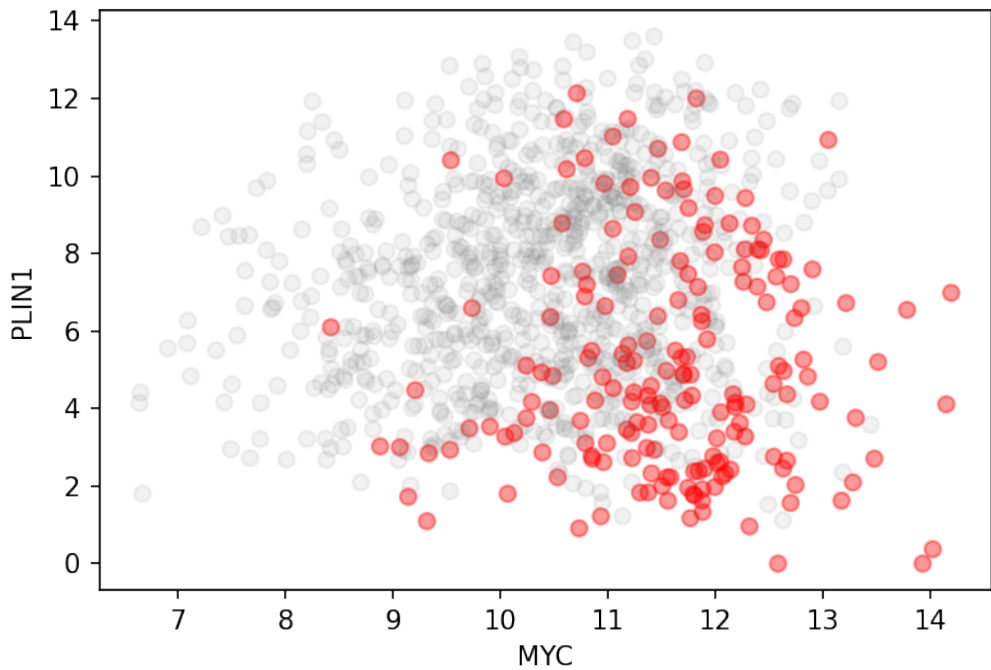

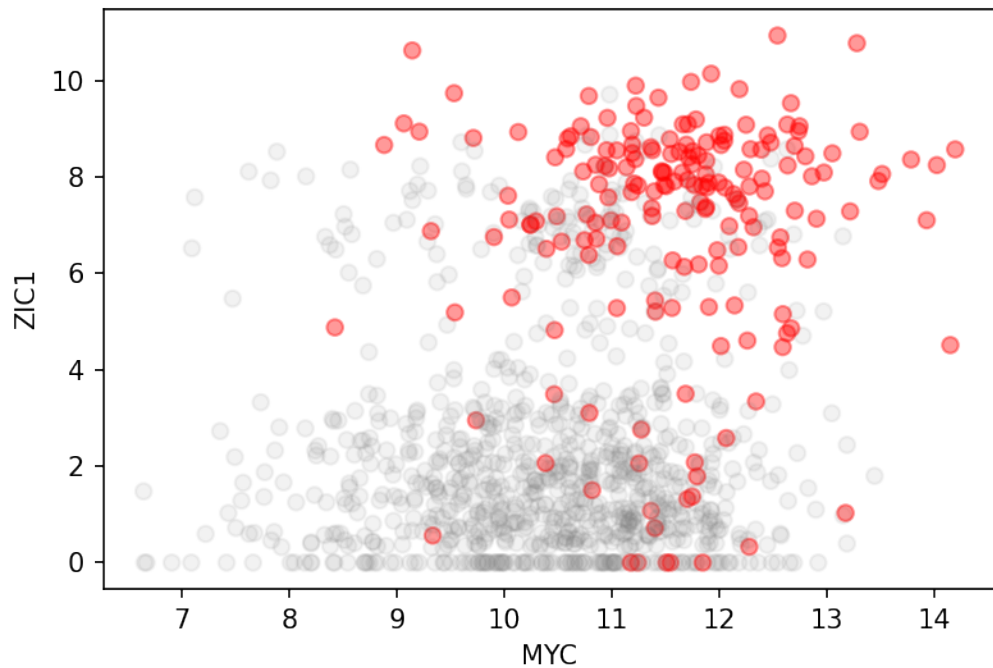

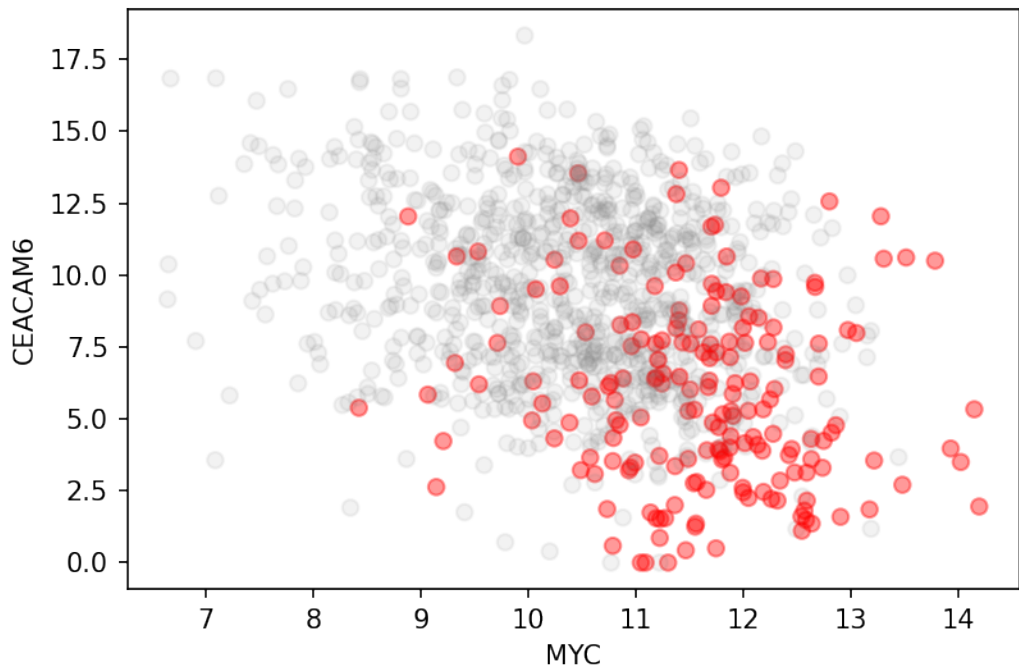

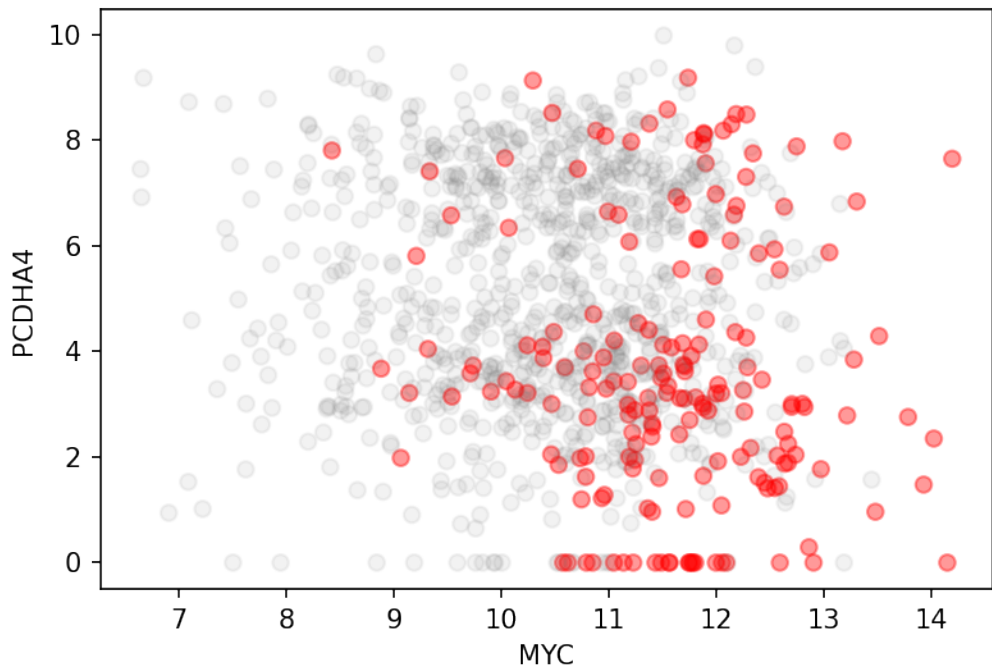

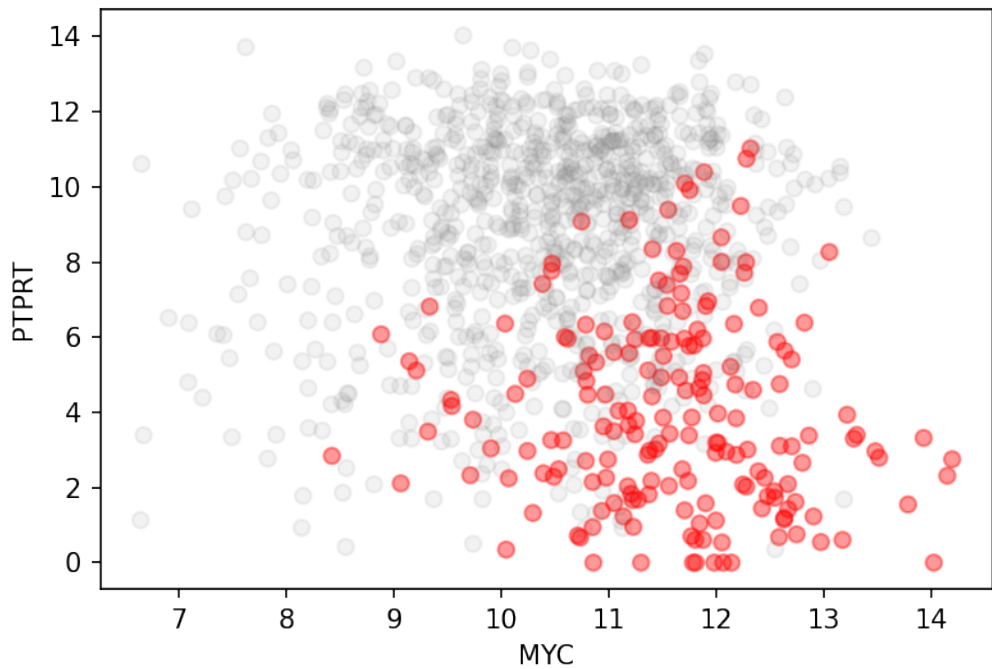

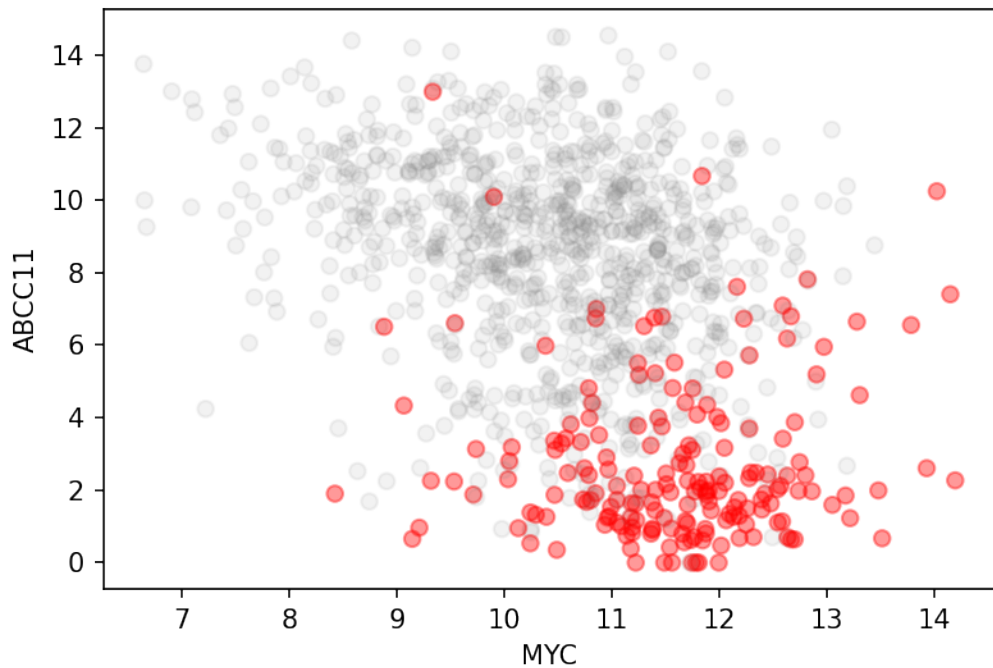

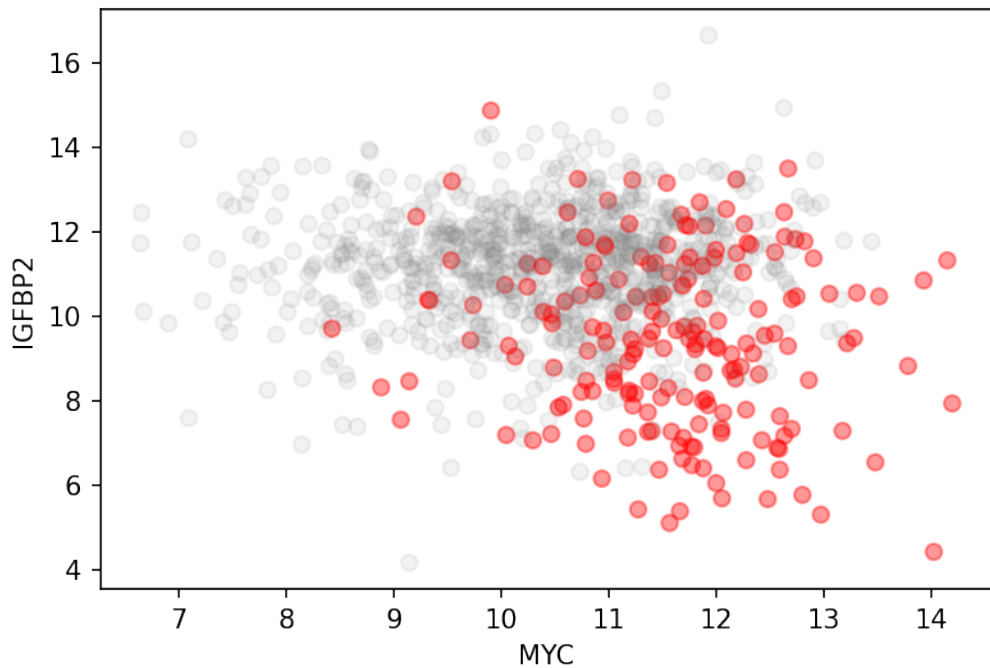

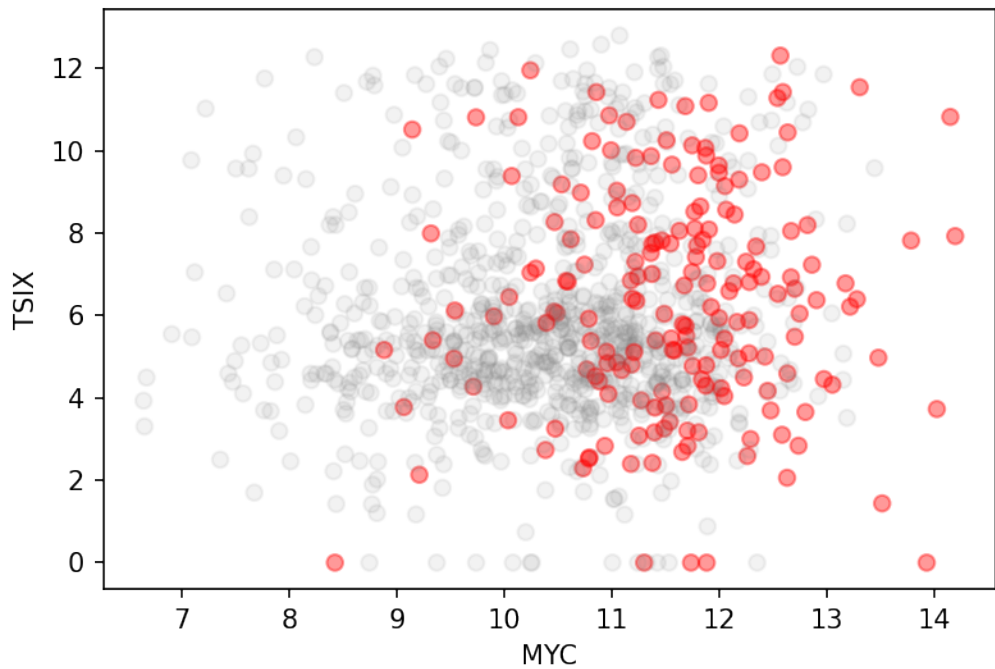

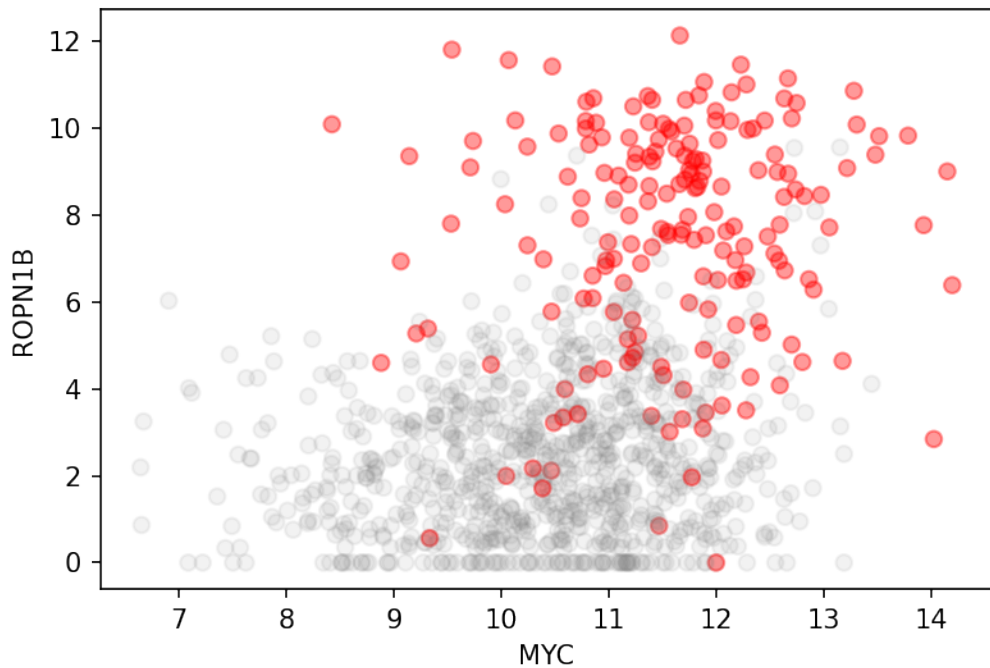

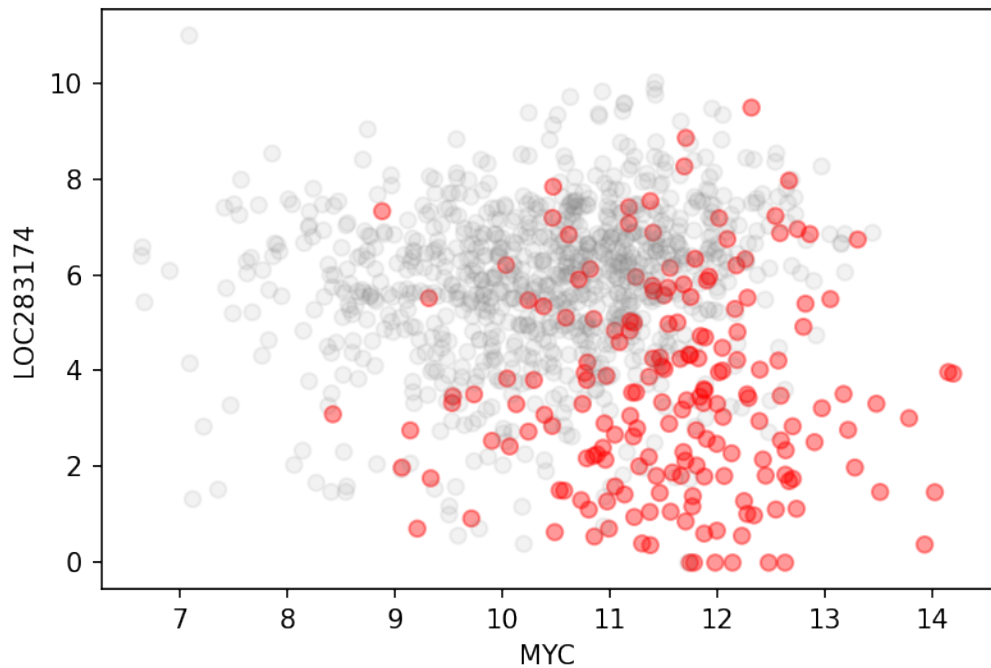

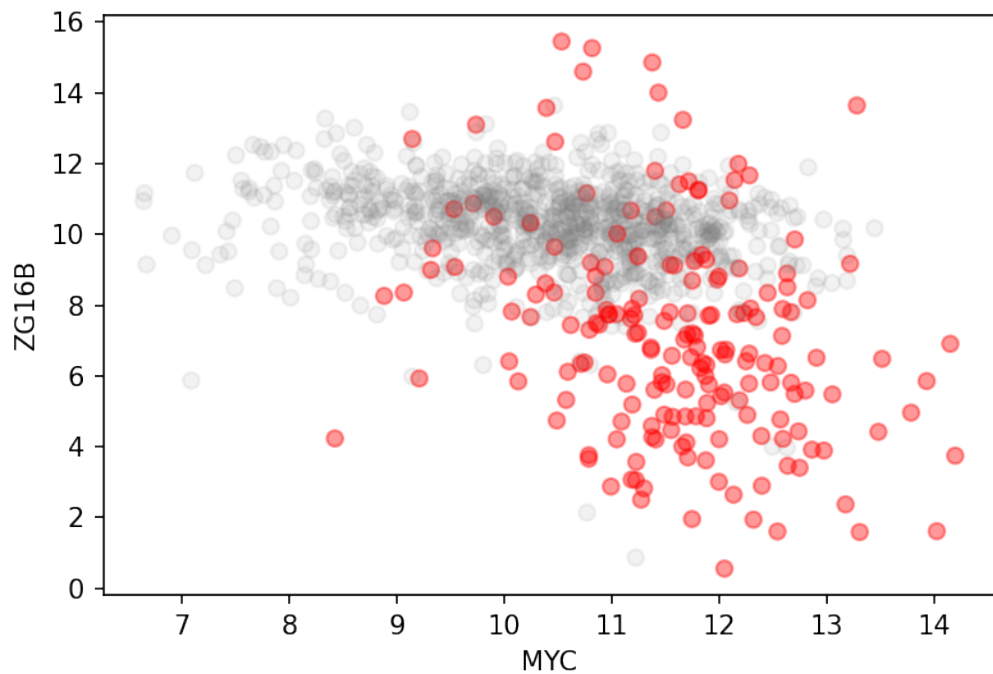

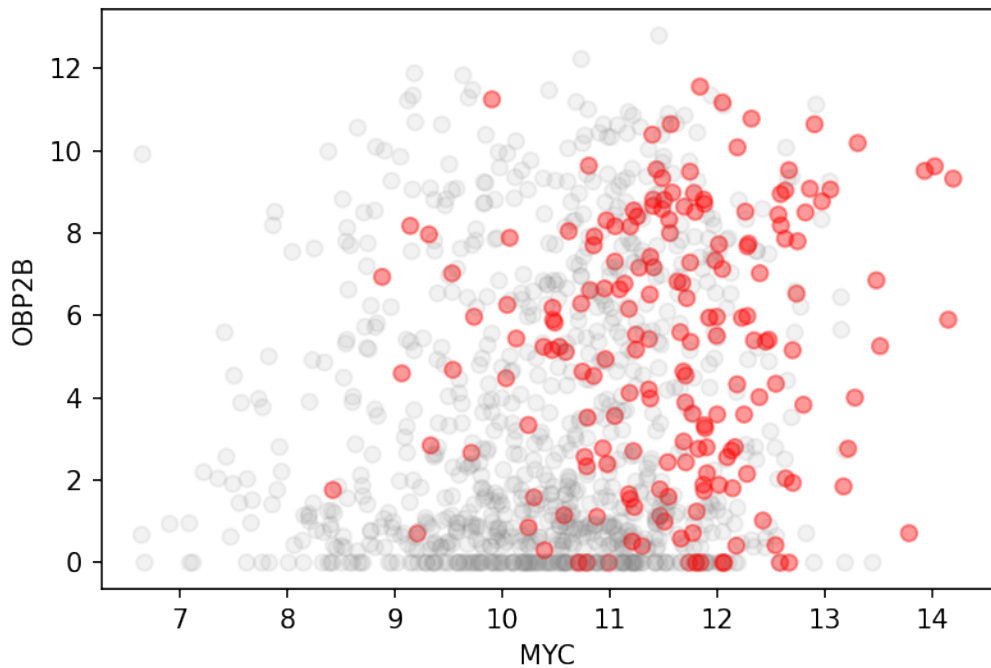

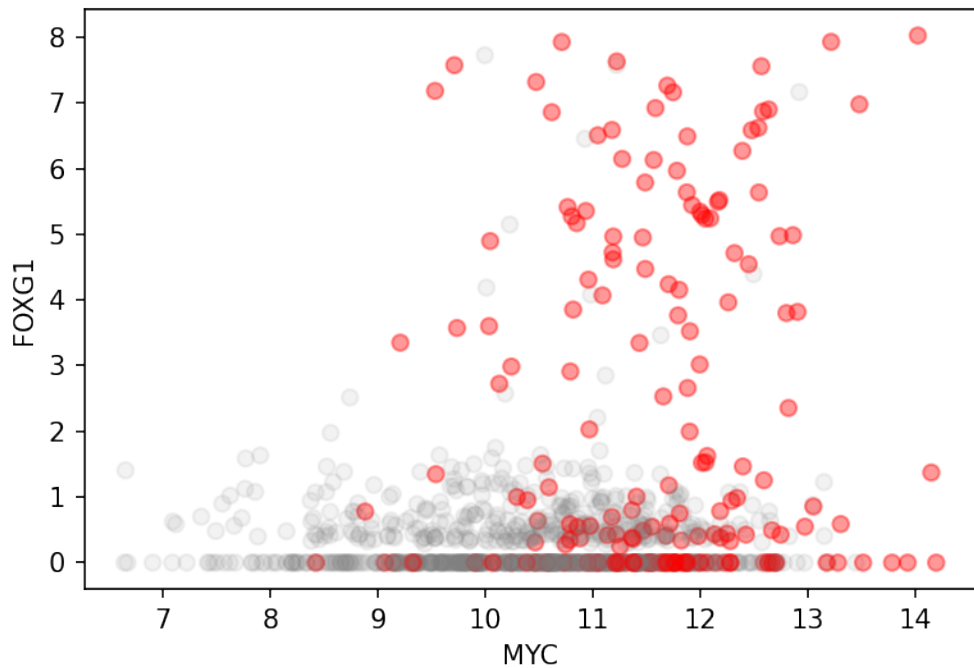

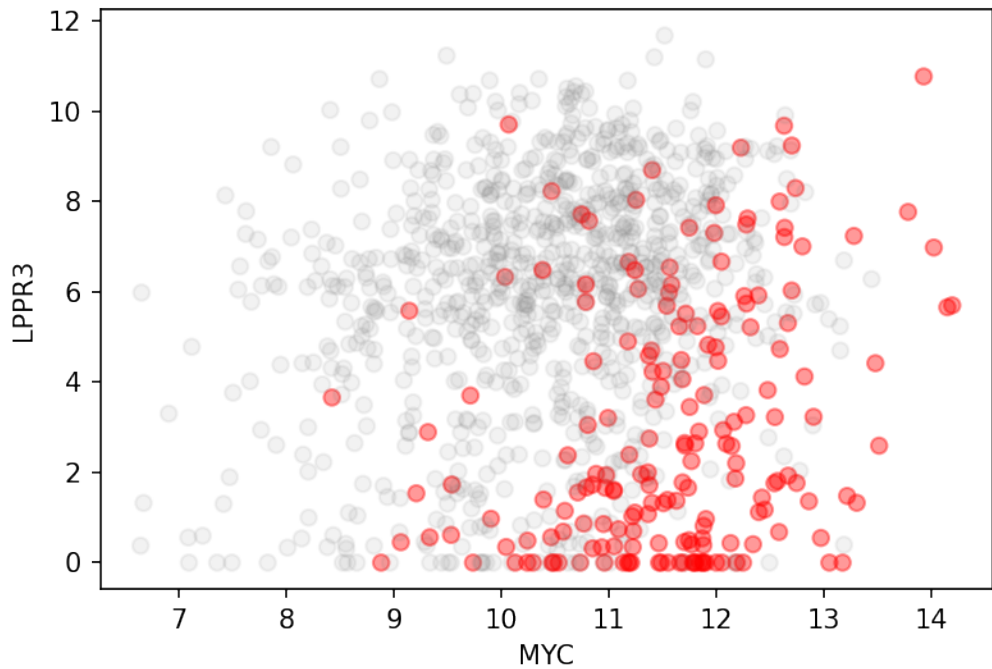

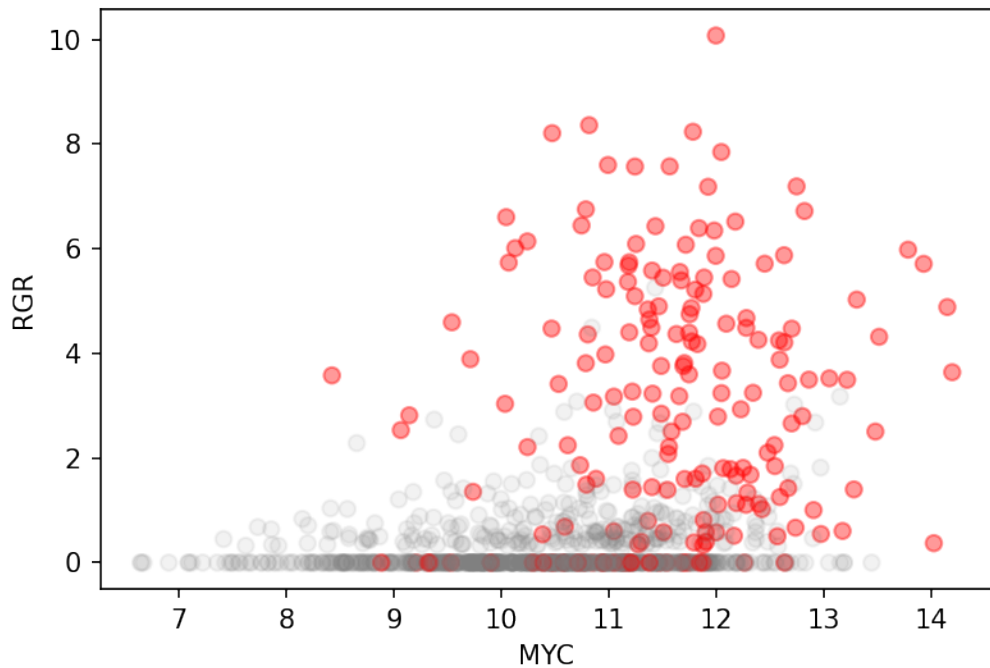

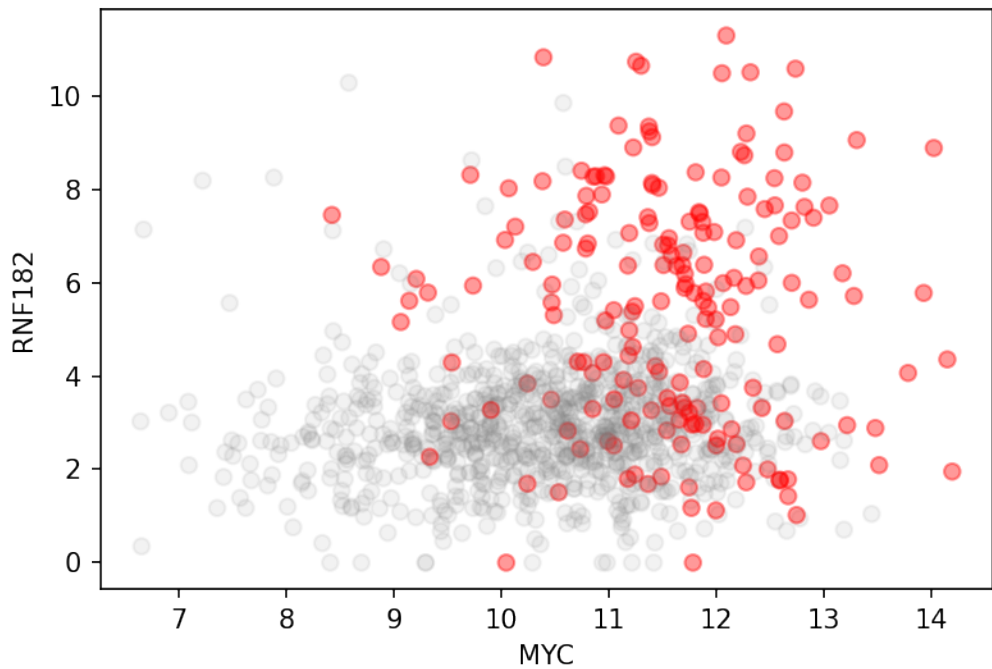

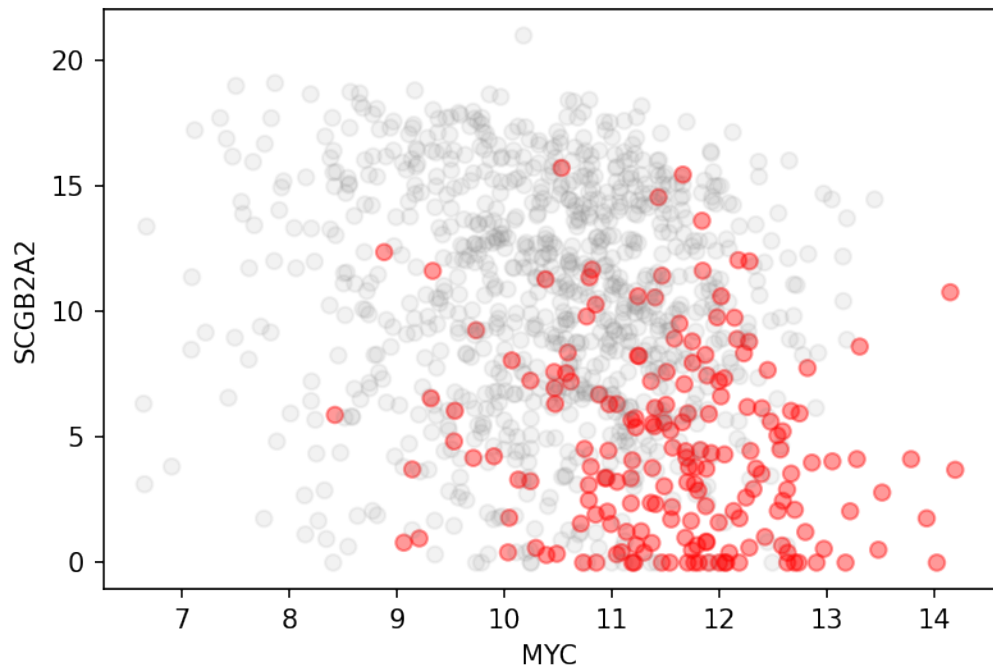

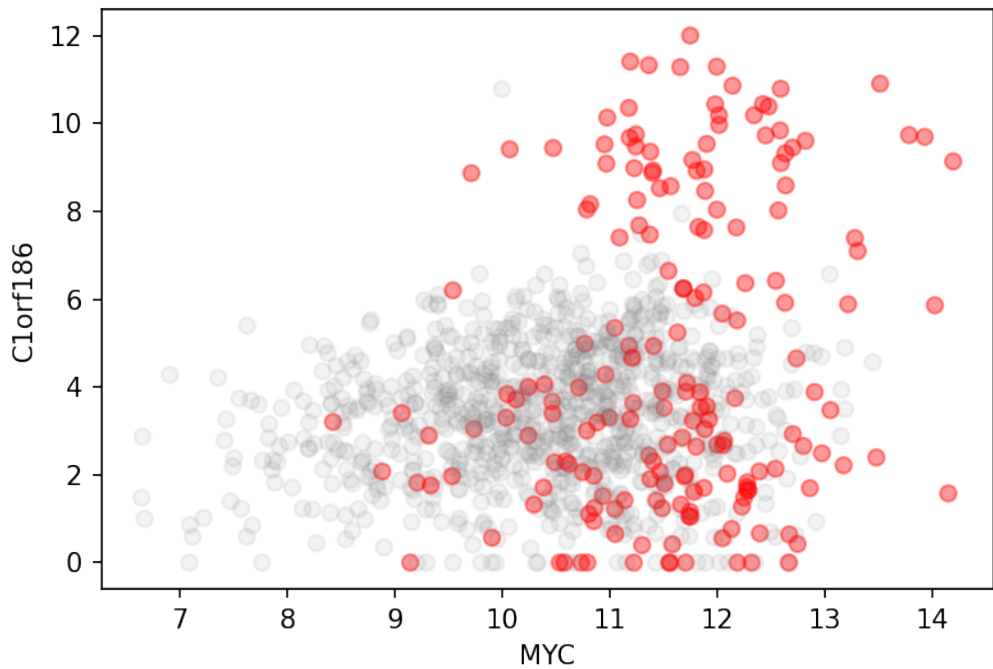

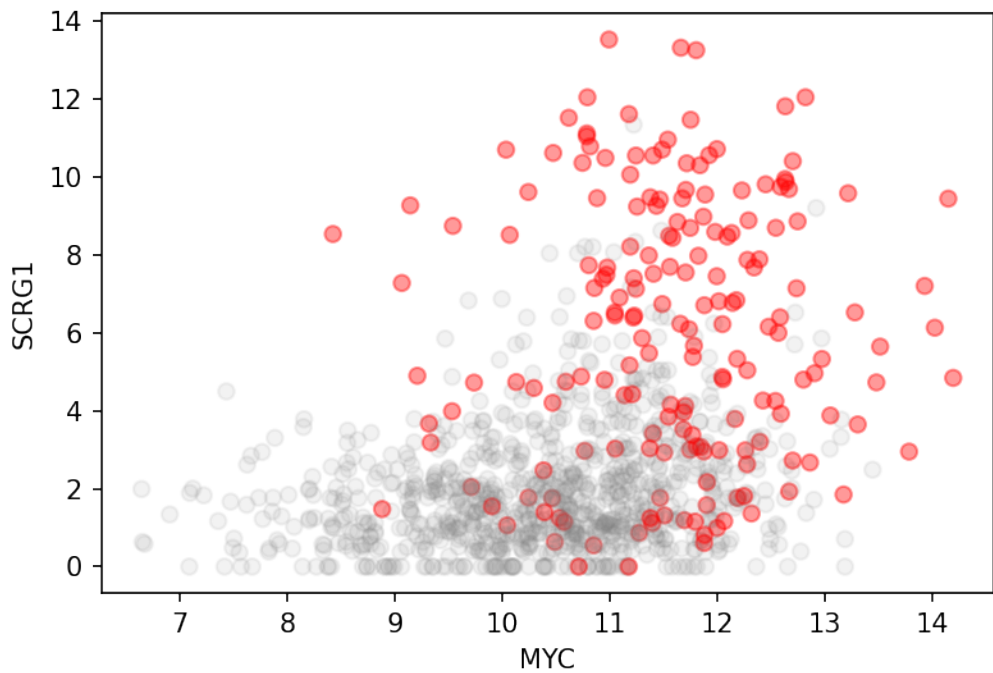

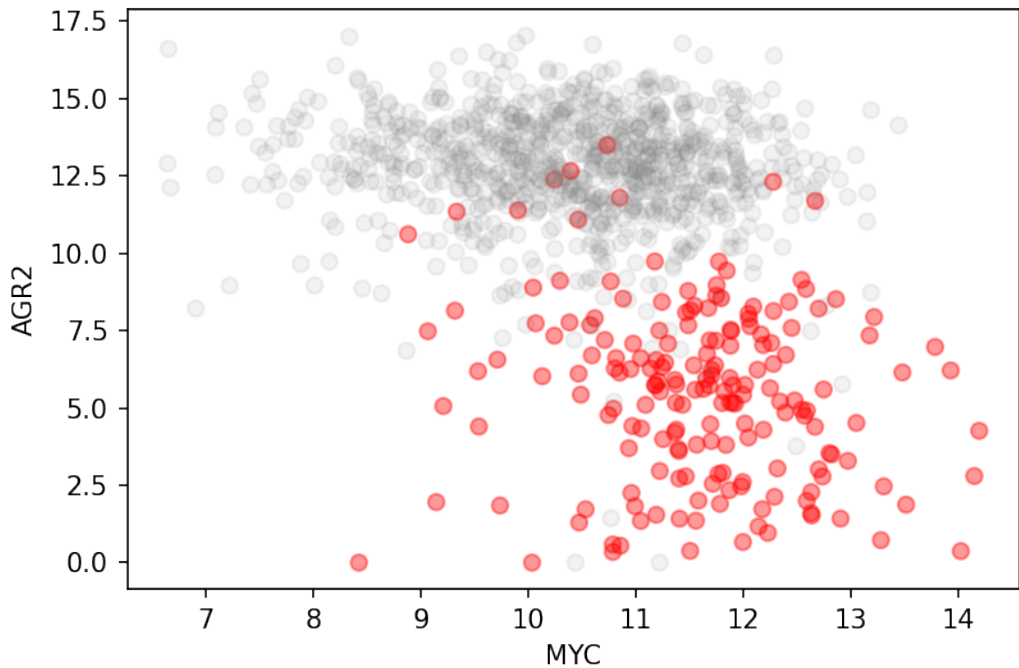

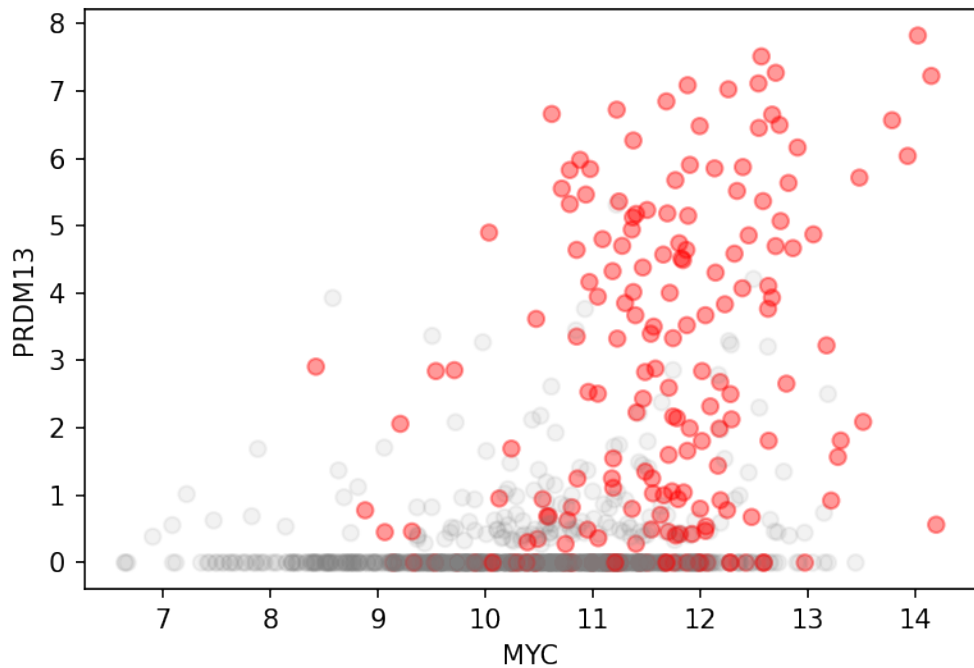

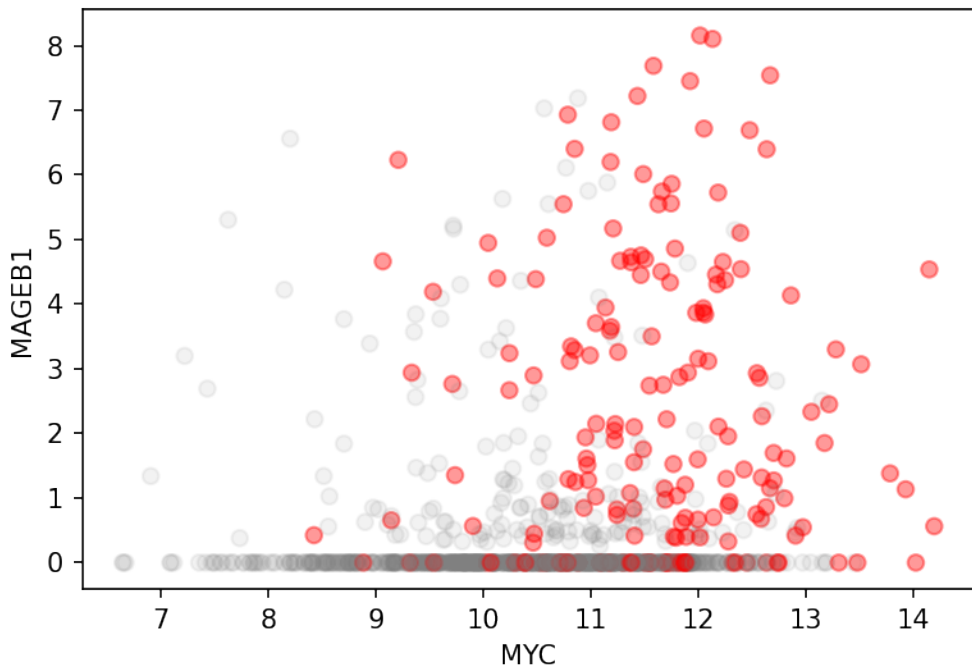

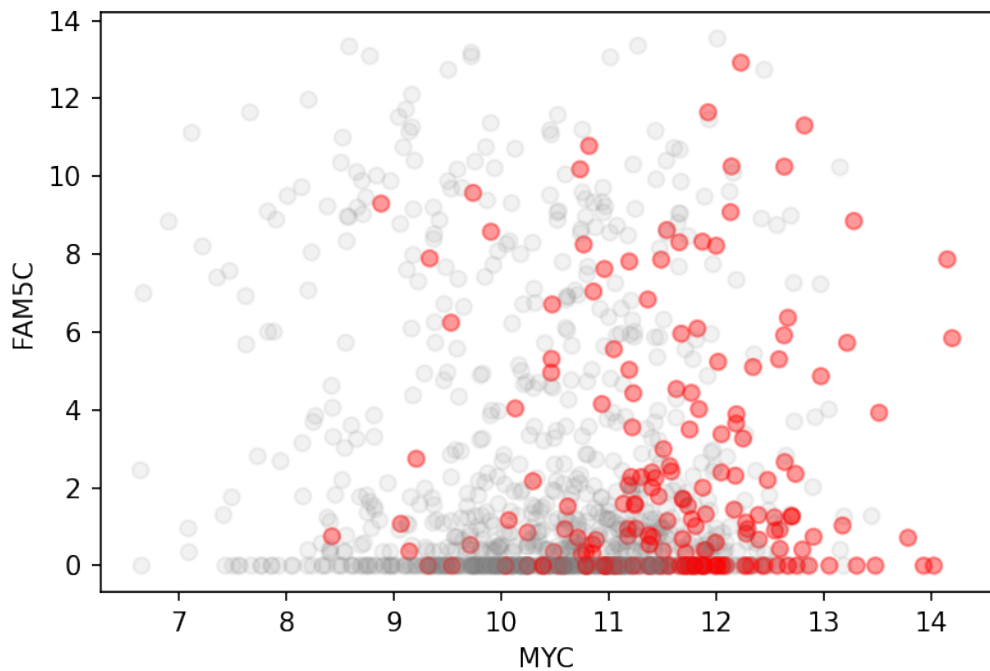

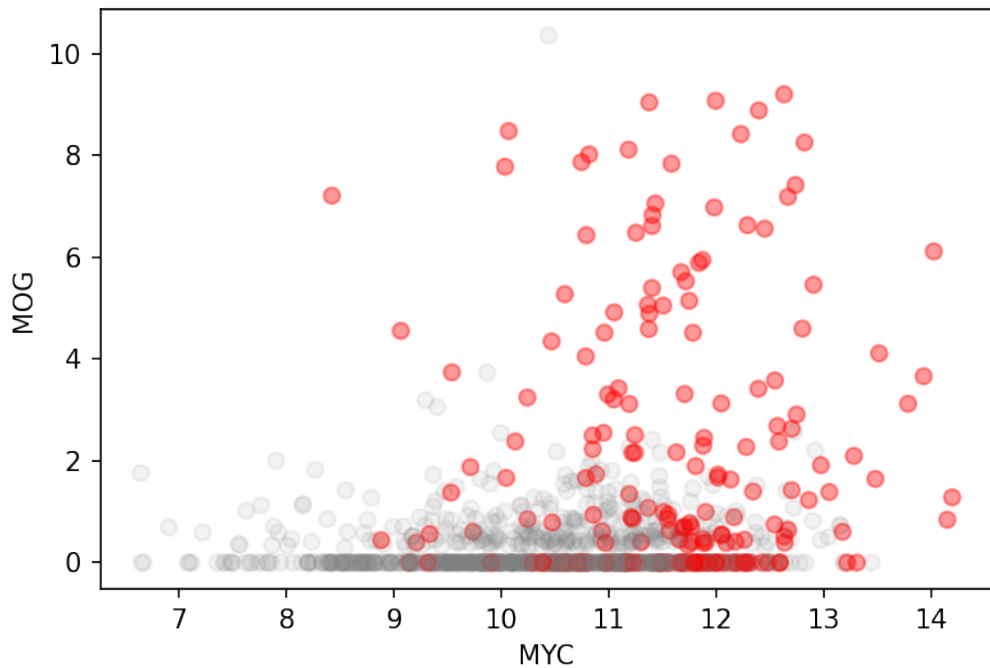

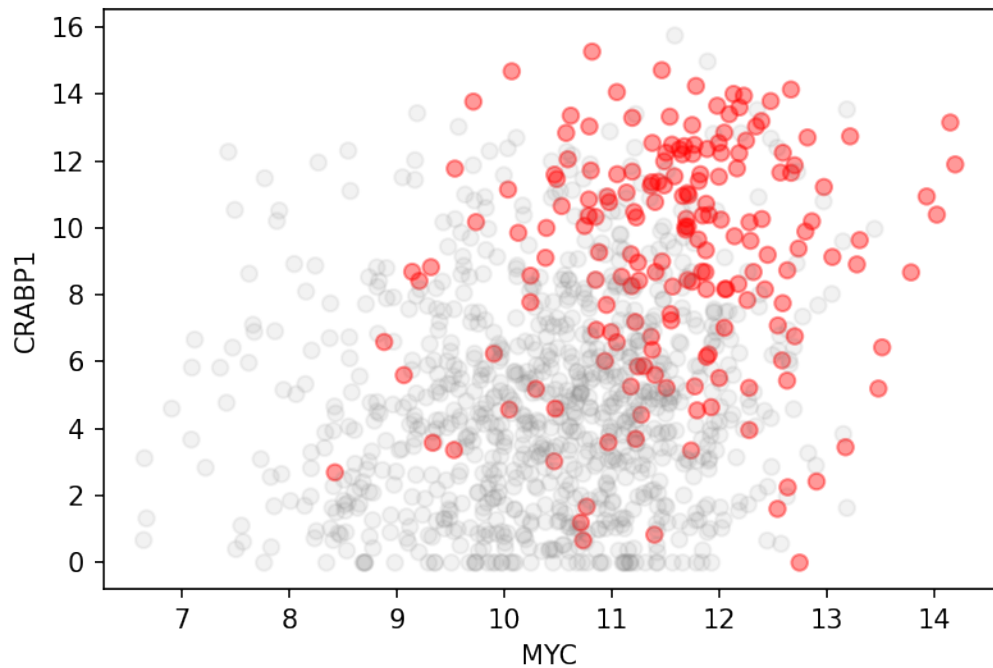

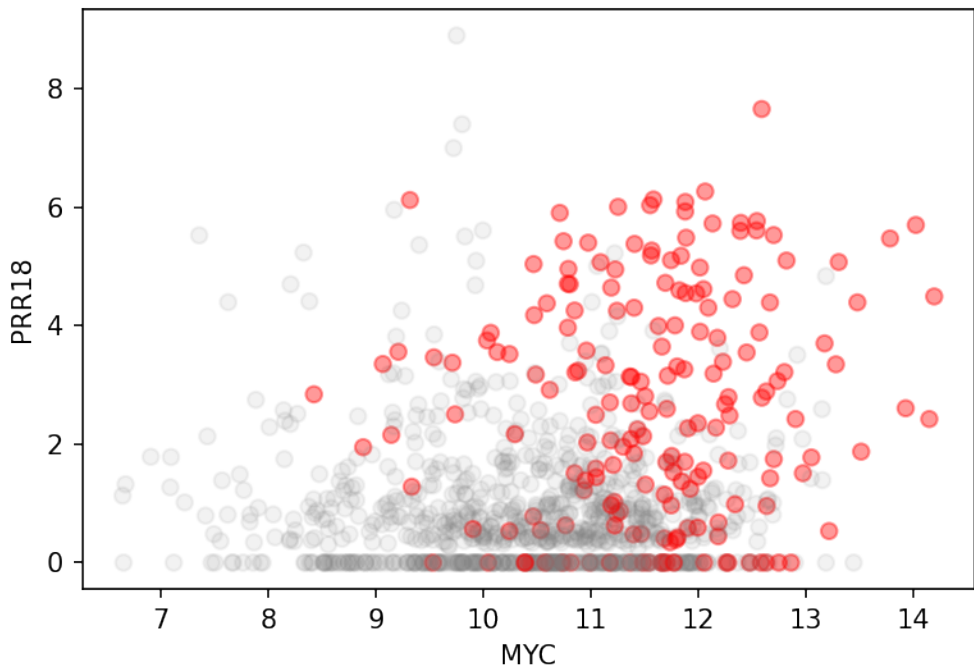

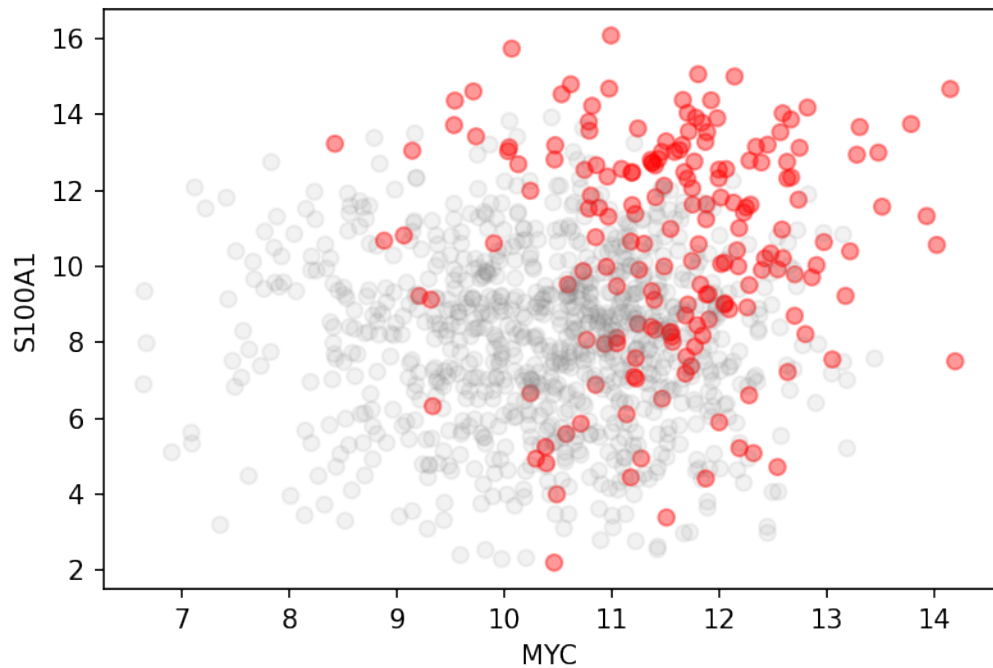

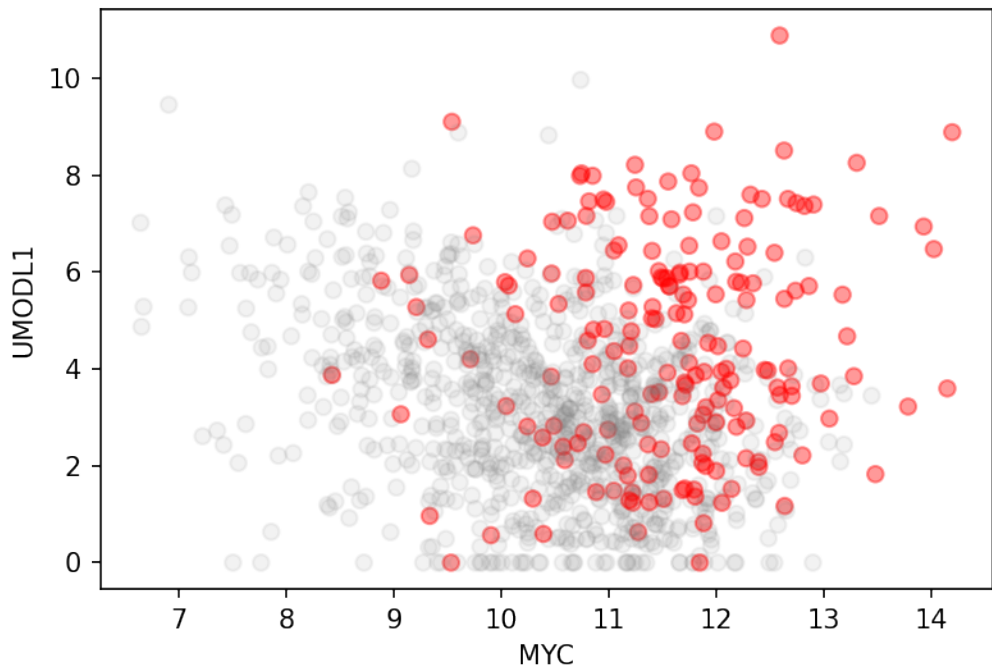

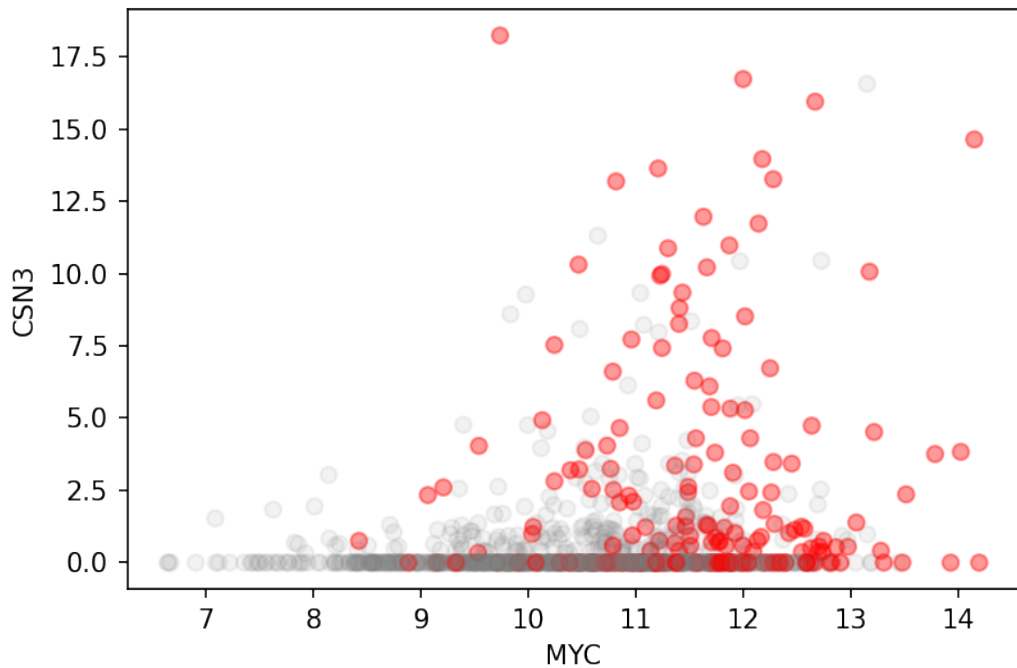

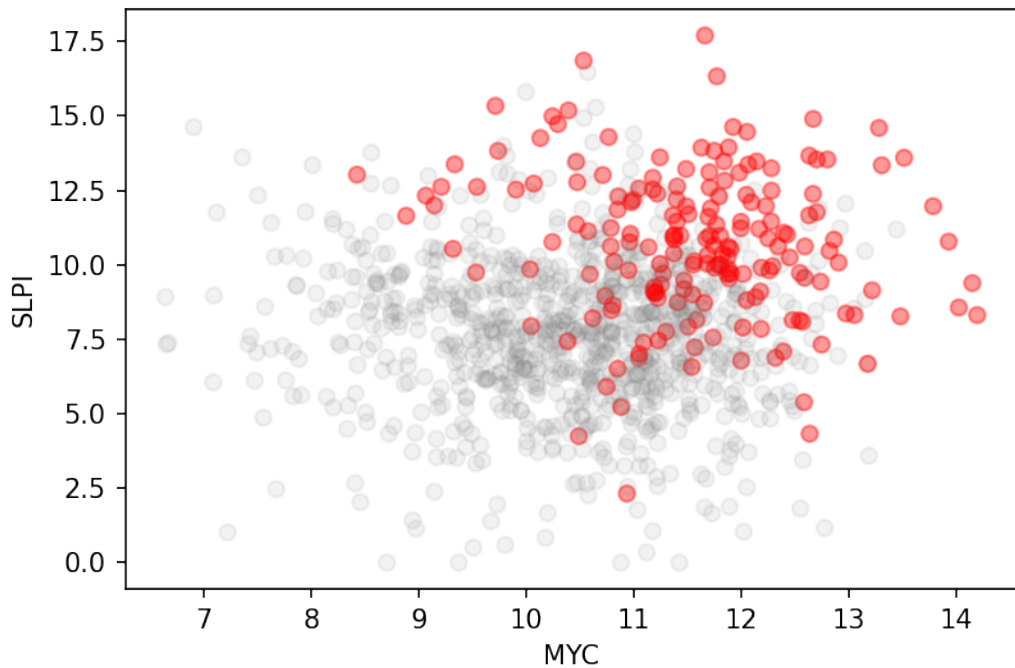

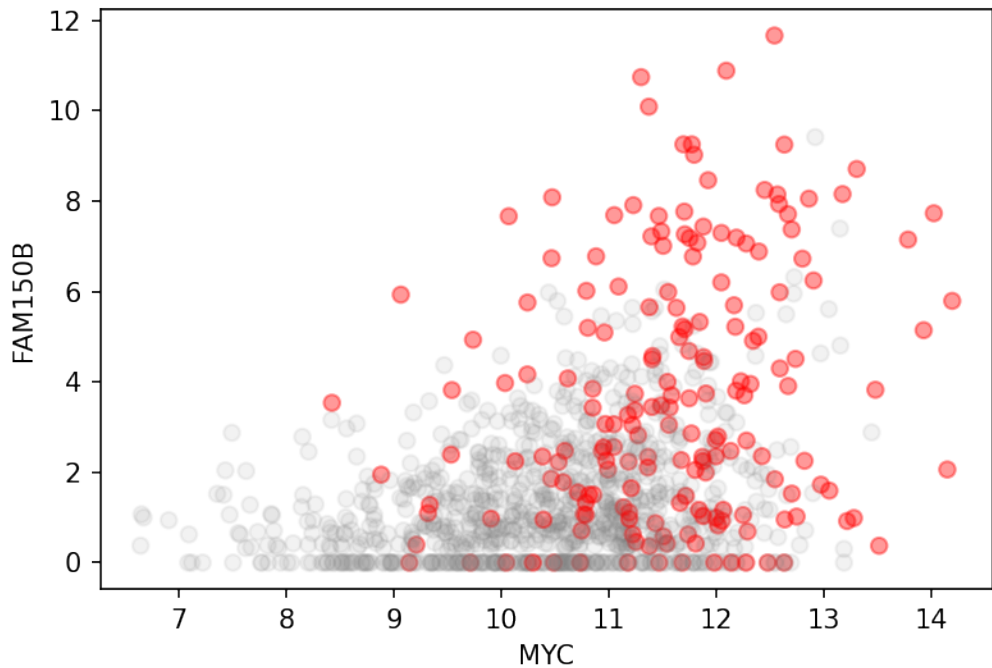

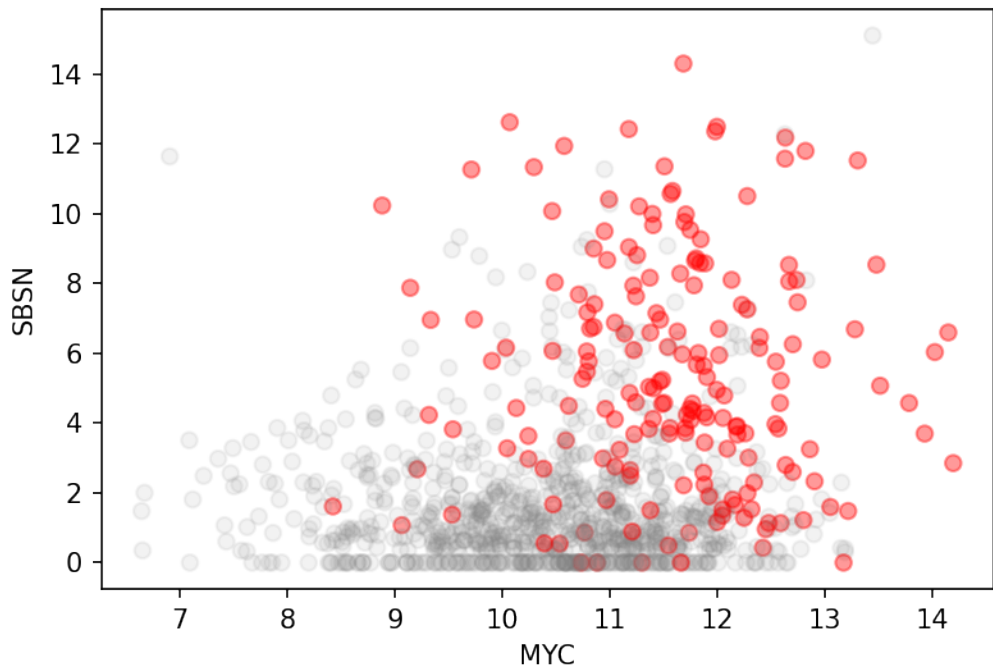

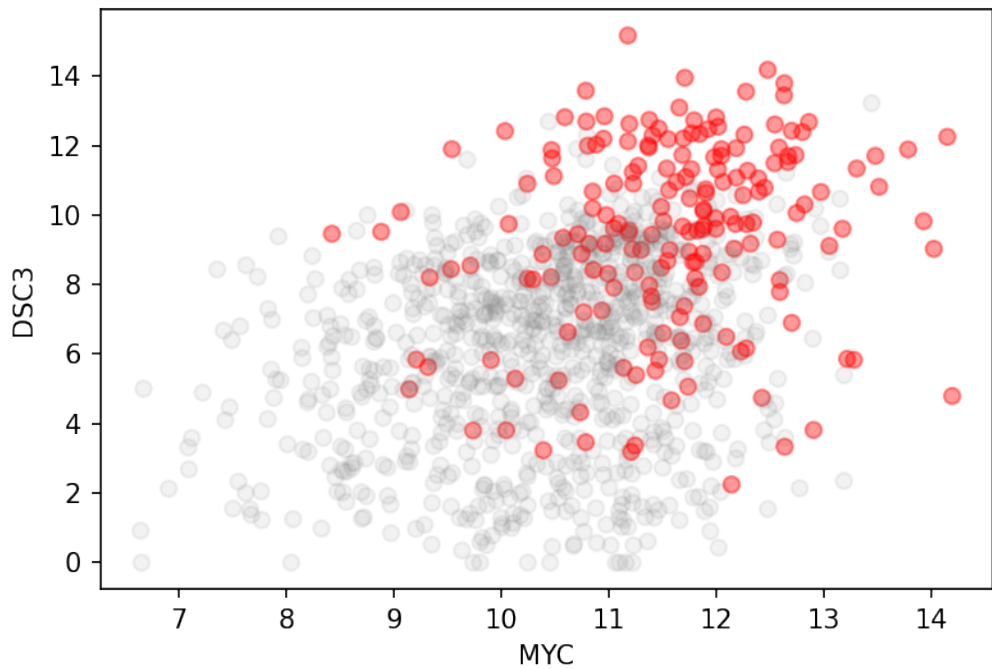

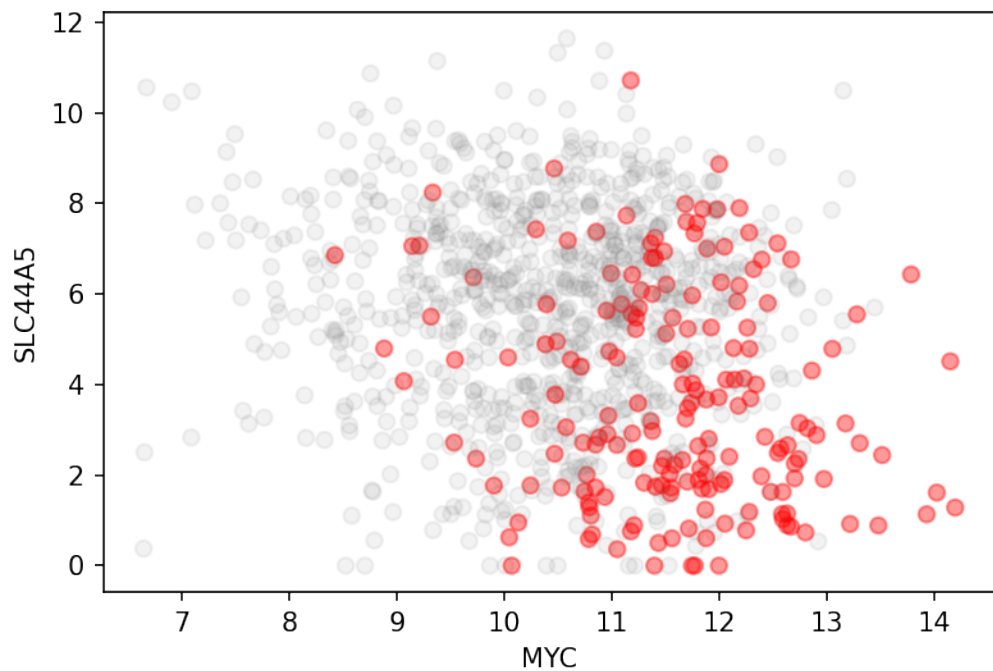

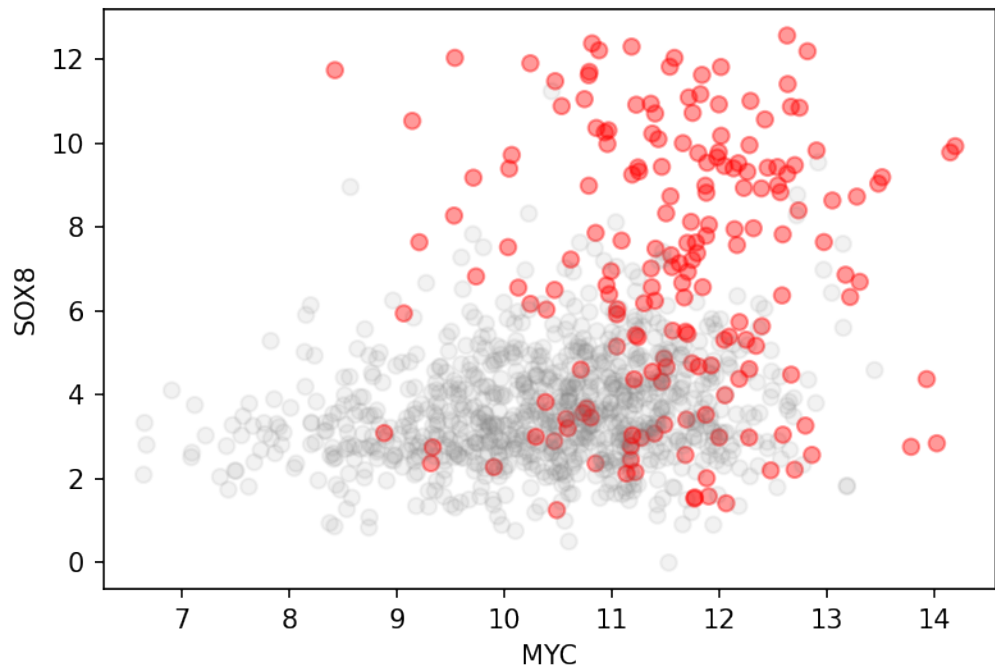

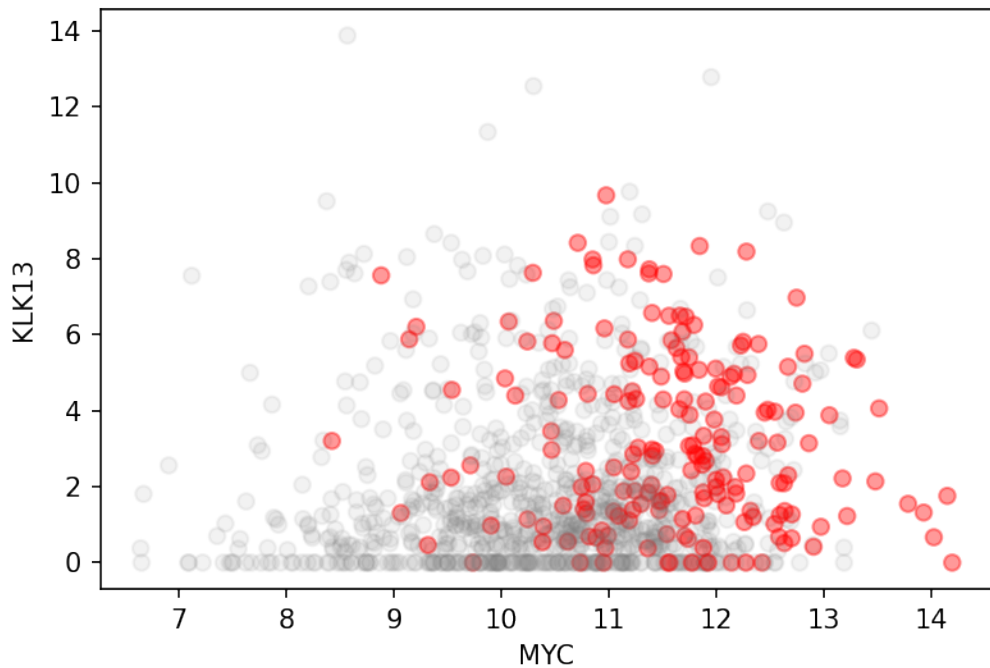

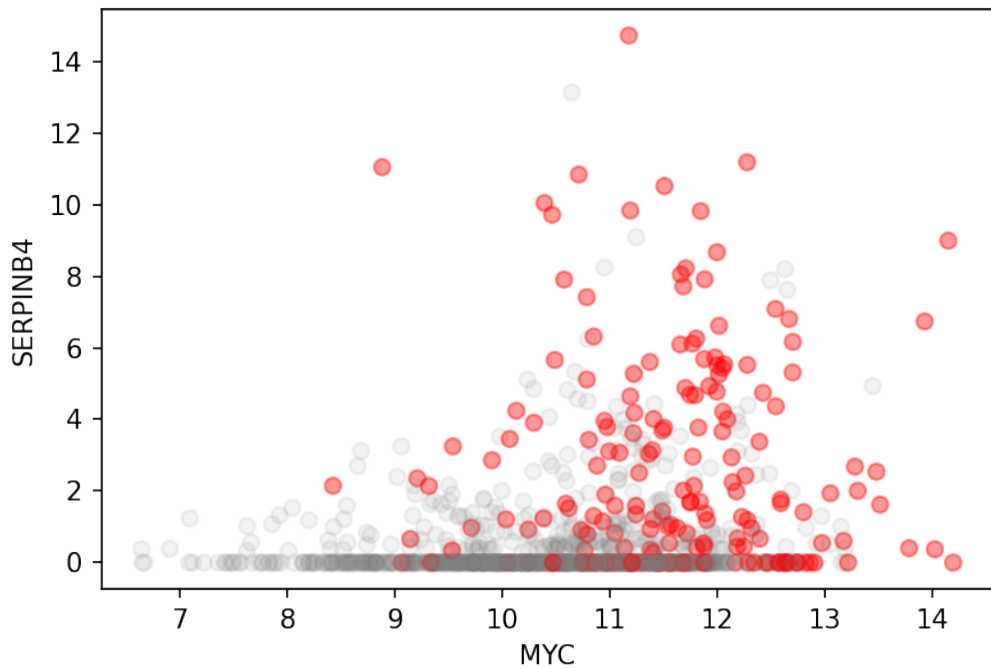

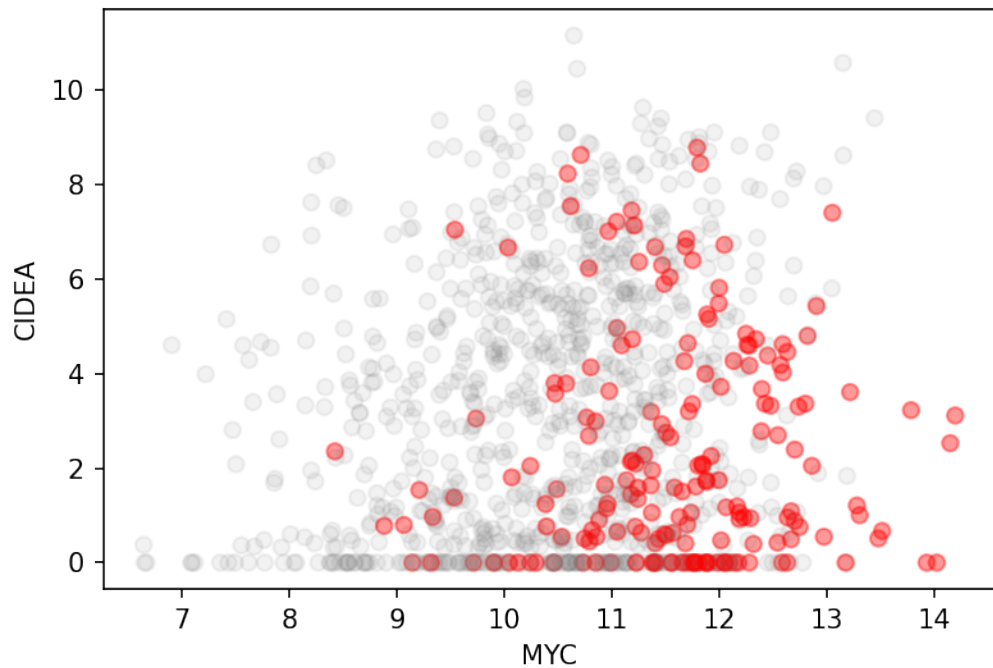

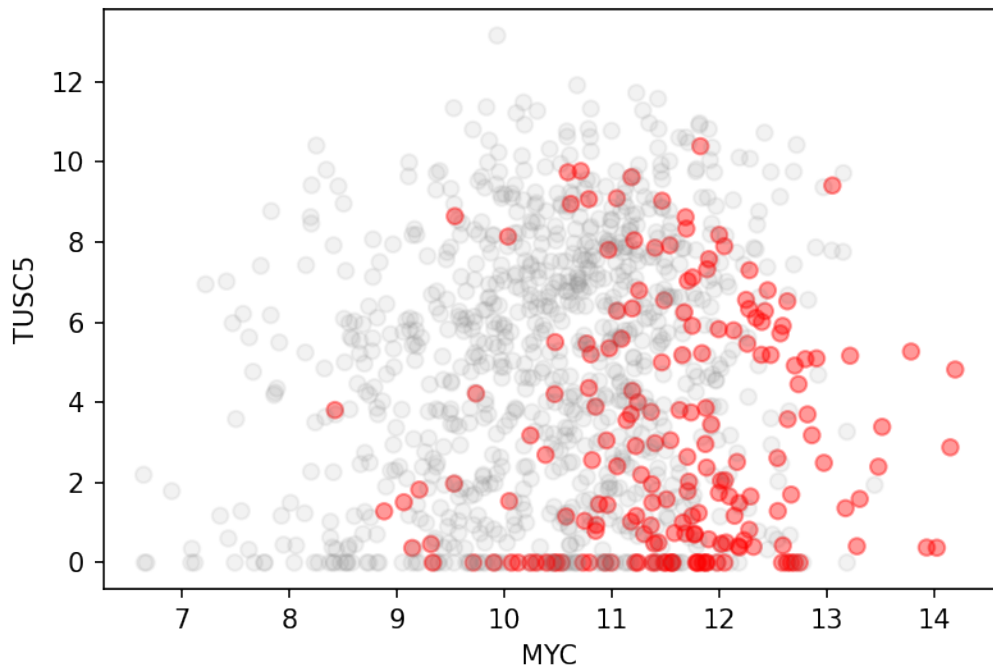

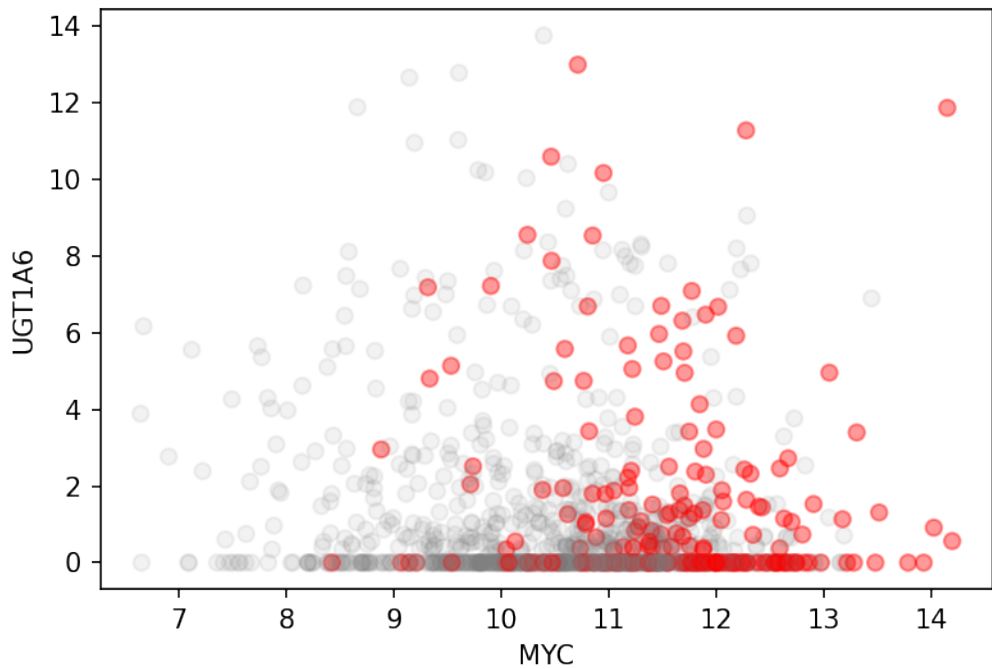

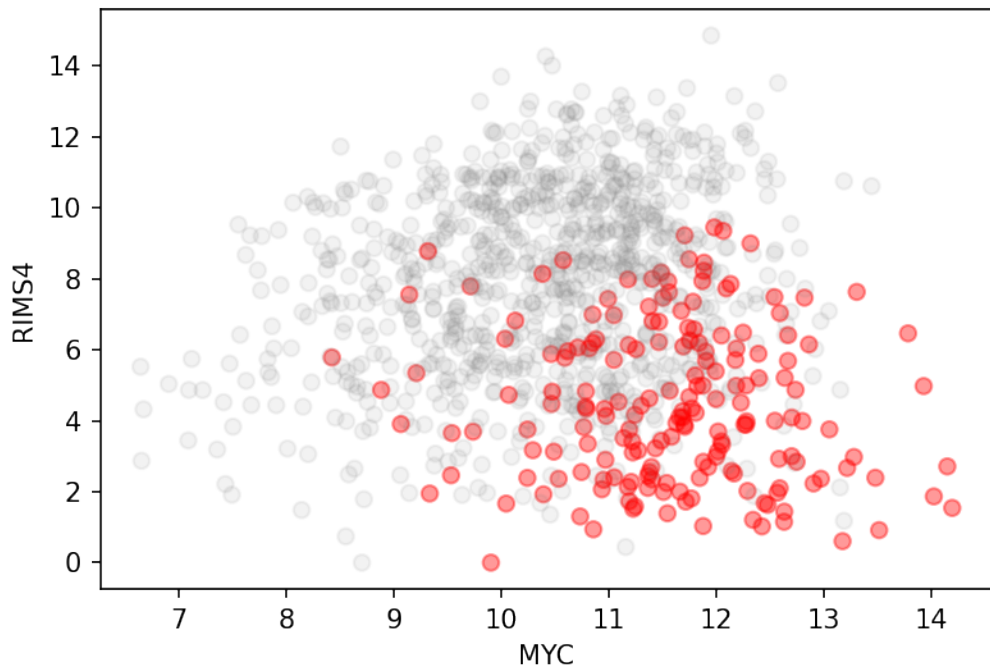

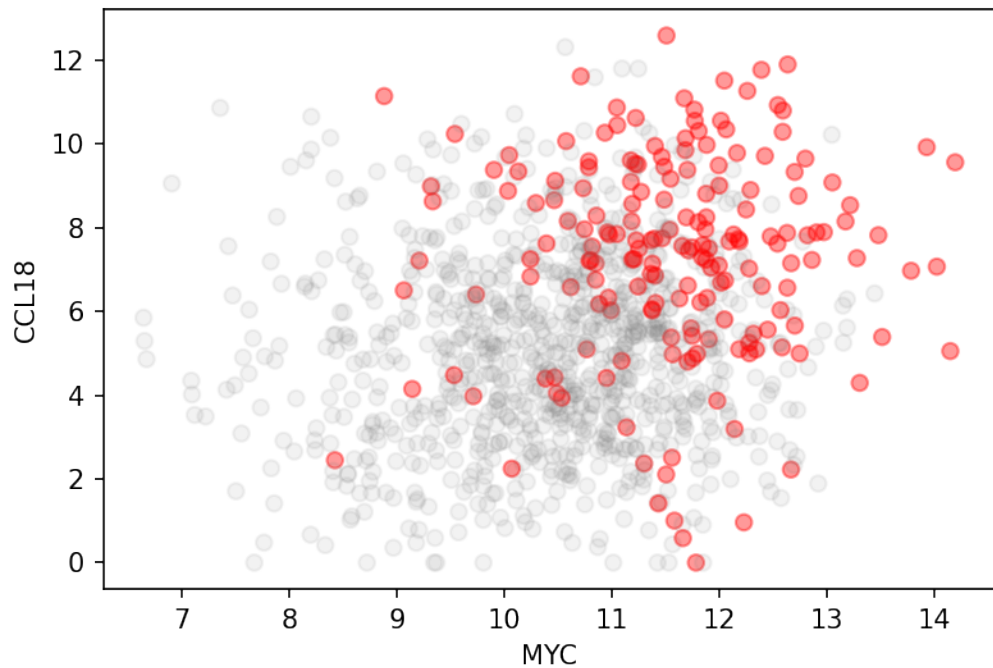

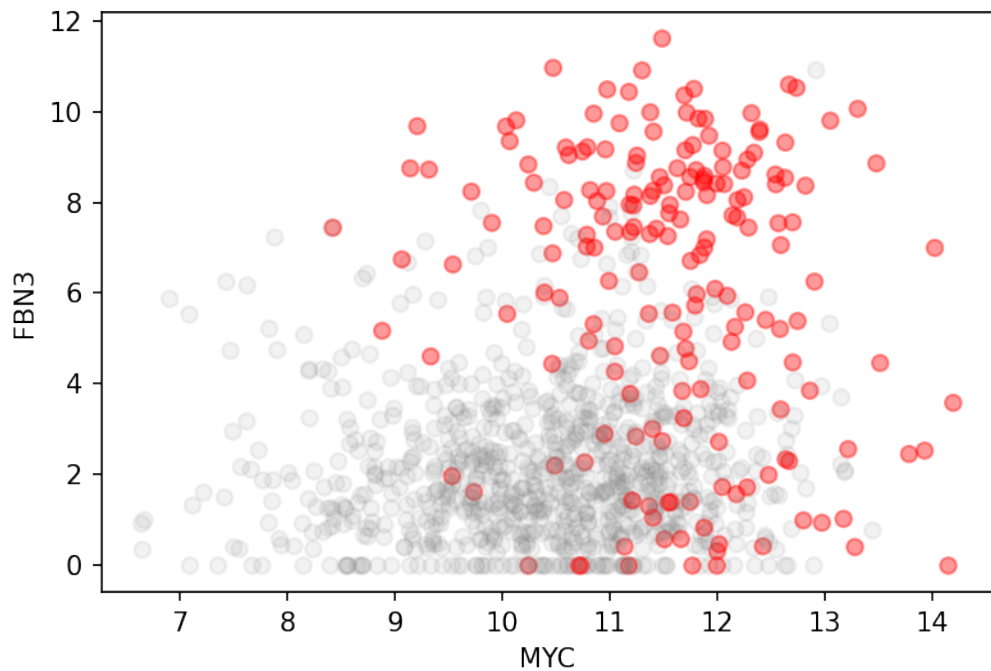

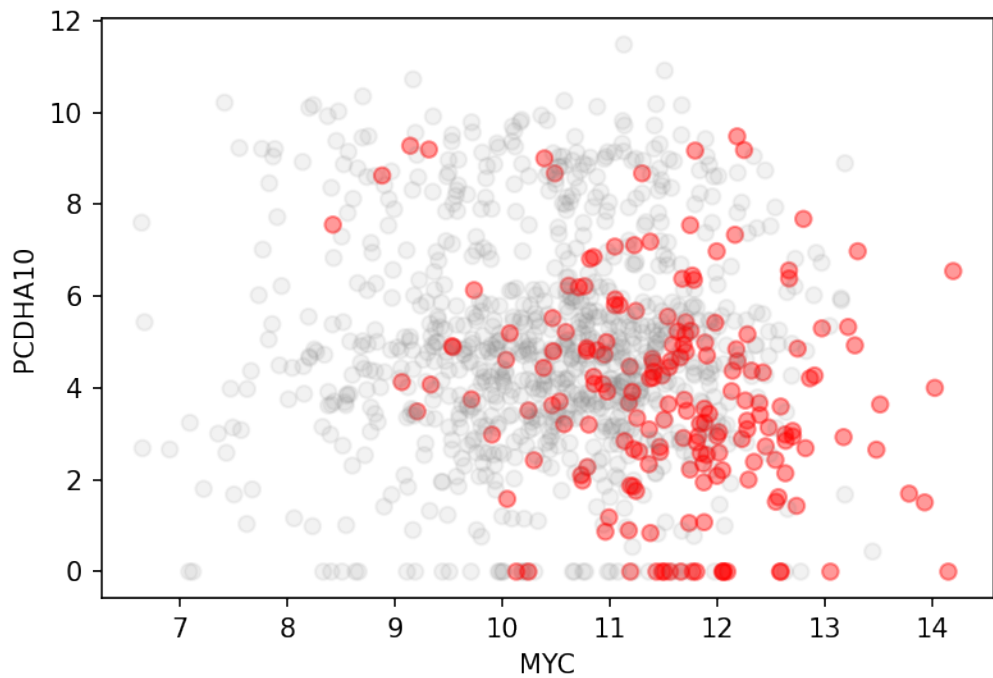

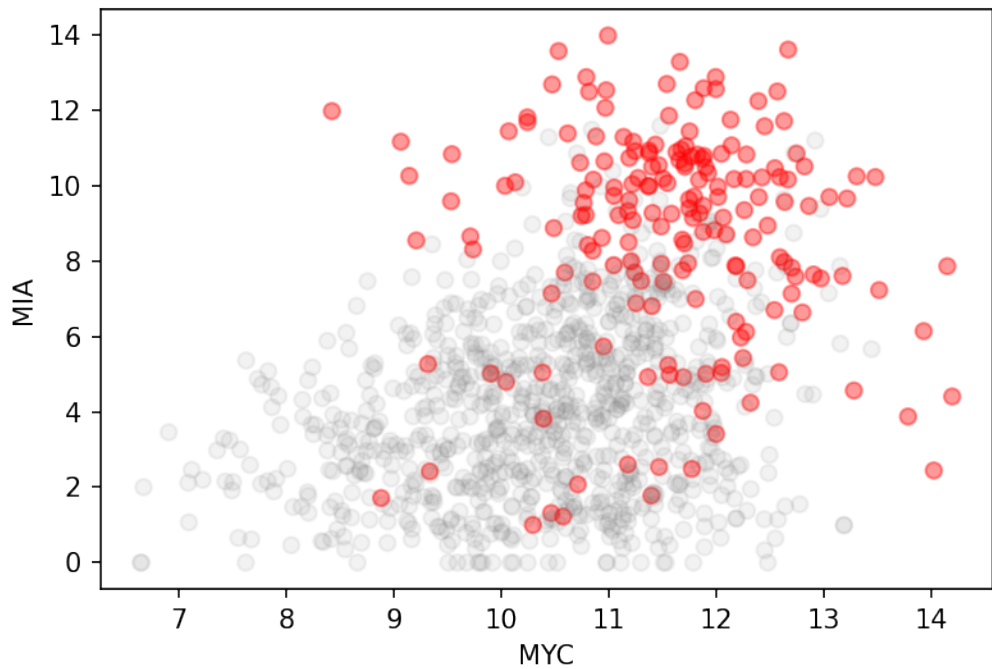

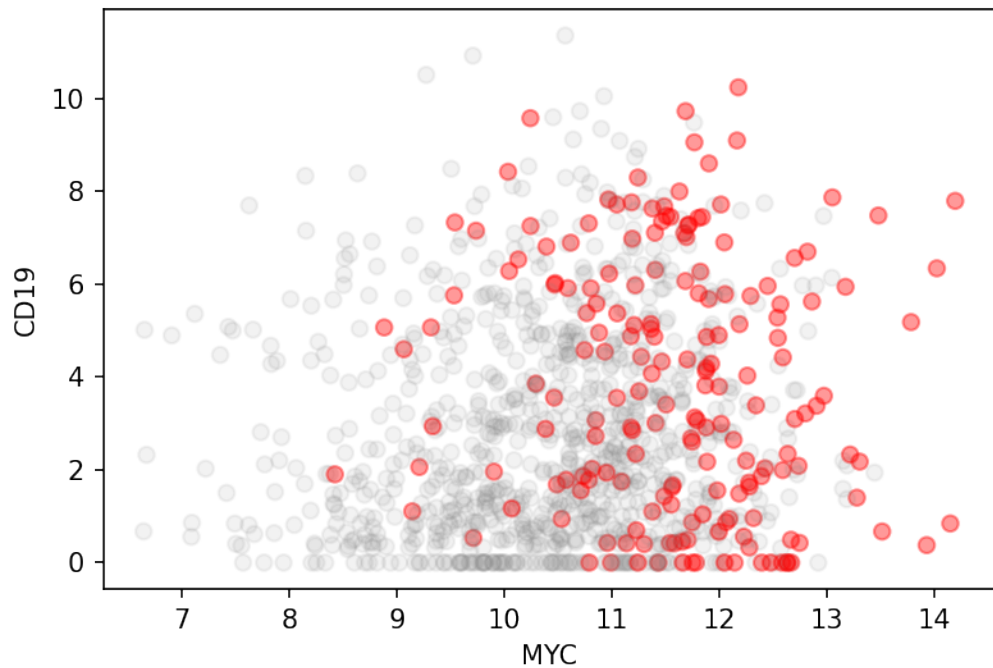

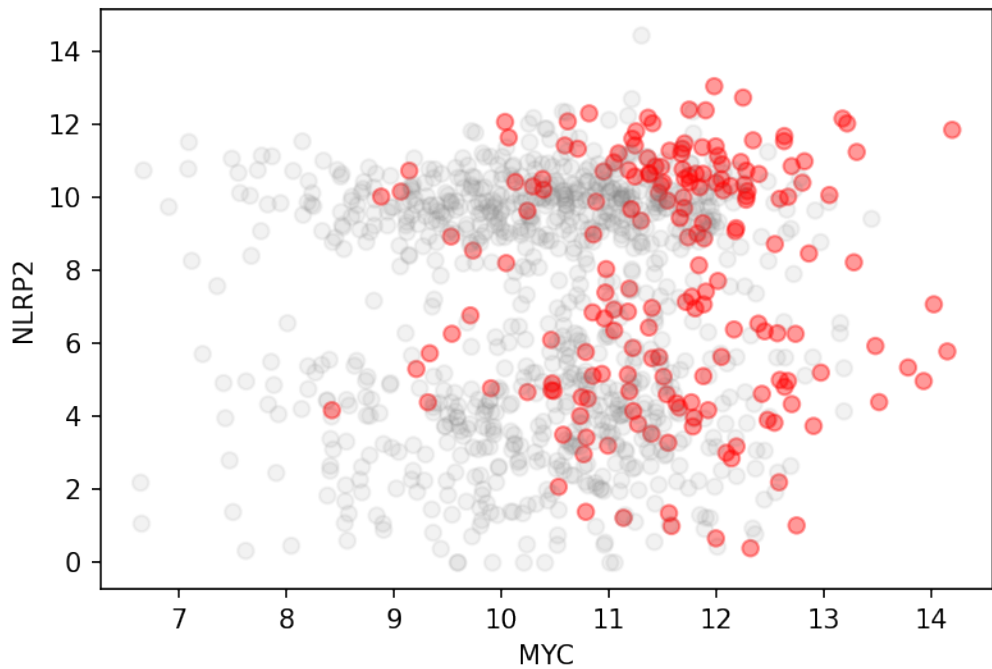

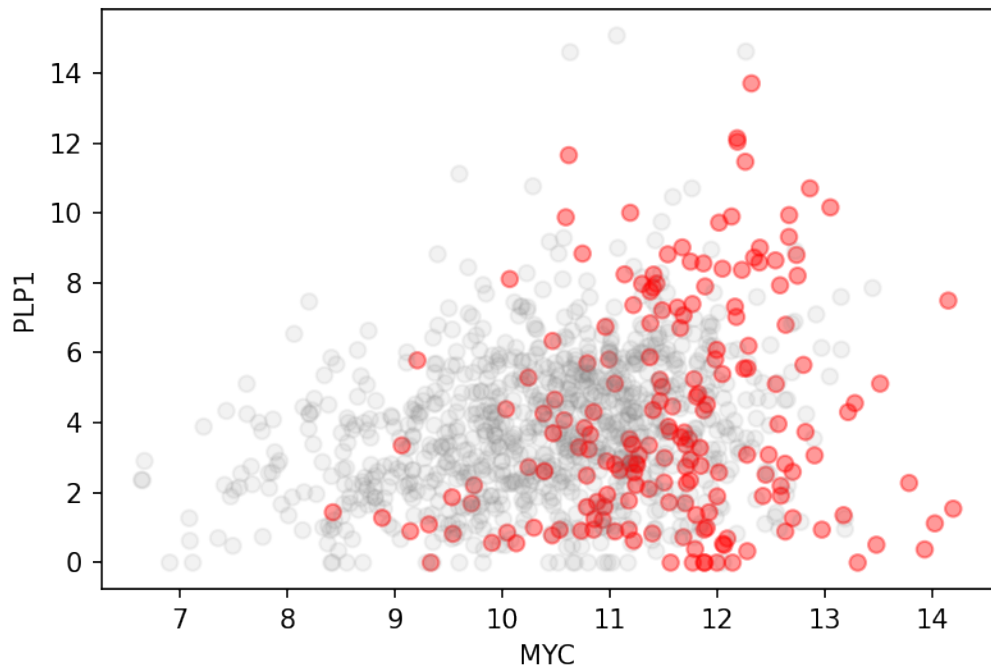

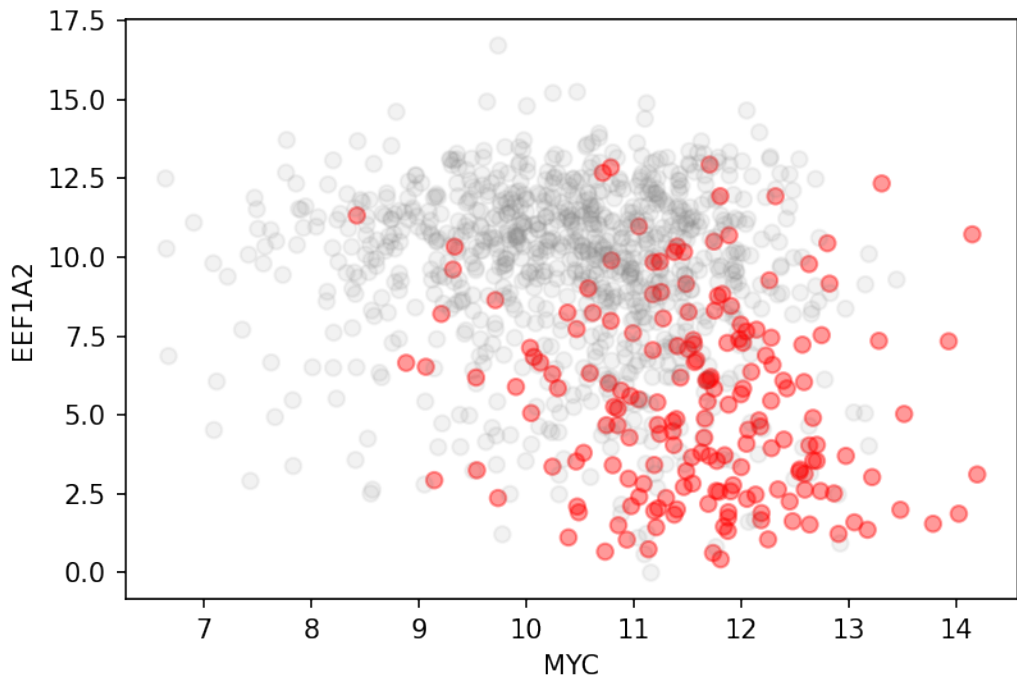

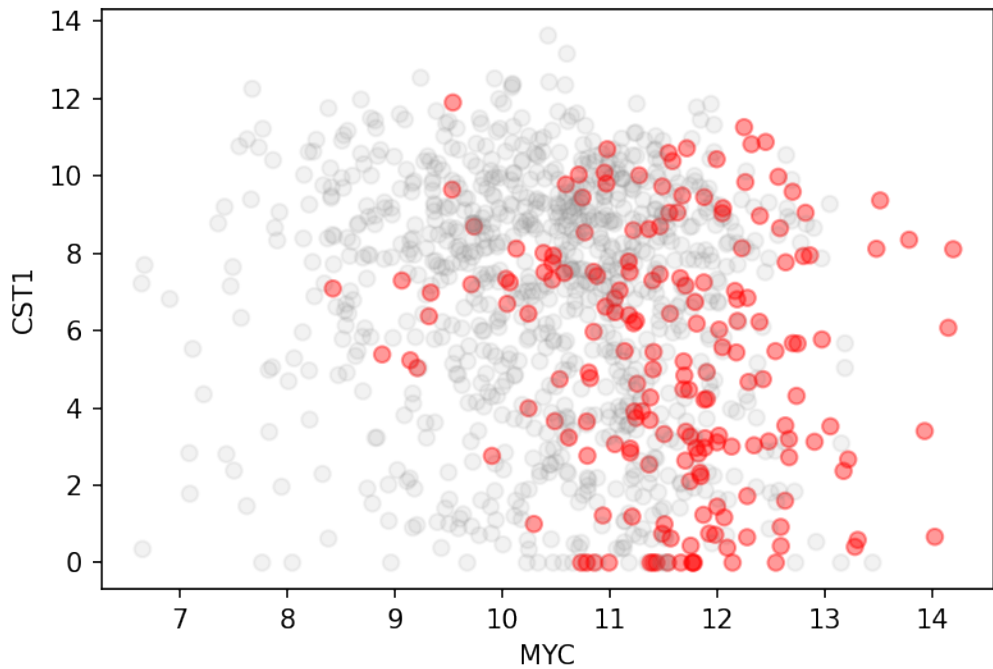

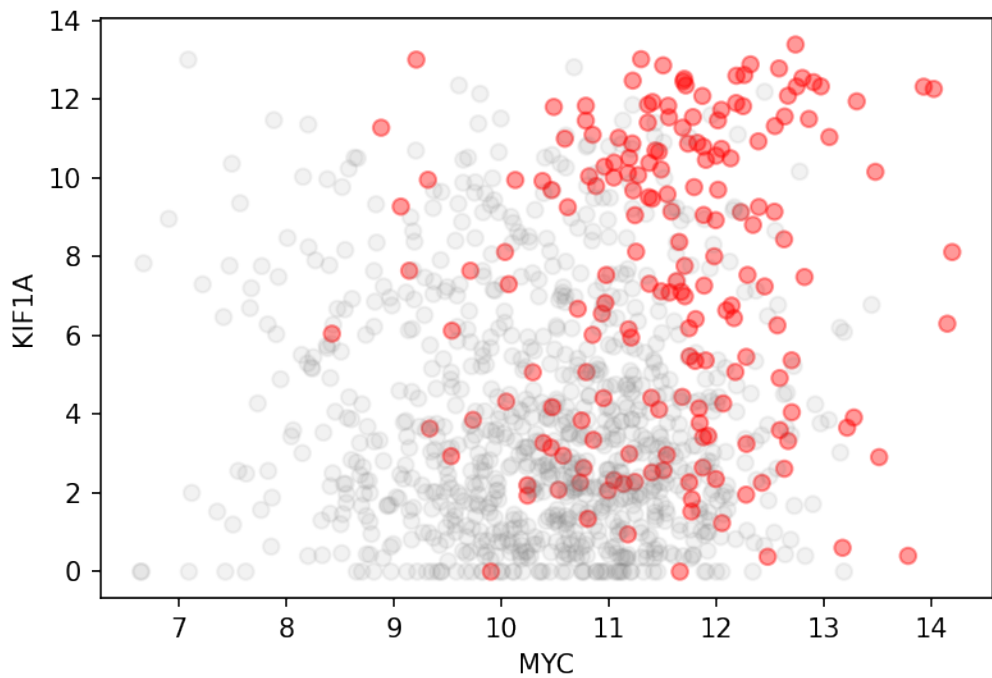

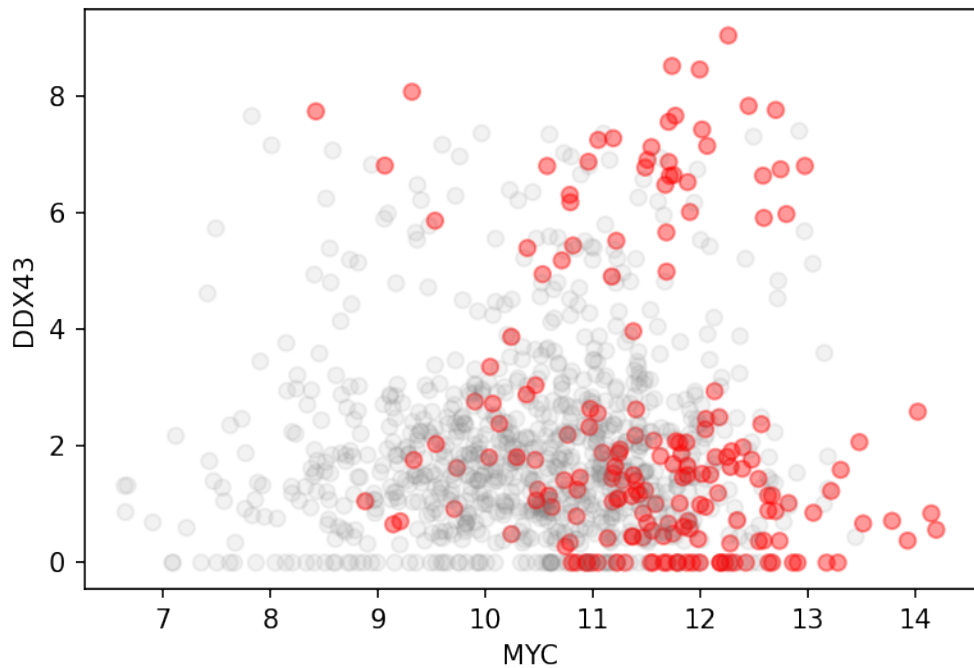

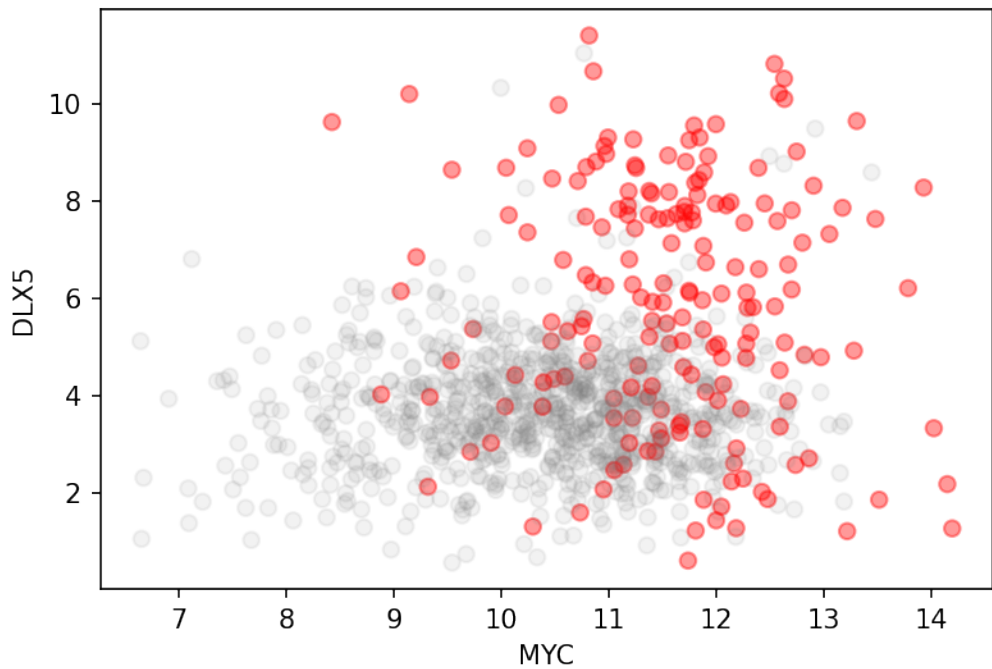

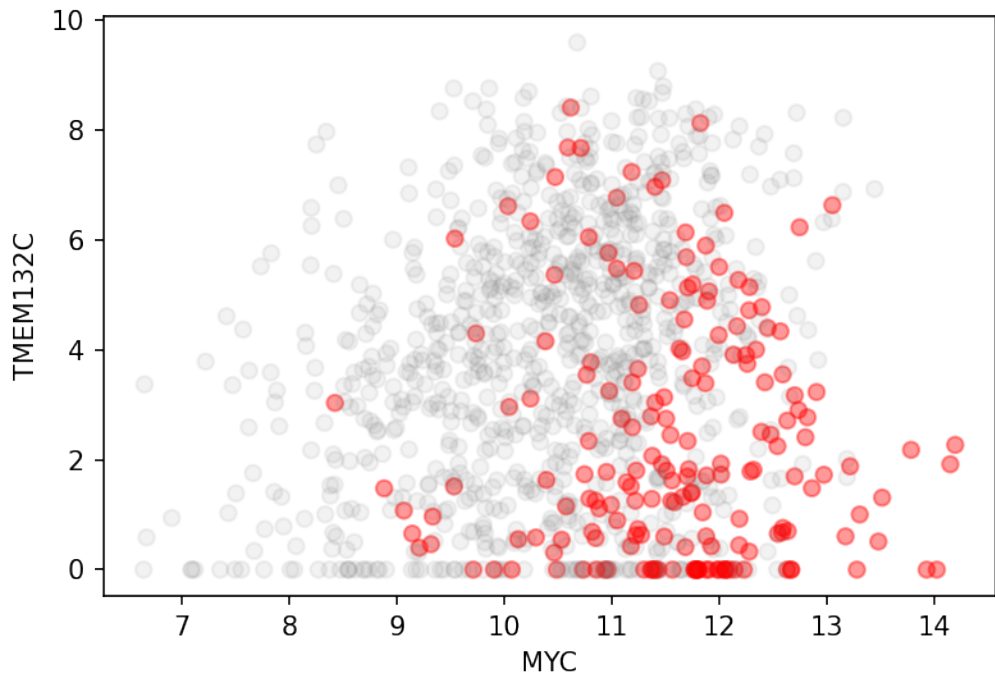

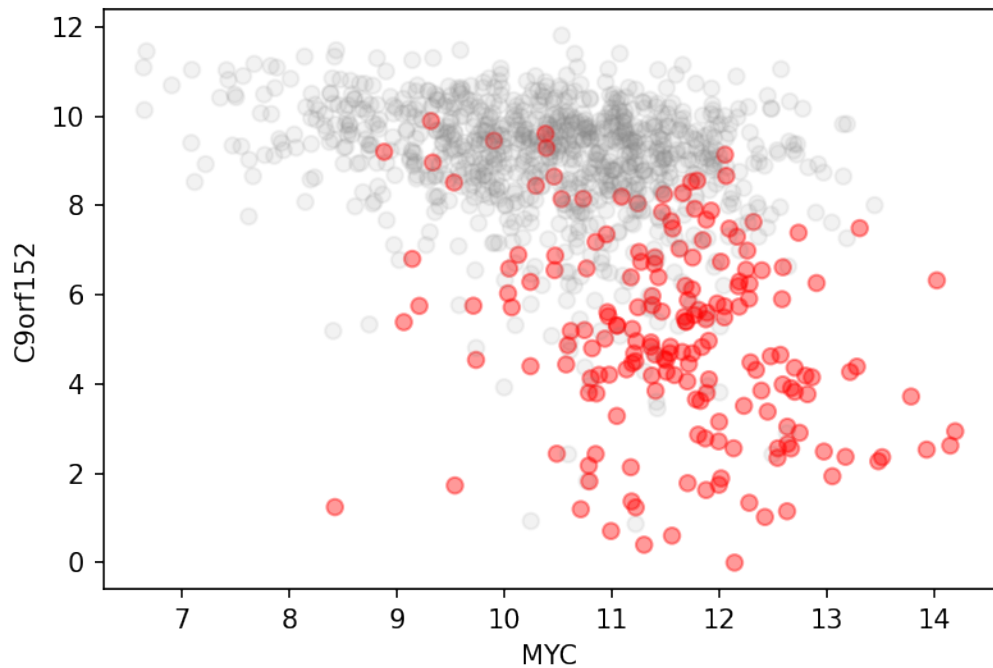

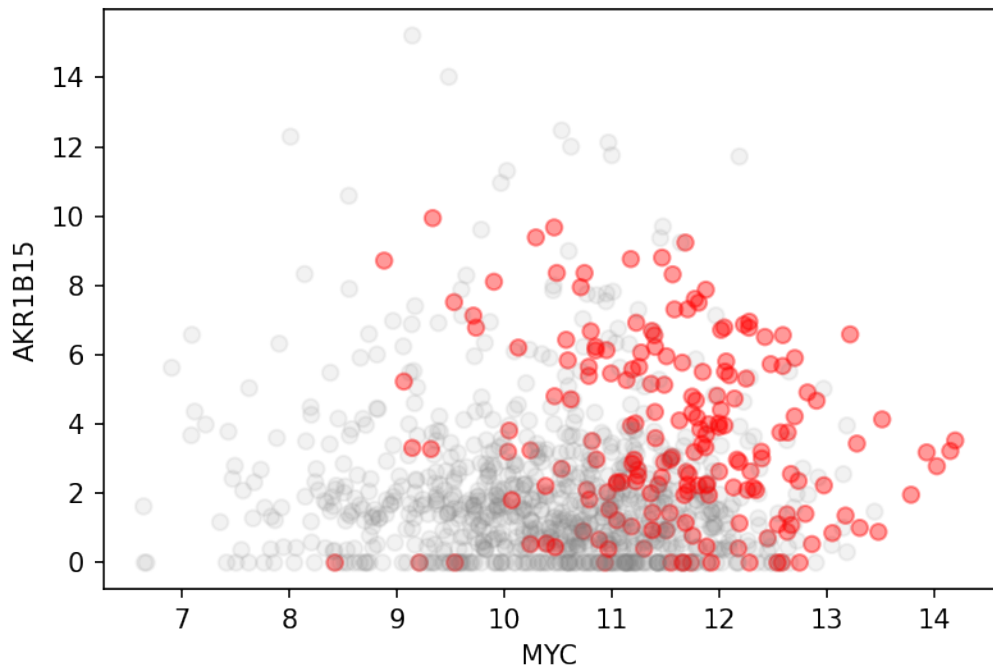

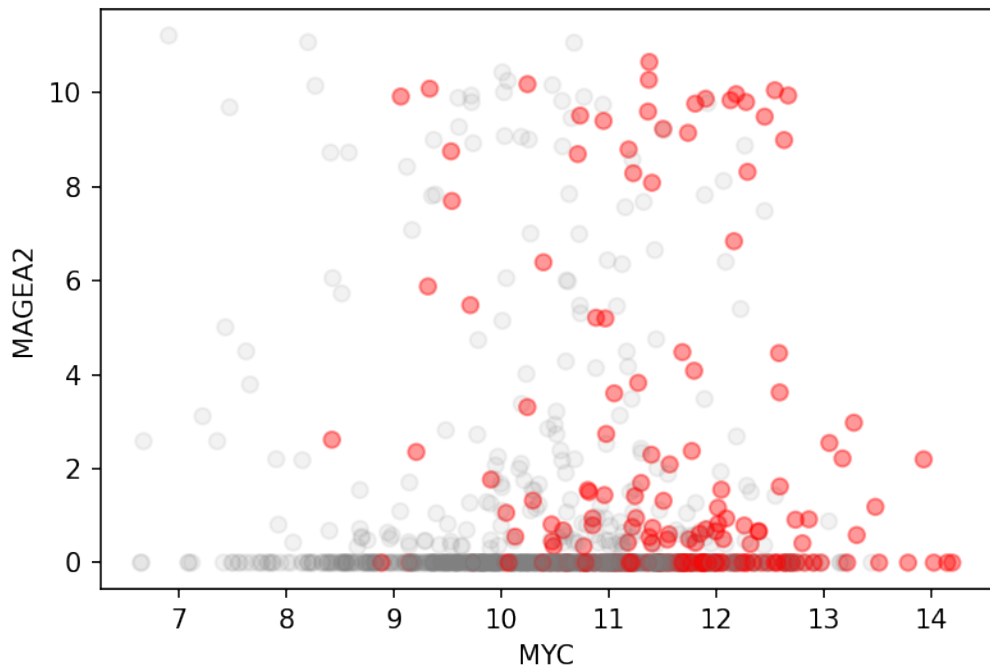

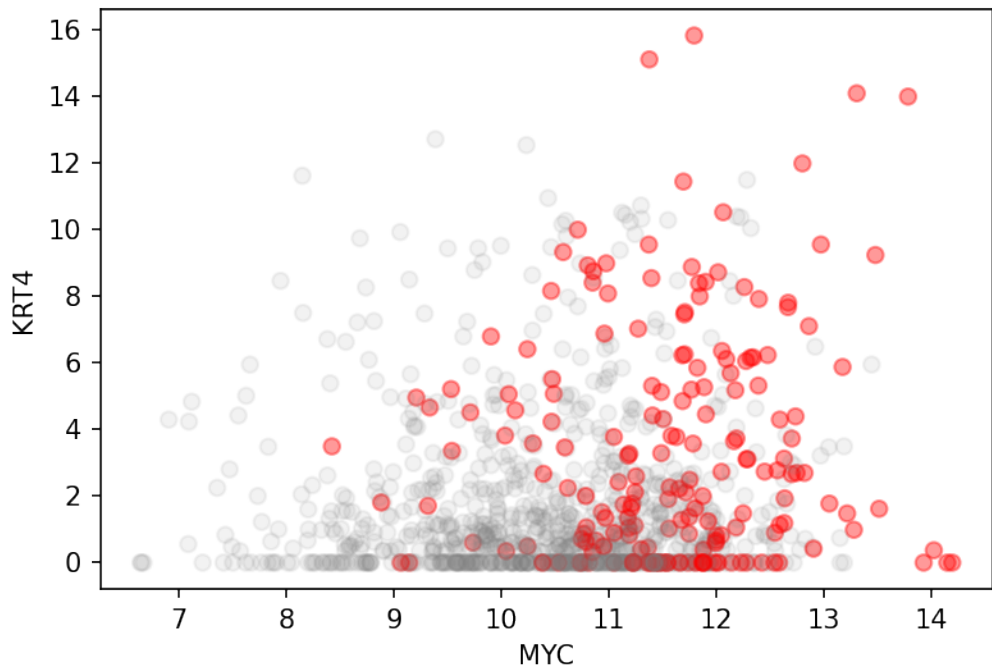

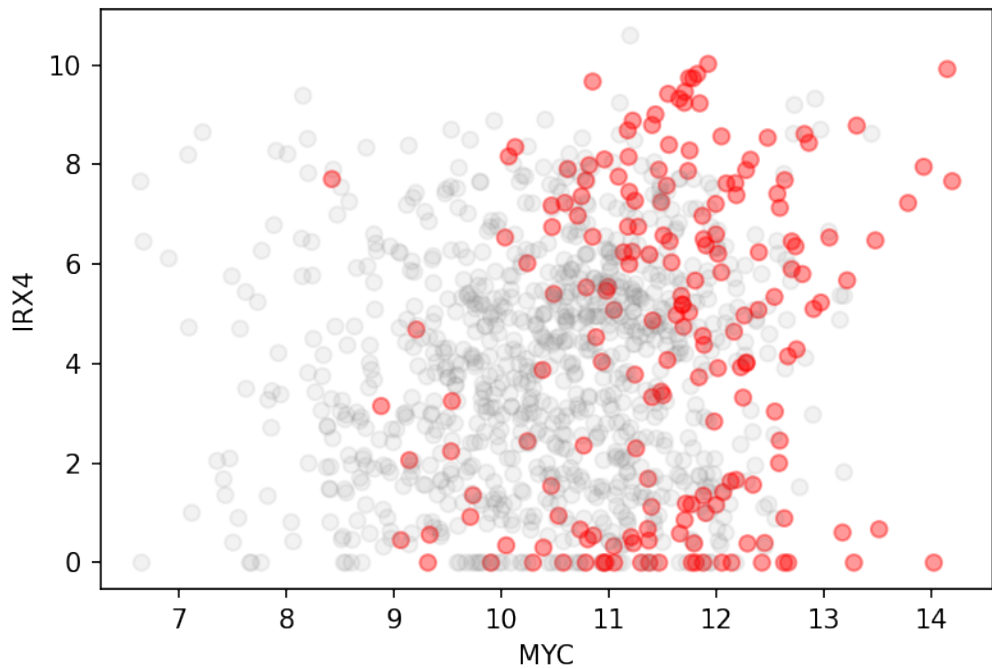

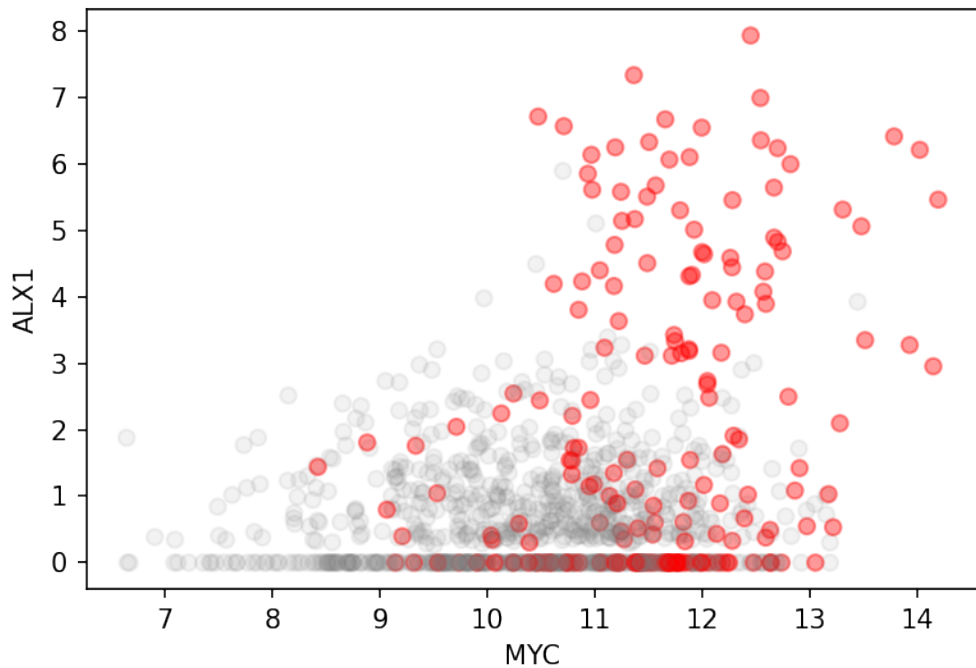

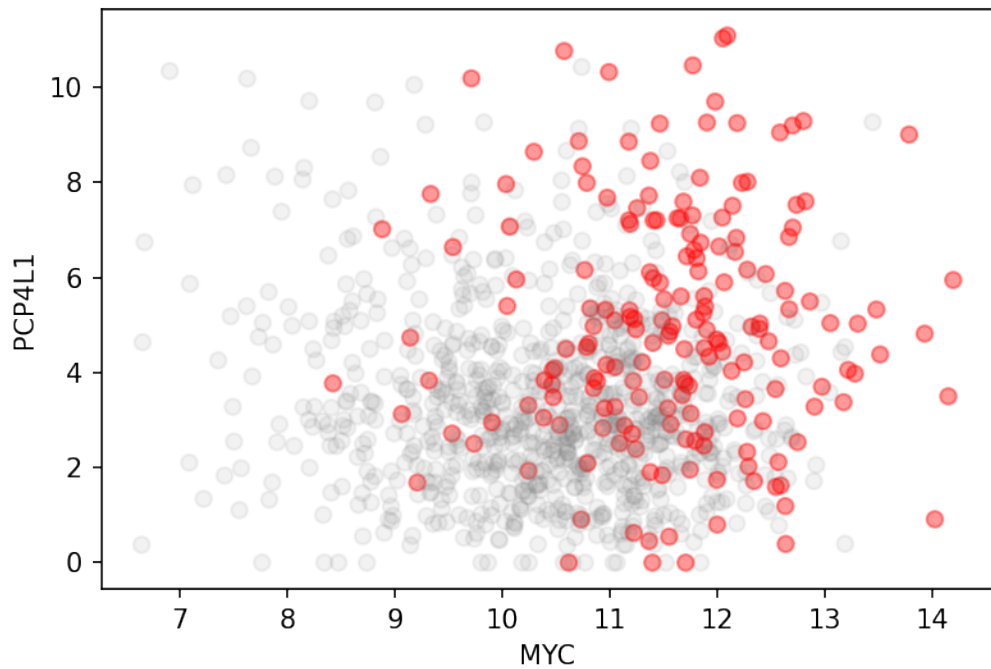

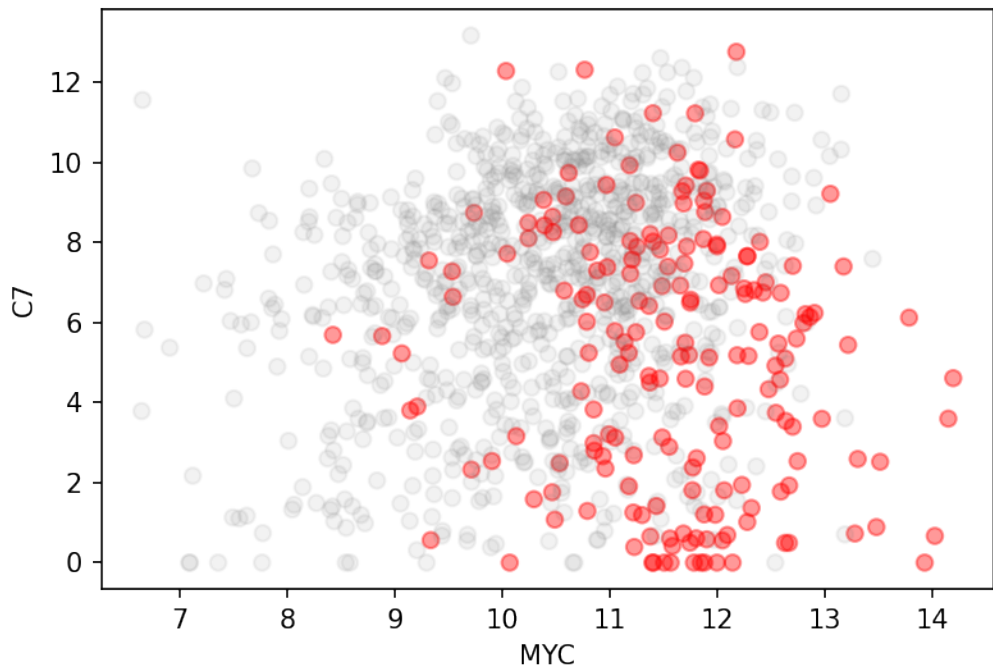

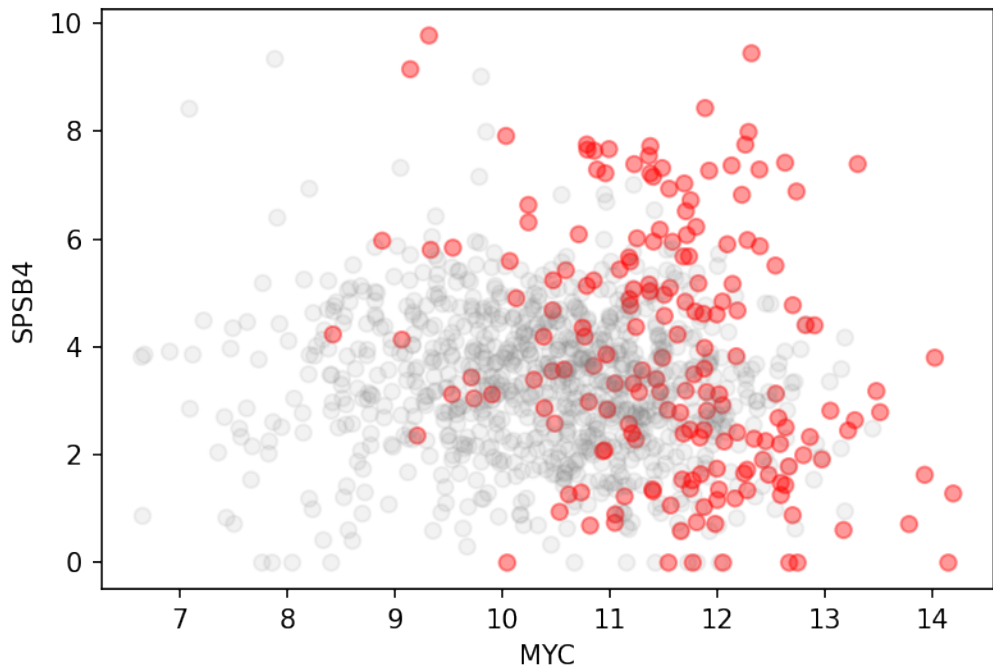

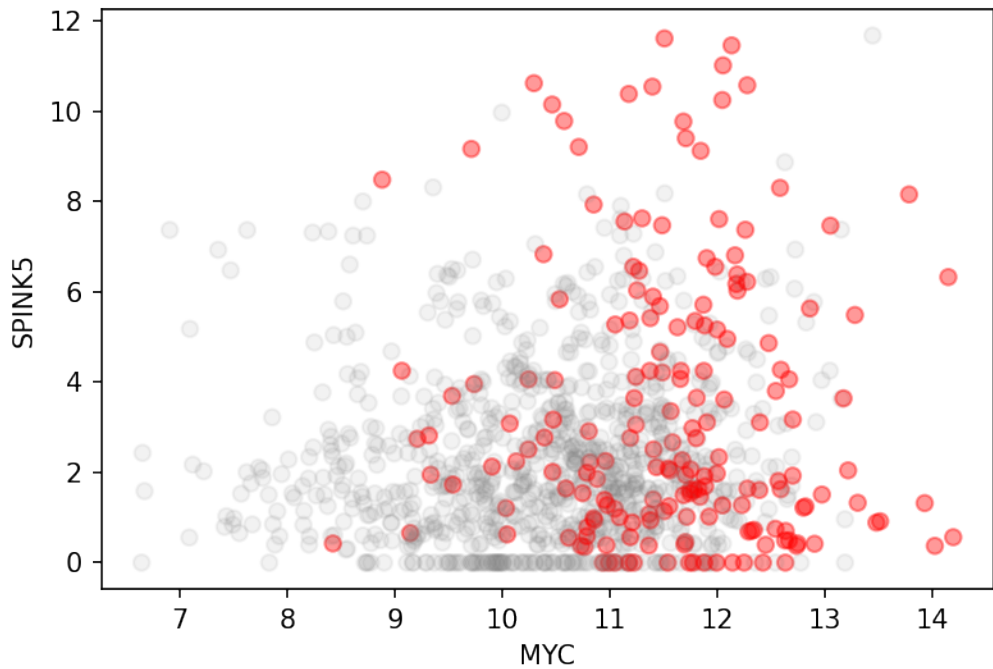

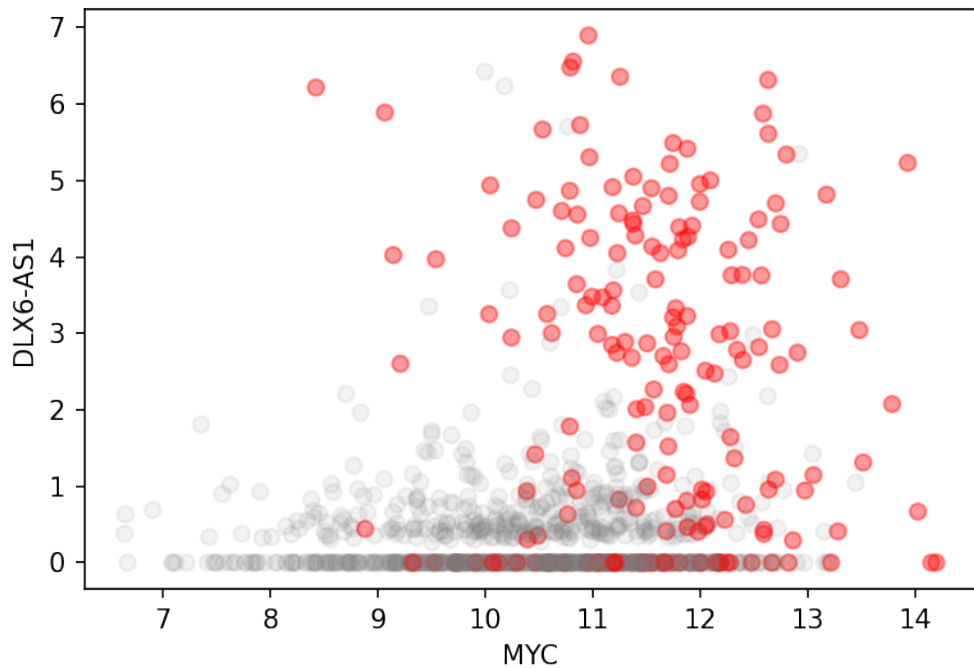

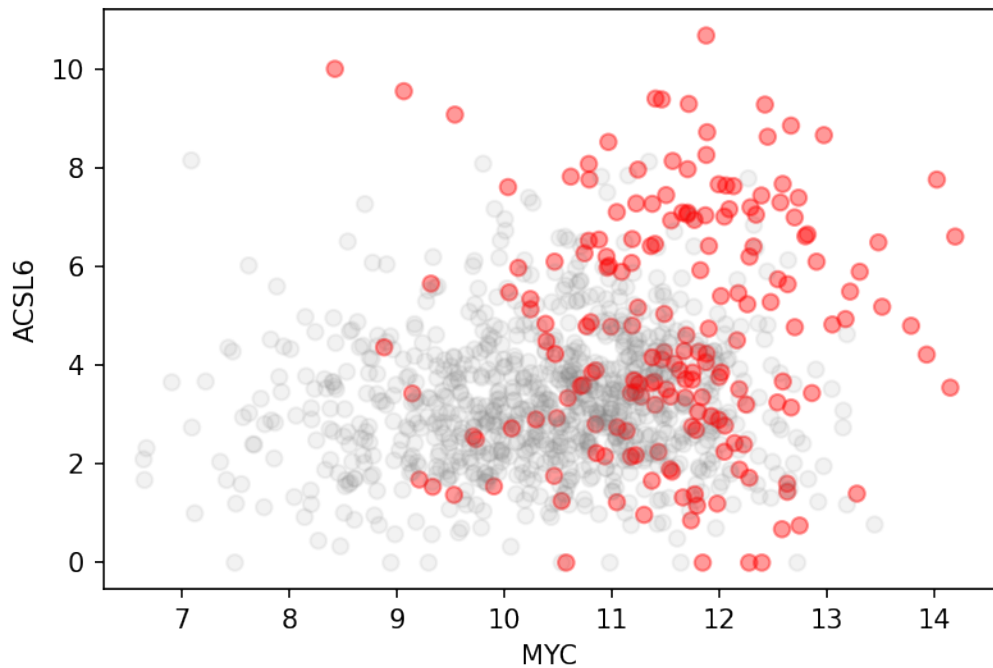

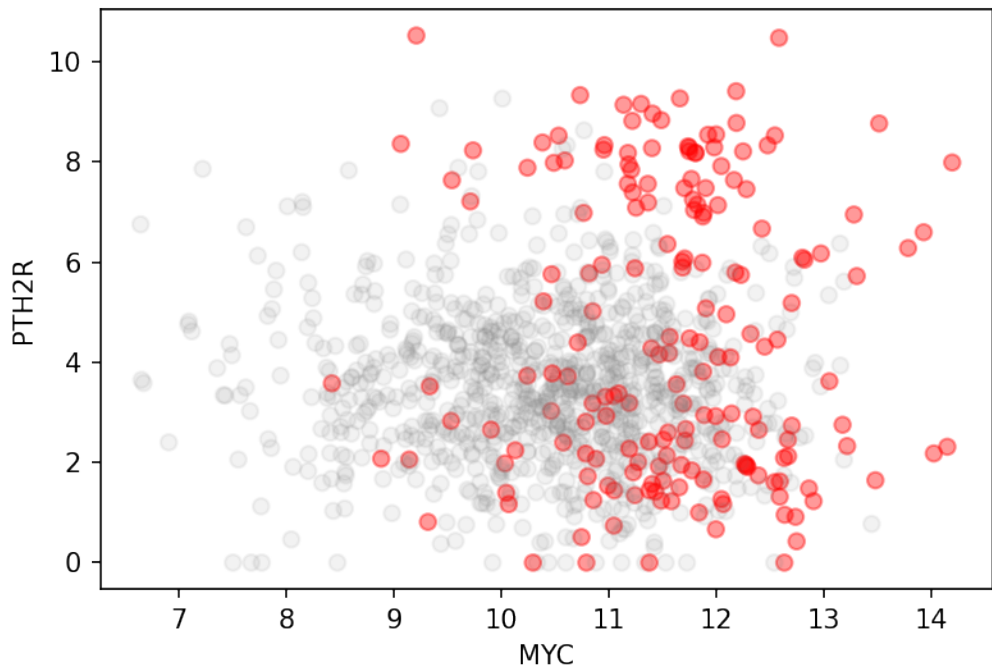

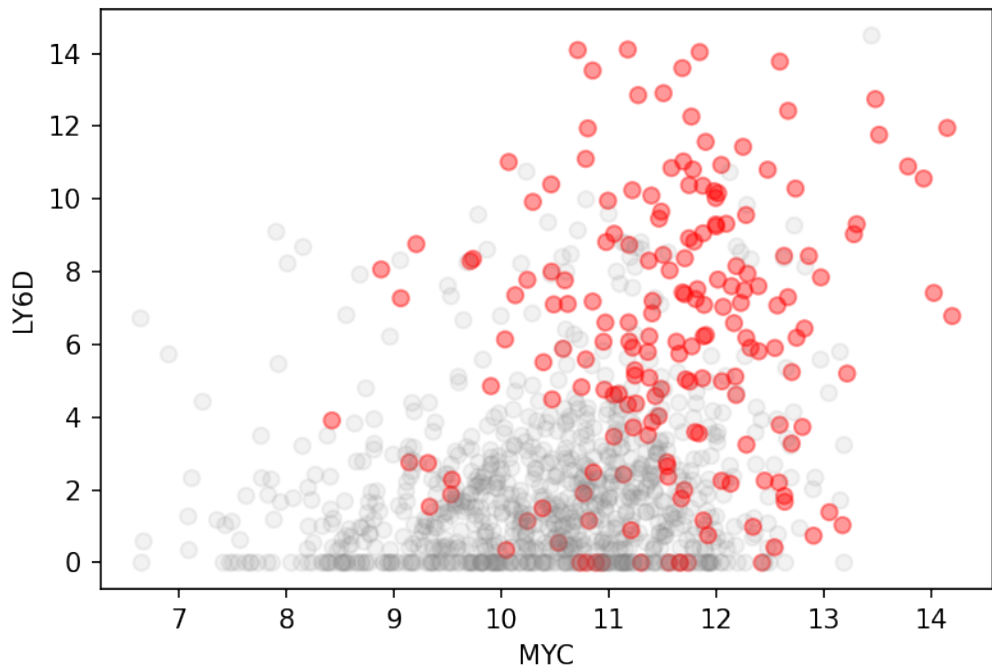

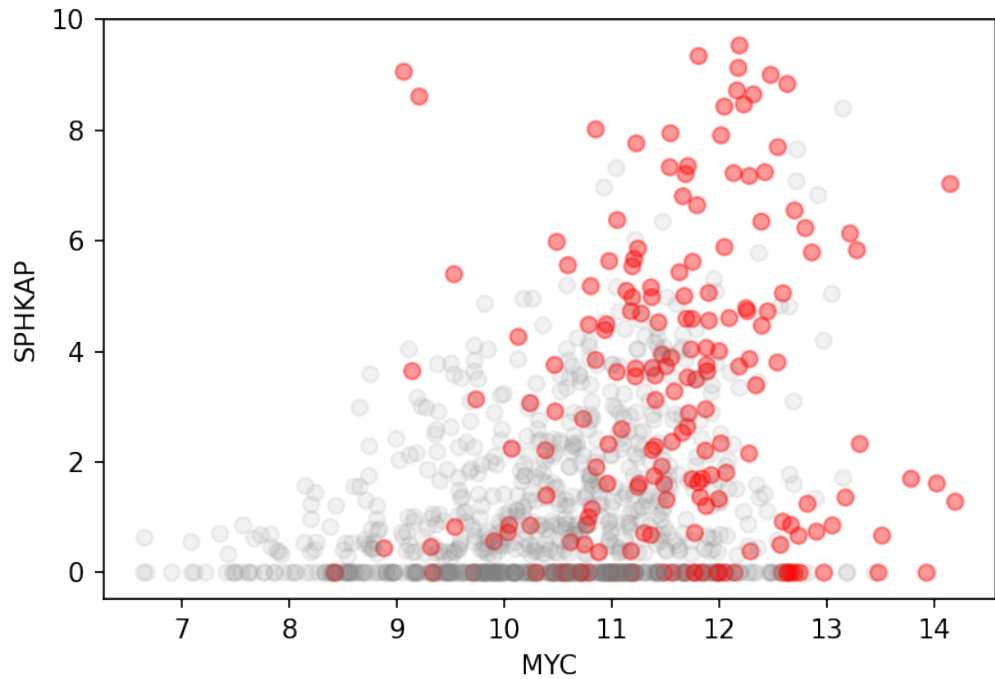

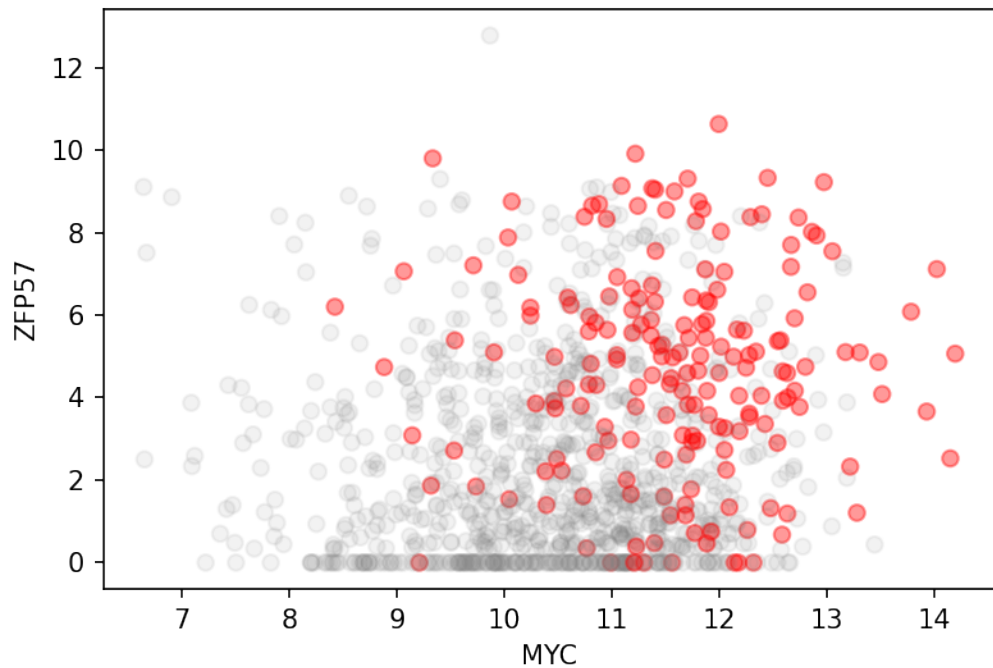

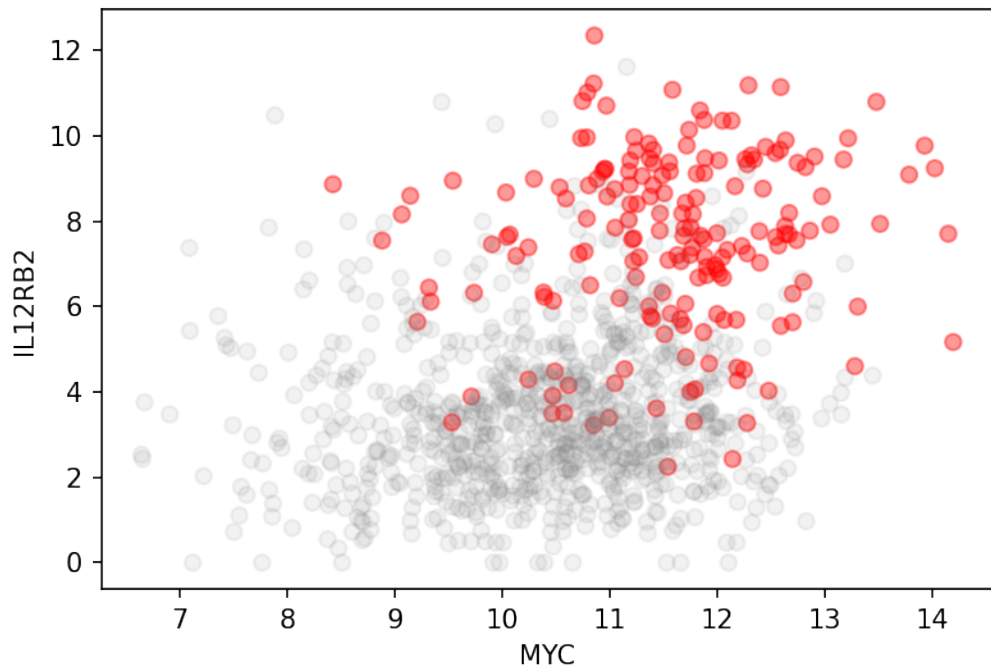

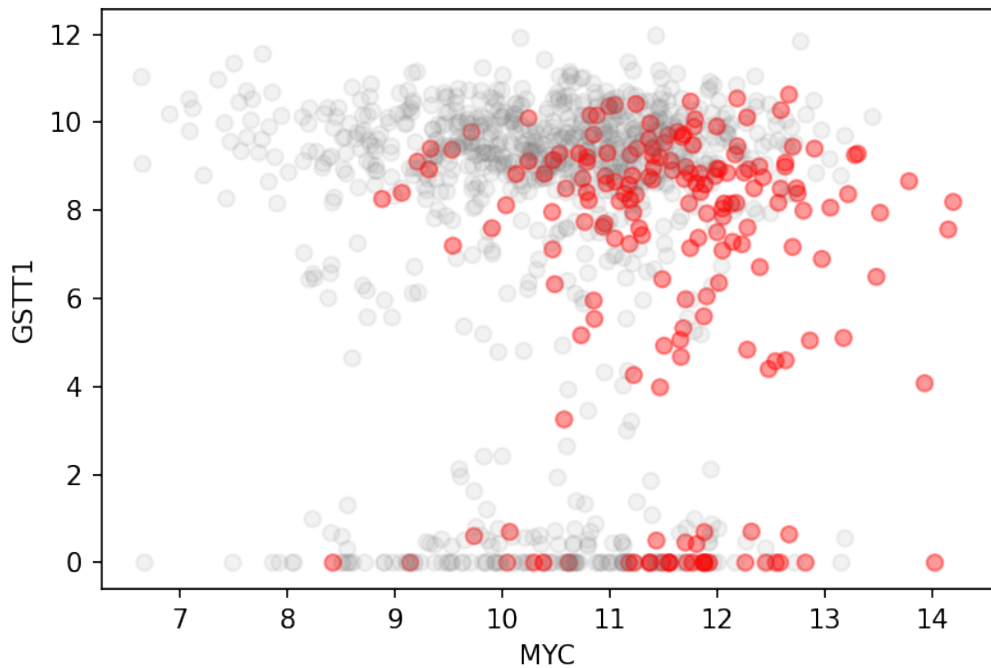

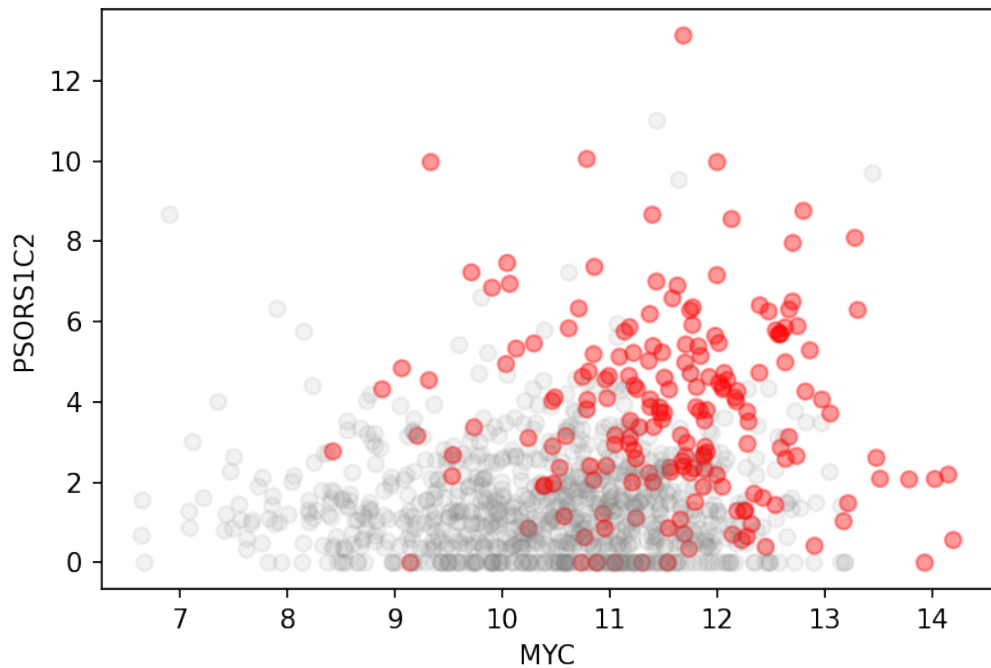

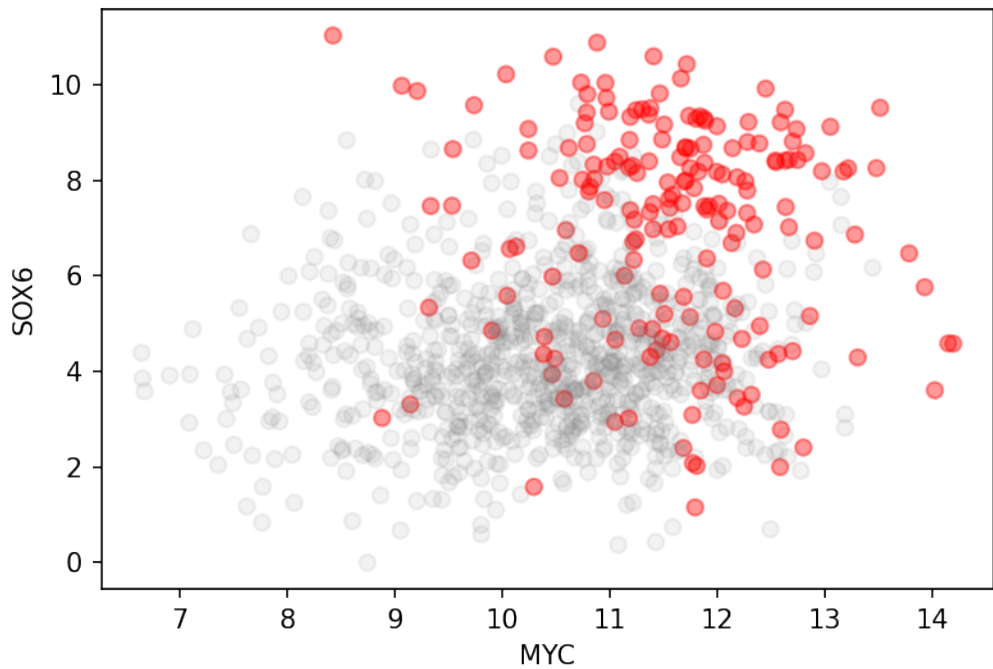

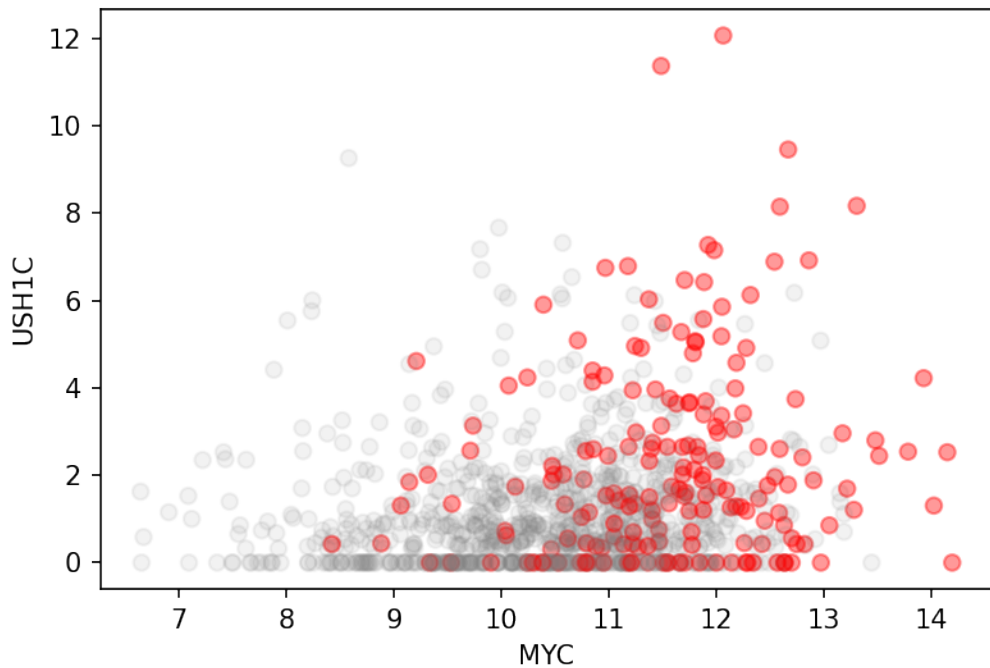

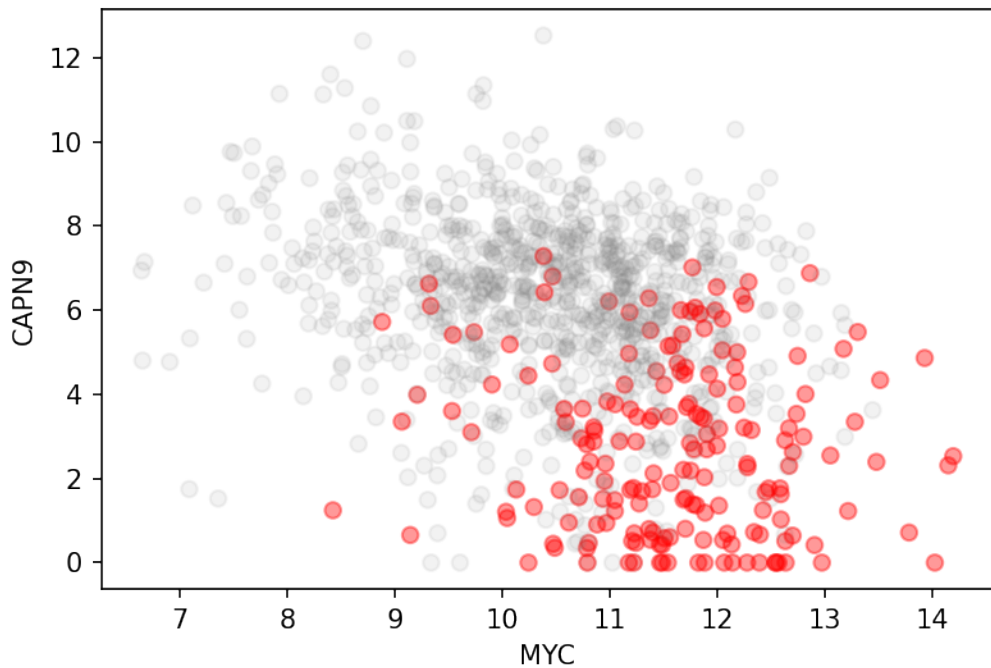

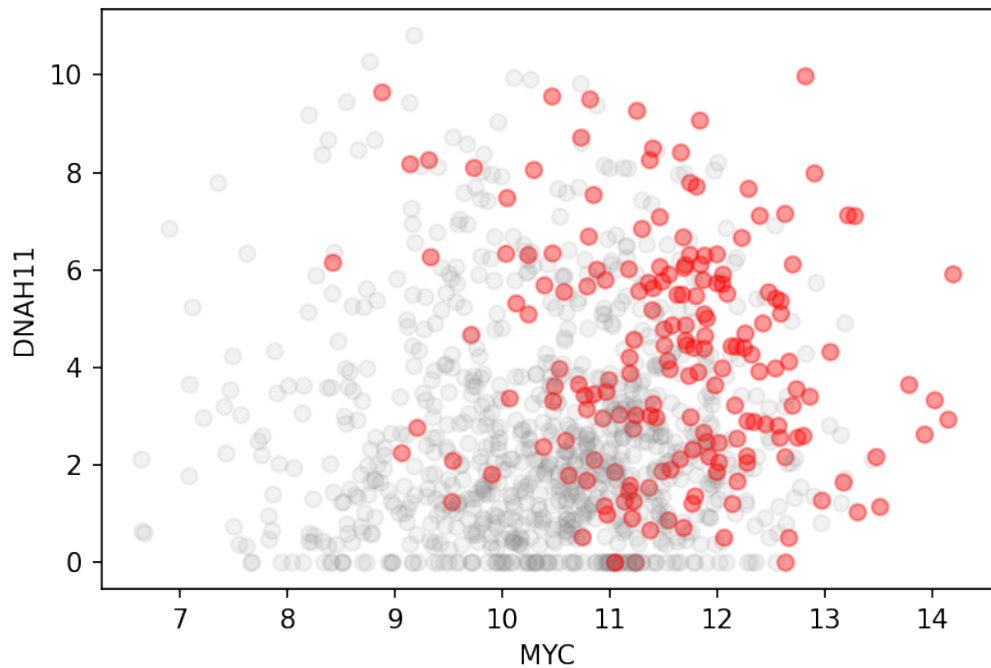

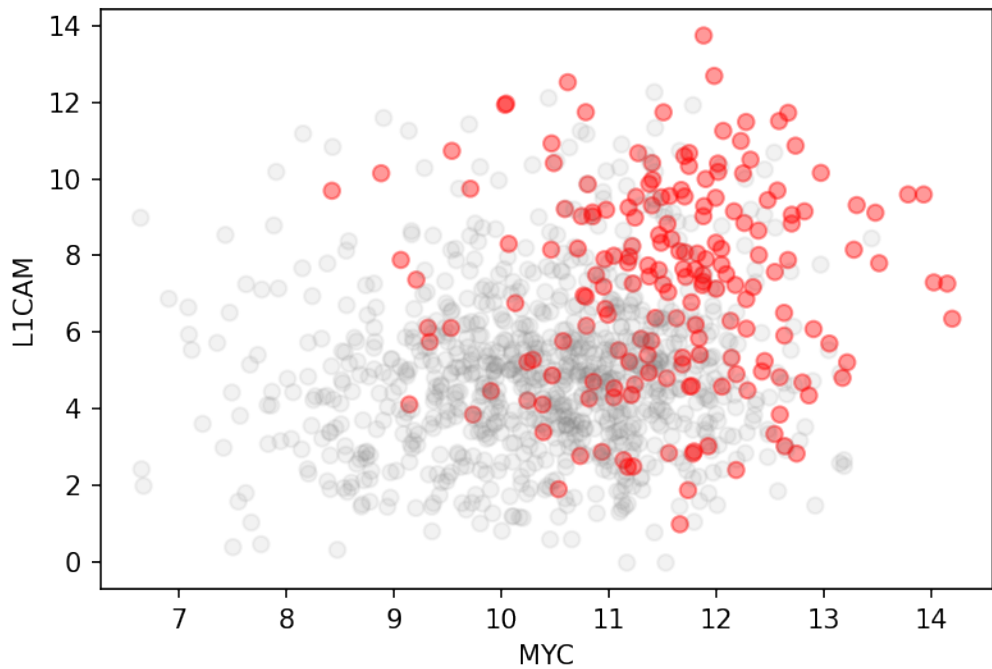

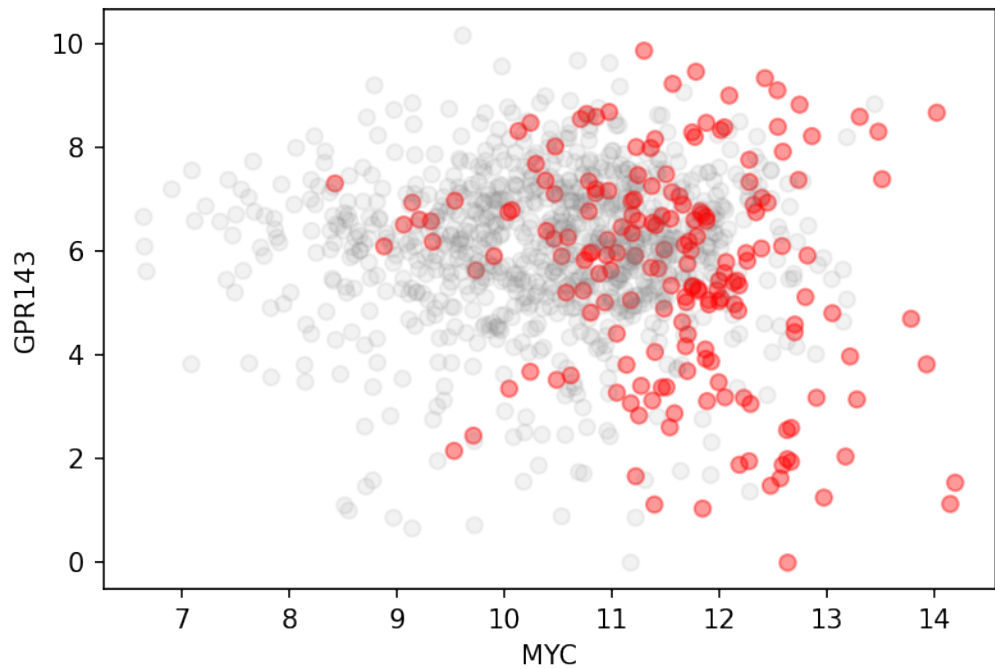

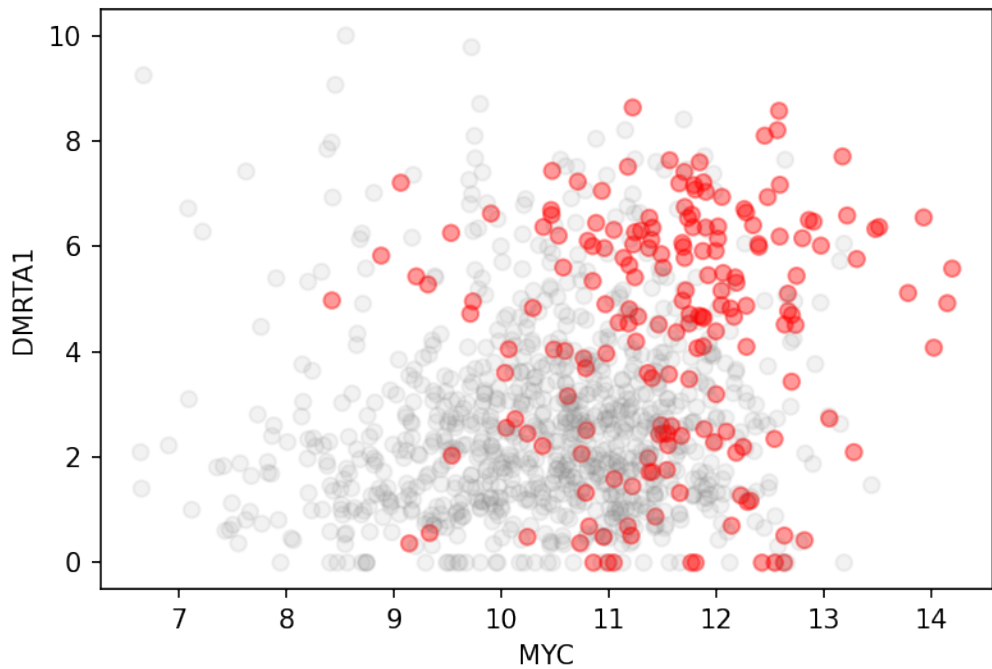

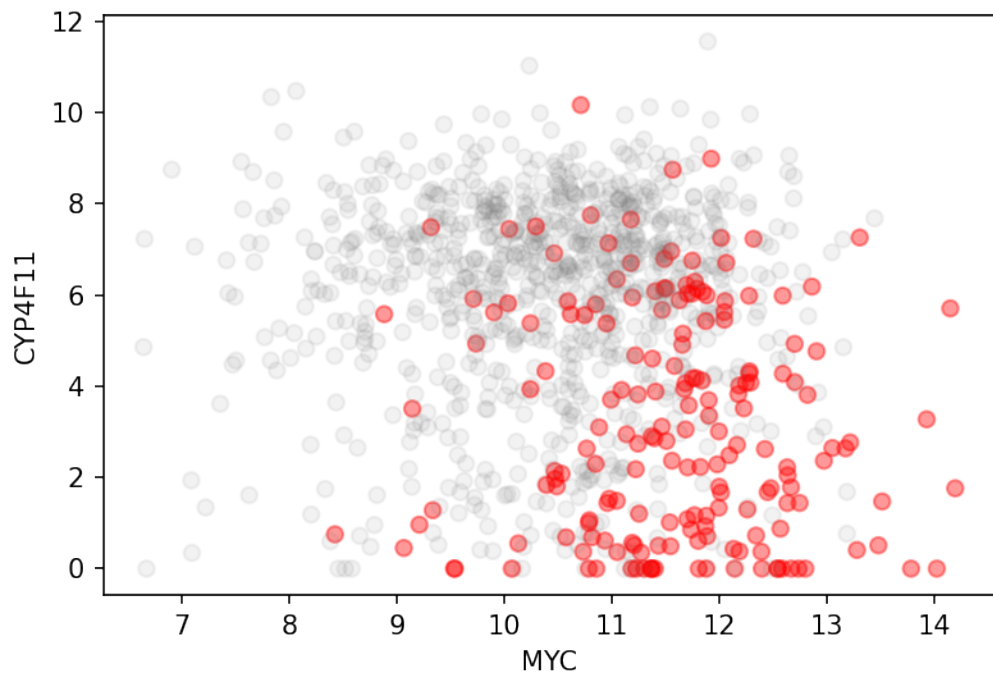

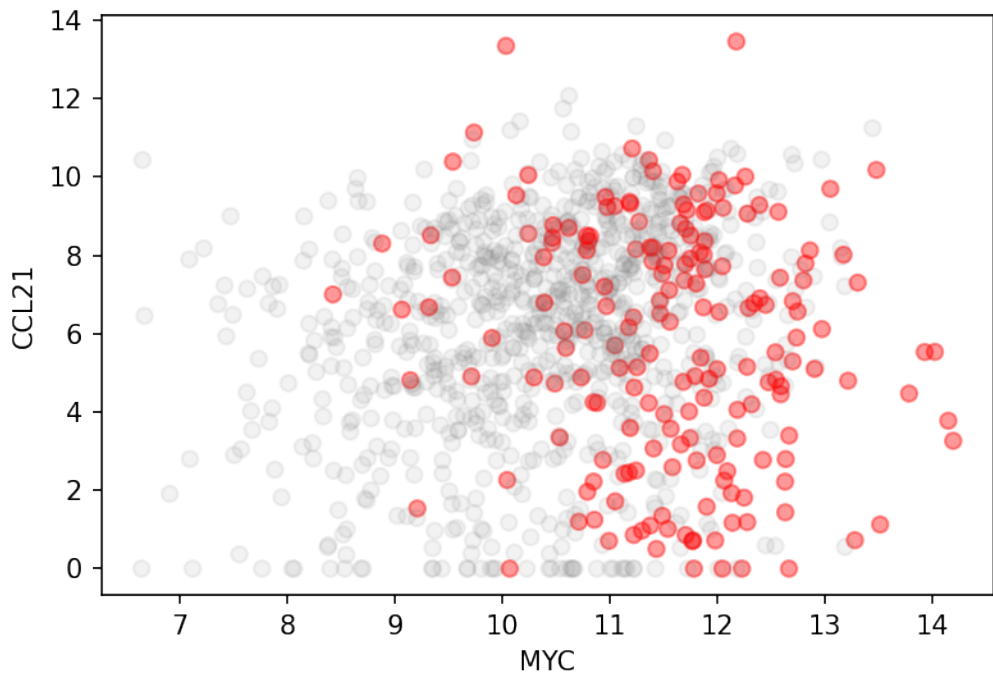

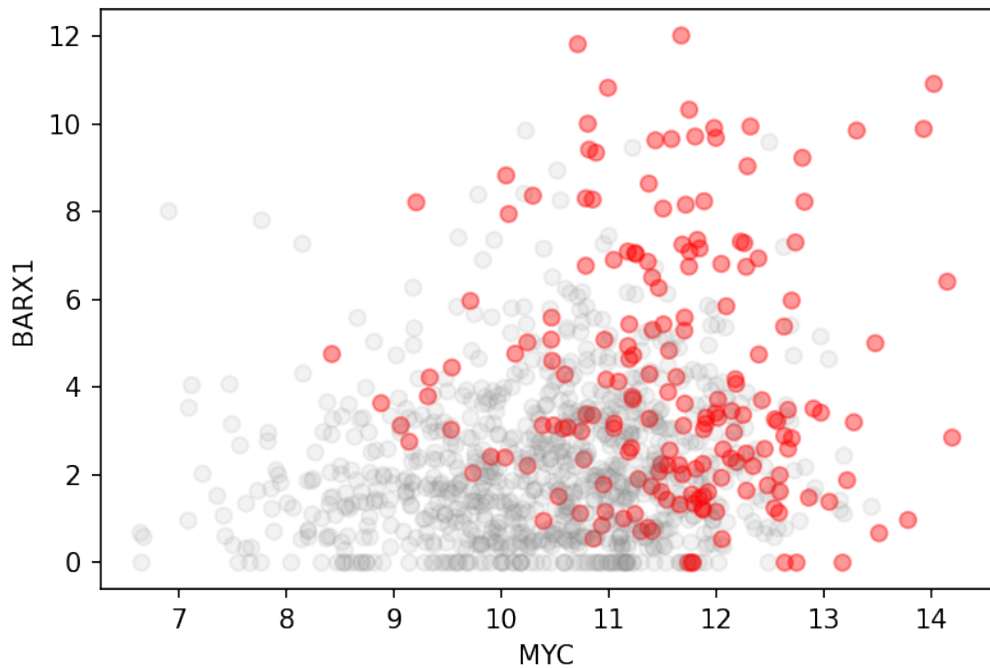

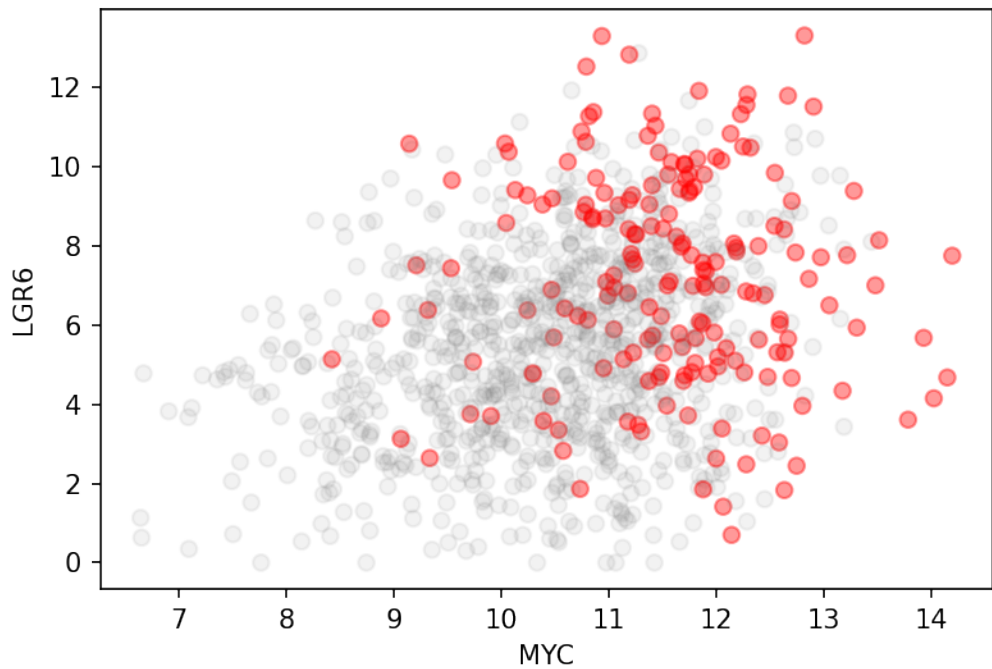

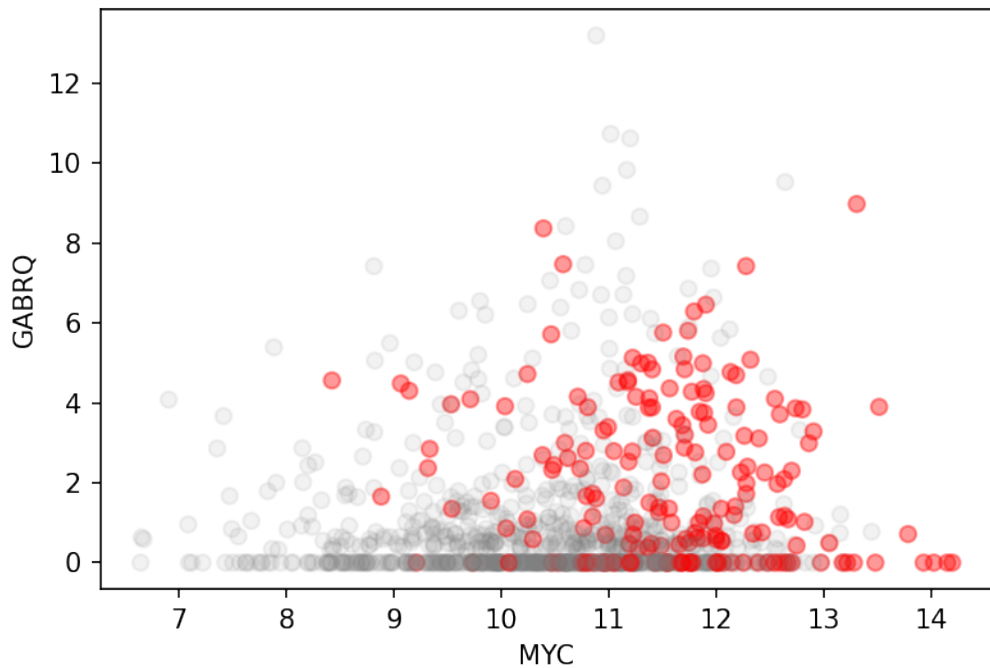

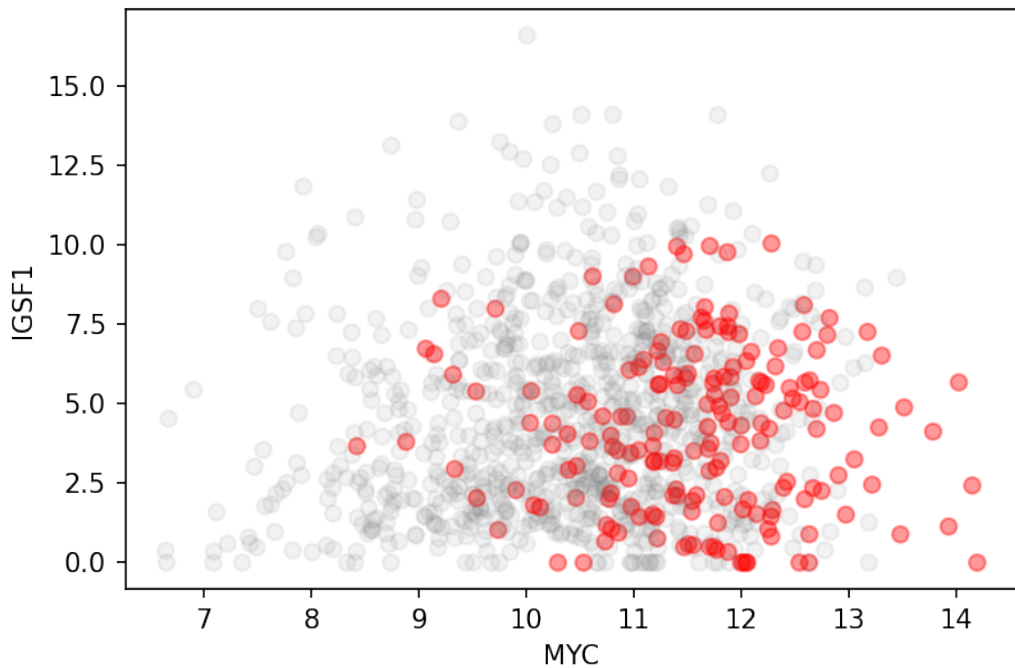

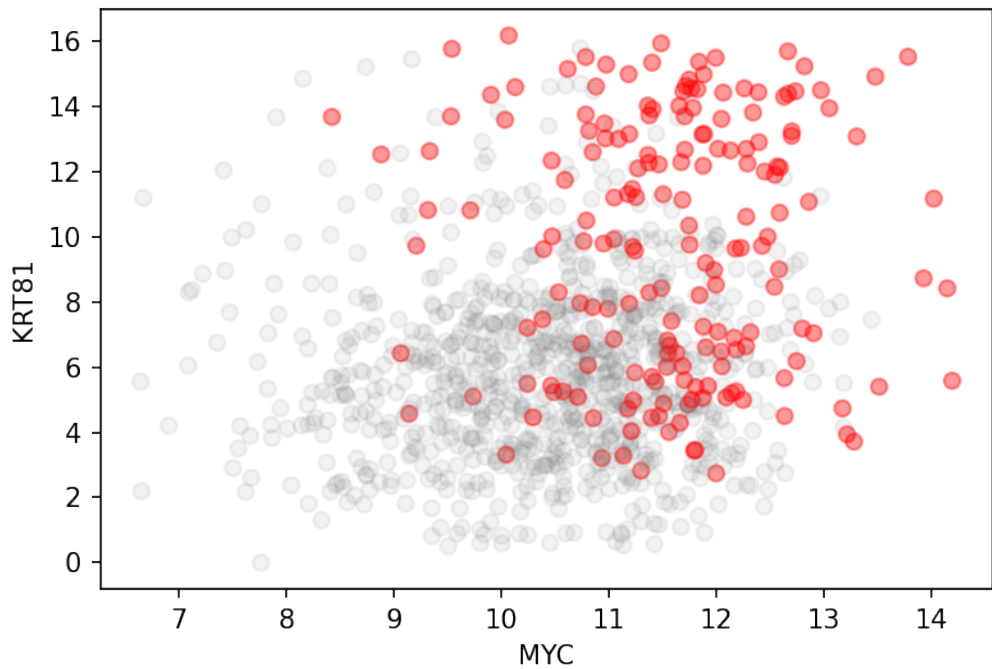

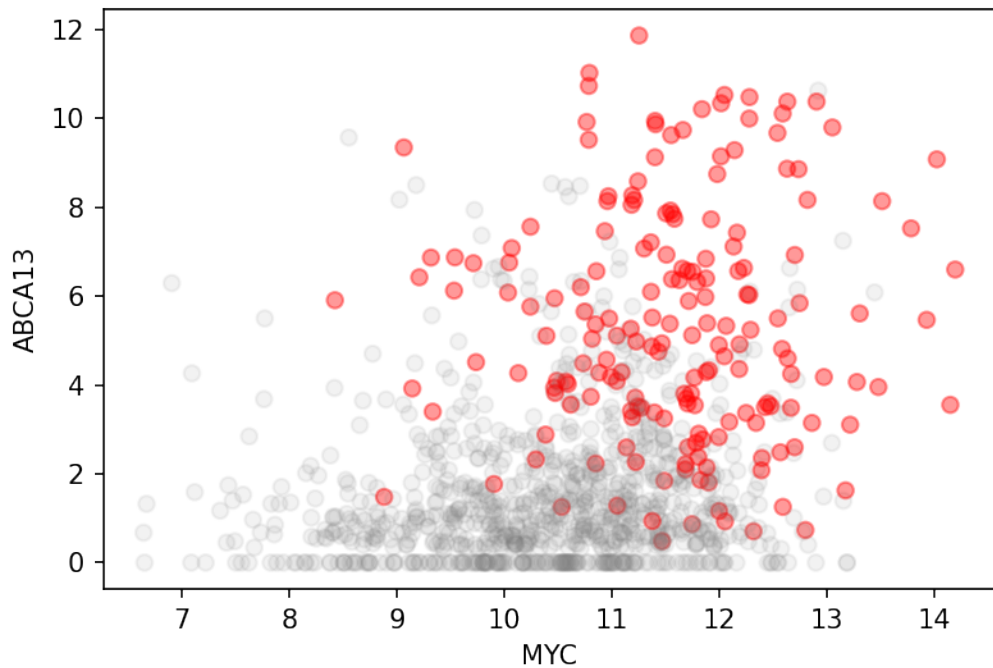

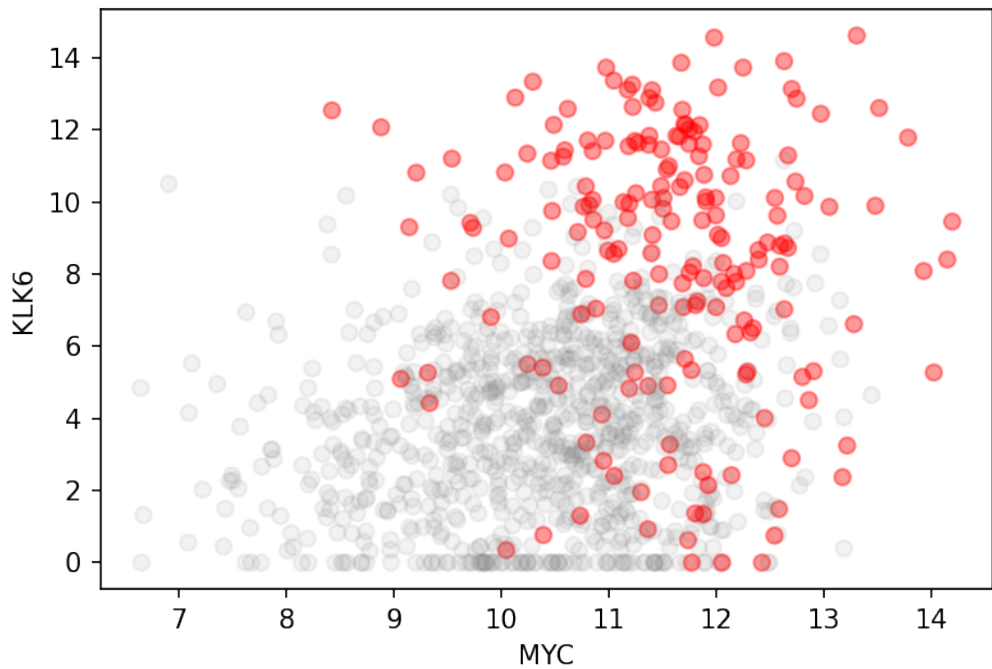

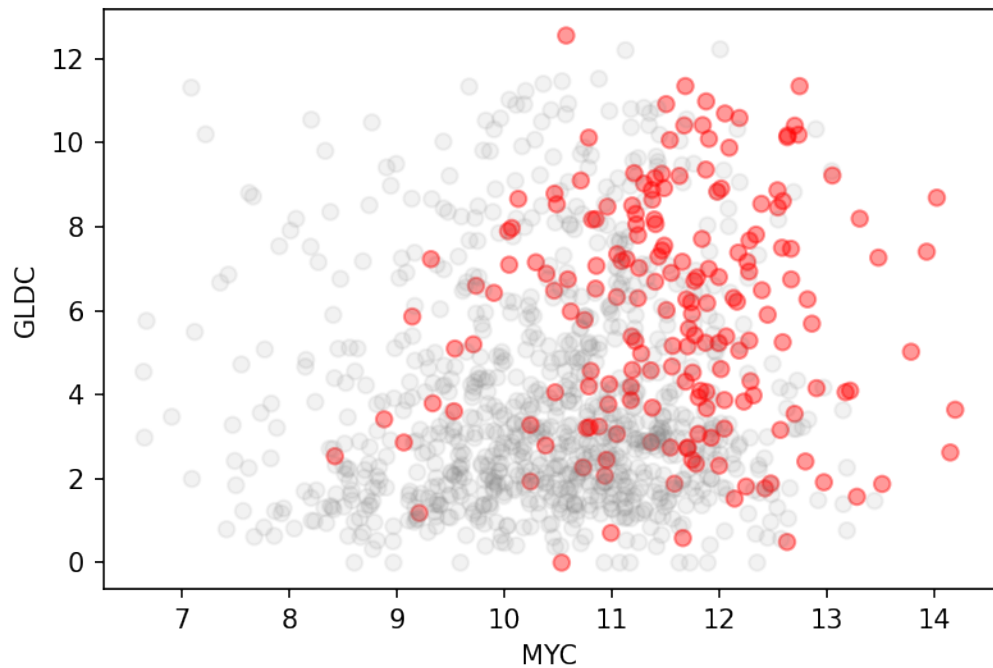

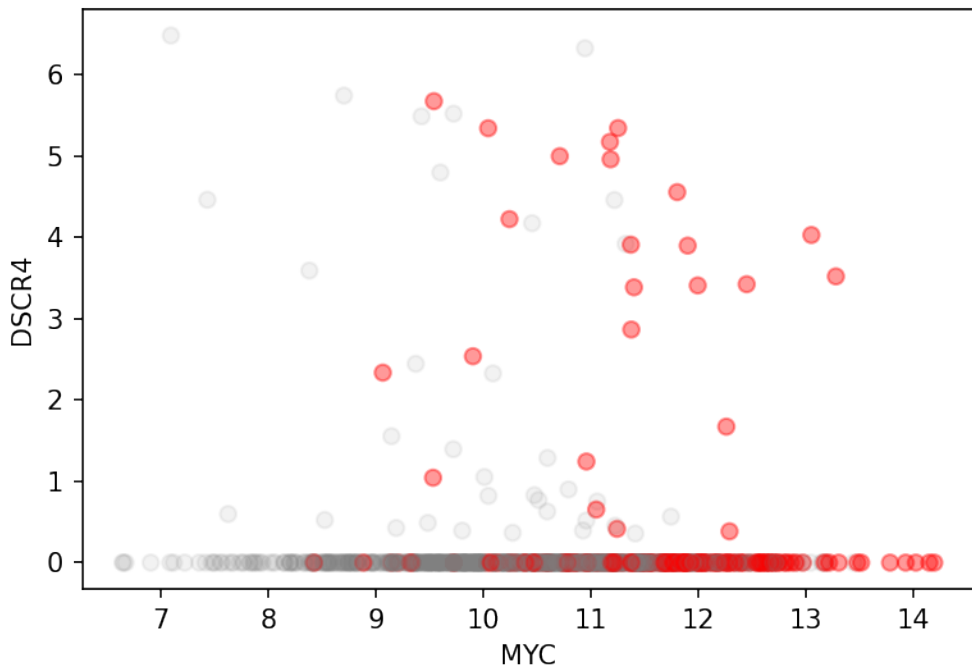

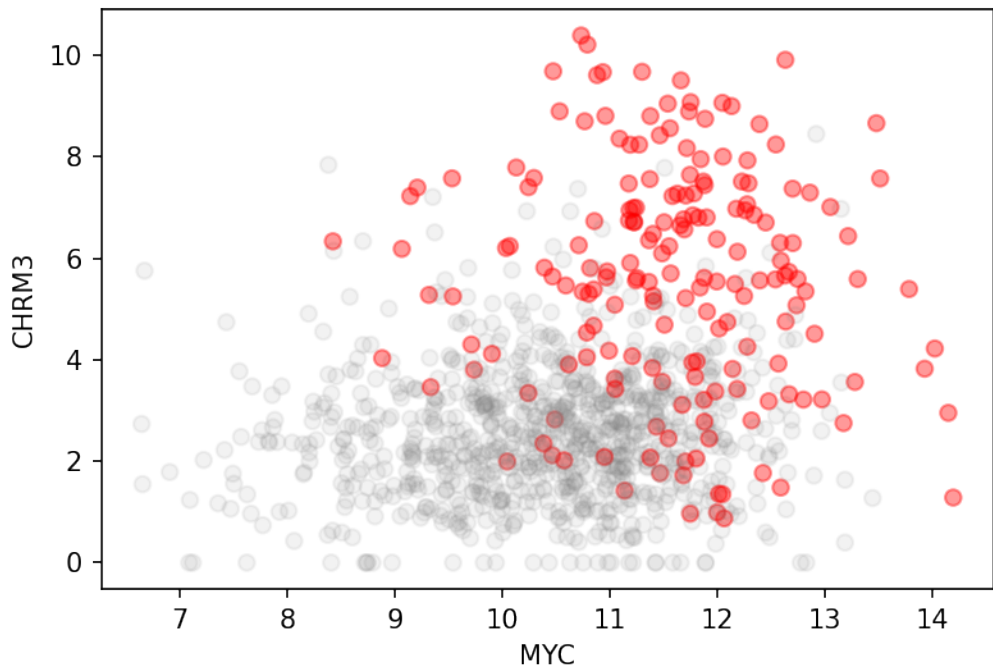

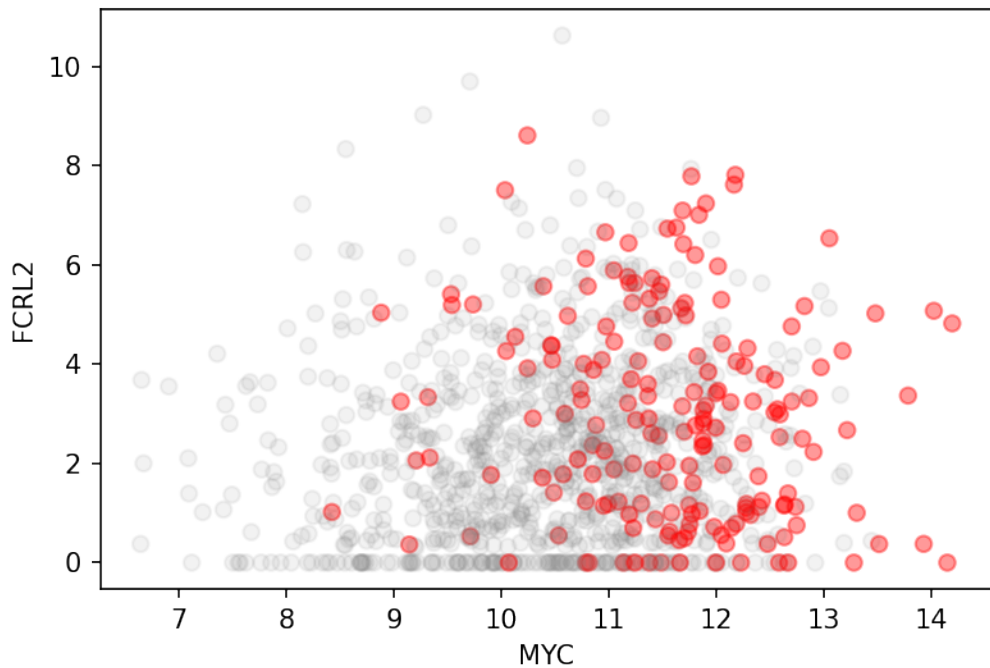

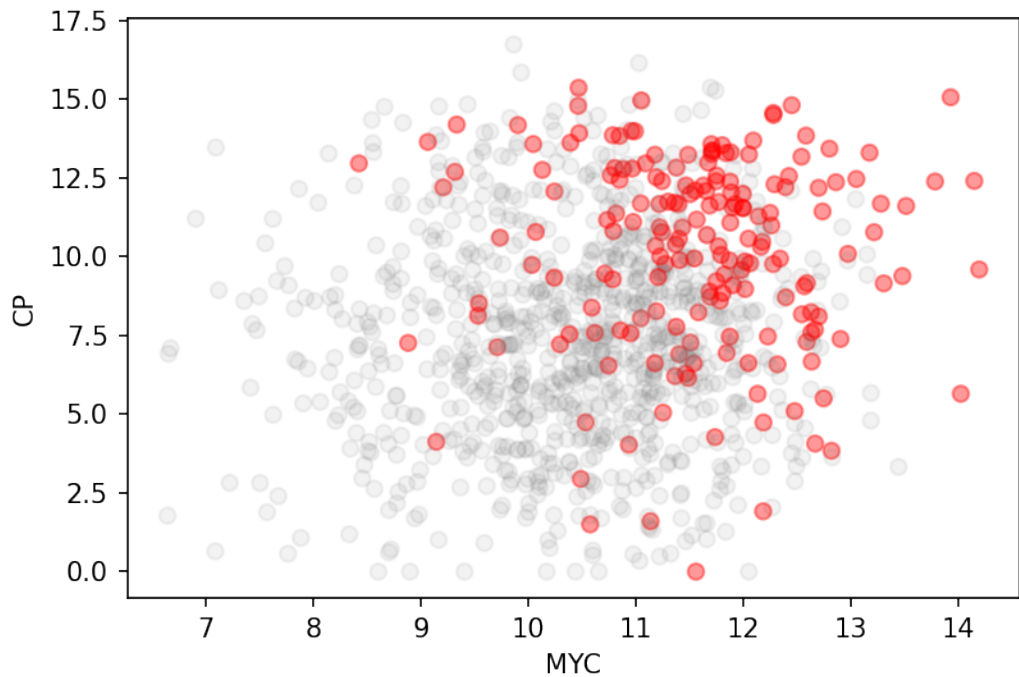

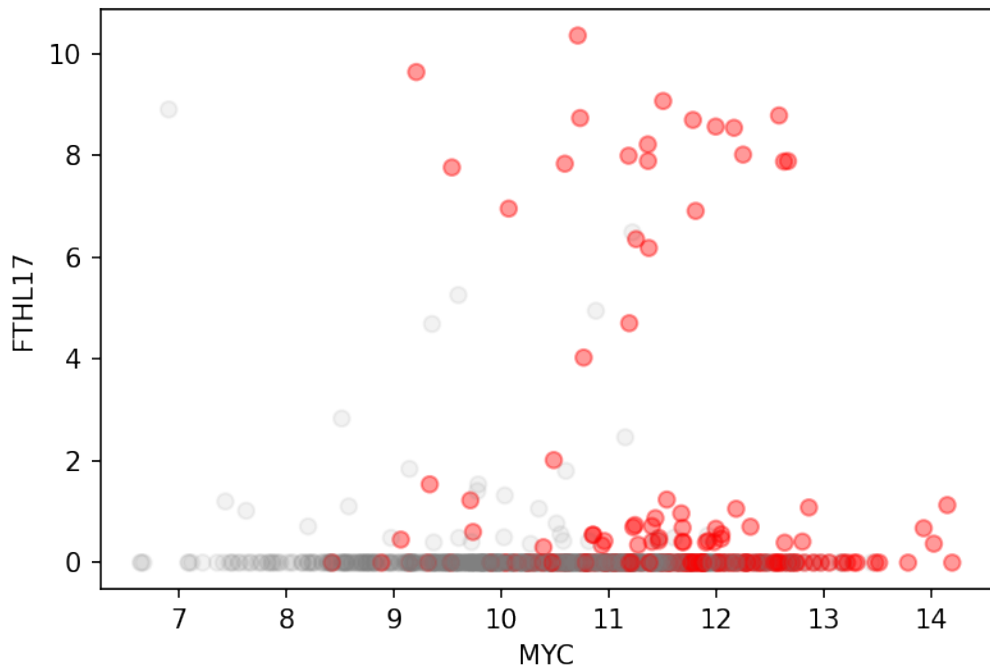

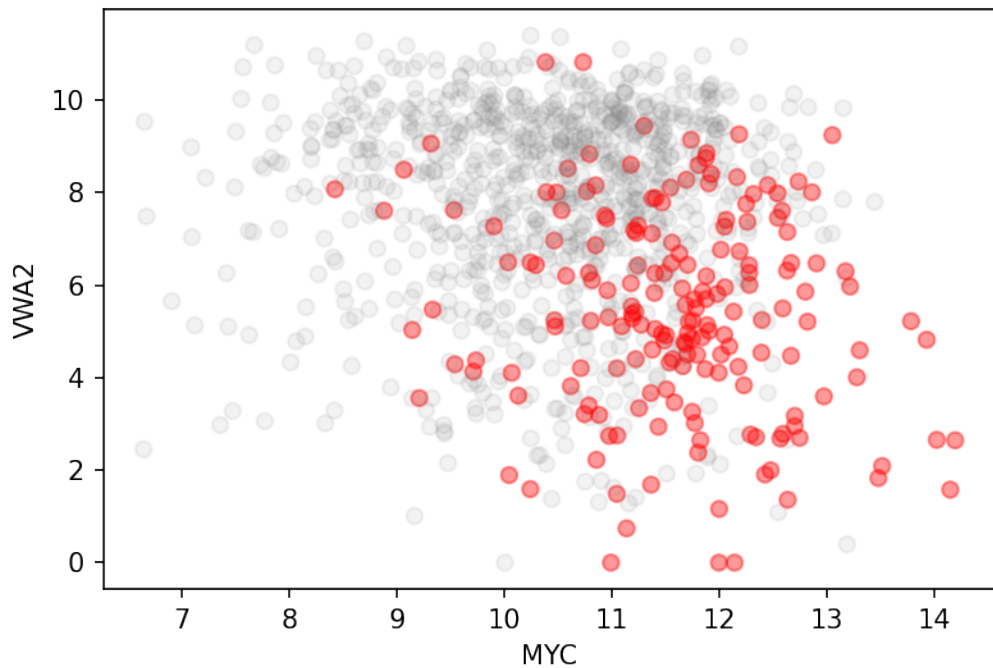

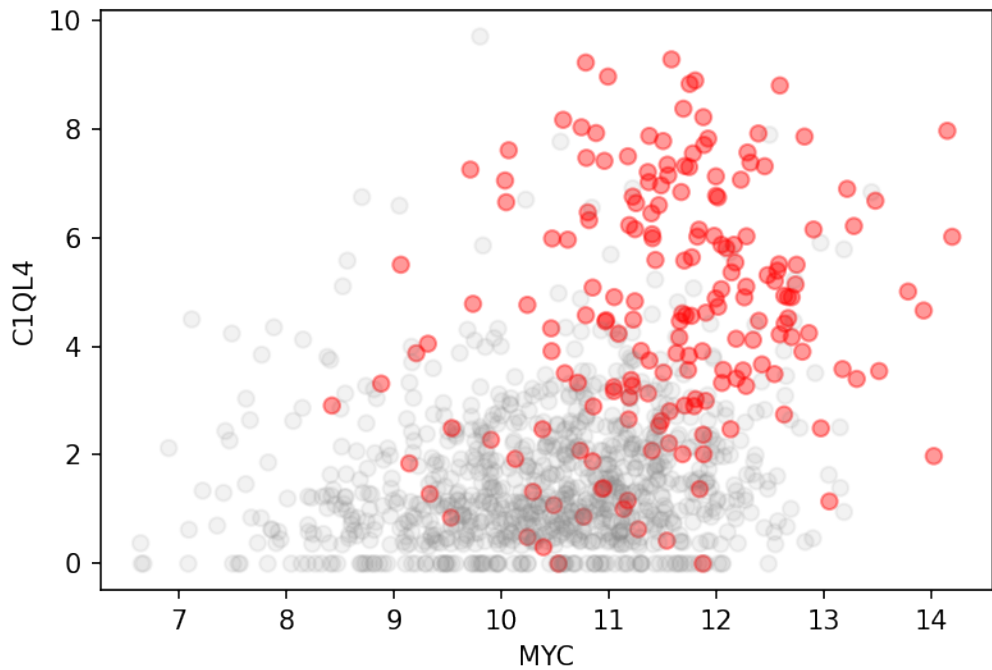

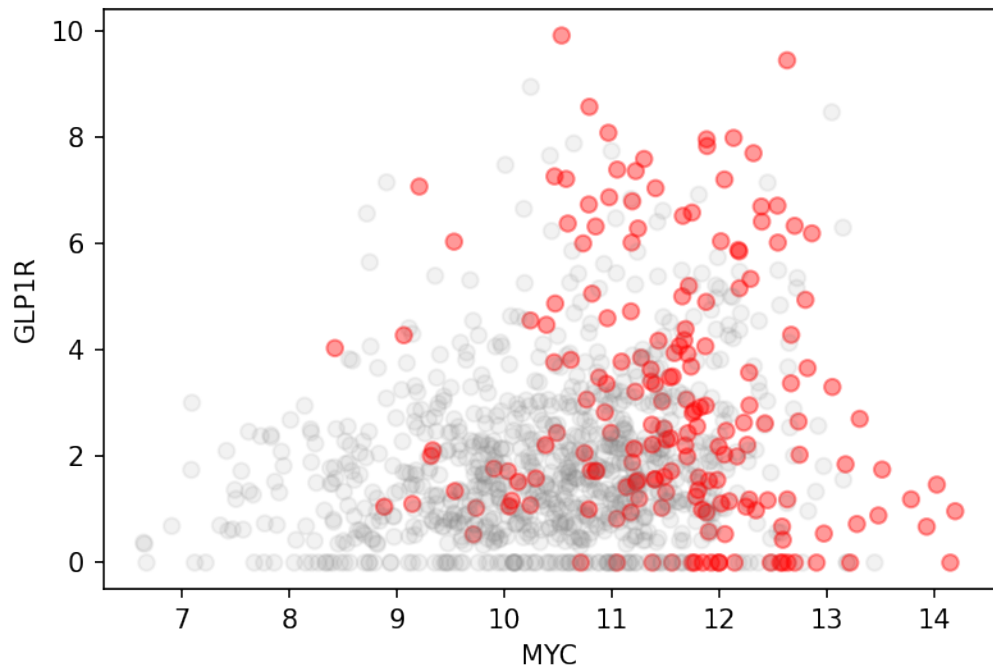

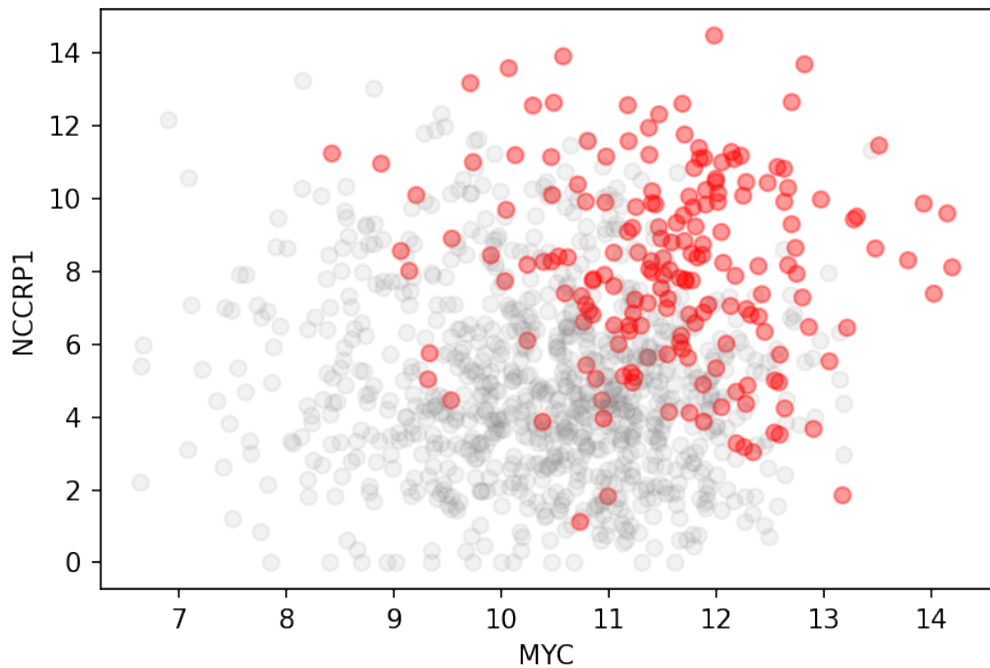

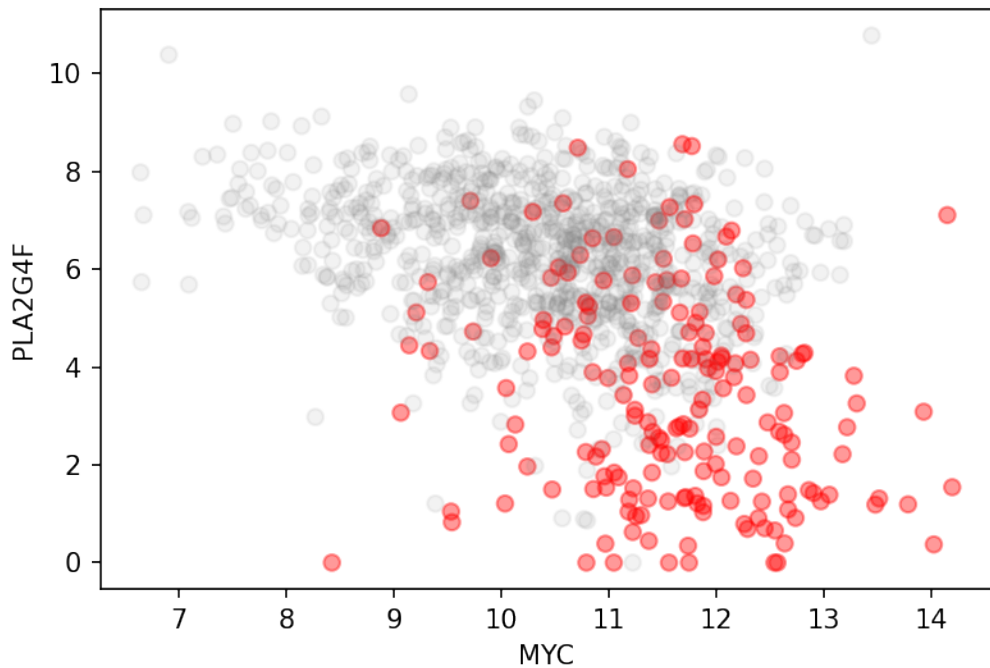

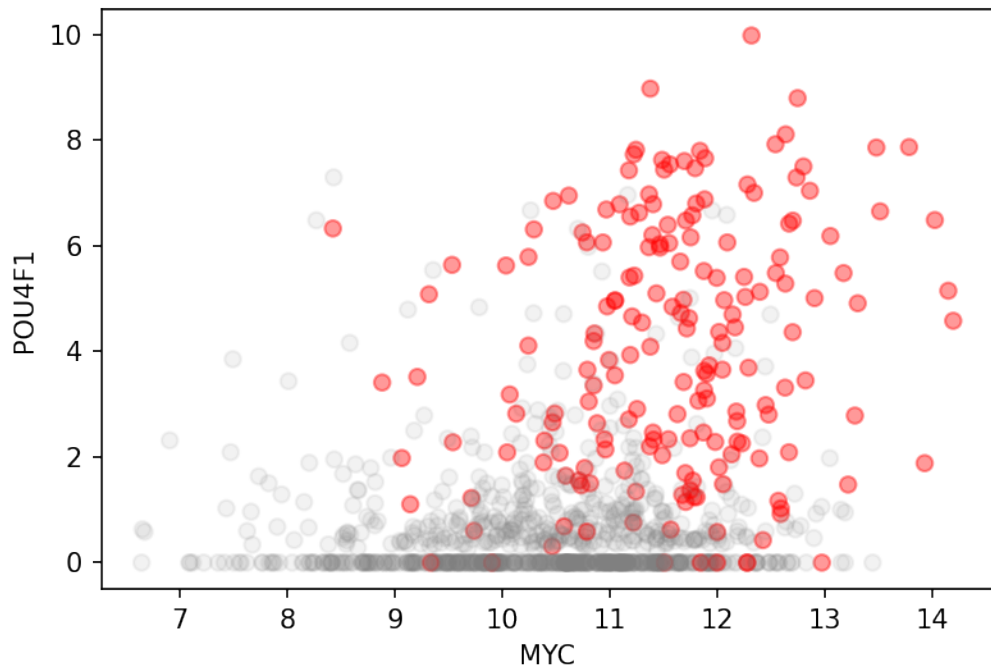

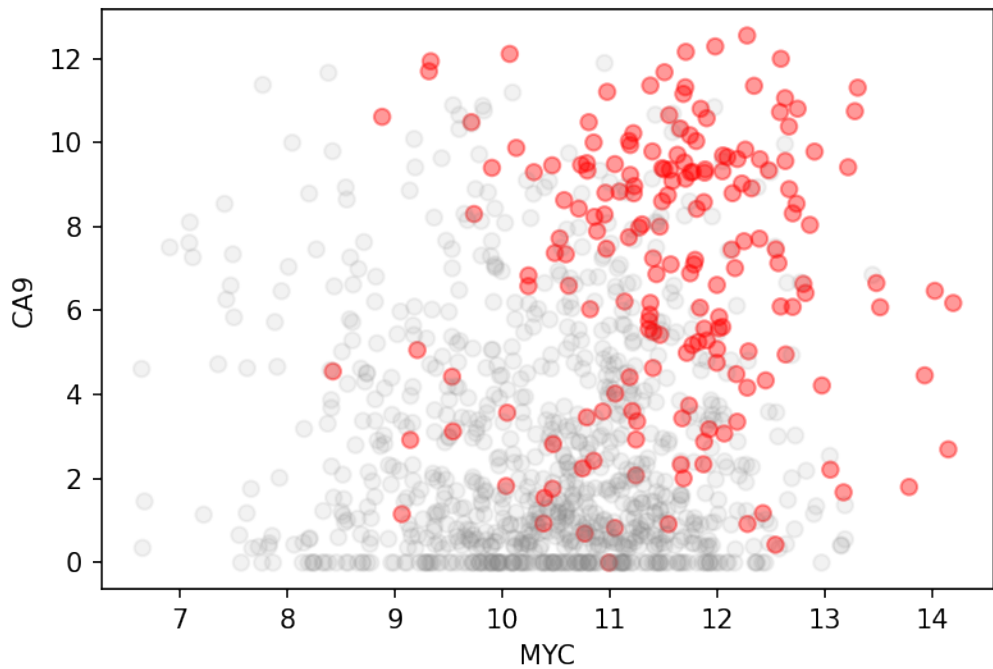

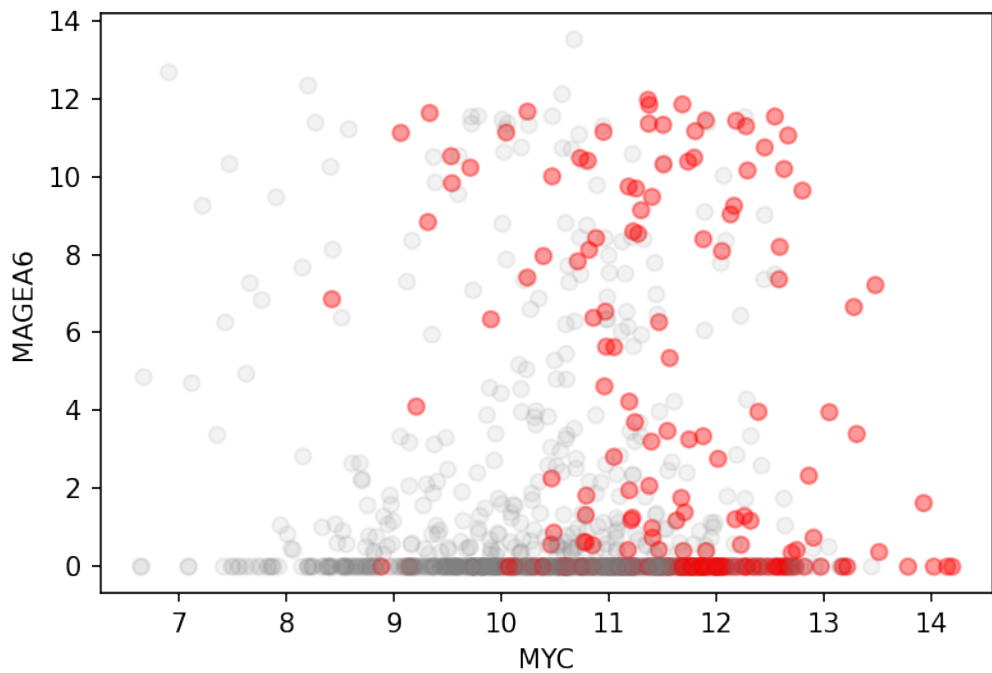

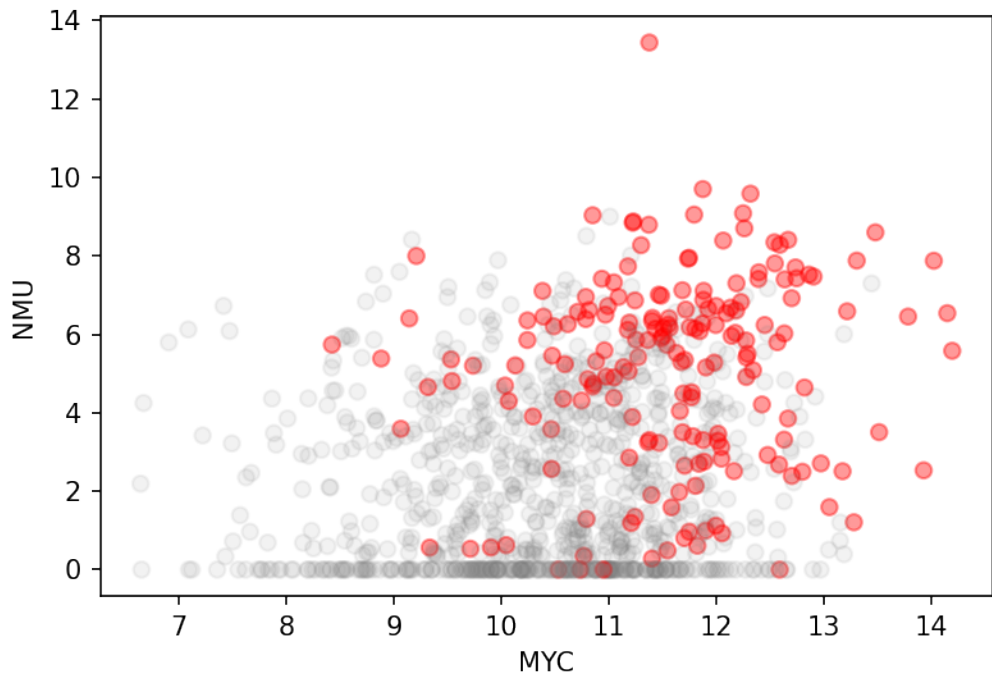

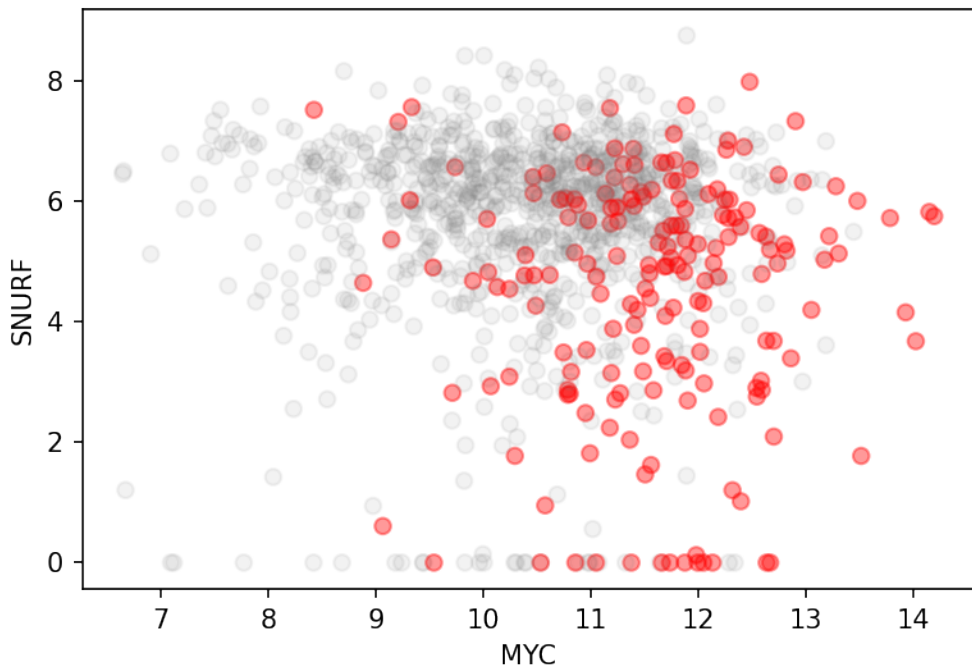

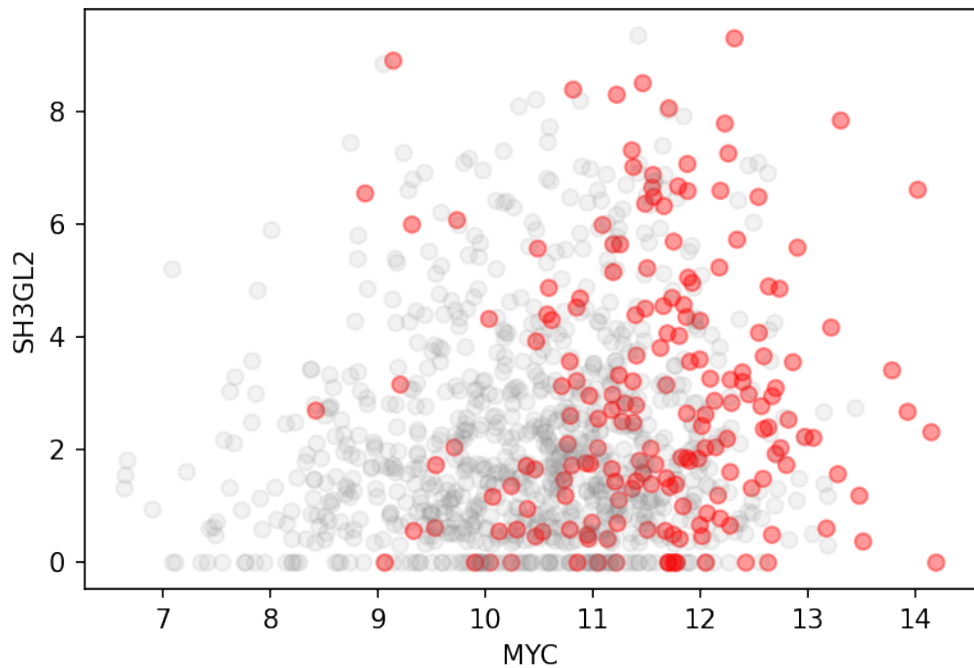

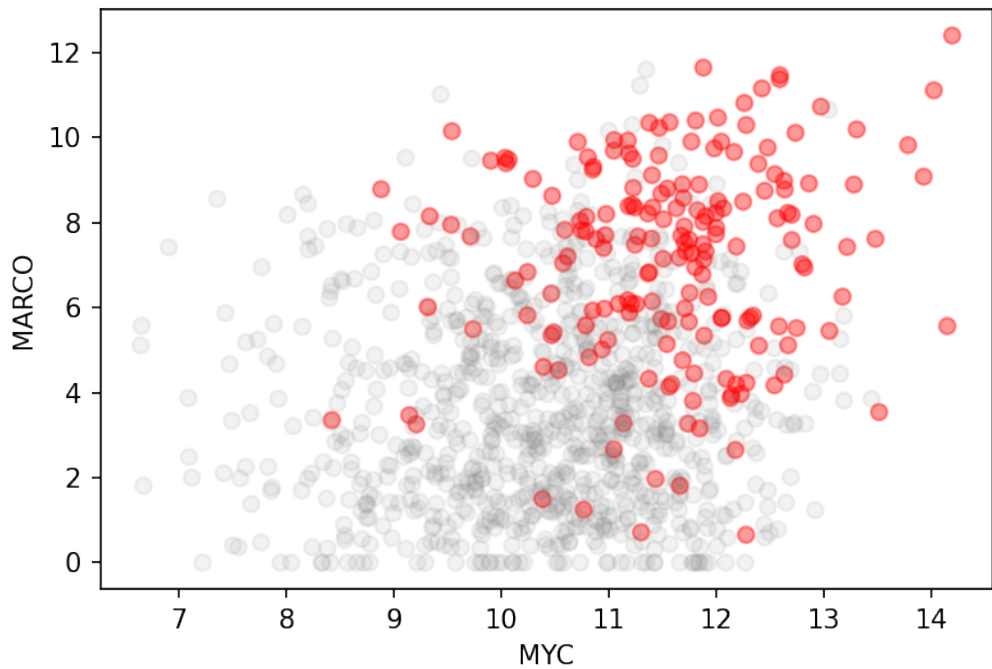

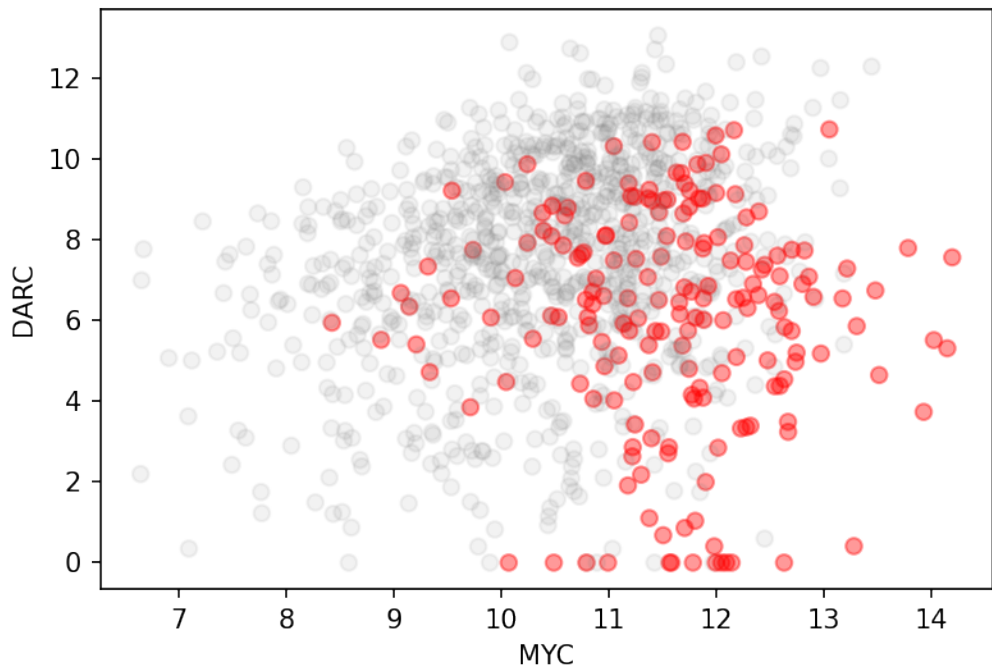

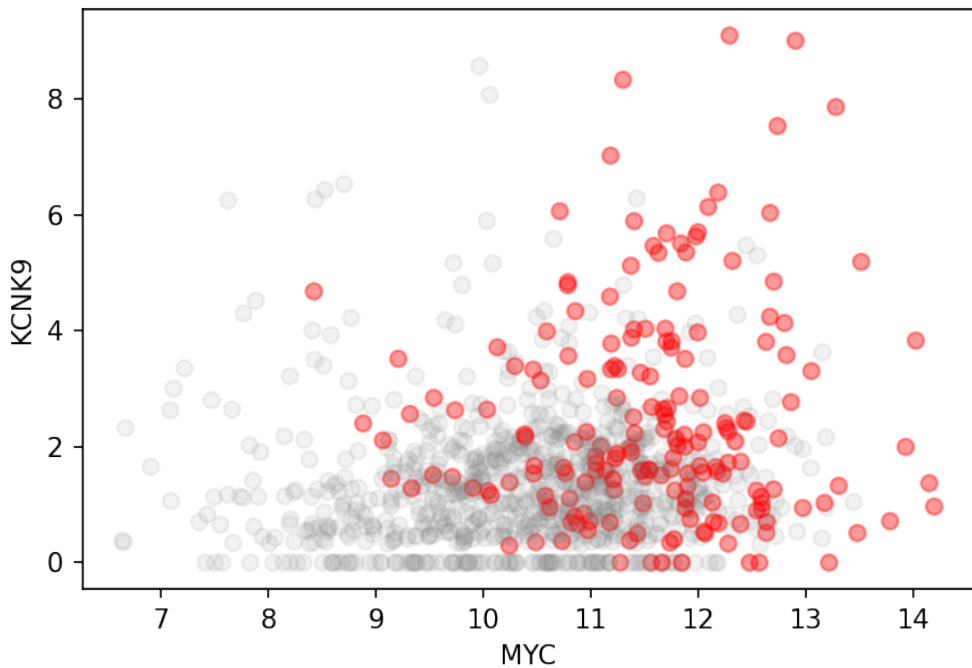

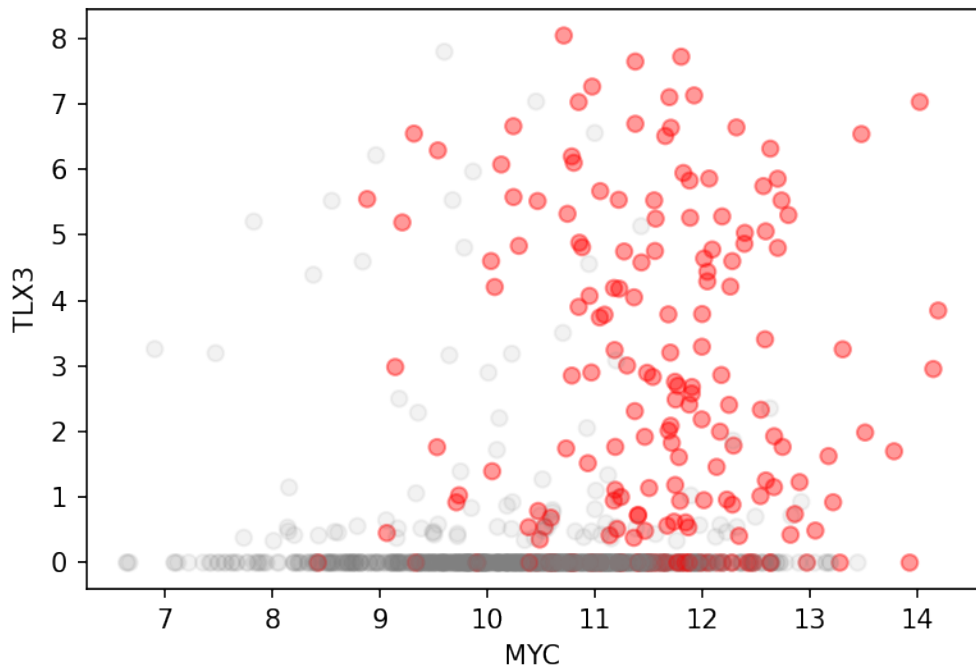

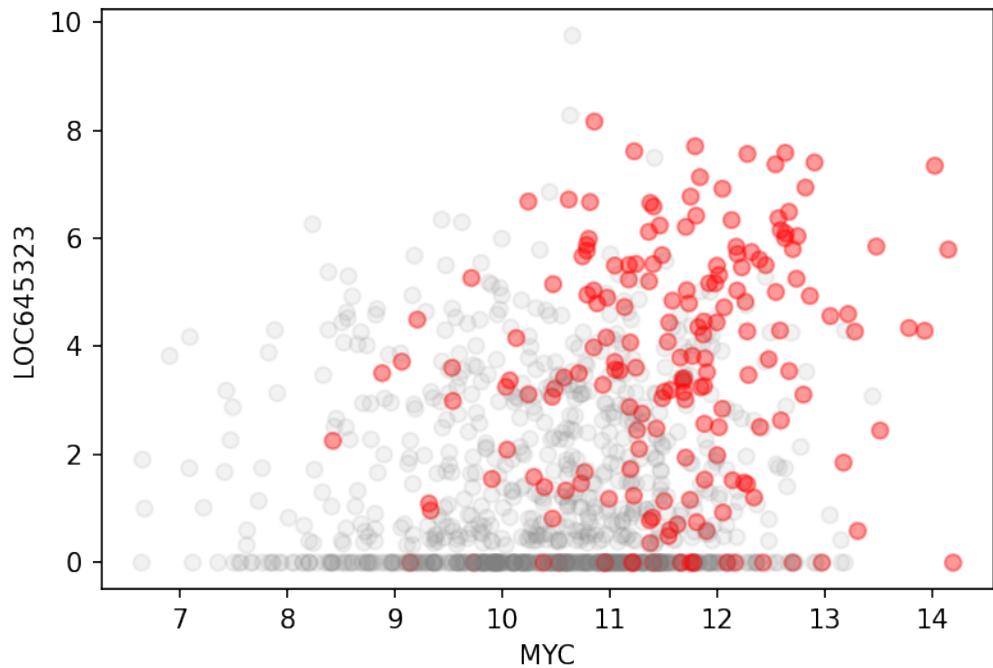

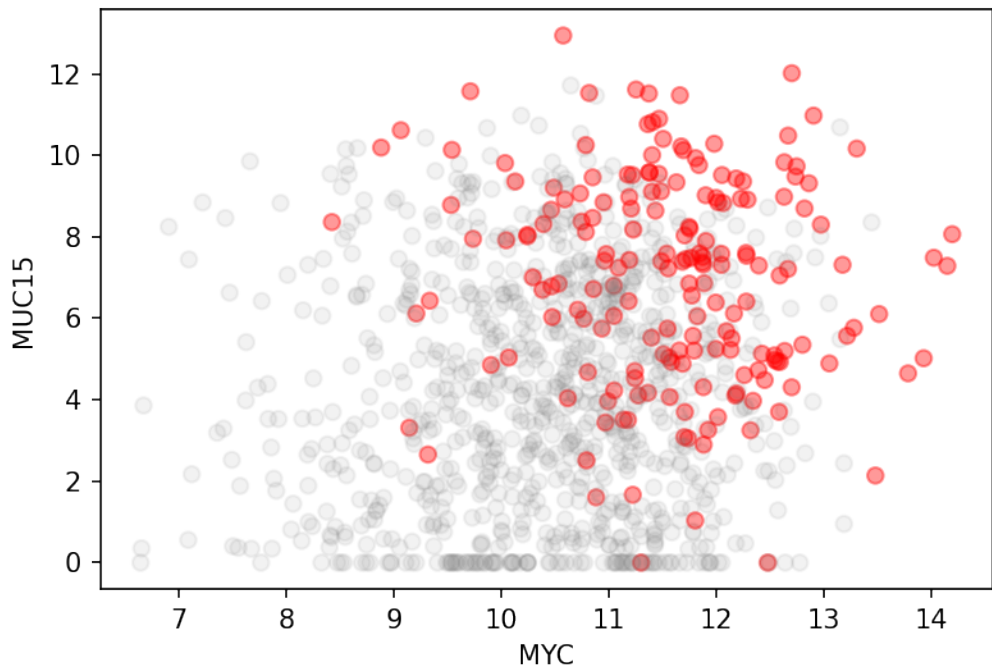

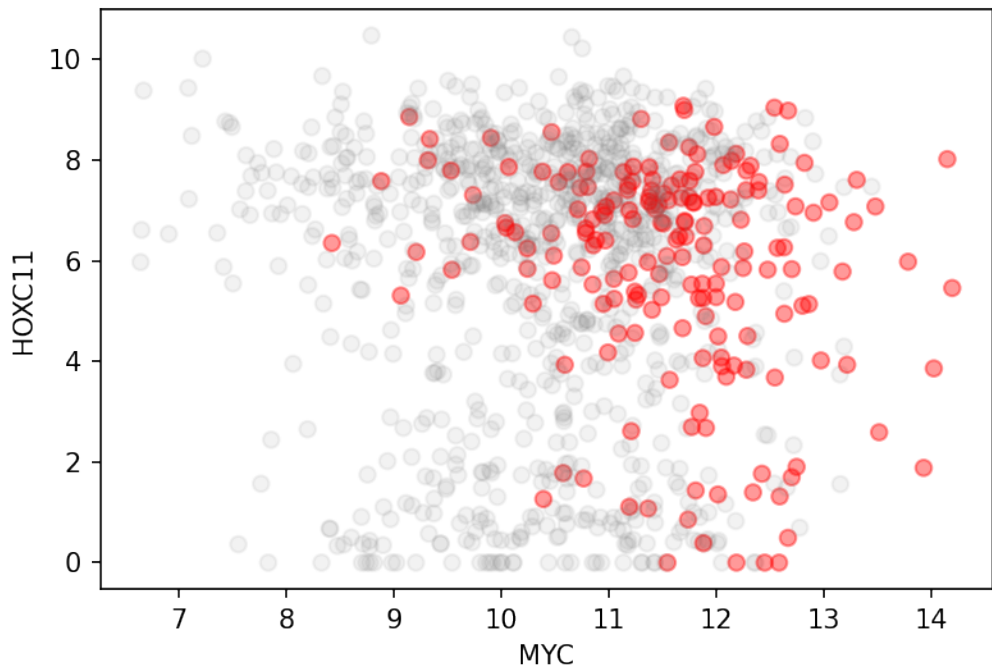

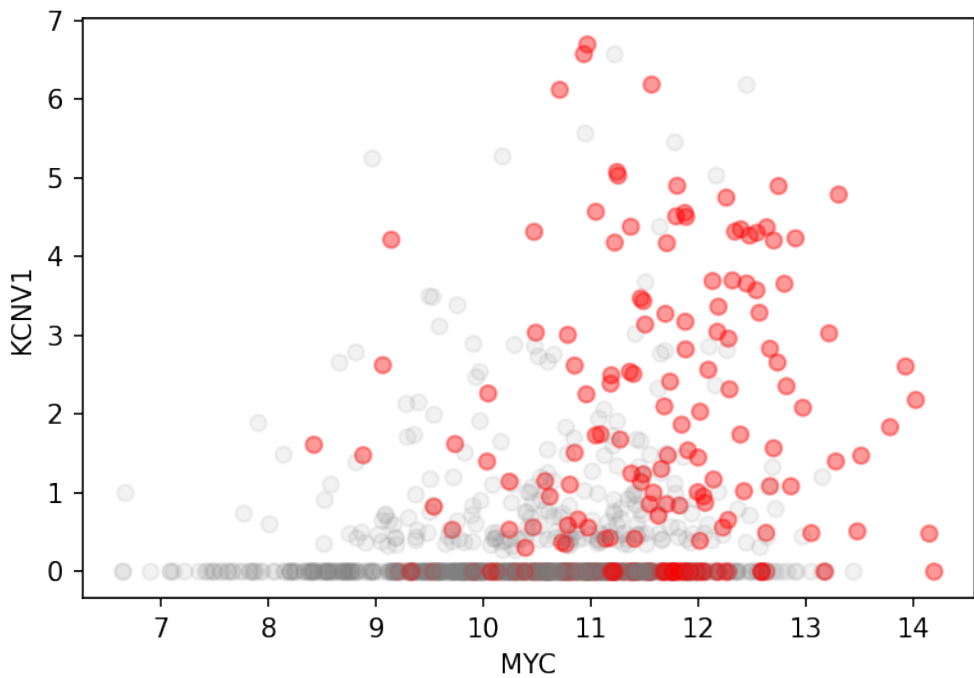

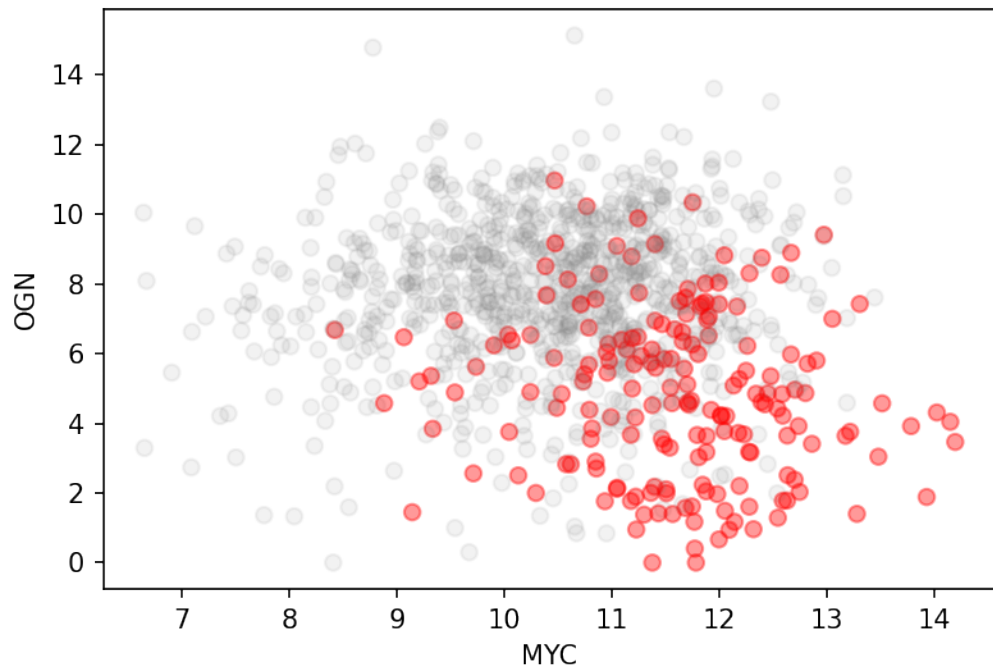

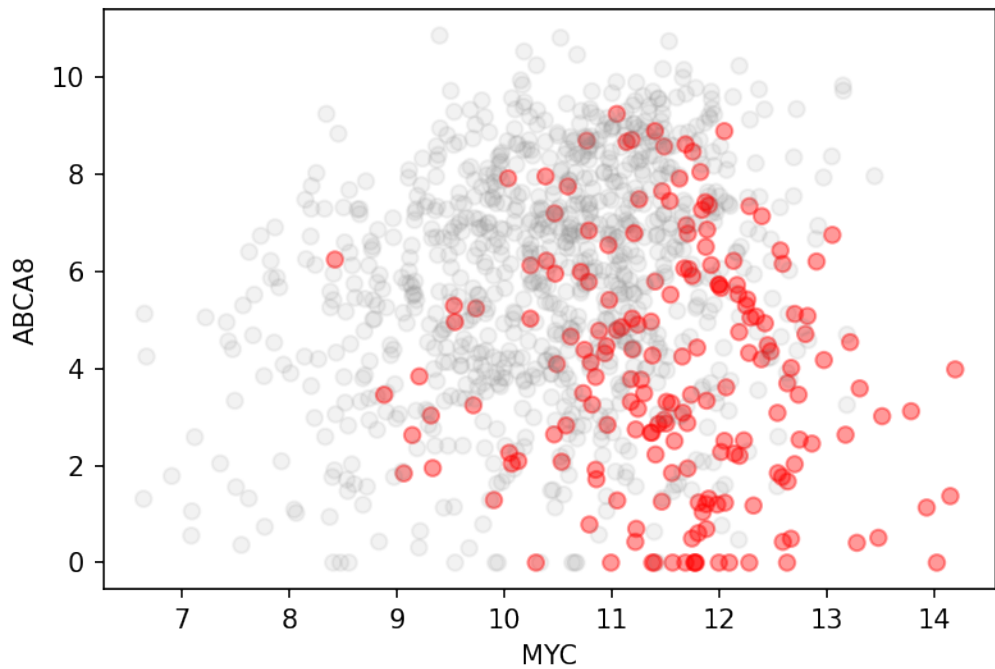

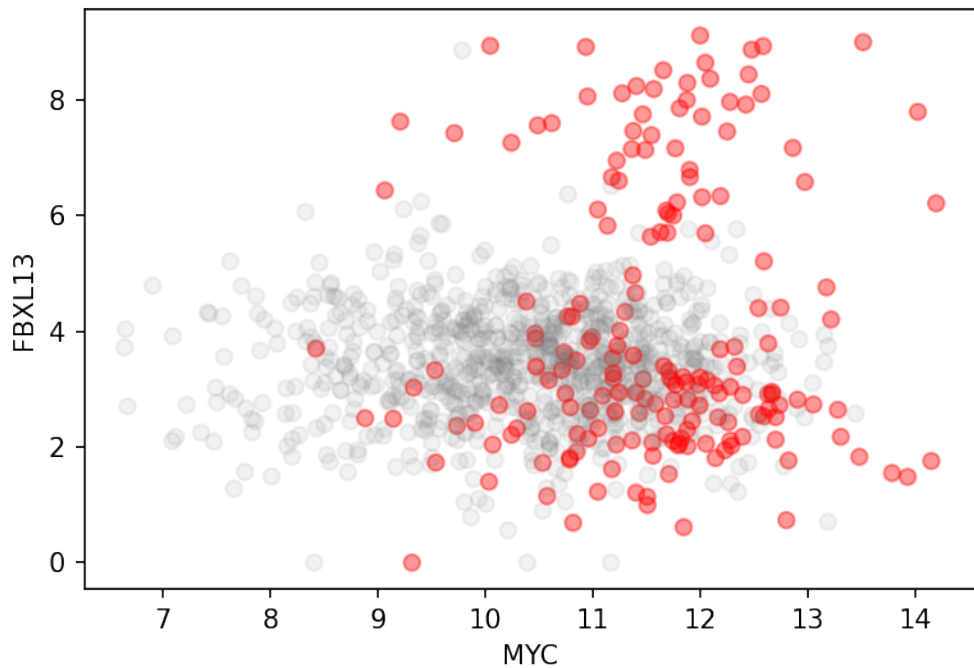

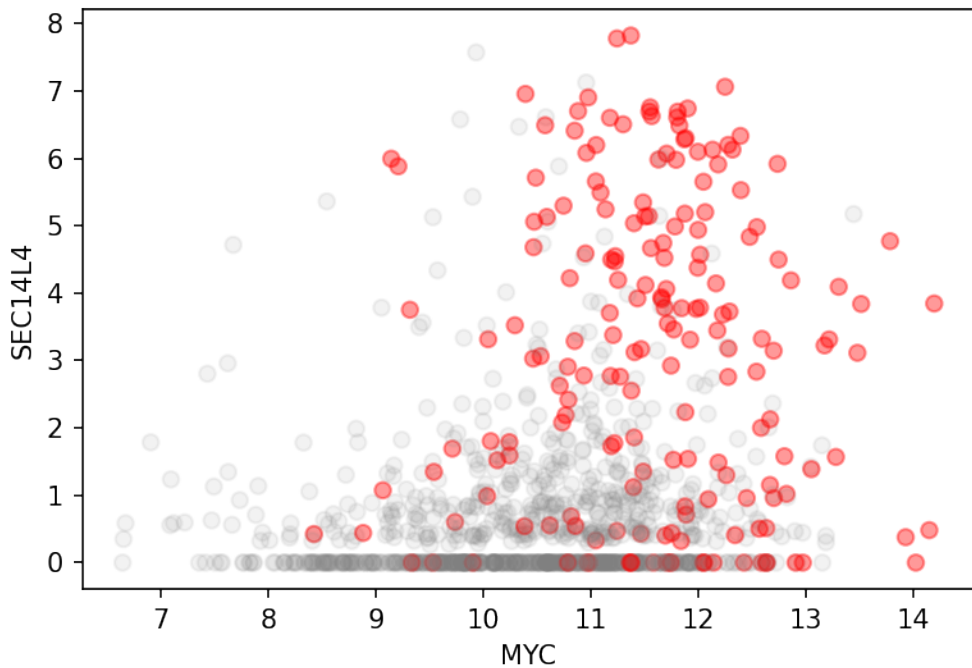

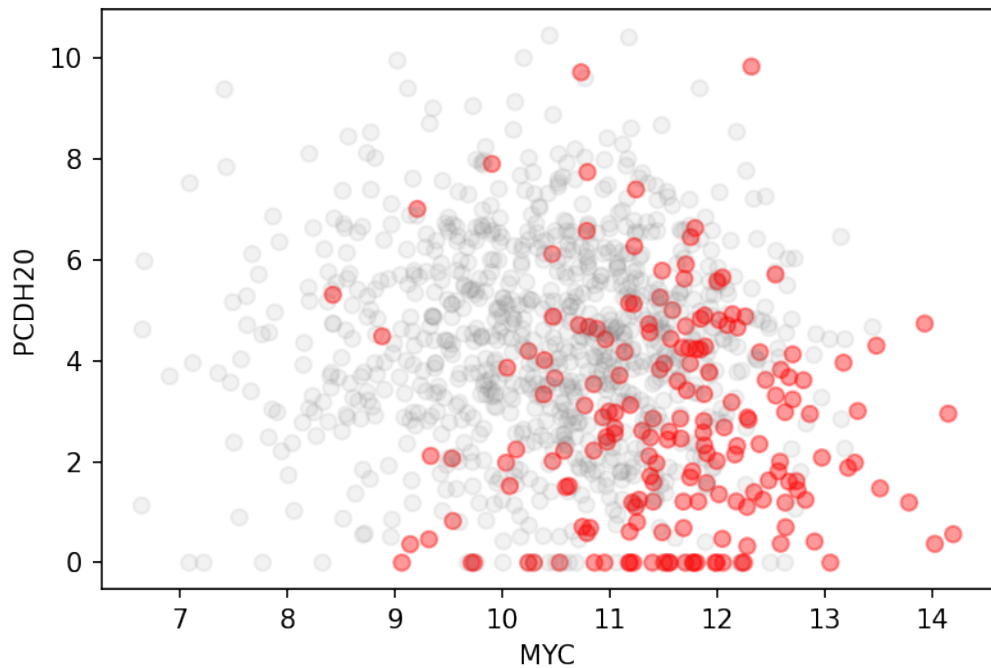

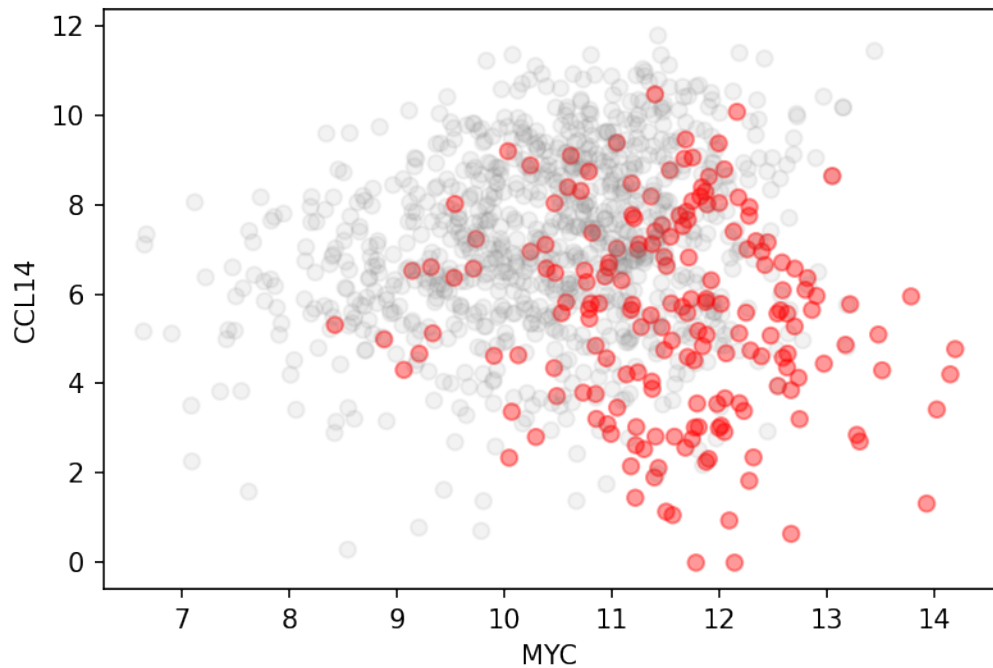

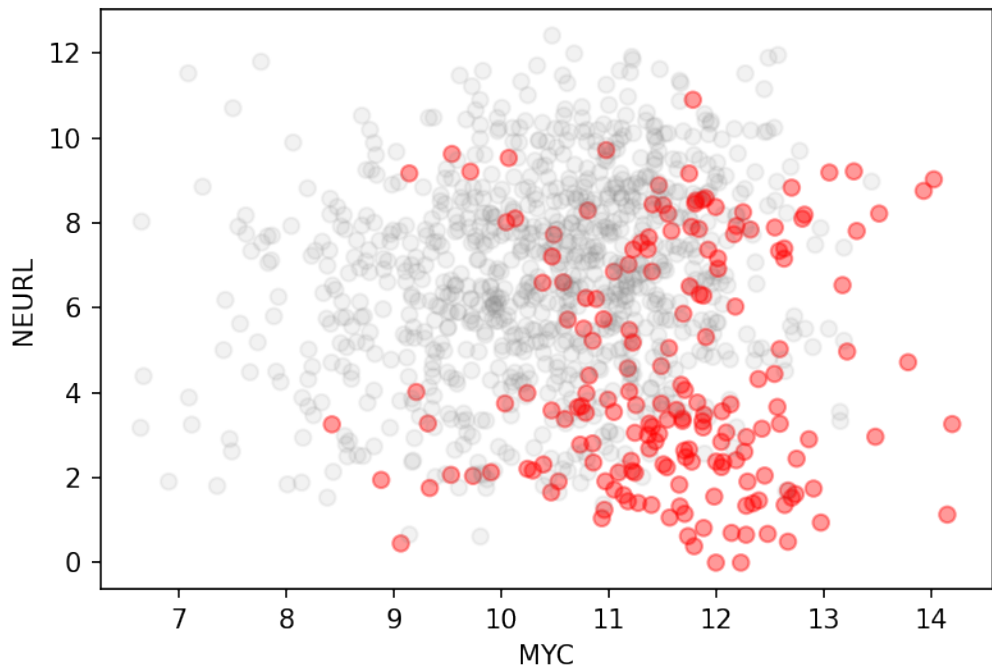

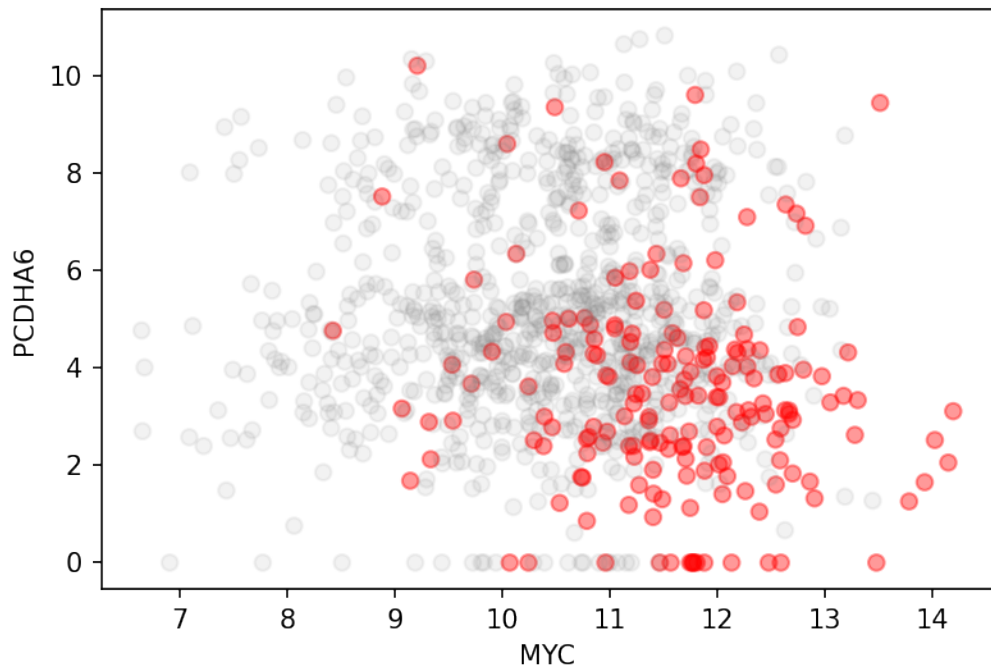

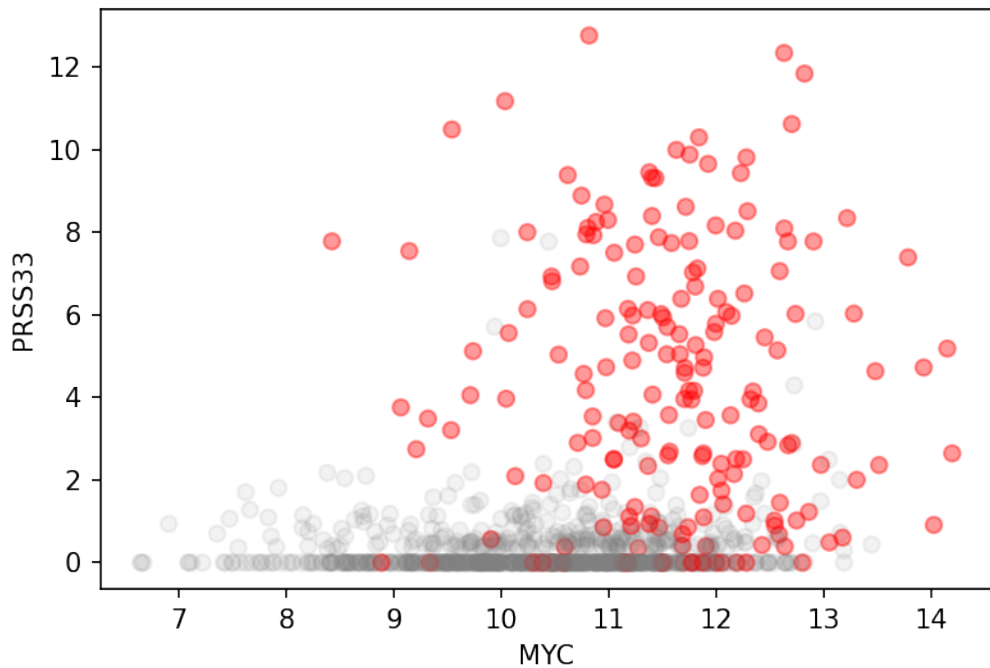

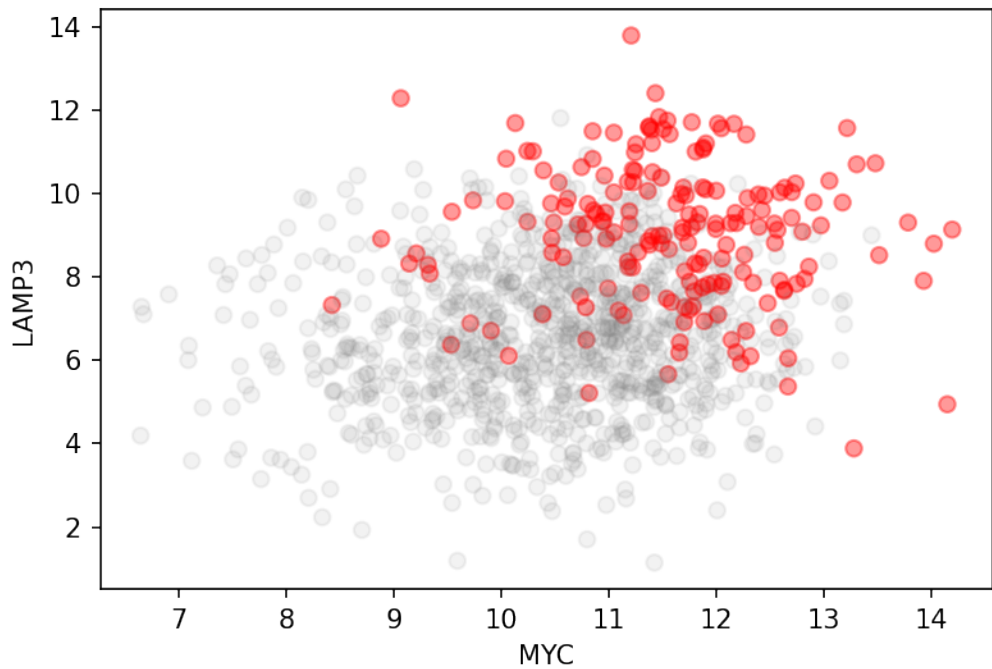

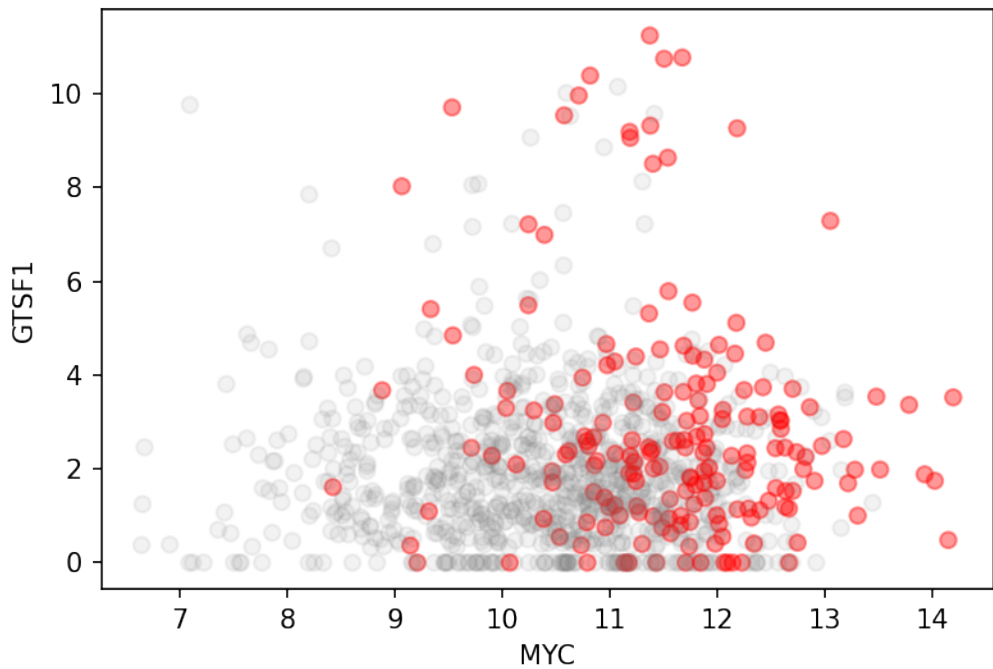

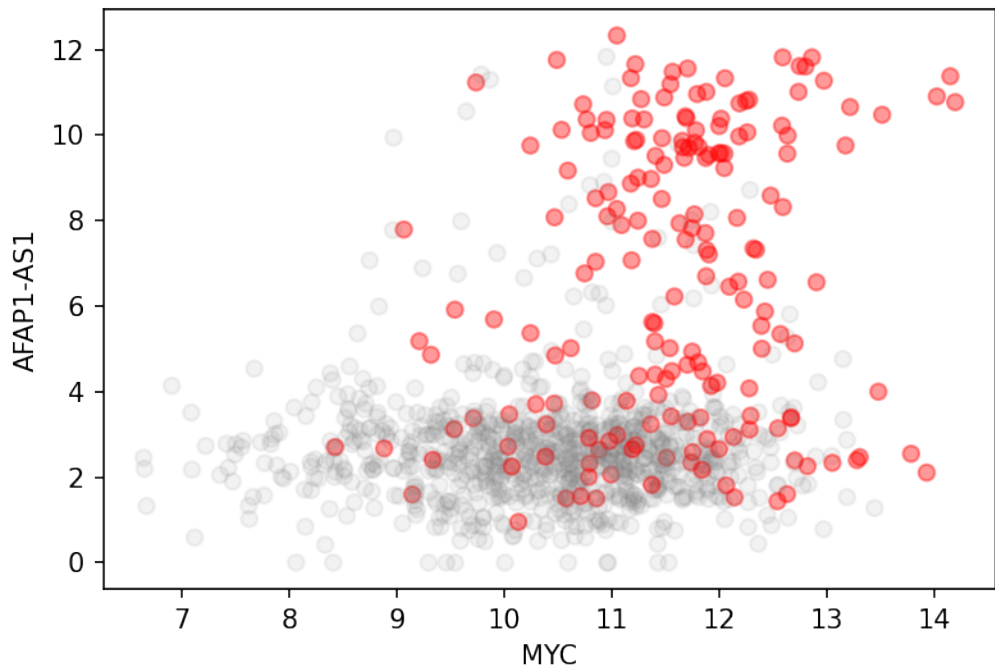

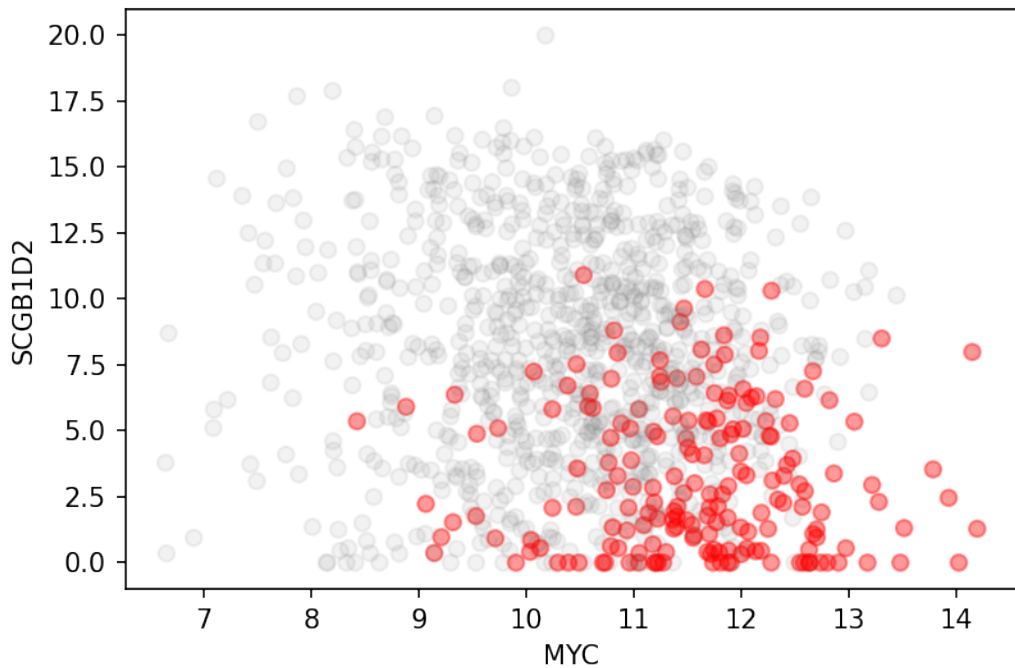

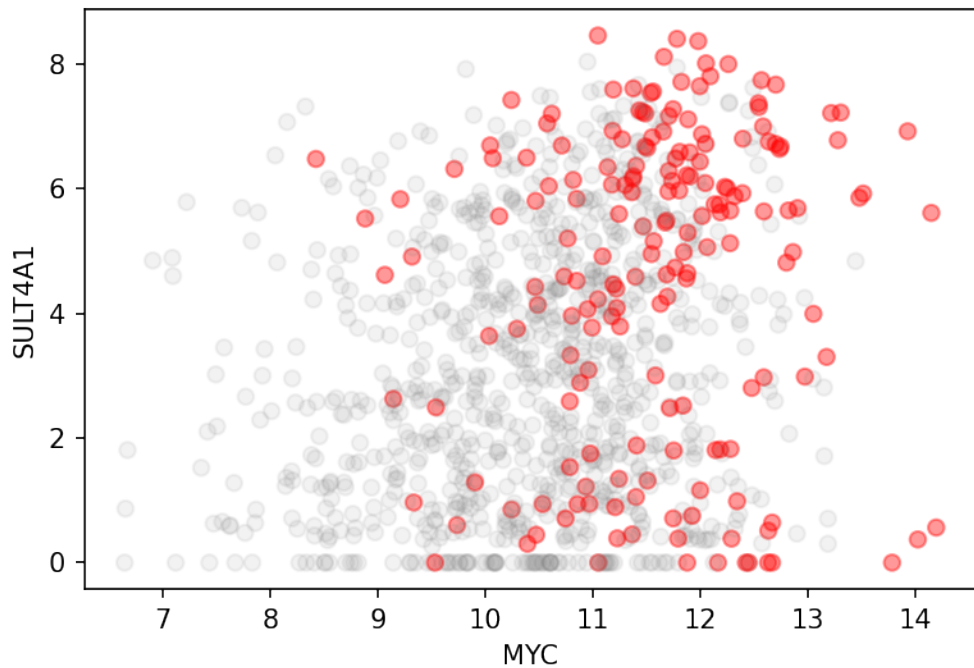

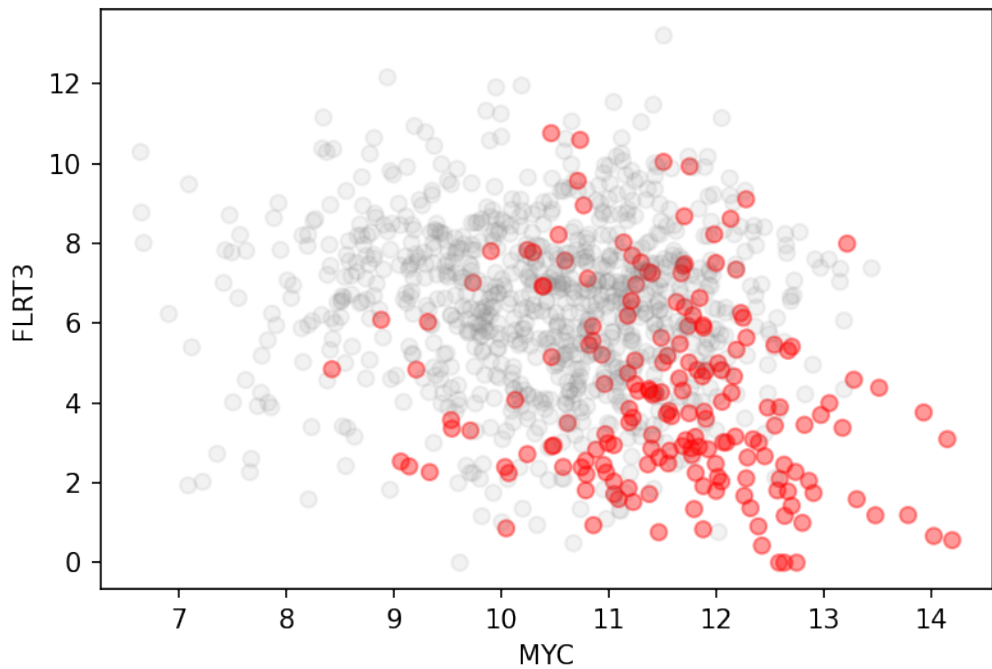

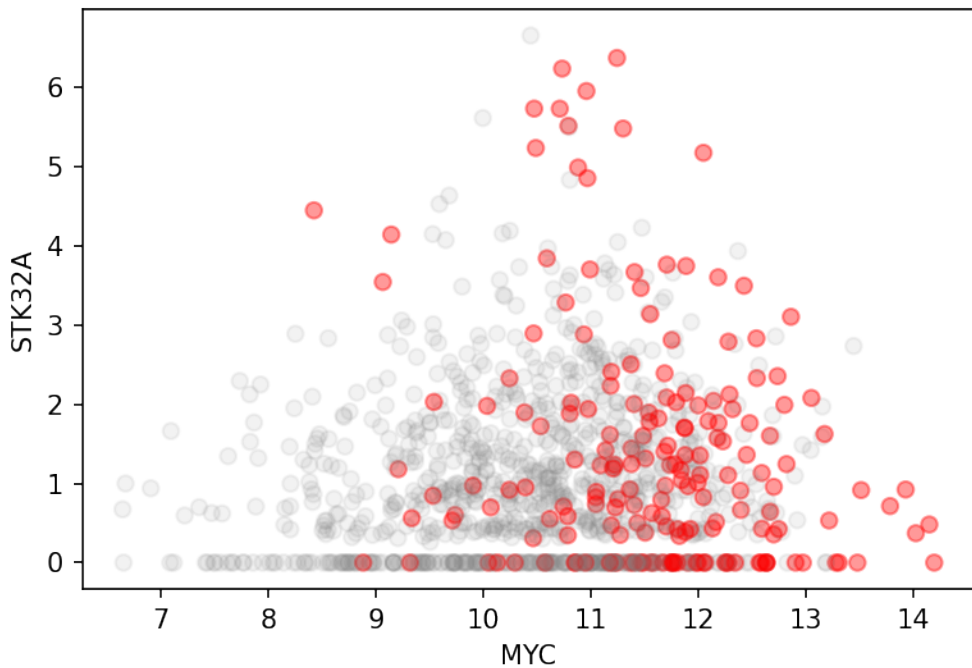

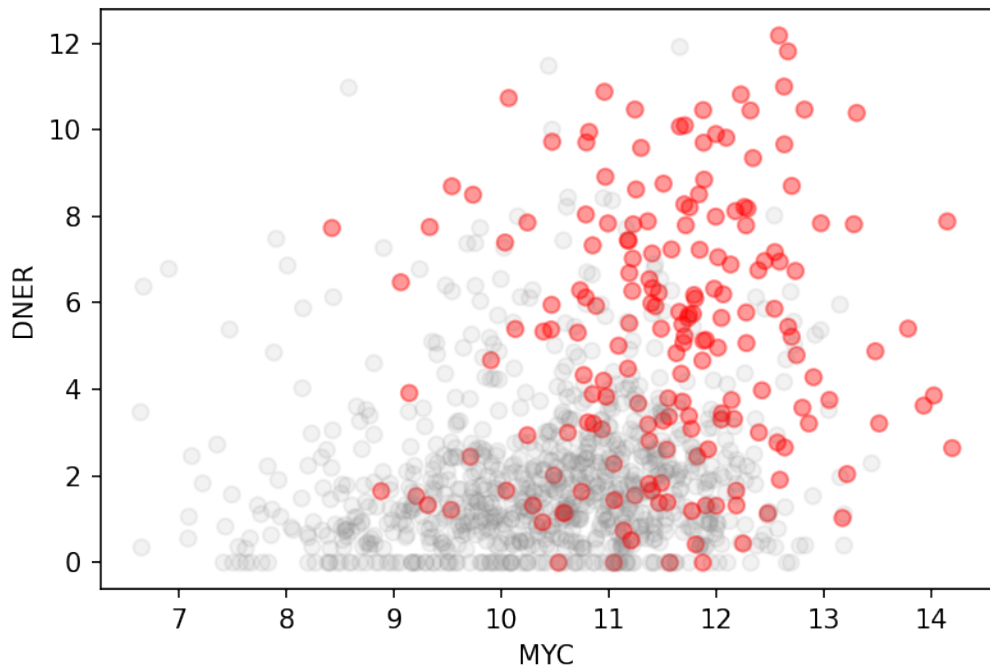

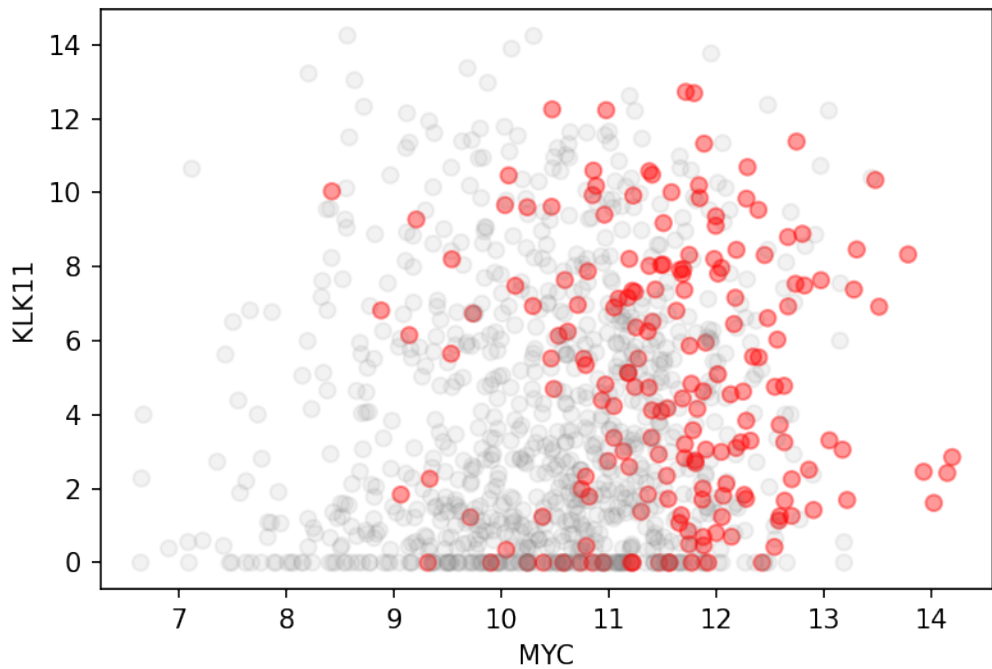

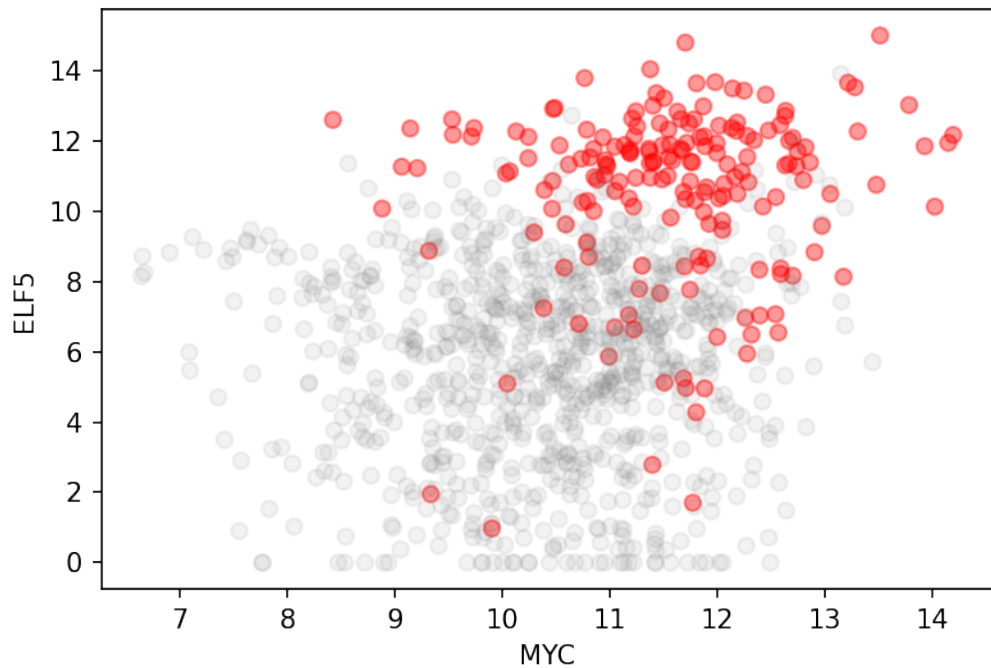

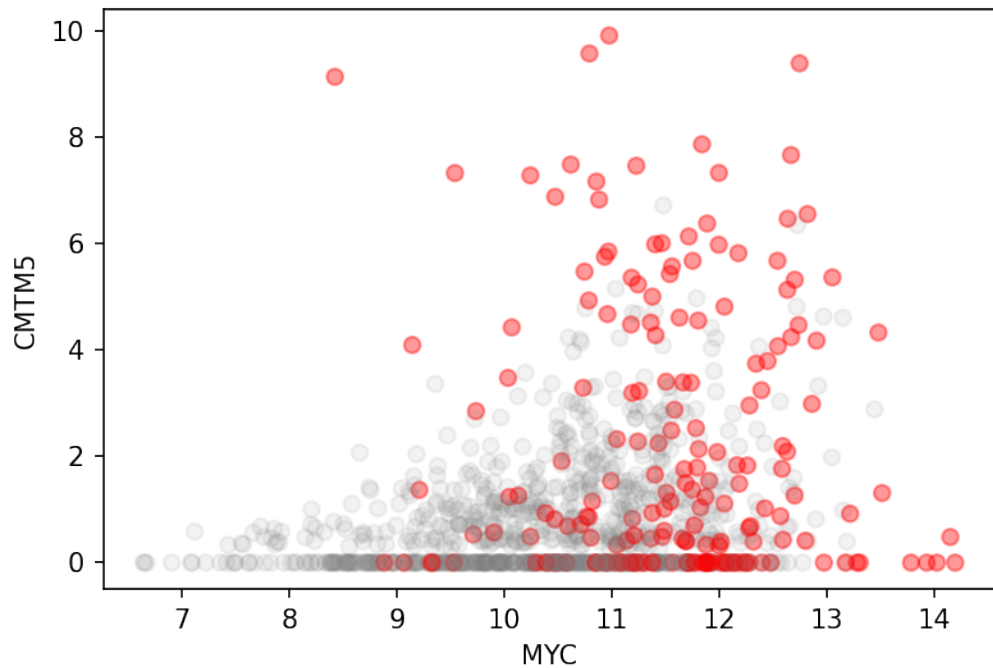

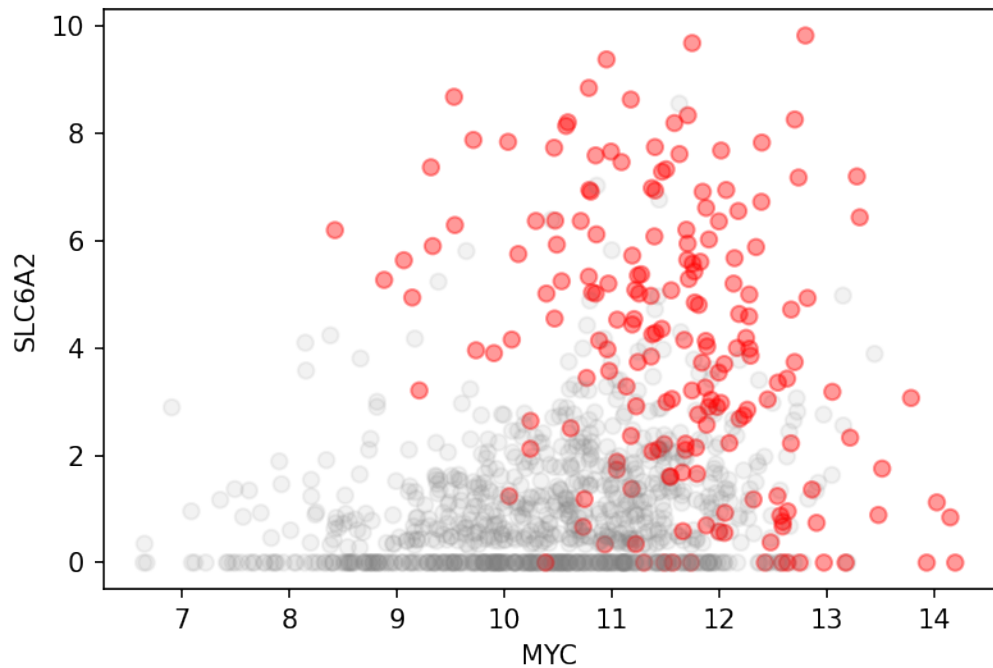

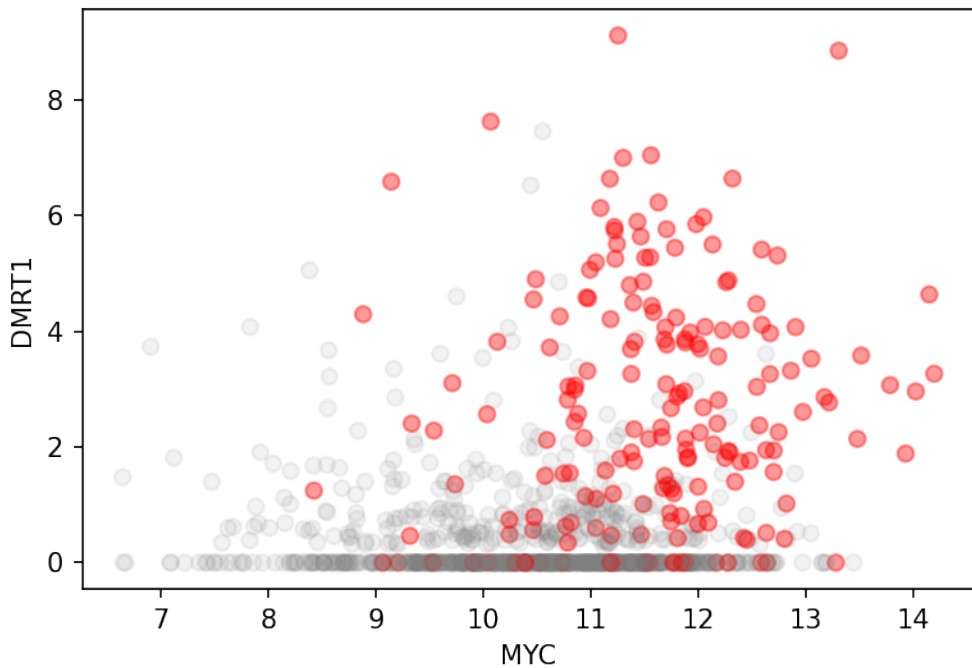

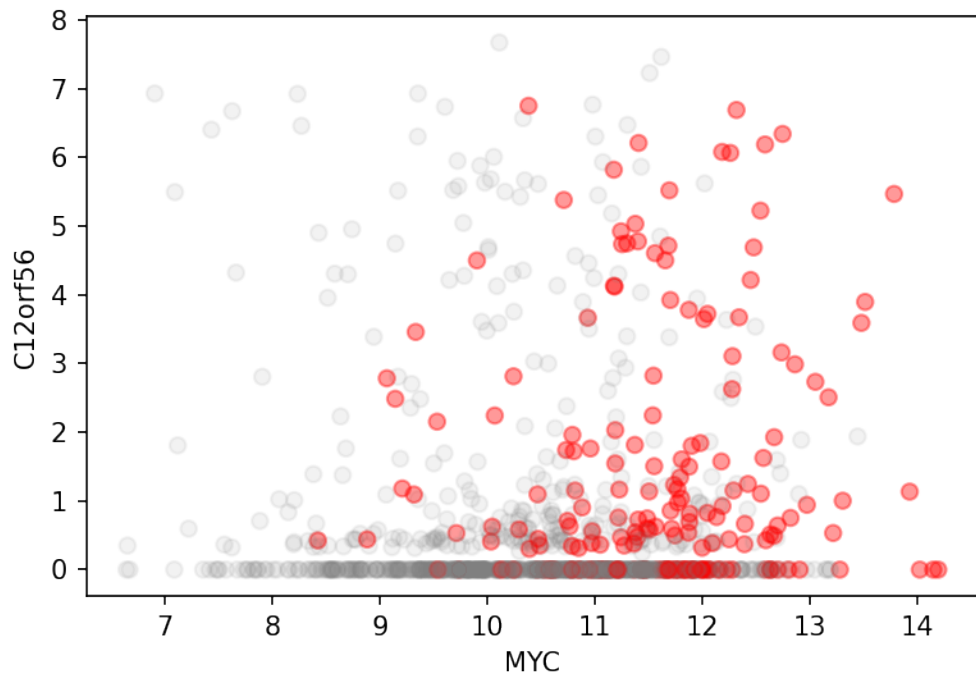

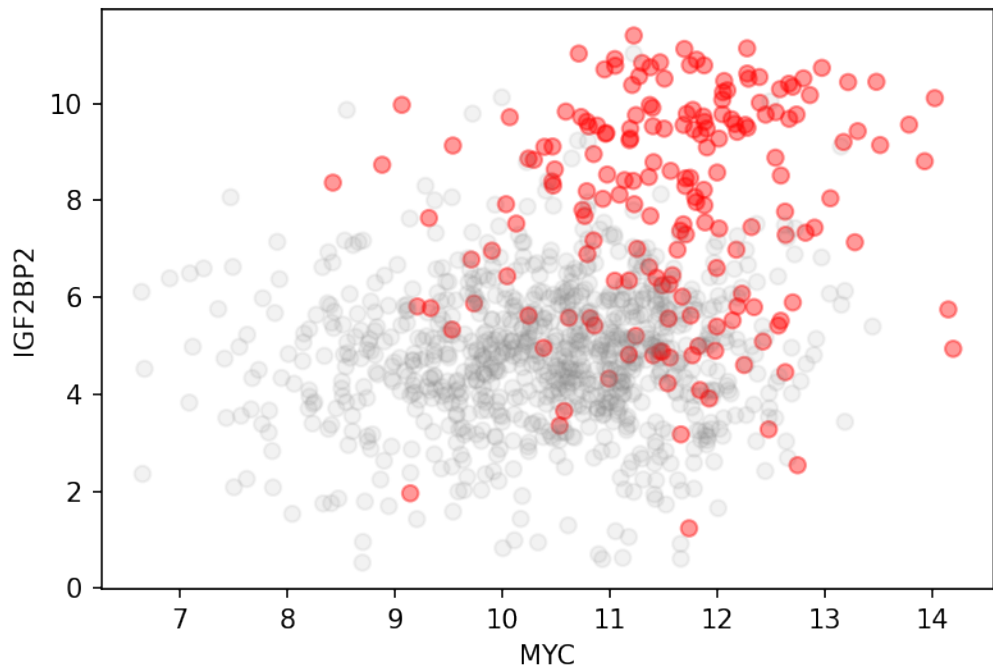

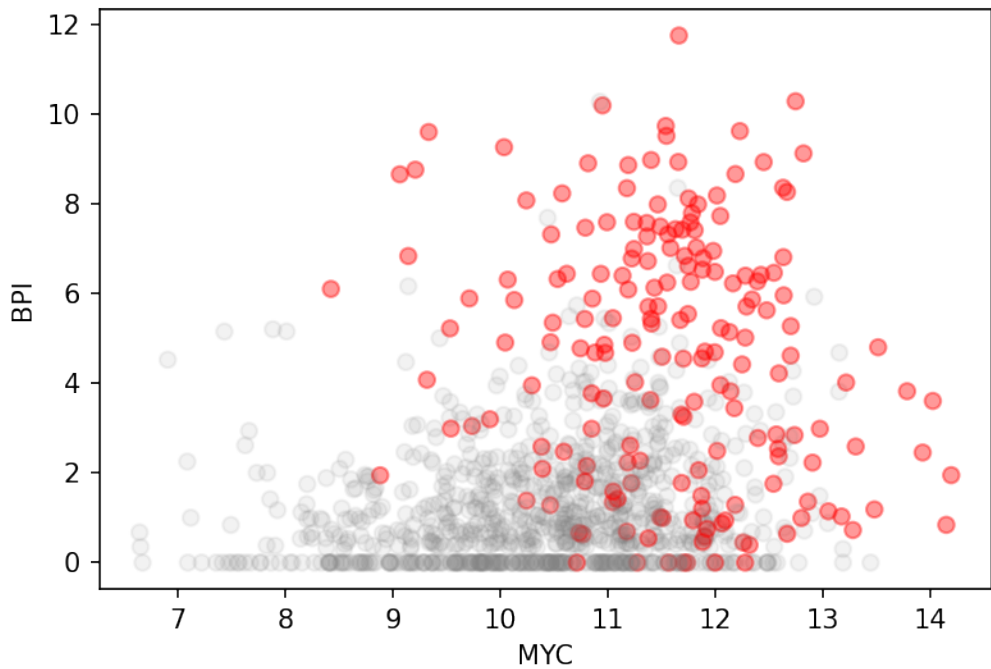

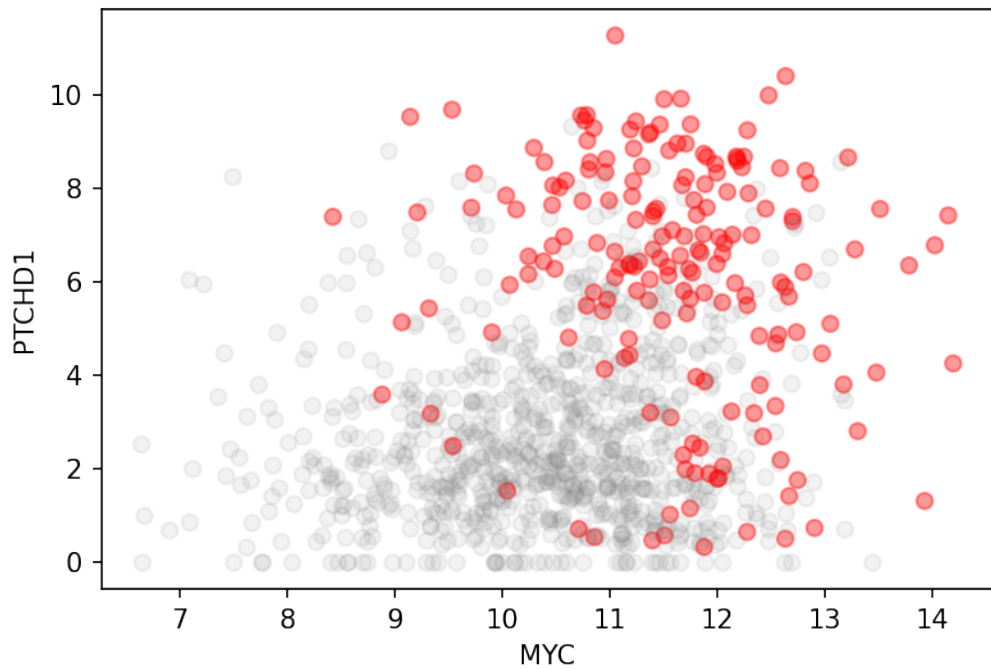

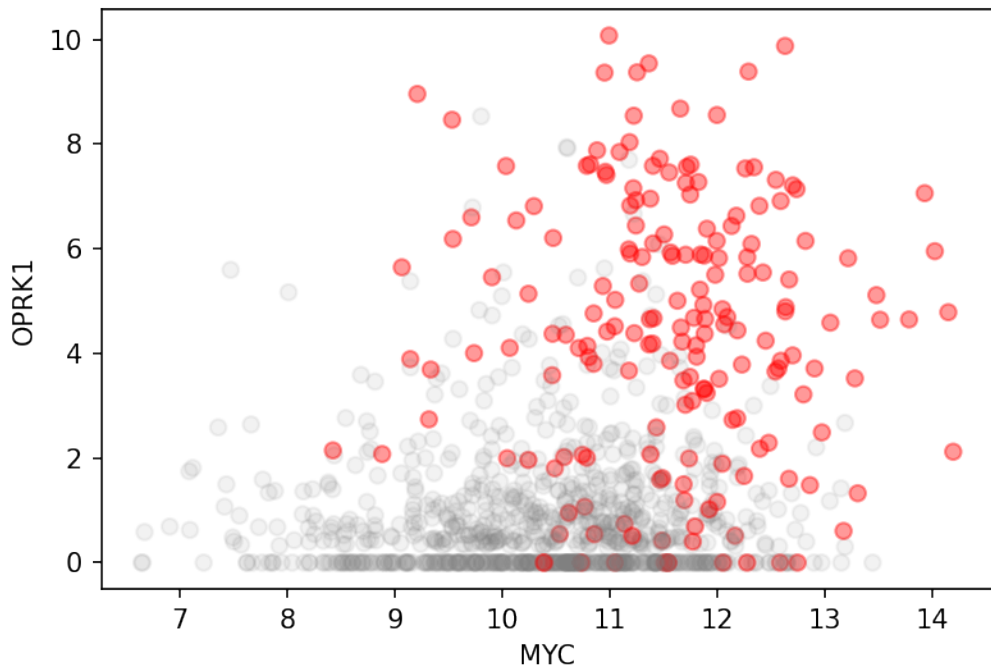

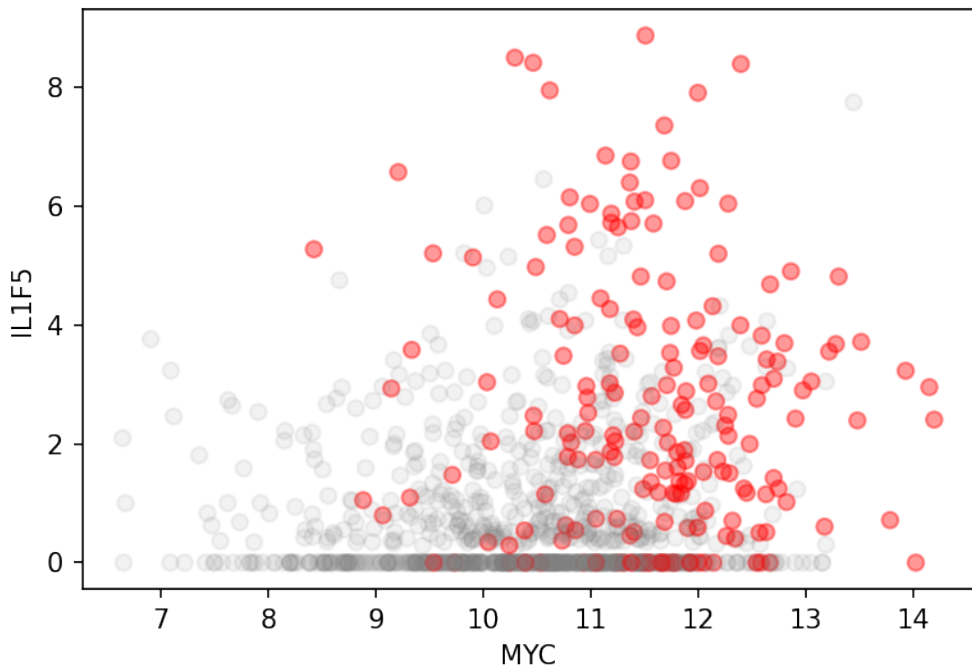

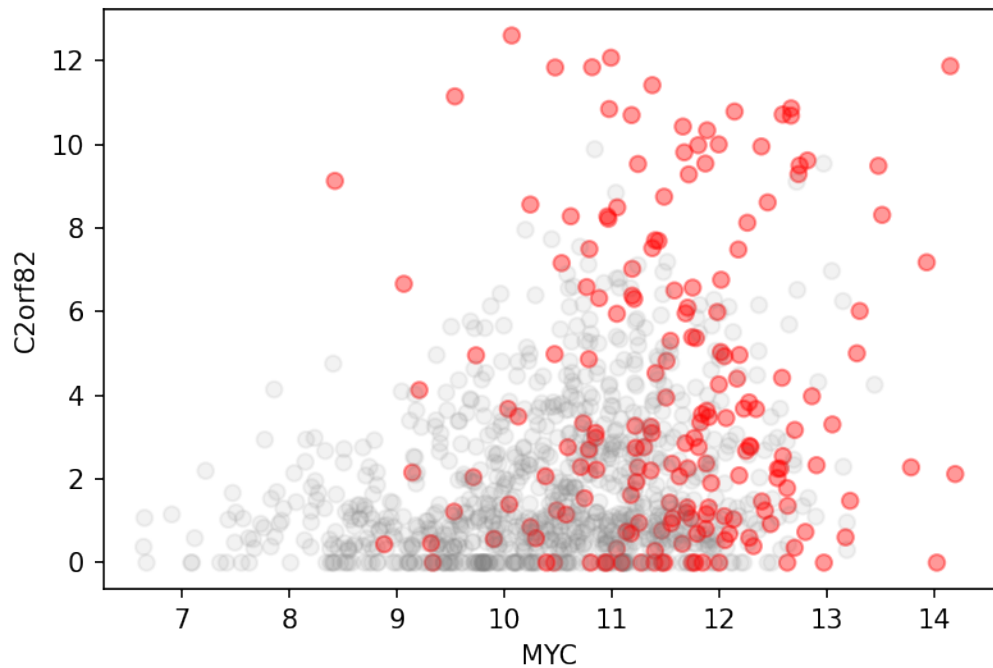

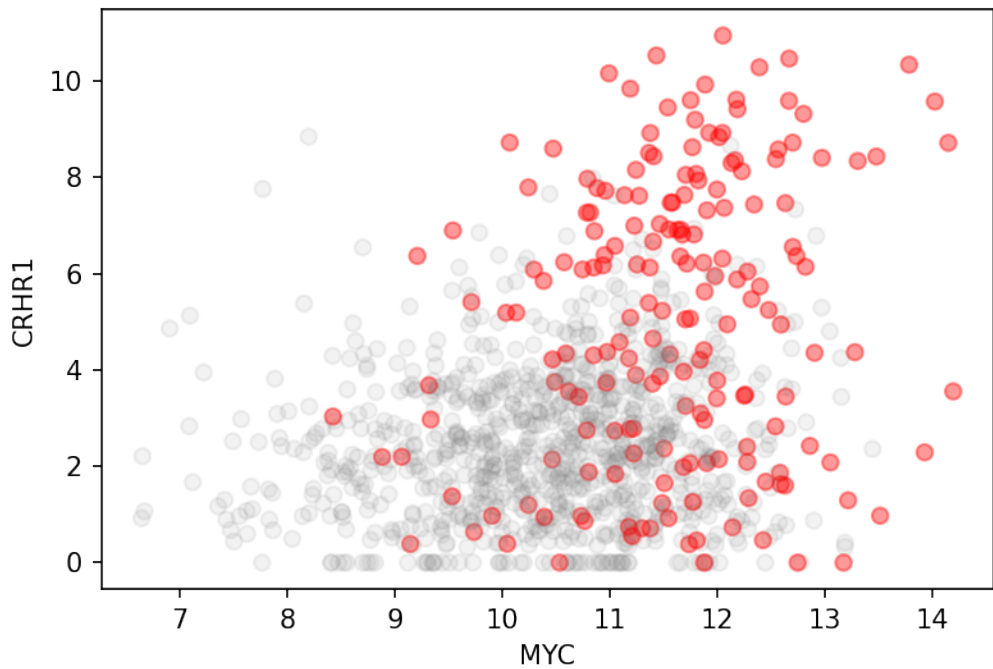

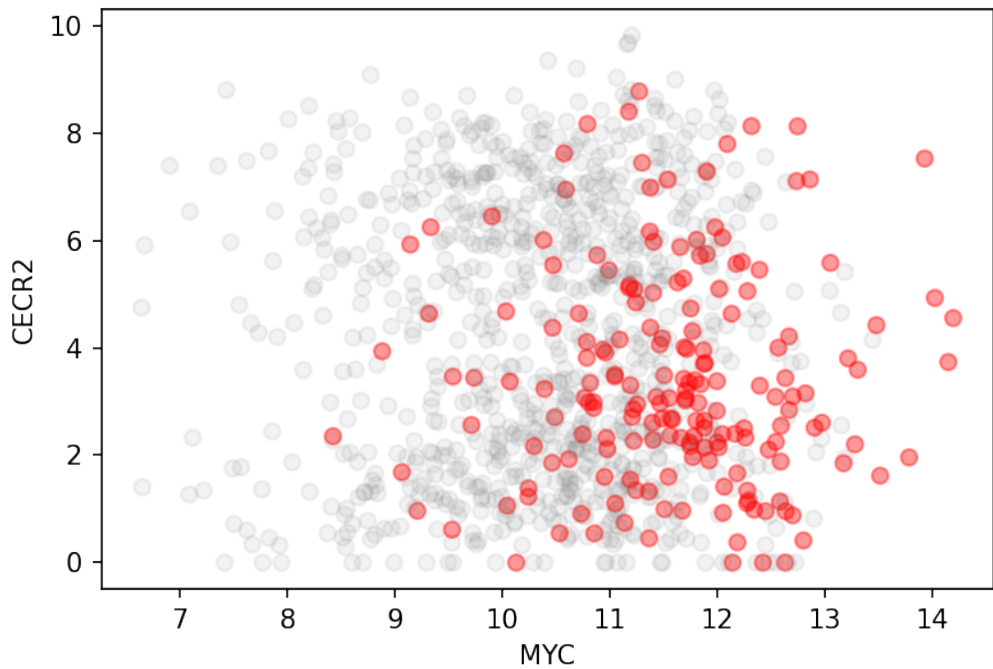

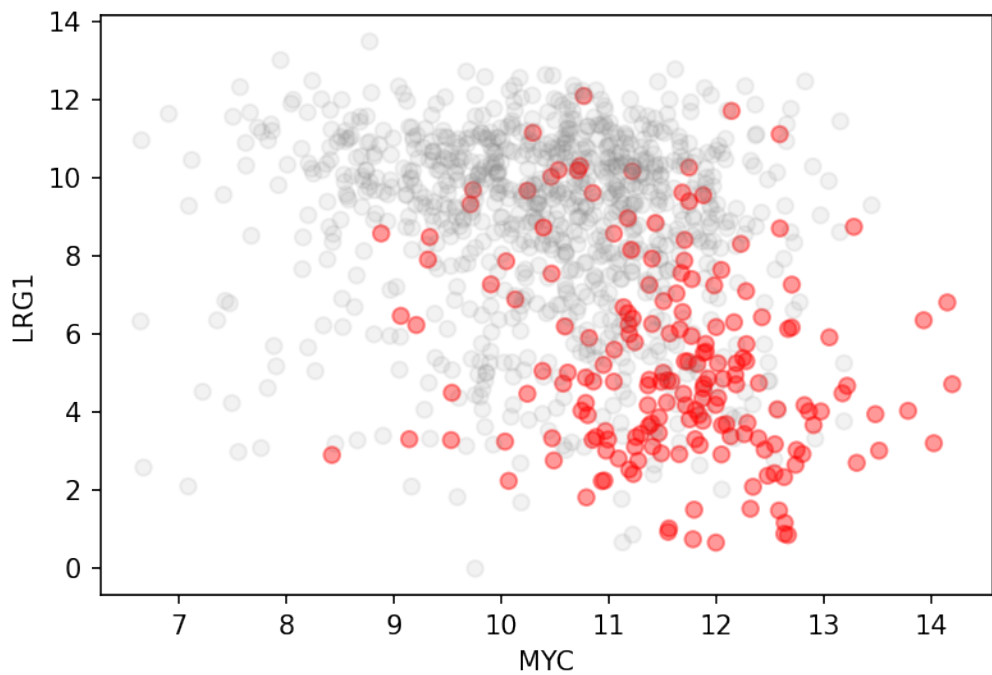

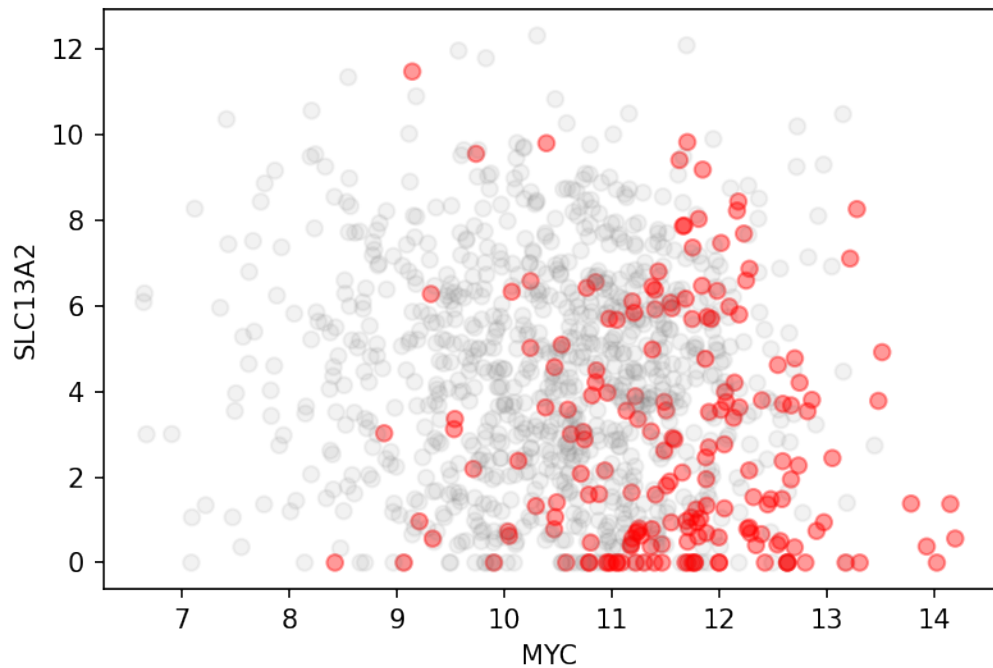

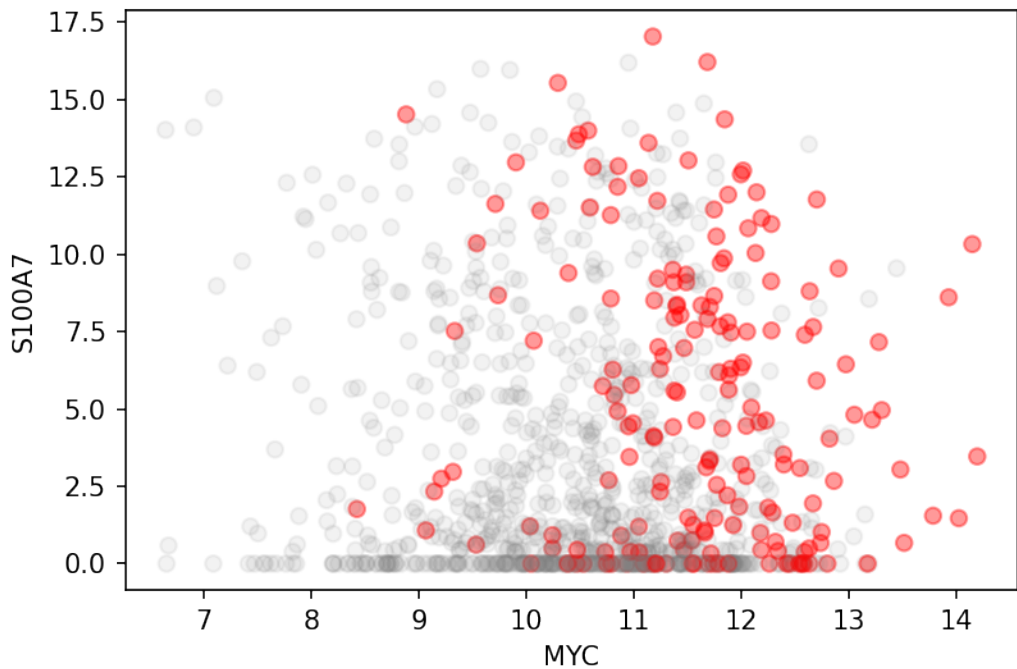

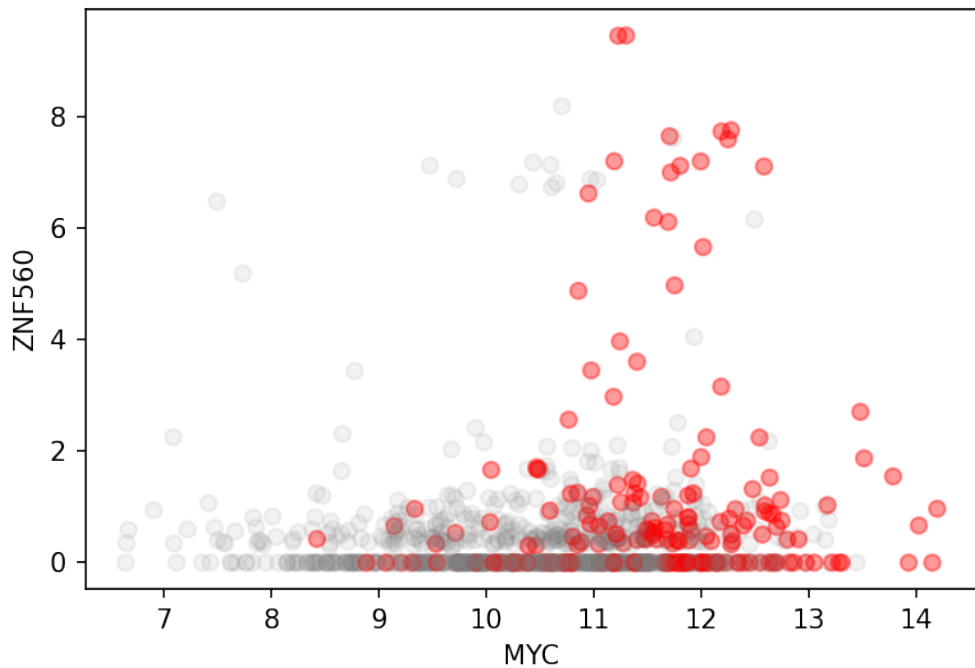

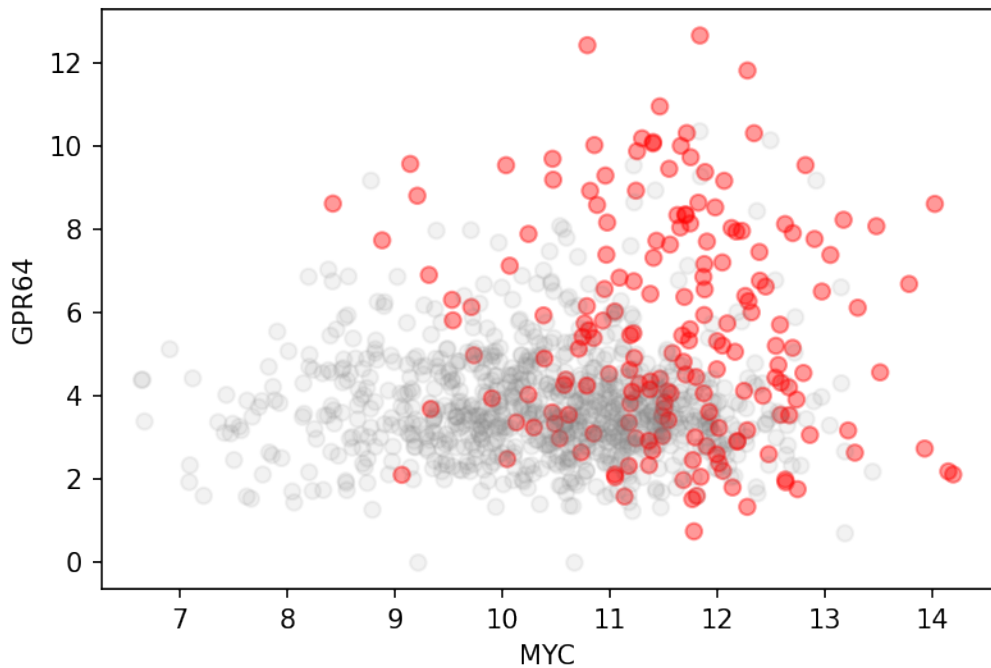

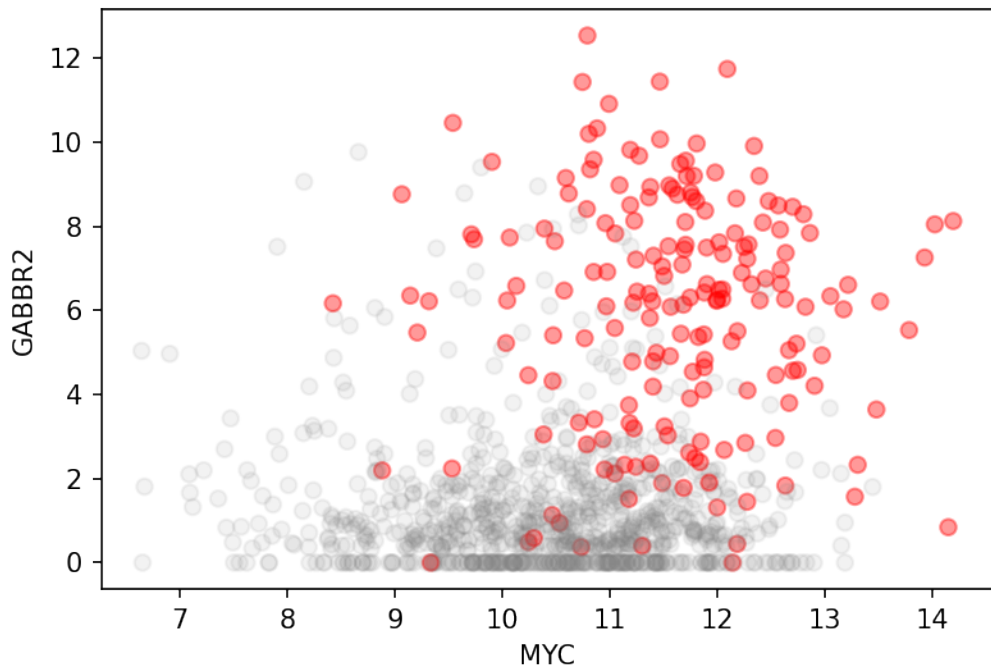

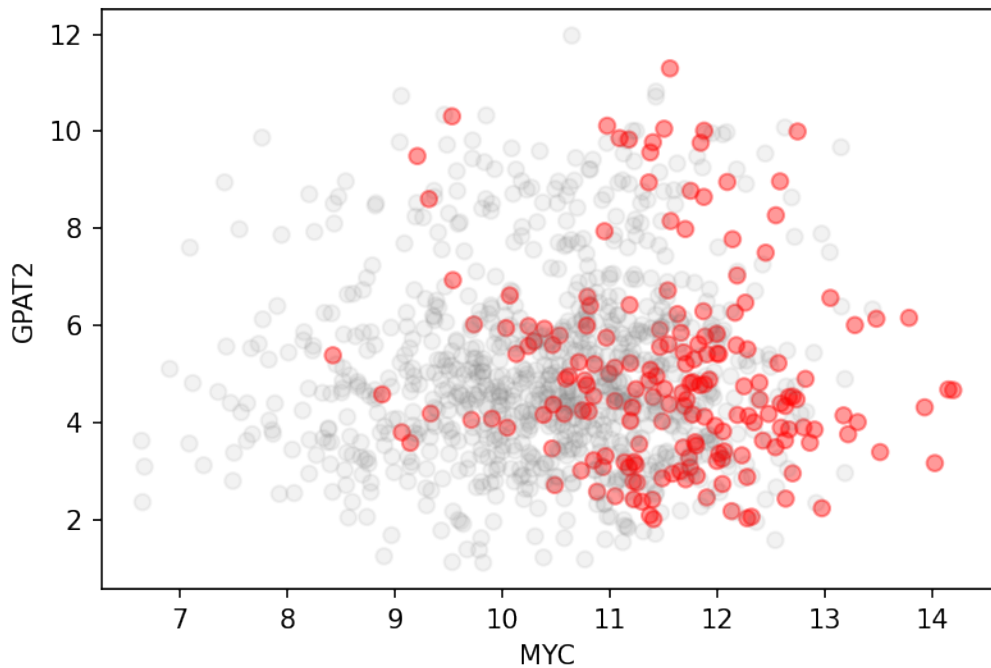

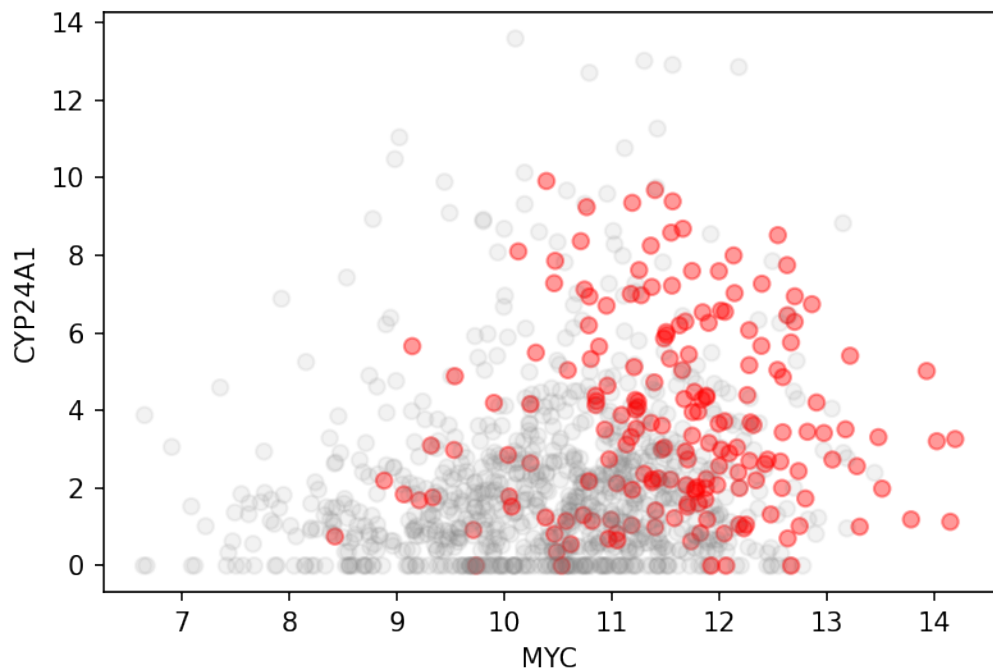

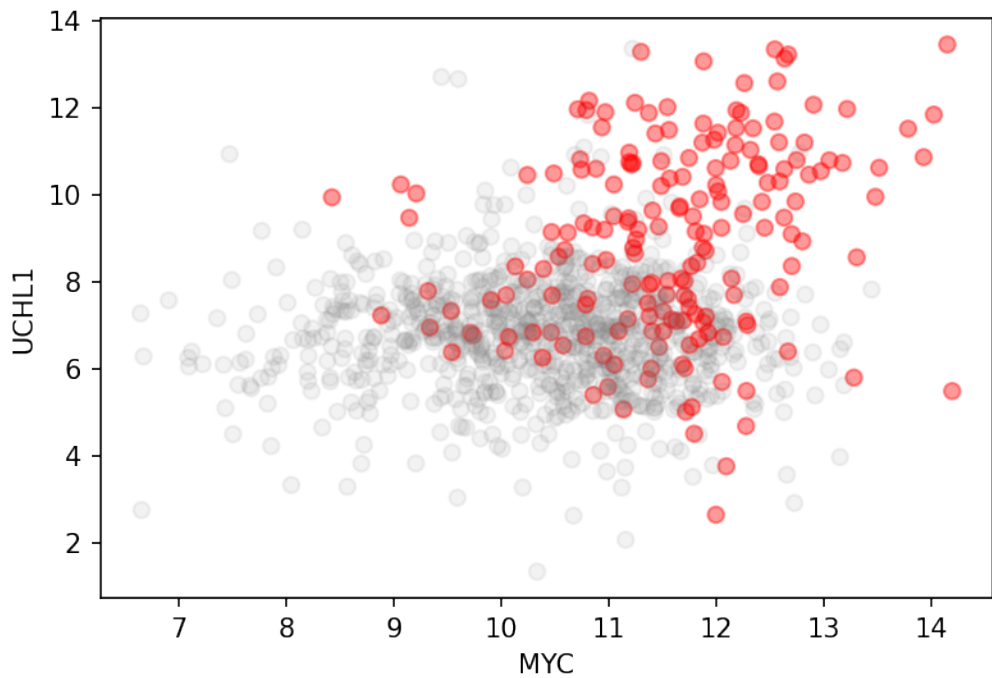

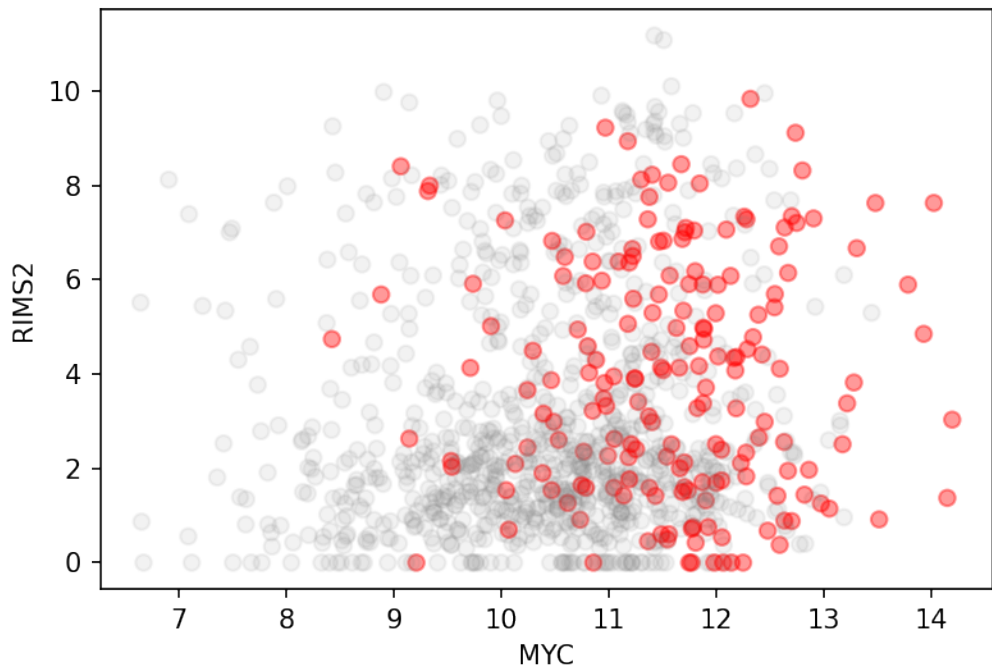

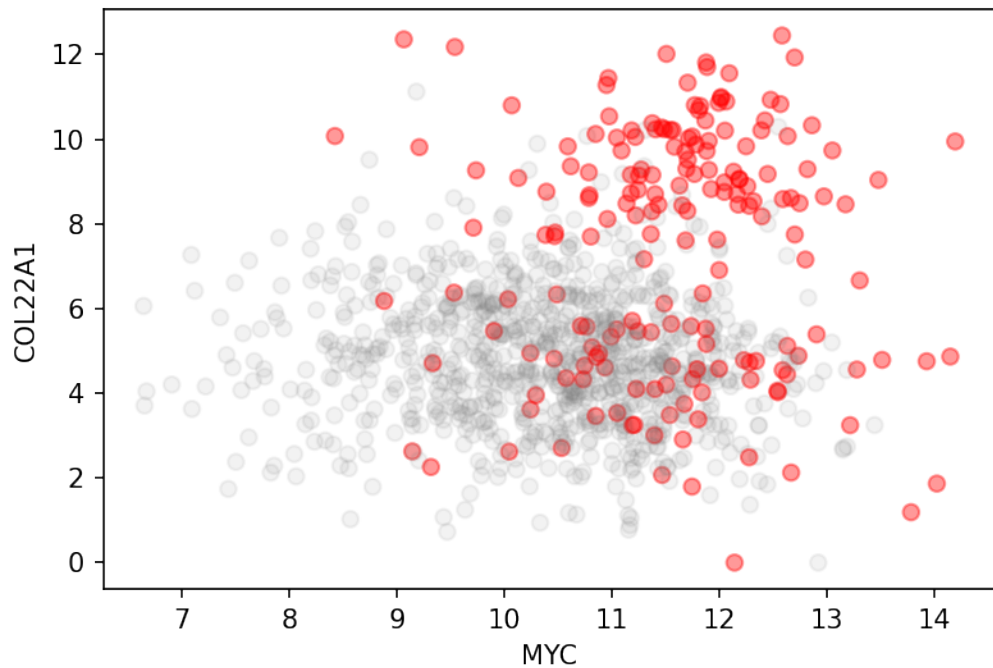

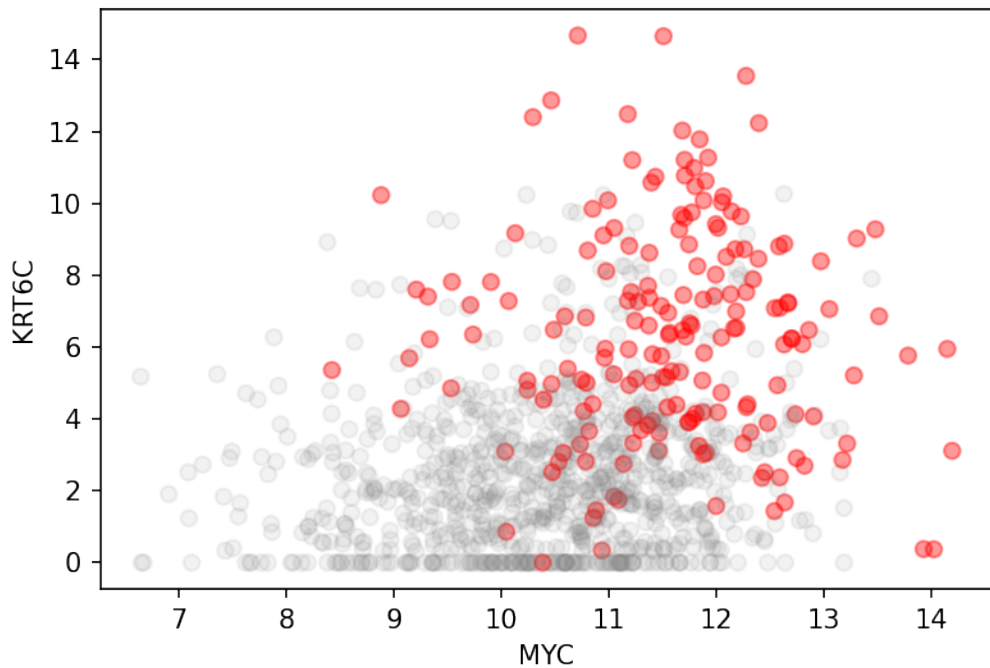

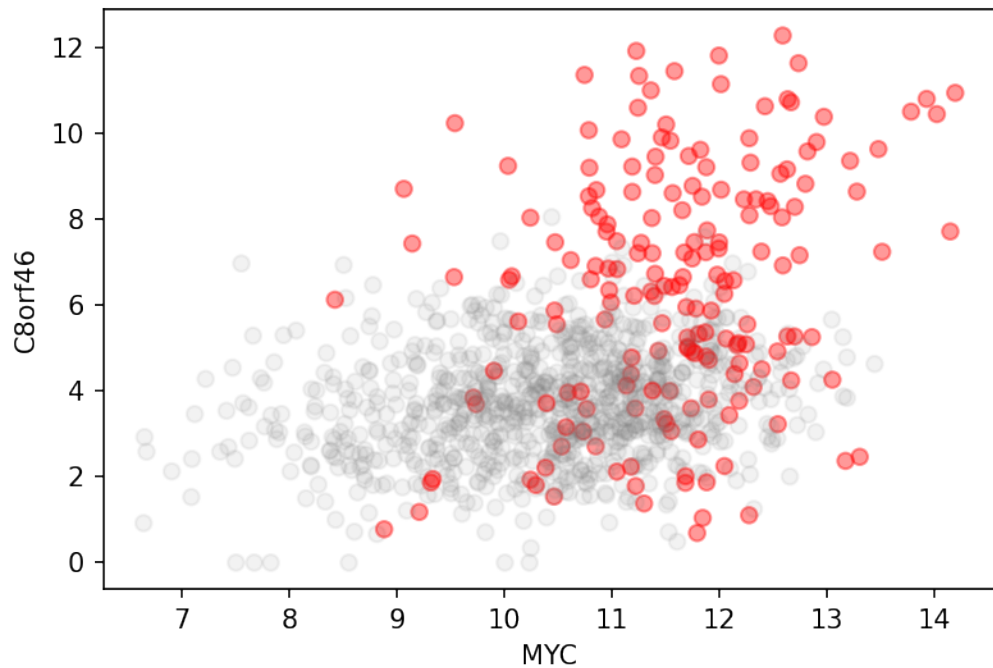

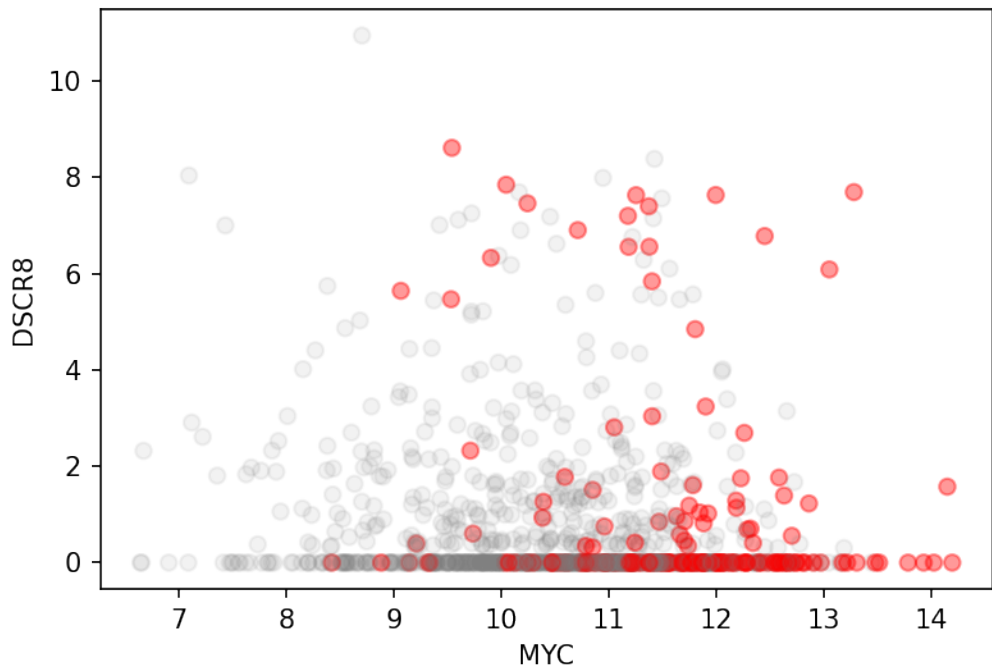

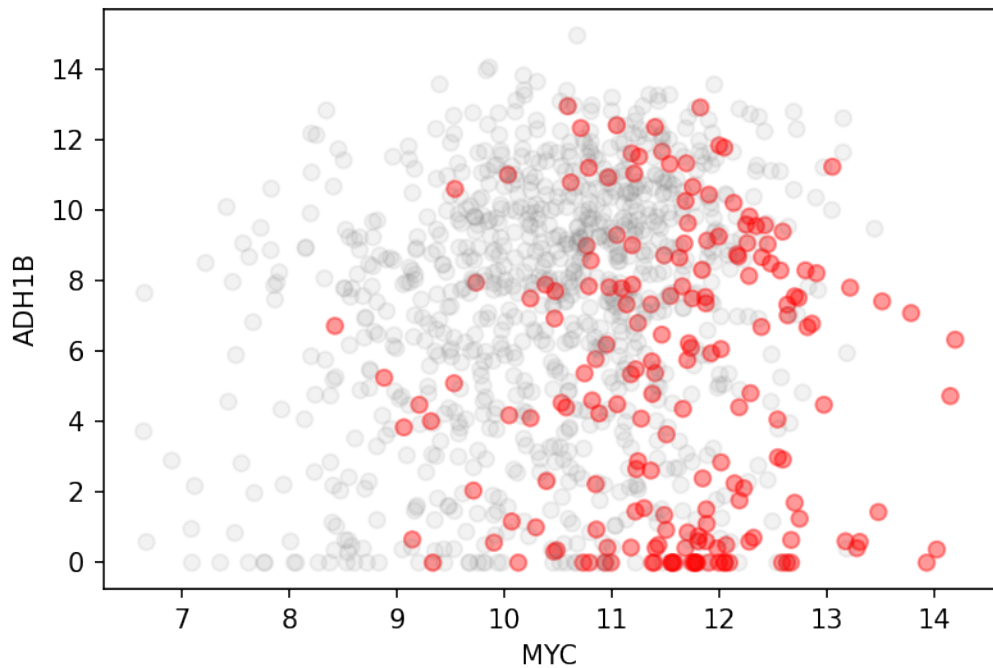

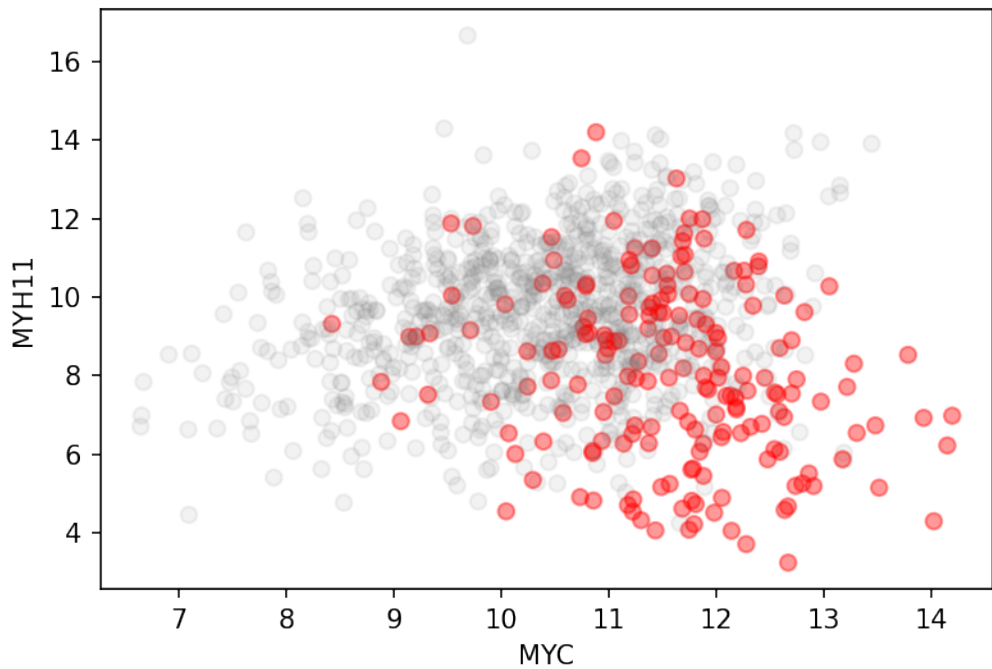

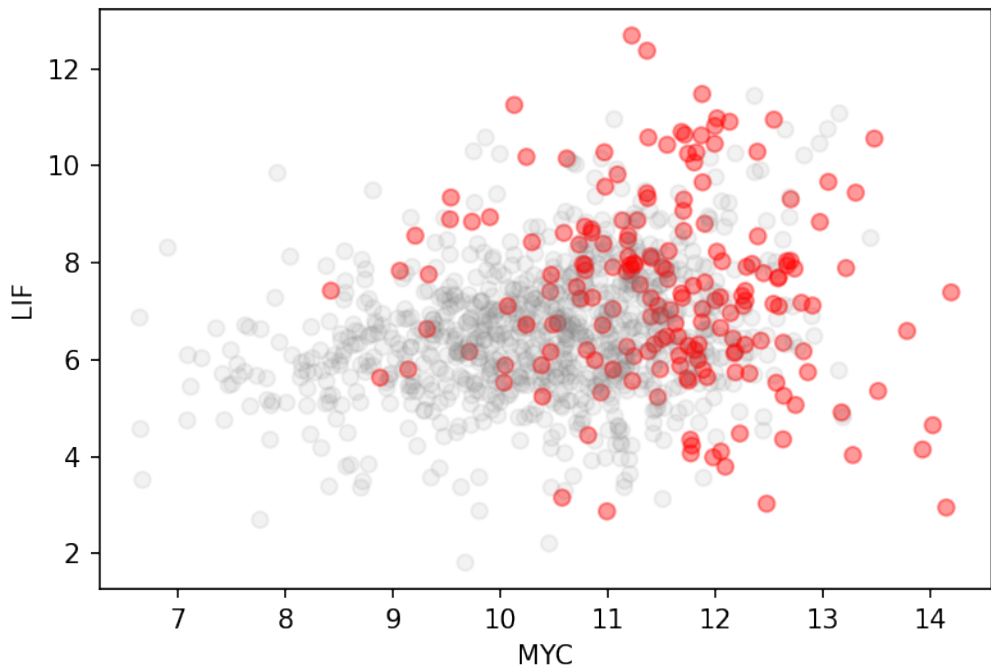

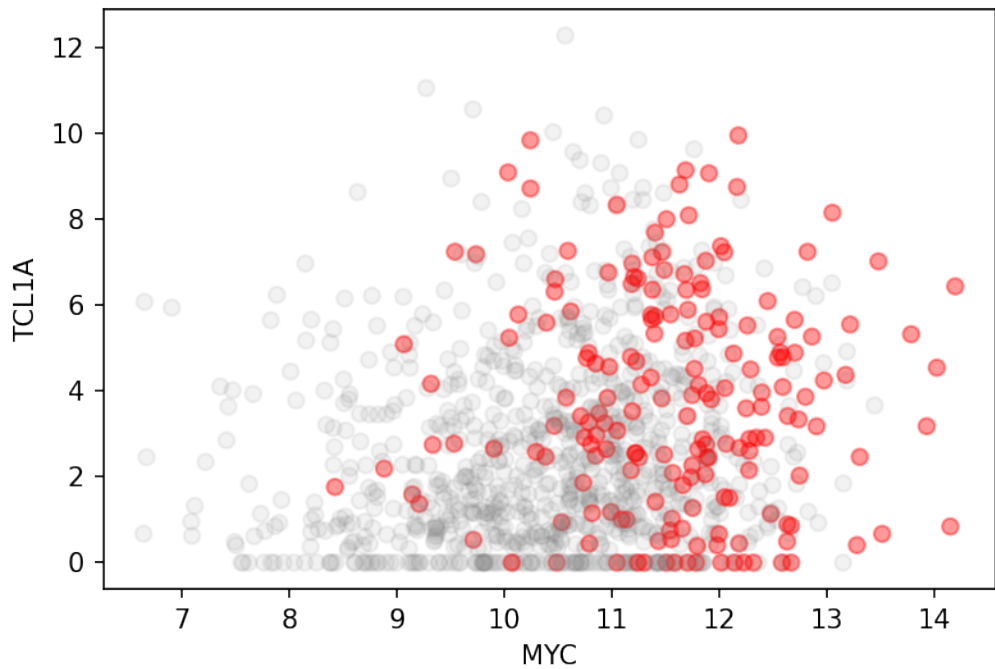

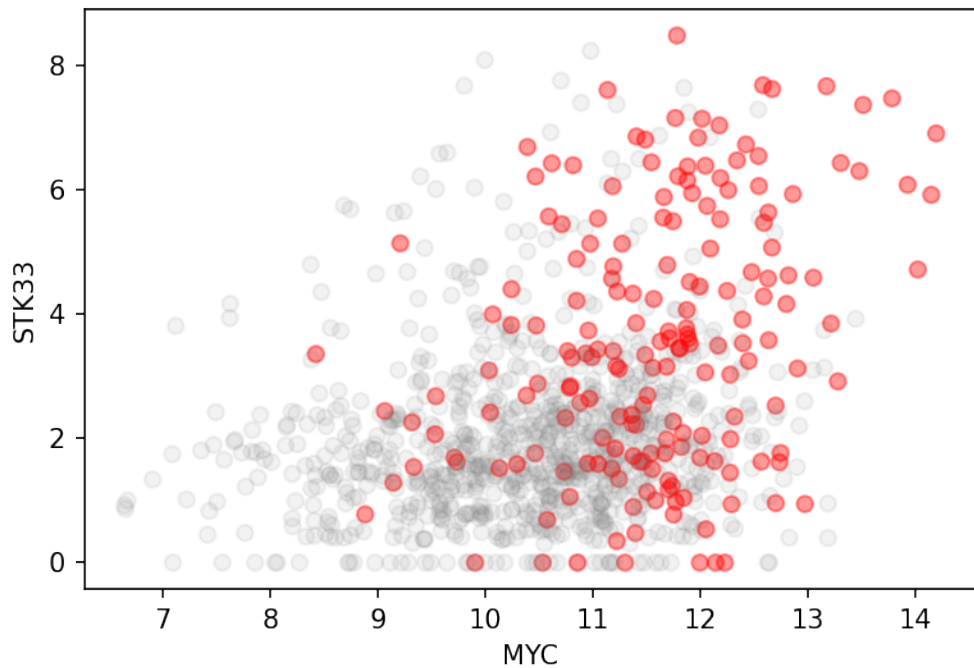

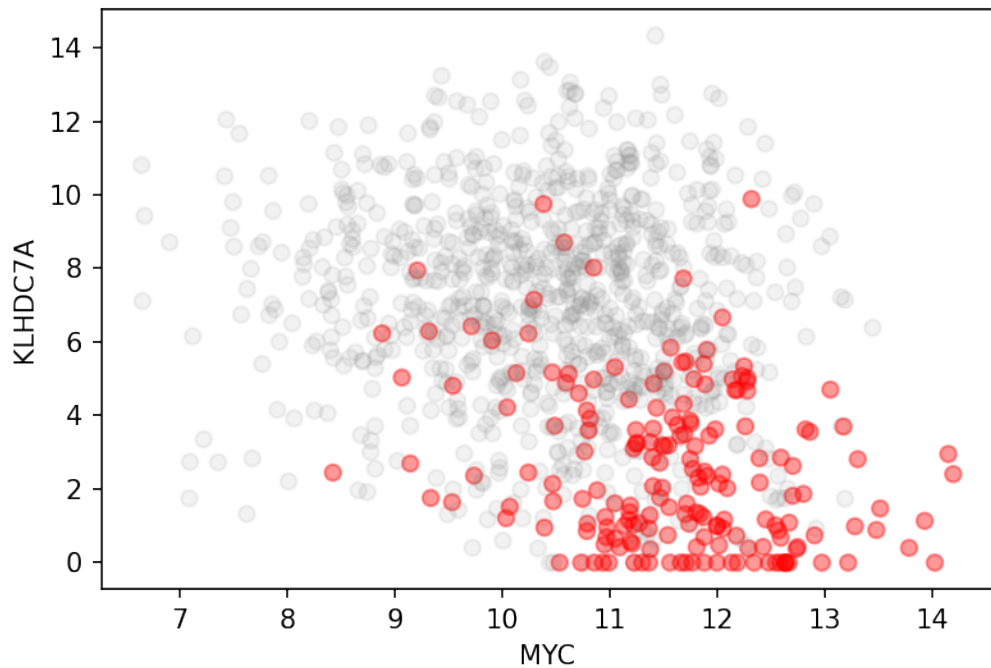

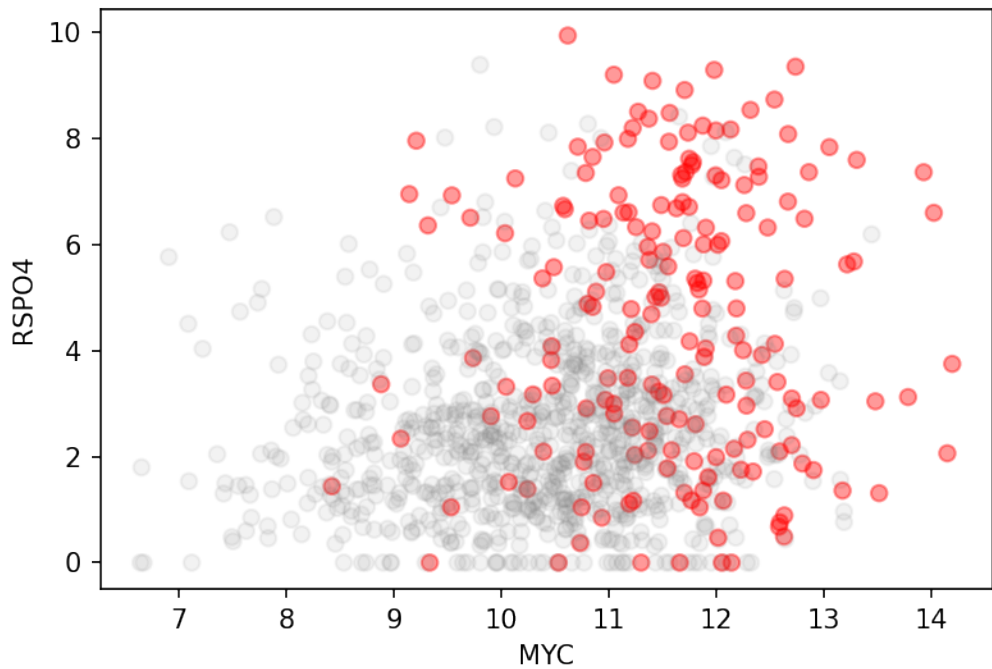

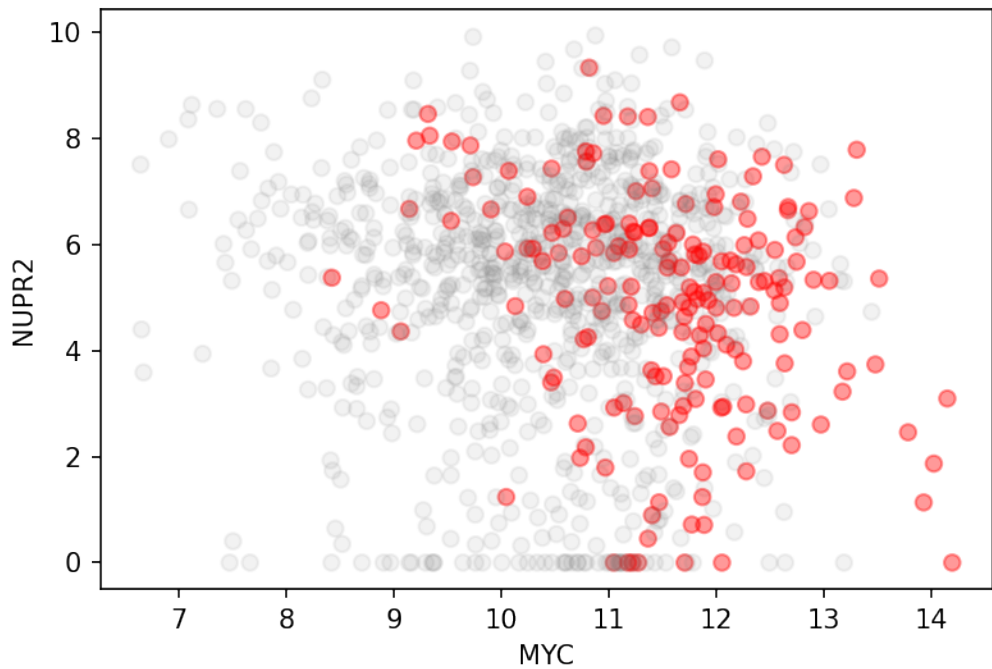

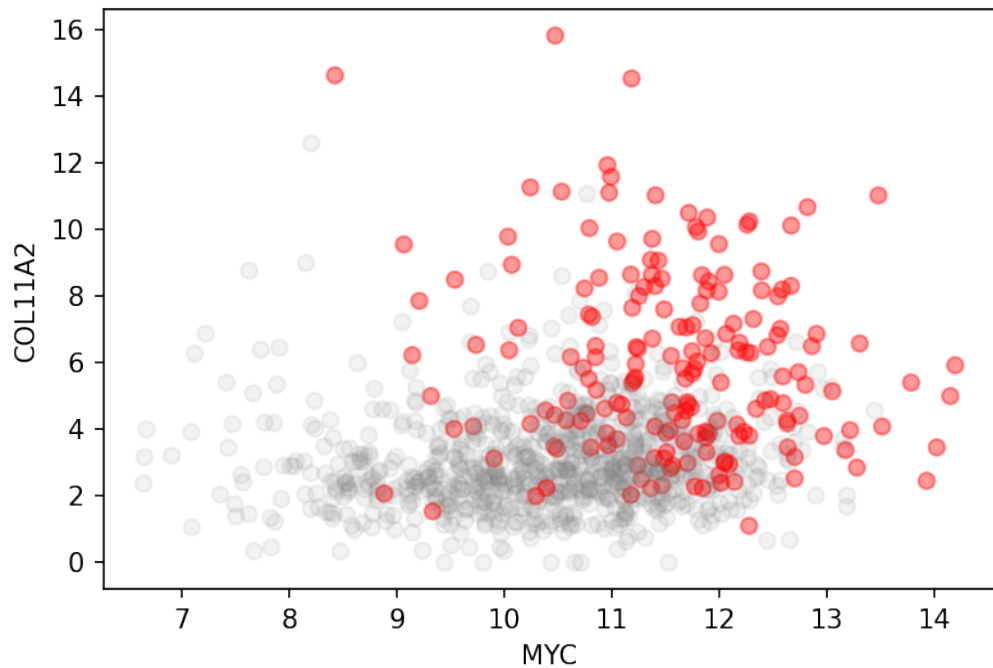

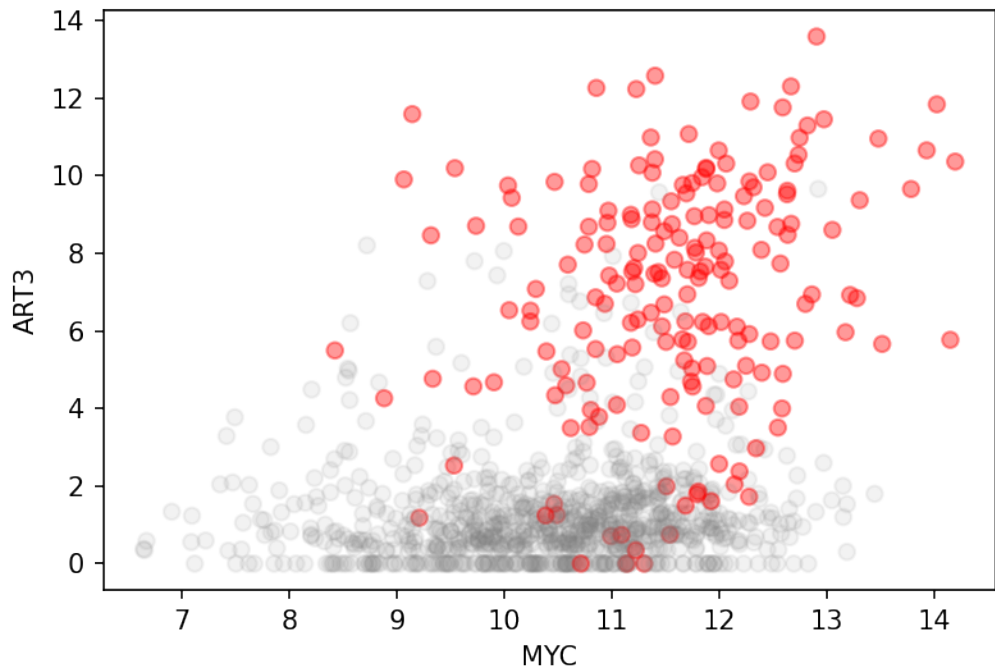

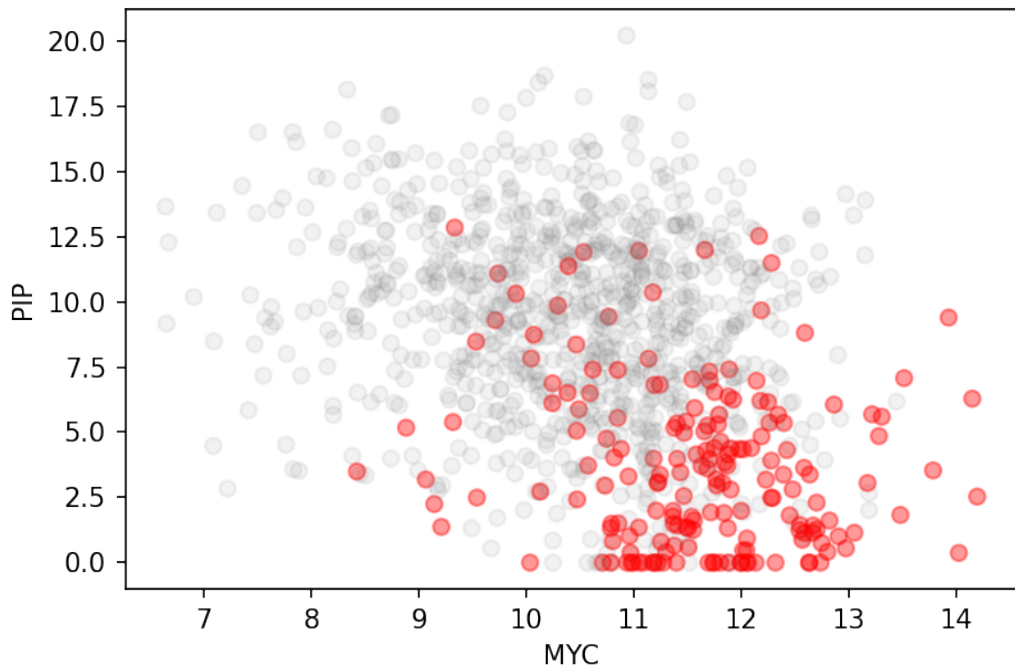

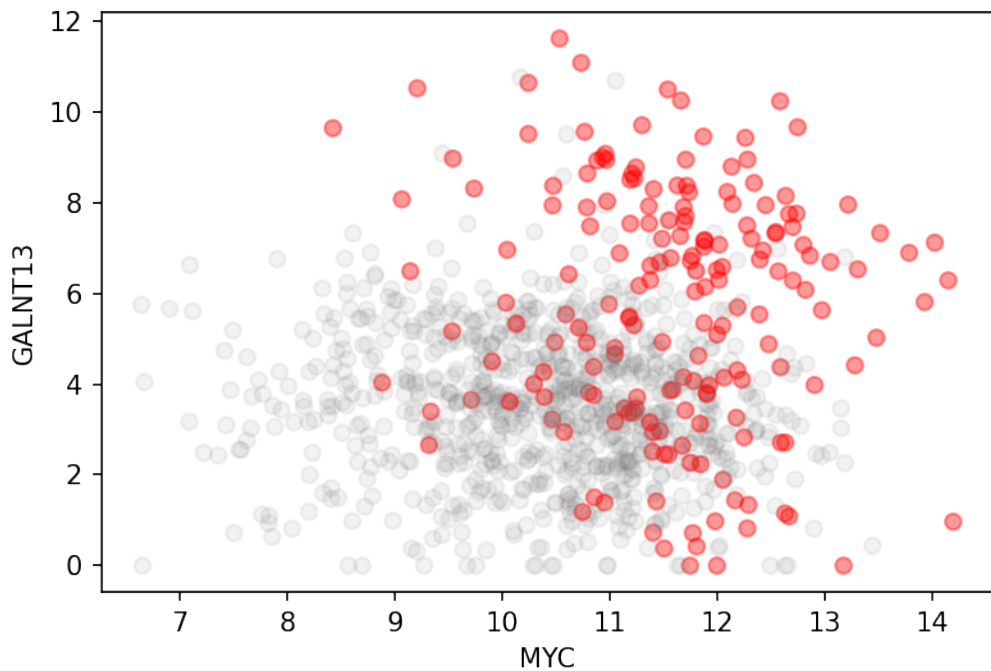

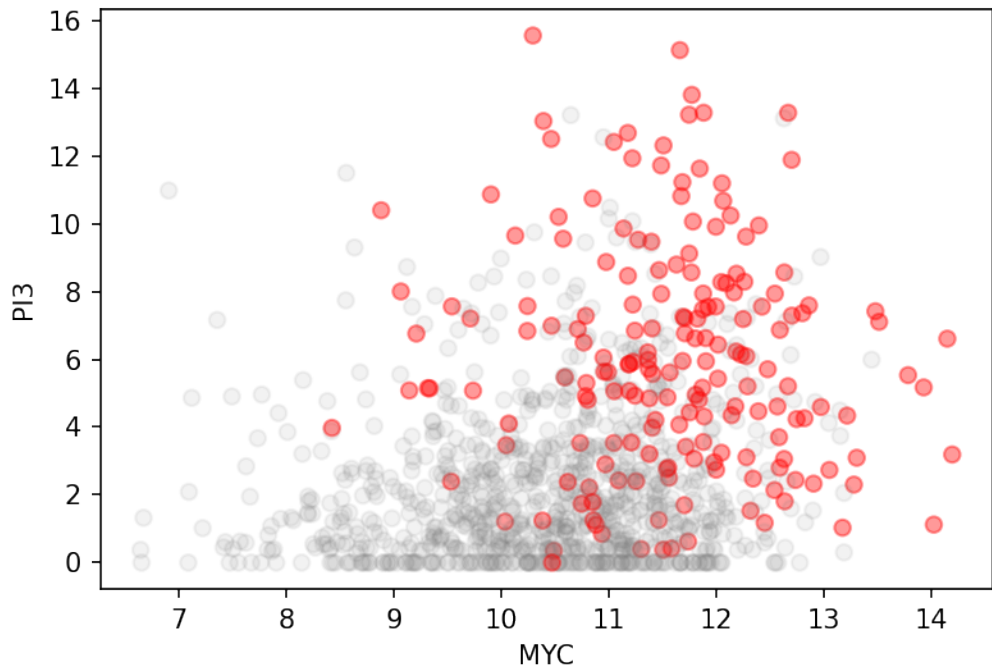

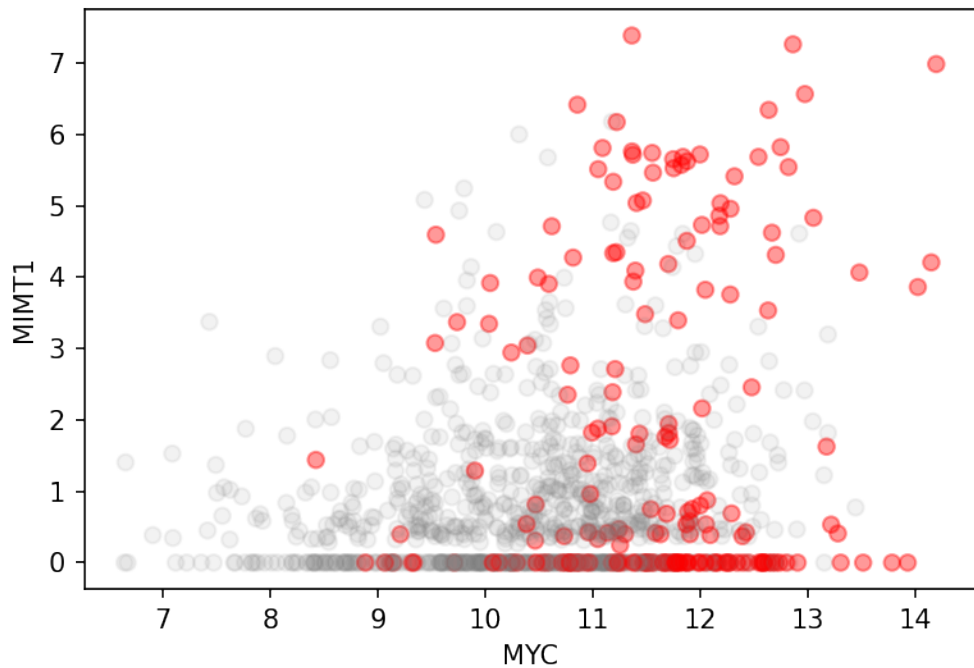

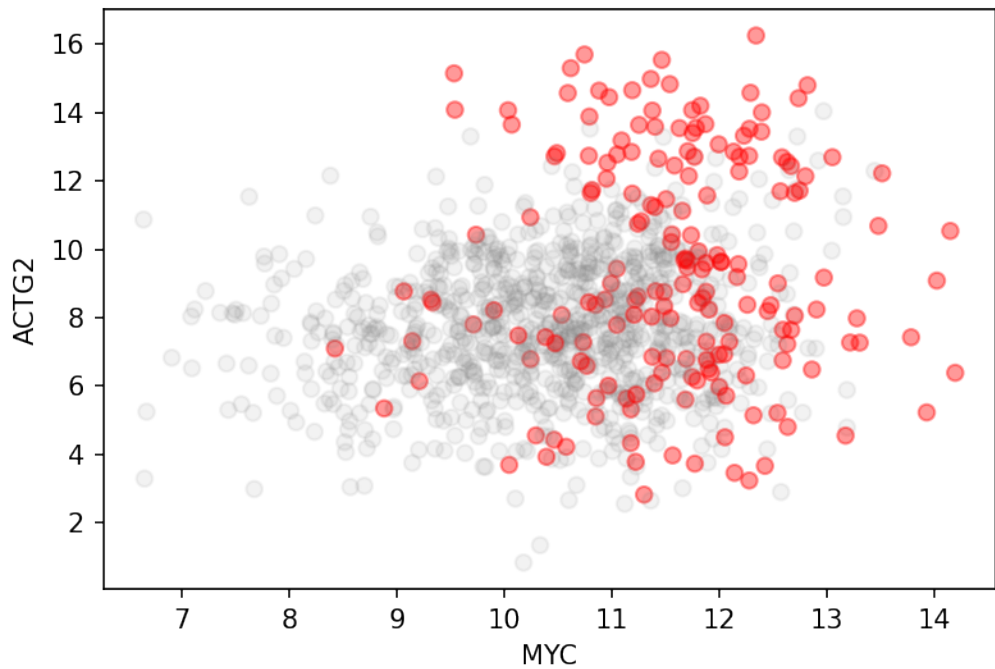

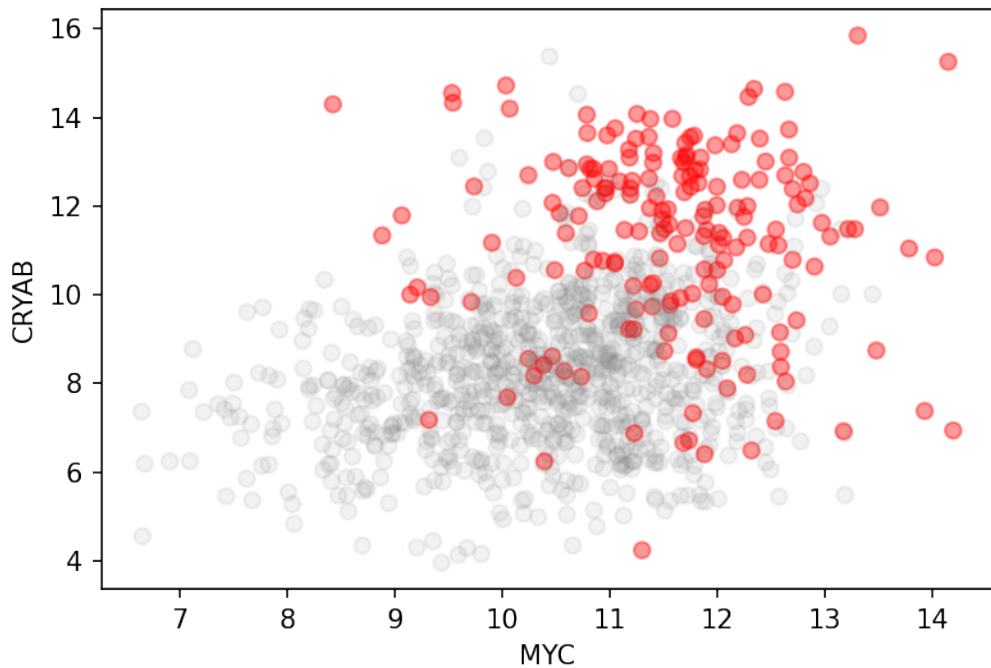

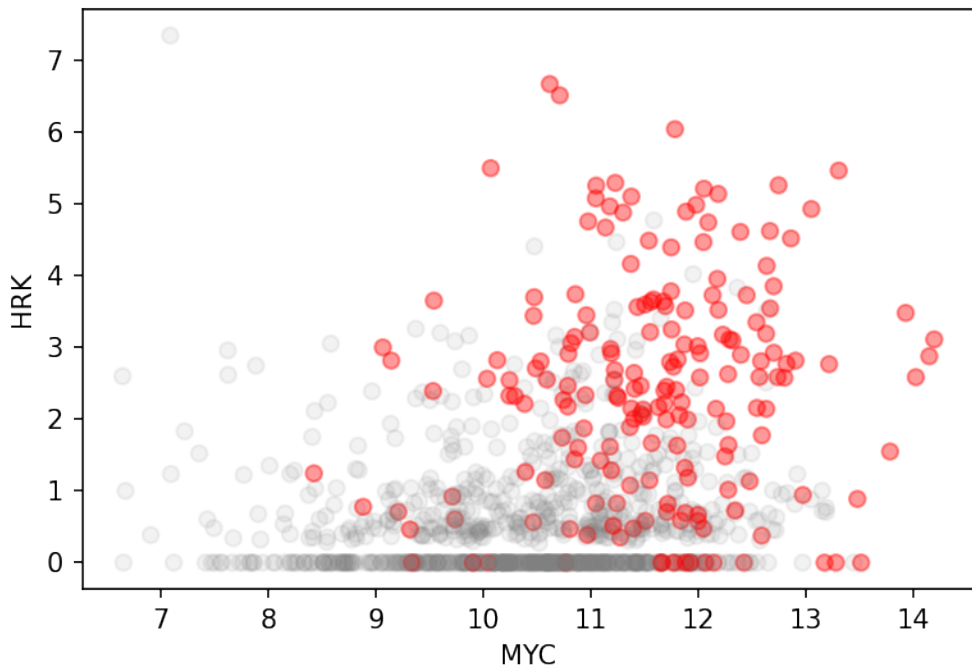

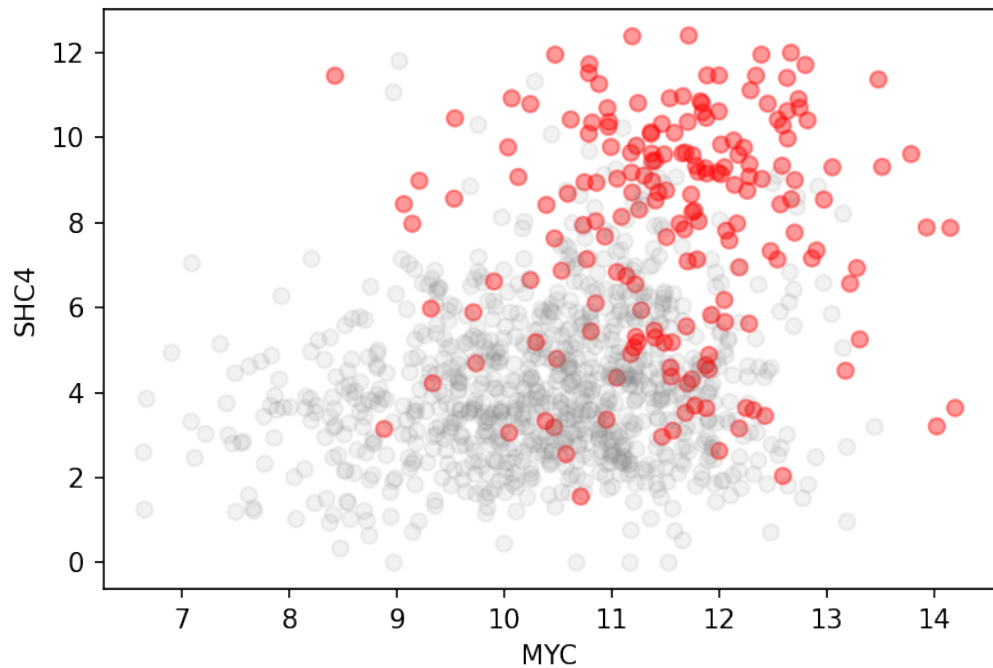

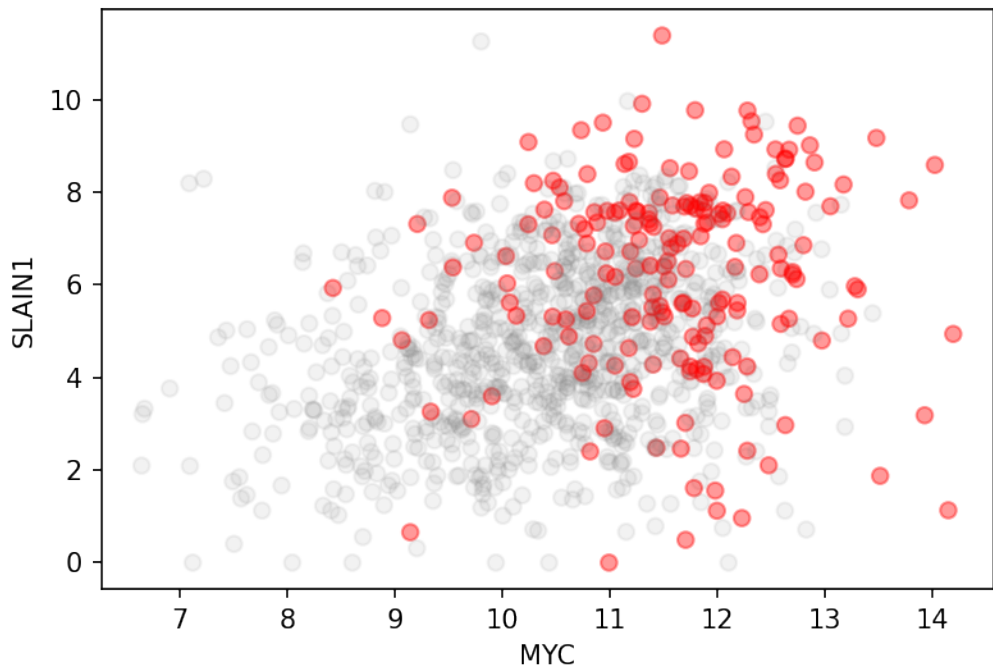

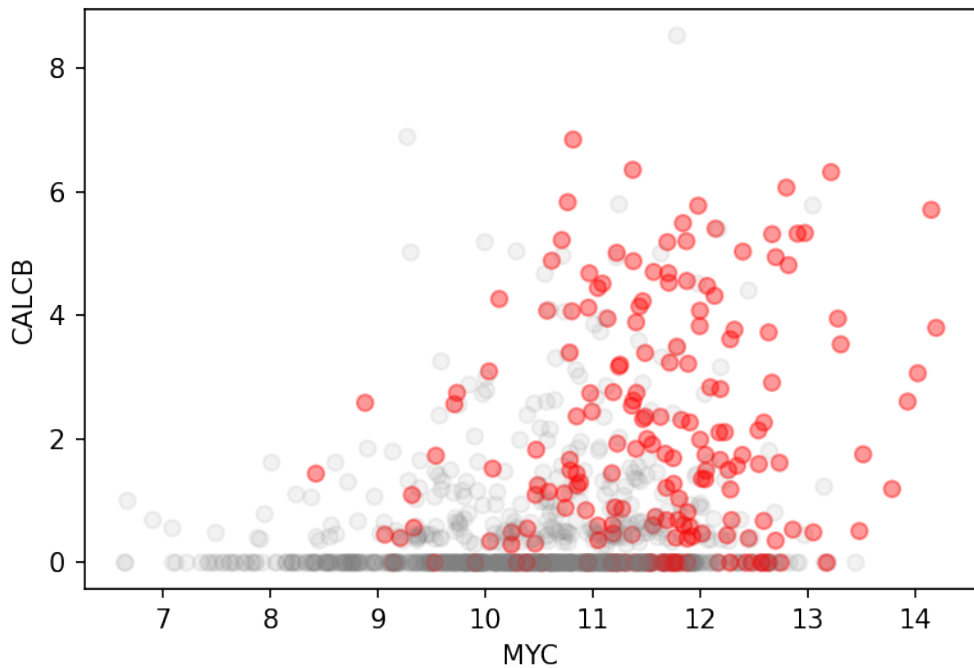

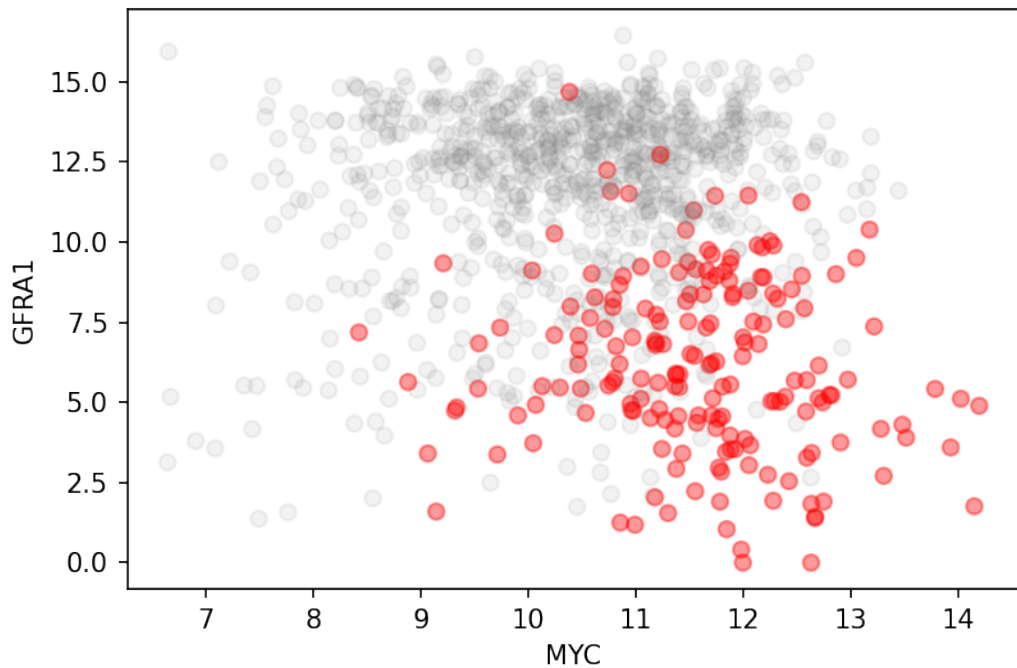

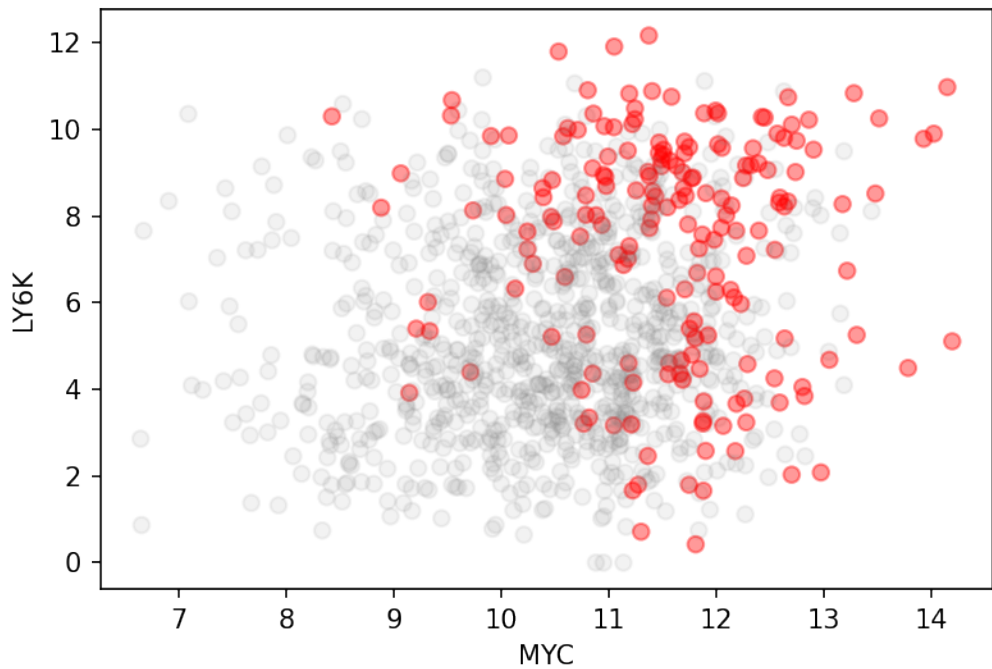

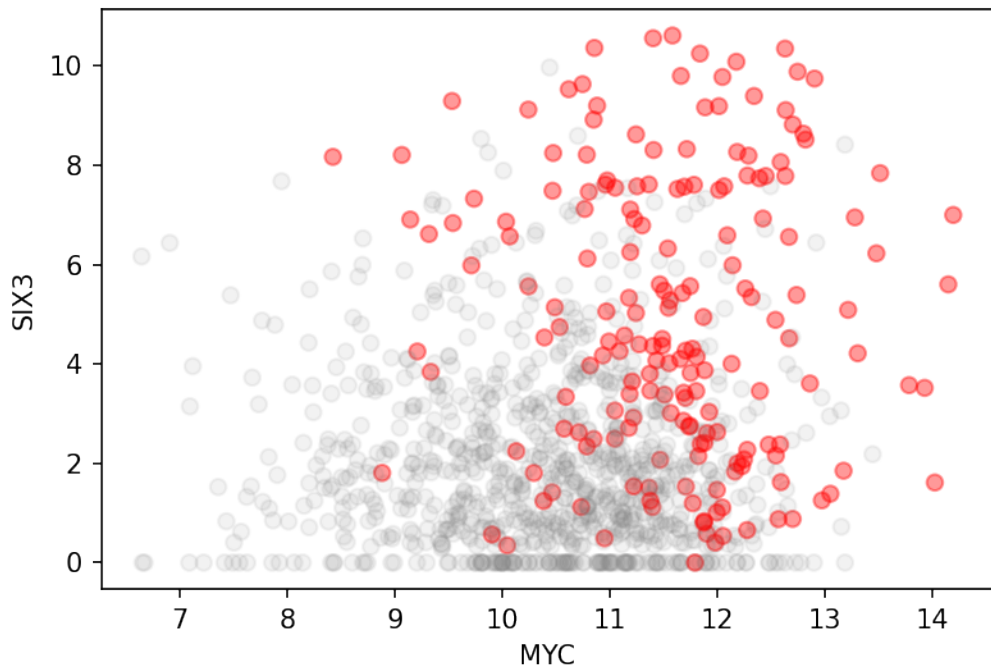

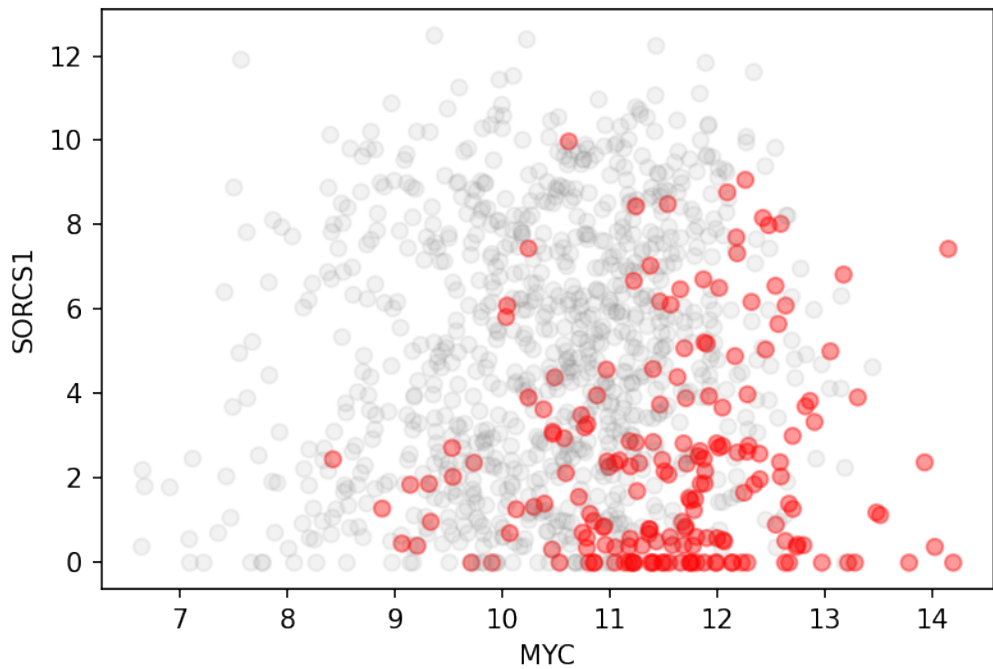

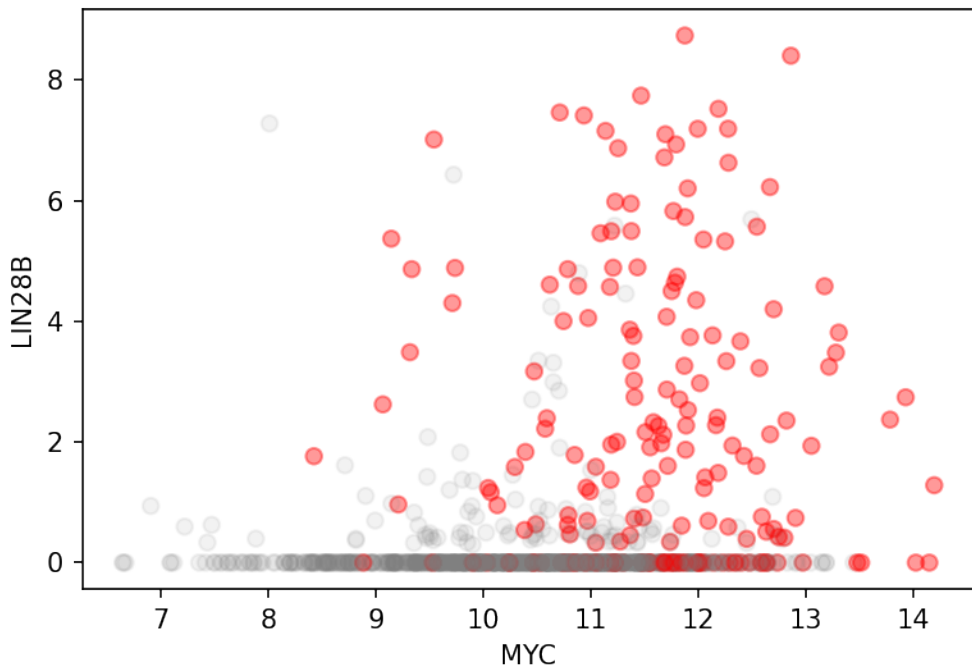

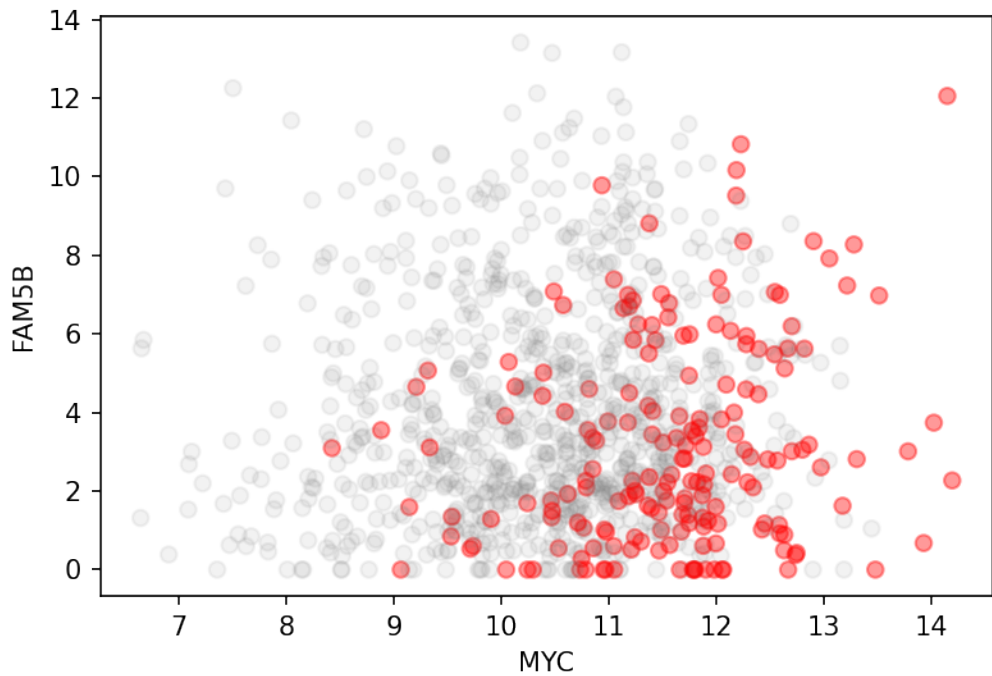

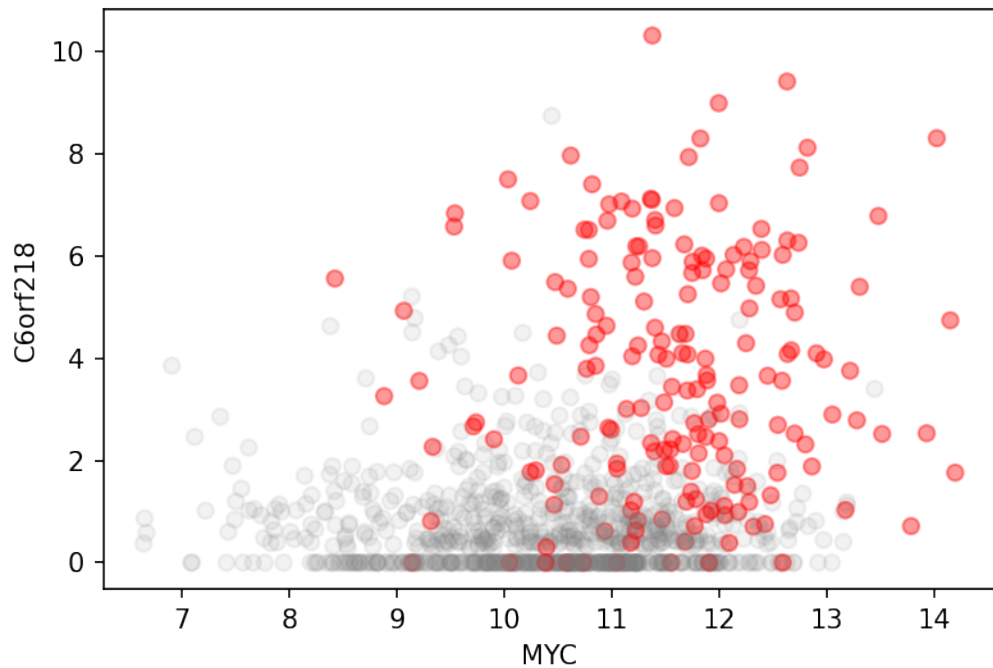

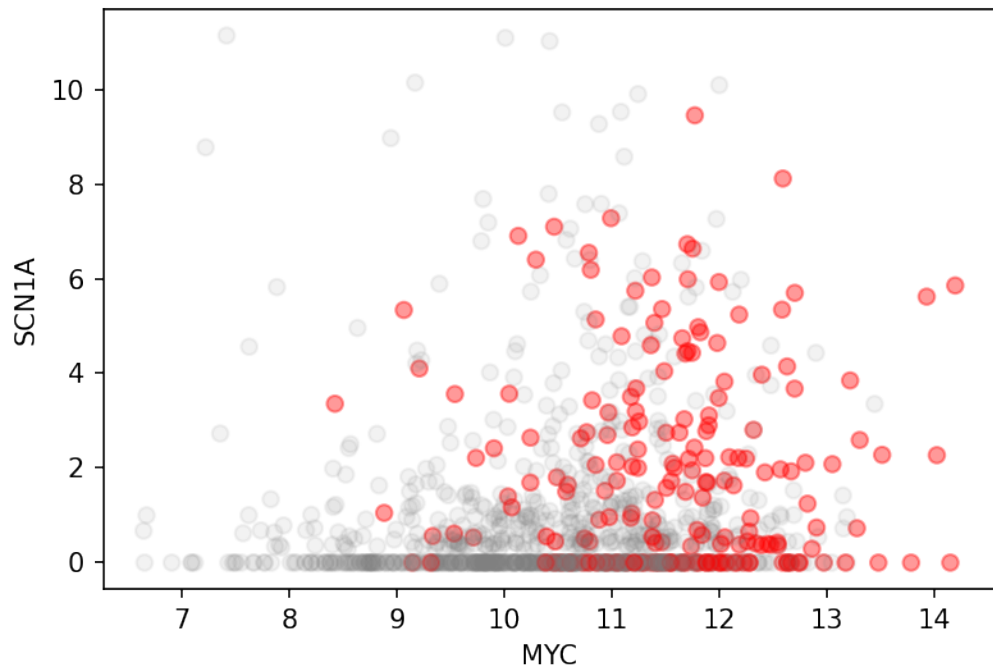

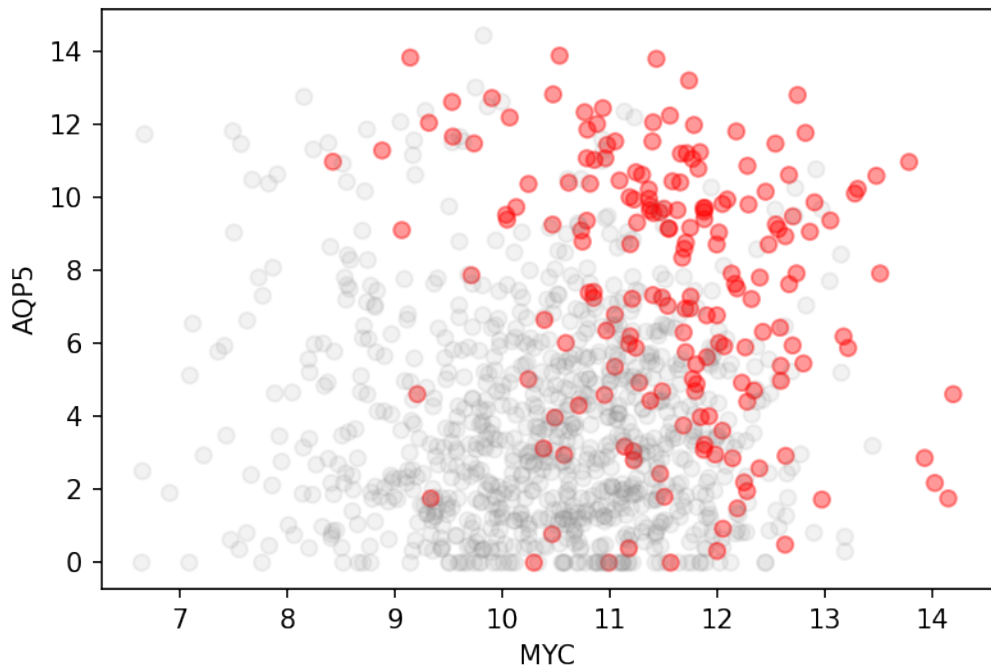

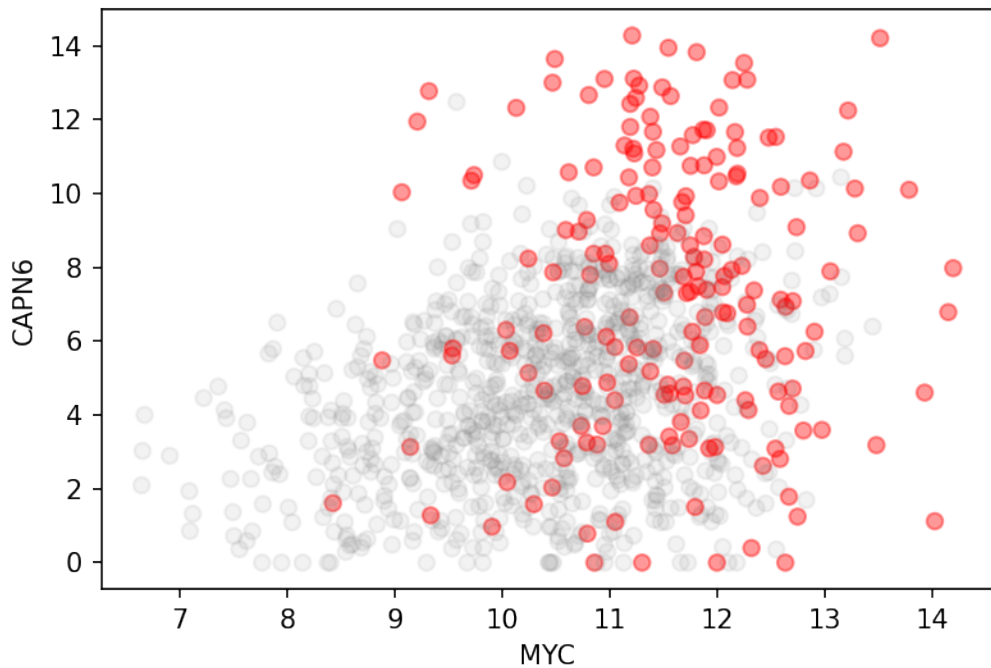

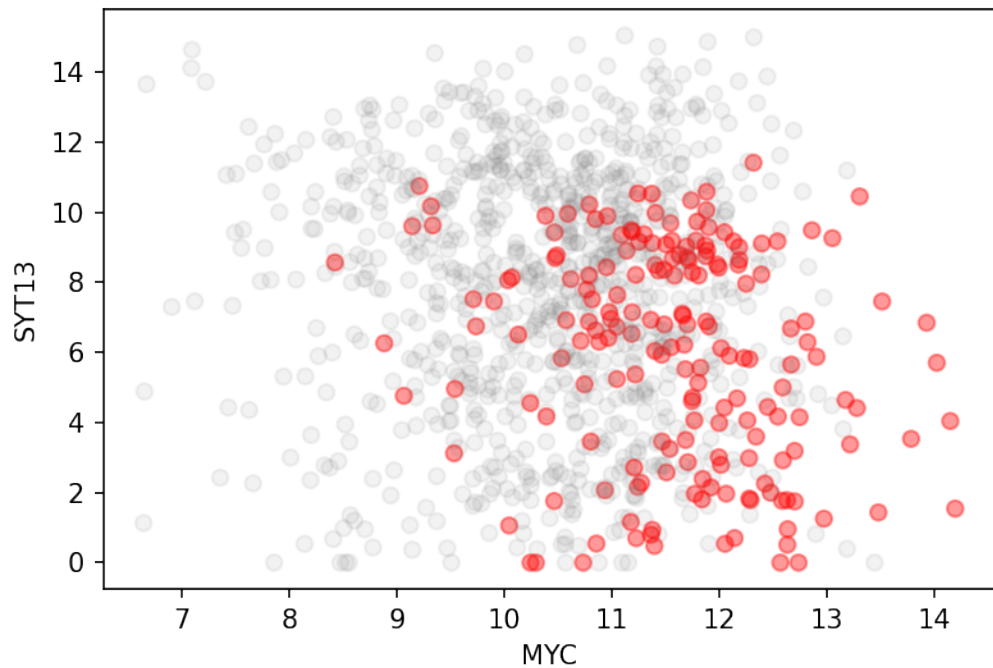

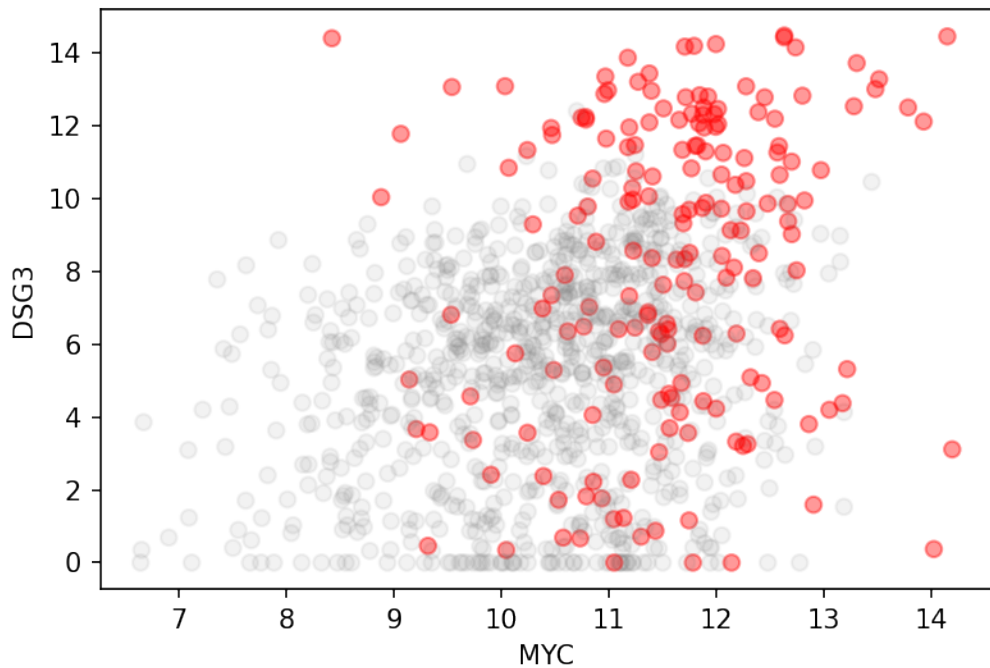

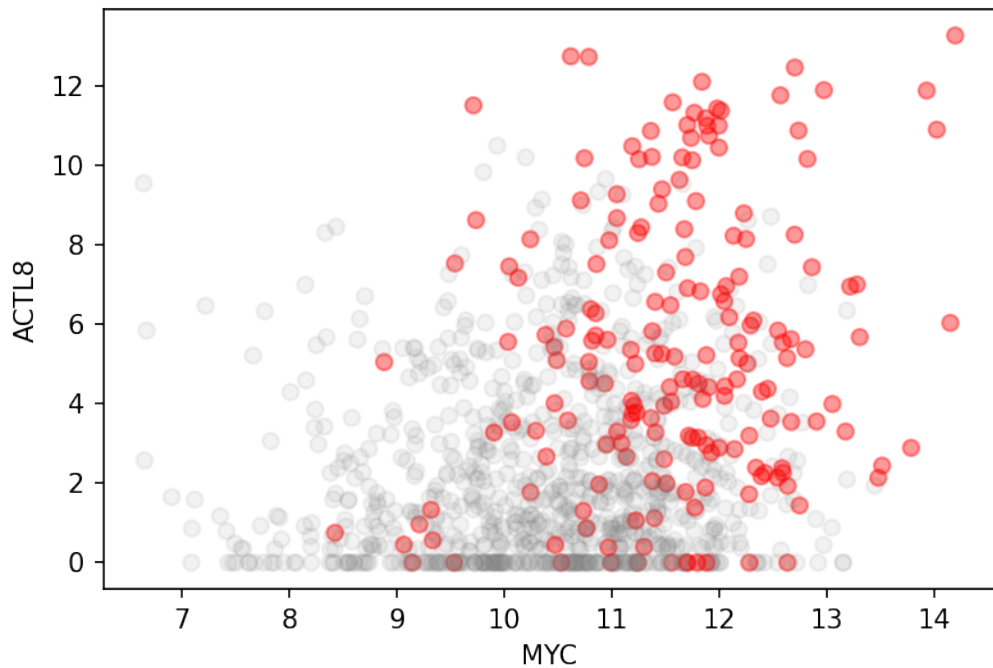

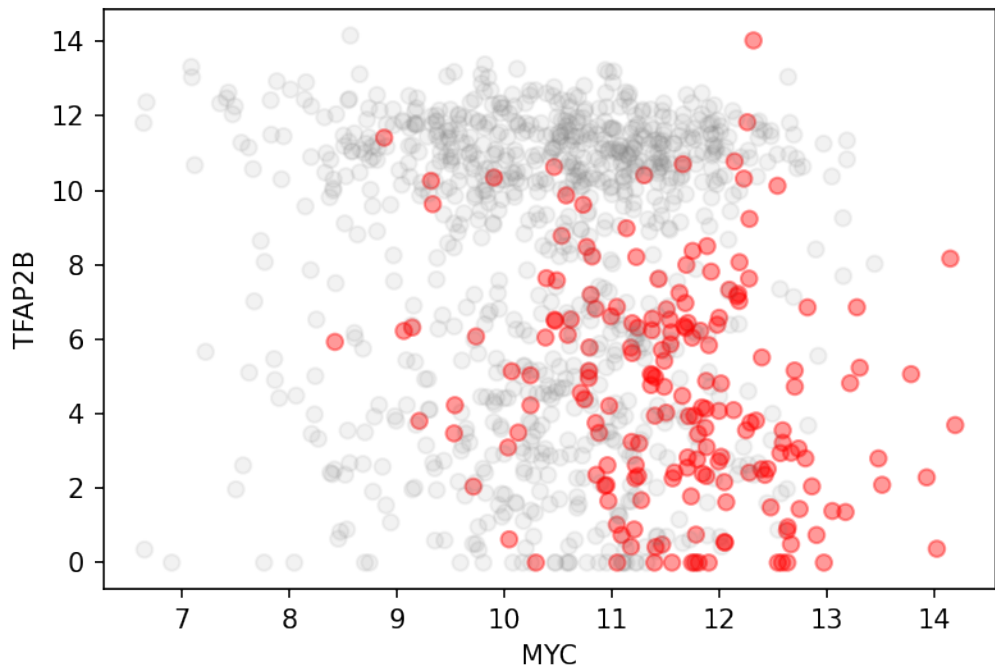

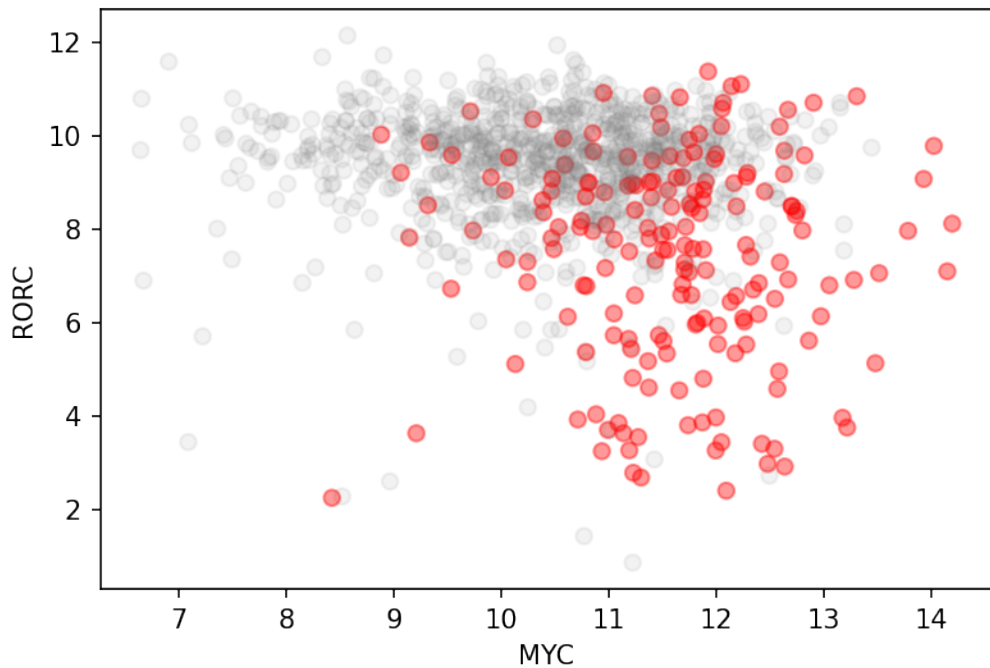

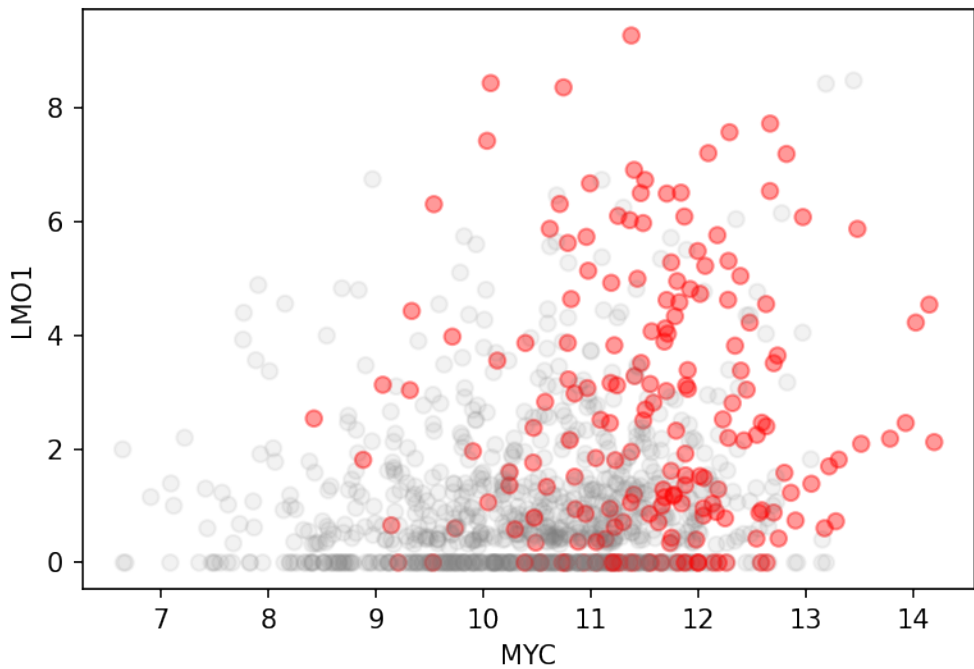

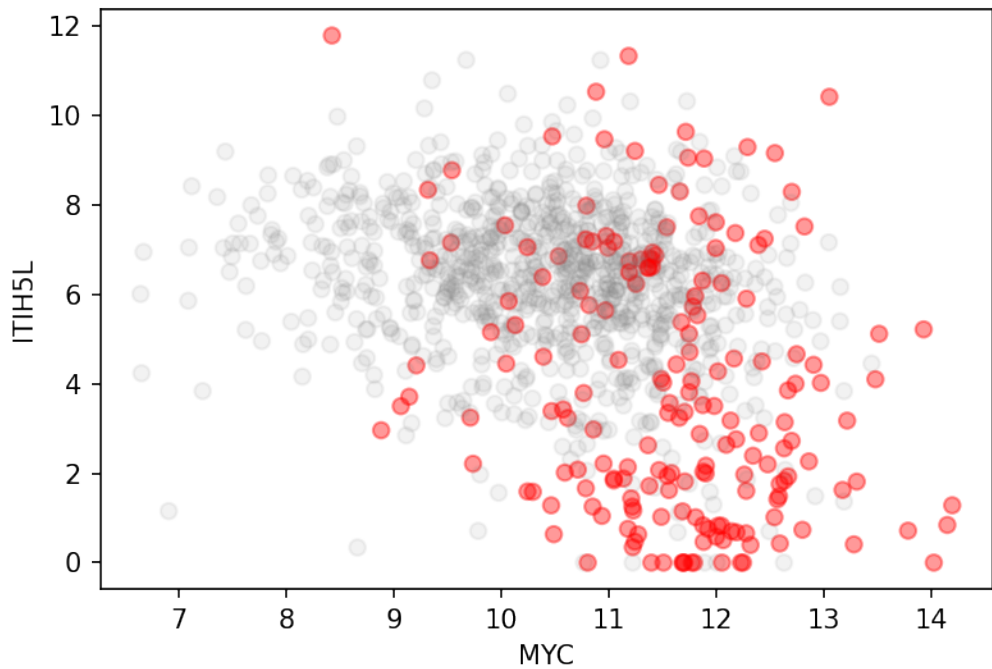

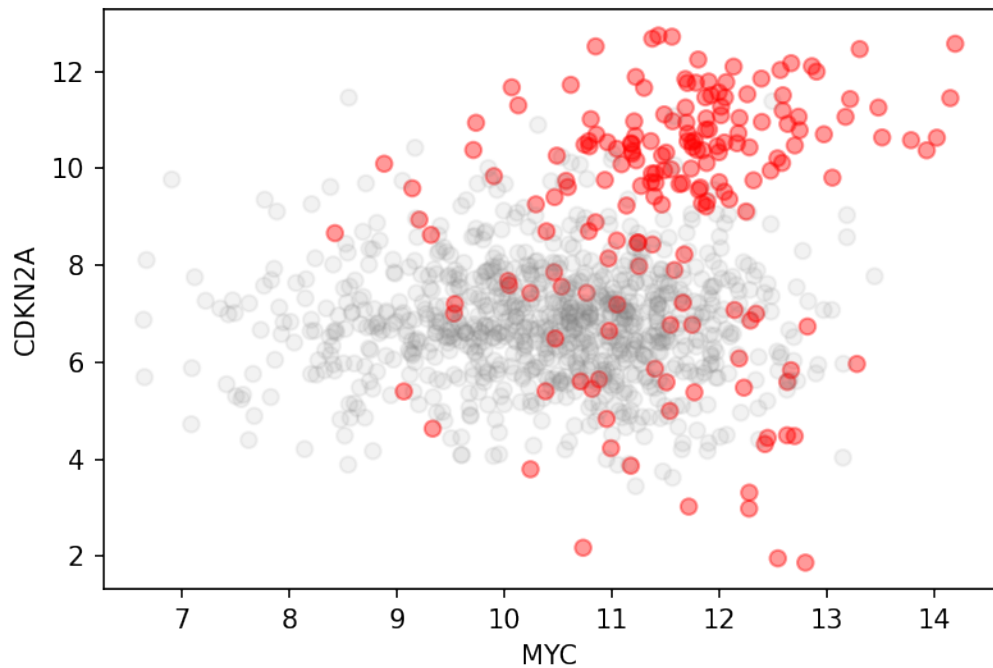

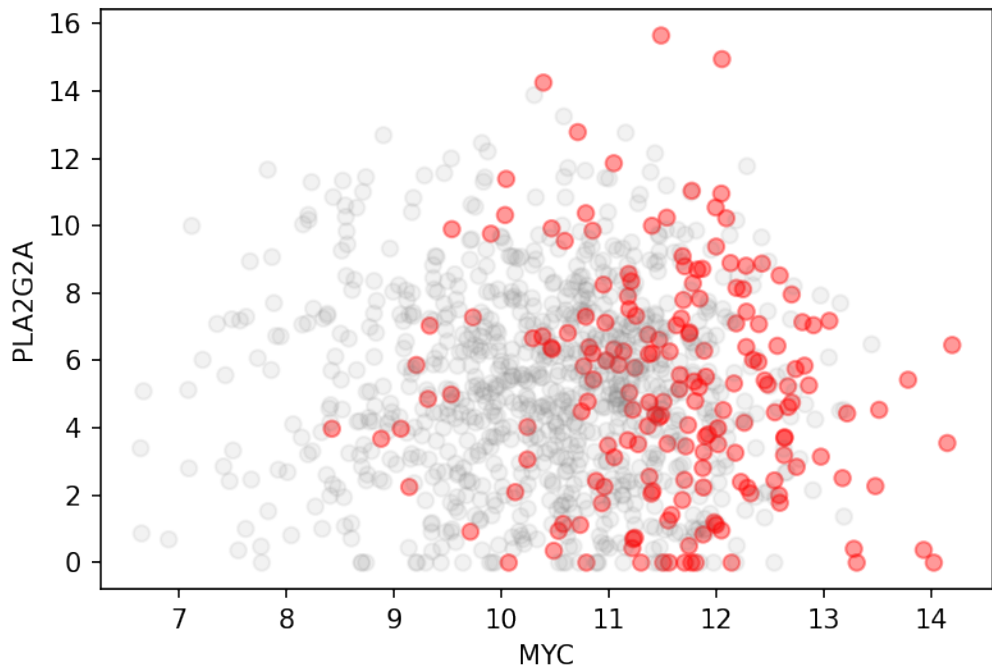

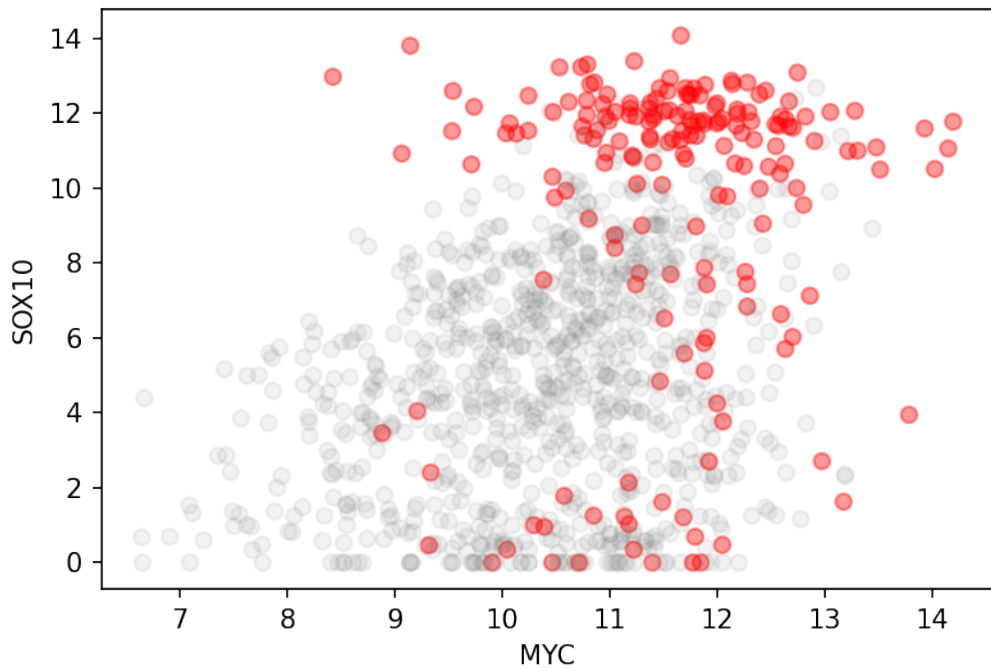

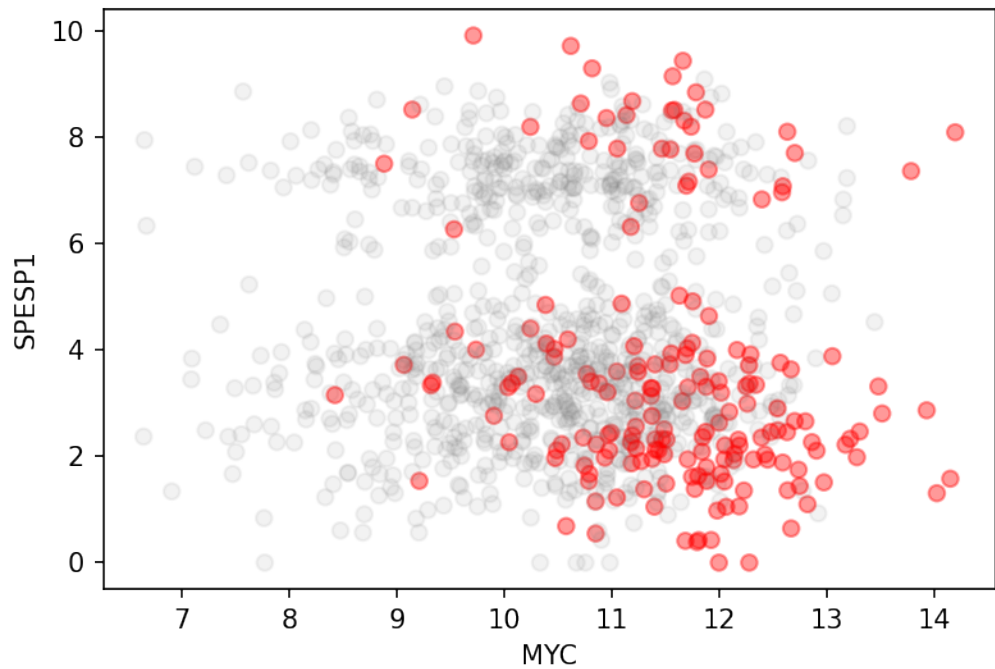

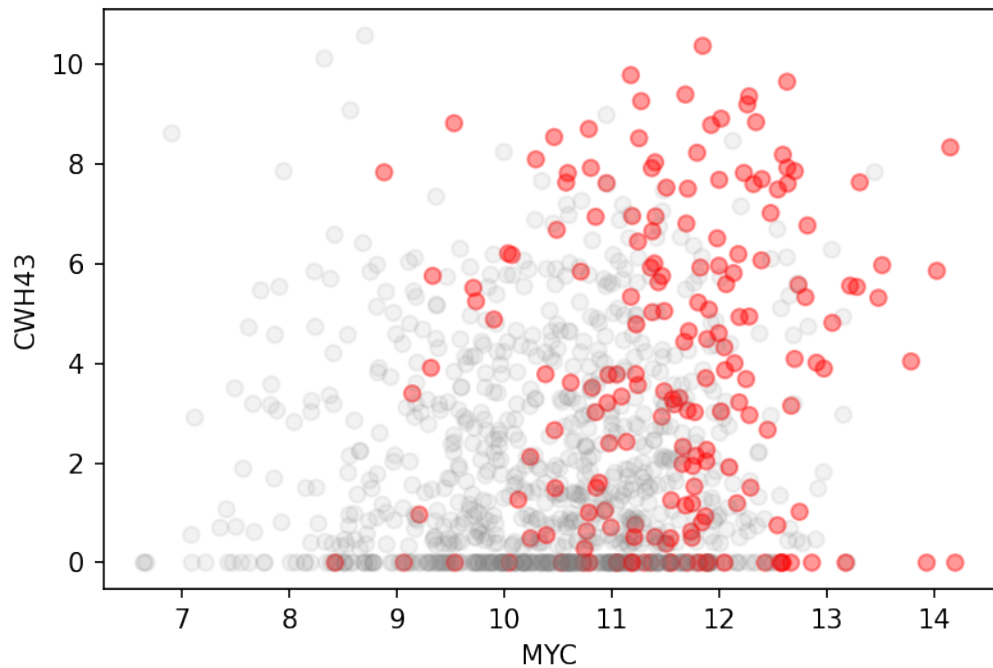

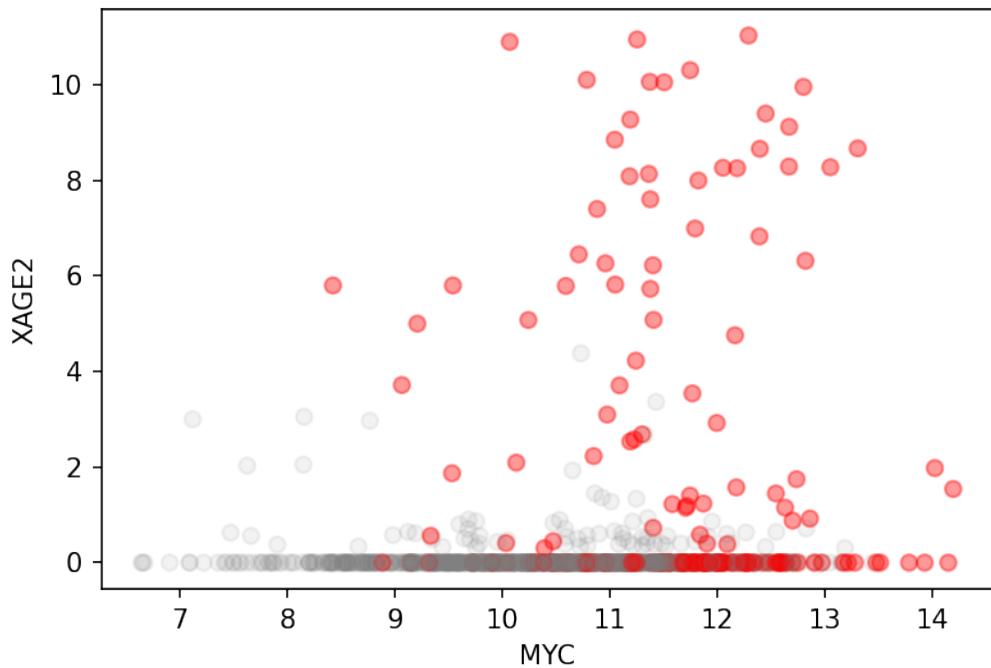

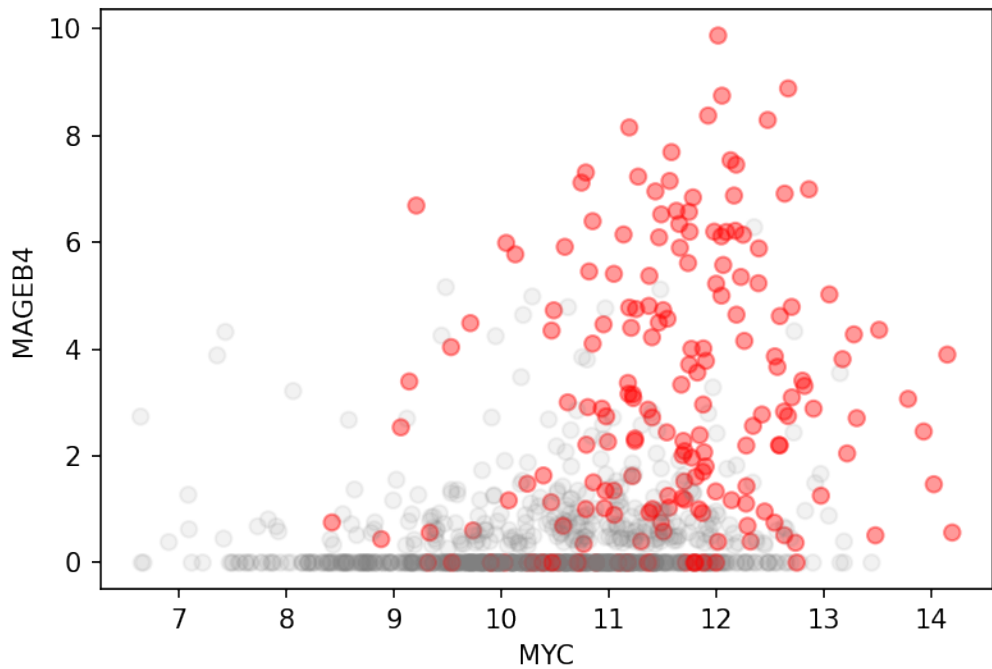

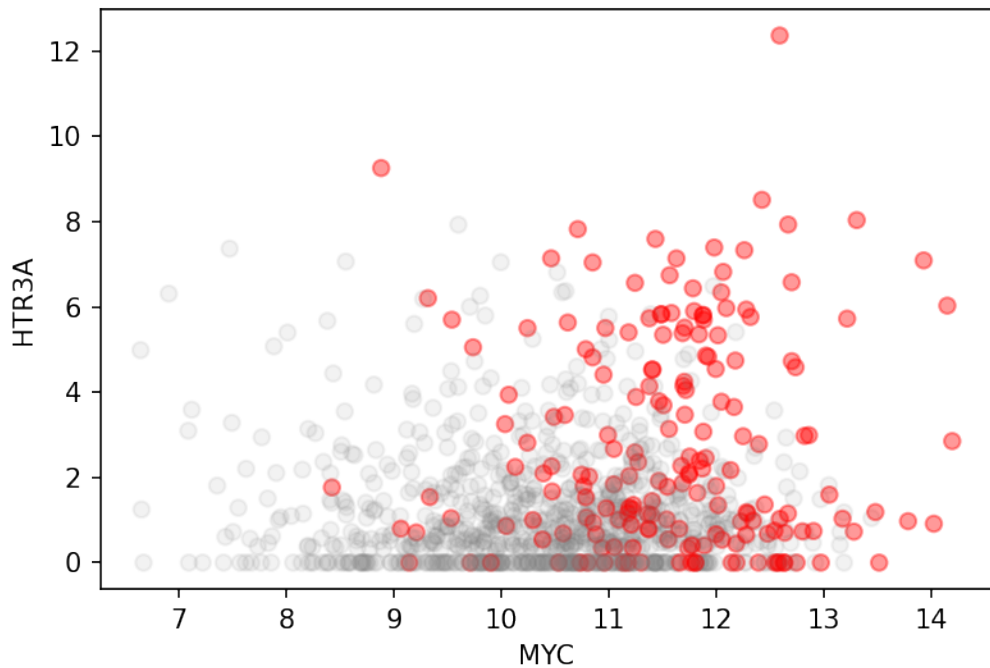

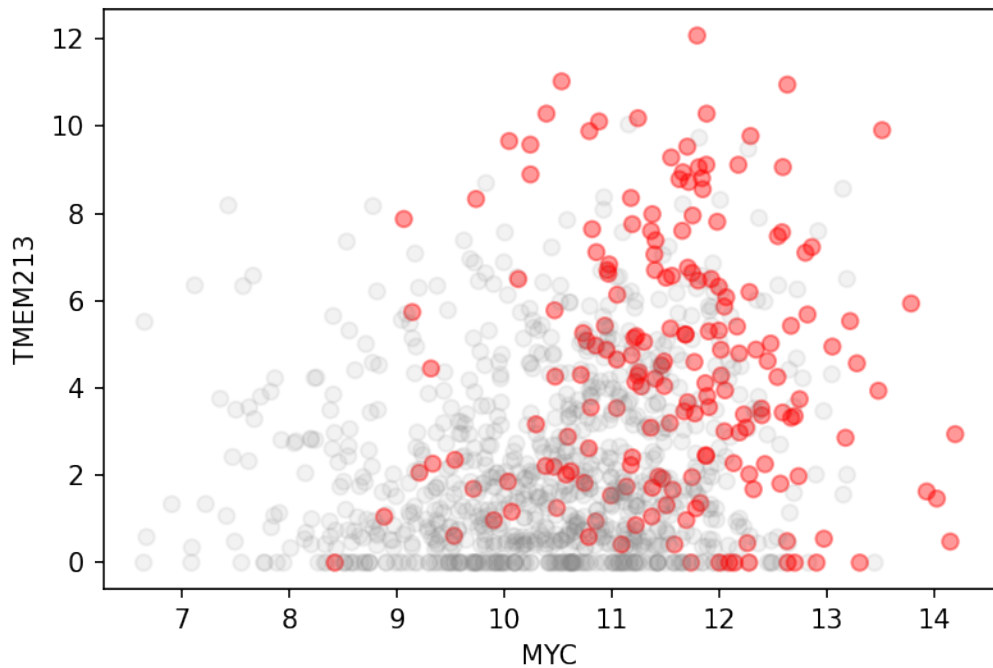

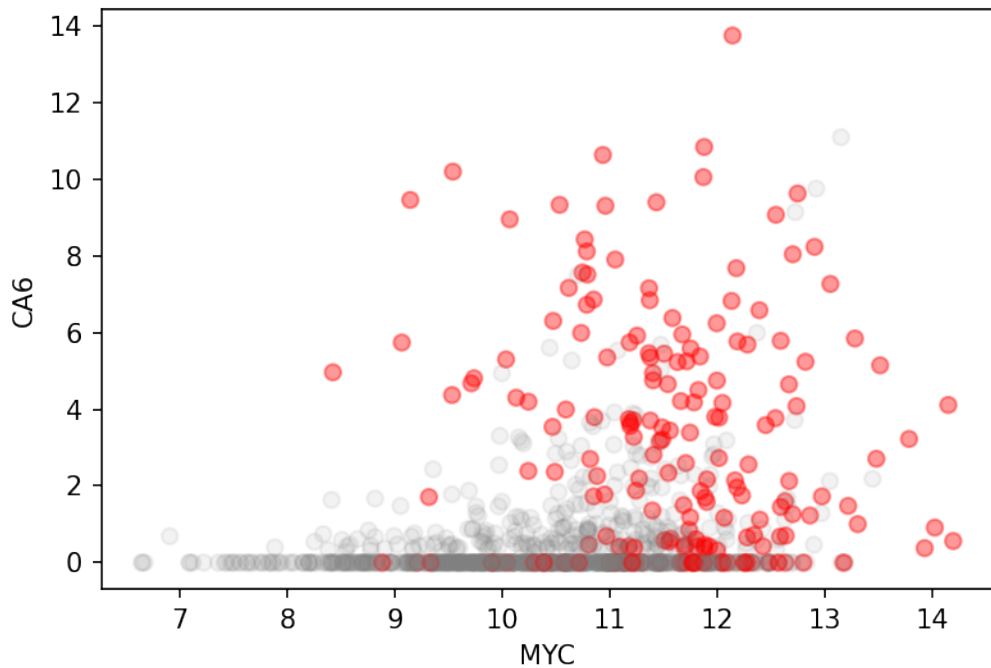

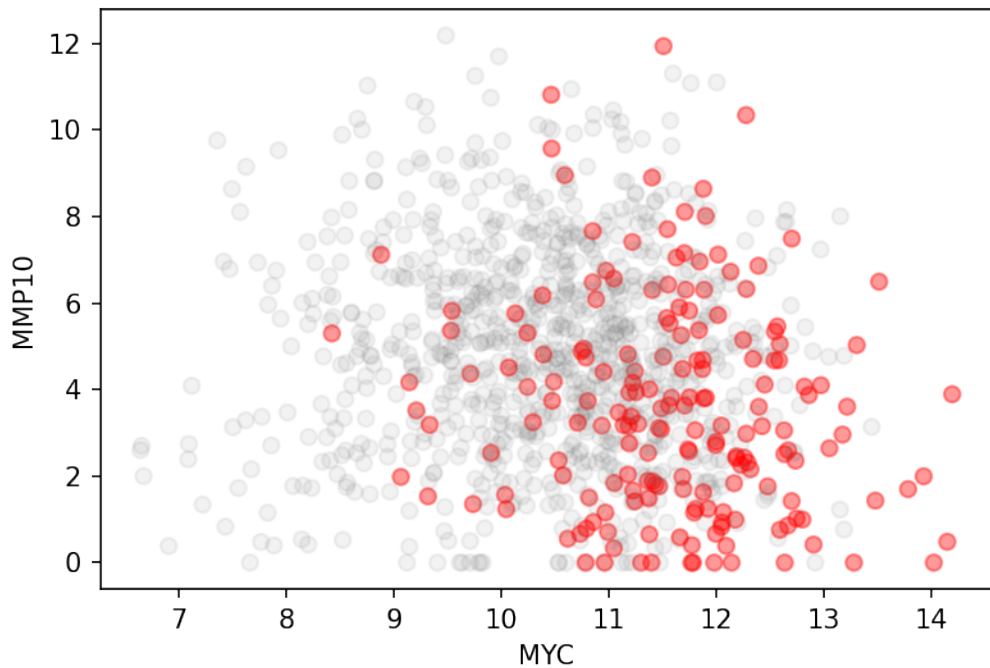

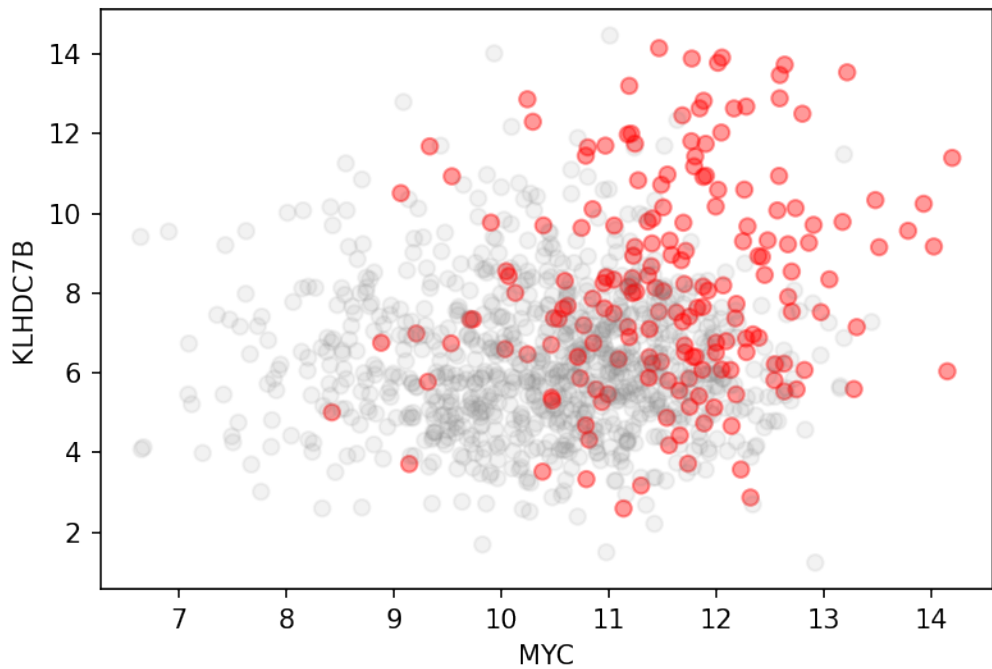

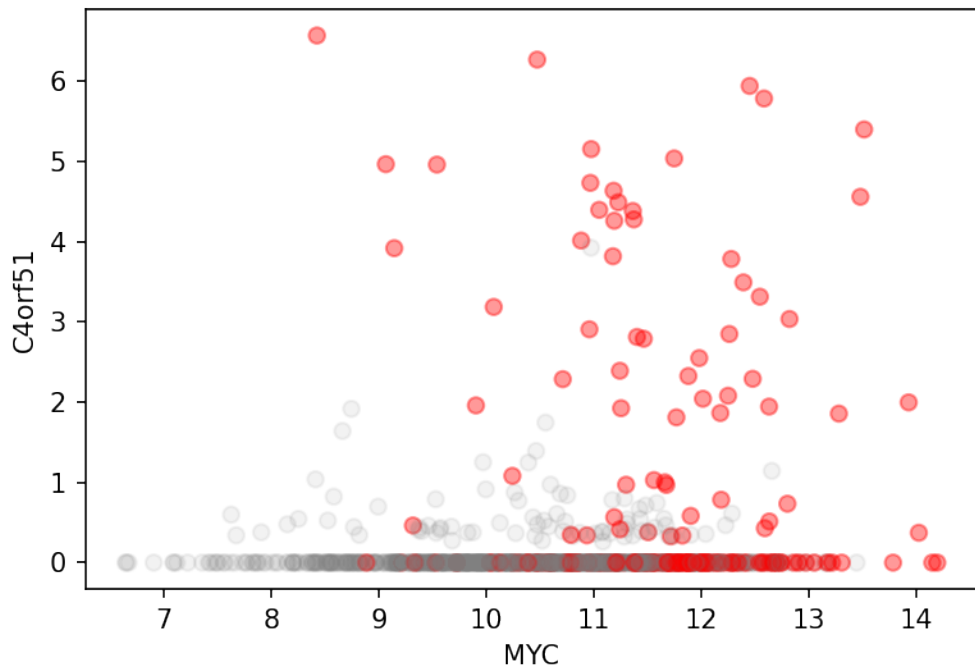

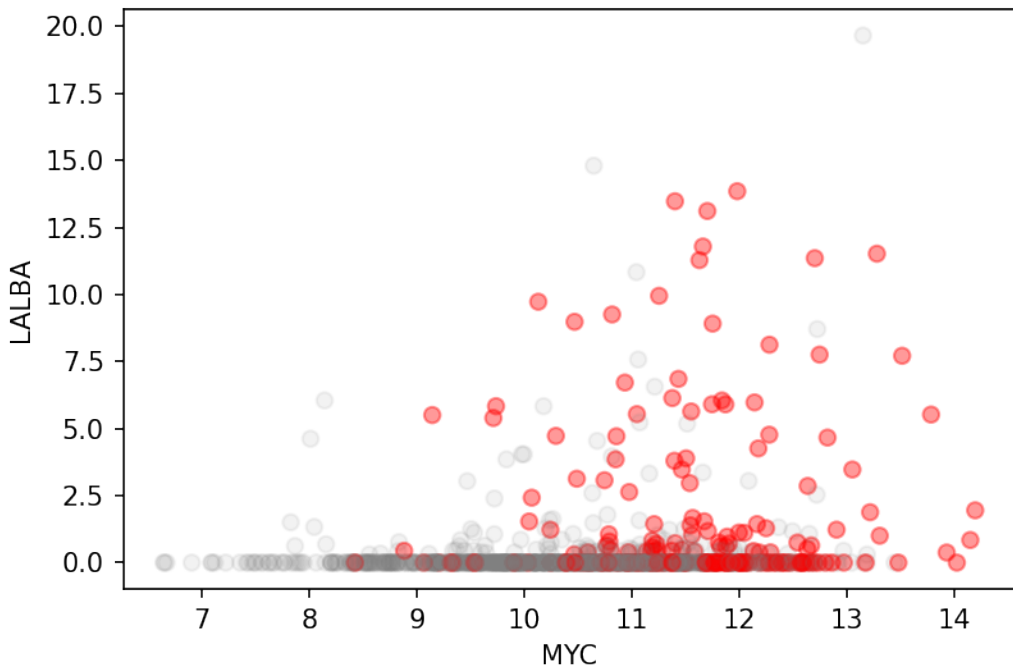

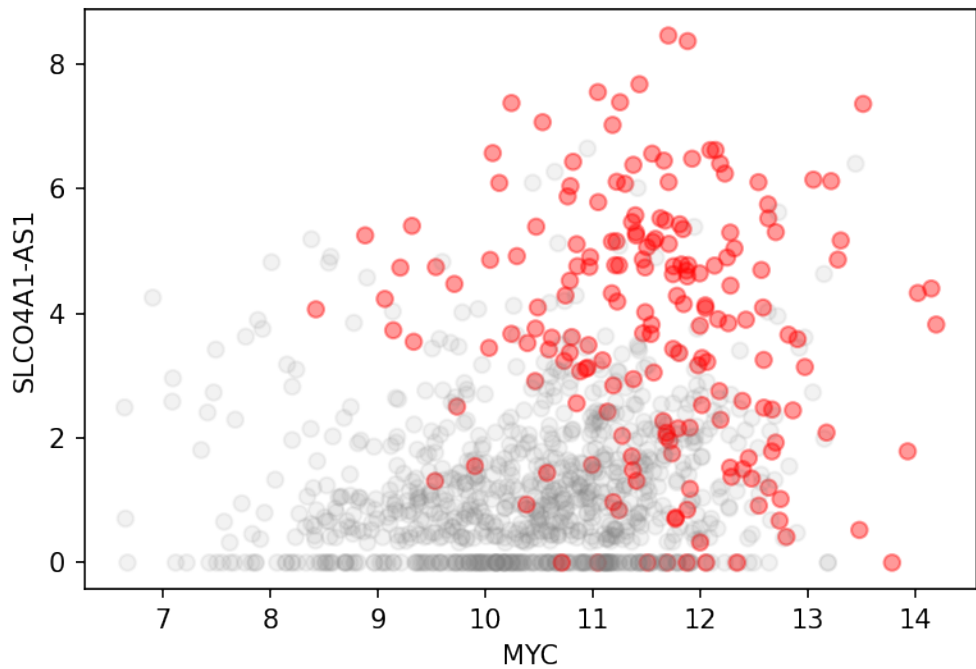

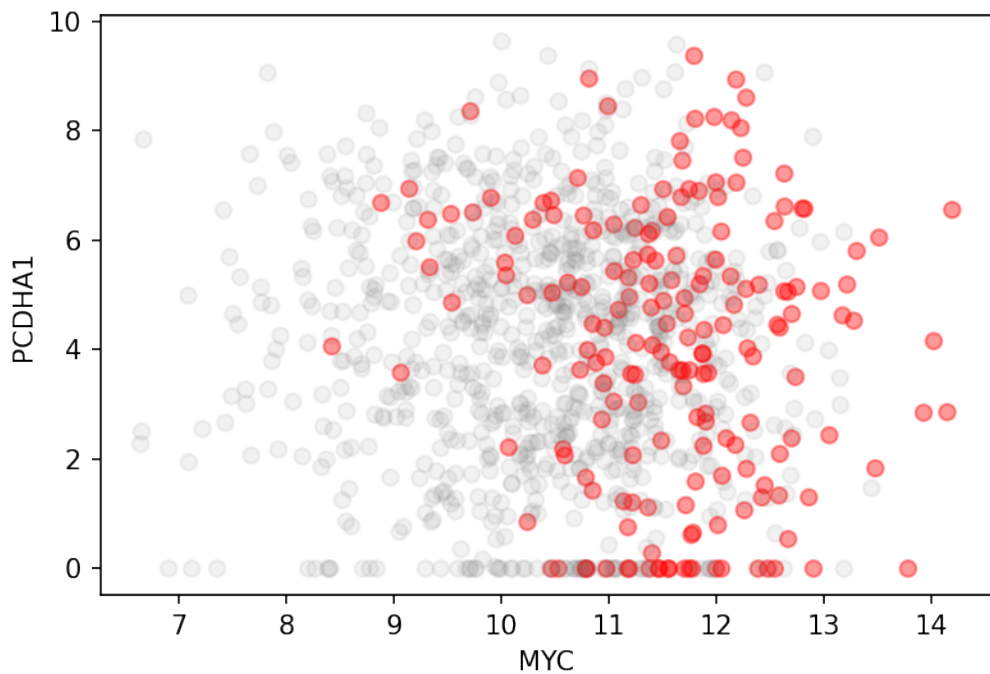

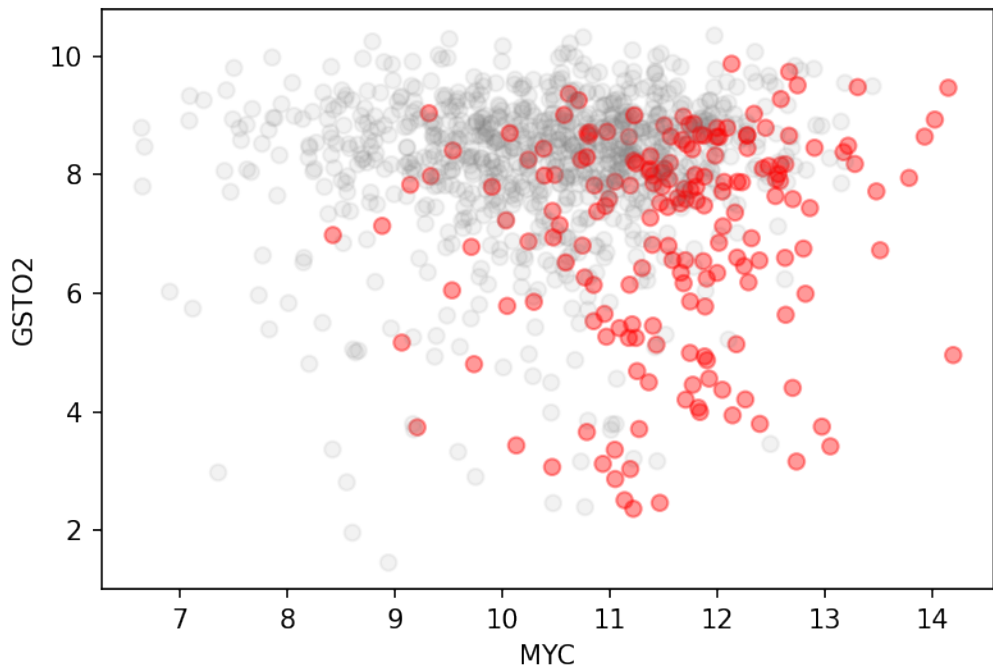

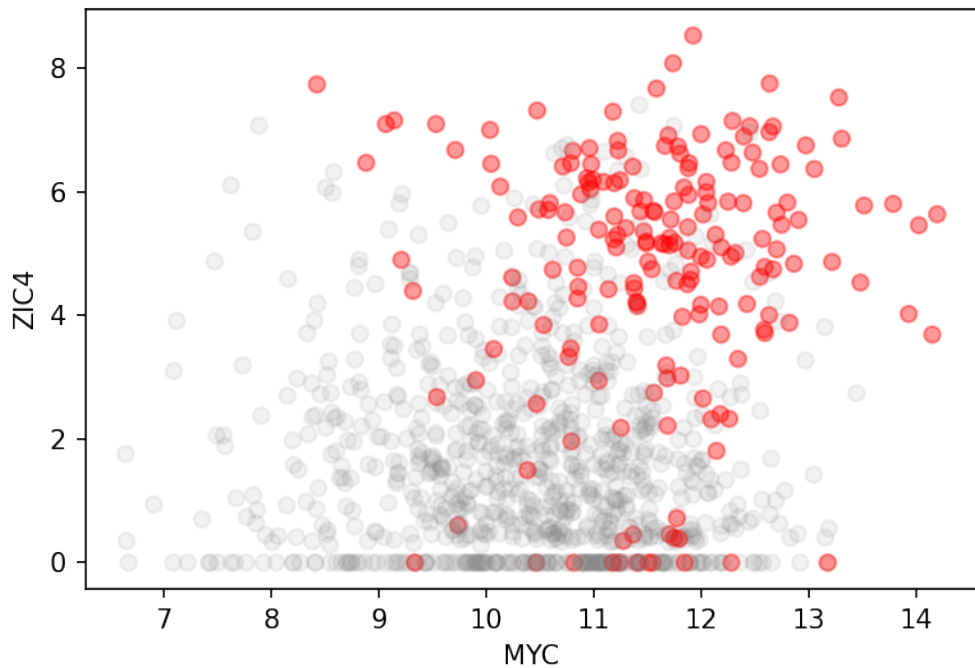

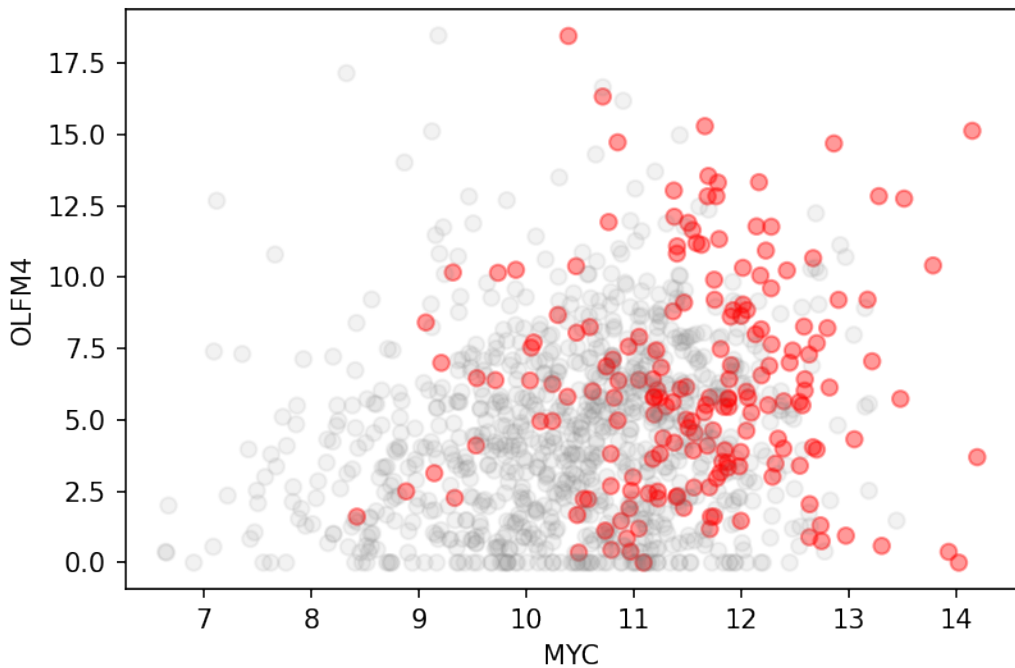

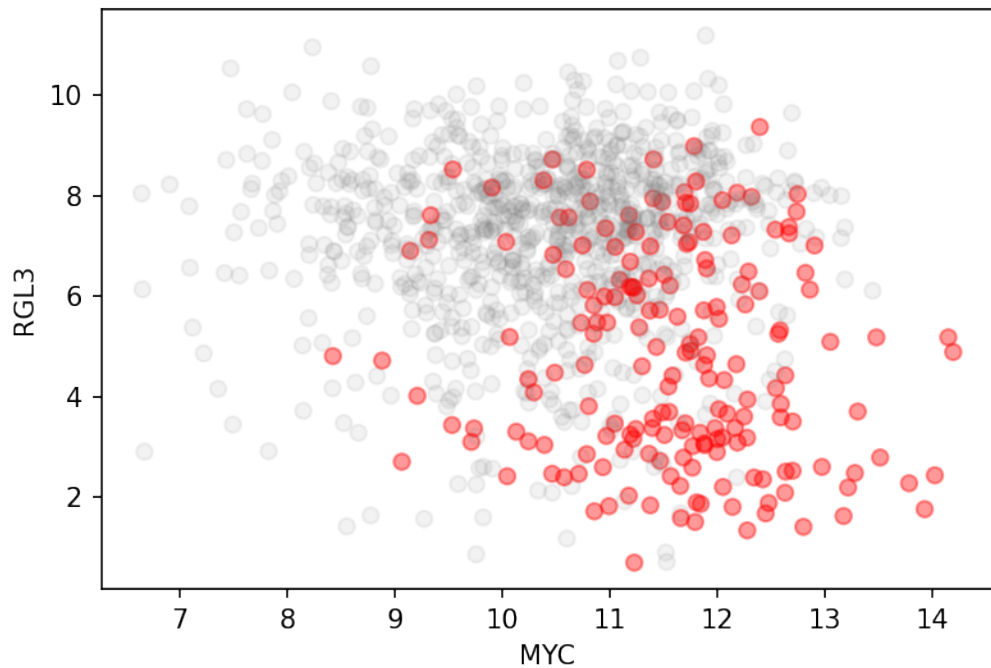

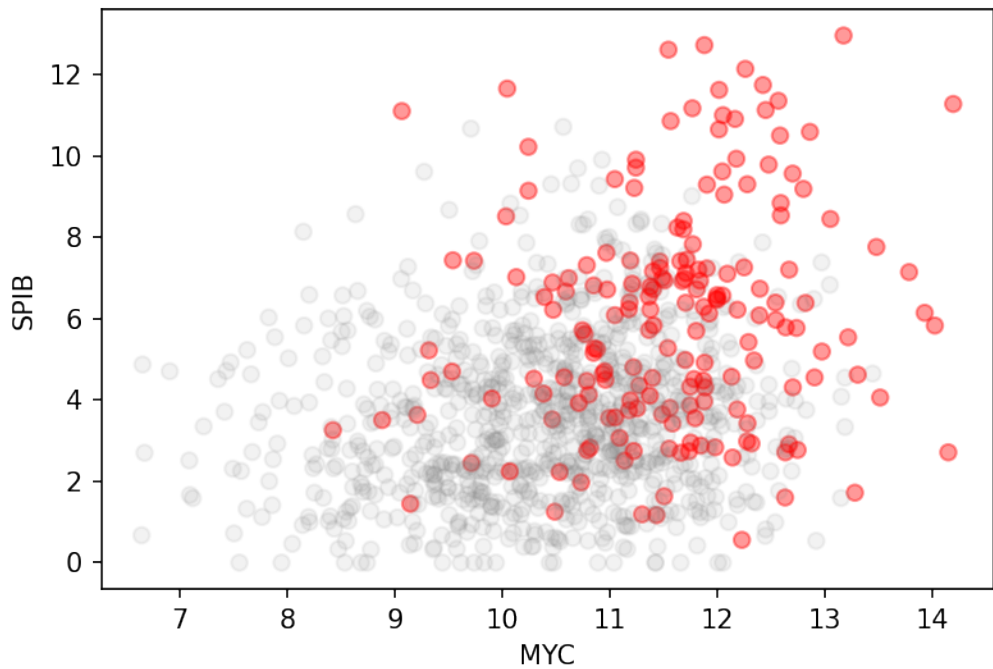

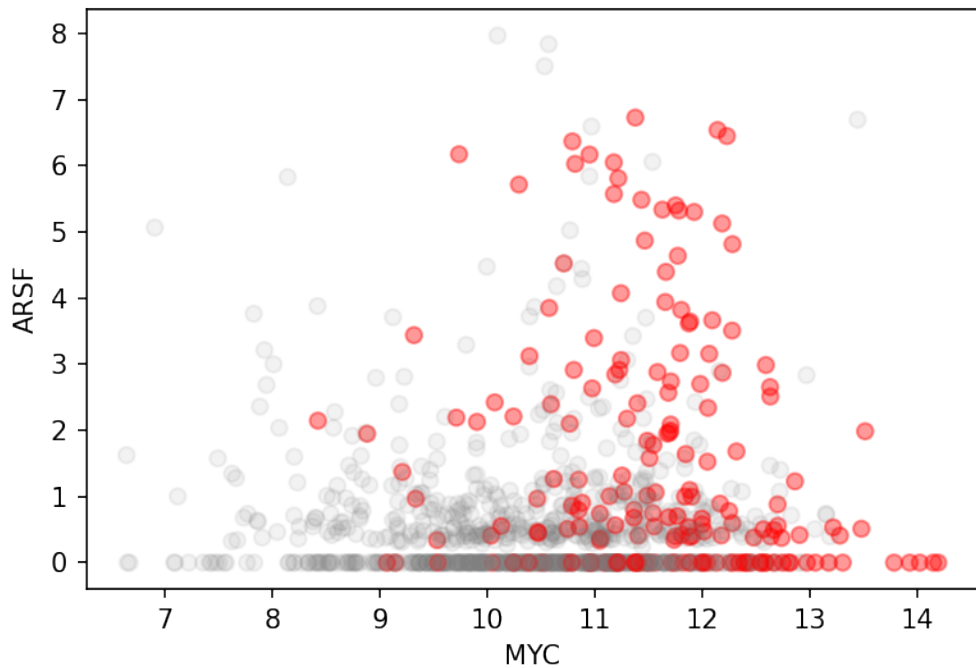

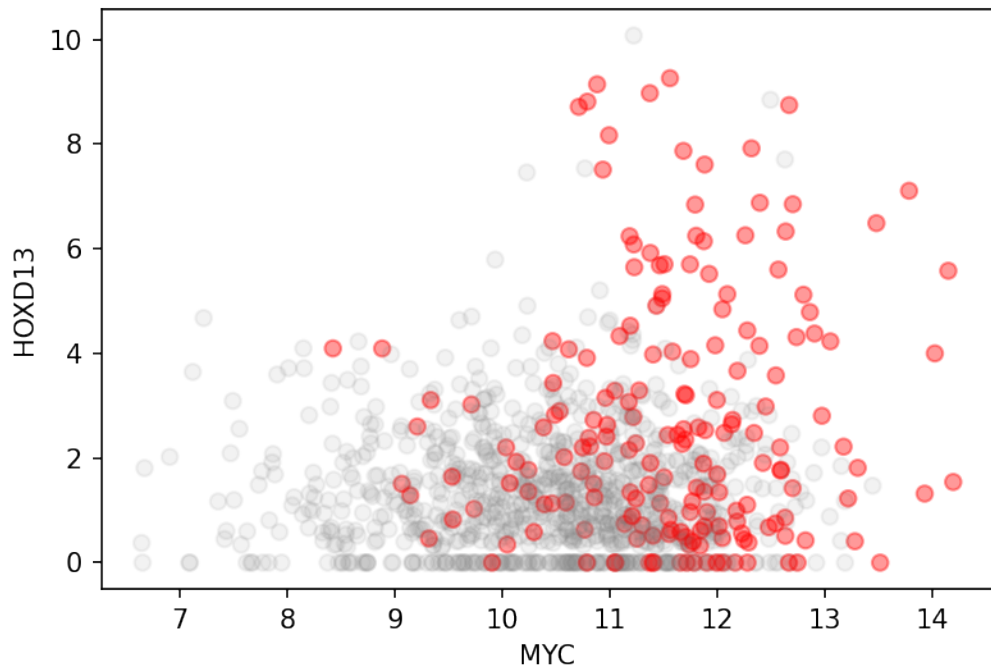

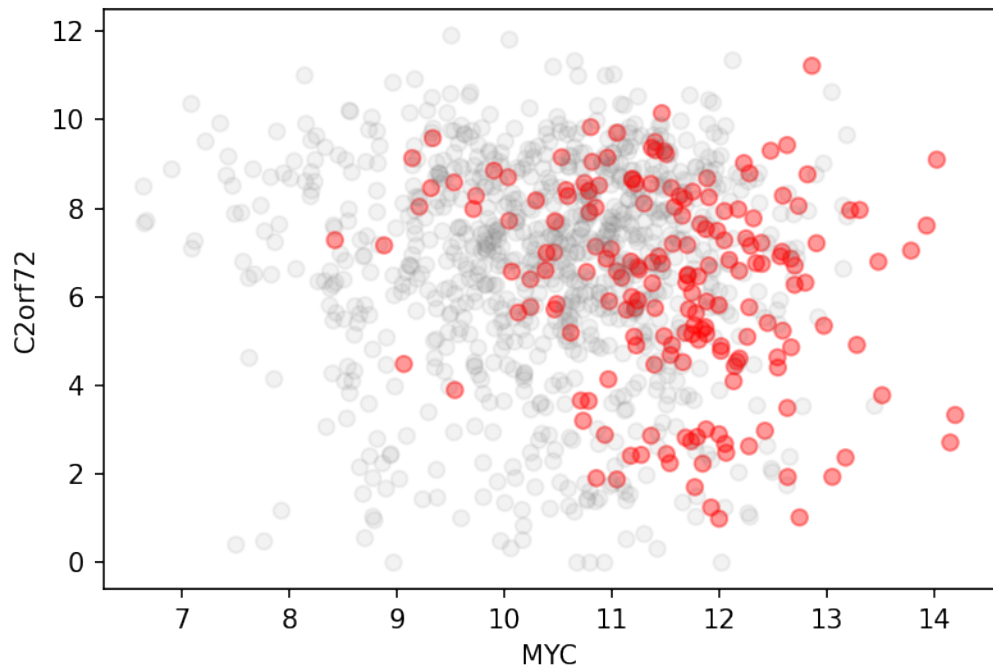

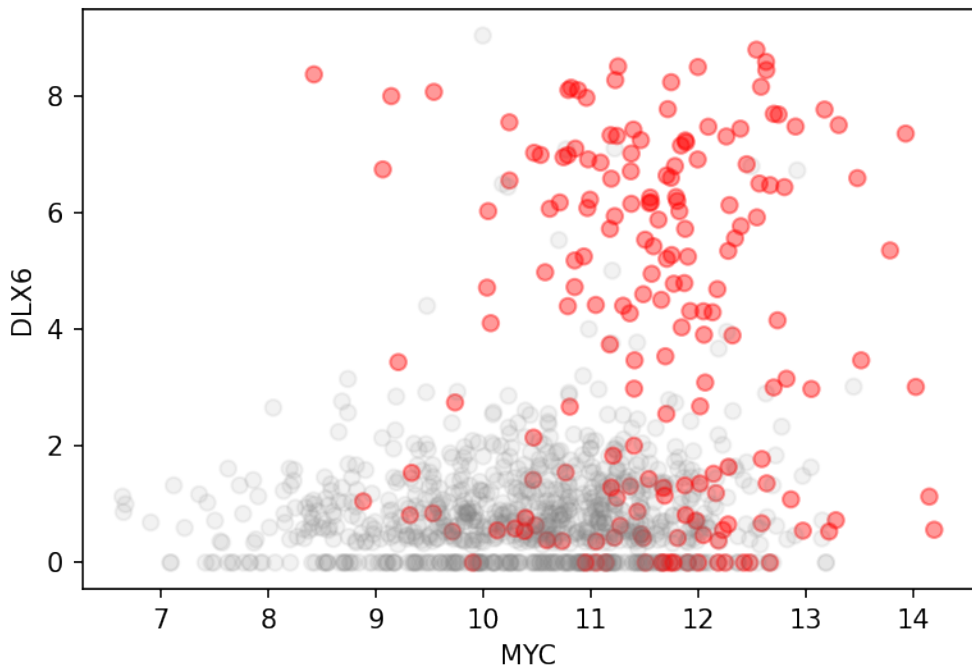

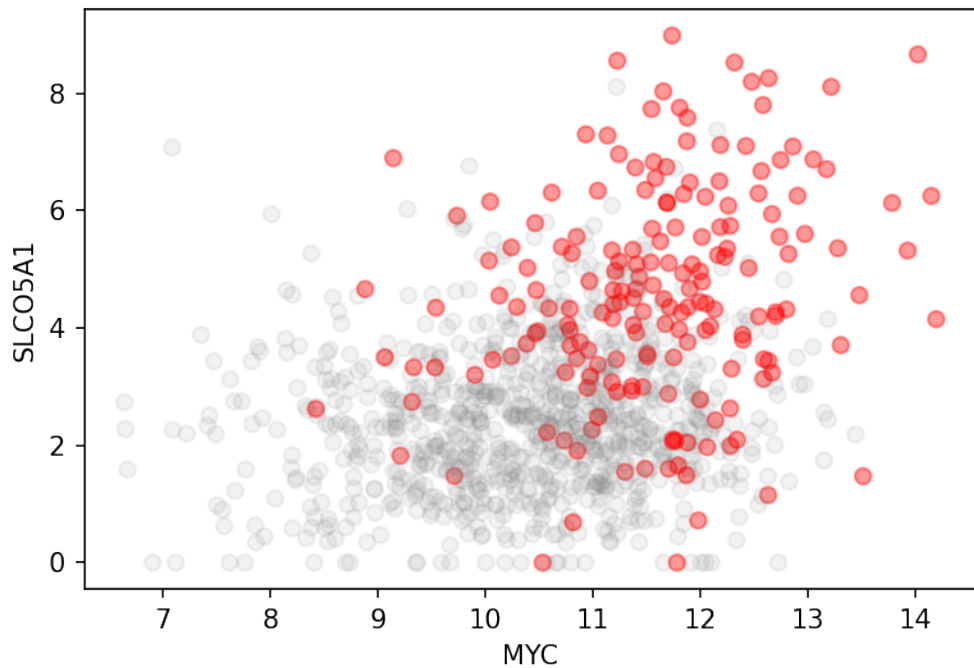

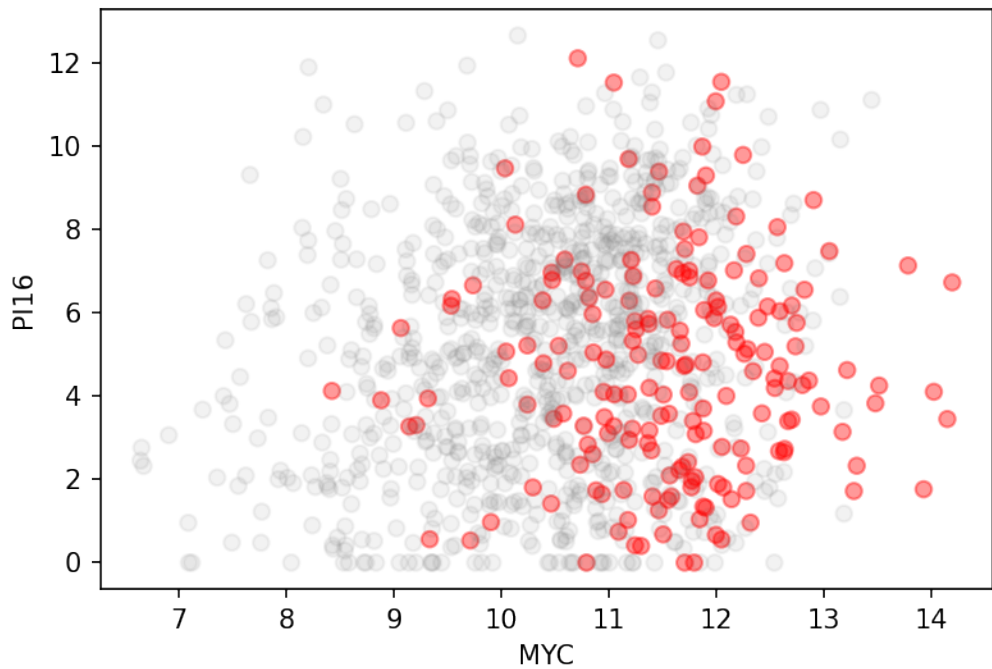

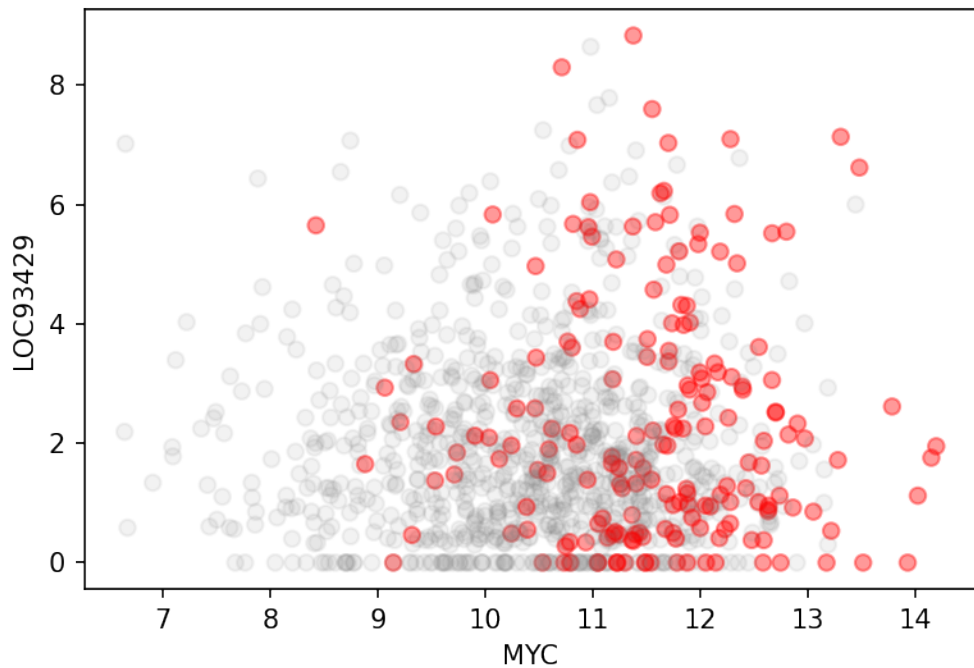

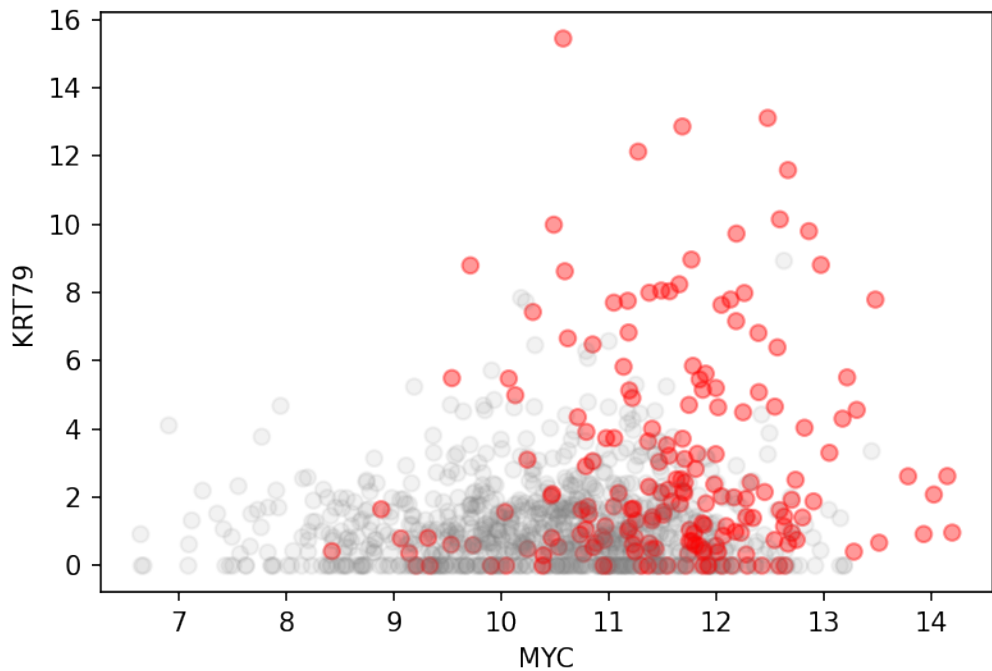

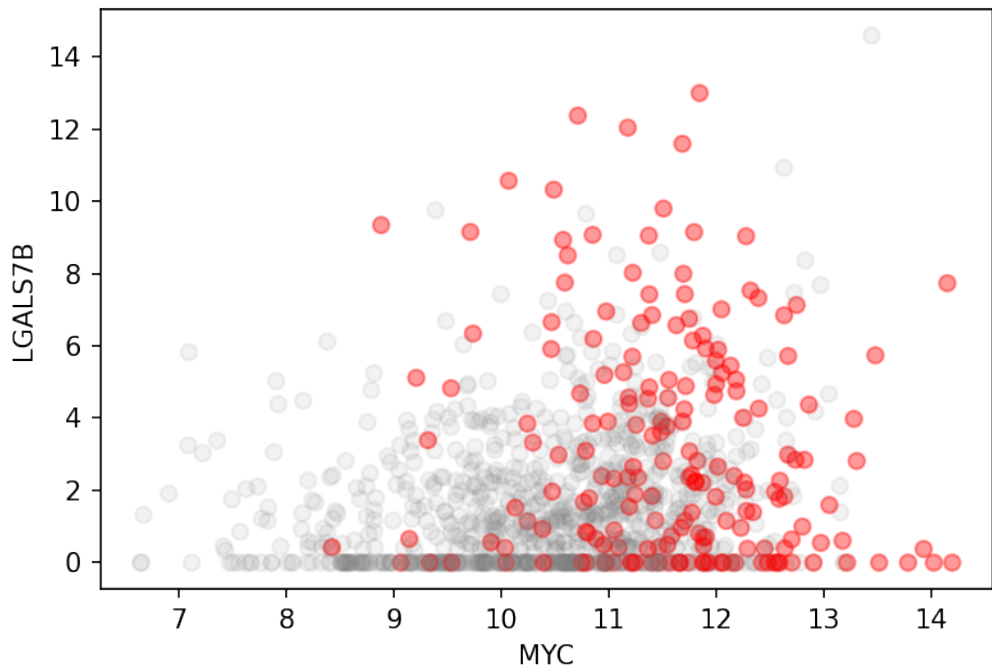

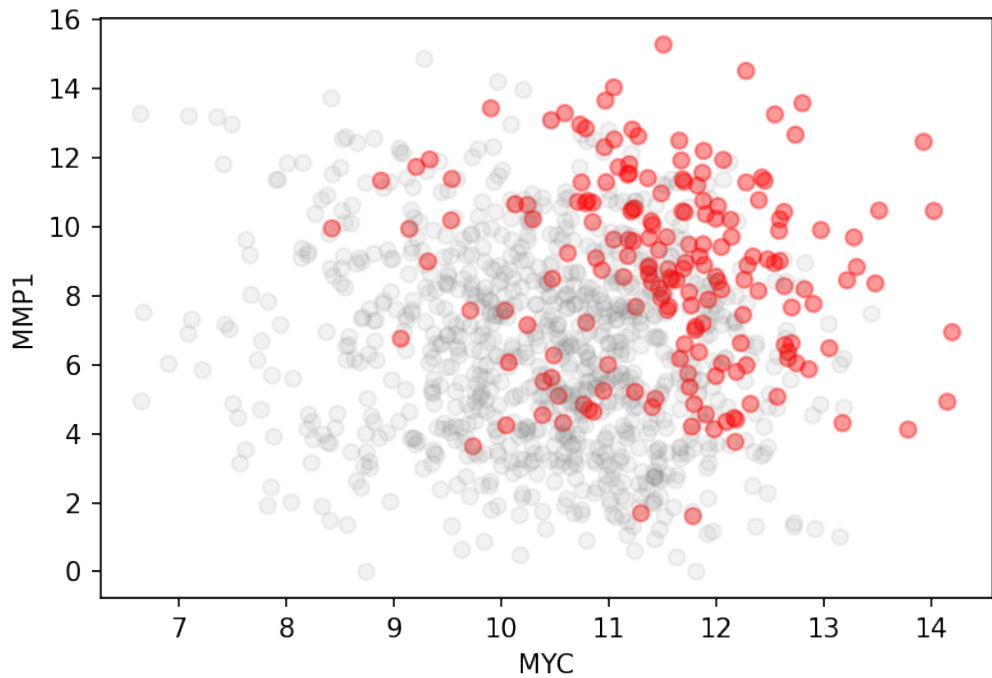

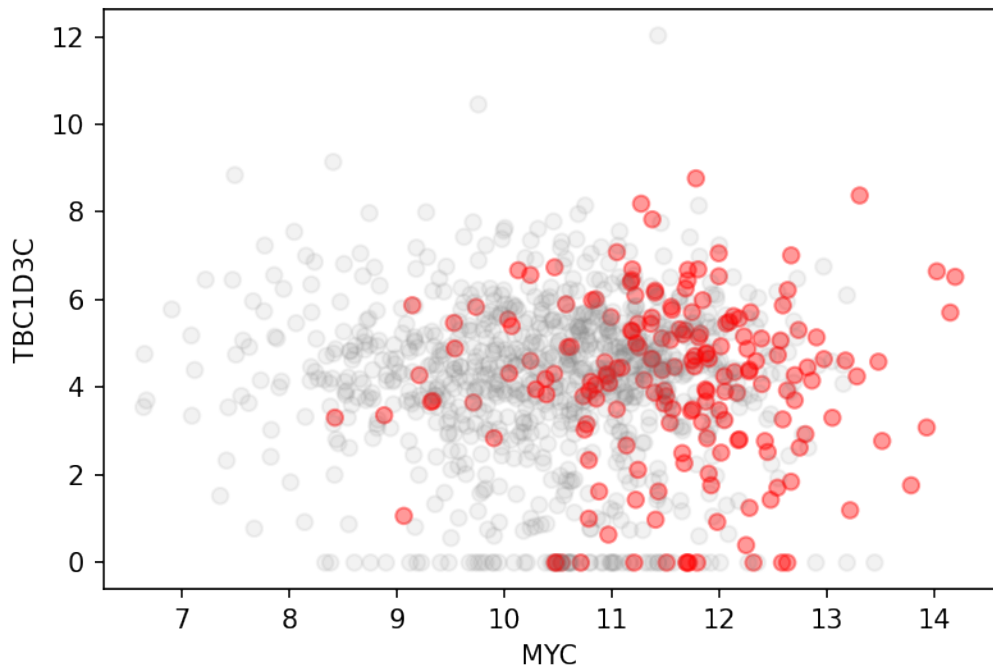

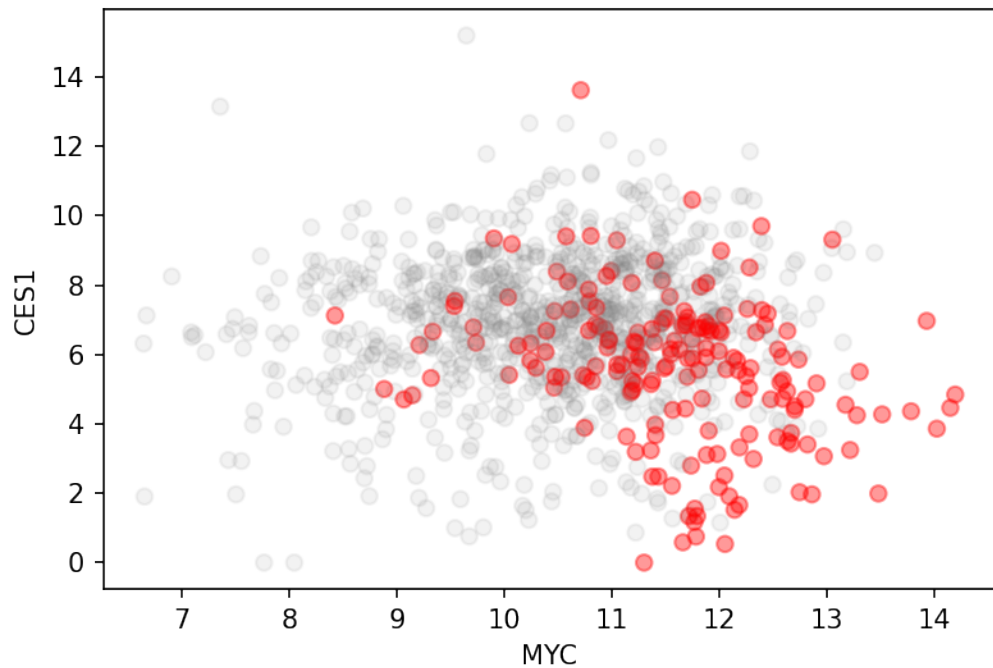

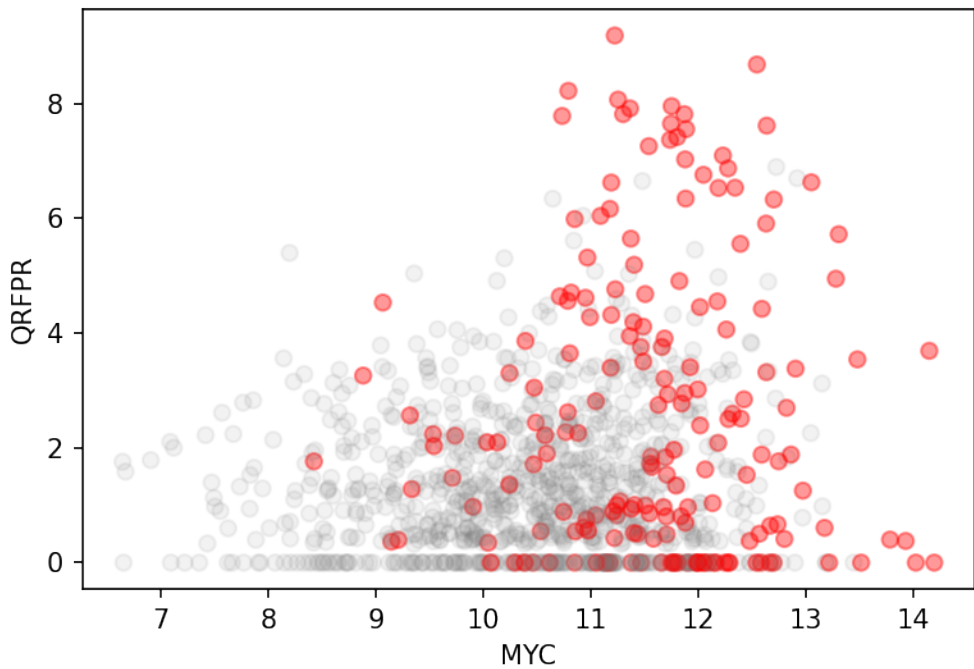

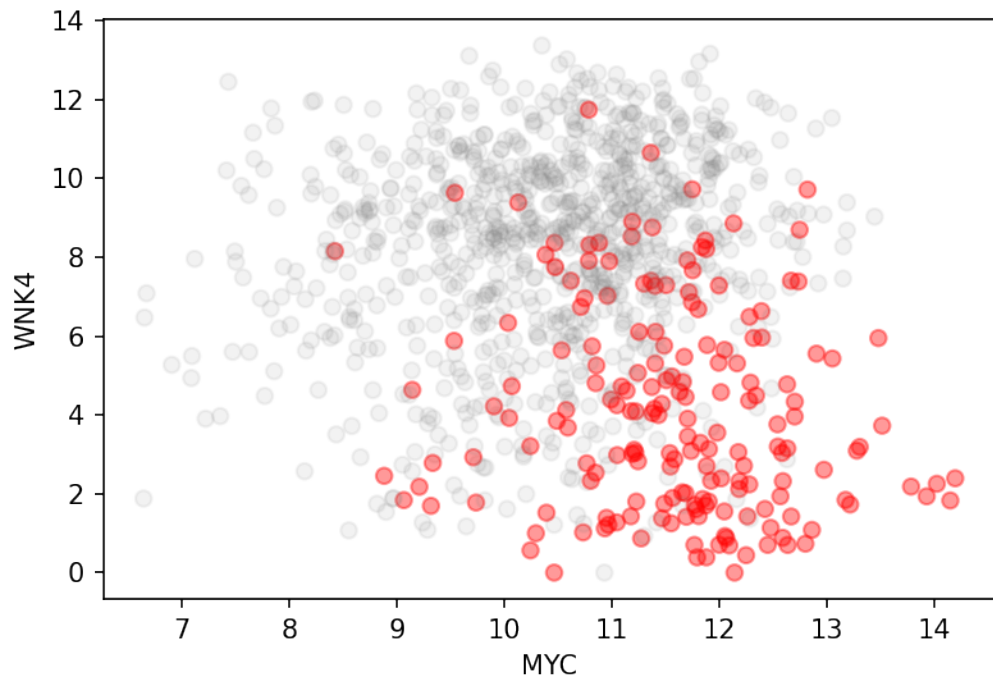

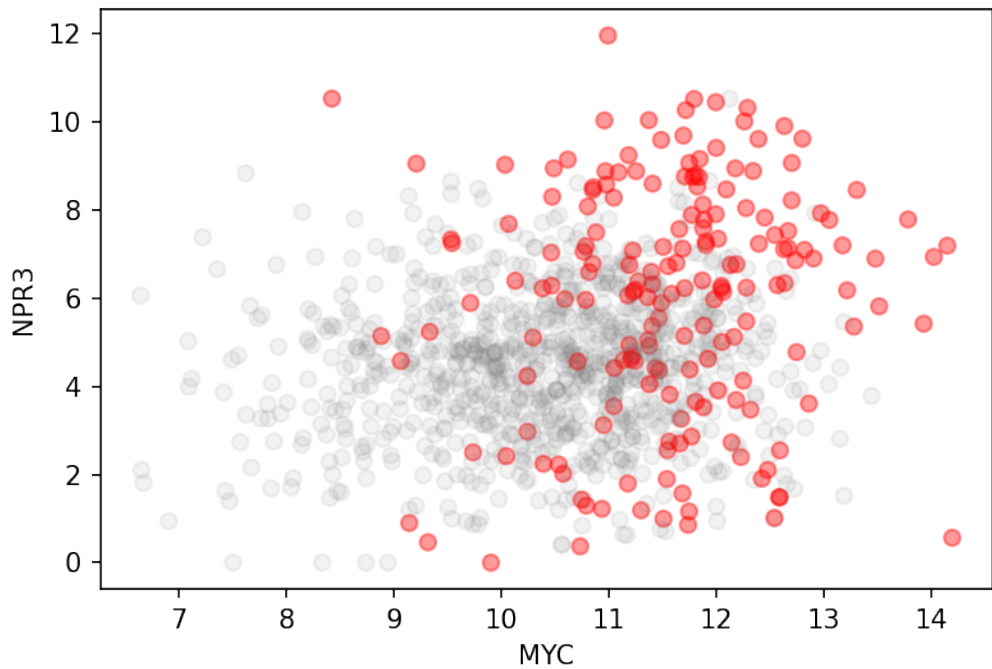

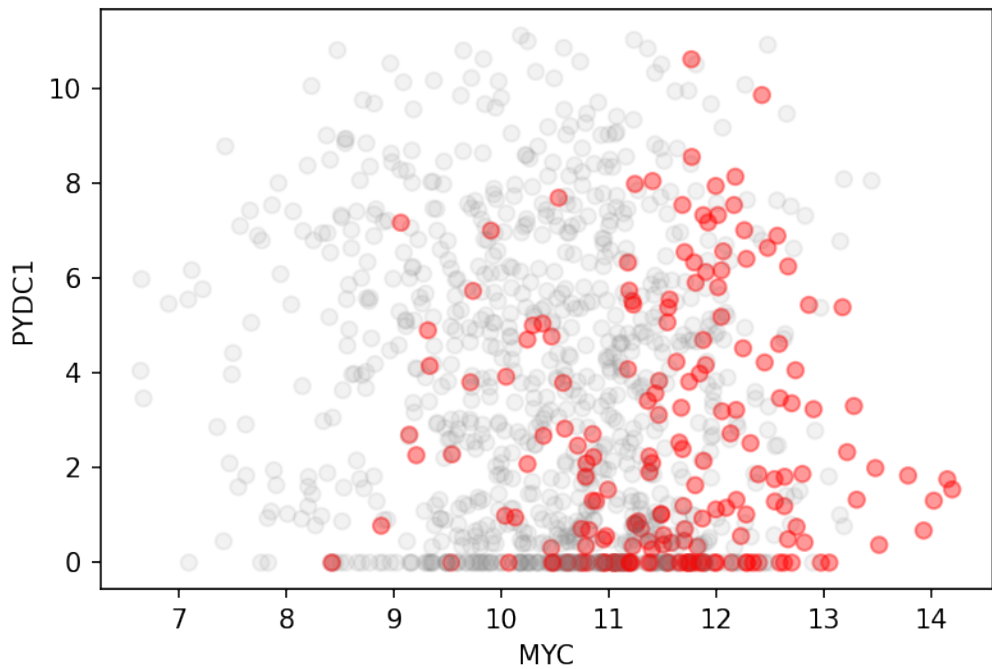

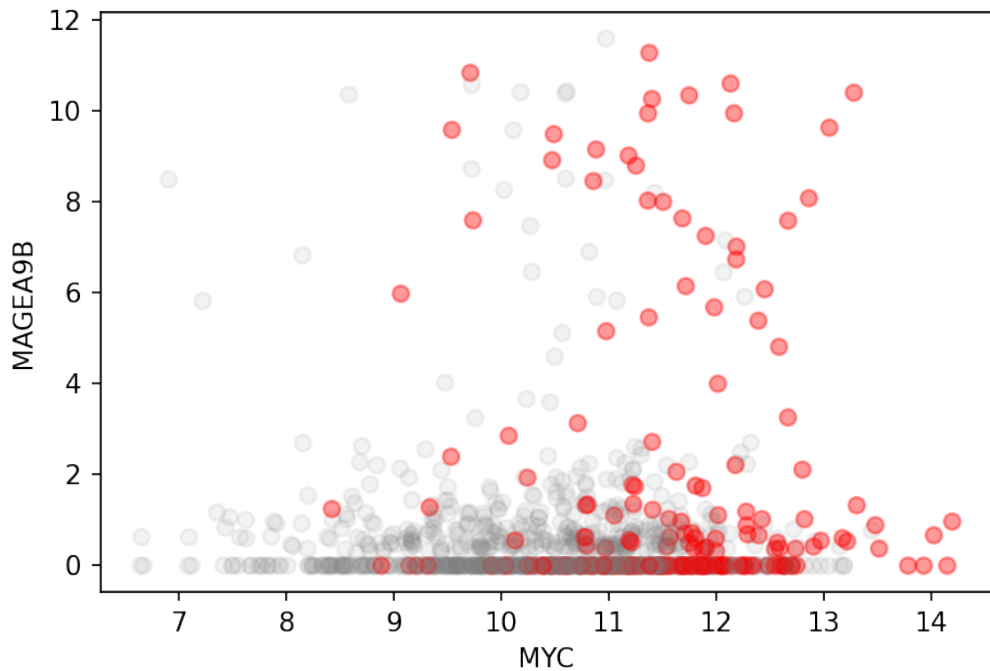

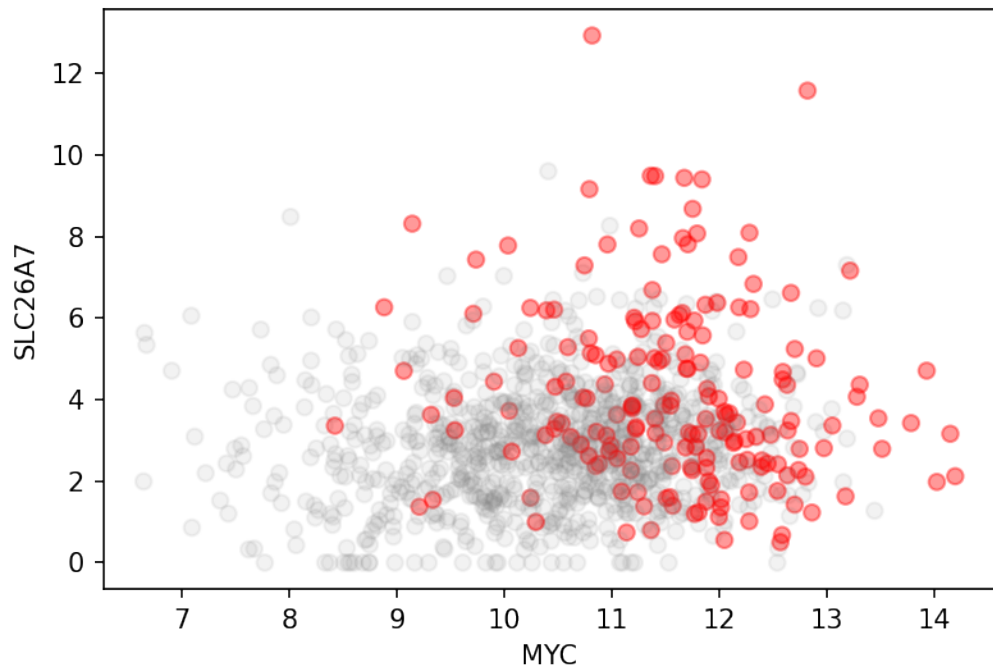

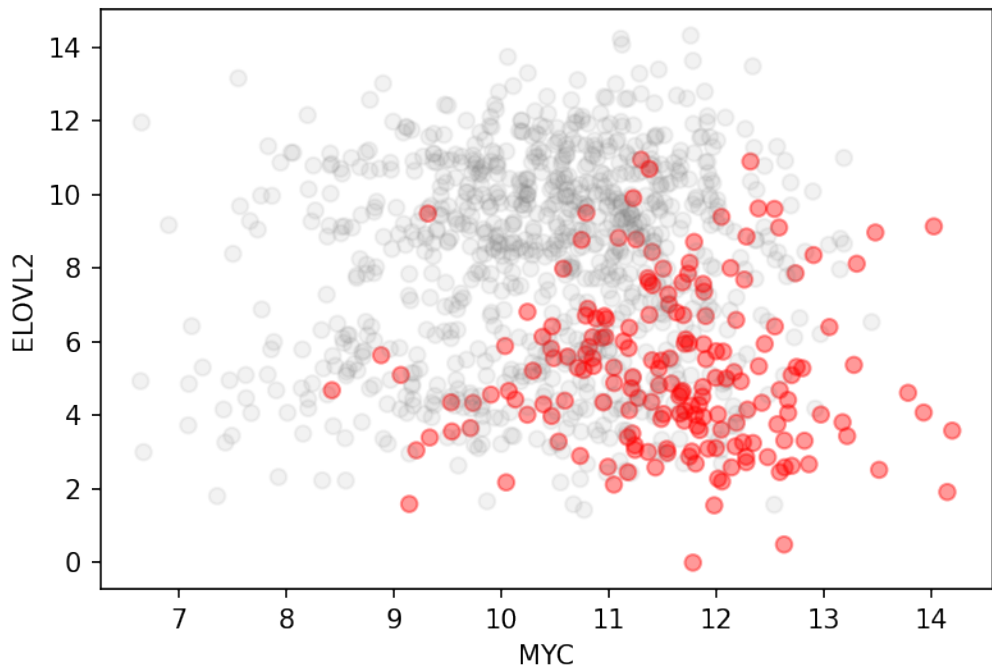

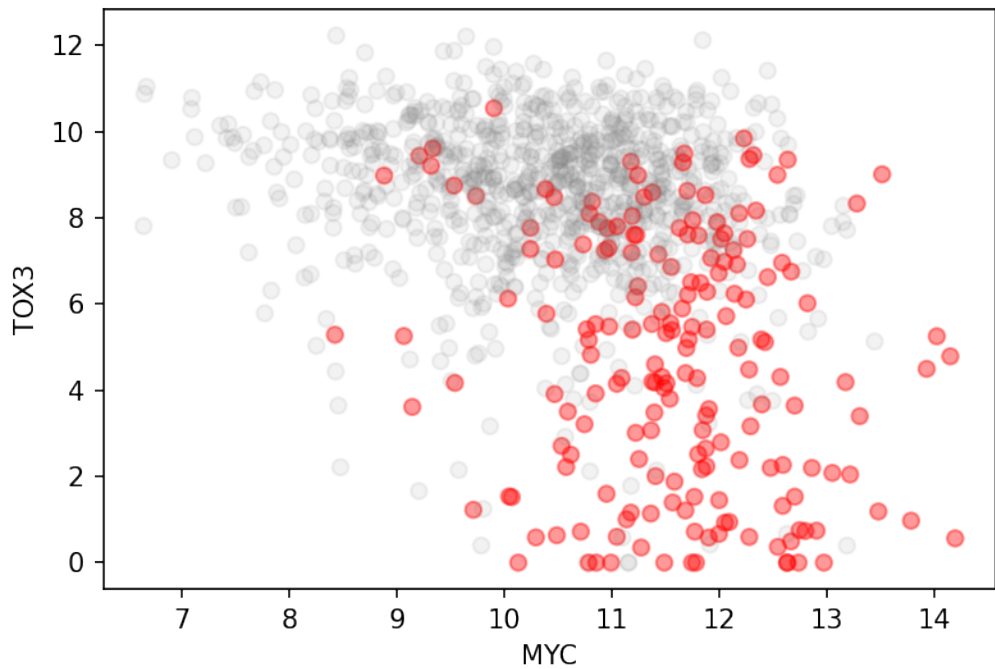

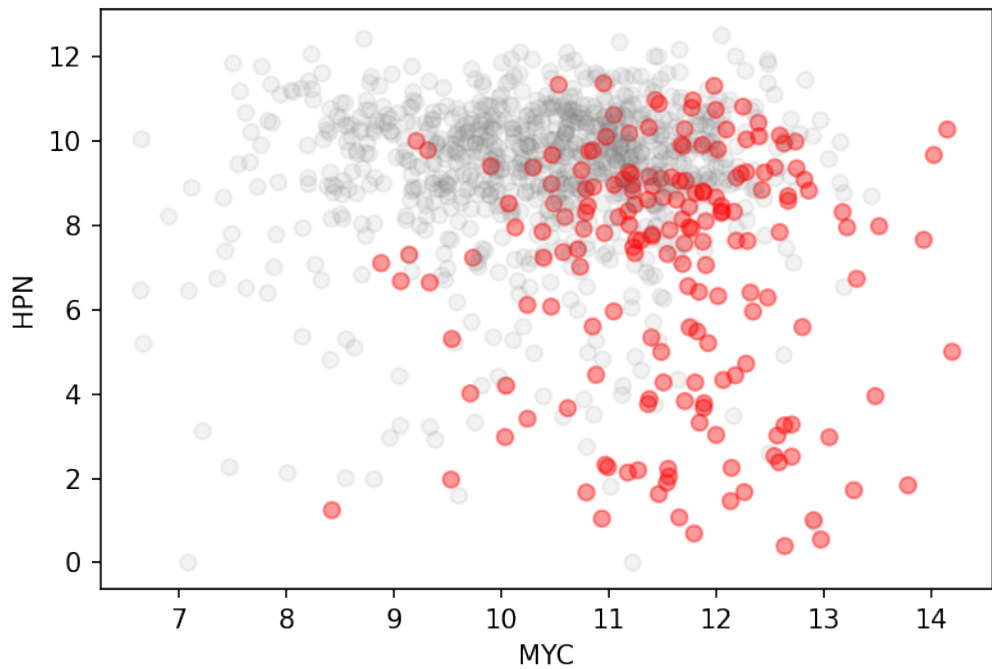

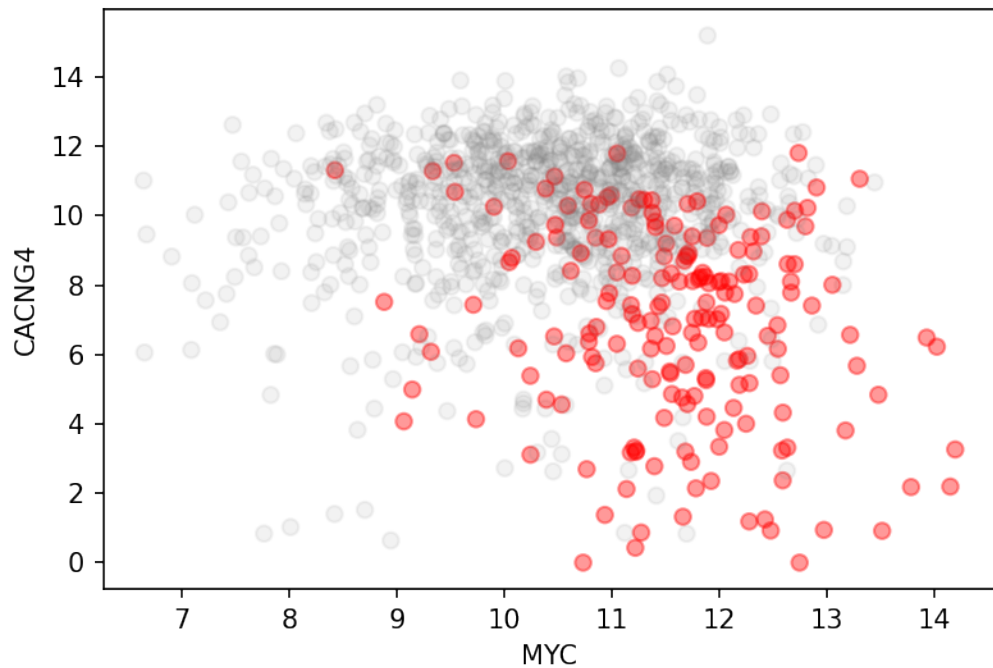

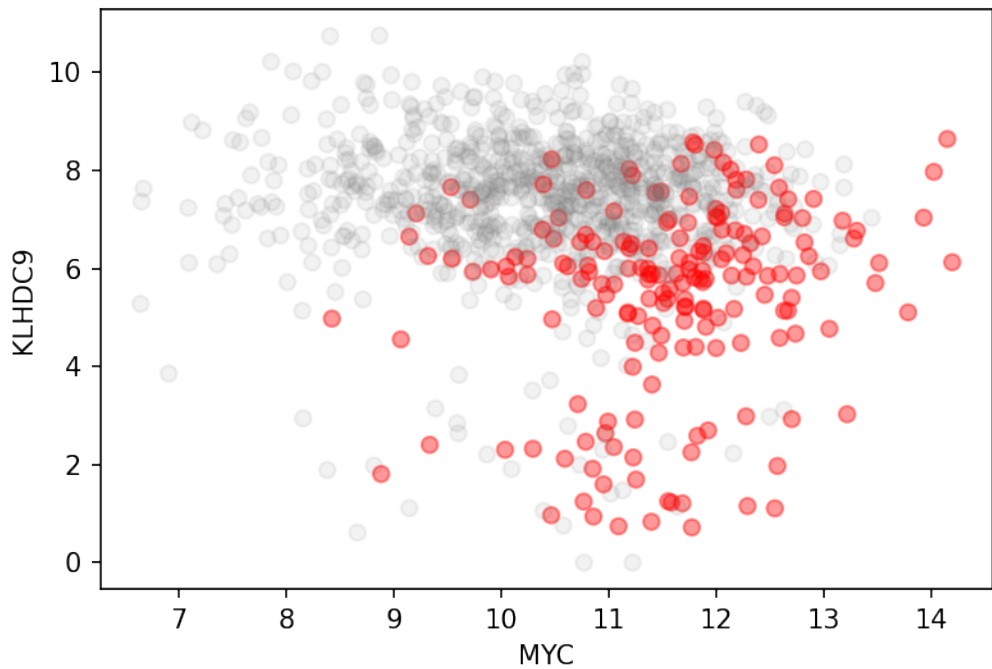

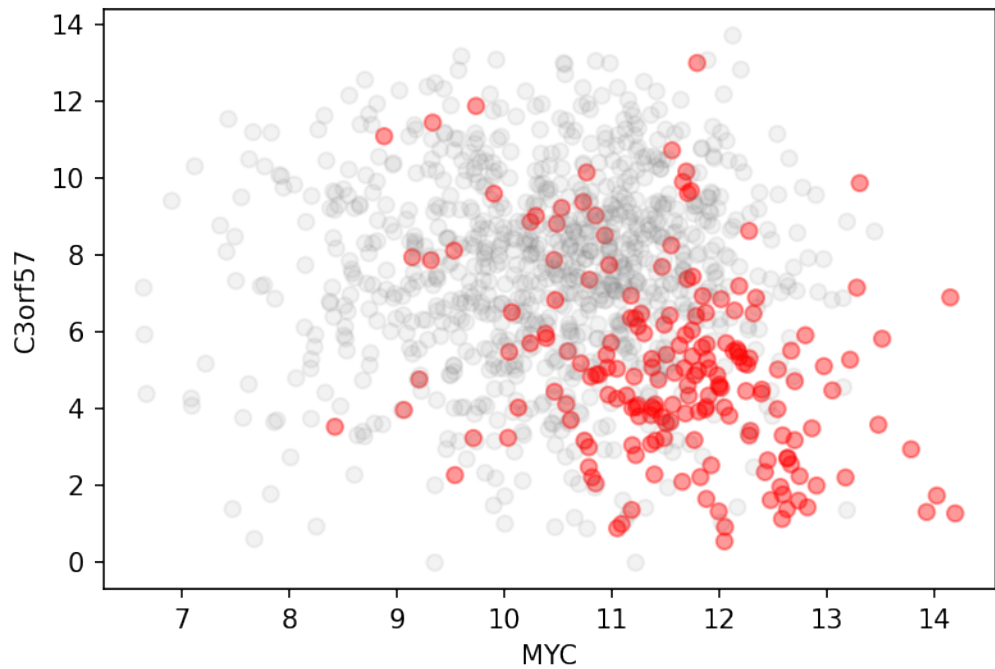

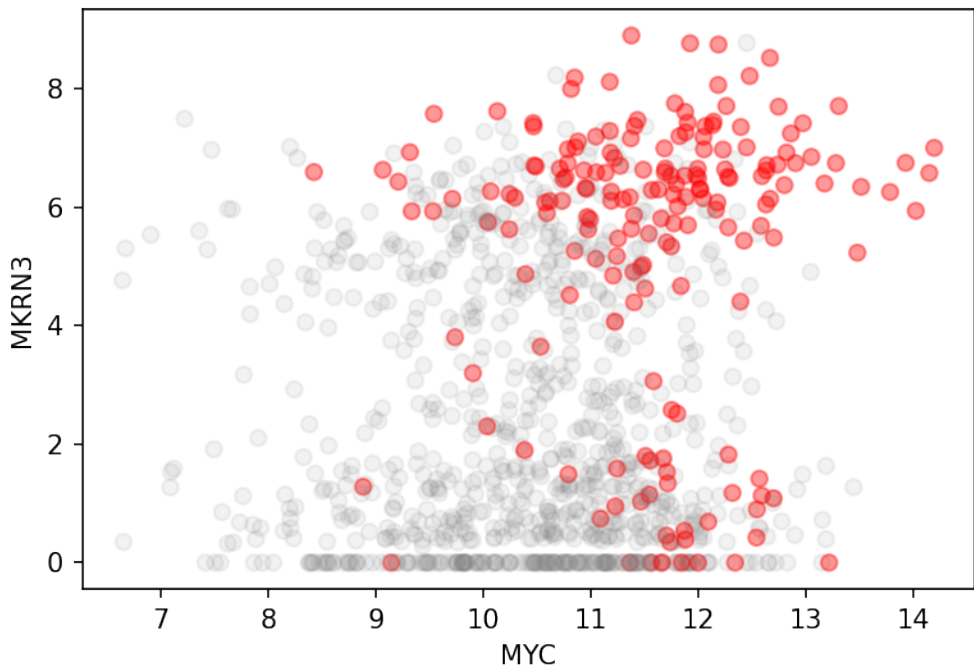

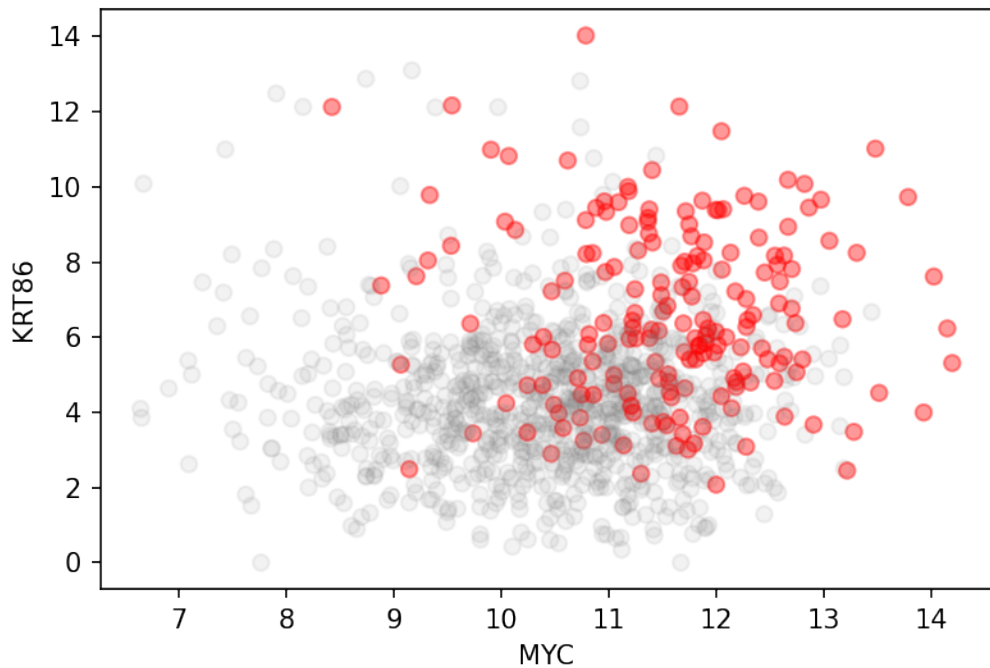

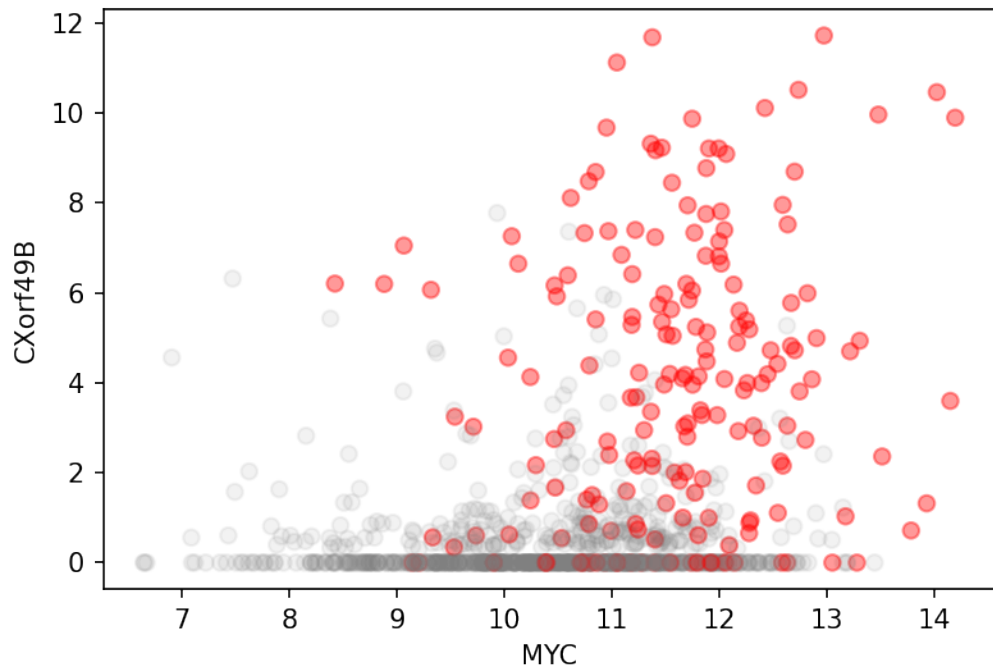

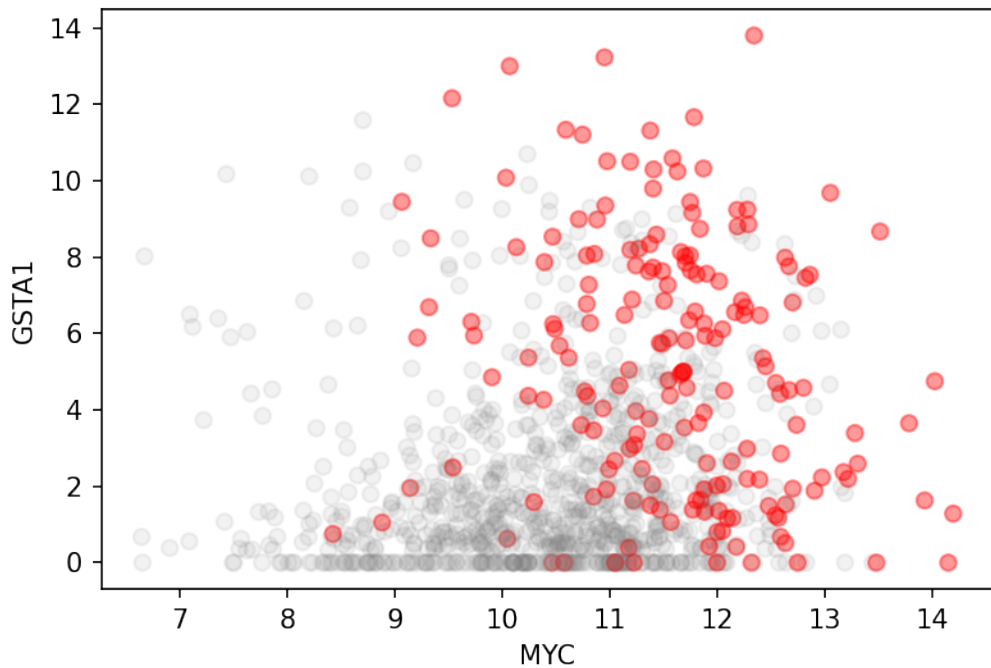

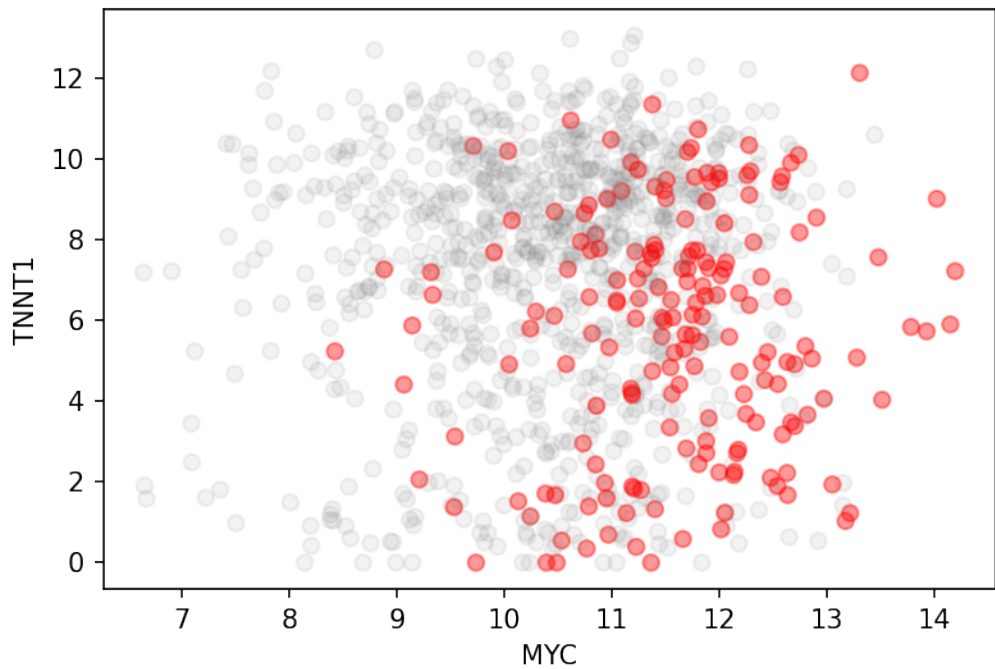

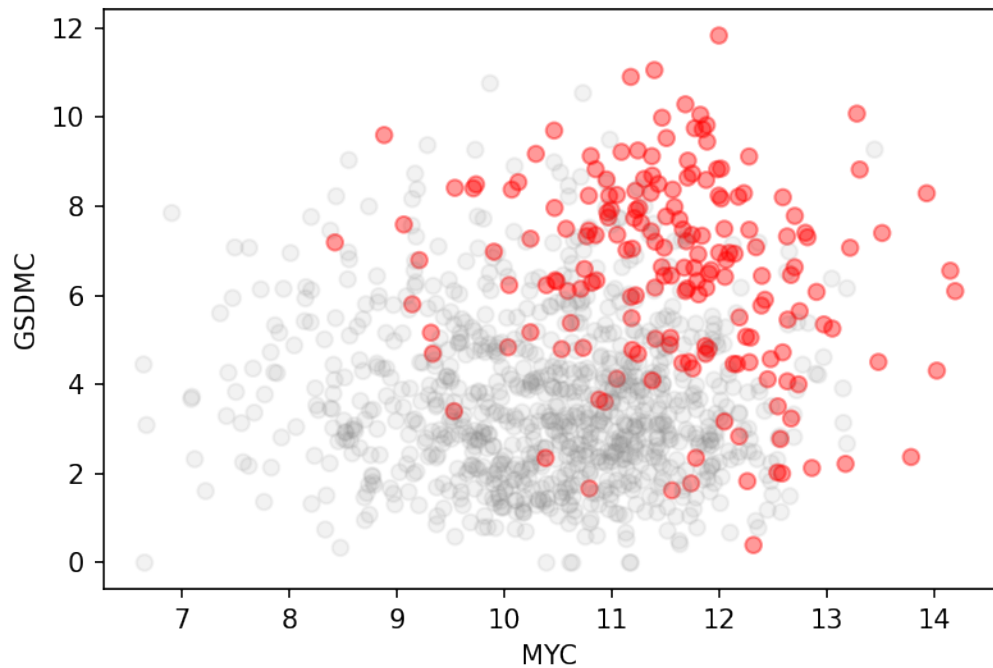

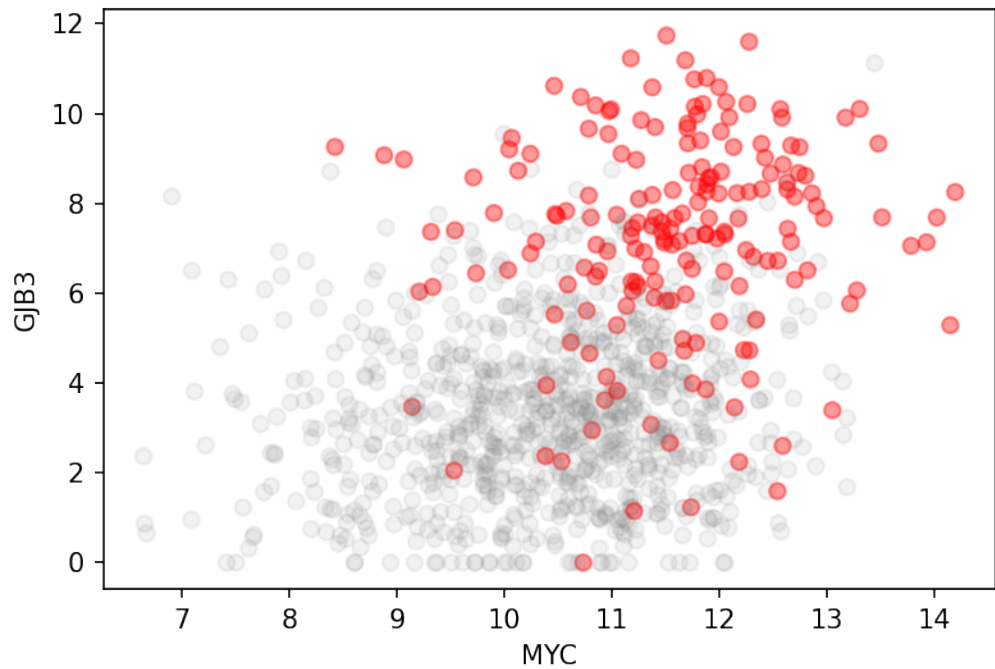

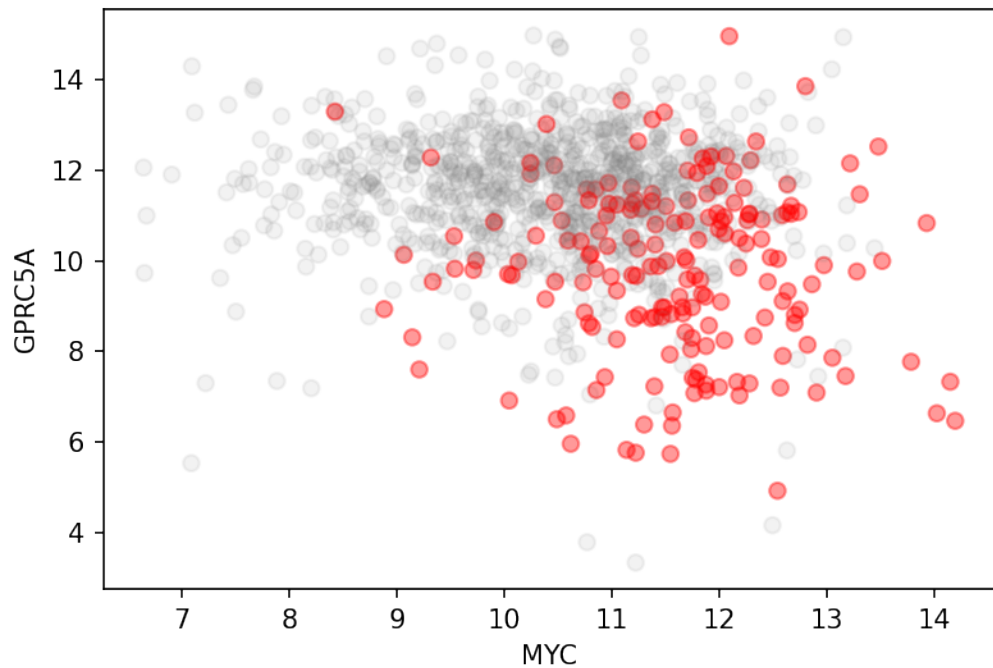

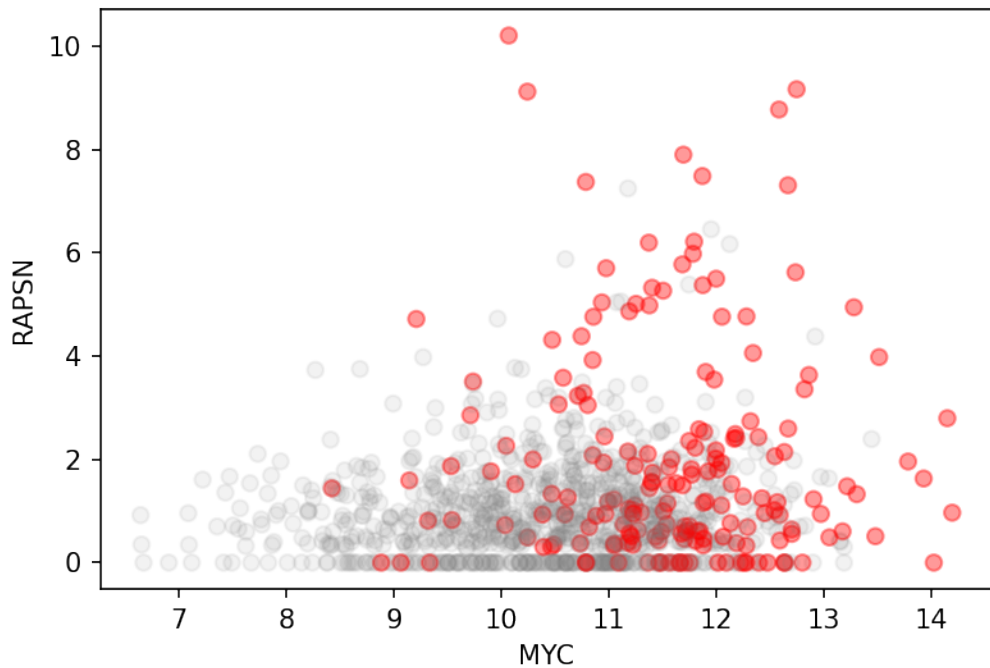

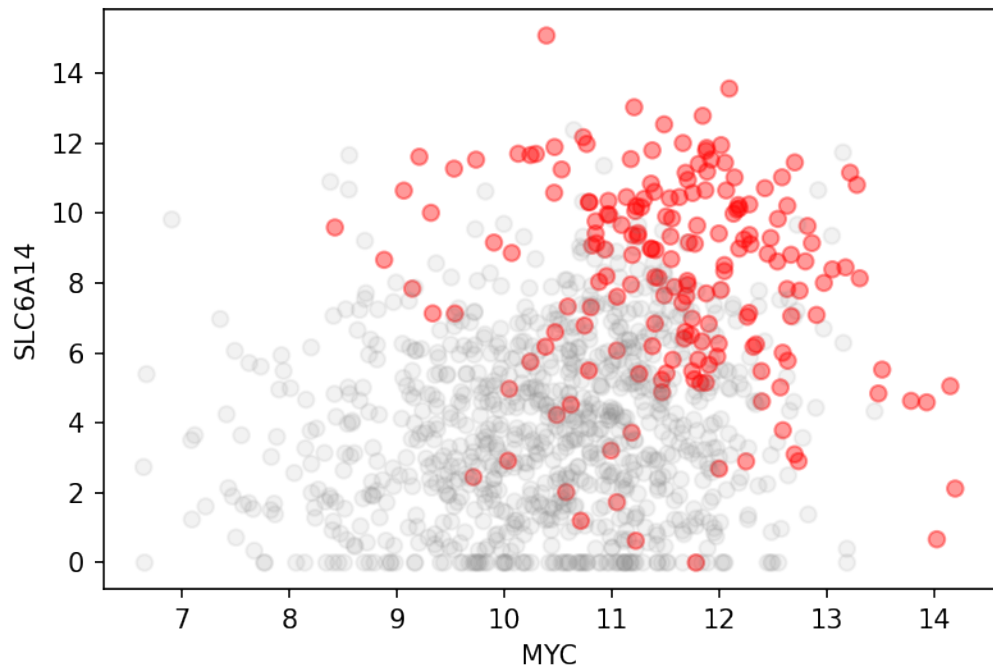

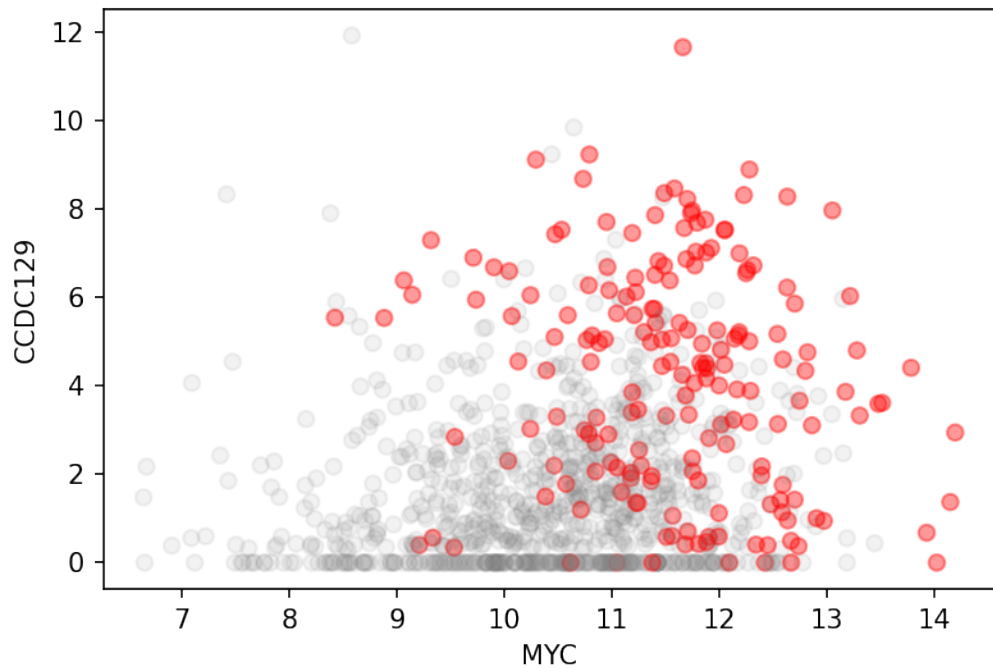

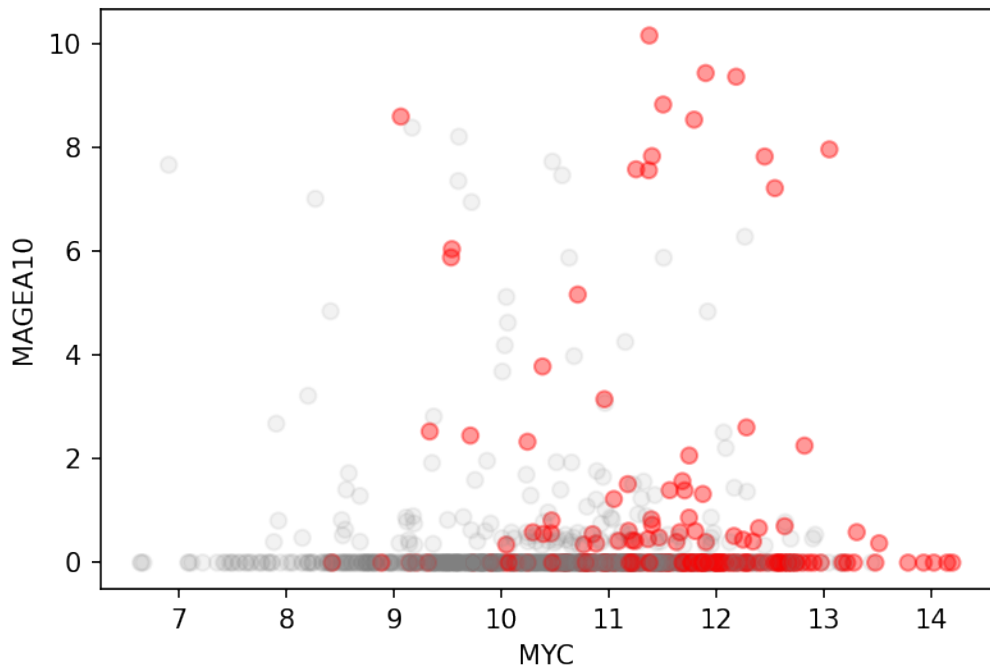

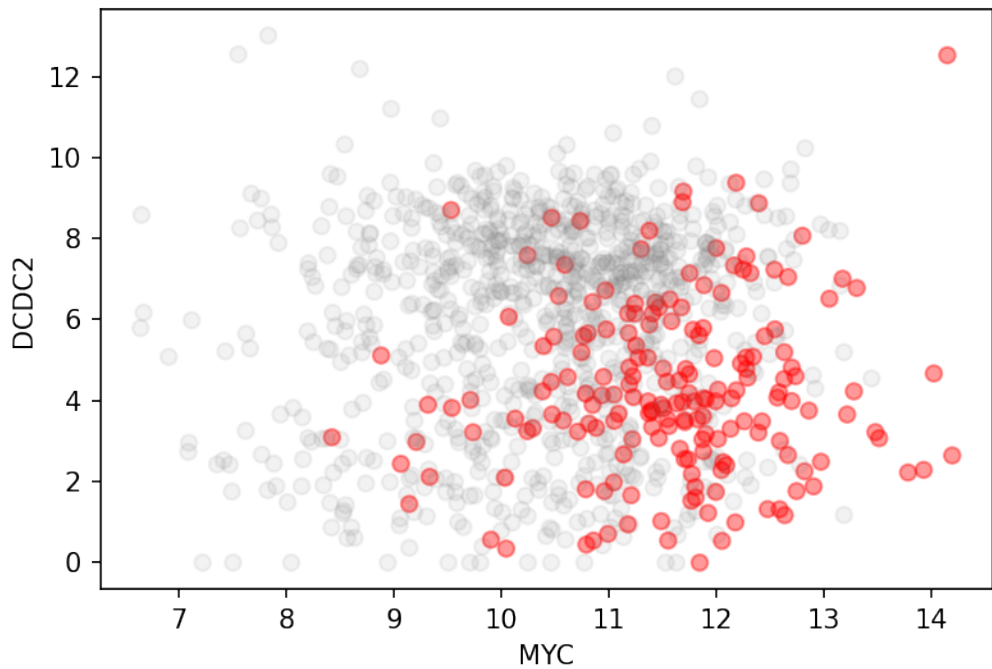

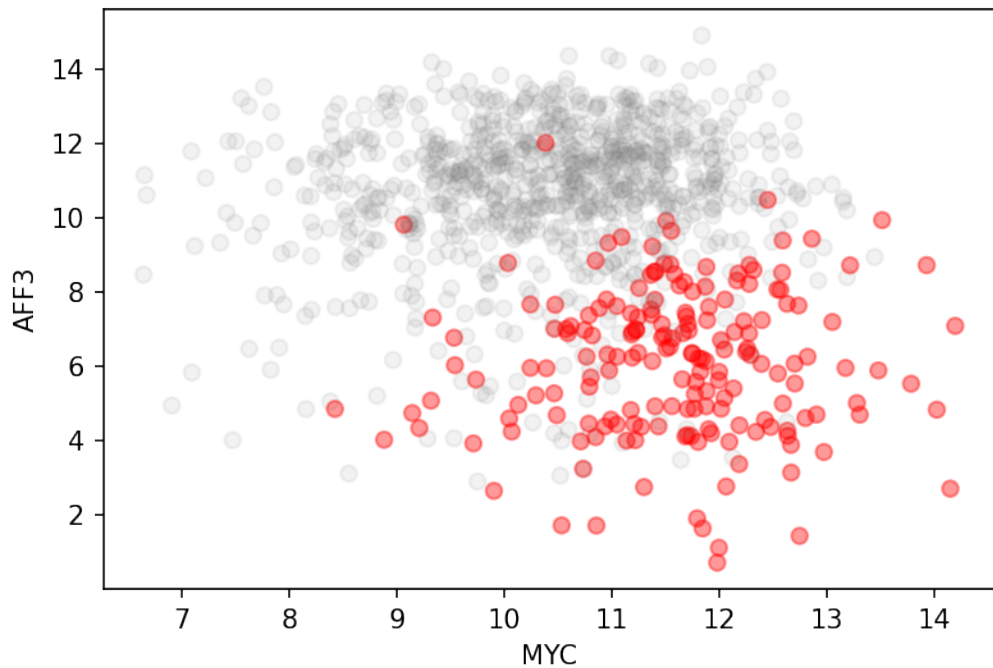

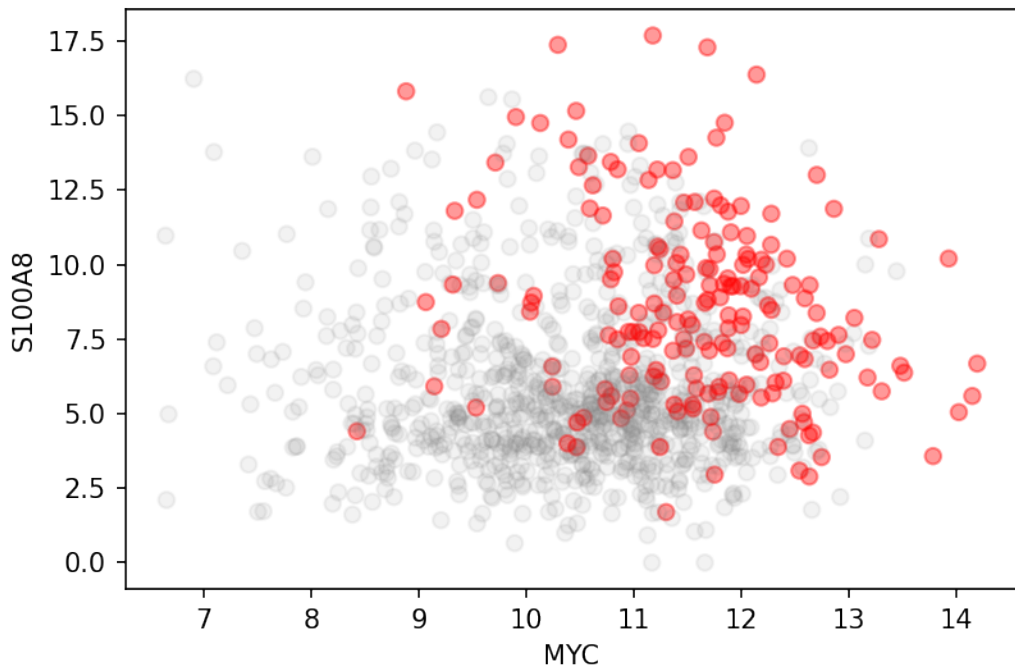

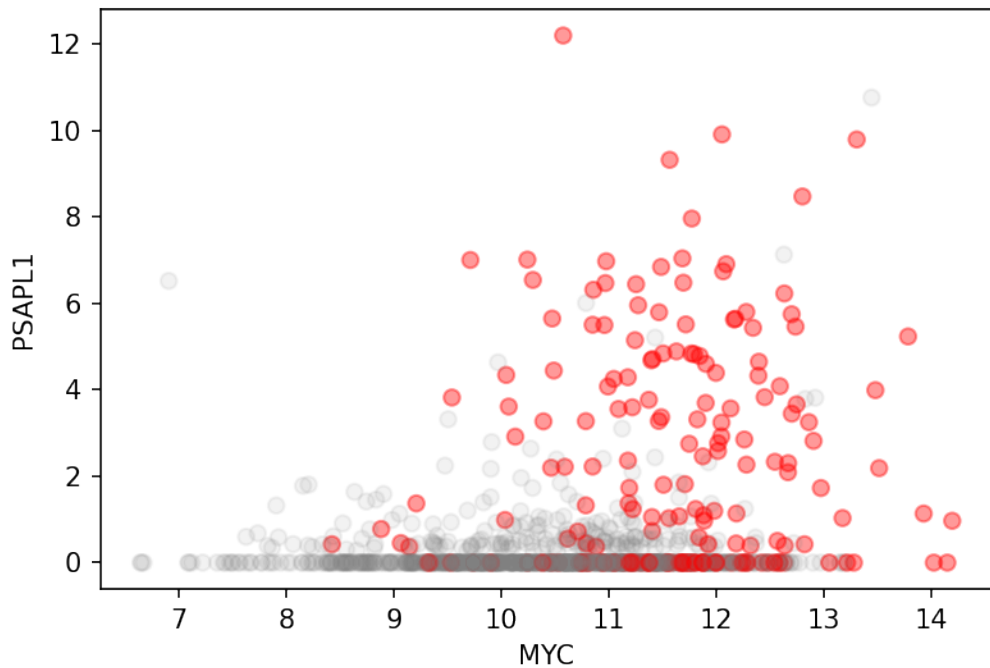

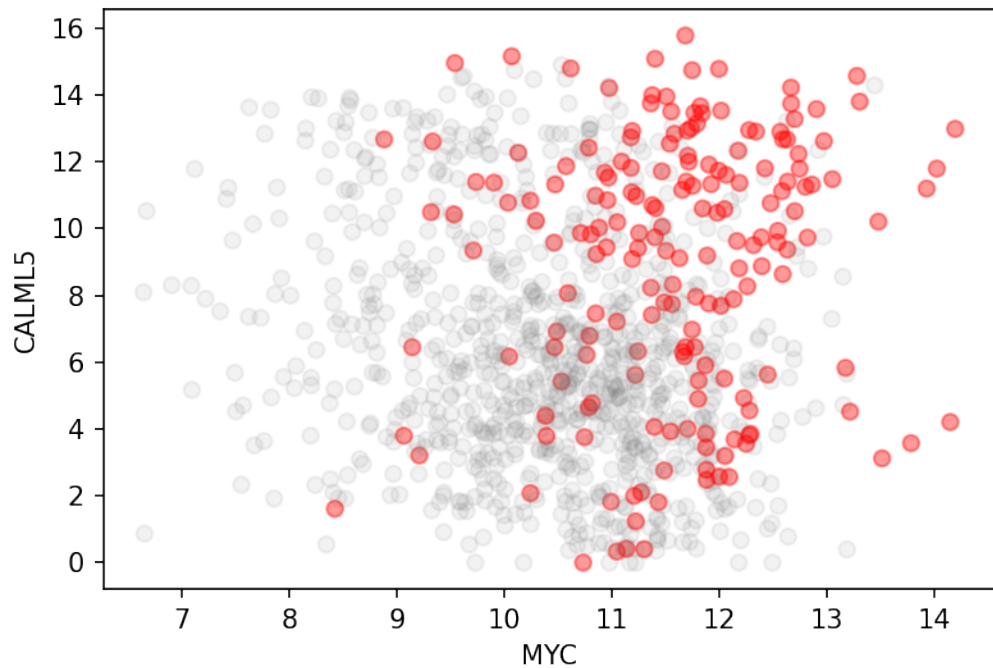

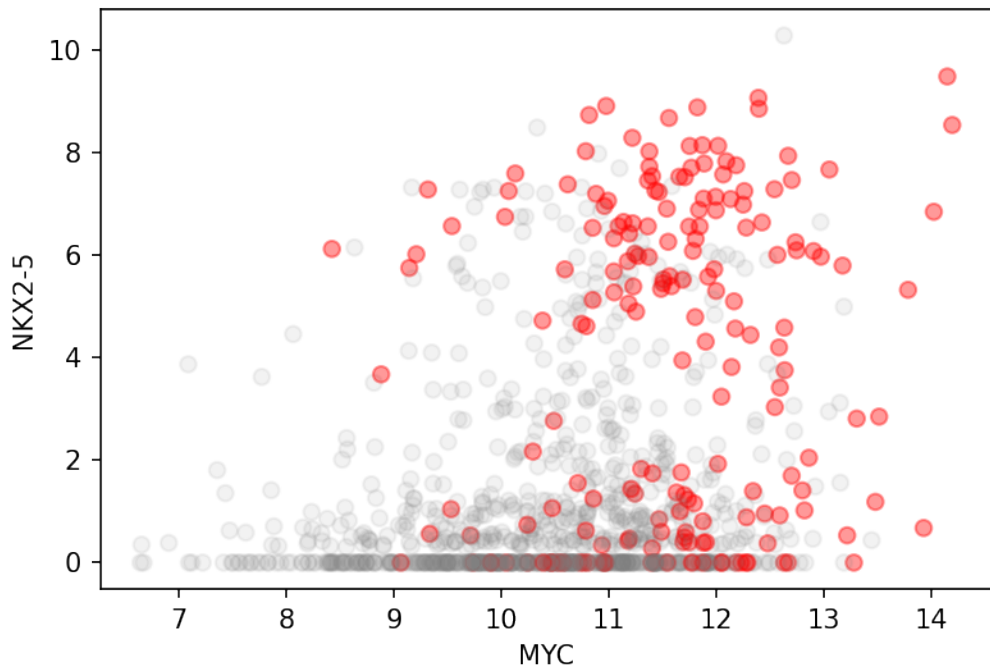

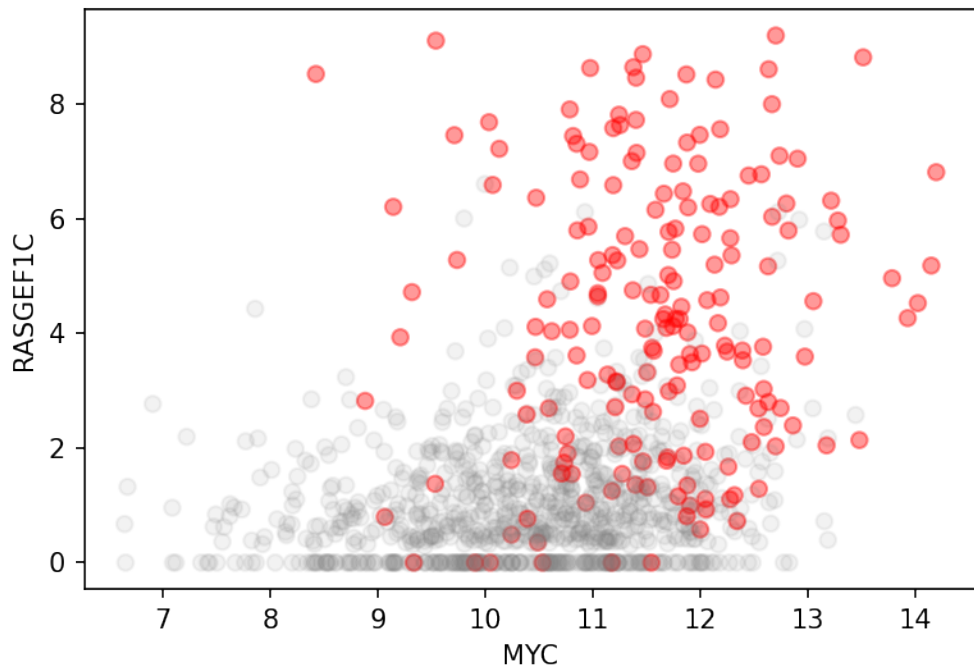

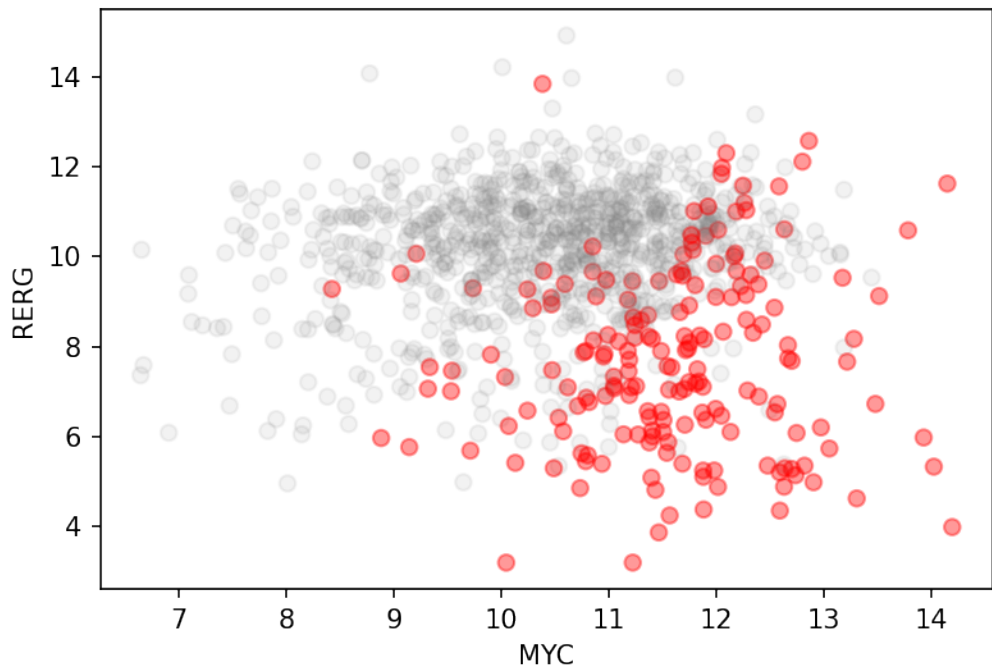

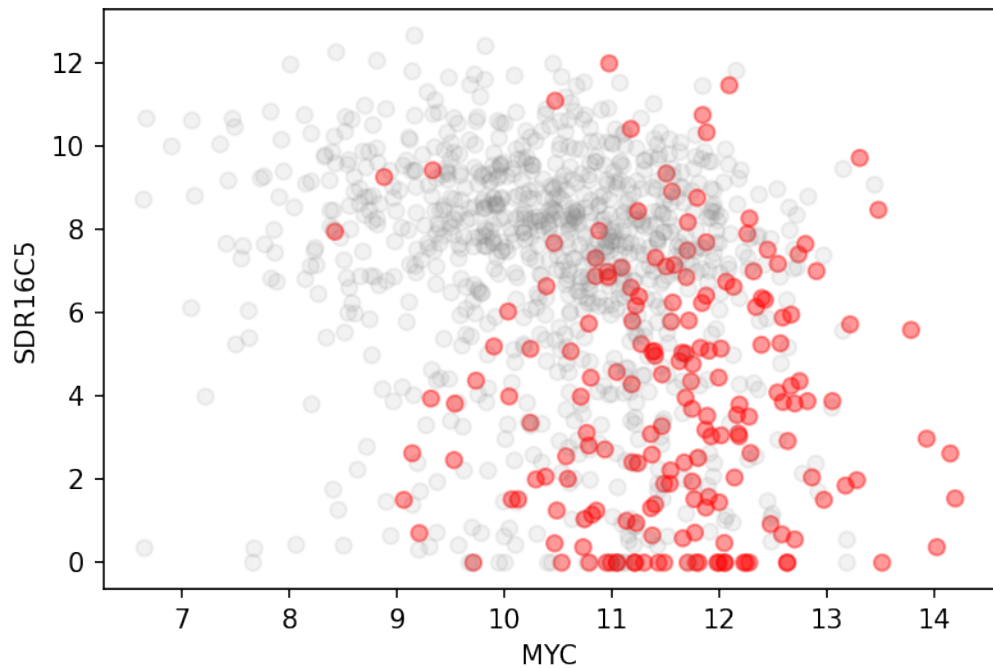

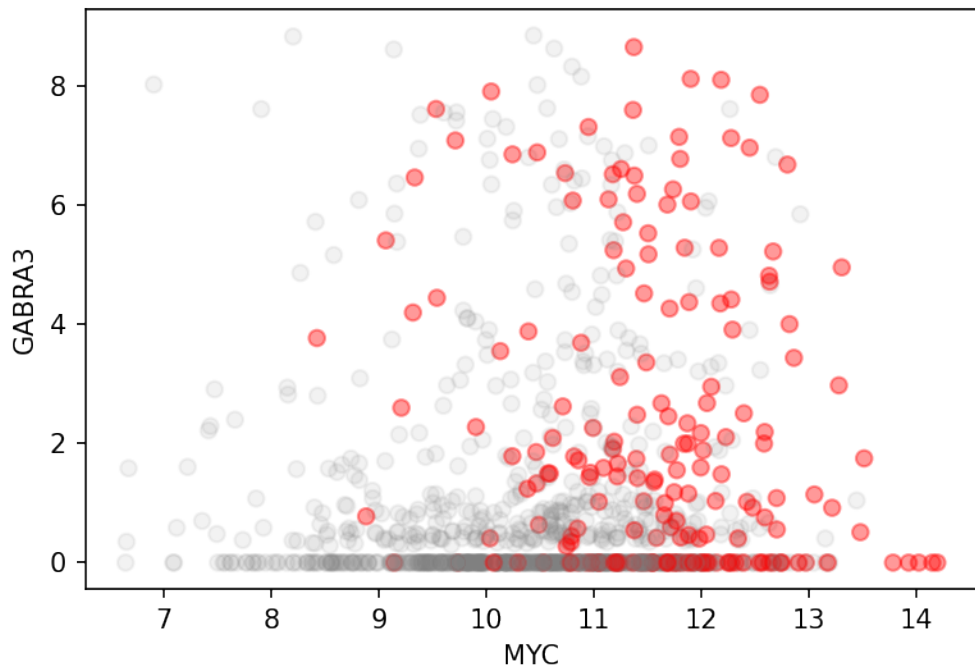

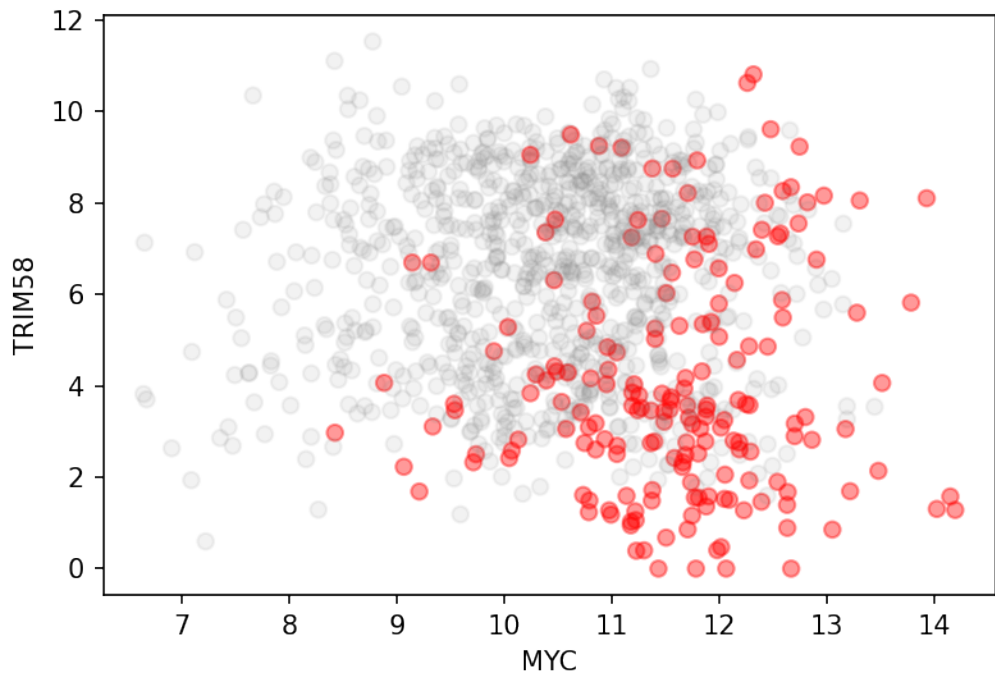

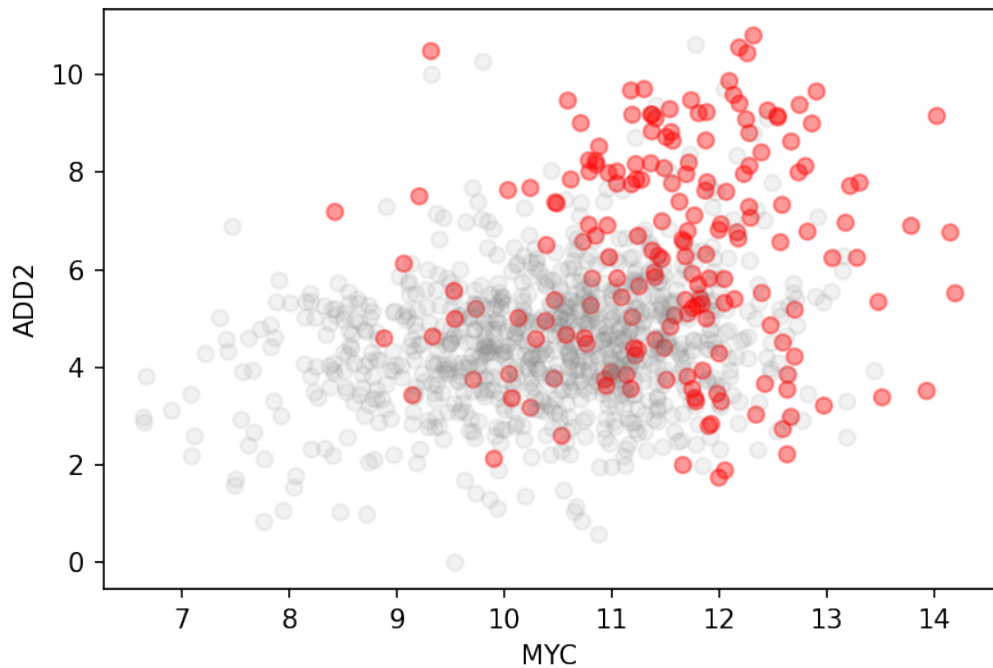

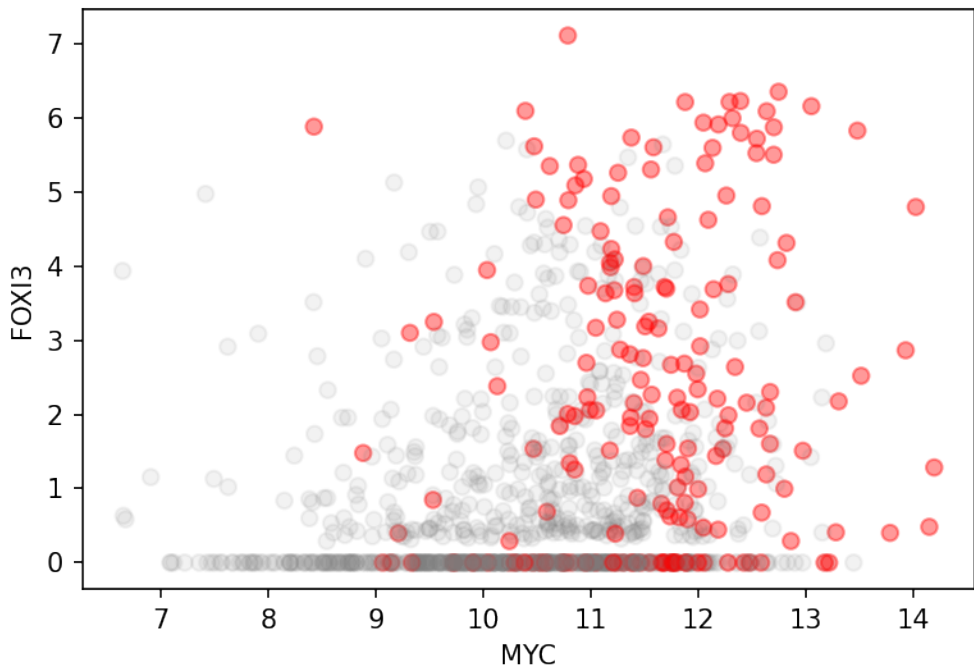

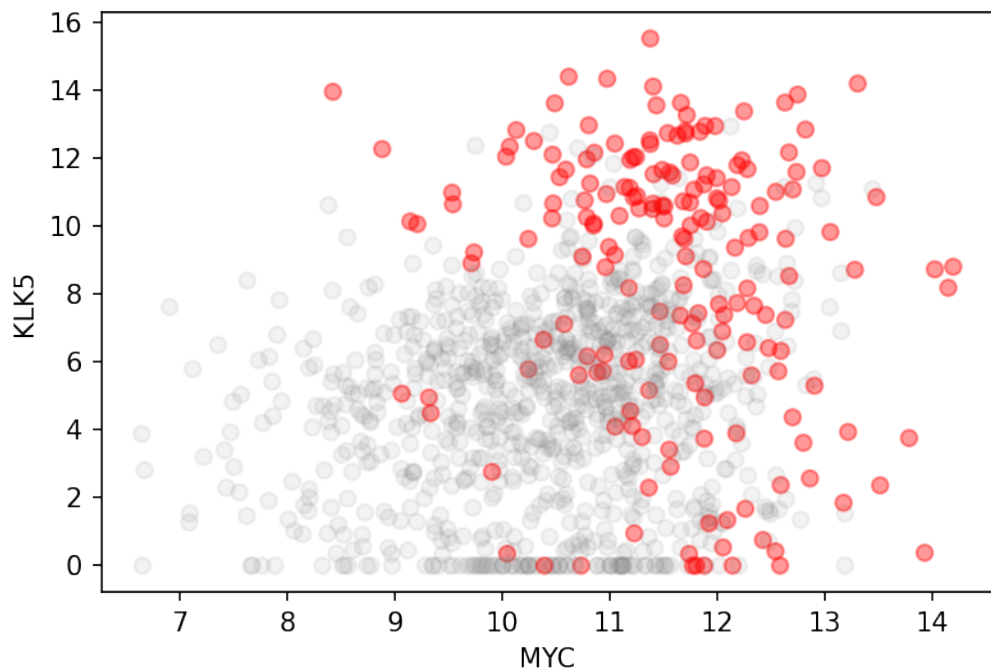

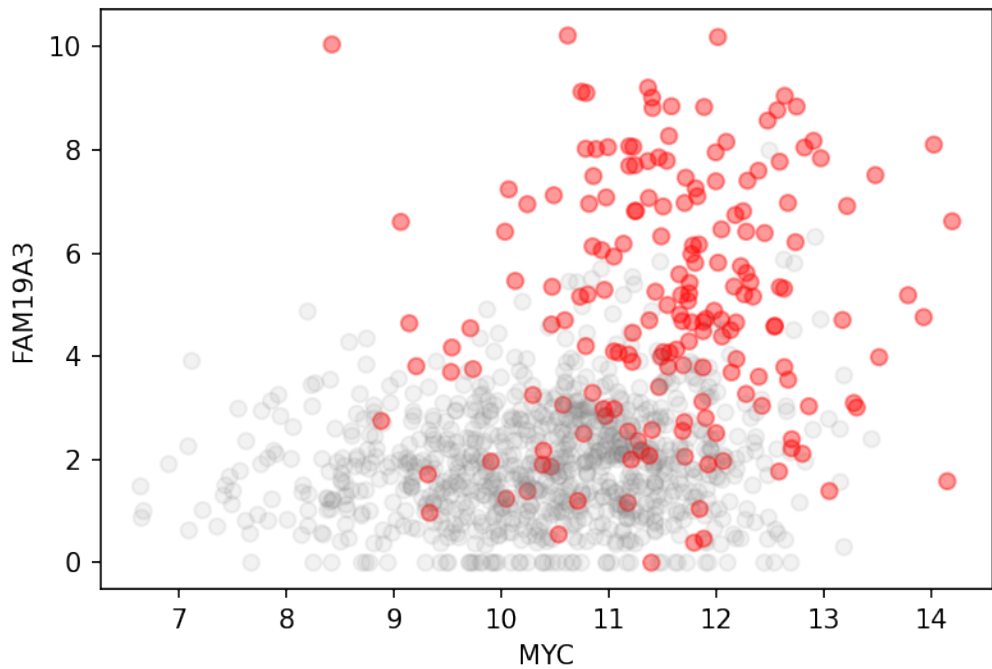

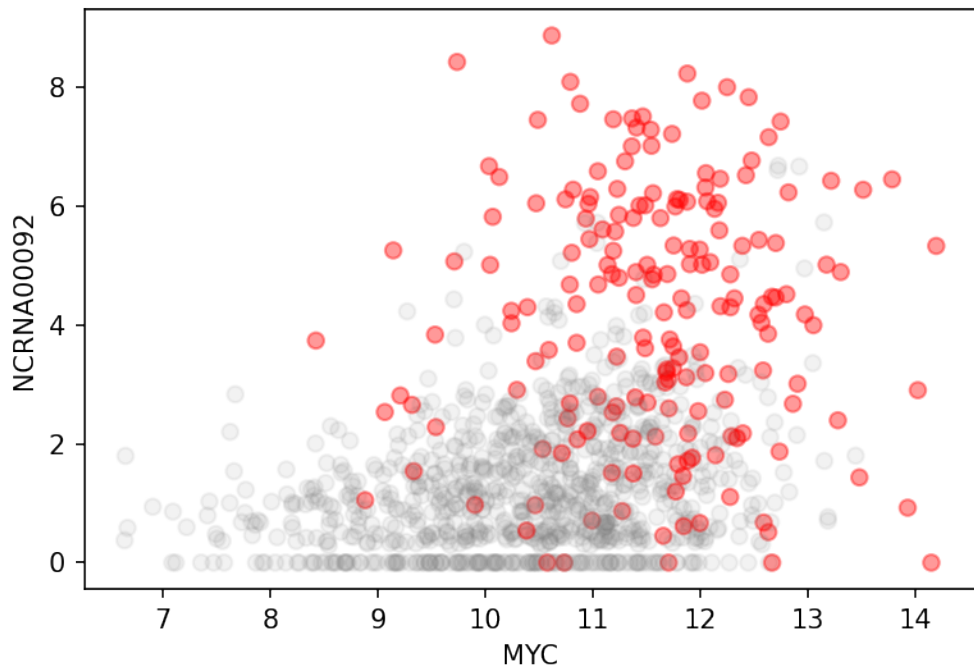

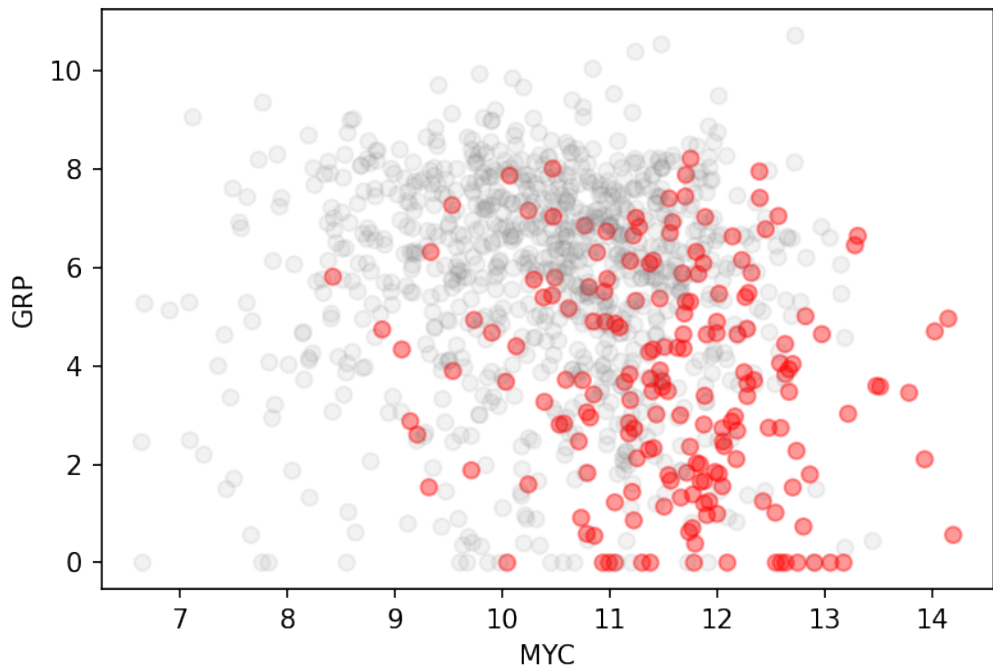

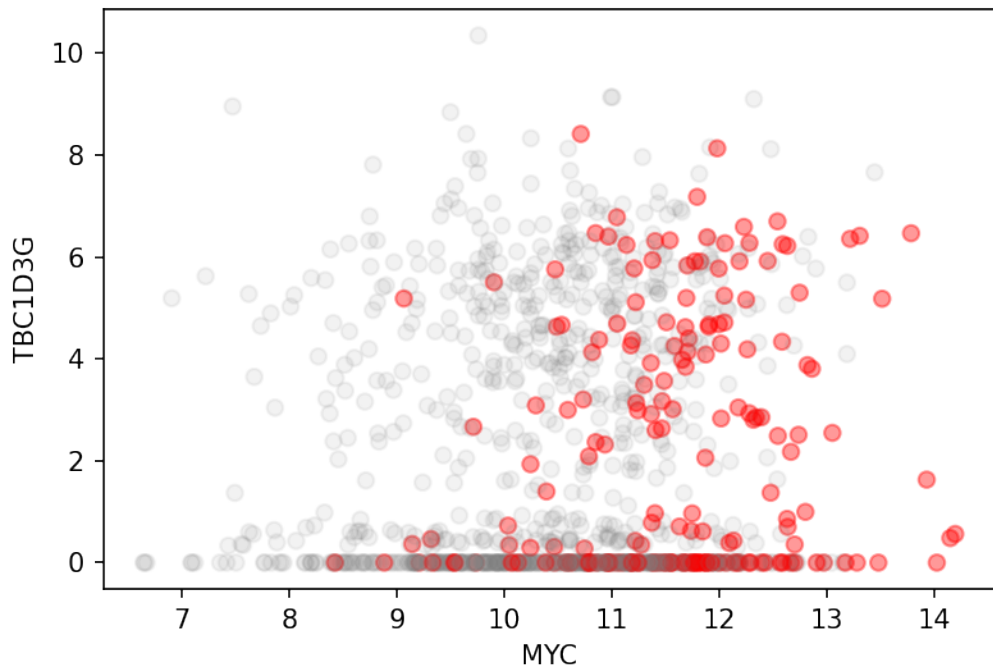

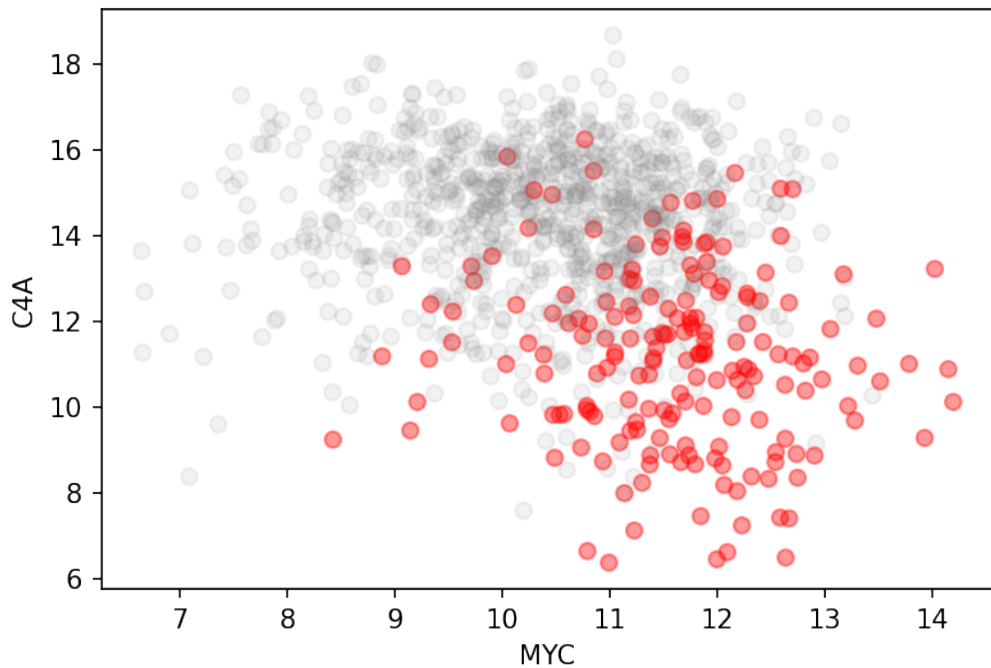

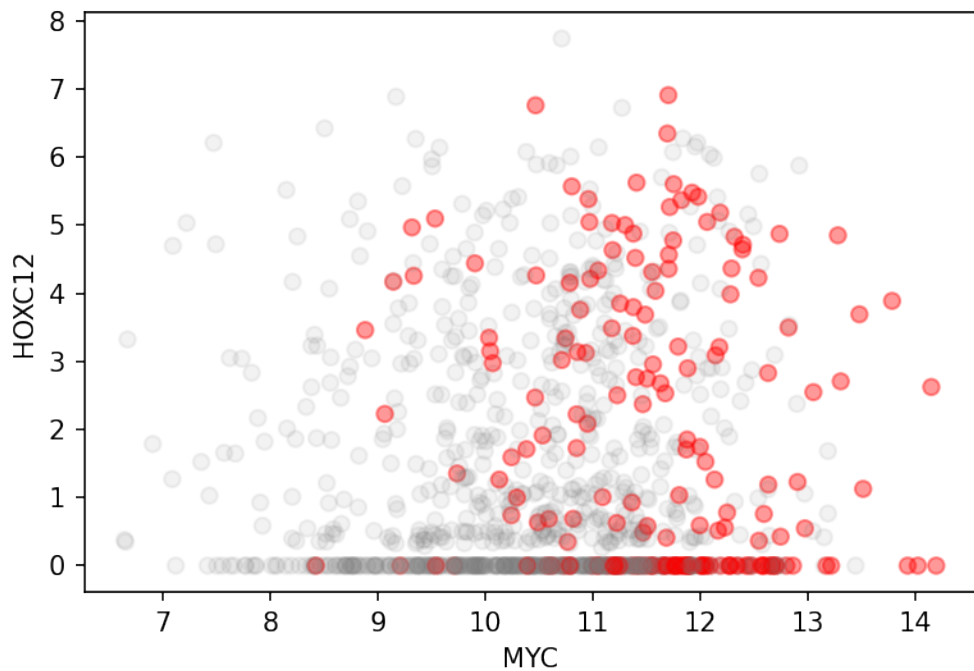

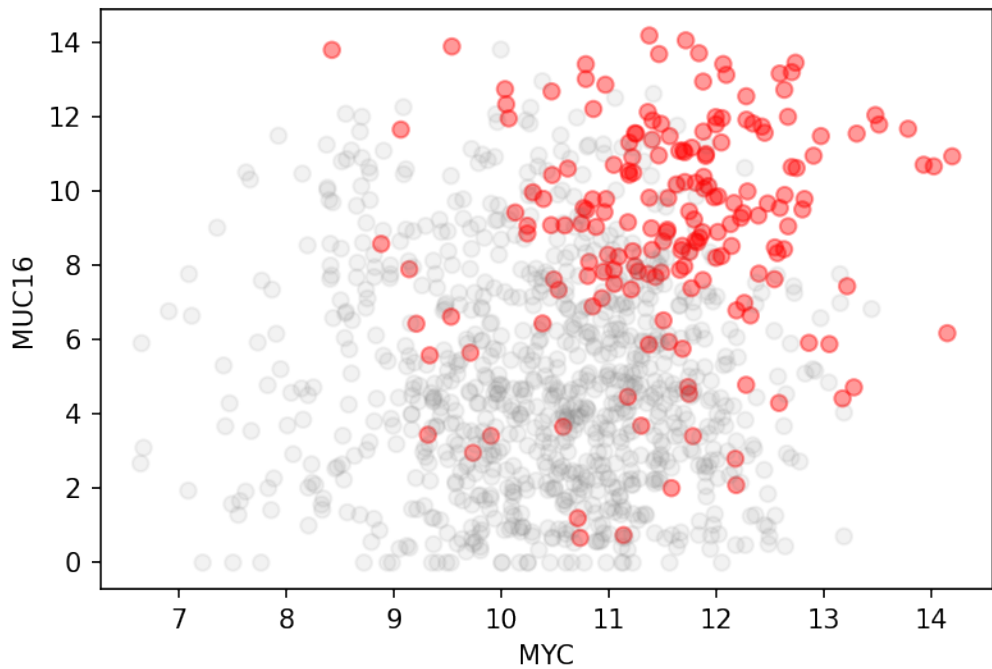

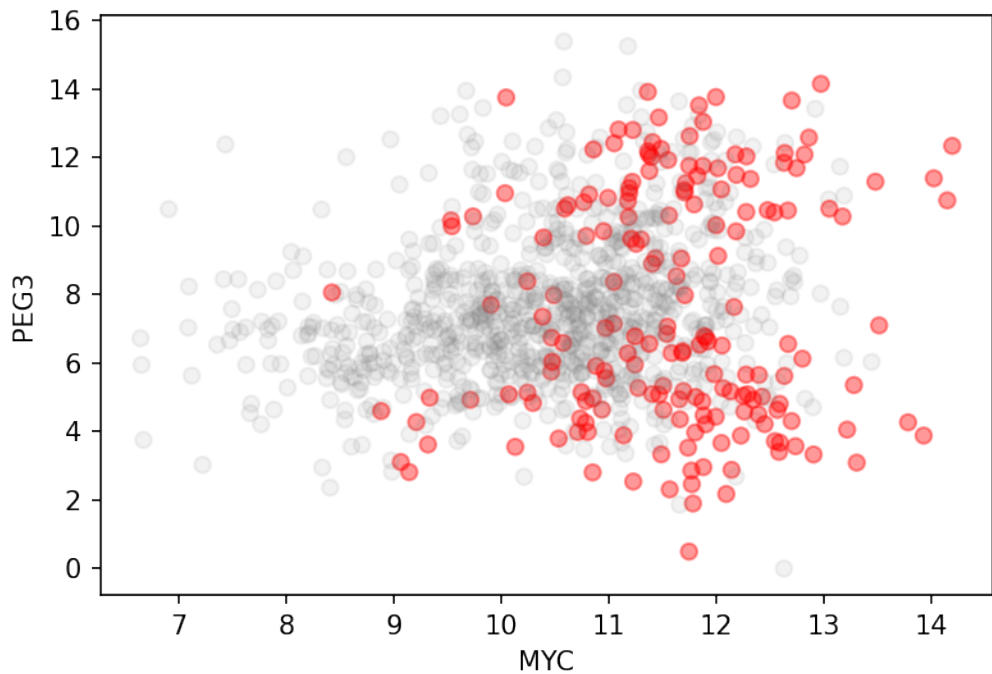

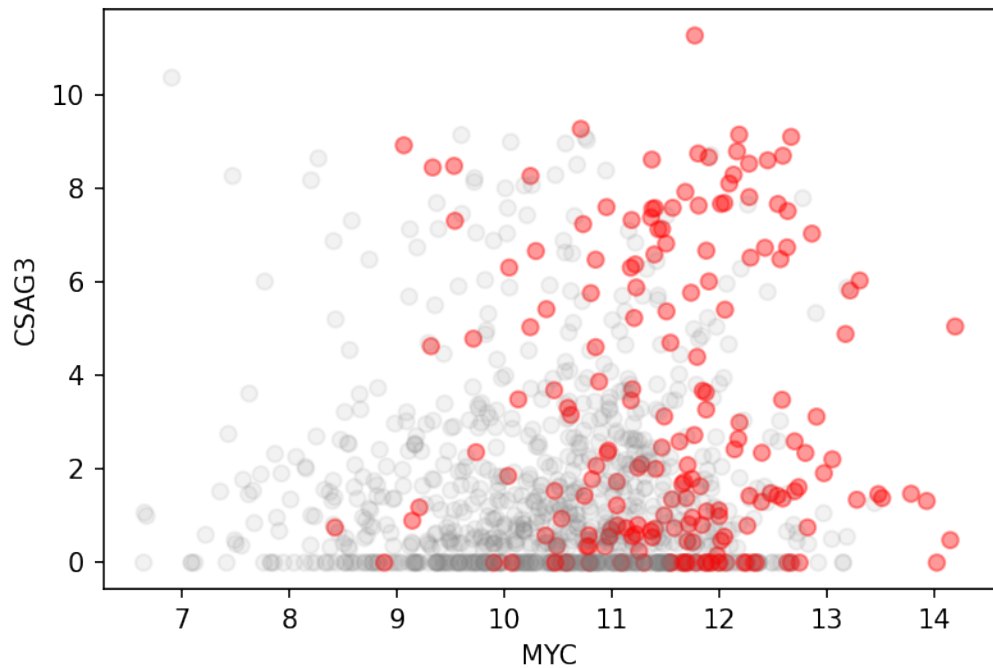

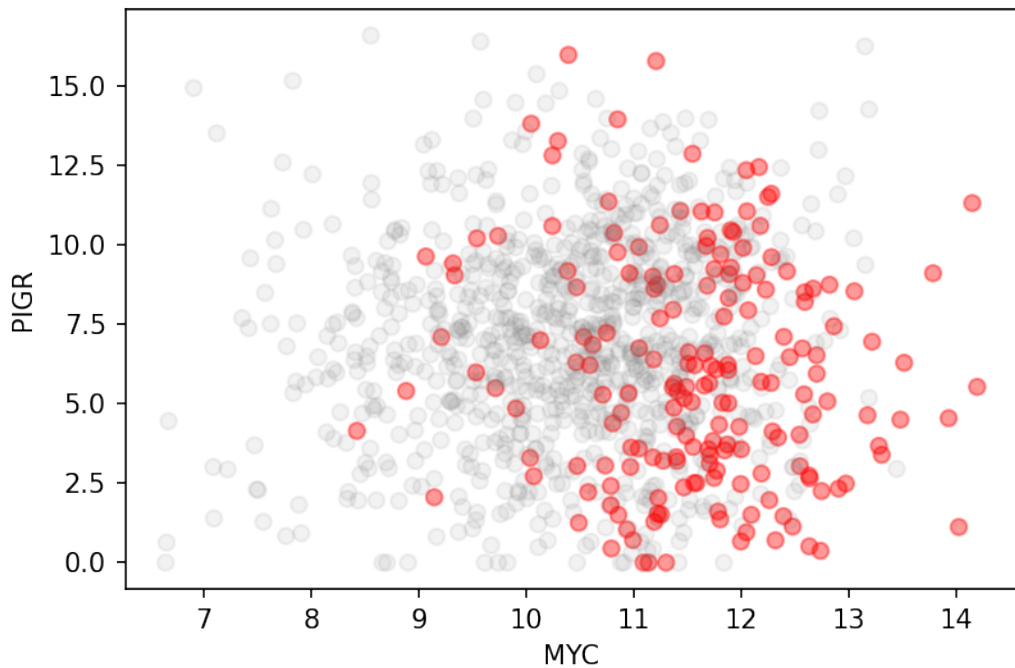

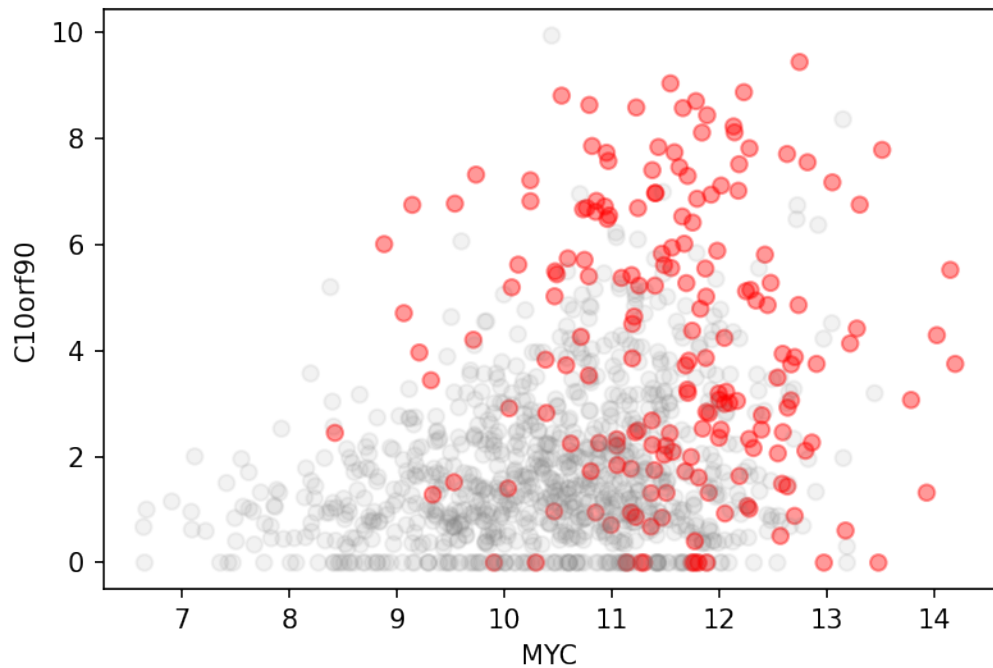

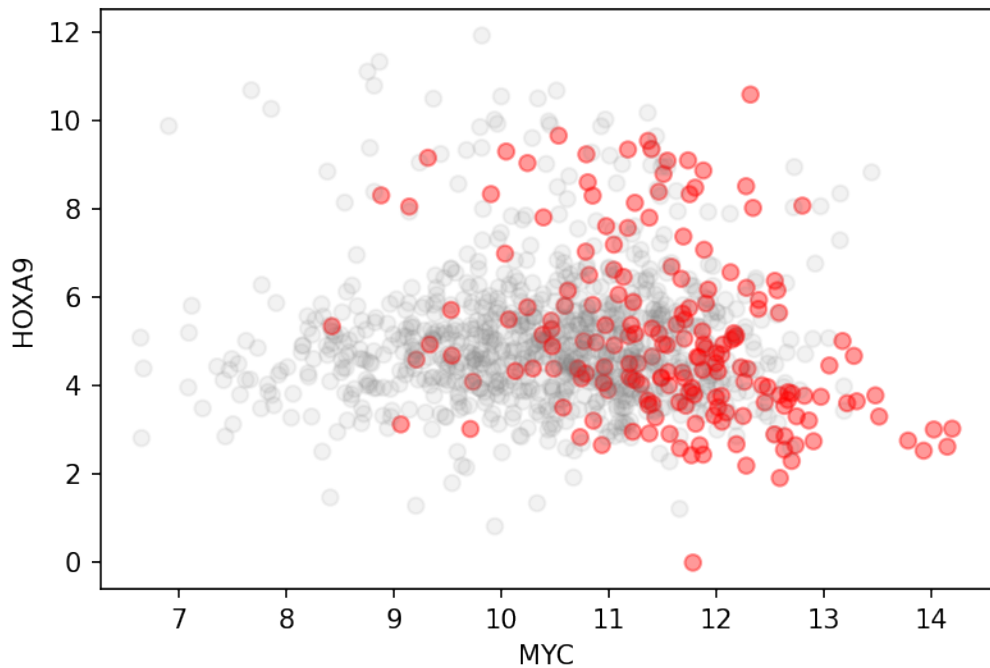

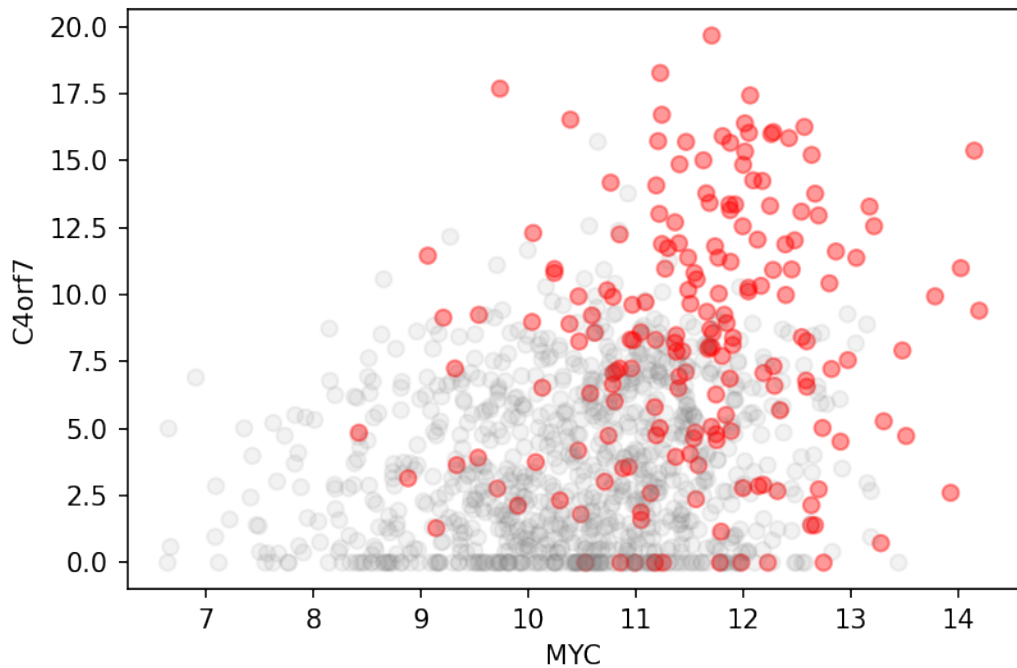

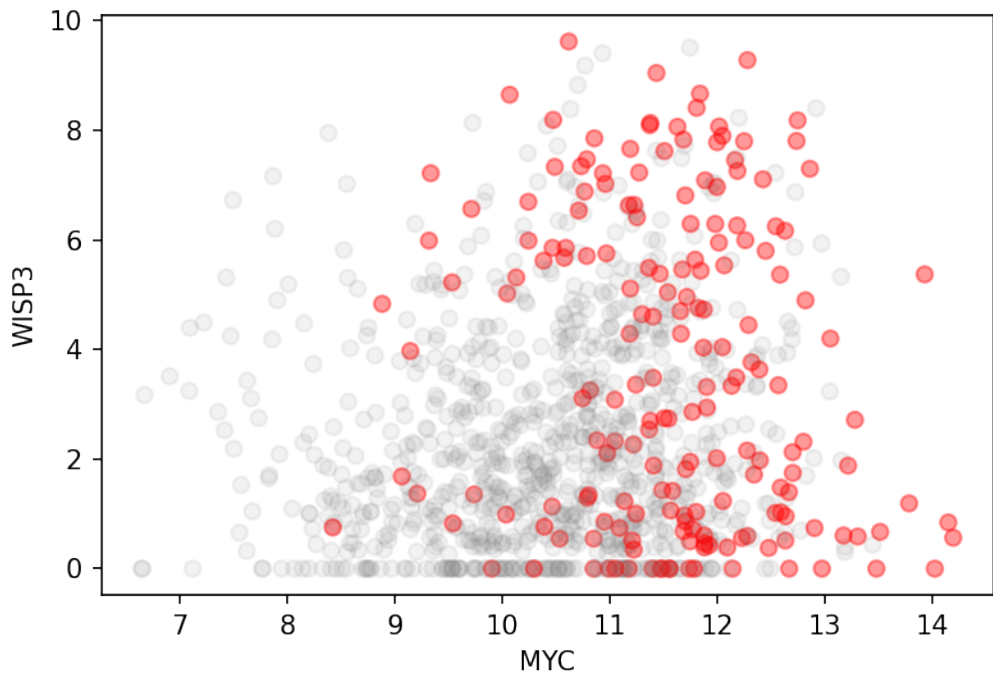

Supplement: Data S2. All 488 selected salient genes in the TCGA TNBC model, related to Figure 1 — For each representative gene, its log-transformed TPM expression and MYC expression are shown on scatterplots across all TCGA breast cancer samples. Each dot represents a TCGA breast cancer sample, with basal-like breast cancer samples highlighted in red. [file mmc9.pdf]
